# Xenobiotic Metabolism and Gut Microbiomes

**DOI:** 10.1371/journal.pone.0163099

**Published:** 2016-10-03

**Authors:** Anubhav Das, Meenakshi Srinivasan, Tarini Shankar Ghosh, Sharmila S. Mande

**Affiliations:** 1 TCS Research, Tata Consultancy Services Ltd., Pune, Maharashtra, India; 2 Manipal College of Pharmaceutical Sciences, Manipal University, Manipal, Karnataka, India; University of Illinois at Urbana-Champaign, UNITED STATES

## Abstract

Humans are exposed to numerous xenobiotics, a majority of which are in the form of pharmaceuticals. Apart from human enzymes, recent studies have indicated the role of the gut bacterial community (microbiome) in metabolizing xenobiotics. However, little is known about the contribution of the plethora of gut microbiome in xenobiotic metabolism. The present study reports the results of analyses on xenobiotic metabolizing enzymes in various human gut microbiomes. A total of 397 available gut metagenomes from individuals of varying age groups from 8 nationalities were analyzed. Based on the diversities and abundances of the xenobiotic metabolizing enzymes, various bacterial taxa were classified into three groups, namely, least versatile, intermediately versatile and highly versatile xenobiotic metabolizers. Most interestingly, specific relationships were observed between the overall drug consumption profile and the abundance and diversity of the xenobiotic metabolizing repertoire in various geographies. The obtained differential abundance patterns of xenobiotic metabolizing enzymes and bacterial genera harboring them, suggest their links to pharmacokinetic variations among individuals. Additional analyses of a few well studied classes of drug modifying enzymes (DMEs) also indicate geographic as well as age specific trends.

## Introduction

Advancements in medicine as well as health care, along with increasing awareness of health and hygiene have resulted in a significant rise in the demand and consumption of pharmaceuticals **[1]**. Reports have indicated that for certain countries (specifically in the middle income group), the consumption of drugs (especially those belonging to the Sulphonyl urea based categories) in the last decade has gone up by more than 250% (http://www.who.int/medicines/areas/policy/world_medicines_situation/en/index.html). Consequently, humans are increasingly exposed to various xenobiotics, which are typically the precursors of almost all categories of drugs/pharmaceuticals. However, given the foreign nature of these compounds (with respect to the human physiology), their metabolism, elimination and toxicity have become a major concern for clinicians and researchers working in areas of health sciences.

Human cells are equipped with a variety of enzymes to counter the probable harmful effects caused by these foreign compounds. For example, drugs administered orally pass through the alimentary canal and subsequently undergo a series of modifications. These modifications (referred to as Phase I of drug metabolism) are mainly carried out by hepatic enzymes, various cytochrome P450 proteins, and other enzymes encoded by our genome **[2, 3]**. The process involves modification of the chemical moiety using either oxidation (typically using the cytochrome P450 monoxygenases or flavin-containing monoxygenases or alcohol/aldehyde dehydrogenases) **[2]**, or other variants of modifications like, reduction (using cytochrome P450 reductases) and hydrolysis (by esterases and epoxide hydrolases) **[3]**. Various enzymes mentioned above are known to transform xenobiotics into less toxic, biocompatible and/or easily excretable forms. Many of these bio-transformations, convert the xenobiotics into bioactive compounds, which then serve their intended purpose **[4, 5]**. However, reports have also indicated that in certain cases, the metabolites formed during the course of xenobiotic metabolism are relatively more toxic than the administered compounds **[6, 7]**. Consequently, the balance between these biochemical events, viz., biotransformation of drugs into active compounds, formation of toxic metabolites and degradation of the xenobiotics consumed, decides the bioavailability, toxicity and other pharmacokinetic properties of the drugs.

Given the above context, drug efficacy, modulation of pharmacological properties (upon administration) and their links to human physiology are critical issues that need to be investigated prior to their administration. Several reports have provided various pharmacokinetic properties (e.g. ADMET) of numerous xenobiotics **[8]**. Combining information on drug properties with other factors like environment and host genetics/genomics, has led to a better understanding of not only drug bioavailability, toxicity and efficacy, but also of inter-individual variations in response to different drugs **[9]**. Although such insights have immense potential for furthering the development of personalized preventive/therapeutic strategies, our understanding regarding the contributions of various clinical and genetic factors in drug response is still incomplete. For example, 50% of the inter-individual variations in response to the drug warfarin are still unexplained **[9]**. In order to comprehensively understand the inter-individual variations in response to different drugs, it is important to investigate not only clinical, environmental and genetic components, but also other possible factors.

Recent studies have indicated the role of gut microbial community in the bioavailability and metabolism of various drugs **[10].** Since a majority of drugs are administered orally, and most of them get absorbed in our gut/intestines, the role of gut microbiota in modulating drug bioavailability, efficacy and toxicity is inevitable. Microbes in various ecosystems are known to have machineries that are utilized by them for metabolizing xenobiotic compounds by modifying and converting them to active/inactive/toxic metabolites **[10]**. Studies have also indicated that microbes can synthesize molecules that may affect the expression levels of host drug modifying enzymes, like cytochrome P450 **[11]**. The mechanisms of xenobiotic modifications and/or metabolism are mostly based on reduction, hydrolysis, mono-/di-oxygenation, cleavage and coupling reactions **[10, 12]**. Recent studies have suggested the metabolic capacity and capability of gut microbiota to be similar to those of the liver **[13]**. Thus, apart from gut microbiome’s role in various diseases/disorders, it seems to play a substantial role in physiological processes, including xenobiotic metabolism **[10, 14–19]**.

The studies pertaining to role of microbes in pharmacology and metabolism of drugs have been referred to as Pharmacomicrobiomics **[10]**. Traditionally, both *in vivo* and *in vitro* approaches have been used in pharmacomicrobiomics **[20–25]**. The *in vivo* strategies include analysis of drug metabolism and drug metabolites concentration using animal models **[20–29].** The use of animal models not only provides an *in vivo* system to study the metabolism and reactions of various drugs, but also bypasses the ethical and clinical issues associated with the use of humans in drug testing. However, accurate extrapolation of the patterns observed in the microbiomes of such animal models to humans has certain limitations, including differences in surface area ratio, presence or absence of appendix (which has been reported as a reservoir of beneficial gut microbes **[30]**) and diet **[31]**. Also, it has been shown that while the composition similarity can be seen only at higher taxonomic levels (like phylum or superkingdom level), almost 85% of microbial composition in humans and mice are not similar **[32]**. In order to overcome this limitation, studies have utilized transplantation of human faecal microbiota into germ free/gnotobiological mice. The faecal microbiota transplantation (FMT) approach, however, has certain caveats which render this approach inefficient to understand the microbial basis of human physiology. These include anatomical anomalies associated with germ free mice like enlarged caecum, reduced villous thickness and villous capillary networks, etc. **[33]**.

In contrast, *in vitro* approaches include assaying specific drug degrading capabilities of various microbes using faecal samples **[25, 34, 35]**. However, adopting conventional techniques for culturable bacteria has a major limitation since a majority of microbial species residing in various environments cannot be cultured using traditional laboratory techniques **[36]**. This inability to study the microbial community structure of the environment (*in vivo*, *in vitro* or *in situ*) can be overcome using the emerging field of metagenomics. Several studies have adopted the metagenomics approach for investigating the role of gut microbiota in various diseases/disorders **[37–41]**.

The available huge repositories of gut microbiome datasets from various geographies and age groups have been utilized by different groups for understanding the role of gut microbiomes in various diseases/metabolic disorders **[42–48]**. These repositories can be mined for predictive profiling of enzymes belonging to various biochemical pathways, including those of xenobiotic metabolism. Such studies are likely to lead to the identification of geographic as well as age-specific variations in the xenobiotics/drug metabolizing capabilities of the gut microbiome.

With the above motivation, we present in this paper an in-depth analysis of xenobiotic metabolism pathways using an informatics approach. In addition to performing a comparative profiling of homologues of 592 xenobiotic metabolizing proteins (belonging to 32 xenobiotic metabolizing pathways) across 850 microbial genera, 397 publicly available metagenomic datasets belonging to 8 regions, viz., American, Danish, Spanish, French, Italian, Chinese, Indian and Japanese (belonging to 6 age groups, viz, AG1, AG2, AG3, AG4, AG5 and AG6), were analyzed with the objective of identifying inter-individual, geographic and inter-age group variations of gut microbiome’s xenobiotic metabolizing capabilities and associated microbial community structure (S1 Table). Variations of selected well studied drug modifying enzymes (DMEs) involved in phase I and phase II reactions of drug metabolisms **[49, 50]** were also evaluated. The phase I drug metabolizing enzymes included cytochrome P450s (CYP), monoamine oxidase (MO), epoxide hydrolase (EH), alcohol (ADH) and aldehyde dehydrogenase (ALDH). Similarly the enzymes involved in phase II reactions involved in drug metabolism, that were considered in the present study included thiopurine methyltransferase (TPMT), N-acetyl transferase (NAT) and glutathione S-transferase (GST).

## Results

### Profiling xenobiotic metabolizing capabilities of microbes

**Fig 1:**
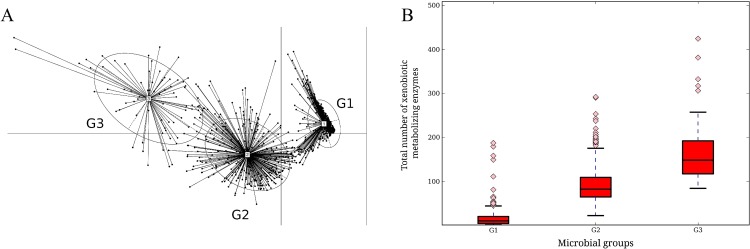
Principal Coordinate analysis (PCoA) of profiles of xenobiotic metabolizing enzymes in microbes (at genera level). **(A)** Profiles of xenobiotic metabolizing enzymes were obtained as abundance of selected enzymes in each genus per genome present in the database. Members of each cluster include microbial genera having similar abundance patterns of xenobiotic metabolizing enzymes. **(B)** Diversity of xenobiotic metabolizing enzymes harbored by members belonging to clusters G1, G2 and G3 were calculated and plotted as box plot. Significant differences (Kruskal-Wallis test p-value <0.05) in abundances of enzymes are observed among members of each cluster. Based on the diversities, the three groups, G1, G2 and G3 are considered as least versatile (LV), intermediately versatile (IV) and highly versatile (HV) xenobiotic metabolizers, respectively.

In order to investigate xenobiotic metabolizing capabilities of various microbes, homologues of such enzymes were first identified across all fully and partially sequenced microbial genomes. The abundance profiles of these enzymes, at the taxonomic level of genus, were obtained for each of the 850 genera. Principle coordinate analysis (PCoA) of these profiles identified three distinct clusters, referred to as G1, G2 and G3 (Fig 1A). Each cluster represented groups of microbial genera having similar patterns of xenobiotic metabolizing enzyme abundances in them. The list of genera, their group affiliations, along with the abundances of the various xenobiotic metabolizing proteins harbored by them is provided in S2 Table. Out of the 850 genera, 442 belonged to G1, 339 belonged to G2 and only 69 belonged to the group G3. Comparison of the members belonging to different clusters (G1, G2, G3) indicated differences in the diversity of xenobiotic metabolizing enzymes (i.e. the total number of detected enzymes) harboured by them (Fig 1B). The diversity of these enzymes was observed to be highest for members belonging to group G3 and lowest for those belonging to G1. Thus, based on the diversity of the enzymes harboured, the members belonging to G1, G2 and G3 could be classified as least versatile (LV), intermediately versatile (IV) and highly versatile (HV) xenobiotic metabolizers, respectively. The fraction of species belonging to the aforementioned genera, harbouring the xenobiotic metabolizing enzymes, also showed variations (S3 Table).

It is to be noted that the enzymes considered in the study encompass a large variety on xenobiotic metabolizing enzymes that are likely to be functional in various pathways. Thus, only a few selected well studied drug modifying enzymes (DMEs) which are involved in phase I and phase II reactions of drug metabolisms were also evaluated. Similar to the variations of the xenobiotic metabolizing abundances in the microbial groups, members of G3 were observed to harbour the highest abundance of the DMEs, followed by members of G2 and G1 (S1 Fig). The details of EC numbers and KO ids, corresponding to these DMEs have been listed in S3 Table. Thus, in addition to the total number of xenobiotic metabolizing enzymes, the abundances of certain drug metabolism enzymes involved in phase I and II reactions of drug metabolism also correlate with the versatilities (i.e. LV, IV and HV) of the xenobiotic metabolizers.

### Profiling homologues of xenobiotic metabolizing enzymes in gut microbiomes

#### Xenobiotic metabolizing enzyme repertoire in gut microbiomes of individuals from various geographies

**Fig 2:**
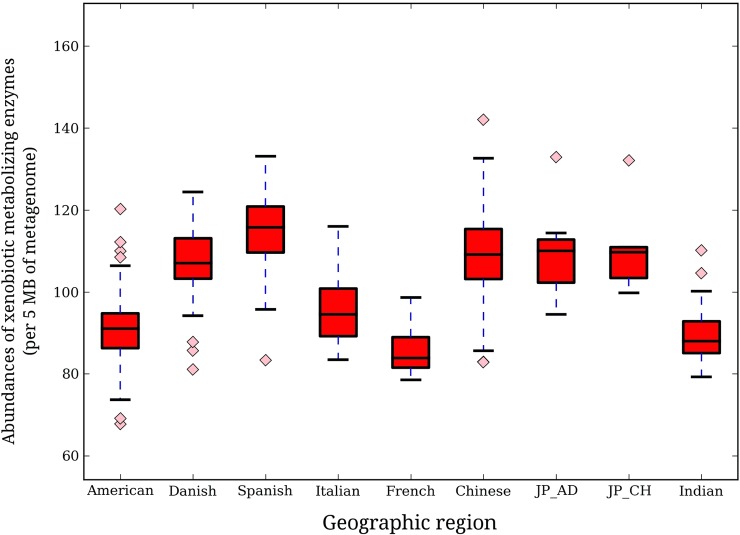
Variation in abundances of xenobiotic metabolizing enzymes in gut microbiomes of individuals belonging to different geographies. Abundance of enzyme homologues in each sample was calculated as number of contigs showing hits with the enzymes of interest per 5 mega bases of respective metagenome volume. Significant differences (Kruskal-Wallis test, p-value< 0.05) in abundances were observed both at intra and inter-regional levels. While Spanish individuals had the highest abundances, Chinese population showed highest inter-individual variations.

**Fig 3:**
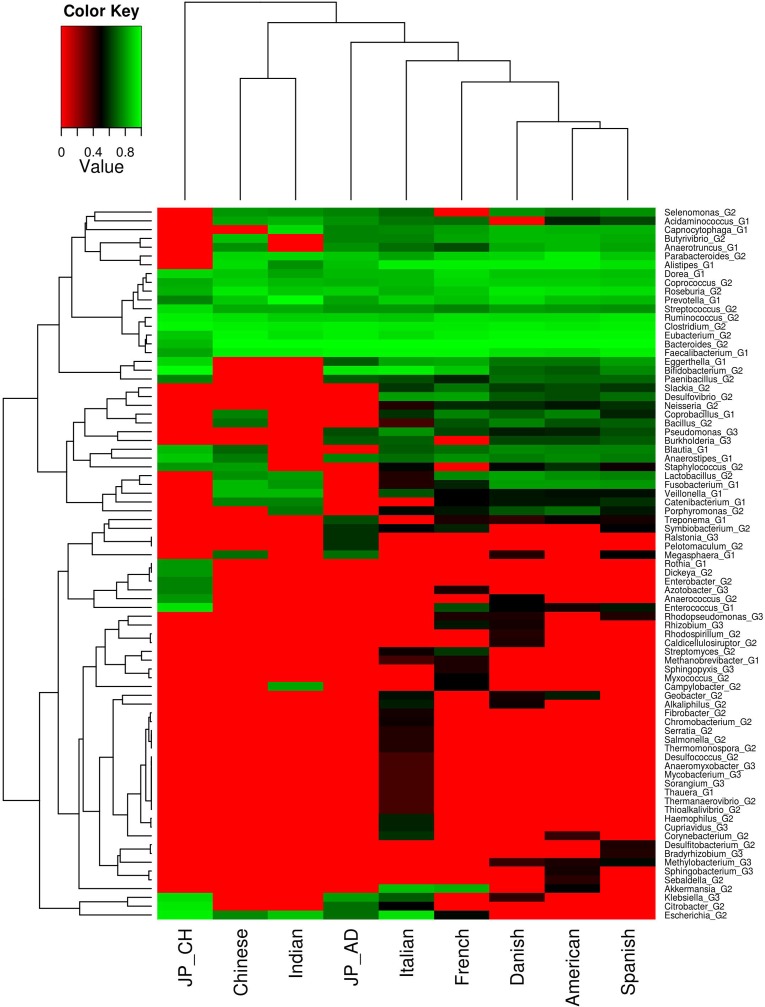
Analyses of microbial groups (genera) harbouring xenobiotic metabolizing enzymes in the gut microbiomes across different geographies. Specific abundance profiles, representing contribution of each genera in harbouring xenobiotic degrading enzymes, in each sample was calculated. Genera which were present in minimum of 30% of samples, belonging to at least one region, were considered. Median values of each microbial genus for each region was calculated and ranked within each regional group, and plotted as heat map. Differential as well as specific microbial compositions were observed in different regions. While some microbial genera were found to have similar abundance in all the regions, some showed region specific preferences. European and American regions clearly clustered distinctly from Asian samples.Kruskal-Wallis H test was performed among groups for each genera and significant genera (p-value < 0.05) have been reported.

To investigate whether there exist any geographic trend with respect to the microbial composition as well as abundances of the xenobiotic metabolizing enzyme repertoire in the gut microbiomes, homologues of various xenobiotic metabolizing enzymes were identified in various metagenomic samples (S5 Table). Abundance of xenobiotic metabolizing enzymes (calculated as the number of homologues identified per 5 million base pairs of metagenome) were obtained for each gut microbiome and compared across various nationalities (Fig 2). As can be seen from the figure, Spanish individuals showed the highest median abundance (115.8), followed by adult Japanese (110.1), Chinese (109.2) and Danish (107.1). The Chinese were observed to have the highest degree of inter-individual variation. Interestingly, in contrast to the noticeable differences in the median abundance of xenobiotic metabolizing enzymes across the European and American nationalities, the East Asian populations (Chinese, Japanese adults and children) were observed to have almost similar median abundances. Based on the specific abundance, representing the contribution of each genusto the xenobiotic metabolizing enzyme repertoire of gut, certain geographic clustering trends were observed. While the European and American regions clustered closer to each other, the Asian populations (Indian, Chinese and Japanese children) clustered separately. The only exceptions to this trend were the Japanese adults that clustered with the European populations but appeared closer to the Asian cohorts (Fig 3). These results suggest that the individuals belonging to different geographies have gut microbiota with distinct xenobiotic metabolizing capacities.

The microbial groups could be further divided into different categories based on their specific abundance patterns (in the xenobiotic metabolizing enzyme repertoire) across various nationalities (Fig 3). The first group consisted of genera which had a high specific abundance across all regions. These included the commonly found gut associated genera like *Prevotella*, *Faecalibacterium*, *Dorea*, *Roseburia*, *Eubacterium*, *Ruminococcus*, *Bacteroides*, etc. The second group included genera like *Neisseria*, *Bacillus*, *Slackia*, *Coprobacillus*, *Treponema*, etc. While, these genera were observed to have higher specific abundance across the European and American populations, they were found to be relatively depleted in the gut microbiomes of individuals belonging to the Asian nationalities. The third microbial group, consisting of the genera like, *Rhizobium*, *Rhodospirillum*, *Bradyrhizobium*, *Rhodopseudomonas*, *Methylobacterium*, were sparsely detected in the xenobiotic metabolizing enzyme repertoire in the gut microbiomes of European and American individuals and were not detected in the gut microbiomes of individuals from Asia. The genus *Escherichia*, as part of the genera harboring the xenobiotic metabolizing enzyme repertoire, was only detected in the gut microbiomes of the Asian individuals. These results indicate that the xenobiotic metabolizing enzyme repertoire in the gut microbiome differs noticeably across nationalities and bears certain geography-specific trends.

Investigating the specific abundances of genera harboring the DMEs showed geography-specific clustering of sample groups. While the American and European groups clustered together, the Asian cohorts showed region specific clustering (S2 Fig). Interestingly, the Japanese groups, i. e., Japanese adults and children appeared to cluster together and were separated from the Indian and Chinese groups. It was also observed that most of the microbial genera had higher specific abundance of DMEs in the European groups. These results suggest that the gut of individuals belonging to different geographies vary with respect to abundances of specific drug metabolizing enzymes that are involved in phase I and II reactions during drug metabolism.

To further explore the variation of enzyme abundance within sample cohorts, median abundance of each xenobiotic metabolizing enzyme (EC number and KO ids present in at least 30% of samples of either of the regions) was calculated for each cohort followed by rank normalization and bi clustering on a heat map (S3 Fig). While cohorts obtained from older age groups (French and Italian samples with age> 60 yrs) were observed to cluster together along with Japanese adults (AG2-AG4), Japanese children were observed to be outliers. All the other sample groups (Indian, Danish, Spanish, American and Chinese) clustered separately according to geography. Although, there was no function specific patterns of the DMEs, the enzyme involved in phase I reactions of drug metabolism (ADH, ALDH, CYP, EH, MO) showed higher numbers in Japanese, French and Italian samples. On the other hand, the DMEs involved in Phase II reactions (GST and NAT) of drug metabolism showed higher abundance in Indian, Chinese, American, Danish and Spanish cohorts. This suggests that along with other xenobiotic metabolizing enzymes, these specific DMEs may have a role in the drug metabolism variations between sample groups.

Cumulative abundance analysis of the selected DMEs showed that these enzymes contributed 40% of the overall xenobiotic metabolism enzyme abundance in each of the cohorts. Based on median abundance in each region, these enzymes were found to cluster according to their functions (S4 Fig). While enzymes involved in phase I of drug metabolism appeared together, enzymes involved in phase II reaction (i.e., conjugation reactions) belonged to a separate cluster. Further, the cohorts showed age-dependent grouping. While Japanese and Indian children appeared to be outliers, eldest sample groups (FR and IT) formed a part of separate cluster and those belonging to middle age groups occurred together. This suggests that the biochemical roles of the drug metabolizing enzymes involved in phase I and phase II drug metabolism reactions co-occur, irrespective of the variations of the abundances across sample groups.

Analysis of the diversity of xenobiotic metabolizing genes and their abundances in the metagenomic samples showed a direct relationship. However, the degree of correlation across various regions was observed to vary (S5 Fig). While a strong positive correlation was seen between the diversity and the abundance of these genes in the Chinese, Spanish and Japanese populations, the degree of positive correlation was observed to be lower for other populations. The Shannon diversity distributions of the microbial genera for the overall microbiome as well as the specific microbiome harboring the xenobiotic metabolism repertoire, for the European populations were observed to have higher median values than the Asian cohorts (S6A and S6B Fig).

**Fig 4:**
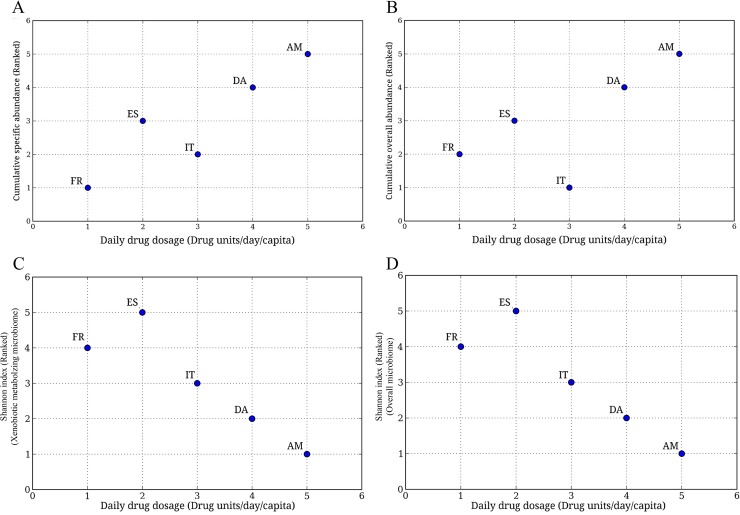
Variation of cumulative abundances of genera harboring xenobiotic metabolizing enzymes with ranked Daily Drug Dosage (DDD). (**A**) Variation of ranked cumulative specific abundance with ranked DDD.Cumulative specific abundances of microbial genera of groups G1, G2 and G3 were calculated for each geography, namely, France (FR), Italy (IT), Spain (ES) and America (AM). The Cumulative specific abundance represents the abundance of each microbial genera in the xenobiotic metabolizing repertoire, and is obtained by calculating number of contigs (in the corresponding metagenome) showing hits against genes corresponding to xenobiotic metabolizing enzymes, assigned to a particular genera, normalized by total number of contigs in the metagenome. Median was calculated for each region and was ranked, and plotted against their respective daily drug dosage (ranked) obtained from earlier reports. A linear increase in abundance with increase in DDD was observed suggesting a role of drug consumption in modulating the abundances of xenobiotic metabolizing enzymes. Kruskal-Wallis H test was performed and the differences were found to be significant (p-value < 0.05). (**B**) Variation of ranked cumulative overall abundance with ranked DDD.Overall abundance of each genus, as a measure of contribution of each microbial genera, in the whole metagenome of each sample, was considered. Cumulative of the overall abundance of microbial genera of groups G1, G2 and G3 were calculated for each sample. Median was calculated for each region and was ranked and plotted against their respective daily drug dosage. A similar (linear) trend obtained, suggest a role of drug consumption in modulating the abundances of xenobiotic metabolizing microbes in the microbiome. Kruskal-Wallis H test was performed and the differences were found to be significant (p-value < 0.05).**(C)** Variation of Shannon index calculated for xenobiotic metabolizing microbiome with ranked DDD. Kruskal-Wallis H test was performed and the differences were found to be significant (p-value < 0.05).**(D)** Variation of Shannon index of overall microbiome with DDD. Kruskal-Wallis H test was performed and the differences were found to be significant (p-value < 0.05).

In order to evaluate whether the observed geography-specific trends of the abundance as well as the microbial composition of the xenobiotic metabolizing enzyme repertoire correlates with the drug consumption, the available daily drug dosage data (Drug units/day/capita) for five nationalities (Americans, Denmark, Spain, France, Italy) was utilized **[1, 51]**. This data was compared with the specific as well as the overall abundances of the three groups of bacteria (namely G1 or LV xenobiotic metabolizers, G2 or IV xenobiotic metabolizers and G3 or the HV xenobiotic metabolizers). Except for the Italian population, a positive relationship between specific (as well as the overall) abundance of drug-metabolizing gut bacteria and the per capita drug consumption was observed (Fig 4). This indicates that high drugs/xenobiotics usage is likely to create an environment that provides a selective survival advantage to drug metabolizing bacteria, thereby increasing their abundance in the gut microbial community.

An interesting observation was obtained when the variation of Shannon diversity values (both overall gut microbiome as well as xenobiotic metabolizing gut microbiome) was compared with drug consumption data available for certain countries. Contrary to the direct relation between cumulative genera abundance and drug consumption, the Shannon diversity showed an inverse relation. In other words, the Shannon diversity was observed to be lower for regions with higher drug consumption as compared to those with low drug intake (Fig 4C and 4D).

#### Xenobiotic metabolizing enzyme repertoire in gut microbiomes of individuals of various age groups

**Fig 5:**
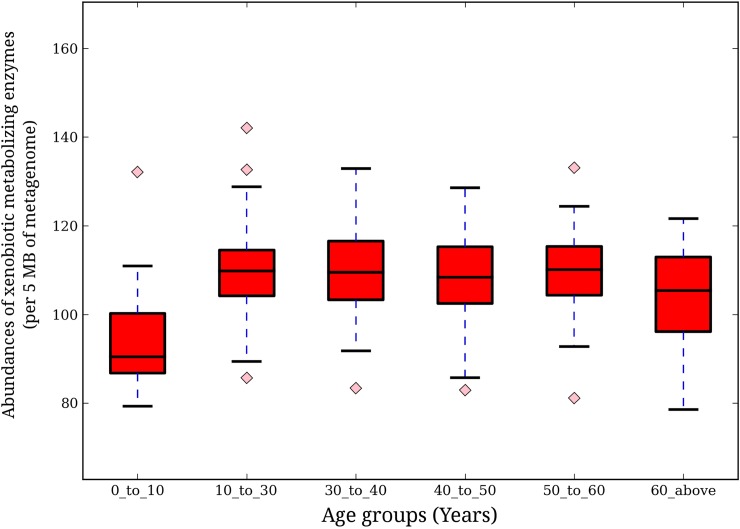
Variation of xenobiotic metabolizing enzyme abundance across age groups. Abundance of enzyme homologues for each sample was calculated as number of contigs showing hits with the enzymes of interest per 5 mega bases (Mbp) of respective metagenome volume. Significant differences (Kruskal-Wallis test, p-value< 0.05) in homologue abundance were observed for both at intra and inter-regional levels. The youngest group (AG1: 0–10 years) showed the least abundance, followed by a sudden increase at age group G2 (10–30 years), and subsequently, a saturation in abundance was observed.

To investigate patterns of xenobiotic metabolizing enzyme repertoire in the gut microbiome of individuals belonging to various age groups, the individuals under study were categorized into 6 age groups (AG1: 0–10 years, AG2: 10–30 years, AG3: 30–40 years, AG4: 40–50 years, AG5: 50–60 years, AG6: 60 years and above). The abundance of xenobiotic metabolizing enzymes showed inter-age group variations (Fig 5). Lowest abundance of xenobiotic metabolizing enzymes was observed in the youngest age group (0–10 years). A sharp increase in their abundances was observed in the age group of 10–30 years, followed by saturation beyond this age group. This suggests that, increase in exposure to wide range of drugs/xenobiotics with age probably leads to an increase in the xenobiotic metabolizing potential of the gut microbiome.

**Fig 6:**
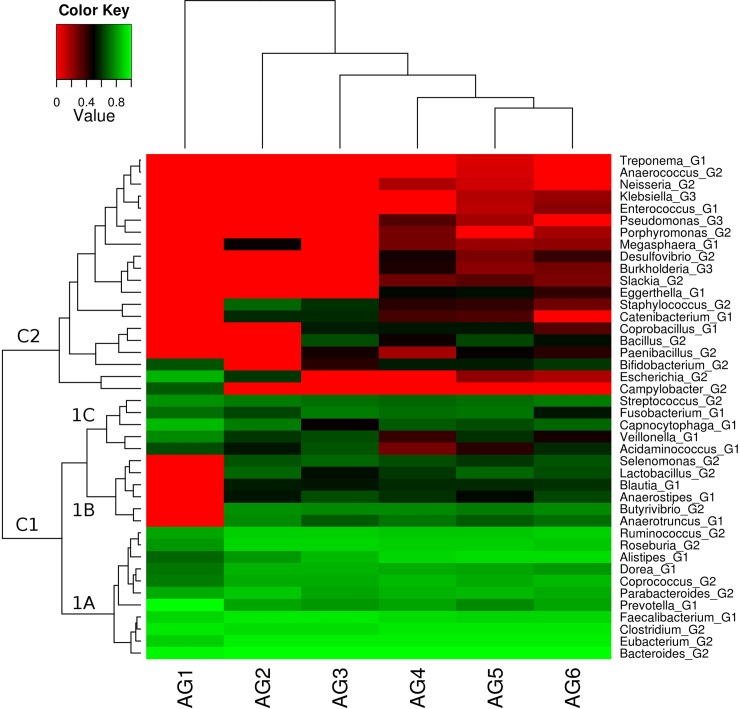
Variations in microbial genera composition and abundance of xenobiotic metabolizing enzymes across different age groups. Specific abundance profile, representing contribution of each genus in harbouring xenobiotic metabolizing enzymes, in each sample was generated. Only those genera were considered which were present in minimum of 30% of samples belonging to at least one age group. Median values of each microbial genus for each region was calculated and ranked within each age group, and plotted as heat map. There is a differential as well as specific microbial composition in different age groups. While some microbial genera were found to have similar abundance in all the age groups, some showed age specific preferences. Age-specific clustering pattern was also observed. Kruskal-Wallis H test was performed among groups for each genera and significant genera (p-value <0.05) have been reported.

Based on the xenobiotic metabolizing enzyme repertoire in the microbiomes of all individuals, age-specific clustering was observed (Fig 6). This suggests that gut microbial composition harbouring the xenobiotic metabolizing enzyme repertoire has age specific signatures. Further it was observed that different genera could be divided into two broad groups based on their specific abundance profiles across age-groups (Fig 6). Members of the first group, comprising equal numbers of LV xenobiotic metabolizers (belonging to group G1) and IV xenobiotic metabolizers (belonging to group G2), were observed to be present across all age-groups. Members of the second group, consisting of mostly IV and a few HV xenobiotic metabolizers (belonging to groups G2 and G3, respectively), were observed to populate the xenobiotic metabolizing enzyme repertoire of individuals above the age of 30 years (AG3-AG6). The first group could further be sub-divided into three sub-groups, namely 1A, 1B and 1C. While members of 1A had a noticeably higher abundance across all age-groups as compared to 1B and 1C, members of 1B was found to be absent in the youngest group (AG1). This indicates that certain genera gain entry into xenobiotic metabolizing enzyme repertoire with increasing age of the individual.

When specific abundance of microbes harbouring the selected DME classes was analyzed, a similar age related clustering of the sample cohorts was observed (S7 Fig). While the youngest age group (AG1) appeared to be outliers, the comparatively older age groups (AG2 and AG3) were observed to cluster together. The eldest age groups appeared as a complete separate cluster. The microbial genera, harbouring the DMEs, also showed age specific signatures. While, those genera, highest in AG1 were observed to be present in other sample groups, microbial genera, specific to older groups, were seen to cluster together. These results suggest that the potential of gut microbes harbouring drug metabolizing enzymes is dependent on age.

Enzyme abundance analysis of the members of age groups, also showed age specific signatures (S8 Fig). The youngest age group appeared as an outlier, followed by seniority based clustering of the sample groups. Interestingly, it was observed that the DMEs involved in phase I reactions (ADH, ALDH and EH) were present in higher abundance in younger age groups. Conversely, the phase II DMEs (GST, NAT, and TPMT) were observed to be higher in the older groups of samples. Analyzing the cumulative abundances of the selected DME classes indicated that while DMEs specific to Phase II drug metabolism are present in high amounts in older age groups, those involved in phase I reactions are found to be low in all the age groups (S9 Fig). The increased abundance of phase II DMEs in the older sample groups may be because of increase in xenobiotic exposure.

**Fig 7:**
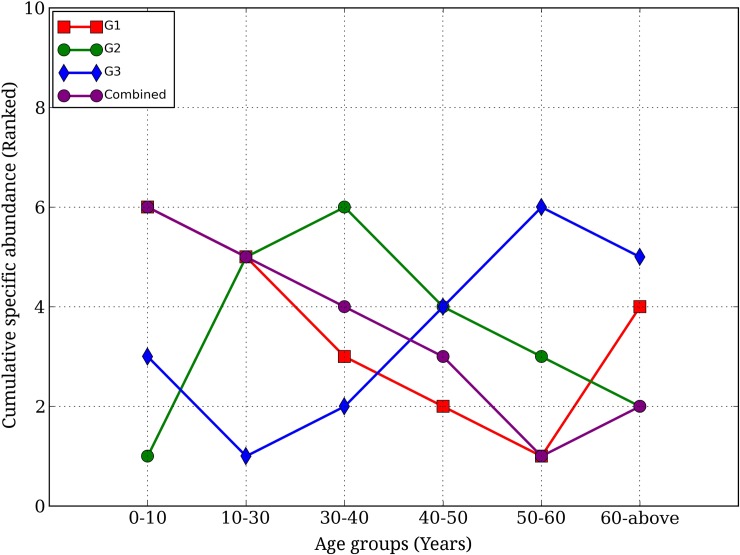
Variation of cumulative specific abundance of genera belonging to the three categories (Least versatile, Intermediately versatile and Highly versatile xenobiotic metabolizers) with respect to age groups. Abundances of members of each category, namely, G1, G2 and G3, were cumulated and variations were checked with increase in age. The cumulative abundances were ranked among each other and plotted against their respective ranked age groups, based on seniority. All reported differences were statistically significant (Kruskal_wallis H-Test, p-value < 0.05).

For each age group, analysis of cumulative specific abundances of three functional categories of genera, namely, LV xenobiotic metabolizers (G1 members), IV xenobiotic metabolizers (G2 members) and HV xenobiotic metabolizers (G3 members), further identified prominent variations with age (Fig 7). While the cumulative abundance of LV xenobiotic metabolizing group (G1) was observed to be highest at the youngest age group (AG1), it was found to decrease till the penultimate group and increase at the oldest age group (AG6). The cumulative abundances of the IV xenobiotic metabolizing group (G2) was found to increase till the age of 40 (AG3), followed by decrease thereafter. The abundances of the HV xenobiotic metabolizing group (G3) were observed to increase from the age of 10 (AG2) till 60 (AG5) and were found to decrease slightly at old age (AG6). These results indicate that the abundances of the three xenobiotic metabolizing groups (i.e. low, intermediate and highly versatile) show distinct trends across age groups.

**Fig 8:**
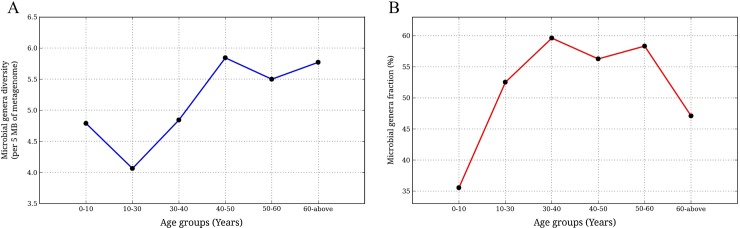
Variation of diversity of microbial genera harboring xenobiotic metabolizing enzymes with age group. (**A**) Variation of diversity of microbial taxa with age groups. Microbial taxa diversity was calculated as number of unique microbial genera, harbouring xenobiotic metabolizing enzymes, per 5 mega bases of the metagenome. The calculated diversity was plotted against respective age groups. (Significance: Kruskal-Wallis H test, p-value < 0.05).(**B**) Variation of fraction of microbial genera, harbouring xenobiotic metabolizing enzymes, with age. Microbial genera fraction was calculated as the ratio of number of microbial genera harbouring xenobiotic metabolizing enzymes and total number of microbial genera present in the metagenome. The obtained fraction, representing microbial contribution towards repertoire of xenobiotic metabolizing enzymes, was plotted against respective age groups (Significance: Kruskal-Wallis H test, p-value < 0.05).

The diversity of microbial genera that harbor xenobiotic metabolizing enzymes was found to decrease from group AG1 (0–10 years) to AG2 (10–30 years), increase until middle age group AG4 (40–50 years), and finally attain saturation beyond the age of 50 (AG5 and AG6) (Fig 8A). Investigation of the percentage of genera harboring the homologues of xenobiotic metabolizing enzymes across individuals of different age groups indicated lower values at the youngest and oldest age groups and a zone of stability in the intermediate ones (Fig 8B). Similar to regional level analysis, an overall positive correlation was observed between Shannon diversity of the genes of xenobiotic metabolizing enzymes and their abundances in the samples (S5 Fig). While a strong correlation was observed in AG2 and AG3, other age groups did not display a strong correlation.

At the age group level, it was seen that the median Shannon diversities (corresponding to the overall as well as the xenobiotic specific microbiome) increased with seniority of age groups (S6C and S6D Fig). This indicates that the overall diversity of the gut microbiota including that of the microbiota harbouring the xenobiotic metabolizing repertoire increases with age. This is further indicated by the strong positive correlation between the Shannon diversity and the age of the individuals, obtained using a windows based approach **[41]** (S6E and S6F Fig).

#### Enzyme level analysis of Gut microbiomes

Different sample groups corresponding to various geographies and age groups are likely to harbor different xenobiotic metabolizing enzymes originating from the same genus. In order to investigate this, the abundances of each xenobiotic metabolizing enzyme (present in at least 30% of samples belonging to either different geographies) in all the microbes which showed significant differences at the regional level (S6 Table) were evaluated. This was followed by pair-wise quantification of dissimilarity using cosine distance as a measure.

The results (S10 Fig) indicate a marked dissimilarity between microbiomes of various cohorts and the xenobiotic metabolising enzymes contributed by them. Interestingly, samples belonging to Indian and Japanese children (i.e. younger age group) grouped separately while the Japanese and Chinese adults were seen to occur with European cohorts. While the Italian and French groups appeared together (majority of samples above age of 60 years), all the other European groups (Danish and Spanish) were seen as a separate class along with the Chinese and American cohorts (majority of samples in the middle age group, 30–60 years). These results further suggest a consorted effect of geographical region as well as age group on the xenobiotic metabolizing repertoire of the gut microbiome.

#### Classification of enzymes based on abundance in samples

A broad level Principal Component Analysis of the enzymes of interest was performed based on their abundances in the samples. It was observed that the enzymes formed 4 distinct clusters (S11 Fig and S7 Table). Based on ranked median cumulative abundances of members of each cluster in each sample group, it was observed that the sample groups clustered strictly based on ethnicity (S12 Fig). While abundance of cluster 1 was observed to be higher in the Asian cohorts, cluster 3’s abundance was high in the European sample groups. This indicates that xenobiotic metabolizing enzyme repertoire differed across populations.

Following PCoA, we characterized the members of the clusters based on COGs (cluster of orthologous groups). We identified the COG affiliations for each EC numbers and KO ids and observed that members of cluster 1 contained the highest number of COGs, followed by cluster 3, cluster 4 and cluster 2, which contained very less COGs as compared to cluster 1 (S13 Fig). The EC numbers and KO ids, along with their corresponding cluster affiliation and COGs have been listed in S7 Table. It was observed that COGs belonging to each cluster were mostly unique and very less COGs were common between the clusters suggesting a functional based separation of the xenobiotic metabolizing enzymes (S14 Fig and S8 Table). We further checked the COG functional category distribution of the clusters. Similar to previous observation, cluster 1 included highest number of functional categories, followed by cluster 3, 4 and 2 (S15 Fig). This suggests that the members of cluster 1 are more dynamic and play major role in different other processes besides metabolism of xenobiotics. Overall specificity of the functional categories identified, showed that COG categories belonging to cellular metabolism were highest in each of the clusters (S16 Fig). The DMEs also belonged to a variety of COGs. It was observed that the enzymes involved in phase I reactions of drug metabolism (ADH, ALDH, EH and MO) belonged to less number of COGs than those involved in phase II reactions of drug metabolism (GST, NAT, TPMT). While the enzymes involved in phase I reactions belonged to an average of 7.7 COGs per enzyme, the phase II DMEs belonged to a higher average COGs per enzyme (12.09). The details of enzymes, their names, number of COGs and the COGs are provided in S4 Table.

## Discussion

The current study reports, for the first time, a genome wide profiling of xenobiotic metabolizing enzymes as well as xenobiotic metabolic capabilities of various bacteria (and their communities) based on genomic (and metagenomic data). The study has identified 850 bacterial genera that can potentially metabolize one or more xenobiotics. Results also indicated the presence of three groups of bacterial genera that have differences in the overall abundances of xenobiotic metabolizing enzyme repertoire. These groups include LV xenobiotic metabolizers (G1), IV xenobiotic metabolizers (G2) and HV xenobiotic metabolizers (G3). Family level analyses of these genera indicated distinct tendencies of the respective families to belong to the groups of LV, IV and HV xenobiotic metabolizers (S17 Fig and S9 Table). These differences in tendencies (to belong to one of the groups) could be a reflection of the ecological/habitat specific preferences. For example, as observed in a previous study **[52]** species belonging to families like Comamondaceae and Burkholderiaceae are present in a larger variety of habitats, as compared to those belonging to the family Enterobactericeae (which are present mainly in gut and aerial environment). Consequently, Comamonadaceae and Burkholderiaceae are expected to be exposed to a higher variety of chemicals (including Xenobiotics) and thus harbor a larger xenobiotic metabolizing repertoire as compared to Enterobacteriaceae. This trend is also revealed in the current analysis, where in, Enterobacteriaceae is observed to be less versatile in its xenobiotic metabolism (and hence tend to belong to the group G2), as compared to Comamondaceae or Burkholderiaceae (which are more versatile and hence tend to belong to G3).

Results of the analyses on 397 gut metagenomes of individuals belonging to different geography also indicate distinct inter-regional variations in the overall abundances of xenobiotic metabolizing enzymes. The study also suggests that an increase in drug consumption may result in a concomitant increase in the number of bacterial lineages harbouring enzymes that metabolize these xenobiotics. It is important to note that not all xenobiotics are drugs and many of them (that utilize pathways for detoxification of environmental pollutants) are not used clinically. However, since the majority of drugs are structurally homologous to some xenobiotic or intermediates (or metabolites) of the corresponding metabolizing pathway, they are likely to be metabolized by enzymes of the same degrading pathways. It can be assumed that if an individual’s gut microbiome has higher abundance of homologues of xenobiotic metabolizing enzymes, then he/she is likely to have higher xenobiotic metabolism capability. This increase in the xenobiotic metabolism capability of the microbiome is likely to result in a decrease in the bioavailability of the xenobiotics in this individual. This in turn may lead to an increase in the toxicity levels for some of the drugs.

We have compiled information on few clinically relevant drugs and their metabolic pathways from widely used reference databases/repositories like KEGG, BioCyc, PubMed and www.drugbank.ca (S10 Table). It is observed that, with the exception of DDT, Nitrotoulene and Atrazine, 25 out of 28 different xenobiotic metabolizing pathways have the potential to metabolize at least one clinically used drug. Thus, there is an overlap between majority of xenobiotic metabolizing pathways (considered in the study) and different classes of drugs.

Another interesting observation from the present study pertains to the xenobiotic metabolizing capability of microbial composition (in terms of the different bacterial genera) in individuals belonging to different ethnicities. For example, Asian individuals (Indian, Chinese and Japanese) are observed to have similar compositions of bacterial groups containing these enzymes, which are distinct from those in American and European individuals. This result is in line with previously published studies, which have shown a similarity in drug response with ethnicity **[53–54]**. Results of the present study also indicates similarity in the xenobiotic metabolizing enzymes within various age groups, an observation that is in line with the earlier reports which suggest xenobiotic metabolism and drug response to be dependent on age **[55–57]**. However, it is interesting to note that this observation is true irrespective of ethnicities of the individuals in each age group.

The present study also indicates that bacterial genera that are LV xenobiotic metabolizers (G1), IV xenobiotic metabolizers (G2) and HV xenobiotic metabolizers (G3) have different representations in the gut microbiomes. While members of IV xenobiotic metabolizers have highest abundance (60%) in the gut microbial communities, members of HV xenobiotic metabolizers have lowest abundance (1–2%). On the other hand, members of LV xenobiotic metabolizers are observed to have an intermediate abundance (around 20%). In spite of having an intermediate diversity of xenobiotic metabolizing enzymes of IV xenobiotic metabolizers, members belonging to this G2 group have the potential to metabolize the most number of drugs (S11 Table). In other words, in spite of having lesser variety of individual enzymes, G2 members are likely to contain enzymes that have the ability to metabolize a much larger number of xenobiotics. Therefore, IV xenobiotic metabolizers (G2 members) may be considered as drivers of the xenobiotic metabolizing enzyme repertoire in a gut microbiome. The observed increase in abundances of HV xenobiotic metabolizers (members of G3) with increase in age of individuals is likely to be due to the accumulation of specialized xenobiotics metabolizing bacteria with time and exposure to xenobiotics.

The reduced contribution of various genera at age extremities suggests a certain degree of disequilibrium in the diversity as well as the contribution of various genera in xenobiotic metabolism. A likely reason for this observation could be that while children have unstable microbial composition (still under development) and thus have low microbial diversity, the microbial community in older people is dysbiotic due to onset of senescence.

The enzyme repertoire as well as the microbes contributing these enzymes showed distinct distribution between sample groups with specific age and geography dependent trends. This suggested that not only the abundance of microbes harboring the enzymes of interest, but also the variety of enzymes in those microbes play a role in determining xenobiotic metabolizing capabilities of a metagenome.

Analyses of abundance of enzymes showed that well established DMEs contributed only 30–40% of total xenobiotic metabolizing enzyme repertoire in each microbiome. The results further indicated role specific clustering of the enzyme types, irrespective of sample groups. Since, the well studied DMEs show only partial contribution to the gut xenobiotic metabolizing repertoire (30–40%), there exists potential to explore the roles of other enzymes (forming rest 60%) in the context of drug metabolism by human microbiome.

A potential issue associated with the current study is the likely presence of cohort- and study-specific biases that could skew the data. Such biases have been discussed in earlier studies **[46, 58, 59]**. In order to reduce additional biases related to analyses, contigs generated by the studies were used and same approaches (softwares and tools) were applied to all the datasets (see methods). Cohort and sample specific biases have also been addressed in this manuscript. Also, the number of samples in different sample groups was not uniform. Thus, to tackle this issue, non parametric central tendency values for each sample group were analyzed. Additionally, in the current study, all the individuals in the French and Italian cohorts belonged to the elderly age group (i.e. greater than 60 years). Similarly, the Indian cohort consisted of the gut microbiomes from 20 children. However, two distinct patterns observed in the current study indicate that the age-specific bias of a few cohorts did not significantly affect the results of this study. The first was the progressive increase of higher xenobiotic metabolism capability with age (in spite of the biases in the nationalities of the individuals belonging to the different age-groups). The second is the similarity between the compositions of the xenobiotic metabolizing enzyme repertoire across the Asian nationalities (in spite of the distinctly lower age of the Indian individuals). Thus, although the age-specific biases may skew the data, such effects are not observed to significantly affect the results obtained herein.

Another aspect which remains critical is the annotation of EC numbers and KO ids to the sequences of the seed database and identified homologues. This is due to the fact that BLAST searches may identify remote homologues which may not perform the desired functions. To ensure minimal errors in this aspect, we have used accurately curatedand widely accepted public databases (like KEGG and NCBI) to populate the reference database. Also, stringent cut offs were used during BLAST searches (see methods) in order to ensure that the homologues identified in the metagenomes are annotated correctly. Further validation was performed to ensure the accuracy of BLAST parameters while assigning annotation of EC numbers on the basis of homology (described in S1 Text). Based on the results, identity percentage between sequences belonging to same EC numbers considered were above 40% at all taxonomic levels. On the other hand, identity percentage of maximum blast hits between sequences of different EC numbers lied below 40%. Thus, an identity cut off of > 40%, apart from stringent subject coverage, was used to assign enzyme function to the identified metagenomic homologues.

Experimental studies, characterizing the xenobiotic degrading capabilities in different bacteria, are currently limited. Therefore, apart from the current analyses on gut microbiomes, the aim of the genome-wide analyses (performed in this study) was to obtain a comprehensive profile of the occurrence likelihoods of the various xenobiotic degrading enzymes across all bacterial clades (which may or may not be associated with humans). Obtaining such a profile is helpful because xenobiotic degradation pathways not only have clinical importance with respect to drug bio-availability, but also have implications in bio-remediation as many of these pathways play a role in the de-toxification of pollutants like DDT, Atrazine, PAHs, etc. Using the information obtained from the current study, researchers working on a given xenobiotic degrading pathway(s) can now focus their investigations on specific clades of bacteria.

It is to be noted that metagenomic studies provide only a static overview of putative enzymes and pathways. To confirm the expression and the functional status of the same, additional experimental studies of the gut microbiota, using transcriptomics/metatranscriptomics, RNA-Seq analysis, meta-proteomics and metabolomics, are required. Such studies will not only give an idea about the gene expression, enzymatic activity and metabolic dynamics of the gut microbiome, but will also help in making the predictions more robust and accurate.

Another aspect that needs investigation is the role of mobile genetic elements in the transfer of xenobiotic metabolizing traits. Extensive use of xenobiotics in the form of pesticides and pharmaceuticals, introduces a selective survival pressure in the environment that may facilitate the spread of such elements across microbial species. Previous studies have identified and elaborated on the role of such mobile genetic elements in the transfer of xenobiotic metabolizing capabilities in bacteria **[60–61]**. Due to continuous exposure to xenobiotics, mainly in the form of pharmaceuticals, there is also a possibility of constant selective pressure maintained in the gut bacterial community. Due to this, the aspect of inter-cellular horizontal acquisition of such genes using mobile genetic elements and its role in providing additional survival advantage to the microbes cannot be ignored. As seen in a previously published report on gut microbiomes, enzymes responsible for conferring antibiotic resistance to bacteria were found to be associated with DNA sequences responsible in horizontal transfer of genes **[43]**. Microbiomic investigations of such elements, specifically with respect tothe transfer of such traits in the gut microbiota could provide a much more holistic and dynamic perspective on the xenobiotic metabolizing capabilities of the gut microbiome.

## Conclusion

The present study reports the first ever comprehensive analyses, with respect to xenobiotic metabolizing enzymes, on gut microbiomes of large cohorts. In contrast to earlier studies which adopted culture-based methodologies for deriving inferences about drug microbe interactions, the present study employs a combination of genome-wide variation and metagenome-wide homologue detection approach for comprehensively profiling (*in silico*) the xenobiotic metabolizing capabilities of various microbes. The study also puts forward interesting insights regarding variations observed in these capabilities (of gut microbiomes) with respect to age and geography.

The results reported in the present study also corroborate previously known pharmacokinetic variations (such as ethnic and age related drug efficacy). Overall, the trends observed (from the present study) suggest a direct role of intestinal microbes in pharmacokinetic variations. The study stratifies microbes into three broad groups based on their xenobiotic metabolizing capabilities. Once experimentally validated, these insights can be potentially employed for devising/designing appropriate (personalized) therapeutic regimens. Furthermore, a combination of gut microbiome based profiles of xenobiotic metabolizing enzymes (as presented in the current study) and wide association studies (along with information from clinical trials) is expected to help in creation of a comprehensive data/knowledge base. This information repository has the potential for predicting the pharmacokinetic parameters and their variations in each individual, with better accuracy. This in turn, is likely to help addressing issues like drug bioavailability, drug overdose, side effects and adverse drug reactions which may further help in advancing the field of predictive medicine and pharmacology.

## Methods

### Database creation of xenobiotic metabolizing enzymes present in various microbial genomes

KEGG Orthology (KO) and Enzyme Commission (EC) numbers of enzymes belonging to various xenobiotics biodegradation and metabolism pathways in KEGG were fetched from KEGG (www.genome.jp/kegg/). A total of 284 KO IDs and 300 EC numbers corresponding to 32 pathways were obtained. The details of these enzymes, in terms of KO IDs, EC numbers, along with their corresponding xenobiotic degradation pathways, are provided in S12 Table. The NCBI GI numbers of the respective EC numbers and KO IDs were identified and sequences were fetched from NCBI-NR (a non-redundant protein sequence database from NCBI) database. Furthermore, the NCBI-NR database was also searched for sequences containing the keyword 'Cytochrome P450'. This created a database of 1,62,054 sequences from 850 microbial genera. In addition, to identify sequences corresponding more xenobiotic metabolizing enzymes, the database was further enriched by performing a BLASTp search of the constituent sequences against the NCBI-NR database (with thresholds of Identity > = 90%, Subject coverage > = 90% and E-Value < 1e-5). Best hits were identified using bit score parameters. The homologues obtained were assigned the same EC and KO annotation as that of the matched sequences in their respective databases. Additionally to check for errors and extent of divergence, two tests were performed (S1 Text).

### Classification of bacterial genera based on genome scale profiling of xenobiotic metabolizing enzymes

Microbial taxa affiliations of xenobiotic metabolizing enzyme sequences present in the final database were considered for genome scale profiling at the genus level. Based on the occurrences of various KO IDs of the sequences in each genomes (belonging to different genera represented in the database), an abundance table consisting of different genera and the occurrences of each EC/KO was created. To remove database bias, the occurrences were normalized by total number of unique genomes of each genus. Using the abundance table, Principle Coordinate analysis was performed using the protocol used earlier by Arumugam *et al*. **[47]**, based on which clusters of bacterial genera having similar profiles of xenobiotic metabolizing enzymes (belonging to various pathways) were identified. To identify the optimum number of clusters, we calculated Calinski-Harabasz (CH) Index (S18 Fig). The cluster number with highest CH index (N = 3) was considered as the optimal one. For cluster validation, we used mean silhouette score as a part of global assessment of the clusters. A positive mean score of 0.247, which along with CH index analysis suggested robust clustering of the microbial genera, into 3 distinct groups, based on enzyme abundances.

### Profiling homologues of xenobiotics metabolizing enzymes in gut microbiomes of individuals from various geographies

#### Datasets used

Gut metagenomic sequences (available as contigs) from 27 individuals belonging to Italian, French and Japanese, previously analyzed by Arumugam *et*. *al*. **[47]**, were downloaded from *http://www.bork.embl.de/Docu/Arumugam_et_al_2011/downloads.html*. Additionally, contigs of metagenomic sequences corresponding to 116 European (81 Danish and 35 Spanish) individuals, previously analyzed by Qin *et al*. **[42]**, were downloaded from *http://gutmeta.genomics.org.cn/*. Further, contigs corresponding to 90 gut metagenomes of American individuals, sequenced as part of the Human Microbiome Project **[62]**, were downloaded from HMP-DACC website (*http://www.hmpdacc.org/HMASM/*). Apart from this, contigs from gut metagenomic datasets from 20 Indian children of varying nutritional status, previously analyzed by Ghosh *et al*.**[41],** were downloaded from *http://www.ncbi.nlm.nih.gov/Traces/sra*. In addition, a set of gut metagenomic contigs from 144 Chinese individuals, previously analyzed by Qin *et al*. **[38],** were also downloaded fromhttp://gigadb.org/dataset/100036. Details on all the datasets are listed in S13 Table.

The basis for using metagenomic contigs (instead of sequence reads) for this analysis is as follows. Firstly, contigs, by virtue of their longer lengths, facilitate the detection of full length genes, enhancing the reliability of the results obtained. Secondly, it is likely to remove the biases associated with using sequence data from multiple studies. Different sequencing platforms and DNA extraction methods may have a possible impact on the comparative analysis of data from multiple studies. The primary issue of comparing datasets obtained using different sequencing platforms is the variations in the read lengths of the sequences. These are likely to have artefactual effects on the taxonomic and functional profiles obtained for the analyzed datasets. By ensuring similar sequence lengths across all datasets, the use of longer high quality contigs is likely to bypass these limitations. For example, in the current analysis, metagenomic contigs of most of the datasets were observed to have lengths greater than 1000 base pairs (S19 Fig). However, biases could also originate due to differences in the DNA extraction method and other experimental protocols. To ensure that such biases were not present in the datasets in the current study, taxonomic classification of the contigs in each of the samples (in each dataset) were first obtained using DiScRIBinATE **[63]**. The taxonomic profiles of each sample (in the different datasets) were then compared by PCA using STAMP **[64]**. It was observed that the samples from different datasets clustered together with no significant biases in any of the components (S19 Fig).

#### Identification of homologues of xenobiotic metabolizing enzymes in gut microbiomes

Homologues of xenobiotic metabolizing enzymes were identified in the gut metagenomes by performing BLASTx searches of the metagenomic contigs against the xenobiotic metabolizing enzyme database (created as described earlier). Preliminary filtering of the BLASTx involved a sequence identity cut-off of >40%, subject coverage cut-off of >75% and e-value cut-off of <1e-5. Alignment with the highest bit score was considered as best hit. The identity cut off of 40% was used because it has been shown earlier that 90% accuracy can be achieved while assigning full EC numbers when sequence identity is more than 40% **[65–67]**. Additionally, two different validation tests were performed to specifically investigate the pattern of sequence divergence observed within the families containing the xenobiotic degrading enzymes and to further check whether the above criteria is stringent enough (so as not to result in false positive associations). The details of these tests have been provided in S1 Text.

#### Abundances of xenobiotic metabolizing enzymes and analysis of microbial community across gut microbiomes

The overall abundance of each xenobiotic metabolizing enzyme in each gut microbiome was calculated as total number of detected genes corresponding to that enzyme per 5 million base pairs of the respective metagenome. The calculated abundances of all xenobiotic metabolizing enzymes in all gut microbiomes were compared for identifying inter-individual, inter/intra-regional and inter-age group variations of the xenobiotic metabolizing enzymes.

In order to investigate the trends in the overall abundances of various xenobiotic metabolizing enzymes, analyses of the microbial communities in the gut microbiomes was performed, with specific focus on the microbial genera harboring various xenobiotic metabolizing enzymes. For this purpose, for each metagenome, the taxa affiliations of all contigs, identified to harbor xenobiotic metabolizing enzymes (using the BLAST search), were first obtained using the DiScRIBinATE method **[63]**. This method, along with a few other methods **[68–69],** utilizes BLAST alignment parameter based thresholds for the accurate taxonomic affiliation of metagenomic sequences/contigs. A recent comparative evaluation of 38 taxonomic classification methods observed DiScRIBinATE to have the highest sensitivity and specificity values in typical metagenomic scenarios, where in a majority of sequences originate from hitherto uncharacterized genomes **[70]**.

Subsequently, based on the taxa assignments for each metagenome, the abundance of various xenobiotic metabolizing enzymes at the taxa level of genera was obtained and was used for all further analyses. The contribution of each genera to the xenobiotic metabolism gene repertoire in each microbiome (i.e. specific abundance) was then obtained by calculating number of contigs (in the corresponding metagenome) showing hits against genes corresponding to xenobiotic metabolizing enzymes, assigned to a particular genera, normalized by total number of contigs in the metagenome. Specific abundance represents the contribution of a microbial genus to the xenobiotic metabolizing enzymes repertoire. On the other hand, the calculated overall taxonomic abundances (overall abundance) represent contributions of a particular microbial genus in the whole microbiome. Both, specific and overall abundances were used for further analyses.

#### Statistical tests for comparison of various groups of gut microbiomes

All multiple group statistical comparisons were performed using Kruskal-Walis H test, followed by multiple test corrections using Benjamini-Hochberg FDR correction wherever applicable. Statistically significant differences with corrected P-value < 0.05 are reported.

### Data Acquisition

We would like to note that no human subjects were used during the course of this study. All human microbiomic sequence data, used as part of this study, were sequenced as part of previous sequencing initiatives and were publicly available. These data were downloaded from the respective repositories as mentioned in the Methods section and re-analyzed in the current study.

## Supporting Information

S1 Fig Variation of abundance of selected drug modifier classes (DMEs) in each of the microbial groups. (PDF)

S2 Fig Variation of Specific abundance (based on selected drug modifying enzyme classes) of microbial genera across regions. (JPG)

S3 Fig Variation of enzyme abundance across regional cohorts. (JPG)

S4 Fig Heat map showing variation of cumulative abundances of the drug modifying enzyme classes (DMEs), in different regional cohorts. These enzymes included cytochrome P450s (CYP), monoamine oxidase (MO), epoxide hydrolase (EH), alcohol dehydrogenase (ADH), aldehyde dehydrogenase (ALDH), thiopurine methyltransferase (TPMT), N-acetyl transferase (NAT) and glutathione S-transferase (GST).
(JPG)

S5 Fig Variation of xenobiotic metabolizing gene diversity with gene abundance at regional and age group levels. (TIF)

S6 Fig Variation of Shannon index (for both overall microbiome and xenobiotic metabolizing microbiome) with region and age. (TIF)

S7 Fig Variation of Specific abundance (based on selected DME classes) of microbial genera across age groups. (JPG)

S8 Fig Variation of enzyme abundance across age groups. (JPG)

S9 Fig Heat map showing variation of cumulative abundances of the drug modifying enzyme classes (DMEs), in different age groups. These enzymes included cytochrome P450s (CYP), monoamine oxidase (MO), epoxide hydrolase (EH), alcohol dehydrogenase (ADH), aldehyde dehydrogenase (ALDH), thiopurine methyltransferase (TPMT), N-acetyl transferase (NAT) and glutathione S-transferase (GST).
(JPG)

S10 Fig Cosine distance comparison as a measure of dissimilarity in enzyme harboured by microbial genera in each region. (JPG)

S11 Fig Clusters obtained from PCoA performed using abundance of each xenobiotic metabolizing enzyme in the metagenomic samples. (JPG)

S12 Fig Heat map showing variation of cumulative abundances of members of each enzyme cluster in different sample groups. (JPG)

S13 Fig Variation of COG abundance in each enzyme cluster. (JPG)

S14 Fig Venn diagram showing number of COGs unique or shared by the enzyme clusters. (JPG)

S15 Fig Variation of COG functional categories abundance in each cluster. (JPG)

S16 Fig Variation and distribution of each COG functional category in each enzyme cluster. (JPG)

S17 Fig Propensities of family to belong to the three functional categories, G1 (Least versatile xenobiotic metabolizers), G2 (Intermediately versatile xenobiotic metabolizers) and G3 (Highly versatile xenobiotic metabolizers). (PDF)

S18 Fig Variation of Calinski-Harabasz (CH) Index with number of clusters. (TIFF)

S19 Fig **(A)** Distribution of the length of contigs in samples belonging to different geographic regions. **(B)** Principal component analysis (PCA) of genera abundances in metagenomic samples (Study1: JP_AD, JP_CH; Study2: Chinese; Study3: Danish, Spanish, French and Italian; Study4: Indian; Study5: American). Statistical analyses (performed using Kruskal Wallis H-test, with Benjamini-Hochberg Correction indicated no significant variations in the different components across the samples belonging to any of the regions (PC1 < 0.784; PC2 < 0.784; PC3 < 0.764).
(TIF)

S1 Table List of regions included in the study and corresponding age groups represented. (PDF)

S2 Table Presence or absence of EC numbers and KO IDs corresponding to the xenobiotic metabolizing enzymes in genera present in the database along with their respective group affiliations and number of proteins present in them. (PDF)

S3 Table List of species and their corresponding genera, harbouring xenobiotic metabolizing enzymes. (XLSX)

S4 Table List of EC numbers, KO ids and COGs corresponding to selected DME classes. (XLSX)

S5 Table Abundances of xenobiotic metabolizing enzymes (with their EC numbers and KO IDs) in each of the metagenomic samples. (PDF)

S6 Table List of microbial genera and xenobiotic metabolizing enzymes harboured by them in each regional group. (XLSX)

S7 Table List of EC numbers and KO ids along with their corresponding cluster affiliations and COGs. (XLSX)

S8 Table List of COGs shared or unique to enzyme clusters. (XLSX)

S9 Table List of taxonomic families and their preferences for three different groups (Least versatile xenobiotic metabolizers or G1; Intermediately versatile xenobiotic metabolizers or G2; Highly versatile xenobiotic metabolizers or G3) along with their cluster affiliations. (PDF)

S10 Table List of clinically used drugs and their respective metabolic pathways. (PDF)

S11 Table List of selected drugs and bacterial genera capable of metabolizing them (information obtained from literature), along with their group affiliations. (PDF)

S12 Table List of EC numbers and KO IDs of xenobiotic metabolizing enzymes along with the pathways in which they function. (PDF)

S13 Table List of metagenomic samples along with their regional affiliations and age groups. (PDF)

S1 Text Details of validation tests performed to check reliability and accuracy of the alignment based filtering parameters. (PDF)
