# Supplementary figures and images for: Xenobiotic Metabolism and Gut Microbiomes
Source: PLoS One. 2016 Oct 3;11(10):e0163099. doi: 10.1371/journal.pone.0163099 (PMC5047465; doi:10.1371/journal.pone.0163099)

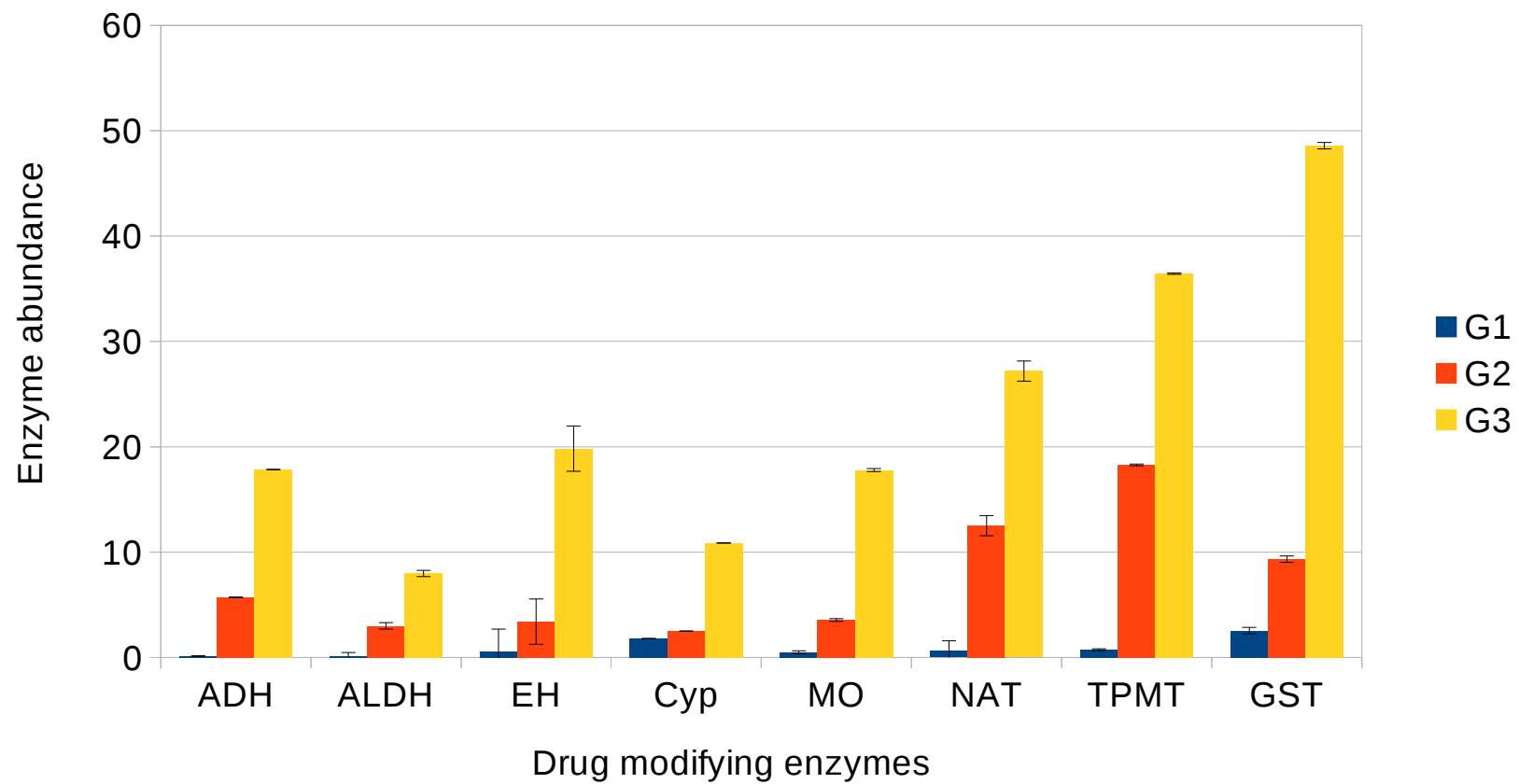

Supplement: S1 Fig — (PDF) [file pone.0163099.s001.pdf]

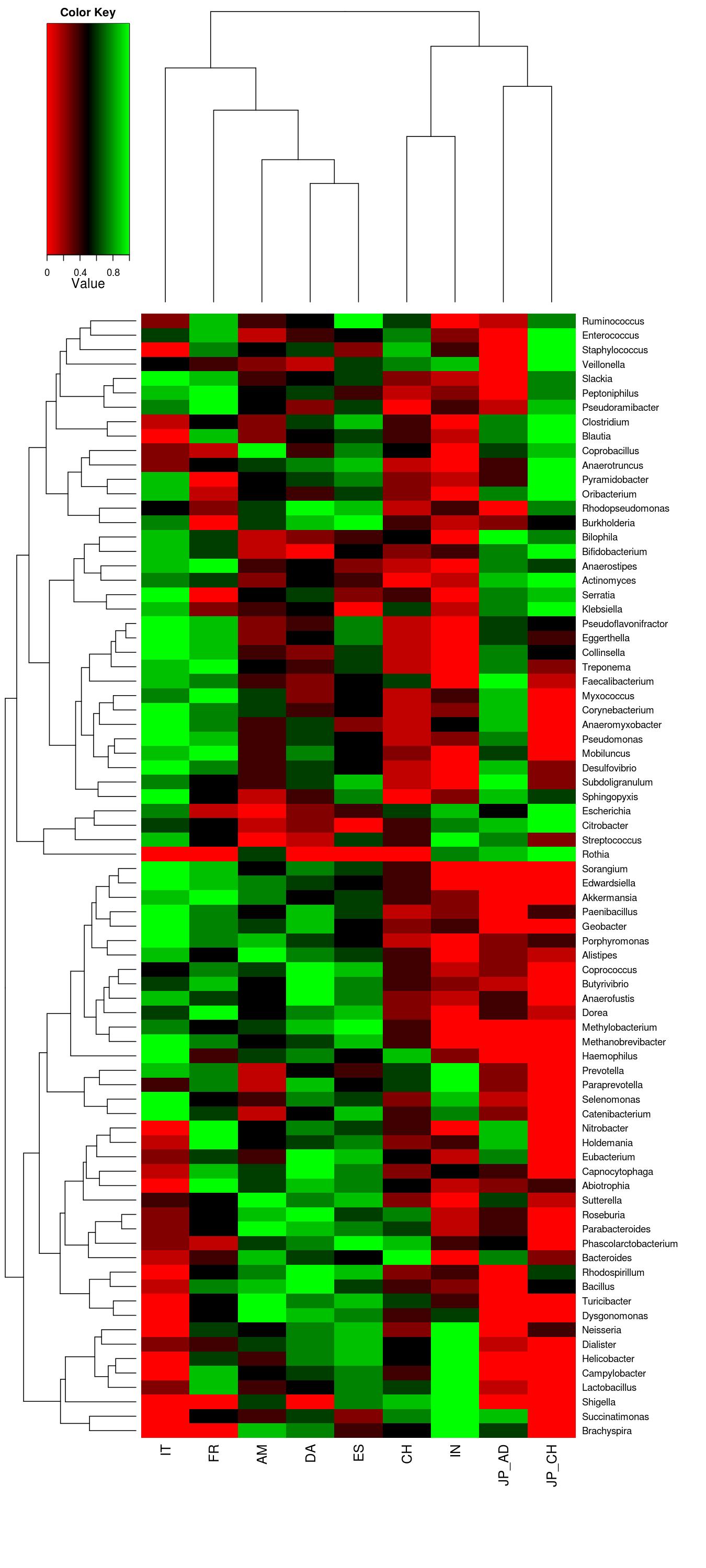

Supplement: S2 Fig — (JPG) [file pone.0163099.s002.jpg]

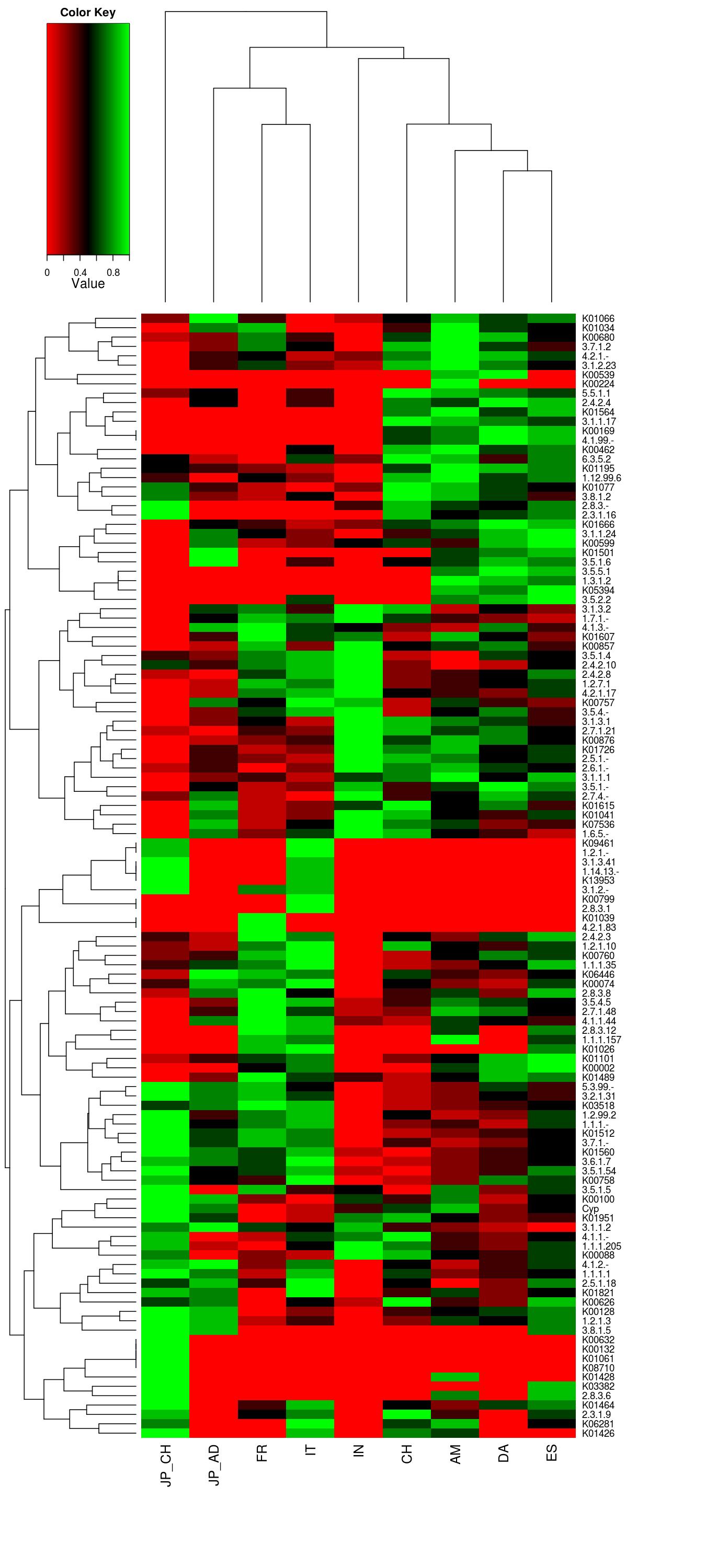

Supplement: S3 Fig — (JPG) [file pone.0163099.s003.jpg]

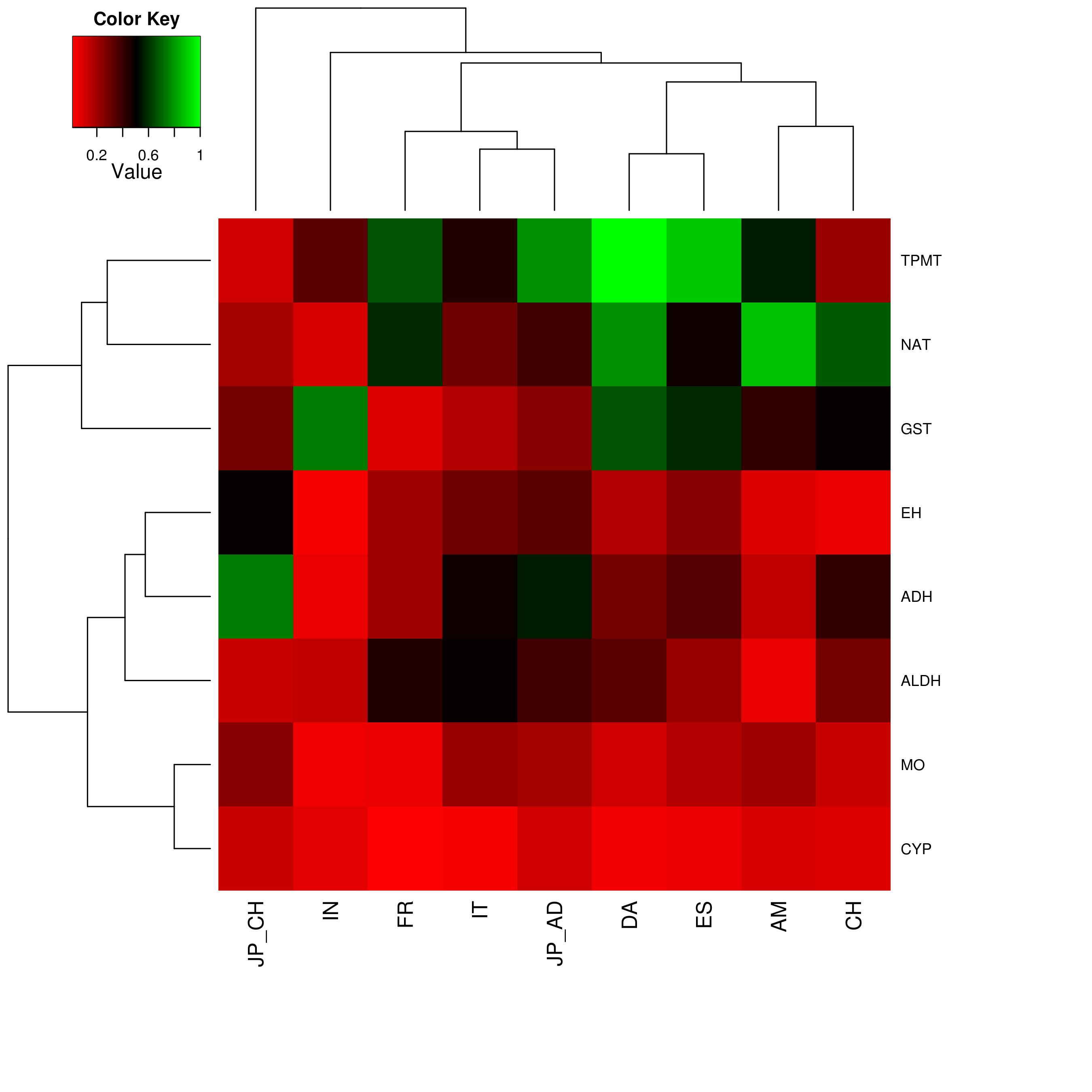

Supplement: S4 Fig — These enzymes included cytochrome P450s (CYP), monoamine oxidase (MO), epoxide hydrolase (EH), alcohol dehydrogenase (ADH), aldehyde dehydrogenase (ALDH), thiopurine methyltransferase (TPMT), N-acetyl transferase (NAT) and glutathione S-transferase (GST). (JPG) [file pone.0163099.s004.jpg]

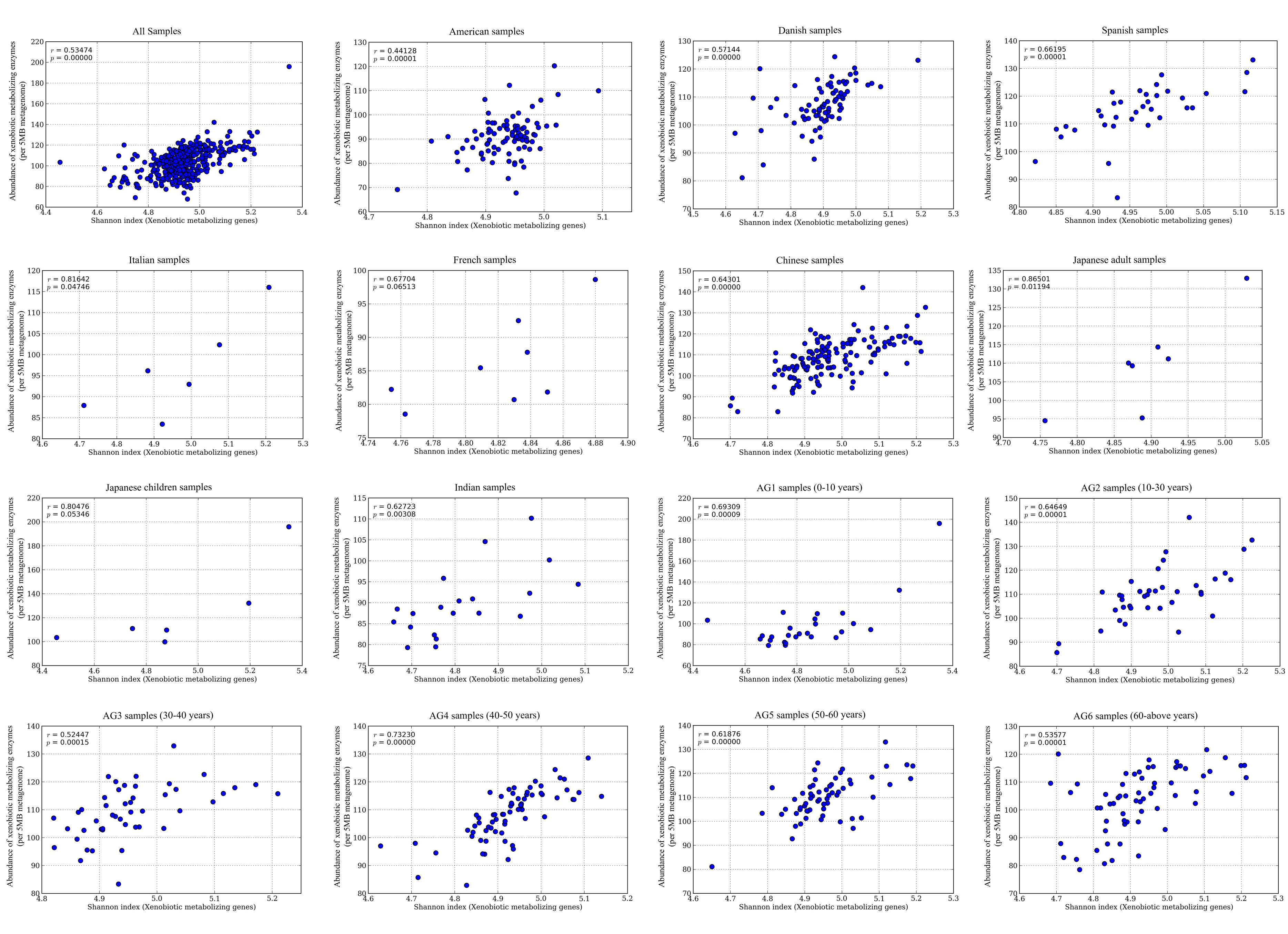

Supplement: S5 Fig — (TIF) [file pone.0163099.s005.tif]

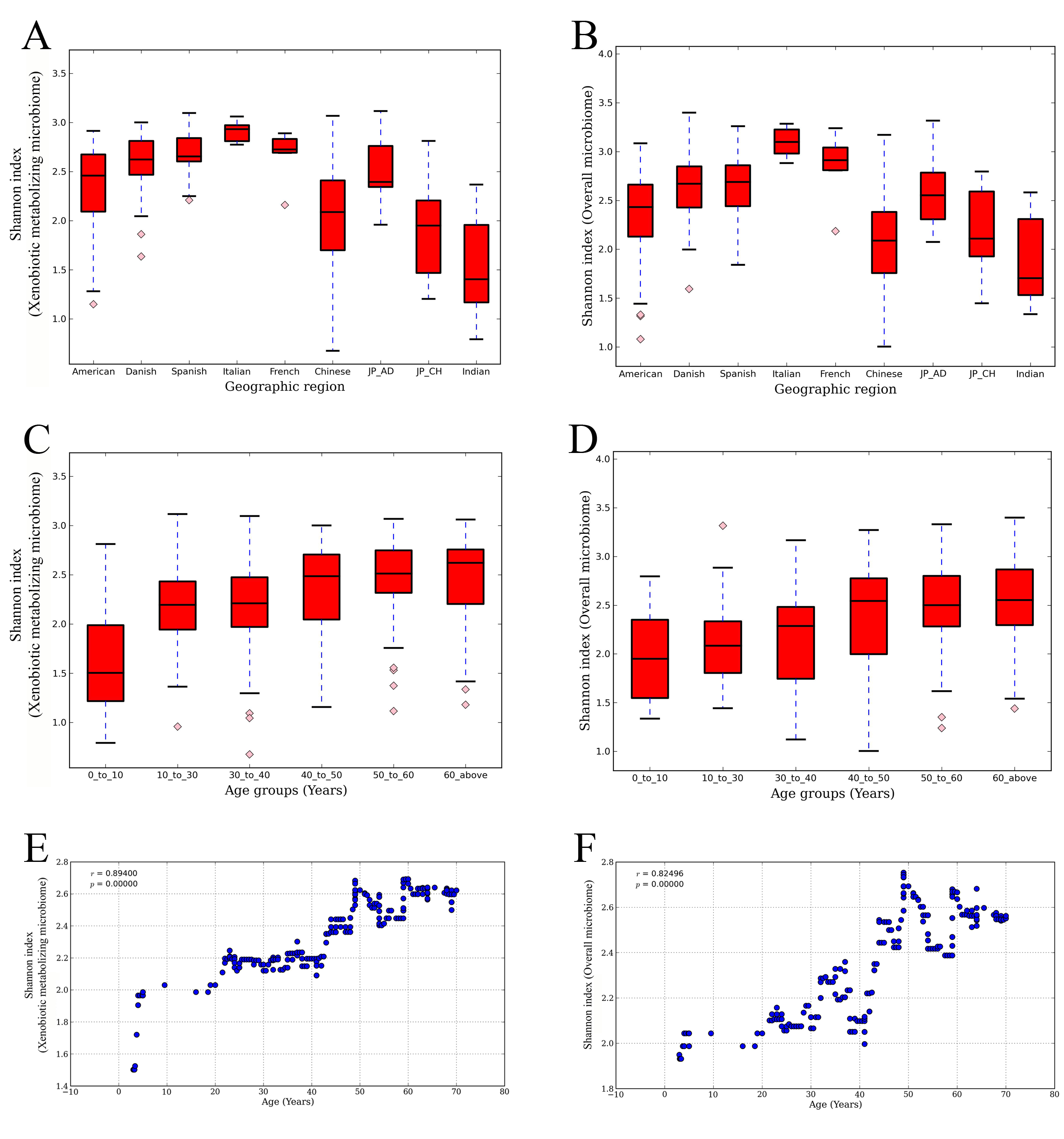

Supplement: S6 Fig — (TIF) [file pone.0163099.s006.tif]

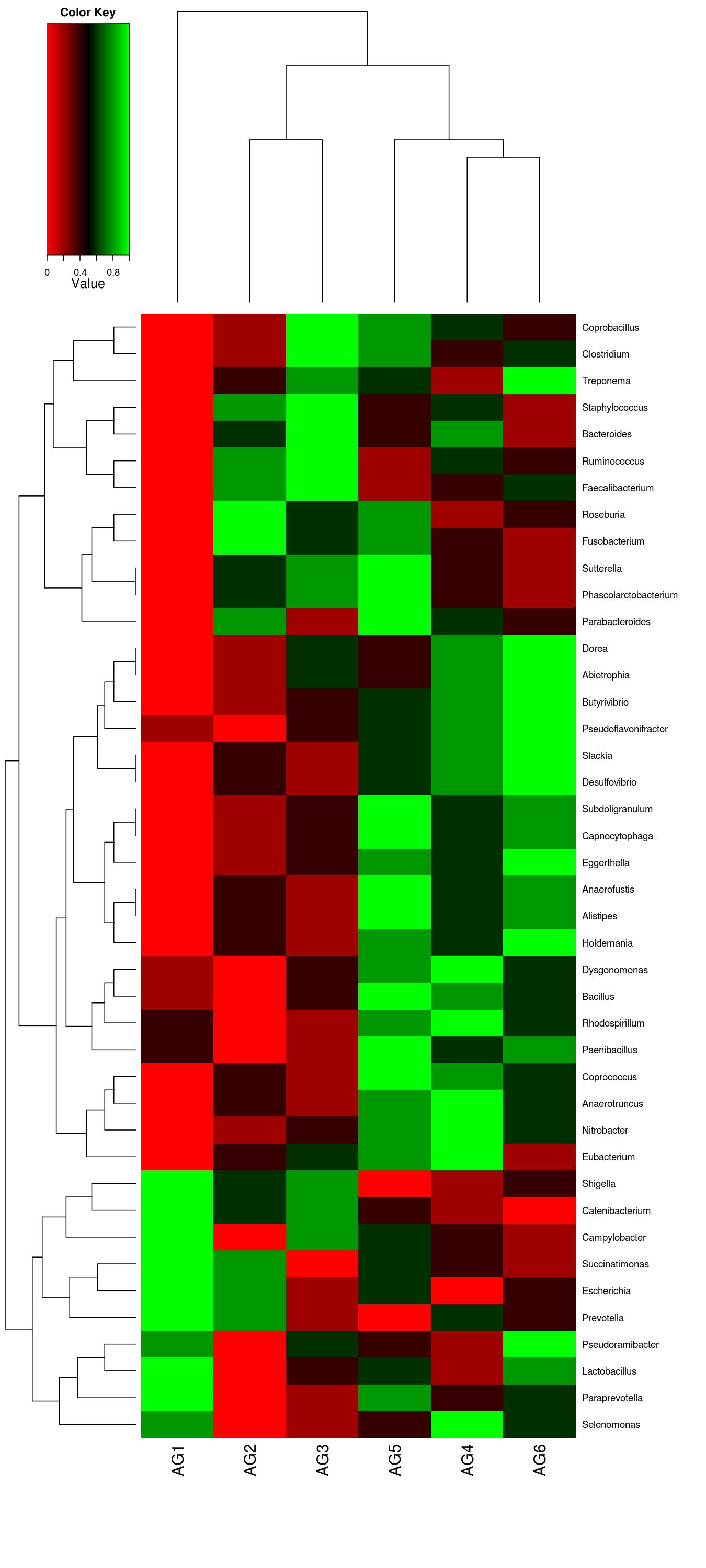

Supplement: S7 Fig — (JPG) [file pone.0163099.s007.jpg]

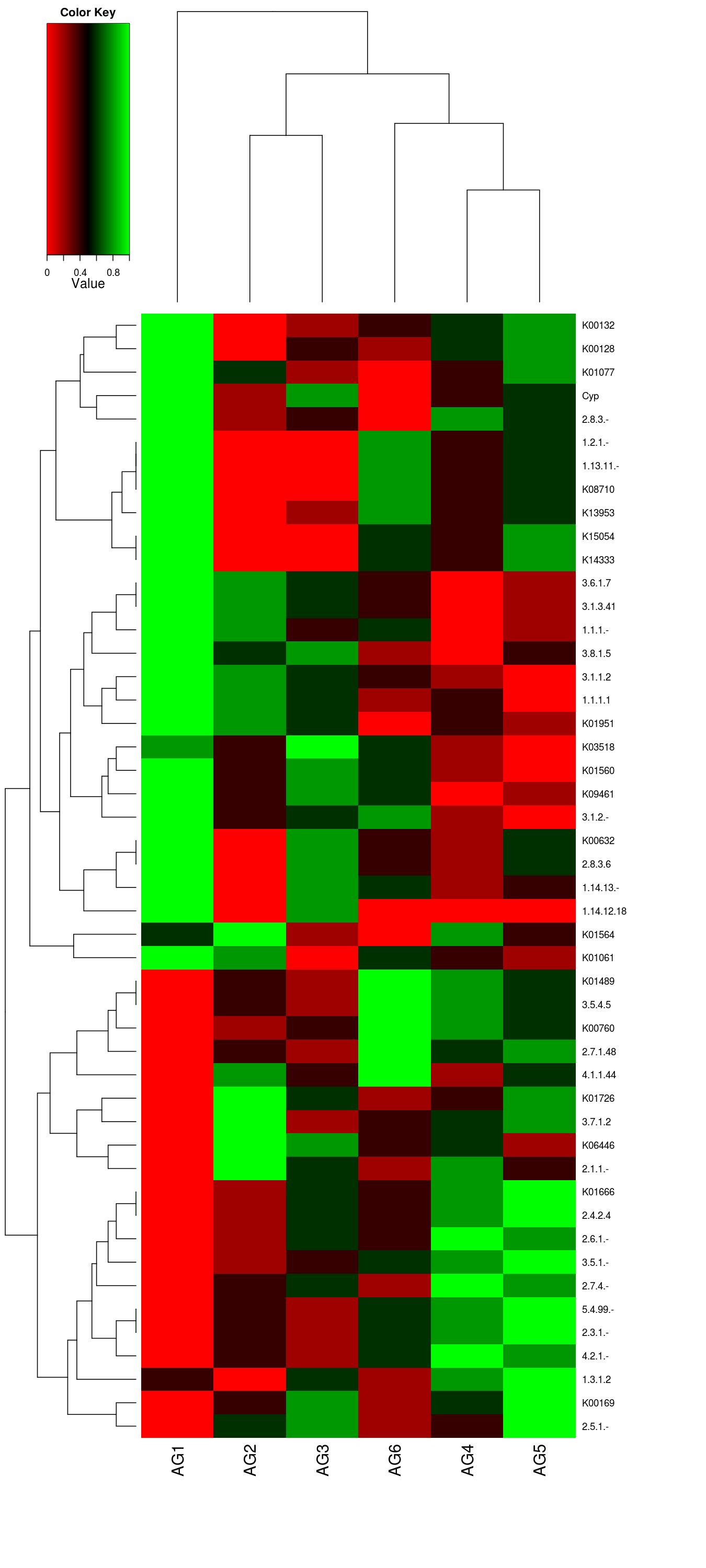

Supplement: S8 Fig — (JPG) [file pone.0163099.s008.jpg]

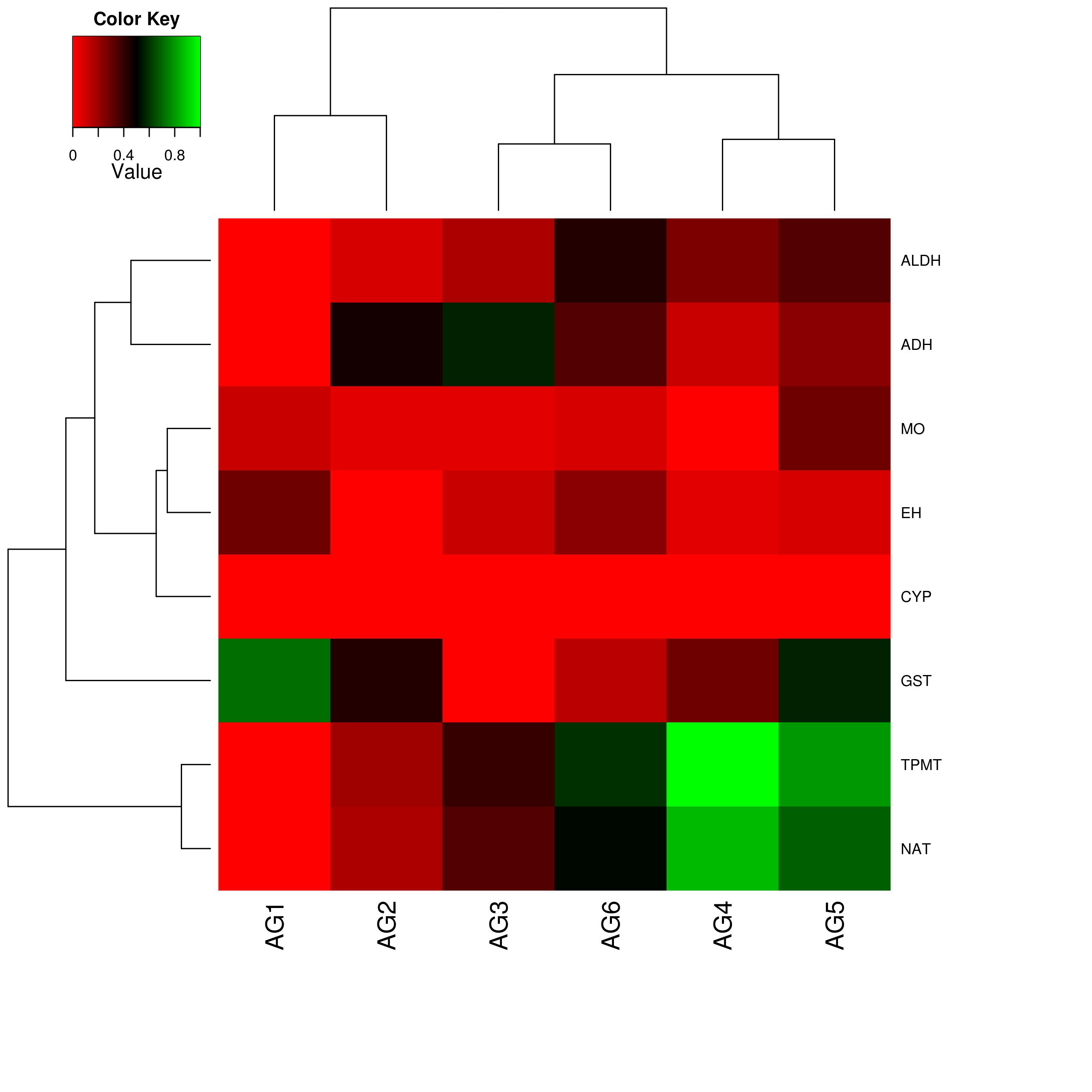

Supplement: S9 Fig — These enzymes included cytochrome P450s (CYP), monoamine oxidase (MO), epoxide hydrolase (EH), alcohol dehydrogenase (ADH), aldehyde dehydrogenase (ALDH), thiopurine methyltransferase (TPMT), N-acetyl transferase (NAT) and glutathione S-transferase (GST). (JPG) [file pone.0163099.s009.jpg]

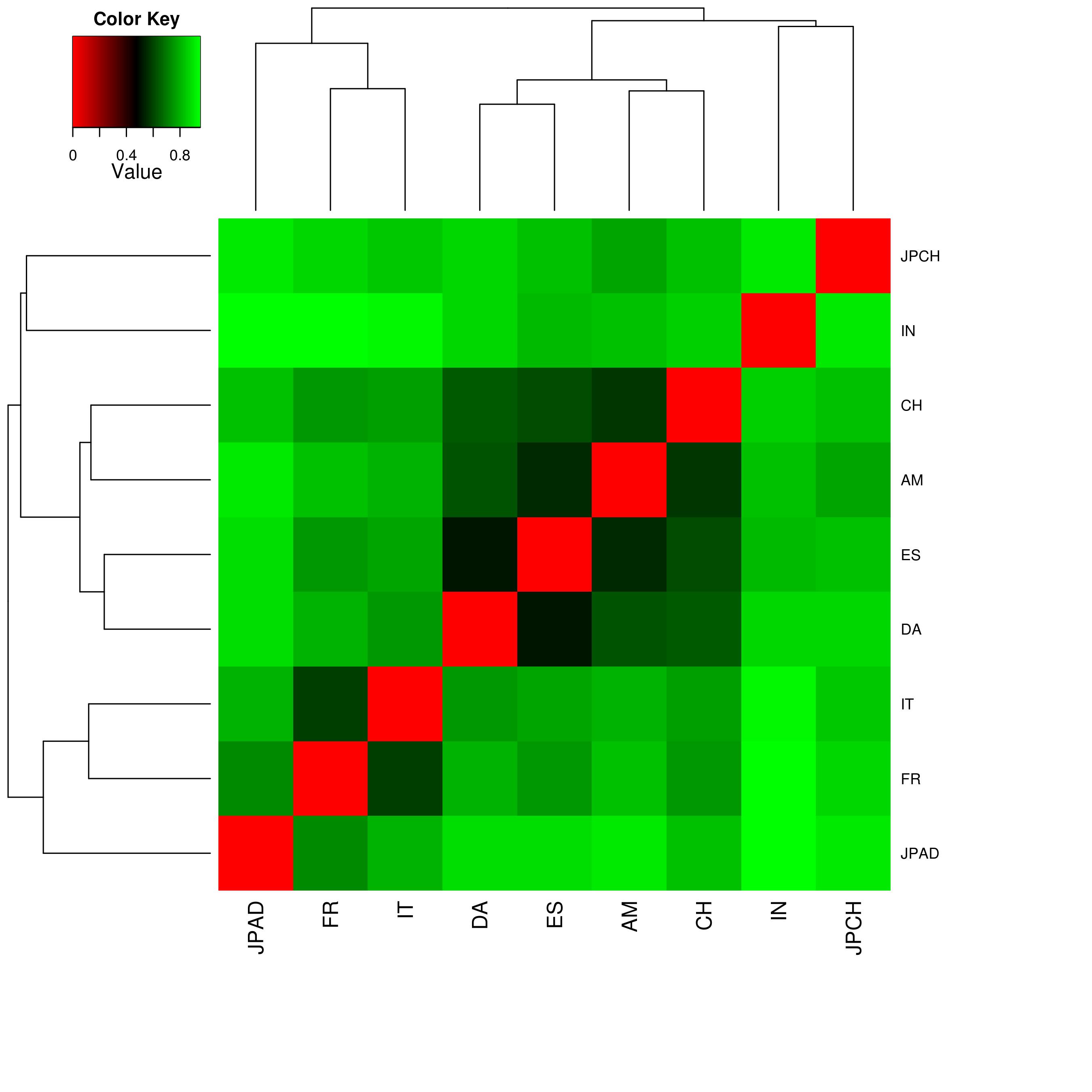

Supplement: S10 Fig — (JPG) [file pone.0163099.s010.jpg]

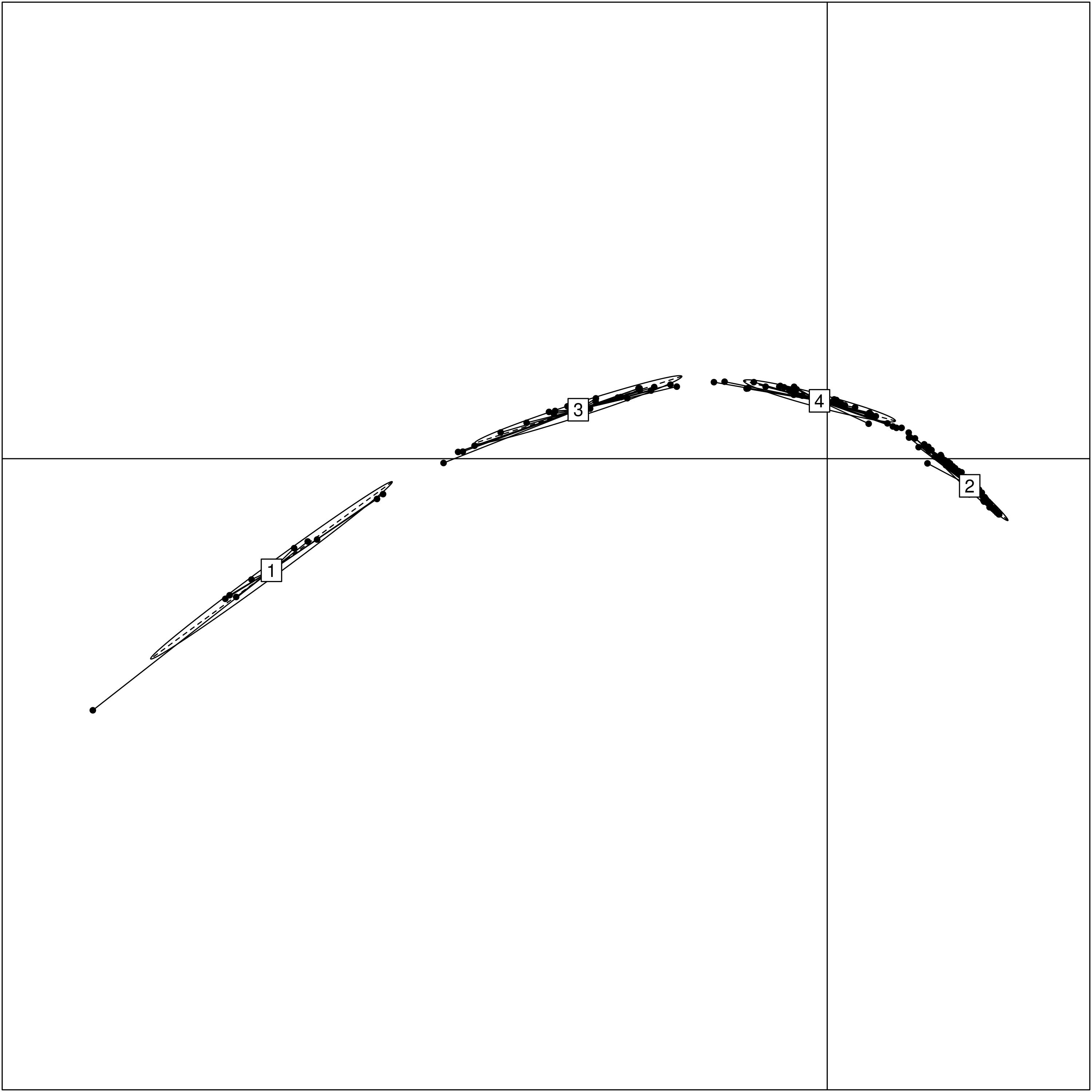

Supplement: S11 Fig — (JPG) [file pone.0163099.s011.jpg]

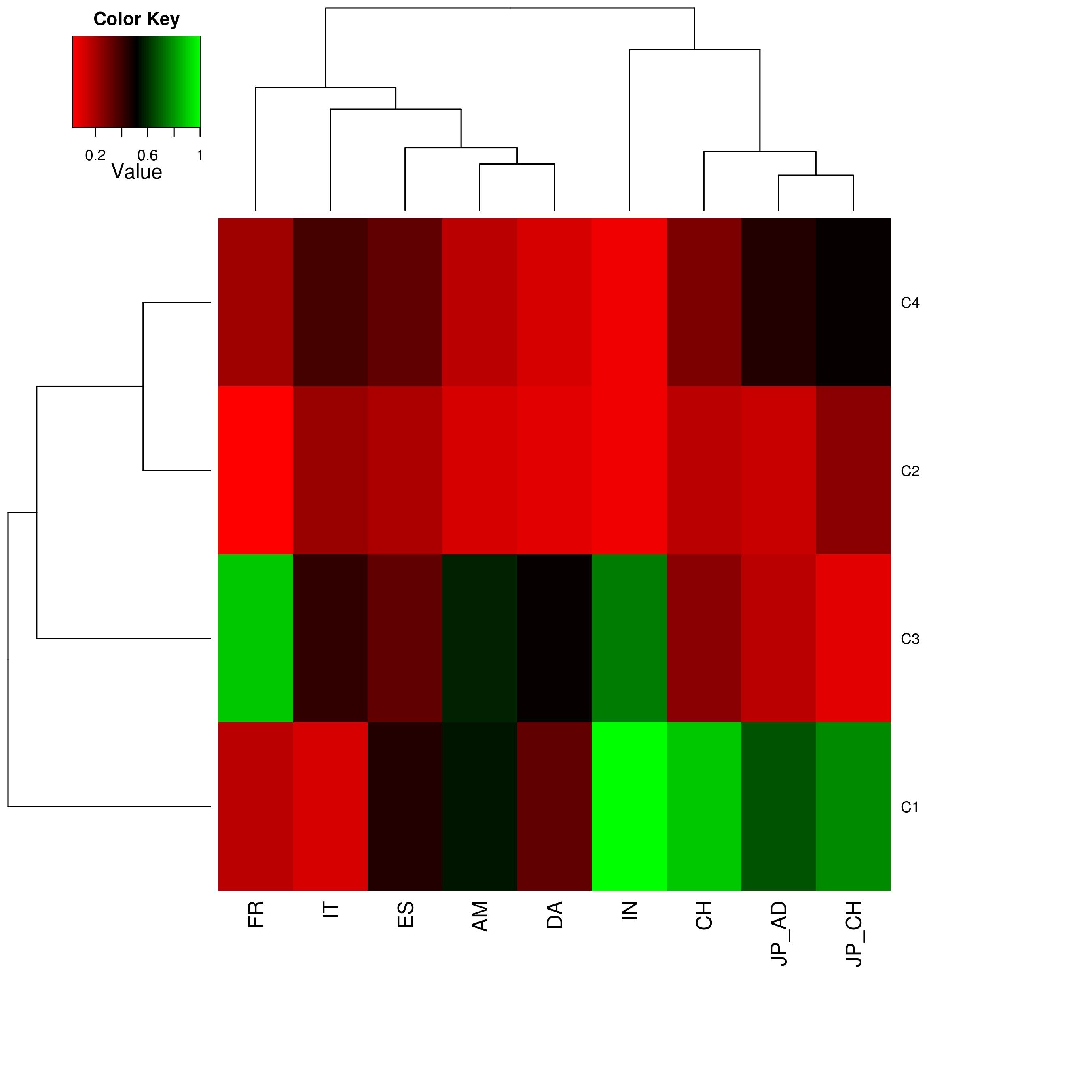

Supplement: S12 Fig — (JPG) [file pone.0163099.s012.jpg]

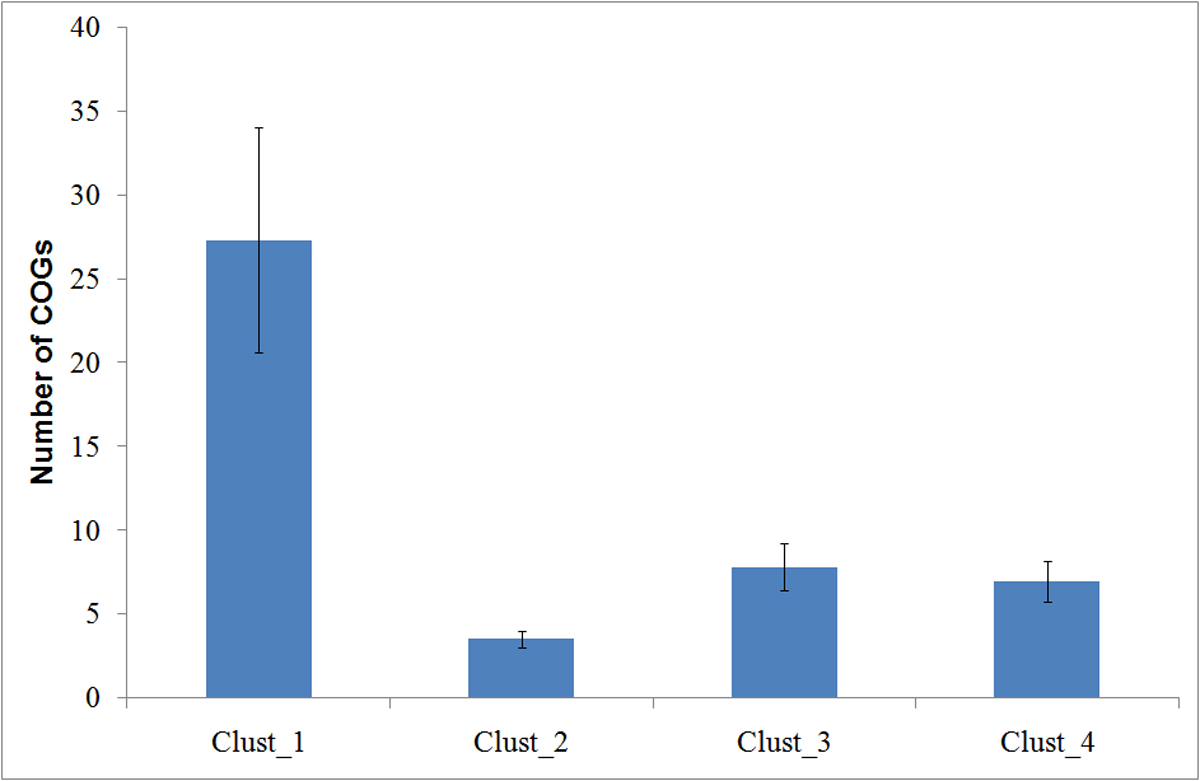

Supplement: S13 Fig — (JPG) [file pone.0163099.s013.jpg]

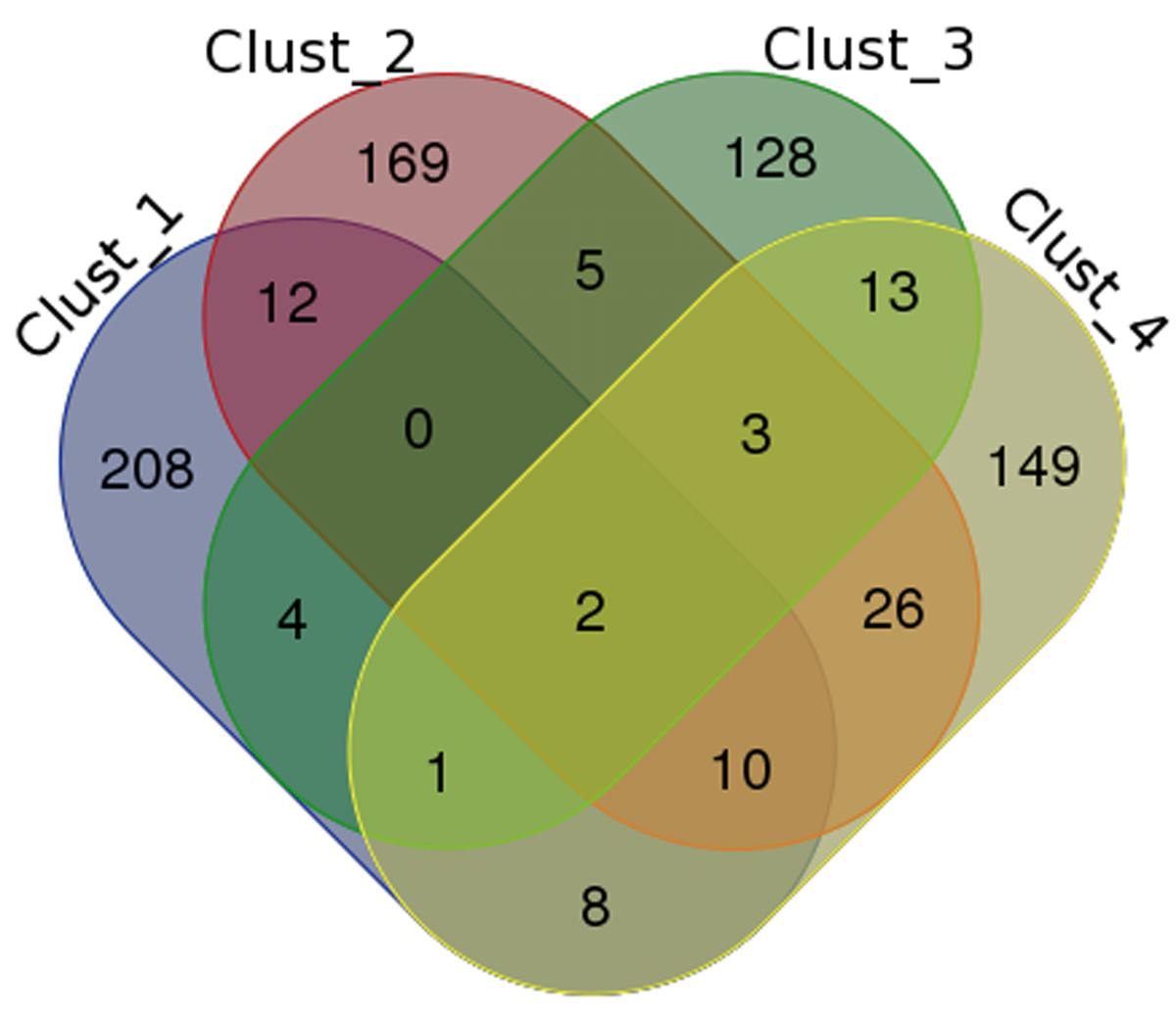

Supplement: S14 Fig — (JPG) [file pone.0163099.s014.jpg]

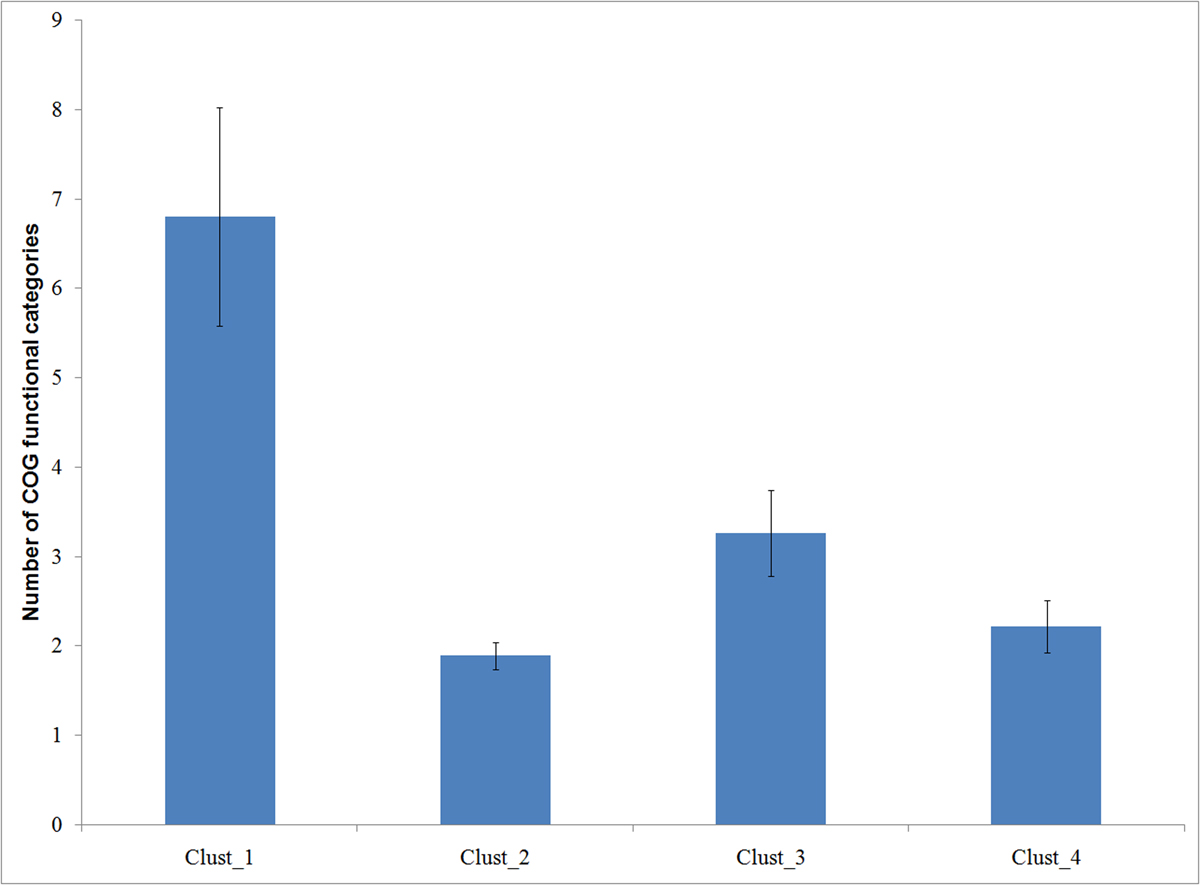

Supplement: S15 Fig — (JPG) [file pone.0163099.s015.jpg]

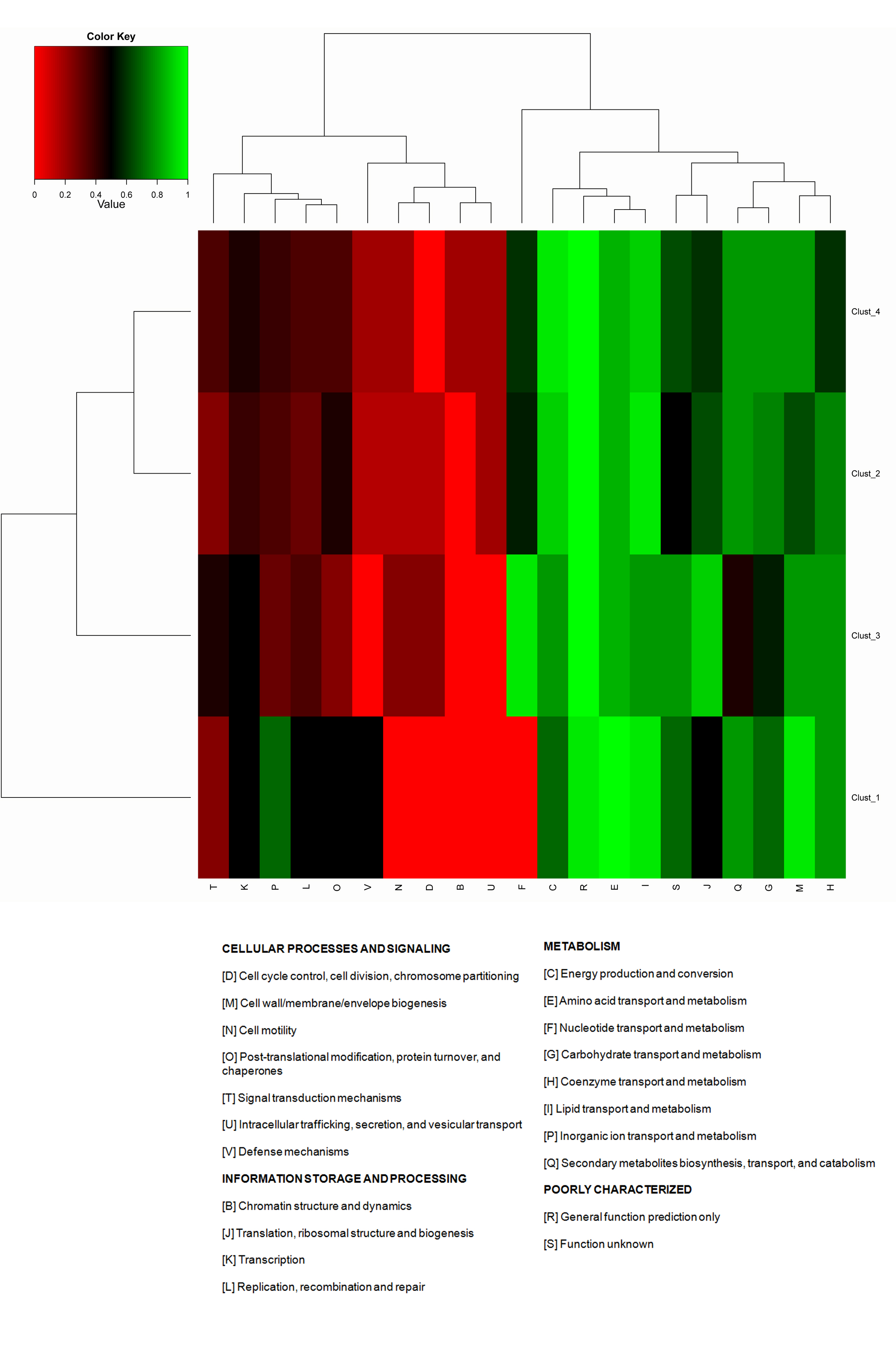

Supplement: S16 Fig — (JPG) [file pone.0163099.s016.jpg]

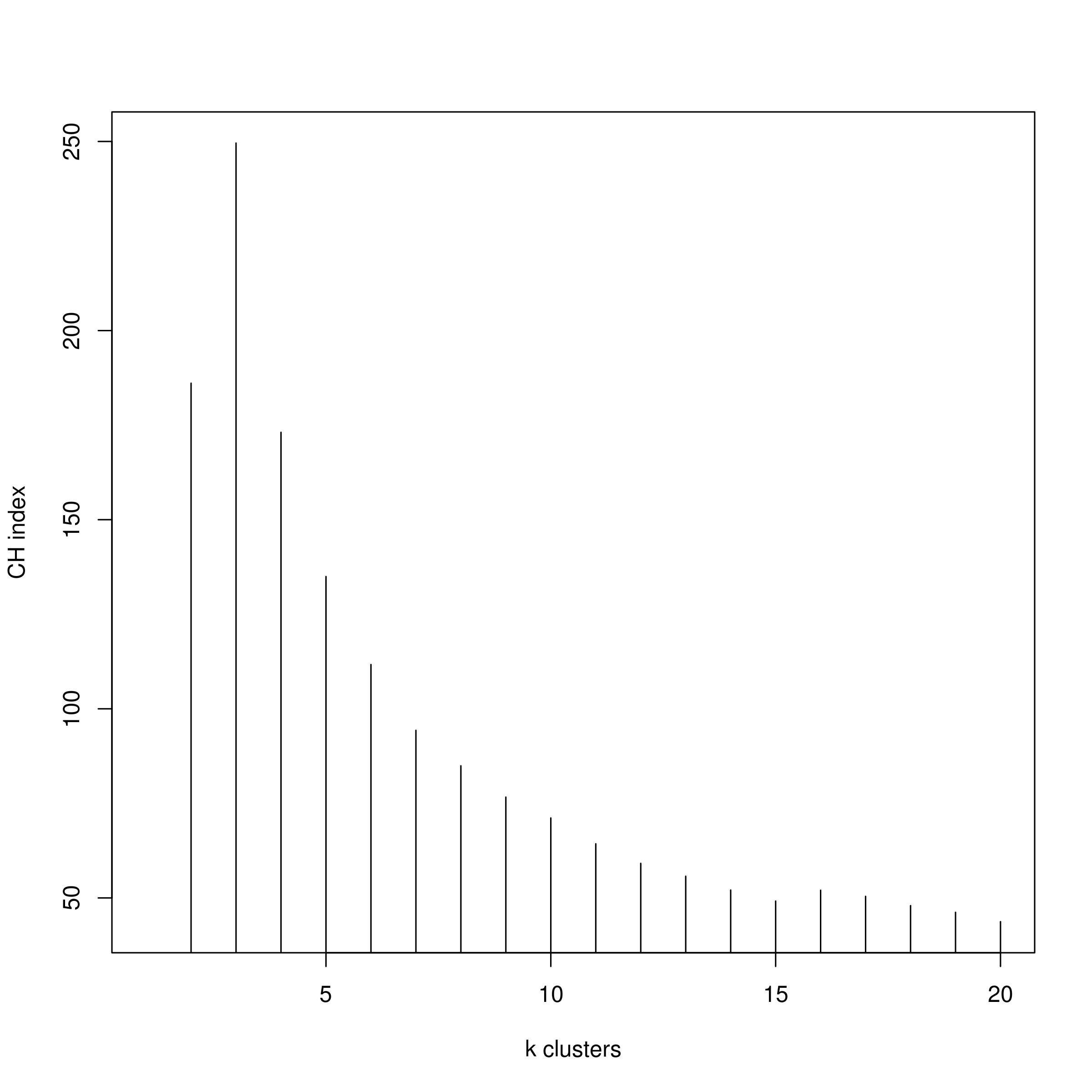

Supplement: S18 Fig — (TIFF) [file pone.0163099.s018.tiff]

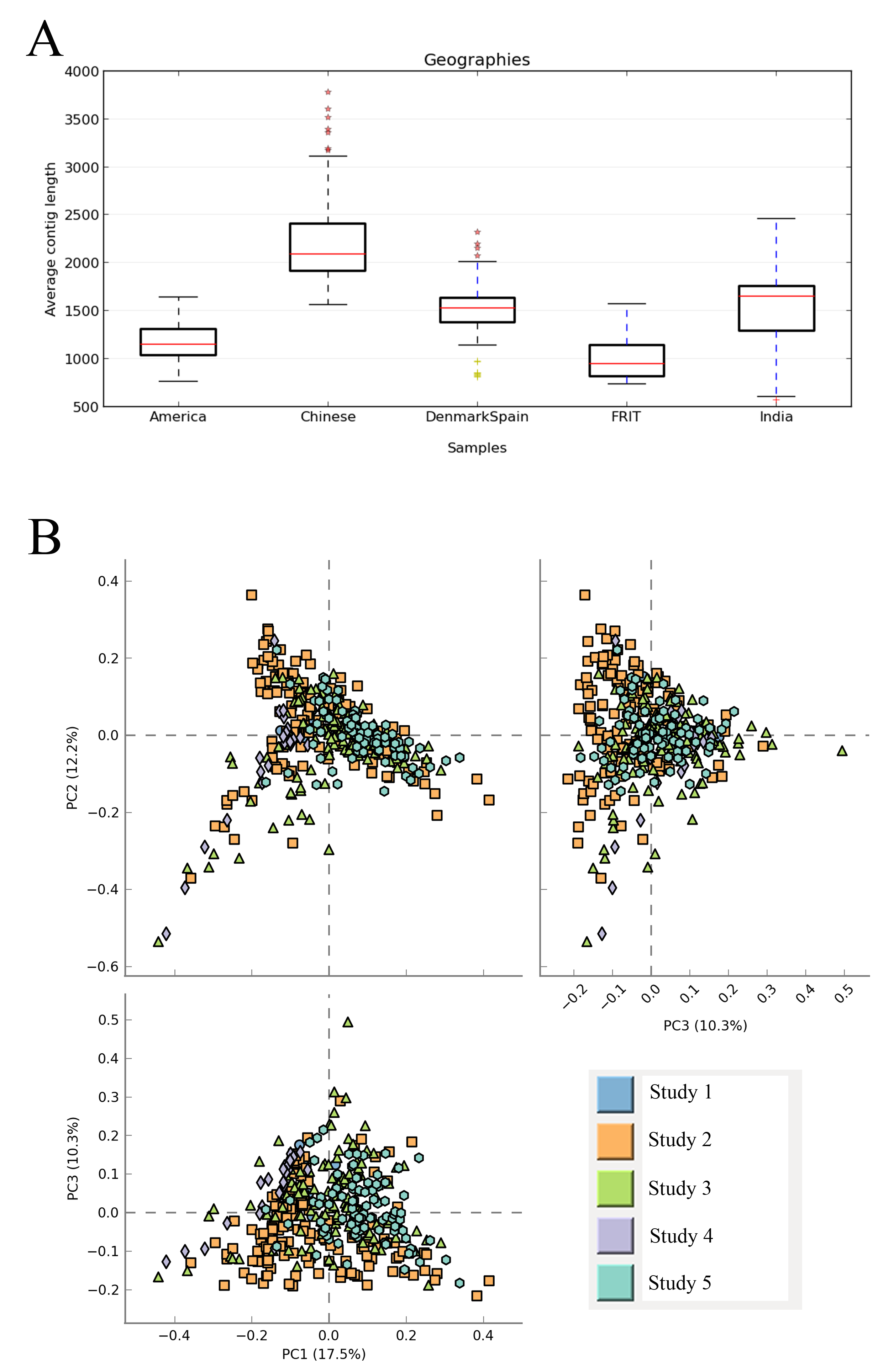

Supplement: S19 Fig — (A) Distribution of the length of contigs in samples belonging to different geographic regions. (B) Principal component analysis (PCA) of genera abundances in metagenomic samples (Study1: JP_AD, JP_CH; Study2: Chinese; Study3: Danish, Spanish, French and Italian; Study4: Indian; Study5: American). Statistical analyses (performed using Kruskal Wallis H-test, with Benjamini-Hochberg Correction indicated no significant variations in the different components across the samples belonging to any of the regions (PC1 < 0.784; PC2 < 0.784; PC3 < 0.764). (TIF) [file pone.0163099.s019.tif]
