# Supplementary material for: Xenobiotic Metabolism and Gut Microbiomes
Source: PLoS One. 2016 Oct 3;11(10):e0163099. doi: 10.1371/journal.pone.0163099 (PMC5047465; doi:10.1371/journal.pone.0163099)
Supplement: S17 Fig — (PDF) [file pone.0163099.s017.pdf]

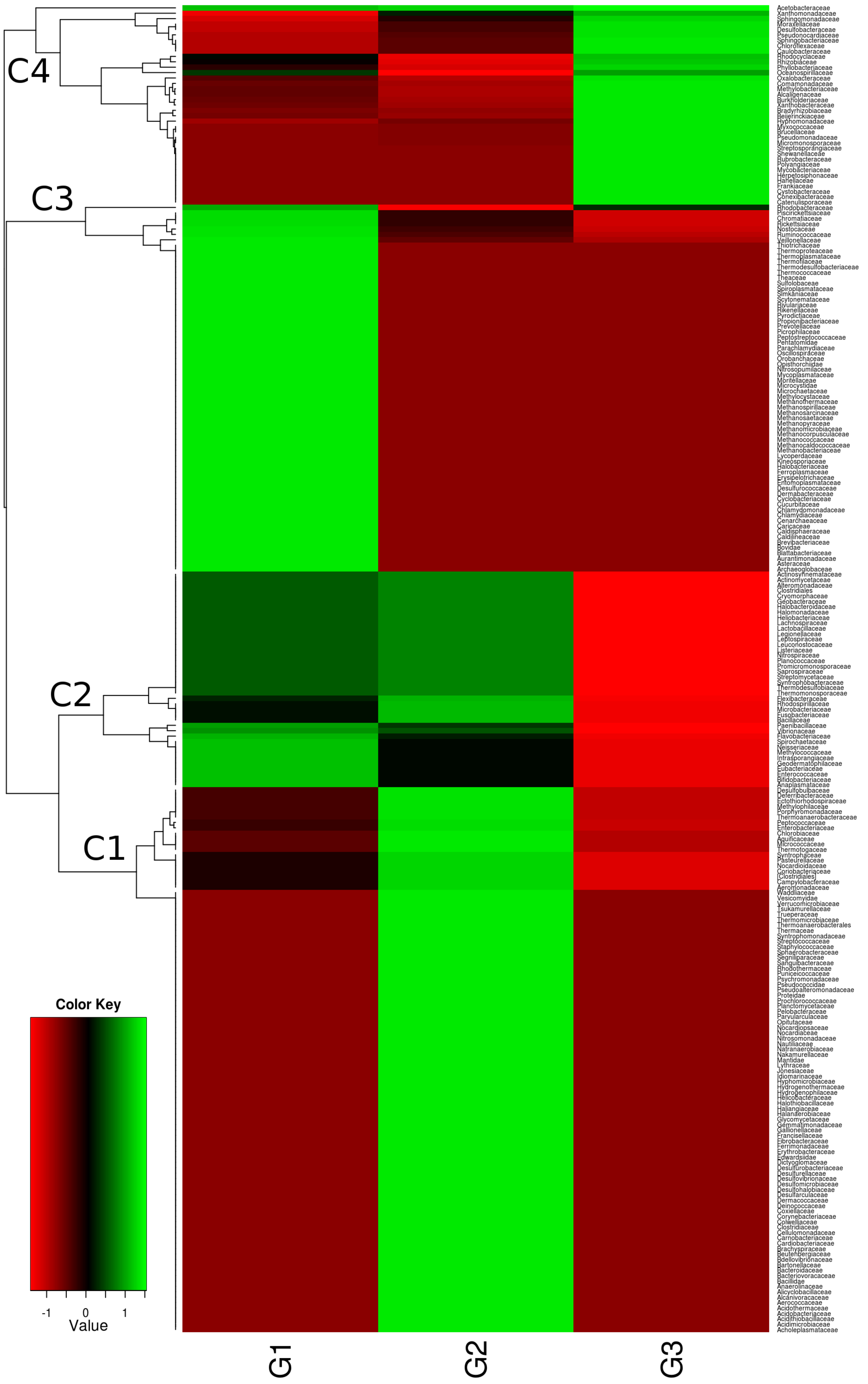
C4
C3
C2
C1
Color Key
-1
0
1
Value
G1
G2
G3
Acetobacteraceae
Xanthomonadaceae
Sphingomonadaceae
Moraxellaceae
Desulfobacteraceae
Pseudonocardiaceae
Sphingobacteriaceae
Chloroflexaceae
Caulobacteraceae
Rhodocyclaceae
Rhizobiaceae
Phyllobacteriaceae
Oceanospirillaceae
Oxalobacteraceae
Comamonadaceae
Methylobacteriaceae
Alcaligenaceae
Burkholderiaceae
Xanthobacteraceae
Bradyrhizobiaceae
Beijerinckiaceae
Hyphomonadaceae
Myxococcaceae
Brucellaceae
Pseudomonadaceae
Micromonosporaceae
Streptosporangiaceae
Shewanellaceae
Rubrobacteraceae
Polyangiaceae
Mycobacteriaceae
Herpetosiphonaceae
Hahellaceae
Frankiaceae
Cystobacteraceae
Conexibacteraceae
Catenulisporaceae
Rhodobacteraceae
Piscirickettsiaceae
Chromatiaceae
Rickettsiaceae
Nostocaceae
Ruminococcaceae
Veillonellaceae
Thiotrichaceae
Thermoproteaceae
Thermoplasmataceae
Thermofilaceae
Thermodesulfobacteriaceae
Thermococcaceae
Theaceae
Sulfolobaceae
Spiroplasmataceae
Simkaniaceae
Scytonemataceae
Rivulariaceae
Rikenellaceae
Pyrodictiaceae
Propionibacteriaceae
Prevotellaceae
Picrophilaceae
Peptostreptococcaceae
Pentatomidae
Parachlamydiaceae
Oscillospiraceae
Orobanchaceae
Opisthorchiidae
Nitrosopumilaceae
Mycoplasmataceae
Moritellaceae
Microcystidae
Microchaetaceae
Methylocystaceae
Methanothermaceae
Methanospirillaceae
Methanosarcinaceae
Methanosaetaceae
Methanopyraceae
Methanomicrobiaceae
Methanocorpusculaceae
Methanococcaceae
Methanocaldococcaceae
Methanobacteriaceae
Lycoperdaceae
Kineosporiaceae
Halobacteriaceae
Ferroplasmaceae
Erysipelotrichaceae
Entomoplasmataceae
Desulfurococcaceae
Dermabacteraceae
Cyclobacteriaceae
Cucurbitaceae
Chlamydomonadaceae
Chlamydiaceae
Cenarchaeaceae
Caricaceae
Caldisphaeraceae
Caldilineaceae
Brevibacteriaceae
Bovidae
Blattabacteriaceae
Aurantimonadaceae
Asteraceae
Archaeoglobaceae
Actinosynnemataceae
Actinomycetaceae
Alteromonadaceae
Clostridiales
Cryomorphaceae
Geobacteraceae
Halobacteroidaceae
Halomonadaceae
Heliobacteriaceae
Lachnospiraceae
Lactobacillaceae
Legionellaceae
Leptospiraceae
Leuconostocaceae
Listeriaceae
Nitrospiraceae
Planococcaceae
Promicromonosporaceae
Saprospiraceae
Streptomycetaceae
Syntrophobacteraceae
Thermodesulfobiaceae
Thermomonosporaceae
Flexibacteraceae
Rhodospirillaceae
Microbacteriaceae
Fusobacteriaceae
Bacillaceae
Paenibacillaceae
Vibrionaceae
Flavobacteriaceae
Spirochaetaceae
Neisseriaceae
Methylococcaceae
Intrasporangiaceae
Geodermatophilaceae
Eubacteriaceae
Enterococcaceae
Bifidobacteriaceae
Anaplasmataceae
Desulfobulbaceae
Deferribacteraceae
Ectothiorhodospiraceae
Methylophilaceae
Porphyromonadaceae
Thermoanaerobacteraceae
Peptococcaceae
Enterobacteriaceae
Chlorobiaceae
Aquificaceae
Micrococcaceae
Thermotogaceae
Syntrophaceae
Pasteurellaceae
Nocardioidaceae
Coriobacteriaceae
[Clostridiales]
Campylobacteraceae
Aeromonadaceae
Waddliaceae
Vesicomyidae
Verrucomicrobiaceae
Tsukamurellaceae
Trueperaceae
Thermomicrobiaceae
Thermoanaerobacterales
Thermaceae
Syntrophomonadaceae
Streptococcaceae
Staphylococcaceae
Sphaerobacteraceae
Segniliparaceae
Sanguibacteraceae
Rhodothermaceae
Puniceicoccaceae
Psychromonadaceae
Pseudococcidae
Pseudoalteromonadaceae
Proteidae
Prochlorococcaceae
Planctomycetaceae
Pelobacteraceae
Parvularculaceae
Opitutaceae
Nocardiopsaceae
Nocardiaceae
Nitrosomonadaceae
Nautiliaceae
Natranaerobiaceae
Nakamurellaceae
Mantidae
Lythraceae
Jonesiaceae
Idiomarinaceae
Hyphomicrobiaceae
Hydrogenothermaceae
Hydrogenophilaceae
Helicobacteraceae
Halothiobacillaceae
Haliangiaceae
Halanaerobiaceae
Glycomycetaceae
Gemmatimonadaceae
Gallionellaceae
Francisellaceae
Fibrobacteraceae
Ferrimonadaceae
Erythrobacteraceae
Edwardsiidae
Dictyoglomaceae
Desulfurobacteriaceae
Desulfurellaceae
Desulfovibrionaceae
Desulfomicrobiaceae
Desulfohalobiaceae
Desulfarculaceae
Dermacoccaceae
Deinococcaceae
Coxiellaceae
Corynebacteriaceae
Colwelliaceae
Clostridiaceae
Cellulomonadaceae
Carnobacteriaceae
Cardiobacteriaceae
Brachyspiraceae
Beutenbergiaceae
Bdellovibrionaceae
Bartonellaceae
Bacteroidaceae
Bacteriovoracaceae
Bacillidae
Anaerolinaceae
Alicyclobacillaceae
Alcanivoracaceae
Aerococcaceae
Acidothermaceae
Acidobacteriaceae
Acidithiobacillaceae
Acidimicrobiaceae
Acholeplasmataceae
