# Supplementary material for: Xenobiotic Metabolism and Gut Microbiomes
Source: PLoS One. 2016 Oct 3;11(10):e0163099. doi: 10.1371/journal.pone.0163099 (PMC5047465; doi:10.1371/journal.pone.0163099)
Supplement: S1 Table — (PDF) [file pone.0163099.s020.pdf]

Region	AG1 (0-10 years)	AG2 (10-30 years)	AG3 (30-40 years)	AG4 (40-50 years)	AG5 (50-60 years)	AG6 (60-above years)	References
**American (AM)**	NA	NA	NA	NA	NA	NA	1
**Danish (DA)**	NA	NA	NA				2
**Spanish (ES)**	NA						2
**French (FR)**	NA	NA	NA	NA	NA		3
**Italian (IT)**	NA	NA	NA	NA	NA		3
**Chinese (CH)**	NA						4
**Indian (IN)**		NA	NA	NA	NA	NA	5
**Japanese (JP)**					NA	NA	3

References:

1. Human Microbiome Project Consortium. Structure, function and diversity of the healthy human microbiome. Nature. 2012;486: 207-214.
2. Qin J, Li R, Raes J, Arumugam M, Burgdorf KS, Manichanh C et al. A human gut microbial gene catalogue established by metagenomic sequencing. Nature. 2010;464: 59-65.
3. Arumugam M, Raes J, Pelletier E, Le Paslier D, Yamada T, Mende DR et al. Enterotypes of the human gut microbiome.Nature. 2011;473: 174-180.
4. Qin J, Li Y, Cai Z, Li S, Zhu J, Zhang F et al. A metagenome-wide association study of gut microbiota in type 2 diabetes. Nature. 2012;490: 55-60.
5. Ghosh TS, Gupta SS, Bhattacharya T, Yadav D, Barik A, ChowdhuryA et. al. Gut microbiomes of Indian children of varying nutritionalstatus. PLoS One. 2014;9: e95547.
