# Supplementary material for: Xenobiotic Metabolism and Gut Microbiomes
Source: PLoS One. 2016 Oct 3;11(10):e0163099. doi: 10.1371/journal.pone.0163099 (PMC5047465; doi:10.1371/journal.pone.0163099)
Supplement: S2 Table — (PDF) [file pone.0163099.s021.pdf]

Genus	Cluster Number	Protein Counts	1.1.1.-	1.1.1.1	1.1.1.157	1.1.1.205	1.1.1.35	1.1.1.90	1.12.99.6	1.13.11.-	1.13.11.1	1.13.11.2	1.13.11.3	1.13.11.39	1.13.11.41	1.13.11.5	1.13.11.8	1.13.12.-	1.14.11.-
**Acaricomes**	1	2	0	0	0	0	0	0	0	0	0	0	0	0	0	0	0	0	0
**Acaryochloris**	2	88	1	1	0	1	0	0	0	0	0	0	0	0	0	0	0	0	0
**Acetivibrio**	1	2	0	0	0	0	0	0	0	0	0	0	0	0	0	0	0	0	0
**Acetobacter**	2	85	1	1	0	1	0	0	0	0	0	0	0	0	0	0	0	0	0
**Acetobacteraceae**	1	2	0	0	0	0	0	0	0	0	0	0	0	0	0	0	0	0	0
**Acetobacterium**	1	8	0	0	0	0	0	0	0	0	0	0	0	0	0	0	0	0	0
**Acetohalobium**	2	79	0	1	0	1	0	0	0	0	0	0	0	0	0	0	0	0	0
**Acholeplasma**	2	38	0	1	0	0	0	0	0	0	0	0	0	0	0	0	0	0	0
**Achromobacter**	2	192	1	1	1	1	1	0	0	0	1	0	0	1	0	1	0	1	1
**Acidaminococcus**	1	65	1	1	1	1	0	0	0	0	0	0	0	0	0	0	0	0	0
**Acidianus**	1	9	0	0	0	0	0	0	0	0	0	0	0	0	0	0	0	0	0
**Acidilobus**	1	8	0	0	0	0	0	0	0	0	0	0	0	0	0	0	0	0	0
**Acidimicrobium**	2	58	1	1	1	1	1	0	0	0	0	0	0	0	0	1	1	0	0
**Acidiphilium**	3	144	1	1	1	1	1	0	1	0	0	0	1	0	0	0	0	0	0
**Acidithiobacillus**	2	70	1	1	0	1	0	0	1	0	0	0	0	0	0	0	0	0	0
**Acidobacterium**	2	91	0	1	1	1	1	0	1	0	0	0	0	0	0	1	0	0	0
**Acidocella**	1	2	0	0	0	0	0	0	0	0	0	0	0	0	0	0	0	0	0
**Acidothermus**	2	68	1	1	0	1	1	0	0	0	0	0	0	1	0	0	0	0	0
**Acidovorax**	3	217	1	1	1	1	1	0	0	0	1	1	0	1	0	0	1	1	0
**Aciduliprofundum**	1	11	0	0	0	0	0	0	0	0	0	0	0	0	0	0	0	0	0
**Acinetobacter**	3	188	1	1	1	1	1	1	0	0	1	0	1	0	0	0	0	0	0
**Actinobacillus**	2	112	1	1	0	1	0	0	1	0	0	0	0	0	0	0	0	0	0
**Actinomadura**	1	1	0	0	0	0	0	0	0	0	0	0	0	0	0	0	0	0	0
**Actinoplanes**	1	52	1	0	0	0	0	0	0	0	0	0	0	0	0	0	0	0	0
**Actinopolyspora**	1	2	0	0	0	0	0	0	0	0	0	0	0	0	0	0	0	0	0
**Actinosynnema**	2	122	1	1	1	1	1	0	0	0	0	0	1	0	0	1	0	0	0
**Advenella**	1	30	0	0	0	0	0	0	0	0	0	0	0	0	0	0	0	0	0
**Aequorivita**	1	12	0	0	0	0	0	0	0	0	0	0	0	0	0	0	0	0	0
**Aerococcus**	2	57	0	1	0	1	1	0	0	0	0	0	0	0	0	0	0	0	0
**Aeromonas**	2	127	1	1	0	1	1	0	1	0	0	0	0	0	0	1	0	0	0
**Aeropyrum**	1	13	0	0	0	0	0	0	0	0	0	0	0	0	0	0	0	0	0
**Afipia**	1	54	0	1	1	1	1	0	0	0	0	0	0	0	0	0	0	0	0
**Aggregatibacter**	1	81	0	1	0	1	0	0	1	0	0	0	0	0	0	0	0	0	0
**Agrobacterium**	2	242	1	1	1	1	1	0	0	0	1	1	1	1	0	1	1	0	1
**Ahrensia**	1	2	0	0	0	0	0	0	0	0	0	0	0	0	0	0	0	0	0
**Akkermansia**	2	47	1	1	0	1	0	0	1	0	0	0	0	0	0	0	0	0	0
**Albidiferax**	3	144	1	1	1	1	1	0	1	0	0	0	0	0	0	0	1	0	0
**Alcaligenes**	1	21	0	0	0	0	0	0	0	0	0	0	0	0	0	0	0	0	0
**Alcanivorax**	2	146	1	1	0	1	1	0	0	0	0	0	0	0	0	0	0	1	0
**Alicycliphilus**	3	152	1	1	1	1	1	0	0	0	1	1	0	0	0	0	0	0	0
**Alicyclobacillus**	2	91	1	1	1	1	1	0	0	0	0	1	0	0	0	0	0	0	0
**Aliivibrio**	2	118	1	1	0	1	1	0	0	0	0	0	0	0	0	0	0	0	0
**Alishewanella**	1	1	0	0	0	0	0	0	0	0	0	0	0	0	0	0	0	0	0
**Alistipes**	1	52	1	0	0	1	0	0	0	0	0	0	0	0	0	0	0	0	0
**Alkalilimnicola**	2	97	0	1	0	1	1	0	1	0	0	0	0	1	0	0	0	0	0
**Alkaliphilus**	2	62	1	1	1	1	0	0	0	0	0	0	0	0	0	0	0	0	0
**Allochromatium**	2	84	1	1	0	1	0	0	1	0	0	0	0	0	0	0	0	0	0
**Alphaproteobacteria**	1	3	0	0	0	0	0	0	0	0	0	0	0	0	0	0	0	0	0
**Alteromonadales**	1	12	0	0	0	0	0	0	0	0	0	0	0	0	0	0	0	0	0
**Alteromonas**	1	37	0	0	0	0	0	0	0	0	0	0	0	0	0	0	0	0	0
**Aminobacter**	1	4	0	0	0	0	0	0	0	0	0	0	0	0	0	0	0	0	0
**Aminobacterium**	2	59	0	1	0	1	0	0	0	0	0	0	0	0	0	0	0	0	0

Genus	Cluster Number	Protein Counts	1.1.1.-	1.1.1.1	1.1.1.157	1.1.1.205	1.1.1.35	1.1.1.90	1.12.99.6	1.13.11.-	1.13.11.1	1.13.11.2	1.13.11.3	1.13.11.39	1.13.11.41	1.13.11.5	1.13.11.8	1.13.12.-	1.14.11.-
**Ammonifex**	2	50	0	1	0	1	0	0	1	0	0	0	0	0	0	0	0	0	0
**Amphibacillus**	1	13	0	0	0	0	0	0	0	0	0	0	0	0	0	0	0	0	0
**Amphritea**	1	2	0	0	0	0	0	0	0	0	0	0	0	0	0	0	0	0	0
**Amycolatopsis**	2	201	1	1	1	1	1	0	1	0	1	1	1	1	0	1	0	0	0
**Amycolicicoccus**	1	43	1	0	0	0	0	0	1	0	0	0	0	0	0	0	0	0	0
**Anabaena**	2	92	1	1	0	1	1	0	1	0	0	0	0	0	0	0	0	0	0
**Anaerobaculum**	1	11	0	0	0	0	0	0	0	0	0	0	0	0	0	0	0	0	0
**Anaerococcus**	2	46	0	1	1	1	1	0	0	0	0	0	0	0	0	0	0	0	0
**Anaerolinea**	2	66	1	1	0	1	0	0	0	0	0	0	0	0	0	0	0	0	0
**Anaeromyxobacter**	3	117	1	1	1	1	1	0	1	0	0	0	0	0	1	1	0	0	0
**Anaerostipes**	1	44	1	0	0	1	1	0	0	0	0	0	0	0	0	0	0	0	0
**Anaerotruncus**	1	2	0	0	0	0	0	0	0	0	0	0	0	0	0	0	0	0	0
**Anaplasma**	1	35	0	0	0	1	0	0	0	0	0	0	1	0	0	0	0	0	0
**Ancylobacter**	1	3	0	0	0	0	0	0	1	0	0	0	0	0	0	0	0	0	0
**Aneurinibacillus**	1	1	0	0	0	0	0	0	0	0	0	0	0	0	0	0	0	0	0
**Angomonas**	1	1	0	0	0	0	0	0	0	0	0	0	0	0	0	0	0	0	0
**Anoxybacillus**	2	72	1	1	0	1	1	0	0	0	0	0	0	0	0	0	0	0	0
**Aquifex**	2	45	0	1	0	1	0	0	1	0	0	0	0	0	0	0	0	0	0
**Aquimarina**	1	2	0	0	0	0	0	0	0	0	0	0	0	0	0	0	0	0	0
**Arcanobacterium**	2	45	0	1	0	1	0	0	0	0	0	0	0	0	0	0	0	0	0
**Archaeoglobus**	1	14	0	0	0	0	0	0	0	0	0	0	0	0	0	0	0	0	0
**Arcobacter**	2	101	1	1	0	1	0	0	1	0	0	0	0	0	0	0	0	0	0
**Aromatoleum**	3	133	1	1	1	1	1	0	0	0	0	0	0	0	0	0	0	0	0
**Arsenophonus**	1	4	0	0	0	0	0	0	0	0	0	0	0	0	0	0	0	0	0
**Arthrobacter**	2	206	1	1	1	1	1	0	0	0	1	1	1	1	0	0	1	1	0
**Arthrospira**	2	67	1	1	0	1	0	0	0	0	0	0	0	0	0	0	0	0	0
**Aster**	1	10	0	0	0	0	0	0	0	0	0	0	0	0	0	0	0	0	0
**Asticcacaulis**	2	103	1	1	1	1	1	0	0	0	0	0	0	0	0	1	1	0	0
**Atopobium**	1	28	1	1	0	1	1	0	0	0	0	0	0	0	0	0	0	0	0
**Aurantimonas**	1	2	0	0	0	0	0	0	0	0	0	0	0	0	0	0	0	0	0
**Avibacterium**	1	17	0	0	0	1	0	0	0	0	0	0	0	0	0	0	0	0	0
**Azoarcus**	3	172	1	1	1	1	0	0	1	0	0	1	0	1	0	0	1	0	0
**Azorhizobium**	3	149	1	1	1	1	1	0	1	0	1	0	1	1	0	0	0	0	0
**Azorhizophilus**	1	4	0	0	0	0	0	0	0	0	0	0	0	0	0	0	0	0	0
**Azospira**	1	19	0	0	0	0	0	0	0	0	0	0	0	0	0	0	0	0	0
**Azospirillum**	2	198	1	1	1	1	1	0	1	0	1	0	1	0	0	1	0	0	0
**Azotobacter**	3	183	1	1	1	1	1	0	1	1	0	1	1	0	0	1	1	1	0
**Bacillales**	1	9	0	0	0	1	0	0	0	0	0	0	0	0	0	0	0	0	0
**Bacilli**	1	3	0	0	0	0	0	0	0	0	0	0	0	0	0	0	0	0	0
**Bacillus**	2	237	1	1	1	1	1	0	0	0	0	1	0	0	0	1	0	0	0
**Bacteria**	2	86	0	1	1	1	1	0	0	0	0	0	0	0	0	0	0	1	0
**Bacteriovorax**	2	98	1	1	1	1	1	0	0	0	0	0	0	0	0	1	0	0	0
**Bacteroidales**	2	59	1	0	0	1	0	0	1	0	0	0	0	0	0	0	0	0	0
**Bacteroides**	2	96	1	1	0	1	0	0	1	0	0	0	0	0	0	0	0	0	0
**Bacteroidetes**	1	2	0	0	0	0	0	0	0	0	0	0	0	0	0	0	0	0	0
**Bartonella**	2	50	1	1	0	1	0	0	0	0	0	0	0	0	0	0	0	0	0
**Baumannia**	2	24	0	0	0	1	0	0	0	0	0	0	0	0	0	0	0	0	0
**Bdellovibrio**	2	87	1	1	1	1	1	0	0	0	0	0	0	0	0	1	0	0	0
**Beijerinckia**	3	123	1	1	1	1	1	0	1	0	0	0	0	0	0	0	0	0	0
**Belliella**	1	14	0	0	0	0	0	0	0	0	0	0	0	0	0	0	0	0	0
**Beutenbergia**	2	82	1	1	0	1	1	0	0	0	0	1	0	0	0	0	0	0	0
**Bifidobacterium**	2	61	1	1	0	1	1	0	0	0	0	0	0	0	0	0	0	0	0

Genus	Cluster Number	Protein Counts	1.1.1.-	1.1.1.1	1.1.1.157	1.1.1.205	1.1.1.35	1.1.1.90	1.12.99.6	1.13.11.-	1.13.11.1	1.13.11.2	1.13.11.3	1.13.11.39	1.13.11.41	1.13.11.5	1.13.11.8	1.13.12.-	1.14.11.-
**Bizionia**	1	1	0	0	0	0	0	0	0	0	0	0	0	0	0	0	0	0	0
**Blastococcus**	1	39	0	0	0	0	0	0	0	0	0	0	0	0	0	0	0	0	0
**Blattabacterium**	1	23	0	0	0	1	0	0	0	0	0	0	0	0	0	0	0	0	0
**Blautia**	1	22	1	0	0	0	0	0	0	0	0	0	0	0	0	0	0	0	0
**Bordetella**	3	227	1	1	1	1	1	0	0	1	1	0	1	1	0	1	0	0	1
**Borrelia**	1	32	1	0	0	1	0	0	0	0	0	0	0	0	0	0	0	0	0
**Brachybacterium**	1	18	0	0	0	0	0	0	0	0	0	0	0	0	0	0	0	0	0
**Brachymonas**	1	4	0	0	0	0	0	0	0	0	0	0	0	0	0	0	0	0	0
**Brachyspira**	2	79	1	1	1	1	1	0	0	0	0	0	0	0	0	0	0	0	0
**Bradyrhizobiaceae**	1	20	0	0	0	1	1	0	0	0	0	0	0	0	0	0	0	0	0
**Bradyrhizobium**	3	254	1	1	1	1	1	0	1	0	1	0	1	1	0	1	1	0	0
**Brenneria**	1	20	0	1	0	1	0	0	0	0	0	0	0	0	0	0	0	0	0
**Brevibacillus**	2	122	1	1	1	1	1	0	0	0	0	0	0	0	0	1	0	0	0
**Brevibacterium**	1	4	0	0	0	0	0	0	0	0	0	0	0	0	0	0	0	1	0
**Brevundimonas**	2	105	1	1	1	1	0	0	0	0	0	0	1	0	0	1	1	0	0
**Brucella**	3	138	1	1	1	1	1	0	0	0	0	0	1	0	0	0	0	0	1
**Buchnera**	1	26	0	0	0	0	0	0	0	0	0	0	0	0	0	0	0	0	0
**Burkholderia**	3	424	1	1	1	1	1	0	1	1	1	1	1	1	1	1	1	1	0
**Burkholderiaceae**	3	143	1	1	1	1	1	0	0	0	0	0	0	0	0	0	0	0	0
**Burkholderiales**	1	24	0	1	0	0	0	0	1	0	0	0	0	0	0	0	0	0	0
**Buttiauxella**	1	2	0	0	0	0	0	0	0	0	0	0	0	0	0	0	0	0	0
**Butyrivibrio**	2	67	1	1	0	1	1	0	0	0	0	0	0	0	0	0	1	0	0
**Caldanaerobacter**	2	63	1	1	1	1	0	0	0	0	0	0	0	0	0	0	0	0	0
**Caldicellulosiruptor**	2	75	1	1	0	1	0	0	0	0	0	0	0	0	0	0	0	0	0
**Caldilinea**	1	23	0	0	0	0	0	0	0	0	0	1	0	0	0	0	0	0	0
**Caldimonas**	1	6	0	0	0	0	0	0	0	0	0	0	0	0	0	0	0	0	0
**Caldisericum**	1	10	0	0	0	0	0	0	0	0	0	0	0	0	0	0	0	0	0
**Caldisphaera**	1	7	0	0	0	0	0	0	0	0	0	0	0	0	0	0	0	0	0
**Calditerrivibrio**	2	74	1	1	0	1	0	0	0	0	0	0	0	0	0	0	0	0	0
**Caldivirga**	1	13	0	0	0	0	0	0	0	0	0	0	0	0	0	0	0	0	0
**Calothrix**	1	43	0	1	0	1	0	0	1	0	0	0	0	0	0	0	0	0	0
**Calyptogena**	2	34	0	0	0	1	0	0	0	0	0	0	0	0	0	0	0	0	0
**Campylobacter**	2	80	1	1	0	1	0	0	1	0	0	0	0	0	0	0	0	0	0
**Candidatus**	2	290	1	1	1	1	1	0	1	0	0	0	1	1	0	1	1	0	0
**Capnocytophaga**	1	50	1	0	0	1	0	0	0	0	0	0	0	0	0	0	0	0	0
**Carbophilus**	1	2	0	0	0	0	0	0	0	0	0	0	0	0	0	0	0	0	0
**Carboxydibrachium**	1	14	0	1	0	1	0	0	0	0	0	0	0	0	0	0	0	0	0
**Carboxydothermus**	2	68	1	1	1	1	1	0	0	0	0	0	0	0	0	0	0	0	0
**Carica**	1	2	0	0	0	0	0	0	0	0	0	0	0	0	0	0	0	0	0
**Carnobacterium**	2	71	1	1	1	1	1	0	0	0	0	0	0	0	0	0	0	0	0
**Catenibacterium**	1	2	0	0	0	0	0	0	0	0	0	0	0	0	0	0	0	0	0
**Catenovulum**	1	2	0	0	0	0	0	0	0	0	0	0	0	0	0	0	0	0	0
**Catenulispora**	3	159	1	1	1	1	1	0	0	0	1	0	1	1	0	1	0	0	0
**Caulobacter**	3	168	1	1	1	1	1	0	0	0	0	0	1	1	0	1	0	0	0
**Cedecea**	1	45	0	1	0	1	0	0	0	0	0	0	0	0	0	0	0	0	0
**Cellulomonas**	2	96	1	1	0	1	1	0	0	0	0	1	0	0	0	0	0	1	0
**Cellulophaga**	2	105	1	1	1	1	1	0	0	0	0	0	0	0	0	1	0	0	0
**Cellulosilyticum**	1	14	0	0	0	0	0	0	0	0	0	0	0	0	0	0	0	0	0
**Cellvibrio**	2	85	0	0	0	1	0	0	0	0	0	0	0	0	0	0	0	1	0
**Cenarchaeum**	1	7	0	0	0	0	0	0	0	0	0	0	0	0	0	0	0	0	0
**Chamaesiphon**	1	13	0	0	0	0	0	0	0	0	0	0	0	0	0	0	0	0	0
**Chelativorans**	3	136	1	1	1	1	1	0	0	0	0	1	1	1	0	1	0	0	1

Genus	Cluster Number	Protein Counts	1.1.1.-	1.1.1.1	1.1.1.157	1.1.1.205	1.1.1.35	1.1.1.90	1.12.99.6	1.13.11.-	1.13.11.1	1.13.11.2	1.13.11.3	1.13.11.39	1.13.11.41	1.13.11.5	1.13.11.8	1.13.12.-	1.14.11.-
Chelatococcus	1	2	0	0	0	0	0	0	0	0	0	0	0	0	0	1	0	0	0
Chitinophaga	2	98	1	1	1	1	0	0	0	0	0	0	0	0	0	1	0	0	0
Chlamydia	1	36	0	0	0	1	0	0	0	0	0	0	0	0	0	0	0	0	0
Chlamydomonas	1	3	0	0	0	0	0	0	0	0	0	0	0	0	0	0	0	0	0
Chlamydophila	1	31	0	0	0	1	0	0	0	0	0	0	0	0	0	0	0	0	0
Chlorobaculum	2	61	0	1	0	1	0	0	1	0	0	0	0	0	0	0	0	0	0
Chlorobium	2	89	1	1	0	1	0	0	1	0	0	0	0	0	0	0	0	0	0
Chlorobium/Pelodictyon	1	2	0	0	0	0	0	0	0	0	0	0	0	0	0	0	0	0	0
Chloroflexus	2	105	0	1	0	1	1	0	1	0	0	1	0	0	0	1	0	0	0
Chlorogloeopsis	1	9	0	0	0	1	0	0	0	0	0	0	0	0	0	0	0	0	0
Chloroherpeton	2	59	0	1	0	1	0	0	1	0	0	0	0	0	0	0	0	0	0
Chromobacterium	2	117	1	1	1	1	1	0	0	0	0	0	0	1	0	1	0	0	0
Chromohalobacter	2	145	1	1	0	1	1	0	0	0	0	0	1	0	0	0	1	0	0
Chroococcidiopsis	1	24	0	0	0	0	0	0	0	0	0	0	0	0	0	0	0	0	0
Chryseobacterium	2	104	1	1	1	1	1	0	0	0	1	0	0	0	0	0	1	0	0
Citreicella	1	13	0	0	0	0	0	0	1	0	0	1	0	0	0	0	0	0	0
Citrobacter	2	190	1	1	0	1	1	0	1	0	0	0	1	0	0	0	1	0	0
Clavibacter	2	83	1	1	0	1	1	0	0	0	0	0	0	0	0	0	0	0	0
Clonorchis	1	1	0	0	0	0	0	0	0	0	0	0	0	0	0	0	0	0	0
Clostridiales	2	77	1	1	0	1	1	0	0	0	0	0	0	0	0	0	0	0	0
Clostridium	2	191	1	1	1	1	1	0	0	0	0	0	0	0	0	0	0	0	0
Cohnella	1	2	0	0	0	0	0	0	0	0	0	0	0	0	0	0	0	0	0
Coleofasciculus	1	4	0	0	0	0	0	0	0	0	0	0	0	0	0	0	0	0	0
Collimonas	1	35	0	0	0	0	0	0	0	0	0	0	0	0	0	0	0	0	0
Colwellia	2	130	1	1	0	1	1	0	0	0	0	0	0	0	0	0	0	0	0
Comamonas	2	196	1	1	1	1	1	0	0	0	1	1	0	0	0	0	1	0	0
Conexibacter	3	117	0	1	1	1	1	0	1	0	0	1	0	0	0	1	0	0	0
Coprobacillus	1	2	0	0	0	0	0	0	0	0	0	0	0	0	0	0	0	0	0
Coprococcus	2	76	1	1	1	1	1	0	0	0	0	0	0	0	0	1	0	0	0
Coprothermobacter	2	45	1	1	0	1	0	0	0	0	0	0	0	0	0	0	0	0	0
Coraliomargarita	2	68	1	1	0	1	0	0	0	0	0	0	0	0	0	1	0	0	0
Corallococcus	1	29	0	0	0	0	0	0	0	0	0	0	0	0	0	0	0	0	0
Coriobacterium	2	36	1	1	1	1	0	0	0	0	0	0	0	0	0	0	0	0	0
Corynebacterineae	1	5	0	0	0	0	0	0	0	0	0	0	0	0	0	0	0	0	0
Corynebacterium	2	175	1	1	1	1	1	0	1	0	1	1	1	0	0	0	0	1	0
Coxiella	2	54	0	0	0	1	1	0	0	0	0	0	0	0	0	0	0	0	0
Crinalium	1	21	0	0	0	1	0	0	0	0	0	0	0	0	0	0	0	0	0
Croceibacter	2	80	1	1	1	1	1	0	0	0	0	0	0	0	0	1	0	0	0
Crocosphaera	1	6	0	0	0	0	0	0	0	0	0	0	0	0	0	0	0	0	0
Cronobacter	2	120	1	1	0	1	1	0	0	0	0	0	0	0	0	0	0	0	0
Cryptobacterium	2	39	0	0	0	1	1	0	1	0	0	0	0	0	0	0	0	0	0
Cucumis	1	9	0	0	0	0	0	0	0	0	0	0	0	0	0	0	0	0	0
Cupriavidus	3	317	1	1	1	1	1	0	1	0	1	1	1	1	0	1	1	1	0
Curtobacterium	1	5	0	0	0	0	0	0	0	0	0	0	0	0	0	0	0	0	0
Curvibacter	1	27	0	0	0	1	0	0	1	0	0	0	0	0	0	0	0	0	0
Cyanobacterium	1	9	0	0	0	0	0	0	0	0	0	0	0	0	0	0	0	0	0
Cyanobium	1	10	0	0	0	0	0	0	0	0	0	0	0	0	0	0	0	0	0
Cyanothece	1	34	0	0	0	0	0	0	0	0	0	0	0	0	0	0	0	0	0
Cyclobacterium	1	19	0	0	0	0	0	0	0	0	0	0	0	0	0	0	0	0	0
Cycloclasticus	1	21	0	0	0	0	0	0	0	0	0	0	0	0	0	0	0	0	0
Cylindrospermopsis	1	4	0	0	0	0	0	0	0	0	0	0	0	0	0	0	0	0	0
Cylindrospermum	1	30	0	1	0	1	0	0	1	0	0	0	0	0	0	0	0	0	0

Genus	Cluster Number	Protein Counts	1.1.1.-	1.1.1.1	1.1.1.157	1.1.1.205	1.1.1.35	1.1.1.90	1.12.99.6	1.13.11.-	1.13.11.1	1.13.11.2	1.13.11.3	1.13.11.39	1.13.11.41	1.13.11.5	1.13.11.8	1.13.12.-	1.14.11.-
Cytophaga	2	69	1	1	1	1	1	0	0	0	0	0	0	0	0	0	0	0	0
Dactylococcopsis	1	8	0	0	0	0	0	0	0	0	0	0	0	0	0	0	0	0	0
Dechloromonas	1	40	0	0	0	0	0	0	0	0	0	0	0	0	0	0	0	0	0
Dechlorosoma	1	19	0	0	0	0	0	0	0	0	0	0	0	0	0	0	0	0	0
Deferribacter	2	73	1	1	1	1	0	0	0	0	0	0	0	0	0	0	0	0	0
Dehalobacter	1	9	0	0	0	0	0	0	0	0	0	0	0	0	0	0	0	0	0
Dehalococcoides	2	41	0	1	0	0	0	0	0	0	0	0	0	0	0	0	0	0	0
Dehalogenimonas	1	37	0	0	0	0	0	0	1	0	0	0	0	0	0	0	0	0	0
Deinococcus	2	140	1	1	1	1	1	0	0	0	0	0	1	0	0	0	0	0	0
Delftia	3	177	1	1	1	1	1	0	0	0	1	1	0	0	0	0	1	1	0
Denitrovibrio	2	67	1	0	0	1	0	0	0	0	0	0	0	0	0	0	0	0	0
Desulfarculus	2	92	0	1	1	1	1	0	0	0	0	0	0	0	0	0	0	0	0
Desulfatibacillum	3	84	1	1	1	1	1	0	1	0	0	0	0	0	0	0	0	0	0
Desulfitobacterium	2	102	0	1	1	1	1	0	1	0	0	0	0	0	0	0	0	0	0
Desulfobacca	2	65	0	1	0	1	1	0	0	0	0	0	0	0	0	0	0	0	0
Desulfobacterium	2	113	1	1	1	1	1	0	1	0	0	0	0	0	0	0	0	0	0
Desulfobacula	1	21	0	0	0	0	0	0	0	0	0	0	0	0	0	0	0	0	0
Desulfobulbus	2	88	1	1	1	1	0	0	1	0	0	0	0	0	0	0	0	0	0
Desulfocapsa	1	9	0	0	0	0	0	0	0	0	0	0	0	0	0	0	0	0	0
Desulfococcus	2	80	0	1	1	1	1	0	0	0	0	0	0	0	0	0	0	0	0
Desulfohalobium	2	52	0	1	0	1	1	0	0	0	0	0	0	0	0	0	0	0	0
Desulfomicrobium	2	65	1	1	0	1	0	0	1	0	0	0	0	0	0	0	0	0	0
Desulfomonile	1	25	0	0	0	0	0	0	0	0	0	0	0	0	0	0	0	0	0
Desulforhabdus	1	2	0	0	0	0	0	0	0	0	0	0	0	0	0	0	0	0	0
Desulfosporosinus	1	30	0	0	0	0	0	0	0	0	0	0	0	0	0	0	0	0	0
Desulfotalea	2	66	1	1	0	1	0	0	0	0	0	0	0	0	0	0	0	0	0
Desulfotomaculum	2	98	0	1	1	1	1	0	0	0	0	0	0	0	0	0	0	0	0
Desulfovibrio	2	144	1	1	0	1	0	0	1	0	0	0	0	0	0	0	0	0	0
Desulfurispirillum	2	78	1	1	1	1	0	0	0	0	0	0	0	0	0	0	0	0	0
Desulfurivibrio	2	71	1	1	0	1	0	0	0	0	0	0	0	0	0	0	0	0	0
Desulfurobacterium	2	53	0	1	0	1	0	0	0	0	0	0	0	0	0	0	0	0	0
Desulfurococcus	1	11	0	0	0	0	0	0	0	0	0	0	0	0	0	0	0	0	0
Diaphorobacter	1	16	0	0	0	0	0	0	0	0	0	1	0	0	0	0	0	0	0
Dichelobacter	2	45	0	0	0	1	0	0	0	0	0	0	0	0	0	0	0	0	0
Dickeya	2	155	1	1	0	1	1	0	1	0	0	0	0	1	0	0	0	0	0
Dictyoglomus	2	62	1	1	0	1	0	0	0	0	0	0	0	0	0	0	0	0	0
Dinoroseobacter	3	148	1	1	1	1	1	0	0	0	1	0	1	0	0	1	0	0	0
Dokdonia	1	3	0	0	0	0	0	0	0	0	0	0	0	0	0	0	0	0	0
Dorea	1	7	0	0	0	0	0	0	0	0	0	0	0	0	0	0	0	0	0
Dyadobacter	2	83	1	1	1	1	0	0	0	0	0	0	0	0	0	1	0	0	0
Echinicola	1	20	0	0	0	0	0	0	0	0	0	0	0	0	0	0	0	0	0
Edwardsiella	2	126	1	1	0	1	0	0	1	0	0	0	0	0	0	0	0	0	0
Eggerthella	1	51	0	1	0	1	0	0	0	0	0	0	0	0	0	0	0	0	0
Ehrlichia	2	37	0	0	0	1	1	0	0	0	0	0	1	0	0	0	0	0	0
Eikenella	1	4	0	0	0	0	0	0	0	0	0	0	0	0	0	0	0	0	0
Elusimicrobium	2	44	1	1	0	1	0	0	0	0	0	0	0	0	0	0	0	0	0
Emticicia	1	21	0	0	0	0	0	0	0	0	0	0	0	0	0	0	0	0	0
Enhydrobacter	1	1	1	0	0	0	0	0	0	0	0	0	0	0	0	0	0	0	0
Ensifer	1	2	0	1	0	0	0	0	0	0	0	0	0	0	0	0	0	0	0
Enterobacter	2	202	1	1	0	1	1	0	1	0	1	0	1	0	0	0	0	0	0
Enterobacteriaceae	2	211	1	1	0	1	1	0	1	0	1	0	1	0	0	0	1	0	0
Enterococcus	1	187	1	1	0	1	1	0	0	1	1	1	1	0	0	0	1	0	0

Genus	Cluster Number	Protein Counts	1.1.1.-	1.1.1.1	1.1.1.157	1.1.1.205	1.1.1.35	1.1.1.90	1.12.99.6	1.13.11.-	1.13.11.1	1.13.11.2	1.13.11.3	1.13.11.39	1.13.11.41	1.13.11.5	1.13.11.8	1.13.12.-	1.14.11.-
Enterovibrio	1	15	0	0	0	1	0	0	0	0	0	0	0	0	0	0	0	0	0
Erwinia	2	151	1	1	0	1	1	0	0	0	0	0	0	0	0	0	1	0	0
Erysipelothrix	1	9	0	0	0	0	0	0	0	0	0	0	0	0	0	0	0	0	0
Erysipelotrichaceae	1	4	0	0	0	0	0	0	0	0	0	0	0	0	0	0	0	0	0
Erythrobacter	2	80	1	1	0	1	1	0	0	0	0	0	0	0	0	0	0	0	0
Escherichia	2	242	1	1	1	1	1	0	1	1	1	0	1	0	0	0	1	0	0
Ethanoligenens	2	69	1	1	0	1	0	0	0	0	0	0	0	0	0	0	0	0	0
Eubacteriaceae	1	2	0	0	0	0	0	0	0	0	0	0	0	0	0	0	0	0	0
Eubacterium	2	103	1	1	1	1	1	0	0	0	0	0	0	0	0	0	0	0	0
Eudoraea	1	4	0	0	0	0	0	0	0	0	0	0	0	0	0	0	0	0	0
Euryarchaeota	1	1	0	0	0	0	0	0	0	0	0	0	0	0	0	0	0	0	0
Exiguobacterium	2	93	1	1	0	1	1	0	0	0	0	1	0	0	0	0	0	0	0
Faecalibacterium	1	12	0	0	0	0	0	0	0	0	0	0	0	0	0	0	0	0	0
Ferrimonas	2	115	1	1	0	1	1	0	1	0	0	0	0	0	0	1	0	0	0
Ferroglobus	1	14	0	0	0	0	0	0	0	0	0	0	0	0	0	0	0	0	0
Ferroplasma	1	11	0	0	0	0	0	0	0	0	0	0	0	0	0	0	0	0	0
Fervidicoccus	1	7	0	0	0	0	0	0	0	0	0	0	0	0	0	0	0	0	0
Fervidobacterium	2	54	1	1	0	1	0	0	0	0	0	0	0	0	0	0	0	0	0
Fibrella	1	17	0	0	0	0	0	0	0	0	0	0	0	0	0	0	0	0	0
Fibrobacter	2	52	1	1	0	1	0	0	0	0	0	0	0	0	0	0	0	0	0
Filifactor	1	9	0	0	0	0	0	0	0	0	0	0	0	0	0	0	0	0	0
Finegoldia	2	55	1	0	0	1	1	0	0	0	0	0	0	0	0	0	0	0	0
Firmicutes	1	15	0	0	0	0	0	0	0	0	0	0	0	0	0	0	0	0	0
Fischerella	1	15	0	0	0	1	0	0	0	0	0	0	0	0	0	0	0	0	0
Flavobacteria	1	7	0	0	1	0	0	0	0	0	0	0	0	0	0	0	0	0	0
Flavobacteriaceae	2	69	0	1	1	1	0	0	0	0	0	0	0	0	0	1	0	0	0
Flavobacteriales	1	3	0	0	0	0	0	0	0	0	0	0	0	0	0	0	0	0	0
Flavobacterium	2	103	1	1	1	1	1	0	1	0	0	0	0	0	0	1	0	0	0
Flexibacter	1	17	0	0	0	0	0	0	0	0	0	0	0	0	0	0	0	0	0
Flexistipes	1	11	0	0	0	0	0	0	0	0	0	0	0	0	0	0	0	0	0
Fluoribacter	1	6	0	0	0	1	0	0	0	0	0	0	0	0	0	0	0	0	0
Fluviicola	2	69	1	0	1	1	1	0	0	0	0	0	0	0	0	1	0	0	0
Formosa	1	2	0	0	0	0	0	0	0	0	0	0	0	0	0	0	0	0	0
Francisella	2	88	1	0	0	1	1	0	0	0	0	0	0	0	0	0	0	0	0
Frankia	3	202	1	1	0	1	1	0	1	1	0	0	1	1	0	0	0	0	0
Frateuria	1	25	0	0	0	0	0	0	0	0	1	0	0	0	0	0	0	0	0
Fructobacillus	1	2	0	0	0	0	0	0	0	0	0	0	0	0	0	0	0	0	0
Fulvimarina	1	2	0	0	0	0	0	0	0	0	0	0	0	0	0	0	0	0	0
Fusobacterium	1	48	1	1	0	1	1	0	0	0	0	0	0	0	0	0	0	0	0
Gallibacterium	2	87	1	1	0	1	1	0	0	0	0	0	0	0	0	0	0	0	0
Gallionella	2	67	1	1	0	1	0	0	0	0	0	0	0	0	0	0	0	0	0
Gammaproteobacteria	1	9	1	0	0	0	0	0	0	0	0	0	0	0	0	0	0	0	0
Gardnerella	1	31	0	1	0	1	0	0	0	0	0	0	0	0	0	0	0	0	0
Geitlerinema	1	9	0	0	0	0	0	0	0	0	0	0	0	0	0	0	0	0	0
Gemmatimonas	2	93	1	1	1	1	1	0	1	0	0	0	0	0	0	1	0	0	0
Geobacillus	2	168	1	1	1	1	1	0	1	0	0	1	0	0	0	0	0	0	0
Geobacter	2	150	1	1	1	1	1	0	1	0	0	0	0	0	0	0	0	0	0
Geodermatophilus	2	136	1	1	1	1	1	0	0	0	1	0	1	1	0	1	0	0	0
Geopsychrobacter	1	2	0	1	0	0	0	0	0	0	0	0	0	0	0	0	0	0	0
Gillisia	1	11	0	0	0	0	0	0	0	0	0	0	0	0	0	1	0	0	0
Gilvimarinus	1	4	0	0	0	0	0	0	0	0	0	0	0	0	0	0	0	0	0
Glaciecola	2	117	1	1	1	1	0	0	0	0	0	0	0	0	0	0	1	0	0

Genus	Cluster Number	Protein Counts	1.1.1.-	1.1.1.1	1.1.1.157	1.1.1.205	1.1.1.35	1.1.1.90	1.12.99.6	1.13.11.-	1.13.11.1	1.13.11.2	1.13.11.3	1.13.11.39	1.13.11.41	1.13.11.5	1.13.11.8	1.13.12.-	1.14.11.-
**Gloeobacter**	2	67	1	1	0	1	0	0	0	0	0	0	0	0	0	0	0	0	1
**Gloeocapsa**	1	20	0	0	0	0	0	0	0	0	0	0	0	0	0	0	0	0	0
**Gloeothece**	1	1	0	0	0	0	0	0	0	0	0	0	0	0	0	0	0	0	0
**Gluconacetobacter**	2	96	1	1	0	1	0	0	0	0	0	0	0	0	0	0	0	0	0
**Gluconobacter**	2	76	1	1	0	1	0	0	0	0	0	0	0	0	0	0	0	0	0
**Gordonia**	1	158	1	1	0	1	1	0	1	0	1	1	0	1	0	0	0	0	0
**Gordonibacter**	1	32	1	0	0	0	0	0	0	0	0	0	0	0	0	0	0	0	0
**Gramella**	2	104	1	1	1	1	1	0	0	0	0	0	1	0	0	1	0	0	0
**Granulibacter**	2	90	1	1	1	1	0	0	0	0	0	0	0	0	0	0	0	0	0
**Granulicella**	2	98	1	1	0	1	1	0	0	0	0	0	0	0	0	0	0	0	0
**Grimontia**	1	9	0	0	0	0	0	0	0	0	0	0	0	0	0	0	0	0	0
**Haemophilus**	2	117	1	1	0	1	1	0	1	0	0	0	1	0	0	0	1	0	0
**Hafnia**	1	29	0	1	0	1	0	0	0	0	0	0	0	0	0	0	0	0	0
**Hahella**	3	130	1	1	0	1	1	0	0	0	0	0	1	0	0	1	0	0	0
**Halalkalicoccus**	1	18	0	0	0	0	0	0	0	0	0	0	0	0	0	0	0	0	0
**Halanaerobium**	2	89	1	1	0	1	1	0	0	0	0	0	0	0	0	0	0	0	0
**Haliangium**	2	108	1	1	1	1	1	0	0	0	0	0	0	0	0	1	0	1	0
**Haliscomenobacter**	2	107	1	1	1	1	0	0	1	0	0	0	1	0	0	1	0	0	0
**Haloarcula**	1	22	0	0	0	0	0	0	0	0	0	0	0	0	0	0	0	0	0
**Halobacillus**	1	19	0	0	0	0	0	0	0	0	0	0	0	0	0	0	0	0	0
**Halobacteriaceae**	1	1	0	0	0	0	0	0	0	0	0	0	0	0	0	0	0	0	0
**Halobacterium**	1	10	0	0	0	0	0	0	0	0	0	0	0	0	0	0	0	0	0
**Halobacteroides**	1	14	0	0	0	0	0	0	0	0	0	0	0	0	0	0	0	0	0
**Haloferax**	1	20	0	0	0	0	0	0	0	0	0	0	0	0	0	0	0	0	0
**Halogeometricum**	1	12	0	0	0	0	0	0	0	0	0	0	0	0	0	0	0	0	0
**Halomicrobium**	1	10	0	0	0	0	0	0	0	0	0	0	0	0	0	0	0	0	0
**Halomonas**	1	149	1	1	1	1	1	0	0	0	0	0	1	0	0	1	0	0	0
**Halopiger**	1	16	0	0	0	0	0	0	0	0	0	0	0	0	0	0	0	0	0
**Haloquadratum**	1	19	0	0	0	0	0	0	0	0	0	0	0	0	0	0	0	0	0
**Halorhabdus**	1	10	0	0	0	0	0	0	0	0	0	0	0	0	0	0	0	0	0
**Halorhodospira**	2	71	1	1	0	1	0	0	0	0	0	0	0	0	0	0	0	0	0
**Halorubrum**	1	16	0	0	0	0	0	0	0	0	0	0	0	0	0	0	0	0	0
**Haloterrigena**	1	19	0	0	0	0	0	0	0	0	0	0	0	0	0	0	0	0	0
**Halothece**	1	9	0	0	0	0	0	0	0	0	0	0	0	0	0	0	0	0	0
**Halothermothrix**	2	50	1	0	0	1	0	0	0	0	0	0	0	0	0	0	0	0	0
**Halothiobacillus**	2	65	1	1	0	1	0	0	0	0	0	0	0	0	0	0	0	0	0
**Halovivax**	1	12	0	0	0	0	0	0	0	0	0	0	0	0	0	0	0	0	0
**Helicobacter**	2	79	1	1	0	1	1	0	1	0	0	0	0	1	0	0	0	0	0
**Heliobacillus**	1	4	0	0	0	1	0	0	0	0	0	0	0	0	0	0	0	0	0
**Heliobacterium**	2	51	0	1	0	1	1	0	0	0	0	0	0	0	0	0	0	0	0
**Herbaspirillum**	2	172	1	1	0	1	1	0	0	0	1	0	1	1	0	1	0	1	0
**Herminiimonas**	3	89	1	1	0	1	1	0	0	0	0	0	0	0	0	0	0	0	0
**Herpetosiphon**	3	90	1	1	0	1	1	0	0	0	0	1	0	0	0	1	0	0	0
**Hippea**	2	44	0	0	0	1	0	0	1	0	0	0	0	0	0	0	0	0	0
**Hirschia**	2	102	1	1	1	1	1	0	0	0	0	0	0	0	0	0	1	0	0
**Histophilus**	2	72	1	0	0	1	0	0	0	0	0	0	0	0	0	0	0	0	0
**Hoeflea**	1	16	0	0	0	1	0	0	0	0	0	0	0	0	0	0	0	0	0
**Hydrogenivirga**	1	2	0	0	0	0	0	0	0	0	0	0	0	0	0	0	0	0	0
**Hydrogenobacter**	2	55	1	1	0	1	0	0	1	0	0	0	0	0	0	0	0	0	0
**Hydrogenobaculum**	2	44	0	1	0	1	0	0	1	0	0	0	0	0	0	0	0	0	0
**Hydrogenophaga**	1	9	0	0	0	0	0	0	0	0	0	1	0	0	0	0	0	0	0
**Hylemonella**	1	4	0	0	0	0	0	0	0	0	0	0	0	0	0	0	0	0	0

Genus	Cluster Number	Protein Counts	1.1.1.-	1.1.1.1	1.1.1.157	1.1.1.205	1.1.1.35	1.1.1.90	1.12.99.6	1.13.11.-	1.13.11.1	1.13.11.2	1.13.11.3	1.13.11.39	1.13.11.41	1.13.11.5	1.13.11.8	1.13.12.-	1.14.11.-
Hyperthermus	1	6	0	0	0	0	0	0	0	0	0	0	0	0	0	0	0	0	0
Hyphomicrobium	2	93	1	1	1	1	0	0	0	0	0	0	0	0	0	0	0	0	0
Hyphomonas	3	105	1	1	1	1	0	0	0	0	0	0	0	1	0	0	0	0	0
Ideonella	1	18	0	0	0	0	0	0	1	0	0	0	0	0	0	0	0	0	0
Idiomarina	2	86	1	1	0	1	1	0	0	0	0	0	0	0	0	1	0	0	0
Ignavibacterium	1	14	0	0	0	0	0	0	0	0	0	0	0	0	0	0	0	0	0
Ignicoccus	1	5	0	0	0	0	0	0	0	0	0	0	0	0	0	0	0	0	0
Ignisphaera	1	8	0	0	0	0	0	0	0	0	0	0	0	0	0	0	0	0	0
Ilyobacter	2	85	1	1	0	1	0	0	0	0	0	0	0	0	0	0	0	0	0
Intrasporangium	2	114	1	1	0	1	1	0	0	0	0	1	0	0	0	1	0	0	0
Isoptericola	1	18	0	0	0	0	0	0	0	0	0	0	0	0	0	0	0	0	0
Isosphaera	2	75	1	1	1	1	0	0	0	0	0	0	1	0	0	0	0	0	0
Jannaschia	3	151	1	1	1	1	1	0	0	0	0	0	1	1	0	1	0	0	0
Janthinobacterium	2	99	1	1	0	1	0	0	0	0	0	0	0	0	0	0	0	0	0
Jonesia	2	56	1	1	0	1	0	0	0	0	0	0	0	0	0	0	0	0	0
Joostella	1	3	0	0	0	0	0	0	0	0	0	0	0	0	0	0	0	0	0
Kaistia	1	2	0	0	0	0	0	0	0	0	0	0	0	0	0	0	0	0	0
Kangiella	2	84	0	1	0	1	1	0	0	0	0	0	0	0	0	1	0	0	0
Ketogulonicigenium	2	102	1	1	0	1	0	0	1	0	0	0	1	0	0	0	0	0	0
Kineococcus	1	24	0	0	0	0	0	0	0	0	0	0	0	0	0	0	0	0	0
Kinetoplastibacterium	1	2	0	0	0	0	0	0	0	0	0	0	0	0	0	0	0	0	0
Kingella	1	4	0	0	0	0	0	0	0	0	0	0	0	0	0	0	0	0	0
Kitasatospora	1	36	0	0	0	0	0	0	0	0	0	0	0	0	0	0	0	0	0
Klebsiella	3	222	1	1	1	1	1	0	0	1	1	0	1	0	0	0	1	0	0
Kluyvera	1	1	0	0	0	0	0	0	0	0	0	0	0	0	0	0	0	0	0
Kocuria	2	87	1	1	1	1	1	0	0	0	0	1	1	0	0	0	0	0	0
Kordia	1	5	0	0	0	0	0	0	0	0	0	0	0	0	0	0	0	0	0
Kosmotoga	2	61	1	1	0	1	0	0	0	0	0	0	0	0	0	0	0	0	0
Kribbella	2	99	1	1	0	1	1	0	0	0	0	0	0	1	0	1	0	0	0
Krokinobacter	2	90	1	1	1	1	1	0	0	0	0	0	0	0	0	1	0	0	0
Kyrpidia	2	113	1	1	1	1	1	0	1	0	0	1	0	0	0	1	1	0	0
Kytococcus	2	70	0	1	0	1	1	0	0	0	0	0	0	0	0	1	0	0	0
Labrenzia	1	4	0	0	0	0	0	0	0	0	0	0	0	0	0	0	0	0	0
Lachnoanaerobaculum	1	1	0	0	0	0	0	0	0	0	0	0	0	0	0	0	0	0	0
Lachnospiraceae	1	51	1	1	0	1	1	0	0	0	0	0	0	0	0	0	0	0	0
Lacinutrix	1	20	0	0	0	0	0	0	0	0	0	0	0	0	0	0	0	0	0
Lactobacillus	2	119	1	1	0	1	1	0	0	0	0	0	0	0	0	0	0	0	0
Lactococcus	2	82	1	1	0	1	0	0	0	0	0	0	0	0	0	0	0	0	0
Lamprocystis	1	2	0	0	0	0	0	0	0	0	0	0	0	0	0	0	0	0	0
Laribacter	2	80	1	0	1	1	1	0	0	0	0	0	0	0	0	0	0	0	0
Lawsonia	2	33	0	0	0	1	0	0	1	0	0	0	0	0	0	0	0	0	0
Leadbetterella	2	79	1	1	0	1	0	0	0	0	0	0	0	0	0	1	0	0	0
Leeuwenhoekiella	1	2	0	0	0	0	0	0	0	0	0	0	0	0	0	0	0	0	0
Legionella	2	117	1	1	0	1	1	0	0	0	0	0	0	0	0	1	0	0	0
Leifsonia	2	48	0	1	0	1	0	0	0	0	0	0	0	0	0	0	0	0	0
Leptolyngbya	1	10	0	0	0	0	0	0	0	0	0	0	0	0	0	0	0	0	0
Leptospira	2	76	1	1	0	1	0	0	0	0	0	0	0	0	0	0	0	0	0
Leptospirillum	1	10	0	0	0	0	0	0	0	0	0	0	0	0	0	0	0	0	0
Leptothrix	3	186	1	1	1	1	1	0	1	0	1	1	1	0	0	1	1	0	0
Leptotrichia	2	43	0	1	0	1	0	0	0	0	0	0	0	0	0	0	0	0	0
Leucobacter	1	2	0	0	0	0	0	0	0	0	0	0	0	0	0	0	1	0	0
Leuconostoc	2	64	1	1	0	1	0	0	0	0	0	0	0	0	0	0	0	0	0

Genus	Cluster Number	Protein Counts	1.1.1.-	1.1.1.1	1.1.1.157	1.1.1.205	1.1.1.35	1.1.1.90	1.12.99.6	1.13.11.-	1.13.11.1	1.13.11.2	1.13.11.3	1.13.11.39	1.13.11.41	1.13.11.5	1.13.11.8	1.13.12.-	1.14.11.-
**Liberibacter**	1	4	0	0	0	0	0	0	0	0	0	0	0	0	0	0	0	0	0
**Limnohabitans**	1	11	0	0	0	0	0	0	0	0	0	0	0	0	0	0	1	0	0
**Listeria**	2	77	1	1	0	1	0	0	0	0	0	0	0	0	0	0	0	0	0
**Listeriaceae**	1	6	0	0	0	0	0	0	0	0	0	0	0	0	0	0	0	0	0
**Listonella**	1	38	0	0	0	1	0	0	0	0	0	0	0	0	0	0	0	0	0
**Loktanella**	1	3	0	0	0	0	0	0	0	0	0	0	0	0	0	0	0	0	0
**Lysinibacillus**	2	97	1	1	1	1	1	0	0	0	0	0	0	0	0	0	0	0	0
**Macrococcus**	2	60	1	0	0	1	1	0	0	0	0	0	0	0	0	0	0	0	0
**Magnetococcus**	2	64	1	0	0	1	1	0	0	0	0	0	0	0	0	0	0	0	0
**Magnetospirillum**	2	113	1	0	1	1	1	0	1	0	0	0	0	0	0	0	1	0	0
**Mahella**	2	58	1	1	0	1	0	0	0	0	0	0	0	0	0	0	0	0	0
**Mannheimia**	2	85	1	1	0	1	0	0	0	0	0	0	0	0	0	0	0	0	0
**Maribacter**	3	87	1	0	1	1	0	0	0	0	0	0	0	0	0	1	0	0	0
**Maricaulis**	3	87	1	1	0	1	0	0	0	0	0	0	0	0	0	0	0	0	0
**Marichromatium**	1	2	0	0	0	0	0	0	0	0	0	0	0	0	0	0	0	0	0
**Marinithermus**	2	77	0	1	1	1	1	0	0	0	0	0	0	0	0	0	0	0	0
**Marinitoga**	1	15	0	0	0	0	0	0	0	0	0	0	0	0	0	0	0	0	0
**Marinobacter**	2	163	1	1	1	1	0	0	0	0	0	1	0	1	0	0	0	0	0
**Marinomonas**	3	192	1	1	0	1	1	0	0	0	1	0	0	0	0	1	1	0	0
**Maritimibacter**	1	7	0	0	0	0	0	0	0	0	0	0	0	0	0	0	0	0	0
**Marivirga**	2	96	1	1	1	1	1	0	0	0	0	0	0	0	0	1	0	0	0
**Martelella**	1	3	1	0	0	0	0	0	0	0	0	0	0	0	0	0	0	0	0
**Marvinbryantia**	1	1	0	0	0	0	0	0	0	0	0	0	0	0	0	0	0	0	0
**Massilia**	1	4	0	0	0	0	0	0	0	0	0	0	0	0	0	0	0	0	0
**Mastigocladopsis**	1	16	1	0	0	1	0	0	0	0	0	0	0	0	0	0	0	0	0
**Megamonas**	1	47	1	1	0	0	0	0	1	0	0	0	0	0	0	0	0	0	0
**Megasphaera**	1	14	0	0	0	0	0	0	0	0	0	0	0	0	0	0	0	0	0
**Meiothermus**	2	110	1	1	1	1	1	0	0	0	0	1	1	0	0	0	0	0	0
**Melissococcus**	2	46	1	1	0	1	0	0	0	0	0	0	0	0	0	0	0	0	0
**Mesoflavibacter**	1	6	0	0	0	0	0	0	0	0	0	0	0	0	0	0	0	0	0
**Mesoplasma**	1	23	0	0	0	1	0	0	0	0	0	0	0	0	0	0	0	0	0
**Mesorhizobium**	2	201	1	1	1	1	1	0	1	0	1	0	1	0	0	1	0	0	0
**Mesotoga**	1	14	0	0	0	0	0	0	0	0	0	0	0	0	0	0	0	0	0
**Metallosphaera**	1	18	0	0	0	0	0	0	0	0	0	0	0	0	0	0	0	0	0
**Methanobacterium**	1	7	0	0	0	0	0	0	0	0	0	0	0	0	0	0	0	0	0
**Methanobrevibacter**	1	9	0	0	0	0	0	0	0	0	0	0	0	0	0	0	0	0	0
**Methanocaldococcus**	1	6	0	0	0	0	0	0	0	0	0	0	0	0	0	0	0	0	0
**Methanocella**	1	15	0	0	0	0	0	0	0	0	0	0	0	0	0	0	0	0	0
**Methanococcaceae**	1	1	0	0	0	0	0	0	0	0	0	0	0	0	0	0	0	0	0
**Methanococcoides**	1	5	0	0	0	0	0	0	0	0	0	0	0	0	0	0	0	0	0
**Methanococcus**	1	9	0	0	0	0	0	0	0	0	0	0	0	0	0	0	0	0	0
**Methanocorpusculum**	1	7	0	0	0	0	0	0	0	0	0	0	0	0	0	0	0	0	0
**Methanoculleus**	1	8	0	0	0	0	0	0	0	0	0	0	0	0	0	0	0	0	0
**Methanohalobium**	1	5	0	0	0	0	0	0	0	0	0	0	0	0	0	0	0	0	0
**Methanohalophilus**	1	5	0	0	0	0	0	0	0	0	0	0	0	0	0	0	0	0	0
**Methanolobus**	1	5	0	0	0	0	0	0	0	0	0	0	0	0	0	0	0	0	0
**Methanomethylovorans**	1	5	0	0	0	0	0	0	0	0	0	0	0	0	0	0	0	0	0
**Methanoplanus**	1	7	0	0	0	0	0	0	0	0	0	0	0	0	0	0	0	0	0
**Methanopyrus**	1	4	0	0	0	0	0	0	0	0	0	0	0	0	0	0	0	0	0
**Methanoregula**	1	8	0	0	0	0	0	0	0	0	0	0	0	0	0	0	0	0	0
**Methanosaeta**	1	9	0	0	0	0	0	0	0	0	0	0	0	0	0	0	0	0	0
**Methanosalsum**	1	7	0	0	0	0	0	0	0	0	0	0	0	0	0	0	0	0	0

Genus	Cluster Number	Protein Counts	1.1.1.-	1.1.1.1	1.1.1.157	1.1.1.205	1.1.1.35	1.1.1.90	1.12.99.6	1.13.11.-	1.13.11.1	1.13.11.2	1.13.11.3	1.13.11.39	1.13.11.41	1.13.11.5	1.13.11.8	1.13.12.-	1.14.11.-
**Methanosarcina**	1	18	0	0	0	0	0	0	0	0	0	0	0	0	0	0	0	0	0
**Methanosphaera**	1	7	0	0	0	0	0	0	0	0	0	0	0	0	0	0	0	0	0
**Methanosphaerula**	1	8	0	0	0	0	0	0	0	0	0	0	0	0	0	0	0	0	0
**Methanospirillum**	1	6	0	0	0	0	0	0	0	0	0	0	0	0	0	0	0	0	0
**Methanothermobacter**	1	6	0	0	0	0	0	0	0	0	0	0	0	0	0	0	0	0	0
**Methanothermococcus**	1	5	0	0	0	0	0	0	0	0	0	0	0	0	0	0	0	0	0
**Methanothermus**	1	5	0	0	0	0	0	0	0	0	0	0	0	0	0	0	0	0	0
**Methanotorris**	1	5	0	0	0	0	0	0	0	0	0	0	0	0	0	0	0	0	0
**Methylacidiphilum**	2	48	0	1	0	1	0	0	1	0	0	0	0	0	0	0	0	0	0
**Methylarcula**	1	2	0	0	0	0	0	0	0	0	0	0	0	0	0	0	0	0	0
**Methylibium**	1	38	0	0	0	0	0	0	1	0	0	0	0	0	0	0	0	0	0
**Methylobacillus**	2	61	1	1	0	1	0	0	0	0	0	0	0	0	0	0	0	0	0
**Methylobacterium**	3	236	1	1	1	1	1	0	0	0	1	0	1	1	0	1	0	1	0
**Methylocella**	3	117	1	1	0	1	1	0	1	0	0	1	0	0	0	0	0	0	0
**Methylococcus**	2	80	1	1	0	1	0	0	1	0	0	0	0	0	0	0	0	0	0
**Methylocystis**	1	21	0	0	0	0	0	0	0	0	0	0	0	0	0	0	0	0	0
**Methyloferula**	1	2	0	0	0	0	0	0	0	0	0	0	0	0	0	0	0	0	0
**Methylomicrobium**	1	12	0	0	0	0	0	0	0	0	0	0	0	0	0	0	0	0	0
**Methylomonas**	1	15	0	0	0	0	0	0	0	0	0	0	0	0	0	0	0	0	0
**Methylophaga**	1	7	0	0	0	0	0	0	0	0	0	0	0	0	0	0	0	0	0
**Methylophilus**	1	2	0	0	0	0	0	0	0	0	0	0	0	0	0	0	0	0	0
**Methylosinus**	1	2	0	0	0	0	0	0	0	0	0	0	0	0	0	0	0	0	0
**Methylotenera**	2	72	1	1	0	1	0	0	0	0	0	0	0	0	0	0	0	0	0
**Methyloversatilis**	1	5	0	0	0	0	0	0	1	0	0	0	0	0	0	0	0	0	0
**Methylovorus**	2	66	1	1	0	1	0	0	0	0	0	0	0	0	0	0	0	0	0
**Micavibrio**	1	9	0	0	0	0	0	0	0	0	0	0	0	0	0	0	0	0	0
**Microbacterium**	2	115	1	1	1	1	0	0	0	0	0	1	1	0	0	0	1	0	0
**Microchaete**	1	23	0	0	0	1	0	0	1	0	0	0	0	0	0	0	0	0	0
**Micrococcus**	2	72	1	1	1	1	1	0	0	0	0	1	0	0	0	0	0	0	0
**Microcoleus**	1	17	0	0	0	0	0	0	0	0	0	0	0	0	0	0	0	0	0
**Microcystis**	2	67	1	1	0	1	0	0	0	0	0	0	0	0	0	0	0	0	0
**Microlunatus**	1	25	0	0	0	0	0	0	0	0	0	0	0	0	0	0	0	0	0
**Micromonospora**	2	115	1	1	1	1	1	0	0	0	0	0	0	1	0	1	0	0	0
**Microvirga**	1	6	0	0	0	0	0	0	0	0	0	0	0	0	0	1	0	0	0
**Mobiluncus**	1	41	0	1	0	1	0	0	1	0	0	0	0	0	0	0	0	0	0
**Modestobacter**	1	43	0	0	0	0	0	0	0	0	0	0	0	0	0	0	0	0	0
**Moorea**	1	2	0	0	0	0	0	0	0	0	0	0	0	0	0	0	0	0	0
**Moorella**	2	69	0	1	0	1	0	0	0	0	0	0	0	0	0	0	0	0	0
**Moraxella**	2	66	1	0	0	1	0	0	0	0	0	0	0	0	0	0	0	0	0
**Morganella**	1	29	0	0	0	1	0	0	0	0	0	0	0	0	0	0	0	0	0
**Moritella**	1	2	0	0	0	0	0	0	0	0	0	0	0	0	0	0	0	0	0
**Muricauda**	1	23	0	0	0	0	0	0	0	0	0	0	0	0	0	0	0	0	0
**Mycobacterium**	3	306	1	1	1	1	1	0	1	1	1	1	1	1	0	1	1	1	0
**Mycoplasma**	1	53	1	1	0	1	0	0	0	0	0	0	0	0	0	0	0	0	0
**Myxococcus**	2	110	1	1	1	1	1	0	0	0	0	0	0	0	0	1	0	0	0
**Nakamurella**	2	115	1	1	0	1	1	0	0	0	0	0	0	1	0	1	0	0	0
**napthalene**	1	2	0	0	0	0	0	0	0	0	0	0	0	0	0	0	0	0	0
**Natranaerobius**	2	55	0	1	1	1	1	0	0	0	0	0	0	0	0	0	0	0	0
**Natrialba**	1	16	0	0	0	0	0	0	0	0	0	0	0	0	0	0	0	0	0
**Natrinema**	1	16	0	0	0	0	0	0	0	0	0	0	0	0	0	0	0	0	0
**Natronobacterium**	1	11	0	0	0	0	0	0	0	0	0	0	0	0	0	0	0	0	0
**Natronococcus**	1	21	0	0	0	0	0	0	0	0	0	0	0	0	0	0	0	0	0

Genus	Cluster Number	Protein Counts	1.1.1.-	1.1.1.1	1.1.1.157	1.1.1.205	1.1.1.35	1.1.1.90	1.12.99.6	1.13.11.-	1.13.11.1	1.13.11.2	1.13.11.3	1.13.11.39	1.13.11.41	1.13.11.5	1.13.11.8	1.13.12.-	1.14.11.-
Natronomonas	1	20	0	0	0	0	0	0	0	0	0	0	0	0	0	0	0	0	0
Nautilia	2	48	1	0	0	1	0	0	1	0	0	0	0	0	0	0	0	0	0
Neisseria	2	67	1	1	0	1	0	0	0	0	0	0	0	0	0	0	0	0	0
Neisseriaceae	1	1	0	0	0	0	0	0	0	0	0	0	0	0	0	0	0	0	0
Neorickettsia	1	31	0	0	0	1	0	0	0	0	0	0	0	0	0	0	0	0	0
Neptuniibacter	1	2	0	0	0	0	0	0	0	0	0	0	0	0	0	0	0	0	0
Niastella	1	21	0	0	0	0	0	0	0	0	0	0	0	0	0	0	0	0	0
Nitratifractor	2	54	1	1	0	1	0	0	0	0	0	0	0	0	0	0	0	0	0
Nitratireductor	1	19	0	1	0	0	0	0	0	0	0	0	0	0	0	0	0	0	0
Nitratiruptor	2	40	0	0	0	1	0	0	1	0	0	0	0	0	0	0	0	0	0
Nitrobacter	2	99	0	1	1	1	1	0	0	0	0	0	0	0	0	1	0	0	0
Nitrococcus	1	2	0	0	0	0	0	0	0	0	0	0	0	0	0	0	0	0	0
Nitrosococcus	2	94	1	1	0	1	1	0	0	0	1	0	0	0	0	1	0	0	0
Nitrosomonas	2	72	1	1	0	1	0	0	0	0	0	0	0	0	0	0	0	0	0
Nitrosopumilaceae	1	7	0	0	0	0	0	0	0	0	0	0	0	0	0	0	0	0	0
Nitrosopumilus	1	11	0	0	0	0	0	0	0	0	0	0	0	0	0	0	0	0	0
Nitrosospira	2	74	1	1	0	1	0	0	0	0	1	0	0	0	0	0	0	1	0
Nocardia	2	185	1	1	1	1	1	0	1	0	1	1	0	1	0	1	0	0	0
Nocardioidaceae	1	2	0	0	0	0	0	0	0	0	0	0	0	0	0	0	0	0	0
Nocardioides	2	157	1	1	1	1	1	0	0	0	1	1	0	1	0	1	0	0	0
Nocardiopsis	2	102	1	1	0	1	1	0	0	0	0	0	0	0	0	1	0	0	0
Nodularia	1	22	0	0	0	1	0	0	1	0	0	0	0	0	0	0	0	0	0
Nonlabens	1	19	0	0	0	0	0	0	0	0	0	0	0	0	0	0	0	0	0
Nostoc	2	111	1	1	0	1	1	0	1	0	0	0	0	0	0	0	0	0	0
Nostocaceae	1	2	0	0	0	0	0	0	0	0	0	0	0	0	0	0	0	0	0
Novosphingobium	2	169	1	1	1	1	1	0	1	0	1	0	0	0	0	0	1	0	0
Oceanibaculum	1	3	0	0	0	0	0	0	0	0	0	0	0	0	0	0	0	0	0
Oceanibulbus	1	12	0	0	0	0	0	0	0	0	0	0	0	0	0	0	0	0	0
Oceanicola	1	9	0	0	0	0	0	0	0	0	0	0	0	0	0	0	0	0	0
Oceanimonas	1	33	0	0	0	0	0	0	0	0	0	0	0	0	0	0	0	0	0
Oceaniovalibus	1	2	0	0	0	0	0	0	0	0	0	0	0	0	0	0	0	0	0
Oceanithermus	2	72	1	1	1	1	0	0	0	0	0	0	0	0	0	0	0	0	0
Oceanobacillus	2	91	1	1	1	1	1	0	0	0	0	0	0	0	0	0	0	0	0
Oceanospirillum	1	2	0	0	0	0	0	0	0	0	0	0	0	0	0	0	0	0	0
Ochrobactrum	2	133	1	1	1	1	1	0	0	0	0	0	1	0	0	0	0	0	1
Octadecabacter	1	29	0	0	0	0	0	0	0	0	0	0	0	0	0	0	0	0	0
Oenococcus	2	41	1	1	0	1	0	0	0	0	0	0	0	0	0	0	0	0	0
Oleomonas	1	2	0	0	0	0	0	0	0	0	0	0	0	0	0	0	0	0	0
Oligotropha	3	87	1	1	1	1	1	0	0	0	0	0	0	0	0	0	0	0	0
Olsenella	2	40	1	1	0	1	1	0	0	0	0	0	0	0	0	0	0	0	0
Opitutus	2	67	1	1	0	1	0	0	0	0	0	0	0	1	0	0	0	0	0
Orientia	1	21	0	0	0	0	0	0	0	0	0	0	0	0	0	0	0	0	0
Ornithobacterium	1	11	0	0	0	0	0	0	0	0	0	0	0	0	0	0	0	0	0
Oscillatoria	1	16	0	0	0	0	0	0	0	0	0	0	0	0	0	0	0	0	0
Oscillatoriales	1	2	0	0	0	0	0	0	0	0	0	0	0	0	0	0	0	0	0
Oscillibacter	1	20	0	0	0	0	0	0	0	0	0	0	0	0	0	0	0	0	0
Owenweeksia	1	15	0	0	0	0	0	0	0	0	0	0	0	0	0	0	0	0	0
Oxalobacteraceae	1	4	0	0	0	0	0	0	0	0	0	0	0	0	0	0	0	0	0
Paenibacillus	2	152	1	1	1	1	1	0	0	0	0	0	0	0	0	0	0	0	0
Paludibacter	2	67	1	1	0	1	0	0	1	0	0	0	0	0	0	0	0	0	0
Pandoraea	1	18	0	0	0	0	0	0	0	0	0	0	0	0	0	0	0	0	0
Pannonibacter	1	3	0	0	0	0	0	0	0	0	0	0	0	0	0	0	0	0	0

Genus	Cluster Number	Protein Counts	1.1.1.-	1.1.1.1	1.1.1.157	1.1.1.205	1.1.1.35	1.1.1.90	1.12.99.6	1.13.11.-	1.13.11.1	1.13.11.2	1.13.11.3	1.13.11.39	1.13.11.41	1.13.11.5	1.13.11.8	1.13.12.-	1.14.11.-
**Pantholops**	1	23	1	1	0	1	0	0	0	0	0	0	0	0	0	0	0	0	0
**Pantoea**	2	181	1	1	0	1	1	0	0	0	0	1	1	0	0	0	0	0	0
**Parabacteroides**	2	64	1	0	0	1	0	0	1	0	0	0	0	0	0	0	0	0	0
**Parachlamydia**	1	11	0	0	0	0	0	0	0	0	0	0	0	0	0	0	0	0	0
**Paracoccus**	2	169	1	1	1	1	1	0	1	0	1	0	1	0	0	1	0	0	0
**Parvibaculum**	3	117	1	1	1	1	1	0	0	0	0	0	0	1	0	0	0	0	0
**Parvularcula**	2	72	1	1	1	1	1	0	0	0	0	0	0	0	0	0	0	0	0
**Pasteurella**	2	85	1	0	0	1	0	0	0	0	0	0	0	0	0	0	0	0	0
**Pasteurellaceae**	1	2	0	0	0	0	0	0	0	0	0	0	0	0	0	0	0	0	0
**Pectobacterium**	1	44	0	1	0	1	0	0	0	0	0	0	0	0	0	0	0	0	0
**Pediococcus**	1	43	1	1	0	1	0	0	0	0	0	0	0	0	0	0	0	0	0
**Pedobacter**	2	88	1	1	1	1	1	0	0	0	0	0	0	0	0	1	0	0	0
**Pelagibaca**	1	10	0	0	0	0	0	0	0	0	0	0	0	0	0	0	0	0	0
**Pelagibacterium**	1	39	0	0	0	0	0	0	0	0	0	0	0	0	0	0	0	0	0
**Pelobacter**	2	85	1	1	1	1	0	0	0	0	0	0	0	0	0	0	0	0	0
**Pelodictyon**	2	62	1	1	0	1	0	0	1	0	0	0	0	0	0	0	0	0	0
**Pelosinus**	1	1	0	0	0	0	0	0	0	0	0	0	0	0	0	0	0	0	0
**Pelotomaculum**	2	60	0	1	1	1	0	0	0	0	0	0	0	0	0	0	0	0	0
**Peptostreptococcus**	1	1	0	0	0	0	0	0	0	0	0	0	0	0	0	0	0	0	0
**Persephonella**	2	55	0	1	0	1	0	0	1	0	0	0	0	0	0	0	0	0	0
**Petrotoga**	2	72	1	1	0	1	0	0	0	0	0	0	0	0	0	0	0	0	0
**Phaeobacter**	2	55	0	1	0	1	0	0	0	0	0	0	0	0	0	1	0	0	0
**Phenylobacterium**	3	103	1	1	1	1	0	0	0	0	0	0	0	0	0	1	0	0	0
**Photobacterium**	2	119	1	1	0	1	1	0	0	0	0	0	0	0	0	1	0	0	0
**Photorhabdus**	2	149	1	1	0	1	0	0	0	0	0	0	0	0	0	0	0	0	0
**Phycisphaera**	1	9	0	0	0	0	0	0	0	0	0	0	0	0	0	0	0	0	0
**Phyllobacterium**	1	6	0	0	0	0	0	0	0	0	0	0	0	0	0	0	0	0	0
**Picrophilus**	1	15	0	0	0	0	0	0	0	0	0	0	0	0	0	0	0	0	0
**Pirellula**	2	85	1	1	0	1	0	0	0	0	0	0	1	0	0	0	0	0	0
**Planctomyces**	2	93	1	1	0	1	1	0	0	0	0	0	1	0	0	0	0	0	0
**Plautia**	1	17	0	0	0	1	0	0	0	0	0	0	0	0	0	0	0	0	0
**Plesiomonas**	1	4	0	0	0	0	0	0	0	0	0	0	0	0	0	0	0	0	0
**Pleurocapsa**	1	23	0	0	0	0	0	0	0	0	0	0	0	0	0	0	0	0	0
**Polaribacter**	2	74	1	0	1	1	1	0	0	0	0	0	0	0	0	1	0	0	0
**Polaromonas**	3	238	1	1	1	1	1	0	1	0	1	0	1	0	0	1	1	0	0
**Polymorphum**	3	173	1	1	1	1	1	0	0	0	1	1	1	0	0	1	1	0	0
**Polynucleobacter**	1	19	0	0	0	0	0	0	0	0	0	0	0	0	0	0	0	0	0
**Ponticaulis**	1	1	0	0	0	0	0	0	0	0	0	0	0	0	0	0	0	0	0
**Porphyromonas**	2	63	1	1	1	1	1	0	0	0	0	0	0	0	0	0	0	0	0
**Prevotella**	1	65	1	0	0	1	0	0	0	0	0	0	0	0	0	0	0	0	0
**Prochlorococcus**	2	68	1	0	0	1	0	0	0	0	0	0	0	0	0	0	0	0	0
**Propionibacterium**	1	62	0	1	0	1	0	0	0	0	0	0	0	0	0	0	0	0	0
**Prosthecochloris**	1	16	0	0	0	1	0	0	0	0	0	0	0	0	0	0	0	0	0
**Proteobacteria**	1	19	0	1	0	0	0	0	0	0	0	0	0	0	0	0	0	0	0
**Proteus**	2	113	1	1	0	1	1	0	1	0	0	0	0	0	0	0	0	0	0
**Providencia**	1	38	0	1	0	0	0	0	0	0	0	0	0	0	0	0	0	0	0
**Pseudaminobacter**	1	10	0	0	0	0	0	0	0	0	0	0	0	0	0	0	0	0	0
**Pseudanabaena**	1	11	0	0	0	0	0	0	0	0	0	0	0	0	0	0	0	0	0
**Pseudoalteromonas**	2	188	1	1	1	1	1	1	0	0	0	0	0	1	0	1	1	0	0
**Pseudochrobactrum**	1	7	0	1	0	0	0	0	0	0	0	0	0	0	0	0	0	0	0
**Pseudogulbenkiania**	1	40	0	0	0	0	0	0	0	0	0	0	0	0	0	0	0	0	0
**pseudomallei**	3	131	1	1	0	1	1	0	0	0	0	0	1	0	1	1	0	0	0

Genus	Cluster Number	Protein Counts	1.1.1.-	1.1.1.1	1.1.1.157	1.1.1.205	1.1.1.35	1.1.1.90	1.12.99.6	1.13.11.-	1.13.11.1	1.13.11.2	1.13.11.3	1.13.11.39	1.13.11.41	1.13.11.5	1.13.11.8	1.13.12.-	1.14.11.-
Pseudomonas	3	381	1	1	1	1	1	1	1	0	1	1	1	1	0	1	1	1	0
Pseudonocardia	3	180	1	1	0	1	1	0	1	0	1	1	1	1	0	1	1	0	0
Pseudorhodobacter	1	4	0	0	0	1	0	0	0	0	0	0	0	0	0	0	0	0	0
Pseudovibrio	1	33	0	0	0	0	0	0	0	0	0	0	0	0	0	0	0	0	0
Pseudoxanthomonas	2	124	1	1	0	1	1	0	0	0	0	0	0	0	0	1	0	0	0
Psychrobacter	2	124	1	1	0	1	1	0	0	0	1	0	0	0	0	0	0	0	0
Psychroflexus	1	22	0	0	0	0	0	0	0	0	0	0	0	0	0	0	0	0	0
Psychromonas	2	110	1	1	0	1	1	0	0	0	0	0	0	0	0	0	0	0	0
Pusillimonas	3	111	1	1	1	1	1	0	0	0	0	0	0	0	0	1	0	0	0
Pyrobaculum	1	15	0	0	0	0	0	0	0	0	0	0	0	0	0	0	0	0	0
Pyrococcus	1	10	0	0	0	0	0	0	0	0	0	0	0	0	0	0	0	0	0
Pyrolobus	1	4	0	0	0	0	0	0	0	0	0	0	0	0	0	0	0	0	0
Rahnella	2	143	1	1	1	1	1	0	0	0	0	0	0	0	0	0	1	0	0
Ralstonia	3	332	1	1	1	1	1	0	0	0	1	1	1	1	0	1	1	0	0
Ramlibacter	1	35	0	0	0	1	0	0	0	0	0	0	0	0	0	0	1	0	0
Raoultella	2	139	1	1	0	1	0	0	0	0	1	0	1	0	0	0	1	0	0
Raphidiopsis	1	5	0	0	0	0	0	0	0	0	0	0	0	0	0	0	0	0	0
Reinekea	1	2	0	0	0	0	0	0	0	0	0	0	0	0	0	0	0	0	0
Renibacterium	2	67	1	1	1	1	1	0	0	0	0	1	0	0	0	0	0	0	0
Rhizobiaceae	1	3	0	0	0	0	0	0	0	0	0	0	0	0	0	0	0	0	0
Rhizobiales	1	6	0	0	0	0	0	0	0	0	0	0	0	0	0	0	0	0	0
Rhizobium	3	257	1	1	1	1	1	0	1	1	1	1	1	0	0	1	0	0	0
Rhizobium/Agrobacterium	2	87	1	1	1	1	1	0	0	0	1	1	0	0	0	0	0	0	0
Rhodanobacter	1	21	0	0	0	0	0	0	0	0	0	0	0	0	0	0	0	0	0
Rhodobacter	3	175	1	1	1	1	1	0	1	0	0	1	0	1	0	0	0	0	0
Rhodobacteraceae	1	21	0	0	0	0	0	0	0	0	0	0	0	0	0	0	0	0	0
Rhodobacterales	1	34	0	1	0	1	1	0	0	0	0	0	0	0	0	0	0	0	0
Rhodococcus	2	292	1	1	1	1	1	0	1	1	1	1	1	1	0	1	0	0	0
Rhodocyclaceae	1	7	0	0	0	0	0	0	1	0	0	0	0	0	0	0	0	0	0
Rhodoferax	3	144	1	1	1	1	1	0	1	0	0	0	0	0	0	0	1	0	0
Rhodomicrobium	2	103	1	1	1	1	1	0	1	0	0	0	0	0	0	0	0	0	0
Rhodopirellula	2	87	1	1	0	1	0	0	0	0	0	0	1	0	0	0	0	0	0
Rhodopseudomonas	3	246	1	1	1	1	1	0	1	0	1	1	1	0	0	1	1	0	0
Rhodospirillum	2	121	1	1	1	1	1	0	1	0	0	0	0	0	0	1	0	0	0
Rhodothermus	2	76	0	1	1	1	1	0	0	0	0	0	0	0	0	0	0	0	0
Rhodovulum	1	4	0	0	0	0	0	0	0	0	0	0	0	0	0	0	0	0	0
Richelia	1	2	0	0	0	0	0	0	0	0	0	0	0	0	0	0	0	0	0
Rickettsia	2	43	0	0	0	0	1	0	0	0	0	0	1	0	0	0	0	0	0
Rickettsiaceae	1	5	0	0	0	0	0	0	0	0	0	0	0	0	0	0	0	0	0
Riemerella	2	61	1	0	1	1	0	0	0	0	0	0	0	0	0	1	0	0	0
Rivularia	1	18	0	0	0	0	0	0	0	0	0	0	0	0	0	0	0	0	0
Robiginitalea	2	83	1	1	1	1	1	0	1	0	0	0	1	0	0	1	0	0	0
Roseburia	2	58	1	1	0	1	1	0	0	0	0	0	0	0	0	0	0	0	0
Roseibium	1	3	0	0	0	0	0	0	0	0	0	0	0	0	0	0	0	0	0
Roseiflexus	3	100	1	1	0	1	0	0	1	0	0	1	0	0	0	0	0	0	0
Roseobacter	2	149	1	1	1	1	1	0	0	0	0	0	1	0	0	1	0	0	0
Roseomonas	1	2	0	0	0	0	0	0	0	0	0	0	0	0	0	0	0	0	0
Roseovarius	1	15	0	0	0	0	0	0	1	0	0	0	0	0	0	0	0	0	0
Rothia	1	44	1	1	0	1	0	0	0	0	0	0	0	0	0	0	0	0	0
Rubrivivax	1	24	0	0	0	0	0	0	0	0	0	0	0	0	0	0	0	0	0
Rubrobacter	3	117	1	1	1	1	1	0	0	0	1	1	1	0	0	0	0	0	0
Ruegeria	2	189	1	1	1	1	1	0	0	0	0	0	1	1	0	1	0	0	0

Genus	Cluster Number	Protein Counts	1.1.1.-	1.1.1.1	1.1.1.157	1.1.1.205	1.1.1.35	1.1.1.90	1.12.99.6	1.13.11.-	1.13.11.1	1.13.11.2	1.13.11.3	1.13.11.39	1.13.11.41	1.13.11.5	1.13.11.8	1.13.12.-	1.14.11.-
Ruminococcaceae	1	2	0	0	0	0	0	0	0	0	0	0	0	0	0	0	0	0	0
Ruminococcus	2	101	1	1	1	1	1	0	0	0	0	0	0	0	0	0	0	0	0
Runella	1	26	0	0	0	0	0	0	0	0	0	0	0	0	0	0	0	0	0
Saccharomonospora	2	132	1	1	0	1	1	0	1	0	1	0	1	0	0	1	1	0	0
Saccharophagus	2	81	1	1	0	1	0	0	0	0	0	0	0	0	0	0	0	0	0
Saccharopolyspora	3	194	1	1	0	1	1	0	1	0	1	0	1	0	0	1	0	0	0
Saccharothrix	1	32	0	0	0	0	0	0	0	0	0	0	0	0	0	0	0	0	0
Sagittula	1	10	0	0	0	0	0	0	1	0	0	1	0	0	0	0	0	0	0
Salinibacter	2	77	1	1	1	1	1	0	0	0	0	0	0	0	0	0	0	0	0
Salinimonas	1	1	0	0	0	0	0	0	0	0	0	0	0	0	0	0	0	0	0
Salinispora	3	127	1	1	1	1	1	0	0	0	0	0	0	1	0	1	0	0	0
Salinivibrio	1	2	0	0	0	0	0	0	0	0	0	0	0	0	0	0	0	0	0
Salmonella	2	151	1	1	0	1	1	0	1	0	0	0	0	0	0	0	0	0	0
Sanguibacter	2	73	1	1	0	1	0	0	0	0	0	0	0	0	0	0	0	0	0
Saprospira	1	13	0	0	0	0	0	0	0	0	0	0	0	0	0	0	0	0	0
Scardovia	1	1	0	1	0	0	0	0	0	0	0	0	0	0	0	0	0	0	0
Scytonema	1	10	0	0	0	0	0	0	0	0	0	0	0	0	0	0	0	0	0
Sebaldella	2	69	1	1	0	1	0	0	0	0	0	0	0	0	0	0	0	0	0
Segniliparus	2	85	1	1	0	1	1	0	0	0	0	0	0	1	0	0	0	0	0
Selenomonas	2	57	1	0	0	1	0	0	1	0	0	0	0	0	0	0	0	0	0
Serinicoccus	1	1	0	0	0	0	0	0	0	0	0	1	0	0	0	0	0	0	0
Serratia	2	186	1	1	1	1	1	0	0	0	0	0	1	1	0	0	1	0	0
Shewanella	3	214	1	1	0	1	1	0	1	0	0	0	0	1	0	1	0	0	0
Shigella	2	172	1	1	0	1	1	0	1	0	0	0	0	0	0	0	0	0	0
Shinella	1	2	0	0	0	0	0	0	0	0	0	0	0	0	0	0	0	0	0
Sideroxydans	2	81	1	1	0	1	0	0	1	0	0	0	0	0	0	0	0	0	0
Silicibacter	2	105	0	1	1	1	1	0	0	0	0	0	1	0	0	1	0	0	0
Simiduia	1	18	0	0	0	0	0	0	0	0	0	0	0	0	0	0	0	0	0
Simkania	1	6	0	0	0	0	0	0	0	0	0	0	0	0	0	0	0	0	0
Simonsiella	1	2	0	0	0	0	0	0	0	0	0	0	0	0	0	0	0	0	0
Singulisphaera	1	18	0	0	0	0	0	0	0	0	0	0	0	0	0	0	0	0	0
Sinorhizobium	3	207	1	1	1	1	1	0	0	1	1	0	1	0	0	1	0	0	0
Slackia	2	46	1	1	0	1	1	0	0	0	0	0	0	0	0	0	0	0	0
Smaragdicoccus	1	3	0	0	0	0	0	0	1	0	0	0	0	0	0	0	0	0	0
Sodalis	2	71	1	1	0	1	0	0	0	0	0	0	0	0	0	0	0	0	0
Solibacillus	1	23	0	0	0	1	0	0	0	0	0	0	0	0	0	0	0	0	0
Solitalea	1	13	0	0	0	0	0	0	0	0	0	0	0	0	0	0	0	0	0
Sorangium	3	115	1	1	0	1	1	0	0	0	1	0	1	0	0	1	0	0	0
Sphaerobacter	2	100	0	1	1	1	1	0	1	0	0	1	1	0	0	0	0	0	0
Sphaerochaeta	1	42	1	1	0	1	0	0	0	0	0	0	0	0	0	0	0	0	0
Sphingobacterium	3	85	1	1	1	1	1	0	0	0	0	0	0	0	0	0	0	0	0
Sphingobium	1	179	1	1	0	1	1	0	0	0	1	1	0	1	0	1	1	0	0
Sphingomonadaceae	1	15	0	1	0	0	0	0	0	0	0	0	0	0	0	0	0	0	0
Sphingomonas	2	220	1	1	1	1	1	0	0	0	1	1	1	1	0	1	1	0	0
Sphingopyxis	3	99	1	1	0	1	1	0	1	0	0	0	0	0	0	0	0	0	0
Spirochaeta	2	103	1	1	0	1	0	0	0	0	0	0	0	0	0	0	0	0	0
Spiroplasma	1	6	0	0	0	0	0	0	0	0	0	0	0	0	0	0	0	0	0
Spirosoma	2	107	1	1	1	1	1	0	1	0	0	1	1	0	0	1	0	0	0
Stackebrandtia	2	110	1	1	1	1	1	0	0	0	1	0	1	0	0	1	0	0	0
Stanieria	1	17	0	0	0	0	0	0	0	0	0	0	0	0	0	0	0	0	0
Staphylococcus	2	108	1	1	0	1	1	0	0	0	0	0	0	0	0	0	0	0	0
Staphylothermus	1	12	0	0	0	0	0	0	0	0	0	0	0	0	0	0	0	0	0

Genus	Cluster Number	Protein Counts	1.1.1.-	1.1.1.1	1.1.1.157	1.1.1.205	1.1.1.35	1.1.1.90	1.12.99.6	1.13.11.-	1.13.11.1	1.13.11.2	1.13.11.3	1.13.11.39	1.13.11.41	1.13.11.5	1.13.11.8	1.13.12.-	1.14.11.-
**Stappia**	1	2	0	0	0	0	0	0	0	0	0	0	0	0	0	0	0	0	0
**Starkeya**	3	134	1	1	1	1	1	0	0	0	0	0	0	0	0	0	0	0	0
**Stenotrophomonas**	3	104	1	1	0	1	1	0	0	0	1	0	1	0	0	1	0	0	0
**Stigmatella**	3	129	1	1	0	1	1	0	0	0	0	0	1	0	0	1	0	0	0
**Streptobacillus**	1	38	0	1	0	1	0	0	0	0	0	0	0	0	0	0	0	0	0
**Streptococcus**	2	121	1	1	0	1	1	0	0	0	0	0	0	0	0	0	0	0	0
**Streptomyces**	2	253	1	1	1	1	1	0	1	1	1	1	1	1	0	1	0	1	0
**Streptosporangium**	3	148	1	1	0	1	1	0	1	0	1	0	1	1	0	1	0	0	0
**Strigomonas**	1	2	0	0	0	0	0	0	0	0	0	0	0	0	0	0	0	0	0
**Sulfitobacter**	1	10	0	0	0	0	0	0	0	0	0	0	0	0	0	0	0	0	0
**Sulfobacillus**	1	30	0	0	0	0	0	0	0	0	0	0	0	0	0	0	0	0	0
**Sulfolobus**	1	23	0	0	0	0	0	0	0	0	0	0	0	0	0	0	0	0	0
**Sulfuricurvum**	2	67	1	0	0	1	0	0	1	0	0	0	0	0	0	0	0	0	0
**Sulfurihydrogenibium**	2	55	1	1	0	1	0	0	1	0	0	0	0	0	0	0	0	0	0
**Sulfurimonas**	2	60	1	0	0	1	0	0	1	0	0	0	0	0	0	0	0	0	0
**Sulfurospirillum**	1	48	1	0	0	1	0	0	0	0	0	0	0	0	0	0	0	0	0
**Sulfurovum**	2	48	0	0	0	1	0	0	1	0	0	0	0	0	0	0	0	0	0
**Symbiobacterium**	2	68	1	1	1	1	1	0	0	0	0	0	0	0	0	0	0	0	0
**Synechococcus**	1	34	0	0	0	0	0	0	0	0	0	0	0	0	0	0	0	0	0
**Synechocystis**	1	40	0	1	0	0	0	0	0	0	0	0	0	0	0	0	0	0	0
**Synergistetes**	1	27	0	1	0	1	0	0	0	0	0	0	0	0	0	0	0	0	0
**Syntrophobacter**	2	101	1	0	0	1	1	0	1	0	0	0	0	0	0	0	0	0	0
**Syntrophobotulus**	2	71	1	1	0	1	0	0	0	0	0	0	0	0	0	0	0	0	0
**Syntrophomonas**	2	58	1	0	1	1	1	0	0	0	0	0	0	0	0	0	0	0	0
**Syntrophothermus**	2	60	0	1	1	1	1	0	0	0	0	0	0	0	0	0	0	0	0
**Syntrophus**	2	66	0	1	0	1	1	0	1	0	0	0	0	0	0	0	0	0	0
**Tannerella**	1	8	0	0	0	0	0	0	0	0	0	0	0	0	0	0	0	0	0
**Taylorella**	1	43	0	0	0	1	0	0	0	0	0	0	0	0	0	0	0	0	0
**Tepidanaerobacter**	2	55	0	1	0	1	0	0	0	0	0	0	0	0	0	0	0	0	0
**Teredinibacter**	2	109	1	0	0	1	1	0	0	0	0	0	0	0	0	0	1	0	0
**Terrabacter**	1	1	0	0	0	0	0	0	0	0	0	0	0	0	0	0	0	0	0
**Terriglobus**	2	83	1	1	0	1	0	0	0	0	0	0	0	1	0	0	0	0	0
**Tetragenococcus**	1	12	0	0	0	0	0	0	0	0	0	0	0	0	0	0	0	0	0
**Thalassiobium**	1	11	0	0	0	0	0	0	0	0	0	0	0	0	0	0	0	0	0
**Thalassobacter**	1	9	0	0	0	0	0	0	0	0	0	0	0	0	0	0	0	0	0
**Thalassolituus**	1	12	0	0	0	0	0	0	0	0	0	0	0	0	0	0	0	0	0
**Thalassospira**	1	2	0	0	0	0	0	0	0	0	0	0	0	0	0	0	0	0	0
**Thauera**	1	111	0	1	1	1	0	0	0	0	0	1	0	0	0	0	1	0	0
**Thermacetogenium**	1	7	0	1	0	0	0	0	0	0	0	0	0	0	0	0	0	0	0
**Thermaerobacter**	2	79	1	1	0	1	1	0	0	0	0	1	0	0	0	0	0	0	0
**Thermanaerovibrio**	2	58	0	1	0	1	0	0	0	0	0	0	0	0	0	0	0	0	0
**Thermincola**	2	62	0	1	1	1	0	0	1	0	0	0	0	0	0	0	0	0	0
**Thermoanaerobacter**	2	76	1	1	1	1	0	0	0	0	0	0	0	0	0	0	0	0	0
**Thermoanaerobacterium**	2	64	1	1	1	1	0	0	0	0	0	0	0	0	0	0	0	0	0
**Thermobacillus**	1	11	0	0	0	0	0	0	0	0	0	0	0	0	0	0	0	0	0
**Thermobaculum**	2	62	0	1	1	1	0	0	0	0	0	0	0	0	0	0	0	0	0
**Thermobifida**	2	72	0	1	0	1	1	0	0	0	0	0	0	0	0	0	0	0	0
**Thermobispora**	2	109	1	1	0	1	0	0	1	0	0	0	1	1	0	1	0	0	0
**Thermococcus**	1	13	0	0	0	0	0	0	0	0	0	0	0	0	0	0	0	0	0
**Thermocrinis**	2	60	1	1	0	1	0	0	1	0	0	0	0	0	0	0	0	0	0
**Thermodesulfatator**	1	10	0	0	0	0	0	0	0	0	0	0	0	0	0	0	0	0	0
**Thermodesulfobacterium**	1	9	0	0	0	0	0	0	0	0	0	0	0	0	0	0	0	0	0

Genus	Cluster Number	Protein Counts	1.1.1.-	1.1.1.1	1.1.1.157	1.1.1.205	1.1.1.35	1.1.1.90	1.12.99.6	1.13.11.-	1.13.11.1	1.13.11.2	1.13.11.3	1.13.11.39	1.13.11.41	1.13.11.5	1.13.11.8	1.13.12.-	1.14.11.-
**Thermodesulfobium**	1	13	0	0	0	0	0	0	0	0	0	0	0	0	0	0	0	0	0
**Thermodesulfovibrio**	2	60	1	1	0	1	0	0	1	0	0	0	0	0	0	0	0	0	0
**Thermofilum**	1	9	0	0	0	0	0	0	0	0	0	0	0	0	0	0	0	0	0
**Thermogladius**	1	7	0	0	0	0	0	0	0	0	0	0	0	0	0	0	0	0	0
**Thermomicrobium**	2	92	0	1	0	1	0	0	1	0	0	1	0	0	0	0	0	0	0
**Thermomonospora**	2	125	0	1	0	1	1	0	0	0	0	1	1	1	0	1	0	0	0
**Thermoplasma**	1	11	0	0	0	0	0	0	0	0	0	0	0	0	0	0	0	0	0
**Thermoplasmatales**	1	6	0	0	0	0	0	0	0	0	0	0	0	0	0	0	0	0	0
**Thermoproteus**	1	12	0	0	0	0	0	0	0	0	0	0	0	0	0	0	0	0	0
**Thermosediminibacter**	2	64	0	1	0	1	0	0	0	0	0	0	0	0	0	0	0	0	0
**Thermosipho**	2	70	1	1	0	1	0	0	0	0	0	0	0	0	0	0	0	0	0
**Thermosphaera**	1	10	0	0	0	0	0	0	0	0	0	0	0	0	0	0	0	0	0
**Thermosynechococcus**	1	6	0	0	0	0	0	0	0	0	0	0	0	0	0	0	0	0	0
**Thermotoga**	2	80	1	1	0	1	0	0	0	0	0	0	0	0	0	0	0	0	0
**Thermovibrio**	2	58	0	1	0	1	0	0	0	0	0	0	0	0	0	0	0	0	0
**Thermovirga**	1	9	0	0	0	0	0	0	0	0	0	0	0	0	0	0	0	0	0
**Thermus**	2	107	1	1	1	1	1	0	0	0	0	1	0	0	0	0	0	0	0
**Thioalkalimicrobium**	1	4	0	0	0	0	0	0	0	0	0	0	0	0	0	0	0	0	0
**Thioalkalivibrio**	2	91	1	1	0	1	0	0	1	0	0	0	0	0	0	0	0	0	0
**Thiobaca**	1	2	0	0	0	0	0	0	0	0	0	0	0	0	0	0	0	0	0
**Thiobacillus**	2	67	1	1	0	1	0	0	1	0	0	0	0	0	0	0	0	0	0
**Thiocapsa**	1	2	0	0	0	0	0	0	0	0	0	0	0	0	0	0	0	0	0
**Thiocystis**	1	11	0	0	0	0	0	0	0	0	0	0	0	0	0	0	0	0	0
**Thioflavicoccus**	1	13	0	0	0	0	0	0	0	0	0	0	0	0	0	0	0	0	0
**Thiomicrospira**	2	50	0	1	0	1	0	0	0	0	0	0	0	0	0	0	0	0	0
**Thiomonas**	2	85	1	1	0	1	0	0	1	0	0	0	0	0	0	0	1	0	0
**Thiorhodococcus**	1	2	0	0	0	0	0	0	0	0	0	0	0	0	0	0	0	0	0
**Thiothrix**	1	2	0	0	0	0	0	0	0	0	0	0	0	0	0	0	0	0	0
**Tistrella**	1	42	0	0	0	0	0	0	0	0	0	0	0	0	0	0	0	0	0
**Tolumonas**	2	100	1	1	0	1	0	0	0	0	0	0	0	0	0	0	0	0	0
**Transposon**	1	2	0	0	0	0	0	0	0	0	0	0	0	0	0	0	0	0	0
**Treponema**	1	63	1	1	0	1	1	0	0	0	0	0	0	0	0	0	0	0	0
**Trichodesmium**	2	71	1	1	0	1	0	0	1	0	0	0	0	0	0	0	0	0	0
**Trichormus**	1	12	0	0	0	1	0	0	0	0	0	0	0	0	0	0	0	0	0
**Tropheryma**	2	22	0	0	0	1	0	0	0	0	0	0	0	0	0	0	0	0	0
**Truepera**	2	104	1	1	0	1	1	0	0	0	1	0	0	0	0	0	0	0	0
**Tsukamurella**	2	123	1	1	0	1	1	0	0	0	0	0	1	1	0	0	0	0	0
**Turneriella**	1	16	0	0	0	0	0	0	0	0	0	0	0	0	0	0	0	0	0
**Uliginosibacterium**	1	2	0	0	0	0	0	0	0	0	0	0	0	0	0	0	0	0	0
**Ureaplasma**	1	24	0	0	0	0	0	0	0	0	0	0	0	0	0	0	0	0	0
**Ureibacillus**	1	2	0	0	0	0	0	0	0	0	0	0	0	0	0	0	0	0	0
**Variovorax**	3	187	1	1	1	1	1	0	0	0	1	0	1	1	0	1	1	1	0
**Veillonella**	1	54	0	1	0	1	0	0	1	0	0	0	0	0	0	0	0	0	0
**Verminephrobacter**	3	188	1	1	1	1	1	0	0	0	1	1	0	0	0	0	1	0	0
**Verrucosispora**	3	126	1	1	1	1	1	0	0	0	0	0	1	1	0	1	0	0	0
**Vibrio**	2	195	1	1	0	1	1	0	0	1	0	0	1	0	0	1	0	0	0
**Vibrionales**	1	46	0	0	0	1	0	0	0	0	0	0	0	0	0	0	0	0	0
**Vitreoscilla**	1	1	0	0	0	0	0	0	0	0	0	0	0	0	0	0	0	0	0
**Vulcanisaeta**	1	14	0	0	0	0	0	0	0	0	0	0	0	0	0	0	0	0	0
**Waddlia**	2	68	1	1	0	1	1	0	0	0	0	0	0	0	0	0	0	0	0
**Wautersia**	1	2	0	0	0	0	0	0	0	0	0	0	0	0	0	0	0	0	0
**Weeksella**	2	65	1	0	1	1	1	0	0	0	0	0	0	0	0	1	1	0	0

Genus	Cluster Number	Protein Counts	1.1.1.-	1.1.1.1	1.1.1.157	1.1.1.205	1.1.1.35	1.1.1.90	1.12.99.6	1.13.11.-	1.13.11.1	1.13.11.2	1.13.11.3	1.13.11.39	1.13.11.41	1.13.11.5	1.13.11.8	1.13.12.-	1.14.11.-
Weissella	1	14	0	0	0	0	0	0	0	0	0	0	0	0	0	0	0	0	0
Wenxinia	1	2	0	0	0	0	0	0	0	0	0	0	0	0	0	0	0	0	0
Wigglesworthia	1	29	0	0	0	1	0	0	0	0	0	0	0	0	0	0	0	0	0
Wolbachia	1	32	1	0	0	1	0	0	0	0	0	0	1	0	0	0	0	0	0
Wolinella	2	51	1	0	0	1	0	0	0	0	0	0	0	0	0	0	0	0	0
Xanthobacter	3	176	1	1	1	1	1	0	1	1	1	0	1	1	0	1	0	1	0
Xanthobacteraceae	1	4	0	0	0	0	0	0	0	0	0	0	0	0	0	0	0	0	0
Xanthomonadaceae	1	39	1	1	0	1	0	0	0	0	0	0	0	0	0	1	0	0	0
Xanthomonas	2	162	1	1	1	1	1	0	0	0	0	0	1	0	0	1	1	0	0
Xenorhabdus	2	127	1	1	0	1	1	0	0	0	0	0	0	0	0	0	0	0	0
Xylanimonas	2	66	0	0	0	1	1	0	0	0	0	0	0	0	0	0	0	0	0
Xylella	2	52	0	1	0	1	0	0	0	0	0	0	0	0	0	0	0	0	0
Yersinia	2	145	1	1	0	1	1	0	1	0	0	0	0	0	0	0	0	0	0
Yokenella	2	61	1	1	0	1	0	0	0	0	0	0	0	0	0	0	0	0	0
Zobellia	1	28	0	0	0	1	0	0	0	0	0	0	0	0	0	0	0	0	0
Zunongwangia	2	98	1	1	1	1	1	0	0	0	0	0	0	0	0	1	0	0	0
Zymomonas	2	67	1	1	0	1	0	0	0	0	0	0	0	0	0	0	0	0	0
Zymophilus	1	2	0	0	0	0	0	0	0	0	0	0	0	0	0	0	0	0	0

Genus	Cluster Number	Protein Counts	1.14.12.10	1.14.12.13	1.14.12.18	1.14.12.3	1.14.12.7	1.14.13.-	1.14.13.1	1.14.13.2	1.14.13.20	1.14.13.22	1.14.13.40	1.14.13.50	1.14.13.7	1.14.13.8	1.14.13.82
**Acaricomes**	1	2	0	0	0	0	0	0	0	0	0	0	0	0	0	0	0
**Acaryochloris**	2	88	0	0	0	0	0	1	0	0	0	0	0	0	0	0	0
**Acetivibrio**	1	2	0	0	0	0	0	0	0	0	0	0	0	0	0	0	0
**Acetobacter**	2	85	1	0	0	0	0	1	1	0	0	0	0	0	0	0	1
**Acetobacteraceae**	1	2	0	0	0	0	0	0	0	0	0	0	0	0	0	0	0
**Acetobacterium**	1	8	0	0	0	0	0	0	0	0	0	0	0	0	0	0	0
**Acetohalobium**	2	79	0	0	0	0	0	1	0	0	0	0	0	0	0	0	0
**Acholeplasma**	2	38	0	0	0	0	0	0	0	0	0	0	0	0	0	0	0
**Achromobacter**	2	192	0	0	1	1	0	1	1	0	0	1	0	0	0	0	0
**Acidaminococcus**	1	65	0	0	0	0	0	0	0	0	0	0	0	0	0	0	0
**Acidianus**	1	9	0	0	0	0	0	0	0	0	0	0	0	0	0	0	0
**Acidilobus**	1	8	0	0	0	0	0	0	0	0	0	0	0	0	0	0	0
**Acidimicrobium**	2	58	0	0	0	0	0	0	0	0	0	0	0	0	0	0	0
**Acidiphilium**	3	144	0	0	0	0	0	1	0	1	0	1	0	0	0	0	0
**Acidithiobacillus**	2	70	0	0	0	0	0	1	0	0	0	0	0	0	0	0	0
**Acidobacterium**	2	91	0	0	0	0	0	1	1	0	0	0	0	0	0	0	0
**Acidocella**	1	2	0	0	0	0	0	0	0	0	0	0	0	0	0	0	0
**Acidothermus**	2	68	0	0	0	0	0	0	0	0	0	0	0	0	0	0	0
**Acidovorax**	3	217	0	0	0	0	0	1	1	0	0	1	1	0	1	0	0
**Aciduliprofundum**	1	11	0	0	0	0	0	0	0	0	0	0	0	0	0	0	0
**Acinetobacter**	3	188	1	0	1	0	0	1	1	1	0	1	0	0	1	0	1
**Actinobacillus**	2	112	0	0	0	0	0	1	0	0	0	0	0	0	0	0	0
**Actinomadura**	1	1	0	0	0	0	0	0	0	0	0	0	0	0	0	0	0
**Actinoplanes**	1	52	0	0	0	0	0	0	0	0	0	0	0	0	0	0	0
**Actinopolyspora**	1	2	0	0	0	0	0	0	0	0	0	0	0	0	0	0	0
**Actinosynnema**	2	122	0	0	0	0	0	1	0	1	0	1	0	0	0	0	0
**Advenella**	1	30	0	0	0	0	0	0	0	0	0	0	0	0	0	0	0
**Aequorivita**	1	12	0	0	0	0	0	0	0	0	0	0	0	0	0	0	0
**Aerococcus**	2	57	0	0	0	0	0	0	0	0	0	0	0	0	0	0	0
**Aeromonas**	2	127	0	0	0	0	0	1	0	0	0	0	0	0	0	0	0
**Aeropyrum**	1	13	0	0	0	0	0	0	0	0	0	0	0	0	0	0	0
**Afipia**	1	54	1	0	0	0	0	0	0	0	0	0	0	0	0	0	0
**Aggregatibacter**	1	81	0	0	0	0	0	1	0	0	0	0	0	0	0	0	0
**Agrobacterium**	2	242	1	0	0	0	0	1	1	1	0	0	0	0	0	0	1
**Ahrensia**	1	2	0	0	0	0	0	0	0	0	0	0	0	0	0	0	0
**Akkermansia**	2	47	0	0	0	0	0	0	0	0	0	0	0	0	0	0	0
**Albidiferax**	3	144	0	0	0	0	0	1	0	1	0	0	0	0	0	0	0
**Alcaligenes**	1	21	0	0	0	0	0	0	0	0	1	0	0	0	1	0	0
**Alcanivorax**	2	146	0	0	0	0	0	1	0	0	0	1	0	0	0	0	1
**Alicycliphilus**	3	152	0	0	0	0	0	1	0	0	0	0	0	0	1	0	0
**Alicyclobacillus**	2	91	0	0	0	0	0	1	0	1	0	0	0	0	1	0	0
**Aliivibrio**	2	118	0	0	0	0	0	1	0	0	0	0	0	0	0	0	0
**Alishewanella**	1	1	0	0	0	0	0	0	0	0	0	0	0	0	0	0	0
**Alistipes**	1	52	0	0	0	0	0	0	0	0	0	0	0	0	0	0	0
**Alkalilimnicola**	2	97	0	0	0	0	0	1	0	0	0	0	0	0	0	0	0
**Alkaliphilus**	2	62	0	0	0	0	0	0	0	0	0	0	0	0	0	0	1
**Allochromatium**	2	84	0	0	0	0	0	1	0	0	0	0	0	0	0	0	0
**Alphaproteobacteria**	1	3	0	0	0	0	0	0	0	0	0	0	0	0	0	0	0
**Alteromonadales**	1	12	0	0	0	0	0	0	0	0	0	0	0	0	0	0	0
**Alteromonas**	1	37	0	0	0	0	0	0	0	0	0	0	0	0	0	0	0
**Aminobacter**	1	4	0	0	0	0	0	0	0	0	0	0	0	0	0	0	0
**Aminobacterium**	2	59	0	0	0	0	0	0	0	0	0	0	0	0	0	0	0

Genus	Cluster Number	Protein Counts	1.14.12.10	1.14.12.13	1.14.12.18	1.14.12.3	1.14.12.7	1.14.13.-	1.14.13.1	1.14.13.2	1.14.13.20	1.14.13.22	1.14.13.40	1.14.13.50	1.14.13.7	1.14.13.8	1.14.13.82
**Ammonifex**	2	50	0	0	0	0	0	0	0	0	0	0	0	0	0	0	0
**Amphibacillus**	1	13	0	0	0	0	0	0	0	0	0	0	0	0	0	0	0
**Amphritea**	1	2	0	0	0	0	0	0	0	0	0	0	0	0	0	0	0
**Amycolatopsis**	2	201	1	0	0	0	0	1	1	1	0	1	1	0	0	0	0
**Amycolicicoccus**	1	43	0	0	0	0	0	0	0	0	0	0	0	0	0	0	0
**Anabaena**	2	92	0	0	0	0	0	1	0	0	0	0	0	0	0	0	0
**Anaerobaculum**	1	11	0	0	0	0	0	0	0	0	0	0	0	0	0	0	0
**Anaerococcus**	2	46	0	0	0	0	0	0	0	0	0	0	0	0	0	0	0
**Anaerolinea**	2	66	0	0	0	0	0	1	0	0	0	0	0	0	0	0	1
**Anaeromyxobacter**	3	117	0	0	0	0	0	1	1	0	0	0	0	0	0	0	0
**Anaerostipes**	1	44	0	0	0	0	0	0	0	0	0	0	0	0	0	0	0
**Anaerotruncus**	1	2	0	0	0	0	0	0	0	0	0	0	0	0	0	0	0
**Anaplasma**	1	35	0	0	0	0	0	1	0	0	0	0	0	0	0	0	0
**Ancylobacter**	1	3	0	0	0	0	0	0	0	0	0	0	0	0	0	0	0
**Aneurinibacillus**	1	1	0	0	0	0	0	0	0	0	0	0	0	0	0	0	0
**Angomonas**	1	1	0	0	0	0	0	0	0	0	0	0	0	0	0	0	0
**Anoxybacillus**	2	72	0	0	0	0	0	0	0	0	0	0	0	0	0	0	0
**Aquifex**	2	45	0	0	0	0	0	0	0	0	0	0	0	0	0	0	0
**Aquimarina**	1	2	0	0	0	0	0	0	0	0	0	0	0	0	0	0	0
**Arcanobacterium**	2	45	0	0	0	0	0	0	0	0	0	0	0	0	0	0	0
**Archaeoglobus**	1	14	0	0	0	0	0	0	0	0	0	0	0	0	0	0	0
**Arcobacter**	2	101	0	0	0	0	0	0	0	0	0	0	0	0	0	0	0
**Aromatoleum**	3	133	0	0	0	0	0	1	1	0	0	0	0	0	0	0	0
**Arsenophonus**	1	4	0	0	0	0	0	0	0	0	0	0	0	0	0	0	0
**Arthrobacter**	2	206	1	0	1	0	0	1	1	1	0	0	0	0	1	0	1
**Arthrospira**	2	67	0	0	0	0	0	1	0	0	0	0	0	0	0	0	0
**Aster**	1	10	0	0	0	0	0	0	0	0	0	0	0	0	0	0	0
**Asticcacaulis**	2	103	0	0	0	0	0	1	0	0	0	0	0	0	0	0	0
**Atopobium**	1	28	0	0	0	0	0	0	0	0	0	0	0	0	0	0	0
**Aurantimonas**	1	2	0	0	0	0	0	0	0	0	0	0	0	0	0	0	0
**Avibacterium**	1	17	0	0	0	0	0	0	0	0	0	0	0	0	0	0	0
**Azoarcus**	3	172	0	1	0	0	0	1	0	1	0	0	1	0	1	0	0
**Azorhizobium**	3	149	0	0	0	0	0	1	1	1	0	0	1	0	0	0	0
**Azorhizophilus**	1	4	0	0	0	0	0	0	0	0	0	0	0	0	0	0	0
**Azospira**	1	19	0	0	0	0	0	0	0	0	0	0	0	0	0	0	0
**Azospirillum**	2	198	1	0	1	0	0	1	0	1	0	0	0	0	0	0	1
**Azotobacter**	3	183	1	0	0	0	0	1	0	1	0	0	0	0	1	0	0
**Bacillales**	1	9	0	0	0	0	0	0	0	0	0	0	0	0	0	0	0
**Bacilli**	1	3	0	0	0	0	0	0	0	0	0	0	0	0	0	0	0
**Bacillus**	2	237	1	0	1	0	0	1	1	1	0	1	0	0	0	0	0
**Bacteria**	2	86	0	0	0	0	0	1	1	0	0	0	0	0	0	0	0
**Bacteriovorax**	2	98	0	0	0	0	0	0	0	0	0	0	0	0	0	0	0
**Bacteroidales**	2	59	0	0	0	0	0	0	0	0	0	0	0	0	0	0	0
**Bacteroides**	2	96	0	0	0	0	0	0	0	0	0	0	0	0	1	0	0
**Bacteroidetes**	1	2	0	0	0	0	0	0	0	0	0	0	0	0	0	0	0
**Bartonella**	2	50	0	0	0	0	0	1	1	0	0	0	0	0	0	0	0
**Baumannia**	2	24	0	0	0	0	0	0	0	0	0	0	0	0	0	0	0
**Bdellovibrio**	2	87	0	0	0	0	0	0	0	0	0	0	0	0	1	0	0
**Beijerinckia**	3	123	0	0	0	0	0	1	1	0	0	0	0	0	0	0	0
**Belliella**	1	14	0	0	0	0	0	0	0	0	0	0	0	0	0	0	0
**Beutenbergia**	2	82	0	0	0	0	0	1	0	0	0	0	0	0	0	0	0
**Bifidobacterium**	2	61	0	0	0	0	0	0	0	0	0	0	0	0	0	0	0

Genus	Cluster Number	Protein Counts	1.14.12.10	1.14.12.13	1.14.12.18	1.14.12.3	1.14.12.7	1.14.13.-	1.14.13.1	1.14.13.2	1.14.13.20	1.14.13.22	1.14.13.40	1.14.13.50	1.14.13.7	1.14.13.8	1.14.13.82
Bizionia	1	1	0	0	0	0	0	0	0	0	0	0	0	0	0	0	0
Blastococcus	1	39	0	0	0	0	0	0	0	0	0	0	0	0	0	0	0
Blattabacterium	1	23	0	0	0	0	0	0	0	0	0	0	0	0	0	0	0
Blautia	1	22	0	0	0	0	0	0	0	0	0	0	0	0	0	0	0
Bordetella	3	227	1	0	1	0	1	1	1	0	0	1	0	0	0	0	0
Borrelia	1	32	0	0	0	0	0	0	0	0	0	0	0	0	0	0	0
Brachybacterium	1	18	0	0	0	0	0	0	0	0	0	0	0	0	0	0	0
Brachymonas	1	4	0	0	0	0	0	0	0	0	0	0	0	0	0	0	0
Brachyspira	2	79	0	0	0	0	0	0	0	0	0	0	0	0	0	0	0
Bradyrhizobiaceae	1	20	1	0	0	0	0	0	0	0	0	0	0	0	0	0	0
Bradyrhizobium	3	254	0	0	0	0	0	1	1	1	0	1	1	0	0	0	1
Brenneria	1	20	0	0	0	0	0	0	0	0	0	0	0	0	0	0	0
Brevibacillus	2	122	0	0	0	0	0	1	0	0	0	0	0	0	0	0	0
Brevibacterium	1	4	0	0	0	0	0	0	0	0	0	0	0	0	0	0	0
Brevundimonas	2	105	0	0	0	0	0	1	0	1	0	1	0	0	0	0	0
Brucella	3	138	0	0	0	0	0	1	1	1	0	0	0	0	0	0	0
Buchnera	1	26	0	0	0	0	0	0	0	0	0	0	0	0	0	0	0
Burkholderia	3	424	1	1	1	1	1	1	1	1	1	1	1	1	1	0	1
Burkholderiaceae	3	143	1	0	0	0	0	1	1	0	0	0	0	0	1	0	1
Burkholderiales	1	24	0	0	0	0	0	1	0	0	0	0	0	0	1	0	0
Buttiauxella	1	2	0	0	0	0	0	0	0	0	0	0	0	0	0	0	0
Butyrivibrio	2	67	0	0	0	0	0	0	0	0	0	0	0	0	0	0	0
Caldanaerobacter	2	63	0	0	0	0	0	0	0	0	0	0	0	0	0	0	0
Caldicellulosiruptor	2	75	0	0	0	0	0	0	0	0	0	0	0	0	0	0	0
Caldilinea	1	23	0	0	0	0	0	0	0	0	0	0	0	0	0	0	0
Caldimonas	1	6	0	0	0	0	0	0	0	0	0	0	0	0	0	0	0
Caldisericum	1	10	0	0	0	0	0	0	0	0	0	0	0	0	0	0	0
Caldisphaera	1	7	0	0	0	0	0	0	0	0	0	0	0	0	0	0	0
Calditerrivibrio	2	74	0	0	0	0	0	0	0	0	0	0	0	0	0	0	0
Caldivirga	1	13	0	0	0	0	0	0	0	0	0	0	0	0	0	0	0
Calothrix	1	43	0	0	0	0	0	1	0	0	0	0	0	0	0	0	0
Calyptogena	2	34	0	0	0	0	0	1	0	0	0	0	0	0	0	0	0
Campylobacter	2	80	0	0	0	0	0	0	0	0	0	0	0	0	0	0	0
Candidatus	2	290	1	0	0	0	1	1	1	1	0	1	0	0	0	1	1
Capnocytophaga	1	50	0	0	0	0	0	0	0	0	0	0	0	0	0	0	0
Carbophilus	1	2	0	0	0	0	0	0	0	0	0	0	0	0	0	0	0
Carboxydibrachium	1	14	0	0	0	0	0	0	0	0	0	0	0	0	0	0	0
Carboxydothermus	2	68	0	0	0	0	0	0	0	0	0	0	0	0	0	0	0
Carica	1	2	0	0	0	0	0	0	0	0	0	0	0	0	0	0	0
Carnobacterium	2	71	0	0	0	0	0	1	0	0	0	0	0	0	0	0	0
Catenibacterium	1	2	0	0	0	0	0	0	0	0	0	0	0	0	0	0	0
Catenovulum	1	2	0	0	0	0	0	0	0	0	0	0	0	0	0	0	0
Catenulispora	3	159	1	0	0	0	0	1	1	1	0	1	1	0	0	0	0
Caulobacter	3	168	0	0	1	0	0	1	0	1	0	1	0	0	0	0	1
Cedecea	1	45	0	0	0	0	0	0	0	0	0	0	0	0	0	0	0
Cellulomonas	2	96	0	0	0	0	0	1	1	0	0	0	0	0	0	0	0
Cellulophaga	2	105	1	0	0	0	0	1	1	0	0	0	0	0	0	0	0
Cellulosilyticum	1	14	0	0	0	0	0	0	0	0	0	0	0	0	0	0	0
Cellvibrio	2	85	0	0	0	0	0	1	0	0	0	0	0	0	0	0	0
Cenarchaeum	1	7	0	0	0	0	0	0	0	0	0	0	0	0	0	0	0
Chamaesiphon	1	13	0	0	0	0	0	0	0	0	0	0	0	0	0	0	0
Chelativorans	3	136	0	0	0	0	0	1	1	1	0	0	0	0	0	0	0

Genus	Cluster Number	Protein Counts	1.14.12.10	1.14.12.13	1.14.12.18	1.14.12.3	1.14.12.7	1.14.13.-	1.14.13.1	1.14.13.2	1.14.13.20	1.14.13.22	1.14.13.40	1.14.13.50	1.14.13.7	1.14.13.8	1.14.13.82
Chelatococcus	1	2	0	0	0	0	0	0	0	0	0	0	0	0	0	0	0
Chitinophaga	2	98	0	0	0	0	0	0	0	1	0	0	0	0	0	0	0
Chlamydia	1	36	0	0	0	0	0	0	0	0	0	0	0	0	0	0	0
Chlamydomonas	1	3	0	0	0	0	0	0	0	0	0	0	0	0	0	0	1
Chlamydophila	1	31	0	0	0	0	0	0	0	0	0	0	0	0	0	0	0
Chlorobaculum	2	61	0	0	0	0	0	0	0	0	0	0	0	0	0	0	0
Chlorobium	2	89	0	0	0	0	0	0	0	0	0	0	0	0	0	0	0
Chlorobium/Pelodictyon	1	2	0	0	0	0	0	0	0	0	0	0	0	0	0	0	0
Chloroflexus	2	105	0	0	0	0	0	1	0	0	0	0	0	0	0	0	0
Chlorogloeopsis	1	9	0	0	0	0	0	0	0	0	0	0	0	0	0	0	0
Chloroherpeton	2	59	0	0	0	0	0	0	0	0	0	0	0	0	0	0	0
Chromobacterium	2	117	0	0	0	0	0	1	0	0	0	0	0	0	0	0	0
Chromohalobacter	2	145	1	0	0	0	0	1	1	1	0	0	0	0	0	0	1
Chroococcidiopsis	1	24	0	0	0	0	0	0	0	0	0	0	0	0	0	0	0
Chryseobacterium	2	104	0	0	0	0	0	1	1	1	0	0	0	0	0	0	1
Citreicella	1	13	0	0	0	0	0	0	0	0	0	0	0	0	0	0	0
Citrobacter	2	190	1	0	0	0	0	1	0	1	0	0	0	0	0	0	0
Clavibacter	2	83	0	0	0	0	0	0	1	0	0	0	0	0	0	0	0
Clonorchis	1	1	0	0	0	0	0	0	0	0	0	0	0	0	0	0	0
Clostridiales	2	77	0	0	0	0	0	0	0	0	0	0	0	0	0	0	0
Clostridium	2	191	0	0	0	0	0	1	0	0	0	0	0	0	0	0	0
Cohnella	1	2	0	0	0	0	0	0	0	0	0	0	0	0	0	0	0
Coleofasciculus	1	4	0	0	0	0	0	0	0	0	0	0	0	0	0	0	0
Collimonas	1	35	0	0	0	0	0	0	0	0	0	0	0	0	0	0	0
Colwellia	2	130	0	0	0	0	0	1	0	0	0	0	0	0	0	0	0
Comamonas	2	196	0	0	0	0	1	1	1	1	0	1	0	0	1	0	1
Conexibacter	3	117	0	0	0	0	0	1	0	0	0	1	1	0	0	0	0
Coprobacillus	1	2	0	0	0	0	0	0	0	0	0	0	0	0	0	0	0
Coprococcus	2	76	0	0	0	0	0	0	0	0	0	0	0	0	0	0	0
Coprothermobacter	2	45	0	0	0	0	0	0	0	0	0	0	0	0	0	0	0
Coraliomargarita	2	68	0	0	0	0	0	0	0	0	0	0	0	0	0	0	0
Corallococcus	1	29	0	0	0	0	0	0	0	0	0	0	0	0	0	0	0
Coriobacterium	2	36	0	0	0	0	0	0	0	0	0	0	0	0	0	0	0
Corynebacterineae	1	5	0	0	0	0	0	0	0	0	0	0	0	0	0	0	0
Corynebacterium	2	175	1	0	0	0	0	1	1	1	0	1	0	0	1	0	1
Coxiella	2	54	0	0	0	0	0	1	0	0	0	0	0	0	0	0	0
Crinalium	1	21	0	0	0	0	0	0	0	0	0	0	0	0	0	0	0
Croceibacter	2	80	0	0	0	0	0	1	0	0	0	0	0	0	0	0	0
Crocosphaera	1	6	0	0	0	0	0	0	0	0	0	0	0	0	0	0	0
Cronobacter	2	120	0	0	0	0	0	1	0	0	0	0	0	0	0	0	0
Cryptobacterium	2	39	0	0	0	0	0	0	0	0	0	0	0	0	0	0	0
Cucumis	1	9	0	0	0	0	0	0	0	0	0	0	0	0	0	0	0
Cupriavidus	3	317	1	0	1	0	1	1	1	1	1	1	0	0	1	0	1
Curtobacterium	1	5	0	0	0	0	0	0	0	0	0	0	0	0	0	0	0
Curvibacter	1	27	0	0	0	0	0	0	0	0	0	0	0	0	0	0	0
Cyanobacterium	1	9	0	0	0	0	0	0	0	0	0	0	0	0	0	0	0
Cyanobium	1	10	0	0	0	0	0	0	0	0	0	0	0	0	0	0	0
Cyanothece	1	34	0	0	0	0	0	0	0	0	0	0	0	0	0	0	0
Cyclobacterium	1	19	0	0	0	0	0	0	0	0	0	0	0	0	0	0	0
Cycloclasticus	1	21	0	0	0	0	0	0	0	0	0	0	0	0	0	0	0
Cylindrospermopsis	1	4	0	0	0	0	0	0	0	0	0	0	0	0	0	0	0
Cylindrospermum	1	30	0	0	0	0	0	1	0	0	0	0	0	0	0	0	0

Genus	Cluster Number	Protein Counts	1.14.12.10	1.14.12.13	1.14.12.18	1.14.12.3	1.14.12.7	1.14.13.-	1.14.13.1	1.14.13.2	1.14.13.20	1.14.13.22	1.14.13.40	1.14.13.50	1.14.13.7	1.14.13.8	1.14.13.82
Cytophaga	2	69	0	0	0	0	0	0	0	0	0	0	0	0	0	0	0
Dactylococcopsis	1	8	0	0	0	0	0	0	0	0	0	0	0	0	0	0	0
Dechloromonas	1	40	0	0	0	0	0	0	0	0	0	0	0	0	0	0	0
Dechlorosoma	1	19	0	0	0	0	0	0	0	0	0	0	0	0	0	0	0
Deferribacter	2	73	0	0	0	0	0	0	0	0	0	0	0	0	0	0	0
Dehalobacter	1	9	0	0	0	0	0	0	0	0	0	0	0	0	0	0	0
Dehalococcoides	2	41	0	0	0	0	0	0	0	0	0	0	0	0	0	0	0
Dehalogenimonas	1	37	0	0	0	0	0	0	0	0	0	0	0	0	0	0	0
Deinococcus	2	140	0	0	0	0	0	1	1	1	0	0	0	0	0	0	0
Delftia	3	177	0	0	0	0	0	1	1	1	0	0	0	0	0	0	1
Denitrovibrio	2	67	0	0	0	0	0	0	0	0	0	0	0	0	0	0	0
Desulfarculus	2	92	0	0	0	0	0	0	0	0	0	0	0	0	0	0	0
Desulfatibacillum	3	84	0	0	0	0	0	0	0	0	0	0	0	0	0	0	0
Desulfitobacterium	2	102	0	0	0	0	0	1	0	0	0	0	0	0	0	0	0
Desulfobacca	2	65	0	0	0	0	0	0	0	0	0	0	0	0	0	0	0
Desulfobacterium	2	113	0	0	0	0	0	0	0	0	0	0	0	0	0	0	0
Desulfobacula	1	21	0	0	0	0	0	0	0	0	0	0	0	0	0	0	0
Desulfobulbus	2	88	0	0	0	0	0	0	0	0	0	0	0	0	0	0	0
Desulfocapsa	1	9	0	0	0	0	0	0	0	0	0	0	0	0	0	0	0
Desulfococcus	2	80	0	0	0	0	0	0	0	0	0	0	0	0	0	0	0
Desulfohalobium	2	52	0	0	0	0	0	0	0	0	0	0	0	0	0	0	0
Desulfomicrobium	2	65	0	0	0	0	0	0	0	0	0	0	0	0	0	0	0
Desulfomonile	1	25	0	0	0	0	0	0	0	0	0	0	0	0	0	0	0
Desulforhabdus	1	2	0	0	0	0	0	0	0	0	0	0	0	0	0	0	0
Desulfosporosinus	1	30	0	0	0	0	0	0	0	0	0	0	0	0	0	0	0
Desulfotalea	2	66	0	0	0	0	0	0	0	0	0	0	0	0	0	0	0
Desulfotomaculum	2	98	0	0	0	0	0	1	0	0	0	0	0	0	0	0	0
Desulfovibrio	2	144	0	0	0	0	0	0	0	0	0	0	0	0	0	0	0
Desulfurispirillum	2	78	0	0	0	0	0	0	0	0	0	0	0	0	0	0	0
Desulfurivibrio	2	71	0	0	0	0	0	0	0	0	0	0	0	0	0	0	0
Desulfurobacterium	2	53	0	0	0	0	0	1	0	0	0	0	0	0	0	0	0
Desulfurococcus	1	11	0	0	0	0	0	0	0	0	0	0	0	0	0	0	0
Diaphorobacter	1	16	0	0	0	0	0	0	0	0	0	0	0	0	1	0	0
Dichelobacter	2	45	0	0	0	0	0	1	0	0	0	0	0	0	0	0	0
Dickeya	2	155	0	0	0	0	0	1	1	0	0	0	0	0	0	0	1
Dictyoglomus	2	62	0	0	0	0	0	0	0	0	0	0	0	0	0	0	0
Dinoroseobacter	3	148	0	0	0	0	0	1	1	1	0	1	0	0	0	0	0
Dokdonia	1	3	0	0	0	0	0	0	0	0	0	0	0	0	0	0	0
Dorea	1	7	0	0	0	0	0	0	0	0	0	0	0	0	0	0	0
Dyadobacter	2	83	0	0	0	0	0	0	1	0	0	0	0	0	0	0	0
Echinicola	1	20	0	0	0	0	0	0	0	0	0	0	0	0	0	0	0
Edwardsiella	2	126	0	0	0	0	0	1	0	0	0	0	0	0	0	1	0
Eggerthella	1	51	0	0	0	0	0	0	0	0	0	0	0	0	0	0	0
Ehrlichia	2	37	0	0	0	0	0	1	0	0	0	0	0	0	0	0	0
Eikenella	1	4	0	0	0	0	0	0	0	0	0	0	0	0	0	0	0
Elusimicrobium	2	44	0	0	0	0	0	0	0	0	0	0	0	0	0	0	0
Emticicia	1	21	0	0	0	0	0	0	0	0	0	0	0	0	0	0	0
Enhydrobacter	1	1	0	0	0	0	0	0	0	0	0	0	0	0	0	0	0
Ensifer	1	2	0	0	0	0	0	0	0	0	0	0	0	0	0	0	0
Enterobacter	2	202	1	0	0	0	0	1	1	0	0	0	0	0	0	0	1
Enterobacteriaceae	2	211	1	0	0	0	0	1	0	1	0	0	0	0	0	0	1
Enterococcus	1	187	1	0	0	0	0	1	0	1	0	0	0	0	0	0	1

Genus	Cluster Number	Protein Counts	1.14.12.10	1.14.12.13	1.14.12.18	1.14.12.3	1.14.12.7	1.14.13.-	1.14.13.1	1.14.13.2	1.14.13.20	1.14.13.22	1.14.13.40	1.14.13.50	1.14.13.7	1.14.13.8	1.14.13.82
Enterovibrio	1	15	0	0	0	0	0	0	0	0	0	0	0	0	0	0	0
Erwinia	2	151	1	0	0	0	0	1	1	1	0	0	0	0	0	1	1
Erysipelothrix	1	9	0	0	0	0	0	0	0	0	0	0	0	0	0	0	0
Erysipelotrichaceae	1	4	0	0	0	0	0	0	0	0	0	0	0	0	0	0	0
Erythrobacter	2	80	0	0	0	0	0	1	0	0	0	1	0	0	0	0	0
Escherichia	2	242	1	0	1	0	0	1	1	1	0	0	0	0	0	1	0
Ethanoligenens	2	69	0	0	0	0	0	0	0	0	0	0	0	0	0	0	0
Eubacteriaceae	1	2	0	0	0	0	0	0	0	0	0	0	0	0	0	0	0
Eubacterium	2	103	0	0	0	0	0	0	0	0	0	0	0	0	0	0	0
Eudoraea	1	4	0	0	0	0	0	0	0	0	0	0	0	0	0	0	0
Euryarchaeota	1	1	0	0	0	0	0	0	0	0	0	0	0	0	0	0	0
Exiguobacterium	2	93	0	0	0	0	0	1	0	0	0	0	0	0	0	0	0
Faecalibacterium	1	12	0	0	0	0	0	0	0	0	0	0	0	0	0	0	0
Ferrimonas	2	115	0	0	0	0	0	1	0	0	0	0	0	0	0	0	0
Ferroglobus	1	14	0	0	0	0	0	0	0	0	0	0	0	0	0	0	0
Ferroplasma	1	11	0	0	0	0	0	0	0	0	0	0	0	0	0	0	0
Fervidicoccus	1	7	0	0	0	0	0	0	0	0	0	0	0	0	0	0	0
Fervidobacterium	2	54	0	0	0	0	0	0	0	0	0	0	0	0	0	0	0
Fibrella	1	17	0	0	0	0	0	0	0	0	0	0	0	0	0	0	0
Fibrobacter	2	52	0	0	0	0	0	0	0	0	0	0	0	0	0	0	0
Filifactor	1	9	0	0	0	0	0	0	0	0	0	0	0	0	0	0	0
Finegoldia	2	55	0	0	0	0	0	0	0	0	0	0	0	0	0	0	0
Firmicutes	1	15	0	0	0	0	0	0	0	0	0	0	0	0	0	0	0
Fischerella	1	15	0	0	0	0	0	0	0	0	0	0	0	0	0	0	0
Flavobacteria	1	7	0	0	0	0	0	0	0	0	0	0	0	0	0	0	0
Flavobacteriaceae	2	69	0	0	0	0	0	0	0	0	0	0	0	0	0	0	0
Flavobacteriales	1	3	0	0	0	0	0	0	0	0	0	0	0	0	0	0	0
Flavobacterium	2	103	0	0	0	0	0	1	0	0	0	0	0	0	0	0	0
Flexibacter	1	17	0	0	0	0	0	0	0	0	0	0	0	0	0	0	0
Flexistipes	1	11	0	0	0	0	0	0	0	0	0	0	0	0	0	0	0
Fluoribacter	1	6	0	0	0	0	0	0	0	0	0	0	0	0	0	0	0
Fluviicola	2	69	0	0	0	0	0	1	0	0	0	0	0	0	0	0	0
Formosa	1	2	0	0	0	0	0	0	0	0	0	0	0	0	0	0	0
Francisella	2	88	0	0	0	0	0	1	0	0	0	0	0	0	1	0	0
Frankia	3	202	1	0	1	0	0	1	1	1	0	1	0	0	0	0	1
Frateuria	1	25	0	0	0	0	0	0	0	0	0	0	0	0	0	0	0
Fructobacillus	1	2	0	0	0	0	0	0	0	0	0	0	0	0	0	0	0
Fulvimarina	1	2	0	0	0	0	0	0	0	0	0	0	0	0	0	0	0
Fusobacterium	1	48	0	0	0	0	0	0	0	0	0	0	0	0	0	0	0
Gallibacterium	2	87	0	0	0	0	0	1	0	0	0	0	0	0	0	0	0
Gallionella	2	67	0	0	0	0	0	1	0	0	0	0	0	0	0	0	0
Gammaproteobacteria	1	9	0	0	0	0	0	0	0	0	0	0	0	0	0	0	1
Gardnerella	1	31	0	0	0	0	0	0	0	0	0	0	0	0	0	0	0
Geitlerinema	1	9	0	0	0	0	0	0	0	0	0	0	0	0	0	0	0
Gemmatimonas	2	93	0	0	0	0	0	1	1	0	0	0	1	0	0	0	0
Geobacillus	2	168	0	0	0	0	0	1	0	0	0	0	0	0	0	0	0
Geobacter	2	150	0	0	0	0	0	0	0	0	0	0	0	0	0	0	0
Geodermatophilus	2	136	1	0	0	0	0	1	1	1	0	1	0	0	0	0	1
Geopsychrobacter	1	2	0	0	0	0	0	0	0	0	0	0	0	0	0	0	0
Gillisia	1	11	0	0	0	0	0	0	0	0	0	0	0	0	0	0	0
Gilvimarinus	1	4	0	0	0	0	0	0	0	0	0	0	0	0	0	0	0
Glaciecola	2	117	0	0	0	0	0	1	1	0	0	0	0	0	0	0	0

Genus	Cluster Number	Protein Counts	1.14.12.10	1.14.12.13	1.14.12.18	1.14.12.3	1.14.12.7	1.14.13.-	1.14.13.1	1.14.13.2	1.14.13.20	1.14.13.22	1.14.13.40	1.14.13.50	1.14.13.7	1.14.13.8	1.14.13.82
Gloeobacter	2	67	0	0	0	0	0	0	0	0	0	0	0	0	0	0	0
Gloeocapsa	1	20	0	0	0	0	0	0	0	0	0	0	0	0	0	0	0
Gloeothece	1	1	0	0	0	0	0	0	0	0	0	0	0	0	0	0	0
Gluconacetobacter	2	96	0	0	0	0	0	1	0	0	0	0	0	0	0	0	0
Gluconobacter	2	76	0	0	0	0	0	1	0	0	0	0	0	0	0	0	1
Gordonia	1	158	1	0	0	0	0	1	1	0	0	1	0	0	0	0	0
Gordonibacter	1	32	0	0	0	0	0	0	0	0	0	0	0	0	0	0	0
Gramella	2	104	0	0	0	0	0	1	0	0	0	0	0	0	0	0	0
Granulibacter	2	90	0	0	0	0	0	1	0	0	0	0	0	0	0	0	1
Granulicella	2	98	0	0	0	0	0	1	0	0	0	0	0	0	0	0	0
Grimontia	1	9	0	0	0	0	0	0	0	0	0	0	0	0	0	0	0
Haemophilus	2	117	0	0	0	0	0	1	0	0	0	0	0	0	0	0	0
Hafnia	1	29	0	0	0	0	0	0	0	0	0	0	0	0	0	0	0
Hahella	3	130	0	0	0	0	0	1	0	0	0	1	0	0	0	0	0
Halalkalicoccus	1	18	0	0	0	0	0	0	0	0	0	0	0	0	0	0	0
Halanaerobium	2	89	0	0	0	0	0	0	0	0	0	0	0	0	0	0	0
Haliangium	2	108	0	0	0	0	0	0	0	0	0	1	1	0	0	0	1
Haliscomenobacter	2	107	0	0	0	0	0	1	1	1	0	0	0	0	0	0	0
Haloarcula	1	22	0	0	0	0	0	0	0	0	0	0	0	0	0	0	0
Halobacillus	1	19	0	0	0	0	0	0	0	0	0	0	0	0	0	0	0
Halobacteriaceae	1	1	0	0	0	0	0	0	0	0	0	0	0	0	0	0	0
Halobacterium	1	10	0	0	0	0	0	0	0	0	0	0	0	0	0	0	0
Halobacteroides	1	14	0	0	0	0	0	0	0	0	0	0	0	0	0	0	0
Haloferax	1	20	0	0	0	0	0	0	0	0	0	0	0	0	0	0	0
Halogeometricum	1	12	0	0	0	0	0	0	0	0	0	0	0	0	0	0	0
Halomicrobium	1	10	0	0	0	0	0	0	0	0	0	0	0	0	0	0	0
Halomonas	1	149	0	0	0	0	0	1	0	1	0	0	0	0	0	0	0
Halopiger	1	16	0	0	0	0	0	0	0	0	0	0	0	0	0	0	0
Haloquadratum	1	19	0	0	0	0	0	0	0	0	0	0	0	0	0	0	0
Halorhabdus	1	10	0	0	0	0	0	0	0	0	0	0	0	0	0	0	0
Halorhodospira	2	71	0	0	0	0	0	1	0	0	0	0	0	0	0	0	0
Halorubrum	1	16	0	0	0	0	0	0	0	0	0	0	0	0	0	0	0
Haloterrigena	1	19	0	0	0	0	0	0	0	0	0	0	0	0	0	0	0
Halothece	1	9	0	0	0	0	0	0	0	0	0	0	0	0	0	0	0
Halothermothrix	2	50	0	0	0	0	0	0	0	0	0	0	0	0	0	0	0
Halothiobacillus	2	65	0	0	0	0	0	1	0	0	0	0	0	0	0	0	0
Halovivax	1	12	0	0	0	0	0	0	0	0	0	0	0	0	0	0	0
Helicobacter	2	79	0	0	0	0	0	1	0	0	0	0	0	0	0	0	0
Heliobacillus	1	4	0	0	0	0	0	0	0	0	0	0	0	0	0	0	0
Heliobacterium	2	51	0	0	0	0	0	0	0	0	0	0	0	0	0	0	0
Herbaspirillum	2	172	1	0	1	0	0	1	1	1	0	0	0	0	0	0	1
Herminiimonas	3	89	1	0	0	0	0	1	0	0	0	0	0	0	0	0	0
Herpetosiphon	3	90	0	0	0	0	0	1	1	0	0	0	0	0	0	0	0
Hippea	2	44	0	0	0	0	0	0	0	0	0	0	0	0	0	0	0
Hirschia	2	102	0	0	0	0	0	1	1	0	0	0	0	0	0	0	0
Histophilus	2	72	0	0	0	0	0	1	0	0	0	0	0	0	0	0	0
Hoeflea	1	16	0	0	0	0	0	0	0	0	0	0	0	0	0	0	0
Hydrogenivirga	1	2	0	0	0	0	0	0	0	0	0	0	0	0	0	0	0
Hydrogenobacter	2	55	0	0	0	0	0	0	0	0	0	0	0	0	0	0	0
Hydrogenobaculum	2	44	0	0	0	0	0	0	0	0	0	0	0	0	0	0	0
Hydrogenophaga	1	9	0	0	0	0	0	0	0	0	0	0	0	0	0	0	0
Hylemonella	1	4	0	0	0	0	0	0	0	0	0	0	0	0	0	0	0

Genus	Cluster Number	Protein Counts	1.14.12.10	1.14.12.13	1.14.12.18	1.14.12.3	1.14.12.7	1.14.13.-	1.14.13.1	1.14.13.2	1.14.13.20	1.14.13.22	1.14.13.40	1.14.13.50	1.14.13.7	1.14.13.8	1.14.13.82
Hyperthermus	1	6	0	0	0	0	0	0	0	0	0	0	0	0	0	0	0
Hyphomicrobium	2	93	0	0	0	0	0	1	0	0	0	0	0	0	0	0	0
Hyphomonas	3	105	0	0	0	0	0	1	1	0	0	1	0	0	0	0	0
Ideonella	1	18	0	0	0	0	0	0	0	0	0	0	0	0	1	0	0
Idiomarina	2	86	0	0	0	0	0	1	0	0	0	0	0	0	0	0	0
Ignavibacterium	1	14	0	0	0	0	0	0	0	0	0	0	0	0	0	0	0
Ignicoccus	1	5	0	0	0	0	0	0	0	0	0	0	0	0	0	0	0
Ignisphaera	1	8	0	0	0	0	0	0	0	0	0	0	0	0	0	0	0
Ilyobacter	2	85	0	0	0	0	0	0	0	0	0	0	0	0	0	0	0
Intrasporangium	2	114	0	0	0	0	0	1	1	0	0	0	0	0	0	0	0
Isoptericola	1	18	0	0	0	0	0	0	0	0	0	0	0	0	0	0	0
Isosphaera	2	75	0	0	0	0	0	0	0	0	0	0	0	0	0	0	0
Jannaschia	3	151	0	0	0	0	0	1	1	1	0	0	1	0	0	0	1
Janthinobacterium	2	99	1	0	0	0	0	1	0	0	0	0	0	0	0	0	1
Jonesia	2	56	0	0	0	0	0	0	0	0	0	0	0	0	0	0	0
Joostella	1	3	0	0	0	0	0	0	0	0	0	0	0	0	0	0	0
Kaistia	1	2	0	0	0	0	0	0	0	0	0	0	0	0	0	0	0
Kangiella	2	84	0	0	0	0	0	1	0	0	0	0	0	0	0	0	0
Ketogulonicigenium	2	102	0	0	0	0	0	1	1	1	0	1	0	0	0	0	0
Kineococcus	1	24	0	0	0	0	0	0	0	0	0	0	0	0	0	0	0
Kinetoplastibacterium	1	2	0	0	0	0	0	0	0	0	0	0	0	0	0	0	0
Kingella	1	4	0	0	0	0	0	0	0	0	0	0	0	0	0	0	0
Kitasatospora	1	36	0	0	0	0	0	0	0	0	0	0	0	0	0	0	0
Klebsiella	3	222	1	0	0	0	0	1	1	1	0	0	0	0	0	0	1
Kluyvera	1	1	0	0	0	0	0	0	0	0	0	0	0	0	0	0	0
Kocuria	2	87	0	0	0	0	0	1	0	0	0	0	0	0	0	0	0
Kordia	1	5	0	0	0	0	0	0	0	0	0	0	0	0	0	0	0
Kosmotoga	2	61	0	0	0	0	0	1	0	0	0	0	0	0	0	0	0
Kribbella	2	99	0	0	0	0	0	1	0	0	0	1	0	0	0	0	0
Krokinobacter	2	90	0	0	0	0	0	1	0	0	0	0	0	0	0	0	0
Kyrpidia	2	113	0	0	1	0	0	1	0	0	0	0	0	0	1	0	0
Kytococcus	2	70	0	0	0	0	0	0	1	0	0	0	0	0	0	0	0
Labrenzia	1	4	0	0	0	0	0	0	0	0	0	0	0	0	0	0	0
Lachnoanaerobaculum	1	1	0	0	0	0	0	0	0	0	0	0	0	0	0	0	0
Lachnospiraceae	1	51	0	0	0	0	0	0	0	0	0	0	0	0	0	0	0
Lacinutrix	1	20	0	0	0	0	0	0	0	0	0	0	0	0	0	0	0
Lactobacillus	2	119	0	0	0	0	0	1	0	0	0	0	0	0	0	0	0
Lactococcus	2	82	0	0	0	0	0	1	0	0	0	0	0	0	0	0	0
Lamprocystis	1	2	0	0	0	0	0	0	0	0	0	0	0	0	0	0	0
Laribacter	2	80	0	0	0	0	0	1	0	0	0	0	0	0	0	0	0
Lawsonia	2	33	0	0	0	0	0	0	0	0	0	0	0	0	0	0	0
Leadbetterella	2	79	0	0	0	0	0	0	0	0	0	0	0	0	0	0	0
Leeuwenhoekiella	1	2	0	0	0	0	0	0	0	0	0	0	0	0	0	0	0
Legionella	2	117	1	0	0	0	0	1	0	0	0	0	0	0	0	0	0
Leifsonia	2	48	0	0	0	0	0	0	0	0	0	0	0	0	0	0	0
Leptolyngbya	1	10	0	0	0	0	0	0	0	0	0	0	0	0	0	0	0
Leptospira	2	76	0	0	0	0	0	1	0	0	0	1	0	0	0	0	0
Leptospirillum	1	10	0	0	0	0	0	0	0	0	0	0	0	0	0	0	0
Leptothrix	3	186	0	0	0	0	0	1	0	1	0	0	0	0	1	0	0
Leptotrichia	2	43	0	0	0	0	0	0	0	0	0	0	0	0	0	0	0
Leucobacter	1	2	0	0	0	0	0	0	0	0	0	0	0	0	0	0	0
Leuconostoc	2	64	0	0	0	0	0	0	0	0	0	0	0	0	0	0	0

Genus	Cluster Number	Protein Counts	1.14.12.10	1.14.12.13	1.14.12.18	1.14.12.3	1.14.12.7	1.14.13.-	1.14.13.1	1.14.13.2	1.14.13.20	1.14.13.22	1.14.13.40	1.14.13.50	1.14.13.7	1.14.13.8	1.14.13.82
Liberibacter	1	4	0	0	0	0	0	0	0	0	0	0	0	0	0	0	0
Limnohabitans	1	11	0	0	0	0	0	0	0	0	0	0	0	0	0	0	0
Listeria	2	77	0	0	0	0	0	1	0	0	0	0	0	0	0	0	0
Listeriaceae	1	6	0	0	0	0	0	0	0	0	0	0	0	0	0	0	0
Listonella	1	38	0	0	0	0	0	0	0	0	0	0	0	0	0	0	0
Loktanella	1	3	0	0	0	0	0	0	0	0	0	0	0	0	0	0	0
Lysinibacillus	2	97	0	0	0	0	0	1	0	0	0	0	0	0	0	0	0
Macrococcus	2	60	0	0	0	0	0	0	1	0	0	0	0	0	0	0	0
Magnetococcus	2	64	0	0	0	0	0	0	0	0	0	0	0	0	0	0	0
Magnetospirillum	2	113	0	0	0	0	0	1	0	1	0	0	0	0	0	0	0
Mahella	2	58	0	0	0	0	0	0	0	0	0	0	0	0	0	0	0
Mannheimia	2	85	0	0	0	0	0	0	0	0	0	0	0	0	0	0	0
Maribacter	3	87	0	0	0	0	0	0	0	0	0	0	0	0	0	0	0
Maricaulis	3	87	1	0	0	0	0	1	0	0	0	1	1	0	0	0	1
Marichromatium	1	2	0	0	0	0	0	0	0	0	0	0	0	0	0	0	0
Marinithermus	2	77	0	0	0	0	0	0	0	0	0	0	0	0	0	0	0
Marinitoga	1	15	0	0	0	0	0	0	0	0	0	0	0	0	0	0	0
Marinobacter	2	163	1	0	1	0	0	1	0	0	0	1	0	0	1	0	1
Marinomonas	3	192	1	0	1	0	0	1	1	1	0	1	0	0	0	0	1
Maritimibacter	1	7	0	0	0	0	0	0	0	0	0	0	0	0	0	0	0
Marivirga	2	96	0	0	0	0	0	1	1	0	0	0	0	0	0	0	0
Martelella	1	3	0	0	0	0	0	0	0	0	0	0	0	0	0	0	0
Marvinbryantia	1	1	0	0	0	0	0	0	0	0	0	0	0	0	0	0	0
Massilia	1	4	0	0	0	0	0	0	0	0	0	0	0	0	0	0	0
Mastigocladopsis	1	16	0	0	0	0	0	0	0	0	0	0	0	0	0	0	0
Megamonas	1	47	0	0	0	0	0	0	0	0	0	0	0	0	0	0	0
Megasphaera	1	14	0	0	0	0	0	0	0	0	0	0	0	0	0	0	0
Meiothermus	2	110	0	0	0	0	0	1	1	1	0	0	1	0	0	0	0
Melissococcus	2	46	0	0	0	0	0	0	0	0	0	0	0	0	0	0	0
Mesoflavibacter	1	6	0	0	0	0	0	0	0	0	0	0	0	0	0	0	0
Mesoplasma	1	23	0	0	0	0	0	0	0	0	0	0	0	0	0	0	0
Mesorhizobium	2	201	1	0	0	0	0	1	1	1	0	0	1	0	0	0	1
Mesotoga	1	14	0	0	0	0	0	0	0	0	0	0	0	0	0	0	0
Metallosphaera	1	18	0	0	0	0	0	0	0	0	0	0	0	0	0	0	0
Methanobacterium	1	7	0	0	0	0	0	0	0	0	0	0	0	0	0	0	0
Methanobrevibacter	1	9	0	0	0	0	0	0	0	0	0	0	0	0	0	0	0
Methanocaldococcus	1	6	0	0	0	0	0	0	0	0	0	0	0	0	0	0	0
Methanocella	1	15	0	0	0	0	0	0	0	0	0	0	0	0	0	0	0
Methanococcaceae	1	1	0	0	0	0	0	0	0	0	0	0	0	0	0	0	0
Methanococcoides	1	5	0	0	0	0	0	0	0	0	0	0	0	0	0	0	0
Methanococcus	1	9	0	0	0	0	0	0	0	0	0	0	0	0	0	0	0
Methanocorpusculum	1	7	0	0	0	0	0	0	0	0	0	0	0	0	0	0	0
Methanoculleus	1	8	0	0	0	0	0	0	0	0	0	0	0	0	0	0	0
Methanohalobium	1	5	0	0	0	0	0	0	0	0	0	0	0	0	0	0	0
Methanohalophilus	1	5	0	0	0	0	0	0	0	0	0	0	0	0	0	0	0
Methanolobus	1	5	0	0	0	0	0	0	0	0	0	0	0	0	0	0	0
Methanomethylovorans	1	5	0	0	0	0	0	0	0	0	0	0	0	0	0	0	0
Methanoplanus	1	7	0	0	0	0	0	0	0	0	0	0	0	0	0	0	0
Methanopyrus	1	4	0	0	0	0	0	0	0	0	0	0	0	0	0	0	0
Methanoregula	1	8	0	0	0	0	0	0	0	0	0	0	0	0	0	0	0
Methanosaeta	1	9	0	0	0	0	0	0	0	0	0	0	0	0	0	0	0
Methanosalsum	1	7	0	0	0	0	0	0	0	0	0	0	0	0	0	0	0

Genus	Cluster Number	Protein Counts	1.14.12.10	1.14.12.13	1.14.12.18	1.14.12.3	1.14.12.7	1.14.13.-	1.14.13.1	1.14.13.2	1.14.13.20	1.14.13.22	1.14.13.40	1.14.13.50	1.14.13.7	1.14.13.8	1.14.13.82
Methanosarcina	1	18	0	0	0	0	0	0	0	0	0	0	0	0	0	0	0
Methanosphaera	1	7	0	0	0	0	0	0	0	0	0	0	0	0	0	0	0
Methanosphaerula	1	8	0	0	0	0	0	0	0	0	0	0	0	0	0	0	0
Methanospirillum	1	6	0	0	0	0	0	0	0	0	0	0	0	0	0	0	0
Methanothermobacter	1	6	0	0	0	0	0	0	0	0	0	0	0	0	0	0	0
Methanothermococcus	1	5	0	0	0	0	0	0	0	0	0	0	0	0	0	0	0
Methanothermus	1	5	0	0	0	0	0	0	0	0	0	0	0	0	0	0	0
Methanotorris	1	5	0	0	0	0	0	0	0	0	0	0	0	0	0	0	0
Methylacidiphilum	2	48	0	0	0	0	0	0	0	0	0	0	0	0	0	0	1
Methylarcula	1	2	0	0	0	0	0	0	0	0	0	0	0	0	0	0	0
Methylibium	1	38	0	0	0	0	0	0	0	0	0	0	0	0	0	0	0
Methylobacillus	2	61	0	0	0	0	0	1	0	0	0	0	0	0	0	0	0
Methylobacterium	3	236	1	0	0	0	0	1	1	1	0	1	0	0	0	0	1
Methylocella	3	117	0	0	0	0	0	1	1	0	1	0	0	0	0	0	0
Methylococcus	2	80	0	0	0	0	0	1	0	0	0	0	0	0	0	0	0
Methylocystis	1	21	0	0	0	0	0	0	0	0	0	0	0	0	0	0	0
Methyloferula	1	2	0	0	0	0	0	0	0	0	0	0	0	0	0	0	0
Methylomicrobium	1	12	0	0	0	0	0	0	0	0	0	0	0	0	0	0	0
Methylomonas	1	15	0	0	0	0	0	0	0	0	0	0	0	0	0	0	0
Methylophaga	1	7	0	0	0	0	0	0	0	0	0	0	0	0	0	0	0
Methylophilus	1	2	0	0	0	0	0	0	0	0	0	0	0	0	0	0	0
Methylosinus	1	2	0	0	0	0	0	0	0	0	0	0	0	0	0	0	0
Methylotenera	2	72	0	0	0	0	0	1	0	0	0	0	0	0	0	0	0
Methyloversatilis	1	5	0	0	0	0	0	0	0	0	0	0	0	0	0	0	0
Methylovorus	2	66	0	0	0	0	0	1	0	0	0	0	0	0	0	0	0
Micavibrio	1	9	0	0	0	0	0	0	0	0	0	0	0	0	0	0	0
Microbacterium	2	115	0	0	0	0	0	1	1	1	0	1	0	0	0	0	1
Microchaete	1	23	0	0	0	0	0	0	0	0	0	0	0	0	0	0	0
Micrococcus	2	72	0	0	0	0	0	0	0	0	0	0	0	0	0	0	0
Microcoleus	1	17	0	0	0	0	0	0	0	0	0	0	0	0	0	0	0
Microcystis	2	67	0	0	0	0	0	1	0	0	0	0	0	0	0	0	0
Microlunatus	1	25	0	0	0	0	0	0	0	0	0	0	0	0	0	0	0
Micromonospora	2	115	0	0	0	0	0	1	0	0	0	0	0	0	0	0	0
Microvirga	1	6	0	0	0	0	0	0	0	0	0	0	0	0	0	0	0
Mobiluncus	1	41	0	0	0	0	0	0	0	0	0	0	0	0	0	0	0
Modestobacter	1	43	0	0	0	0	0	0	0	0	0	0	0	0	0	0	0
Moorea	1	2	0	0	0	0	0	0	0	0	0	0	0	0	0	0	0
Moorella	2	69	0	0	0	0	0	0	0	0	0	0	0	0	0	0	0
Moraxella	2	66	0	0	0	0	0	1	0	0	0	0	0	0	0	0	0
Morganella	1	29	0	0	0	0	0	0	0	0	0	0	0	0	0	0	0
Moritella	1	2	0	0	0	0	0	0	0	0	0	0	0	0	0	0	0
Muricauda	1	23	0	0	0	0	0	0	0	0	0	0	0	0	0	0	0
Mycobacterium	3	306	1	0	1	0	0	1	1	0	0	1	0	0	0	0	1
Mycoplasma	1	53	0	0	0	0	0	1	0	0	0	0	0	0	0	0	0
Myxococcus	2	110	0	0	0	0	0	1	0	0	0	1	0	0	0	0	0
Nakamurella	2	115	0	0	0	0	0	1	0	1	0	0	0	0	0	0	0
napthalene	1	2	0	0	0	0	0	0	0	0	0	0	0	0	0	0	0
Natranaerobius	2	55	0	0	0	0	0	0	0	0	0	0	0	0	0	0	0
Natrialba	1	16	0	0	0	0	0	0	0	0	0	0	0	0	0	0	0
Natrinema	1	16	0	0	0	0	0	0	0	0	0	0	0	0	0	0	0
Natronobacterium	1	11	0	0	0	0	0	0	0	0	0	0	0	0	0	0	0
Natronococcus	1	21	0	0	0	0	0	0	0	0	0	0	0	0	0	0	0

Genus	Cluster Number	Protein Counts	1.14.12.10	1.14.12.13	1.14.12.18	1.14.12.3	1.14.12.7	1.14.13.-	1.14.13.1	1.14.13.2	1.14.13.20	1.14.13.22	1.14.13.40	1.14.13.50	1.14.13.7	1.14.13.8	1.14.13.82
Natronomonas	1	20	0	0	0	0	0	0	0	0	0	0	0	0	0	0	0
Nautilia	2	48	0	0	0	0	0	1	0	0	0	0	0	0	0	0	0
Neisseria	2	67	1	0	0	0	0	1	0	0	0	0	0	0	0	0	0
Neisseriaceae	1	1	0	0	0	0	0	0	0	0	0	0	0	0	0	0	0
Neorickettsia	1	31	0	0	0	0	0	1	0	0	0	0	0	0	0	0	0
Neptuniibacter	1	2	0	0	0	0	0	0	0	0	0	0	0	0	0	0	0
Niastella	1	21	0	0	0	0	0	0	0	0	0	0	0	0	0	0	0
Nitratifractor	2	54	0	0	0	0	0	0	0	0	0	0	0	0	0	0	0
Nitratireductor	1	19	0	0	0	0	0	0	0	0	0	0	0	0	0	0	0
Nitratiruptor	2	40	0	0	0	0	0	0	0	0	0	0	0	0	0	0	0
Nitrobacter	2	99	0	0	0	0	0	1	1	0	0	0	0	0	0	0	0
Nitrococcus	1	2	0	0	0	0	0	0	0	0	0	0	0	0	0	0	0
Nitrosococcus	2	94	0	0	0	0	0	1	0	0	0	0	0	0	0	0	0
Nitrosomonas	2	72	0	0	0	0	0	1	0	0	0	0	0	0	0	0	0
Nitrosopumilaceae	1	7	0	0	0	0	0	0	0	0	0	0	0	0	0	0	0
Nitrosopumilus	1	11	0	0	0	0	0	0	0	0	0	0	0	0	0	0	0
Nitrosospira	2	74	0	0	0	0	0	1	0	0	0	1	0	0	0	0	0
Nocardia	2	185	1	0	0	0	0	1	1	0	0	1	1	0	0	0	0
Nocardioidaceae	1	2	1	0	0	0	0	0	0	0	0	0	0	0	0	0	0
Nocardioides	2	157	1	0	0	0	0	1	0	0	1	1	0	0	0	0	0
Nocardiopsis	2	102	0	0	0	0	0	1	0	0	0	1	0	0	0	0	0
Nodularia	1	22	0	0	0	0	0	0	0	0	0	0	0	0	0	0	0
Nonlabens	1	19	0	0	0	0	0	0	0	0	0	0	0	0	0	0	0
Nostoc	2	111	0	0	0	0	0	1	0	0	0	1	0	0	0	0	0
Nostocaceae	1	2	0	0	0	0	0	0	0	0	0	0	0	0	0	0	0
Novosphingobium	2	169	1	1	1	0	0	1	0	1	0	1	0	0	0	0	0
Oceanibaculum	1	3	0	0	0	0	0	0	0	0	0	0	0	0	0	0	0
Oceanibulbus	1	12	0	0	0	0	0	0	0	0	0	0	0	0	0	0	1
Oceanicola	1	9	0	0	0	0	0	0	0	0	0	0	0	0	0	0	0
Oceanimonas	1	33	0	0	0	0	0	0	0	0	0	0	0	0	0	0	0
Oceaniovalibus	1	2	0	0	0	0	0	0	0	0	0	0	0	0	0	0	0
Oceanithermus	2	72	0	0	0	0	0	0	0	0	0	0	0	0	0	0	0
Oceanobacillus	2	91	0	0	0	0	0	1	0	0	0	0	0	0	0	0	0
Oceanospirillum	1	2	0	0	0	0	0	0	0	0	0	0	0	0	0	0	0
Ochrobactrum	2	133	0	0	0	0	0	1	1	1	0	0	1	0	0	0	0
Octadecabacter	1	29	0	0	0	0	0	0	0	0	0	0	0	0	0	0	0
Oenococcus	2	41	0	0	0	0	0	0	0	0	0	0	0	0	0	0	0
Oleomonas	1	2	0	0	0	0	0	0	0	0	0	0	0	0	1	0	0
Oligotropha	3	87	0	0	0	0	0	1	1	0	0	0	0	0	0	0	0
Olsenella	2	40	0	0	0	0	0	0	0	0	0	0	0	0	0	0	0
Opitutus	2	67	0	0	0	0	0	0	0	0	0	0	0	0	0	0	0
Orientia	1	21	0	0	0	0	0	1	0	0	0	0	0	0	0	0	0
Ornithobacterium	1	11	0	0	0	0	0	0	0	0	0	0	0	0	0	0	0
Oscillatoria	1	16	0	0	0	0	0	0	0	0	0	0	0	0	0	0	0
Oscillatoriales	1	2	0	0	0	0	0	0	0	0	0	0	0	0	0	0	0
Oscillibacter	1	20	0	0	0	0	0	0	0	0	0	0	0	0	0	0	0
Owenweeksia	1	15	0	0	0	0	0	0	0	0	0	0	0	0	0	0	0
Oxalobacteraceae	1	4	0	0	0	0	0	0	0	0	0	0	0	0	0	0	0
Paenibacillus	2	152	0	0	0	0	0	1	0	0	0	0	0	0	0	0	1
Paludibacter	2	67	0	0	0	0	0	0	0	0	0	0	0	0	0	0	0
Pandoraea	1	18	0	0	0	0	0	1	0	0	0	0	0	0	0	0	0
Pannonibacter	1	3	0	0	0	0	0	0	0	0	0	0	0	0	0	0	0

Genus	Cluster Number	Protein Counts	1.14.12.10	1.14.12.13	1.14.12.18	1.14.12.3	1.14.12.7	1.14.13.-	1.14.13.1	1.14.13.2	1.14.13.20	1.14.13.22	1.14.13.40	1.14.13.50	1.14.13.7	1.14.13.8	1.14.13.82
Pantholops	1	23	1	0	0	0	0	0	0	0	0	0	0	0	0	0	0
Pantoea	2	181	0	0	0	0	0	1	1	0	0	0	0	0	0	0	1
Parabacteroides	2	64	0	0	0	0	0	0	0	0	0	0	0	0	0	0	0
Parachlamydia	1	11	0	0	0	0	0	0	0	0	0	0	0	0	0	0	0
Paracoccus	2	169	1	0	0	0	0	1	1	1	0	0	1	0	0	0	0
Parvibaculum	3	117	1	0	0	0	0	1	0	0	0	1	0	0	0	0	0
Parvularcula	2	72	0	0	0	0	0	1	0	0	0	0	0	0	0	0	0
Pasteurella	2	85	0	0	0	0	0	1	0	0	0	0	0	0	0	0	0
Pasteurellaceae	1	2	0	0	0	0	0	0	0	0	0	0	0	0	0	0	0
Pectobacterium	1	44	0	0	0	0	0	0	0	0	0	0	0	0	0	0	0
Pediococcus	1	43	0	0	0	0	0	0	0	0	0	0	0	0	0	0	0
Pedobacter	2	88	0	0	0	0	0	0	0	0	0	0	0	0	0	0	0
Pelagibaca	1	10	0	0	0	0	0	0	0	0	0	0	0	0	0	0	0
Pelagibacterium	1	39	0	0	0	0	0	0	0	0	0	0	0	0	0	0	0
Pelobacter	2	85	0	0	0	0	0	0	0	0	0	0	0	0	0	0	0
Pelodictyon	2	62	0	0	0	0	0	0	0	0	0	0	0	0	0	0	0
Pelosinus	1	1	0	0	0	0	0	0	0	0	0	0	0	0	0	0	0
Pelotomaculum	2	60	0	0	0	0	0	0	0	0	0	0	0	0	0	0	0
Peptostreptococcus	1	1	0	0	0	0	0	0	0	0	0	0	0	0	0	0	0
Persephonella	2	55	0	0	0	0	0	1	0	0	0	0	0	0	0	0	0
Petrotoga	2	72	0	0	0	0	0	0	0	0	0	0	0	0	0	0	0
Phaeobacter	2	55	0	0	0	0	0	0	0	0	0	0	0	0	0	0	0
Phenylobacterium	3	103	0	0	0	0	0	1	0	0	0	1	0	0	0	0	0
Photobacterium	2	119	0	0	0	0	0	1	0	0	0	0	0	0	0	0	0
Photorhabdus	2	149	1	0	0	0	0	1	1	0	0	1	0	0	0	0	0
Phycisphaera	1	9	0	0	0	0	0	0	0	0	0	0	0	0	0	0	0
Phyllobacterium	1	6	0	0	0	0	0	0	0	0	0	0	0	0	0	0	0
Picrophilus	1	15	0	0	0	0	0	0	0	0	0	0	0	0	0	0	0
Pirellula	2	85	0	0	0	0	0	0	0	0	0	0	0	0	0	0	0
Planctomyces	2	93	0	0	0	0	0	0	0	0	0	1	0	0	0	0	0
Plautia	1	17	0	0	0	0	0	0	0	0	0	0	0	0	0	0	0
Plesiomonas	1	4	0	0	0	0	0	0	0	0	0	0	0	0	0	0	0
Pleurocapsa	1	23	0	0	0	0	0	0	0	0	0	0	0	0	0	0	0
Polaribacter	2	74	0	0	0	0	0	0	0	0	0	0	0	0	0	0	0
Polaromonas	3	238	1	0	1	0	1	1	1	1	0	1	0	0	0	0	1
Polymorphum	3	173	0	0	1	0	0	1	1	1	0	0	1	0	0	0	1
Polynucleobacter	1	19	0	0	0	0	0	0	0	0	0	0	0	0	0	0	0
Ponticaulis	1	1	0	0	0	0	0	0	0	0	0	0	0	0	0	0	0
Porphyromonas	2	63	0	0	0	0	0	0	0	0	0	0	0	0	0	0	0
Prevotella	1	65	1	0	0	0	0	0	0	0	0	0	0	0	0	0	0
Prochlorococcus	2	68	0	0	0	0	0	1	0	0	0	0	0	0	0	0	0
Propionibacterium	1	62	0	0	0	0	0	0	0	0	0	0	0	0	0	0	0
Prosthecochloris	1	16	0	0	0	0	0	0	0	0	0	0	0	0	0	0	0
Proteobacteria	1	19	0	0	1	0	0	0	0	0	0	0	0	0	0	0	0
Proteus	2	113	0	0	0	0	0	1	0	0	0	0	0	0	0	0	0
Providencia	1	38	0	0	0	0	0	0	0	0	0	0	0	0	0	0	0
Pseudaminobacter	1	10	0	0	0	0	0	0	0	0	0	0	0	0	0	0	0
Pseudanabaena	1	11	0	0	0	0	0	0	0	0	0	0	0	0	0	0	0
Pseudoalteromonas	2	188	0	0	0	0	0	1	1	1	0	0	0	0	0	0	1
Pseudochrobactrum	1	7	0	0	0	0	0	0	0	0	0	0	0	0	0	0	0
Pseudogulbenkiania	1	40	0	0	0	0	0	0	0	0	0	0	0	0	0	0	0
pseudomallei	3	131	1	0	0	0	0	1	0	0	0	1	0	0	0	0	1

Genus	Cluster Number	Protein Counts	1.14.12.10	1.14.12.13	1.14.12.18	1.14.12.3	1.14.12.7	1.14.13.-	1.14.13.1	1.14.13.2	1.14.13.20	1.14.13.22	1.14.13.40	1.14.13.50	1.14.13.7	1.14.13.8	1.14.13.82
Pseudomonas	3	381	1	1	1	0	0	1	1	1	1	1	0	0	1	0	1
Pseudonocardia	3	180	1	0	0	0	1	1	1	1	0	1	1	0	1	0	1
Pseudorhodobacter	1	4	0	0	0	0	0	0	0	0	0	0	0	0	0	0	0
Pseudovibrio	1	33	0	0	0	0	0	0	0	0	0	0	0	0	0	0	0
Pseudoxanthomonas	2	124	0	0	1	0	0	1	0	0	0	0	0	0	0	0	0
Psychrobacter	2	124	1	0	0	0	0	1	0	0	0	0	0	0	0	0	0
Psychroflexus	1	22	0	0	0	0	0	0	0	0	0	0	0	0	0	0	0
Psychromonas	2	110	0	0	0	0	0	1	0	0	0	0	0	0	0	0	0
Pusillimonas	3	111	0	0	0	0	0	1	0	0	0	0	0	0	0	0	1
Pyrobaculum	1	15	0	0	0	0	0	0	0	0	0	0	0	0	0	0	0
Pyrococcus	1	10	0	0	0	0	0	0	0	0	0	0	0	0	0	0	0
Pyrolobus	1	4	0	0	0	0	0	0	0	0	0	0	0	0	0	0	0
Rahnella	2	143	0	0	0	0	0	1	1	0	0	0	0	0	0	0	0
Ralstonia	3	332	1	0	1	0	1	1	1	1	1	1	1	0	1	0	1
Ramlibacter	1	35	0	0	0	0	0	0	0	0	0	0	0	0	0	0	0
Raoultella	2	139	1	0	0	0	0	1	0	0	0	0	0	0	0	0	1
Raphidiopsis	1	5	0	0	0	0	0	0	0	0	0	0	0	0	0	0	0
Reinekea	1	2	0	0	0	0	0	0	0	0	0	0	0	0	0	0	0
Renibacterium	2	67	0	0	0	0	0	1	1	0	0	1	0	0	0	0	0
Rhizobiaceae	1	3	0	0	0	0	0	0	0	0	0	0	0	0	0	0	0
Rhizobiales	1	6	0	0	0	0	0	0	0	0	0	0	0	0	0	0	0
Rhizobium	3	257	1	0	0	0	0	1	1	1	1	0	0	0	0	0	1
Rhizobium/Agrobacterium	2	87	0	0	0	0	0	1	0	0	0	0	0	0	0	0	0
Rhodanobacter	1	21	0	0	0	0	0	0	0	0	0	0	0	0	0	0	0
Rhodobacter	3	175	1	0	0	0	0	1	1	0	0	0	0	0	0	0	0
Rhodobacteraceae	1	21	0	0	0	0	0	0	0	0	0	0	0	0	0	0	0
Rhodobacterales	1	34	0	0	0	0	0	0	0	0	0	0	0	0	0	0	0
Rhodococcus	2	292	1	0	1	0	0	1	1	1	0	1	0	0	1	0	1
Rhodocyclaceae	1	7	0	0	0	0	0	0	0	0	0	0	0	0	0	0	0
Rhodoferax	3	144	0	0	0	0	0	1	0	1	0	0	0	0	0	0	0
Rhodomicrobium	2	103	0	0	0	0	0	1	1	0	0	0	0	0	0	0	0
Rhodopirellula	2	87	0	0	0	0	0	0	0	0	0	0	0	0	0	0	0
Rhodopseudomonas	3	246	1	0	1	0	0	1	1	1	0	1	0	0	0	0	1
Rhodospirillum	2	121	0	0	0	0	0	1	0	0	0	1	0	0	0	0	0
Rhodothermus	2	76	0	0	0	0	0	0	0	0	0	0	0	0	0	0	0
Rhodovulum	1	4	0	0	0	0	0	0	0	0	0	0	0	0	0	0	0
Richelia	1	2	0	0	0	0	0	0	0	0	0	0	0	0	0	0	0
Rickettsia	2	43	0	0	0	0	0	1	0	0	0	0	0	0	0	0	0
Rickettsiaceae	1	5	0	0	0	0	0	0	0	0	0	0	0	0	0	0	0
Riemerella	2	61	0	0	0	0	0	0	1	0	0	0	0	0	0	0	0
Rivularia	1	18	0	0	0	0	0	0	0	0	0	0	0	0	0	0	0
Robiginitalea	2	83	0	0	0	0	0	1	0	0	0	0	0	0	0	0	0
Roseburia	2	58	0	0	0	0	0	0	0	0	0	0	0	0	0	0	0
Roseibium	1	3	0	0	0	0	0	0	0	0	0	0	0	0	0	0	0
Roseiflexus	3	100	0	0	0	0	0	1	0	0	0	0	0	0	0	0	0
Roseobacter	2	149	1	0	0	0	0	1	1	1	0	0	0	0	0	0	1
Roseomonas	1	2	0	0	0	0	0	0	0	0	0	0	0	0	0	0	0
Roseovarius	1	15	0	0	0	0	0	0	0	0	0	0	0	0	0	0	0
Rothia	1	44	0	0	0	0	0	0	0	0	0	0	0	0	0	0	0
Rubrivivax	1	24	0	0	0	0	0	0	0	0	0	0	0	0	0	0	0
Rubrobacter	3	117	0	0	0	0	0	1	1	1	0	0	1	0	0	0	0
Ruegeria	2	189	1	0	0	0	0	1	1	1	0	0	1	0	0	0	0

Genus	Cluster Number	Protein Counts	1.14.12.10	1.14.12.13	1.14.12.18	1.14.12.3	1.14.12.7	1.14.13.-	1.14.13.1	1.14.13.2	1.14.13.20	1.14.13.22	1.14.13.40	1.14.13.50	1.14.13.7	1.14.13.8	1.14.13.82
Ruminococcaceae	1	2	0	0	0	0	0	0	0	0	0	0	0	0	0	0	0
Ruminococcus	2	101	0	0	0	0	0	0	0	0	0	0	0	0	0	0	0
Runella	1	26	0	0	0	0	0	0	0	0	0	0	0	0	0	0	0
Saccharomonospora	2	132	1	0	0	0	0	1	0	1	0	1	0	0	0	0	1
Saccharophagus	2	81	0	0	0	0	0	1	0	0	0	0	0	0	0	0	0
Saccharopolyspora	3	194	1	0	0	0	0	1	1	1	0	1	1	0	0	0	1
Saccharothrix	1	32	0	0	0	0	0	0	0	0	0	0	0	0	0	0	0
Sagittula	1	10	0	0	0	0	0	0	0	0	0	0	0	0	0	0	0
Salinibacter	2	77	0	0	0	0	0	0	0	0	0	1	0	0	0	0	0
Salinimonas	1	1	0	0	0	0	0	0	0	0	0	0	0	0	0	0	0
Salinispora	3	127	0	0	0	0	0	1	1	0	0	0	0	0	0	0	0
Salinivibrio	1	2	0	0	0	0	0	0	0	0	0	0	0	0	0	0	0
Salmonella	2	151	0	0	0	0	0	1	0	0	0	0	0	0	0	0	0
Sanguibacter	2	73	0	0	0	0	0	1	0	0	0	1	0	0	0	0	0
Saprospira	1	13	0	0	0	0	0	0	0	0	0	0	0	0	0	0	0
Scardovia	1	1	0	0	0	0	0	0	0	0	0	0	0	0	0	0	0
Scytonema	1	10	0	0	0	0	0	1	0	0	0	0	0	0	0	0	0
Sebaldella	2	69	0	0	0	0	0	0	0	0	0	0	0	0	0	0	0
Segniliparus	2	85	0	0	0	0	0	1	1	0	0	1	0	0	0	0	0
Selenomonas	2	57	0	0	0	0	0	0	0	0	0	0	0	0	0	0	0
Serinicoccus	1	1	0	0	0	0	0	0	0	0	0	0	0	0	0	0	0
Serratia	2	186	0	0	0	0	0	1	1	1	0	0	0	0	0	0	1
Shewanella	3	214	0	0	0	0	0	1	0	0	0	1	0	0	0	0	0
Shigella	2	172	1	0	0	0	0	1	0	0	0	0	0	0	0	0	0
Shinella	1	2	0	0	0	0	0	0	0	0	0	0	0	0	0	0	0
Sideroxydans	2	81	0	0	0	0	0	1	0	0	0	0	0	0	0	0	0
Silicibacter	2	105	0	0	0	0	0	1	0	1	0	0	1	0	0	0	0
Simiduia	1	18	0	0	0	0	0	0	0	0	0	0	0	0	0	0	0
Simkania	1	6	0	0	0	0	0	0	0	0	0	0	0	0	0	0	0
Simonsiella	1	2	0	0	0	0	0	0	0	0	0	0	0	0	0	0	0
Singulisphaera	1	18	0	0	0	0	0	0	0	0	0	0	0	0	0	0	0
Sinorhizobium	3	207	1	0	0	0	0	1	1	1	0	1	0	0	0	0	0
Slackia	2	46	0	0	0	0	0	0	0	0	0	0	0	0	0	0	0
Smaragdicoccus	1	3	0	0	0	0	0	0	0	0	0	0	0	0	0	0	0
Sodalis	2	71	0	0	0	0	0	1	0	0	0	0	0	0	0	0	0
Solibacillus	1	23	0	0	0	0	0	0	0	0	0	0	0	0	0	0	0
Solitalea	1	13	0	0	0	0	0	0	0	0	0	0	0	0	0	0	0
Sorangium	3	115	0	0	0	0	0	1	0	0	0	1	0	0	0	0	0
Sphaerobacter	2	100	0	0	0	0	0	0	0	1	0	0	0	0	0	0	0
Sphaerochaeta	1	42	0	0	0	0	0	0	0	0	0	0	0	0	0	0	0
Sphingobacterium	3	85	0	0	0	0	0	1	1	0	0	0	0	0	0	0	0
Sphingobium	1	179	0	0	0	0	1	1	0	0	1	1	0	0	1	0	0
Sphingomonadaceae	1	15	0	0	0	0	0	0	0	0	0	0	0	0	0	0	0
Sphingomonas	2	220	1	1	1	1	1	1	1	0	1	1	1	0	0	0	1
Sphingopyxis	3	99	0	0	0	0	0	1	0	0	0	0	0	0	0	0	0
Spirochaeta	2	103	0	0	0	0	0	1	0	0	0	0	0	0	0	0	0
Spiroplasma	1	6	0	0	0	0	0	0	0	0	0	0	0	0	0	0	0
Spirosoma	2	107	0	0	0	0	0	1	1	1	0	0	0	0	0	0	0
Stackebrandtia	2	110	0	0	0	0	0	1	1	0	0	0	0	0	0	0	0
Stanieria	1	17	0	0	0	0	0	0	0	0	0	0	0	0	0	0	0
Staphylococcus	2	108	0	0	0	0	0	1	1	0	0	0	0	0	0	0	0
Staphylothermus	1	12	0	0	0	0	0	0	0	0	0	0	0	0	0	0	0

Genus	Cluster Number	Protein Counts	1.14.12.10	1.14.12.13	1.14.12.18	1.14.12.3	1.14.12.7	1.14.13.-	1.14.13.1	1.14.13.2	1.14.13.20	1.14.13.22	1.14.13.40	1.14.13.50	1.14.13.7	1.14.13.8	1.14.13.82
Stappia	1	2	0	0	0	0	0	0	0	0	0	0	0	0	0	0	0
Starkeya	3	134	1	0	0	0	0	1	1	0	0	0	0	0	0	0	0
Stenotrophomonas	3	104	0	0	0	0	0	1	0	0	0	0	0	0	0	0	0
Stigmatella	3	129	0	0	0	0	0	1	0	1	0	0	0	0	0	0	1
Streptobacillus	1	38	0	0	0	0	0	0	0	0	0	0	0	0	0	0	0
Streptococcus	2	121	0	0	0	0	0	1	0	0	0	0	0	0	0	0	0
Streptomyces	2	253	1	0	1	0	0	1	1	1	0	1	1	0	0	0	1
Streptosporangium	3	148	0	0	0	0	0	1	1	1	0	1	0	0	0	0	0
Strigomonas	1	2	0	0	0	0	0	0	0	0	0	0	0	0	0	0	0
Sulfitobacter	1	10	0	0	0	0	0	0	0	0	0	0	0	0	0	0	0
Sulfobacillus	1	30	0	0	0	0	0	0	0	0	0	0	0	0	0	0	0
Sulfolobus	1	23	0	0	0	0	0	0	0	0	0	0	0	0	0	0	0
Sulfuricurvum	2	67	0	0	0	0	0	1	0	0	0	0	0	0	0	0	0
Sulfurihydrogenibium	2	55	0	0	0	0	0	0	0	0	0	0	0	0	0	0	0
Sulfurimonas	2	60	0	0	0	0	0	0	0	0	0	0	0	0	0	0	0
Sulfurospirillum	1	48	0	0	0	0	0	0	0	0	0	0	0	0	0	0	0
Sulfurovum	2	48	0	0	0	0	0	1	0	0	0	0	0	0	0	0	0
Symbiobacterium	2	68	0	0	0	0	0	0	0	0	0	0	0	0	0	0	0
Synechococcus	1	34	0	0	0	0	0	0	0	0	0	0	0	0	0	0	0
Synechocystis	1	40	0	0	0	0	0	0	0	0	0	0	0	0	0	0	0
Synergistetes	1	27	0	0	0	0	0	0	0	0	0	0	0	0	0	0	0
Syntrophobacter	2	101	0	0	0	0	0	0	0	0	0	0	0	0	0	0	0
Syntrophobotulus	2	71	0	0	0	0	0	0	0	0	0	0	0	0	0	0	0
Syntrophomonas	2	58	0	0	0	0	0	0	0	0	0	0	0	0	0	0	0
Syntrophothermus	2	60	0	0	0	0	0	0	0	0	0	0	0	0	0	0	0
Syntrophus	2	66	0	0	0	0	0	0	0	0	0	0	0	0	0	0	0
Tannerella	1	8	0	0	0	0	0	0	0	0	0	0	0	0	0	0	0
Taylorella	1	43	0	0	0	0	0	1	0	0	0	0	0	0	0	0	0
Tepidanaerobacter	2	55	0	0	0	0	0	0	0	0	0	0	0	0	0	0	0
Teredinibacter	2	109	0	0	0	0	0	1	0	0	0	0	0	0	0	0	1
Terrabacter	1	1	0	0	1	0	0	0	0	0	0	0	0	0	0	0	0
Terriglobus	2	83	0	0	0	0	0	0	0	1	0	0	0	0	0	0	0
Tetragenococcus	1	12	0	0	0	0	0	0	0	0	0	0	0	0	0	0	0
Thalassiobium	1	11	0	0	0	0	0	0	0	0	0	0	0	0	0	0	1
Thalassobacter	1	9	0	0	0	0	0	0	0	0	0	0	0	0	0	0	1
Thalassolituus	1	12	0	0	0	0	0	0	0	0	0	0	0	0	0	0	0
Thalassospira	1	2	0	0	0	0	0	0	0	0	0	0	0	0	0	0	0
Thauera	1	111	1	0	0	0	0	1	0	0	0	0	0	0	1	0	0
Thermacetogenium	1	7	0	0	0	0	0	0	0	0	0	0	0	0	0	0	0
Thermaerobacter	2	79	0	0	0	0	0	1	0	0	0	0	0	0	0	0	0
Thermanaerovibrio	2	58	0	0	0	0	0	0	0	0	0	0	0	0	0	0	0
Thermincola	2	62	0	0	0	0	0	0	0	0	0	0	0	0	0	0	0
Thermoanaerobacter	2	76	0	0	0	0	0	0	0	0	0	0	0	0	0	0	0
Thermoanaerobacterium	2	64	0	0	0	0	0	0	0	0	0	0	0	0	0	0	0
Thermobacillus	1	11	0	0	0	0	0	0	0	0	0	0	0	0	0	0	0
Thermobaculum	2	62	0	0	0	0	0	0	0	0	0	0	0	0	0	0	0
Thermobifida	2	72	0	0	0	0	0	1	0	0	0	1	0	0	0	0	0
Thermobispora	2	109	0	0	0	0	0	1	1	1	0	1	0	0	0	0	0
Thermococcus	1	13	0	0	0	0	0	0	0	0	0	0	0	0	0	0	0
Thermocrinis	2	60	0	0	0	0	0	0	0	0	0	0	0	0	0	0	0
Thermodesulfatator	1	10	0	0	0	0	0	0	0	0	0	0	0	0	0	0	0
Thermodesulfobacterium	1	9	0	0	0	0	0	0	0	0	0	0	0	0	0	0	0

Genus	Cluster Number	Protein Counts	1.14.12.10	1.14.12.13	1.14.12.18	1.14.12.3	1.14.12.7	1.14.13.-	1.14.13.1	1.14.13.2	1.14.13.20	1.14.13.22	1.14.13.40	1.14.13.50	1.14.13.7	1.14.13.8	1.14.13.82
Thermodesulfobium	1	13	0	0	0	0	0	0	0	0	0	0	0	0	0	0	0
Thermodesulfovibrio	2	60	0	0	0	0	0	0	0	0	0	0	0	0	0	0	0
Thermofilum	1	9	0	0	0	0	0	0	0	0	0	0	0	0	0	0	0
Thermogladius	1	7	0	0	0	0	0	0	0	0	0	0	0	0	0	0	0
Thermomicrobium	2	92	0	0	1	0	0	1	0	0	0	0	0	0	0	0	0
Thermomonospora	2	125	0	0	0	0	0	1	1	1	0	1	0	0	1	0	0
Thermoplasma	1	11	0	0	0	0	0	0	0	0	0	0	0	0	0	0	0
Thermoplasmatales	1	6	0	0	0	0	0	0	0	0	0	0	0	0	0	0	0
Thermoproteus	1	12	0	0	0	0	0	0	0	0	0	0	0	0	0	0	0
Thermosediminibacter	2	64	0	0	0	0	0	0	0	0	0	0	0	0	0	0	0
Thermosipho	2	70	0	0	0	0	0	1	0	0	0	0	0	0	0	0	0
Thermosphaera	1	10	0	0	0	0	0	0	0	0	0	0	0	0	0	0	0
Thermosynechococcus	1	6	0	0	0	0	0	0	0	0	0	0	0	0	0	0	0
Thermotoga	2	80	0	0	0	0	0	1	0	0	0	0	0	0	0	0	0
Thermovibrio	2	58	0	0	0	0	0	1	0	0	0	0	0	0	0	0	0
Thermovirga	1	9	0	0	0	0	0	0	0	0	0	0	0	0	0	0	0
Thermus	2	107	0	0	0	0	0	1	0	1	0	0	0	0	0	0	0
Thioalkalimicrobium	1	4	0	0	0	0	0	0	0	0	0	0	0	0	0	0	0
Thioalkalivibrio	2	91	0	0	0	0	0	1	0	0	0	0	0	0	0	0	0
Thiobaca	1	2	0	0	0	0	0	0	0	0	0	0	0	0	0	0	0
Thiobacillus	2	67	0	0	0	0	0	1	0	0	0	0	0	0	0	0	0
Thiocapsa	1	2	0	0	0	0	0	0	0	0	0	0	0	0	0	0	0
Thiocystis	1	11	0	0	0	0	0	0	0	0	0	0	0	0	0	0	0
Thioflavicoccus	1	13	0	0	0	0	0	0	0	0	0	0	0	0	0	0	0
Thiomicrospira	2	50	0	0	0	0	0	1	0	0	0	0	0	0	0	0	0
Thiomonas	2	85	0	0	0	0	0	1	0	0	0	0	0	0	0	0	0
Thiorhodococcus	1	2	0	0	0	0	0	0	0	0	0	0	0	0	0	0	0
Thiothrix	1	2	0	0	0	0	0	0	0	0	0	0	0	0	0	0	0
Tistrella	1	42	0	0	0	0	0	0	0	0	0	0	0	0	0	0	0
Tolumonas	2	100	0	0	0	0	0	1	0	0	0	0	0	0	0	0	0
Transposon	1	2	0	0	0	0	0	0	0	0	0	0	0	0	0	0	0
Treponema	1	63	0	0	0	0	0	0	0	0	0	0	0	0	0	0	0
Trichodesmium	2	71	0	0	0	0	0	1	1	0	0	1	0	0	0	1	0
Trichormus	1	12	0	0	0	0	0	0	0	0	0	0	0	0	0	0	0
Tropheryma	2	22	0	0	0	0	0	0	0	0	0	0	0	0	0	0	0
Truepera	2	104	0	0	0	0	0	1	0	0	0	0	0	0	0	0	0
Tsukamurella	2	123	0	0	0	0	0	1	1	0	0	1	0	0	0	0	0
Turneriella	1	16	0	0	0	0	0	0	0	0	0	0	0	0	0	0	0
Uliginosibacterium	1	2	0	0	0	0	0	0	0	0	0	0	0	0	0	0	0
Ureaplasma	1	24	0	0	0	0	0	0	0	0	0	0	0	0	0	0	0
Ureibacillus	1	2	0	0	0	0	0	0	0	0	0	0	0	0	0	0	0
Variovorax	3	187	0	1	0	0	1	1	1	1	0	0	1	0	0	0	1
Veillonella	1	54	0	0	0	0	0	0	0	0	0	0	0	0	0	0	0
Verminephrobacter	3	188	0	1	0	1	1	1	0	1	0	0	0	0	1	0	1
Verrucosispora	3	126	0	0	0	0	0	1	1	1	0	0	0	0	0	0	0
Vibrio	2	195	0	0	0	0	0	1	0	0	0	0	0	0	0	0	0
Vibrionales	1	46	0	0	0	0	0	0	0	0	0	0	0	0	0	0	0
Vitreoscilla	1	1	0	0	0	0	0	0	0	0	0	0	0	0	0	0	0
Vulcanisaeta	1	14	0	0	0	0	0	0	0	0	0	0	0	0	0	0	0
Waddlia	2	68	0	0	0	0	0	0	0	0	0	0	0	0	1	0	0
Wautersia	1	2	0	0	0	0	0	0	0	0	0	0	0	0	1	0	0
Weeksella	2	65	0	0	0	0	0	1	0	0	0	0	0	0	0	0	0

Genus	Cluster Number	Protein Counts	1.14.12.10	1.14.12.13	1.14.12.18	1.14.12.3	1.14.12.7	1.14.13.-	1.14.13.1	1.14.13.2	1.14.13.20	1.14.13.22	1.14.13.40	1.14.13.50	1.14.13.7	1.14.13.8	1.14.13.82
**Weissella**	1	14	0	0	0	0	0	0	0	0	0	0	0	0	0	0	0
**Wenxinia**	1	2	0	0	0	0	0	0	0	0	0	0	0	0	0	0	0
**Wigglesworthia**	1	29	0	0	0	0	0	1	0	0	0	0	0	0	0	0	0
**Wolbachia**	1	32	0	0	0	0	0	1	0	0	0	0	0	0	0	0	0
**Wolinella**	2	51	0	0	0	0	0	0	0	0	0	0	0	0	0	0	0
**Xanthobacter**	3	176	1	0	0	0	0	1	1	1	0	0	0	0	0	0	0
**Xanthobacteraceae**	1	4	0	0	0	0	0	0	0	0	0	0	0	0	0	0	0
**Xanthomonadaceae**	1	39	0	0	0	0	0	1	0	0	0	0	0	0	0	0	0
**Xanthomonas**	2	162	0	0	0	0	0	1	0	1	0	0	0	0	0	0	1
**Xenorhabdus**	2	127	0	0	0	0	0	1	0	0	0	1	1	0	0	0	0
**Xylanimonas**	2	66	0	0	0	0	0	1	0	0	0	0	1	0	0	0	0
**Xylella**	2	52	0	0	0	0	0	1	0	0	0	0	0	0	0	0	0
**Yersinia**	2	145	1	0	0	0	0	1	0	0	0	0	0	0	0	0	1
**Yokenella**	2	61	0	0	0	0	0	0	0	0	0	0	0	0	0	0	1
**Zobellia**	1	28	0	0	0	0	0	0	0	0	0	0	0	0	0	0	0
**Zunongwangia**	2	98	0	0	0	0	0	1	0	0	0	0	0	0	0	0	0
**Zymomonas**	2	67	0	0	0	0	0	1	0	0	0	0	0	0	0	0	0
**Zymophilus**	1	2	0	0	0	0	0	0	0	0	0	0	0	0	0	0	0

Genus	Cluster Number	Protein Counts	1.14.14.1	1.14.99.-	1.17.3.2	1.17.99.1	1.17.99.2	1.18.6.1	1.2.1.-	1.2.1.10	1.2.1.28	1.2.1.3	1.2.1.39	1.2.1.5	1.2.1.65	1.2.7.1	1.2.99.2	1.3.1.-	1.3.1.2	1.3.1.25
**Acaricomes**	1	2	0	0	0	0	0	0	0	0	0	0	0	0	0	0	0	0	0	0
**Acaryochloris**	2	88	0	1	0	0	0	0	0	1	0	1	0	0	0	0	0	0	0	0
**Acetivibrio**	1	2	0	0	0	0	0	0	0	0	0	0	0	0	0	1	0	0	0	0
**Acetobacter**	2	85	0	1	0	0	0	0	0	0	0	1	1	0	0	0	0	0	1	0
**Acetobacteraceae**	1	2	0	0	0	0	0	0	0	0	0	0	0	0	0	0	0	0	0	0
**Acetobacterium**	1	8	0	0	0	0	0	0	0	0	0	0	0	0	0	0	0	0	0	0
**Acetohalobium**	2	79	0	0	0	0	0	0	0	0	0	0	0	0	0	1	1	0	0	0
**Acholeplasma**	2	38	0	1	0	0	0	0	0	0	0	1	0	0	0	0	0	0	0	0
**Achromobacter**	2	192	0	0	0	1	0	0	0	1	0	1	1	0	0	0	1	0	0	0
**Acidaminococcus**	1	65	0	0	0	0	0	0	0	0	0	0	0	0	0	0	0	0	0	0
**Acidianus**	1	9	0	0	0	0	0	0	0	0	0	0	0	0	0	0	0	0	0	0
**Acidilobus**	1	8	0	0	0	0	0	0	0	0	0	0	0	0	0	0	0	0	0	0
**Acidimicrobium**	2	58	0	1	0	0	0	0	0	0	0	1	0	0	0	0	1	0	0	0
**Acidiphilium**	3	144	0	1	0	0	0	0	0	0	0	1	0	0	0	0	1	0	0	0
**Acidithiobacillus**	2	70	0	0	0	0	0	1	0	0	0	1	0	0	0	0	0	0	0	0
**Acidobacterium**	2	91	0	0	0	0	0	0	0	0	0	1	0	0	0	0	0	0	0	0
**Acidocella**	1	2	0	0	0	0	0	0	0	0	0	0	0	0	0	0	0	0	0	0
**Acidothermus**	2	68	0	0	0	0	0	0	0	0	0	1	0	0	0	1	0	0	0	0
**Acidovorax**	3	217	0	1	0	0	0	0	0	1	1	1	0	0	0	1	0	1	1	0
**Aciduliprofundum**	1	11	0	0	0	0	0	0	0	0	0	0	0	0	0	0	0	0	0	0
**Acinetobacter**	3	188	0	0	0	0	0	0	0	0	1	1	1	0	0	0	0	0	0	1
**Actinobacillus**	2	112	0	0	0	0	0	0	0	0	0	1	0	0	0	0	0	0	0	0
**Actinomadura**	1	1	0	0	0	0	0	0	0	0	0	0	0	0	0	0	0	0	0	0
**Actinoplanes**	1	52	0	0	0	0	0	0	0	0	0	0	0	0	0	0	0	0	0	0
**Actinopolyspora**	1	2	0	0	0	0	0	0	0	0	0	0	0	0	0	0	0	0	0	0
**Actinosynnema**	2	122	0	1	0	0	0	0	0	0	1	1	0	0	0	0	0	0	0	0
**Advenella**	1	30	0	0	0	0	0	0	0	0	0	0	0	0	0	0	0	0	0	0
**Aequorivita**	1	12	0	0	0	0	0	0	0	0	0	0	0	0	0	0	0	0	0	0
**Aerococcus**	2	57	0	0	0	0	0	0	0	0	0	1	0	0	0	0	0	0	0	0
**Aeromonas**	2	127	0	0	0	0	0	0	0	1	0	1	0	0	0	0	0	0	0	0
**Aeropyrum**	1	13	0	0	0	0	0	0	0	0	0	0	0	0	0	0	0	0	0	0
**Afipia**	1	54	0	0	0	0	0	0	0	0	0	1	0	0	0	0	1	0	0	0
**Aggregatibacter**	1	81	0	0	0	0	0	0	0	0	0	0	0	0	0	0	0	0	0	0
**Agrobacterium**	2	242	0	0	1	0	0	0	0	1	0	1	0	0	1	0	1	0	1	1
**Ahrensia**	1	2	0	0	0	0	0	0	0	0	0	0	0	0	0	0	0	0	0	0
**Akkermansia**	2	47	0	0	0	0	0	0	0	0	0	0	0	0	0	0	0	0	0	0
**Albidiferax**	3	144	0	0	0	0	0	0	0	1	0	1	0	0	0	1	0	0	0	0
**Alcaligenes**	1	21	0	0	0	0	0	1	0	0	0	0	0	0	0	0	0	0	0	0
**Alcanivorax**	2	146	0	1	0	0	0	0	0	0	0	1	0	0	0	0	0	0	0	0
**Alicycliphilus**	3	152	0	0	0	0	1	0	0	1	0	1	0	0	0	0	1	0	0	1
**Alicyclobacillus**	2	91	0	1	0	0	0	0	0	1	0	1	0	0	0	0	0	0	0	0
**Aliivibrio**	2	118	0	0	0	0	0	0	0	1	0	1	0	0	0	0	0	0	0	0
**Alishewanella**	1	1	0	0	0	0	0	0	0	0	0	0	0	0	0	0	0	0	0	0
**Alistipes**	1	52	0	0	0	0	0	0	0	0	0	1	0	0	0	0	0	0	0	0
**Alkalilimnicola**	2	97	0	0	0	0	0	0	0	1	0	1	0	0	0	0	1	0	0	0
**Alkaliphilus**	2	62	0	0	0	0	0	0	0	0	0	1	0	0	0	0	0	0	0	0
**Allochromatium**	2	84	0	1	0	0	0	1	0	0	0	0	0	0	0	0	0	0	0	0
**Alphaproteobacteria**	1	3	0	0	0	0	0	0	0	0	0	0	0	0	0	0	0	0	0	0
**Alteromonadales**	1	12	0	0	0	0	0	0	0	0	0	0	0	0	0	0	0	0	0	0
**Alteromonas**	1	37	0	0	0	0	0	0	0	0	0	0	0	0	0	0	0	0	0	0
**Aminobacter**	1	4	0	0	0	0	0	0	0	0	0	0	0	0	0	0	0	0	0	0
**Aminobacterium**	2	59	0	0	0	0	0	0	0	0	0	0	0	0	0	1	0	0	0	0

Genus	Cluster Number	Protein Counts	1.14.14.1	1.14.99.-	1.17.3.2	1.17.99.1	1.17.99.2	1.18.6.1	1.2.1.-	1.2.1.10	1.2.1.28	1.2.1.3	1.2.1.39	1.2.1.5	1.2.1.65	1.2.7.1	1.2.99.2	1.3.1.-	1.3.1.2	1.3.1.25
**Ammonifex**	2	50	0	0	0	0	0	0	0	0	0	0	0	0	0	0	1	0	0	0
**Amphibacillus**	1	13	0	0	0	0	0	0	0	0	0	0	0	0	0	0	0	0	0	0
**Amphritea**	1	2	0	0	0	0	0	0	0	0	0	0	0	0	0	0	0	0	0	0
**Amycolatopsis**	2	201	0	1	0	0	0	0	0	1	0	1	0	0	0	0	1	0	0	0
**Amycolicicoccus**	1	43	0	0	0	0	0	0	0	0	0	0	0	0	0	0	0	0	0	0
**Anabaena**	2	92	0	1	0	0	0	1	0	0	0	1	0	0	0	0	0	0	0	0
**Anaerobaculum**	1	11	0	0	0	0	0	0	0	0	0	0	0	0	0	0	0	0	0	0
**Anaerococcus**	2	46	0	0	0	0	0	0	0	0	0	1	0	0	0	0	0	0	0	0
**Anaerolinea**	2	66	0	1	0	0	0	0	0	0	0	0	0	0	0	0	1	0	0	0
**Anaeromyxobacter**	3	117	0	0	0	0	0	1	0	0	0	1	0	0	0	0	0	0	0	0
**Anaerostipes**	1	44	0	0	0	0	0	0	0	0	0	1	0	0	0	1	0	0	1	0
**Anaerotruncus**	1	2	0	0	0	0	0	0	0	0	0	0	0	0	0	0	0	0	0	0
**Anaplasma**	1	35	0	0	0	0	0	0	0	0	0	0	0	0	0	0	0	0	0	0
**Ancylobacter**	1	3	0	0	0	0	0	0	0	0	0	0	0	0	0	0	0	0	0	0
**Aneurinibacillus**	1	1	0	0	0	0	0	0	0	0	0	1	0	0	0	0	0	0	0	0
**Angomonas**	1	1	0	0	0	0	0	0	0	0	0	0	0	0	0	0	0	0	0	0
**Anoxybacillus**	2	72	0	1	0	0	0	0	0	0	0	1	0	0	0	0	0	0	0	0
**Aquifex**	2	45	0	0	0	0	0	0	0	0	0	1	0	0	0	1	0	0	0	0
**Aquimarina**	1	2	0	0	0	0	0	0	0	0	0	0	0	0	0	0	0	0	0	0
**Arcanobacterium**	2	45	0	0	0	0	0	0	0	0	0	0	0	0	0	0	0	0	0	0
**Archaeoglobus**	1	14	0	0	0	0	0	0	0	0	0	0	0	0	0	0	0	0	0	0
**Arcobacter**	2	101	0	0	0	0	0	1	0	0	0	1	0	0	0	1	0	0	0	0
**Aromatoleum**	3	133	0	0	0	1	1	0	0	0	1	1	0	0	0	1	0	0	0	0
**Arsenophonus**	1	4	0	0	0	0	0	0	0	0	0	0	0	0	0	0	0	0	0	0
**Arthrobacter**	2	206	0	1	0	0	0	0	0	0	0	1	0	0	0	0	1	1	0	1
**Arthrospira**	2	67	0	1	0	0	0	0	0	1	0	1	0	0	0	0	0	0	0	0
**Aster**	1	10	0	0	0	0	0	0	0	0	0	0	0	0	0	0	0	0	0	0
**Asticcacaulis**	2	103	0	0	0	0	0	0	1	0	0	1	0	0	0	0	0	0	0	0
**Atopobium**	1	28	0	0	0	0	0	0	0	0	0	0	0	0	0	0	0	0	0	0
**Aurantimonas**	1	2	0	0	0	0	0	0	0	0	0	0	0	0	0	0	0	0	0	0
**Avibacterium**	1	17	0	0	0	0	0	0	0	0	0	0	0	0	0	0	0	0	0	0
**Azoarcus**	3	172	0	0	0	0	1	1	0	1	1	1	0	0	0	0	0	0	0	0
**Azorhizobium**	3	149	0	0	0	0	0	1	0	0	0	1	0	0	0	0	1	0	0	0
**Azorhizophilus**	1	4	0	0	0	0	0	1	0	0	0	0	0	0	0	0	0	0	0	0
**Azospira**	1	19	0	0	0	0	0	0	0	0	0	0	0	0	0	0	0	0	0	0
**Azospirillum**	2	198	0	1	0	0	0	1	0	0	1	1	0	0	0	0	1	0	1	1
**Azotobacter**	3	183	0	0	0	0	0	1	0	1	0	1	0	0	0	0	1	1	0	1
**Bacillales**	1	9	0	0	0	0	0	0	0	0	0	0	0	0	0	0	0	0	0	0
**Bacilli**	1	3	0	0	0	0	0	0	0	0	0	0	0	0	0	0	0	0	0	0
**Bacillus**	2	237	0	1	0	0	0	0	0	1	0	1	1	0	0	1	0	0	1	0
**Bacteria**	2	86	0	0	0	0	0	0	0	0	0	1	0	0	0	0	0	0	1	0
**Bacteriovorax**	2	98	0	1	0	0	0	0	0	0	0	1	0	0	0	0	0	0	0	0
**Bacteroidales**	2	59	0	0	0	0	0	0	0	0	0	0	0	0	0	0	0	0	0	0
**Bacteroides**	2	96	0	0	0	0	0	0	0	0	0	0	0	0	0	0	0	1	1	0
**Bacteroidetes**	1	2	0	0	0	0	0	0	0	0	0	0	0	0	0	0	0	0	0	0
**Bartonella**	2	50	0	0	0	0	0	0	0	0	0	1	0	0	0	0	0	0	0	0
**Baumannia**	2	24	0	0	0	0	0	0	0	0	0	0	0	0	0	0	0	0	0	0
**Bdellovibrio**	2	87	0	0	0	0	0	0	0	0	0	1	0	0	0	0	0	0	0	0
**Beijerinckia**	3	123	0	1	0	0	0	1	0	0	0	1	0	0	0	0	0	0	0	0
**Belliella**	1	14	0	0	0	0	0	0	0	0	0	0	0	0	0	0	0	0	0	0
**Beutenbergia**	2	82	0	1	0	0	0	0	0	0	0	1	0	0	0	0	0	0	0	0
**Bifidobacterium**	2	61	0	0	0	0	0	0	0	1	0	1	0	0	0	0	0	0	0	0

Genus	Cluster Number	Protein Counts	1.14.14.1	1.14.99.-	1.17.3.2	1.17.99.1	1.17.99.2	1.18.6.1	1.2.1.-	1.2.1.10	1.2.1.28	1.2.1.3	1.2.1.39	1.2.1.5	1.2.1.65	1.2.7.1	1.2.99.2	1.3.1.-	1.3.1.2	1.3.1.25
**Bizionia**	1	1	0	0	0	0	0	0	0	0	0	0	0	0	0	0	0	0	0	0
**Blastococcus**	1	39	0	0	0	0	0	0	0	0	0	0	0	0	0	0	0	0	0	0
**Blattabacterium**	1	23	0	0	0	0	0	0	0	0	0	0	0	0	0	0	0	0	0	0
**Blautia**	1	22	0	0	0	0	0	0	0	0	0	0	0	0	0	0	0	0	0	0
**Bordetella**	3	227	0	0	0	0	0	0	0	1	1	1	0	0	0	0	1	1	0	1
**Borrelia**	1	32	0	0	0	0	0	0	0	0	0	0	0	0	0	0	0	0	0	0
**Brachybacterium**	1	18	0	0	0	0	0	0	0	0	0	0	0	0	0	0	0	0	0	0
**Brachymonas**	1	4	0	0	0	0	0	0	0	0	0	0	0	0	0	0	0	0	0	0
**Brachyspira**	2	79	0	0	0	0	0	0	0	1	0	1	0	0	0	1	0	0	0	0
**Bradyrhizobiaceae**	1	20	0	0	0	0	0	0	0	0	0	0	0	0	0	0	1	0	0	0
**Bradyrhizobium**	3	254	0	1	0	0	0	1	0	0	1	1	0	0	0	0	1	0	1	0
**Brenneria**	1	20	0	0	0	0	0	0	0	0	0	0	0	0	0	0	0	0	0	0
**Brevibacillus**	2	122	0	1	0	0	0	0	0	1	0	1	0	0	0	0	0	0	1	0
**Brevibacterium**	1	4	0	0	0	0	0	0	0	0	0	0	0	0	0	0	0	0	0	0
**Brevundimonas**	2	105	0	1	0	0	0	0	0	0	0	1	0	0	0	0	0	0	0	0
**Brucella**	3	138	0	0	0	0	0	0	0	0	0	1	0	0	0	0	0	0	1	0
**Buchnera**	1	26	0	0	0	0	0	0	0	0	0	0	0	0	0	0	0	0	0	0
**Burkholderia**	3	424	0	1	0	1	0	1	1	1	1	1	1	0	0	0	1	1	1	1
**Burkholderiaceae**	3	143	0	0	0	0	0	0	0	1	0	1	0	0	0	0	1	0	0	0
**Burkholderiales**	1	24	0	0	0	0	0	1	0	1	0	0	0	0	0	0	0	0	0	0
**Buttiauxella**	1	2	0	0	0	0	0	0	0	0	0	0	0	0	0	0	0	0	0	0
**Butyrivibrio**	2	67	0	0	0	0	0	1	0	0	0	1	0	0	0	0	0	0	0	0
**Caldanaerobacter**	2	63	0	0	0	0	0	0	0	0	0	0	0	0	0	0	1	0	0	0
**Caldicellulosiruptor**	2	75	0	0	0	0	0	1	0	0	0	0	0	0	0	1	1	0	0	0
**Caldilinea**	1	23	0	0	0	0	0	0	0	0	0	0	0	0	0	0	0	0	0	0
**Caldimonas**	1	6	0	0	0	0	0	0	0	0	0	0	0	0	0	0	0	0	0	0
**Caldisericum**	1	10	0	0	0	0	0	0	0	0	0	0	0	0	0	0	0	0	0	0
**Caldisphaera**	1	7	0	0	0	0	0	0	0	0	0	0	0	0	0	0	0	0	0	0
**Calditerrivibrio**	2	74	0	0	0	0	0	1	0	0	0	1	0	0	0	0	0	0	0	0
**Caldivirga**	1	13	0	0	0	0	0	0	0	0	0	0	0	0	0	0	0	0	0	0
**Calothrix**	1	43	0	0	0	0	0	1	0	0	0	0	0	0	0	0	0	0	0	0
**Calyptogena**	2	34	0	0	0	0	0	0	0	0	0	0	0	0	0	0	0	0	0	0
**Campylobacter**	2	80	0	0	0	0	0	0	0	0	0	0	0	0	0	0	1	0	0	0
**Candidatus**	2	290	0	1	0	1	0	1	0	1	0	1	0	0	0	1	1	0	1	0
**Capnocytophaga**	1	50	0	0	0	0	0	0	0	0	0	0	0	0	0	0	0	0	0	0
**Carbophilus**	1	2	0	0	0	0	0	0	0	0	0	0	0	0	0	0	1	0	0	0
**Carboxydibrachium**	1	14	0	0	0	0	0	0	0	0	0	0	0	0	0	0	0	0	0	0
**Carboxydothermus**	2	68	0	0	0	0	0	0	0	1	0	0	0	0	0	0	1	0	0	0
**Carica**	1	2	0	0	0	0	0	0	0	0	0	0	0	0	0	0	0	0	0	0
**Carnobacterium**	2	71	0	1	0	0	0	0	0	0	0	1	0	0	0	0	0	0	0	0
**Catenibacterium**	1	2	0	0	0	0	0	0	0	0	0	0	0	0	0	0	0	0	0	0
**Catenovulum**	1	2	0	0	0	0	0	0	0	0	0	0	0	0	0	0	0	0	0	0
**Catenulispora**	3	159	0	0	0	0	0	0	0	0	1	1	0	0	0	0	1	0	0	0
**Caulobacter**	3	168	0	1	0	0	0	0	1	0	1	1	0	0	0	0	0	0	0	0
**Cedecea**	1	45	0	0	0	0	0	0	0	0	0	1	0	0	0	0	0	0	0	0
**Cellulomonas**	2	96	0	1	0	0	0	0	0	1	0	1	0	0	0	0	0	0	0	0
**Cellulophaga**	2	105	0	1	0	1	0	0	0	0	0	1	0	0	0	0	0	0	0	0
**Cellulosilyticum**	1	14	0	0	0	0	0	0	0	0	0	0	0	0	0	0	0	0	0	0
**Cellvibrio**	2	85	0	0	0	1	0	0	0	1	0	0	0	0	0	0	0	0	0	0
**Cenarchaeum**	1	7	0	0	0	0	0	0	0	0	0	0	0	0	0	0	0	0	0	0
**Chamaesiphon**	1	13	0	0	0	0	0	0	0	0	0	0	0	0	0	0	0	0	0	0
**Chelativorans**	3	136	0	0	0	0	0	0	0	0	0	1	0	0	0	0	1	0	1	0

Genus	Cluster Number	Protein Counts	1.14.14.1	1.14.99.-	1.17.3.2	1.17.99.1	1.17.99.2	1.18.6.1	1.2.1.-	1.2.1.10	1.2.1.28	1.2.1.3	1.2.1.39	1.2.1.5	1.2.1.65	1.2.7.1	1.2.99.2	1.3.1.-	1.3.1.2	1.3.1.25
Chelatococcus	1	2	0	0	0	0	0	0	0	0	0	0	0	0	0	0	0	0	0	0
Chitinophaga	2	98	0	1	0	0	0	0	0	0	0	1	0	0	0	0	0	0	1	0
Chlamydia	1	36	0	0	0	0	0	0	0	0	0	0	0	0	0	0	0	0	0	0
Chlamydomonas	1	3	0	0	0	0	0	0	0	0	0	0	0	0	0	0	0	0	0	0
Chlamydophila	1	31	0	0	0	0	0	0	0	0	0	0	0	0	0	0	0	0	0	0
Chlorobaculum	2	61	0	1	0	0	0	1	0	0	0	1	0	0	0	0	0	0	0	0
Chlorobium	2	89	0	1	0	0	0	1	0	0	0	1	0	0	0	0	0	0	0	0
Chlorobium/Pelodictyon	1	2	0	0	0	0	0	0	0	0	0	0	0	0	0	0	0	0	0	0
Chloroflexus	2	105	0	1	0	0	0	0	0	1	0	1	0	0	0	0	1	0	0	0
Chlorogloeopsis	1	9	0	0	0	0	0	0	0	0	0	0	0	0	0	0	0	0	0	0
Chloroherpeton	2	59	0	0	0	0	0	1	0	0	0	0	0	0	0	0	0	0	0	0
Chromobacterium	2	117	0	0	0	0	0	0	1	0	0	1	0	0	0	0	0	0	0	0
Chromohalobacter	2	145	0	0	0	0	0	0	0	0	0	1	0	0	0	0	0	0	0	0
Chroococcidiopsis	1	24	0	0	0	0	0	0	0	0	0	0	0	0	0	0	0	0	0	0
Chryseobacterium	2	104	0	0	0	0	0	0	0	0	0	1	0	0	0	0	1	0	0	0
Citreicella	1	13	0	0	0	0	0	0	0	0	0	0	0	0	0	0	0	0	0	0
Citrobacter	2	190	0	0	0	0	0	0	1	1	0	1	0	0	0	0	0	1	1	0
Clavibacter	2	83	0	1	0	0	0	0	0	0	0	1	0	0	0	0	0	1	0	0
Clonorchis	1	1	0	0	0	0	0	0	0	0	0	0	0	0	0	0	0	0	0	0
Clostridiales	2	77	0	0	0	0	0	1	0	1	0	1	0	0	0	1	0	0	1	0
Clostridium	2	191	0	1	0	0	0	1	0	1	0	1	0	0	0	1	1	0	1	0
Cohnella	1	2	0	0	0	0	0	0	0	0	0	0	0	0	0	0	0	0	0	0
Coleofasciculus	1	4	0	0	0	0	0	0	0	0	0	0	0	0	0	0	0	0	0	0
Collimonas	1	35	0	0	0	0	0	0	0	0	0	0	0	0	0	0	0	0	0	0
Colwellia	2	130	0	1	0	1	0	0	0	0	0	1	0	0	0	0	0	0	0	0
Comamonas	2	196	0	0	0	1	0	0	0	1	0	1	0	0	0	0	1	1	1	0
Conexibacter	3	117	0	1	0	0	0	0	0	1	0	1	0	1	0	0	1	0	0	0
Coprobacillus	1	2	0	0	0	0	0	0	0	0	0	0	0	0	0	0	0	0	0	0
Coprococcus	2	76	0	0	0	0	0	0	0	0	0	1	0	0	0	1	0	0	0	0
Coprothermobacter	2	45	0	0	0	0	0	0	0	0	0	0	0	0	0	1	0	0	0	0
Coraliomargarita	2	68	0	0	0	0	0	1	0	0	0	1	0	0	0	0	0	0	0	0
Corallococcus	1	29	0	0	0	0	0	0	0	0	0	0	0	0	0	0	0	0	0	0
Coriobacterium	2	36	0	0	0	0	0	0	0	0	0	0	0	0	0	0	0	0	0	0
Corynebacterineae	1	5	0	0	0	0	0	0	0	0	0	0	0	0	0	0	0	0	0	0
Corynebacterium	2	175	0	1	0	0	0	0	0	0	0	1	0	0	0	0	0	0	0	1
Coxiella	2	54	0	0	0	0	0	0	0	0	0	0	0	0	0	0	0	0	0	0
Crinalium	1	21	0	0	0	0	0	0	0	0	0	0	0	0	0	0	0	0	0	0
Croceibacter	2	80	0	1	0	0	0	0	0	0	0	1	0	0	0	0	0	0	0	0
Crocosphaera	1	6	0	0	0	0	0	0	0	0	0	0	0	0	0	0	0	0	0	0
Cronobacter	2	120	0	1	0	0	0	0	1	1	0	1	0	0	0	0	0	0	0	0
Cryptobacterium	2	39	0	0	0	0	0	0	0	0	0	0	0	0	0	1	0	0	0	0
Cucumis	1	9	0	0	0	0	0	0	0	0	0	0	0	0	0	0	0	0	0	0
Cupriavidus	3	317	0	0	0	0	0	1	0	1	1	1	0	0	0	0	1	0	0	1
Curtobacterium	1	5	0	1	0	0	0	0	0	0	0	0	0	0	0	0	0	0	0	0
Curvibacter	1	27	0	0	0	0	0	1	0	0	0	1	0	0	0	0	0	0	0	0
Cyanobacterium	1	9	0	0	0	0	0	0	0	0	0	0	0	0	0	0	0	0	0	0
Cyanobium	1	10	0	0	0	0	0	0	0	0	0	0	0	0	0	0	0	0	0	0
Cyanothece	1	34	0	0	0	0	0	0	0	0	0	0	0	0	0	0	0	0	0	0
Cyclobacterium	1	19	0	0	0	0	0	0	0	0	0	0	0	0	0	0	0	0	0	0
Cycloclasticus	1	21	0	0	0	0	0	0	0	0	0	0	0	0	0	0	0	0	0	0
Cylindrospermopsis	1	4	0	0	0	0	0	0	0	0	0	0	0	0	0	0	0	0	0	0
Cylindrospermum	1	30	0	0	0	0	0	1	0	0	0	0	0	0	0	0	0	0	0	0

Genus	Cluster Number	Protein Counts	1.14.14.1	1.14.99.-	1.17.3.2	1.17.99.1	1.17.99.2	1.18.6.1	1.2.1.-	1.2.1.10	1.2.1.28	1.2.1.3	1.2.1.39	1.2.1.5	1.2.1.65	1.2.7.1	1.2.99.2	1.3.1.-	1.3.1.2	1.3.1.25
Cytophaga	2	69	0	0	0	0	0	0	0	0	0	0	0	0	0	0	0	0	0	0
Dactylococcopsis	1	8	0	0	0	0	0	0	0	0	0	0	0	0	0	0	0	0	0	0
Dechloromonas	1	40	0	0	0	0	0	0	0	0	0	0	0	0	0	0	0	0	0	0
Dechlorosoma	1	19	0	0	0	0	0	0	0	0	0	0	0	0	0	0	0	0	0	0
Deferribacter	2	73	0	0	0	0	0	0	0	0	0	1	0	0	0	0	0	0	0	0
Dehalobacter	1	9	0	0	0	0	0	0	0	0	0	0	0	0	0	0	0	0	0	0
Dehalococcoides	2	41	0	0	0	0	0	1	0	0	0	0	0	0	0	1	0	0	0	0
Dehalogenimonas	1	37	0	0	0	0	0	0	0	0	0	0	0	0	0	1	1	0	0	0
Deinococcus	2	140	0	1	0	0	0	0	1	0	0	1	0	0	0	0	1	0	0	0
Delftia	3	177	0	0	0	0	0	1	0	1	0	1	0	0	0	0	1	0	1	0
Denitrovibrio	2	67	0	0	0	0	0	1	0	0	0	1	0	0	0	0	0	0	0	0
Desulfarculus	2	92	0	0	0	0	0	1	0	0	0	1	0	0	0	1	1	0	0	0
Desulfatibacillum	3	84	0	0	0	0	0	1	0	0	0	1	0	0	0	0	1	0	0	0
Desulfitobacterium	2	102	0	0	0	0	0	1	0	1	0	1	0	0	0	0	1	0	0	0
Desulfobacca	2	65	0	0	0	0	0	1	0	0	0	0	0	0	0	1	1	0	0	0
Desulfobacterium	2	113	0	0	0	0	0	1	0	1	0	1	0	0	0	0	1	0	0	0
Desulfobacula	1	21	0	0	0	0	0	0	0	0	0	0	0	0	0	0	0	0	0	0
Desulfobulbus	2	88	0	0	0	0	0	1	0	0	0	0	0	0	0	0	0	0	0	1
Desulfocapsa	1	9	0	0	0	0	0	0	0	0	0	0	0	0	0	0	0	0	0	0
Desulfococcus	2	80	0	0	0	0	1	0	0	0	0	1	0	0	0	1	1	0	0	0
Desulfohalobium	2	52	0	0	0	0	0	0	0	0	0	0	0	0	0	0	1	0	0	0
Desulfomicrobium	2	65	0	0	0	0	0	1	0	1	0	0	0	0	0	0	1	0	0	0
Desulfomonile	1	25	0	0	0	0	0	0	0	0	0	0	0	0	0	0	0	0	0	0
Desulforhabdus	1	2	0	0	0	0	0	0	0	0	0	0	0	0	0	0	0	0	0	0
Desulfosporosinus	1	30	0	0	0	0	0	0	0	0	0	0	0	0	0	0	0	0	0	0
Desulfotalea	2	66	0	0	0	0	0	0	0	1	0	0	0	0	0	1	0	0	0	0
Desulfotomaculum	2	98	0	0	0	0	0	1	0	0	0	1	0	0	0	0	1	0	0	0
Desulfovibrio	2	144	0	0	0	0	0	1	0	1	0	1	0	0	0	0	1	0	0	0
Desulfurispirillum	2	78	0	0	0	0	0	1	0	0	0	0	0	0	0	1	1	0	0	0
Desulfurivibrio	2	71	0	0	0	0	0	1	0	0	0	0	0	0	0	0	1	0	0	0
Desulfurobacterium	2	53	0	0	0	0	0	0	0	0	0	1	0	0	0	1	1	0	0	0
Desulfurococcus	1	11	0	0	0	0	0	0	0	0	0	0	0	0	0	0	0	0	0	0
Diaphorobacter	1	16	0	0	0	0	0	0	0	1	0	0	0	0	0	0	0	0	0	0
Dichelobacter	2	45	0	0	0	0	0	0	0	0	0	0	0	0	0	0	0	0	0	0
Dickeya	2	155	0	0	0	0	0	1	0	1	0	1	0	0	0	0	0	0	1	0
Dictyoglomus	2	62	0	0	0	0	0	0	0	0	0	1	0	0	0	1	0	0	0	0
Dinoroseobacter	3	148	0	1	0	0	0	0	0	0	0	1	0	0	0	0	1	0	1	0
Dokdonia	1	3	0	0	0	0	0	0	0	0	0	0	0	0	0	0	0	0	0	0
Dorea	1	7	0	0	0	0	0	0	0	0	0	0	0	0	0	0	0	0	0	0
Dyadobacter	2	83	0	1	0	0	0	0	0	0	0	1	0	0	0	0	0	1	0	0
Echinicola	1	20	0	0	0	0	0	0	0	0	0	0	0	0	0	0	0	0	0	0
Edwardsiella	2	126	0	0	0	0	0	0	0	1	0	1	0	0	0	0	0	0	0	0
Eggerthella	1	51	0	0	0	0	0	0	0	0	0	1	0	0	0	1	0	0	0	0
Ehrlichia	2	37	0	0	0	0	0	0	0	0	0	0	0	0	0	0	0	0	0	0
Eikenella	1	4	0	0	0	0	0	0	0	0	0	0	0	0	0	0	0	0	0	0
Elusimicrobium	2	44	0	0	0	0	0	0	0	1	0	0	0	0	0	0	0	0	0	0
Emticicia	1	21	0	0	0	0	0	0	0	0	0	0	0	0	0	0	0	0	0	0
Enhydrobacter	1	1	0	0	0	0	0	0	0	0	0	0	0	0	0	0	0	0	0	0
Ensifer	1	2	0	0	0	0	0	0	0	0	0	0	0	0	0	0	0	0	0	0
Enterobacter	2	202	0	0	0	0	0	1	1	1	0	1	1	0	0	0	0	0	0	1
Enterobacteriaceae	2	211	0	0	0	0	0	0	1	1	0	1	1	0	0	0	0	1	1	1
Enterococcus	1	187	0	1	0	0	0	0	1	1	0	1	0	0	0	0	0	0	0	1

Genus	Cluster Number	Protein Counts	1.14.14.1	1.14.99.-	1.17.3.2	1.17.99.1	1.17.99.2	1.18.6.1	1.2.1.-	1.2.1.10	1.2.1.28	1.2.1.3	1.2.1.39	1.2.1.5	1.2.1.65	1.2.7.1	1.2.99.2	1.3.1.-	1.3.1.2	1.3.1.25
Enterovibrio	1	15	0	0	0	0	0	0	0	1	0	0	0	0	0	0	0	0	0	0
Erwinia	2	151	0	0	0	0	0	0	0	1	0	1	0	0	0	0	0	0	0	0
Erysipelothrix	1	9	0	0	0	0	0	0	0	0	0	0	0	0	0	0	0	0	0	0
Erysipelotrichaceae	1	4	0	0	0	0	0	0	0	0	0	0	0	0	0	0	0	0	0	0
Erythrobacter	2	80	1	1	0	0	0	0	0	0	0	1	0	0	0	0	0	0	0	0
Escherichia	2	242	0	0	0	0	0	0	1	1	0	1	1	0	0	0	0	1	1	1
Ethanoligenens	2	69	0	0	0	0	0	1	0	0	0	0	0	0	0	0	0	0	1	0
Eubacteriaceae	1	2	0	0	0	0	0	0	0	0	0	0	0	0	0	0	0	0	0	0
Eubacterium	2	103	0	1	0	0	0	0	0	1	0	1	0	0	0	1	1	0	1	0
Eudoraea	1	4	0	0	0	0	0	0	0	0	0	0	0	0	0	0	0	0	0	0
Euryarchaeota	1	1	0	0	0	0	0	0	0	0	0	0	0	0	0	0	0	0	0	0
Exiguobacterium	2	93	0	1	0	0	0	0	0	1	0	1	1	0	0	0	0	0	0	0
Faecalibacterium	1	12	0	0	0	0	0	0	0	0	0	0	0	0	0	0	0	0	0	0
Ferrimonas	2	115	0	0	0	0	0	0	0	0	0	1	0	0	0	0	0	0	0	0
Ferroglobus	1	14	0	0	0	0	0	0	0	0	0	0	0	0	0	0	0	0	0	0
Ferroplasma	1	11	0	0	0	0	0	0	0	0	0	0	0	0	0	0	0	0	0	0
Fervidicoccus	1	7	0	0	0	0	0	0	0	0	0	0	0	0	0	0	0	0	0	0
Fervidobacterium	2	54	0	0	0	0	0	0	0	0	0	0	0	0	0	1	0	0	0	0
Fibrella	1	17	0	0	0	0	0	0	0	0	0	0	0	0	0	0	0	0	0	0
Fibrobacter	2	52	0	0	0	0	0	1	0	0	0	1	0	0	0	0	0	0	0	0
Filifactor	1	9	0	0	0	0	0	0	0	0	0	0	0	0	0	0	0	0	0	0
Finegoldia	2	55	0	0	0	0	0	0	0	1	0	1	0	0	0	1	1	0	0	0
Firmicutes	1	15	0	0	0	0	0	0	0	0	0	0	0	0	0	0	0	0	0	0
Fischerella	1	15	0	0	0	0	0	1	0	0	0	0	0	0	0	0	0	0	0	0
Flavobacteria	1	7	0	0	0	0	0	0	0	0	0	0	0	0	0	0	0	0	0	0
Flavobacteriaceae	2	69	0	1	0	0	0	0	0	0	0	1	0	0	0	0	0	0	0	0
Flavobacteriales	1	3	0	0	0	0	0	0	0	0	0	0	0	0	0	0	0	0	0	0
Flavobacterium	2	103	0	1	0	0	0	0	0	0	0	1	0	0	0	0	0	0	0	0
Flexibacter	1	17	0	0	0	0	0	0	0	0	0	0	0	0	0	0	0	0	0	0
Flexistipes	1	11	0	0	0	0	0	0	0	0	0	0	0	0	0	0	0	0	0	0
Fluoribacter	1	6	0	0	0	0	0	0	0	0	0	0	0	0	0	0	0	0	0	0
Fluviicola	2	69	0	1	0	0	0	0	0	0	0	1	0	0	0	0	0	0	0	0
Formosa	1	2	0	0	0	0	0	0	0	0	0	0	0	0	0	0	0	0	0	0
Francisella	2	88	0	0	0	0	0	0	0	0	0	0	0	0	0	0	0	0	0	0
Frankia	3	202	0	1	0	0	0	1	0	1	0	1	0	0	0	1	1	1	0	0
Frateuria	1	25	0	0	0	0	0	0	0	0	0	0	0	0	0	0	0	0	0	0
Fructobacillus	1	2	0	0	0	0	0	0	0	0	0	0	0	0	0	0	0	0	0	0
Fulvimarina	1	2	0	0	0	0	0	0	0	0	0	0	0	0	0	0	0	0	0	0
Fusobacterium	1	48	0	0	0	0	0	0	0	0	0	0	0	0	0	0	0	0	0	0
Gallibacterium	2	87	0	0	0	0	0	0	0	0	0	0	0	0	0	0	0	0	0	0
Gallionella	2	67	0	0	0	0	0	0	0	0	0	0	0	0	0	1	0	0	0	0
Gammaproteobacteria	1	9	0	0	0	0	0	0	0	0	0	0	0	0	0	0	0	0	0	0
Gardnerella	1	31	0	0	0	0	0	0	0	0	0	0	0	0	0	0	0	0	0	0
Geitlerinema	1	9	0	0	0	0	0	0	0	0	0	0	0	0	0	0	0	0	0	0
Gemmatimonas	2	93	0	1	0	0	0	0	0	0	0	1	0	0	0	0	0	0	0	0
Geobacillus	2	168	0	1	0	0	0	0	0	1	0	1	1	0	0	1	1	0	1	0
Geobacter	2	150	0	0	0	1	0	1	0	1	0	1	0	0	0	0	1	0	0	0
Geodermatophilus	2	136	0	1	0	0	0	0	0	0	1	1	0	0	0	0	1	0	0	1
Geopsychrobacter	1	2	0	0	0	0	0	0	0	0	0	0	0	0	0	0	0	0	0	0
Gillisia	1	11	0	0	0	0	0	0	0	0	0	0	0	0	0	0	0	0	0	0
Gilvimarinus	1	4	0	0	0	0	0	0	0	0	0	0	0	0	0	0	0	0	0	0
Glaciecola	2	117	0	0	0	0	0	0	0	0	0	1	0	0	0	0	0	0	0	0

Genus	Cluster Number	Protein Counts	1.14.14.1	1.14.99.-	1.17.3.2	1.17.99.1	1.17.99.2	1.18.6.1	1.2.1.-	1.2.1.10	1.2.1.28	1.2.1.3	1.2.1.39	1.2.1.5	1.2.1.65	1.2.7.1	1.2.99.2	1.3.1.-	1.3.1.2	1.3.1.25
Gloeobacter	2	67	0	1	0	0	0	0	0	0	0	0	0	0	0	0	0	0	0	0
Gloeocapsa	1	20	0	0	0	0	0	0	0	0	0	0	0	0	0	0	0	0	0	0
Gloeothece	1	1	0	0	0	0	0	0	0	0	0	0	0	0	0	0	0	0	0	0
Gluconacetobacter	2	96	0	1	0	0	0	1	0	0	0	1	1	0	0	0	0	0	1	0
Gluconobacter	2	76	0	0	0	0	0	0	0	0	0	1	0	0	0	0	0	0	0	0
Gordonia	1	158	0	1	0	0	0	0	0	1	0	1	0	0	0	0	1	0	0	1
Gordonibacter	1	32	0	0	0	0	0	0	0	0	0	1	0	0	0	1	0	0	0	0
Gramella	2	104	0	1	0	0	0	0	0	0	0	1	0	0	0	0	0	0	0	0
Granulibacter	2	90	0	1	0	0	0	0	0	0	0	1	0	0	0	0	0	1	0	0
Granulicella	2	98	0	0	0	0	0	0	0	0	0	1	0	0	0	0	0	1	1	0
Grimontia	1	9	0	0	0	0	0	0	0	1	0	0	0	0	0	0	0	0	0	0
Haemophilus	2	117	0	0	0	0	0	0	0	0	0	0	0	0	0	0	0	0	0	0
Hafnia	1	29	0	0	0	0	0	0	0	0	0	0	0	0	0	0	0	0	0	0
Hahella	3	130	0	0	0	0	0	0	0	0	0	1	0	0	0	0	0	0	0	0
Halalkalicoccus	1	18	0	0	0	0	0	0	0	0	0	0	0	0	0	0	0	0	0	0
Halanaerobium	2	89	0	0	0	0	0	0	0	0	0	0	0	0	0	1	0	0	1	0
Haliangium	2	108	0	1	0	0	0	0	0	0	0	1	0	0	0	0	1	1	0	0
Haliscomenobacter	2	107	0	1	0	0	0	0	0	0	0	1	0	0	0	0	1	0	0	0
Haloarcula	1	22	0	0	0	0	0	0	0	0	0	0	0	0	0	0	0	0	0	0
Halobacillus	1	19	0	0	0	0	0	0	0	0	0	0	0	0	0	0	0	0	0	0
Halobacteriaceae	1	1	0	0	0	0	0	0	0	0	0	0	0	0	0	0	0	0	0	0
Halobacterium	1	10	0	0	0	0	0	0	0	0	0	0	0	0	0	0	0	0	0	0
Halobacteroides	1	14	0	0	0	0	0	0	0	0	0	0	0	0	0	0	0	0	0	0
Haloferax	1	20	0	0	0	0	0	0	0	0	0	0	0	0	0	0	0	0	0	0
Halogeometricum	1	12	0	0	0	0	0	0	0	0	0	0	0	0	0	0	0	0	0	0
Halomicrobium	1	10	0	0	0	0	0	0	0	0	0	0	0	0	0	0	0	0	0	0
Halomonas	1	149	0	0	0	0	0	0	0	0	0	1	0	0	0	0	0	0	0	0
Halopiger	1	16	0	0	0	0	0	0	0	0	0	0	0	0	0	0	0	0	0	0
Haloquadratum	1	19	0	0	0	0	0	0	0	0	0	0	0	0	0	0	0	0	0	0
Halorhabdus	1	10	0	0	0	0	0	0	0	0	0	0	0	0	0	0	0	0	0	0
Halorhodospira	2	71	0	1	0	0	0	1	0	0	0	0	0	0	0	0	0	0	0	0
Halorubrum	1	16	0	0	0	0	0	0	0	0	0	0	0	0	0	0	0	0	0	0
Haloterrigena	1	19	0	0	0	0	0	0	0	0	0	0	0	0	0	0	0	0	0	0
Halothece	1	9	0	0	0	0	0	0	0	0	0	0	0	0	0	0	0	0	0	0
Halothermothrix	2	50	0	0	0	0	0	0	0	1	0	0	0	0	0	1	0	0	0	0
Halothiobacillus	2	65	0	0	0	0	0	0	0	0	0	1	0	0	0	0	0	0	0	0
Halovivax	1	12	0	0	0	0	0	0	0	0	0	0	0	0	0	0	0	0	0	0
Helicobacter	2	79	0	0	0	0	0	0	0	0	0	0	0	0	0	1	0	0	0	0
Heliobacillus	1	4	0	0	0	0	0	0	0	0	0	0	0	0	0	0	0	0	0	0
Heliobacterium	2	51	0	0	0	0	0	1	0	0	0	0	0	0	0	0	0	0	0	0
Herbaspirillum	2	172	0	0	0	0	0	1	0	1	1	1	0	0	0	0	0	1	0	1
Herminiimonas	3	89	0	0	0	0	0	0	0	0	0	0	0	0	0	0	0	0	0	0
Herpetosiphon	3	90	0	1	0	0	0	0	0	0	0	1	0	0	0	0	0	0	0	0
Hippea	2	44	0	0	0	0	0	0	0	0	0	1	0	0	0	1	0	0	0	0
Hirschia	2	102	0	0	0	0	0	0	0	0	0	1	0	0	0	0	0	0	0	0
Histophilus	2	72	0	0	0	0	0	0	0	0	0	0	0	0	0	0	0	0	0	0
Hoeflea	1	16	0	0	0	0	0	0	0	0	0	0	0	0	0	0	1	0	0	0
Hydrogenivirga	1	2	0	0	0	0	0	0	0	0	0	0	0	0	0	0	0	0	0	0
Hydrogenobacter	2	55	0	0	0	0	0	0	0	0	0	1	0	0	0	1	1	0	0	0
Hydrogenobaculum	2	44	0	0	0	0	0	0	0	0	0	0	0	0	0	1	0	0	0	0
Hydrogenophaga	1	9	0	0	0	0	0	0	0	0	0	0	0	0	0	0	0	0	0	0
Hylemonella	1	4	0	0	0	0	0	0	0	0	0	0	0	0	0	0	0	0	0	0

Genus	Cluster Number	Protein Counts	1.14.14.1	1.14.99.-	1.17.3.2	1.17.99.1	1.17.99.2	1.18.6.1	1.2.1.-	1.2.1.10	1.2.1.28	1.2.1.3	1.2.1.39	1.2.1.5	1.2.1.65	1.2.7.1	1.2.99.2	1.3.1.-	1.3.1.2	1.3.1.25
**Hyperthermus**	1	6	0	0	0	0	0	0	0	0	0	0	0	0	0	0	0	0	0	0
**Hyphomicrobium**	2	93	0	1	0	0	0	1	0	0	0	1	0	0	0	0	0	0	0	0
**Hyphomonas**	3	105	0	0	0	0	0	0	0	0	0	1	0	0	0	0	0	0	0	0
**Ideonella**	1	18	0	0	0	0	1	1	0	1	0	0	0	0	0	0	0	0	0	0
**Idiomarina**	2	86	0	0	0	0	0	0	0	0	0	1	0	0	0	0	0	0	0	0
**Ignavibacterium**	1	14	0	0	0	0	0	0	0	0	0	0	0	0	0	0	0	0	0	0
**Ignicoccus**	1	5	0	0	0	0	0	0	0	0	0	0	0	0	0	0	0	0	0	0
**Ignisphaera**	1	8	0	0	0	0	0	0	0	0	0	0	0	0	0	0	0	0	0	0
**Ilyobacter**	2	85	0	0	0	0	0	1	0	1	0	0	0	0	0	1	0	0	0	0
**Intrasporangium**	2	114	0	0	0	0	0	0	0	1	0	1	0	0	0	0	1	0	1	0
**Isoptericola**	1	18	0	0	0	0	0	0	0	0	0	0	0	0	0	0	0	0	0	0
**Isosphaera**	2	75	0	1	0	0	0	0	0	0	0	1	0	0	0	0	0	0	0	0
**Jannaschia**	3	151	0	1	0	0	0	0	0	0	0	1	0	0	0	0	1	0	1	0
**Janthinobacterium**	2	99	0	0	0	0	0	0	0	0	0	0	0	0	0	0	1	0	0	0
**Jonesia**	2	56	0	1	0	0	0	0	0	0	0	1	0	0	0	0	0	0	0	0
**Joostella**	1	3	0	0	0	0	0	0	0	0	0	0	0	0	0	0	0	0	0	0
**Kaistia**	1	2	0	0	0	0	0	0	0	0	0	0	0	0	0	0	0	0	0	0
**Kangiella**	2	84	0	0	0	0	0	0	0	0	0	1	0	0	0	0	0	0	0	0
**Ketogulonicigenium**	2	102	0	0	0	0	0	0	0	0	0	1	0	0	0	0	0	0	0	0
**Kineococcus**	1	24	0	0	0	0	0	0	0	0	0	0	0	0	0	0	0	0	0	0
**Kinetoplastibacterium**	1	2	0	0	0	0	0	0	0	0	0	0	0	0	0	0	0	0	0	0
**Kingella**	1	4	0	0	0	0	0	0	0	0	0	0	0	0	0	0	0	0	0	0
**Kitasatospora**	1	36	0	0	0	0	0	0	0	0	0	0	0	0	0	0	0	0	0	0
**Klebsiella**	3	222	0	0	0	0	0	1	1	1	0	1	1	0	0	0	0	0	0	1
**Kluyvera**	1	1	0	0	0	0	0	0	0	0	0	0	0	0	0	0	0	0	0	0
**Kocuria**	2	87	0	1	0	0	0	0	0	0	0	1	0	0	0	0	0	0	0	0
**Kordia**	1	5	0	0	0	0	0	0	0	0	0	0	0	0	0	0	0	0	0	0
**Kosmotoga**	2	61	0	0	0	0	0	0	0	0	0	0	0	0	0	1	0	0	0	0
**Kribbella**	2	99	0	1	0	0	0	0	0	0	0	1	0	0	0	0	0	0	0	0
**Krokinobacter**	2	90	0	1	0	0	0	0	0	0	0	1	0	0	0	0	0	0	0	0
**Kyrpidia**	2	113	0	0	0	0	0	0	0	1	0	1	1	0	0	0	1	0	0	0
**Kytococcus**	2	70	0	1	0	0	0	0	0	0	0	1	0	0	0	0	0	0	0	0
**Labrenzia**	1	4	0	0	0	0	0	0	0	0	0	0	0	0	0	0	0	0	0	0
**Lachnoanaerobaculum**	1	1	0	0	0	0	0	0	0	0	0	0	0	0	0	0	0	0	0	0
**Lachnospiraceae**	1	51	0	0	0	0	0	0	0	0	0	1	0	0	0	1	0	0	1	0
**Lacinutrix**	1	20	0	0	0	0	0	0	0	0	0	0	0	0	0	0	0	0	0	0
**Lactobacillus**	2	119	0	1	0	0	0	0	0	1	0	1	0	0	0	0	0	0	1	0
**Lactococcus**	2	82	0	0	0	0	0	0	0	1	0	0	0	0	0	0	0	0	0	0
**Lamprocystis**	1	2	0	0	0	0	0	0	0	0	0	0	0	0	0	0	0	0	0	0
**Laribacter**	2	80	0	0	0	0	0	0	0	0	0	1	0	0	0	0	0	0	0	0
**Lawsonia**	2	33	0	0	0	0	0	0	0	0	0	0	0	0	0	0	0	0	0	0
**Leadbetterella**	2	79	0	0	0	0	0	0	0	0	0	1	0	0	0	0	0	0	0	0
**Leeuwenhoekiella**	1	2	0	0	0	0	0	0	0	0	0	0	0	0	0	0	0	0	0	0
**Legionella**	2	117	0	0	0	0	0	0	0	0	0	1	0	0	0	0	0	0	0	0
**Leifsonia**	2	48	0	1	0	0	0	0	0	0	0	1	0	0	0	0	0	0	0	0
**Leptolyngbya**	1	10	0	0	0	0	0	0	0	0	0	0	0	0	0	0	0	0	0	0
**Leptospira**	2	76	0	0	0	0	0	0	0	0	0	1	0	0	0	0	0	0	0	0
**Leptospirillum**	1	10	0	0	0	0	0	0	0	0	0	0	0	0	0	0	0	0	0	0
**Leptothrix**	3	186	0	0	0	0	0	1	0	1	0	1	0	0	0	1	0	0	0	0
**Leptotrichia**	2	43	0	0	0	0	0	0	0	0	0	0	0	0	0	0	0	0	0	0
**Leucobacter**	1	2	0	0	0	0	0	0	0	0	0	0	0	0	0	0	0	0	0	0
**Leuconostoc**	2	64	0	1	0	0	0	0	0	1	0	1	0	0	0	0	0	0	0	0

Genus	Cluster Number	Protein Counts	1.14.14.1	1.14.99.-	1.17.3.2	1.17.99.1	1.17.99.2	1.18.6.1	1.2.1.-	1.2.1.10	1.2.1.28	1.2.1.3	1.2.1.39	1.2.1.5	1.2.1.65	1.2.7.1	1.2.99.2	1.3.1.-	1.3.1.2	1.3.1.25
**Liberibacter**	1	4	0	0	0	0	0	0	0	0	0	0	0	0	0	0	0	0	0	0
**Limnohabitans**	1	11	0	0	0	0	0	0	0	0	0	1	0	0	0	0	0	0	0	0
**Listeria**	2	77	0	0	0	0	0	0	1	1	0	0	0	0	0	0	0	0	0	0
**Listeriaceae**	1	6	0	0	0	0	0	0	0	1	0	0	0	0	0	0	0	0	0	0
**Listonella**	1	38	0	0	0	0	0	0	0	1	0	0	0	0	0	0	0	0	0	0
**Loktanella**	1	3	0	0	0	0	0	0	0	0	0	0	0	0	0	0	0	0	0	0
**Lysinibacillus**	2	97	0	1	0	0	0	0	0	0	0	1	0	0	0	0	0	0	1	0
**Macrococcus**	2	60	0	1	0	0	0	0	0	0	0	1	0	0	0	0	0	0	0	0
**Magnetococcus**	2	64	0	0	0	0	0	1	0	0	0	1	0	0	0	0	0	0	0	0
**Magnetospirillum**	2	113	0	1	0	0	0	1	0	0	0	1	0	0	0	1	0	0	0	0
**Mahella**	2	58	0	0	0	0	0	0	0	1	0	1	0	0	0	1	0	0	0	0
**Mannheimia**	2	85	0	0	0	0	0	0	0	1	0	1	0	0	0	0	0	0	0	0
**Maribacter**	3	87	0	1	0	0	0	0	0	0	0	1	0	0	0	0	0	0	0	0
**Maricaulis**	3	87	0	0	0	0	0	0	0	0	0	1	0	0	0	0	0	0	0	0
**Marichromatium**	1	2	0	0	0	0	0	0	0	0	0	0	0	0	0	0	0	0	0	0
**Marinithermus**	2	77	0	0	0	0	0	0	1	0	0	1	0	0	0	0	0	0	0	0
**Marinitoga**	1	15	0	0	0	0	0	0	0	0	0	0	0	0	0	0	0	0	0	0
**Marinobacter**	2	163	0	1	0	0	0	0	0	1	0	1	0	0	0	0	0	0	0	1
**Marinomonas**	3	192	0	1	0	0	0	0	0	1	1	1	1	0	0	0	0	1	0	1
**Maritimibacter**	1	7	0	0	0	0	0	0	0	0	0	0	0	0	0	0	0	0	0	0
**Marivirga**	2	96	0	1	0	0	0	0	0	0	0	1	0	0	0	0	0	0	0	0
**Martelella**	1	3	0	0	0	0	0	0	0	0	0	0	0	0	0	0	0	0	0	0
**Marvinbryantia**	1	1	0	0	0	0	0	0	0	0	0	0	0	0	0	0	0	0	0	0
**Massilia**	1	4	0	0	0	0	0	0	0	0	0	0	0	0	0	0	0	0	0	0
**Mastigocladopsis**	1	16	0	0	0	0	0	1	0	0	0	0	0	0	0	0	0	0	0	0
**Megamonas**	1	47	0	0	0	0	0	0	0	0	0	0	0	0	0	0	0	0	0	0
**Megasphaera**	1	14	0	0	0	0	0	0	0	0	0	0	0	0	0	0	0	0	0	0
**Meiothermus**	2	110	0	1	0	0	0	0	1	0	0	1	0	0	0	0	1	0	1	0
**Melissococcus**	2	46	0	0	0	0	0	0	0	0	0	0	0	0	0	0	0	0	0	0
**Mesoflavibacter**	1	6	0	0	0	0	0	0	0	0	0	0	0	0	0	0	0	0	0	0
**Mesoplasma**	1	23	0	0	0	0	0	0	0	0	0	0	0	0	0	0	0	0	0	0
**Mesorhizobium**	2	201	0	0	0	0	0	1	0	0	0	1	0	0	0	0	1	1	1	0
**Mesotoga**	1	14	0	0	0	0	0	0	0	0	0	0	0	0	0	0	0	0	0	0
**Metallosphaera**	1	18	0	0	0	0	0	0	0	0	0	0	0	0	0	0	0	0	0	0
**Methanobacterium**	1	7	0	0	0	0	0	0	0	0	0	0	0	0	0	0	0	0	0	0
**Methanobrevibacter**	1	9	0	0	0	0	0	0	0	0	0	0	0	0	0	0	0	0	0	0
**Methanocaldococcus**	1	6	0	0	0	0	0	0	0	0	0	0	0	0	0	0	0	0	0	0
**Methanocella**	1	15	0	0	0	0	0	0	0	0	0	0	0	0	0	0	0	0	0	0
**Methanococcaceae**	1	1	0	0	0	0	0	0	0	0	0	0	0	0	0	0	0	0	0	0
**Methanococcoides**	1	5	0	0	0	0	0	0	0	0	0	0	0	0	0	0	0	0	0	0
**Methanococcus**	1	9	0	0	0	0	0	0	0	0	0	0	0	0	0	0	0	0	0	0
**Methanocorpusculum**	1	7	0	0	0	0	0	0	0	0	0	0	0	0	0	0	0	0	0	0
**Methanoculleus**	1	8	0	0	0	0	0	0	0	0	0	0	0	0	0	0	0	0	0	0
**Methanohalobium**	1	5	0	0	0	0	0	0	0	0	0	0	0	0	0	0	0	0	0	0
**Methanohalophilus**	1	5	0	0	0	0	0	0	0	0	0	0	0	0	0	0	0	0	0	0
**Methanolobus**	1	5	0	0	0	0	0	0	0	0	0	0	0	0	0	0	0	0	0	0
**Methanomethylovorans**	1	5	0	0	0	0	0	0	0	0	0	0	0	0	0	0	0	0	0	0
**Methanoplanus**	1	7	0	0	0	0	0	0	0	0	0	0	0	0	0	0	0	0	0	0
**Methanopyrus**	1	4	0	0	0	0	0	0	0	0	0	0	0	0	0	0	0	0	0	0
**Methanoregula**	1	8	0	0	0	0	0	0	0	0	0	0	0	0	0	0	0	0	0	0
**Methanosaeta**	1	9	0	0	0	0	0	0	0	0	0	0	0	0	0	0	0	0	0	0
**Methanosalsum**	1	7	0	0	0	0	0	0	0	0	0	0	0	0	0	0	0	0	0	0

Genus	Cluster Number	Protein Counts	1.14.14.1	1.14.99.-	1.17.3.2	1.17.99.1	1.17.99.2	1.18.6.1	1.2.1.-	1.2.1.10	1.2.1.28	1.2.1.3	1.2.1.39	1.2.1.5	1.2.1.65	1.2.7.1	1.2.99.2	1.3.1.-	1.3.1.2	1.3.1.25
Methanosarcina	1	18	0	0	0	0	0	0	0	0	0	0	0	0	0	0	0	0	0	0
Methanosphaera	1	7	0	0	0	0	0	0	0	0	0	0	0	0	0	0	0	0	0	0
Methanosphaerula	1	8	0	0	0	0	0	0	0	0	0	0	0	0	0	0	0	0	0	0
Methanospirillum	1	6	0	0	0	0	0	0	0	0	0	0	0	0	0	0	0	0	0	0
Methanothermobacter	1	6	0	0	0	0	0	0	0	0	0	0	0	0	0	0	0	0	0	0
Methanothermococcus	1	5	0	0	0	0	0	0	0	0	0	0	0	0	0	0	0	0	0	0
Methanothermus	1	5	0	0	0	0	0	0	0	0	0	0	0	0	0	0	0	0	0	0
Methanotorris	1	5	0	0	0	0	0	0	0	0	0	0	0	0	0	0	0	0	0	0
Methylacidiphilum	2	48	0	0	0	0	0	1	0	0	0	1	0	0	0	0	0	0	1	0
Methylarcula	1	2	0	0	0	0	0	0	0	0	0	0	0	0	0	0	0	0	0	0
Methylibium	1	38	0	0	0	0	0	0	0	0	0	1	0	0	0	0	0	0	0	0
Methylobacillus	2	61	0	0	0	0	0	0	0	0	0	1	0	0	0	0	0	0	0	0
Methylobacterium	3	236	0	1	0	0	0	1	0	0	0	1	0	0	0	0	1	0	0	1
Methylocella	3	117	0	1	0	0	0	1	0	1	0	1	0	0	0	0	0	0	0	0
Methylococcus	2	80	0	0	0	0	0	1	0	0	0	0	0	0	0	0	0	0	0	0
Methylocystis	1	21	0	0	0	0	0	1	0	0	0	0	0	0	0	0	0	0	0	0
Methyloferula	1	2	0	0	0	0	0	1	0	0	0	0	0	0	0	0	0	0	0	0
Methylomicrobium	1	12	0	0	0	0	0	0	0	0	0	0	0	0	0	0	0	0	0	0
Methylomonas	1	15	0	0	0	0	0	0	0	0	0	0	0	0	0	0	0	0	0	0
Methylophaga	1	7	0	0	0	0	0	0	0	0	0	0	0	0	0	0	0	0	0	0
Methylophilus	1	2	0	0	0	0	0	0	0	0	0	0	0	0	0	0	0	0	0	0
Methylosinus	1	2	0	0	0	0	0	1	0	0	0	0	0	0	0	0	0	0	0	0
Methylotenera	2	72	0	1	0	0	0	0	0	0	0	1	0	0	0	0	0	0	0	0
Methyloversatilis	1	5	0	0	0	0	0	1	0	0	0	0	0	0	0	0	0	0	0	0
Methylovorus	2	66	0	0	0	0	0	0	0	0	0	1	0	0	0	0	0	0	0	0
Micavibrio	1	9	0	0	0	0	0	0	0	0	0	0	0	0	0	0	0	0	0	0
Microbacterium	2	115	0	1	0	0	0	0	0	0	0	1	0	0	0	0	0	0	0	0
Microchaete	1	23	0	0	0	0	0	1	0	0	0	0	0	0	0	0	0	0	0	0
Micrococcus	2	72	0	1	0	0	0	0	0	0	0	1	0	0	0	0	0	0	0	0
Micrococleus	1	17	0	0	0	0	0	0	0	0	0	0	0	0	0	0	0	0	0	0
Microcystis	2	67	0	0	0	0	0	0	0	0	0	1	0	0	0	0	0	0	0	0
Microlunatus	1	25	0	0	0	0	0	0	0	0	0	0	0	0	0	0	0	0	0	0
Micromonospora	2	115	0	1	0	0	0	0	0	1	0	1	0	0	0	0	1	0	0	0
Microvirga	1	6	0	0	0	0	0	1	0	0	0	0	0	0	0	0	0	0	0	0
Mobiluncus	1	41	0	0	0	0	0	0	0	0	0	1	0	0	0	1	0	0	0	0
Modestobacter	1	43	0	0	0	0	0	0	0	0	0	0	0	0	0	0	0	0	0	0
Moorea	1	2	0	0	0	0	0	0	0	0	0	0	0	0	0	0	0	0	0	0
Moorella	2	69	0	0	0	0	0	1	0	1	0	0	0	0	0	1	1	0	0	0
Moraxella	2	66	0	0	0	0	0	0	0	0	0	1	0	0	0	0	0	0	0	0
Morganella	1	29	0	0	0	0	0	0	0	0	0	0	0	0	0	0	0	0	0	0
Moritella	1	2	0	0	0	0	0	0	0	0	0	0	0	0	0	0	0	0	0	0
Muricauda	1	23	0	0	0	0	0	0	0	0	0	0	0	0	0	0	0	0	0	0
Mycobacterium	3	306	0	1	0	0	0	0	0	1	0	1	0	0	0	0	1	1	0	1
Mycoplasma	1	53	0	0	0	0	0	0	0	0	0	1	0	0	0	0	0	0	0	0
Myxococcus	2	110	0	1	0	0	0	0	0	0	0	1	0	0	0	0	0	1	0	0
Nakamurella	2	115	0	1	0	0	0	0	0	1	0	1	0	0	0	0	1	0	0	0
napthalene	1	2	0	0	0	0	0	0	0	0	0	0	0	0	0	0	0	0	0	0
Natranaerobius	2	55	0	0	0	0	0	0	0	0	0	0	0	0	0	0	0	0	0	0
Natrialba	1	16	0	0	0	0	0	0	0	0	0	0	0	0	0	0	0	0	0	0
Natrinema	1	16	0	0	0	0	0	0	0	0	0	0	0	0	0	0	0	0	0	0
Natronobacterium	1	11	0	0	0	0	0	0	0	0	0	0	0	0	0	0	0	0	0	0
Natronococcus	1	21	0	0	0	0	0	0	0	0	0	0	0	0	0	0	0	0	0	0

Genus	Cluster Number	Protein Counts	1.14.14.1	1.14.99.-	1.17.3.2	1.17.99.1	1.17.99.2	1.18.6.1	1.2.1.-	1.2.1.10	1.2.1.28	1.2.1.3	1.2.1.39	1.2.1.5	1.2.1.65	1.2.7.1	1.2.99.2	1.3.1.-	1.3.1.2	1.3.1.25
**Natronomonas**	1	20	0	0	0	0	0	0	0	0	0	0	0	0	0	0	0	0	0	0
**Nautilia**	2	48	0	0	0	0	0	0	0	0	0	0	0	0	0	1	0	0	0	0
**Neisseria**	2	67	0	0	0	0	0	0	0	0	0	0	0	0	0	0	0	0	0	0
**Neisseriaceae**	1	1	0	0	0	0	0	0	0	0	0	0	0	0	0	0	0	0	0	0
**Neorickettsia**	1	31	0	0	0	0	0	0	0	0	0	0	0	0	0	0	0	0	0	0
**Neptuniibacter**	1	2	0	0	0	0	0	0	0	0	0	0	0	0	0	0	0	0	0	0
**Niastella**	1	21	0	0	0	0	0	0	0	0	0	0	0	0	0	0	0	0	0	0
**Nitratifractor**	2	54	0	0	0	0	0	0	0	0	0	0	0	0	0	1	0	0	0	0
**Nitratireductor**	1	19	0	0	0	0	0	0	0	0	0	0	0	0	0	0	0	0	1	0
**Nitratiruptor**	2	40	0	0	0	0	0	0	0	0	0	0	0	0	0	1	0	0	0	0
**Nitrobacter**	2	99	0	0	0	0	0	0	0	0	0	1	0	0	0	0	1	0	0	0
**Nitrococcus**	1	2	0	0	0	0	0	0	0	0	0	0	0	0	0	0	0	0	0	0
**Nitrosococcus**	2	94	0	1	0	1	0	0	0	0	0	1	0	0	0	0	0	0	0	0
**Nitrosomonas**	2	72	0	0	0	1	0	0	0	0	0	1	0	0	0	0	0	0	0	0
**Nitrosopumilaceae**	1	7	0	0	0	0	0	0	0	0	0	0	0	0	0	0	0	0	0	0
**Nitrosopumilus**	1	11	0	0	0	0	0	0	0	0	0	0	0	0	0	0	0	0	0	0
**Nitrosospira**	2	74	0	0	0	1	0	0	0	0	0	0	0	0	0	0	0	0	0	0
**Nocardia**	2	185	0	1	0	0	0	0	0	1	0	1	0	0	0	0	0	0	0	0
**Nocardioidaceae**	1	2	0	0	0	0	0	0	0	0	0	0	0	0	0	0	0	0	0	0
**Nocardioides**	2	157	0	1	0	0	0	0	0	1	0	1	0	0	0	1	1	0	0	0
**Nocardiopsis**	2	102	0	1	0	0	0	0	0	0	0	1	0	0	0	0	0	0	0	0
**Nodularia**	1	22	0	0	0	0	0	1	0	0	0	0	0	0	0	0	0	0	0	0
**Nonlabens**	1	19	0	0	0	0	0	0	0	0	0	0	0	0	0	0	0	0	0	0
**Nostoc**	2	111	1	1	0	0	0	1	0	0	0	1	0	0	0	0	0	0	0	0
**Nostocaceae**	1	2	0	0	0	0	0	0	0	0	0	0	0	0	0	0	0	0	0	0
**Novosphingobium**	2	169	0	0	0	1	0	0	0	1	0	1	0	0	0	0	0	0	0	1
**Oceanibaculum**	1	3	0	0	0	0	0	0	0	0	0	0	0	0	0	0	0	0	0	0
**Oceanibulbus**	1	12	0	0	0	0	0	0	0	0	0	0	0	0	0	0	0	0	0	0
**Oceanicola**	1	9	0	0	0	0	0	0	0	0	0	1	0	0	0	0	0	0	0	0
**Oceanimonas**	1	33	0	0	0	0	0	0	0	0	0	0	0	0	0	0	0	0	0	0
**Oceaniovalibus**	1	2	0	0	0	0	0	0	0	0	0	0	0	0	0	0	0	0	0	0
**Oceanithermus**	2	72	0	0	0	0	0	0	1	0	0	1	0	0	0	0	0	0	0	0
**Oceanobacillus**	2	91	0	1	0	0	0	0	0	0	1	1	0	0	0	0	0	0	0	0
**Oceanospirillum**	1	2	0	0	0	0	0	0	0	0	0	0	0	0	0	0	0	0	0	0
**Ochrobactrum**	2	133	0	0	0	0	0	0	0	0	0	1	0	0	0	0	0	0	1	0
**Octadecabacter**	1	29	0	0	0	0	0	0	0	0	0	0	0	0	0	0	0	0	0	0
**Oenococcus**	2	41	0	0	0	0	0	0	0	1	0	0	0	0	0	0	0	0	0	0
**Oleomonas**	1	2	0	0	0	0	0	0	0	0	0	0	0	0	0	0	0	0	0	0
**Oligotropha**	3	87	0	0	0	0	0	0	0	0	0	1	0	0	0	0	0	0	0	0
**Olsenella**	2	40	0	0	0	0	0	0	0	0	0	0	0	0	0	0	0	0	0	0
**Opitutus**	2	67	0	0	0	0	0	0	0	0	0	1	0	0	0	0	0	0	0	0
**Orientia**	1	21	0	0	0	0	0	0	0	0	0	0	0	0	0	0	0	0	0	0
**Ornithobacterium**	1	11	0	0	0	0	0	0	0	0	0	0	0	0	0	0	0	0	0	0
**Oscillatoria**	1	16	0	0	0	0	0	0	0	0	0	0	0	0	0	0	0	0	0	0
**Oscillatoriales**	1	2	0	0	0	0	0	0	0	0	0	0	0	0	0	0	0	0	0	0
**Oscillibacter**	1	20	0	0	0	0	0	0	0	0	0	0	0	0	0	0	0	0	0	0
**Owenweeksia**	1	15	0	0	0	0	0	0	0	0	0	0	0	0	0	0	0	0	0	0
**Oxalobacteraceae**	1	4	0	0	0	0	0	0	0	0	0	0	0	0	0	0	0	0	0	0
**Paenibacillus**	2	152	0	1	0	0	0	0	0	0	0	1	0	0	0	1	0	0	1	0
**Paludibacter**	2	67	0	1	0	0	0	1	0	0	0	0	0	0	0	0	0	0	0	0
**Pandoraea**	1	18	0	0	0	0	0	0	0	0	0	0	0	0	0	0	0	1	0	0
**Pannonibacter**	1	3	0	0	0	0	0	0	0	0	0	0	0	0	0	0	0	0	0	0

Genus	Cluster Number	Protein Counts	1.14.14.1	1.14.99.-	1.17.3.2	1.17.99.1	1.17.99.2	1.18.6.1	1.2.1.-	1.2.1.10	1.2.1.28	1.2.1.3	1.2.1.39	1.2.1.5	1.2.1.65	1.2.7.1	1.2.99.2	1.3.1.-	1.3.1.2	1.3.1.25
**Pantholops**	1	23	0	0	0	0	0	0	0	0	0	0	0	0	0	0	0	0	0	0
**Pantoea**	2	181	0	1	0	0	0	1	0	1	0	1	0	0	0	0	1	0	0	0
**Parabacteroides**	2	64	0	0	0	0	0	0	0	0	0	0	0	0	0	0	0	0	1	0
**Parachlamydia**	1	11	0	0	0	0	0	0	0	0	0	0	0	0	0	0	0	0	0	0
**Paracoccus**	2	169	0	0	0	0	0	0	0	0	0	1	0	0	0	0	0	0	0	1
**Parvibaculum**	3	117	0	0	0	0	0	0	0	0	0	1	0	0	0	0	1	0	0	0
**Parvularcula**	2	72	0	1	0	0	0	0	0	0	0	1	0	0	0	0	0	0	0	0
**Pasteurella**	2	85	0	0	0	0	0	0	0	1	0	0	0	0	0	0	0	0	0	0
**Pasteurellaceae**	1	2	0	0	0	0	0	0	0	0	0	0	0	0	0	0	0	0	0	0
**Pectobacterium**	1	44	0	0	0	0	0	0	0	0	0	0	0	0	0	0	0	0	0	0
**Pediococcus**	1	43	0	0	0	0	0	0	0	0	0	0	0	0	0	0	0	0	0	0
**Pedobacter**	2	88	0	1	0	0	0	0	0	0	0	1	0	0	0	0	0	0	0	0
**Pelagibaca**	1	10	0	0	0	0	0	0	0	0	0	0	0	0	0	0	0	0	0	0
**Pelagibacterium**	1	39	0	0	0	0	0	0	0	0	0	0	0	0	0	0	0	0	0	0
**Pelobacter**	2	85	0	0	0	0	0	1	0	1	0	1	0	0	0	1	1	0	0	0
**Pelodictyon**	2	62	0	1	0	0	0	1	0	0	0	1	0	0	0	0	0	0	0	0
**Pelosinus**	1	1	0	0	0	0	0	0	0	0	0	0	0	0	0	0	0	0	0	0
**Pelotomaculum**	2	60	0	0	0	0	0	0	0	1	0	0	0	0	0	0	0	0	0	0
**Peptostreptococcus**	1	1	0	0	0	0	0	0	0	0	0	0	0	0	0	0	0	0	0	0
**Persephonella**	2	55	0	0	0	0	0	0	0	0	0	0	0	0	0	1	0	0	0	0
**Petrotoga**	2	72	0	0	0	0	0	0	0	0	0	0	0	0	0	1	0	0	0	0
**Phaeobacter**	2	55	0	0	0	0	0	0	0	0	0	0	0	0	0	0	1	0	1	0
**Phenylobacterium**	3	103	0	0	0	0	0	0	0	0	0	1	0	0	0	0	0	0	0	0
**Photobacterium**	2	119	0	0	0	0	0	0	0	1	0	1	0	0	0	0	0	1	0	0
**Photorhabdus**	2	149	0	1	0	0	0	0	0	0	0	1	1	0	0	0	0	1	0	0
**Phycisphaera**	1	9	0	0	0	0	0	0	0	0	0	0	0	0	0	0	0	0	0	0
**Phyllobacterium**	1	6	0	0	0	0	0	0	0	0	0	0	0	0	0	0	0	0	0	0
**Picrophilus**	1	15	0	0	0	0	0	0	0	0	0	0	0	0	0	0	0	0	0	0
**Pirellula**	2	85	0	1	0	0	0	0	0	0	0	1	0	0	0	0	0	0	0	0
**Planctomyces**	2	93	0	1	0	0	0	0	0	0	0	1	0	0	0	0	0	0	0	0
**Plautia**	1	17	0	0	0	0	0	0	0	0	0	0	0	0	0	0	0	0	0	0
**Plesiomonas**	1	4	0	0	0	0	0	0	0	1	0	0	0	0	0	0	0	0	0	0
**Pleurocapsa**	1	23	0	0	0	0	0	0	0	0	0	0	0	0	0	0	0	0	0	0
**Polaribacter**	2	74	0	1	0	0	0	0	0	0	0	1	0	0	0	0	0	0	0	0
**Polaromonas**	3	238	0	1	0	1	0	1	0	1	1	1	0	0	0	0	1	1	1	1
**Polymorphum**	3	173	0	0	0	0	0	0	0	1	0	1	0	0	0	0	1	1	1	0
**Polynucleobacter**	1	19	0	0	0	0	0	0	0	0	0	0	0	0	0	0	0	0	0	0
**Ponticaulis**	1	1	0	0	0	0	0	0	0	0	0	0	0	0	0	0	0	0	0	0
**Porphyromonas**	2	63	0	0	0	0	0	0	0	0	0	0	0	0	0	0	0	0	0	0
**Prevotella**	1	65	0	0	0	0	0	0	0	0	0	0	0	0	0	0	0	1	1	0
**Prochlorococcus**	2	68	0	1	0	0	0	0	0	0	0	1	0	0	0	0	0	0	0	0
**Propionibacterium**	1	62	0	0	0	0	0	0	0	0	0	0	0	0	0	0	0	0	0	0
**Prosthecochloris**	1	16	0	0	0	0	0	1	0	0	0	0	0	0	0	0	0	0	0	0
**Proteobacteria**	1	19	0	0	0	0	0	0	0	1	0	0	0	0	0	0	0	0	0	0
**Proteus**	2	113	0	0	0	0	0	0	0	0	0	1	0	0	0	0	0	0	0	0
**Providencia**	1	38	0	0	0	0	0	0	0	0	0	0	0	0	0	0	0	0	0	0
**Pseudaminobacter**	1	10	0	0	0	0	0	0	0	0	0	0	0	0	0	0	0	0	1	0
**Pseudanabaena**	1	11	0	0	0	0	0	0	0	0	0	0	0	0	0	0	0	0	0	0
**Pseudoalteromonas**	2	188	0	0	0	1	0	0	0	1	0	1	0	0	0	0	0	0	0	0
**Pseudochrobactrum**	1	7	0	0	0	0	0	0	0	0	0	1	0	0	0	0	0	0	0	0
**Pseudogulbenkiania**	1	40	0	0	0	0	0	0	0	0	0	0	0	0	0	0	0	0	0	0
**pseudomallei**	3	131	0	0	0	0	0	0	0	0	0	1	1	0	0	0	0	0	0	0

Genus	Cluster Number	Protein Counts	1.14.14.1	1.14.99.-	1.17.3.2	1.17.99.1	1.17.99.2	1.18.6.1	1.2.1.-	1.2.1.10	1.2.1.28	1.2.1.3	1.2.1.39	1.2.1.5	1.2.1.65	1.2.7.1	1.2.99.2	1.3.1.-	1.3.1.2	1.3.1.25
**Pseudomonas**	3	381	0	1	0	0	0	1	1	1	1	1	1	0	0	0	0	1	1	1
**Pseudonocardia**	3	180	0	0	0	0	0	0	0	1	0	1	0	0	0	0	1	0	0	0
**Pseudorhodobacter**	1	4	0	0	0	0	0	0	0	0	0	0	0	0	0	0	0	0	0	0
**Pseudovibrio**	1	33	0	0	0	0	0	0	0	0	0	0	0	0	0	0	0	0	0	0
**Pseudoxanthomonas**	2	124	0	0	0	0	0	0	0	1	0	1	0	0	0	0	0	0	0	0
**Psychrobacter**	2	124	0	0	0	0	0	0	0	0	0	1	0	0	0	0	0	0	0	1
**Psychroflexus**	1	22	0	0	0	0	0	0	0	0	0	0	0	0	0	0	0	0	0	0
**Psychromonas**	2	110	0	0	0	0	0	0	0	0	0	1	0	0	0	0	0	0	0	0
**Pusillimonas**	3	111	0	0	0	0	0	0	0	1	0	1	0	0	0	0	0	0	0	0
**Pyrobaculum**	1	15	0	0	0	0	0	0	0	0	0	0	0	0	0	0	0	0	0	0
**Pyrococcus**	1	10	0	0	0	0	0	0	0	0	0	0	0	0	0	0	0	0	0	0
**Pyrolobus**	1	4	0	0	0	0	0	0	0	0	0	0	0	0	0	0	0	0	0	0
**Rahnella**	2	143	0	0	0	0	0	0	0	0	0	1	0	0	0	0	0	0	0	0
**Ralstonia**	3	332	0	1	0	0	0	0	0	1	1	1	0	0	0	0	1	1	1	1
**Ramlibacter**	1	35	0	0	0	0	0	0	0	0	0	0	0	0	0	0	0	0	0	0
**Raoultella**	2	139	0	0	0	0	0	1	0	1	0	1	1	0	0	0	0	0	0	0
**Raphidiopsis**	1	5	0	0	0	0	0	0	0	0	0	0	0	0	0	0	0	0	0	0
**Reinekea**	1	2	0	0	0	0	0	0	0	0	0	0	0	0	0	0	0	0	0	0
**Renibacterium**	2	67	0	0	0	0	0	0	0	0	0	1	0	0	0	0	0	0	0	0
**Rhizobiaceae**	1	3	0	0	0	0	0	0	0	0	0	0	0	0	0	0	0	0	0	0
**Rhizobiales**	1	6	0	0	0	0	0	0	0	0	0	0	0	0	0	0	1	0	0	0
**Rhizobium**	3	257	0	0	0	0	0	1	0	0	0	1	0	0	0	0	1	1	1	0
**Rhizobium/Agrobacterium**	2	87	0	0	0	0	0	0	0	0	0	1	0	0	0	0	1	0	1	0
**Rhodanobacter**	1	21	0	0	0	0	0	0	0	0	0	0	0	0	0	0	0	0	0	0
**Rhodobacter**	3	175	0	1	0	0	0	1	0	0	0	1	0	0	0	0	1	0	1	0
**Rhodobacteraceae**	1	21	0	0	0	0	0	0	0	0	0	0	0	0	0	0	0	0	0	0
**Rhodobacterales**	1	34	0	0	0	0	0	0	0	0	0	0	0	0	0	0	0	0	1	0
**Rhodococcus**	2	292	0	1	0	1	0	0	0	1	1	1	0	0	0	0	1	1	0	1
**Rhodocyclaceae**	1	7	0	0	0	0	0	1	0	0	0	0	0	0	0	0	0	0	0	0
**Rhodoferax**	3	144	0	0	0	0	0	0	0	1	0	1	0	0	0	1	0	0	0	0
**Rhodomicrobium**	2	103	0	1	0	0	0	1	0	0	0	1	0	0	0	0	0	0	0	0
**Rhodopirellula**	2	87	0	1	0	0	0	0	0	0	0	1	0	0	0	0	0	0	0	0
**Rhodopseudomonas**	3	246	0	1	0	0	0	1	0	0	0	1	0	0	0	1	1	0	0	0
**Rhodospirillum**	2	121	0	1	0	0	0	1	0	0	0	1	0	0	0	0	1	0	0	0
**Rhodothermus**	2	76	0	1	0	0	0	0	0	0	0	1	0	0	0	1	1	0	0	0
**Rhodovulum**	1	4	0	0	0	0	0	1	0	0	0	0	0	0	0	0	0	0	0	0
**Richelia**	1	2	0	0	0	0	0	0	0	0	0	0	0	0	0	0	0	0	0	0
**Rickettsia**	2	43	0	0	0	0	0	0	0	0	0	0	0	0	0	0	0	0	0	0
**Rickettsiaceae**	1	5	0	0	0	0	0	0	0	0	0	0	0	0	0	0	0	0	0	0
**Riemerella**	2	61	0	0	0	0	0	0	0	0	0	1	0	0	0	0	0	0	0	0
**Rivularia**	1	18	0	0	0	0	0	0	0	0	0	0	0	0	0	0	0	0	0	0
**Robiginitalea**	2	83	0	1	0	0	0	0	0	0	0	0	0	0	0	0	0	0	0	0
**Roseburia**	2	58	0	0	0	0	0	0	0	0	0	0	0	0	0	1	0	0	0	0
**Roseibium**	1	3	0	0	0	0	0	0	0	0	0	0	0	0	0	0	0	0	0	0
**Roseiflexus**	3	100	0	1	0	0	0	1	1	1	0	1	0	1	0	0	1	0	0	0
**Roseobacter**	2	149	0	1	0	0	0	0	0	0	0	1	0	0	0	0	1	0	1	0
**Roseomonas**	1	2	0	0	0	0	0	0	0	0	0	0	0	0	0	0	0	0	0	0
**Roseovarius**	1	15	0	0	0	0	0	0	0	0	0	0	0	0	0	0	0	0	0	0
**Rothia**	1	44	0	0	0	0	0	0	0	0	0	1	0	0	0	0	0	0	0	0
**Rubrivivax**	1	24	0	0	0	0	0	1	0	0	0	0	0	0	0	0	0	0	0	0
**Rubrobacter**	3	117	0	0	0	0	0	0	0	0	0	1	0	0	0	1	1	0	0	0
**Ruegeria**	2	189	0	0	0	0	0	0	0	0	0	1	0	0	0	0	1	0	1	0

Genus	Cluster Number	Protein Counts	1.14.14.1	1.14.99.-	1.17.3.2	1.17.99.1	1.17.99.2	1.18.6.1	1.2.1.-	1.2.1.10	1.2.1.28	1.2.1.3	1.2.1.39	1.2.1.5	1.2.1.65	1.2.7.1	1.2.99.2	1.3.1.-	1.3.1.2	1.3.1.25
Ruminococcaceae	1	2	0	0	0	0	0	0	0	0	0	0	0	0	0	0	0	0	0	0
Ruminococcus	2	101	0	0	0	0	0	1	0	1	0	1	0	0	0	0	1	0	1	0
Runella	1	26	0	0	0	0	0	0	0	0	0	0	0	0	0	0	0	0	0	0
Saccharomonospora	2	132	0	1	0	0	0	0	0	0	1	1	0	0	0	0	1	0	0	1
Saccharophagus	2	81	0	0	0	0	0	0	0	0	0	0	0	0	0	0	0	0	0	0
Saccharopolyspora	3	194	0	0	0	0	0	0	0	0	1	1	0	0	0	0	1	0	0	1
Saccharothrix	1	32	0	0	0	0	0	0	0	0	0	0	0	0	0	0	0	0	0	0
Sagittula	1	10	0	0	0	0	0	0	0	0	0	0	0	0	0	0	1	0	0	0
Salinibacter	2	77	0	1	0	0	0	0	0	0	0	1	0	0	0	0	0	0	0	0
Salinimonas	1	1	0	0	0	0	0	0	0	0	0	0	0	0	0	0	0	0	0	0
Salinispora	3	127	0	1	0	0	0	0	0	1	0	1	0	0	0	0	0	0	0	0
Salinivibrio	1	2	0	0	0	0	0	0	0	0	0	0	0	0	0	0	0	0	0	0
Salmonella	2	151	0	0	0	0	0	0	0	1	0	1	0	0	0	0	0	0	1	0
Sanguibacter	2	73	0	0	0	0	0	0	0	0	0	1	0	0	0	0	0	0	0	0
Saprospira	1	13	0	0	0	0	0	0	0	0	0	0	0	0	0	0	0	0	0	0
Scardovia	1	1	0	0	0	0	0	0	0	0	0	0	0	0	0	0	0	0	0	0
Scytonema	1	10	0	0	0	0	0	1	0	0	0	0	0	0	0	0	0	0	0	0
Sebaldella	2	69	0	0	0	0	0	0	0	0	0	1	0	0	0	0	0	0	0	0
Segniliparus	2	85	0	0	0	0	0	0	0	1	0	1	0	0	0	0	0	0	0	0
Selenomonas	2	57	0	0	0	0	0	0	0	0	0	0	0	0	0	0	1	0	0	0
Serinicoccus	1	1	0	0	0	0	0	0	0	0	0	0	0	0	0	0	0	0	0	0
Serratia	2	186	0	0	0	0	0	0	1	1	0	1	1	0	0	0	0	0	0	0
Shewanella	3	214	0	1	0	1	0	0	1	1	0	1	0	0	0	0	1	1	0	0
Shigella	2	172	0	0	0	0	0	0	1	1	0	1	1	0	0	0	0	1	1	0
Shinella	1	2	0	0	0	0	0	0	0	0	0	0	0	0	0	0	1	0	0	0
Sideroxydans	2	81	0	0	0	0	0	1	0	0	0	0	0	0	0	0	0	0	0	0
Silicibacter	2	105	0	0	0	0	0	0	0	0	0	1	0	0	0	0	1	0	1	0
Simiduia	1	18	0	0	0	0	0	0	0	0	0	0	0	0	0	0	0	0	0	0
Simkania	1	6	0	0	0	0	0	0	0	0	0	0	0	0	0	0	0	0	0	0
Simonsiella	1	2	0	0	0	0	0	0	0	0	0	0	0	0	0	0	0	0	0	0
Singulisphaera	1	18	0	0	0	0	0	0	0	0	0	0	0	0	0	0	0	0	0	0
Sinorhizobium	3	207	0	1	0	0	0	1	0	0	0	1	1	0	0	0	1	0	1	0
Slackia	2	46	0	0	0	0	0	0	0	1	0	1	0	0	0	0	0	0	0	0
Smaragdicoccus	1	3	0	0	0	0	0	0	0	0	0	0	0	0	0	0	0	0	0	0
Sodalis	2	71	0	0	0	0	0	0	0	0	0	0	0	0	0	0	0	0	0	0
Solibacillus	1	23	0	0	0	0	0	0	0	0	0	1	0	0	0	0	0	0	0	0
Solitalea	1	13	0	0	0	0	0	0	0	0	0	0	0	0	0	0	0	0	0	0
Sorangium	3	115	0	1	0	0	0	0	0	0	0	1	0	0	0	0	0	0	1	0
Sphaerobacter	2	100	0	0	0	0	0	0	0	0	0	1	0	0	0	0	1	0	0	0
Sphaerochaeta	1	42	0	0	0	0	0	0	0	1	0	1	0	0	0	0	0	0	0	0
Sphingobacterium	3	85	0	0	0	0	0	0	0	0	0	1	0	0	0	0	0	0	0	0
Sphingobium	1	179	0	1	0	0	0	0	0	1	0	1	0	0	0	0	0	0	0	0
Sphingomonadaceae	1	15	0	0	0	0	0	0	0	0	0	1	0	0	0	0	0	0	0	0
Sphingomonas	2	220	0	0	0	1	0	0	0	1	0	1	0	0	0	0	1	0	0	1
Sphingopyxis	3	99	0	1	0	0	0	0	0	0	0	1	0	0	0	0	0	0	0	0
Spirochaeta	2	103	0	0	0	0	0	1	0	1	0	1	0	0	0	0	1	0	0	0
Spiroplasma	1	6	0	0	0	0	0	0	0	0	0	0	0	0	0	0	0	0	0	0
Spirosoma	2	107	0	1	0	0	0	0	0	0	0	1	0	0	0	0	0	1	0	0
Stackebrandtia	2	110	0	0	0	0	0	0	0	0	0	1	0	0	0	0	0	0	0	0
Stanieria	1	17	0	0	0	0	0	0	0	0	0	0	0	0	0	0	0	0	0	0
Staphylococcus	2	108	0	1	0	0	0	0	0	1	0	1	0	0	0	0	0	0	0	0
Staphylothermus	1	12	0	0	0	0	0	0	0	0	0	0	0	0	0	0	0	0	0	0

Genus	Cluster Number	Protein Counts	1.14.14.1	1.14.99.-	1.17.3.2	1.17.99.1	1.17.99.2	1.18.6.1	1.2.1.-	1.2.1.10	1.2.1.28	1.2.1.3	1.2.1.39	1.2.1.5	1.2.1.65	1.2.7.1	1.2.99.2	1.3.1.-	1.3.1.2	1.3.1.25
Stappia	1	2	0	0	0	0	0	0	0	0	0	0	0	0	0	0	0	0	0	0
Starkeya	3	134	0	0	0	0	0	0	0	0	0	1	0	0	0	0	1	0	0	0
Stenotrophomonas	3	104	0	1	0	0	0	0	0	0	1	1	0	0	0	0	0	0	0	0
Stigmatella	3	129	0	1	0	0	0	0	0	0	0	1	0	0	0	0	0	0	1	0
Streptobacillus	1	38	0	0	0	0	0	0	0	1	0	0	0	0	0	0	0	0	0	0
Streptococcus	2	121	0	0	0	0	0	0	0	1	0	0	0	0	0	0	0	0	0	0
Streptomyces	2	253	0	1	0	0	0	0	0	1	1	1	0	0	0	0	1	1	0	0
Streptosporangium	3	148	0	1	0	0	0	0	0	1	0	1	0	0	0	0	1	0	0	0
Strigomonas	1	2	0	0	0	0	0	0	0	0	0	0	0	0	0	0	0	0	0	0
Sulfitobacter	1	10	0	0	0	0	0	0	0	0	0	0	0	0	0	0	0	0	0	0
Sulfobacillus	1	30	0	0	0	0	0	0	0	0	0	0	0	0	0	0	0	0	0	0
Sulfolobus	1	23	0	0	0	0	0	0	0	0	0	0	0	0	0	0	0	0	0	0
Sulfuricurvum	2	67	0	0	0	1	0	1	0	0	0	1	0	0	0	1	0	0	0	0
Sulfurihydrogenibium	2	55	0	0	0	0	0	0	0	0	0	1	0	0	0	1	0	0	0	0
Sulfurimonas	2	60	0	0	0	0	0	0	0	0	0	1	0	0	0	1	0	0	0	0
Sulfurospirillum	1	48	0	0	0	0	0	0	0	1	0	1	0	0	0	0	0	0	0	0
Sulfurovum	2	48	0	0	0	0	0	0	0	0	0	0	0	0	0	1	0	0	0	0
Symbiobacterium	2	68	0	0	0	0	0	0	0	0	0	0	0	0	0	1	0	0	0	0
Synechococcus	1	34	0	0	0	0	0	0	0	0	0	0	0	0	0	0	0	0	0	0
Synechocystis	1	40	0	1	0	0	0	0	0	0	0	1	0	0	0	0	0	0	0	0
Synergistetes	1	27	0	0	0	0	0	0	0	0	0	0	0	0	0	0	0	0	0	0
Syntrophobacter	2	101	0	1	0	0	0	1	0	0	0	1	0	0	0	1	1	0	0	0
Syntrophobotulus	2	71	0	0	0	0	0	1	0	0	0	1	0	0	0	0	0	0	1	0
Syntrophomonas	2	58	0	0	0	0	0	1	0	0	0	0	0	0	0	1	0	0	0	0
Syntrophothermus	2	60	0	0	0	0	0	1	0	0	0	0	0	0	0	0	0	0	0	0
Syntrophus	2	66	0	0	0	0	0	0	0	0	0	1	0	0	0	1	0	0	0	0
Tannerella	1	8	0	0	0	0	0	0	0	0	0	0	0	0	0	0	0	0	0	0
Taylorella	1	43	0	0	0	0	0	0	0	0	0	0	0	0	0	0	0	0	0	0
Tepidanaerobacter	2	55	0	0	0	0	0	0	0	0	0	0	0	0	0	1	1	0	0	0
Teredinibacter	2	109	0	0	0	0	0	1	0	1	0	0	0	0	0	0	0	0	0	0
Terrabacter	1	1	0	0	0	0	0	0	0	0	0	0	0	0	0	0	0	0	0	0
Terriglobus	2	83	0	0	0	0	0	0	0	0	0	0	0	0	0	0	0	0	0	0
Tetragenococcus	1	12	0	0	0	0	0	0	0	0	0	0	0	0	0	0	0	0	0	0
Thalassiobium	1	11	0	0	0	0	0	0	0	0	0	0	0	0	0	0	0	0	0	0
Thalassobacter	1	9	0	0	0	0	0	0	0	0	0	0	0	0	0	0	0	0	0	0
Thalassolituus	1	12	0	0	0	0	0	0	0	0	0	0	0	0	0	0	0	0	0	0
Thalassospira	1	2	0	0	0	0	0	0	0	0	0	0	0	0	0	0	0	0	0	0
Thauera	1	111	0	0	0	0	0	0	0	1	0	1	0	0	0	1	0	0	0	1
Thermacetogenium	1	7	0	0	0	0	0	0	0	0	0	0	0	0	0	0	0	0	0	0
Thermaerobacter	2	79	0	1	0	0	0	0	0	1	0	1	0	0	0	0	1	0	0	0
Thermanaerovibrio	2	58	0	0	0	0	0	0	0	0	0	0	0	0	0	0	0	0	0	0
Thermincola	2	62	0	0	0	0	0	1	0	1	0	0	0	0	0	0	1	0	0	0
Thermoanaerobacter	2	76	0	0	0	0	0	0	0	0	0	0	0	0	0	0	1	0	0	0
Thermoanaerobacterium	2	64	0	0	0	0	0	1	0	0	0	0	0	0	0	0	0	0	0	0
Thermobacillus	1	11	0	0	0	0	0	0	0	0	0	0	0	0	0	0	0	0	0	0
Thermobaculum	2	62	0	1	0	0	0	0	0	0	0	1	0	0	0	0	1	0	0	0
Thermobifida	2	72	0	1	0	0	0	0	0	0	0	1	0	0	0	0	1	0	0	0
Thermobispora	2	109	0	0	0	0	0	0	0	0	0	1	0	0	0	0	1	0	0	0
Thermococcus	1	13	0	0	0	0	0	0	0	0	0	0	0	0	0	0	0	0	0	0
Thermocrinis	2	60	0	0	0	0	0	1	0	0	0	1	0	0	0	1	0	0	0	0
Thermodesulfatator	1	10	0	0	0	0	0	0	0	0	0	0	0	0	0	0	0	0	0	0
Thermodesulfobacterium	1	9	0	0	0	0	0	0	0	0	0	0	0	0	0	0	0	0	0	0

Genus	Cluster Number	Protein Counts	1.14.14.1	1.14.99.-	1.17.3.2	1.17.99.1	1.17.99.2	1.18.6.1	1.2.1.-	1.2.1.10	1.2.1.28	1.2.1.3	1.2.1.39	1.2.1.5	1.2.1.65	1.2.7.1	1.2.99.2	1.3.1.-	1.3.1.2	1.3.1.25
**Thermodesulfobium**	1	13	0	0	0	0	0	0	0	0	0	0	0	0	0	0	0	0	0	0
**Thermodesulfovibrio**	2	60	0	0	0	0	0	1	0	0	0	0	0	0	0	1	0	0	0	0
**Thermofilum**	1	9	0	0	0	0	0	0	0	0	0	0	0	0	0	0	0	0	0	0
**Thermogladius**	1	7	0	0	0	0	0	0	0	0	0	0	0	0	0	0	0	0	0	0
**Thermomicrobium**	2	92	0	1	0	0	0	0	0	1	0	1	0	0	0	0	1	1	0	0
**Thermomonospora**	2	125	0	0	0	0	0	0	0	1	0	1	0	0	0	0	1	0	0	0
**Thermoplasma**	1	11	0	0	0	0	0	0	0	0	0	0	0	0	0	0	0	0	0	0
**Thermoplasmatales**	1	6	0	0	0	0	0	0	0	0	0	0	0	0	0	0	0	0	0	0
**Thermoproteus**	1	12	0	0	0	0	0	0	0	0	0	0	0	0	0	0	0	0	0	0
**Thermosediminibacter**	2	64	0	0	0	0	0	0	0	1	0	0	0	0	0	1	1	0	0	0
**Thermosipho**	2	70	0	0	0	0	0	0	0	0	0	1	0	0	0	1	0	0	0	0
**Thermosphaera**	1	10	0	0	0	0	0	0	0	0	0	0	0	0	0	0	0	0	0	0
**Thermosynechococcus**	1	6	0	0	0	0	0	0	0	0	0	0	0	0	0	0	0	0	0	0
**Thermotoga**	2	80	0	0	0	0	0	0	0	0	0	1	0	0	0	1	0	0	0	0
**Thermovibrio**	2	58	0	0	0	0	0	0	0	0	0	1	0	0	0	1	0	0	0	0
**Thermovirga**	1	9	0	0	0	0	0	0	0	0	0	0	0	0	0	0	0	0	0	0
**Thermus**	2	107	0	0	0	0	0	0	1	1	0	1	0	0	0	0	0	0	0	0
**Thioalkalimicrobium**	1	4	0	0	0	0	0	0	0	0	0	0	0	0	0	0	0	0	0	0
**Thioalkalivibrio**	2	91	0	0	0	0	0	0	0	0	0	0	0	0	0	0	0	0	0	0
**Thiobaca**	1	2	0	0	0	0	0	0	0	0	0	0	0	0	0	0	0	0	0	0
**Thiobacillus**	2	67	0	0	0	0	0	0	0	0	0	0	0	0	0	0	0	0	0	0
**Thiocapsa**	1	2	0	0	0	0	0	0	0	0	0	0	0	0	0	0	0	0	0	0
**Thiocystis**	1	11	0	0	0	0	0	0	0	0	0	0	0	0	0	0	0	0	0	0
**Thioflavicoccus**	1	13	0	0	0	0	0	0	0	0	0	0	0	0	0	0	0	0	0	0
**Thiomicrospira**	2	50	0	0	0	0	0	0	0	0	0	0	0	0	0	0	0	0	0	0
**Thiomonas**	2	85	0	0	0	0	0	0	0	0	0	1	0	0	0	0	0	0	0	0
**Thiorhodococcus**	1	2	0	0	0	0	0	0	0	0	0	0	0	0	0	0	0	0	0	0
**Thiothrix**	1	2	0	0	0	0	0	0	0	0	0	1	0	0	0	0	0	0	0	0
**Tistrella**	1	42	0	0	0	0	0	0	0	0	0	0	0	0	0	0	0	0	0	0
**Tolumonas**	2	100	0	0	0	0	0	1	0	0	0	0	0	0	0	0	0	0	0	0
**Transposon**	1	2	0	0	0	0	0	0	0	0	0	0	0	0	0	0	0	0	0	0
**Treponema**	1	63	0	0	0	0	0	0	0	0	0	1	0	0	0	0	0	0	0	0
**Trichodesmium**	2	71	0	1	0	0	0	1	0	0	0	1	0	0	0	0	0	0	0	0
**Trichormus**	1	12	0	0	0	0	0	0	0	0	0	0	0	0	0	0	0	0	0	0
**Tropheryma**	2	22	0	0	0	0	0	0	0	0	0	0	0	0	0	0	0	0	0	0
**Truepera**	2	104	0	1	0	0	0	0	1	0	0	1	0	0	0	0	0	0	1	0
**Tsukamurella**	2	123	0	1	0	0	0	0	0	1	0	1	0	0	0	0	0	0	0	0
**Turneriella**	1	16	0	0	0	0	0	0	0	0	0	0	0	0	0	0	0	0	0	0
**Uliginosibacterium**	1	2	0	0	0	0	0	0	0	0	0	0	0	0	0	0	0	0	0	0
**Ureaplasma**	1	24	0	0	0	0	0	0	0	0	0	0	0	0	0	0	0	0	0	0
**Ureibacillus**	1	2	0	0	0	0	0	0	0	0	0	0	0	0	0	0	0	0	0	0
**Variovorax**	3	187	0	0	0	0	0	0	0	0	0	1	0	0	0	0	1	0	0	0
**Veillonella**	1	54	0	0	0	0	0	0	0	0	0	0	0	0	0	0	0	0	0	0
**Verminephrobacter**	3	188	0	0	0	0	0	0	0	1	0	1	0	0	0	0	1	0	1	0
**Verrucosispora**	3	126	0	1	0	0	0	0	0	1	1	1	0	0	0	0	0	0	0	0
**Vibrio**	2	195	0	1	0	0	0	0	1	1	0	1	0	0	0	0	0	1	0	0
**Vibrionales**	1	46	0	0	0	0	0	0	0	0	0	0	0	0	0	0	0	0	0	0
**Vitreoscilla**	1	1	0	0	0	0	0	0	0	0	0	0	0	0	0	0	0	0	0	0
**Vulcanisaeta**	1	14	0	0	0	0	0	0	0	0	0	0	0	0	0	0	0	0	0	0
**Waddlia**	2	68	0	0	0	0	0	0	0	0	0	0	0	0	0	0	0	0	0	0
**Wautersia**	1	2	0	0	0	0	0	0	0	0	0	0	0	0	0	0	0	0	0	0
**Weeksella**	2	65	0	0	0	0	0	0	0	0	0	1	0	0	0	0	0	0	0	0

Genus	Cluster Number	Protein Counts	1.14.14.1	1.14.99.-	1.17.3.2	1.17.99.1	1.17.99.2	1.18.6.1	1.2.1.-	1.2.1.10	1.2.1.28	1.2.1.3	1.2.1.39	1.2.1.5	1.2.1.65	1.2.7.1	1.2.99.2	1.3.1.-	1.3.1.2	1.3.1.25
**Weissella**	1	14	0	0	0	0	0	0	0	0	0	0	0	0	0	0	0	0	0	0
**Wenxinia**	1	2	0	0	0	0	0	0	0	0	0	0	0	0	0	0	0	0	0	0
**Wigglesworthia**	1	29	0	0	0	0	0	0	0	0	0	0	0	0	0	0	0	0	0	0
**Wolbachia**	1	32	0	0	0	0	0	0	0	0	0	0	0	0	0	0	0	0	0	0
**Wolinella**	2	51	0	0	0	0	0	1	0	0	0	0	0	0	0	1	0	0	0	0
**Xanthobacter**	3	176	0	1	0	0	0	1	0	1	0	1	0	0	0	0	1	0	0	1
**Xanthobacteraceae**	1	4	0	0	0	0	0	0	0	0	0	0	0	0	0	0	0	0	0	0
**Xanthomonadaceae**	1	39	0	0	0	0	0	0	0	0	0	0	0	0	0	0	0	0	0	0
**Xanthomonas**	2	162	0	1	0	0	0	0	0	0	1	1	0	0	0	0	0	1	0	0
**Xenorhabdus**	2	127	0	0	0	0	0	0	0	1	0	1	1	0	0	0	0	0	0	0
**Xylanimonas**	2	66	0	0	0	0	0	0	0	1	0	1	0	0	0	0	0	0	0	0
**Xylella**	2	52	0	0	0	0	0	0	0	0	0	0	0	0	0	0	0	0	0	0
**Yersinia**	2	145	0	0	0	0	0	0	0	1	0	1	0	0	0	0	0	0	0	0
**Yokenella**	2	61	0	0	0	0	0	0	0	0	0	1	0	0	0	0	0	0	0	0
**Zobellia**	1	28	0	0	0	0	0	0	0	0	0	0	0	0	0	0	0	0	0	0
**Zunongwangia**	2	98	0	1	0	0	0	0	0	0	0	1	0	0	0	0	0	0	0	0
**Zymomonas**	2	67	0	1	0	0	0	1	0	0	0	0	0	0	0	0	0	0	0	0
**Zymophilus**	1	2	0	0	0	0	0	0	0	0	0	0	0	0	0	0	0	0	0	0

Genus	Cluster Number	Protein Counts	1.3.1.32	1.3.1.56	1.3.99.-	1.4.3.4	1.6.5.-	1.7.1.-	1.8.99.3	1.97.1.8	2.1.1.-	2.1.1.67	2.3.1.-	2.3.1.16	2.3.1.5	2.3.1.9	2.4.2.10	2.4.2.3	2.4.2.4	2.4.2.8	2.5.1.-	2.5.1.18
Acaricomes	1	2	0	0	0	0	0	0	0	0	0	0	0	0	0	0	0	0	0	1	0	0
Acaryochloris	2	88	0	0	0	0	0	0	0	0	1	0	1	0	0	1	1	1	0	0	1	1
Acetivibrio	1	2	0	0	0	0	0	0	0	0	0	0	0	0	0	0	0	0	0	0	0	0
Acetobacter	2	85	0	0	0	0	0	0	0	0	1	1	1	0	0	0	1	0	0	0	1	1
Acetobacteraceae	1	2	0	0	0	0	0	0	0	0	0	0	0	0	0	0	0	0	0	0	0	0
Acetobacterium	1	8	0	0	0	0	0	0	0	0	0	0	0	0	0	0	0	0	0	0	0	0
Acetohalobium	2	79	0	0	0	0	1	0	0	0	1	0	1	1	0	0	1	0	0	1	1	0
Acholeplasma	2	38	0	0	0	0	1	0	0	0	1	0	1	1	0	0	0	0	0	1	0	1
Achromobacter	2	192	0	0	1	0	0	0	0	0	1	0	1	1	0	1	1	0	1	0	1	1
Acidaminococcus	1	65	0	0	0	0	0	0	0	0	1	0	1	1	0	1	1	1	0	1	1	0
Acidianus	1	9	0	0	0	0	0	0	0	0	0	0	0	0	0	0	0	0	0	0	0	0
Acidilobus	1	8	0	0	0	0	0	0	0	0	0	0	0	0	0	0	0	0	0	0	0	0
Acidimicrobium	2	58	0	0	0	0	0	0	0	0	1	0	1	0	0	1	1	0	0	1	1	0
Acidiphilium	3	144	0	0	0	0	0	1	0	0	1	0	1	1	0	1	1	0	0	0	1	1
Acidithiobacillus	2	70	0	0	0	0	0	1	0	0	1	0	1	0	0	0	1	0	0	1	1	1
Acidobacterium	2	91	0	0	0	0	1	0	0	0	1	0	1	1	0	1	1	0	0	1	1	1
Acidocella	1	2	0	0	0	0	0	0	0	0	0	0	0	0	0	0	0	0	0	0	0	0
Acidothermus	2	68	0	0	0	0	0	0	0	0	1	0	1	1	0	1	1	0	0	1	1	0
Acidovorax	3	217	0	0	0	0	0	1	0	0	1	0	1	1	0	1	1	0	1	0	1	1
Aciduliprofundum	1	11	0	0	0	0	0	0	0	0	0	0	0	0	0	0	0	0	0	0	0	0
Acinetobacter	3	188	0	0	0	0	0	1	0	0	1	1	1	1	0	1	1	0	0	1	1	1
Actinobacillus	2	112	0	0	0	0	1	1	0	0	1	0	1	1	0	1	1	1	0	1	1	1
Actinomadura	1	1	0	0	0	0	0	0	0	0	0	0	0	0	0	0	0	0	0	0	0	0
Actinoplanes	1	52	0	0	0	0	0	0	0	0	0	0	0	0	0	0	0	0	0	0	0	0
Actinopolyspora	1	2	0	0	0	0	0	0	0	0	1	0	0	0	0	0	0	0	0	0	0	0
Actinosynnema	2	122	0	0	0	0	0	0	0	0	1	1	1	1	1	1	1	1	1	1	1	1
Advenella	1	30	0	0	0	0	0	0	0	0	0	0	0	0	0	0	0	0	0	0	0	0
Aequorivita	1	12	0	0	0	0	0	0	0	0	0	0	0	0	0	0	0	0	0	0	0	0
Aerococcus	2	57	0	0	0	0	0	0	0	0	1	0	1	1	0	0	1	0	0	1	0	1
Aeromonas	2	127	0	0	0	0	1	1	0	0	1	1	1	1	0	1	1	1	1	1	1	1
Aeropyrum	1	13	0	0	0	0	0	0	0	0	0	0	0	0	0	0	0	0	0	0	0	0
Afipia	1	54	0	0	0	0	0	1	0	0	1	0	1	1	0	1	0	0	0	0	1	1
Aggregatibacter	1	81	0	0	0	0	1	0	0	0	1	0	1	0	0	0	1	1	0	1	1	0
Agrobacterium	2	242	0	0	0	0	0	0	0	0	1	0	1	1	0	1	1	1	1	1	1	1
Ahrensia	1	2	0	0	0	0	0	0	0	0	0	0	0	0	0	0	0	0	0	0	0	0
Akkermansia	2	47	0	0	0	0	0	1	0	0	1	0	1	0	0	0	1	0	0	1	1	0
Albidiferax	3	144	0	0	0	0	0	1	0	0	1	0	1	1	0	1	1	0	1	0	1	1
Alcaligenes	1	21	0	0	0	0	0	0	0	0	0	0	0	1	0	0	0	0	0	0	0	0
Alcanivorax	2	146	0	0	0	0	1	1	0	0	1	0	1	1	0	1	1	0	1	1	1	1
Alicycliphilus	3	152	0	0	1	0	0	0	0	0	1	0	1	1	0	1	1	0	1	0	1	1
Alicyclobacillus	2	91	0	0	0	0	0	1	0	0	1	0	1	1	0	1	1	0	0	1	1	0
Aliivibrio	2	118	0	0	0	0	1	1	0	0	1	1	1	1	0	1	1	1	1	1	1	1
Alishewanella	1	1	0	0	0	0	0	0	0	0	0	0	0	0	0	0	0	0	0	0	0	1
Alistipes	1	52	0	0	0	0	1	0	0	0	1	0	1	0	0	0	1	0	0	1	1	0
Alkalilimnicola	2	97	0	0	0	0	0	1	0	0	1	0	1	1	0	1	1	0	1	1	1	1
Alkaliphilus	2	62	0	0	0	0	0	0	0	0	1	0	1	0	0	1	1	0	0	1	0	0
Allochromatium	2	84	0	0	0	0	0	0	1	0	1	1	1	1	0	0	1	0	1	1	1	0
Alphaproteobacteria	1	3	0	0	0	0	0	0	0	0	0	0	0	0	0	0	0	0	1	0	0	0
Alteromonadales	1	12	0	0	0	0	1	0	0	0	0	0	0	1	0	0	0	0	0	1	0	1
Alteromonas	1	37	0	0	0	0	1	0	0	0	0	0	0	0	0	0	0	0	0	0	0	0
Aminobacter	1	4	0	0	0	0	0	0	0	0	0	0	0	0	0	0	0	0	0	0	0	0
Aminobacterium	2	59	0	0	0	0	0	0	0	0	1	0	1	0	0	1	1	0	0	1	1	0

Genus	Cluster Number	Protein Counts	1.3.1.32	1.3.1.56	1.3.99.-	1.4.3.4	1.6.5.-	1.7.1.-	1.8.99.3	1.97.1.8	2.1.1.-	2.1.1.67	2.3.1.-	2.3.1.16	2.3.1.5	2.3.1.9	2.4.2.10	2.4.2.3	2.4.2.4	2.4.2.8	2.5.1.-	2.5.1.18
**Ammonifex**	2	50	0	0	0	0	0	0	1	0	1	0	1	0	0	0	1	0	0	1	1	0
**Amphibacillus**	1	13	0	0	0	0	0	0	0	0	1	0	0	0	0	0	0	0	0	0	0	0
**Amphritea**	1	2	0	0	0	0	0	0	0	0	0	0	0	0	0	0	0	0	0	0	0	0
**Amycolatopsis**	2	201	0	0	1	1	0	0	0	0	1	0	1	1	0	1	1	0	1	1	1	1
**Amycolicicoccus**	1	43	0	0	0	0	0	0	0	0	0	0	0	0	0	0	0	0	0	0	0	0
**Anabaena**	2	92	0	0	0	0	0	1	0	0	1	0	1	0	0	0	1	0	0	0	1	1
**Anaerobaculum**	1	11	0	0	0	0	0	0	0	0	0	0	0	0	0	0	0	0	0	0	0	0
**Anaerococcus**	2	46	0	0	0	0	1	0	0	0	1	0	1	1	0	0	1	1	0	1	0	0
**Anaerolinea**	2	66	0	0	0	0	0	0	0	0	1	0	1	1	0	1	0	1	0	1	1	0
**Anaeromyxobacter**	3	117	0	0	0	1	0	1	0	0	1	0	1	1	0	1	1	0	0	1	1	1
**Anaerostipes**	1	44	0	0	0	0	0	0	0	0	1	0	1	1	0	0	1	0	0	0	0	0
**Anaerotruncus**	1	2	0	0	0	0	0	0	0	0	1	0	0	0	0	0	0	0	0	0	0	0
**Anaplasma**	1	35	0	0	0	0	0	0	0	0	1	0	0	0	0	0	1	0	0	0	1	0
**Ancylobacter**	1	3	0	0	0	0	0	0	0	0	0	0	0	0	0	0	0	0	0	0	0	0
**Aneurinibacillus**	1	1	0	0	0	0	0	0	0	0	0	0	0	0	0	0	0	0	0	0	0	0
**Angomonas**	1	1	0	0	0	0	0	0	0	0	0	0	0	0	0	0	0	0	0	0	0	0
**Anoxybacillus**	2	72	0	0	0	0	0	1	0	0	1	0	1	1	0	0	1	0	0	1	1	0
**Aquifex**	2	45	0	0	0	0	0	1	0	0	1	0	1	0	0	0	1	0	0	1	1	0
**Aquimarina**	1	2	0	0	0	0	0	0	0	0	0	0	0	0	0	1	0	0	0	0	0	0
**Arcanobacterium**	2	45	0	0	1	0	0	0	0	0	1	0	1	0	0	0	1	0	1	0	0	0
**Archaeoglobus**	1	14	0	0	0	0	0	0	0	0	0	0	0	0	0	0	0	0	0	0	0	0
**Arcobacter**	2	101	0	0	0	0	0	1	0	0	1	0	1	0	0	1	1	0	0	0	0	0
**Aromatoleum**	3	133	0	0	0	0	1	1	0	0	1	0	1	1	0	1	1	0	0	1	1	1
**Arsenophonus**	1	4	0	0	0	0	1	0	0	0	0	0	0	0	0	0	0	0	0	0	0	0
**Arthrobacter**	2	206	0	0	0	0	0	0	0	0	1	0	1	1	0	1	1	0	1	1	1	1
**Arthrospira**	2	67	0	0	0	0	0	0	0	0	1	0	1	0	0	1	1	0	0	0	1	1
**Aster**	1	10	0	0	0	0	0	0	0	0	1	0	0	0	0	0	0	0	0	0	0	0
**Asticcacaulis**	2	103	0	0	0	0	0	0	0	0	1	0	1	1	0	1	1	0	0	1	1	1
**Atopobium**	1	28	0	0	0	0	0	0	0	0	1	0	1	0	0	0	1	0	0	1	0	0
**Aurantimonas**	1	2	0	0	0	0	0	0	0	0	0	0	0	0	0	0	0	0	0	0	0	0
**Avibacterium**	1	17	0	0	0	0	1	0	0	0	1	0	0	0	0	0	0	0	0	0	0	0
**Azoarcus**	3	172	0	0	0	0	0	1	0	0	1	0	1	1	1	1	1	0	0	1	1	1
**Azorhizobium**	3	149	0	0	0	0	0	1	0	0	1	0	1	1	0	1	1	0	1	1	1	1
**Azorhizophilus**	1	4	0	0	0	0	0	0	0	0	0	0	0	0	0	0	0	0	0	0	0	0
**Azospira**	1	19	0	0	0	0	0	0	0	0	0	0	0	0	0	0	0	0	0	0	0	0
**Azospirillum**	2	198	0	0	1	1	0	0	0	0	1	0	1	1	0	1	1	0	1	0	1	1
**Azotobacter**	3	183	0	0	0	1	1	0	0	0	1	1	1	1	0	1	1	1	0	1	1	1
**Bacillales**	1	9	0	0	0	0	0	0	0	0	0	0	0	0	0	0	0	0	0	1	0	0
**Bacilli**	1	3	0	0	0	0	0	0	0	0	1	0	1	0	0	0	0	0	0	0	0	0
**Bacillus**	2	237	0	0	0	0	0	1	0	0	1	0	1	1	0	1	1	1	0	1	1	1
**Bacteria**	2	86	0	0	1	0	0	0	0	0	1	0	1	1	0	1	0	0	0	0	1	1
**Bacteriovorax**	2	98	0	0	0	0	1	1	0	0	1	1	1	1	0	1	1	0	0	1	1	1
**Bacteroidales**	2	59	0	0	0	0	1	0	0	0	1	0	1	0	0	0	1	0	0	0	1	0
**Bacteroides**	2	96	0	0	0	0	1	1	0	0	1	0	1	0	0	0	1	0	0	1	1	0
**Bacteroidetes**	1	2	0	0	0	0	0	1	0	0	0	0	0	0	0	0	0	0	0	0	0	0
**Bartonella**	2	50	0	0	0	0	0	0	0	0	1	0	1	0	0	0	1	0	0	0	1	0
**Baumannia**	2	24	0	0	0	0	0	1	0	0	1	0	1	0	0	0	1	0	0	0	1	0
**Bdellovibrio**	2	87	0	0	0	0	0	1	0	0	1	0	1	1	0	1	1	0	0	1	1	1
**Beijerinckia**	3	123	0	0	0	0	0	0	0	0	1	0	1	1	0	1	1	0	0	0	1	1
**Belliella**	1	14	0	0	0	0	0	0	0	0	0	0	0	0	0	0	0	0	0	0	0	0
**Beutenbergia**	2	82	0	0	0	0	0	0	0	0	1	0	1	1	0	1	1	0	1	1	1	1
**Bifidobacterium**	2	61	0	0	0	0	0	0	0	0	1	0	1	0	0	0	1	0	0	1	1	0

Genus	Cluster Number	Protein Counts	1.3.1.32	1.3.1.56	1.3.99.-	1.4.3.4	1.6.5.-	1.7.1.-	1.8.99.3	1.97.1.8	2.1.1.-	2.1.1.67	2.3.1.-	2.3.1.16	2.3.1.5	2.3.1.9	2.4.2.10	2.4.2.3	2.4.2.4	2.4.2.8	2.5.1.-	2.5.1.18
Bizionia	1	1	0	0	0	0	0	0	0	0	0	0	0	0	0	0	0	0	0	0	0	0
Blastococcus	1	39	0	0	0	0	0	0	0	0	0	0	0	0	0	0	0	0	0	0	0	0
Blattabacterium	1	23	0	0	0	0	0	0	0	0	1	0	1	0	0	0	1	0	0	0	1	0
Blautia	1	22	0	0	0	0	0	0	0	0	1	0	1	0	0	0	1	0	0	1	0	0
Bordetella	3	227	0	0	1	0	0	1	0	0	1	1	1	1	0	1	1	0	0	0	1	1
Borrelia	1	32	0	0	0	0	0	0	0	0	1	0	1	0	0	1	0	0	0	1	0	0
Brachybacterium	1	18	0	0	0	0	0	0	0	0	0	0	0	0	0	0	0	0	0	0	0	0
Brachymonas	1	4	0	0	0	0	0	0	0	0	0	0	0	0	0	0	0	0	1	0	0	0
Brachyspira	2	79	0	0	0	0	1	0	0	0	1	0	1	0	0	1	1	1	0	1	1	1
Bradyrhizobiaceae	1	20	0	0	0	0	0	0	0	0	0	0	0	0	0	0	0	0	0	0	0	1
Bradyrhizobium	3	254	0	0	0	0	0	1	0	0	1	0	1	1	1	1	1	0	1	0	1	1
Brenneria	1	20	0	0	0	0	0	0	0	0	0	0	0	0	0	0	0	0	0	0	1	0
Brevibacillus	2	122	0	0	0	0	0	0	0	0	1	0	1	1	0	1	1	1	0	1	1	1
Brevibacterium	1	4	0	0	0	0	0	0	0	0	0	0	0	0	0	0	0	0	0	0	0	0
Brevundimonas	2	105	0	0	0	0	0	0	0	0	1	0	1	1	0	1	1	0	0	1	1	1
Brucella	3	138	0	0	0	0	0	1	0	0	1	0	1	1	0	1	1	0	0	1	1	1
Buchnera	1	26	0	0	0	0	0	1	0	0	1	0	0	0	0	0	1	0	0	1	1	0
Burkholderia	3	424	0	1	1	1	0	1	0	0	1	0	1	1	1	1	1	0	1	1	1	1
Burkholderiaceae	3	143	0	0	0	0	0	1	0	0	1	0	1	1	0	1	0	0	0	0	1	1
Burkholderiales	1	24	0	0	0	0	0	0	0	0	0	0	0	0	0	1	0	0	0	0	1	0
Buttiauxella	1	2	0	0	0	0	0	0	0	0	0	0	0	0	0	0	0	0	0	0	0	0
Butyrivibrio	2	67	0	0	0	0	0	0	0	0	1	0	1	0	0	1	1	1	0	1	1	0
Caldanaerobacter	2	63	0	0	0	0	0	1	0	0	1	0	1	0	0	1	1	0	1	1	1	0
Caldicellulosiruptor	2	75	0	0	0	0	0	1	0	0	1	0	1	0	0	0	1	0	0	1	1	1
Caldilinea	1	23	0	0	0	0	0	0	0	0	0	0	0	0	0	0	0	0	0	0	0	0
Caldimonas	1	6	0	0	0	0	0	0	0	0	0	0	0	0	0	0	0	0	0	0	0	0
Caldisericum	1	10	0	0	0	0	0	0	0	0	0	0	0	0	0	0	0	0	0	0	0	0
Caldisphaera	1	7	0	0	0	0	0	0	0	0	0	0	0	0	0	0	0	0	0	0	0	0
Calditerrivibrio	2	74	0	0	0	0	1	0	0	0	1	0	1	1	0	1	1	0	0	1	1	0
Caldivirga	1	13	0	0	0	0	0	0	0	0	0	0	0	0	0	0	0	0	0	0	0	0
Calothrix	1	43	0	0	0	0	0	1	0	0	1	0	0	0	0	0	0	0	0	0	0	1
Calyptogena	2	34	0	0	0	0	0	0	1	0	1	0	1	0	0	0	1	0	0	0	1	0
Campylobacter	2	80	0	0	0	0	0	1	0	0	1	0	1	0	0	0	1	0	0	0	1	0
Candidatus	2	290	0	0	0	1	1	1	1	0	1	1	1	1	0	1	1	1	1	1	1	1
Capnocytophaga	1	50	0	0	0	0	1	0	0	0	1	0	1	0	0	0	1	0	0	0	1	0
Carbophilus	1	2	0	0	0	0	0	0	0	0	0	0	0	0	0	0	0	0	0	0	0	0
Carboxydibrachium	1	14	0	0	0	0	0	0	0	0	0	0	0	0	0	0	1	0	0	0	0	0
Carboxydothermus	2	68	0	0	0	0	0	0	0	0	1	0	1	1	0	1	0	0	0	1	0	0
Carica	1	2	0	0	0	0	0	0	0	0	0	0	0	0	0	0	0	0	0	0	0	0
Carnobacterium	2	71	0	0	0	0	0	0	0	0	1	0	1	0	0	1	1	0	0	1	0	1
Catenibacterium	1	2	0	0	0	0	0	0	0	0	0	0	0	0	0	0	0	0	0	0	0	0
Catenovulum	1	2	0	0	0	0	0	0	0	0	0	0	0	0	0	0	0	0	0	0	0	0
Catenulispora	3	159	0	0	0	0	0	0	0	0	1	0	1	1	0	1	1	0	1	1	1	1
Caulobacter	3	168	0	0	0	0	0	1	0	0	1	0	1	1	1	1	1	0	1	1	1	1
Cedecea	1	45	0	0	1	0	0	0	0	0	1	0	0	1	0	1	1	1	1	1	1	0
Cellulomonas	2	96	0	0	0	0	0	0	0	0	1	0	1	1	0	1	1	0	1	1	1	1
Cellulophaga	2	105	0	0	0	0	1	0	0	0	1	0	1	1	0	1	1	0	0	0	1	1
Cellulosilyticum	1	14	0	0	0	0	0	0	0	0	0	0	0	0	0	0	0	0	0	0	0	0
Cellvibrio	2	85	0	0	0	0	1	1	0	0	1	0	1	0	0	1	1	0	1	1	1	1
Cenarchaeum	1	7	0	0	0	0	0	0	0	0	0	0	0	0	0	0	0	0	0	0	0	0
Chamaesiphon	1	13	0	0	0	0	0	0	0	0	0	0	0	0	0	0	0	0	0	0	0	0
Chelativorans	3	136	0	0	0	0	0	1	0	0	1	0	1	1	0	1	1	0	1	1	1	1

Genus	Cluster Number	Protein Counts	1.3.1.32	1.3.1.56	1.3.99.-	1.4.3.4	1.6.5.-	1.7.1.-	1.8.99.3	1.97.1.8	2.1.1.-	2.1.1.67	2.3.1.-	2.3.1.16	2.3.1.5	2.3.1.9	2.4.2.10	2.4.2.3	2.4.2.4	2.4.2.8	2.5.1.-	2.5.1.18
Chelatococcus	1	2	0	0	0	0	0	0	0	0	0	0	0	0	0	0	0	0	0	0	0	0
Chitinophaga	2	98	0	0	0	0	0	0	0	0	1	0	1	1	0	1	1	0	0	1	1	1
Chlamydia	1	36	0	0	0	0	1	0	0	0	1	0	1	0	0	0	1	0	0	0	1	0
Chlamydomonas	1	3	0	0	0	0	0	0	0	0	0	0	0	0	0	0	0	0	0	0	0	0
Chlamydophila	1	31	0	0	0	0	1	0	0	0	1	0	1	0	0	0	1	0	0	0	1	0
Chlorobaculum	2	61	0	0	0	0	0	1	0	0	1	0	1	0	0	0	1	0	0	1	1	0
Chlorobium	2	89	0	0	0	0	1	1	0	0	1	0	1	0	0	0	1	0	0	1	1	0
Chlorobium/Pelodictyon	1	2	0	0	0	0	0	0	0	0	0	0	0	0	0	0	0	0	0	0	0	0
Chloroflexus	2	105	0	0	0	0	0	0	0	0	1	0	1	1	1	1	1	0	0	1	1	0
Chlorogloeopsis	1	9	0	0	0	0	0	0	0	0	0	0	0	0	0	0	0	0	0	0	0	0
Chloroherpeton	2	59	0	0	0	0	0	1	0	0	1	0	1	0	0	0	1	0	0	1	0	0
Chromobacterium	2	117	0	0	0	0	0	1	0	0	1	0	1	1	0	1	1	0	1	1	1	1
Chromohalobacter	2	145	0	0	0	0	1	1	0	0	1	1	1	0	0	1	1	0	1	1	1	1
Chroococcidiopsis	1	24	0	0	0	0	0	0	0	0	0	0	0	0	0	0	0	0	0	0	0	0
Chryseobacterium	2	104	0	0	0	0	0	0	0	0	1	0	1	1	0	1	1	0	0	0	0	1
Citreicella	1	13	0	0	0	0	1	0	0	0	0	0	0	0	0	1	0	0	0	0	0	0
Citrobacter	2	190	0	0	1	0	1	1	0	0	1	0	1	1	0	1	1	1	1	1	1	1
Clavibacter	2	83	0	0	0	0	0	0	0	0	1	0	1	1	0	0	1	0	1	1	1	1
Clonorchis	1	1	0	0	0	0	0	0	0	0	0	0	0	0	0	0	0	0	0	0	0	0
Clostridiales	2	77	0	0	0	0	0	0	0	0	1	0	1	1	0	0	1	0	0	1	1	0
Clostridium	2	191	0	0	1	0	0	1	0	0	1	0	1	1	0	1	1	1	1	1	1	1
Cohnella	1	2	0	0	0	0	0	0	0	0	0	0	0	0	0	0	0	0	0	0	0	0
Coleofasciculus	1	4	0	0	0	0	0	0	0	0	0	0	0	0	0	0	0	0	0	0	0	0
Collimonas	1	35	0	0	0	0	0	0	0	0	1	0	0	0	0	0	0	0	0	0	0	0
Colwellia	2	130	0	0	0	0	1	1	0	0	1	1	1	1	0	1	1	0	1	1	1	1
Comamonas	2	196	0	0	0	0	0	0	0	0	1	0	1	1	0	1	1	0	1	0	1	1
Conexibacter	3	117	0	0	0	1	0	0	0	0	1	0	1	1	0	1	1	0	0	1	1	1
Coprobacillus	1	2	0	0	0	0	0	0	0	0	1	0	0	0	0	0	0	0	0	0	0	0
Coprococcus	2	76	0	0	0	0	1	0	0	0	1	0	1	1	0	1	1	1	0	1	1	0
Coprothermobacter	2	45	0	0	0	0	0	0	0	0	1	0	1	1	0	0	1	0	0	1	0	0
Coraliomargarita	2	68	0	0	0	0	0	0	0	0	1	0	1	0	0	0	1	0	1	1	1	0
Corallococcus	1	29	0	0	0	0	0	0	0	0	0	0	0	0	0	0	0	0	0	0	0	1
Coriobacterium	2	36	0	0	0	0	0	0	0	0	1	0	1	0	0	0	1	0	0	1	1	0
Corynebacterineae	1	5	0	0	0	0	0	0	0	0	0	0	0	1	0	1	0	0	0	0	0	0
Corynebacterium	2	175	0	0	0	0	0	0	0	0	1	0	1	1	0	1	1	0	1	1	1	0
Coxiella	2	54	0	0	0	0	0	1	0	0	1	0	1	1	0	1	1	1	0	1	1	0
Crinalium	1	21	0	0	0	0	0	0	0	0	0	0	0	0	0	0	0	0	0	0	0	0
Croceibacter	2	80	0	0	0	0	1	0	0	0	1	0	1	0	0	1	1	0	0	0	1	0
Crocosphaera	1	6	0	0	0	0	0	0	0	0	0	0	0	0	0	0	0	0	0	0	0	0
Cronobacter	2	120	0	0	0	0	0	0	0	0	1	0	1	1	0	1	1	1	1	1	1	1
Cryptobacterium	2	39	0	0	0	0	0	1	0	0	1	0	1	0	0	0	1	0	0	1	0	0
Cucumis	1	9	0	0	0	0	1	0	0	0	1	0	0	0	0	0	0	0	0	1	0	0
Cupriavidus	3	317	0	1	1	1	0	1	0	0	1	0	1	1	1	1	1	0	1	1	1	1
Curtobacterium	1	5	0	0	0	0	0	0	0	0	0	0	0	0	0	0	0	0	0	0	0	0
Curvibacter	1	27	0	0	0	0	0	0	0	0	0	0	0	1	0	0	0	0	0	0	0	0
Cyanobacterium	1	9	0	0	0	0	0	0	0	0	0	0	0	0	0	0	0	0	0	0	0	0
Cyanobium	1	10	0	0	0	0	0	0	0	0	0	0	0	0	0	0	0	0	0	0	0	0
Cyanothece	1	34	0	0	0	0	0	0	0	0	0	0	0	0	0	0	0	0	0	0	0	0
Cyclobacterium	1	19	0	0	0	0	0	0	0	0	0	0	0	0	0	0	0	0	0	0	0	0
Cycloclasticus	1	21	0	0	0	0	0	0	0	0	0	0	0	0	0	0	0	0	0	0	0	0
Cylindrospermopsis	1	4	0	0	0	0	0	0	0	0	0	0	0	0	0	0	0	0	0	0	0	0
Cylindrospermum	1	30	0	0	0	0	0	0	0	0	1	0	0	0	0	0	0	0	0	0	0	0

Genus	Cluster Number	Protein Counts	1.3.1.32	1.3.1.56	1.3.99.-	1.4.3.4	1.6.5.-	1.7.1.-	1.8.99.3	1.97.1.8	2.1.1.-	2.1.1.67	2.3.1.-	2.3.1.16	2.3.1.5	2.3.1.9	2.4.2.10	2.4.2.3	2.4.2.4	2.4.2.8	2.5.1.-	2.5.1.18
**Cytophaga**	2	69	0	0	0	0	0	0	0	0	1	0	1	0	0	1	1	0	0	0	1	1
**Dactylococcopsis**	1	8	0	0	0	0	0	0	0	0	0	0	0	0	0	0	0	0	0	0	0	0
**Dechloromonas**	1	40	0	0	0	0	0	0	0	0	0	0	0	0	0	0	0	0	0	0	0	0
**Dechlorosoma**	1	19	0	0	0	0	0	0	0	0	0	0	0	0	0	0	0	0	0	0	0	0
**Deferribacter**	2	73	0	0	0	0	1	0	0	0	1	0	1	1	0	1	1	0	0	1	1	0
**Dehalobacter**	1	9	0	0	0	0	0	0	0	0	0	0	0	0	0	0	0	0	0	0	0	0
**Dehalococcoides**	2	41	0	0	0	0	0	0	0	1	1	0	1	0	0	0	1	0	0	1	1	0
**Dehalogenimonas**	1	37	0	0	0	0	0	0	0	0	1	0	1	0	0	0	1	0	0	1	0	0
**Deinococcus**	2	140	0	0	0	0	0	0	0	0	1	0	1	1	0	1	1	0	0	1	1	1
**Delftia**	3	177	0	0	0	0	0	0	0	0	1	0	1	1	0	1	1	0	1	0	1	1
**Denitrovibrio**	2	67	0	0	0	0	1	0	0	0	1	0	1	0	0	0	1	0	0	1	1	0
**Desulfarculus**	2	92	0	0	0	0	0	0	1	0	1	0	1	1	0	1	1	0	0	1	0	0
**Desulfatibacillum**	3	84	0	0	0	0	0	1	0	0	1	0	1	1	0	1	1	1	0	1	1	0
**Desulfitobacterium**	2	102	0	0	1	0	0	0	0	1	1	0	1	1	1	1	1	0	0	1	1	0
**Desulfobacca**	2	65	0	0	0	0	0	0	1	0	1	0	1	0	0	0	1	0	0	1	1	0
**Desulfobacterium**	2	113	0	0	0	0	1	0	1	0	1	0	1	1	0	1	1	1	0	1	1	0
**Desulfobacula**	1	21	0	0	0	0	0	0	0	0	0	0	0	0	0	0	0	0	0	0	0	0
**Desulfobulbus**	2	88	0	0	0	0	0	0	1	0	1	0	1	0	0	1	1	0	0	1	1	0
**Desulfocapsa**	1	9	0	0	0	0	0	0	0	0	0	0	0	0	0	0	0	0	0	0	0	0
**Desulfococcus**	2	80	0	0	0	0	1	1	0	0	1	0	1	1	0	1	1	1	0	1	1	0
**Desulfohalobium**	2	52	0	0	0	0	1	0	0	0	1	0	1	0	0	0	1	0	0	1	1	0
**Desulfomicrobium**	2	65	0	0	0	0	1	0	0	0	1	0	1	0	0	0	1	0	0	1	1	0
**Desulfomonile**	1	25	0	0	0	0	0	0	0	0	0	0	0	0	0	0	0	0	0	0	0	0
**Desulforhabdus**	1	2	0	0	0	0	0	0	0	0	0	0	0	0	0	0	0	0	0	0	0	0
**Desulfosporosinus**	1	30	0	0	0	0	0	0	0	0	0	0	0	0	0	0	0	0	0	0	0	0
**Desulfotalea**	2	66	0	0	0	0	1	1	1	0	1	0	1	0	0	0	1	0	0	1	0	0
**Desulfotomaculum**	2	98	0	0	1	0	0	0	0	0	1	0	1	1	0	1	1	0	0	1	0	0
**Desulfovibrio**	2	144	0	0	0	0	1	1	1	0	1	0	1	0	0	0	1	0	1	1	1	0
**Desulfurispirillum**	2	78	0	0	0	0	0	0	0	0	1	0	1	1	0	1	1	0	0	1	1	1
**Desulfurivibrio**	2	71	0	0	0	0	1	0	1	0	1	0	1	0	0	0	1	0	0	1	1	0
**Desulfurobacterium**	2	53	0	0	0	0	0	0	0	0	1	0	1	0	0	0	1	0	0	1	1	0
**Desulfurococcus**	1	11	0	0	0	0	0	0	0	0	0	0	0	0	0	0	0	0	0	0	0	0
**Diaphorobacter**	1	16	0	0	0	0	0	0	0	0	0	0	0	0	0	0	0	0	0	0	0	0
**Dichelobacter**	2	45	0	0	0	0	1	1	0	0	1	0	1	0	0	0	1	0	1	1	0	1
**Dickeya**	2	155	0	0	1	0	0	0	0	0	1	0	1	1	0	1	1	1	1	1	1	1
**Dictyoglomus**	2	62	0	0	0	0	0	0	0	0	1	0	1	0	0	0	1	0	0	1	0	0
**Dinoroseobacter**	3	148	0	0	0	0	0	0	0	0	1	0	1	1	0	1	1	0	1	1	1	1
**Dokdonia**	1	3	0	0	0	0	0	0	0	0	0	0	0	0	0	1	0	0	0	0	0	0
**Dorea**	1	7	0	0	0	0	0	0	0	0	1	0	0	0	0	0	1	0	0	0	0	0
**Dyadobacter**	2	83	0	0	0	1	0	0	0	0	1	0	1	1	0	1	1	0	0	1	1	1
**Echinicola**	1	20	0	0	0	0	0	0	0	0	0	0	0	0	0	0	0	0	0	0	0	0
**Edwardsiella**	2	126	0	0	1	0	0	0	0	0	1	0	1	1	0	0	1	1	1	1	1	1
**Eggerthella**	1	51	0	0	1	0	0	0	0	0	1	0	1	0	0	0	1	0	0	1	1	0
**Ehrlichia**	2	37	0	0	0	0	0	0	0	0	1	0	1	0	0	0	1	0	0	0	1	0
**Eikenella**	1	4	0	0	0	0	0	0	0	0	0	0	0	0	0	0	0	0	0	0	0	0
**Elusimicrobium**	2	44	0	0	0	0	0	1	0	0	1	0	1	0	0	0	1	1	0	1	0	0
**Emticicia**	1	21	0	0	0	0	0	0	0	0	0	0	0	0	0	0	0	0	0	0	0	0
**Enhydrobacter**	1	1	0	0	0	0	0	0	0	0	0	0	0	0	0	0	0	0	0	0	0	0
**Ensifer**	1	2	0	0	0	0	0	0	0	0	0	0	0	0	0	0	0	0	0	0	0	0
**Enterobacter**	2	202	0	0	0	0	1	1	0	0	1	0	1	1	0	1	1	1	1	1	1	1
**Enterobacteriaceae**	2	211	0	0	1	0	1	1	0	0	1	0	1	1	0	1	1	1	1	1	1	1
**Enterococcus**	1	187	0	0	0	0	1	1	0	0	1	1	1	1	0	1	1	1	1	1	1	1

Genus	Cluster Number	Protein Counts	1.3.1.32	1.3.1.56	1.3.99.-	1.4.3.4	1.6.5.-	1.7.1.-	1.8.99.3	1.97.1.8	2.1.1.-	2.1.1.67	2.3.1.-	2.3.1.16	2.3.1.5	2.3.1.9	2.4.2.10	2.4.2.3	2.4.2.4	2.4.2.8	2.5.1.-	2.5.1.18
Enterovibrio	1	15	0	0	0	0	1	0	0	0	0	0	0	0	0	0	0	0	0	0	0	0
Erwinia	2	151	0	0	0	0	0	0	0	0	1	0	1	1	0	1	1	1	1	1	1	1
Erysipelothrix	1	9	0	0	0	0	0	0	0	0	0	0	0	0	0	0	0	0	0	0	0	0
Erysipelotrichaceae	1	4	0	0	0	0	0	0	0	0	1	0	1	0	0	0	0	0	0	0	0	0
Erythrobacter	2	80	0	0	0	0	0	1	0	0	1	0	1	0	0	1	1	0	0	0	1	1
Escherichia	2	242	0	0	1	0	1	1	0	0	1	0	1	1	0	1	1	1	1	1	1	1
Ethanoligenens	2	69	0	0	0	0	0	0	0	0	1	0	1	0	0	0	1	1	0	1	1	0
Eubacteriaceae	1	2	0	0	0	0	0	0	0	0	1	0	0	0	0	0	0	0	0	0	0	0
Eubacterium	2	103	0	0	0	0	1	0	0	0	1	0	1	1	0	0	1	1	0	1	1	0
Eudoraea	1	4	0	0	0	0	1	0	0	0	0	0	0	0	0	0	0	0	0	0	0	0
Euryarchaeota	1	1	0	0	0	0	0	0	0	0	0	0	0	0	0	0	0	0	0	0	0	0
Exiguobacterium	2	93	0	0	0	0	0	1	0	0	1	0	1	1	0	1	1	0	0	1	1	1
Faecalibacterium	1	12	0	0	0	0	0	0	0	0	0	0	1	0	0	0	0	0	0	0	0	0
Ferrimonas	2	115	0	0	0	0	1	0	0	0	1	1	1	1	0	0	1	1	1	1	1	1
Ferroglobus	1	14	0	0	0	0	0	0	0	0	0	0	0	0	0	0	0	0	0	0	0	0
Ferroplasma	1	11	0	0	0	0	0	0	0	0	0	0	0	0	0	0	0	0	0	0	0	0
Fervidicoccus	1	7	0	0	0	0	0	0	0	0	0	0	0	0	0	0	0	0	0	0	0	0
Fervidobacterium	2	54	0	0	0	0	1	0	0	0	1	0	1	1	0	0	1	0	0	1	0	0
Fibrella	1	17	0	0	0	0	0	0	0	0	0	0	0	0	0	0	0	0	0	0	0	0
Fibrobacter	2	52	0	0	0	0	0	0	0	0	1	0	1	0	0	0	1	0	0	1	1	0
Filifactor	1	9	0	0	0	0	0	0	0	0	0	0	0	0	0	0	0	0	0	0	0	0
Finegoldia	2	55	0	0	0	0	1	0	0	0	1	0	1	0	0	0	1	1	0	1	1	1
Firmicutes	1	15	0	0	0	0	0	0	0	0	1	0	1	0	0	0	0	0	0	0	0	0
Fischerella	1	15	0	0	0	0	0	0	0	0	0	0	0	0	0	0	0	0	0	0	0	0
Flavobacteria	1	7	0	0	0	0	1	0	0	0	0	0	0	0	0	0	0	0	0	0	0	0
Flavobacteriaceae	2	69	0	0	0	0	1	0	0	0	1	0	1	1	0	1	1	0	0	1	1	1
Flavobacteriales	1	3	0	0	0	0	0	0	0	0	0	0	0	0	0	0	0	0	0	0	0	0
Flavobacterium	2	103	0	0	0	0	0	0	0	0	1	0	1	0	0	1	1	0	0	1	1	1
Flexibacter	1	17	0	0	0	0	0	0	0	0	0	0	0	0	0	0	0	0	0	0	0	0
Flexistipes	1	11	0	0	0	0	0	0	0	0	0	0	0	0	0	0	0	0	0	0	0	0
Fluoribacter	1	6	0	0	0	0	0	0	0	0	0	0	0	0	0	0	0	0	0	0	0	0
Fluviicola	2	69	0	0	0	0	0	0	0	0	1	0	1	1	0	1	1	0	0	1	1	1
Formosa	1	2	0	0	0	0	1	0	0	0	0	0	0	0	0	0	0	0	0	0	0	0
Francisella	2	88	0	0	0	0	0	0	0	0	1	1	1	1	0	1	1	1	0	1	1	0
Frankia	3	202	0	0	1	0	0	1	0	0	1	0	1	1	0	1	1	1	1	1	1	1
Frateuria	1	25	0	0	0	0	0	0	0	0	0	0	0	0	0	0	0	0	0	0	0	0
Fructobacillus	1	2	0	0	0	0	0	0	0	0	0	0	0	0	0	0	0	0	0	0	0	0
Fulvimarina	1	2	0	0	0	0	0	0	0	0	0	0	0	0	0	0	0	0	0	0	0	0
Fusobacterium	1	48	0	0	0	0	0	0	0	0	1	0	1	0	0	1	1	0	0	1	0	0
Gallibacterium	2	87	0	0	0	0	1	0	0	0	1	0	1	1	0	0	1	1	0	1	1	0
Gallionella	2	67	0	0	0	0	0	0	0	0	1	0	1	0	0	1	1	0	0	1	1	0
Gammaproteobacteria	1	9	0	0	0	0	0	0	0	0	0	0	1	0	0	0	0	0	0	0	0	1
Gardnerella	1	31	0	0	0	0	0	0	0	0	1	0	1	0	0	1	1	1	0	1	1	0
Geitlerinema	1	9	0	0	0	0	0	0	0	0	0	0	0	0	0	0	0	0	0	0	0	0
Gemmatimonas	2	93	0	0	0	0	0	0	0	0	1	0	1	0	0	1	1	0	0	1	1	1
Geobacillus	2	168	0	0	0	0	0	1	0	0	1	0	1	1	0	1	1	0	0	1	1	0
Geobacter	2	150	0	0	0	0	1	0	0	1	1	0	1	1	0	1	1	1	0	1	1	1
Geodermatophilus	2	136	0	0	0	0	0	0	0	0	1	0	1	1	0	1	1	0	1	1	1	1
Geopsychrobacter	1	2	0	0	0	0	0	0	0	0	0	0	0	0	0	0	0	0	0	0	0	0
Gillisia	1	11	0	0	0	0	1	0	0	0	0	0	0	0	0	0	0	0	0	0	0	0
Gilvimarinus	1	4	0	0	0	0	0	0	0	0	0	0	0	0	0	0	0	0	0	0	0	0
Glaciecola	2	117	0	0	0	0	1	1	0	0	1	0	1	1	0	0	1	0	0	1	1	1

Genus	Cluster Number	Protein Counts	1.3.1.32	1.3.1.56	1.3.99.-	1.4.3.4	1.6.5.-	1.7.1.-	1.8.99.3	1.97.1.8	2.1.1.-	2.1.1.67	2.3.1.-	2.3.1.16	2.3.1.5	2.3.1.9	2.4.2.10	2.4.2.3	2.4.2.4	2.4.2.8	2.5.1.-	2.5.1.18
Gloeobacter	2	67	0	0	0	0	0	1	0	0	1	0	1	0	0	0	1	0	0	1	1	1
Gloeocapsa	1	20	0	0	0	0	0	0	0	0	0	0	0	0	0	0	0	0	0	0	0	0
Gloeothece	1	1	0	0	0	0	0	0	0	0	0	0	0	0	0	0	0	0	0	0	0	0
Gluconacetobacter	2	96	0	0	0	0	0	0	0	0	1	0	1	1	0	1	1	1	0	0	1	1
Gluconobacter	2	76	0	0	0	0	0	1	0	0	1	0	1	0	0	0	1	0	0	0	1	1
Gordonia	1	158	0	0	1	1	0	0	0	0	1	0	1	1	0	1	1	0	1	1	1	1
Gordonibacter	1	32	0	0	0	0	0	0	1	0	1	0	0	0	0	0	1	0	0	0	1	0
Gramella	2	104	0	0	0	0	1	0	0	0	1	0	1	1	0	1	1	0	0	0	1	1
Granulibacter	2	90	0	0	0	0	0	1	0	0	1	0	1	1	0	0	1	0	0	0	1	1
Granulicella	2	98	0	0	0	0	0	0	0	0	1	0	1	1	0	0	1	0	0	1	1	1
Grimontia	1	9	0	0	0	0	1	0	0	0	0	0	0	0	0	0	0	0	0	0	0	0
Haemophilus	2	117	0	0	0	0	1	1	0	0	1	0	1	0	0	1	1	1	0	1	1	1
Hafnia	1	29	0	0	1	0	1	0	0	0	1	0	0	0	0	0	1	1	0	1	0	0
Hahella	3	130	0	0	0	0	1	0	0	0	1	1	1	1	0	1	1	0	1	1	1	1
Halalkalicoccus	1	18	0	0	0	0	0	0	0	0	0	0	0	0	0	0	0	0	0	0	0	0
Halanaerobium	2	89	0	0	0	0	0	0	0	0	1	0	1	1	0	1	1	0	0	1	1	1
Haliangium	2	108	0	0	0	0	0	0	0	0	1	1	1	1	0	1	1	0	0	1	1	1
Haliscomenobacter	2	107	0	0	0	0	1	0	0	0	1	0	1	0	0	1	1	0	0	1	1	1
Haloarcula	1	22	0	0	0	0	0	0	0	0	0	0	0	0	0	0	0	0	0	0	0	0
Halobacillus	1	19	0	0	0	0	0	0	0	0	0	0	0	0	0	0	0	0	0	0	0	0
Halobacteriaceae	1	1	0	0	0	0	0	0	0	0	0	0	0	0	0	0	0	0	0	0	0	0
Halobacterium	1	10	0	0	0	0	0	0	0	0	0	0	0	0	0	0	0	0	0	0	0	0
Halobacteroides	1	14	0	0	0	0	0	0	0	0	0	0	0	0	0	0	0	0	0	0	0	0
Haloferax	1	20	0	0	0	0	0	0	0	0	0	0	0	0	0	0	0	0	0	0	0	0
Halogeometricum	1	12	0	0	0	0	0	0	0	0	0	0	0	0	0	0	0	0	0	0	0	0
Halomicrobium	1	10	0	0	0	0	0	0	0	0	0	0	0	0	0	0	0	0	0	0	0	0
Halomonas	1	149	0	0	0	0	1	0	0	0	1	1	1	1	0	1	1	0	1	1	1	1
Halopiger	1	16	0	0	0	0	0	0	0	0	0	0	0	0	0	0	0	0	0	0	0	0
Haloquadratum	1	19	0	0	0	0	0	0	0	0	0	0	0	0	0	0	0	0	0	0	0	0
Halorhabdus	1	10	0	0	0	0	0	0	0	0	0	0	0	0	0	0	0	0	0	0	0	0
Halorhodospira	2	71	0	0	0	0	0	1	0	0	1	0	1	0	0	1	1	0	1	1	0	1
Halorubrum	1	16	0	0	0	0	0	0	0	0	0	0	0	0	0	0	0	0	0	0	0	0
Haloterrigena	1	19	0	0	0	0	0	0	0	0	0	0	0	0	0	0	0	0	0	0	0	0
Halothece	1	9	0	0	0	0	0	0	0	0	0	0	0	0	0	0	0	0	0	0	0	0
Halothermothrix	2	50	0	0	0	0	0	0	0	0	1	0	1	0	0	0	1	0	0	1	0	0
Halothiobacillus	2	65	0	0	0	0	0	0	0	0	1	0	1	0	0	0	1	0	0	1	1	0
Halovivax	1	12	0	0	0	0	0	0	0	0	0	0	0	0	0	0	0	0	0	0	0	0
Helicobacter	2	79	0	0	0	0	0	1	0	0	1	0	1	1	0	1	1	0	0	0	1	0
Heliobacillus	1	4	0	0	0	0	0	0	0	0	0	0	0	0	0	0	0	0	0	0	0	0
Heliobacterium	2	51	0	0	0	0	0	0	0	0	1	0	1	1	0	0	0	0	0	1	1	0
Herbaspirillum	2	172	0	0	0	0	0	0	0	0	1	1	1	1	0	1	1	0	0	0	1	1
Herminiimonas	3	89	0	0	0	0	0	1	0	0	1	0	1	0	0	1	1	0	1	0	1	1
Herpetosiphon	3	90	0	0	1	0	0	0	0	0	1	1	1	1	0	1	1	0	0	1	1	1
Hippea	2	44	0	0	0	0	0	0	0	0	1	0	1	0	0	1	1	0	0	0	1	0
Hirschia	2	102	0	0	0	0	0	0	0	0	1	0	1	1	0	1	1	0	0	1	1	1
Histophilus	2	72	0	0	0	0	1	1	0	0	1	0	1	0	0	1	0	1	0	1	0	0
Hoeflea	1	16	0	0	0	0	0	0	0	0	1	0	0	0	0	1	0	0	0	1	0	0
Hydrogenivirga	1	2	0	0	0	0	0	1	0	0	0	0	0	0	0	0	0	0	0	0	0	0
Hydrogenobacter	2	55	0	0	0	0	0	0	0	0	1	0	1	0	0	0	1	0	0	1	1	0
Hydrogenobaculum	2	44	0	0	0	0	0	1	0	0	1	0	1	0	0	0	1	0	0	1	1	0
Hydrogenophaga	1	9	0	0	0	0	0	0	0	0	0	0	1	0	0	0	0	0	1	0	0	0
Hylemonella	1	4	0	0	0	0	0	0	0	0	0	0	0	0	0	0	0	0	0	0	0	0

Genus	Cluster Number	Protein Counts	1.3.1.32	1.3.1.56	1.3.99.-	1.4.3.4	1.6.5.-	1.7.1.-	1.8.99.3	1.97.1.8	2.1.1.-	2.1.1.67	2.3.1.-	2.3.1.16	2.3.1.5	2.3.1.9	2.4.2.10	2.4.2.3	2.4.2.4	2.4.2.8	2.5.1.-	2.5.1.18
Hyperthermus	1	6	0	0	0	0	0	0	0	0	0	0	0	0	0	0	0	0	0	0	0	0
Hyphomicrobium	2	93	0	0	0	0	0	0	0	0	1	1	1	0	0	1	1	0	0	1	1	1
Hyphomonas	3	105	0	0	0	0	0	1	0	0	1	0	1	1	0	1	1	0	0	1	1	1
Ideonella	1	18	0	0	0	0	0	0	0	0	0	0	0	0	0	0	0	0	0	0	0	0
Idiomarina	2	86	0	0	0	0	1	1	0	0	1	0	1	1	0	0	1	0	1	0	1	1
Ignavibacterium	1	14	0	0	0	0	0	0	0	0	0	0	0	0	0	0	0	0	0	0	0	0
Ignicoccus	1	5	0	0	0	0	0	0	0	0	0	0	0	0	0	0	0	0	0	0	0	0
Ignisphaera	1	8	0	0	0	0	0	0	0	0	0	0	0	0	0	0	0	0	0	0	0	0
Ilyobacter	2	85	0	0	0	0	1	0	0	0	1	0	1	1	0	0	1	0	0	1	0	0
Intrasporangium	2	114	0	0	0	0	0	0	1	0	1	0	1	1	0	1	1	0	1	1	1	1
Isoptericola	1	18	0	0	0	0	0	0	0	0	0	0	0	0	0	0	0	0	0	0	0	0
Isosphaera	2	75	0	0	0	0	0	0	0	0	1	0	1	0	0	1	1	0	0	1	1	0
Jannaschia	3	151	0	0	0	0	0	0	0	0	1	0	1	1	0	1	1	0	1	1	1	1
Janthinobacterium	2	99	0	0	0	0	0	1	0	0	1	0	1	1	0	1	1	0	1	0	1	1
Jonesia	2	56	0	0	0	0	0	0	0	0	1	0	1	1	0	0	1	0	1	1	1	1
Joostella	1	3	0	0	0	0	1	0	0	0	0	0	0	0	0	0	0	0	0	0	0	0
Kaistia	1	2	0	0	0	0	0	0	0	0	0	0	0	0	0	0	0	0	0	0	0	0
Kangiella	2	84	0	0	0	0	1	0	0	0	1	1	1	0	0	1	1	0	1	1	1	1
Ketogulonicigenium	2	102	0	0	0	0	0	0	0	0	1	0	1	0	0	1	1	0	1	1	1	1
Kineococcus	1	24	0	0	0	0	0	0	0	0	0	0	0	0	0	0	0	0	0	0	0	0
Kinetoplastibacterium	1	2	0	0	0	0	0	0	0	0	0	0	0	0	0	0	0	0	0	0	0	0
Kingella	1	4	0	0	0	0	0	0	0	0	0	0	0	0	0	0	0	0	0	0	0	0
Kitasatospora	1	36	0	0	0	0	0	0	0	0	0	0	0	0	0	0	0	0	0	0	0	0
Klebsiella	3	222	0	0	0	0	1	1	0	0	1	0	1	1	0	1	1	1	1	1	1	1
Kluyvera	1	1	0	0	0	0	0	0	0	0	0	0	0	0	0	0	0	0	0	0	0	0
Kocuria	2	87	0	0	0	1	0	0	0	0	1	0	1	1	0	1	1	0	1	1	1	0
Kordia	1	5	0	0	0	0	1	0	0	0	0	0	0	0	0	1	0	0	0	0	0	0
Kosmotoga	2	61	0	0	0	0	0	0	0	0	1	0	1	1	0	0	0	1	0	1	1	0
Kribbella	2	99	0	0	0	0	0	0	0	0	1	0	1	1	0	1	1	0	1	1	1	1
Krokinobacter	2	90	0	0	0	0	1	0	0	0	1	0	1	0	0	1	1	0	0	0	1	0
Kyrpidia	2	113	0	0	0	0	1	0	0	0	1	0	1	1	0	1	1	0	0	1	1	0
Kytococcus	2	70	0	0	0	0	0	0	0	0	1	0	1	1	0	1	1	0	1	1	1	0
Labrenzia	1	4	0	0	0	0	1	0	0	0	0	0	0	0	0	0	0	0	0	0	0	0
Lachnoanaerobaculum	1	1	0	0	0	0	0	0	0	0	0	0	0	0	0	0	0	0	0	0	0	0
Lachnospiraceae	1	51	0	0	0	0	0	0	0	0	1	0	1	1	0	0	1	0	0	0	0	0
Lacinutrix	1	20	0	0	0	0	0	0	0	0	0	0	0	0	0	1	0	0	0	0	0	0
Lactobacillus	2	119	0	0	0	0	0	0	0	0	1	0	1	1	0	1	1	1	0	1	1	1
Lactococcus	2	82	0	0	0	0	0	0	0	0	1	0	1	1	0	1	1	0	0	1	1	0
Lamprocystis	1	2	0	0	0	0	0	0	0	0	0	0	0	0	0	0	0	0	0	0	0	0
Laribacter	2	80	0	0	0	0	0	0	0	0	1	0	1	1	0	1	1	0	0	1	1	1
Lawsonia	2	33	0	0	0	0	0	1	1	0	1	0	1	0	0	0	1	0	0	0	0	0
Leadbetterella	2	79	0	0	0	0	0	0	0	0	1	0	1	0	0	1	1	0	0	1	1	1
Leeuwenhoekiella	1	2	0	0	0	0	1	0	0	0	0	0	0	0	0	0	0	0	0	0	0	0
Legionella	2	117	0	0	1	0	0	1	0	0	1	1	1	1	0	1	1	0	1	1	1	1
Leifsonia	2	48	0	0	0	0	0	0	0	0	1	0	1	1	0	1	1	0	1	1	1	0
Leptolyngbya	1	10	0	0	0	0	0	0	0	0	0	0	0	0	0	0	0	0	0	0	0	0
Leptospira	2	76	0	0	0	0	0	0	0	0	1	1	1	0	0	1	1	0	0	1	1	1
Leptospirillum	1	10	0	0	0	0	0	0	0	0	0	0	0	0	0	0	0	0	0	0	0	0
Leptothrix	3	186	0	0	0	0	0	0	0	0	1	0	1	1	0	1	1	0	1	0	1	1
Leptotrichia	2	43	0	0	0	0	0	0	0	0	1	0	1	0	0	0	1	0	0	1	0	0
Leucobacter	1	2	0	0	0	0	0	0	0	0	0	0	0	0	0	0	0	0	0	0	0	0
Leuconostoc	2	64	0	0	0	0	0	0	0	0	1	0	1	0	0	1	1	0	0	1	1	0

Genus	Cluster Number	Protein Counts	1.3.1.32	1.3.1.56	1.3.99.-	1.4.3.4	1.6.5.-	1.7.1.-	1.8.99.3	1.97.1.8	2.1.1.-	2.1.1.67	2.3.1.-	2.3.1.16	2.3.1.5	2.3.1.9	2.4.2.10	2.4.2.3	2.4.2.4	2.4.2.8	2.5.1.-	2.5.1.18
Liberibacter	1	4	0	0	0	0	0	0	0	0	0	0	0	0	0	0	0	0	0	0	0	0
Limnohabitans	1	11	0	0	0	0	0	0	0	0	0	0	0	0	0	0	0	0	0	0	0	0
Listeria	2	77	0	0	0	0	0	0	0	0	1	0	1	1	0	1	1	0	0	1	1	0
Listeriaceae	1	6	0	0	0	0	0	0	0	0	0	0	0	0	0	0	0	0	0	0	0	0
Listonella	1	38	0	0	0	0	1	0	0	0	1	0	0	0	0	1	1	1	0	1	0	0
Loktanella	1	3	0	0	0	0	0	0	0	0	0	0	0	0	0	0	0	0	0	0	0	0
Lysinibacillus	2	97	0	0	0	0	0	0	0	0	1	0	1	1	0	0	1	0	0	1	1	0
Macrococcus	2	60	0	0	0	0	0	0	0	0	1	0	1	0	0	0	1	0	0	1	1	0
Magnetococcus	2	64	0	0	0	0	0	1	0	0	1	1	1	1	0	1	1	0	0	1	0	1
Magnetospirillum	2	113	0	0	0	0	0	1	0	0	1	0	1	1	0	1	1	0	0	0	1	1
Mahella	2	58	0	0	0	0	0	0	0	0	1	0	1	0	0	0	1	0	0	1	1	0
Mannheimia	2	85	0	0	0	0	1	1	0	0	1	0	1	0	0	0	1	1	0	1	0	1
Maribacter	3	87	0	0	0	0	1	0	0	0	1	0	1	0	0	1	1	0	0	0	1	0
Maricaulis	3	87	0	0	0	1	0	1	0	0	1	0	1	1	0	1	1	0	1	1	1	1
Marichromatium	1	2	0	0	0	0	0	0	0	0	0	0	0	0	0	0	0	0	0	0	0	0
Marinithermus	2	77	0	0	0	0	0	0	0	0	1	0	1	1	0	1	1	0	0	1	0	0
Marinitoga	1	15	0	0	0	0	0	0	0	0	0	0	0	0	0	0	0	0	0	0	0	0
Marinobacter	2	163	0	0	0	0	1	1	0	0	1	1	1	1	0	1	1	0	1	1	1	1
Marinomonas	3	192	0	0	0	0	1	1	0	0	1	1	1	1	0	1	1	0	0	1	1	1
Maritimibacter	1	7	0	0	0	0	1	0	0	0	0	0	1	0	0	0	0	0	0	0	0	0
Marivirga	2	96	0	0	0	0	1	0	0	0	1	0	1	1	0	1	1	0	1	1	1	1
Martelella	1	3	0	0	0	0	0	0	0	0	0	0	0	0	0	0	0	0	0	0	0	0
Marvinbryantia	1	1	0	0	0	0	0	0	0	0	1	0	0	0	0	0	0	0	0	0	0	0
Massilia	1	4	0	0	0	0	0	0	0	0	0	0	0	0	0	0	0	0	0	0	0	0
Mastigocladopsis	1	16	0	0	0	0	0	1	0	0	0	0	0	0	0	0	0	0	0	0	0	0
Megamonas	1	47	0	0	0	0	0	0	0	0	1	0	1	0	0	0	1	0	0	1	1	0
Megasphaera	1	14	0	0	0	0	0	0	0	0	0	0	0	0	0	0	0	0	0	0	0	0
Meiothermus	2	110	0	0	0	0	0	0	0	0	1	0	1	1	0	1	1	1	0	1	1	0
Melissococcus	2	46	0	0	0	0	0	0	0	0	1	0	1	0	0	0	1	0	0	1	1	0
Mesoflavibacter	1	6	0	0	0	0	1	0	0	0	0	0	0	0	0	1	0	0	0	0	0	0
Mesoplasma	1	23	0	0	0	0	0	0	0	0	1	0	1	0	0	0	0	0	1	1	0	0
Mesorhizobium	2	201	0	0	0	0	0	1	0	0	1	0	1	1	1	1	1	0	1	1	1	1
Mesotoga	1	14	0	0	0	0	0	0	0	0	0	0	0	0	0	0	0	0	0	0	0	0
Metallosphaera	1	18	0	0	0	0	0	0	0	0	0	0	0	0	0	0	0	0	0	0	0	0
Methanobacterium	1	7	0	0	0	0	0	0	0	0	0	0	0	0	0	0	0	0	0	0	0	0
Methanobrevibacter	1	9	0	0	0	0	0	0	0	0	0	0	0	0	0	0	0	0	0	0	0	0
Methanocaldococcus	1	6	0	0	0	0	0	0	0	0	0	0	0	0	0	0	0	0	0	0	0	0
Methanocella	1	15	0	0	0	0	0	0	0	0	0	0	0	0	0	0	0	0	0	0	0	0
Methanococcaceae	1	1	0	0	0	0	0	0	0	0	0	0	0	0	0	0	0	0	0	0	0	0
Methanococcoides	1	5	0	0	0	0	0	0	0	0	0	0	0	0	0	0	0	0	0	0	0	0
Methanococcus	1	9	0	0	0	0	0	0	0	0	0	0	0	0	0	0	0	0	0	0	0	0
Methanocorpusculum	1	7	0	0	0	0	0	0	0	0	0	0	0	0	0	0	0	0	0	0	0	0
Methanoculleus	1	8	0	0	0	0	0	0	0	0	0	0	0	0	0	0	0	0	0	0	0	0
Methanohalobium	1	5	0	0	0	0	0	0	0	0	0	0	0	0	0	0	0	0	0	0	0	0
Methanohalophilus	1	5	0	0	0	0	0	0	0	0	0	0	0	0	0	0	0	0	0	0	0	0
Methanolobus	1	5	0	0	0	0	0	0	0	0	0	0	0	0	0	0	0	0	0	0	0	0
Methanomethylovorans	1	5	0	0	0	0	0	0	0	0	0	0	0	0	0	0	0	0	0	0	0	0
Methanoplanus	1	7	0	0	0	0	0	0	0	0	0	0	0	0	0	0	0	0	0	0	0	0
Methanopyrus	1	4	0	0	0	0	0	0	0	0	0	0	0	0	0	0	0	0	0	0	0	0
Methanoregula	1	8	0	0	0	0	0	0	0	0	0	0	0	0	0	0	0	0	0	0	0	0
Methanosaeta	1	9	0	0	0	0	0	0	0	0	0	0	0	0	0	0	0	0	0	0	0	0
Methanosalsum	1	7	0	0	0	0	0	0	0	0	0	0	0	0	0	0	0	0	0	0	0	0

Genus	Cluster Number	Protein Counts	1.3.1.32	1.3.1.56	1.3.99.-	1.4.3.4	1.6.5.-	1.7.1.-	1.8.99.3	1.97.1.8	2.1.1.-	2.1.1.67	2.3.1.-	2.3.1.16	2.3.1.5	2.3.1.9	2.4.2.10	2.4.2.3	2.4.2.4	2.4.2.8	2.5.1.-	2.5.1.18
Methanosarcina	1	18	0	0	0	0	0	0	0	0	0	0	0	0	0	0	0	0	0	0	0	0
Methanosphaera	1	7	0	0	0	0	0	0	0	0	0	0	0	0	0	0	0	0	0	0	0	0
Methanosphaerula	1	8	0	0	0	0	0	0	0	0	0	0	0	0	0	0	0	0	0	0	0	0
Methanospirillum	1	6	0	0	0	0	0	0	0	0	0	0	0	0	0	0	0	0	0	0	0	0
Methanothermobacter	1	6	0	0	0	0	0	0	0	0	0	0	0	0	0	0	0	0	0	0	0	0
Methanothermococcus	1	5	0	0	0	0	0	0	0	0	0	0	0	0	0	0	0	0	0	0	0	0
Methanothermus	1	5	0	0	0	0	0	0	0	0	0	0	0	0	0	0	0	0	0	0	0	0
Methanotorris	1	5	0	0	0	0	0	0	0	0	0	0	0	0	0	0	0	0	0	0	0	0
Methylacidiphilum	2	48	0	0	0	0	0	0	0	0	1	0	1	0	0	0	1	0	0	1	1	0
Methylarcula	1	2	0	0	0	0	0	0	0	0	0	0	0	0	0	0	0	0	0	0	0	0
Methylibium	1	38	0	0	0	0	0	0	0	0	0	0	0	0	0	0	0	0	0	0	0	0
Methylobacillus	2	61	0	0	0	0	0	0	0	0	1	0	1	0	0	0	1	0	0	1	1	0
Methylobacterium	3	236	0	0	0	0	0	1	0	0	1	0	1	1	0	1	1	1	1	1	1	1
Methylocella	3	117	0	0	0	0	0	1	0	0	1	0	1	0	0	1	1	0	0	0	1	1
Methylococcus	2	80	0	0	0	0	1	1	0	0	1	1	1	0	0	0	1	0	1	1	1	1
Methylocystis	1	21	0	0	0	0	0	0	0	0	0	0	0	0	0	0	0	0	0	0	0	0
Methyloferula	1	2	0	0	0	0	0	0	0	0	0	0	0	0	0	0	0	0	0	0	0	0
Methylomicrobium	1	12	0	0	0	0	0	0	0	0	0	0	0	0	0	0	0	0	0	0	0	0
Methylomonas	1	15	0	0	0	0	0	0	0	0	0	0	0	0	0	0	0	0	0	0	0	0
Methylophaga	1	7	0	0	0	0	0	0	0	0	0	0	0	0	0	0	0	0	0	0	0	0
Methylophilus	1	2	0	0	0	0	0	0	0	0	0	0	0	0	0	0	0	0	0	0	0	0
Methylosinus	1	2	0	0	0	0	0	0	0	0	0	0	0	0	0	0	0	0	0	0	0	0
Methylotenera	2	72	0	0	0	0	0	0	0	0	1	1	1	0	0	0	1	0	0	1	1	0
Methyloversatilis	1	5	0	0	0	0	0	0	0	0	0	0	0	0	0	0	0	0	0	0	0	0
Methylovorus	2	66	0	0	0	0	0	0	0	0	1	1	1	0	0	0	1	0	0	1	1	0
Micavibrio	1	9	0	0	0	0	0	0	0	0	0	0	0	0	0	0	0	0	0	0	0	0
Microbacterium	2	115	0	0	0	0	0	0	0	0	1	0	1	1	0	1	1	0	1	1	1	1
Microchaete	1	23	0	0	0	0	0	1	0	0	1	0	0	0	0	0	0	0	0	0	0	0
Micrococcus	2	72	0	0	0	0	0	0	0	0	1	0	1	0	0	1	1	0	1	1	1	0
Microcoleus	1	17	0	0	0	0	0	0	0	0	0	0	0	0	0	0	0	0	0	0	0	0
Microcystis	2	67	0	0	0	1	0	1	0	0	1	0	1	0	0	1	1	1	0	0	1	0
Microlunatus	1	25	0	0	0	0	0	0	0	0	0	0	0	0	0	0	0	0	0	0	0	0
Micromonospora	2	115	0	0	0	1	0	0	0	0	1	0	1	1	0	1	1	0	1	1	1	1
Microvirga	1	6	0	0	0	0	0	0	0	0	0	0	0	0	0	0	0	0	0	0	0	0
Mobiluncus	1	41	0	0	0	0	0	0	0	0	1	0	1	0	0	0	1	0	0	1	0	0
Modestobacter	1	43	0	0	0	0	0	0	0	0	0	0	0	0	0	0	0	0	0	0	0	0
Moorea	1	2	0	0	0	0	0	0	0	0	0	0	0	0	0	0	0	0	0	0	0	0
Moorella	2	69	0	0	0	0	0	0	1	0	1	0	1	0	0	1	1	0	0	1	0	0
Moraxella	2	66	0	0	0	0	1	0	0	0	1	0	1	1	0	0	1	0	0	1	1	1
Morganella	1	29	0	0	0	0	1	0	0	0	0	0	0	0	0	0	0	0	0	0	0	0
Moritella	1	2	0	0	0	0	0	0	0	0	0	0	0	0	0	0	0	0	0	0	0	0
Muricauda	1	23	0	0	0	0	1	0	0	0	0	0	0	0	0	0	0	0	0	0	0	0
Mycobacterium	3	306	1	1	1	1	0	0	0	0	1	0	1	1	1	1	1	1	1	1	1	1
Mycoplasma	1	53	0	0	0	0	0	0	0	0	1	0	1	0	0	0	1	0	1	1	0	0
Myxococcus	2	110	0	0	0	0	0	0	0	0	1	0	1	1	0	1	1	0	0	1	1	1
Nakamurella	2	115	0	0	0	0	0	0	0	0	1	0	1	1	0	1	1	0	1	1	1	1
napthalene	1	2	0	0	0	0	0	0	0	0	0	0	0	0	0	0	0	0	0	0	0	0
Natranaerobius	2	55	0	0	0	0	1	0	0	0	1	0	1	1	0	0	1	0	0	1	0	0
Natrialba	1	16	0	0	0	0	0	0	0	0	0	0	0	0	0	0	0	0	0	0	0	0
Natrinema	1	16	0	0	0	0	0	0	0	0	0	0	0	0	0	0	0	0	0	0	0	0
Natronobacterium	1	11	0	0	0	0	0	0	0	0	0	0	0	0	0	0	0	0	0	0	0	0
Natronococcus	1	21	0	0	0	0	0	0	0	0	0	0	0	0	0	0	0	0	0	0	0	0

Genus	Cluster Number	Protein Counts	1.3.1.32	1.3.1.56	1.3.99.-	1.4.3.4	1.6.5.-	1.7.1.-	1.8.99.3	1.97.1.8	2.1.1.-	2.1.1.67	2.3.1.-	2.3.1.16	2.3.1.5	2.3.1.9	2.4.2.10	2.4.2.3	2.4.2.4	2.4.2.8	2.5.1.-	2.5.1.18
Natronomonas	1	20	0	0	0	0	0	0	0	0	0	0	0	0	0	0	0	0	0	0	0	0
Nautilia	2	48	0	0	0	0	0	0	0	0	1	0	1	0	0	0	1	0	0	0	1	0
Neisseria	2	67	0	0	0	0	1	1	0	0	1	0	1	0	0	0	1	0	0	1	1	0
Neisseriaceae	1	1	0	0	0	0	0	0	0	0	0	0	0	0	0	0	0	0	0	0	0	0
Neorickettsia	1	31	0	0	0	0	0	0	0	0	1	0	1	0	0	0	1	0	0	0	1	0
Neptuniibacter	1	2	0	0	0	0	0	0	0	0	0	0	0	0	0	0	0	0	0	0	0	0
Niastella	1	21	0	0	0	0	0	0	0	0	0	0	0	0	0	0	0	0	0	0	0	0
Nitratifractor	2	54	0	0	0	0	0	0	0	0	1	0	1	0	0	0	1	0	0	0	1	0
Nitratireductor	1	19	0	0	0	0	1	0	0	0	1	0	0	0	0	1	0	0	0	0	0	0
Nitratiruptor	2	40	0	0	0	0	0	1	0	0	1	0	1	0	0	0	1	0	0	0	0	0
Nitrobacter	2	99	0	0	0	0	1	0	0	0	1	0	1	1	0	1	1	1	1	0	1	1
Nitrococcus	1	2	0	0	0	0	0	0	0	0	0	0	0	0	0	0	0	0	0	0	0	0
Nitrosococcus	2	94	0	0	0	0	0	1	0	0	1	0	1	1	0	1	1	0	1	1	1	1
Nitrosomonas	2	72	0	0	0	0	1	1	0	0	1	1	1	0	0	1	1	0	0	0	1	1
Nitrosopumilaceae	1	7	0	0	0	0	0	0	0	0	0	0	0	0	0	0	0	0	0	0	0	0
Nitrosopumilus	1	11	0	0	0	0	0	0	0	0	0	0	0	0	0	0	0	0	0	0	0	0
Nitrosospira	2	74	0	0	0	0	0	1	0	0	1	1	1	0	0	1	1	0	0	1	1	1
Nocardia	2	185	0	0	1	1	0	0	0	0	1	0	1	1	1	1	1	0	0	1	1	1
Nocardioidaceae	1	2	0	0	0	0	0	0	0	0	0	0	0	0	0	0	0	0	0	0	0	0
Nocardioides	2	157	0	1	1	1	0	0	0	0	1	0	1	1	0	1	1	0	1	1	1	1
Nocardiopsis	2	102	0	0	0	0	0	0	0	0	1	0	1	1	0	1	1	0	1	1	1	1
Nodularia	1	22	0	0	0	0	0	0	0	0	1	0	0	0	0	0	0	0	0	0	0	0
Nonlabens	1	19	0	0	0	0	0	0	0	0	0	0	0	0	0	0	0	0	0	0	0	0
Nostoc	2	111	0	0	0	1	0	1	0	0	1	0	1	0	0	0	1	1	0	0	1	1
Nostocaceae	1	2	0	0	0	0	0	0	0	0	0	0	0	0	0	0	0	0	0	0	0	0
Novosphingobium	2	169	1	0	1	0	0	0	0	0	1	0	1	1	0	1	1	0	1	0	1	1
Oceanibaculum	1	3	0	0	0	0	0	0	0	0	0	0	0	0	0	0	0	0	0	0	0	0
Oceanibulbus	1	12	0	0	0	0	0	0	0	0	0	0	0	0	0	1	0	0	0	0	0	0
Oceanicola	1	9	0	0	0	0	1	0	0	0	0	0	1	0	0	0	0	0	0	0	0	0
Oceanimonas	1	33	0	0	0	0	0	0	0	0	0	0	0	0	0	0	0	0	0	0	0	0
Oceaniovalibus	1	2	0	0	0	0	0	0	0	0	0	0	0	0	0	0	0	0	0	0	0	0
Oceanithermus	2	72	0	0	0	0	0	0	0	0	1	0	1	0	0	1	1	0	0	1	1	0
Oceanobacillus	2	91	0	0	0	0	0	1	0	0	1	0	1	1	0	1	1	0	0	1	1	0
Oceanospirillum	1	2	0	0	0	0	0	0	0	0	0	0	0	0	0	0	0	0	0	0	0	0
Ochrobactrum	2	133	0	0	0	0	0	1	0	0	1	0	1	1	0	1	1	0	0	1	1	1
Octadecabacter	1	29	0	0	0	0	0	0	0	0	0	0	0	0	0	0	0	0	0	0	0	0
Oenococcus	2	41	0	0	0	0	0	0	0	0	1	0	1	0	0	1	1	0	0	1	0	0
Oleomonas	1	2	0	0	0	0	0	0	0	0	0	0	0	0	0	0	0	0	0	0	0	0
Oligotropha	3	87	0	0	0	0	0	1	0	0	1	0	1	1	0	1	1	0	0	0	1	1
Olsenella	2	40	0	0	0	0	0	0	0	0	1	0	1	0	0	0	1	1	0	1	0	0
Opitutus	2	67	0	0	0	0	0	1	0	0	1	0	1	0	0	0	1	1	0	1	1	1
Orientia	1	21	0	0	0	0	0	0	0	0	1	0	0	0	0	0	0	0	0	0	1	0
Ornithobacterium	1	11	0	0	0	0	0	0	0	0	0	0	0	0	0	0	0	0	0	0	0	0
Oscillatoria	1	16	0	0	0	0	0	0	0	0	0	0	0	0	0	0	0	0	0	0	0	0
Oscillatoriales	1	2	0	0	0	0	0	0	0	0	0	0	0	0	0	0	0	0	0	0	0	0
Oscillibacter	1	20	0	0	0	0	0	0	0	0	1	0	0	0	0	0	0	0	0	0	0	0
Owenweeksia	1	15	0	0	0	0	0	0	0	0	0	0	0	0	0	0	0	0	0	0	0	0
Oxalobacteraceae	1	4	0	0	0	0	0	0	0	0	1	0	0	0	0	0	0	0	0	0	0	0
Paenibacillus	2	152	0	0	0	0	0	0	0	0	1	0	1	1	0	1	1	1	0	1	1	1
Paludibacter	2	67	0	0	0	0	0	0	0	0	1	0	1	0	0	0	1	0	0	1	1	1
Pandoraea	1	18	0	0	0	0	0	0	0	0	0	0	0	0	0	0	0	0	0	0	0	0
Pannonibacter	1	3	0	0	0	0	0	0	0	0	0	0	0	0	0	0	0	0	0	0	0	0

Genus	Cluster Number	Protein Counts	1.3.1.32	1.3.1.56	1.3.99.-	1.4.3.4	1.6.5.-	1.7.1.-	1.8.99.3	1.97.1.8	2.1.1.-	2.1.1.67	2.3.1.-	2.3.1.16	2.3.1.5	2.3.1.9	2.4.2.10	2.4.2.3	2.4.2.4	2.4.2.8	2.5.1.-	2.5.1.18
Pantholops	1	23	0	0	0	0	0	0	0	0	1	0	0	1	0	1	0	0	0	0	1	1
Pantoea	2	181	0	0	0	0	1	0	0	0	1	0	1	1	0	1	1	1	1	1	1	1
Parabacteroides	2	64	0	0	0	0	1	0	0	0	1	0	1	0	0	0	1	0	0	1	1	0
Parachlamydia	1	11	0	0	0	0	0	0	0	0	0	0	0	0	0	0	0	0	0	0	0	0
Paracoccus	2	169	0	0	0	0	1	1	0	0	1	0	1	1	0	1	1	0	1	0	1	1
Parvibaculum	3	117	0	0	0	0	0	1	0	0	1	0	1	1	0	1	1	0	1	1	1	1
Parvularcula	2	72	0	0	0	0	0	0	0	0	1	0	1	0	0	1	1	0	0	1	1	1
Pasteurella	2	85	0	0	0	0	1	1	0	0	1	0	1	0	0	0	1	1	0	1	0	1
Pasteurellaceae	1	2	0	0	0	0	1	0	0	0	0	0	0	0	0	0	0	0	0	0	0	0
Pectobacterium	1	44	0	0	0	0	0	0	0	0	1	0	0	0	0	0	1	0	0	0	1	0
Pediococcus	1	43	0	0	0	0	0	0	0	0	1	0	1	0	0	0	0	0	0	1	0	0
Pedobacter	2	88	0	0	0	0	0	0	0	0	1	0	1	0	0	1	1	0	0	1	1	1
Pelagibaca	1	10	0	0	0	0	1	0	0	0	0	0	1	0	0	1	0	0	0	0	0	0
Pelagibacterium	1	39	0	0	0	0	0	0	0	0	0	0	0	0	0	0	0	0	0	0	0	0
Pelobacter	2	85	0	0	0	0	0	0	0	0	1	0	1	0	0	0	1	1	0	1	1	0
Pelodictyon	2	62	0	0	0	0	0	1	0	0	1	0	1	0	0	0	1	0	0	1	1	0
Pelosinus	1	1	0	0	0	0	0	0	0	0	0	0	0	0	0	0	0	0	0	0	0	0
Pelotomaculum	2	60	0	0	0	0	0	0	0	0	1	0	1	1	0	0	1	0	0	1	0	0
Peptostreptococcus	1	1	0	0	0	0	0	0	0	0	0	0	0	0	0	0	0	0	0	0	0	0
Persephonella	2	55	0	0	0	0	0	0	0	0	1	0	1	0	0	0	1	0	0	1	1	0
Petrotoga	2	72	0	0	0	0	1	0	0	0	1	0	1	0	0	1	1	0	0	1	0	0
Phaeobacter	2	55	0	0	0	0	1	0	0	0	1	0	0	0	0	1	1	0	0	0	0	0
Phenylobacterium	3	103	0	0	0	0	0	0	0	0	1	0	1	1	1	1	1	0	1	1	1	1
Photobacterium	2	119	0	0	0	0	1	1	0	0	1	1	1	1	0	1	1	1	1	1	1	1
Photorhabdus	2	149	0	0	0	0	1	1	0	0	1	0	1	1	0	1	1	1	1	1	1	1
Phycisphaera	1	9	0	0	0	0	0	0	0	0	0	0	0	0	0	0	0	0	0	0	0	0
Phyllobacterium	1	6	0	0	0	0	0	0	0	0	0	0	0	1	0	0	0	0	0	0	0	0
Picrophilus	1	15	0	0	0	0	0	0	0	0	0	0	0	0	0	0	0	0	0	0	0	0
Pirellula	2	85	0	0	0	0	0	0	0	0	1	0	1	1	0	0	1	0	1	1	1	0
Planctomyces	2	93	0	0	0	0	1	1	0	0	1	0	1	0	0	1	1	0	0	1	1	1
Plautia	1	17	0	0	0	0	0	0	0	0	1	0	0	0	0	0	0	0	0	0	1	0
Plesiomonas	1	4	0	0	0	0	0	0	0	0	0	0	0	0	0	0	0	0	0	0	0	0
Pleurocapsa	1	23	0	0	0	0	0	0	0	0	0	0	0	0	0	0	0	0	0	0	0	0
Polaribacter	2	74	0	0	0	0	1	0	0	0	1	0	1	0	0	1	1	0	0	0	1	0
Polaromonas	3	238	0	1	0	0	0	1	0	0	1	0	1	1	0	1	1	0	1	0	1	1
Polymorphum	3	173	0	0	0	0	0	0	0	0	1	0	1	1	0	1	1	0	1	1	1	1
Polynucleobacter	1	19	0	0	0	0	0	0	0	0	0	0	0	0	0	0	0	0	0	0	0	0
Ponticaulis	1	1	0	0	0	0	0	0	0	0	0	0	0	0	0	0	0	0	0	0	0	0
Porphyromonas	2	63	0	0	0	0	1	1	0	0	1	0	1	0	0	0	1	0	0	1	1	0
Prevotella	1	65	0	0	0	0	1	0	0	0	1	0	1	0	0	0	1	0	0	1	1	0
Prochlorococcus	2	68	0	0	0	0	0	1	0	0	1	0	1	0	0	0	1	0	0	0	1	1
Propionibacterium	1	62	0	0	0	0	0	0	0	0	1	0	1	0	0	0	1	0	1	1	1	0
Prosthecochloris	1	16	0	0	0	0	0	0	0	0	0	0	0	0	0	0	0	0	0	0	0	0
Proteobacteria	1	19	0	0	0	0	0	0	0	0	0	0	1	0	0	0	0	0	1	0	0	1
Proteus	2	113	0	0	1	0	1	1	0	0	1	0	1	1	0	1	1	1	1	1	1	1
Providencia	1	38	0	0	1	0	1	0	0	0	1	0	0	0	0	0	0	0	0	0	0	0
Pseudaminobacter	1	10	0	0	0	0	0	0	0	0	0	0	0	1	0	0	0	0	0	1	0	0
Pseudanabaena	1	11	0	0	0	0	0	0	0	0	0	0	0	0	0	0	0	0	0	0	0	0
Pseudoalteromonas	2	188	0	0	0	0	1	1	0	0	1	1	1	1	0	1	1	1	0	1	1	1
Pseudochrobactrum	1	7	0	0	0	0	0	0	0	0	0	0	0	1	0	0	0	0	0	0	0	0
Pseudogulbenkiania	1	40	0	0	0	0	0	0	0	0	1	0	0	0	0	0	0	0	0	0	0	0
pseudomallei	3	131	0	0	0	0	0	1	0	0	1	0	1	1	0	1	1	0	1	1	1	1

Genus	Cluster Number	Protein Counts	1.3.1.32	1.3.1.56	1.3.99.-	1.4.3.4	1.6.5.-	1.7.1.-	1.8.99.3	1.97.1.8	2.1.1.-	2.1.1.67	2.3.1.-	2.3.1.16	2.3.1.5	2.3.1.9	2.4.2.10	2.4.2.3	2.4.2.4	2.4.2.8	2.5.1.-	2.5.1.18
Pseudomonas	3	381	0	1	0	1	1	1	0	0	1	1	1	1	1	1	1	0	1	1	1	1
Pseudonocardia	3	180	0	0	1	0	0	0	0	0	1	0	1	1	0	1	1	1	0	1	1	1
Pseudorhodobacter	1	4	0	0	0	0	0	0	0	0	0	0	0	0	0	0	0	0	0	0	0	0
Pseudovibrio	1	33	0	0	0	0	0	0	0	0	0	0	0	0	0	0	0	0	0	0	0	0
Pseudoxanthomonas	2	124	0	0	0	0	0	0	0	0	1	1	1	0	0	1	1	0	1	1	1	1
Psychrobacter	2	124	0	0	0	0	1	1	0	0	1	1	1	1	0	1	1	0	0	1	0	1
Psychroflexus	1	22	0	0	0	0	1	0	0	0	0	0	0	0	0	0	0	0	0	0	0	0
Psychromonas	2	110	0	0	0	0	1	1	0	0	1	0	1	0	0	1	1	1	1	1	1	1
Pusillimonas	3	111	0	0	1	0	0	0	0	0	1	0	1	1	0	1	1	0	0	0	1	1
Pyrobaculum	1	15	0	0	0	0	0	0	0	0	0	0	0	0	0	0	0	0	0	0	0	0
Pyrococcus	1	10	0	0	0	0	0	0	0	0	0	0	0	0	0	0	0	0	0	0	0	0
Pyrolobus	1	4	0	0	0	0	0	0	0	0	0	0	0	0	0	0	0	0	0	0	0	0
Rahnella	2	143	0	0	0	0	0	0	0	0	1	0	1	0	0	1	1	1	0	1	1	1
Ralstonia	3	332	0	0	1	1	0	1	0	0	1	0	1	1	1	1	1	0	1	1	1	1
Ramlibacter	1	35	0	0	0	0	0	0	0	0	0	0	0	0	0	0	0	0	0	0	0	0
Raoultella	2	139	0	0	0	0	1	1	0	0	1	0	1	1	0	1	1	1	1	1	1	1
Raphidiopsis	1	5	0	0	0	0	0	0	0	0	0	0	0	0	0	0	0	0	0	0	0	0
Reinekea	1	2	0	0	0	0	0	0	0	0	0	0	0	0	0	0	0	0	0	0	0	0
Renibacterium	2	67	0	0	0	0	0	0	0	0	1	0	1	1	0	1	1	0	1	1	1	1
Rhizobiaceae	1	3	0	0	0	0	0	0	0	0	0	0	0	0	0	0	0	0	0	0	0	0
Rhizobiales	1	6	0	0	0	0	0	0	0	0	0	0	0	1	0	0	0	0	0	0	0	0
Rhizobium	3	257	1	0	0	0	0	1	0	0	1	0	1	1	0	1	1	0	1	1	1	1
Rhizobium/Agrobacterium	2	87	0	0	0	0	0	0	0	0	1	0	1	0	0	1	1	0	0	1	1	1
Rhodanobacter	1	21	0	0	0	0	0	0	0	0	0	0	0	0	0	0	0	0	0	0	0	0
Rhodobacter	3	175	0	0	0	0	0	0	0	0	1	0	1	1	0	1	1	0	1	1	1	1
Rhodobacteraceae	1	21	0	0	0	0	1	0	0	0	1	0	1	0	0	1	0	0	0	0	0	0
Rhodobacterales	1	34	0	0	0	0	1	0	0	0	1	0	1	0	0	1	1	0	0	0	0	0
Rhodococcus	2	292	0	1	1	1	0	0	0	0	1	0	1	1	1	1	1	0	1	1	1	1
Rhodocyclaceae	1	7	0	0	0	0	0	0	0	0	0	0	0	0	0	0	0	0	0	0	0	0
Rhodoferax	3	144	0	0	0	0	0	1	0	0	1	0	1	1	0	1	1	0	1	0	1	1
Rhodomicrobium	2	103	0	0	0	0	0	0	1	0	1	0	1	0	0	1	1	0	0	0	1	1
Rhodopirellula	2	87	0	0	0	0	1	1	0	0	1	0	1	0	0	0	1	0	1	1	1	0
Rhodopseudomonas	3	246	0	0	0	0	0	1	0	0	1	0	1	1	0	1	1	0	1	0	1	1
Rhodospirillum	2	121	0	0	0	0	0	0	0	0	1	0	1	1	0	1	1	0	1	0	1	1
Rhodothermus	2	76	0	0	1	0	0	0	0	0	1	0	1	1	0	1	1	0	0	1	1	0
Rhodovulum	1	4	0	0	0	0	0	0	0	0	0	0	0	0	0	0	0	0	0	0	0	0
Richelia	1	2	0	0	0	0	0	0	0	0	0	0	0	0	0	0	0	0	0	0	0	0
Rickettsia	2	43	0	0	0	0	0	1	0	0	1	0	1	1	0	1	0	0	0	0	1	0
Rickettsiaceae	1	5	0	0	0	0	0	0	0	0	1	0	0	0	0	0	0	0	0	0	1	0
Riemerella	2	61	0	0	0	0	0	0	0	0	1	0	1	0	0	1	0	0	0	1	0	0
Rivularia	1	18	0	0	0	0	0	0	0	0	0	0	0	0	0	0	0	0	0	0	0	0
Robiginitalea	2	83	0	0	0	0	1	0	0	0	1	0	1	1	0	0	1	0	1	0	1	0
Roseburia	2	58	0	0	0	0	0	0	0	0	1	0	1	0	0	1	1	1	0	0	1	0
Roseibium	1	3	0	0	0	0	1	0	0	0	0	0	0	0	0	0	0	0	0	0	0	0
Roseiflexus	3	100	0	0	1	0	0	0	0	0	1	0	1	1	0	1	1	1	0	0	1	0
Roseobacter	2	149	0	0	0	0	1	0	0	0	1	0	1	0	0	1	1	0	1	1	1	1
Roseomonas	1	2	0	0	0	0	0	0	0	0	0	0	0	0	0	0	0	0	0	0	0	0
Roseovarius	1	15	0	0	0	0	1	0	0	0	0	0	1	0	0	1	0	0	0	0	0	0
Rothia	1	44	0	0	0	0	0	0	0	0	1	0	1	0	0	0	1	0	0	1	1	0
Rubrivivax	1	24	0	0	0	0	0	0	0	0	0	0	0	0	0	0	0	0	0	0	0	0
Rubrobacter	3	117	1	0	0	0	0	0	0	0	1	0	1	1	0	1	1	0	0	1	1	1
Ruegeria	2	189	0	0	1	0	1	1	0	0	1	0	1	1	0	1	1	0	1	1	1	1

Genus	Cluster Number	Protein Counts	1.3.1.32	1.3.1.56	1.3.99.-	1.4.3.4	1.6.5.-	1.7.1.-	1.8.99.3	1.97.1.8	2.1.1.-	2.1.1.67	2.3.1.-	2.3.1.16	2.3.1.5	2.3.1.9	2.4.2.10	2.4.2.3	2.4.2.4	2.4.2.8	2.5.1.-	2.5.1.18
Ruminococcaceae	1	2	0	0	0	0	0	0	0	0	1	0	0	0	0	0	0	0	0	0	0	0
Ruminococcus	2	101	0	0	0	0	0	0	0	0	1	0	1	1	0	1	1	1	0	1	1	1
Runella	1	26	0	0	0	0	0	0	0	0	0	0	0	0	0	0	0	0	0	0	0	0
Saccharomonospora	2	132	0	0	0	0	0	0	0	0	1	0	1	1	0	1	1	0	1	1	1	0
Saccharophagus	2	81	0	0	0	0	1	1	0	0	1	0	1	1	0	1	1	0	1	1	1	1
Saccharopolyspora	3	194	0	0	0	1	0	0	0	0	1	0	1	1	1	1	1	0	1	1	1	1
Saccharothrix	1	32	0	0	0	0	0	0	0	0	0	0	0	0	0	0	0	0	0	0	0	0
Sagittula	1	10	0	0	0	0	0	0	0	0	0	0	0	0	0	1	0	0	0	0	0	0
Salinibacter	2	77	0	0	0	1	0	0	0	0	1	0	1	1	0	1	1	0	0	1	1	0
Salinimonas	1	1	0	0	0	0	0	0	0	0	0	0	0	0	0	0	0	0	0	0	0	0
Salinispora	3	127	0	0	1	0	0	0	0	0	1	0	1	1	0	1	1	0	1	1	1	1
Salinivibrio	1	2	0	0	0	0	1	0	0	0	0	0	0	0	0	0	0	0	0	0	0	0
Salmonella	2	151	0	0	1	0	1	1	0	0	1	0	1	1	0	1	1	1	1	1	1	1
Sanguibacter	2	73	0	0	0	0	0	0	0	0	1	0	1	1	0	0	1	0	1	1	1	1
Saprospira	1	13	0	0	0	0	0	0	0	0	0	0	0	0	0	0	0	0	0	0	0	0
Scardovia	1	1	0	0	0	0	0	0	0	0	0	0	0	0	0	0	0	0	0	0	0	0
Scytonema	1	10	0	0	0	0	0	0	0	0	0	0	0	0	0	0	0	0	0	0	0	0
Sebaldella	2	69	0	0	0	0	0	0	0	0	1	0	1	0	0	0	1	1	0	1	0	0
Segniliparus	2	85	0	0	0	0	0	0	0	0	1	0	1	1	0	1	1	0	1	1	1	0
Selenomonas	2	57	0	0	0	0	0	0	0	0	1	0	1	0	0	0	1	0	0	1	1	0
Serinicoccus	1	1	0	0	0	0	0	0	0	0	0	0	0	0	0	0	0	0	0	0	0	0
Serratia	2	186	0	0	1	0	1	1	0	0	1	0	1	1	1	1	1	1	1	1	1	1
Shewanella	3	214	0	0	1	0	1	1	0	1	1	1	1	1	0	1	1	1	1	1	1	1
Shigella	2	172	0	0	1	0	0	1	0	0	1	0	1	1	0	1	1	1	1	1	1	1
Shinella	1	2	0	0	0	0	0	0	0	0	0	0	0	0	0	0	0	0	0	0	0	0
Sideroxydans	2	81	0	0	0	0	0	0	1	0	1	1	1	1	0	0	1	0	0	1	1	1
Silicibacter	2	105	0	0	1	0	1	0	0	0	1	0	1	1	0	1	1	0	0	1	1	1
Simiduia	1	18	0	0	0	0	0	0	0	0	0	0	0	0	0	0	0	0	0	0	0	0
Simkania	1	6	0	0	0	0	0	0	0	0	0	0	0	0	0	0	0	0	0	0	0	0
Simonsiella	1	2	0	0	0	0	0	0	0	0	0	0	0	0	0	0	0	0	0	0	0	0
Singulisphaera	1	18	0	0	0	0	0	0	0	0	0	0	0	0	0	0	0	0	0	0	0	0
Sinorhizobium	3	207	0	0	0	0	0	1	0	0	1	0	1	1	0	1	1	0	1	1	1	1
Slackia	2	46	0	0	0	0	0	1	0	0	1	0	1	0	0	0	1	0	0	1	1	0
Smaragdicoccus	1	3	0	0	0	0	0	0	0	0	0	0	0	0	0	0	0	0	0	0	0	0
Sodalis	2	71	0	0	0	0	0	1	0	0	1	0	1	0	0	0	1	1	1	1	1	1
Solibacillus	1	23	0	0	0	0	0	0	0	0	0	0	0	0	0	0	0	0	0	1	0	0
Solitalea	1	13	0	0	0	0	0	0	0	0	0	0	0	0	0	0	0	0	0	0	0	0
Sorangium	3	115	0	0	1	0	0	0	0	0	1	0	1	1	1	1	1	0	0	1	1	1
Sphaerobacter	2	100	0	0	0	0	0	1	0	0	1	0	1	1	0	1	1	0	0	1	1	0
Sphaerochaeta	1	42	0	0	0	0	0	0	0	0	1	0	1	0	0	0	1	1	0	1	0	0
Sphingobacterium	3	85	0	0	0	0	0	0	0	0	1	0	1	1	1	1	1	0	0	1	1	1
Sphingobium	1	179	0	0	0	0	0	0	0	0	1	0	1	1	0	1	1	0	1	0	1	1
Sphingomonadaceae	1	15	0	0	0	0	0	0	0	0	0	0	0	0	0	1	0	0	0	0	0	1
Sphingomonas	2	220	0	0	1	1	0	1	0	0	1	0	1	1	0	1	1	0	0	0	1	1
Sphingopyxis	3	99	0	0	0	0	0	0	0	0	1	0	1	1	0	1	1	0	1	1	1	1
Spirochaeta	2	103	0	0	0	0	1	0	0	0	1	0	1	0	0	0	1	1	0	1	1	1
Spiroplasma	1	6	0	0	0	0	0	0	0	0	0	0	0	0	0	0	0	0	0	0	0	0
Spirosoma	2	107	0	0	0	0	0	0	0	0	1	1	1	0	0	1	1	0	0	1	1	1
Stackebrandtia	2	110	1	0	1	0	0	0	0	0	1	0	1	1	1	1	1	0	1	1	1	1
Stanieria	1	17	0	0	0	0	0	0	0	0	0	0	0	0	0	0	0	0	0	0	0	0
Staphylococcus	2	108	0	0	0	0	0	1	0	0	1	0	1	1	0	1	1	0	0	1	1	1
Staphylothermus	1	12	0	0	0	0	0	0	0	0	0	0	0	0	0	0	0	0	0	0	0	0

Genus	Cluster Number	Protein Counts	1.3.1.32	1.3.1.56	1.3.99.-	1.4.3.4	1.6.5.-	1.7.1.-	1.8.99.3	1.97.1.8	2.1.1.-	2.1.1.67	2.3.1.-	2.3.1.16	2.3.1.5	2.3.1.9	2.4.2.10	2.4.2.3	2.4.2.4	2.4.2.8	2.5.1.-	2.5.1.18
Stappia	1	2	0	0	0	0	0	0	0	0	0	0	0	0	0	0	0	0	0	0	0	0
Starkeya	3	134	0	0	0	0	0	0	0	0	1	1	1	1	0	1	1	0	1	1	1	1
Stenotrophomonas	3	104	0	0	0	0	0	1	0	0	1	0	1	1	0	1	1	0	1	1	1	1
Stigmatella	3	129	0	0	0	0	0	0	0	0	1	0	1	1	0	1	1	0	0	1	1	1
Streptobacillus	1	38	0	0	0	0	0	0	0	0	1	0	1	1	0	0	0	0	0	1	0	0
Streptococcus	2	121	0	0	0	0	0	1	0	0	1	0	1	1	0	1	1	1	0	1	1	1
Streptomyces	2	253	0	0	1	1	0	0	0	0	1	0	1	1	0	1	1	1	1	1	1	1
Streptosporangium	3	148	0	0	1	0	0	0	0	0	1	0	1	1	0	1	1	0	1	1	1	1
Strigomonas	1	2	0	0	0	0	0	0	0	0	0	0	0	0	0	0	0	0	0	0	0	0
Sulfitobacter	1	10	0	0	0	0	0	0	0	0	0	0	1	0	0	1	0	0	0	0	0	0
Sulfobacillus	1	30	0	0	0	0	0	0	0	0	0	0	0	0	0	0	0	0	0	0	0	0
Sulfolobus	1	23	0	0	0	0	0	0	0	0	0	0	0	0	0	0	0	0	0	0	0	0
Sulfuricurvum	2	67	0	0	0	0	0	0	0	0	1	0	1	0	0	0	1	0	0	0	1	0
Sulfurihydrogenibium	2	55	0	0	0	0	0	1	0	0	1	0	1	0	0	0	1	0	0	1	1	0
Sulfurimonas	2	60	0	0	0	0	1	1	0	0	1	0	1	0	0	0	1	0	0	0	1	0
Sulfurospirillum	1	48	0	0	0	0	0	1	0	0	1	0	1	0	0	0	1	0	0	0	0	0
Sulfurovum	2	48	0	0	0	0	0	1	0	0	1	0	1	0	0	0	1	0	0	0	1	0
Symbiobacterium	2	68	0	0	0	0	0	0	0	0	1	0	1	1	0	1	1	0	0	1	1	1
Synechococcus	1	34	0	0	0	0	0	0	0	0	0	0	0	0	0	0	0	0	0	0	0	0
Synechocystis	1	40	0	0	0	0	0	1	0	0	1	0	1	0	0	0	1	0	0	0	1	0
Synergistetes	1	27	0	0	0	0	0	0	0	0	1	0	1	0	0	0	1	0	0	1	0	0
Syntrophobacter	2	101	0	0	0	0	0	1	1	0	1	0	1	1	0	1	1	0	0	1	1	0
Syntrophobotulus	2	71	0	0	0	0	0	0	0	0	1	0	1	0	0	0	1	0	0	1	0	0
Syntrophomonas	2	58	0	0	0	0	0	0	0	0	1	0	1	0	0	1	1	0	0	1	0	0
Syntrophothermus	2	60	0	0	0	0	0	0	0	0	1	0	1	1	0	1	1	0	0	1	1	0
Syntrophus	2	66	0	0	0	0	0	1	0	0	1	0	1	1	0	1	1	0	0	1	0	0
Tannerella	1	8	0	0	0	0	0	0	0	0	1	0	0	0	0	0	0	0	0	0	0	0
Taylorella	1	43	0	0	0	0	0	0	0	0	1	0	1	0	0	0	1	0	0	0	1	1
Tepidanaerobacter	2	55	0	0	0	0	0	0	0	0	1	0	1	0	0	0	1	0	0	1	1	0
Teredinibacter	2	109	0	0	0	0	1	0	0	0	1	1	1	1	0	1	1	0	1	1	1	1
Terrabacter	1	1	0	0	0	0	0	0	0	0	0	0	0	0	0	0	0	0	0	0	0	0
Terriglobus	2	83	0	0	0	0	0	0	0	0	1	0	1	0	0	0	1	0	0	1	1	1
Tetragenococcus	1	12	0	0	0	0	0	0	0	0	0	0	0	0	0	0	0	0	0	0	0	0
Thalassiobium	1	11	0	0	0	0	0	0	0	0	0	0	0	0	0	1	0	0	0	0	0	0
Thalassobacter	1	9	0	0	0	0	0	0	0	0	0	0	0	0	0	1	0	0	0	0	0	0
Thalassolituus	1	12	0	0	0	0	0	0	0	0	0	0	0	0	0	0	0	0	0	0	0	0
Thalassospira	1	2	0	0	0	0	0	0	0	0	0	0	0	0	0	0	0	0	0	0	0	0
Thauera	1	111	0	0	0	0	1	0	0	0	1	0	1	1	0	1	1	0	1	0	0	0
Thermacetogenium	1	7	0	0	0	0	0	0	0	0	0	0	0	0	0	0	0	0	0	0	0	0
Thermaerobacter	2	79	0	0	0	0	0	0	0	0	1	0	1	1	0	1	1	0	0	1	1	0
Thermanaerovibrio	2	58	0	0	0	0	0	0	0	0	1	0	1	0	0	0	1	0	0	1	0	0
Thermincola	2	62	0	0	0	0	0	0	0	0	1	0	1	0	0	0	1	0	0	1	1	0
Thermoanaerobacter	2	76	0	0	0	0	0	1	0	0	1	0	1	0	0	1	1	0	1	1	1	0
Thermoanaerobacterium	2	64	0	0	0	0	0	0	0	0	1	0	1	0	0	1	1	0	0	1	1	0
Thermobacillus	1	11	0	0	0	0	0	0	0	0	0	0	0	0	0	0	0	0	0	0	0	0
Thermobaculum	2	62	0	0	0	0	0	0	0	0	1	0	1	1	0	0	1	0	0	1	1	0
Thermobifida	2	72	0	0	0	0	0	0	0	0	1	0	1	1	0	1	1	0	0	1	1	0
Thermobispora	2	109	0	0	0	0	0	0	0	0	1	0	1	1	0	1	1	0	1	1	1	1
Thermococcus	1	13	0	0	0	0	0	0	0	0	0	0	0	0	0	0	0	0	0	0	0	0
Thermocrinis	2	60	0	0	0	0	0	0	0	0	1	0	1	0	0	0	1	0	0	1	1	0
Thermodesulfatator	1	10	0	0	0	0	0	0	0	0	0	0	0	0	0	0	0	0	0	0	0	0
Thermodesulfobacterium	1	9	0	0	0	0	0	0	0	0	0	0	0	0	0	0	0	0	0	0	0	0

Genus	Cluster Number	Protein Counts	1.3.1.32	1.3.1.56	1.3.99.-	1.4.3.4	1.6.5.-	1.7.1.-	1.8.99.3	1.97.1.8	2.1.1.-	2.1.1.67	2.3.1.-	2.3.1.16	2.3.1.5	2.3.1.9	2.4.2.10	2.4.2.3	2.4.2.4	2.4.2.8	2.5.1.-	2.5.1.18
Thermodesulfobium	1	13	0	0	0	0	0	0	0	0	0	0	0	0	0	0	0	0	0	0	0	0
Thermodesulfovibrio	2	60	0	0	0	0	0	0	1	0	1	0	1	0	0	0	1	0	0	1	1	0
Thermofilum	1	9	0	0	0	0	0	0	0	0	0	0	0	0	0	0	0	0	0	0	0	0
Thermogladius	1	7	0	0	0	0	0	0	0	0	0	0	0	0	0	0	0	0	0	0	0	0
Thermomicrobium	2	92	0	0	0	0	0	0	0	0	1	0	1	1	0	0	1	0	0	1	1	0
Thermomonospora	2	125	0	0	0	0	0	0	0	0	1	0	1	1	0	1	1	0	1	1	1	1
Thermoplasma	1	11	0	0	0	0	0	0	0	0	0	0	0	0	0	0	0	0	0	0	0	0
Thermoplasmatales	1	6	0	0	0	0	0	0	0	0	0	0	0	0	0	0	0	0	0	0	0	0
Thermoproteus	1	12	0	0	0	0	0	0	0	0	0	0	0	0	0	0	0	0	0	0	0	0
Thermosediminibacter	2	64	0	0	0	0	0	0	0	0	1	0	1	0	0	0	0	0	0	1	1	0
Thermosipho	2	70	0	0	0	0	1	0	0	0	1	0	1	1	0	1	1	0	0	1	1	0
Thermosphaera	1	10	0	0	0	0	0	0	0	0	0	0	0	0	0	0	0	0	0	0	0	0
Thermosynechococcus	1	6	0	0	0	0	0	0	0	0	0	0	0	0	0	0	0	0	0	0	0	0
Thermotoga	2	80	0	0	0	0	1	0	0	0	1	0	1	0	0	1	1	1	0	1	1	0
Thermovibrio	2	58	0	0	0	0	0	0	0	0	1	0	1	0	0	0	1	0	1	1	1	0
Thermovirga	1	9	0	0	0	0	0	0	0	0	0	0	0	0	0	0	0	0	0	0	0	0
Thermus	2	107	0	0	0	0	0	0	0	0	1	0	0	1	0	1	1	0	0	1	1	0
Thioalkalimicrobium	1	4	0	0	0	0	0	0	0	0	0	0	0	0	0	0	0	0	0	0	0	0
Thioalkalivibrio	2	91	0	0	0	0	0	1	0	0	1	0	1	1	0	0	1	0	1	1	1	1
Thiobaca	1	2	0	0	0	0	0	0	0	0	0	0	0	0	0	0	0	0	0	0	0	0
Thiobacillus	2	67	0	0	0	0	0	1	1	0	1	0	1	0	0	0	1	0	1	1	1	1
Thiocapsa	1	2	0	0	0	0	0	0	0	0	0	0	0	0	0	0	0	0	0	0	0	0
Thiocystis	1	11	0	0	0	0	0	0	0	0	0	0	0	0	0	0	0	0	0	0	0	0
Thioflavicoccus	1	13	0	0	0	0	0	0	0	0	0	0	0	0	0	0	0	0	0	0	0	0
Thiomicrospira	2	50	0	0	0	0	0	1	0	0	1	1	1	0	0	0	1	0	0	0	0	0
Thiomonas	2	85	0	0	0	0	0	0	0	0	1	0	1	1	0	1	1	0	0	0	1	1
Thiorhodococcus	1	2	0	0	0	0	0	0	0	0	0	0	0	0	0	0	0	0	0	0	0	0
Thiothrix	1	2	0	0	0	0	0	0	0	0	0	0	0	0	0	0	0	0	0	0	0	0
Tistrella	1	42	0	0	0	0	0	0	0	0	0	0	1	0	0	0	0	0	0	0	0	0
Tolumonas	2	100	0	0	0	0	1	0	0	0	1	1	1	0	0	0	1	0	1	1	1	1
Transposon	1	2	0	0	0	0	0	0	0	0	0	0	0	0	0	0	0	0	0	0	0	0
Treponema	1	63	0	0	0	0	1	0	0	0	1	0	1	0	0	0	1	1	0	1	0	0
Trichodesmium	2	71	0	0	0	0	0	0	0	0	1	0	1	0	0	0	1	0	0	0	1	1
Trichormus	1	12	0	0	0	0	0	0	0	0	0	0	0	0	0	0	0	0	0	0	0	0
Tropheryma	2	22	0	0	0	0	0	0	0	0	1	0	1	0	0	0	1	0	0	1	1	0
Truepera	2	104	0	0	0	1	0	0	0	0	1	0	1	1	0	1	1	0	0	1	1	0
Tsukamurella	2	123	0	0	1	1	0	0	0	0	1	0	1	1	1	1	1	0	1	1	1	1
Turneriella	1	16	0	0	0	0	0	0	0	0	0	0	0	0	0	0	0	0	0	0	0	0
Uliginosibacterium	1	2	0	0	0	0	0	0	0	0	0	0	0	0	0	0	0	0	0	0	0	0
Ureaplasma	1	24	0	0	0	0	0	0	0	0	1	0	1	0	0	0	0	0	1	1	0	0
Ureibacillus	1	2	0	0	0	0	0	0	0	0	0	0	0	0	0	0	0	0	0	0	0	0
Variovorax	3	187	0	0	0	0	0	0	0	0	1	0	1	1	0	1	1	0	1	0	1	1
Veillonella	1	54	0	0	0	0	0	1	0	0	1	0	1	0	0	1	1	0	0	1	0	0
Verminephrobacter	3	188	0	0	0	0	1	1	0	0	1	0	1	1	0	1	1	0	1	0	1	1
Verrucosispora	3	126	0	0	0	0	0	0	0	0	1	0	1	1	0	1	1	0	1	1	1	1
Vibrio	2	195	0	0	0	0	1	1	0	0	1	1	1	1	0	1	1	1	1	1	1	1
Vibrionales	1	46	0	0	0	0	1	1	0	0	1	0	1	0	0	1	1	1	1	1	0	1
Vitreoscilla	1	1	0	0	0	0	0	0	0	0	0	0	0	0	0	0	0	0	0	0	0	0
Vulcanisaeta	1	14	0	0	0	0	0	0	0	0	0	0	0	0	0	0	0	0	0	0	0	0
Waddlia	2	68	0	0	0	0	1	0	0	0	1	0	1	1	0	1	1	0	0	0	1	0
Wautersia	1	2	0	0	0	0	0	0	0	0	0	0	0	0	0	0	0	0	0	0	0	0
Weeksella	2	65	0	0	0	0	0	0	0	0	1	0	1	0	0	1	1	0	0	1	1	0

Genus	Cluster Number	Protein Counts	1.3.1.32	1.3.1.56	1.3.99.-	1.4.3.4	1.6.5.-	1.7.1.-	1.8.99.3	1.97.1.8	2.1.1.-	2.1.1.67	2.3.1.-	2.3.1.16	2.3.1.5	2.3.1.9	2.4.2.10	2.4.2.3	2.4.2.4	2.4.2.8	2.5.1.-	2.5.1.18
**Weissella**	1	14	0	0	0	0	0	0	0	0	0	0	0	0	0	0	0	0	0	0	0	0
**Wenxinia**	1	2	0	0	0	0	0	0	0	0	0	0	0	0	0	0	0	0	0	0	0	0
**Wigglesworthia**	1	29	0	0	0	0	0	0	0	0	1	0	1	0	0	0	1	0	0	0	1	0
**Wolbachia**	1	32	0	0	0	0	0	0	0	0	1	0	0	0	0	0	1	0	0	0	1	0
**Wolinella**	2	51	0	0	0	0	0	1	0	0	1	0	1	0	0	0	1	0	0	0	0	0
**Xanthobacter**	3	176	0	0	0	0	0	0	0	0	1	1	1	1	0	1	1	1	0	1	1	1
**Xanthobacteraceae**	1	4	0	0	0	0	0	0	0	0	0	0	0	0	0	0	0	0	0	0	0	0
**Xanthomonadaceae**	1	39	0	0	0	0	0	0	0	0	1	0	1	1	0	0	1	0	0	1	1	1
**Xanthomonas**	2	162	0	0	0	0	0	1	0	0	1	1	1	1	0	1	1	0	1	1	1	1
**Xenorhabdus**	2	127	0	0	0	0	1	0	0	0	1	0	1	1	0	1	1	1	1	1	1	1
**Xylanimonas**	2	66	0	0	0	0	0	0	0	0	1	0	1	1	0	1	1	0	1	1	1	0
**Xylella**	2	52	0	0	0	0	0	1	0	0	1	0	1	0	0	0	1	0	1	1	1	1
**Yersinia**	2	145	0	0	1	0	1	1	0	0	1	0	1	1	0	1	1	1	1	1	1	1
**Yokenella**	2	61	0	0	1	0	1	0	0	0	1	0	1	0	0	1	1	1	1	1	1	1
**Zobellia**	1	28	0	0	0	0	1	0	0	0	0	0	0	0	0	1	0	0	0	0	0	0
**Zunongwangia**	2	98	0	0	0	0	1	0	0	0	1	0	1	1	0	1	1	0	1	1	1	0
**Zymomonas**	2	67	0	0	0	0	0	1	0	0	1	0	1	0	0	0	1	0	0	0	1	1
**Zymophilus**	1	2	0	0	0	0	0	0	0	0	0	0	0	0	0	0	0	0	0	0	0	0

Genus	Cluster Number	Protein Counts	2.6.1.-	2.7.1.21	2.7.1.48	2.7.4.-	2.8.3.-	2.8.3.1	2.8.3.12	2.8.3.15	2.8.3.6	2.8.3.8	3.1.1.-	3.1.1.1	3.1.1.17	3.1.1.2	3.1.1.24	3.1.1.45	3.1.2.-	3.1.2.23	3.1.3.1	3.1.3.2
Acaricomes	1	2	0	0	0	0	0	0	0	0	0	0	0	0	0	0	0	0	0	0	0	0
Acaryochloris	2	88	1	0	1	1	0	0	0	0	0	0	1	1	0	0	1	0	0	0	1	0
Acetivibrio	1	2	0	0	0	0	0	0	0	0	0	0	0	0	0	0	0	0	0	0	0	0
Acetobacter	2	85	0	0	0	1	0	1	0	0	0	0	0	0	1	0	0	0	0	0	0	0
Acetobacteraceae	1	2	0	0	0	0	0	0	0	0	0	0	0	0	0	0	0	0	0	0	0	0
Acetobacterium	1	8	0	0	0	0	0	0	0	0	0	0	0	0	0	0	0	0	0	0	0	0
Acetohalobium	2	79	0	0	0	1	0	0	0	0	0	0	1	0	0	0	0	0	0	0	0	0
Acholeplasma	2	38	0	1	1	1	0	0	0	0	0	0	0	1	0	0	0	0	0	0	0	0
Achromobacter	2	192	0	0	0	1	0	0	0	0	1	0	1	0	1	1	1	0	0	1	1	0
Acidaminococcus	1	65	0	0	1	1	0	0	1	0	0	0	0	0	0	0	0	0	0	0	1	0
Acidianus	1	9	0	0	0	0	0	0	0	0	0	0	0	0	0	0	0	0	0	0	0	0
Acidilobus	1	8	0	0	0	0	0	0	0	0	0	0	0	0	0	0	0	0	0	0	0	0
Acidimicrobium	2	58	0	0	1	1	0	0	0	0	0	0	0	0	0	0	0	0	1	0	0	0
Acidiphilium	3	144	0	0	0	1	0	0	0	0	1	0	1	0	1	0	1	0	0	0	0	1
Acidithiobacillus	2	70	0	0	0	1	0	0	0	0	0	0	1	0	0	0	0	0	0	0	0	0
Acidobacterium	2	91	1	0	1	1	0	0	0	0	0	0	1	0	1	1	1	0	0	0	0	1
Acidocella	1	2	0	0	0	0	0	0	0	0	0	0	0	0	0	0	0	0	0	0	0	0
Acidothermus	2	68	0	0	0	1	0	0	1	0	0	0	0	0	1	0	1	0	0	0	0	1
Acidovorax	3	217	0	0	0	1	0	0	0	0	1	0	1	1	1	1	1	0	0	1	1	0
Aciduliprofundum	1	11	0	0	0	0	0	0	0	0	0	0	0	0	0	0	0	0	0	0	0	0
Acinetobacter	3	188	0	0	0	1	0	0	0	0	1	1	0	0	0	1	1	0	1	0	1	0
Actinobacillus	2	112	0	1	1	1	0	0	0	0	0	0	1	0	1	0	0	0	1	0	1	1
Actinomadura	1	1	0	0	0	0	0	0	0	0	0	0	0	0	0	0	0	0	0	0	0	0
Actinoplanes	1	52	0	0	0	0	0	0	0	0	0	0	0	0	0	0	0	0	0	0	0	0
Actinopolyspora	1	2	0	0	0	0	0	0	0	0	0	0	0	0	0	0	0	0	0	0	0	0
Actinosynnema	2	122	0	1	1	1	0	0	1	0	1	0	0	0	1	0	1	0	1	0	1	0
Advenella	1	30	0	0	0	0	0	0	0	0	0	0	0	0	0	0	0	0	0	0	0	0
Aequorivita	1	12	0	0	0	0	0	0	0	0	0	0	0	0	0	0	0	0	0	0	0	0
Aerococcus	2	57	0	1	0	1	0	0	0	0	0	1	0	1	0	0	0	0	0	0	0	0
Aeromonas	2	127	1	1	1	1	0	0	0	0	0	0	1	0	0	1	1	0	1	0	1	1
Aeropyrum	1	13	0	0	0	0	0	0	0	0	0	0	0	0	0	0	0	0	0	0	0	0
Afipia	1	54	0	0	0	1	0	0	1	0	0	0	0	0	0	0	0	0	0	0	0	0
Aggregatibacter	1	81	0	1	1	1	0	0	0	0	0	0	1	0	0	0	0	0	1	0	0	1
Agrobacterium	2	242	0	1	1	1	0	0	0	0	1	1	1	1	1	1	1	0	1	0	1	1
Ahrensia	1	2	0	0	0	0	0	0	0	0	0	0	0	0	0	0	0	0	0	0	0	0
Akkermansia	2	47	0	0	0	1	0	0	0	0	0	0	1	0	0	0	0	0	0	0	1	0
Albidiferax	3	144	1	1	0	1	0	0	0	0	1	0	1	1	1	1	1	0	1	0	1	0
Alcaligenes	1	21	0	0	0	1	0	0	0	0	0	0	0	0	0	0	0	0	0	0	0	0
Alcanivorax	2	146	0	0	0	1	0	0	0	0	0	0	0	0	0	1	0	0	1	1	1	0
Alicycliphilus	3	152	0	0	0	1	0	0	0	0	1	0	1	1	0	1	1	0	0	0	1	0
Alicyclobacillus	2	91	1	1	0	1	0	0	0	0	0	0	1	0	0	0	0	0	0	0	0	0
Aliivibrio	2	118	0	1	1	1	0	0	0	0	0	0	1	0	0	1	0	0	1	0	1	0
Alishewanella	1	1	0	0	0	0	0	0	0	0	0	0	0	0	0	0	0	0	0	0	0	0
Alistipes	1	52	0	1	1	1	0	0	0	0	0	0	0	1	0	0	0	0	0	1	0	0
Alkalilimnicola	2	97	1	0	0	1	0	0	0	0	0	0	0	0	0	1	1	0	0	0	0	0
Alkaliphilus	2	62	0	1	1	1	0	0	0	0	0	1	0	0	0	0	0	0	0	0	1	0
Allochromatium	2	84	1	0	0	1	0	0	0	0	0	0	1	0	0	1	0	0	0	0	1	0
Alphaproteobacteria	1	3	0	0	0	0	0	0	0	0	0	0	0	0	0	0	0	0	0	0	0	0
Alteromonadales	1	12	0	0	1	0	0	0	0	0	0	0	0	0	0	0	0	0	0	0	0	0
Alteromonas	1	37	0	0	0	0	0	0	0	0	0	0	0	0	0	0	0	0	0	0	1	0
Aminobacter	1	4	0	0	0	0	0	0	0	0	0	0	0	0	0	0	0	0	0	0	0	0
Aminobacterium	2	59	1	0	1	1	0	0	0	0	0	0	1	0	0	0	0	0	0	0	1	0

Genus	Cluster Number	Protein Counts	2.6.1.-	2.7.1.21	2.7.1.48	2.7.4.-	2.8.3.-	2.8.3.1	2.8.3.12	2.8.3.15	2.8.3.6	2.8.3.8	3.1.1.-	3.1.1.1	3.1.1.17	3.1.1.2	3.1.1.24	3.1.1.45	3.1.2.-	3.1.2.23	3.1.3.1	3.1.3.2
Ammonifex	2	50	0	0	1	1	0	0	0	0	0	0	0	0	0	0	0	0	0	0	0	0
Amphibacillus	1	13	0	0	0	0	0	0	0	0	0	0	0	0	0	0	0	0	0	0	0	0
Amphritea	1	2	0	0	0	0	0	0	0	0	0	0	0	0	0	0	0	0	0	0	0	0
Amycolatopsis	2	201	0	1	1	1	1	0	1	0	0	0	1	0	1	0	1	0	1	0	1	1
Amycolicicoccus	1	43	0	0	0	0	0	0	0	0	0	0	0	0	0	0	0	0	0	0	0	0
Anabaena	2	92	0	0	1	1	0	0	0	0	0	0	0	0	0	1	0	0	0	0	1	1
Anaerobaculum	1	11	0	0	0	0	0	0	0	0	0	0	0	0	0	0	0	0	0	0	0	0
Anaerococcus	2	46	1	1	1	1	0	0	0	0	0	0	0	0	0	0	0	0	0	0	0	0
Anaerolinea	2	66	1	1	1	1	0	0	1	0	0	0	1	0	0	0	1	0	0	1	1	0
Anaeromyxobacter	3	117	0	0	1	1	0	0	0	0	1	0	0	1	0	0	1	0	0	0	1	1
Anaerostipes	1	44	0	0	1	1	0	0	0	0	0	0	0	0	0	0	0	0	0	0	0	0
Anaerotruncus	1	2	0	0	0	0	0	0	0	0	0	0	0	0	0	0	0	0	0	0	0	0
Anaplasma	1	35	0	0	0	1	0	0	0	0	0	0	1	0	0	0	0	0	0	0	0	0
Ancylobacter	1	3	0	0	0	0	0	0	0	0	0	0	0	0	0	0	0	0	0	0	0	0
Aneurinibacillus	1	1	0	0	0	0	0	0	0	0	0	0	0	0	0	0	0	0	0	0	0	0
Angomonas	1	1	0	0	0	0	0	0	0	0	0	0	0	0	0	0	0	0	0	0	0	0
Anoxybacillus	2	72	1	1	1	1	0	0	0	0	0	0	1	1	0	0	0	0	0	0	0	0
Aquifex	2	45	0	0	0	1	0	0	0	0	0	0	0	0	0	0	0	0	0	0	0	0
Aquimarina	1	2	0	0	0	0	0	0	0	0	0	0	0	0	0	0	0	0	0	0	0	0
Arcanobacterium	2	45	0	1	1	1	1	0	0	0	0	0	1	0	0	0	1	0	1	0	0	0
Archaeoglobus	1	14	0	0	0	0	0	0	0	0	0	0	0	0	0	0	0	0	0	0	0	0
Arcobacter	2	101	1	0	0	1	0	0	0	0	0	0	1	0	0	0	1	0	0	0	1	0
Aromatoleum	3	133	0	0	0	1	0	0	0	1	0	0	1	0	0	1	1	0	0	1	1	0
Arsenophonus	1	4	0	0	0	1	0	0	0	0	0	0	0	0	0	0	0	0	0	0	0	0
Arthrobacter	2	206	1	1	1	1	0	1	0	0	1	0	1	0	1	0	1	0	1	1	1	0
Arthrospira	2	67	0	0	1	1	0	0	0	0	0	0	0	0	0	0	0	0	0	0	1	0
Aster	1	10	0	1	1	1	0	0	0	0	0	0	0	0	0	0	0	0	0	0	0	0
Asticcacaulis	2	103	0	1	0	1	0	0	0	0	0	0	1	0	1	0	0	0	0	0	1	0
Atopobium	1	28	0	0	0	1	0	0	0	0	0	0	1	0	0	0	0	0	0	0	0	0
Aurantimonas	1	2	0	0	0	0	0	0	0	0	0	0	0	0	0	0	0	0	0	0	0	0
Avibacterium	1	17	0	0	0	1	0	0	0	0	0	0	1	0	0	0	0	0	0	0	0	0
Azoarcus	3	172	0	0	0	1	0	0	0	0	0	0	1	0	1	1	0	0	0	1	1	0
Azorhizobium	3	149	0	0	0	1	0	0	0	0	1	0	1	1	1	1	1	0	1	0	1	1
Azorhizophilus	1	4	0	0	0	0	0	0	0	0	0	0	0	0	0	0	0	0	0	0	0	0
Azospira	1	19	0	0	0	0	0	0	0	0	0	0	0	0	0	0	0	0	0	0	0	0
Azospirillum	2	198	1	0	0	1	0	0	1	0	0	1	1	0	1	1	1	0	0	1	1	0
Azotobacter	3	183	0	0	0	1	0	0	1	0	0	1	0	0	1	1	1	0	1	0	1	0
Bacillales	1	9	0	0	0	0	0	0	0	0	0	0	0	0	0	0	0	0	0	0	0	0
Bacilli	1	3	0	0	0	0	0	0	0	0	0	0	0	0	0	0	0	0	0	0	0	0
Bacillus	2	237	1	1	1	1	0	0	0	0	0	1	1	1	1	1	1	0	0	0	1	1
Bacteria	2	86	0	0	0	1	0	0	0	0	1	0	1	1	0	1	1	0	0	1	0	0
Bacteriovorax	2	98	0	1	1	1	0	0	0	0	0	0	1	1	0	1	0	0	0	0	1	1
Bacteroidales	2	59	0	1	1	1	0	0	0	0	0	0	0	1	1	0	0	0	0	0	1	0
Bacteroides	2	96	1	1	1	1	0	0	0	0	0	0	0	1	1	0	0	0	0	1	1	1
Bacteroidetes	1	2	0	0	0	0	0	0	0	0	0	0	0	0	0	0	0	0	0	0	0	0
Bartonella	2	50	0	0	0	1	0	0	0	0	0	0	1	0	0	0	0	0	1	0	0	0
Baumannia	2	24	0	0	0	1	0	0	0	0	0	0	0	0	0	0	0	0	0	0	0	0
Bdellovibrio	2	87	0	1	1	1	0	0	0	0	0	0	1	1	0	1	1	0	0	0	0	0
Beijerinckia	3	123	0	0	1	1	0	0	0	0	0	0	1	0	1	1	1	0	1	0	1	1
Belliella	1	14	0	0	0	0	0	0	0	0	0	0	0	0	0	0	0	0	0	0	0	0
Beutenbergia	2	82	0	1	1	1	0	0	0	0	0	0	0	1	1	0	1	0	1	0	1	0
Bifidobacterium	2	61	0	0	1	1	0	0	0	0	0	0	0	0	0	0	0	0	1	0	1	0

Genus	Cluster Number	Protein Counts	2.6.1.-	2.7.1.21	2.7.1.48	2.7.4.-	2.8.3.-	2.8.3.1	2.8.3.12	2.8.3.15	2.8.3.6	2.8.3.8	3.1.1.-	3.1.1.1	3.1.1.17	3.1.1.2	3.1.1.24	3.1.1.45	3.1.2.-	3.1.2.23	3.1.3.1	3.1.3.2
**Bizionia**	1	1	0	0	0	0	0	0	0	0	0	0	0	0	0	0	0	0	0	0	0	0
**Blastococcus**	1	39	0	0	0	0	0	0	0	0	0	0	0	0	0	0	0	0	0	0	0	0
**Blattabacterium**	1	23	0	0	0	1	0	0	0	0	0	0	0	0	0	0	0	0	0	0	0	0
**Blautia**	1	22	0	0	0	0	0	0	0	0	0	0	1	0	0	0	0	0	0	0	0	0
**Bordetella**	3	227	1	0	0	1	0	0	1	0	1	0	1	0	1	1	1	1	0	1	0	0
**Borrelia**	1	32	0	1	1	1	0	0	0	0	0	0	0	0	0	0	0	0	0	0	0	0
**Brachybacterium**	1	18	0	0	0	0	0	0	0	0	0	0	0	0	0	0	0	0	0	0	0	0
**Brachymonas**	1	4	0	0	0	0	0	0	0	0	0	0	0	0	0	0	0	0	0	0	0	0
**Brachyspira**	2	79	1	0	0	1	0	0	0	0	0	1	1	0	0	0	0	0	0	0	1	0
**Bradyrhizobiaceae**	1	20	0	0	0	0	0	0	0	0	0	0	0	0	0	0	0	0	0	0	0	0
**Bradyrhizobium**	3	254	0	0	1	1	0	0	0	0	0	0	1	1	1	1	0	0	1	0	0	0
**Brenneria**	1	20	0	0	1	1	0	0	0	0	0	0	1	0	0	0	0	0	0	0	0	0
**Brevibacillus**	2	122	0	1	1	1	0	0	0	0	0	1	1	1	0	0	1	0	0	0	1	0
**Brevibacterium**	1	4	0	0	0	0	0	0	0	0	0	0	0	0	0	0	0	0	0	0	0	0
**Brevundimonas**	2	105	0	0	1	1	0	0	0	0	1	0	1	0	0	1	1	0	0	0	1	0
**Brucella**	3	138	0	0	0	1	0	1	0	0	1	0	1	1	1	1	1	0	1	0	1	0
**Buchnera**	1	26	0	0	0	1	0	0	0	0	0	0	0	0	0	0	0	0	0	0	0	0
**Burkholderia**	3	424	1	0	0	1	0	0	1	0	1	1	1	1	1	1	1	1	1	1	1	1
**Burkholderiaceae**	3	143	0	0	0	1	0	0	0	0	1	1	1	0	1	0	1	0	0	1	0	0
**Burkholderiales**	1	24	0	0	0	1	0	0	0	0	0	0	0	0	0	0	0	1	0	0	0	0
**Buttiauxella**	1	2	0	0	0	0	0	0	0	0	0	0	0	0	0	0	0	0	0	0	0	0
**Butyrivibrio**	2	67	1	1	1	1	0	0	0	0	0	0	1	0	0	0	1	0	0	0	0	0
**Caldanaerobacter**	2	63	1	1	1	1	0	0	0	0	0	0	0	0	0	0	0	0	0	0	0	0
**Caldicellulosiruptor**	2	75	0	0	1	1	0	0	0	0	0	0	0	0	0	0	0	0	0	0	1	0
**Caldilinea**	1	23	0	0	0	0	0	0	0	0	0	0	0	0	0	0	0	0	0	0	0	0
**Caldimonas**	1	6	0	0	0	1	0	0	0	0	0	1	0	0	0	0	0	0	0	0	0	0
**Caldisericum**	1	10	0	0	0	0	0	0	0	0	0	0	0	0	0	0	0	0	0	0	0	0
**Caldisphaera**	1	7	0	0	0	0	0	0	0	0	0	0	0	0	0	0	0	0	0	0	0	0
**Calditerrivibrio**	2	74	1	0	0	1	0	0	0	0	0	0	1	0	0	0	1	0	0	0	1	0
**Caldivirga**	1	13	0	0	0	0	0	0	0	0	0	0	0	0	0	0	0	0	0	0	0	0
**Calothrix**	1	43	0	0	1	1	0	0	0	0	0	0	0	0	0	0	0	0	0	0	0	0
**Calyptogena**	2	34	0	0	0	1	0	0	0	0	0	0	0	0	0	0	0	0	0	0	0	0
**Campylobacter**	2	80	1	1	0	1	0	0	0	0	0	0	1	0	0	0	0	0	1	0	1	0
**Candidatus**	2	290	1	1	1	1	0	0	1	0	1	1	1	0	1	1	1	1	0	0	1	1
**Capnocytophaga**	1	50	0	1	1	1	0	0	0	0	0	0	0	0	0	0	0	0	0	0	1	1
**Carbophilus**	1	2	0	0	0	0	0	0	0	0	0	0	0	0	0	0	0	0	0	0	0	0
**Carboxydibrachium**	1	14	0	0	1	1	0	0	0	0	0	0	0	0	0	0	0	0	0	0	0	0
**Carboxydothermus**	2	68	0	1	0	1	0	0	0	0	0	0	0	0	0	0	0	0	0	0	0	0
**Carica**	1	2	0	0	0	0	0	0	0	0	0	0	0	0	0	0	0	0	0	0	0	0
**Carnobacterium**	2	71	1	1	1	1	0	0	0	0	0	0	0	1	0	0	0	0	0	0	0	0
**Catenibacterium**	1	2	0	0	0	0	0	0	0	0	0	0	0	0	0	0	0	0	0	0	0	0
**Catenovulum**	1	2	0	0	0	0	0	0	0	0	0	0	0	0	0	0	0	0	0	0	0	0
**Catenulispora**	3	159	1	1	1	1	1	0	1	0	1	0	0	0	0	0	1	1	1	0	0	1
**Caulobacter**	3	168	0	0	0	1	0	0	1	0	1	0	1	0	1	1	1	0	1	0	1	0
**Cedecea**	1	45	0	0	1	1	1	0	0	0	0	0	1	0	0	0	0	0	1	0	0	0
**Cellulomonas**	2	96	0	1	1	1	0	0	0	0	0	0	1	0	0	0	0	0	1	0	1	0
**Cellulophaga**	2	105	0	1	1	1	0	0	0	0	0	0	0	1	1	1	0	0	0	0	1	0
**Cellulosilyticum**	1	14	0	0	0	0	0	0	0	0	0	0	0	0	0	0	0	0	0	0	0	0
**Cellvibrio**	2	85	1	0	0	1	0	0	0	0	0	0	1	0	1	1	1	0	0	0	1	0
**Cenarchaeum**	1	7	0	0	0	0	0	0	0	0	0	0	0	0	0	0	0	0	0	0	0	0
**Chamaesiphon**	1	13	0	0	0	0	0	0	0	0	0	0	0	0	0	0	0	0	0	0	0	0
**Chelativorans**	3	136	0	0	0	1	0	0	1	0	1	0	1	1	1	1	1	0	1	0	1	0

Genus	Cluster Number	Protein Counts	2.6.1.-	2.7.1.21	2.7.1.48	2.7.4.-	2.8.3.-	2.8.3.1	2.8.3.12	2.8.3.15	2.8.3.6	2.8.3.8	3.1.1.-	3.1.1.1	3.1.1.17	3.1.1.2	3.1.1.24	3.1.1.45	3.1.2.-	3.1.2.23	3.1.3.1	3.1.3.2
Chelatococcus	1	2	0	0	0	0	0	0	0	0	0	0	0	0	0	0	0	0	0	0	0	0
Chitinophaga	2	98	0	1	0	1	0	0	0	0	0	0	1	0	1	0	0	0	0	0	1	1
Chlamydia	1	36	0	0	1	1	0	0	0	0	0	0	0	0	0	0	0	0	0	0	0	0
Chlamydomonas	1	3	0	0	0	0	0	0	0	0	0	0	1	0	0	0	0	0	0	0	0	0
Chlamydophila	1	31	0	0	1	1	0	0	0	0	0	0	0	0	0	0	0	0	0	0	0	0
Chlorobaculum	2	61	0	0	1	1	0	0	0	0	0	0	1	1	0	0	1	0	0	0	1	0
Chlorobium	2	89	1	0	1	1	0	0	0	0	0	0	1	1	0	0	1	0	0	0	1	1
Chlorobium/Pelodictyon	1	2	0	0	0	0	0	0	0	0	0	0	0	0	0	0	0	0	0	0	0	0
Chloroflexus	2	105	1	1	1	1	0	0	0	0	0	0	1	1	0	0	1	0	0	0	1	0
Chlorogloeopsis	1	9	0	0	0	1	0	0	0	0	0	0	0	0	0	0	0	0	0	0	0	0
Chloroherpeton	2	59	0	1	0	1	0	0	0	0	0	0	1	0	0	1	1	0	0	1	1	0
Chromobacterium	2	117	1	1	1	1	0	0	0	0	0	0	1	0	0	1	0	0	0	0	1	0
Chromohalobacter	2	145	0	0	0	1	0	0	0	0	1	0	1	0	1	1	1	0	1	0	0	0
Chroococcidiopsis	1	24	0	0	0	0	0	0	0	0	0	0	0	0	0	0	0	0	0	0	0	0
Chryseobacterium	2	104	0	0	0	0	0	0	0	0	0	0	1	0	0	1	1	0	0	0	1	0
Citreicella	1	13	0	0	0	0	0	0	0	0	0	0	0	0	0	0	0	0	0	0	0	0
Citrobacter	2	190	0	1	1	1	1	0	0	0	1	1	1	0	0	1	1	1	1	0	1	1
Clavibacter	2	83	0	1	1	1	0	0	0	0	0	0	1	1	1	0	1	1	1	0	0	0
Clonorchis	1	1	0	0	0	0	0	0	0	0	0	0	0	0	0	0	0	0	0	0	0	0
Clostridiales	2	77	0	1	1	1	0	0	0	0	0	1	1	0	0	0	0	0	0	0	0	0
Clostridium	2	191	1	1	1	1	1	0	0	0	1	1	1	0	0	1	1	0	0	0	1	1
Cohnella	1	2	0	0	0	1	0	0	0	0	0	0	0	0	0	0	0	0	0	0	0	0
Coleofasciculus	1	4	0	0	0	0	0	0	0	0	0	0	0	0	0	0	0	0	0	0	0	0
Collimonas	1	35	0	0	0	1	0	0	0	0	0	0	0	0	0	0	0	0	0	0	0	0
Colwellia	2	130	0	1	1	1	0	0	0	0	1	0	1	0	0	1	0	0	1	0	1	1
Comamonas	2	196	0	0	0	1	0	0	1	0	1	0	1	1	0	1	0	0	0	0	1	0
Conexibacter	3	117	0	0	0	1	1	0	1	0	0	0	1	1	1	0	1	0	0	0	1	0
Coprobacillus	1	2	0	0	0	0	0	0	0	0	0	0	0	0	0	0	0	0	0	0	0	0
Coprococcus	2	76	0	0	1	1	0	0	0	0	0	1	1	0	0	0	1	0	0	0	0	0
Coprothermobacter	2	45	0	1	1	1	0	0	0	0	0	0	0	0	0	0	0	0	0	0	0	0
Coraliomargarita	2	68	0	0	0	1	0	0	0	0	0	0	0	0	1	1	0	0	0	0	1	0
Corallococcus	1	29	0	0	1	0	0	0	0	0	0	0	0	0	0	0	0	0	0	0	0	0
Coriobacterium	2	36	0	0	0	1	0	0	0	0	0	0	1	0	0	0	0	0	0	0	0	0
Corynebacterineae	1	5	0	0	0	0	0	0	0	0	0	0	0	0	0	0	0	0	0	0	0	0
Corynebacterium	2	175	0	0	0	1	0	0	0	0	1	0	1	1	0	0	1	0	1	1	1	0
Coxiella	2	54	1	1	1	1	0	0	0	0	0	0	1	0	0	0	0	0	0	0	0	0
Crinalium	1	21	0	0	0	0	0	0	0	0	0	0	0	0	0	0	0	0	0	0	0	0
Croceibacter	2	80	0	1	1	1	0	0	0	0	0	0	0	1	0	0	0	0	0	0	1	0
Crocosphaera	1	6	0	0	0	0	0	0	0	0	0	0	0	0	0	0	0	0	0	0	0	0
Cronobacter	2	120	1	1	1	1	0	0	0	0	0	0	1	0	0	1	0	1	1	0	0	1
Cryptobacterium	2	39	1	0	0	1	0	0	0	0	0	0	0	0	0	0	1	0	0	0	0	0
Cucumis	1	9	0	0	1	0	0	0	0	0	0	0	0	0	0	0	0	0	0	0	0	0
Cupriavidus	3	317	1	0	0	1	1	0	1	0	1	1	1	0	1	1	1	0	0	1	1	1
Curtobacterium	1	5	0	0	0	0	0	0	0	0	0	0	0	0	0	0	0	0	0	0	0	0
Curvibacter	1	27	0	0	0	0	0	0	0	0	0	0	0	0	0	0	0	0	0	0	0	0
Cyanobacterium	1	9	0	0	0	0	0	0	0	0	0	0	0	0	0	0	0	0	0	0	0	0
Cyanobium	1	10	0	0	0	0	0	0	0	0	0	0	0	0	0	0	0	0	0	0	0	0
Cyanothece	1	34	0	0	0	1	0	0	0	0	0	0	0	0	0	0	0	0	0	0	0	0
Cyclobacterium	1	19	0	0	0	0	0	0	0	0	0	0	0	0	0	0	0	0	0	0	0	0
Cycloclasticus	1	21	0	0	0	0	0	0	0	0	0	0	0	0	0	0	0	0	0	0	0	0
Cylindrospermopsis	1	4	0	0	0	1	0	0	0	0	0	0	0	0	0	0	0	0	0	0	0	0
Cylindrospermum	1	30	0	0	1	1	0	0	0	0	0	0	0	0	0	0	0	0	0	0	0	0

Genus	Cluster Number	Protein Counts	2.6.1.-	2.7.1.21	2.7.1.48	2.7.4.-	2.8.3.-	2.8.3.1	2.8.3.12	2.8.3.15	2.8.3.6	2.8.3.8	3.1.1.-	3.1.1.1	3.1.1.17	3.1.1.2	3.1.1.24	3.1.1.45	3.1.2.-	3.1.2.23	3.1.3.1	3.1.3.2
Cytophaga	2	69	1	0	1	1	0	0	0	0	0	0	1	0	1	0	1	0	0	0	1	0
Dactylococcopsis	1	8	0	0	0	0	0	0	0	0	0	0	0	0	0	0	0	0	0	0	0	0
Dechloromonas	1	40	0	0	0	0	0	0	0	0	0	0	0	0	0	0	0	0	0	0	0	0
Dechlorosoma	1	19	0	0	0	0	0	0	0	0	0	0	0	0	0	0	0	0	0	0	0	0
Deferribacter	2	73	1	0	0	1	0	0	0	0	0	1	1	1	0	0	0	0	0	0	1	0
Dehalobacter	1	9	0	0	0	0	0	0	0	0	0	0	0	0	0	0	0	0	0	0	0	0
Dehalococcoides	2	41	0	0	0	1	0	0	0	0	0	0	1	0	0	0	1	0	0	0	0	0
Dehalogenimonas	1	37	0	0	0	1	0	0	0	0	0	0	0	0	0	0	0	0	0	0	1	0
Deinococcus	2	140	1	1	1	1	0	0	0	0	1	1	0	1	1	0	1	0	0	1	1	0
Delftia	3	177	0	0	0	1	0	0	0	0	1	0	1	1	1	1	1	0	0	1	1	0
Denitrovibrio	2	67	0	0	0	1	0	0	0	0	0	0	1	1	0	0	0	0	0	0	1	0
Desulfarculus	2	92	1	0	0	1	0	0	0	0	0	0	1	0	0	0	0	0	0	0	1	0
Desulfatibacillum	3	84	0	0	0	1	0	0	1	0	1	0	0	0	0	0	1	0	0	0	0	0
Desulfitobacterium	2	102	1	0	1	1	1	0	1	0	1	0	0	0	0	0	1	0	0	0	1	0
Desulfobacca	2	65	1	0	0	1	0	0	0	0	0	0	1	0	0	0	0	0	0	0	1	0
Desulfobacterium	2	113	0	0	0	1	0	0	1	0	0	0	1	0	0	1	1	0	1	0	1	0
Desulfobacula	1	21	0	0	0	0	0	0	0	0	0	0	0	0	0	0	0	0	0	0	0	0
Desulfobulbus	2	88	0	0	0	1	0	0	1	0	1	0	1	0	0	1	1	0	0	0	1	0
Desulfocapsa	1	9	0	0	0	0	0	0	0	0	0	0	0	0	0	0	0	0	0	0	0	0
Desulfococcus	2	80	0	0	0	1	0	0	0	0	0	0	1	0	0	0	1	0	0	0	0	0
Desulfohalobium	2	52	0	0	0	1	0	0	0	0	0	0	1	0	0	0	1	0	0	0	1	0
Desulfomicrobium	2	65	0	0	0	1	0	0	0	0	0	0	1	0	0	1	0	0	0	0	1	0
Desulfomonile	1	25	0	0	0	0	0	0	0	0	0	0	0	0	0	0	0	0	0	0	0	0
Desulforhabdus	1	2	0	0	0	0	0	0	0	0	0	0	0	0	0	0	0	0	0	0	0	0
Desulfosporosinus	1	30	0	0	0	0	0	0	0	0	0	0	0	0	0	0	0	0	0	0	0	0
Desulfotalea	2	66	0	0	0	1	0	0	0	0	0	0	0	0	0	0	0	0	0	0	0	0
Desulfotomaculum	2	98	0	0	1	1	1	0	1	0	1	0	0	1	0	0	0	0	0	0	0	0
Desulfovibrio	2	144	1	0	1	1	0	0	1	0	0	0	1	0	0	1	1	0	1	0	1	1
Desulfurispirillum	2	78	0	0	0	1	0	0	0	0	0	0	1	0	0	1	0	0	0	0	1	0
Desulfurivibrio	2	71	1	0	0	1	0	0	0	0	0	0	1	0	0	0	0	0	0	0	0	0
Desulfurobacterium	2	53	0	0	0	1	0	0	0	0	0	0	0	0	0	0	0	0	0	0	0	0
Desulfurococcus	1	11	0	0	0	0	0	0	0	0	0	0	0	0	0	0	0	0	0	0	0	0
Diaphorobacter	1	16	0	0	0	0	0	0	0	0	0	0	0	0	0	0	0	0	0	0	0	0
Dichelobacter	2	45	0	0	0	1	0	0	0	0	0	0	0	0	0	0	1	0	0	0	1	0
Dickeya	2	155	1	1	1	1	0	0	0	0	0	1	1	0	0	1	1	1	1	0	1	0
Dictyoglomus	2	62	0	1	1	1	0	0	0	0	0	0	1	0	0	0	0	0	0	0	1	0
Dinoroseobacter	3	148	0	1	0	1	0	0	0	0	0	1	1	0	0	1	1	0	1	0	1	0
Dokdonia	1	3	0	0	0	0	0	0	0	0	0	0	0	0	0	0	0	0	0	0	0	0
Dorea	1	7	0	0	0	0	0	0	0	0	0	0	0	0	0	0	0	0	0	0	0	0
Dyadobacter	2	83	1	1	1	1	0	0	0	0	0	0	1	0	1	1	1	0	0	0	1	1
Echinicola	1	20	0	0	0	0	0	0	0	0	0	0	0	0	0	0	0	0	0	0	0	0
Edwardsiella	2	126	1	1	1	1	1	0	0	0	0	1	1	0	0	1	1	0	1	0	1	1
Eggerthella	1	51	1	0	0	1	1	0	0	0	0	0	1	0	0	0	0	0	0	0	0	0
Ehrlichia	2	37	0	0	0	1	0	0	0	0	0	0	1	0	0	0	0	0	0	0	0	0
Eikenella	1	4	0	0	0	0	0	0	0	0	0	0	0	0	0	0	0	0	0	0	0	0
Elusimicrobium	2	44	1	1	0	1	0	0	0	0	0	0	1	0	0	0	0	0	0	0	1	0
Emticicia	1	21	0	0	0	0	0	0	0	0	0	0	0	0	0	0	0	0	0	0	0	0
Enhydrobacter	1	1	0	0	0	0	0	0	0	0	0	0	0	0	0	0	0	0	0	0	0	0
Ensifer	1	2	0	0	0	0	0	0	0	0	0	0	0	0	0	0	0	0	0	0	0	0
Enterobacter	2	202	1	1	1	1	0	0	0	0	1	0	1	0	1	1	1	1	1	0	1	1
Enterobacteriaceae	2	211	1	1	1	1	1	0	0	0	1	1	1	0	0	1	0	1	1	0	1	1
Enterococcus	1	187	1	1	1	1	0	0	0	0	1	0	1	1	1	1	1	1	1	0	0	0

Genus	Cluster Number	Protein Counts	2.6.1.-	2.7.1.21	2.7.1.48	2.7.4.-	2.8.3.-	2.8.3.1	2.8.3.12	2.8.3.15	2.8.3.6	2.8.3.8	3.1.1.-	3.1.1.1	3.1.1.17	3.1.1.2	3.1.1.24	3.1.1.45	3.1.2.-	3.1.2.23	3.1.3.1	3.1.3.2
Enterovibrio	1	15	0	0	0	1	0	0	0	0	0	0	0	0	0	0	0	0	0	0	0	0
Erwinia	2	151	1	1	1	1	0	1	0	0	0	0	1	0	1	1	1	1	1	0	1	1
Erysipelothrix	1	9	0	0	0	0	0	0	0	0	0	0	0	0	0	0	0	0	0	0	0	0
Erysipelotrichaceae	1	4	0	0	0	0	0	0	0	0	0	0	0	0	0	0	0	0	0	0	0	0
Erythrobacter	2	80	0	1	0	1	0	0	0	0	0	0	1	0	0	1	1	0	1	0	1	0
Escherichia	2	242	1	1	1	1	1	1	0	0	1	1	1	0	0	1	1	1	1	0	1	1
Ethanoligenens	2	69	0	0	1	1	0	0	0	0	0	0	0	0	0	0	0	0	0	0	0	0
Eubacteriaceae	1	2	0	0	0	0	0	0	0	0	0	0	0	0	0	0	0	0	0	0	0	0
Eubacterium	2	103	1	0	1	1	0	0	0	0	0	1	0	0	1	0	0	0	0	0	0	0
Eudoraea	1	4	0	0	0	0	0	0	0	0	0	0	0	0	0	0	0	0	0	0	0	0
Euryarchaeota	1	1	0	0	0	0	0	0	0	0	0	0	0	0	0	0	0	0	0	0	0	0
Exiguobacterium	2	93	1	1	1	1	0	0	0	0	0	0	0	1	1	0	0	0	0	0	1	0
Faecalibacterium	1	12	0	0	0	0	0	0	0	0	0	0	0	0	0	0	0	0	0	0	0	0
Ferrimonas	2	115	0	1	1	1	0	0	0	0	0	0	1	0	0	1	0	0	1	0	1	0
Ferroglobus	1	14	0	0	0	0	0	0	0	0	0	0	0	0	0	0	0	0	0	0	0	0
Ferroplasma	1	11	0	0	0	0	0	0	0	0	0	0	0	0	0	0	0	0	0	0	0	0
Fervidicoccus	1	7	0	0	0	0	0	0	0	0	0	0	0	0	0	0	0	0	0	0	0	0
Fervidobacterium	2	54	0	1	1	1	0	0	0	0	0	0	0	1	0	0	0	0	0	0	1	0
Fibrella	1	17	0	0	0	0	0	0	0	0	0	0	0	0	0	0	0	0	0	0	0	0
Fibrobacter	2	52	0	0	0	1	0	0	0	0	0	0	0	0	0	0	0	0	0	0	1	0
Filifactor	1	9	0	0	0	0	0	0	0	0	0	0	0	0	0	0	0	0	0	0	0	0
Finegoldia	2	55	1	1	1	1	0	0	0	0	0	0	0	0	0	0	0	0	0	0	0	0
Firmicutes	1	15	0	0	0	0	0	0	0	0	0	0	0	0	0	0	0	0	0	0	0	0
Fischerella	1	15	0	0	0	1	0	0	0	0	0	0	0	0	0	0	0	0	0	0	0	0
Flavobacteria	1	7	0	0	0	0	0	0	0	0	0	0	0	0	0	0	0	0	0	0	0	0
Flavobacteriaceae	2	69	1	1	1	1	0	0	0	0	0	0	0	0	0	0	0	0	0	0	1	0
Flavobacteriales	1	3	0	0	0	0	0	0	0	0	0	0	0	0	0	0	0	0	0	0	0	0
Flavobacterium	2	103	1	1	1	1	0	0	0	0	0	0	0	1	0	0	1	0	0	0	1	1
Flexibacter	1	17	0	0	0	0	0	0	0	0	0	0	0	0	0	0	0	0	0	0	0	0
Flexistipes	1	11	0	0	0	0	0	0	0	0	0	0	0	0	0	0	0	0	0	0	0	0
Fluoribacter	1	6	0	0	0	1	0	0	0	0	0	0	0	0	0	0	0	0	0	0	0	0
Fluviicola	2	69	1	1	0	1	0	0	0	0	0	0	0	0	0	0	0	0	0	0	1	0
Formosa	1	2	0	0	0	0	0	0	0	0	0	0	0	0	0	0	0	0	0	0	0	0
Francisella	2	88	1	1	1	1	0	0	0	0	0	0	0	1	0	0	0	0	0	0	1	1
Frankia	3	202	0	0	1	1	1	0	1	0	0	0	1	0	0	0	1	0	1	0	0	1
Frateuria	1	25	0	0	0	0	0	0	0	0	0	0	0	0	0	0	0	0	0	0	0	0
Fructobacillus	1	2	0	0	0	0	0	0	0	0	0	0	0	0	0	0	0	0	0	0	0	0
Fulvimarina	1	2	0	0	0	0	0	0	0	0	0	0	0	0	0	0	0	0	0	0	0	0
Fusobacterium	1	48	0	0	0	1	0	0	1	0	0	1	0	0	0	0	0	0	0	0	0	0
Gallibacterium	2	87	0	1	1	1	0	0	0	0	0	0	1	0	0	0	0	0	1	0	1	1
Gallionella	2	67	1	0	0	1	0	0	0	0	0	0	0	0	0	1	0	0	0	0	1	0
Gammaproteobacteria	1	9	0	0	0	1	0	0	0	0	0	0	0	0	0	0	0	0	0	0	0	0
Gardnerella	1	31	0	0	0	1	0	0	0	0	0	0	0	0	0	0	0	0	0	0	0	0
Geitlerinema	1	9	0	0	0	0	0	0	0	0	0	0	0	0	0	0	0	0	0	0	0	0
Gemmatimonas	2	93	0	1	1	1	0	0	0	0	0	0	1	1	0	1	1	0	1	1	1	0
Geobacillus	2	168	1	1	1	1	0	0	0	0	0	0	1	1	0	0	0	0	0	0	1	0
Geobacter	2	150	1	0	0	1	0	0	1	1	1	0	1	0	0	1	1	0	0	0	1	0
Geodermatophilus	2	136	1	1	0	1	0	0	0	0	1	1	1	0	0	0	1	0	1	0	0	0
Geopsychrobacter	1	2	0	0	0	0	0	0	0	0	0	0	0	0	0	0	0	0	0	0	0	0
Gillisia	1	11	0	1	0	1	0	0	0	0	0	0	0	0	0	0	0	0	0	0	0	0
Gilvimarinus	1	4	0	0	0	1	0	0	0	0	0	0	0	0	0	0	0	0	0	0	0	0
Glaciecola	2	117	0	1	1	1	0	0	0	0	0	0	0	0	0	1	1	0	1	0	1	1

Genus	Cluster Number	Protein Counts	2.6.1.-	2.7.1.21	2.7.1.48	2.7.4.-	2.8.3.-	2.8.3.1	2.8.3.12	2.8.3.15	2.8.3.6	2.8.3.8	3.1.1.-	3.1.1.1	3.1.1.17	3.1.1.2	3.1.1.24	3.1.1.45	3.1.2.-	3.1.2.23	3.1.3.1	3.1.3.2
Gloeobacter	2	67	1	0	1	1	0	0	0	0	0	0	0	0	1	0	0	0	0	0	1	0
Gloeocapsa	1	20	0	0	0	0	0	0	0	0	0	0	0	0	0	0	0	0	0	0	0	0
Gloeothece	1	1	0	0	0	0	0	0	0	0	0	0	0	0	0	0	0	0	0	0	0	0
Gluconacetobacter	2	96	0	0	0	1	1	0	0	0	0	0	0	0	0	1	1	0	0	0	0	0
Gluconobacter	2	76	0	1	0	1	0	0	0	0	0	0	0	0	1	0	0	0	0	0	0	1
Gordonia	1	158	0	1	0	1	1	0	0	0	1	0	0	0	0	0	1	0	1	0	0	0
Gordonibacter	1	32	0	0	0	1	0	0	0	0	0	0	0	0	0	0	0	0	0	0	0	0
Gramella	2	104	1	1	1	1	0	0	0	0	0	0	0	1	0	1	1	0	0	0	1	0
Granulibacter	2	90	0	0	0	1	0	0	0	0	0	0	1	0	0	0	1	1	0	0	1	1
Granulicella	2	98	0	1	1	1	0	0	0	0	0	0	1	0	0	1	0	0	0	0	0	0
Grimontia	1	9	0	0	0	1	0	0	0	0	0	0	0	0	0	0	0	0	0	0	0	0
Haemophilus	2	117	0	1	1	1	0	0	0	0	0	1	1	0	1	0	0	0	1	0	1	1
Hafnia	1	29	0	0	1	1	0	0	0	0	0	0	1	0	0	0	0	0	0	0	0	0
Hahella	3	130	0	0	1	1	0	0	0	0	0	1	1	0	0	1	1	0	1	0	1	1
Halalkalicoccus	1	18	0	0	0	0	0	0	0	0	0	0	0	0	0	0	0	0	0	0	0	0
Halanaerobium	2	89	1	1	1	1	0	0	0	0	0	1	0	1	0	0	0	0	0	0	1	0
Haliangium	2	108	0	0	1	1	0	0	0	0	0	0	1	0	1	0	0	0	0	0	1	0
Haliscomenobacter	2	107	0	1	0	1	0	0	0	0	0	0	0	0	1	0	0	0	1	0	1	0
Haloarcula	1	22	0	0	0	0	0	0	0	0	0	0	0	0	0	0	0	0	0	0	0	0
Halobacillus	1	19	0	0	0	0	0	0	0	0	0	0	0	0	0	0	0	0	0	0	0	0
Halobacteriaceae	1	1	0	0	0	0	0	0	0	0	0	0	0	0	0	0	0	0	0	0	0	0
Halobacterium	1	10	0	0	0	0	0	0	0	0	0	0	0	0	0	0	0	0	0	0	0	0
Halobacteroides	1	14	0	0	0	0	0	0	0	0	0	0	0	0	0	0	0	0	0	0	0	0
Haloferax	1	20	0	0	0	0	0	0	0	0	0	0	0	0	0	0	0	0	0	0	0	0
Halogeometricum	1	12	0	0	0	0	0	0	0	0	0	0	0	0	0	0	0	0	0	0	0	0
Halomicrobium	1	10	0	0	0	0	0	0	0	0	0	0	0	0	0	0	0	0	0	0	0	0
Halomonas	1	149	0	0	0	1	0	0	1	0	0	1	1	0	1	1	0	0	1	0	1	1
Halopiger	1	16	0	0	0	0	0	0	0	0	0	0	0	0	0	0	0	0	0	0	0	0
Haloquadratum	1	19	0	0	0	0	0	0	0	0	0	0	0	0	0	0	0	0	0	0	0	0
Halorhabdus	1	10	0	0	0	0	0	0	0	0	0	0	0	0	0	0	0	0	0	0	0	0
Halorhodospira	2	71	0	0	0	1	0	0	0	0	0	0	0	0	0	1	0	0	0	0	0	0
Halorubrum	1	16	0	0	0	0	0	0	0	0	0	0	0	0	0	0	0	0	0	0	0	0
Haloterrigena	1	19	0	0	0	0	0	0	0	0	0	0	0	0	0	0	0	0	0	0	0	0
Halothece	1	9	0	0	0	0	0	0	0	0	0	0	0	0	0	0	0	0	0	0	0	0
Halothermothrix	2	50	0	1	1	1	0	0	0	0	0	0	0	1	0	0	0	0	0	0	0	0
Halothiobacillus	2	65	0	0	0	1	0	0	0	0	0	0	0	0	0	1	0	0	0	0	1	0
Halovivax	1	12	0	0	0	0	0	0	0	0	0	0	0	0	0	0	0	0	0	0	0	0
Helicobacter	2	79	1	0	0	1	0	0	0	0	0	0	1	0	0	0	0	0	0	0	1	1
Heliobacillus	1	4	0	0	0	0	0	0	0	0	0	0	0	0	0	0	0	0	0	0	0	0
Heliobacterium	2	51	0	0	1	1	0	0	0	0	0	0	1	0	0	0	0	0	0	0	1	0
Herbaspirillum	2	172	1	0	0	1	0	0	0	0	1	0	1	1	1	1	1	0	0	1	1	0
Herminiimonas	3	89	0	0	0	1	0	0	0	0	0	0	0	0	1	1	1	0	1	0	1	0
Herpetosiphon	3	90	1	1	1	1	0	0	0	0	0	0	1	1	0	0	1	0	0	0	1	0
Hippea	2	44	0	0	0	1	0	0	0	0	0	1	0	0	0	0	0	0	0	0	0	0
Hirschia	2	102	0	1	1	1	0	0	0	0	0	0	1	0	1	0	0	0	0	0	1	0
Histophilus	2	72	0	1	1	1	0	0	0	0	0	1	1	0	1	0	0	0	0	0	1	0
Hoeflea	1	16	0	1	0	0	0	0	0	0	0	0	0	0	0	0	0	0	0	0	0	0
Hydrogenivirga	1	2	0	0	0	0	0	0	0	0	0	0	0	0	0	0	0	0	0	0	0	0
Hydrogenobacter	2	55	0	0	0	1	0	0	0	0	0	0	0	0	0	0	0	0	0	0	1	0
Hydrogenobaculum	2	44	0	0	0	1	0	0	0	0	0	0	0	0	0	0	0	0	0	0	0	0
Hydrogenophaga	1	9	0	0	0	0	0	0	0	0	0	0	0	0	0	0	0	0	0	0	0	0
Hylemonella	1	4	0	0	0	1	0	0	0	0	0	0	0	0	0	0	0	0	0	0	0	0

Genus	Cluster Number	Protein Counts	2.6.1.-	2.7.1.21	2.7.1.48	2.7.4.-	2.8.3.-	2.8.3.1	2.8.3.12	2.8.3.15	2.8.3.6	2.8.3.8	3.1.1.-	3.1.1.1	3.1.1.17	3.1.1.2	3.1.1.24	3.1.1.45	3.1.2.-	3.1.2.23	3.1.3.1	3.1.3.2
**Hyperthermus**	1	6	0	0	0	0	0	0	0	0	0	0	0	0	0	0	0	0	0	0	0	0
**Hyphomicrobium**	2	93	1	0	0	1	0	0	0	0	0	1	1	0	0	1	1	0	1	0	0	0
**Hyphomonas**	3	105	0	1	1	1	0	0	0	0	0	0	1	0	1	0	0	0	1	0	1	1
**Ideonella**	1	18	0	0	0	1	0	0	0	0	0	0	0	0	0	0	0	0	0	0	0	0
**Idiomarina**	2	86	0	1	1	1	0	0	0	0	0	0	0	0	0	1	0	0	1	0	1	0
**Ignavibacterium**	1	14	0	0	0	0	0	0	0	0	0	0	0	0	0	0	0	0	0	0	0	0
**Ignicoccus**	1	5	0	0	0	0	0	0	0	0	0	0	0	0	0	0	0	0	0	0	0	0
**Ignisphaera**	1	8	0	0	0	0	0	0	0	0	0	0	0	0	0	0	0	0	0	0	0	0
**Ilyobacter**	2	85	0	1	1	1	0	0	0	0	0	1	1	0	0	0	1	0	0	0	1	0
**Intrasporangium**	2	114	0	1	0	1	0	0	0	0	1	0	0	0	1	0	0	0	1	0	0	0
**Isoptericola**	1	18	0	0	0	0	0	0	0	0	0	0	0	0	0	0	0	0	0	0	0	0
**Isosphaera**	2	75	1	0	0	1	0	0	0	0	0	0	1	0	1	0	1	0	0	0	1	1
**Jannaschia**	3	151	0	1	0	1	0	1	0	0	0	0	1	0	0	1	1	0	0	1	1	0
**Janthinobacterium**	2	99	0	0	0	1	0	0	0	0	0	0	1	0	1	1	1	0	1	0	1	0
**Jonesia**	2	56	0	1	0	1	0	0	0	0	0	0	0	0	0	0	0	0	1	0	0	0
**Joostella**	1	3	0	0	0	0	0	0	0	0	0	0	0	0	0	0	0	0	0	0	0	0
**Kaistia**	1	2	0	0	0	0	0	0	0	0	0	0	0	0	0	0	0	0	0	0	0	0
**Kangiella**	2	84	0	0	0	1	0	0	0	0	0	0	0	0	0	1	0	0	0	0	0	0
**Ketogulonicigenium**	2	102	0	1	0	1	0	0	0	0	1	0	1	1	1	0	0	0	0	0	1	0
**Kineococcus**	1	24	0	0	0	0	0	0	0	0	0	0	0	0	0	0	0	0	0	0	1	0
**Kinetoplastibacterium**	1	2	0	0	0	0	0	0	0	0	0	0	0	0	0	0	0	0	0	0	0	0
**Kingella**	1	4	0	0	0	0	0	0	0	0	0	0	0	0	0	0	0	0	0	0	0	0
**Kitasatospora**	1	36	0	0	0	0	0	0	0	0	0	0	0	0	0	0	0	0	0	0	0	0
**Klebsiella**	3	222	1	1	1	1	0	0	0	0	1	1	1	0	1	1	1	1	1	0	1	0
**Kluyvera**	1	1	0	0	0	0	0	0	0	0	0	0	0	0	0	0	0	0	0	0	0	0
**Kocuria**	2	87	0	1	0	1	0	0	0	0	1	0	0	0	1	0	0	0	1	0	0	0
**Kordia**	1	5	0	0	0	0	0	0	0	0	0	0	0	0	0	0	0	0	0	0	0	0
**Kosmotoga**	2	61	0	1	1	1	0	0	0	0	0	0	0	1	0	0	0	0	0	0	1	0
**Kribbella**	2	99	0	1	0	1	0	0	0	0	0	0	1	0	1	0	1	0	1	0	0	0
**Krokinobacter**	2	90	0	1	1	1	0	0	0	0	0	0	0	1	0	1	0	0	0	0	1	0
**Kyrpidia**	2	113	0	0	1	1	0	0	0	0	0	0	1	1	0	0	1	0	0	0	1	0
**Kytococcus**	2	70	0	1	0	1	0	0	0	0	0	0	0	0	1	0	1	0	1	0	1	0
**Labrenzia**	1	4	0	0	0	0	0	0	0	0	0	0	0	0	0	0	0	0	0	0	0	0
**Lachnoanaerobaculum**	1	1	0	0	0	0	0	0	0	0	0	0	0	0	0	0	0	0	0	0	0	0
**Lachnospiraceae**	1	51	0	0	1	1	0	0	0	0	0	1	1	0	0	0	0	0	0	0	0	0
**Lacinutrix**	1	20	0	0	0	0	0	0	0	0	0	0	0	0	0	0	0	0	0	0	0	0
**Lactobacillus**	2	119	1	1	1	1	0	0	0	0	0	0	1	1	0	1	0	0	0	0	1	0
**Lactococcus**	2	82	1	1	1	1	0	0	0	0	0	0	1	1	0	0	0	0	0	0	0	0
**Lamprocystis**	1	2	0	0	0	0	0	0	0	0	0	0	0	0	0	0	0	0	0	0	0	0
**Laribacter**	2	80	1	0	0	1	0	0	0	0	0	0	0	0	0	1	1	0	0	0	0	1
**Lawsonia**	2	33	0	0	0	1	0	0	0	0	0	0	1	0	0	0	0	0	0	0	0	1
**Leadbetterella**	2	79	1	1	1	1	0	0	0	0	0	0	0	0	1	0	0	0	1	0	1	0
**Leeuwenhoekiella**	1	2	0	0	0	0	0	0	0	0	0	0	0	0	0	0	0	0	0	0	0	0
**Legionella**	2	117	0	1	1	1	0	0	0	0	0	0	0	0	1	0	1	0	0	0	1	1
**Leifsonia**	2	48	0	1	0	1	0	0	0	0	0	0	0	0	0	0	0	0	1	0	0	0
**Leptolyngbya**	1	10	0	0	0	0	0	0	0	0	0	0	0	0	0	0	0	0	0	0	0	0
**Leptospira**	2	76	1	0	0	1	0	0	0	0	0	0	0	0	0	1	0	0	0	0	1	0
**Leptospirillum**	1	10	0	0	0	0	0	0	0	0	0	0	0	0	0	0	0	0	0	0	0	0
**Leptothrix**	3	186	1	0	0	1	0	0	0	0	1	1	1	1	1	1	0	1	0	0	1	0
**Leptotrichia**	2	43	1	1	1	1	0	0	0	0	0	0	1	0	0	0	0	0	0	0	0	0
**Leucobacter**	1	2	0	0	0	0	0	0	0	0	0	0	0	0	0	0	0	0	0	0	0	0
**Leuconostoc**	2	64	1	1	1	1	0	0	0	0	0	0	1	0	0	0	0	0	0	0	0	0

Genus	Cluster Number	Protein Counts	2.6.1.-	2.7.1.21	2.7.1.48	2.7.4.-	2.8.3.-	2.8.3.1	2.8.3.12	2.8.3.15	2.8.3.6	2.8.3.8	3.1.1.-	3.1.1.1	3.1.1.17	3.1.1.2	3.1.1.24	3.1.1.45	3.1.2.-	3.1.2.23	3.1.3.1	3.1.3.2
**Liberibacter**	1	4	0	0	0	0	0	0	0	0	0	0	0	0	0	0	0	0	0	0	0	0
**Limnohabitans**	1	11	0	0	0	1	0	0	0	0	0	0	0	0	0	0	0	0	0	0	0	0
**Listeria**	2	77	1	1	1	1	0	0	0	0	0	1	1	1	0	0	0	0	0	0	0	0
**Listeriaceae**	1	6	0	0	0	1	0	0	0	0	0	0	0	0	0	0	0	0	0	0	0	0
**Listonella**	1	38	0	0	1	1	0	0	0	0	0	0	0	0	0	0	0	0	1	0	0	0
**Loktanella**	1	3	0	0	0	0	0	0	0	0	0	0	0	0	0	0	0	0	0	0	0	0
**Lysinibacillus**	2	97	0	1	1	1	0	0	0	0	0	0	1	1	0	0	0	0	0	0	1	0
**Macrococcus**	2	60	0	1	1	1	0	0	0	0	0	0	1	1	0	0	0	0	0	0	1	0
**Magnetococcus**	2	64	0	0	0	1	0	0	0	0	0	0	0	0	0	0	0	0	0	0	0	0
**Magnetospirillum**	2	113	1	0	0	1	0	0	0	0	0	0	1	0	0	0	1	0	0	0	1	0
**Mahella**	2	58	0	0	1	1	0	0	0	0	0	0	0	0	0	0	0	0	0	0	0	0
**Mannheimia**	2	85	0	1	1	1	0	0	0	0	0	0	1	0	1	0	0	0	1	0	0	0
**Maribacter**	3	87	1	1	1	1	0	0	0	0	0	0	0	1	1	1	0	0	0	0	1	0
**Maricaulis**	3	87	0	1	1	1	0	0	0	0	0	0	0	0	1	0	1	0	1	1	0	0
**Marichromatium**	1	2	0	0	0	0	0	0	0	0	0	0	0	0	0	0	0	0	0	0	0	0
**Marinithermus**	2	77	1	1	0	1	0	0	0	0	0	0	1	0	0	0	0	0	0	0	0	0
**Marinitoga**	1	15	0	0	0	0	0	0	0	0	0	0	0	0	0	0	0	0	0	0	0	0
**Marinobacter**	2	163	1	0	0	1	0	0	0	0	0	0	1	0	0	1	1	0	1	0	1	0
**Marinomonas**	3	192	0	1	1	1	0	0	1	0	1	0	1	0	1	1	1	0	1	0	1	0
**Maritimibacter**	1	7	0	0	0	0	0	0	0	0	0	0	0	0	0	0	0	0	0	0	0	0
**Marivirga**	2	96	1	1	1	1	0	0	0	0	0	0	1	0	1	1	0	0	0	0	1	1
**Martelella**	1	3	0	0	0	0	0	0	0	0	0	0	0	0	0	0	0	0	0	0	0	0
**Marvinbryantia**	1	1	0	0	0	0	0	0	0	0	0	0	0	0	0	0	0	0	0	0	0	0
**Massilia**	1	4	0	0	0	1	0	0	0	0	0	0	0	0	0	0	0	0	0	0	0	0
**Mastigocladopsis**	1	16	0	0	0	1	0	0	0	0	0	0	0	0	0	0	0	0	0	0	0	0
**Megamonas**	1	47	1	0	0	1	0	0	0	0	0	0	0	1	0	0	0	0	0	0	0	0
**Megasphaera**	1	14	0	0	0	0	0	0	0	0	0	0	0	0	0	0	0	0	0	0	0	0
**Meiothermus**	2	110	1	1	1	1	0	0	0	0	0	0	1	1	0	0	1	0	0	0	1	0
**Melissococcus**	2	46	0	1	1	1	0	0	0	0	0	0	0	0	0	0	0	0	0	0	0	0
**Mesoflavibacter**	1	6	0	0	0	0	0	0	0	0	0	0	0	0	0	0	0	0	0	0	0	0
**Mesoplasma**	1	23	0	1	1	1	0	0	0	0	0	0	0	0	0	0	0	0	0	0	0	0
**Mesorhizobium**	2	201	0	1	0	1	0	1	1	0	0	0	1	1	1	1	1	0	1	0	1	0
**Mesotoga**	1	14	0	0	0	0	0	0	0	0	0	0	0	0	0	0	0	0	0	0	0	0
**Metallosphaera**	1	18	0	0	0	0	0	0	0	0	0	0	0	0	0	0	0	0	0	0	0	0
**Methanobacterium**	1	7	0	0	0	0	0	0	0	0	0	0	0	0	0	0	0	0	0	0	0	0
**Methanobrevibacter**	1	9	0	0	0	0	0	0	0	0	0	0	0	0	0	0	0	0	0	0	0	0
**Methanocaldococcus**	1	6	0	0	0	0	0	0	0	0	0	0	0	0	0	0	0	0	0	0	0	0
**Methanocella**	1	15	0	0	0	0	0	0	0	0	0	0	0	0	0	0	0	0	0	0	0	0
**Methanococcaceae**	1	1	0	0	0	0	0	0	0	0	0	0	0	0	0	0	0	0	0	0	0	0
**Methanococcoides**	1	5	0	0	0	0	0	0	0	0	0	0	0	0	0	0	0	0	0	0	0	0
**Methanococcus**	1	9	0	0	0	0	0	0	0	0	0	0	0	0	0	0	0	0	0	0	0	0
**Methanocorpusculum**	1	7	0	0	0	0	0	0	0	0	0	0	0	0	0	0	0	0	0	0	0	0
**Methanoculleus**	1	8	0	0	0	0	0	0	0	0	0	0	0	0	0	0	0	0	0	0	0	0
**Methanohalobium**	1	5	0	0	0	0	0	0	0	0	0	0	0	0	0	0	0	0	0	0	0	0
**Methanohalophilus**	1	5	0	0	0	0	0	0	0	0	0	0	0	0	0	0	0	0	0	0	0	0
**Methanolobus**	1	5	0	0	0	0	0	0	0	0	0	0	0	0	0	0	0	0	0	0	0	0
**Methanomethylovorans**	1	5	0	0	0	0	0	0	0	0	0	0	0	0	0	0	0	0	0	0	0	0
**Methanoplanus**	1	7	0	0	0	0	0	0	0	0	0	0	0	0	0	0	0	0	0	0	0	0
**Methanopyrus**	1	4	0	0	0	0	0	0	0	0	0	0	0	0	0	0	0	0	0	0	0	0
**Methanoregula**	1	8	0	0	0	0	0	0	0	0	0	0	0	0	0	0	0	0	0	0	0	0
**Methanosaeta**	1	9	0	0	0	0	0	0	0	0	0	0	0	0	0	0	0	0	0	0	0	0
**Methanosalsum**	1	7	0	0	0	0	0	0	0	0	0	0	0	0	0	0	0	0	0	0	0	0

Genus	Cluster Number	Protein Counts	2.6.1.-	2.7.1.21	2.7.1.48	2.7.4.-	2.8.3.-	2.8.3.1	2.8.3.12	2.8.3.15	2.8.3.6	2.8.3.8	3.1.1.-	3.1.1.1	3.1.1.17	3.1.1.2	3.1.1.24	3.1.1.45	3.1.2.-	3.1.2.23	3.1.3.1	3.1.3.2
**Methanosarcina**	1	18	0	0	0	0	0	0	0	0	0	0	0	0	0	0	0	0	0	0	0	0
**Methanosphaera**	1	7	0	0	0	0	0	0	0	0	0	0	0	0	0	0	0	0	0	0	0	0
**Methanosphaerula**	1	8	0	0	0	0	0	0	0	0	0	0	0	0	0	0	0	0	0	0	0	0
**Methanospirillum**	1	6	0	0	0	0	0	0	0	0	0	0	0	0	0	0	0	0	0	0	0	0
**Methanothermobacter**	1	6	0	0	0	0	0	0	0	0	0	0	0	0	0	0	0	0	0	0	0	0
**Methanothermococcus**	1	5	0	0	0	0	0	0	0	0	0	0	0	0	0	0	0	0	0	0	0	0
**Methanothermus**	1	5	0	0	0	0	0	0	0	0	0	0	0	0	0	0	0	0	0	0	0	0
**Methanotorris**	1	5	0	0	0	0	0	0	0	0	0	0	0	0	0	0	0	0	0	0	0	0
**Methylacidiphilum**	2	48	0	0	1	1	0	0	0	0	0	0	1	0	0	0	0	0	0	0	0	0
**Methylarcula**	1	2	0	0	0	0	0	0	0	0	0	0	0	0	0	0	0	0	0	0	0	0
**Methylibium**	1	38	0	0	0	0	0	0	0	0	0	0	0	0	0	0	0	0	0	0	0	0
**Methylobacillus**	2	61	0	0	0	1	0	0	0	0	0	0	1	0	0	1	0	0	0	0	1	0
**Methylobacterium**	3	236	1	0	0	1	0	1	0	0	1	1	1	1	1	1	1	0	1	0	1	0
**Methylocella**	3	117	0	0	0	1	0	0	0	0	0	1	1	0	0	1	1	0	1	0	1	0
**Methylococcus**	2	80	1	0	0	1	0	0	0	0	0	0	1	0	0	1	0	0	0	0	1	1
**Methylocystis**	1	21	0	0	0	0	0	0	0	0	0	0	0	0	0	0	0	0	0	0	0	0
**Methyloferula**	1	2	0	0	0	0	0	0	0	0	0	0	0	0	0	0	0	0	0	0	0	0
**Methylomicrobium**	1	12	0	0	0	0	0	0	0	0	0	0	0	0	0	0	0	0	0	0	0	0
**Methylomonas**	1	15	0	0	0	0	0	0	0	0	0	0	0	0	0	0	0	0	0	0	0	0
**Methylophaga**	1	7	0	0	0	0	0	0	0	0	0	0	0	0	0	0	0	0	0	0	0	0
**Methylophilus**	1	2	0	0	0	0	0	0	0	0	0	0	0	0	0	0	0	0	0	0	0	0
**Methylosinus**	1	2	0	0	0	0	0	0	0	0	0	0	0	0	0	0	0	0	0	0	0	0
**Methylotenera**	2	72	0	0	0	1	0	0	0	0	0	0	1	0	0	1	0	0	0	0	1	0
**Methyloversatilis**	1	5	0	0	0	0	0	0	0	0	0	0	0	0	0	0	0	0	0	0	0	0
**Methylovorus**	2	66	0	0	0	1	0	0	0	0	0	0	1	1	0	1	0	0	0	0	1	0
**Micavibrio**	1	9	0	0	0	0	0	0	0	0	0	0	0	0	0	0	0	0	0	0	0	0
**Microbacterium**	2	115	0	1	0	1	0	0	0	0	1	0	1	0	1	0	1	0	1	0	1	0
**Microchaete**	1	23	0	0	1	1	0	0	0	0	0	0	0	0	0	0	0	0	0	0	0	0
**Micrococcus**	2	72	0	1	0	1	0	0	0	0	0	0	0	0	1	0	0	0	1	0	0	0
**Microcoleus**	1	17	0	0	0	0	0	0	0	0	0	0	0	0	0	0	0	0	0	0	0	0
**Microcystis**	2	67	0	0	1	1	0	0	0	0	0	0	1	0	0	0	0	0	0	0	1	0
**Microlunatus**	1	25	0	0	0	0	0	0	0	0	0	0	0	0	0	0	0	0	0	0	0	0
**Micromonospora**	2	115	1	1	1	1	0	0	0	0	0	0	1	0	0	0	1	0	1	0	0	0
**Microvirga**	1	6	0	0	0	0	0	0	0	0	0	0	0	0	0	0	0	0	0	0	0	0
**Mobiluncus**	1	41	1	0	0	1	0	0	0	0	0	0	0	0	0	0	1	0	0	0	0	0
**Modestobacter**	1	43	0	0	0	0	0	0	0	0	0	0	0	0	0	0	0	0	0	0	0	0
**Moorea**	1	2	0	0	0	0	0	0	0	0	0	0	0	0	0	0	0	0	0	0	0	0
**Moorella**	2	69	0	0	0	1	0	0	0	0	0	0	0	0	0	0	0	0	0	0	0	0
**Moraxella**	2	66	0	0	0	1	0	0	0	0	0	0	0	0	0	0	0	0	1	0	0	0
**Morganella**	1	29	0	0	0	1	0	0	0	0	0	0	0	0	0	1	0	0	0	0	0	0
**Moritella**	1	2	0	0	0	0	0	0	0	0	0	0	0	0	0	0	0	0	0	0	0	0
**Muricauda**	1	23	0	0	0	0	0	0	0	0	0	0	0	0	0	0	0	0	0	0	0	0
**Mycobacterium**	3	306	0	0	0	1	1	0	1	0	1	1	1	1	1	1	1	0	1	0	1	1
**Mycoplasma**	1	53	1	1	1	1	0	0	0	0	0	0	0	0	0	0	0	0	0	0	0	0
**Myxococcus**	2	110	1	1	1	1	0	0	1	0	1	0	0	0	0	0	1	0	1	0	1	0
**Nakamurella**	2	115	0	1	0	1	0	0	0	0	1	0	0	0	0	0	0	0	1	0	0	0
**napthalene**	1	2	0	0	0	0	0	0	0	0	0	0	0	0	0	0	0	0	0	0	0	0
**Natranaerobius**	2	55	0	1	0	1	0	0	0	0	0	0	0	1	0	0	1	0	0	0	0	0
**Natrialba**	1	16	0	0	0	0	0	0	0	0	0	0	0	0	0	0	0	0	0	0	0	0
**Natrinema**	1	16	0	0	0	0	0	0	0	0	0	0	0	0	0	0	0	0	0	0	0	0
**Natronobacterium**	1	11	0	0	0	0	0	0	0	0	0	0	0	0	0	0	0	0	0	0	0	0
**Natronococcus**	1	21	0	0	0	0	0	0	0	0	0	0	0	0	0	0	0	0	0	0	0	0

Genus	Cluster Number	Protein Counts	2.6.1.-	2.7.1.21	2.7.1.48	2.7.4.-	2.8.3.-	2.8.3.1	2.8.3.12	2.8.3.15	2.8.3.6	2.8.3.8	3.1.1.-	3.1.1.1	3.1.1.17	3.1.1.2	3.1.1.24	3.1.1.45	3.1.2.-	3.1.2.23	3.1.3.1	3.1.3.2
**Natronomonas**	1	20	0	0	0	0	0	0	0	0	0	0	0	0	0	0	0	0	0	0	0	0
**Nautilia**	2	48	0	0	0	1	0	0	0	0	0	0	1	0	0	0	0	0	0	0	0	0
**Neisseria**	2	67	0	0	0	1	0	0	0	0	0	0	1	0	0	0	0	0	0	0	1	0
**Neisseriaceae**	1	1	0	0	0	0	0	0	0	0	0	0	0	0	0	0	0	0	0	0	0	0
**Neorickettsia**	1	31	1	0	0	1	0	0	0	0	0	0	1	0	0	0	0	0	0	0	0	0
**Neptuniibacter**	1	2	0	0	0	0	0	0	0	0	0	0	0	0	0	0	0	0	0	0	0	0
**Niastella**	1	21	0	0	0	0	0	0	0	0	0	0	0	0	0	0	0	0	0	0	0	0
**Nitratifractor**	2	54	1	0	0	1	0	0	0	0	0	0	1	0	0	0	0	0	0	0	0	0
**Nitratireductor**	1	19	0	0	0	0	0	0	1	0	0	0	0	0	0	0	0	0	0	0	0	0
**Nitratiruptor**	2	40	1	0	0	1	0	0	0	0	0	0	1	0	0	0	0	0	0	0	0	0
**Nitrobacter**	2	99	1	0	0	1	0	0	0	0	1	0	1	0	0	1	0	0	1	0	0	0
**Nitrococcus**	1	2	0	0	0	1	0	0	0	0	0	0	0	0	0	0	0	0	0	0	0	0
**Nitrosococcus**	2	94	0	0	0	1	0	0	0	0	0	0	0	0	1	1	1	0	0	0	0	0
**Nitrosomonas**	2	72	0	0	0	1	0	0	0	0	0	0	1	0	0	1	0	0	0	0	0	0
**Nitrosopumilaceae**	1	7	0	0	0	0	0	0	0	0	0	0	0	0	0	0	0	0	0	0	0	0
**Nitrosopumilus**	1	11	0	0	0	0	0	0	0	0	0	0	0	0	0	0	0	0	0	0	0	0
**Nitrosospira**	2	74	0	0	0	1	0	0	0	0	0	0	1	0	0	1	0	0	0	0	0	0
**Nocardia**	2	185	0	0	0	1	1	0	1	0	0	0	1	0	0	0	1	1	1	0	0	0
**Nocardioidaceae**	1	2	0	0	0	0	0	0	0	0	0	0	0	0	0	0	0	0	0	0	0	0
**Nocardioides**	2	157	1	1	0	1	1	0	1	0	1	0	1	0	0	0	1	0	1	0	0	1
**Nocardiopsis**	2	102	0	1	0	1	0	0	1	0	0	0	1	0	0	0	1	0	0	0	1	0
**Nodularia**	1	22	0	0	0	1	0	0	0	0	0	0	0	0	0	0	0	0	0	0	0	0
**Nonlabens**	1	19	0	0	0	0	0	0	0	0	0	0	0	0	0	0	0	0	0	0	0	0
**Nostoc**	2	111	0	0	1	1	0	0	0	0	0	0	0	0	1	1	1	0	0	0	1	1
**Nostocaceae**	1	2	0	0	1	0	0	0	0	0	0	0	0	0	0	0	0	0	0	0	0	0
**Novosphingobium**	2	169	0	1	0	1	1	0	0	0	1	0	1	0	0	1	1	0	1	0	1	0
**Oceanibaculum**	1	3	0	0	0	0	0	0	0	0	0	0	0	0	0	0	0	0	0	0	0	0
**Oceanibulbus**	1	12	0	0	0	1	0	0	0	0	0	0	0	0	0	0	0	0	0	0	0	0
**Oceanicola**	1	9	0	0	0	0	0	0	0	0	0	0	0	0	0	0	0	0	0	0	0	0
**Oceanimonas**	1	33	0	0	0	1	0	0	0	0	0	0	0	0	0	0	0	0	0	0	0	0
**Oceaniovalibus**	1	2	0	0	0	0	0	0	0	0	0	0	0	0	0	0	0	0	0	0	0	0
**Oceanithermus**	2	72	1	1	1	1	0	0	0	0	0	0	0	0	0	0	1	0	0	0	0	0
**Oceanobacillus**	2	91	1	1	1	1	0	0	0	0	0	0	1	1	0	0	0	0	0	0	1	0
**Oceanospirillum**	1	2	0	0	0	0	0	0	0	0	0	0	0	0	0	0	0	0	0	0	0	0
**Ochrobactrum**	2	133	0	0	0	1	0	1	0	0	1	0	1	1	1	1	1	0	1	0	1	0
**Octadecabacter**	1	29	0	0	0	0	0	0	0	0	0	0	0	0	0	0	0	0	0	0	0	0
**Oenococcus**	2	41	1	1	1	1	0	0	0	0	0	0	0	0	0	0	0	0	0	0	0	0
**Oleomonas**	1	2	0	0	0	0	0	0	0	0	0	0	0	0	0	0	0	0	0	0	0	0
**Oligotropha**	3	87	0	0	0	1	0	0	1	0	1	0	1	0	0	1	0	0	1	0	0	0
**Olsenella**	2	40	0	1	0	1	0	0	0	0	0	0	0	0	0	0	0	0	0	0	0	0
**Opitutus**	2	67	1	0	1	1	0	0	0	0	0	0	0	1	1	1	0	0	0	0	0	0
**Orientia**	1	21	0	0	0	1	0	0	0	0	0	0	1	0	0	0	0	0	0	0	0	0
**Ornithobacterium**	1	11	0	0	0	0	0	0	0	0	0	0	0	0	0	0	0	0	0	0	0	0
**Oscillatoria**	1	16	0	0	0	0	0	0	0	0	0	0	0	0	0	0	0	0	0	0	0	0
**Oscillatoriales**	1	2	0	0	0	0	0	0	0	0	0	0	0	0	0	0	0	0	0	0	0	0
**Oscillibacter**	1	20	0	0	0	0	0	0	0	0	0	0	0	0	0	0	0	0	0	0	0	0
**Owenweeksia**	1	15	0	0	0	0	0	0	0	0	0	0	0	0	0	0	0	0	0	0	0	0
**Oxalobacteraceae**	1	4	0	0	0	1	0	0	0	0	0	0	0	0	0	0	0	0	0	0	0	0
**Paenibacillus**	2	152	1	1	1	1	0	0	0	0	0	0	1	1	1	0	1	0	0	0	1	1
**Paludibacter**	2	67	0	1	1	1	1	0	0	0	0	0	0	0	0	0	0	0	0	1	1	0
**Pandoraea**	1	18	0	0	0	1	0	0	0	0	0	0	0	0	0	0	0	0	0	0	0	0
**Pannonibacter**	1	3	0	0	0	0	0	0	0	0	0	0	0	0	0	0	0	0	0	0	0	0

Genus	Cluster Number	Protein Counts	2.6.1.-	2.7.1.21	2.7.1.48	2.7.4.-	2.8.3.-	2.8.3.1	2.8.3.12	2.8.3.15	2.8.3.6	2.8.3.8	3.1.1.-	3.1.1.1	3.1.1.17	3.1.1.2	3.1.1.24	3.1.1.45	3.1.2.-	3.1.2.23	3.1.3.1	3.1.3.2
Pantholops	1	23	0	0	0	0	0	0	0	0	0	0	0	0	0	0	0	0	0	0	0	0
Pantoea	2	181	1	1	1	1	0	0	0	0	1	0	1	0	1	1	1	1	1	0	1	0
Parabacteroides	2	64	1	1	1	1	0	0	0	0	0	0	0	1	1	0	0	0	0	0	1	0
Parachlamydia	1	11	0	0	0	0	0	0	0	0	0	0	0	0	0	0	0	0	0	0	0	0
Paracoccus	2	169	0	1	0	1	0	0	0	0	1	0	1	1	0	1	1	0	0	1	1	1
Parvibaculum	3	117	0	0	0	1	0	0	0	0	0	0	1	0	1	1	1	0	1	0	0	0
Parvularcula	2	72	0	0	0	1	0	0	0	0	0	0	1	0	0	0	0	0	1	0	1	0
Pasteurella	2	85	0	1	1	1	0	0	0	0	0	0	1	0	1	0	0	0	1	0	0	0
Pasteurellaceae	1	2	0	0	0	0	0	0	0	0	0	0	0	0	0	0	0	0	0	0	0	0
Pectobacterium	1	44	0	0	0	1	0	0	0	0	0	0	0	0	0	0	0	0	0	0	0	0
Pediococcus	1	43	1	1	1	1	0	0	0	0	0	0	0	0	0	0	0	0	0	0	0	0
Pedobacter	2	88	1	1	1	1	0	0	0	0	0	0	1	1	1	1	0	0	0	0	1	1
Pelagibaca	1	10	0	0	0	0	0	0	0	0	0	0	0	0	0	0	0	0	0	0	0	0
Pelagibacterium	1	39	0	0	0	0	0	0	0	0	0	0	0	0	0	0	0	0	0	0	0	0
Pelobacter	2	85	1	0	0	1	0	0	0	0	0	0	1	0	0	1	0	0	0	0	1	0
Pelodictyon	2	62	0	0	1	1	0	0	0	0	0	0	1	1	0	0	1	0	0	0	1	1
Pelosinus	1	1	0	0	0	0	0	0	0	0	0	0	0	0	0	0	0	0	0	0	0	0
Pelotomaculum	2	60	0	0	0	1	0	0	1	0	1	1	0	0	0	0	0	0	0	0	1	0
Peptostreptococcus	1	1	0	0	0	0	0	0	0	0	0	0	0	0	0	0	0	0	0	0	0	0
Persephonella	2	55	0	0	0	1	0	0	0	0	0	0	1	0	0	0	0	0	0	0	0	0
Petrotoga	2	72	1	1	1	1	0	0	0	0	0	1	0	1	0	0	0	0	0	0	1	0
Phaeobacter	2	55	0	0	0	0	0	0	0	0	0	0	0	0	0	0	0	0	0	0	0	0
Phenylobacterium	3	103	1	0	0	1	0	0	0	0	0	0	1	0	1	1	1	0	1	0	1	0
Photobacterium	2	119	0	1	1	1	0	0	0	0	0	0	1	0	0	1	1	0	1	0	1	0
Photorhabdus	2	149	1	1	1	1	0	0	1	0	1	1	1	0	0	0	1	0	1	0	1	0
Phycisphaera	1	9	0	0	0	0	0	0	0	0	0	0	0	0	0	0	0	0	0	0	0	0
Phyllobacterium	1	6	0	0	0	0	0	0	0	0	0	0	0	0	0	0	0	0	0	0	0	0
Picrophilus	1	15	0	0	0	0	0	0	0	0	0	0	0	0	0	0	0	0	0	0	0	0
Pirellula	2	85	0	0	0	1	0	0	0	0	0	0	0	1	1	0	1	0	0	0	1	0
Planctomyces	2	93	0	0	0	1	0	0	0	0	0	0	0	0	1	1	0	0	0	0	1	0
Plautia	1	17	0	0	1	1	0	0	0	0	0	0	0	0	0	0	0	0	0	0	0	0
Plesiomonas	1	4	0	0	0	1	0	0	0	0	0	0	0	0	0	0	0	0	0	0	0	0
Pleurocapsa	1	23	0	0	0	0	0	0	0	0	0	0	0	0	0	0	0	0	0	0	0	0
Polaribacter	2	74	0	1	1	1	0	0	0	0	0	0	0	1	0	0	0	0	1	0	1	0
Polaromonas	3	238	0	0	0	1	0	0	0	0	1	1	1	1	1	1	1	0	0	1	0	0
Polymorphum	3	173	0	1	0	1	0	0	1	0	1	0	1	1	1	1	1	0	1	0	0	0
Polynucleobacter	1	19	0	0	0	0	0	0	0	0	0	0	0	0	0	0	0	0	0	0	0	0
Ponticaulis	1	1	0	0	0	0	0	0	0	0	0	0	0	0	0	0	0	0	0	0	0	0
Porphyromonas	2	63	0	1	1	1	0	0	0	0	0	0	0	0	0	0	0	0	0	0	1	1
Prevotella	1	65	0	1	1	1	0	0	0	0	0	0	0	1	0	0	0	0	0	1	1	0
Prochlorococcus	2	68	0	0	0	1	0	0	0	0	0	0	1	0	0	0	0	0	0	0	1	1
Propionibacterium	1	62	1	1	0	1	0	0	0	0	0	0	0	0	0	0	0	0	1	0	0	0
Prosthecochloris	1	16	0	0	0	0	0	0	0	0	0	0	0	0	0	0	0	0	0	0	0	0
Proteobacteria	1	19	0	0	0	0	0	0	0	0	0	0	0	0	0	0	0	0	0	0	0	0
Proteus	2	113	1	1	1	1	1	0	0	0	0	0	1	0	0	1	0	0	1	0	1	0
Providencia	1	38	0	0	0	1	0	0	0	0	0	0	1	0	0	0	0	0	0	0	0	0
Pseudaminobacter	1	10	0	0	0	0	0	0	0	0	0	0	0	0	0	0	0	0	0	0	0	0
Pseudanabaena	1	11	0	0	0	0	0	0	0	0	0	0	0	0	0	0	0	0	0	0	0	0
Pseudoalteromonas	2	188	1	1	1	1	0	0	0	0	1	0	1	0	1	1	1	0	1	0	1	1
Pseudochrobactrum	1	7	0	0	0	0	0	0	0	0	0	0	0	0	0	0	0	0	0	0	0	0
Pseudogulbenkiania	1	40	0	0	0	0	0	0	0	0	0	0	0	0	0	0	0	0	0	0	0	0
pseudomallei	3	131	1	0	0	1	0	0	0	0	1	1	1	1	1	1	1	0	0	0	0	0

Genus	Cluster Number	Protein Counts	2.6.1.-	2.7.1.21	2.7.1.48	2.7.4.-	2.8.3.-	2.8.3.1	2.8.3.12	2.8.3.15	2.8.3.6	2.8.3.8	3.1.1.-	3.1.1.1	3.1.1.17	3.1.1.2	3.1.1.24	3.1.1.45	3.1.2.-	3.1.2.23	3.1.3.1	3.1.3.2
Pseudomonas	3	381	1	1	0	1	0	0	1	0	1	1	1	1	1	1	1	1	1	1	1	1
Pseudonocardia	3	180	0	0	1	1	0	0	1	0	1	0	0	0	1	0	1	0	1	0	0	0
Pseudorhodobacter	1	4	0	0	0	0	0	0	0	0	0	0	0	0	0	0	0	0	0	0	0	0
Pseudovibrio	1	33	0	0	0	0	0	0	0	0	0	0	0	0	0	0	0	0	0	0	0	0
Pseudoxanthomonas	2	124	0	1	0	1	0	0	0	0	0	0	1	0	1	1	0	0	1	1	1	1
Psychrobacter	2	124	0	0	0	1	0	0	0	0	1	1	0	1	0	1	1	0	1	0	1	0
Psychroflexus	1	22	0	0	0	0	0	0	0	0	0	0	0	0	0	0	0	0	0	0	0	0
Psychromonas	2	110	0	1	1	1	0	0	0	0	0	0	1	0	0	1	0	0	1	0	1	0
Pusillimonas	3	111	0	0	0	1	0	0	0	0	1	0	1	0	0	1	0	0	0	0	0	0
Pyrobaculum	1	15	0	0	0	0	0	0	0	0	0	0	0	0	0	0	0	0	0	0	0	0
Pyrococcus	1	10	0	0	0	0	0	0	0	0	0	0	0	0	0	0	0	0	0	0	0	0
Pyrolobus	1	4	0	0	0	0	0	0	0	0	0	0	0	0	0	0	0	0	0	0	0	0
Rahnella	2	143	1	1	1	1	0	0	0	0	0	0	1	0	0	1	1	1	1	0	1	0
Ralstonia	3	332	1	0	0	1	1	0	1	0	1	1	1	0	1	1	1	0	0	1	1	1
Ramlibacter	1	35	0	0	0	0	0	0	0	0	0	0	0	0	0	0	0	0	0	0	0	0
Raoultella	2	139	1	1	1	1	0	0	0	0	1	0	1	0	0	1	0	1	1	0	1	0
Raphidiopsis	1	5	0	0	0	1	0	0	0	0	0	0	0	0	0	0	0	0	0	0	0	0
Reinekea	1	2	0	0	0	0	0	0	0	0	0	0	0	0	0	0	0	0	0	0	0	0
Renibacterium	2	67	0	1	0	1	0	0	0	0	0	0	0	0	0	0	0	0	1	0	0	0
Rhizobiaceae	1	3	0	0	0	0	0	0	0	0	0	0	0	0	0	0	0	0	0	0	0	0
Rhizobiales	1	6	0	0	0	0	0	0	0	0	0	0	0	0	0	0	0	0	0	0	0	0
Rhizobium	3	257	1	1	0	1	1	1	1	0	1	1	1	1	1	1	1	0	1	0	1	1
Rhizobium/Agrobacterium	2	87	0	0	0	1	0	0	0	0	1	0	0	0	0	0	0	0	0	0	1	1
Rhodanobacter	1	21	0	0	0	0	0	0	0	0	0	0	0	0	0	0	0	0	0	0	0	0
Rhodobacter	3	175	1	1	0	1	0	0	0	0	1	1	1	1	1	1	1	0	1	0	1	0
Rhodobacteraceae	1	21	0	0	0	1	0	0	0	0	0	0	0	0	0	0	0	0	0	0	0	0
Rhodobacterales	1	34	0	0	0	0	0	0	0	0	0	0	0	0	0	0	0	0	0	0	0	0
Rhodococcus	2	292	1	0	1	1	1	1	1	0	1	1	1	0	1	0	1	0	1	0	1	0
Rhodocyclaceae	1	7	0	0	0	0	0	0	0	0	0	0	0	0	0	0	0	0	0	0	0	0
Rhodoferax	3	144	1	1	0	1	0	0	0	0	1	0	1	1	1	1	1	0	1	0	1	0
Rhodomicrobium	2	103	0	0	0	1	0	0	0	0	0	0	1	0	0	1	1	0	1	0	1	0
Rhodopirellula	2	87	0	1	0	1	0	0	0	0	0	0	0	0	1	1	0	0	0	0	1	0
Rhodopseudomonas	3	246	1	0	0	1	0	0	1	0	1	1	1	1	1	1	1	0	1	0	1	0
Rhodospirillum	2	121	0	1	0	1	0	0	0	0	0	1	1	0	1	1	1	0	0	0	1	0
Rhodothermus	2	76	0	1	1	1	0	0	0	0	0	0	0	0	0	1	0	0	1	0	1	1
Rhodovulum	1	4	0	0	0	0	0	0	0	0	0	0	0	0	0	0	0	0	0	0	0	0
Richelia	1	2	0	0	0	1	0	0	0	0	0	0	0	0	0	0	0	0	0	0	0	0
Rickettsia	2	43	0	0	0	1	0	0	0	0	0	0	1	0	0	0	0	0	0	0	0	0
Rickettsiaceae	1	5	0	0	0	0	0	0	0	0	0	0	0	0	0	0	0	0	0	0	0	0
Riemerella	2	61	0	1	1	1	0	0	0	0	0	0	0	0	0	0	0	0	0	0	1	0
Rivularia	1	18	0	0	0	0	0	0	0	0	0	0	0	0	0	0	0	0	0	0	0	0
Robiginitalea	2	83	0	1	1	1	0	0	0	0	0	0	0	1	1	1	0	0	0	0	1	0
Roseburia	2	58	0	0	1	1	0	0	0	0	0	0	0	0	0	0	0	0	0	0	0	0
Roseibium	1	3	0	0	0	0	0	0	0	0	0	0	0	0	0	0	0	0	0	0	0	0
Roseiflexus	3	100	0	1	0	1	0	0	0	0	0	0	1	1	1	0	1	0	0	0	1	0
Roseobacter	2	149	0	1	0	1	0	1	0	0	0	0	1	1	1	1	1	0	0	0	1	0
Roseomonas	1	2	0	0	0	0	0	0	0	0	0	0	0	0	0	0	0	0	0	0	0	0
Roseovarius	1	15	0	0	0	0	0	0	0	0	0	0	0	0	0	0	0	0	0	0	0	0
Rothia	1	44	0	0	0	1	0	0	0	0	0	0	0	0	0	0	0	0	1	0	0	0
Rubrivivax	1	24	0	0	0	0	0	0	0	0	0	0	0	0	0	0	0	0	0	0	0	0
Rubrobacter	3	117	0	1	0	1	0	0	1	0	0	0	0	1	0	0	1	0	0	0	1	0
Ruegeria	2	189	0	1	0	1	0	0	0	0	1	0	1	0	1	1	1	0	1	1	1	0

Genus	Cluster Number	Protein Counts	2.6.1.-	2.7.1.21	2.7.1.48	2.7.4.-	2.8.3.-	2.8.3.1	2.8.3.12	2.8.3.15	2.8.3.6	2.8.3.8	3.1.1.-	3.1.1.1	3.1.1.17	3.1.1.2	3.1.1.24	3.1.1.45	3.1.2.-	3.1.2.23	3.1.3.1	3.1.3.2
**Ruminococcaceae**	1	2	0	0	0	0	0	0	0	0	0	0	0	0	0	0	0	0	0	0	0	0
**Ruminococcus**	2	101	1	1	1	1	0	0	0	0	0	1	1	0	0	1	0	0	0	0	1	0
**Runella**	1	26	0	0	0	0	0	0	0	0	0	0	0	0	0	0	0	0	0	0	0	0
**Saccharomonospora**	2	132	0	1	1	1	0	0	1	0	1	0	1	1	0	0	1	0	1	0	0	0
**Saccharophagus**	2	81	0	0	0	1	0	0	0	0	0	0	0	0	1	1	0	0	1	0	1	0
**Saccharopolyspora**	3	194	1	0	1	1	0	0	1	0	1	0	1	1	1	0	1	1	1	0	0	0
**Saccharothrix**	1	32	0	0	0	0	0	0	0	0	0	0	0	0	0	0	0	0	0	0	0	0
**Sagittula**	1	10	0	0	0	0	0	0	0	0	0	0	0	0	0	0	0	0	0	0	0	0
**Salinibacter**	2	77	0	1	1	1	0	0	0	0	0	0	0	0	0	1	0	0	0	0	1	1
**Salinimonas**	1	1	0	0	0	0	0	0	0	0	0	0	0	0	0	0	0	0	0	0	0	0
**Salinispora**	3	127	1	1	1	1	1	0	0	0	0	0	1	0	1	0	1	0	1	0	0	0
**Salinivibrio**	1	2	0	0	0	0	0	0	0	0	0	0	0	0	0	0	0	0	0	0	0	0
**Salmonella**	2	151	1	1	1	1	1	0	0	0	0	1	1	0	0	1	0	1	1	0	0	1
**Sanguibacter**	2	73	1	1	1	1	0	0	0	0	0	0	1	0	1	0	1	0	1	0	1	0
**Saprospira**	1	13	0	0	0	0	0	0	0	0	0	0	0	0	0	0	0	0	0	0	0	0
**Scardovia**	1	1	0	0	0	0	0	0	0	0	0	0	0	0	0	0	0	0	0	0	0	0
**Scytonema**	1	10	0	0	0	1	0	0	0	0	0	0	0	0	0	0	0	0	0	0	0	0
**Sebaldella**	2	69	0	1	1	1	0	0	0	0	0	0	1	0	0	0	0	0	0	0	1	1
**Segniliparus**	2	85	0	0	0	1	0	0	0	0	0	1	0	0	0	0	0	0	1	0	0	0
**Selenomonas**	2	57	0	0	1	1	0	0	0	0	0	0	0	1	0	0	0	0	0	0	0	0
**Serinicoccus**	1	1	0	0	0	0	0	0	0	0	0	0	0	0	0	0	0	0	0	0	0	0
**Serratia**	2	186	1	1	1	1	0	0	0	0	1	1	1	0	1	1	1	1	1	0	1	1
**Shewanella**	3	214	1	1	1	1	1	0	1	0	1	0	1	0	1	1	1	0	1	0	1	0
**Shigella**	2	172	1	1	1	1	1	0	0	0	0	1	1	0	0	1	0	1	1	0	1	1
**Shinella**	1	2	0	0	0	0	0	0	0	0	0	0	0	0	0	0	0	0	0	0	0	0
**Sideroxydans**	2	81	1	0	0	1	0	0	0	0	0	0	0	0	0	1	0	0	0	0	1	1
**Silicibacter**	2	105	0	0	0	1	0	0	0	0	0	0	0	0	0	0	0	0	0	0	1	0
**Simiduia**	1	18	0	0	0	1	0	0	0	0	0	0	0	0	0	0	0	0	0	0	0	0
**Simkania**	1	6	0	0	0	0	0	0	0	0	0	0	0	0	0	0	0	0	0	0	0	0
**Simonsiella**	1	2	0	0	0	0	0	0	0	0	0	0	0	0	0	0	0	0	0	0	0	0
**Singulisphaera**	1	18	0	0	0	0	0	0	0	0	0	0	0	0	0	0	0	0	0	0	0	0
**Sinorhizobium**	3	207	1	1	0	1	0	1	1	0	1	0	1	1	1	1	1	0	1	0	1	0
**Slackia**	2	46	0	0	0	1	0	0	0	0	0	0	0	0	0	0	1	0	0	0	0	0
**Smaragdicoccus**	1	3	0	0	0	0	0	0	0	0	0	0	0	0	0	0	0	0	0	0	0	0
**Sodalis**	2	71	1	1	1	1	0	0	0	0	0	0	1	0	0	0	0	0	0	0	0	0
**Solibacillus**	1	23	0	0	0	0	0	0	0	0	0	0	0	0	0	0	0	0	0	0	0	0
**Solitalea**	1	13	0	0	0	0	0	0	0	0	0	0	0	0	0	0	0	0	0	0	0	0
**Sorangium**	3	115	1	0	0	1	0	0	0	0	0	0	1	0	1	0	1	0	0	1	0	0
**Sphaerobacter**	2	100	1	0	0	1	0	0	1	0	0	0	1	1	0	0	0	0	0	0	0	0
**Sphaerochaeta**	1	42	0	0	1	0	0	0	0	0	0	0	0	0	0	0	0	0	0	0	1	0
**Sphingobacterium**	3	85	1	1	0	1	0	0	0	0	0	0	1	1	1	0	1	0	0	0	1	1
**Sphingobium**	1	179	0	1	0	1	0	0	0	0	1	0	1	0	1	1	1	0	1	0	1	0
**Sphingomonadaceae**	1	15	0	0	0	0	0	0	0	0	0	0	0	0	0	0	0	0	0	0	0	0
**Sphingomonas**	2	220	0	1	0	1	1	0	1	0	1	0	1	1	1	1	1	0	1	1	1	1
**Sphingopyxis**	3	99	1	1	0	1	0	0	0	0	1	0	1	0	1	1	1	0	1	0	1	0
**Spirochaeta**	2	103	1	1	1	0	0	0	0	0	0	0	1	0	0	0	0	0	0	0	1	0
**Spiroplasma**	1	6	0	0	0	0	0	0	0	0	0	0	0	0	0	0	0	0	0	0	0	0
**Spirosoma**	2	107	1	1	1	1	0	0	0	0	0	0	1	0	1	1	1	0	0	0	1	0
**Stackebrandtia**	2	110	0	0	1	1	0	0	1	0	0	0	0	0	1	0	1	0	1	0	1	0
**Stanieria**	1	17	0	0	0	0	0	0	0	0	0	0	0	0	0	0	0	0	0	0	0	0
**Staphylococcus**	2	108	1	1	1	1	0	0	0	0	0	0	1	1	0	0	0	0	0	0	1	1
**Staphylothermus**	1	12	0	0	0	0	0	0	0	0	0	0	0	0	0	0	0	0	0	0	0	0

Genus	Cluster Number	Protein Counts	2.6.1.-	2.7.1.21	2.7.1.48	2.7.4.-	2.8.3.-	2.8.3.1	2.8.3.12	2.8.3.15	2.8.3.6	2.8.3.8	3.1.1.-	3.1.1.1	3.1.1.17	3.1.1.2	3.1.1.24	3.1.1.45	3.1.2.-	3.1.2.23	3.1.3.1	3.1.3.2
Stappia	1	2	0	0	0	0	0	0	0	0	0	0	0	0	0	0	0	0	0	0	0	0
Starkeya	3	134	0	0	0	1	0	0	1	0	0	0	1	1	1	1	1	0	1	0	1	0
Stenotrophomonas	3	104	0	1	0	1	0	0	0	0	0	0	1	0	0	1	1	0	1	1	1	1
Stigmatella	3	129	1	1	1	1	0	0	1	0	1	0	0	0	0	0	1	0	1	0	1	0
Streptobacillus	1	38	1	1	1	1	0	0	0	0	0	0	1	0	0	0	0	0	0	0	0	0
Streptococcus	2	121	1	1	1	1	1	0	0	0	0	1	1	1	0	0	1	0	0	0	0	1
Streptomyces	2	253	1	1	1	1	1	0	1	0	1	0	1	0	1	0	1	0	1	0	1	0
Streptosporangium	3	148	0	1	1	1	1	0	1	0	1	0	0	0	1	0	1	0	1	0	0	0
Strigomonas	1	2	0	0	0	0	0	0	0	0	0	0	0	0	0	0	0	0	0	0	0	0
Sulfitobacter	1	10	0	0	0	1	0	0	0	0	0	0	0	0	0	0	0	0	0	0	0	0
Sulfobacillus	1	30	0	0	0	0	0	0	0	0	0	0	0	0	0	0	0	0	0	0	0	0
Sulfolobus	1	23	0	0	0	0	0	0	0	0	0	0	0	0	0	0	0	0	0	0	0	0
Sulfuricurvum	2	67	1	0	0	1	0	0	0	0	0	0	1	0	0	1	0	0	0	0	1	0
Sulfurihydrogenibium	2	55	0	0	0	1	0	0	0	0	0	0	1	0	0	0	0	0	0	0	0	0
Sulfurimonas	2	60	1	0	0	1	0	0	0	0	0	0	1	0	0	0	0	0	0	0	0	0
Sulfurospirillum	1	48	1	0	0	1	0	0	0	0	0	0	1	0	0	0	0	0	0	0	1	0
Sulfurovum	2	48	0	0	0	1	0	0	0	0	0	0	0	0	0	0	0	0	0	0	1	0
Symbiobacterium	2	68	0	1	1	1	0	0	0	0	0	0	0	0	0	1	1	0	0	0	0	0
Synechococcus	1	34	0	0	0	1	0	0	0	0	0	0	0	0	0	0	0	0	0	0	0	0
Synechocystis	1	40	0	0	0	1	0	0	0	0	0	0	0	0	0	0	0	0	0	0	0	0
Synergistetes	1	27	0	0	1	1	0	0	0	0	1	0	0	0	0	0	0	0	0	0	0	0
Syntrophobacter	2	101	1	0	0	1	0	0	1	0	1	1	1	0	0	1	1	0	0	0	0	0
Syntrophobotulus	2	71	1	0	0	1	0	0	0	0	0	0	1	1	0	0	1	0	0	0	1	0
Syntrophomonas	2	58	1	1	1	1	0	0	0	0	1	0	0	0	0	0	0	0	0	0	0	0
Syntrophothermus	2	60	0	0	0	1	0	0	1	0	1	0	0	1	0	0	0	0	0	0	0	0
Syntrophus	2	66	1	0	0	1	0	0	0	0	0	1	0	0	0	0	0	0	0	0	0	0
Tannerella	1	8	0	0	0	0	0	0	0	0	0	0	0	0	0	0	0	0	0	0	0	0
Taylorella	1	43	0	0	0	1	0	0	0	0	0	0	1	0	0	0	0	0	0	0	1	0
Tepidanaerobacter	2	55	0	0	1	1	0	0	0	0	0	1	0	0	0	0	0	0	0	0	1	0
Teredinibacter	2	109	0	0	0	1	0	0	0	0	0	0	0	0	1	1	0	0	1	0	1	0
Terrabacter	1	1	0	0	0	0	0	0	0	0	0	0	0	0	0	0	0	0	0	0	0	0
Terriglobus	2	83	0	1	1	1	0	0	0	0	0	0	1	0	1	1	0	0	0	0	1	1
Tetragenococcus	1	12	0	0	0	0	0	0	0	0	0	0	0	0	0	0	0	0	0	0	0	0
Thalassiobium	1	11	0	0	0	1	0	0	0	0	0	0	0	0	0	0	0	0	0	0	0	0
Thalassobacter	1	9	0	0	0	1	0	0	0	0	0	0	0	0	0	0	0	0	0	0	0	0
Thalassolituus	1	12	0	0	0	0	0	0	0	0	0	0	0	0	0	0	0	0	0	0	0	0
Thalassospira	1	2	0	0	0	0	0	0	0	0	0	0	0	0	0	0	0	0	0	0	0	0
Thauera	1	111	0	0	0	1	0	0	0	0	0	1	0	0	0	0	0	0	0	0	1	0
Thermacetogenium	1	7	0	0	0	0	0	0	0	0	0	0	0	0	0	0	0	0	0	0	0	0
Thermaerobacter	2	79	0	1	1	1	0	0	0	0	0	0	0	1	0	0	0	0	0	0	0	0
Thermanaerovibrio	2	58	1	0	1	1	0	0	0	0	0	0	1	0	0	0	0	0	0	0	1	0
Thermincola	2	62	0	0	0	1	0	0	0	0	0	0	0	0	0	0	0	0	0	0	0	0
Thermoanaerobacter	2	76	1	1	1	1	0	0	0	0	0	0	1	0	0	0	0	0	0	0	0	0
Thermoanaerobacterium	2	64	1	1	1	1	0	0	0	0	0	0	0	0	0	0	0	0	0	0	0	0
Thermobacillus	1	11	0	0	0	0	0	0	0	0	0	0	0	0	0	0	0	0	0	0	0	0
Thermobaculum	2	62	1	0	0	1	0	0	0	0	0	0	1	0	0	0	0	0	0	0	1	1
Thermobifida	2	72	0	0	0	1	0	0	0	0	0	0	1	0	0	0	1	0	1	0	0	0
Thermobispora	2	109	0	0	0	1	0	0	1	0	1	0	1	0	0	0	1	0	1	0	0	0
Thermococcus	1	13	0	0	0	0	0	0	0	0	0	0	0	0	0	0	0	0	0	0	0	0
Thermocrinis	2	60	0	1	0	1	0	0	0	0	0	0	0	0	0	0	0	0	0	0	0	0
Thermodesulfatator	1	10	0	0	0	0	0	0	0	0	0	0	0	0	0	0	0	0	0	0	0	0
Thermodesulfobacterium	1	9	0	0	0	0	0	0	0	0	0	0	0	0	0	0	0	0	0	0	0	0

Genus	Cluster Number	Protein Counts	2.6.1.-	2.7.1.21	2.7.1.48	2.7.4.-	2.8.3.-	2.8.3.1	2.8.3.12	2.8.3.15	2.8.3.6	2.8.3.8	3.1.1.-	3.1.1.1	3.1.1.17	3.1.1.2	3.1.1.24	3.1.1.45	3.1.2.-	3.1.2.23	3.1.3.1	3.1.3.2
Thermodesulfobium	1	13	0	0	0	0	0	0	0	0	0	0	0	0	0	0	0	0	0	0	0	0
Thermodesulfovibrio	2	60	1	0	0	1	0	0	0	0	0	0	0	0	0	0	0	0	0	0	1	0
Thermofilum	1	9	0	0	0	0	0	0	0	0	0	0	0	0	0	0	0	0	0	0	0	0
Thermogladius	1	7	0	0	0	0	0	0	0	0	0	0	0	0	0	0	0	0	0	0	0	0
Thermomicrobium	2	92	0	0	0	1	0	0	0	0	1	0	0	0	1	0	1	0	0	0	1	0
Thermomonospora	2	125	0	0	1	1	1	0	1	0	0	0	0	0	0	0	1	0	1	0	0	0
Thermoplasma	1	11	0	0	0	0	0	0	0	0	0	0	0	0	0	0	0	0	0	0	0	0
Thermoplasmatales	1	6	0	0	0	0	0	0	0	0	0	0	0	0	0	0	0	0	0	0	0	0
Thermoproteus	1	12	0	0	0	0	0	0	0	0	0	0	0	0	0	0	0	0	0	0	0	0
Thermosediminibacter	2	64	1	0	1	1	0	0	0	0	0	0	0	0	0	0	0	0	0	0	0	0
Thermosipho	2	70	0	1	1	1	0	0	0	0	0	1	0	1	0	0	0	0	0	0	1	0
Thermosphaera	1	10	0	0	0	0	0	0	0	0	0	0	0	0	0	0	0	0	0	0	0	0
Thermosynechococcus	1	6	0	0	0	0	0	0	0	0	0	0	0	0	0	0	0	0	0	0	0	0
Thermotoga	2	80	0	1	1	1	0	0	0	0	0	0	0	1	0	0	1	0	0	0	1	0
Thermovibrio	2	58	0	0	0	1	0	0	0	0	0	0	1	0	0	0	0	0	0	0	0	0
Thermovirga	1	9	0	0	0	0	0	0	0	0	0	0	0	0	0	0	0	0	0	0	0	0
Thermus	2	107	1	1	1	1	0	0	0	0	0	0	0	0	0	0	1	0	0	0	1	0
Thioalkalimicrobium	1	4	0	0	0	0	0	0	0	0	0	0	0	0	0	0	0	0	0	0	0	0
Thioalkalivibrio	2	91	0	0	0	1	0	0	0	0	0	0	1	0	0	1	0	0	0	0	1	0
Thiobaca	1	2	0	0	0	0	0	0	0	0	0	0	0	0	0	0	0	0	0	0	0	0
Thiobacillus	2	67	0	0	0	1	0	0	0	0	0	0	1	0	0	1	0	0	0	0	0	0
Thiocapsa	1	2	0	0	0	0	0	0	0	0	0	0	0	0	0	0	0	0	0	0	0	0
Thiocystis	1	11	0	0	0	0	0	0	0	0	0	0	0	0	0	0	0	0	0	0	0	0
Thioflavicoccus	1	13	0	0	0	0	0	0	0	0	0	0	0	0	0	0	0	0	0	0	0	0
Thiomicrospira	2	50	0	0	0	1	0	0	0	0	0	0	1	0	0	1	0	0	0	0	1	0
Thiomonas	2	85	1	0	0	1	0	0	0	0	0	0	1	0	0	1	0	0	0	0	1	0
Thiorhodococcus	1	2	0	0	0	0	0	0	0	0	0	0	0	0	0	0	0	0	0	0	0	0
Thiothrix	1	2	0	0	0	0	0	0	0	0	0	0	0	0	0	0	0	0	0	0	0	0
Tistrella	1	42	0	0	0	0	0	0	0	0	0	0	0	0	0	0	0	0	0	0	0	0
Tolumonas	2	100	0	0	1	1	0	0	0	0	0	0	1	0	1	1	0	0	0	0	1	0
Transposon	1	2	0	0	0	0	0	0	0	0	0	0	0	0	0	0	0	0	0	0	0	0
Treponema	1	63	1	0	1	0	0	0	0	0	0	0	0	1	0	0	0	0	0	0	1	0
Trichodesmium	2	71	0	0	1	1	0	0	0	0	0	0	0	0	0	1	0	0	0	0	1	0
Trichormus	1	12	0	0	0	1	0	0	0	0	0	0	0	0	0	0	0	0	0	0	0	0
Tropheryma	2	22	0	0	0	1	0	0	0	0	0	0	0	0	0	0	0	0	0	0	0	0
Truepera	2	104	1	1	1	1	0	0	0	0	0	0	0	0	1	0	1	0	0	0	1	0
Tsukamurella	2	123	0	0	0	1	1	0	0	0	1	1	1	0	0	0	1	0	1	0	1	0
Turneriella	1	16	0	0	0	0	0	0	0	0	0	0	0	0	0	0	0	0	0	0	0	0
Uliginosibacterium	1	2	0	0	0	0	0	0	0	0	0	0	0	0	0	0	0	0	0	0	0	0
Ureaplasma	1	24	0	1	1	1	0	0	0	0	0	0	0	0	0	0	0	0	0	0	0	0
Ureibacillus	1	2	0	0	0	1	0	0	0	0	0	0	0	0	0	0	0	0	0	0	0	0
Variovorax	3	187	0	0	0	1	0	0	0	0	1	0	1	0	1	1	1	0	1	1	1	0
Veillonella	1	54	1	0	0	1	0	0	0	0	0	0	1	0	0	0	0	0	0	0	0	0
Verminephrobacter	3	188	1	0	0	1	0	1	1	0	1	1	1	1	1	0	0	0	1	0	0	0
Verrucosispora	3	126	0	1	1	1	0	0	1	0	1	0	1	0	0	0	1	0	1	0	1	0
Vibrio	2	195	1	1	1	1	0	0	0	0	0	1	1	0	0	1	1	0	1	0	1	0
Vibrionales	1	46	0	1	1	1	0	0	0	0	0	0	1	0	0	0	0	0	1	0	0	0
Vitreoscilla	1	1	0	0	0	0	0	0	0	0	0	0	0	0	0	0	0	0	0	0	0	0
Vulcanisaeta	1	14	0	0	0	0	0	0	0	0	0	0	0	0	0	0	0	0	0	0	0	0
Waddlia	2	68	1	1	1	1	0	0	1	0	0	0	0	1	0	0	0	0	0	0	1	0
Wautersia	1	2	0	0	0	0	0	0	0	0	0	0	0	0	0	0	0	0	0	0	0	0
Weeksella	2	65	0	1	1	1	0	0	0	0	0	0	0	1	0	0	0	0	1	0	1	0

Genus	Cluster Number	Protein Counts	2.6.1.-	2.7.1.21	2.7.1.48	2.7.4.-	2.8.3.-	2.8.3.1	2.8.3.12	2.8.3.15	2.8.3.6	2.8.3.8	3.1.1.-	3.1.1.1	3.1.1.17	3.1.1.2	3.1.1.24	3.1.1.45	3.1.2.-	3.1.2.23	3.1.3.1	3.1.3.2
**Weissella**	1	14	0	0	0	0	0	0	0	0	0	0	0	0	0	0	0	0	0	0	0	0
**Wenxinia**	1	2	0	0	0	0	0	0	0	0	0	0	0	0	0	0	0	0	0	0	0	0
**Wigglesworthia**	1	29	0	0	0	1	0	0	0	0	0	0	0	0	0	0	0	0	0	0	0	0
**Wolbachia**	1	32	0	0	0	1	0	0	0	0	0	0	1	0	0	0	0	0	0	0	0	0
**Wolinella**	2	51	0	0	0	1	0	0	0	0	0	0	1	0	0	0	0	0	0	0	0	0
**Xanthobacter**	3	176	0	0	0	1	0	0	1	0	1	0	1	1	1	1	1	0	1	0	0	0
**Xanthobacteraceae**	1	4	0	0	0	0	0	0	0	0	0	0	0	0	0	0	0	0	0	0	0	0
**Xanthomonadaceae**	1	39	0	0	0	1	0	0	0	0	0	0	0	0	0	0	0	0	1	1	0	1
**Xanthomonas**	2	162	0	1	0	1	0	0	1	0	1	1	1	0	1	1	1	0	1	1	1	1
**Xenorhabdus**	2	127	1	1	1	1	0	0	0	0	0	0	1	0	0	1	0	0	1	0	0	1
**Xylanimonas**	2	66	0	1	0	1	0	0	0	0	0	0	0	0	0	0	1	0	1	0	0	0
**Xylella**	2	52	0	0	0	1	0	0	0	0	0	0	0	0	1	0	0	0	1	0	1	0
**Yersinia**	2	145	1	1	1	1	0	0	0	0	0	0	1	0	0	1	0	1	1	0	1	1
**Yokenella**	2	61	0	0	1	1	0	0	0	0	0	0	1	0	0	0	0	0	1	0	0	0
**Zobellia**	1	28	0	0	0	0	0	0	0	0	0	0	0	0	0	0	0	0	0	0	0	0
**Zunongwangia**	2	98	0	1	1	1	0	0	0	0	0	0	1	1	1	1	1	0	0	0	1	0
**Zymomonas**	2	67	0	1	0	1	0	0	0	0	0	0	1	0	1	0	1	0	0	0	1	1
**Zymophilus**	1	2	0	0	0	0	0	0	0	0	0	0	0	0	0	0	0	0	0	0	0	0

Genus	Cluster Number	Protein Counts	3.1.3.41	3.1.8.1	3.2.1.31	3.3.2.10	3.3.2.9	3.5.1.-	3.5.1.4	3.5.1.5	3.5.1.54	3.5.1.6	3.5.2.-	3.5.2.15	3.5.2.2	3.5.4.-	3.5.4.5	3.5.5.1	3.5.5.7	3.5.99.3	3.6.1.7	3.7.1.-
Acaricomes	1	2	0	0	0	0	0	0	0	0	0	0	0	0	0	0	0	0	0	0	0	0
Acaryochloris	2	88	0	0	0	0	1	0	1	1	0	0	0	0	0	1	0	0	1	0	1	0
Acetivibrio	1	2	0	0	0	0	0	0	0	0	0	0	0	0	0	0	0	0	0	0	0	0
Acetobacter	2	85	0	0	0	0	0	0	0	1	1	1	0	0	1	1	0	0	1	1	0	0
Acetobacteraceae	1	2	0	0	0	0	0	0	0	1	0	0	0	0	0	0	0	0	0	0	0	0
Acetobacterium	1	8	0	0	0	0	0	0	0	0	0	0	0	0	0	0	0	0	0	0	0	0
Acetohalobium	2	79	0	0	0	0	0	1	0	0	1	0	0	0	0	1	1	0	0	0	1	0
Acholeplasma	2	38	0	0	0	0	0	0	0	0	0	0	0	0	0	1	1	0	0	0	0	0
Achromobacter	2	192	0	0	0	0	1	1	1	0	1	1	0	0	0	1	0	1	0	0	1	1
Acidaminococcus	1	65	0	0	0	0	0	0	0	0	1	0	0	0	0	1	1	1	0	0	0	0
Acidianus	1	9	0	0	0	0	0	0	0	0	0	0	0	0	0	0	0	0	0	0	0	0
Acidilobus	1	8	0	0	0	0	0	0	0	0	0	0	0	0	0	0	0	0	0	0	0	0
Acidimicrobium	2	58	1	0	0	0	0	0	0	0	0	0	0	0	0	1	0	0	0	0	0	0
Acidiphilium	3	144	0	0	0	0	0	1	0	0	1	1	0	0	0	1	0	0	0	0	1	1
Acidithiobacillus	2	70	0	0	0	0	0	1	0	0	0	0	0	0	0	1	0	0	0	0	0	0
Acidobacterium	2	91	0	0	0	0	0	1	0	0	0	0	0	0	0	1	1	0	0	0	1	0
Acidocella	1	2	0	0	0	0	0	0	0	1	0	0	0	0	0	0	0	0	0	0	0	0
Acidothermus	2	68	1	0	0	0	1	0	0	0	1	0	0	0	0	1	0	0	1	0	1	1
Acidovorax	3	217	0	0	0	0	0	1	1	1	1	1	0	0	1	1	0	1	0	1	1	1
Aciduliprofundum	1	11	0	0	0	0	0	0	0	0	0	0	0	0	0	0	0	0	0	0	0	0
Acinetobacter	3	188	0	0	0	0	1	1	1	1	1	0	0	0	0	1	0	0	0	1	0	0
Actinobacillus	2	112	0	0	0	0	0	0	0	1	0	0	0	0	0	1	1	0	0	0	1	0
Actinomadura	1	1	0	0	0	0	1	0	0	0	0	0	0	0	0	0	0	0	0	0	0	0
Actinoplanes	1	52	0	0	0	0	0	0	0	0	0	0	0	0	0	0	0	0	0	0	0	0
Actinopolyspora	1	2	0	0	0	0	0	0	0	0	0	0	0	0	0	0	0	0	0	0	0	0
Actinosynnema	2	122	1	0	0	0	1	1	1	1	1	0	0	0	1	1	1	0	0	0	1	1
Advenella	1	30	0	0	0	0	0	0	0	0	0	0	0	0	0	0	0	0	0	0	0	0
Aequorivita	1	12	0	0	0	0	0	0	0	0	0	0	0	0	0	0	0	0	0	0	0	0
Aerococcus	2	57	0	0	1	0	0	1	1	0	0	0	0	0	0	1	1	0	0	0	0	0
Aeromonas	2	127	0	0	0	0	0	0	0	0	0	0	0	0	0	1	1	0	0	0	1	0
Aeropyrum	1	13	0	0	0	0	0	0	0	0	0	0	0	0	0	0	0	0	0	0	0	0
Afipia	1	54	0	0	0	0	0	0	0	1	0	0	0	0	0	0	0	0	0	0	0	0
Aggregatibacter	1	81	0	0	0	0	0	1	0	0	0	0	0	0	0	1	1	0	0	0	1	1
Agrobacterium	2	242	0	0	0	0	1	1	1	1	1	1	0	1	1	1	1	1	1	0	1	1
Ahrensia	1	2	0	0	0	0	0	0	0	1	0	0	0	0	0	0	0	0	0	0	0	0
Akkermansia	2	47	0	0	0	0	0	0	0	0	0	0	0	0	0	1	0	0	0	0	0	0
Albidiferax	3	144	0	0	0	0	0	1	0	1	1	1	0	0	0	0	0	0	0	0	1	0
Alcaligenes	1	21	0	0	0	0	0	0	0	0	0	0	0	0	0	0	0	0	0	0	0	0
Alcanivorax	2	146	0	0	0	0	1	0	1	1	1	0	0	0	0	1	0	0	0	0	1	1
Alicycliphilus	3	152	0	0	0	0	0	1	0	0	1	1	0	0	0	0	0	0	0	0	1	1
Alicyclobacillus	2	91	0	0	0	0	0	1	1	0	1	0	0	0	0	1	1	0	0	0	0	0
Aliivibrio	2	118	0	0	0	0	0	0	0	1	1	0	0	0	0	1	1	0	0	0	1	1
Alishewanella	1	1	0	0	0	0	0	0	0	0	0	0	0	0	0	0	0	0	0	0	0	0
Alistipes	1	52	0	0	0	0	0	1	0	0	0	0	0	0	0	1	1	0	0	0	0	0
Alkalilimnicola	2	97	0	0	0	0	0	0	0	1	1	0	0	0	0	1	0	0	0	0	1	0
Alkaliphilus	2	62	0	0	0	0	0	1	1	0	0	0	0	0	0	1	1	0	0	0	0	0
Allochromatium	2	84	0	0	0	0	0	1	0	0	0	0	0	0	0	1	0	0	0	0	1	0
Alphaproteobacteria	1	3	0	0	0	0	0	0	0	0	0	0	0	0	0	0	0	0	0	0	0	0
Alteromonadales	1	12	0	0	0	0	0	0	0	0	0	0	0	0	0	0	0	0	0	0	0	0
Alteromonas	1	37	0	0	0	0	0	0	0	0	0	0	0	0	0	0	0	0	0	0	0	0
Aminobacter	1	4	0	0	0	0	0	0	0	0	0	0	0	0	0	0	0	0	0	1	0	0
Aminobacterium	2	59	0	0	0	0	0	1	1	0	1	0	0	0	0	1	1	0	0	0	1	0

Genus	Cluster Number	Protein Counts	3.1.3.41	3.1.8.1	3.2.1.31	3.3.2.10	3.3.2.9	3.5.1.-	3.5.1.4	3.5.1.5	3.5.1.54	3.5.1.6	3.5.2.-	3.5.2.15	3.5.2.2	3.5.4.-	3.5.4.5	3.5.5.1	3.5.5.7	3.5.99.3	3.6.1.7	3.7.1.-
**Ammonifex**	2	50	0	0	0	0	0	1	0	0	0	0	0	0	0	0	1	0	0	0	1	0
**Amphibacillus**	1	13	0	0	0	0	0	0	0	0	0	0	0	0	0	0	0	0	0	0	0	0
**Amphritea**	1	2	0	0	0	0	0	0	0	1	0	0	0	0	0	0	0	0	0	0	0	0
**Amycolatopsis**	2	201	1	0	0	0	1	1	1	1	1	1	0	0	1	1	1	0	0	0	1	1
**Amycolicicoccus**	1	43	0	0	0	0	0	0	0	0	0	0	0	0	0	0	0	0	0	0	0	0
**Anabaena**	2	92	0	0	0	0	1	0	1	1	0	0	0	0	0	1	0	0	0	0	1	0
**Anaerobaculum**	1	11	0	0	0	0	0	0	0	0	0	0	0	0	0	0	0	0	0	0	0	0
**Anaerococcus**	2	46	0	0	0	0	0	0	0	1	0	0	0	0	1	1	1	0	0	0	1	0
**Anaerolinea**	2	66	0	0	0	0	0	1	1	0	1	0	0	0	1	0	1	0	0	0	0	0
**Anaeromyxobacter**	3	117	0	0	0	0	1	0	0	1	0	0	0	0	0	1	1	0	0	0	1	1
**Anaerostipes**	1	44	0	0	0	0	0	1	0	0	1	0	0	0	1	0	0	0	0	0	1	0
**Anaerotruncus**	1	2	0	0	0	0	0	0	0	0	0	0	0	0	0	0	0	0	0	0	0	0
**Anaplasma**	1	35	0	0	0	0	0	0	0	0	0	0	0	0	0	1	0	0	0	0	0	0
**Ancylobacter**	1	3	0	0	0	0	0	0	0	0	0	0	0	0	0	0	0	0	0	0	0	0
**Aneurinibacillus**	1	1	0	0	0	0	0	0	0	0	0	0	0	0	0	0	0	0	0	0	0	0
**Angomonas**	1	1	0	0	0	0	0	0	0	0	0	0	0	0	0	0	0	0	0	0	0	0
**Anoxybacillus**	2	72	0	0	0	0	0	1	1	0	0	0	0	0	0	1	1	0	0	0	1	0
**Aquifex**	2	45	0	0	0	0	0	0	1	0	0	0	0	0	0	1	0	0	0	0	0	0
**Aquimarina**	1	2	0	0	0	0	0	0	0	0	0	0	0	0	0	0	0	0	0	0	0	0
**Arcanobacterium**	2	45	1	0	0	0	1	0	0	0	0	0	0	0	0	1	1	0	0	0	0	1
**Archaeoglobus**	1	14	0	0	0	0	0	0	0	0	0	0	0	0	0	0	0	0	0	0	0	0
**Arcobacter**	2	101	0	0	0	0	0	1	1	1	1	0	0	0	0	0	0	0	0	0	1	0
**Aromatoleum**	3	133	1	0	0	0	0	0	0	0	0	0	0	0	0	1	0	0	0	0	0	0
**Arsenophonus**	1	4	0	0	0	0	0	0	0	0	0	0	0	0	0	0	0	0	0	0	0	0
**Arthrobacter**	2	206	1	0	0	0	1	1	1	1	1	0	0	0	0	1	1	1	0	1	1	1
**Arthrospira**	2	67	0	0	0	0	0	1	1	1	0	0	0	0	0	1	1	0	0	0	1	0
**Aster**	1	10	0	0	0	0	0	0	0	0	0	0	0	0	0	1	0	0	0	0	0	0
**Asticcacaulis**	2	103	0	0	0	0	0	1	0	0	1	0	0	0	0	1	1	0	0	0	0	1
**Atopobium**	1	28	0	0	0	0	0	0	0	0	0	0	0	0	1	1	1	0	0	0	0	0
**Aurantimonas**	1	2	0	0	0	0	0	0	0	1	0	0	0	0	0	0	0	0	0	0	0	0
**Avibacterium**	1	17	0	0	0	0	0	1	0	0	0	0	0	0	0	0	0	0	0	0	0	0
**Azoarcus**	3	172	0	0	0	0	0	0	0	1	1	0	0	0	0	1	0	0	0	0	1	1
**Azorhizobium**	3	149	0	0	0	0	0	1	1	1	1	1	0	0	1	1	0	0	1	0	1	1
**Azorhizophilus**	1	4	0	0	0	0	0	0	0	0	0	0	0	0	0	0	0	0	0	0	0	0
**Azospira**	1	19	0	0	0	0	0	0	0	0	0	0	0	0	0	0	0	0	0	0	0	0
**Azospirillum**	2	198	0	0	0	0	1	1	1	1	1	1	1	0	1	1	1	0	1	0	1	1
**Azotobacter**	3	183	0	0	0	0	1	1	0	1	1	0	0	0	0	1	0	0	0	0	1	1
**Bacillales**	1	9	0	0	0	0	0	0	0	0	0	0	0	0	0	0	0	0	0	0	0	0
**Bacilli**	1	3	0	0	0	0	0	0	0	0	0	0	0	0	0	0	0	0	0	0	0	0
**Bacillus**	2	237	0	0	1	0	1	1	1	1	1	1	0	0	1	1	1	0	1	0	1	1
**Bacteria**	2	86	0	0	0	0	1	1	1	1	1	0	0	0	0	1	0	0	0	1	0	0
**Bacteriovorax**	2	98	0	0	0	0	1	0	0	0	0	0	0	0	0	1	1	0	0	0	0	0
**Bacteroidales**	2	59	0	0	0	0	0	0	0	0	0	0	0	0	0	1	1	0	0	0	0	0
**Bacteroides**	2	96	0	0	0	0	0	0	0	0	0	0	0	0	0	1	1	0	0	0	0	0
**Bacteroidetes**	1	2	0	0	0	0	0	0	0	0	0	0	0	0	0	0	0	0	0	0	0	0
**Bartonella**	2	50	0	0	0	0	0	0	0	0	0	0	0	0	0	1	0	0	0	0	0	0
**Baumannia**	2	24	0	0	0	0	0	0	0	0	0	0	0	0	0	1	0	0	0	0	0	0
**Bdellovibrio**	2	87	0	0	0	0	0	1	0	0	0	0	0	0	0	1	1	0	0	0	0	1
**Beijerinckia**	3	123	0	0	0	0	1	0	0	1	1	0	1	0	0	1	1	0	0	0	1	1
**Belliella**	1	14	0	0	0	0	0	0	0	0	0	0	0	0	0	0	0	0	0	0	0	0
**Beutenbergia**	2	82	1	0	0	0	1	0	0	0	1	0	0	0	1	1	1	0	0	0	1	1
**Bifidobacterium**	2	61	1	0	1	0	0	1	0	1	1	0	0	0	0	1	0	0	0	0	1	0

Genus	Cluster Number	Protein Counts	3.1.3.41	3.1.8.1	3.2.1.31	3.3.2.10	3.3.2.9	3.5.1.-	3.5.1.4	3.5.1.5	3.5.1.54	3.5.1.6	3.5.2.-	3.5.2.15	3.5.2.2	3.5.4.-	3.5.4.5	3.5.5.1	3.5.5.7	3.5.99.3	3.6.1.7	3.7.1.-
Bizionia	1	1	0	0	0	0	0	0	0	0	0	0	0	0	0	0	0	0	0	0	0	0
Blastococcus	1	39	0	0	0	0	0	0	0	0	0	0	0	0	0	0	0	0	0	0	0	0
Blattabacterium	1	23	0	0	0	0	0	0	0	1	0	0	0	0	0	1	0	0	0	0	0	1
Blautia	1	22	0	0	0	0	0	0	0	0	0	0	0	0	0	0	1	0	0	0	0	1
Bordetella	3	227	0	0	0	0	1	1	1	1	1	1	0	0	1	1	0	1	0	0	1	1
Borrelia	1	32	0	0	0	0	0	0	0	0	0	0	0	0	0	0	1	0	0	0	1	0
Brachybacterium	1	18	0	0	0	0	0	0	0	0	0	0	0	0	0	0	0	0	0	0	0	0
Brachymonas	1	4	0	0	0	0	0	0	0	0	0	0	0	0	0	0	0	0	0	0	0	0
Brachyspira	2	79	0	0	1	0	0	1	1	0	0	0	0	0	1	0	0	0	0	1	0	0
Bradyrhizobiaceae	1	20	0	0	0	0	0	0	0	1	0	0	0	0	0	0	0	0	0	0	0	0
Bradyrhizobium	3	254	0	0	0	0	1	1	1	1	1	1	0	1	1	1	1	0	1	0	1	1
Brenneria	1	20	0	0	0	0	0	0	0	0	0	0	0	0	0	0	0	0	0	0	0	0
Brevibacillus	2	122	0	0	0	0	0	1	1	0	1	1	0	0	1	1	1	1	0	0	0	0
Brevibacterium	1	4	0	0	0	0	0	0	0	0	0	0	0	0	0	0	0	0	0	0	0	0
Brevundimonas	2	105	0	0	0	0	0	1	0	0	0	0	0	0	0	1	0	0	0	0	0	0
Brucella	3	138	0	0	0	0	0	0	0	1	0	1	0	0	1	1	0	0	0	0	0	1
Buchnera	1	26	0	0	0	0	0	0	0	0	0	0	0	0	0	1	0	0	0	0	0	0
Burkholderia	3	424	0	0	0	0	1	1	1	1	1	1	1	0	1	1	1	1	1	1	1	1
Burkholderiaceae	3	143	0	0	0	0	1	1	0	1	1	0	0	0	0	1	0	1	1	1	1	0
Burkholderiales	1	24	0	0	0	0	0	0	0	0	0	0	0	0	0	0	0	0	0	0	0	0
Buttiauxella	1	2	0	0	0	0	0	0	0	0	0	0	0	0	0	0	0	0	0	0	0	0
Butyrivibrio	2	67	0	0	0	0	0	1	0	0	0	0	0	0	0	1	1	0	0	0	1	0
Caldanaerobacter	2	63	0	0	0	0	0	1	1	0	1	0	0	0	0	1	1	0	0	0	1	0
Caldicellulosiruptor	2	75	0	0	1	0	0	1	1	1	0	0	0	0	0	1	1	0	0	0	0	0
Caldilinea	1	23	0	0	0	0	0	0	0	0	0	0	0	0	0	0	0	0	0	0	0	0
Caldimonas	1	6	0	0	0	0	0	0	0	1	0	0	0	0	0	0	0	0	0	0	0	0
Caldisericum	1	10	0	0	0	0	0	0	0	0	0	0	0	0	0	0	0	0	0	0	0	0
Caldisphaera	1	7	0	0	0	0	0	0	0	0	0	0	0	0	0	0	0	0	0	0	0	0
Calditerrivibrio	2	74	0	0	0	0	0	1	1	0	1	0	0	0	0	1	0	0	0	0	1	0
Caldivirga	1	13	0	0	0	0	0	0	0	0	0	0	0	0	0	0	0	0	0	0	0	0
Calothrix	1	43	0	0	0	0	0	0	0	1	0	0	0	0	0	0	0	0	0	0	0	0
Calyptogena	2	34	0	0	0	0	0	0	0	0	0	0	0	0	0	1	0	0	0	0	0	0
Campylobacter	2	80	0	0	0	0	0	1	1	0	1	0	0	0	0	0	0	0	0	0	0	1
Candidatus	2	290	0	0	0	0	1	1	1	1	1	1	0	0	1	1	1	0	1	0	1	1
Capnocytophaga	1	50	0	0	0	0	0	0	0	0	0	0	0	0	0	1	1	0	0	0	0	1
Carbophilus	1	2	0	0	0	0	0	0	0	0	0	0	0	0	0	0	0	0	0	0	0	0
Carboxydibrachium	1	14	0	0	0	0	0	1	0	0	0	0	0	0	0	0	0	0	0	0	0	0
Carboxydothermus	2	68	0	0	0	0	0	1	0	0	1	0	0	0	0	1	1	0	0	0	0	0
Carica	1	2	0	0	1	0	0	0	0	0	0	0	0	0	0	0	0	0	0	0	0	0
Carnobacterium	2	71	0	0	1	0	1	0	0	0	0	0	0	0	0	1	1	0	0	0	1	0
Catenibacterium	1	2	0	0	0	0	0	0	0	0	0	0	0	0	0	0	0	0	0	0	0	0
Catenovulum	1	2	0	0	0	0	0	0	0	1	0	0	0	0	0	0	0	0	0	0	0	0
Catenulispora	3	159	1	0	0	0	1	1	1	1	1	0	0	0	0	1	1	1	0	0	0	1
Caulobacter	3	168	0	0	0	0	1	1	1	0	1	0	0	0	0	1	1	1	0	0	0	1
Cedecea	1	45	0	0	0	0	0	0	0	0	0	0	0	0	0	0	0	0	0	0	0	0
Cellulomonas	2	96	1	0	0	0	1	1	1	1	1	0	0	0	1	1	1	0	0	0	1	0
Cellulophaga	2	105	0	0	0	0	0	0	0	0	1	0	0	0	0	1	1	0	1	0	0	1
Cellulosilyticum	1	14	0	0	0	0	0	0	0	0	0	0	0	0	0	0	0	0	0	0	0	0
Cellvibrio	2	85	0	0	0	0	0	1	0	0	1	0	0	0	0	1	0	0	0	1	0	0
Cenarchaeum	1	7	0	0	0	0	0	0	0	0	0	0	0	0	0	0	0	0	0	0	0	0
Chamaesiphon	1	13	0	0	0	0	0	0	0	1	0	0	0	0	0	0	0	0	0	0	0	0
Chelativorans	3	136	0	0	0	0	0	0	0	1	0	1	0	0	1	1	1	0	0	0	0	0

Genus	Cluster Number	Protein Counts	3.1.3.41	3.1.8.1	3.2.1.31	3.3.2.10	3.3.2.9	3.5.1.-	3.5.1.4	3.5.1.5	3.5.1.54	3.5.1.6	3.5.2.-	3.5.2.15	3.5.2.2	3.5.4.-	3.5.4.5	3.5.5.1	3.5.5.7	3.5.99.3	3.6.1.7	3.7.1.-
Chelatococcus	1	2	0	0	0	0	0	0	0	0	0	0	0	0	0	0	0	0	0	0	0	0
Chitinophaga	2	98	0	0	0	0	1	0	0	1	0	0	0	0	1	1	1	0	0	0	1	1
Chlamydia	1	36	0	0	0	0	0	1	0	0	0	0	0	0	0	1	0	0	0	0	0	0
Chlamydomonas	1	3	0	0	0	0	0	0	0	0	0	0	0	0	0	0	0	0	0	0	0	0
Chlamydophila	1	31	0	0	0	0	0	0	0	0	0	0	0	0	0	1	0	0	0	0	0	0
Chlorobaculum	2	61	0	0	0	0	0	0	0	0	0	0	0	0	0	1	0	0	0	0	1	0
Chlorobium	2	89	0	0	0	0	0	0	0	0	0	0	0	0	0	1	0	0	0	0	1	0
Chlorobium/Pelodictyon	1	2	0	0	0	0	0	0	0	0	0	0	0	0	0	0	0	0	0	0	0	0
Chloroflexus	2	105	0	0	0	0	0	1	0	0	1	0	0	0	0	0	1	0	0	0	1	0
Chlorogloeopsis	1	9	0	0	0	0	0	0	0	1	0	0	0	0	0	0	0	0	0	0	0	0
Chloroherpeton	2	59	0	0	0	0	0	0	0	0	0	0	0	0	0	1	1	0	0	0	1	0
Chromobacterium	2	117	0	0	0	0	1	1	0	0	1	0	0	0	0	1	1	0	0	0	0	0
Chromohalobacter	2	145	0	0	0	0	0	0	1	1	1	1	0	0	0	1	1	0	0	0	1	0
Chroococcidiopsis	1	24	0	0	0	0	0	0	0	0	0	0	0	0	0	0	0	0	0	0	0	0
Chryseobacterium	2	104	0	0	0	0	0	1	1	1	1	1	0	0	1	0	0	0	0	0	0	1
Citreicella	1	13	0	0	0	0	0	0	0	1	0	0	0	0	0	0	0	0	0	0	0	0
Citrobacter	2	190	0	0	0	0	0	0	0	1	1	1	1	0	0	1	1	0	0	0	1	1
Clavibacter	2	83	1	0	0	0	1	0	1	0	1	0	0	0	0	1	1	0	0	0	0	1
Clonorchis	1	1	0	0	0	0	0	0	0	0	0	0	0	0	0	0	0	0	0	0	0	0
Clostridiales	2	77	0	0	0	0	0	1	0	0	1	1	0	0	1	1	1	0	0	0	1	1
Clostridium	2	191	0	0	1	0	1	1	1	1	1	1	0	0	1	1	1	1	0	1	1	1
Cohnella	1	2	0	0	0	0	0	0	0	0	0	0	0	0	0	0	0	0	0	0	0	0
Coleofasciculus	1	4	0	0	0	0	0	0	0	1	0	0	0	0	0	0	0	0	0	0	0	0
Collimonas	1	35	0	0	0	0	0	0	0	0	0	0	0	0	0	0	0	0	0	0	0	0
Colwellia	2	130	0	0	0	0	0	1	1	0	1	1	0	0	1	1	1	0	0	0	1	0
Comamonas	2	196	0	0	0	0	1	1	1	1	1	1	0	0	1	0	0	0	0	1	0	1
Conexibacter	3	117	0	0	0	0	1	1	0	1	1	0	0	0	0	1	0	0	1	0	0	1
Coprobacillus	1	2	0	0	0	0	0	0	0	0	0	0	0	0	0	0	0	0	0	0	0	0
Coprococcus	2	76	0	0	0	0	0	1	0	0	0	0	0	0	1	1	1	0	0	0	1	0
Coprothermobacter	2	45	0	0	0	0	0	0	0	0	1	0	0	0	0	1	1	0	0	0	1	0
Coraliomargarita	2	68	0	0	0	0	0	1	0	1	1	0	0	0	0	1	1	0	0	0	0	0
Corallococcus	1	29	0	0	0	0	0	0	0	0	0	0	0	0	0	0	0	0	0	0	0	0
Coriobacterium	2	36	0	0	0	0	0	0	0	0	0	0	0	0	0	1	1	0	0	0	0	0
Corynebacterineae	1	5	0	0	0	0	0	0	0	0	0	0	0	0	0	0	0	0	0	0	0	0
Corynebacterium	2	175	1	0	1	0	1	1	0	1	1	1	0	0	0	1	1	0	0	0	1	1
Coxiella	2	54	0	0	0	0	0	0	0	0	0	0	0	0	0	1	0	0	0	0	1	0
Crinalium	1	21	0	0	0	0	0	0	0	1	0	0	0	0	0	0	0	0	0	0	0	0
Croceibacter	2	80	0	0	0	0	0	0	0	0	1	0	0	0	0	1	1	0	1	0	0	1
Crocosphaera	1	6	0	0	0	0	0	0	0	0	0	0	0	0	0	0	0	0	0	0	0	0
Cronobacter	2	120	0	0	0	0	0	0	0	0	1	0	0	0	0	1	1	0	0	0	1	1
Cryptobacterium	2	39	0	0	0	0	0	0	0	0	1	0	0	0	0	1	0	0	0	0	0	0
Cucumis	1	9	0	0	0	0	0	0	0	0	0	0	0	0	0	0	0	0	0	0	0	0
Cupriavidus	3	317	0	0	0	0	1	1	0	1	1	1	0	0	1	1	0	0	1	1	1	1
Curtobacterium	1	5	0	0	0	0	0	0	0	0	0	0	0	0	0	0	0	0	0	0	0	0
Curvibacter	1	27	0	0	0	0	0	0	0	1	0	0	0	0	0	0	0	0	0	0	0	0
Cyanobacterium	1	9	0	0	0	0	0	0	0	0	0	0	0	0	0	0	0	0	0	0	0	0
Cyanobium	1	10	0	0	0	0	0	0	0	0	0	0	0	0	0	0	0	0	0	0	0	0
Cyanothece	1	34	0	0	0	0	0	0	0	0	0	0	0	0	0	0	0	0	0	0	0	0
Cyclobacterium	1	19	0	0	0	0	0	0	0	0	0	0	0	0	0	0	0	0	0	0	0	0
Cycloclasticus	1	21	0	0	0	0	0	0	0	0	0	0	0	0	0	0	0	0	0	0	0	0
Cylindrospermopsis	1	4	0	0	0	0	0	0	0	0	0	0	0	0	0	0	0	0	0	0	0	0
Cylindrospermum	1	30	0	0	0	0	0	0	0	0	0	0	0	0	0	0	0	0	0	0	0	0

Genus	Cluster Number	Protein Counts	3.1.3.41	3.1.8.1	3.2.1.31	3.3.2.10	3.3.2.9	3.5.1.-	3.5.1.4	3.5.1.5	3.5.1.54	3.5.1.6	3.5.2.-	3.5.2.15	3.5.2.2	3.5.4.-	3.5.4.5	3.5.5.1	3.5.5.7	3.5.99.3	3.6.1.7	3.7.1.-
Cytophaga	2	69	0	0	0	0	0	0	0	1	0	0	0	0	0	1	0	0	0	0	0	1
Dactylococcopsis	1	8	0	0	0	0	0	0	0	0	0	0	0	0	0	0	0	0	0	0	0	0
Dechloromonas	1	40	0	0	0	0	0	0	0	0	0	0	0	0	0	0	0	0	0	0	0	0
Dechlorosoma	1	19	0	0	0	0	0	0	0	0	0	0	0	0	0	0	0	0	0	0	0	0
Deferribacter	2	73	0	0	0	0	0	0	1	0	0	0	0	0	0	1	0	0	0	0	1	0
Dehalobacter	1	9	0	0	0	0	0	0	0	0	0	0	0	0	0	0	0	0	0	0	0	0
Dehalococcoides	2	41	0	0	0	0	0	0	0	0	0	0	0	0	0	0	0	0	0	0	1	0
Dehalogenimonas	1	37	0	0	0	0	0	0	0	0	0	0	0	0	0	0	0	0	0	0	1	0
Deinococcus	2	140	0	0	0	0	1	1	1	1	1	0	0	0	0	1	1	0	1	0	1	0
Delftia	3	177	0	0	0	0	1	1	1	1	1	1	0	0	1	0	0	0	0	1	0	1
Denitrovibrio	2	67	0	0	0	0	0	1	1	0	0	0	0	0	0	1	0	0	0	0	1	0
Desulfarculus	2	92	0	0	0	0	0	1	1	0	1	0	0	0	0	1	1	0	0	0	1	0
Desulfatibacillum	3	84	0	0	0	0	0	0	1	0	0	0	0	0	0	1	0	1	0	0	1	0
Desulfitobacterium	2	102	0	0	0	0	0	1	1	0	0	1	0	0	1	1	1	0	0	0	0	1
Desulfobacca	2	65	0	0	0	0	0	1	0	0	0	0	0	0	0	1	1	0	0	0	0	1
Desulfobacterium	2	113	0	0	0	0	0	0	0	0	1	0	0	0	0	1	0	0	0	0	0	0
Desulfobacula	1	21	0	0	0	0	0	0	0	0	0	0	0	0	0	0	0	0	0	0	0	0
Desulfobulbus	2	88	0	0	0	0	0	1	0	0	0	0	0	0	0	1	0	0	0	0	0	1
Desulfocapsa	1	9	0	0	0	0	0	0	0	0	0	0	0	0	0	0	0	0	0	0	0	0
Desulfococcus	2	80	0	0	0	0	0	0	1	0	0	0	0	0	0	1	0	0	0	0	1	0
Desulfohalobium	2	52	0	0	0	0	0	0	1	0	0	0	0	0	0	1	0	0	0	0	1	0
Desulfomicrobium	2	65	0	0	0	0	0	1	0	0	0	0	1	0	0	1	1	0	0	0	0	0
Desulfomonile	1	25	0	0	0	0	0	0	0	0	0	0	0	0	0	0	0	0	0	0	0	0
Desulforhabdus	1	2	0	0	0	0	0	0	0	0	0	0	0	0	0	0	0	0	0	0	0	0
Desulfosporosinus	1	30	0	0	0	0	0	0	0	0	0	0	0	0	0	0	0	0	0	0	0	0
Desulfotalea	2	66	0	0	0	0	0	0	0	0	1	0	0	0	0	1	1	0	0	0	0	0
Desulfotomaculum	2	98	0	0	0	0	0	1	1	0	0	0	0	0	1	1	1	0	0	0	0	0
Desulfovibrio	2	144	0	0	0	0	0	1	0	1	0	1	1	0	1	1	1	0	0	0	1	0
Desulfurispirillum	2	78	0	0	0	0	0	1	1	0	0	0	0	0	0	1	0	0	0	0	1	0
Desulfurivibrio	2	71	0	0	0	0	0	1	1	0	0	0	0	0	0	1	0	0	0	0	0	0
Desulfurobacterium	2	53	0	0	0	0	0	1	1	0	0	0	0	0	0	0	0	0	0	0	0	0
Desulfurococcus	1	11	0	0	0	0	0	0	0	0	0	0	0	0	0	0	0	0	0	0	0	0
Diaphorobacter	1	16	0	0	0	0	0	0	0	0	0	0	0	0	0	0	0	0	0	0	0	1
Dichelobacter	2	45	0	0	0	0	0	0	0	0	0	0	0	0	0	1	0	0	0	0	0	0
Dickeya	2	155	0	0	0	0	0	1	1	0	1	1	0	0	1	1	1	1	0	0	1	1
Dictyoglomus	2	62	0	0	1	0	0	1	0	0	0	0	0	0	0	1	1	0	0	0	1	0
Dinoroseobacter	3	148	0	0	0	0	0	0	0	1	1	1	0	0	1	1	1	0	0	0	0	1
Dokdonia	1	3	0	0	0	0	0	0	0	0	0	0	0	0	0	0	0	0	0	0	0	0
Dorea	1	7	0	0	0	0	0	0	0	0	0	0	0	0	0	0	0	0	0	0	0	0
Dyadobacter	2	83	0	0	0	0	0	0	0	0	0	0	0	0	0	1	0	0	0	0	0	1
Echinicola	1	20	0	0	0	0	0	0	0	0	0	0	0	0	0	0	0	0	0	0	0	0
Edwardsiella	2	126	0	0	1	0	0	1	0	1	0	0	0	0	0	1	1	0	0	1	1	1
Eggerthella	1	51	0	0	0	0	0	0	1	0	1	0	0	0	0	1	0	0	0	0	0	0
Ehrlichia	2	37	0	0	0	0	0	0	0	0	0	0	0	0	0	1	0	0	0	0	0	0
Eikenella	1	4	0	0	0	0	0	0	0	0	0	0	0	0	0	0	0	0	0	0	0	0
Elusimicrobium	2	44	0	0	0	0	0	1	0	0	0	0	0	0	0	0	0	0	0	0	0	0
Emticicia	1	21	0	0	0	0	0	0	0	0	0	0	0	0	0	0	0	0	0	0	0	0
Enhydrobacter	1	1	0	0	0	0	0	0	0	0	0	0	0	0	0	0	0	0	0	0	0	0
Ensifer	1	2	0	0	0	0	0	0	0	0	0	0	0	0	0	0	0	0	0	0	0	0
Enterobacter	2	202	0	0	0	0	1	0	0	1	1	1	1	0	0	1	1	1	0	0	1	1
Enterobacteriaceae	2	211	0	0	1	0	1	0	0	1	1	1	0	0	0	1	1	0	0	0	1	1
Enterococcus	1	187	0	0	0	0	1	1	0	1	1	1	0	0	1	1	1	0	0	0	1	1

Genus	Cluster Number	Protein Counts	3.1.3.41	3.1.8.1	3.2.1.31	3.3.2.10	3.3.2.9	3.5.1.-	3.5.1.4	3.5.1.5	3.5.1.54	3.5.1.6	3.5.2.-	3.5.2.15	3.5.2.2	3.5.4.-	3.5.4.5	3.5.5.1	3.5.5.7	3.5.99.3	3.6.1.7	3.7.1.-
Enterovibrio	1	15	0	0	0	0	0	0	0	0	0	0	0	0	0	0	0	0	0	0	0	0
Erwinia	2	151	0	0	0	0	0	1	0	0	1	1	0	0	0	1	1	0	1	1	1	1
Erysipelothrix	1	9	0	0	0	0	0	0	0	0	0	0	0	0	0	0	0	0	0	0	0	0
Erysipelotrichaceae	1	4	0	0	0	0	0	0	0	0	0	0	0	0	0	0	0	0	0	0	0	0
Erythrobacter	2	80	0	0	0	0	1	0	0	0	0	0	0	0	0	1	1	0	1	0	0	0
Escherichia	2	242	0	0	1	0	1	0	1	1	1	1	0	0	1	1	1	1	0	0	1	1
Ethanoligenens	2	69	0	0	0	0	0	1	0	1	1	1	0	0	1	1	1	0	0	0	1	0
Eubacteriaceae	1	2	0	0	0	0	0	0	0	0	0	0	0	0	0	0	0	0	0	0	0	0
Eubacterium	2	103	0	0	1	0	0	1	0	0	0	1	0	0	1	1	1	0	0	0	1	0
Eudoraea	1	4	0	0	0	0	0	0	0	0	0	0	0	0	0	0	0	0	0	0	0	0
Euryarchaeota	1	1	0	0	0	0	0	0	0	0	0	0	0	0	0	0	0	0	0	0	0	0
Exiguobacterium	2	93	0	0	0	0	0	1	0	0	0	0	0	0	0	1	1	0	0	0	1	0
Faecalibacterium	1	12	0	0	0	0	0	0	0	0	0	0	0	0	0	0	0	0	0	0	0	0
Ferrimonas	2	115	0	0	0	0	0	1	0	0	1	0	0	0	0	1	1	0	0	0	0	0
Ferroglobus	1	14	0	0	0	0	0	0	0	0	0	0	0	0	0	0	0	0	0	0	0	0
Ferroplasma	1	11	0	0	0	0	0	0	0	0	0	0	0	0	0	0	0	0	0	0	0	0
Fervidicoccus	1	7	0	0	0	0	0	0	0	0	0	0	0	0	0	0	0	0	0	0	0	0
Fervidobacterium	2	54	0	0	0	0	0	0	0	0	0	0	0	0	0	0	1	0	0	0	1	0
Fibrella	1	17	0	0	0	0	0	0	0	0	0	0	0	0	0	0	0	0	0	0	0	0
Fibrobacter	2	52	0	0	0	0	0	1	0	0	0	0	0	0	0	1	0	0	0	0	0	0
Filifactor	1	9	0	0	0	0	0	0	0	0	0	0	0	0	0	0	0	0	0	0	0	0
Finegoldia	2	55	0	0	0	0	0	1	0	0	0	0	0	0	0	1	1	0	0	0	0	0
Firmicutes	1	15	0	0	0	0	0	0	0	0	0	0	0	0	0	0	0	0	0	0	0	0
Fischerella	1	15	0	0	0	0	0	0	0	1	0	0	0	0	0	0	0	0	0	0	0	0
Flavobacteria	1	7	0	0	0	0	0	0	0	0	0	0	0	0	0	0	0	0	0	0	0	0
Flavobacteriaceae	2	69	0	0	0	0	0	0	0	0	0	0	0	0	0	1	1	0	0	0	0	1
Flavobacteriales	1	3	0	0	0	0	0	0	0	0	0	0	0	0	0	0	0	0	0	0	0	0
Flavobacterium	2	103	0	0	1	1	1	1	0	1	0	0	0	0	0	1	1	0	0	0	0	1
Flexibacter	1	17	0	0	0	0	0	0	0	0	0	0	0	0	0	0	0	0	0	0	0	0
Flexistipes	1	11	0	0	0	0	0	0	0	0	0	0	0	0	0	0	0	0	0	0	0	0
Fluoribacter	1	6	0	0	0	0	0	0	0	0	0	0	0	0	0	0	0	0	0	0	0	0
Fluviicola	2	69	0	0	0	0	0	1	0	0	0	0	0	0	0	1	1	0	0	0	0	0
Formosa	1	2	0	0	0	0	0	0	0	0	0	0	0	0	0	0	0	0	0	0	0	0
Francisella	2	88	0	0	0	0	0	1	0	0	1	0	0	0	0	1	1	0	0	0	0	0
Frankia	3	202	1	0	1	0	1	1	1	1	1	0	0	0	1	1	1	0	0	1	1	1
Frateuria	1	25	0	0	0	0	0	0	0	0	0	0	0	0	0	0	0	0	0	0	0	0
Fructobacillus	1	2	0	0	0	0	0	0	0	0	0	0	0	0	0	0	0	0	0	0	0	0
Fulvimarina	1	2	0	0	0	0	0	0	0	1	0	0	0	0	0	0	0	0	0	0	0	0
Fusobacterium	1	48	0	0	0	0	0	0	0	0	1	0	0	0	0	0	0	0	0	0	0	0
Gallibacterium	2	87	0	0	0	0	0	1	0	0	1	0	0	0	0	1	1	0	0	0	0	1
Gallionella	2	67	0	0	0	0	0	1	0	0	1	0	0	0	0	1	0	0	0	0	0	0
Gammaproteobacteria	1	9	0	0	0	0	0	0	0	0	0	0	0	0	0	0	0	0	0	0	0	0
Gardnerella	1	31	1	0	0	0	0	0	0	0	0	0	0	0	0	1	0	0	0	0	0	0
Geitlerinema	1	9	0	0	0	0	0	0	0	0	0	0	0	0	0	0	0	0	0	0	0	0
Gemmatimonas	2	93	0	0	0	0	0	1	0	0	0	0	0	0	0	1	1	0	0	0	0	0
Geobacillus	2	168	0	1	0	0	0	1	1	1	1	1	0	0	1	1	1	1	0	0	1	1
Geobacter	2	150	0	0	0	0	0	1	1	0	1	0	0	0	0	1	0	0	0	0	1	0
Geodermatophilus	2	136	1	0	0	0	1	0	0	1	1	0	0	0	0	1	1	0	0	0	1	1
Geopsychrobacter	1	2	0	0	0	0	0	0	0	0	0	0	0	0	0	0	0	0	0	0	0	0
Gillisia	1	11	0	0	0	0	0	0	1	0	0	0	0	0	0	0	0	0	0	0	0	0
Gilvimarinus	1	4	0	0	0	0	0	0	1	0	0	0	0	0	0	0	0	0	0	0	0	0
Glaciecola	2	117	0	0	0	0	0	0	0	1	0	0	0	0	0	1	1	1	0	0	0	0

Genus	Cluster Number	Protein Counts	3.1.3.41	3.1.8.1	3.2.1.31	3.3.2.10	3.3.2.9	3.5.1.-	3.5.1.4	3.5.1.5	3.5.1.54	3.5.1.6	3.5.2.-	3.5.2.15	3.5.2.2	3.5.4.-	3.5.4.5	3.5.5.1	3.5.5.7	3.5.99.3	3.6.1.7	3.7.1.-
**Gloeobacter**	2	67	0	0	0	0	1	1	0	0	1	0	0	0	0	1	0	0	0	0	1	0
**Gloeocapsa**	1	20	0	0	0	0	0	0	0	0	0	0	0	0	0	0	0	0	0	0	0	0
**Gloeothece**	1	1	0	0	0	0	0	0	0	0	0	0	0	0	0	0	0	0	0	0	0	0
**Gluconacetobacter**	2	96	0	0	0	0	1	0	0	0	1	1	0	0	1	1	1	0	1	0	0	1
**Gluconobacter**	2	76	0	0	0	0	0	0	0	0	1	0	0	0	0	1	0	0	0	0	0	0
**Gordonia**	1	158	1	0	0	0	1	1	1	1	1	0	0	0	0	1	1	0	0	0	1	1
**Gordonibacter**	1	32	0	0	0	0	0	0	1	0	1	0	0	0	0	0	0	0	0	0	0	0
**Gramella**	2	104	0	0	0	0	0	0	1	0	1	0	0	0	0	1	1	0	0	0	0	1
**Granulibacter**	2	90	0	0	0	0	1	0	1	1	1	1	0	0	0	1	1	0	0	0	1	0
**Granulicella**	2	98	0	0	0	0	1	1	0	0	1	0	0	0	1	1	1	0	0	0	1	0
**Grimontia**	1	9	0	0	0	0	0	0	0	0	0	0	0	0	0	0	0	0	0	0	0	0
**Haemophilus**	2	117	0	0	1	0	0	1	0	1	1	0	0	0	0	1	1	0	0	0	1	1
**Hafnia**	1	29	0	0	0	0	0	0	0	0	0	0	0	0	0	0	0	0	0	0	0	0
**Hahella**	3	130	0	0	0	0	0	0	0	1	0	0	0	0	0	1	0	0	0	0	1	1
**Halalkalicoccus**	1	18	0	0	0	0	0	0	0	0	0	0	0	0	0	0	0	0	0	0	0	0
**Halanaerobium**	2	89	0	0	1	0	0	1	1	0	0	0	0	0	1	1	1	1	0	0	1	0
**Haliangium**	2	108	0	0	0	0	1	1	0	1	0	0	0	0	1	1	1	0	1	0	1	1
**Haliscomenobacter**	2	107	0	0	0	0	0	1	0	0	1	0	0	0	0	1	1	0	0	0	0	1
**Haloarcula**	1	22	0	0	0	0	0	0	0	0	0	0	0	0	0	0	0	0	0	0	0	0
**Halobacillus**	1	19	0	0	0	0	0	0	0	0	0	0	0	0	0	0	0	0	0	0	0	0
**Halobacteriaceae**	1	1	0	0	0	0	0	0	0	0	0	0	0	0	0	0	0	0	0	0	0	0
**Halobacterium**	1	10	0	0	0	0	0	0	0	0	0	0	0	0	0	0	0	0	0	0	0	0
**Halobacteroides**	1	14	0	0	0	0	0	0	0	0	0	0	0	0	0	0	0	0	0	0	0	0
**Haloferax**	1	20	0	0	0	0	0	0	0	0	0	0	0	0	0	0	0	0	0	0	0	0
**Halogeometricum**	1	12	0	0	0	0	0	0	0	0	0	0	0	0	0	0	0	0	0	0	0	0
**Halomicrobium**	1	10	0	0	0	0	0	0	0	0	0	0	0	0	0	0	0	0	0	0	0	0
**Halomonas**	1	149	0	0	0	0	0	1	0	1	1	1	0	0	0	1	1	0	0	0	1	1
**Halopiger**	1	16	0	0	0	0	0	0	0	0	0	0	0	0	0	0	0	0	0	0	0	0
**Haloquadratum**	1	19	0	0	0	0	0	0	0	0	0	0	0	0	0	0	0	0	0	0	0	0
**Halorhabdus**	1	10	0	0	0	0	0	0	0	0	0	0	0	0	0	0	0	0	0	0	0	0
**Halorhodospira**	2	71	0	0	0	0	0	0	1	0	1	0	0	0	0	1	0	0	0	0	1	0
**Halorubrum**	1	16	0	0	0	0	0	0	0	0	0	0	0	0	0	0	0	0	0	0	0	0
**Haloterrigena**	1	19	0	0	0	0	0	0	0	0	0	0	0	0	0	0	0	0	0	0	0	0
**Halothece**	1	9	0	0	0	0	0	0	0	0	0	0	0	0	0	0	0	0	0	0	0	0
**Halothermothrix**	2	50	0	0	0	0	0	1	1	0	0	0	0	0	0	0	1	0	0	0	1	0
**Halothiobacillus**	2	65	0	0	0	0	0	1	1	0	1	0	0	0	0	1	0	0	0	1	0	0
**Halovivax**	1	12	0	0	0	0	0	0	0	0	0	0	0	0	0	0	0	0	0	0	0	0
**Helicobacter**	2	79	0	0	0	0	0	1	1	1	1	0	0	0	0	0	0	0	0	0	1	0
**Heliobacillus**	1	4	0	0	0	0	0	0	0	0	0	0	0	0	0	0	0	0	0	0	0	0
**Heliobacterium**	2	51	0	0	0	0	0	1	0	0	1	0	0	0	1	1	1	0	0	0	0	0
**Herbaspirillum**	2	172	0	0	0	0	1	1	1	1	1	1	0	0	0	1	0	0	0	1	0	1
**Herminiimonas**	3	89	0	0	0	0	0	0	0	0	0	0	0	0	0	1	0	0	0	0	0	0
**Herpetosiphon**	3	90	0	0	0	0	0	1	0	1	1	0	0	0	0	0	1	0	0	0	1	0
**Hippea**	2	44	0	0	0	0	0	1	0	0	0	0	0	0	0	0	0	0	0	0	1	0
**Hirschia**	2	102	0	0	0	0	0	0	0	1	1	0	0	0	0	1	0	0	0	0	0	1
**Histophilus**	2	72	0	0	1	0	0	0	0	0	1	0	0	0	0	1	1	0	0	0	0	1
**Hoeflea**	1	16	0	0	0	0	0	0	0	1	0	0	0	0	0	0	0	0	0	0	0	0
**Hydrogenivirga**	1	2	0	0	0	0	0	0	0	0	0	0	0	0	0	0	0	0	0	0	0	0
**Hydrogenobacter**	2	55	0	0	0	0	0	0	0	0	0	0	0	0	0	1	0	0	0	0	1	0
**Hydrogenobaculum**	2	44	0	0	0	0	0	1	0	0	0	0	0	0	0	1	0	0	0	0	1	0
**Hydrogenophaga**	1	9	0	0	0	0	0	0	0	0	0	0	0	0	0	0	0	0	0	0	0	0
**Hylemonella**	1	4	0	0	0	0	0	0	0	0	0	0	0	0	0	0	0	0	0	0	0	0

Genus	Cluster Number	Protein Counts	3.1.3.41	3.1.8.1	3.2.1.31	3.3.2.10	3.3.2.9	3.5.1.-	3.5.1.4	3.5.1.5	3.5.1.54	3.5.1.6	3.5.2.-	3.5.2.15	3.5.2.2	3.5.4.-	3.5.4.5	3.5.5.1	3.5.5.7	3.5.99.3	3.6.1.7	3.7.1.-
Hyperthermus	1	6	0	0	0	0	0	0	0	0	0	0	0	0	0	0	0	0	0	0	0	0
Hyphomicrobium	2	93	0	0	0	0	0	1	0	1	0	0	0	0	0	1	0	0	0	0	0	0
Hyphomonas	3	105	0	0	0	0	1	0	0	0	1	0	0	0	0	1	1	0	0	0	0	1
Ideonella	1	18	0	0	0	0	0	0	0	1	0	0	0	0	0	0	0	0	0	0	0	0
Idiomarina	2	86	0	0	0	0	0	0	0	0	1	0	0	0	0	1	0	0	0	0	0	1
Ignavibacterium	1	14	0	0	0	0	0	0	0	0	0	0	0	0	0	0	0	0	0	0	0	0
Ignicoccus	1	5	0	0	0	0	0	0	0	0	0	0	0	0	0	0	0	0	0	0	0	0
Ignisphaera	1	8	0	0	0	0	0	0	0	0	0	0	0	0	0	0	0	0	0	0	0	0
Ilyobacter	2	85	0	0	0	0	0	1	1	0	1	0	0	0	1	0	1	0	0	0	1	1
Intrasporangium	2	114	1	0	0	0	1	0	1	0	1	0	0	0	0	1	1	0	0	0	1	1
Isoptericola	1	18	0	0	0	0	0	0	0	0	0	0	0	0	0	0	0	0	0	0	0	0
Isosphaera	2	75	0	0	0	0	0	0	0	0	1	0	0	0	0	1	1	0	0	0	0	0
Jannaschia	3	151	0	0	0	0	1	1	1	1	1	1	0	0	1	1	1	1	0	0	0	1
Janthinobacterium	2	99	0	0	0	0	1	1	0	1	1	0	0	0	0	1	0	1	0	1	0	0
Jonesia	2	56	1	0	0	0	1	1	0	0	0	0	0	0	0	1	1	0	0	0	0	0
Joostella	1	3	0	0	0	0	0	0	0	0	0	0	0	0	0	0	0	0	0	0	0	0
Kaistia	1	2	0	0	0	0	0	0	0	1	0	0	0	0	0	0	0	0	0	0	0	0
Kangiella	2	84	0	0	0	0	0	1	0	0	1	0	0	0	0	1	0	0	0	0	0	0
Ketogulonicigenium	2	102	0	0	0	0	0	1	0	1	1	1	0	0	1	1	1	0	0	0	0	0
Kineococcus	1	24	0	0	0	0	0	0	0	0	0	0	0	0	0	0	0	0	0	0	0	0
Kinetoplastibacterium	1	2	0	0	0	0	0	0	0	0	0	0	0	0	0	0	0	0	0	0	0	0
Kingella	1	4	0	0	0	0	0	0	0	0	0	0	0	0	0	0	0	0	0	0	0	0
Kitasatospora	1	36	0	0	0	0	0	0	0	0	0	0	0	0	0	0	0	0	0	0	0	0
Klebsiella	3	222	0	0	0	0	1	0	0	1	1	1	0	0	0	1	1	0	0	0	1	1
Kluyvera	1	1	0	0	0	0	0	0	0	0	0	0	0	0	0	0	0	0	0	0	0	0
Kocuria	2	87	1	0	0	0	1	0	0	1	1	0	0	0	0	1	1	0	0	0	1	0
Kordia	1	5	0	0	0	0	0	0	0	0	0	0	0	0	0	0	0	0	0	0	0	0
Kosmotoga	2	61	0	0	0	0	0	0	0	0	0	0	0	0	1	0	1	0	0	1	1	1
Kribbella	2	99	1	0	0	0	1	1	0	0	1	0	0	0	1	1	1	1	0	0	0	1
Krokinobacter	2	90	0	0	0	0	0	0	0	0	1	0	0	0	0	1	1	0	1	0	0	1
Kyrpidia	2	113	0	0	0	0	0	1	1	1	1	0	0	0	0	1	1	0	0	0	1	1
Kytococcus	2	70	1	0	0	0	0	0	0	0	1	0	0	0	0	1	1	0	0	0	1	0
Labrenzia	1	4	0	0	0	0	0	0	0	1	0	0	0	0	0	0	0	0	0	0	0	0
Lachnoanaerobaculum	1	1	0	0	0	0	0	0	0	0	0	0	0	0	0	0	0	0	0	0	0	0
Lachnospiraceae	1	51	0	0	0	0	0	1	0	0	1	1	0	0	1	0	0	0	0	0	1	1
Lacinutrix	1	20	0	0	0	0	0	0	0	0	0	0	0	0	0	0	0	0	0	0	0	0
Lactobacillus	2	119	0	0	1	0	0	1	1	0	1	0	0	0	0	1	1	0	0	0	1	1
Lactococcus	2	82	0	0	0	0	0	1	0	0	0	0	0	0	0	1	1	0	0	0	0	0
Lamprocystis	1	2	0	0	0	0	0	0	0	0	0	0	0	0	0	0	0	0	0	0	0	0
Laribacter	2	80	0	0	0	0	0	0	0	1	0	0	0	0	0	1	0	0	0	0	1	0
Lawsonia	2	33	0	0	0	0	0	0	0	0	0	0	0	0	0	1	0	0	0	0	0	0
Leadbetterella	2	79	0	0	0	0	0	1	0	0	1	1	0	0	0	1	1	0	0	0	0	1
Leeuwenhoekiella	1	2	0	0	0	0	0	0	0	0	0	0	0	0	0	0	0	0	0	0	0	0
Legionella	2	117	0	0	0	0	0	0	1	0	0	0	0	0	0	1	1	0	0	0	1	1
Leifsonia	2	48	1	0	0	0	0	0	0	0	0	0	0	0	0	1	1	0	0	0	0	0
Leptolyngbya	1	10	0	0	0	0	0	0	0	1	0	0	0	0	0	0	0	0	0	0	0	0
Leptospira	2	76	0	0	0	0	1	0	0	0	0	0	0	0	0	1	0	0	0	0	1	0
Leptospirillum	1	10	0	0	0	0	0	0	0	0	0	0	0	0	0	0	0	0	0	0	0	0
Leptothrix	3	186	0	0	0	0	0	0	0	1	1	0	0	0	0	1	0	0	0	0	1	1
Leptotrichia	2	43	0	0	0	0	0	1	0	0	0	0	0	0	0	0	1	0	0	0	0	0
Leucobacter	1	2	0	0	0	0	0	0	0	0	0	0	0	0	0	0	0	0	0	0	0	0
Leuconostoc	2	64	0	0	0	0	0	0	1	0	1	1	0	0	0	1	1	0	0	0	1	0

Genus	Cluster Number	Protein Counts	3.1.3.41	3.1.8.1	3.2.1.31	3.3.2.10	3.3.2.9	3.5.1.-	3.5.1.4	3.5.1.5	3.5.1.54	3.5.1.6	3.5.2.-	3.5.2.15	3.5.2.2	3.5.4.-	3.5.4.5	3.5.5.1	3.5.5.7	3.5.99.3	3.6.1.7	3.7.1.-
Liberibacter	1	4	0	0	0	0	0	0	0	0	0	0	0	0	0	0	0	0	0	0	0	0
Limnohabitans	1	11	0	0	0	0	0	0	0	1	0	0	0	0	0	0	0	0	0	0	0	0
Listeria	2	77	0	0	0	0	0	1	1	0	0	0	0	0	0	1	1	0	0	0	0	1
Listeriaceae	1	6	0	0	0	0	0	0	0	0	0	0	0	0	0	0	0	0	0	0	0	0
Listonella	1	38	0	0	0	0	0	0	0	0	0	0	0	0	0	0	0	0	0	0	0	0
Loktanella	1	3	0	0	0	0	0	0	0	1	0	0	0	0	0	0	0	0	0	0	0	0
Lysinibacillus	2	97	0	0	0	0	0	1	1	1	1	1	0	0	1	1	1	0	0	0	0	0
Macrococcus	2	60	0	0	0	0	0	0	1	0	1	0	0	0	0	1	1	0	0	0	0	0
Magnetococcus	2	64	0	0	0	0	0	0	0	1	0	0	0	0	0	1	0	0	0	0	0	0
Magnetospirillum	2	113	0	0	0	0	0	1	0	0	1	0	0	0	0	1	0	0	0	0	0	0
Mahella	2	58	0	0	0	0	0	1	0	0	0	0	0	0	0	1	1	0	0	0	0	1
Mannheimia	2	85	0	0	0	0	0	0	0	0	0	0	0	0	0	1	1	0	0	0	1	1
Maribacter	3	87	0	0	0	0	0	0	0	0	1	0	0	0	0	1	1	0	1	0	0	1
Maricaulis	3	87	0	0	0	0	0	0	0	0	0	0	0	0	0	1	0	0	0	0	0	0
Marichromatium	1	2	0	0	0	0	0	0	0	0	0	0	0	0	0	0	0	0	0	0	0	0
Marinithermus	2	77	0	0	0	0	0	1	0	0	1	0	0	0	0	1	1	0	0	0	1	1
Marinitoga	1	15	0	0	0	0	0	0	0	0	0	0	0	0	0	0	0	0	0	0	0	0
Marinobacter	2	163	0	0	0	0	0	0	1	1	1	0	0	0	0	1	0	0	0	0	1	1
Marinomonas	3	192	0	0	0	0	0	1	1	1	1	1	0	0	0	1	1	0	0	0	0	1
Maritimibacter	1	7	0	0	0	0	0	0	0	1	0	0	0	0	0	0	0	0	0	0	0	0
Marivirga	2	96	0	0	0	0	0	0	0	0	1	0	0	0	0	1	1	1	0	0	0	1
Martelella	1	3	0	0	0	0	0	0	0	1	0	0	0	0	0	0	0	0	0	0	0	0
Marvinbryantia	1	1	0	0	0	0	0	0	0	0	0	0	0	0	0	0	0	0	0	0	0	0
Massilia	1	4	0	0	0	0	0	0	0	0	0	0	0	0	0	0	0	0	0	0	0	0
Mastigocladopsis	1	16	0	0	0	0	0	0	0	1	0	0	0	0	0	0	0	0	0	0	0	0
Megamonas	1	47	0	0	0	0	0	1	0	0	0	0	0	0	0	1	0	0	0	0	1	1
Megasphaera	1	14	0	0	0	0	0	0	0	0	0	0	0	0	0	0	0	0	0	0	0	0
Meiothermus	2	110	0	0	0	0	0	1	0	0	1	0	0	0	1	1	1	0	0	0	1	1
Melissococcus	2	46	0	0	0	0	0	0	0	0	0	0	0	0	0	1	1	0	0	0	0	0
Mesoflavibacter	1	6	0	0	0	0	0	0	0	0	0	0	0	0	0	0	0	0	0	0	0	0
Mesoplasma	1	23	0	0	0	0	0	1	0	0	0	0	0	0	0	1	1	0	0	0	0	0
Mesorhizobium	2	201	0	0	0	0	1	1	1	1	1	1	0	0	1	1	1	0	0	0	1	1
Mesotoga	1	14	0	0	0	0	0	0	0	0	0	0	0	0	0	0	0	0	0	0	0	0
Metallosphaera	1	18	0	0	0	0	0	0	0	0	0	0	0	0	0	0	0	0	0	0	0	0
Methanobacterium	1	7	0	0	0	0	0	0	0	0	0	0	0	0	0	0	0	0	0	0	0	0
Methanobrevibacter	1	9	0	0	0	0	0	0	0	0	0	0	0	0	0	0	0	0	0	0	0	0
Methanocaldococcus	1	6	0	0	0	0	0	0	0	0	0	0	0	0	0	0	0	0	0	0	0	0
Methanocella	1	15	0	0	0	0	0	0	0	0	0	0	0	0	0	0	0	0	0	0	0	0
Methanococcaceae	1	1	0	0	0	0	0	0	0	0	0	0	0	0	0	0	0	0	0	0	0	0
Methanococcoides	1	5	0	0	0	0	0	0	0	0	0	0	0	0	0	0	0	0	0	0	0	0
Methanococcus	1	9	0	0	0	0	0	0	0	0	0	0	0	0	0	0	0	0	0	0	0	0
Methanocorpusculum	1	7	0	0	0	0	0	0	0	0	0	0	0	0	0	0	0	0	0	0	0	0
Methanoculleus	1	8	0	0	0	0	0	0	0	0	0	0	0	0	0	0	0	0	0	0	0	0
Methanohalobium	1	5	0	0	0	0	0	0	0	0	0	0	0	0	0	0	0	0	0	0	0	0
Methanohalophilus	1	5	0	0	0	0	0	0	0	0	0	0	0	0	0	0	0	0	0	0	0	0
Methanolobus	1	5	0	0	0	0	0	0	0	0	0	0	0	0	0	0	0	0	0	0	0	0
Methanomethylovorans	1	5	0	0	0	0	0	0	0	0	0	0	0	0	0	0	0	0	0	0	0	0
Methanoplanus	1	7	0	0	0	0	0	0	0	0	0	0	0	0	0	0	0	0	0	0	0	0
Methanopyrus	1	4	0	0	0	0	0	0	0	0	0	0	0	0	0	0	0	0	0	0	0	0
Methanoregula	1	8	0	0	0	0	0	0	0	0	0	0	0	0	0	0	0	0	0	0	0	0
Methanosaeta	1	9	0	0	0	0	0	0	0	0	0	0	0	0	0	0	0	0	0	0	0	0
Methanosalsum	1	7	0	0	0	0	0	0	0	0	0	0	0	0	0	0	0	0	0	0	0	0

Genus	Cluster Number	Protein Counts	3.1.3.41	3.1.8.1	3.2.1.31	3.3.2.10	3.3.2.9	3.5.1.-	3.5.1.4	3.5.1.5	3.5.1.54	3.5.1.6	3.5.2.-	3.5.2.15	3.5.2.2	3.5.4.-	3.5.4.5	3.5.5.1	3.5.5.7	3.5.99.3	3.6.1.7	3.7.1.-
Methanosarcina	1	18	0	0	0	0	0	0	0	0	0	0	0	0	0	0	0	0	0	0	0	0
Methanosphaera	1	7	0	0	0	0	0	0	0	0	0	0	0	0	0	0	0	0	0	0	0	0
Methanosphaerula	1	8	0	0	0	0	0	0	0	0	0	0	0	0	0	0	0	0	0	0	0	0
Methanospirillum	1	6	0	0	0	0	0	0	0	0	0	0	0	0	0	0	0	0	0	0	0	0
Methanothermobacter	1	6	0	0	0	0	0	0	0	0	0	0	0	0	0	0	0	0	0	0	0	0
Methanothermococcus	1	5	0	0	0	0	0	0	0	0	0	0	0	0	0	0	0	0	0	0	0	0
Methanothermus	1	5	0	0	0	0	0	0	0	0	0	0	0	0	0	0	0	0	0	0	0	0
Methanotorris	1	5	0	0	0	0	0	0	0	0	0	0	0	0	0	0	0	0	0	0	0	0
Methylacidiphilum	2	48	0	0	0	0	0	0	0	0	0	0	0	0	1	1	0	0	0	0	1	0
Methylarcula	1	2	0	0	0	0	0	0	0	0	0	0	0	0	0	0	0	0	0	0	0	0
Methylibium	1	38	0	0	0	0	0	0	0	0	0	0	0	0	0	0	0	0	0	0	0	0
Methylobacillus	2	61	0	0	0	0	0	0	1	1	0	0	0	0	0	1	0	0	0	0	0	0
Methylobacterium	3	236	0	0	0	0	1	1	1	1	1	1	0	1	1	1	1	1	1	1	1	1
Methylocella	3	117	0	0	0	0	0	0	0	1	1	0	0	0	0	1	1	0	1	0	1	0
Methylococcus	2	80	0	0	0	0	0	0	0	0	1	0	0	0	0	1	0	0	0	0	1	0
Methylocystis	1	21	0	0	0	0	0	0	0	0	0	0	0	0	0	0	0	0	0	0	0	0
Methyloferula	1	2	0	0	0	0	0	0	0	0	0	0	0	0	0	0	0	0	0	0	0	0
Methylomicrobium	1	12	0	0	0	0	0	0	0	0	0	0	0	0	0	0	0	0	0	0	0	0
Methylomonas	1	15	0	0	0	0	0	0	0	0	0	0	0	0	0	0	0	0	0	0	0	0
Methylophaga	1	7	0	0	0	0	0	0	0	0	0	0	0	0	0	0	0	0	0	0	0	0
Methylophilus	1	2	0	0	0	0	0	0	0	1	0	0	0	0	0	0	0	0	0	0	0	0
Methylosinus	1	2	0	0	0	0	0	0	0	0	0	0	0	0	0	0	0	0	0	0	0	0
Methylotenera	2	72	0	0	0	0	0	1	0	1	1	0	0	0	0	1	0	0	0	0	0	0
Methyloversatilis	1	5	0	0	0	0	0	0	0	0	0	0	0	0	0	0	0	0	0	0	0	0
Methylovorus	2	66	0	0	0	0	0	0	0	1	1	0	0	0	0	1	0	0	0	0	0	0
Micavibrio	1	9	0	0	0	0	0	0	0	0	0	0	0	0	0	0	0	0	0	0	0	0
Microbacterium	2	115	1	0	0	0	1	0	0	0	1	0	0	0	1	1	1	0	0	0	1	0
Microchaete	1	23	0	0	0	0	0	0	0	1	0	0	0	0	0	0	0	0	0	0	0	0
Micrococcus	2	72	1	0	0	0	1	0	0	1	1	0	0	0	0	1	1	0	0	0	0	0
Microcoleus	1	17	0	0	0	0	0	0	0	1	0	0	0	0	0	0	0	0	0	0	0	0
Microcystis	2	67	0	0	0	0	0	0	0	1	0	0	0	0	0	1	1	0	0	0	0	1
Microlunatus	1	25	0	0	0	0	0	0	0	0	0	0	0	0	0	0	0	0	0	0	0	0
Micromonospora	2	115	1	0	0	0	1	1	1	1	1	0	0	0	0	1	1	0	0	0	0	0
Microvirga	1	6	0	0	0	0	0	0	0	0	0	0	0	0	0	0	0	0	0	0	0	0
Mobiluncus	1	41	0	0	0	0	0	1	0	0	0	0	0	0	0	1	0	0	0	0	0	0
Modestobacter	1	43	0	0	0	0	0	0	0	0	0	0	0	0	0	0	0	0	0	0	0	0
Moorea	1	2	0	0	0	0	0	0	0	0	0	0	0	0	0	0	0	0	0	0	0	0
Moorella	2	69	0	0	0	0	0	0	0	0	0	0	0	0	1	1	1	0	0	0	1	0
Moraxella	2	66	0	0	0	0	0	1	0	0	0	0	0	0	0	1	0	0	0	0	0	0
Morganella	1	29	0	0	0	0	0	0	0	0	0	0	0	0	0	0	0	0	0	0	0	0
Moritella	1	2	0	0	0	0	0	0	0	0	0	0	0	0	0	0	0	0	0	0	0	0
Muricauda	1	23	0	0	0	0	0	0	0	0	0	0	0	0	0	0	0	0	0	0	0	1
Mycobacterium	3	306	1	0	0	0	1	1	1	1	1	1	0	0	1	1	1	0	1	0	1	1
Mycoplasma	1	53	0	0	0	0	0	0	0	0	0	0	0	0	0	1	1	0	0	0	0	1
Myxococcus	2	110	0	0	0	0	1	1	0	0	1	0	0	0	0	1	1	0	0	0	1	0
Nakamurella	2	115	1	0	0	0	1	1	0	1	1	0	0	0	0	1	1	0	0	0	1	1
napthalene	1	2	0	0	0	0	0	0	0	0	0	0	0	0	0	0	0	0	0	0	0	0
Natranaerobius	2	55	0	0	0	0	0	1	1	0	1	0	0	0	0	1	1	1	0	0	1	0
Natrialba	1	16	0	0	0	0	0	0	0	0	0	0	0	0	0	0	0	0	0	0	0	0
Natrinema	1	16	0	0	0	0	0	0	0	0	0	0	0	0	0	0	0	0	0	0	0	0
Natronobacterium	1	11	0	0	0	0	0	0	0	0	0	0	0	0	0	0	0	0	0	0	0	0
Natronococcus	1	21	0	0	0	0	0	0	0	0	0	0	0	0	0	0	0	0	0	0	0	0

Genus	Cluster Number	Protein Counts	3.1.3.41	3.1.8.1	3.2.1.31	3.3.2.10	3.3.2.9	3.5.1.-	3.5.1.4	3.5.1.5	3.5.1.54	3.5.1.6	3.5.2.-	3.5.2.15	3.5.2.2	3.5.4.-	3.5.4.5	3.5.5.1	3.5.5.7	3.5.99.3	3.6.1.7	3.7.1.-
Natronomonas	1	20	0	0	0	0	0	0	0	0	0	0	0	0	0	0	0	0	0	0	0	0
Nautilia	2	48	0	0	0	0	0	0	0	0	1	0	0	0	0	0	0	0	0	0	1	0
Neisseria	2	67	0	0	0	0	0	0	0	0	1	0	0	0	0	1	0	0	0	0	0	0
Neisseriaceae	1	1	0	0	0	0	0	0	0	0	0	0	0	0	0	0	0	0	0	0	0	0
Neorickettsia	1	31	0	0	0	0	0	0	0	0	0	0	0	0	0	1	0	0	0	0	0	0
Neptuniibacter	1	2	0	0	0	0	0	0	0	1	0	0	0	0	0	0	0	0	0	0	0	0
Niastella	1	21	0	0	0	0	0	0	0	0	0	0	0	0	0	0	0	0	0	0	0	0
Nitratifractor	2	54	0	0	0	0	0	1	1	0	0	0	0	0	0	1	0	0	0	0	1	0
Nitratireductor	1	19	0	0	0	0	0	0	0	1	0	0	0	0	0	0	0	0	0	0	0	0
Nitratiruptor	2	40	0	0	0	0	0	0	0	0	0	0	0	0	0	0	0	0	0	0	0	0
Nitrobacter	2	99	0	0	0	0	0	0	0	0	1	0	0	0	0	1	0	0	0	0	1	0
Nitrococcus	1	2	0	0	0	0	0	0	0	0	0	0	0	0	0	0	0	0	0	0	0	0
Nitrosococcus	2	94	0	0	0	0	0	1	0	1	0	0	0	0	0	1	0	0	0	0	1	0
Nitrosomonas	2	72	0	0	0	0	0	0	0	0	0	0	0	0	0	1	0	0	0	0	0	0
Nitrosopumilaceae	1	7	0	0	0	0	0	0	0	0	0	0	0	0	0	0	0	0	0	0	0	0
Nitrosopumilus	1	11	0	0	0	0	0	0	0	0	0	0	0	0	0	0	0	0	0	0	0	0
Nitrosospira	2	74	0	0	0	0	0	1	0	1	1	0	0	0	0	1	0	0	0	0	0	0
Nocardia	2	185	1	0	0	0	1	1	1	1	1	0	0	0	0	1	1	0	1	0	1	1
Nocardioidaceae	1	2	0	0	0	0	0	0	0	0	0	0	0	0	0	0	0	0	0	0	0	0
Nocardioides	2	157	1	0	0	0	1	1	1	0	1	0	0	1	1	1	1	0	0	1	1	1
Nocardiopsis	2	102	1	0	0	0	1	0	1	0	1	0	0	0	1	1	1	0	0	0	1	0
Nodularia	1	22	0	0	0	0	0	0	0	1	0	0	0	0	0	0	0	0	0	0	0	0
Nonlabens	1	19	0	0	0	0	0	0	0	0	0	0	0	0	0	0	0	0	0	0	0	0
Nostoc	2	111	0	0	0	0	1	0	1	1	1	0	0	0	0	1	0	0	0	0	1	0
Nostocaceae	1	2	0	0	0	0	0	0	0	0	0	0	0	0	0	0	0	0	0	0	0	0
Novosphingobium	2	169	1	0	0	0	1	0	1	1	1	0	0	0	0	1	1	0	0	0	0	1
Oceanibaculum	1	3	0	0	0	0	0	0	0	1	0	0	0	0	0	0	0	0	0	0	0	0
Oceanibulbus	1	12	0	0	0	0	0	0	0	1	0	0	0	0	0	0	0	0	0	0	0	0
Oceanicola	1	9	0	0	0	0	0	0	0	1	0	0	0	0	0	0	0	0	0	0	0	0
Oceanimonas	1	33	0	0	0	0	0	0	0	0	0	0	0	0	0	0	0	0	0	0	0	0
Oceaniovalibus	1	2	0	0	0	0	0	0	0	0	0	0	0	0	0	0	0	0	0	0	0	0
Oceanithermus	2	72	0	0	0	0	0	1	0	0	1	0	0	0	0	1	1	0	0	0	1	0
Oceanobacillus	2	91	0	0	0	0	0	0	0	0	1	0	0	0	0	1	1	0	0	0	1	0
Oceanospirillum	1	2	0	0	0	0	0	0	0	1	0	0	0	0	0	0	0	0	0	0	0	0
Ochrobactrum	2	133	0	0	0	0	0	0	1	1	0	1	1	0	1	1	0	0	0	0	0	1
Octadecabacter	1	29	0	0	0	0	0	0	0	1	0	0	0	0	0	0	0	0	0	0	0	0
Oenococcus	2	41	0	0	0	0	0	0	0	0	0	0	0	0	0	1	1	0	0	0	0	0
Oleomonas	1	2	0	0	0	0	0	0	0	0	0	0	0	0	0	0	0	0	0	0	0	0
Oligotropha	3	87	0	0	0	0	1	0	0	0	1	1	0	0	0	1	0	0	0	0	1	0
Olsenella	2	40	0	0	0	0	0	0	0	0	0	0	0	0	0	1	1	0	0	0	1	0
Opitutus	2	67	0	0	1	0	1	0	0	1	1	0	0	0	0	1	1	0	0	0	0	0
Orientia	1	21	0	0	0	0	0	0	0	0	0	0	0	0	0	1	0	0	0	0	0	0
Ornithobacterium	1	11	0	0	0	0	0	0	0	0	0	0	0	0	0	0	0	0	0	0	0	0
Oscillatoria	1	16	0	0	0	0	0	0	0	1	0	0	0	0	0	0	0	0	0	0	0	0
Oscillatoriales	1	2	0	0	0	0	0	0	0	1	0	0	0	0	0	0	0	0	0	0	0	0
Oscillibacter	1	20	0	0	0	0	0	0	0	0	0	0	0	0	0	0	0	0	0	0	0	0
Owenweeksia	1	15	0	0	0	0	0	0	0	0	0	0	0	0	0	0	0	0	0	0	0	0
Oxalobacteraceae	1	4	0	0	0	0	0	0	0	0	0	0	0	0	0	0	0	0	0	0	0	0
Paenibacillus	2	152	0	0	0	0	1	1	1	1	1	1	0	1	1	1	1	0	0	0	0	1
Paludibacter	2	67	0	0	0	0	0	0	0	0	0	0	0	0	0	1	1	0	0	0	0	0
Pandoraea	1	18	0	0	0	0	0	0	1	1	0	0	0	0	0	0	0	0	0	0	0	0
Pannonibacter	1	3	0	0	0	0	0	0	0	1	0	0	0	0	0	0	0	0	0	0	0	0

Genus	Cluster Number	Protein Counts	3.1.3.41	3.1.8.1	3.2.1.31	3.3.2.10	3.3.2.9	3.5.1.-	3.5.1.4	3.5.1.5	3.5.1.54	3.5.1.6	3.5.2.-	3.5.2.15	3.5.2.2	3.5.4.-	3.5.4.5	3.5.5.1	3.5.5.7	3.5.99.3	3.6.1.7	3.7.1.-
Pantholops	1	23	0	0	0	0	0	0	0	0	0	0	0	0	0	0	0	0	0	0	0	0
Pantoea	2	181	0	0	0	0	1	1	1	0	1	1	0	0	1	1	1	1	1	1	1	1
Parabacteroides	2	64	0	0	0	0	0	0	0	0	0	0	0	0	0	1	1	0	0	0	0	0
Parachlamydia	1	11	0	0	0	0	0	0	0	0	0	0	0	0	0	0	0	0	0	0	0	0
Paracoccus	2	169	0	0	0	0	0	1	1	1	1	1	0	0	1	1	1	0	0	0	0	1
Parvibaculum	3	117	0	0	0	0	1	1	0	0	1	0	0	0	0	1	0	0	0	0	0	0
Parvularcula	2	72	0	0	0	0	0	1	0	0	1	0	0	0	0	1	0	0	0	0	0	0
Pasteurella	2	85	0	0	0	0	0	1	0	1	0	0	0	0	0	1	1	0	0	0	1	0
Pasteurellaceae	1	2	0	0	0	0	0	0	0	0	0	0	0	0	0	0	0	0	0	0	0	0
Pectobacterium	1	44	0	0	0	0	0	0	0	0	0	0	0	0	0	0	0	0	0	0	0	0
Pediococcus	1	43	0	0	0	0	0	0	0	0	0	0	0	0	0	1	0	0	0	0	0	0
Pedobacter	2	88	0	0	0	0	0	0	0	0	0	0	0	0	0	1	0	0	0	0	1	1
Pelagibaca	1	10	0	0	0	0	0	0	0	0	0	0	0	0	0	0	0	0	0	0	0	0
Pelagibacterium	1	39	0	0	0	0	0	0	0	1	0	0	0	0	0	0	0	0	0	0	0	0
Pelobacter	2	85	0	0	0	0	0	0	1	0	0	0	0	0	0	1	1	0	0	0	1	0
Pelodictyon	2	62	0	0	0	0	0	0	0	0	0	0	0	0	0	1	0	0	0	0	1	0
Pelosinus	1	1	0	0	0	0	0	0	0	0	0	0	0	0	0	0	0	0	0	0	0	0
Pelotomaculum	2	60	0	0	0	0	0	0	0	0	1	0	0	0	0	1	1	0	0	0	1	0
Peptostreptococcus	1	1	0	0	0	0	0	0	0	0	0	0	0	0	0	0	0	0	0	0	0	0
Persephonella	2	55	0	0	0	0	0	0	0	0	0	0	0	0	0	1	0	0	0	0	1	0
Petrotoga	2	72	0	0	0	0	0	0	1	0	0	0	0	0	1	0	1	0	0	0	1	0
Phaeobacter	2	55	0	0	0	0	0	0	0	1	0	1	0	0	0	0	0	0	0	0	0	0
Phenylobacterium	3	103	0	0	0	0	1	0	1	0	0	0	0	0	0	0	0	0	0	0	1	0
Photobacterium	2	119	0	0	0	0	1	0	0	0	1	0	0	0	0	1	1	0	0	1	1	1
Photorhabdus	2	149	0	0	0	0	0	0	0	1	1	0	0	0	0	1	1	0	1	0	1	1
Phycisphaera	1	9	0	0	0	0	0	0	0	0	0	0	0	0	0	0	0	0	0	0	0	0
Phyllobacterium	1	6	0	0	0	0	0	0	0	1	0	0	0	0	0	0	0	0	0	0	0	0
Picrophilus	1	15	0	0	0	0	0	0	0	0	0	0	0	0	0	0	0	0	0	0	0	0
Pirellula	2	85	0	0	0	0	1	1	1	0	0	0	0	0	0	1	1	1	0	0	0	0
Planctomyces	2	93	0	0	0	0	0	1	1	0	0	0	0	0	0	1	0	0	0	0	0	0
Plautia	1	17	0	0	0	0	0	0	0	0	0	0	0	0	0	0	0	0	0	0	0	0
Plesiomonas	1	4	0	0	0	0	0	0	0	0	0	0	0	0	0	0	0	0	0	0	0	0
Pleurocapsa	1	23	0	0	0	0	0	0	0	1	0	0	0	0	0	0	0	0	0	0	0	0
Polaribacter	2	74	0	0	0	0	0	0	0	0	1	0	0	0	0	1	1	0	1	0	0	1
Polaromonas	3	238	0	0	0	0	0	1	1	1	1	1	0	0	1	0	0	0	1	0	1	1
Polymorphum	3	173	0	0	0	0	0	1	0	1	1	1	0	0	1	1	1	0	0	1	1	0
Polynucleobacter	1	19	0	0	0	0	0	0	0	0	0	0	0	0	0	0	0	0	0	0	0	0
Ponticaulis	1	1	0	0	0	0	0	0	0	0	0	0	0	0	0	0	0	0	0	0	0	0
Porphyromonas	2	63	0	0	0	0	0	1	0	0	0	0	0	0	0	1	1	0	0	0	0	0
Prevotella	1	65	0	0	1	0	0	1	0	0	0	0	0	0	0	1	1	0	0	0	0	0
Prochlorococcus	2	68	0	0	0	0	0	0	0	1	0	0	0	0	0	1	0	0	1	0	0	0
Propionibacterium	1	62	1	0	1	0	0	0	0	0	1	0	0	0	0	1	1	0	0	0	1	1
Prosthecochloris	1	16	0	0	0	0	0	0	0	0	0	0	0	0	0	0	0	0	0	0	0	0
Proteobacteria	1	19	0	0	0	0	0	0	0	0	0	0	0	0	0	0	0	0	0	0	0	0
Proteus	2	113	0	0	0	0	0	0	0	1	1	0	0	0	0	1	1	1	0	0	1	0
Providencia	1	38	0	0	0	0	0	0	0	1	0	0	0	0	0	0	0	0	0	0	0	0
Pseudaminobacter	1	10	0	0	0	0	0	0	0	1	0	0	0	0	0	0	0	0	0	0	0	0
Pseudanabaena	1	11	0	0	0	0	0	0	0	0	0	0	0	0	0	0	0	0	0	0	0	0
Pseudoalteromonas	2	188	0	0	0	0	1	1	0	1	1	0	0	0	0	1	1	1	0	0	0	1
Pseudochrobactrum	1	7	0	0	0	0	0	0	0	0	0	0	0	0	0	0	0	0	0	0	0	0
Pseudogulbenkiania	1	40	0	0	0	0	0	0	0	0	0	0	0	0	0	0	0	0	0	0	0	0
pseudomallei	3	131	0	0	0	0	1	1	0	1	1	1	1	0	0	1	1	0	0	0	1	1

Genus	Cluster Number	Protein Counts	3.1.3.41	3.1.8.1	3.2.1.31	3.3.2.10	3.3.2.9	3.5.1.-	3.5.1.4	3.5.1.5	3.5.1.54	3.5.1.6	3.5.2.-	3.5.2.15	3.5.2.2	3.5.4.-	3.5.4.5	3.5.5.1	3.5.5.7	3.5.99.3	3.6.1.7	3.7.1.-
Pseudomonas	3	381	0	0	0	0	1	1	1	1	1	1	0	0	1	1	0	1	1	1	1	1
Pseudonocardia	3	180	1	0	0	0	1	1	1	1	1	0	0	0	1	1	0	0	1	0	1	0
Pseudorhodobacter	1	4	0	0	0	0	0	0	0	0	0	0	0	0	0	0	0	0	0	0	0	0
Pseudovibrio	1	33	0	0	0	0	0	0	0	0	0	0	0	0	0	0	0	0	0	0	0	0
Pseudoxanthomonas	2	124	0	0	0	0	0	1	0	0	0	0	0	0	0	1	0	0	0	0	1	1
Psychrobacter	2	124	0	0	0	0	0	0	0	1	1	0	0	0	0	1	0	0	0	1	0	0
Psychroflexus	1	22	0	0	0	0	0	0	0	0	0	0	0	0	0	0	0	0	0	0	0	0
Psychromonas	2	110	0	0	0	0	0	0	0	1	1	0	0	0	0	1	1	0	0	0	1	0
Pusillimonas	3	111	0	0	0	0	0	0	1	0	1	0	0	0	1	1	0	0	0	0	0	0
Pyrobaculum	1	15	0	0	0	0	0	0	0	0	0	0	0	0	0	0	0	0	0	0	0	0
Pyrococcus	1	10	0	0	0	0	0	0	0	0	0	0	0	0	0	0	0	0	0	0	0	0
Pyrolobus	1	4	0	0	0	0	0	0	0	0	0	0	0	0	0	0	0	0	0	0	0	0
Rahnella	2	143	0	0	0	0	0	1	0	0	1	0	0	0	0	1	1	1	0	1	1	0
Ralstonia	3	332	0	0	0	0	1	1	0	1	1	1	0	0	1	1	0	1	1	1	1	1
Ramlibacter	1	35	0	0	0	0	0	0	0	1	0	0	0	0	0	0	0	0	0	0	0	0
Raoultella	2	139	0	0	0	0	0	0	0	1	0	0	0	0	0	0	0	0	0	0	0	1
Raphidiopsis	1	5	0	0	0	0	0	0	0	0	0	0	0	0	0	0	0	0	0	0	0	0
Reinekea	1	2	0	0	0	0	0	0	0	1	0	0	0	0	0	0	0	0	0	0	0	0
Renibacterium	2	67	1	0	0	0	1	0	0	0	1	0	0	0	0	1	0	0	0	0	0	1
Rhizobiaceae	1	3	0	0	0	0	0	0	0	0	0	0	0	0	0	0	0	0	0	0	0	1
Rhizobiales	1	6	0	0	0	0	0	0	0	1	0	0	0	0	0	0	0	0	0	0	0	0
Rhizobium	3	257	0	0	1	0	1	1	1	1	1	1	0	1	1	1	1	0	1	1	1	1
Rhizobium/Agrobacterium	2	87	0	0	0	0	1	0	0	1	0	0	0	0	0	1	0	0	0	0	0	1
Rhodanobacter	1	21	0	0	0	0	0	0	0	0	0	0	0	0	0	0	0	0	0	0	0	0
Rhodobacter	3	175	0	0	0	0	0	1	1	1	1	1	0	0	1	1	1	0	0	0	0	1
Rhodobacteraceae	1	21	0	0	0	0	0	0	0	0	0	0	0	0	1	0	0	0	0	0	0	0
Rhodobacterales	1	34	0	0	0	0	0	0	0	1	0	0	0	0	0	0	0	0	0	0	0	0
Rhodococcus	2	292	1	0	0	0	1	1	1	1	1	0	0	0	0	1	1	0	0	0	1	1
Rhodocyclaceae	1	7	0	0	0	0	0	0	0	1	0	0	0	0	0	0	0	0	0	0	0	0
Rhodoferax	3	144	0	0	0	0	0	1	0	1	1	1	0	0	0	0	0	0	0	0	1	0
Rhodomicrobium	2	103	0	0	0	0	0	1	0	0	1	0	0	0	0	1	0	0	0	0	0	0
Rhodopirellula	2	87	0	0	0	0	0	0	1	0	0	0	0	0	0	1	1	0	0	0	0	0
Rhodopseudomonas	3	246	0	0	0	0	1	1	1	1	1	1	1	0	0	1	1	1	0	0	1	1
Rhodospirillum	2	121	0	0	0	0	1	0	1	0	1	0	1	0	0	1	1	0	0	0	1	0
Rhodothermus	2	76	0	0	0	0	0	0	1	0	0	0	0	0	0	1	0	0	0	0	1	1
Rhodovulum	1	4	0	0	0	0	0	0	0	1	0	0	0	0	0	0	0	0	0	0	0	0
Richelia	1	2	0	0	0	0	0	0	0	0	0	0	0	0	0	0	0	0	0	0	0	0
Rickettsia	2	43	0	0	0	0	0	0	0	0	0	0	0	0	0	1	0	0	0	0	0	0
Rickettsiaceae	1	5	0	0	0	0	0	0	0	0	0	0	0	0	0	1	0	0	0	0	0	0
Riemerella	2	61	0	0	0	0	0	0	0	1	0	0	0	0	0	1	1	0	0	0	0	1
Rivularia	1	18	0	0	0	0	0	0	0	0	0	0	0	0	0	0	0	0	0	0	0	0
Robiginitalea	2	83	0	0	0	0	0	0	0	0	1	0	0	0	0	1	1	0	0	0	0	1
Roseburia	2	58	0	0	1	0	0	1	0	0	1	0	0	0	0	1	1	0	0	0	1	0
Roseibium	1	3	0	0	0	0	0	0	0	0	0	0	0	0	0	0	0	0	0	0	0	0
Roseiflexus	3	100	0	0	0	0	0	1	0	0	0	0	0	0	0	0	1	0	0	0	1	0
Roseobacter	2	149	0	0	0	0	0	0	1	1	1	1	0	0	1	1	1	0	0	0	0	1
Roseomonas	1	2	0	0	0	0	0	0	0	1	0	0	0	0	0	0	0	0	0	0	0	0
Roseovarius	1	15	0	0	0	0	0	0	0	1	0	0	0	0	0	0	0	0	0	0	0	0
Rothia	1	44	1	0	0	0	1	0	0	0	0	0	0	0	0	1	0	0	0	0	1	0
Rubrivivax	1	24	0	0	0	0	0	0	0	0	0	0	0	0	0	0	0	0	0	0	0	0
Rubrobacter	3	117	0	0	0	0	0	1	1	0	0	1	0	0	1	1	1	0	0	0	1	1
Ruegeria	2	189	0	0	0	0	1	1	1	1	1	1	0	0	1	1	1	1	0	0	0	1

Genus	Cluster Number	Protein Counts	3.1.3.41	3.1.8.1	3.2.1.31	3.3.2.10	3.3.2.9	3.5.1.-	3.5.1.4	3.5.1.5	3.5.1.54	3.5.1.6	3.5.2.-	3.5.2.15	3.5.2.2	3.5.4.-	3.5.4.5	3.5.5.1	3.5.5.7	3.5.99.3	3.6.1.7	3.7.1.-
Ruminococcaceae	1	2	0	0	0	0	0	0	0	0	0	0	0	0	0	0	0	0	0	0	0	0
Ruminococcus	2	101	0	0	0	0	0	1	0	1	0	1	0	0	1	1	1	0	0	0	1	1
Runella	1	26	0	0	0	0	0	0	0	0	0	0	0	0	0	0	0	0	0	0	0	0
Saccharomonospora	2	132	1	0	0	0	1	1	1	0	1	0	0	0	0	1	1	0	1	0	1	1
Saccharophagus	2	81	0	0	0	0	0	0	0	1	1	0	0	0	0	1	0	0	0	0	1	0
Saccharopolyspora	3	194	1	0	0	0	1	1	1	1	1	0	0	0	1	1	1	0	0	0	1	1
Saccharothrix	1	32	0	0	0	0	0	0	0	0	0	0	0	0	0	0	0	0	0	0	0	0
Sagittula	1	10	0	0	0	0	0	0	0	1	0	0	0	0	0	0	0	0	0	0	0	0
Salinibacter	2	77	0	0	0	0	0	0	1	0	0	0	0	0	0	1	1	0	0	0	1	1
Salinimonas	1	1	0	0	0	0	0	0	0	0	0	0	0	0	0	0	0	0	0	0	0	0
Salinispora	3	127	1	0	0	1	1	1	1	0	1	0	0	0	0	1	1	0	0	0	0	1
Salinivibrio	1	2	0	0	0	0	0	0	0	0	0	0	0	0	0	0	0	0	0	0	0	0
Salmonella	2	151	0	0	1	0	0	0	0	0	1	0	0	0	0	1	1	0	0	0	1	1
Sanguibacter	2	73	1	0	0	0	1	0	1	0	1	0	0	0	1	1	1	0	0	0	0	0
Saprospira	1	13	0	0	0	0	0	0	0	0	0	0	0	0	0	0	0	0	0	0	0	0
Scardovia	1	1	0	0	0	0	0	0	0	0	0	0	0	0	0	0	0	0	0	0	0	0
Scytonema	1	10	0	0	0	0	0	0	0	1	0	0	0	0	0	0	0	0	0	0	0	0
Sebaldella	2	69	0	0	0	0	0	1	0	0	0	0	0	0	1	0	1	0	0	0	0	1
Segniliparus	2	85	1	0	0	0	1	1	0	1	1	1	0	0	1	1	1	0	0	0	0	0
Selenomonas	2	57	0	0	0	0	0	1	0	0	0	0	0	0	0	1	1	0	0	0	1	0
Serinicoccus	1	1	0	0	0	0	0	0	0	0	0	0	0	0	0	0	0	0	0	0	0	0
Serratia	2	186	0	0	1	0	1	0	0	0	1	1	1	0	0	1	1	1	0	0	1	1
Shewanella	3	214	0	0	0	0	1	1	1	1	1	0	0	0	0	1	1	1	1	0	1	1
Shigella	2	172	0	0	1	0	0	0	0	1	1	0	0	0	0	1	1	0	0	0	1	1
Shinella	1	2	0	0	0	0	0	0	0	0	0	0	0	0	0	0	0	0	0	0	0	0
Sideroxydans	2	81	0	0	0	0	0	1	0	0	1	0	0	0	0	1	0	0	0	0	1	0
Silicibacter	2	105	0	0	0	0	0	0	1	1	1	1	0	0	1	0	1	0	0	0	0	0
Simiduia	1	18	0	0	0	0	0	0	0	0	0	0	0	0	0	0	0	0	0	0	0	0
Simkania	1	6	0	0	0	0	0	0	0	0	0	0	0	0	0	0	0	0	0	0	0	0
Simonsiella	1	2	0	0	0	0	0	0	0	0	0	0	0	0	0	0	0	0	0	0	0	0
Singulisphaera	1	18	0	0	0	0	0	0	0	0	0	0	0	0	0	0	0	0	0	0	0	0
Sinorhizobium	3	207	0	0	0	0	1	1	1	1	1	1	0	0	1	1	1	0	0	0	1	1
Slackia	2	46	0	0	0	0	0	1	1	0	0	0	0	0	0	1	0	0	0	0	0	0
Smaragdicoccus	1	3	0	0	0	0	0	0	0	0	0	0	0	0	0	0	0	0	0	0	0	0
Sodalis	2	71	0	0	0	0	0	0	1	0	1	0	0	0	0	1	1	0	0	0	1	0
Solibacillus	1	23	0	0	0	0	0	0	0	0	0	0	0	0	0	0	0	0	0	0	0	0
Solitalea	1	13	0	0	0	0	0	0	0	0	0	0	0	0	0	0	0	0	0	0	0	0
Sorangium	3	115	0	0	0	0	1	0	1	1	1	0	0	0	1	1	1	0	1	0	1	0
Sphaerobacter	2	100	0	0	0	0	0	1	1	0	1	1	0	0	1	1	0	1	0	0	1	0
Sphaerochaeta	1	42	0	0	0	0	0	0	0	0	0	0	0	0	0	0	1	0	0	0	0	1
Sphingobacterium	3	85	0	0	0	0	0	0	0	0	1	0	0	0	0	1	0	0	0	0	1	1
Sphingobium	1	179	0	0	0	0	0	0	0	1	1	0	0	0	0	1	1	0	0	0	1	0
Sphingomonadaceae	1	15	0	0	0	0	0	0	0	0	0	0	0	0	0	0	0	0	0	0	0	0
Sphingomonas	2	220	0	0	0	0	1	1	1	0	0	0	0	0	0	1	1	0	1	0	1	1
Sphingopyxis	3	99	0	0	0	0	1	0	0	0	0	0	0	0	0	1	1	0	1	0	0	1
Spirochaeta	2	103	0	0	0	0	0	1	0	0	0	0	0	0	1	0	1	0	0	1	1	1
Spiroplasma	1	6	0	0	0	0	0	0	0	0	0	0	0	0	0	0	0	0	0	0	0	0
Spirosoma	2	107	0	0	0	0	1	1	1	0	0	0	0	0	0	1	0	1	0	0	0	1
Stackebrandtia	2	110	1	0	0	0	1	1	1	0	1	0	0	0	0	1	1	0	0	0	1	1
Stanieria	1	17	0	0	0	0	0	0	0	0	0	0	0	0	0	0	0	0	0	0	0	0
Staphylococcus	2	108	0	0	1	0	0	0	1	1	1	1	0	0	0	1	1	0	0	0	1	0
Staphylothermus	1	12	0	0	0	0	0	0	0	0	0	0	0	0	0	0	0	0	0	0	0	0

Genus	Cluster Number	Protein Counts	3.1.3.41	3.1.8.1	3.2.1.31	3.3.2.10	3.3.2.9	3.5.1.-	3.5.1.4	3.5.1.5	3.5.1.54	3.5.1.6	3.5.2.-	3.5.2.15	3.5.2.2	3.5.4.-	3.5.4.5	3.5.5.1	3.5.5.7	3.5.99.3	3.6.1.7	3.7.1.-
Stappia	1	2	0	0	0	0	0	0	0	1	0	0	0	0	0	0	0	0	0	0	0	0
Starkeya	3	134	0	0	0	0	1	1	1	1	1	1	0	0	1	1	0	0	1	1	1	1
Stenotrophomonas	3	104	0	0	0	0	1	0	0	0	0	0	0	0	0	1	0	0	0	0	0	0
Stigmatella	3	129	0	0	0	0	1	1	0	1	0	0	0	0	1	1	1	0	0	0	1	1
Streptobacillus	1	38	0	0	1	0	0	0	0	0	0	0	0	0	0	0	1	0	0	0	0	0
Streptococcus	2	121	0	0	1	0	0	1	1	1	0	0	0	0	0	1	1	0	0	0	1	0
Streptomyces	2	253	1	0	0	0	1	1	1	1	1	1	0	0	1	1	1	1	0	0	1	1
Streptosporangium	3	148	1	0	0	0	1	1	1	1	1	0	0	0	0	1	1	0	0	0	1	1
Strigomonas	1	2	0	0	0	0	0	0	0	0	0	0	0	0	0	0	0	0	0	0	0	0
Sulfitobacter	1	10	0	0	0	0	0	0	0	1	0	0	0	0	0	0	0	0	0	0	0	0
Sulfobacillus	1	30	0	0	0	0	0	0	0	0	0	0	0	0	0	0	0	0	0	0	0	0
Sulfolobus	1	23	0	0	0	0	0	0	0	0	0	0	0	0	0	0	0	0	0	0	0	0
Sulfuricurvum	2	67	0	0	0	0	0	1	1	0	1	0	0	0	0	1	0	0	0	0	0	0
Sulfurihydrogenibium	2	55	0	0	0	0	0	0	0	0	0	0	0	0	0	1	0	0	0	0	1	0
Sulfurimonas	2	60	0	0	0	0	0	1	1	0	0	0	0	0	0	1	0	0	0	0	0	0
Sulfurospirillum	1	48	0	0	0	0	0	1	0	0	1	0	0	0	0	0	0	0	0	0	1	1
Sulfurovum	2	48	0	0	0	0	0	0	1	0	0	0	0	0	0	0	0	0	0	0	1	0
Symbiobacterium	2	68	0	0	0	0	0	1	0	0	1	0	0	0	0	1	1	0	0	0	0	0
Synechococcus	1	34	0	0	0	0	0	0	0	0	0	0	0	0	0	0	0	0	0	0	0	0
Synechocystis	1	40	0	0	0	0	0	0	0	0	0	0	0	0	0	0	0	0	0	0	0	0
Synergistetes	1	27	0	0	0	0	0	1	1	0	1	0	0	0	0	0	0	0	0	0	0	0
Syntrophobacter	2	101	0	0	0	0	0	0	1	0	1	0	0	0	0	1	0	1	0	0	1	0
Syntrophobotulus	2	71	0	0	0	0	0	0	0	1	1	0	0	0	1	1	1	0	0	0	0	1
Syntrophomonas	2	58	0	0	0	0	0	1	0	0	0	0	0	0	0	1	1	0	0	0	0	0
Syntrophothermus	2	60	0	0	0	0	0	1	0	0	0	0	0	0	0	1	1	0	0	0	0	0
Syntrophus	2	66	0	0	0	0	0	0	0	0	0	0	0	0	0	1	1	0	0	0	1	0
Tannerella	1	8	0	0	0	0	0	0	0	0	0	0	0	0	0	0	0	0	0	0	0	0
Taylorella	1	43	0	0	0	0	0	0	0	0	0	0	0	0	0	1	0	0	0	0	0	0
Tepidanaerobacter	2	55	0	0	0	0	0	0	1	0	0	0	0	0	0	1	1	0	0	0	0	0
Teredinibacter	2	109	0	0	0	0	0	0	0	1	1	0	0	0	0	1	0	0	1	1	0	0
Terrabacter	1	1	0	0	0	0	0	0	0	0	0	0	0	0	0	0	0	0	0	0	0	0
Terriglobus	2	83	0	0	0	0	0	1	0	0	0	0	0	0	0	1	1	0	0	0	1	0
Tetragenococcus	1	12	0	0	0	0	0	0	0	0	0	0	0	0	0	0	0	0	0	0	0	0
Thalassiobium	1	11	0	0	0	0	0	0	0	1	0	0	0	0	1	0	0	0	0	0	0	0
Thalassobacter	1	9	0	0	0	0	0	0	0	0	0	0	0	0	0	0	0	0	0	0	0	0
Thalassolituus	1	12	0	0	0	0	0	0	0	0	0	0	0	0	0	0	0	0	0	0	0	0
Thalassospira	1	2	0	0	0	0	0	0	0	1	0	0	0	0	0	0	0	0	0	0	0	0
Thauera	1	111	0	0	0	0	0	0	0	1	0	0	0	0	0	1	0	0	0	0	0	0
Thermacetogenium	1	7	0	0	0	0	0	0	0	0	0	0	0	0	0	0	0	0	0	0	0	0
Thermaerobacter	2	79	0	0	0	0	0	1	1	0	1	0	0	0	0	1	0	0	0	0	1	0
Thermanaerovibrio	2	58	0	0	0	0	0	1	1	0	1	0	1	0	1	1	1	0	0	0	1	0
Thermincola	2	62	0	0	0	0	0	0	1	1	0	0	0	0	0	1	1	0	0	0	0	0
Thermoanaerobacter	2	76	0	0	0	0	0	1	1	0	1	0	0	0	0	1	1	0	0	0	1	0
Thermoanaerobacterium	2	64	0	0	0	0	0	1	1	0	0	0	0	0	0	1	1	0	0	0	1	0
Thermobacillus	1	11	0	0	0	0	0	0	0	0	0	0	0	0	0	0	0	0	0	0	0	0
Thermobaculum	2	62	0	0	0	0	0	0	0	0	0	0	0	0	0	0	0	0	0	0	1	1
Thermobifida	2	72	1	0	0	0	1	0	0	0	1	0	0	0	0	1	0	0	0	0	1	0
Thermobispora	2	109	1	0	0	0	1	1	0	1	1	0	0	0	0	1	1	0	0	0	1	1
Thermococcus	1	13	0	0	0	0	0	0	0	0	0	0	0	0	0	0	0	0	0	0	0	0
Thermocrinis	2	60	0	0	0	0	0	1	1	1	0	0	0	0	0	1	0	0	0	0	1	0
Thermodesulfatator	1	10	0	0	0	0	0	0	0	0	0	0	0	0	0	0	0	0	0	0	0	0
Thermodesulfobacterium	1	9	0	0	0	0	0	0	0	0	0	0	0	0	0	0	0	0	0	0	0	0

Genus	Cluster Number	Protein Counts	3.1.3.41	3.1.8.1	3.2.1.31	3.3.2.10	3.3.2.9	3.5.1.-	3.5.1.4	3.5.1.5	3.5.1.54	3.5.1.6	3.5.2.-	3.5.2.15	3.5.2.2	3.5.4.-	3.5.4.5	3.5.5.1	3.5.5.7	3.5.99.3	3.6.1.7	3.7.1.-
**Thermodesulfobium**	1	13	0	0	0	0	0	0	0	0	0	0	0	0	0	0	0	0	0	0	0	0
**Thermodesulfovibrio**	2	60	0	0	0	0	0	1	0	0	0	0	0	0	0	1	0	0	0	0	1	0
**Thermofilum**	1	9	0	0	0	0	0	0	0	0	0	0	0	0	0	0	0	0	0	0	0	0
**Thermogladius**	1	7	0	0	0	0	0	0	0	0	0	0	0	0	0	0	0	0	0	0	0	0
**Thermomicrobium**	2	92	0	0	0	0	1	1	1	1	1	0	0	0	0	0	0	0	0	0	1	0
**Thermomonospora**	2	125	1	0	0	0	1	0	0	1	0	0	0	0	0	1	1	0	0	0	1	1
**Thermoplasma**	1	11	0	0	0	0	0	0	0	0	0	0	0	0	0	0	0	0	0	0	0	0
**Thermoplasmatales**	1	6	0	0	0	0	0	0	0	0	0	0	0	0	0	0	0	0	0	0	0	0
**Thermoproteus**	1	12	0	0	0	0	0	0	0	0	0	0	0	0	0	0	0	0	0	0	0	0
**Thermosediminibacter**	2	64	0	0	0	0	0	1	1	0	1	0	0	0	0	1	1	0	0	0	1	0
**Thermosipho**	2	70	0	0	0	0	0	0	0	0	0	0	0	0	1	0	1	0	0	1	0	0
**Thermosphaera**	1	10	0	0	0	0	0	0	0	0	0	0	0	0	0	0	0	0	0	0	0	0
**Thermosynechococcus**	1	6	0	0	0	0	0	0	0	0	0	0	0	0	0	0	0	0	0	0	0	0
**Thermotoga**	2	80	0	0	0	0	0	0	0	0	0	0	0	0	0	1	1	0	0	0	1	0
**Thermovibrio**	2	58	0	0	0	0	0	1	1	0	0	0	0	0	0	1	1	0	0	0	1	0
**Thermovirga**	1	9	0	0	0	0	0	0	0	0	0	0	0	0	0	0	0	0	0	0	0	0
**Thermus**	2	107	0	0	0	0	0	1	0	0	1	0	0	0	0	0	1	0	0	0	1	0
**Thioalkalimicrobium**	1	4	0	0	0	0	0	0	0	0	0	0	0	0	0	0	0	0	0	0	0	0
**Thioalkalivibrio**	2	91	0	0	0	0	0	1	1	1	1	0	0	0	0	1	0	0	0	0	1	0
**Thiobaca**	1	2	0	0	0	0	0	0	0	0	0	0	0	0	0	0	0	0	0	0	0	0
**Thiobacillus**	2	67	0	0	0	0	0	0	0	0	1	0	0	0	0	1	0	0	0	0	1	0
**Thiocapsa**	1	2	0	0	0	0	0	0	0	0	0	0	0	0	0	0	0	0	0	0	0	0
**Thiocystis**	1	11	0	0	0	0	0	0	0	0	0	0	0	0	0	0	0	0	0	0	0	0
**Thioflavicoccus**	1	13	0	0	0	0	0	0	0	0	0	0	0	0	0	0	0	0	0	0	0	0
**Thiomicrospira**	2	50	0	0	0	0	0	0	0	0	1	0	0	0	0	1	0	0	0	0	0	0
**Thiomonas**	2	85	0	0	0	0	0	1	0	0	0	0	0	0	0	1	0	0	0	0	1	0
**Thiorhodococcus**	1	2	0	0	0	0	0	0	0	0	0	0	0	0	0	0	0	0	0	0	0	0
**Thiothrix**	1	2	0	0	0	0	0	0	0	0	0	0	0	0	0	0	0	0	0	0	0	0
**Tistrella**	1	42	0	0	0	0	0	0	0	0	0	0	0	0	0	0	0	0	0	0	0	0
**Tolumonas**	2	100	0	0	1	0	0	0	1	1	1	0	0	0	0	1	1	0	0	0	1	0
**Transposon**	1	2	0	0	1	0	0	0	0	0	0	0	0	0	0	0	0	0	0	0	0	0
**Treponema**	1	63	0	0	0	0	1	0	0	0	0	0	0	0	0	0	1	0	0	0	1	0
**Trichodesmium**	2	71	0	0	0	0	1	0	0	1	0	0	0	0	0	1	0	0	0	0	0	0
**Trichormus**	1	12	0	0	0	0	0	0	0	0	0	0	0	0	0	0	0	0	0	0	0	0
**Tropheryma**	2	22	0	0	0	0	0	0	0	0	0	0	0	0	0	1	0	0	0	0	0	0
**Truepera**	2	104	0	0	0	0	1	1	1	0	1	0	1	0	1	1	1	0	0	0	1	1
**Tsukamurella**	2	123	1	0	0	0	1	1	0	1	1	0	0	0	1	1	1	0	0	0	1	1
**Turneriella**	1	16	0	0	0	0	0	0	0	0	0	0	0	0	0	0	0	0	0	0	0	0
**Uliginosibacterium**	1	2	0	0	0	0	0	0	0	1	0	0	0	0	0	0	0	0	0	0	0	0
**Ureaplasma**	1	24	0	0	0	0	0	0	0	1	0	0	0	0	0	0	1	0	0	0	0	0
**Ureibacillus**	1	2	0	0	0	0	0	0	0	0	0	0	0	0	0	0	0	0	0	0	0	0
**Variovorax**	3	187	0	0	0	0	1	1	1	1	1	1	0	0	1	1	0	1	1	1	1	1
**Veillonella**	1	54	0	0	0	0	0	1	1	0	1	0	0	0	1	0	1	0	0	0	0	0
**Verminephrobacter**	3	188	0	0	0	0	0	0	1	1	1	1	0	0	1	0	0	0	1	0	0	1
**Verrucosispora**	3	126	1	0	0	0	1	1	1	1	1	0	0	0	0	1	1	0	0	0	0	0
**Vibrio**	2	195	0	0	0	0	0	0	0	1	1	0	0	0	0	1	1	0	0	0	1	1
**Vibrionales**	1	46	0	0	0	0	0	0	0	0	0	0	0	0	0	0	0	0	0	0	0	0
**Vitreoscilla**	1	1	0	0	0	0	0	0	0	0	0	0	0	0	0	0	0	0	0	0	0	0
**Vulcanisaeta**	1	14	0	0	0	0	0	0	0	0	0	0	0	0	0	0	0	0	0	0	0	0
**Waddlia**	2	68	0	0	0	0	0	1	0	0	1	0	0	0	0	1	0	0	0	0	1	0
**Wautersia**	1	2	0	0	0	0	0	0	0	0	0	0	0	0	0	0	0	0	0	0	0	0
**Weeksella**	2	65	0	0	0	0	0	0	0	0	0	0	0	0	0	1	1	0	0	0	0	1

Genus	Cluster Number	Protein Counts	3.1.3.41	3.1.8.1	3.2.1.31	3.3.2.10	3.3.2.9	3.5.1.-	3.5.1.4	3.5.1.5	3.5.1.54	3.5.1.6	3.5.2.-	3.5.2.15	3.5.2.2	3.5.4.-	3.5.4.5	3.5.5.1	3.5.5.7	3.5.99.3	3.6.1.7	3.7.1.-
**Weissella**	1	14	0	0	0	0	0	0	0	0	0	0	0	0	0	0	0	0	0	0	0	0
**Wenxinia**	1	2	0	0	0	0	0	0	0	1	0	0	0	0	0	0	0	0	0	0	0	0
**Wigglesworthia**	1	29	0	0	0	0	0	0	0	0	0	0	0	0	0	1	0	0	0	0	0	0
**Wolbachia**	1	32	0	0	0	0	0	0	0	0	0	0	0	0	0	1	0	0	0	0	0	0
**Wolinella**	2	51	0	0	0	0	0	0	0	0	1	0	0	0	0	0	0	0	0	0	0	0
**Xanthobacter**	3	176	0	0	0	0	0	1	0	1	1	1	0	0	0	1	1	1	0	0	1	1
**Xanthobacteraceae**	1	4	0	0	0	0	0	0	1	1	0	0	0	0	0	0	0	0	0	0	0	0
**Xanthomonadaceae**	1	39	0	0	0	0	1	0	0	0	0	0	0	0	0	0	0	0	0	0	0	0
**Xanthomonas**	2	162	0	0	0	0	0	0	0	0	1	0	0	0	0	1	0	0	0	0	1	0
**Xenorhabdus**	2	127	0	0	0	0	0	1	0	0	1	0	0	0	0	1	1	0	1	0	1	1
**Xylanimonas**	2	66	1	0	0	0	1	0	0	0	1	0	0	0	0	1	1	0	0	0	0	0
**Xylella**	2	52	0	0	0	0	0	0	0	0	0	0	0	0	0	1	0	0	0	0	0	0
**Yersinia**	2	145	0	0	0	0	0	0	1	1	1	0	0	0	0	1	1	1	0	0	1	1
**Yokenella**	2	61	0	0	0	0	0	0	0	0	0	0	0	0	0	0	0	0	0	0	0	0
**Zobellia**	1	28	0	0	0	0	0	0	0	0	0	0	0	0	0	0	0	0	0	0	0	0
**Zunongwangia**	2	98	0	0	0	0	0	0	0	0	1	0	0	0	0	1	1	0	0	0	0	1
**Zymomonas**	2	67	0	0	0	0	0	0	0	0	0	0	0	0	0	0	1	1	0	0	0	0
**Zymophilus**	1	2	0	0	0	0	0	0	0	0	0	0	0	0	0	0	0	0	0	0	0	0

Genus	Cluster Number	Protein Counts	3.7.1.2	3.7.1.9	3.8.1.-	3.8.1.2	3.8.1.3	3.8.1.5	4.1.1.-	4.1.1.44	4.1.1.55	4.1.1.7	4.1.1.70	4.1.1.77	4.1.2.-	4.1.3.-	4.1.3.39	4.1.99.-	4.1.99.11	4.2.1.-	4.2.1.17
Acaricomes	1	2	0	0	0	0	0	0	0	0	0	0	0	0	0	0	0	0	0	0	0
Acaryochloris	2	88	0	0	0	1	0	1	1	0	0	0	0	0	0	1	0	0	0	1	1
Acetivibrio	1	2	0	0	0	0	0	0	0	0	0	0	0	0	0	0	0	0	0	0	0
Acetobacter	2	85	0	0	0	0	0	0	1	0	0	1	0	0	1	1	0	0	0	1	0
Acetobacteraceae	1	2	0	0	0	0	0	0	0	0	0	0	0	0	0	0	0	0	0	0	0
Acetobacterium	1	8	0	0	0	0	0	0	0	0	0	0	0	0	0	0	0	0	0	0	0
Acetohalobium	2	79	0	0	0	0	0	0	1	0	0	0	0	0	1	1	0	1	0	1	0
Acholeplasma	2	38	0	0	0	1	0	0	0	0	0	0	0	0	0	0	0	0	0	1	0
Achromobacter	2	192	1	0	0	0	0	1	1	1	0	0	0	1	1	1	1	0	0	1	1
Acidaminococcus	1	65	0	0	0	0	0	1	1	1	0	0	1	0	0	1	0	0	0	1	1
Acidianus	1	9	0	0	0	0	0	0	0	0	0	0	0	0	0	0	0	0	0	0	0
Acidilobus	1	8	0	0	0	0	0	0	0	0	0	0	0	0	0	0	0	0	0	0	0
Acidimicrobium	2	58	1	0	0	0	0	0	0	1	0	0	0	0	0	1	0	0	0	1	1
Acidiphilium	3	144	1	0	1	1	0	1	0	1	0	0	0	0	1	1	0	0	0	1	1
Acidithiobacillus	2	70	0	0	0	0	0	0	1	0	0	1	0	0	0	1	0	0	0	1	0
Acidobacterium	2	91	1	0	0	0	0	0	1	0	0	1	0	0	0	1	0	0	0	1	1
Acidocella	1	2	0	0	0	0	0	0	0	0	0	0	0	0	0	0	0	0	0	0	0
Acidothermus	2	68	0	0	0	1	0	0	1	1	0	0	0	0	0	1	0	0	0	0	1
Acidovorax	3	217	1	1	0	1	0	1	1	1	0	0	0	1	1	1	1	0	0	1	1
Aciduliprofundum	1	11	0	0	0	0	0	0	0	0	0	0	0	0	0	0	0	0	0	0	0
Acinetobacter	3	188	1	0	0	0	0	0	1	1	0	0	0	0	0	1	0	0	0	1	1
Actinobacillus	2	112	0	0	0	1	0	0	1	1	0	0	0	0	1	1	0	0	0	1	1
Actinomadura	1	1	0	0	0	0	0	0	0	0	0	0	0	0	0	0	0	0	0	0	0
Actinoplanes	1	52	0	0	0	0	0	0	0	0	0	0	0	0	0	0	0	0	0	0	0
Actinopolyspora	1	2	0	0	0	0	0	0	0	0	0	0	0	0	0	0	0	0	0	0	0
Actinosynnema	2	122	1	0	0	1	0	0	0	1	0	0	0	0	1	1	0	0	0	0	1
Advenella	1	30	0	0	0	0	0	0	0	0	0	0	0	0	0	0	0	0	0	0	0
Aequorivita	1	12	0	0	0	0	0	0	0	0	0	0	0	0	0	0	0	0	0	0	0
Aerococcus	2	57	0	0	0	1	0	0	0	1	0	0	0	0	0	0	0	0	0	1	1
Aeromonas	2	127	1	0	0	1	0	0	1	1	0	0	0	0	0	1	0	0	0	1	1
Aeropyrum	1	13	0	0	0	0	0	0	0	0	0	0	0	0	0	0	0	0	0	0	0
Afipia	1	54	1	0	0	1	0	0	0	1	0	0	0	0	0	1	0	0	0	1	0
Aggregatibacter	1	81	0	0	0	1	0	0	1	1	0	0	0	0	0	1	0	0	0	1	0
Agrobacterium	2	242	1	1	0	1	0	1	1	1	1	1	0	1	1	1	0	0	0	1	1
Ahrensia	1	2	0	0	0	0	0	0	0	0	0	0	0	0	0	0	0	0	0	0	0
Akkermansia	2	47	0	0	0	0	0	0	1	1	0	0	0	0	0	1	0	0	0	0	0
Albidiferax	3	144	1	0	0	1	0	1	1	1	0	0	0	0	1	1	1	0	0	1	1
Alcaligenes	1	21	0	0	0	0	0	0	0	0	0	0	0	1	0	0	0	0	0	0	0
Alcanivorax	2	146	0	0	0	1	0	1	1	0	0	0	0	0	0	1	0	0	0	1	1
Alicycliphilus	3	152	0	1	0	0	0	1	1	0	0	0	0	1	1	1	1	0	0	1	1
Alicyclobacillus	2	91	0	0	0	1	0	0	0	0	0	0	0	1	0	1	1	0	0	1	1
Aliivibrio	2	118	0	0	0	0	0	0	1	0	0	0	0	0	0	1	0	0	0	1	1
Alishewanella	1	1	0	0	0	0	0	0	0	0	0	0	0	0	0	0	0	0	0	0	0
Alistipes	1	52	0	0	0	0	0	0	1	1	0	0	0	0	0	1	0	0	0	1	0
Alkalilimnicola	2	97	0	1	0	1	0	1	1	1	0	0	0	1	0	1	1	0	0	1	1
Alkaliphilus	2	62	0	0	0	0	0	0	0	1	0	0	0	0	0	0	0	1	0	1	0
Allochromatium	2	84	0	0	0	0	0	1	1	0	0	0	0	0	0	1	0	0	0	1	0
Alphaproteobacteria	1	3	0	0	0	0	0	0	0	0	0	0	0	0	0	0	0	0	0	0	1
Alteromonadales	1	12	0	0	0	0	0	0	0	0	0	0	0	0	0	0	0	0	0	1	0
Alteromonas	1	37	0	0	0	0	0	0	0	0	0	0	0	0	0	0	0	0	0	0	0
Aminobacter	1	4	0	0	0	0	0	0	0	0	0	0	0	0	0	0	0	0	0	0	0
Aminobacterium	2	59	0	0	0	0	0	0	0	0	0	0	0	0	1	1	0	0	0	1	0

Genus	Cluster Number	Protein Counts	3.7.1.2	3.7.1.9	3.8.1.-	3.8.1.2	3.8.1.3	3.8.1.5	4.1.1.-	4.1.1.44	4.1.1.55	4.1.1.7	4.1.1.70	4.1.1.77	4.1.2.-	4.1.3.-	4.1.3.39	4.1.99.-	4.1.99.11	4.2.1.-	4.2.1.17
**Ammonifex**	2	50	0	0	0	0	0	0	1	0	0	0	0	0	0	1	0	0	0	1	0
**Amphibacillus**	1	13	0	0	0	0	0	0	0	0	0	0	0	0	0	0	0	0	0	0	0
**Amphritea**	1	2	0	0	0	0	0	0	0	0	0	0	0	0	0	0	0	0	0	0	0
**Amycolatopsis**	2	201	1	1	0	1	0	1	0	1	1	1	0	1	0	1	0	0	0	1	1
**Amycolicicoccus**	1	43	0	0	0	0	0	0	0	0	0	0	0	0	0	0	0	0	0	0	0
**Anabaena**	2	92	0	0	0	1	0	1	1	0	0	0	0	0	0	1	0	0	0	1	1
**Anaerobaculum**	1	11	0	0	0	0	0	0	0	0	0	0	0	0	0	0	0	0	0	0	0
**Anaerococcus**	2	46	0	0	0	0	0	0	0	0	0	0	0	0	0	0	0	0	0	1	0
**Anaerolinea**	2	66	0	0	0	0	0	0	0	0	0	0	0	0	0	1	0	0	0	1	0
**Anaeromyxobacter**	3	117	1	1	0	1	1	1	1	0	0	0	0	0	0	1	0	0	0	1	1
**Anaerostipes**	1	44	0	0	0	1	0	0	1	1	0	0	0	0	0	1	0	0	0	0	0
**Anaerotruncus**	1	2	0	0	0	0	0	0	0	0	0	0	0	0	0	0	0	0	0	0	0
**Anaplasma**	1	35	0	0	0	0	0	0	1	0	0	0	0	0	0	0	0	0	0	1	1
**Ancylobacter**	1	3	0	0	0	0	0	0	0	0	0	0	0	0	0	0	0	0	0	0	0
**Aneurinibacillus**	1	1	0	0	0	0	0	0	0	0	0	0	0	0	0	0	0	0	0	0	0
**Angomonas**	1	1	0	0	0	0	0	0	0	0	0	0	0	0	0	0	0	0	0	0	0
**Anoxybacillus**	2	72	0	0	0	1	0	0	0	1	0	0	0	0	0	1	0	1	0	1	1
**Aquifex**	2	45	0	0	0	0	0	0	0	0	0	0	0	0	0	1	0	0	0	1	0
**Aquimarina**	1	2	0	0	0	0	0	0	0	0	0	0	0	0	0	0	0	0	0	0	0
**Arcanobacterium**	2	45	0	0	0	0	0	0	0	0	0	0	0	0	0	0	0	0	0	0	0
**Archaeoglobus**	1	14	0	0	0	0	0	0	0	0	0	0	0	0	0	0	0	0	0	0	0
**Arcobacter**	2	101	0	0	0	0	0	0	1	1	0	0	0	0	0	1	1	1	0	1	1
**Aromatoleum**	3	133	1	0	0	1	0	0	1	0	0	0	0	0	0	1	0	0	1	1	1
**Arsenophonus**	1	4	0	0	0	0	0	0	0	0	0	0	0	0	0	0	0	0	0	0	0
**Arthrobacter**	2	206	1	0	0	1	1	1	0	1	0	1	0	0	1	1	1	1	0	1	1
**Arthrospira**	2	67	0	0	0	0	0	1	1	0	0	0	0	0	1	1	0	0	0	0	0
**Aster**	1	10	0	0	0	0	0	0	0	0	0	0	0	0	0	0	0	0	0	0	0
**Asticcacaulis**	2	103	1	0	0	0	0	0	1	1	0	0	0	0	0	1	0	0	0	1	1
**Atopobium**	1	28	0	0	0	0	0	0	0	0	0	0	0	0	0	0	0	0	0	0	0
**Aurantimonas**	1	2	0	0	0	0	0	0	0	0	0	0	0	0	0	0	0	0	0	0	0
**Avibacterium**	1	17	0	0	0	0	0	0	0	0	0	0	0	0	0	0	0	0	0	1	0
**Azoarcus**	3	172	1	0	0	0	0	0	1	1	0	0	0	1	0	1	1	0	0	1	1
**Azorhizobium**	3	149	0	0	0	0	0	1	0	1	0	0	0	0	1	1	0	0	0	1	1
**Azorhizophilus**	1	4	0	0	0	0	0	0	0	0	0	0	0	0	0	0	0	0	0	0	0
**Azospira**	1	19	0	0	0	0	0	0	0	0	0	0	0	0	0	0	0	0	0	0	0
**Azospirillum**	2	198	1	0	0	1	0	0	1	1	0	0	0	0	1	1	0	0	0	1	1
**Azotobacter**	3	183	1	1	0	1	1	1	1	1	0	0	0	1	1	1	1	0	0	1	1
**Bacillales**	1	9	0	0	0	0	0	0	0	0	0	0	0	0	0	0	0	0	0	0	0
**Bacilli**	1	3	0	0	0	0	0	0	0	0	0	0	0	0	0	0	0	0	0	0	0
**Bacillus**	2	237	1	0	0	1	0	1	1	1	0	0	0	1	0	1	1	1	0	1	1
**Bacteria**	2	86	0	0	0	0	0	0	0	0	0	0	0	0	1	1	0	0	0	1	1
**Bacteriovorax**	2	98	1	0	0	0	0	1	0	0	0	1	0	0	1	1	0	1	0	1	1
**Bacteroidales**	2	59	0	0	0	1	0	0	1	1	0	0	0	0	0	1	0	0	0	1	0
**Bacteroides**	2	96	0	0	0	1	0	0	1	1	0	0	0	0	0	1	1	0	0	1	0
**Bacteroidetes**	1	2	0	0	0	0	0	0	0	0	0	0	0	0	0	0	0	0	0	0	0
**Bartonella**	2	50	0	0	0	0	0	0	1	0	0	0	0	0	0	1	0	0	0	1	0
**Baumannia**	2	24	0	0	0	1	0	0	0	0	0	0	0	0	0	1	0	0	0	1	0
**Bdellovibrio**	2	87	1	0	0	0	0	1	0	0	0	0	0	0	0	1	0	0	0	1	1
**Beijerinckia**	3	123	0	0	0	1	0	1	1	1	0	0	0	0	1	1	0	0	0	1	1
**Belliella**	1	14	0	0	0	0	0	0	0	0	0	0	0	0	0	0	0	0	0	0	0
**Beutenbergia**	2	82	0	0	0	1	0	1	0	1	0	0	0	0	0	1	0	0	0	0	0
**Bifidobacterium**	2	61	0	0	0	0	0	1	0	1	0	0	0	0	1	1	0	0	0	0	0

Genus	Cluster Number	Protein Counts	3.7.1.2	3.7.1.9	3.8.1.-	3.8.1.2	3.8.1.3	3.8.1.5	4.1.1.-	4.1.1.44	4.1.1.55	4.1.1.7	4.1.1.70	4.1.1.77	4.1.2.-	4.1.3.-	4.1.3.39	4.1.99.-	4.1.99.11	4.2.1.-	4.2.1.17
**Bizionia**	1	1	0	0	0	0	0	0	0	0	0	0	0	0	0	0	0	0	0	0	0
**Blastococcus**	1	39	0	0	0	0	0	0	0	0	0	0	0	0	0	0	0	0	0	0	0
**Blattabacterium**	1	23	0	0	0	0	0	0	0	0	0	0	0	0	0	1	0	0	0	0	0
**Blautia**	1	22	0	0	0	0	0	0	0	1	0	0	0	0	0	0	0	0	0	0	0
**Bordetella**	3	227	1	1	0	1	0	1	1	1	1	0	0	0	1	1	1	0	0	1	1
**Borrelia**	1	32	0	0	0	1	0	0	0	0	0	0	0	0	0	0	0	0	0	0	1
**Brachybacterium**	1	18	0	0	0	0	0	0	0	0	0	0	0	0	0	0	0	0	0	0	0
**Brachymonas**	1	4	0	0	0	0	0	0	0	0	0	0	0	0	0	0	0	0	0	0	0
**Brachyspira**	2	79	0	0	0	0	0	0	0	1	0	0	0	0	0	1	0	0	0	1	0
**Bradyrhizobiaceae**	1	20	0	0	0	0	0	0	0	0	0	0	0	0	0	1	0	0	0	0	0
**Bradyrhizobium**	3	254	1	0	0	1	0	1	1	1	0	1	0	0	1	1	0	0	0	1	1
**Brenneria**	1	20	0	0	0	0	0	0	1	0	0	0	0	0	0	1	0	0	0	1	0
**Brevibacillus**	2	122	1	0	0	1	0	0	1	1	0	0	0	1	0	1	0	1	0	1	1
**Brevibacterium**	1	4	0	0	0	0	0	0	0	0	0	0	0	0	0	0	0	0	0	0	0
**Brevundimonas**	2	105	1	0	0	0	0	0	1	0	0	1	0	0	1	1	0	0	0	1	1
**Brucella**	3	138	0	0	0	1	0	1	1	1	0	0	0	0	1	1	0	0	0	1	1
**Buchnera**	1	26	0	0	0	0	0	0	0	0	0	0	0	0	0	1	0	0	0	1	0
**Burkholderia**	3	424	1	1	0	1	1	1	1	1	1	1	0	1	1	1	1	0	0	1	1
**Burkholderiaceae**	3	143	1	1	0	1	0	1	1	0	0	0	0	1	0	1	0	0	0	1	1
**Burkholderiales**	1	24	0	0	0	0	0	0	0	0	0	0	0	1	0	0	0	0	0	0	1
**Buttiauxella**	1	2	0	0	0	0	0	0	0	0	0	0	0	0	0	0	0	0	0	0	0
**Butyrivibrio**	2	67	0	0	0	1	0	0	1	0	0	0	0	0	0	1	0	0	0	1	0
**Caldanaerobacter**	2	63	0	0	0	0	0	0	0	1	0	0	0	0	0	1	0	1	0	1	0
**Caldicellulosiruptor**	2	75	0	0	1	0	0	0	0	1	0	0	0	0	0	1	0	1	0	1	0
**Caldilinea**	1	23	0	0	0	0	0	0	0	0	0	0	0	0	0	0	0	0	0	0	0
**Caldimonas**	1	6	0	0	0	0	0	0	0	0	0	0	0	0	0	0	0	0	0	0	0
**Caldisericum**	1	10	0	0	0	0	0	0	0	0	0	0	0	0	0	0	0	0	0	0	0
**Caldisphaera**	1	7	0	0	0	0	0	0	0	0	0	0	0	0	0	0	0	0	0	0	0
**Calditerrivibrio**	2	74	0	0	0	0	0	0	1	0	0	0	0	0	0	1	0	0	0	1	1
**Caldivirga**	1	13	0	0	0	0	0	0	0	0	0	0	0	0	0	0	0	0	0	0	0
**Calothrix**	1	43	0	0	0	0	0	0	1	0	0	0	0	0	0	1	0	0	0	0	0
**Calyptogena**	2	34	0	0	0	0	0	0	0	0	0	0	0	0	0	1	0	0	0	1	0
**Campylobacter**	2	80	0	0	0	0	0	0	1	1	0	0	0	0	0	1	1	1	0	1	0
**Candidatus**	2	290	1	0	0	1	1	1	1	1	0	0	0	0	1	1	0	1	0	1	1
**Capnocytophaga**	1	50	0	0	0	0	0	0	0	0	0	0	0	0	0	0	0	0	0	1	0
**Carbophilus**	1	2	0	0	0	0	0	0	0	0	0	0	0	0	0	0	0	0	0	0	0
**Carboxydibrachium**	1	14	0	0	0	0	0	0	0	0	0	0	0	0	0	0	0	0	0	0	0
**Carboxydothermus**	2	68	0	0	0	0	0	1	1	1	0	0	0	0	0	1	1	0	0	1	1
**Carica**	1	2	0	0	0	0	0	0	0	0	0	0	0	0	0	0	0	0	0	0	0
**Carnobacterium**	2	71	0	0	0	0	0	0	0	0	0	0	0	0	0	0	0	0	0	1	1
**Catenibacterium**	1	2	0	0	0	0	0	0	0	1	0	0	0	0	0	0	0	0	0	0	0
**Catenovulum**	1	2	0	0	0	0	0	0	0	0	0	0	0	0	0	0	0	0	0	0	0
**Catenulispora**	3	159	1	0	0	1	0	1	1	1	1	0	0	0	1	1	0	0	0	1	1
**Caulobacter**	3	168	1	0	0	0	0	0	1	1	0	0	0	0	1	1	0	0	0	1	1
**Cedecea**	1	45	0	0	0	0	0	0	1	0	0	0	0	0	0	1	0	0	0	1	0
**Cellulomonas**	2	96	1	0	0	1	0	0	0	0	0	0	0	0	1	1	0	0	0	1	1
**Cellulophaga**	2	105	1	0	0	1	0	0	0	1	0	0	0	0	0	1	0	0	0	1	1
**Cellulosilyticum**	1	14	0	0	0	0	0	0	0	0	0	0	0	0	0	0	0	0	0	0	0
**Cellvibrio**	2	85	0	0	0	0	0	0	1	0	0	0	0	0	0	1	0	0	0	1	1
**Cenarchaeum**	1	7	0	0	0	0	0	0	0	0	0	0	0	0	0	0	0	0	0	0	0
**Chamaesiphon**	1	13	0	0	0	0	0	0	0	0	0	0	0	0	0	0	0	0	0	0	0
**Chelativorans**	3	136	1	0	0	1	0	0	1	1	1	0	0	0	1	1	0	0	0	1	1

Genus	Cluster Number	Protein Counts	3.7.1.2	3.7.1.9	3.8.1.-	3.8.1.2	3.8.1.3	3.8.1.5	4.1.1.-	4.1.1.44	4.1.1.55	4.1.1.7	4.1.1.70	4.1.1.77	4.1.2.-	4.1.3.-	4.1.3.39	4.1.99.-	4.1.99.11	4.2.1.-	4.2.1.17
Chelatococcus	1	2	0	0	0	0	0	0	0	0	0	0	0	0	0	0	0	0	0	0	0
Chitinophaga	2	98	1	0	0	1	0	0	1	0	0	1	0	0	0	1	0	0	0	1	1
Chlamydia	1	36	0	0	0	0	0	0	1	0	0	0	0	0	0	0	0	0	0	1	0
Chlamydomonas	1	3	0	0	0	0	0	0	0	0	0	0	0	0	0	0	0	0	0	0	0
Chlamydophila	1	31	0	0	0	0	0	0	1	0	0	0	0	0	0	0	0	0	0	1	0
Chlorobaculum	2	61	0	0	0	0	0	1	0	0	0	0	0	0	0	1	0	0	0	1	0
Chlorobium	2	89	0	0	0	1	0	1	0	0	0	0	0	0	1	1	0	0	0	1	1
Chlorobium/Pelodictyon	1	2	0	0	0	0	0	0	0	0	0	0	0	0	0	0	0	0	0	1	0
Chloroflexus	2	105	1	1	0	1	0	1	0	0	0	0	0	1	1	1	1	0	0	1	1
Chlorogloeopsis	1	9	0	0	0	0	0	0	1	0	0	0	0	0	0	0	0	0	0	0	0
Chloroherpeton	2	59	0	0	0	1	0	1	0	0	0	0	0	0	0	1	0	0	0	1	0
Chromobacterium	2	117	1	0	0	1	0	1	1	1	0	1	0	0	0	1	0	0	0	1	1
Chromohalobacter	2	145	0	0	0	1	0	0	1	1	0	1	0	0	1	1	0	0	0	1	1
Chroococcidiopsis	1	24	0	0	0	0	0	0	1	0	0	0	0	0	0	0	0	0	0	0	0
Chryseobacterium	2	104	1	0	0	0	1	1	0	1	0	1	0	0	1	1	0	0	0	0	1
Citreicella	1	13	0	0	0	0	0	0	0	0	0	0	0	0	0	0	0	0	0	1	0
Citrobacter	2	190	1	0	0	1	0	0	1	1	0	0	0	0	1	1	1	0	0	1	1
Clavibacter	2	83	0	1	0	1	0	0	1	1	0	0	0	0	0	1	0	0	0	0	1
Clonorchis	1	1	0	0	0	0	0	0	0	0	0	0	0	0	0	0	0	0	0	0	0
Clostridiales	2	77	0	0	0	1	0	0	1	1	0	0	0	0	0	1	0	0	0	0	0
Clostridium	2	191	0	0	0	1	0	1	1	1	0	0	0	0	0	1	0	1	0	1	1
Cohnella	1	2	0	0	0	0	0	0	0	0	0	0	0	0	0	0	0	0	0	0	0
Coleofasciculus	1	4	0	0	0	0	0	0	1	0	0	0	0	0	0	0	0	0	0	0	0
Collimonas	1	35	0	0	0	0	0	0	1	0	0	0	0	0	0	0	0	0	0	0	0
Colwellia	2	130	0	0	0	1	0	0	1	1	0	0	0	0	0	1	0	0	0	1	1
Comamonas	2	196	1	1	0	0	0	1	0	1	1	1	0	1	1	1	1	0	0	1	1
Conexibacter	3	117	1	1	0	0	0	0	0	0	0	0	0	1	0	1	0	0	0	1	1
Coprobacillus	1	2	0	0	0	0	0	0	0	0	0	0	0	0	0	0	0	0	0	0	0
Coprococcus	2	76	0	0	0	0	0	0	1	1	0	0	0	0	0	1	0	0	0	1	1
Coprothermobacter	2	45	0	0	0	0	0	0	0	1	0	0	0	0	0	0	0	0	0	1	0
Coraliomargarita	2	68	0	0	0	0	0	0	1	0	0	0	0	1	0	1	0	0	0	1	1
Corallococcus	1	29	0	0	0	0	0	0	0	0	0	0	0	0	0	0	0	0	0	0	1
Coriobacterium	2	36	0	0	0	0	0	0	1	0	0	0	0	0	0	0	0	0	0	0	0
Corynebacterineae	1	5	0	0	0	0	0	0	0	0	0	0	0	0	0	0	0	0	0	0	1
Corynebacterium	2	175	0	0	0	1	0	0	1	1	0	0	0	0	1	1	0	0	0	1	1
Coxiella	2	54	0	0	0	0	0	0	0	0	0	0	0	0	0	0	0	0	0	1	1
Crinalium	1	21	0	0	0	0	0	0	1	0	0	0	0	0	0	0	0	0	0	0	0
Croceibacter	2	80	1	0	0	1	0	1	0	1	0	0	0	0	0	1	0	0	0	1	1
Crocosphaera	1	6	0	0	0	0	0	0	0	0	0	0	0	0	0	0	0	0	0	0	0
Cronobacter	2	120	0	0	0	1	0	0	1	1	0	0	0	0	0	1	0	0	0	1	1
Cryptobacterium	2	39	0	0	0	0	0	0	0	0	0	0	0	0	0	0	0	0	0	1	0
Cucumis	1	9	0	0	0	0	0	0	0	0	0	0	0	0	0	0	0	0	0	0	0
Cupriavidus	3	317	1	1	0	1	1	1	1	1	0	1	0	1	1	1	1	0	0	1	1
Curtobacterium	1	5	0	0	0	0	0	0	0	0	0	0	0	0	0	0	0	0	0	0	0
Curvibacter	1	27	0	0	0	0	0	0	0	0	0	0	0	0	0	1	0	0	0	0	0
Cyanobacterium	1	9	0	0	0	0	0	0	0	0	0	0	0	0	0	0	0	0	0	0	0
Cyanobium	1	10	0	0	0	0	0	0	0	0	0	0	0	0	0	0	0	0	0	0	0
Cyanothece	1	34	0	0	0	0	0	0	0	0	0	0	0	0	0	0	0	0	0	0	0
Cyclobacterium	1	19	0	0	0	0	0	0	0	0	0	0	0	0	0	0	0	0	0	0	0
Cycloclasticus	1	21	0	0	0	0	0	0	0	0	0	0	0	0	0	0	0	0	0	0	0
Cylindrospermopsis	1	4	0	0	0	0	0	0	1	0	0	0	0	0	0	0	0	0	0	0	0
Cylindrospermum	1	30	0	0	0	0	0	0	1	0	0	0	0	0	0	1	0	0	0	0	0

Genus	Cluster Number	Protein Counts	3.7.1.2	3.7.1.9	3.8.1.-	3.8.1.2	3.8.1.3	3.8.1.5	4.1.1.-	4.1.1.44	4.1.1.55	4.1.1.7	4.1.1.70	4.1.1.77	4.1.2.-	4.1.3.-	4.1.3.39	4.1.99.-	4.1.99.11	4.2.1.-	4.2.1.17
Cytophaga	2	69	0	0	0	1	0	1	0	0	0	0	0	0	0	1	0	0	0	1	1
Dactylococcopsis	1	8	0	0	0	0	0	0	0	0	0	0	0	0	0	0	0	0	0	0	0
Dechloromonas	1	40	0	0	0	0	0	0	0	0	0	0	0	0	0	0	0	0	0	0	0
Dechlorosoma	1	19	0	0	0	0	0	0	0	0	0	0	0	0	0	0	0	0	0	0	0
Deferribacter	2	73	0	0	0	1	0	0	1	0	0	0	0	0	0	1	0	0	0	1	1
Dehalobacter	1	9	0	0	0	0	0	0	0	0	0	0	0	0	0	0	0	0	0	0	0
Dehalococcoides	2	41	0	0	0	1	0	1	0	0	0	0	0	0	0	1	0	0	0	0	0
Dehalogenimonas	1	37	0	0	0	0	0	0	0	0	0	0	0	0	0	1	0	0	0	1	0
Deinococcus	2	140	1	0	0	1	1	0	1	1	0	0	0	0	1	1	0	1	0	1	1
Delftia	3	177	1	0	0	0	1	1	0	1	0	1	0	1	1	1	1	0	0	1	1
Denitrovibrio	2	67	0	0	0	0	0	0	1	0	0	0	0	0	0	1	0	0	0	1	1
Desulfarculus	2	92	0	0	0	0	0	0	1	1	0	0	0	0	0	1	0	0	0	1	1
Desulfatibacillum	3	84	1	0	0	0	0	0	0	1	0	0	0	0	0	1	0	1	0	1	1
Desulfitobacterium	2	102	0	1	0	1	0	0	0	1	0	0	0	0	1	1	1	1	0	1	1
Desulfobacca	2	65	0	0	0	0	0	0	1	0	0	0	0	0	0	1	0	0	0	1	1
Desulfobacterium	2	113	0	0	0	1	0	1	1	1	0	0	0	0	0	1	0	1	0	1	1
Desulfobacula	1	21	0	0	0	0	0	0	0	0	0	0	0	0	0	0	0	0	0	0	0
Desulfobulbus	2	88	0	0	0	0	0	0	1	0	0	0	0	0	0	1	0	0	0	1	1
Desulfocapsa	1	9	0	0	0	0	0	0	0	0	0	0	0	0	0	0	0	0	0	0	0
Desulfococcus	2	80	0	0	0	0	0	0	1	0	0	0	0	0	0	1	0	1	0	1	1
Desulfohalobium	2	52	0	0	0	0	0	0	0	0	0	0	0	0	0	1	0	1	0	1	0
Desulfomicrobium	2	65	0	0	0	0	0	0	0	0	0	0	0	0	0	1	0	1	0	1	0
Desulfomonile	1	25	0	0	0	0	0	0	0	0	0	0	0	0	0	0	0	0	0	0	0
Desulforhabdus	1	2	0	0	0	0	0	0	0	0	0	0	0	0	0	0	0	0	0	0	0
Desulfosporosinus	1	30	0	0	0	0	0	0	0	0	0	0	0	0	0	0	0	0	0	0	0
Desulfotalea	2	66	0	0	0	0	0	0	1	0	0	0	0	0	0	1	0	1	0	1	0
Desulfotomaculum	2	98	0	0	0	0	0	0	1	0	0	0	0	0	0	1	0	1	0	1	1
Desulfovibrio	2	144	0	0	0	1	0	1	1	1	0	1	0	0	1	1	0	1	0	1	1
Desulfurispirillum	2	78	0	0	0	0	0	0	1	0	0	0	0	0	0	1	0	0	0	1	1
Desulfurivibrio	2	71	0	0	0	0	0	0	1	0	0	0	0	0	0	1	0	0	0	1	0
Desulfurobacterium	2	53	0	0	0	0	0	0	1	0	0	0	0	0	0	1	0	0	0	1	0
Desulfurococcus	1	11	0	0	0	0	0	0	0	0	0	0	0	0	0	0	0	0	0	0	0
Diaphorobacter	1	16	0	0	0	0	0	0	0	0	0	0	0	1	0	0	1	0	0	0	0
Dichelobacter	2	45	0	0	0	0	0	0	0	0	0	0	0	0	0	0	0	0	0	1	1
Dickeya	2	155	0	0	0	1	0	0	1	1	1	0	0	0	1	1	0	0	0	1	1
Dictyoglomus	2	62	0	0	0	1	0	0	0	0	0	0	0	0	0	1	0	1	0	1	0
Dinoroseobacter	3	148	1	0	0	1	0	1	1	1	1	0	0	1	1	1	0	0	0	1	1
Dokdonia	1	3	0	0	0	0	0	0	0	1	0	0	0	0	0	0	0	0	0	0	0
Dorea	1	7	0	0	0	0	0	0	0	1	0	0	0	0	0	0	0	0	0	0	0
Dyadobacter	2	83	1	0	0	0	0	0	1	0	0	0	0	0	1	1	0	0	0	1	1
Echinicola	1	20	0	0	0	0	0	0	0	0	0	0	0	0	0	0	0	0	0	0	0
Edwardsiella	2	126	0	0	0	1	0	0	1	0	0	0	0	0	0	1	0	0	0	1	0
Eggerthella	1	51	0	0	0	0	0	0	0	1	0	0	0	0	0	1	0	0	0	1	1
Ehrlichia	2	37	0	0	0	0	0	0	0	0	0	0	0	0	0	0	0	0	0	1	1
Eikenella	1	4	0	0	0	0	0	0	1	0	0	0	0	0	0	0	0	0	0	0	0
Elusimicrobium	2	44	0	0	0	0	0	0	0	0	0	0	0	0	0	1	0	1	0	1	0
Emticicia	1	21	0	0	0	0	0	0	0	0	0	0	0	0	0	0	0	0	0	0	0
Enhydrobacter	1	1	0	0	0	0	0	0	0	0	0	0	0	0	0	0	0	0	0	0	0
Ensifer	1	2	0	0	0	0	0	0	0	0	0	0	0	0	0	0	0	0	0	0	0
Enterobacter	2	202	1	0	0	1	0	0	1	1	0	0	0	0	1	1	0	0	0	1	1
Enterobacteriaceae	2	211	1	0	0	1	0	0	1	1	0	0	0	0	1	1	1	0	0	1	1
Enterococcus	1	187	0	0	0	1	0	0	1	1	0	0	0	0	0	1	0	0	0	1	1

Genus	Cluster Number	Protein Counts	3.7.1.2	3.7.1.9	3.8.1.-	3.8.1.2	3.8.1.3	3.8.1.5	4.1.1.-	4.1.1.44	4.1.1.55	4.1.1.7	4.1.1.70	4.1.1.77	4.1.2.-	4.1.3.-	4.1.3.39	4.1.99.-	4.1.99.11	4.2.1.-	4.2.1.17
Enterovibrio	1	15	0	0	0	0	0	0	0	0	0	0	0	0	0	1	0	0	0	1	0
Erwinia	2	151	0	0	0	1	0	1	1	1	1	0	0	0	0	1	0	0	0	1	1
Erysipelothrix	1	9	0	0	0	0	0	0	0	0	0	0	0	0	0	0	0	0	0	0	0
Erysipelotrichaceae	1	4	0	0	0	0	0	0	0	0	0	0	0	0	0	0	0	0	0	0	0
Erythrobacter	2	80	0	0	0	0	0	1	0	0	0	0	0	0	0	1	0	0	0	1	1
Escherichia	2	242	1	0	0	1	0	0	1	1	0	0	0	0	1	1	1	0	0	1	1
Ethanoligenens	2	69	0	0	0	1	0	0	1	1	0	0	0	0	0	1	0	1	0	1	0
Eubacteriaceae	1	2	0	0	0	0	0	0	0	0	0	0	0	0	0	0	0	0	0	0	0
Eubacterium	2	103	0	0	0	0	0	1	1	1	0	0	0	0	0	1	0	1	0	1	0
Eudoraea	1	4	0	0	0	0	0	0	0	0	0	0	0	0	0	0	0	0	0	0	0
Euryarchaeota	1	1	0	0	0	0	0	0	0	0	0	0	0	0	0	0	0	0	0	0	0
Exiguobacterium	2	93	0	0	0	1	0	1	0	1	0	0	0	0	0	1	0	0	0	1	1
Faecalibacterium	1	12	0	0	0	0	0	0	0	0	0	0	0	0	0	0	0	0	0	0	0
Ferrimonas	2	115	1	0	0	1	0	1	1	0	0	0	0	0	0	1	0	0	0	1	1
Ferroglobus	1	14	0	0	0	0	0	0	0	0	0	0	0	0	0	0	0	0	0	0	0
Ferroplasma	1	11	0	0	0	0	0	0	0	0	0	0	0	0	0	0	0	0	0	0	0
Fervidicoccus	1	7	0	0	0	0	0	0	0	0	0	0	0	0	0	0	0	0	0	0	0
Fervidobacterium	2	54	0	0	0	0	0	1	0	0	0	0	0	0	0	0	0	0	0	1	0
Fibrella	1	17	0	0	0	0	0	0	0	0	0	0	0	0	0	0	0	0	0	0	0
Fibrobacter	2	52	0	0	0	0	0	0	1	1	0	0	0	0	0	1	0	0	0	1	0
Filifactor	1	9	0	0	0	0	0	0	0	0	0	0	0	0	0	0	0	0	0	0	0
Finegoldia	2	55	0	0	0	0	0	0	0	1	0	0	0	0	0	0	0	0	0	1	0
Firmicutes	1	15	0	0	0	0	0	0	0	1	0	0	0	0	0	0	0	0	0	0	0
Fischerella	1	15	0	0	0	0	0	0	1	0	0	0	0	0	0	0	0	0	0	0	0
Flavobacteria	1	7	0	0	0	0	0	0	0	1	0	0	0	0	0	0	0	0	0	0	0
Flavobacteriaceae	2	69	1	0	0	1	0	0	0	0	0	0	0	0	0	1	0	0	0	1	1
Flavobacteriales	1	3	0	0	0	0	0	0	0	1	0	0	0	0	0	0	0	0	0	0	0
Flavobacterium	2	103	1	0	0	1	0	1	1	0	0	0	0	0	0	1	0	0	0	1	1
Flexibacter	1	17	0	0	0	0	0	0	0	0	0	0	0	0	0	0	0	0	0	0	0
Flexistipes	1	11	0	0	0	0	0	0	0	0	0	0	0	0	0	0	0	0	0	0	0
Fluoribacter	1	6	0	0	0	0	0	0	1	0	0	0	0	0	0	0	0	0	0	0	0
Fluviicola	2	69	1	0	0	0	0	0	0	0	0	0	0	0	0	1	0	0	0	1	1
Formosa	1	2	0	0	0	0	0	0	0	0	0	0	0	0	0	0	0	0	0	0	0
Francisella	2	88	0	0	0	0	0	0	1	0	0	0	0	0	0	1	0	0	0	1	1
Frankia	3	202	1	1	0	1	0	0	1	1	0	0	0	1	1	1	1	0	0	1	1
Frateuria	1	25	0	0	0	0	0	0	0	0	0	0	0	0	0	0	0	0	0	0	0
Fructobacillus	1	2	0	0	0	0	0	0	0	0	0	0	0	0	0	0	0	0	0	1	0
Fulvimarina	1	2	0	0	0	0	0	0	0	0	0	0	0	0	0	0	0	0	0	0	0
Fusobacterium	1	48	0	0	0	0	0	1	0	0	0	0	1	0	0	0	0	1	0	1	1
Gallibacterium	2	87	1	0	0	1	0	0	1	1	0	0	0	0	0	1	0	0	0	1	0
Gallionella	2	67	0	0	0	0	0	0	1	0	0	0	0	0	0	1	0	0	0	1	1
Gammaproteobacteria	1	9	0	0	0	0	0	0	0	0	0	0	0	0	0	0	0	0	0	0	0
Gardnerella	1	31	0	0	0	0	0	0	0	0	0	0	0	0	0	0	0	0	0	1	0
Geitlerinema	1	9	0	0	0	0	0	0	0	0	0	0	0	0	0	0	0	0	0	0	0
Gemmatimonas	2	93	1	0	0	0	0	1	1	0	0	0	0	0	1	1	0	0	0	0	1
Geobacillus	2	168	0	1	0	1	0	0	1	1	0	0	0	1	1	1	0	1	0	1	1
Geobacter	2	150	0	0	0	1	0	1	1	1	0	0	0	0	0	1	0	0	1	1	1
Geodermatophilus	2	136	1	0	0	1	0	0	0	1	0	0	0	0	0	1	0	1	0	0	1
Geopsychrobacter	1	2	0	0	0	0	0	0	0	0	0	0	0	0	0	0	0	0	0	0	0
Gillisia	1	11	0	0	0	0	0	0	0	1	0	0	0	0	0	0	0	0	0	0	0
Gilvimarinus	1	4	0	0	0	0	0	0	0	0	0	0	0	0	0	0	0	0	0	0	0
Glaciecola	2	117	0	1	0	1	0	0	1	1	0	0	0	0	0	1	0	0	0	1	1

Genus	Cluster Number	Protein Counts	3.7.1.2	3.7.1.9	3.8.1.-	3.8.1.2	3.8.1.3	3.8.1.5	4.1.1.-	4.1.1.44	4.1.1.55	4.1.1.7	4.1.1.70	4.1.1.77	4.1.2.-	4.1.3.-	4.1.3.39	4.1.99.-	4.1.99.11	4.2.1.-	4.2.1.17
Gloeobacter	2	67	0	0	0	0	0	0	1	0	0	0	0	0	0	1	0	0	0	1	1
Gloeocapsa	1	20	0	0	0	0	0	0	1	0	0	0	0	0	0	0	0	0	0	0	0
Gloeothece	1	1	0	0	0	0	0	0	0	0	0	0	0	0	0	0	0	0	0	0	0
Gluconacetobacter	2	96	0	0	0	0	0	0	1	1	0	0	0	0	0	1	0	0	0	1	1
Gluconobacter	2	76	0	0	0	0	0	0	1	0	0	0	0	0	0	1	0	0	0	1	0
Gordonia	1	158	0	1	0	0	0	0	0	1	0	0	0	1	0	1	1	0	0	1	1
Gordonibacter	1	32	0	0	0	1	0	0	0	1	0	0	0	0	0	0	0	0	0	0	0
Gramella	2	104	1	0	0	1	0	0	1	1	0	0	0	1	0	1	0	0	0	1	1
Granulibacter	2	90	0	0	0	0	0	0	0	1	0	0	0	0	0	1	0	0	0	1	0
Granulicella	2	98	0	0	0	0	0	0	1	1	1	0	0	0	0	1	0	0	0	1	1
Grimontia	1	9	0	0	0	0	0	0	0	0	0	0	0	0	0	1	0	0	0	0	0
Haemophilus	2	117	0	0	0	1	0	0	1	1	0	0	0	0	0	1	0	0	0	1	0
Hafnia	1	29	0	0	0	0	0	0	1	0	0	0	0	0	0	1	0	0	0	0	0
Hahella	3	130	1	0	0	0	0	1	1	0	0	0	0	0	0	1	0	0	0	1	1
Halalkalicoccus	1	18	0	0	0	0	0	0	0	0	0	0	0	0	0	0	0	0	0	0	0
Halanaerobium	2	89	0	0	0	0	0	0	0	0	0	0	0	0	0	1	0	0	0	1	1
Haliangium	2	108	1	0	0	0	0	1	0	0	0	0	0	0	0	1	1	0	0	1	1
Haliscomenobacter	2	107	1	0	0	0	0	0	1	0	0	0	0	0	1	1	0	0	0	0	1
Haloarcula	1	22	0	0	0	0	0	0	0	0	0	0	0	0	0	0	0	0	0	0	0
Halobacillus	1	19	0	0	0	0	0	0	0	0	0	0	0	0	0	0	0	0	0	0	0
Halobacteriaceae	1	1	0	0	0	0	0	0	0	0	0	0	0	0	0	0	0	0	0	0	0
Halobacterium	1	10	0	0	0	0	0	0	0	0	0	0	0	0	0	0	0	0	0	0	0
Halobacteroides	1	14	0	0	0	0	0	0	0	0	0	0	0	0	0	0	0	0	0	0	0
Haloferax	1	20	0	0	0	0	0	0	0	0	0	0	0	0	0	0	0	0	0	0	0
Halogeometricum	1	12	0	0	0	0	0	0	0	0	0	0	0	0	0	0	0	0	0	0	0
Halomicrobium	1	10	0	0	0	0	0	0	0	0	0	0	0	0	0	0	0	0	0	0	0
Halomonas	1	149	0	0	0	1	0	0	1	1	0	1	0	0	1	1	0	0	0	1	1
Halopiger	1	16	0	0	0	0	0	0	0	0	0	0	0	0	0	0	0	0	0	0	0
Haloquadratum	1	19	0	0	0	0	0	0	0	0	0	0	0	0	0	0	0	0	0	0	0
Halorhabdus	1	10	0	0	0	0	0	0	0	0	0	0	0	0	0	0	0	0	0	0	0
Halorhodospira	2	71	0	0	0	1	0	0	1	0	0	0	0	0	0	1	0	0	0	1	0
Halorubrum	1	16	0	0	0	0	0	0	0	0	0	0	0	0	0	0	0	0	0	0	0
Haloterrigena	1	19	0	0	0	0	0	0	0	0	0	0	0	0	0	0	0	0	0	0	0
Halothece	1	9	0	0	0	0	0	0	0	0	0	0	0	0	0	0	0	0	0	0	0
Halothermothrix	2	50	0	0	0	0	0	0	0	0	0	0	0	0	0	1	0	1	0	1	0
Halothiobacillus	2	65	0	0	0	0	0	0	0	1	0	0	0	0	0	1	0	0	0	1	0
Halovivax	1	12	0	0	0	0	0	0	0	0	0	0	0	0	0	0	0	0	0	0	0
Helicobacter	2	79	0	0	0	0	0	0	1	1	0	0	0	0	0	1	0	0	0	1	0
Heliobacillus	1	4	0	0	0	0	0	0	0	0	0	0	0	0	0	1	0	0	0	0	0
Heliobacterium	2	51	0	0	0	0	0	0	0	0	0	0	0	0	0	1	0	1	0	1	0
Herbaspirillum	2	172	1	1	0	1	0	1	1	0	0	1	0	1	0	1	0	0	0	1	1
Herminiimonas	3	89	1	0	0	1	1	1	1	0	1	0	0	0	0	1	0	0	0	1	1
Herpetosiphon	3	90	1	0	0	0	0	0	1	0	0	0	0	0	0	1	0	0	0	1	1
Hippea	2	44	0	0	0	0	0	0	1	0	0	0	0	0	0	1	0	0	0	1	1
Hirschia	2	102	0	0	0	1	0	0	1	1	1	0	0	0	1	1	0	0	0	1	1
Histophilus	2	72	0	0	0	0	0	0	1	1	0	0	0	0	0	0	0	0	0	1	0
Hoeflea	1	16	0	0	0	0	0	0	0	0	0	0	0	0	0	1	0	0	0	0	0
Hydrogenivirga	1	2	0	0	0	0	0	0	0	0	0	0	0	0	0	0	0	0	0	0	0
Hydrogenobacter	2	55	0	0	0	0	0	0	1	0	0	0	0	0	0	1	0	0	0	1	0
Hydrogenobaculum	2	44	0	0	0	0	0	0	0	0	0	0	0	0	0	1	0	0	0	1	0
Hydrogenophaga	1	9	0	0	0	0	0	0	0	0	0	0	0	0	0	0	0	0	0	0	0
Hylemonella	1	4	0	0	0	0	0	0	0	0	0	0	0	0	0	0	0	0	0	0	0

Genus	Cluster Number	Protein Counts	3.7.1.2	3.7.1.9	3.8.1.-	3.8.1.2	3.8.1.3	3.8.1.5	4.1.1.-	4.1.1.44	4.1.1.55	4.1.1.7	4.1.1.70	4.1.1.77	4.1.2.-	4.1.3.-	4.1.3.39	4.1.99.-	4.1.99.11	4.2.1.-	4.2.1.17
Hyperthermus	1	6	0	0	0	0	0	0	0	0	0	0	0	0	0	0	0	0	0	0	0
Hyphomicrobium	2	93	0	0	0	0	0	0	1	0	0	0	0	0	0	1	0	0	0	1	1
Hyphomonas	3	105	1	0	0	0	0	1	1	1	0	0	0	0	0	1	0	0	0	1	1
Ideonella	1	18	0	0	0	0	0	0	0	0	0	0	0	0	0	0	0	0	0	0	0
Idiomarina	2	86	1	0	0	1	0	0	1	0	0	0	0	0	0	1	0	0	0	1	1
Ignavibacterium	1	14	0	0	0	0	0	0	0	0	0	0	0	0	0	0	0	0	0	0	0
Ignicoccus	1	5	0	0	0	0	0	0	0	0	0	0	0	0	0	0	0	0	0	0	0
Ignisphaera	1	8	0	0	0	0	0	0	0	0	0	0	0	0	0	0	0	0	0	0	0
Ilyobacter	2	85	0	0	0	1	0	0	0	0	0	0	0	0	0	1	0	0	0	1	0
Intrasporangium	2	114	1	1	0	1	0	0	0	1	0	0	0	1	1	1	0	0	0	1	1
Isoptericola	1	18	0	0	0	0	0	0	0	0	0	0	0	0	0	0	0	0	0	0	0
Isosphaera	2	75	0	0	0	0	0	0	1	0	0	0	0	0	1	1	0	0	0	1	1
Jannaschia	3	151	1	0	0	1	0	1	0	1	0	0	0	0	1	1	0	0	0	1	1
Janthinobacterium	2	99	1	0	0	1	1	0	1	1	0	0	0	0	0	1	0	0	0	1	1
Jonesia	2	56	0	0	0	1	0	0	0	0	0	0	0	0	0	1	0	0	0	0	1
Joostella	1	3	0	0	0	0	0	0	0	1	0	0	0	0	0	0	0	0	0	0	0
Kaistia	1	2	0	0	0	0	0	0	0	0	0	0	0	0	0	0	0	0	0	0	0
Kangiella	2	84	0	0	0	1	0	0	1	0	0	0	0	0	0	1	0	0	0	1	1
Ketogulonicigenium	2	102	0	0	0	0	0	1	1	1	0	0	0	0	1	1	0	0	0	1	0
Kineococcus	1	24	0	0	0	0	0	0	0	0	0	0	0	0	0	0	0	0	0	0	0
Kinetoplastibacterium	1	2	0	0	0	0	0	0	0	0	0	0	0	0	0	0	0	0	0	0	0
Kingella	1	4	0	0	0	0	0	0	1	0	0	0	0	0	0	0	0	0	0	0	0
Kitasatospora	1	36	0	0	0	0	0	0	0	0	0	0	0	0	0	0	0	0	0	0	0
Klebsiella	3	222	1	0	0	1	0	0	1	1	0	1	0	0	1	1	1	0	0	1	1
Kluyvera	1	1	0	0	0	0	0	0	1	0	0	0	0	0	0	0	0	0	0	0	0
Kocuria	2	87	0	0	0	0	0	1	0	1	0	0	0	0	1	1	0	0	0	1	1
Kordia	1	5	0	0	0	0	0	0	0	0	0	0	0	0	0	0	0	0	0	0	0
Kosmotoga	2	61	0	0	0	1	0	0	0	1	0	0	0	0	0	0	0	0	0	1	0
Kribbella	2	99	1	0	0	1	1	1	0	0	0	0	0	0	1	1	0	1	0	1	1
Krokinobacter	2	90	1	0	0	1	0	1	0	1	0	0	0	0	0	1	0	0	0	1	1
Kyrpidia	2	113	1	1	0	0	0	0	1	0	0	0	0	1	0	1	0	1	0	1	1
Kytococcus	2	70	1	0	0	0	0	0	0	0	0	0	0	0	1	1	0	0	0	1	1
Labrenzia	1	4	0	0	0	0	0	0	0	0	0	0	0	0	0	0	0	0	0	0	0
Lachnoanaerobaculum	1	1	0	0	0	0	0	0	0	1	0	0	0	0	0	0	0	0	0	0	0
Lachnospiraceae	1	51	0	0	0	1	0	0	1	1	0	0	0	0	0	0	0	0	0	0	0
Lacinutrix	1	20	0	0	0	0	0	0	0	1	0	0	0	0	0	0	0	0	0	0	0
Lactobacillus	2	119	0	0	0	1	0	1	1	1	0	0	0	0	0	1	0	0	0	1	0
Lactococcus	2	82	0	0	0	1	0	1	1	1	0	0	0	0	0	1	0	0	0	1	0
Lamprocystis	1	2	0	0	0	0	0	0	0	0	0	0	0	0	0	0	0	0	0	0	0
Laribacter	2	80	0	0	0	0	0	1	1	1	0	0	0	0	0	1	0	0	0	1	1
Lawsonia	2	33	0	0	0	0	0	0	0	0	0	0	0	0	0	0	0	0	0	1	0
Leadbetterella	2	79	1	0	0	1	0	1	1	0	0	0	0	0	0	1	0	0	0	1	1
Leeuwenhoekiella	1	2	0	0	0	0	0	0	0	0	0	0	0	0	0	0	0	0	0	0	0
Legionella	2	117	1	0	0	0	0	1	1	1	0	1	0	0	0	1	1	0	0	1	1
Leifsonia	2	48	0	0	0	0	0	0	0	0	0	0	0	0	0	1	0	0	0	1	1
Leptolyngbya	1	10	0	0	0	0	0	0	0	0	0	0	0	0	0	0	0	0	0	0	0
Leptospira	2	76	0	0	0	0	0	1	1	0	0	0	0	0	0	1	0	1	0	1	1
Leptospirillum	1	10	0	0	0	0	0	0	0	0	0	0	0	0	0	0	0	0	0	0	0
Leptothrix	3	186	1	1	0	1	0	1	1	1	0	0	0	1	1	1	0	0	0	1	1
Leptotrichia	2	43	0	0	0	0	0	0	0	0	0	0	0	0	1	1	0	0	0	1	0
Leucobacter	1	2	0	0	0	0	0	0	0	0	0	0	0	0	0	0	0	0	0	0	0
Leuconostoc	2	64	0	0	0	0	0	0	0	1	0	0	0	0	0	1	0	0	0	1	0

Genus	Cluster Number	Protein Counts	3.7.1.2	3.7.1.9	3.8.1.-	3.8.1.2	3.8.1.3	3.8.1.5	4.1.1.-	4.1.1.44	4.1.1.55	4.1.1.7	4.1.1.70	4.1.1.77	4.1.2.-	4.1.3.-	4.1.3.39	4.1.99.-	4.1.99.11	4.2.1.-	4.2.1.17
Liberibacter	1	4	0	0	0	0	0	0	0	0	0	0	0	0	0	0	0	0	0	0	0
Limnohabitans	1	11	0	0	0	0	0	0	0	0	0	0	0	0	0	0	0	0	0	0	0
Listeria	2	77	0	0	0	1	0	0	0	1	0	0	0	0	0	1	0	0	0	1	0
Listeriaceae	1	6	0	0	0	0	0	0	0	0	0	0	0	0	0	0	0	0	0	0	0
Listonella	1	38	0	0	0	0	0	0	0	0	0	0	0	0	0	1	0	0	0	0	0
Loktanella	1	3	0	0	0	0	0	0	0	0	0	0	0	0	0	0	0	0	0	0	0
Lysinibacillus	2	97	1	0	0	1	0	0	1	0	0	0	0	0	0	1	0	1	0	1	1
Macrococcus	2	60	0	0	0	0	0	0	1	0	0	0	0	1	0	0	0	0	0	1	0
Magnetococcus	2	64	0	0	0	0	0	1	1	0	0	0	0	0	0	1	0	0	0	1	0
Magnetospirillum	2	113	0	0	0	1	0	0	1	1	0	0	0	0	1	1	0	0	0	1	1
Mahella	2	58	0	0	0	0	0	0	1	1	0	0	0	0	0	1	0	0	0	1	0
Mannheimia	2	85	0	0	0	1	0	0	1	1	0	0	0	0	1	1	0	0	0	1	0
Maribacter	3	87	1	0	0	1	0	1	0	1	0	0	0	0	0	1	0	0	0	1	1
Maricaulis	3	87	0	0	0	0	0	0	1	0	0	0	0	0	0	1	0	0	0	1	1
Marichromatium	1	2	0	0	0	0	0	0	0	0	0	0	0	0	0	0	0	0	0	0	0
Marinithermus	2	77	0	0	0	0	0	0	1	1	0	0	0	0	0	1	0	0	0	1	1
Marinitoga	1	15	0	0	0	0	0	0	0	0	0	0	0	0	0	0	0	0	0	0	0
Marinobacter	2	163	0	1	0	1	0	1	1	0	0	0	0	1	0	1	0	0	0	1	1
Marinomonas	3	192	1	0	0	1	0	1	0	1	0	0	0	0	1	1	0	0	0	1	1
Maritimibacter	1	7	0	0	0	0	0	0	0	0	0	0	0	0	0	0	0	0	0	0	0
Marivirga	2	96	1	0	0	1	0	0	0	0	0	0	0	0	0	1	0	0	0	1	1
Martelella	1	3	0	0	0	0	0	0	0	0	0	0	0	0	0	0	0	0	0	0	0
Marvinbryantia	1	1	0	0	0	0	0	0	0	0	0	0	0	0	0	0	0	0	0	0	0
Massilia	1	4	0	0	0	0	0	0	0	0	0	0	0	0	0	0	1	0	0	0	0
Mastigocladopsis	1	16	0	0	0	0	0	0	1	0	0	0	0	0	0	1	0	0	0	0	0
Megamonas	1	47	0	0	0	0	0	0	1	1	0	0	0	0	0	1	0	0	0	0	1
Megasphaera	1	14	0	0	0	0	0	0	0	0	0	0	0	0	0	0	0	0	0	0	0
Meiothermus	2	110	0	0	0	1	0	0	0	1	0	0	0	0	0	1	0	0	0	1	1
Melissococcus	2	46	0	0	0	0	0	0	1	1	0	0	0	0	0	0	0	0	0	1	0
Mesoflavibacter	1	6	0	0	0	0	0	0	0	1	0	0	0	0	0	0	0	0	0	0	0
Mesoplasma	1	23	0	0	0	0	0	0	0	0	0	0	0	0	0	0	0	0	0	0	0
Mesorhizobium	2	201	1	0	0	1	0	1	0	1	0	0	0	0	1	1	0	0	0	1	1
Mesotoga	1	14	0	0	0	0	0	0	0	0	0	0	0	0	0	0	0	0	0	0	0
Metallosphaera	1	18	0	0	0	0	0	0	0	0	0	0	0	0	0	0	0	0	0	0	0
Methanobacterium	1	7	0	0	0	0	0	0	0	0	0	0	0	0	0	0	0	0	0	0	0
Methanobrevibacter	1	9	0	0	0	0	0	0	0	0	0	0	0	0	0	0	0	0	0	0	0
Methanocaldococcus	1	6	0	0	0	0	0	0	0	0	0	0	0	0	0	0	0	0	0	0	0
Methanocella	1	15	0	0	0	0	0	0	0	0	0	0	0	0	0	0	0	0	0	0	0
Methanococcaceae	1	1	0	0	0	0	0	0	0	0	0	0	0	0	0	0	0	0	0	0	0
Methanococcoides	1	5	0	0	0	0	0	0	0	0	0	0	0	0	0	0	0	0	0	0	0
Methanococcus	1	9	0	0	0	0	0	0	0	0	0	0	0	0	0	0	0	0	0	0	0
Methanocorpusculum	1	7	0	0	0	0	0	0	0	0	0	0	0	0	0	0	0	0	0	0	0
Methanoculleus	1	8	0	0	0	0	0	0	0	0	0	0	0	0	0	0	0	0	0	0	0
Methanohalobium	1	5	0	0	0	0	0	0	0	0	0	0	0	0	0	0	0	0	0	0	0
Methanohalophilus	1	5	0	0	0	0	0	0	0	0	0	0	0	0	0	0	0	0	0	0	0
Methanolobus	1	5	0	0	0	0	0	0	0	0	0	0	0	0	0	0	0	0	0	0	0
Methanomethylovorans	1	5	0	0	0	0	0	0	0	0	0	0	0	0	0	0	0	0	0	0	0
Methanoplanus	1	7	0	0	0	0	0	0	0	0	0	0	0	0	0	0	0	0	0	0	0
Methanopyrus	1	4	0	0	0	0	0	0	0	0	0	0	0	0	0	0	0	0	0	0	0
Methanoregula	1	8	0	0	0	0	0	0	0	0	0	0	0	0	0	0	0	0	0	0	0
Methanosaeta	1	9	0	0	0	0	0	0	0	0	0	0	0	0	0	0	0	0	0	0	0
Methanosalsum	1	7	0	0	0	0	0	0	0	0	0	0	0	0	0	0	0	0	0	0	0

Genus	Cluster Number	Protein Counts	3.7.1.2	3.7.1.9	3.8.1.-	3.8.1.2	3.8.1.3	3.8.1.5	4.1.1.-	4.1.1.44	4.1.1.55	4.1.1.7	4.1.1.70	4.1.1.77	4.1.2.-	4.1.3.-	4.1.3.39	4.1.99.-	4.1.99.11	4.2.1.-	4.2.1.17
**Methanosarcina**	1	18	0	0	0	0	0	0	0	0	0	0	0	0	0	0	0	0	0	0	0
**Methanosphaera**	1	7	0	0	0	0	0	0	0	0	0	0	0	0	0	0	0	0	0	0	0
**Methanosphaerula**	1	8	0	0	0	0	0	0	0	0	0	0	0	0	0	0	0	0	0	0	0
**Methanospirillum**	1	6	0	0	0	0	0	0	0	0	0	0	0	0	0	0	0	0	0	0	0
**Methanothermobacter**	1	6	0	0	0	0	0	0	0	0	0	0	0	0	0	0	0	0	0	0	0
**Methanothermococcus**	1	5	0	0	0	0	0	0	0	0	0	0	0	0	0	0	0	0	0	0	0
**Methanothermus**	1	5	0	0	0	0	0	0	0	0	0	0	0	0	0	0	0	0	0	0	0
**Methanotorris**	1	5	0	0	0	0	0	0	0	0	0	0	0	0	0	0	0	0	0	0	0
**Methylacidiphilum**	2	48	0	0	0	0	0	0	1	0	0	0	0	0	0	1	0	0	0	0	0
**Methylarcula**	1	2	0	0	0	0	0	0	0	0	0	0	0	0	0	0	0	0	0	1	0
**Methylibium**	1	38	0	0	0	0	0	0	0	0	0	0	0	0	0	0	0	0	0	0	0
**Methylobacillus**	2	61	0	0	0	0	0	0	0	0	0	0	0	0	0	1	0	0	0	1	0
**Methylobacterium**	3	236	1	0	0	1	0	1	1	1	0	1	0	1	1	1	0	1	0	1	1
**Methylocella**	3	117	0	0	0	1	0	0	1	1	0	1	0	1	0	1	1	0	0	1	1
**Methylococcus**	2	80	0	0	0	0	0	0	1	0	0	0	0	0	0	1	0	0	0	1	0
**Methylocystis**	1	21	0	0	0	0	0	0	0	0	0	0	0	0	0	0	0	0	0	0	0
**Methyloferula**	1	2	0	0	0	0	0	0	0	0	0	0	0	0	0	0	0	0	0	0	0
**Methylomicrobium**	1	12	0	0	0	0	0	0	0	0	0	0	0	0	0	0	0	0	0	0	0
**Methylomonas**	1	15	0	0	0	0	0	0	0	0	0	0	0	0	0	0	0	0	0	0	0
**Methylophaga**	1	7	0	0	0	0	0	0	0	0	0	0	0	0	0	0	0	0	0	0	0
**Methylophilus**	1	2	0	0	0	0	0	0	0	0	0	0	0	0	0	0	0	0	0	0	0
**Methylosinus**	1	2	0	0	0	0	0	0	0	0	0	0	0	0	0	0	0	0	0	0	0
**Methylotenera**	2	72	0	0	0	0	0	0	0	1	0	0	0	0	0	1	0	0	0	1	1
**Methyloversatilis**	1	5	0	0	0	0	0	0	0	0	0	0	0	0	0	0	0	0	0	0	0
**Methylovorus**	2	66	0	0	0	0	0	0	0	0	0	0	0	0	0	1	0	0	0	1	0
**Micavibrio**	1	9	0	0	0	0	0	0	0	0	0	0	0	0	0	0	0	0	0	0	0
**Microbacterium**	2	115	1	0	0	0	0	0	1	1	0	0	0	0	1	1	0	1	0	0	1
**Microchaete**	1	23	0	0	0	0	0	0	1	0	0	0	0	0	0	0	0	0	0	0	0
**Micrococcus**	2	72	0	0	0	0	0	0	0	0	0	0	0	0	1	1	0	0	0	1	1
**Microcoleus**	1	17	0	0	0	0	0	0	1	0	0	0	0	0	0	0	0	0	0	0	0
**Microcystis**	2	67	0	0	0	0	0	0	1	0	0	0	0	0	0	1	0	0	0	1	0
**Microlunatus**	1	25	0	0	0	0	0	0	0	0	0	0	0	0	0	0	0	0	0	0	0
**Micromonospora**	2	115	1	0	0	1	0	0	1	0	0	1	0	1	0	1	1	0	0	1	1
**Microvirga**	1	6	0	0	0	0	0	0	0	0	0	0	0	0	0	0	0	0	0	0	0
**Mobiluncus**	1	41	0	0	0	0	0	0	0	0	0	0	0	0	0	1	0	0	0	0	0
**Modestobacter**	1	43	0	0	0	0	0	0	0	0	0	0	0	0	0	0	0	0	0	0	0
**Moorea**	1	2	0	0	0	0	0	0	1	0	0	0	0	0	0	0	0	0	0	0	0
**Moorella**	2	69	0	0	0	0	0	0	1	1	0	0	0	1	0	1	1	1	0	1	0
**Moraxella**	2	66	0	0	0	0	0	0	1	1	0	0	0	0	0	1	0	0	0	1	1
**Morganella**	1	29	0	0	0	0	0	0	1	0	0	0	0	0	0	0	0	0	0	0	0
**Moritella**	1	2	0	0	0	0	0	0	0	0	0	0	0	0	0	0	0	0	0	0	0
**Muricauda**	1	23	0	0	0	0	0	0	0	1	0	0	0	0	0	0	0	0	0	0	0
**Mycobacterium**	3	306	1	1	0	1	1	1	1	1	1	1	0	1	1	1	1	1	0	1	1
**Mycoplasma**	1	53	0	0	0	0	0	0	1	1	0	0	0	0	0	0	0	0	0	0	0
**Myxococcus**	2	110	0	0	0	0	0	1	0	0	0	0	0	1	0	1	0	0	0	1	1
**Nakamurella**	2	115	1	1	0	0	0	1	0	0	0	0	0	1	1	1	1	0	0	1	1
**napthalene**	1	2	0	0	0	0	0	0	0	0	0	0	0	0	0	0	0	0	0	0	0
**Natranaerobius**	2	55	0	0	0	0	0	0	0	1	0	0	0	0	0	1	0	0	0	1	0
**Natrialba**	1	16	0	0	0	0	0	0	0	0	0	0	0	0	0	0	0	0	0	0	0
**Natrinema**	1	16	0	0	0	0	0	0	0	0	0	0	0	0	0	0	0	0	0	0	0
**Natronobacterium**	1	11	0	0	0	0	0	0	0	0	0	0	0	0	0	0	0	0	0	0	0
**Natronococcus**	1	21	0	0	0	0	0	0	0	0	0	0	0	0	0	0	0	0	0	0	0

Genus	Cluster Number	Protein Counts	3.7.1.2	3.7.1.9	3.8.1.-	3.8.1.2	3.8.1.3	3.8.1.5	4.1.1.-	4.1.1.44	4.1.1.55	4.1.1.7	4.1.1.70	4.1.1.77	4.1.2.-	4.1.3.-	4.1.3.39	4.1.99.-	4.1.99.11	4.2.1.-	4.2.1.17
Natronomonas	1	20	0	0	0	0	0	0	0	0	0	0	0	0	0	0	0	0	0	0	0
Nautilia	2	48	0	0	0	0	0	0	1	0	0	0	0	0	0	1	0	1	0	1	0
Neisseria	2	67	0	0	0	1	0	0	1	0	0	0	0	0	0	1	0	0	0	1	0
Neisseriaceae	1	1	0	0	0	0	0	0	0	1	0	0	0	0	0	0	0	0	0	0	0
Neorickettsia	1	31	0	0	0	0	0	0	0	0	0	0	0	0	0	0	0	0	0	1	0
Neptuniibacter	1	2	0	0	0	0	0	0	0	0	0	0	0	0	0	0	0	0	0	0	0
Niastella	1	21	0	0	0	0	0	0	0	0	0	0	0	0	0	0	0	0	0	0	0
Nitratifractor	2	54	0	0	0	0	0	0	1	0	0	0	0	0	0	1	0	0	0	1	0
Nitratireductor	1	19	0	0	0	0	0	0	1	0	0	0	0	0	0	1	0	0	0	0	0
Nitratiruptor	2	40	0	0	0	0	0	0	0	0	0	0	0	0	0	1	0	1	0	1	0
Nitrobacter	2	99	1	0	0	0	0	0	0	1	0	1	0	0	0	1	0	0	0	1	1
Nitrococcus	1	2	0	0	0	0	0	0	0	0	0	0	0	0	0	0	0	0	0	0	0
Nitrosococcus	2	94	1	0	0	1	0	0	1	0	0	0	0	0	0	1	0	0	0	1	1
Nitrosomonas	2	72	0	0	0	0	0	0	1	0	0	0	0	0	1	1	0	0	0	1	1
Nitrosopumilaceae	1	7	0	0	0	0	0	0	0	0	0	0	0	0	0	0	0	0	0	0	0
Nitrosopumilus	1	11	0	0	0	0	0	0	0	0	0	0	0	0	0	0	0	0	0	0	0
Nitrosospira	2	74	0	0	0	0	0	0	1	0	0	0	0	0	1	1	0	0	0	1	1
Nocardia	2	185	1	0	0	0	0	1	0	1	0	0	0	1	1	1	1	0	0	1	1
Nocardioidaceae	1	2	0	0	0	0	0	0	0	0	0	0	0	0	0	0	0	0	0	0	0
Nocardioides	2	157	1	0	0	1	0	1	0	1	0	0	0	1	1	1	1	0	0	1	1
Nocardiopsis	2	102	1	0	0	1	0	0	0	0	0	0	0	0	0	1	1	0	0	1	1
Nodularia	1	22	0	0	0	0	0	0	1	0	0	0	0	0	0	1	0	0	0	0	0
Nonlabens	1	19	0	0	0	0	0	0	0	0	0	0	0	0	0	0	0	0	0	0	0
Nostoc	2	111	0	0	0	1	0	1	1	1	0	1	0	0	1	1	0	1	0	1	1
Nostocaceae	1	2	0	0	0	0	0	0	0	0	0	0	0	0	0	0	0	0	0	0	0
Novosphingobium	2	169	1	0	0	0	0	1	1	0	0	1	0	0	0	1	1	0	0	1	1
Oceanibaculum	1	3	0	0	0	0	0	0	0	0	0	0	0	0	0	0	0	0	0	0	0
Oceanibulbus	1	12	0	0	0	0	0	0	0	0	0	0	0	0	0	0	0	0	0	1	0
Oceanicola	1	9	0	0	0	0	0	0	0	0	0	0	0	0	0	0	0	0	0	0	0
Oceanimonas	1	33	0	0	0	0	0	0	0	0	0	0	0	0	0	0	0	0	0	0	0
Oceaniovalibus	1	2	0	0	0	0	0	0	0	0	0	0	0	0	0	1	0	0	0	0	0
Oceanithermus	2	72	0	0	0	0	0	0	1	1	0	0	0	0	0	1	0	0	0	1	1
Oceanobacillus	2	91	0	0	0	1	0	0	0	1	0	0	0	0	0	1	0	1	0	1	1
Oceanospirillum	1	2	0	0	0	0	0	0	0	0	0	0	0	0	0	0	0	0	0	0	0
Ochrobactrum	2	133	0	0	0	1	0	1	1	1	0	0	0	0	0	1	0	0	0	1	1
Octadecabacter	1	29	0	0	0	0	0	0	0	0	0	0	0	0	0	0	0	0	0	0	0
Oenococcus	2	41	0	0	0	0	0	0	1	0	0	0	0	0	0	0	0	0	0	1	0
Oleomonas	1	2	0	0	0	0	0	0	0	0	0	0	0	0	0	0	0	0	0	0	0
Oligotropha	3	87	1	0	0	1	0	0	1	1	0	0	0	0	0	1	0	0	0	1	1
Olsenella	2	40	0	0	0	0	0	0	1	0	0	0	0	0	0	1	0	0	0	0	1
Opitutus	2	67	0	0	0	0	0	0	0	0	0	0	0	0	0	1	0	0	0	1	0
Orientia	1	21	0	0	0	0	0	0	1	0	0	0	0	0	0	0	0	0	0	1	0
Ornithobacterium	1	11	0	0	0	0	0	0	0	0	0	0	0	0	0	0	0	0	0	0	0
Oscillatoria	1	16	0	0	0	0	0	0	1	0	0	0	0	0	0	0	0	0	0	0	0
Oscillatoriales	1	2	0	0	0	0	0	0	0	0	0	0	0	0	0	0	0	0	0	0	0
Oscillibacter	1	20	0	0	0	0	0	0	0	0	0	0	0	0	0	0	0	0	0	0	0
Owenweeksia	1	15	0	0	0	0	0	0	0	0	0	0	0	0	0	0	0	0	0	0	0
Oxalobacteraceae	1	4	0	0	0	0	0	0	0	0	0	0	0	0	0	0	0	0	0	0	0
Paenibacillus	2	152	1	0	0	1	0	1	1	1	0	0	0	0	0	1	0	1	0	1	1
Paludibacter	2	67	0	0	0	0	0	0	1	0	0	0	0	0	0	1	0	0	0	1	0
Pandoraea	1	18	0	0	0	0	0	0	0	0	1	0	0	0	0	1	0	0	0	0	0
Pannonibacter	1	3	0	0	0	0	0	0	0	0	0	0	0	0	0	0	0	0	0	0	0

Genus	Cluster Number	Protein Counts	3.7.1.2	3.7.1.9	3.8.1.-	3.8.1.2	3.8.1.3	3.8.1.5	4.1.1.-	4.1.1.44	4.1.1.55	4.1.1.7	4.1.1.70	4.1.1.77	4.1.2.-	4.1.3.-	4.1.3.39	4.1.99.-	4.1.99.11	4.2.1.-	4.2.1.17
Pantholops	1	23	0	0	0	0	0	0	0	0	0	0	0	0	0	0	0	0	0	0	1
Pantoea	2	181	0	0	0	1	0	1	1	1	1	0	0	0	1	1	0	0	0	1	1
Parabacteroides	2	64	0	0	0	1	0	0	1	1	0	0	0	0	0	1	0	0	0	1	0
Parachlamydia	1	11	0	0	0	0	0	0	0	0	0	0	0	0	0	0	0	0	0	0	0
Paracoccus	2	169	1	0	0	1	0	1	1	1	1	0	0	0	1	1	0	0	0	1	1
Parvibaculum	3	117	1	0	0	1	0	1	1	0	0	0	0	0	0	1	0	0	0	1	1
Parvularcula	2	72	0	0	0	0	0	1	1	0	0	0	0	0	0	1	0	0	0	1	1
Pasteurella	2	85	0	0	0	1	0	0	1	1	0	0	0	0	1	1	0	0	0	1	0
Pasteurellaceae	1	2	0	0	0	0	0	0	0	0	0	0	0	0	0	0	0	0	0	0	0
Pectobacterium	1	44	0	0	0	0	0	0	1	0	0	0	0	0	0	1	0	0	0	1	0
Pediococcus	1	43	0	0	0	0	0	1	1	1	0	0	0	0	0	0	0	0	0	1	0
Pedobacter	2	88	1	0	0	1	0	0	1	0	0	0	0	0	0	1	0	0	0	1	1
Pelagibaca	1	10	0	0	0	0	0	0	0	0	0	0	0	0	0	1	0	0	0	1	0
Pelagibacterium	1	39	0	0	0	0	0	0	0	0	0	0	0	0	0	0	0	0	0	0	0
Pelobacter	2	85	0	0	0	0	0	0	1	0	0	0	0	0	0	1	0	0	0	1	1
Pelodictyon	2	62	0	0	0	0	0	1	0	0	0	0	0	0	0	1	0	0	0	1	0
Pelosinus	1	1	0	0	0	0	0	0	0	0	0	0	0	0	0	0	0	0	0	0	1
Pelotomaculum	2	60	0	0	0	0	0	0	1	1	0	0	0	0	0	1	1	0	0	1	1
Peptostreptococcus	1	1	0	0	0	0	0	0	0	0	0	0	0	0	0	0	0	0	0	0	0
Persephonella	2	55	0	0	0	0	0	0	1	0	0	0	0	0	0	1	0	0	0	1	0
Petrotoga	2	72	0	0	0	1	0	0	0	1	0	0	0	0	0	1	0	1	0	1	0
Phaeobacter	2	55	0	0	0	0	0	0	0	0	0	0	0	0	0	1	0	0	0	0	1
Phenylobacterium	3	103	1	0	0	1	0	0	1	0	0	0	0	0	0	1	0	0	0	1	1
Photobacterium	2	119	0	0	0	1	0	0	1	1	0	0	0	0	0	1	0	0	0	1	1
Photorhabdus	2	149	0	0	0	1	0	0	1	1	0	0	0	0	1	1	0	0	0	1	1
Phycisphaera	1	9	0	0	0	0	0	0	0	0	0	0	0	0	0	0	0	0	0	0	0
Phyllobacterium	1	6	0	0	0	0	0	0	0	0	0	0	0	0	0	1	0	0	0	0	0
Picrophilus	1	15	0	0	0	0	0	0	0	0	0	0	0	0	0	0	0	0	0	0	0
Pirellula	2	85	0	0	0	0	0	0	1	0	0	1	0	0	1	1	0	0	0	1	1
Planctomyces	2	93	0	0	0	0	0	0	1	0	0	0	0	0	1	1	0	0	0	1	1
Plautia	1	17	0	0	0	0	0	0	1	0	0	0	0	0	0	0	0	0	0	1	0
Plesiomonas	1	4	0	0	0	0	0	0	0	0	0	0	0	0	0	0	0	0	0	0	0
Pleurocapsa	1	23	0	0	0	0	0	0	1	0	0	0	0	0	0	0	0	0	0	0	0
Polaribacter	2	74	1	0	0	0	0	1	0	1	0	0	0	0	0	1	0	0	0	1	1
Polaromonas	3	238	1	0	0	1	1	1	1	1	1	1	0	1	1	1	1	0	0	1	1
Polymorphum	3	173	1	0	0	1	0	0	1	1	1	0	0	0	1	1	0	0	0	1	1
Polynucleobacter	1	19	0	0	0	0	0	0	0	0	0	0	0	0	0	0	0	0	0	0	0
Ponticaulis	1	1	0	0	0	0	0	0	0	0	0	0	0	0	0	0	0	0	0	0	1
Porphyromonas	2	63	0	0	0	0	0	0	1	0	0	0	0	0	0	0	0	0	0	1	1
Prevotella	1	65	0	0	0	1	0	0	1	1	0	0	0	0	0	1	0	0	0	1	0
Prochlorococcus	2	68	0	0	0	0	0	0	1	0	0	0	0	0	0	1	0	0	0	1	0
Propionibacterium	1	62	0	0	0	0	0	0	0	0	0	0	0	0	0	1	0	0	0	0	0
Prosthecochloris	1	16	0	0	0	0	0	0	0	0	0	0	0	0	0	0	0	0	0	1	0
Proteobacteria	1	19	0	0	0	0	0	0	0	1	0	0	0	1	0	0	1	0	0	0	1
Proteus	2	113	0	0	0	1	0	0	1	1	0	0	0	0	0	1	0	0	0	1	1
Providencia	1	38	0	0	0	0	0	0	1	0	0	0	0	0	0	0	0	0	0	1	0
Pseudaminobacter	1	10	0	0	0	0	0	0	0	0	0	0	0	0	0	1	0	0	0	0	0
Pseudanabaena	1	11	0	0	0	0	0	0	0	0	0	0	0	0	0	0	0	0	0	0	0
Pseudoalteromonas	2	188	1	1	0	1	0	1	1	1	0	0	0	0	0	1	1	0	0	1	1
Pseudochrobactrum	1	7	0	0	0	0	0	0	0	0	0	0	0	0	0	0	0	0	0	0	0
Pseudogulbenkiania	1	40	0	0	0	0	0	0	0	1	0	0	0	0	0	0	0	0	0	0	0
pseudomallei	3	131	0	0	0	1	0	0	1	1	0	0	0	0	0	0	0	0	0	1	1

Genus	Cluster Number	Protein Counts	3.7.1.2	3.7.1.9	3.8.1.-	3.8.1.2	3.8.1.3	3.8.1.5	4.1.1.-	4.1.1.44	4.1.1.55	4.1.1.7	4.1.1.70	4.1.1.77	4.1.2.-	4.1.3.-	4.1.3.39	4.1.99.-	4.1.99.11	4.2.1.-	4.2.1.17
Pseudomonas	3	381	1	1	0	1	1	1	1	1	0	1	0	1	1	1	1	0	0	1	1
Pseudonocardia	3	180	1	0	0	0	0	0	1	1	0	0	0	1	1	1	0	0	0	1	1
Pseudorhodobacter	1	4	0	0	0	0	0	0	0	0	0	0	0	0	0	1	0	0	0	0	0
Pseudovibrio	1	33	0	0	0	0	0	0	0	0	0	0	0	0	0	0	0	0	0	0	0
Pseudoxanthomonas	2	124	0	0	0	1	0	0	0	1	0	0	0	1	0	1	1	0	0	1	1
Psychrobacter	2	124	1	0	0	1	0	1	1	1	0	0	0	0	0	1	0	0	0	1	1
Psychroflexus	1	22	0	0	0	0	0	0	0	0	0	0	0	0	0	0	0	0	0	0	0
Psychromonas	2	110	0	0	0	1	0	0	1	0	0	0	0	0	0	1	0	0	0	1	1
Pusillimonas	3	111	1	0	0	1	1	0	1	1	0	0	0	0	1	1	0	0	0	1	1
Pyrobaculum	1	15	0	0	0	0	0	0	0	0	0	0	0	0	0	0	0	0	0	0	0
Pyrococcus	1	10	0	0	0	0	0	0	0	0	0	0	0	0	0	0	0	0	0	0	0
Pyrolobus	1	4	0	0	0	0	0	0	0	0	0	0	0	0	0	0	0	0	0	0	0
Rahnella	2	143	0	0	0	1	0	0	1	1	1	0	0	0	0	1	0	0	0	1	1
Ralstonia	3	332	1	1	0	1	1	1	1	1	1	1	0	1	1	1	1	0	0	1	1
Ramlibacter	1	35	0	0	0	0	0	0	0	0	0	0	0	0	0	0	0	0	0	0	0
Raoultella	2	139	0	0	0	0	0	0	1	1	0	0	0	0	1	1	0	0	0	1	1
Raphidiopsis	1	5	0	0	0	0	0	0	1	0	0	0	0	0	0	0	0	0	0	0	0
Reinekea	1	2	0	0	0	0	0	0	0	0	0	0	0	0	0	0	0	0	0	0	0
Renibacterium	2	67	0	0	0	1	0	0	0	1	0	0	0	0	0	1	0	0	0	1	1
Rhizobiaceae	1	3	0	0	0	0	0	0	0	0	0	0	0	0	0	0	0	0	0	1	0
Rhizobiales	1	6	0	0	0	0	0	0	0	0	0	0	0	0	0	0	0	0	0	0	0
Rhizobium	3	257	1	0	0	1	0	1	1	1	1	0	0	0	1	1	0	0	0	1	1
Rhizobium/Agrobacterium	2	87	0	0	0	1	0	1	0	0	0	0	0	0	1	1	0	0	0	1	1
Rhodanobacter	1	21	0	0	0	0	0	0	0	0	0	0	0	0	0	0	0	0	0	0	0
Rhodobacter	3	175	1	0	0	1	1	1	1	1	0	0	0	0	1	1	0	0	0	1	1
Rhodobacteraceae	1	21	0	0	0	0	0	0	0	0	0	0	0	0	0	1	0	0	0	0	1
Rhodobacterales	1	34	0	0	0	0	0	0	0	0	0	0	0	0	0	1	0	0	0	0	1
Rhodococcus	2	292	1	1	0	0	0	1	0	1	1	1	0	1	1	1	1	0	0	1	1
Rhodocyclaceae	1	7	0	0	0	0	0	0	0	0	0	0	0	0	0	0	0	0	0	0	0
Rhodoferax	3	144	1	0	0	1	0	1	1	1	0	0	0	0	1	1	1	0	0	1	1
Rhodomicrobium	2	103	0	0	0	0	0	0	0	1	0	0	0	0	0	1	0	0	0	1	1
Rhodopirellula	2	87	0	0	0	0	0	0	1	0	0	0	0	0	0	1	0	0	0	1	1
Rhodopseudomonas	3	246	1	0	0	1	1	1	1	1	0	1	0	0	1	1	0	0	0	1	1
Rhodospirillum	2	121	1	0	0	0	0	1	1	0	0	0	0	0	0	1	0	0	0	1	1
Rhodothermus	2	76	0	0	0	1	0	0	0	0	0	0	0	0	0	1	0	0	0	0	1
Rhodovulum	1	4	0	0	0	0	0	0	0	0	0	0	0	0	0	0	0	0	0	0	0
Richelia	1	2	0	0	0	0	0	0	0	0	0	0	0	0	0	0	0	0	0	0	0
Rickettsia	2	43	0	0	0	0	0	0	1	0	0	0	0	0	0	0	0	1	0	1	1
Rickettsiaceae	1	5	0	0	0	0	0	0	0	0	0	0	0	0	0	0	0	0	0	0	0
Riemerella	2	61	1	0	0	1	0	0	0	0	0	0	0	0	0	0	0	0	0	1	1
Rivularia	1	18	0	0	0	0	0	0	1	0	0	0	0	0	0	0	0	0	0	0	0
Robiginitalea	2	83	1	0	0	0	0	0	0	1	0	0	0	0	0	1	0	0	0	1	1
Roseburia	2	58	0	0	0	0	0	0	1	1	0	0	0	0	1	1	0	0	0	1	0
Roseibium	1	3	0	0	0	0	0	0	0	0	0	0	0	0	0	0	0	0	0	0	0
Roseiflexus	3	100	0	1	0	1	0	1	0	0	0	0	0	1	1	1	1	0	0	1	1
Roseobacter	2	149	1	0	0	1	0	0	0	1	0	0	0	0	1	1	0	0	0	1	1
Roseomonas	1	2	0	0	0	0	0	0	0	0	0	0	0	0	0	0	0	0	0	0	0
Roseovarius	1	15	0	0	0	0	0	0	0	0	0	0	0	0	0	1	0	0	0	1	0
Rothia	1	44	0	0	0	0	0	0	0	1	0	0	0	0	0	0	0	0	0	1	1
Rubrivivax	1	24	0	0	0	0	0	0	0	0	0	0	0	0	0	0	0	0	0	0	0
Rubrobacter	3	117	1	0	0	1	0	0	0	1	0	1	0	0	0	1	0	0	0	1	1
Ruegeria	2	189	1	0	0	1	0	0	0	1	0	0	0	0	1	1	0	0	0	1	1

Genus	Cluster Number	Protein Counts	3.7.1.2	3.7.1.9	3.8.1.-	3.8.1.2	3.8.1.3	3.8.1.5	4.1.1.-	4.1.1.44	4.1.1.55	4.1.1.7	4.1.1.70	4.1.1.77	4.1.2.-	4.1.3.-	4.1.3.39	4.1.99.-	4.1.99.11	4.2.1.-	4.2.1.17
Ruminococcaceae	1	2	0	0	0	0	0	0	0	0	0	0	0	0	0	0	0	0	0	0	0
Ruminococcus	2	101	0	0	0	1	0	0	1	1	0	0	0	0	1	1	0	0	0	1	1
Runella	1	26	0	0	0	0	0	0	0	0	0	0	0	0	0	0	0	0	0	0	0
Saccharomonospora	2	132	1	0	0	1	0	0	0	1	0	0	0	0	0	1	0	0	0	1	1
Saccharophagus	2	81	0	0	0	0	0	0	1	0	0	0	0	0	0	1	0	0	0	1	1
Saccharopolyspora	3	194	1	0	0	1	0	1	1	1	0	1	0	0	1	1	0	0	0	1	1
Saccharothrix	1	32	0	0	0	0	0	0	0	0	0	0	0	0	0	0	0	0	0	0	0
Sagittula	1	10	0	0	0	0	0	0	0	0	0	0	0	0	0	0	0	0	0	0	0
Salinibacter	2	77	1	0	0	1	0	0	0	0	0	0	0	0	0	1	0	0	0	0	1
Salinimonas	1	1	0	0	0	0	0	0	0	0	0	0	0	0	0	0	0	0	0	0	0
Salinispora	3	127	1	0	0	1	0	0	1	0	0	0	0	1	0	1	1	0	0	1	1
Salinivibrio	1	2	0	0	0	0	0	0	0	0	0	0	0	0	0	0	0	0	0	0	0
Salmonella	2	151	1	0	0	1	0	0	1	0	0	0	0	0	1	1	0	0	0	1	1
Sanguibacter	2	73	0	0	0	1	0	1	0	1	0	0	0	0	0	1	0	0	0	0	0
Saprospira	1	13	0	0	0	0	0	0	0	0	0	0	0	0	0	0	0	0	0	0	0
Scardovia	1	1	0	0	0	0	0	0	0	0	0	0	0	0	0	0	0	0	0	0	0
Scytonema	1	10	0	0	0	0	0	0	1	0	0	0	0	0	0	0	0	0	0	0	0
Sebaldella	2	69	0	0	0	0	0	0	0	1	0	0	0	0	0	1	0	1	0	1	0
Segniliparus	2	85	1	0	0	0	0	1	1	1	0	0	0	0	0	1	0	0	0	0	1
Selenomonas	2	57	0	0	0	0	0	0	1	0	0	0	0	0	0	1	0	0	0	1	0
Serinicoccus	1	1	0	0	0	0	0	0	0	0	0	0	0	0	0	0	0	0	0	0	0
Serratia	2	186	1	0	0	1	0	0	1	1	0	0	0	0	1	1	0	0	0	1	1
Shewanella	3	214	0	1	0	1	0	1	1	1	0	0	0	1	0	1	1	0	0	1	1
Shigella	2	172	1	0	0	1	0	0	1	1	0	0	0	0	1	1	1	0	0	1	1
Shinella	1	2	0	0	0	0	0	0	0	0	0	0	0	0	0	0	0	0	0	0	0
Sideroxydans	2	81	0	0	0	0	0	0	1	1	0	0	0	0	0	1	0	0	0	1	1
Silicibacter	2	105	1	0	0	1	0	0	0	1	0	0	0	0	1	1	0	0	0	1	1
Simiduia	1	18	0	0	0	0	0	0	0	0	0	0	0	0	0	0	0	0	0	0	0
Simkania	1	6	0	0	0	0	0	0	0	0	0	0	0	0	0	0	0	0	0	0	0
Simonsiella	1	2	0	0	0	0	0	0	0	0	0	0	0	0	0	0	0	0	0	0	0
Singulisphaera	1	18	0	0	0	0	0	0	0	0	0	0	0	0	0	0	0	0	0	0	0
Sinorhizobium	3	207	1	1	0	1	0	1	1	1	0	0	0	0	1	1	0	0	0	1	1
Slackia	2	46	0	1	0	0	0	0	0	1	0	0	0	0	0	1	0	0	0	1	0
Smaragdicoccus	1	3	0	0	0	0	0	0	0	0	0	0	0	0	0	0	0	0	0	0	0
Sodalis	2	71	0	0	0	1	0	0	1	0	0	0	0	0	0	1	0	0	0	1	0
Solibacillus	1	23	0	0	0	0	0	0	0	0	0	0	0	0	0	0	0	0	0	0	0
Solitalea	1	13	0	0	0	0	0	0	0	0	0	0	0	0	0	0	0	0	0	0	0
Sorangium	3	115	1	0	0	1	0	1	0	1	0	0	0	0	0	1	0	0	0	1	1
Sphaerobacter	2	100	0	0	0	1	0	0	1	1	0	0	0	0	0	1	0	0	0	1	1
Sphaerochaeta	1	42	0	0	0	0	0	0	0	0	0	0	0	0	0	1	0	0	0	1	0
Sphingobacterium	3	85	0	0	0	1	0	1	1	1	0	0	0	0	0	1	0	0	0	1	1
Sphingobium	1	179	1	0	0	0	0	1	1	0	0	0	0	1	0	1	1	0	0	1	1
Sphingomonadaceae	1	15	0	0	0	0	0	0	0	0	0	0	0	0	0	0	0	0	0	0	0
Sphingomonas	2	220	1	1	0	0	0	0	1	1	0	1	0	1	1	1	1	0	0	1	1
Sphingopyxis	3	99	1	0	0	0	0	1	1	0	0	0	0	0	0	1	0	0	0	1	1
Spirochaeta	2	103	0	0	0	1	0	1	1	1	0	0	0	0	0	1	0	1	0	1	1
Spiroplasma	1	6	0	0	0	0	0	0	0	0	0	0	0	0	0	0	0	0	0	0	0
Spirosoma	2	107	1	0	0	1	0	0	0	0	0	0	0	0	1	1	0	1	0	1	1
Stackebrandtia	2	110	1	0	0	0	0	0	0	1	0	0	0	0	0	1	0	0	0	1	1
Stanieria	1	17	0	0	0	0	0	0	1	0	0	0	0	0	0	0	0	0	0	0	0
Staphylococcus	2	108	0	0	0	1	0	0	1	1	0	0	0	0	0	1	0	0	0	1	1
Staphylothermus	1	12	0	0	0	0	0	0	0	0	0	0	0	0	0	0	0	0	0	0	0

Genus	Cluster Number	Protein Counts	3.7.1.2	3.7.1.9	3.8.1.-	3.8.1.2	3.8.1.3	3.8.1.5	4.1.1.-	4.1.1.44	4.1.1.55	4.1.1.7	4.1.1.70	4.1.1.77	4.1.2.-	4.1.3.-	4.1.3.39	4.1.99.-	4.1.99.11	4.2.1.-	4.2.1.17
Stappia	1	2	0	0	0	0	0	0	0	0	0	0	0	0	0	0	0	0	0	0	0
Starkeya	3	134	0	0	0	0	1	0	1	1	0	0	0	0	1	1	0	0	0	1	1
Stenotrophomonas	3	104	0	1	0	1	0	1	0	1	0	0	0	0	0	1	0	0	0	1	1
Stigmatella	3	129	0	0	0	1	0	0	0	1	0	0	0	1	0	1	0	1	0	1	1
Streptobacillus	1	38	0	0	0	1	0	0	0	0	0	0	0	0	0	0	0	0	0	0	0
Streptococcus	2	121	0	0	0	1	0	1	1	1	0	0	0	0	0	1	0	0	0	1	1
Streptomyces	2	253	1	0	0	1	1	1	1	1	1	1	0	1	1	1	1	1	0	1	1
Streptosporangium	3	148	1	1	0	1	0	0	0	1	0	0	0	1	0	1	1	0	0	1	1
Strigomonas	1	2	0	0	0	0	0	0	0	0	0	0	0	0	0	0	0	0	0	0	0
Sulfitobacter	1	10	0	0	0	0	0	0	0	0	0	0	0	0	0	0	0	0	0	0	0
Sulfobacillus	1	30	0	0	0	0	0	0	0	0	0	0	0	0	0	0	0	0	0	0	0
Sulfolobus	1	23	0	0	0	0	0	0	0	0	0	0	0	0	0	0	0	0	0	0	0
Sulfuricurvum	2	67	0	0	0	0	0	0	1	0	0	0	0	0	0	1	0	1	0	1	0
Sulfurihydrogenibium	2	55	0	0	0	0	0	0	1	0	0	0	0	0	0	1	0	0	0	1	0
Sulfurimonas	2	60	0	0	0	0	0	0	1	0	0	0	0	0	0	1	0	1	0	1	0
Sulfurospirillum	1	48	0	0	0	0	0	0	0	0	0	0	0	0	0	1	0	1	0	1	0
Sulfurovum	2	48	0	0	0	0	0	0	0	1	0	0	0	0	0	1	1	0	0	1	0
Symbiobacterium	2	68	0	0	0	1	0	1	0	0	0	0	0	0	0	1	0	0	0	1	1
Synechococcus	1	34	0	0	0	0	0	0	0	0	0	0	0	0	0	0	0	0	0	1	0
Synechocystis	1	40	0	0	0	1	0	0	1	0	0	0	0	0	0	0	0	1	0	1	1
Synergistetes	1	27	0	0	0	0	0	1	0	1	0	0	0	0	0	0	0	0	0	1	0
Syntrophobacter	2	101	0	0	0	0	0	0	1	1	0	0	0	0	0	1	0	1	0	1	1
Syntrophobotulus	2	71	0	0	0	0	0	0	1	0	0	0	0	0	0	1	0	1	0	1	0
Syntrophomonas	2	58	0	0	0	0	0	0	1	1	0	0	0	0	0	1	0	1	0	1	1
Syntrophothermus	2	60	0	0	0	1	0	0	1	1	0	0	0	0	0	1	0	0	0	1	1
Syntrophus	2	66	0	0	0	0	0	0	0	1	0	0	0	0	0	1	0	0	0	1	1
Tannerella	1	8	0	0	0	0	0	0	0	0	0	0	0	0	0	0	0	0	0	0	0
Taylorella	1	43	0	0	0	0	0	1	1	0	0	0	0	0	0	0	0	0	0	1	0
Tepidanaerobacter	2	55	0	0	0	0	0	0	0	0	0	0	0	0	0	1	0	0	0	1	1
Teredinibacter	2	109	0	1	0	0	0	0	1	1	0	0	0	0	0	1	0	0	0	1	1
Terrabacter	1	1	0	0	0	0	0	0	0	0	0	0	0	0	0	0	0	0	0	0	0
Terriglobus	2	83	0	0	0	0	0	0	1	1	0	0	0	0	1	1	0	0	0	1	0
Tetragenococcus	1	12	0	0	0	0	0	0	0	0	0	0	0	0	0	0	0	0	0	0	0
Thalassiobium	1	11	0	0	0	0	0	0	0	0	0	0	0	0	0	0	0	0	0	0	0
Thalassobacter	1	9	1	0	0	0	0	0	0	0	0	0	0	0	0	0	0	0	0	0	0
Thalassolituus	1	12	0	0	0	0	0	0	0	0	0	0	0	0	0	0	0	0	0	0	0
Thalassospira	1	2	0	0	0	0	0	0	0	0	0	0	0	0	0	0	0	0	0	0	0
Thauera	1	111	0	1	0	0	0	0	1	0	0	0	0	1	0	1	1	0	0	1	1
Thermacetogenium	1	7	0	0	0	0	0	0	0	0	0	0	0	0	0	0	0	0	0	0	0
Thermaerobacter	2	79	1	0	0	0	0	0	0	0	0	0	0	1	0	1	0	0	0	1	1
Thermanaerovibrio	2	58	0	0	0	0	0	1	1	0	0	0	0	0	0	1	0	0	0	1	0
Thermincola	2	62	0	0	0	0	0	0	1	1	0	0	0	0	0	1	0	1	0	1	0
Thermoanaerobacter	2	76	0	0	0	0	0	0	0	1	0	0	0	0	0	1	0	1	0	1	0
Thermoanaerobacterium	2	64	0	0	0	0	0	0	1	1	0	0	0	0	0	1	0	1	0	1	0
Thermobacillus	1	11	0	0	0	0	0	0	0	0	0	0	0	0	0	0	0	0	0	0	0
Thermobaculum	2	62	0	0	0	1	0	1	0	0	0	0	0	0	0	1	0	0	0	1	1
Thermobifida	2	72	0	0	0	1	0	1	0	0	0	0	0	0	0	1	0	0	0	1	1
Thermobispora	2	109	1	0	0	0	0	0	0	1	0	1	0	0	0	1	0	0	0	1	1
Thermococcus	1	13	0	0	0	0	0	0	0	0	0	0	0	0	0	0	0	0	0	0	0
Thermocrinis	2	60	0	0	0	1	0	0	1	0	0	0	0	0	0	1	0	0	0	1	0
Thermodesulfatator	1	10	0	0	0	0	0	0	0	0	0	0	0	0	0	0	0	0	0	0	0
Thermodesulfobacterium	1	9	0	0	0	0	0	0	0	0	0	0	0	0	0	0	0	0	0	0	0

Genus	Cluster Number	Protein Counts	3.7.1.2	3.7.1.9	3.8.1.-	3.8.1.2	3.8.1.3	3.8.1.5	4.1.1.-	4.1.1.44	4.1.1.55	4.1.1.7	4.1.1.70	4.1.1.77	4.1.2.-	4.1.3.-	4.1.3.39	4.1.99.-	4.1.99.11	4.2.1.-	4.2.1.17
Thermodesulfobium	1	13	0	0	0	0	0	0	0	0	0	0	0	0	0	0	0	0	0	0	0
Thermodesulfovibrio	2	60	0	0	0	0	0	0	1	0	0	0	0	0	0	1	0	0	0	1	0
Thermofilum	1	9	0	0	0	0	0	0	0	0	0	0	0	0	0	0	0	0	0	0	0
Thermogladius	1	7	0	0	0	0	0	0	0	0	0	0	0	0	0	0	0	0	0	0	0
Thermomicrobium	2	92	0	1	0	1	0	0	0	0	0	0	0	1	1	1	0	0	0	1	1
Thermomonospora	2	125	1	1	0	1	0	0	0	1	0	0	0	1	0	1	1	0	0	1	1
Thermoplasma	1	11	0	0	0	0	0	0	0	0	0	0	0	0	0	0	0	0	0	0	0
Thermoplasmatales	1	6	0	0	0	0	0	0	0	0	0	0	0	0	0	0	0	0	0	0	0
Thermoproteus	1	12	0	0	0	0	0	0	0	0	0	0	0	0	0	0	0	0	0	0	0
Thermosediminibacter	2	64	0	0	0	0	0	0	1	0	0	0	0	1	0	1	0	1	0	1	0
Thermosipho	2	70	0	0	0	1	0	0	0	0	0	0	0	0	0	0	0	0	0	1	0
Thermosphaera	1	10	0	0	0	0	0	0	0	0	0	0	0	0	0	0	0	0	0	0	0
Thermosynechococcus	1	6	0	0	0	0	0	0	0	0	0	0	0	0	0	0	0	0	0	0	0
Thermotoga	2	80	0	0	0	1	0	1	0	1	0	0	0	0	0	1	0	0	0	1	0
Thermovibrio	2	58	0	0	0	0	0	0	1	0	0	0	0	0	0	1	0	0	0	1	0
Thermovirga	1	9	0	0	0	0	0	0	0	0	0	0	0	0	0	0	0	0	0	0	0
Thermus	2	107	0	1	0	1	0	0	1	1	0	0	0	1	0	1	1	0	0	1	1
Thioalkalimicrobium	1	4	0	0	0	0	0	0	0	0	0	0	0	0	0	0	0	0	0	0	0
Thioalkalivibrio	2	91	1	0	0	1	0	0	1	1	0	0	0	0	0	1	0	0	0	1	0
Thiobaca	1	2	0	0	0	0	0	0	0	0	0	0	0	0	0	0	0	0	0	0	0
Thiobacillus	2	67	0	0	0	0	0	0	1	0	0	0	0	0	0	1	0	0	0	1	1
Thiocapsa	1	2	0	0	0	0	0	0	0	0	0	0	0	0	0	0	0	0	0	0	0
Thiocystis	1	11	0	0	0	0	0	0	0	0	0	0	0	0	0	0	0	0	0	0	0
Thioflavicoccus	1	13	0	0	0	0	0	0	0	0	0	0	0	0	0	0	0	0	0	0	0
Thiomicrospira	2	50	0	0	0	0	0	0	0	0	0	0	0	0	0	1	0	0	0	1	0
Thiomonas	2	85	0	0	0	0	0	0	1	1	0	0	0	0	0	1	0	0	0	1	1
Thiorhodococcus	1	2	0	0	0	0	0	0	0	0	0	0	0	0	0	0	0	0	0	0	0
Thiothrix	1	2	0	0	0	0	0	0	0	0	0	0	0	0	0	0	0	0	0	0	0
Tistrella	1	42	0	0	0	0	0	0	0	0	0	0	0	0	0	0	0	0	0	0	0
Tolumonas	2	100	0	0	0	1	0	0	1	1	0	0	0	0	0	1	0	0	0	1	0
Transposon	1	2	0	0	0	0	0	0	0	0	0	0	0	0	0	0	0	0	0	0	0
Treponema	1	63	0	0	0	0	0	0	0	1	0	0	0	0	0	1	0	0	0	1	0
Trichodesmium	2	71	0	0	0	0	0	1	1	1	0	0	0	0	0	1	0	0	0	1	0
Trichormus	1	12	0	0	0	0	0	0	1	0	0	0	0	0	0	0	0	0	0	0	0
Tropheryma	2	22	0	0	0	0	0	0	0	0	0	0	0	0	0	0	0	0	0	0	0
Truepera	2	104	0	0	0	1	0	0	1	1	0	0	0	0	1	1	0	1	0	1	1
Tsukamurella	2	123	0	0	0	1	0	0	0	0	0	0	0	0	0	1	1	0	0	1	1
Turneriella	1	16	0	0	0	0	0	0	0	0	0	0	0	0	0	0	0	0	0	0	0
Uliginosibacterium	1	2	0	0	0	0	0	0	0	0	0	0	0	0	0	0	0	0	0	0	0
Ureaplasma	1	24	0	0	0	0	0	0	0	0	0	0	0	0	0	0	0	0	0	0	0
Ureibacillus	1	2	0	0	0	0	0	0	0	0	0	0	0	0	0	0	0	0	0	0	0
Variovorax	3	187	1	1	0	1	0	1	0	1	0	0	0	0	1	1	0	0	0	1	1
Veillonella	1	54	0	0	0	0	0	0	0	1	0	0	0	0	0	1	0	0	0	1	0
Verminephrobacter	3	188	1	1	0	1	0	1	0	1	1	0	0	1	1	1	1	0	0	1	1
Verrucosispora	3	126	1	0	0	1	0	0	1	1	0	1	0	1	0	1	1	0	0	1	1
Vibrio	2	195	1	0	0	1	0	1	1	1	0	0	0	0	1	1	0	0	0	1	1
Vibrionales	1	46	1	0	0	0	0	0	0	0	0	0	0	0	0	1	0	0	0	1	0
Vitreoscilla	1	1	0	0	0	0	0	0	0	0	0	0	0	0	0	0	0	0	0	0	1
Vulcanisaeta	1	14	0	0	0	0	0	0	0	0	0	0	0	0	0	0	0	0	0	0	0
Waddlia	2	68	0	0	0	0	0	0	1	0	0	0	0	0	0	0	0	1	0	1	1
Wautersia	1	2	0	0	0	0	0	0	0	0	0	0	0	0	0	0	0	0	0	0	0
Weeksella	2	65	1	0	0	0	0	0	0	0	0	0	0	0	0	0	0	0	0	1	1

Genus	Cluster Number	Protein Counts	3.7.1.2	3.7.1.9	3.8.1.-	3.8.1.2	3.8.1.3	3.8.1.5	4.1.1.-	4.1.1.44	4.1.1.55	4.1.1.7	4.1.1.70	4.1.1.77	4.1.2.-	4.1.3.-	4.1.3.39	4.1.99.-	4.1.99.11	4.2.1.-	4.2.1.17
**Weissella**	1	14	0	0	0	0	0	0	1	0	0	0	0	0	0	0	0	0	0	0	0
**Wenxinia**	1	2	0	0	0	0	0	0	0	0	0	0	0	0	0	0	0	0	0	0	0
**Wigglesworthia**	1	29	0	0	0	1	0	0	1	0	0	0	0	0	0	0	0	0	0	0	0
**Wolbachia**	1	32	0	0	0	0	0	0	1	0	0	0	0	0	0	0	0	0	0	1	0
**Wolinella**	2	51	0	0	0	0	0	0	0	0	0	0	0	0	0	1	0	0	0	1	0
**Xanthobacter**	3	176	1	0	0	1	0	1	0	1	0	0	0	0	0	1	0	0	0	1	1
**Xanthobacteraceae**	1	4	0	0	0	0	0	0	0	0	0	0	0	0	0	0	0	0	0	0	0
**Xanthomonadaceae**	1	39	0	0	0	0	0	0	0	1	0	0	0	0	0	0	0	0	0	1	1
**Xanthomonas**	2	162	0	1	0	1	0	1	0	1	0	0	0	0	1	1	0	0	0	1	1
**Xenorhabdus**	2	127	0	0	0	1	0	0	1	0	0	0	0	0	1	1	0	0	0	1	1
**Xylanimonas**	2	66	0	0	0	0	0	0	0	0	0	1	0	0	0	1	0	0	0	0	0
**Xylella**	2	52	0	0	0	0	0	0	0	0	0	0	0	0	0	1	0	0	0	1	0
**Yersinia**	2	145	0	0	0	1	0	0	1	1	0	0	0	0	1	1	0	0	0	1	1
**Yokenella**	2	61	0	0	0	0	0	0	1	0	0	0	0	0	0	1	0	0	0	1	0
**Zobellia**	1	28	0	0	0	0	0	0	0	0	0	0	0	0	0	1	0	0	0	0	1
**Zunongwangia**	2	98	1	0	0	1	0	1	0	1	0	0	0	0	0	1	0	0	0	1	1
**Zymomonas**	2	67	0	0	0	0	0	0	1	1	0	0	0	0	0	1	0	0	0	1	0
**Zymophilus**	1	2	0	0	0	0	0	0	1	0	0	0	0	0	0	0	0	0	0	0	0

Genus	Cluster Number	Protein Counts	4.2.1.80	4.2.1.83	4.2.1.84	4.99.1.7	5.1.2.2	5.2.1.2	5.3.3.1	5.3.3.4	5.3.99.-	5.4.99.-	5.5.1.1	5.5.1.2	6.2.1.-	6.2.1.25	6.2.1.32	6.3.5.2	K00002	K00055
Acaricomes	1	2	0	0	0	0	0	0	0	0	0	0	0	0	0	0	0	0	0	0
Acaryochloris	2	88	0	0	1	0	0	1	0	0	0	0	0	0	0	0	0	1	0	0
Acetivibrio	1	2	0	0	0	0	0	0	0	0	0	0	0	0	0	0	0	0	0	0
Acetobacter	2	85	0	0	0	0	0	0	0	0	0	0	0	0	0	0	0	1	0	0
Acetobacteraceae	1	2	0	0	0	0	0	0	0	0	0	0	0	0	0	0	0	0	0	0
Acetobacterium	1	8	0	0	0	0	0	0	0	0	0	0	0	0	0	0	0	0	0	0
Acetohalobium	2	79	0	0	0	0	0	0	0	0	0	0	0	0	0	0	0	1	0	0
Acholeplasma	2	38	0	0	0	0	0	0	0	0	0	0	0	0	0	0	0	0	0	0
Achromobacter	2	192	0	0	0	0	1	1	0	1	1	0	1	0	0	1	0	1	0	0
Acidaminococcus	1	65	0	0	0	0	0	0	0	0	0	0	0	0	0	0	0	1	0	0
Acidianus	1	9	0	0	0	0	0	0	0	0	0	0	0	0	0	0	0	0	0	0
Acidilobus	1	8	0	0	0	0	0	0	0	0	0	0	0	0	0	0	0	0	0	0
Acidimicrobium	2	58	0	0	0	0	0	0	0	0	0	0	0	0	0	0	0	1	0	0
Acidiphilium	3	144	0	1	1	0	0	0	0	0	1	1	0	1	0	0	0	1	0	0
Acidithiobacillus	2	70	0	0	0	0	0	0	0	0	0	0	0	0	0	0	0	1	0	0
Acidobacterium	2	91	0	0	0	0	1	0	0	0	1	0	0	0	0	0	0	1	0	0
Acidocella	1	2	0	0	0	0	0	0	0	0	0	0	0	0	0	0	0	0	0	0
Acidothermus	2	68	0	0	0	0	0	0	0	0	1	0	0	0	0	0	0	1	0	0
Acidovorax	3	217	1	0	1	0	1	1	0	1	0	0	1	0	0	0	0	1	0	0
Aciduliprofundum	1	11	0	0	0	0	0	0	0	0	0	0	0	0	0	0	0	0	0	0
Acinetobacter	3	188	0	0	1	0	0	1	0	1	1	0	1	1	0	0	0	1	0	1
Actinobacillus	2	112	0	0	0	0	0	0	0	1	1	0	0	0	0	0	0	1	0	0
Actinomadura	1	1	0	0	0	0	0	0	0	0	0	0	0	0	0	0	0	0	0	0
Actinoplanes	1	52	0	0	0	0	0	0	0	0	0	0	0	0	0	0	0	0	0	1
Actinopolyspora	1	2	0	0	0	0	0	0	0	0	0	0	0	0	0	0	0	0	0	0
Actinosynnema	2	122	0	0	0	1	0	0	0	0	1	0	0	1	0	0	0	1	0	1
Advenella	1	30	0	0	0	0	0	0	0	0	0	0	0	0	0	0	0	0	0	0
Aequorivita	1	12	0	0	0	0	0	0	0	0	0	0	0	0	0	0	0	0	0	0
Aerococcus	2	57	0	0	0	0	0	0	0	0	0	0	0	0	0	0	0	1	0	0
Aeromonas	2	127	0	0	0	0	0	1	0	0	0	0	0	0	0	0	0	1	0	0
Aeropyrum	1	13	0	0	0	0	0	0	0	0	0	0	0	0	0	0	0	0	0	0
Afipia	1	54	0	0	0	0	0	0	0	1	0	0	0	0	0	0	0	1	0	0
Aggregatibacter	1	81	0	0	0	0	0	0	0	0	0	0	0	0	0	0	0	1	0	0
Agrobacterium	2	242	0	0	1	0	0	1	0	1	1	0	1	1	0	1	0	1	1	1
Ahrensia	1	2	0	0	0	0	0	0	0	0	0	0	0	0	0	0	0	0	0	0
Akkermansia	2	47	0	0	0	0	0	0	0	0	0	0	0	0	0	0	0	1	0	0
Albidiferax	3	144	0	1	0	0	0	0	0	0	0	0	0	1	0	1	0	1	0	0
Alcaligenes	1	21	0	0	0	0	0	0	0	0	0	0	1	0	0	0	0	0	0	0
Alcanivorax	2	146	0	0	0	0	0	0	0	0	0	0	0	0	1	0	0	1	1	0
Alicycliphilus	3	152	0	0	0	0	0	1	0	1	0	0	1	0	1	0	0	1	0	0
Alicyclobacillus	2	91	0	0	0	0	0	0	0	0	1	0	0	0	0	0	0	1	0	0
Aliivibrio	2	118	1	0	0	0	0	1	0	0	0	0	0	0	0	0	0	1	0	0
Alishewanella	1	1	0	0	0	0	0	0	0	0	0	0	0	0	0	0	0	0	0	0
Alistipes	1	52	0	0	0	0	0	0	0	0	0	0	0	0	0	0	0	1	0	0
Alkalilimnicola	2	97	0	0	0	0	0	0	0	0	0	0	0	0	0	0	0	1	0	0
Alkaliphilus	2	62	0	0	0	0	0	0	0	0	0	0	0	0	0	0	0	1	0	0
Allochromatium	2	84	0	0	0	0	0	0	0	0	0	0	0	0	0	0	0	1	0	0
Alphaproteobacteria	1	3	0	0	0	0	0	0	0	0	0	0	0	0	0	0	0	0	0	0
Alteromonadales	1	12	0	0	0	0	0	0	0	0	0	0	0	0	0	0	0	0	0	0
Alteromonas	1	37	0	0	0	0	0	0	0	0	0	0	0	0	0	0	0	0	0	0
Aminobacter	1	4	0	0	0	0	0	0	0	0	0	0	0	0	0	0	0	0	0	0
Aminobacterium	2	59	0	0	0	0	0	0	0	0	0	0	0	0	0	0	0	1	0	0

Genus	Cluster Number	Protein Counts	4.2.1.80	4.2.1.83	4.2.1.84	4.99.1.7	5.1.2.2	5.2.1.2	5.3.3.1	5.3.3.4	5.3.99.-	5.4.99.-	5.5.1.1	5.5.1.2	6.2.1.-	6.2.1.25	6.2.1.32	6.3.5.2	K00002	K00055
Ammonifex	2	50	0	0	0	0	0	0	0	0	0	0	0	0	0	0	0	1	0	0
Amphibacillus	1	13	0	0	0	0	0	0	0	0	0	0	0	0	0	0	0	0	0	0
Amphritea	1	2	0	0	0	0	0	0	0	0	0	0	0	0	0	0	0	0	0	0
Amycolatopsis	2	201	0	0	0	0	1	0	0	0	1	0	1	1	0	0	0	1	0	1
Amycolicicoccus	1	43	0	0	1	0	0	0	0	0	0	0	0	0	0	0	0	0	0	1
Anabaena	2	92	0	0	0	0	0	0	0	0	0	0	0	0	0	0	0	1	0	0
Anaerobaculum	1	11	0	0	0	0	0	0	0	0	0	0	0	0	0	0	0	0	0	0
Anaerococcus	2	46	0	0	0	0	0	0	0	0	0	0	0	0	0	0	0	1	0	0
Anaerolinea	2	66	0	0	0	0	0	0	0	0	1	0	0	0	0	0	0	1	0	0
Anaeromyxobacter	3	117	0	0	0	0	0	0	0	0	0	0	0	0	0	0	0	1	0	0
Anaerostipes	1	44	0	0	0	0	0	0	0	0	1	0	0	0	0	0	0	0	0	0
Anaerotruncus	1	2	0	0	0	0	0	0	0	0	0	0	0	0	0	0	0	0	0	0
Anaplasma	1	35	0	0	0	0	0	0	0	0	0	0	0	0	0	0	0	1	0	0
Ancylobacter	1	3	0	0	0	0	0	0	0	0	0	0	0	0	0	0	0	0	0	0
Aneurinibacillus	1	1	0	0	0	0	0	0	0	0	0	0	0	0	0	0	0	0	0	0
Angomonas	1	1	0	0	0	0	0	0	0	0	0	0	0	0	0	0	0	0	0	0
Anoxybacillus	2	72	0	0	0	0	0	0	0	0	0	0	0	0	0	0	0	1	0	0
Aquifex	2	45	0	0	0	0	0	0	0	0	0	0	0	0	0	0	0	1	0	0
Aquimarina	1	2	0	0	0	0	0	0	0	0	0	0	0	0	0	0	0	0	0	0
Arcanobacterium	2	45	0	0	0	0	0	0	0	0	0	0	0	0	0	0	0	1	0	0
Archaeoglobus	1	14	0	0	0	0	0	0	0	0	0	0	0	0	0	0	0	0	0	0
Arcobacter	2	101	1	0	0	0	0	0	0	0	0	0	0	0	0	0	0	1	1	0
Aromatoleum	3	133	0	0	0	0	1	0	0	0	0	0	0	0	0	1	0	1	0	1
Arsenophonus	1	4	0	0	0	0	0	0	0	0	0	0	0	0	0	0	0	0	0	0
Arthrobacter	2	206	0	1	1	0	0	0	0	1	1	0	1	1	0	0	0	1	0	1
Arthrospira	2	67	0	0	0	0	0	0	0	0	0	0	0	0	0	0	0	1	0	0
Aster	1	10	0	0	0	0	0	0	0	0	0	0	0	0	0	0	0	0	0	0
Asticcacaulis	2	103	0	1	0	0	0	0	0	0	0	0	1	0	0	0	0	1	0	0
Atopobium	1	28	0	0	0	0	0	0	0	0	0	0	0	0	0	0	0	1	0	0
Aurantimonas	1	2	0	0	0	0	0	0	0	0	0	0	0	0	0	0	0	0	0	0
Avibacterium	1	17	0	0	0	0	0	0	0	0	0	0	0	0	0	0	0	1	0	0
Azoarcus	3	172	1	1	0	0	0	1	0	0	0	0	0	0	0	1	0	1	0	0
Azorhizobium	3	149	1	0	1	0	0	0	0	1	0	0	1	1	0	0	0	1	0	0
Azorhizophilus	1	4	0	0	0	0	0	0	0	0	0	0	0	0	0	0	0	0	0	0
Azospira	1	19	0	0	0	0	0	0	0	0	0	0	0	0	0	0	0	0	0	0
Azospirillum	2	198	0	0	0	0	0	0	0	1	0	0	1	1	0	0	0	1	0	1
Azotobacter	3	183	1	0	0	0	0	0	0	0	1	0	0	1	0	0	0	1	0	0
Bacillales	1	9	0	0	0	0	0	0	0	0	0	0	0	0	0	0	0	0	0	0
Bacilli	1	3	0	0	0	0	0	0	0	0	0	0	0	0	0	0	0	0	0	0
Bacillus	2	237	0	0	1	0	0	0	0	0	1	1	1	0	1	0	0	1	1	1
Bacteria	2	86	0	0	0	0	0	1	0	1	1	0	0	0	0	0	0	0	0	0
Bacteriovorax	2	98	0	0	0	0	0	0	0	0	0	0	0	0	0	0	0	1	0	0
Bacteroidales	2	59	0	0	0	0	0	0	0	0	1	0	0	0	0	0	0	1	0	0
Bacteroides	2	96	0	0	0	0	0	0	0	0	1	0	0	0	0	0	0	1	0	0
Bacteroidetes	1	2	0	0	0	0	0	0	0	0	0	0	0	0	0	0	0	0	0	0
Bartonella	2	50	0	0	0	0	0	0	0	0	0	0	0	0	0	0	0	1	0	0
Baumannia	2	24	0	0	0	0	0	0	0	0	0	0	0	0	0	0	0	1	0	0
Bdellovibrio	2	87	0	0	0	0	0	0	0	0	0	0	0	0	0	0	0	1	0	0
Beijerinckia	3	123	0	0	0	0	0	1	0	0	1	0	1	0	0	0	0	1	0	0
Belliella	1	14	0	0	0	0	0	0	0	0	0	0	0	0	0	0	0	0	0	0
Beutenbergia	2	82	0	0	0	0	0	0	0	0	1	0	0	0	0	0	0	1	0	0
Bifidobacterium	2	61	0	0	0	0	0	0	0	0	0	0	0	0	0	0	0	1	0	0

Genus	Cluster Number	Protein Counts	4.2.1.80	4.2.1.83	4.2.1.84	4.99.1.7	5.1.2.2	5.2.1.2	5.3.3.1	5.3.3.4	5.3.99.-	5.4.99.-	5.5.1.1	5.5.1.2	6.2.1.-	6.2.1.25	6.2.1.32	6.3.5.2	K00002	K00055
Bizionia	1	1	0	0	0	0	0	0	0	0	0	0	0	0	0	0	0	0	0	0
Blastococcus	1	39	0	0	0	0	0	0	0	0	0	0	0	0	0	0	0	0	0	0
Blattabacterium	1	23	0	0	0	0	0	0	0	0	0	0	0	0	0	0	0	1	0	0
Blautia	1	22	0	0	0	0	0	0	0	0	1	0	0	0	0	0	0	0	0	0
Bordetella	3	227	1	0	1	0	1	1	0	1	0	0	1	1	0	1	0	1	0	1
Borrelia	1	32	0	0	0	0	0	0	0	0	0	0	0	0	0	0	0	1	0	0
Brachybacterium	1	18	0	0	0	0	0	0	0	0	0	0	0	0	0	0	0	0	0	0
Brachymonas	1	4	0	0	0	0	0	0	0	0	0	0	0	0	0	0	0	1	0	0
Brachyspira	2	79	0	0	0	0	0	0	0	0	0	1	0	0	0	0	0	1	0	0
Bradyrhizobiaceae	1	20	0	0	0	0	0	0	0	0	0	0	0	0	0	0	0	1	0	0
Bradyrhizobium	3	254	1	1	1	0	1	1	0	1	1	0	0	0	0	1	0	1	0	1
Brenneria	1	20	0	0	0	0	0	0	0	0	0	0	0	0	0	0	0	1	0	0
Brevibacillus	2	122	0	0	0	0	0	0	0	0	1	0	1	0	0	0	0	1	0	0
Brevibacterium	1	4	0	0	1	0	0	0	0	0	0	0	0	0	0	0	0	0	0	0
Brevundimonas	2	105	0	1	0	0	0	0	0	0	0	0	0	0	0	0	0	1	0	0
Brucella	3	138	0	0	0	0	0	1	0	0	1	0	1	1	0	0	0	1	0	0
Buchnera	1	26	0	0	0	0	0	0	0	0	0	0	0	0	0	0	0	0	0	0
Burkholderia	3	424	1	1	1	0	1	1	0	1	1	0	1	1	1	1	1	1	0	1
Burkholderiaceae	3	143	0	0	0	0	0	1	0	0	0	0	1	0	0	0	0	1	0	0
Burkholderiales	1	24	0	0	0	0	0	0	0	0	0	0	0	0	0	0	0	0	0	0
Buttiauxella	1	2	0	0	1	0	0	0	0	0	0	0	0	0	0	0	0	0	0	0
Butyrivibrio	2	67	0	1	1	0	0	0	0	0	0	0	0	0	0	0	0	1	0	0
Caldanaerobacter	2	63	0	0	0	0	0	0	0	0	1	0	0	0	0	0	0	1	0	0
Caldicellulosiruptor	2	75	0	0	0	0	0	0	0	0	1	0	0	0	0	0	0	1	0	0
Caldilinea	1	23	0	0	0	0	0	0	0	0	0	0	0	0	0	0	0	0	0	0
Caldimonas	1	6	0	0	0	0	0	0	0	0	0	0	0	0	0	0	0	0	0	0
Caldisericum	1	10	0	0	0	0	0	0	0	0	0	0	0	0	0	0	0	0	0	0
Caldisphaera	1	7	0	0	0	0	0	0	0	0	0	0	0	0	0	0	0	0	0	0
Calditerrivibrio	2	74	0	0	0	0	0	0	0	0	0	0	0	0	0	0	0	1	0	0
Caldivirga	1	13	0	0	0	0	0	0	0	0	0	0	0	0	0	0	0	0	0	0
Calothrix	1	43	0	0	0	0	0	0	0	0	0	0	0	0	0	0	0	1	0	0
Calyptogena	2	34	0	0	0	0	0	0	0	0	0	0	0	0	0	0	0	1	0	0
Campylobacter	2	80	0	0	0	0	0	0	0	0	0	0	0	0	0	0	0	1	0	0
Candidatus	2	290	0	1	1	0	1	1	0	0	1	0	1	1	0	0	0	1	0	1
Capnocytophaga	1	50	0	0	0	0	0	0	0	0	0	0	0	0	0	0	0	1	0	0
Carbophilus	1	2	0	0	0	0	0	0	0	0	0	0	0	0	0	0	0	0	0	0
Carboxydibrachium	1	14	0	0	0	0	0	0	0	0	0	0	0	0	0	0	0	1	0	0
Carboxydothermus	2	68	1	0	0	0	0	0	0	0	0	0	0	0	0	0	0	1	0	0
Carica	1	2	0	0	0	0	0	0	0	0	0	0	0	0	0	0	0	0	0	0
Carnobacterium	2	71	0	0	0	0	0	0	0	0	0	0	0	0	0	0	0	1	0	0
Catenibacterium	1	2	0	0	0	0	0	0	0	0	0	0	0	0	0	0	0	0	0	0
Catenovulum	1	2	0	0	0	0	0	0	0	0	0	0	0	0	0	0	0	0	0	0
Catenulispora	3	159	0	1	0	0	0	0	0	1	1	0	0	1	0	0	0	1	0	1
Caulobacter	3	168	1	0	0	0	0	1	0	0	1	0	1	1	1	0	0	1	0	1
Cedecea	1	45	0	0	0	0	0	0	0	0	0	0	0	0	0	0	0	1	0	0
Cellulomonas	2	96	0	0	0	0	0	0	0	0	1	0	0	0	0	0	0	1	0	0
Cellulophaga	2	105	0	0	0	0	0	0	0	1	1	0	1	0	0	0	0	1	0	0
Cellulosilyticum	1	14	0	0	0	0	0	0	0	0	0	0	0	0	0	0	0	0	0	0
Cellvibrio	2	85	0	0	0	0	0	1	0	0	1	0	0	0	0	0	0	1	0	0
Cenarchaeum	1	7	0	0	0	0	0	0	0	0	0	0	0	0	0	0	0	0	0	0
Chamaesiphon	1	13	0	0	0	0	0	0	0	0	0	0	0	0	0	0	0	0	0	0
Chelativorans	3	136	1	0	0	0	0	0	0	0	1	0	1	1	0	0	0	1	0	0

Genus	Cluster Number	Protein Counts	4.2.1.80	4.2.1.83	4.2.1.84	4.99.1.7	5.1.2.2	5.2.1.2	5.3.3.1	5.3.3.4	5.3.99.-	5.4.99.-	5.5.1.1	5.5.1.2	6.2.1.-	6.2.1.25	6.2.1.32	6.3.5.2	K00002	K00055
Chelatococcus	1	2	0	0	0	0	0	0	0	0	0	0	0	0	0	0	0	0	0	0
Chitinophaga	2	98	0	0	0	0	0	0	0	0	1	0	0	0	0	0	0	1	0	0
Chlamydia	1	36	0	0	0	0	0	0	0	0	0	0	0	0	0	0	0	1	0	0
Chlamydomonas	1	3	0	0	0	0	0	0	0	0	0	0	0	0	0	0	0	0	0	0
Chlamydophila	1	31	0	0	0	0	0	0	0	0	0	0	0	0	0	0	0	1	0	0
Chlorobaculum	2	61	0	0	0	0	0	0	0	0	0	0	0	0	0	0	0	1	0	0
Chlorobium	2	89	0	0	0	0	0	0	0	0	0	0	0	0	0	0	0	1	0	0
Chlorobium/Pelodictyon	1	2	0	0	0	0	0	0	0	0	0	0	0	0	0	0	0	0	0	0
Chloroflexus	2	105	0	0	0	0	0	0	0	0	0	0	0	0	0	0	0	1	0	0
Chlorogloeopsis	1	9	0	0	0	0	0	0	0	0	0	0	0	0	0	0	0	0	0	0
Chloroherpeton	2	59	0	0	0	0	0	0	0	0	0	0	0	0	0	0	0	1	0	0
Chromobacterium	2	117	0	0	0	0	0	1	0	0	0	0	0	0	0	0	0	1	0	0
Chromohalobacter	2	145	0	0	0	0	1	0	0	0	1	0	0	1	0	0	0	1	0	1
Chroococcidiopsis	1	24	0	0	0	0	0	0	0	0	0	0	0	0	0	0	0	0	0	0
Chryseobacterium	2	104	1	1	0	0	1	1	0	0	0	0	1	1	0	1	0	1	0	0
Citreicella	1	13	0	0	0	0	0	0	0	0	0	0	0	0	0	0	0	0	0	0
Citrobacter	2	190	1	0	0	0	0	1	0	0	1	0	0	1	0	0	0	1	0	0
Clavibacter	2	83	0	0	0	0	0	0	0	0	1	0	0	0	0	0	0	1	0	0
Clonorchis	1	1	0	0	0	0	0	0	0	0	0	0	0	0	0	0	0	0	0	0
Clostridiales	2	77	0	0	0	0	0	0	0	0	1	0	0	0	0	0	0	1	0	0
Clostridium	2	191	0	0	0	0	0	0	0	0	1	0	1	0	0	0	0	1	1	1
Cohnella	1	2	0	0	0	0	0	0	0	0	0	0	0	0	0	0	0	0	0	0
Coleofasciculus	1	4	0	0	0	0	0	0	0	0	0	0	0	0	0	0	0	0	0	0
Collimonas	1	35	0	0	0	0	0	0	0	0	0	0	0	0	0	0	0	1	0	0
Colwellia	2	130	1	0	0	0	0	1	0	0	0	0	0	0	0	0	0	1	0	0
Comamonas	2	196	0	0	1	0	1	1	1	1	1	0	1	0	0	1	0	1	0	0
Conexibacter	3	117	0	0	0	0	0	0	0	1	0	0	1	0	0	0	0	1	0	0
Coprobacillus	1	2	0	0	0	0	0	0	0	0	0	0	0	0	0	0	0	0	0	0
Coprococcus	2	76	0	0	0	0	0	0	0	0	0	0	1	0	0	0	0	1	0	0
Coprothermobacter	2	45	0	0	0	0	0	0	0	0	0	0	0	0	0	0	0	1	0	0
Coraliomargarita	2	68	0	0	0	0	0	0	0	1	1	0	0	0	0	0	0	1	0	0
Corallococcus	1	29	0	0	0	0	0	0	0	0	0	0	0	0	0	0	0	1	0	1
Coriobacterium	2	36	0	0	0	0	0	0	0	0	1	0	0	0	0	0	0	1	0	0
Corynebacterineae	1	5	0	0	0	0	0	0	0	0	0	0	0	0	0	0	0	0	0	0
Corynebacterium	2	175	0	0	0	0	0	0	0	1	0	0	1	1	0	0	0	1	1	1
Coxiella	2	54	0	0	0	0	0	0	0	0	0	0	0	0	0	0	0	1	0	0
Crinalium	1	21	0	0	0	0	0	0	0	0	0	0	0	0	0	0	0	0	0	0
Croceibacter	2	80	0	0	0	0	0	0	0	0	0	0	0	0	0	0	0	1	1	0
Crocosphaera	1	6	0	0	0	0	0	0	0	0	0	0	0	0	0	0	0	0	0	0
Cronobacter	2	120	0	0	0	0	0	0	0	0	1	0	0	0	0	0	0	1	0	0
Cryptobacterium	2	39	0	0	0	0	0	0	0	0	0	0	0	0	0	0	0	1	0	0
Cucumis	1	9	0	0	0	0	0	0	0	0	0	0	0	0	0	0	0	0	0	0
Cupriavidus	3	317	1	1	0	0	1	1	0	1	0	0	1	1	1	1	0	1	0	1
Curtobacterium	1	5	0	0	1	0	0	0	0	0	0	0	0	0	0	0	0	1	0	0
Curvibacter	1	27	0	1	0	0	0	0	0	0	0	0	0	1	0	0	0	1	0	0
Cyanobacterium	1	9	0	0	0	0	0	0	0	0	0	0	0	0	0	0	0	0	0	0
Cyanobium	1	10	0	0	0	0	0	0	0	0	0	0	0	0	0	0	0	0	0	0
Cyanothece	1	34	0	0	0	0	0	0	0	0	0	0	0	0	0	0	0	0	1	0
Cyclobacterium	1	19	0	0	0	0	0	0	0	0	0	0	0	0	0	0	0	0	1	0
Cycloclasticus	1	21	0	0	0	0	0	0	0	0	0	0	0	0	0	0	0	0	0	1
Cylindrospermopsis	1	4	0	0	0	0	0	0	0	0	0	0	0	0	0	0	0	0	0	0
Cylindrospermum	1	30	0	0	0	0	0	0	0	0	0	0	0	0	0	0	0	1	0	0

Genus	Cluster Number	Protein Counts	4.2.1.80	4.2.1.83	4.2.1.84	4.99.1.7	5.1.2.2	5.2.1.2	5.3.3.1	5.3.3.4	5.3.99.-	5.4.99.-	5.5.1.1	5.5.1.2	6.2.1.-	6.2.1.25	6.2.1.32	6.3.5.2	K00002	K00055
Cytophaga	2	69	0	0	0	0	0	0	0	0	1	0	1	0	0	0	0	1	0	0
Dactylococcopsis	1	8	0	0	0	0	0	0	0	0	0	0	0	0	0	0	0	0	0	0
Dechloromonas	1	40	0	0	0	0	0	0	0	0	0	0	0	0	0	0	0	0	0	0
Dechlorosoma	1	19	0	0	0	0	0	0	0	0	0	0	0	0	0	0	0	0	0	0
Deferribacter	2	73	0	0	0	0	0	0	0	0	0	0	0	0	0	0	0	1	0	0
Dehalobacter	1	9	0	0	0	0	0	0	0	0	0	0	0	0	0	0	0	0	0	0
Dehalococcoides	2	41	0	0	0	0	0	0	0	0	0	0	0	0	0	0	0	1	0	0
Dehalogenimonas	1	37	0	0	0	0	0	0	0	0	0	0	0	0	0	0	0	1	0	0
Deinococcus	2	140	0	0	0	0	0	0	0	0	1	0	1	1	0	0	0	1	0	0
Delftia	3	177	1	1	0	0	1	1	0	1	1	0	1	1	0	1	0	1	0	0
Denitrovibrio	2	67	0	0	0	0	0	0	0	0	0	0	0	0	1	0	0	1	0	0
Desulfarculus	2	92	0	0	0	0	0	0	0	0	0	0	0	0	1	0	0	1	0	0
Desulfatibacillum	3	84	0	0	0	0	0	0	0	0	0	0	0	0	1	0	0	1	0	0
Desulfitobacterium	2	102	0	0	0	0	0	0	0	0	0	0	0	0	0	0	0	1	0	0
Desulfobacca	2	65	0	0	0	0	0	0	0	0	0	0	0	0	0	0	0	1	0	0
Desulfobacterium	2	113	0	0	0	0	0	0	0	0	0	0	1	0	1	0	0	1	0	0
Desulfobacula	1	21	0	0	0	0	0	0	0	0	0	0	0	0	0	0	0	0	0	0
Desulfobulbus	2	88	0	0	0	0	0	0	0	0	0	0	0	0	1	0	0	1	1	0
Desulfocapsa	1	9	0	0	0	0	0	0	0	0	0	0	0	0	0	0	0	0	0	0
Desulfococcus	2	80	0	0	0	0	0	0	0	0	0	0	0	0	0	0	0	1	0	0
Desulfohalobium	2	52	0	0	0	0	0	0	0	0	0	0	0	0	0	0	0	1	0	0
Desulfomicrobium	2	65	0	0	0	0	0	0	0	0	0	0	0	0	0	0	0	1	0	0
Desulfomonile	1	25	0	0	0	0	0	0	0	0	0	0	0	0	0	0	0	0	0	1
Desulforhabdus	1	2	0	0	0	0	0	0	0	0	0	0	0	0	0	0	0	0	0	0
Desulfosporosinus	1	30	0	0	0	0	0	0	0	0	0	0	0	0	0	0	0	0	0	0
Desulfotalea	2	66	0	0	0	0	0	0	0	0	0	0	0	0	0	0	0	1	0	0
Desulfotomaculum	2	98	0	0	0	0	0	0	0	0	0	0	0	0	0	0	0	1	0	1
Desulfovibrio	2	144	0	0	0	0	0	0	0	0	0	0	1	0	0	1	0	1	0	0
Desulfurispirillum	2	78	0	0	0	0	0	0	0	0	0	0	0	0	0	0	0	1	0	0
Desulfurivibrio	2	71	0	0	0	0	0	0	0	0	0	0	0	0	0	0	0	1	0	0
Desulfurobacterium	2	53	0	0	0	0	0	0	0	0	0	0	0	0	0	0	0	1	0	0
Desulfurococcus	1	11	0	0	0	0	0	0	0	0	0	0	0	0	0	0	0	0	0	0
Diaphorobacter	1	16	0	0	0	0	0	0	0	0	0	0	0	0	0	0	0	0	0	0
Dichelobacter	2	45	0	0	0	0	0	0	0	0	0	0	0	0	0	0	0	1	0	0
Dickeya	2	155	0	0	0	0	0	0	0	0	1	0	1	1	0	0	0	1	0	0
Dictyoglomus	2	62	0	0	0	0	0	0	0	0	0	0	0	0	0	0	0	1	0	0
Dinoroseobacter	3	148	0	0	1	0	1	1	0	0	0	0	0	1	0	0	0	1	0	0
Dokdonia	1	3	0	0	0	0	0	0	0	0	0	0	0	0	0	0	0	0	0	0
Dorea	1	7	0	0	0	0	0	0	0	0	0	0	0	0	0	0	0	0	0	0
Dyadobacter	2	83	0	0	0	0	0	0	0	0	1	0	0	0	0	0	0	1	0	0
Echinicola	1	20	0	0	0	0	0	0	0	0	0	0	0	0	0	0	0	0	0	0
Edwardsiella	2	126	0	0	0	0	0	0	0	0	0	0	0	0	0	0	0	1	0	0
Eggerthella	1	51	0	0	0	0	0	0	0	0	0	0	0	0	0	0	0	1	0	0
Ehrlichia	2	37	0	0	0	0	0	0	0	0	0	0	0	0	0	0	0	1	0	0
Eikenella	1	4	0	0	0	0	0	0	0	0	0	0	0	0	0	0	0	1	0	0
Elusimicrobium	2	44	0	0	0	0	0	0	0	0	0	0	0	0	0	0	0	1	0	0
Emticicia	1	21	0	0	0	0	0	0	0	0	0	0	0	0	0	0	0	0	0	0
Enhydrobacter	1	1	0	0	0	0	0	0	0	0	0	0	0	0	0	0	0	0	0	0
Ensifer	1	2	0	0	0	0	0	0	0	0	0	0	0	0	0	0	0	0	0	0
Enterobacter	2	202	0	0	0	0	0	0	0	1	1	0	1	0	0	0	0	1	0	1
Enterobacteriaceae	2	211	0	0	0	0	0	0	0	1	1	0	1	0	0	0	0	1	0	0
Enterococcus	1	187	0	0	0	0	0	0	0	1	1	0	1	0	0	0	0	1	0	1

Genus	Cluster Number	Protein Counts	4.2.1.80	4.2.1.83	4.2.1.84	4.99.1.7	5.1.2.2	5.2.1.2	5.3.3.1	5.3.3.4	5.3.99.-	5.4.99.-	5.5.1.1	5.5.1.2	6.2.1.-	6.2.1.25	6.2.1.32	6.3.5.2	K00002	K00055
Enterovibrio	1	15	0	0	0	0	0	0	0	0	0	0	0	0	0	0	0	1	0	0
Erwinia	2	151	0	0	0	0	0	0	0	0	1	0	1	0	0	0	0	1	0	0
Erysipelothrix	1	9	0	0	0	0	0	0	0	0	0	0	0	0	0	0	0	0	0	0
Erysipelotrichaceae	1	4	0	0	0	0	0	0	0	0	0	0	0	0	0	0	0	0	0	0
Erythrobacter	2	80	0	0	0	0	0	1	0	0	0	0	0	0	1	0	0	1	0	0
Escherichia	2	242	1	0	0	0	0	1	0	1	1	0	1	1	1	0	0	1	0	1
Ethanoligenens	2	69	0	0	0	0	0	0	0	0	0	0	0	0	0	0	0	1	0	0
Eubacteriaceae	1	2	0	0	0	0	0	0	0	0	0	0	0	0	0	0	0	0	0	0
Eubacterium	2	103	0	0	0	0	0	0	0	0	0	0	1	0	0	0	0	1	0	0
Eudoraea	1	4	0	0	0	0	0	0	0	0	1	0	0	0	0	0	0	0	0	0
Euryarchaeota	1	1	0	0	0	0	0	0	0	0	0	0	0	0	0	0	0	0	0	0
Exiguobacterium	2	93	0	0	0	0	0	0	0	0	0	0	0	0	0	0	0	1	0	0
Faecalibacterium	1	12	0	0	0	0	0	0	0	0	0	0	0	0	0	0	0	0	0	0
Ferrimonas	2	115	0	0	0	0	0	1	0	0	0	0	0	0	0	0	0	1	0	0
Ferroglobus	1	14	0	0	0	0	0	0	0	0	0	0	0	0	0	0	0	0	0	0
Ferroplasma	1	11	0	0	0	0	0	0	0	0	0	0	0	0	0	0	0	0	0	0
Fervidicoccus	1	7	0	0	0	0	0	0	0	0	0	0	0	0	0	0	0	0	0	0
Fervidobacterium	2	54	0	0	0	0	0	0	0	0	0	0	1	0	0	0	0	1	0	0
Fibrella	1	17	0	0	0	0	0	0	0	0	0	0	0	0	0	0	0	0	0	0
Fibrobacter	2	52	0	0	0	0	0	0	0	0	0	0	0	0	0	0	0	1	0	0
Filifactor	1	9	0	0	0	0	0	0	0	0	0	0	0	0	0	0	0	0	0	0
Finegoldia	2	55	0	0	0	0	0	0	0	0	0	0	0	0	0	0	0	1	0	0
Firmicutes	1	15	0	0	0	0	0	0	0	0	0	0	0	0	0	0	0	0	0	0
Fischerella	1	15	0	0	0	0	0	0	0	0	0	0	0	0	0	0	0	1	0	0
Flavobacteria	1	7	0	0	0	0	0	0	0	0	0	0	0	0	0	0	0	0	0	0
Flavobacteriaceae	2	69	0	0	0	0	0	0	0	0	0	0	1	0	0	0	0	1	0	0
Flavobacteriales	1	3	0	0	0	0	0	0	0	0	0	0	0	0	0	0	0	0	0	0
Flavobacterium	2	103	0	0	0	0	0	0	0	0	0	0	0	0	0	0	0	1	0	0
Flexibacter	1	17	0	0	0	0	0	0	0	0	0	0	0	0	0	0	0	0	0	0
Flexistipes	1	11	0	0	0	0	0	0	0	0	0	0	0	0	0	0	0	0	0	0
Fluoribacter	1	6	0	0	0	0	0	0	0	0	0	0	0	0	0	0	0	0	0	0
Fluviicola	2	69	0	0	0	0	0	0	0	0	0	0	0	0	0	0	0	1	0	0
Formosa	1	2	0	0	0	0	0	0	0	0	0	0	0	0	0	0	0	0	0	0
Francisella	2	88	1	0	0	0	0	1	0	0	0	0	1	0	0	0	0	1	0	0
Frankia	3	202	0	0	0	0	1	0	0	0	1	0	0	1	0	0	0	1	0	1
Frateuria	1	25	0	0	0	0	0	0	0	1	0	0	1	0	0	0	0	0	0	0
Fructobacillus	1	2	0	0	0	0	0	0	0	0	0	0	0	0	0	0	0	0	0	0
Fulvimarina	1	2	0	0	0	0	0	0	0	0	0	0	0	0	0	0	0	0	0	0
Fusobacterium	1	48	0	0	0	0	0	0	0	0	0	0	0	0	0	0	0	1	0	0
Gallibacterium	2	87	0	0	0	0	0	0	0	0	0	0	0	0	0	0	0	1	0	0
Gallionella	2	67	0	0	0	0	0	0	0	0	0	0	0	0	0	0	0	1	0	0
Gammaproteobacteria	1	9	0	0	0	0	0	0	0	0	0	0	0	0	0	0	0	0	0	0
Gardnerella	1	31	0	0	0	0	0	0	0	0	0	0	0	0	0	0	0	1	0	0
Geitlerinema	1	9	0	0	0	0	0	0	0	0	0	0	0	0	0	0	0	0	0	0
Gemmatimonas	2	93	0	0	0	0	0	0	0	0	0	0	1	0	0	0	0	1	0	0
Geobacillus	2	168	0	0	0	0	0	0	0	0	1	0	0	0	1	1	0	1	0	0
Geobacter	2	150	0	0	0	0	1	0	0	0	0	0	1	0	1	1	0	1	0	0
Geodermatophilus	2	136	0	0	1	0	0	0	0	1	1	0	1	1	0	0	0	1	0	0
Geopsychrobacter	1	2	0	0	0	0	0	0	0	0	0	0	0	0	0	0	0	0	0	0
Gillisia	1	11	0	0	0	0	0	0	0	0	0	0	0	0	0	0	0	0	0	0
Gilvimarinus	1	4	0	0	0	0	0	0	0	0	0	0	0	0	0	0	0	0	0	0
Glaciecola	2	117	0	1	0	0	0	1	0	0	1	0	0	0	1	0	0	1	0	0

Genus	Cluster Number	Protein Counts	4.2.1.80	4.2.1.83	4.2.1.84	4.99.1.7	5.1.2.2	5.2.1.2	5.3.3.1	5.3.3.4	5.3.99.-	5.4.99.-	5.5.1.1	5.5.1.2	6.2.1.-	6.2.1.25	6.2.1.32	6.3.5.2	K00002	K00055
Gloeobacter	2	67	0	0	0	0	0	0	0	0	0	0	0	0	0	0	0	1	0	0
Gloeocapsa	1	20	0	0	0	0	0	0	0	0	0	0	0	0	0	0	0	0	0	0
Gloeothece	1	1	0	0	0	0	0	0	0	0	0	0	0	0	0	0	0	0	0	0
Gluconacetobacter	2	96	0	0	0	0	0	0	0	0	0	0	0	1	0	0	0	1	0	0
Gluconobacter	2	76	0	0	0	0	0	0	0	0	0	0	0	0	0	0	0	1	0	0
Gordonia	1	158	0	0	0	0	0	0	0	1	0	0	1	0	0	0	0	1	0	1
Gordonibacter	1	32	0	0	0	0	0	0	0	0	0	0	1	0	0	0	0	1	0	1
Gramella	2	104	0	0	0	0	0	0	0	0	1	0	0	0	0	0	0	1	1	0
Granulibacter	2	90	0	0	0	0	0	0	0	0	0	0	0	0	0	0	0	1	0	0
Granulicella	2	98	0	0	0	0	1	0	0	0	0	0	0	0	0	0	0	1	0	0
Grimontia	1	9	0	0	0	0	0	0	0	0	0	0	0	0	0	0	0	0	0	0
Haemophilus	2	117	0	0	0	0	0	0	0	0	0	0	0	0	0	0	0	1	0	0
Hafnia	1	29	0	0	0	0	0	0	0	0	0	0	0	0	0	0	0	1	0	0
Hahella	3	130	0	0	0	0	0	1	0	0	1	0	0	0	0	0	0	1	0	0
Halalkalicoccus	1	18	0	0	0	0	0	0	0	0	0	0	0	0	0	0	0	0	0	0
Halanaerobium	2	89	0	0	0	0	0	0	0	0	0	0	0	0	0	0	0	1	0	0
Haliangium	2	108	0	0	0	0	0	1	0	0	0	1	0	0	0	1	0	1	0	1
Haliscomenobacter	2	107	0	0	0	0	0	0	0	0	1	0	0	1	0	0	0	1	0	0
Haloarcula	1	22	0	0	0	0	0	0	0	0	0	0	0	0	0	0	0	0	0	0
Halobacillus	1	19	0	0	0	0	0	0	0	0	0	0	0	0	0	0	0	0	0	0
Halobacteriaceae	1	1	0	0	0	0	0	0	0	0	0	0	0	0	0	0	0	0	0	0
Halobacterium	1	10	0	0	0	0	0	0	0	0	0	0	0	0	0	0	0	0	0	0
Halobacteroides	1	14	0	0	0	0	0	0	0	0	0	0	0	0	0	0	0	0	0	0
Haloferax	1	20	0	0	0	0	0	0	0	0	0	0	0	0	0	0	0	0	0	1
Halogeometricum	1	12	0	0	0	0	0	0	0	0	0	0	0	0	0	0	0	0	0	0
Halomicrobium	1	10	0	0	0	0	0	0	0	0	0	0	0	0	0	0	0	0	0	0
Halomonas	1	149	0	0	0	0	1	0	0	0	1	0	0	1	0	0	0	1	0	0
Halopiger	1	16	0	0	0	0	0	0	0	0	0	0	0	0	0	0	0	0	0	0
Haloquadratum	1	19	0	0	0	0	0	0	0	0	0	0	0	0	0	0	0	0	0	0
Halorhabdus	1	10	0	0	0	0	0	0	0	0	0	0	0	0	0	0	0	0	0	0
Halorhodospira	2	71	0	0	0	0	0	0	0	0	0	0	0	0	0	0	0	1	0	0
Halorubrum	1	16	0	0	0	0	0	0	0	0	0	0	0	0	0	0	0	0	0	0
Haloterrigena	1	19	0	0	0	0	0	0	0	0	0	0	0	0	0	0	0	0	0	0
Halothece	1	9	0	0	0	0	0	0	0	0	0	0	0	0	0	0	0	0	0	0
Halothermothrix	2	50	0	0	0	0	0	0	0	0	0	0	0	0	0	0	0	1	0	0
Halothiobacillus	2	65	0	0	0	0	0	0	0	0	0	0	0	0	0	0	0	1	0	0
Halovivax	1	12	0	0	0	0	0	0	0	0	0	0	0	0	0	0	0	0	0	0
Helicobacter	2	79	0	0	0	0	0	0	0	0	0	0	0	0	0	0	0	1	1	0
Heliobacillus	1	4	0	0	0	0	0	0	0	0	0	0	0	0	0	0	0	0	0	0
Heliobacterium	2	51	0	0	0	0	0	0	0	0	0	0	0	0	0	0	0	1	0	0
Herbaspirillum	2	172	0	0	1	0	0	0	0	1	0	0	1	1	1	0	0	1	0	0
Herminiimonas	3	89	0	0	0	0	0	0	0	0	0	0	0	0	0	0	0	1	0	0
Herpetosiphon	3	90	0	0	0	0	0	0	0	0	0	0	0	0	0	0	0	1	0	0
Hippea	2	44	0	0	0	0	0	0	0	0	0	0	0	0	0	0	0	1	0	0
Hirschia	2	102	0	1	0	0	0	1	0	0	0	0	0	0	0	0	0	1	0	0
Histophilus	2	72	0	0	0	0	0	0	0	0	0	0	0	0	0	0	0	1	0	0
Hoeflea	1	16	0	0	0	0	0	0	0	0	0	0	0	0	0	0	0	0	0	0
Hydrogenivirga	1	2	0	0	0	0	0	0	0	0	0	0	0	0	0	0	0	0	0	0
Hydrogenobacter	2	55	0	0	0	0	0	0	0	0	0	0	0	0	0	0	0	1	0	0
Hydrogenobaculum	2	44	0	0	0	0	0	0	0	0	0	0	0	0	0	0	0	1	0	0
Hydrogenophaga	1	9	0	0	0	0	0	0	0	0	0	0	0	0	0	0	0	1	0	0
Hylemonella	1	4	0	0	0	0	0	0	0	0	0	0	0	0	0	0	0	1	0	0

Genus	Cluster Number	Protein Counts	4.2.1.80	4.2.1.83	4.2.1.84	4.99.1.7	5.1.2.2	5.2.1.2	5.3.3.1	5.3.3.4	5.3.99.-	5.4.99.-	5.5.1.1	5.5.1.2	6.2.1.-	6.2.1.25	6.2.1.32	6.3.5.2	K00002	K00055
**Hyperthermus**	1	6	0	0	0	0	0	0	0	0	0	0	0	0	0	0	0	0	0	0
**Hyphomicrobium**	2	93	0	0	0	0	0	0	0	0	0	0	1	0	0	0	0	1	0	0
**Hyphomonas**	3	105	0	0	0	0	0	0	0	0	0	0	0	0	0	0	0	1	0	1
**Ideonella**	1	18	0	1	0	0	0	0	0	0	0	0	0	0	0	0	0	0	0	0
**Idiomarina**	2	86	0	0	0	0	0	1	0	0	0	0	0	0	0	0	0	1	0	0
**Ignavibacterium**	1	14	0	0	0	0	0	0	0	0	0	0	0	0	0	0	0	0	0	0
**Ignicoccus**	1	5	0	0	0	0	0	0	0	0	0	0	0	0	0	0	0	0	0	0
**Ignisphaera**	1	8	0	0	0	0	0	0	0	0	0	0	0	0	0	0	0	0	0	0
**Ilyobacter**	2	85	0	0	0	0	0	0	0	0	1	0	0	0	0	0	0	1	0	0
**Intrasporangium**	2	114	0	0	0	0	0	0	0	0	0	0	0	0	0	0	0	1	0	0
**Isoptericola**	1	18	0	0	0	0	0	0	0	0	0	0	0	0	0	0	0	0	0	0
**Isosphaera**	2	75	0	0	0	0	0	0	0	0	1	0	1	0	0	0	0	1	0	0
**Jannaschia**	3	151	0	0	0	0	0	1	0	1	1	0	1	1	0	1	0	1	0	0
**Janthinobacterium**	2	99	0	0	0	0	0	0	0	0	0	0	0	0	0	0	0	1	0	0
**Jonesia**	2	56	0	0	0	0	0	0	0	0	0	0	0	0	0	0	0	1	0	0
**Joostella**	1	3	0	0	0	0	0	0	0	0	0	0	0	0	0	0	0	0	0	0
**Kaistia**	1	2	0	0	0	0	0	0	0	0	0	0	0	0	0	0	0	0	0	0
**Kangiella**	2	84	0	0	0	0	0	1	0	0	0	0	0	0	0	0	0	1	0	0
**Ketogulonicigenium**	2	102	0	0	0	0	0	0	0	0	1	0	0	1	0	0	0	1	0	0
**Kineococcus**	1	24	0	0	0	0	0	0	0	0	0	0	0	0	0	0	0	0	0	0
**Kinetoplastibacterium**	1	2	0	0	0	0	0	0	0	0	0	0	0	0	0	0	0	0	0	0
**Kingella**	1	4	0	0	0	0	0	0	0	0	0	0	0	0	0	0	0	1	0	0
**Kitasatospora**	1	36	0	0	0	0	0	0	0	0	0	0	0	0	0	0	0	1	0	0
**Klebsiella**	3	222	0	0	1	0	0	0	0	1	1	0	1	1	1	0	0	1	0	1
**Kluyvera**	1	1	0	0	0	0	0	0	0	0	0	0	0	0	0	0	0	0	0	0
**Kocuria**	2	87	0	0	0	0	0	0	0	0	0	0	0	1	0	0	0	1	1	0
**Kordia**	1	5	0	0	0	0	0	0	0	0	0	0	0	0	0	0	0	0	0	0
**Kosmotoga**	2	61	0	0	0	0	0	0	0	0	0	0	1	0	0	0	0	1	0	0
**Kribbella**	2	99	0	0	0	0	0	0	0	0	1	0	0	0	0	0	0	1	0	0
**Krokinobacter**	2	90	0	0	0	0	0	0	0	0	1	0	0	0	0	0	0	1	0	0
**Kyrpidia**	2	113	0	0	0	0	0	0	0	0	0	0	0	0	0	0	0	1	0	0
**Kytococcus**	2	70	0	0	0	0	0	0	0	0	0	0	0	0	0	0	0	1	0	0
**Labrenzia**	1	4	0	0	0	0	0	0	0	0	0	0	0	0	0	0	0	0	0	0
**Lachnoanaerobaculum**	1	1	0	0	0	0	0	0	0	0	0	0	0	0	0	0	0	0	0	0
**Lachnospiraceae**	1	51	0	0	0	0	0	0	0	0	1	0	0	0	0	0	0	0	0	0
**Lacinutrix**	1	20	0	0	0	0	0	0	0	0	0	0	0	0	0	0	0	0	0	0
**Lactobacillus**	2	119	0	0	0	0	0	0	0	0	1	0	1	0	0	0	0	1	0	1
**Lactococcus**	2	82	0	0	0	0	0	0	0	0	0	0	0	0	0	0	0	1	0	0
**Lamprocystis**	1	2	0	0	0	0	0	0	0	0	0	0	0	0	0	0	0	0	0	0
**Laribacter**	2	80	0	0	0	0	0	0	0	0	0	0	0	0	0	0	0	1	0	0
**Lawsonia**	2	33	0	0	0	0	0	0	0	0	0	0	0	0	0	0	0	1	0	0
**Leadbetterella**	2	79	0	0	0	0	0	0	0	0	1	0	1	0	0	0	0	1	0	0
**Leeuwenhoekiella**	1	2	0	0	0	0	0	0	0	0	0	0	0	0	0	0	0	0	0	0
**Legionella**	2	117	0	0	1	0	0	0	0	0	0	0	1	0	0	0	0	1	0	0
**Leifsonia**	2	48	0	0	0	0	0	0	0	0	1	0	0	0	0	0	0	1	0	0
**Leptolyngbya**	1	10	0	0	0	0	0	0	0	0	0	0	0	0	0	0	0	0	0	0
**Leptospira**	2	76	0	0	0	0	0	1	0	0	0	0	0	0	0	0	0	1	0	0
**Leptospirillum**	1	10	0	0	0	0	0	0	0	0	0	0	0	0	0	0	0	0	0	0
**Leptothrix**	3	186	0	1	0	0	0	1	0	0	1	0	0	1	1	1	0	1	0	0
**Leptotrichia**	2	43	0	0	0	0	0	0	0	0	0	0	0	0	0	0	0	1	0	0
**Leucobacter**	1	2	0	0	0	0	0	0	0	0	0	0	0	0	0	0	0	0	0	0
**Leuconostoc**	2	64	0	0	0	0	0	0	0	0	0	0	0	0	0	0	0	1	0	0

Genus	Cluster Number	Protein Counts	4.2.1.80	4.2.1.83	4.2.1.84	4.99.1.7	5.1.2.2	5.2.1.2	5.3.3.1	5.3.3.4	5.3.99.-	5.4.99.-	5.5.1.1	5.5.1.2	6.2.1.-	6.2.1.25	6.2.1.32	6.3.5.2	K00002	K00055
Liberibacter	1	4	0	0	0	0	0	0	0	0	0	0	0	0	0	0	0	0	0	0
Limnohabitans	1	11	0	0	0	0	0	0	0	0	0	0	0	0	0	0	0	1	0	0
Listeria	2	77	0	0	0	0	0	0	0	0	1	0	1	0	0	0	0	1	0	0
Listeriaceae	1	6	0	0	0	0	0	0	0	0	0	0	0	0	0	0	0	1	0	0
Listonella	1	38	0	0	0	0	0	0	0	0	0	0	0	0	0	0	0	1	0	0
Loktanella	1	3	0	0	0	0	0	0	0	0	0	0	0	0	0	0	0	0	0	0
Lysinibacillus	2	97	0	0	0	0	0	0	0	0	0	0	1	0	1	0	0	1	0	0
Macrococcus	2	60	0	0	0	0	0	0	0	0	1	0	1	0	0	0	0	1	0	0
Magnetococcus	2	64	0	0	0	0	0	0	0	0	0	0	0	0	0	0	0	1	0	0
Magnetospirillum	2	113	0	1	0	0	0	0	0	0	0	0	0	0	0	1	0	1	0	0
Mahella	2	58	0	0	0	0	0	0	0	0	1	0	0	0	0	0	0	1	0	0
Mannheimia	2	85	0	0	0	0	0	0	0	0	1	0	0	0	0	0	0	1	0	0
Maribacter	3	87	0	0	0	0	0	0	0	0	1	0	0	0	0	0	0	1	0	0
Maricaulis	3	87	0	0	0	0	0	1	0	0	0	0	0	0	0	0	0	1	0	0
Marichromatium	1	2	0	0	0	0	0	0	0	0	0	0	0	0	0	0	0	0	0	0
Marinithermus	2	77	0	0	0	0	0	0	0	0	0	0	1	0	0	0	0	1	0	0
Marinitoga	1	15	0	0	0	0	0	0	0	0	0	0	0	0	0	0	0	0	0	0
Marinobacter	2	163	0	0	0	0	0	1	0	0	0	0	0	0	1	0	0	1	0	0
Marinomonas	3	192	0	1	0	0	0	0	0	1	1	0	1	0	0	0	0	1	0	1
Maritimibacter	1	7	0	0	0	0	0	0	0	0	0	0	0	0	0	0	0	0	0	0
Marivirga	2	96	0	0	0	0	0	0	0	0	0	0	0	0	0	0	0	1	1	0
Martelella	1	3	0	0	0	0	0	0	0	0	0	0	0	0	0	0	0	0	0	0
Marvinbryantia	1	1	0	0	0	0	0	0	0	0	0	0	0	0	0	0	0	0	0	0
Massilia	1	4	0	0	0	0	0	0	0	0	0	0	0	0	0	0	0	0	0	0
Mastigocladopsis	1	16	0	0	0	0	0	0	0	0	0	0	0	0	0	0	0	0	0	0
Megamonas	1	47	0	0	0	0	0	0	0	0	1	0	0	0	0	0	0	1	0	0
Megasphaera	1	14	0	0	0	0	0	0	0	0	0	0	0	0	0	0	0	0	0	0
Meiothermus	2	110	0	0	0	0	0	0	0	0	1	0	1	1	0	0	0	1	0	0
Melissococcus	2	46	0	0	0	0	0	0	0	0	0	0	0	0	0	0	0	1	0	0
Mesoflavibacter	1	6	0	0	0	0	0	0	0	0	0	0	0	0	0	0	0	0	0	0
Mesoplasma	1	23	0	0	0	0	0	0	0	0	0	0	0	0	0	0	0	1	0	0
Mesorhizobium	2	201	0	0	1	0	0	1	0	1	1	0	1	1	0	0	0	1	0	0
Mesotoga	1	14	0	0	0	0	0	0	0	0	0	0	0	0	0	0	0	0	0	0
Metallosphaera	1	18	0	0	0	0	0	0	0	0	0	0	0	0	0	0	0	0	0	0
Methanobacterium	1	7	0	0	0	0	0	0	0	0	0	0	0	0	0	0	0	0	0	0
Methanobrevibacter	1	9	0	0	0	0	0	0	0	0	0	0	0	0	0	0	0	0	0	0
Methanocaldococcus	1	6	0	0	0	0	0	0	0	0	0	0	0	0	0	0	0	0	0	0
Methanocella	1	15	0	0	0	0	0	0	0	0	0	0	0	0	0	0	0	0	0	0
Methanococcaceae	1	1	0	0	0	0	0	0	0	0	0	0	0	0	0	0	0	0	0	0
Methanococcoides	1	5	0	0	0	0	0	0	0	0	0	0	0	0	0	0	0	0	0	0
Methanococcus	1	9	0	0	0	0	0	0	0	0	0	0	0	0	0	0	0	0	0	0
Methanocorpusculum	1	7	0	0	0	0	0	0	0	0	0	0	0	0	0	0	0	0	0	0
Methanoculleus	1	8	0	0	0	0	0	0	0	0	0	0	0	0	0	0	0	0	0	0
Methanohalobium	1	5	0	0	0	0	0	0	0	0	0	0	0	0	0	0	0	0	0	0
Methanohalophilus	1	5	0	0	0	0	0	0	0	0	0	0	0	0	0	0	0	0	0	0
Methanolobus	1	5	0	0	0	0	0	0	0	0	0	0	0	0	0	0	0	0	0	0
Methanomethylovorans	1	5	0	0	0	0	0	0	0	0	0	0	0	0	0	0	0	0	0	0
Methanoplanus	1	7	0	0	0	0	0	0	0	0	0	0	0	0	0	0	0	0	0	0
Methanopyrus	1	4	0	0	0	0	0	0	0	0	0	0	0	0	0	0	0	0	0	0
Methanoregula	1	8	0	0	0	0	0	0	0	0	0	0	0	0	0	0	0	0	0	0
Methanosaeta	1	9	0	0	0	0	0	0	0	0	0	0	0	0	0	0	0	0	0	0
Methanosalsum	1	7	0	0	0	0	0	0	0	0	0	0	0	0	0	0	0	0	0	0

Genus	Cluster Number	Protein Counts	4.2.1.80	4.2.1.83	4.2.1.84	4.99.1.7	5.1.2.2	5.2.1.2	5.3.3.1	5.3.3.4	5.3.99.-	5.4.99.-	5.5.1.1	5.5.1.2	6.2.1.-	6.2.1.25	6.2.1.32	6.3.5.2	K00002	K00055
Methanosarcina	1	18	0	0	0	0	0	0	0	0	0	0	0	0	0	0	0	0	1	0
Methanosphaera	1	7	0	0	0	0	0	0	0	0	0	0	0	0	0	0	0	0	1	0
Methanosphaerula	1	8	0	0	0	0	0	0	0	0	0	0	0	0	0	0	0	0	0	0
Methanospirillum	1	6	0	0	0	0	0	0	0	0	0	0	0	0	0	0	0	0	0	0
Methanothermobacter	1	6	0	0	0	0	0	0	0	0	0	0	0	0	0	0	0	0	0	0
Methanothermococcus	1	5	0	0	0	0	0	0	0	0	0	0	0	0	0	0	0	0	0	0
Methanothermus	1	5	0	0	0	0	0	0	0	0	0	0	0	0	0	0	0	0	0	0
Methanotorris	1	5	0	0	0	0	0	0	0	0	0	0	0	0	0	0	0	0	0	0
Methylacidiphilum	2	48	0	0	0	0	0	0	0	0	0	0	0	0	0	0	0	1	0	0
Methylarcula	1	2	0	0	0	0	0	0	0	0	0	0	0	0	0	0	0	0	0	0
Methylibium	1	38	0	0	0	0	0	0	0	0	0	0	0	0	0	0	0	0	0	0
Methylobacillus	2	61	0	0	0	0	0	0	0	0	0	0	0	0	0	0	0	1	0	0
Methylobacterium	3	236	0	1	1	0	1	1	0	1	1	0	1	1	0	0	0	1	0	0
Methylocella	3	117	0	0	1	0	0	0	0	0	0	0	0	0	0	0	0	1	0	1
Methylococcus	2	80	0	0	0	0	0	0	0	0	0	0	0	0	0	0	0	1	0	0
Methylocystis	1	21	0	0	0	0	0	0	0	0	0	0	0	0	0	0	0	0	0	0
Methyloferula	1	2	0	0	0	0	0	0	0	0	0	0	0	0	0	0	0	0	0	0
Methylomicrobium	1	12	0	0	0	0	0	0	0	0	0	0	0	0	0	0	0	0	0	0
Methylomonas	1	15	0	0	0	0	0	0	0	0	0	0	0	0	0	0	0	0	0	0
Methylophaga	1	7	0	0	0	0	0	0	0	0	0	0	0	0	0	0	0	0	0	0
Methylophilus	1	2	0	0	0	0	0	0	0	0	0	0	0	0	0	0	0	0	0	0
Methylosinus	1	2	0	0	0	0	0	0	0	0	0	0	0	0	0	0	0	0	0	0
Methylotenera	2	72	0	0	0	0	0	0	0	0	0	0	0	0	0	0	0	1	0	0
Methyloversatilis	1	5	0	0	0	0	0	0	0	0	0	0	0	0	0	0	0	0	0	0
Methylovorus	2	66	0	0	0	0	0	0	0	0	0	0	0	0	0	0	0	1	0	0
Micavibrio	1	9	0	0	0	0	0	0	0	0	0	0	0	0	0	0	0	0	0	0
Microbacterium	2	115	0	0	1	0	0	0	0	0	1	0	1	1	0	0	0	1	0	1
Microchaete	1	23	0	0	0	0	0	0	0	0	0	0	0	0	0	0	0	1	0	0
Micrococcus	2	72	0	0	0	0	0	0	0	0	0	0	0	0	0	0	0	1	0	0
Microcoleus	1	17	0	0	0	0	0	0	0	0	0	0	0	0	0	0	0	0	0	0
Microcystis	2	67	0	0	0	0	0	0	0	0	0	0	0	0	0	0	0	1	0	0
Microlunatus	1	25	0	0	0	0	0	0	0	0	0	0	0	0	0	0	0	0	0	0
Micromonospora	2	115	0	0	0	0	0	0	0	0	1	0	0	0	0	0	0	1	0	0
Microvirga	1	6	0	0	0	0	0	0	0	1	0	0	0	0	0	0	0	0	0	0
Mobiluncus	1	41	0	0	0	0	0	0	0	0	0	0	0	0	0	0	0	1	0	0
Modestobacter	1	43	0	0	0	0	0	0	0	0	0	0	0	0	0	0	0	0	0	1
Moorea	1	2	0	0	0	0	0	0	0	0	0	0	0	0	0	0	0	0	0	0
Moorella	2	69	0	0	0	0	0	0	0	0	0	0	0	0	0	0	0	1	0	0
Moraxella	2	66	0	0	0	0	0	1	0	0	0	0	0	0	0	0	0	1	0	0
Morganella	1	29	0	0	0	0	0	0	0	0	0	0	0	0	0	0	0	1	0	0
Moritella	1	2	0	0	0	0	0	0	0	0	0	0	0	0	0	0	0	1	0	0
Muricauda	1	23	0	0	0	0	0	0	0	0	1	0	0	0	0	0	0	0	0	0
Mycobacterium	3	306	1	0	1	0	1	0	1	1	0	0	1	1	1	0	0	1	0	1
Mycoplasma	1	53	0	0	0	0	0	0	0	0	0	0	0	0	0	0	0	1	0	0
Myxococcus	2	110	0	0	0	0	0	1	0	0	0	0	0	0	0	0	0	1	0	0
Nakamurella	2	115	0	0	0	0	1	0	0	0	1	0	0	0	0	0	0	1	0	0
napthalene	1	2	0	0	0	0	0	0	0	0	0	0	0	0	0	0	0	0	0	0
Natranaerobius	2	55	0	0	0	0	0	0	0	0	0	0	0	0	0	0	0	1	0	0
Natrialba	1	16	0	0	0	0	0	0	0	0	0	0	0	0	0	0	0	0	0	0
Natrinema	1	16	0	0	0	0	0	0	0	0	0	0	0	0	0	0	0	0	0	0
Natronobacterium	1	11	0	0	0	0	0	0	0	0	0	0	0	0	0	0	0	0	0	0
Natronococcus	1	21	0	0	0	0	0	0	0	0	0	0	0	0	0	0	0	0	0	0

Genus	Cluster Number	Protein Counts	4.2.1.80	4.2.1.83	4.2.1.84	4.99.1.7	5.1.2.2	5.2.1.2	5.3.3.1	5.3.3.4	5.3.99.-	5.4.99.-	5.5.1.1	5.5.1.2	6.2.1.-	6.2.1.25	6.2.1.32	6.3.5.2	K00002	K00055
Natronomonas	1	20	0	0	0	0	0	0	0	0	0	0	0	0	0	0	0	0	0	0
Nautilia	2	48	0	0	0	0	0	0	0	0	0	0	0	0	0	0	0	1	0	0
Neisseria	2	67	0	0	0	0	0	0	0	0	0	0	0	0	0	0	0	1	0	0
Neisseriaceae	1	1	0	0	0	0	0	0	0	0	0	0	0	0	0	0	0	0	0	0
Neorickettsia	1	31	0	0	0	0	0	0	0	0	0	0	0	0	0	0	0	1	0	0
Neptuniibacter	1	2	0	0	0	0	0	0	0	0	0	0	0	0	0	0	0	0	0	0
Niastella	1	21	0	0	0	0	0	0	0	0	0	0	0	0	0	0	0	0	0	0
Nitratifractor	2	54	0	0	0	0	0	0	0	0	0	0	0	0	0	0	0	1	0	0
Nitratireductor	1	19	0	0	0	0	0	0	0	0	0	0	0	0	0	0	0	1	0	0
Nitratiruptor	2	40	1	0	0	0	0	0	0	0	0	0	0	0	0	0	0	1	0	0
Nitrobacter	2	99	0	0	0	0	1	1	0	0	0	0	1	0	0	0	0	1	0	0
Nitrococcus	1	2	0	0	0	0	0	0	0	0	0	0	0	0	0	0	0	0	0	0
Nitrosococcus	2	94	0	0	0	0	1	0	0	0	0	0	0	0	0	0	0	1	0	0
Nitrosomonas	2	72	0	0	0	0	0	0	0	0	0	0	0	0	0	0	0	1	0	1
Nitrosopumilaceae	1	7	0	0	0	0	0	0	0	0	0	0	0	0	0	0	0	0	0	0
Nitrosopumilus	1	11	0	0	0	0	0	0	0	0	0	0	0	0	0	0	0	0	0	0
Nitrosospira	2	74	0	0	0	0	0	0	0	0	0	0	0	0	0	0	0	1	0	1
Nocardia	2	185	0	0	1	0	0	0	1	1	0	0	1	0	0	0	0	1	0	1
Nocardioidaceae	1	2	0	0	0	0	0	0	0	0	0	0	0	0	0	0	0	0	0	0
Nocardioides	2	157	1	0	0	0	1	0	1	1	0	0	1	0	0	0	0	1	0	1
Nocardiopsis	2	102	0	0	0	0	0	0	0	0	1	0	0	0	0	0	0	1	0	0
Nodularia	1	22	0	0	0	0	0	0	0	0	0	0	0	0	0	0	0	1	0	0
Nonlabens	1	19	0	0	0	0	0	0	0	0	0	0	0	0	0	0	0	0	0	0
Nostoc	2	111	0	0	0	0	0	1	0	0	0	0	1	0	0	0	0	1	0	0
Nostocaceae	1	2	0	0	0	0	0	0	0	0	0	0	0	0	0	0	0	0	0	0
Novosphingobium	2	169	0	1	0	0	0	1	0	1	1	0	1	0	0	0	0	1	0	1
Oceanibaculum	1	3	0	0	0	0	0	0	0	1	0	0	0	0	0	0	0	0	0	0
Oceanibulbus	1	12	0	0	0	0	0	0	0	0	0	0	0	0	0	0	0	0	0	0
Oceanicola	1	9	0	0	0	0	0	0	0	0	0	0	0	0	0	0	0	0	0	0
Oceanimonas	1	33	0	0	0	0	0	0	0	0	0	0	0	0	0	0	0	0	0	0
Oceaniovalibus	1	2	0	0	0	0	0	0	0	0	0	0	0	0	0	0	0	0	0	0
Oceanithermus	2	72	0	0	0	0	0	0	0	0	0	0	1	0	0	0	0	1	0	0
Oceanobacillus	2	91	0	0	0	0	0	0	0	0	1	0	1	0	0	0	0	1	0	0
Oceanospirillum	1	2	0	0	0	0	0	0	0	0	0	0	0	0	0	0	0	0	0	0
Ochrobactrum	2	133	0	0	0	0	0	0	0	0	1	0	1	1	0	0	0	1	0	0
Octadecabacter	1	29	0	0	0	0	0	0	0	0	0	0	0	0	0	0	0	0	0	0
Oenococcus	2	41	0	0	0	0	0	0	0	0	0	0	0	0	0	0	0	1	0	0
Oleomonas	1	2	0	0	0	0	0	0	0	0	0	0	0	0	0	0	0	0	0	0
Oligotropha	3	87	0	0	0	0	0	0	0	1	0	0	0	0	0	0	0	1	0	0
Olsenella	2	40	0	0	0	0	0	0	0	0	0	0	0	0	0	0	0	1	0	0
Opitutus	2	67	0	0	0	0	0	0	0	0	1	0	0	0	0	0	0	1	0	0
Orientia	1	21	0	0	0	0	0	0	0	0	0	0	0	0	0	0	0	0	0	0
Ornithobacterium	1	11	0	0	0	0	0	0	0	0	0	0	0	0	0	0	0	0	0	0
Oscillatoria	1	16	0	0	0	0	0	0	0	0	0	0	0	0	0	0	0	0	0	0
Oscillatoriales	1	2	0	0	0	0	0	0	0	0	0	0	0	0	0	0	0	0	0	0
Oscillibacter	1	20	0	0	0	0	0	0	0	0	0	0	0	0	0	0	0	0	0	0
Owenweeksia	1	15	0	0	0	0	0	0	0	0	0	0	0	0	0	0	0	0	0	0
Oxalobacteraceae	1	4	0	0	0	0	0	0	0	0	0	0	0	0	0	0	0	0	0	0
Paenibacillus	2	152	0	0	1	0	0	0	0	0	1	0	0	0	0	0	0	1	0	1
Paludibacter	2	67	0	0	0	0	0	0	0	0	0	0	0	0	0	0	0	1	1	0
Pandoraea	1	18	0	0	0	0	0	0	0	0	0	0	1	0	0	0	0	0	0	0
Pannonibacter	1	3	0	0	0	0	0	0	0	0	0	0	0	0	0	0	0	0	0	0

Genus	Cluster Number	Protein Counts	4.2.1.80	4.2.1.83	4.2.1.84	4.99.1.7	5.1.2.2	5.2.1.2	5.3.3.1	5.3.3.4	5.3.99.-	5.4.99.-	5.5.1.1	5.5.1.2	6.2.1.-	6.2.1.25	6.2.1.32	6.3.5.2	K00002	K00055
**Pantholops**	1	23	0	1	0	0	0	0	0	0	0	0	0	0	0	0	0	0	0	0
**Pantoea**	2	181	0	0	1	0	1	0	0	0	1	0	1	1	0	0	0	1	0	0
**Parabacteroides**	2	64	0	0	0	0	0	0	0	0	1	0	0	0	0	0	0	1	0	0
**Parachlamydia**	1	11	0	0	0	0	0	0	0	0	0	0	0	0	0	0	0	0	0	0
**Paracoccus**	2	169	1	1	0	0	0	1	0	1	1	0	1	1	0	1	0	1	0	0
**Parvibaculum**	3	117	0	0	0	0	0	1	0	0	0	0	1	0	0	0	0	1	0	1
**Parvularcula**	2	72	0	0	0	0	0	0	0	0	0	0	0	0	0	0	0	1	0	0
**Pasteurella**	2	85	0	0	0	0	0	0	0	0	0	0	0	0	0	0	0	1	0	0
**Pasteurellaceae**	1	2	0	0	0	0	0	0	0	0	0	0	0	0	0	0	0	0	0	0
**Pectobacterium**	1	44	0	0	0	0	0	0	0	0	0	0	0	0	0	0	0	1	0	0
**Pediococcus**	1	43	0	0	0	0	0	0	0	0	0	0	0	0	0	0	0	1	0	1
**Pedobacter**	2	88	0	0	0	0	0	0	0	0	1	0	1	0	0	0	0	1	0	0
**Pelagibaca**	1	10	0	0	0	0	0	0	0	0	0	0	0	0	0	0	0	0	0	0
**Pelagibacterium**	1	39	0	0	0	0	0	0	0	0	0	0	0	0	0	0	0	0	0	0
**Pelobacter**	2	85	0	0	0	0	0	0	0	0	0	0	0	0	0	0	0	1	0	0
**Pelodictyon**	2	62	0	0	0	0	0	0	0	0	0	0	0	0	0	0	0	1	0	0
**Pelosinus**	1	1	0	0	0	0	0	0	0	0	0	0	0	0	0	0	0	0	0	0
**Pelotomaculum**	2	60	1	0	0	0	0	0	0	0	0	0	0	0	0	0	0	1	0	0
**Peptostreptococcus**	1	1	0	0	0	0	0	0	0	0	0	0	0	0	0	0	0	0	0	0
**Persephonella**	2	55	0	0	0	0	0	0	0	0	0	0	0	0	0	0	0	1	0	0
**Petrotoga**	2	72	0	0	0	0	0	0	0	0	0	0	1	0	0	0	0	1	0	0
**Phaeobacter**	2	55	0	0	0	0	0	0	0	0	0	0	1	0	0	0	0	1	0	0
**Phenylobacterium**	3	103	0	0	0	0	0	0	0	0	1	0	1	0	0	0	0	1	0	0
**Photobacterium**	2	119	1	0	0	0	0	0	0	0	1	0	0	0	0	0	0	1	0	0
**Photorhabdus**	2	149	0	0	0	0	0	0	0	0	0	1	0	0	0	0	0	1	0	0
**Phycisphaera**	1	9	0	0	0	0	0	0	0	0	0	0	0	0	0	0	0	0	0	0
**Phyllobacterium**	1	6	0	0	0	0	0	0	0	0	0	0	0	0	0	0	0	0	0	0
**Picrophilus**	1	15	0	0	0	0	0	0	0	0	0	0	0	0	0	0	0	0	0	0
**Pirellula**	2	85	0	0	0	0	0	0	0	0	1	0	1	0	0	0	0	1	0	0
**Planctomyces**	2	93	0	0	0	0	0	0	0	0	1	0	1	0	0	0	0	1	0	0
**Plautia**	1	17	0	0	0	0	0	0	0	0	0	0	0	0	0	0	0	1	0	0
**Plesiomonas**	1	4	0	0	0	0	0	0	0	0	0	0	0	0	0	0	0	0	0	0
**Pleurocapsa**	1	23	0	0	0	0	0	0	0	0	0	0	0	0	0	0	0	0	0	0
**Polaribacter**	2	74	0	0	0	0	0	0	0	0	1	0	0	0	0	0	0	1	0	0
**Polaromonas**	3	238	0	1	0	0	1	1	0	1	0	0	1	1	1	1	0	1	0	0
**Polymorphum**	3	173	0	0	1	0	0	1	0	0	0	0	1	1	0	0	0	1	0	0
**Polynucleobacter**	1	19	0	0	0	0	0	0	0	0	0	0	0	0	0	0	0	0	0	0
**Ponticaulis**	1	1	0	0	0	0	0	0	0	0	0	0	0	0	0	0	0	0	0	0
**Porphyromonas**	2	63	0	0	0	0	0	0	0	0	0	0	0	0	0	0	0	1	0	0
**Prevotella**	1	65	0	0	0	0	0	0	0	0	0	0	0	0	0	0	0	1	0	0
**Prochlorococcus**	2	68	0	0	0	0	0	1	0	0	0	0	0	0	0	0	0	1	0	0
**Propionibacterium**	1	62	0	0	0	0	0	0	0	0	1	0	0	0	0	0	0	1	1	0
**Prosthecochloris**	1	16	0	0	0	0	0	0	0	0	0	0	0	0	0	0	0	0	0	0
**Proteobacteria**	1	19	0	0	0	0	0	0	0	0	0	0	1	0	0	0	0	0	0	0
**Proteus**	2	113	0	0	0	0	0	0	0	0	0	0	0	0	0	0	0	1	0	0
**Providencia**	1	38	0	0	0	0	0	0	0	0	0	0	0	0	0	0	0	0	0	0
**Pseudaminobacter**	1	10	0	0	0	0	0	0	0	0	0	0	0	0	0	0	0	0	0	0
**Pseudanabaena**	1	11	0	0	0	0	0	0	0	0	0	0	0	0	0	0	0	0	0	0
**Pseudoalteromonas**	2	188	1	1	0	0	0	1	0	0	1	0	0	0	1	0	0	1	0	1
**Pseudochrobactrum**	1	7	0	0	0	0	0	0	0	0	0	0	0	0	0	0	0	0	0	0
**Pseudogulbenkiania**	1	40	0	0	0	0	0	0	0	0	0	0	0	0	0	0	0	0	0	0
**pseudomallei**	3	131	0	0	0	0	0	0	0	1	0	0	0	1	1	0	0	1	0	0

Genus	Cluster Number	Protein Counts	4.2.1.80	4.2.1.83	4.2.1.84	4.99.1.7	5.1.2.2	5.2.1.2	5.3.3.1	5.3.3.4	5.3.99.-	5.4.99.-	5.5.1.1	5.5.1.2	6.2.1.-	6.2.1.25	6.2.1.32	6.3.5.2	K00002	K00055
Pseudomonas	3	381	1	0	1	0	1	1	0	1	1	1	1	1	1	0	1	1	1	1
Pseudonocardia	3	180	0	0	1	0	1	0	0	1	0	0	1	1	0	0	0	1	0	0
Pseudorhodobacter	1	4	0	0	0	0	0	0	0	0	0	0	0	0	0	0	0	0	0	0
Pseudovibrio	1	33	0	0	0	0	0	0	0	0	0	0	0	0	0	0	0	0	0	0
Pseudoxanthomonas	2	124	0	0	0	0	0	1	0	0	0	0	0	0	0	0	0	1	0	1
Psychrobacter	2	124	0	0	0	0	0	0	0	1	0	0	1	0	1	0	0	1	0	0
Psychroflexus	1	22	0	0	0	0	0	0	0	0	0	0	0	0	0	0	0	0	0	0
Psychromonas	2	110	1	0	0	0	0	0	0	0	0	0	0	0	0	0	0	1	0	0
Pusillimonas	3	111	0	0	0	0	0	1	0	0	0	0	1	1	0	0	0	1	0	0
Pyrobaculum	1	15	0	0	0	0	0	0	0	0	0	0	0	0	0	0	0	0	0	0
Pyrococcus	1	10	0	0	0	0	0	0	0	0	0	0	0	0	0	0	0	0	0	0
Pyrolobus	1	4	0	0	0	0	0	0	0	0	0	0	0	0	0	0	0	0	0	0
Rahnella	2	143	0	0	1	0	0	0	0	0	0	0	1	0	0	0	0	1	0	1
Ralstonia	3	332	1	1	0	0	1	1	0	1	1	0	1	1	1	1	0	1	1	0
Ramlibacter	1	35	0	0	0	0	0	0	0	0	0	0	0	0	0	0	0	0	0	0
Raoultella	2	139	0	0	0	0	0	0	0	0	0	0	1	0	0	0	0	1	0	0
Raphidiopsis	1	5	0	0	0	0	0	0	0	0	0	0	0	0	0	0	0	0	0	0
Reinekea	1	2	0	0	0	0	0	0	0	0	0	0	0	0	0	0	0	0	0	0
Renibacterium	2	67	0	0	0	0	0	0	0	0	1	0	0	0	0	0	0	1	0	0
Rhizobiaceae	1	3	0	0	0	0	0	0	0	0	0	0	0	0	0	0	0	0	0	0
Rhizobiales	1	6	0	0	0	0	0	0	0	0	0	0	0	0	0	0	0	0	0	0
Rhizobium	3	257	1	1	1	0	0	1	0	1	1	0	1	1	0	1	0	1	0	1
Rhizobium/Agrobacterium	2	87	0	0	0	0	0	1	0	0	1	0	0	0	0	1	0	1	0	0
Rhodanobacter	1	21	0	0	0	0	0	0	0	0	0	0	0	0	0	0	0	0	0	0
Rhodobacter	3	175	0	0	0	0	0	0	0	1	1	0	0	0	1	0	0	1	0	0
Rhodobacteraceae	1	21	0	0	0	0	0	0	0	0	0	0	0	0	0	0	0	0	0	0
Rhodobacterales	1	34	0	0	0	0	0	0	0	0	0	0	0	0	0	0	0	0	0	0
Rhodococcus	2	292	1	1	1	0	1	0	1	1	1	1	1	1	1	0	0	1	0	1
Rhodocyclaceae	1	7	0	0	0	0	0	0	0	0	0	0	0	0	0	0	0	0	0	0
Rhodoferax	3	144	0	1	0	0	0	0	0	0	0	0	0	1	0	1	0	1	0	0
Rhodomicrobium	2	103	0	0	1	0	0	0	0	0	0	0	1	0	0	1	0	1	0	0
Rhodopirellula	2	87	0	0	0	0	0	0	0	0	1	0	1	0	0	0	0	1	0	0
Rhodopseudomonas	3	246	0	1	1	0	0	1	0	0	0	0	1	1	0	1	0	1	0	0
Rhodospirillum	2	121	0	0	0	0	0	0	0	0	0	0	0	0	0	0	0	1	0	0
Rhodothermus	2	76	0	0	0	0	0	0	0	0	1	0	0	0	0	0	0	1	0	0
Rhodovulum	1	4	0	0	0	0	0	0	0	0	0	0	0	0	0	0	0	0	0	0
Richelia	1	2	0	0	0	0	0	0	0	0	0	0	0	0	0	0	0	0	0	0
Rickettsia	2	43	0	0	0	0	0	0	0	0	0	0	0	0	0	0	0	0	0	0
Rickettsiaceae	1	5	0	0	0	0	0	0	0	0	0	0	0	0	0	0	0	0	0	0
Riemerella	2	61	0	0	0	0	0	0	0	0	0	0	0	0	0	0	0	1	0	0
Rivularia	1	18	0	0	0	0	0	0	0	0	0	0	0	0	0	0	0	0	0	0
Robiginitalea	2	83	0	0	0	0	0	0	0	0	1	0	0	0	0	0	0	1	0	0
Roseburia	2	58	0	0	0	0	0	0	0	0	0	0	0	0	0	0	0	1	0	0
Roseibium	1	3	0	0	0	0	0	0	0	0	0	0	0	0	0	0	0	0	0	0
Roseiflexus	3	100	0	0	0	0	0	0	0	0	1	0	0	0	0	0	0	1	0	0
Roseobacter	2	149	0	0	1	0	0	0	0	1	1	0	0	1	0	0	0	1	0	0
Roseomonas	1	2	0	0	0	0	0	0	0	0	0	0	0	0	0	0	0	0	0	0
Roseovarius	1	15	0	0	0	0	0	0	0	0	0	0	0	0	0	0	0	0	0	0
Rothia	1	44	0	0	0	0	0	0	0	0	0	0	0	0	0	0	0	1	0	0
Rubrivivax	1	24	0	0	0	0	0	0	0	0	0	0	0	0	0	0	0	0	0	0
Rubrobacter	3	117	0	0	1	0	0	0	0	0	1	0	0	1	0	1	0	1	0	1
Ruegeria	2	189	1	0	1	0	1	1	0	1	0	0	1	1	0	1	0	1	0	0

Genus	Cluster Number	Protein Counts	4.2.1.80	4.2.1.83	4.2.1.84	4.99.1.7	5.1.2.2	5.2.1.2	5.3.3.1	5.3.3.4	5.3.99.-	5.4.99.-	5.5.1.1	5.5.1.2	6.2.1.-	6.2.1.25	6.2.1.32	6.3.5.2	K00002	K00055
Ruminococcaceae	1	2	0	0	0	0	0	0	0	0	0	0	0	0	0	0	0	0	0	0
Ruminococcus	2	101	0	0	0	0	0	0	0	0	1	0	0	0	0	0	0	1	0	0
Runella	1	26	0	0	0	0	0	0	0	0	0	0	0	0	0	0	0	0	0	0
Saccharomonospora	2	132	0	0	0	0	0	0	0	1	1	0	0	1	0	0	0	1	0	1
Saccharophagus	2	81	0	0	0	0	0	1	0	0	1	0	0	0	0	0	0	1	0	0
Saccharopolyspora	3	194	0	0	0	0	0	0	1	1	1	0	1	1	0	0	0	1	1	0
Saccharothrix	1	32	0	0	0	0	0	0	0	0	0	0	0	0	0	0	0	0	0	0
Sagittula	1	10	0	0	0	0	0	0	0	0	0	0	0	0	0	0	0	0	0	0
Salinibacter	2	77	0	0	0	0	0	0	0	0	1	0	0	0	0	0	0	1	1	0
Salinimonas	1	1	0	0	0	0	0	0	0	0	0	0	0	0	0	0	0	0	0	0
Salinispora	3	127	1	0	0	0	0	0	0	0	0	0	0	1	0	0	0	1	0	0
Salinivibrio	1	2	0	0	0	0	0	0	0	0	0	0	0	0	0	0	0	0	0	0
Salmonella	2	151	0	0	0	0	0	1	0	0	1	0	0	0	0	0	0	1	0	0
Sanguibacter	2	73	0	0	0	0	0	0	0	0	1	0	0	0	0	0	0	1	0	0
Saprospira	1	13	0	0	0	0	0	0	0	0	0	0	0	0	0	0	0	0	0	0
Scardovia	1	1	0	0	0	0	0	0	0	0	0	0	0	0	0	0	0	0	0	0
Scytonema	1	10	0	0	0	0	0	0	0	0	0	0	0	0	0	0	0	0	0	0
Sebaldella	2	69	0	0	0	0	0	0	0	0	1	0	0	0	0	0	0	1	0	0
Segniliparus	2	85	0	0	0	0	0	0	0	0	0	0	0	0	0	0	0	1	0	0
Selenomonas	2	57	0	0	0	0	0	0	0	0	0	0	0	0	0	0	0	1	0	0
Serinicoccus	1	1	0	0	0	0	0	0	0	0	0	0	0	0	0	0	0	0	0	0
Serratia	2	186	0	0	1	0	0	0	0	0	1	0	0	1	0	0	0	1	0	1
Shewanella	3	214	1	0	0	1	0	1	1	0	1	0	0	0	0	0	0	1	0	0
Shigella	2	172	1	0	0	0	0	1	0	0	0	0	0	0	0	0	0	1	0	0
Shinella	1	2	0	0	0	0	0	0	0	0	0	0	0	0	0	0	0	0	0	0
Sideroxydans	2	81	0	0	0	0	0	0	0	0	0	0	0	0	0	0	0	1	0	0
Silicibacter	2	105	0	0	0	0	0	1	0	1	0	0	1	0	0	0	0	0	0	0
Simiduia	1	18	0	0	0	0	0	0	0	0	0	0	0	0	0	0	0	0	0	0
Simkania	1	6	0	0	0	0	0	0	0	0	0	0	0	0	0	0	0	0	0	0
Simonsiella	1	2	0	0	0	0	0	0	0	0	0	0	0	0	0	0	0	1	0	0
Singulisphaera	1	18	0	0	0	0	0	0	0	0	0	0	0	0	0	0	0	0	0	0
Sinorhizobium	3	207	0	1	1	0	0	1	0	0	1	0	1	1	0	0	0	1	0	1
Slackia	2	46	0	0	0	0	0	0	0	0	0	0	0	0	0	0	0	1	0	0
Smaragdicoccus	1	3	0	0	0	0	0	0	0	0	0	0	0	0	0	0	0	0	0	0
Sodalis	2	71	0	0	0	0	0	0	0	0	0	0	0	0	0	0	0	1	0	0
Solibacillus	1	23	0	0	0	0	0	0	0	0	0	0	0	0	0	0	0	0	0	1
Solitalea	1	13	0	0	0	0	0	0	0	0	0	0	0	0	0	0	0	0	0	0
Sorangium	3	115	0	0	0	0	1	1	0	0	0	0	0	0	0	1	0	1	0	0
Sphaerobacter	2	100	0	0	0	0	0	0	0	0	0	0	0	1	0	0	0	1	0	0
Sphaerochaeta	1	42	0	0	0	0	0	0	0	0	1	0	0	0	0	0	0	1	0	0
Sphingobacterium	3	85	0	0	0	0	0	0	0	0	1	0	1	0	0	0	0	1	0	0
Sphingobium	1	179	0	1	0	0	0	0	0	1	0	0	1	0	0	0	0	1	0	1
Sphingomonadaceae	1	15	0	0	0	0	0	0	0	0	0	0	0	0	0	0	0	0	0	1
Sphingomonas	2	220	1	1	0	1	0	1	0	1	1	0	1	1	0	1	0	1	0	1
Sphingopyxis	3	99	0	1	0	0	0	0	0	0	0	0	1	0	0	0	0	1	0	0
Spirochaeta	2	103	0	0	0	0	0	0	0	0	1	0	1	0	0	0	0	1	0	0
Spiroplasma	1	6	0	0	0	0	0	0	0	0	0	0	0	0	0	0	0	0	0	0
Spirosoma	2	107	0	0	0	0	0	0	0	0	1	0	0	1	1	0	0	1	0	0
Stackebrandtia	2	110	0	0	0	0	0	0	0	0	1	0	1	1	0	0	0	1	0	0
Stanieria	1	17	0	0	0	0	0	0	0	0	0	0	0	0	0	0	0	0	0	0
Staphylococcus	2	108	0	0	0	0	0	0	0	0	1	0	0	0	0	0	0	1	0	0
Staphylothermus	1	12	0	0	0	0	0	0	0	0	0	0	0	0	0	0	0	0	0	0

Genus	Cluster Number	Protein Counts	4.2.1.80	4.2.1.83	4.2.1.84	4.99.1.7	5.1.2.2	5.2.1.2	5.3.3.1	5.3.3.4	5.3.99.-	5.4.99.-	5.5.1.1	5.5.1.2	6.2.1.-	6.2.1.25	6.2.1.32	6.3.5.2	K00002	K00055
Stappia	1	2	0	0	0	0	0	0	0	0	0	0	0	0	0	0	0	0	0	0
Starkeya	3	134	0	0	0	0	0	1	0	0	1	0	1	0	0	0	0	1	0	0
Stenotrophomonas	3	104	0	0	0	0	0	0	0	0	1	0	0	0	1	0	0	1	0	1
Stigmatella	3	129	0	0	0	0	0	1	0	0	1	0	1	1	0	0	0	1	0	0
Streptobacillus	1	38	0	0	0	0	0	0	0	0	0	0	0	0	0	0	0	1	0	0
Streptococcus	2	121	0	0	0	0	0	0	0	0	1	0	0	0	0	0	0	1	0	0
Streptomyces	2	253	0	0	1	0	1	0	0	1	1	0	1	1	0	0	0	1	0	1
Streptosporangium	3	148	0	0	0	0	0	0	0	0	1	0	0	1	0	0	0	1	0	0
Strigomonas	1	2	0	0	0	0	0	0	0	0	0	0	0	0	0	0	0	0	0	0
Sulfitobacter	1	10	0	0	0	0	0	0	0	0	0	0	0	0	0	0	0	0	0	0
Sulfobacillus	1	30	0	0	0	0	0	0	0	0	0	0	0	0	0	0	0	0	0	0
Sulfolobus	1	23	0	0	0	0	0	0	0	0	0	0	0	0	0	0	0	0	0	0
Sulfuricurvum	2	67	0	0	0	0	0	0	0	0	0	0	0	0	0	0	0	1	0	0
Sulfurihydrogenibium	2	55	0	0	0	0	0	0	0	0	0	0	0	0	0	0	0	1	0	0
Sulfurimonas	2	60	0	0	0	0	0	0	0	0	0	0	0	0	0	0	0	1	0	0
Sulfurospirillum	1	48	1	0	0	0	0	0	0	0	0	0	0	0	0	0	0	1	0	0
Sulfurovum	2	48	1	0	0	0	0	0	0	0	0	0	0	0	0	0	0	1	0	0
Symbiobacterium	2	68	0	0	0	0	0	0	0	0	0	0	0	0	0	0	0	1	0	0
Synechococcus	1	34	0	0	0	0	0	0	0	0	0	0	0	0	0	0	0	0	1	0
Synechocystis	1	40	0	0	0	0	0	0	0	0	0	0	0	0	0	0	0	0	1	0
Synergistetes	1	27	0	0	0	0	0	0	0	0	0	0	1	0	0	0	0	0	0	0
Syntrophobacter	2	101	0	0	0	0	0	0	0	0	0	0	0	0	0	0	0	1	0	1
Syntrophobotulus	2	71	0	0	0	0	0	0	0	0	0	0	0	0	0	0	0	1	0	0
Syntrophomonas	2	58	0	0	0	0	0	0	0	0	0	0	0	0	0	0	0	1	0	0
Syntrophothermus	2	60	0	0	0	0	0	0	0	0	0	0	0	0	0	0	0	1	0	0
Syntrophus	2	66	0	0	0	0	0	0	0	0	0	0	0	0	0	0	0	1	0	0
Tannerella	1	8	0	0	0	0	0	0	0	0	0	0	0	0	0	0	0	0	0	0
Taylorella	1	43	0	0	0	0	0	0	0	0	0	0	0	0	0	0	0	1	0	0
Tepidanaerobacter	2	55	0	0	0	0	0	0	0	0	1	0	0	0	0	0	0	1	0	0
Teredinibacter	2	109	0	0	0	0	0	0	0	0	0	0	1	0	0	0	0	1	0	0
Terrabacter	1	1	0	0	0	0	0	0	0	0	0	0	0	0	0	0	0	0	0	0
Terriglobus	2	83	0	0	0	0	0	0	0	0	1	0	0	0	0	0	0	1	0	0
Tetragenococcus	1	12	0	0	0	0	0	0	0	0	0	0	0	0	0	0	0	0	0	0
Thalassiobium	1	11	0	0	0	0	0	0	0	0	0	0	0	0	0	0	0	0	0	0
Thalassobacter	1	9	0	0	0	0	0	0	0	0	0	0	0	0	0	0	0	0	0	0
Thalassolituus	1	12	0	0	0	0	0	0	0	0	0	0	0	0	0	0	0	0	0	0
Thalassospira	1	2	0	0	0	0	0	0	0	0	0	0	0	0	0	0	0	0	0	0
Thauera	1	111	0	1	0	0	0	0	0	0	0	0	0	0	0	1	0	1	0	0
Thermacetogenium	1	7	0	0	0	0	0	0	0	0	0	0	0	0	0	0	0	0	0	0
Thermaerobacter	2	79	0	0	0	0	0	0	0	0	0	0	0	0	0	0	0	1	0	0
Thermanaerovibrio	2	58	0	0	0	0	0	0	0	0	0	0	0	0	0	0	0	1	0	0
Thermincola	2	62	0	0	0	0	0	0	0	0	0	0	0	0	0	0	0	1	0	0
Thermoanaerobacter	2	76	0	0	0	0	0	0	0	0	1	0	1	0	0	0	0	1	0	0
Thermoanaerobacterium	2	64	0	0	0	0	0	0	0	0	1	0	0	0	0	0	0	1	0	0
Thermobacillus	1	11	0	0	0	0	0	0	0	0	0	0	0	0	0	0	0	0	0	0
Thermobaculum	2	62	0	0	0	0	0	0	0	0	1	0	1	0	0	0	0	1	0	0
Thermobifida	2	72	0	0	0	0	0	0	0	0	0	0	0	0	0	0	0	1	0	0
Thermobispora	2	109	0	0	0	0	0	0	0	0	1	0	0	1	0	0	0	1	0	0
Thermococcus	1	13	0	0	0	0	0	0	0	0	0	0	0	0	0	0	0	0	0	0
Thermocrinis	2	60	0	0	0	0	0	0	0	0	0	0	0	0	0	0	0	1	0	0
Thermodesulfatator	1	10	0	0	0	0	0	0	0	0	0	0	0	0	0	0	0	0	0	0
Thermodesulfobacterium	1	9	0	0	0	0	0	0	0	0	0	0	0	0	0	0	0	0	0	0

Genus	Cluster Number	Protein Counts	4.2.1.80	4.2.1.83	4.2.1.84	4.99.1.7	5.1.2.2	5.2.1.2	5.3.3.1	5.3.3.4	5.3.99.-	5.4.99.-	5.5.1.1	5.5.1.2	6.2.1.-	6.2.1.25	6.2.1.32	6.3.5.2	K00002	K00055
Thermodesulfobium	1	13	0	0	0	0	0	0	0	0	0	0	0	0	0	0	0	0	0	0
Thermodesulfovibrio	2	60	0	0	0	0	0	0	0	0	0	0	0	0	0	0	0	1	0	0
Thermofilum	1	9	0	0	0	0	0	0	0	0	0	0	0	0	0	0	0	0	0	0
Thermogladius	1	7	0	0	0	0	0	0	0	0	0	0	0	0	0	0	0	0	0	0
Thermomicrobium	2	92	0	0	0	0	0	0	0	0	0	0	0	0	0	0	0	1	0	0
Thermomonospora	2	125	0	0	0	0	0	0	0	0	0	0	0	1	0	0	0	1	0	1
Thermoplasma	1	11	0	0	0	0	0	0	0	0	0	0	0	0	0	0	0	0	0	0
Thermoplasmatales	1	6	0	0	0	0	0	0	0	0	0	0	0	0	0	0	0	0	0	0
Thermoproteus	1	12	0	0	0	0	0	0	0	0	0	0	0	0	0	0	0	0	0	0
Thermosediminibacter	2	64	0	0	0	0	0	0	0	0	0	0	0	0	0	0	0	1	0	0
Thermosipho	2	70	0	0	0	0	0	0	0	0	0	0	0	0	0	0	0	1	0	0
Thermosphaera	1	10	0	0	0	0	0	0	0	0	0	0	0	0	0	0	0	0	0	0
Thermosynechococcus	1	6	0	0	0	0	0	0	0	0	0	0	0	0	0	0	0	0	0	0
Thermotoga	2	80	0	0	0	0	0	0	0	0	1	0	1	0	0	0	0	1	0	0
Thermovibrio	2	58	0	0	0	0	0	0	0	0	0	0	0	0	0	0	0	1	0	0
Thermovirga	1	9	0	0	0	0	0	0	0	0	0	0	0	0	0	0	0	0	0	0
Thermus	2	107	0	0	0	0	0	0	0	0	0	0	1	0	0	0	0	1	0	0
Thioalkalimicrobium	1	4	0	0	0	0	0	0	0	0	0	0	0	0	0	0	0	0	0	0
Thioalkalivibrio	2	91	0	0	0	0	0	0	0	0	0	0	0	0	0	0	0	1	0	0
Thiobaca	1	2	0	0	0	0	0	0	0	0	0	0	0	0	0	0	0	0	0	0
Thiobacillus	2	67	0	0	0	0	0	0	0	0	0	0	0	0	0	0	0	1	0	0
Thiocapsa	1	2	0	0	0	0	0	0	0	0	0	0	0	0	0	0	0	0	0	0
Thiocystis	1	11	0	0	0	0	0	0	0	0	0	0	0	0	0	0	0	0	0	0
Thioflavicoccus	1	13	0	0	0	0	0	0	0	0	0	0	0	0	0	0	0	0	0	0
Thiomicrospira	2	50	0	0	0	0	0	0	0	0	0	0	0	0	0	0	0	1	0	0
Thiomonas	2	85	0	0	0	0	0	0	0	0	0	0	0	0	0	0	0	1	0	0
Thiorhodococcus	1	2	0	0	0	0	0	0	0	0	0	0	0	0	0	0	0	0	0	0
Thiothrix	1	2	0	0	0	0	0	0	0	0	0	0	0	0	0	0	0	0	0	0
Tistrella	1	42	0	0	0	0	0	0	0	0	0	0	0	0	0	0	0	0	0	0
Tolumonas	2	100	0	0	0	0	0	0	0	0	0	0	1	0	0	0	0	1	0	0
Transposon	1	2	0	0	0	0	0	0	0	0	0	0	0	0	0	0	0	0	0	0
Treponema	1	63	0	0	0	0	0	0	0	0	0	0	0	0	0	0	0	1	0	0
Trichodesmium	2	71	0	0	0	0	0	1	0	0	0	0	0	0	0	0	0	1	0	0
Trichormus	1	12	0	0	0	0	0	0	0	0	0	0	0	0	0	0	0	1	0	0
Tropheryma	2	22	0	0	0	0	0	0	0	0	0	0	0	0	0	0	0	1	0	0
Truepera	2	104	0	0	0	0	0	1	0	0	1	0	1	0	0	0	0	1	0	0
Tsukamurella	2	123	0	0	0	0	0	0	0	0	0	0	0	1	0	0	0	1	0	1
Turneriella	1	16	0	0	0	0	0	0	0	0	0	0	0	0	0	0	0	0	0	0
Uliginosibacterium	1	2	0	0	0	0	0	0	0	0	0	0	0	0	0	0	0	0	0	0
Ureaplasma	1	24	0	0	0	0	0	0	0	0	0	0	0	0	0	0	0	0	0	0
Ureibacillus	1	2	0	0	0	0	0	0	0	0	0	0	0	0	0	0	0	0	0	0
Variovorax	3	187	0	1	1	0	0	0	0	1	1	0	1	1	0	1	0	1	0	0
Veillonella	1	54	0	0	0	0	0	0	0	0	0	0	0	0	0	0	0	1	0	0
Verminephrobacter	3	188	0	1	0	0	1	0	0	1	1	0	0	1	0	1	0	1	0	0
Verrucosispora	3	126	0	0	0	0	0	0	0	0	0	0	1	1	0	0	0	1	0	0
Vibrio	2	195	1	0	0	0	0	1	0	0	0	0	1	0	0	0	0	1	0	0
Vibrionales	1	46	0	0	0	0	0	0	0	0	0	0	0	0	0	0	0	1	0	0
Vitreoscilla	1	1	0	0	0	0	0	0	0	0	0	0	0	0	0	0	0	0	0	0
Vulcanisaeta	1	14	0	0	0	0	0	0	0	0	0	0	0	0	0	0	0	0	0	0
Waddlia	2	68	0	0	0	0	0	0	0	0	0	0	0	0	0	0	0	1	0	0
Wautersia	1	2	0	0	0	0	0	0	0	0	0	0	0	0	0	0	0	0	0	0
Weeksella	2	65	0	1	0	0	0	0	0	0	0	0	0	0	0	0	0	1	0	0

Genus	Cluster Number	Protein Counts	4.2.1.80	4.2.1.83	4.2.1.84	4.99.1.7	5.1.2.2	5.2.1.2	5.3.3.1	5.3.3.4	5.3.99.-	5.4.99.-	5.5.1.1	5.5.1.2	6.2.1.-	6.2.1.25	6.2.1.32	6.3.5.2	K00002	K00055
Weissella	1	14	0	0	0	0	0	0	0	0	0	0	0	0	0	0	0	0	0	0
Wenxinia	1	2	0	0	0	0	0	0	0	0	0	0	0	0	0	0	0	0	0	0
Wigglesworthia	1	29	0	0	0	0	0	0	0	0	0	0	0	0	0	0	0	1	0	0
Wolbachia	1	32	0	0	0	0	0	0	0	0	0	0	0	0	0	0	0	1	0	0
Wolinella	2	51	0	0	0	0	0	0	0	0	0	0	0	0	0	0	0	1	0	0
Xanthobacter	3	176	0	0	0	0	0	0	0	1	0	0	1	1	0	0	0	1	0	0
Xanthobacteraceae	1	4	0	0	0	0	0	0	0	0	0	0	0	0	0	0	0	0	0	0
Xanthomonadaceae	1	39	0	0	0	0	0	0	0	0	0	0	0	0	0	0	0	1	0	0
Xanthomonas	2	162	1	1	0	0	0	1	0	0	1	0	0	1	0	0	0	1	0	0
Xenorhabdus	2	127	0	0	0	0	0	0	0	0	0	0	0	0	0	0	0	1	0	0
Xylanimonas	2	66	0	0	0	0	0	0	0	0	1	0	0	0	0	0	0	1	0	0
Xylella	2	52	0	0	0	0	0	0	0	0	0	0	0	0	0	0	0	1	1	0
Yersinia	2	145	0	0	0	0	0	0	0	0	0	0	0	0	0	0	0	1	0	0
Yokenella	2	61	0	0	0	0	0	0	0	0	0	0	0	0	0	0	0	1	0	0
Zobellia	1	28	0	0	0	0	0	0	0	0	1	0	0	0	0	0	0	0	0	0
Zunongwangia	2	98	0	0	0	0	0	0	0	0	1	0	1	0	0	0	0	1	1	0
Zymomonas	2	67	0	0	0	0	0	0	0	0	0	0	0	0	0	0	0	1	0	0
Zymophilus	1	2	0	0	0	0	0	0	0	0	0	0	0	0	0	0	0	0	0	0

Genus	Cluster Number	Protein Counts	K00074	K00088	K00100	K00106	K00114	K00128	K00129	K00132	K00141	K00146	K00148	K00152	K00155	K00169	K00217	K00224	K00274	K00446
Acaricomes	1	2	0	0	0	0	0	0	0	0	0	0	0	0	0	0	0	0	0	0
Acaryochloris	2	88	0	1	0	0	0	1	0	0	0	0	0	0	0	0	0	0	0	0
Acetivibrio	1	2	0	0	0	0	0	0	0	0	0	0	0	0	0	0	0	0	0	0
Acetobacter	2	85	0	1	1	0	1	1	0	0	0	1	0	0	0	0	0	0	0	0
Acetobacteraceae	1	2	0	0	0	0	0	0	0	0	0	0	0	0	0	0	0	0	0	0
Acetobacterium	1	8	0	1	0	0	0	0	0	0	0	0	0	0	0	0	0	0	0	0
Acetohalobium	2	79	0	1	0	0	0	0	0	0	0	0	0	0	0	1	0	0	0	0
Acholeplasma	2	38	0	0	0	0	0	1	0	0	0	0	0	0	0	0	0	0	0	0
Achromobacter	2	192	1	1	0	0	0	1	0	0	0	1	1	0	0	0	0	0	0	0
Acidaminococcus	1	65	1	1	0	0	0	0	0	0	0	0	0	0	0	0	0	0	0	0
Acidianus	1	9	1	0	0	0	0	0	0	0	0	0	0	0	0	1	0	0	0	0
Acidilobus	1	8	0	1	0	0	0	0	0	0	0	0	0	0	0	1	0	0	0	0
Acidimicrobium	2	58	1	1	0	0	0	0	0	0	0	0	0	0	0	0	0	0	0	0
Acidiphilium	3	144	1	1	1	0	1	1	0	0	0	0	0	0	1	0	0	0	1	0
Acidithiobacillus	2	70	0	1	0	0	0	1	0	0	0	0	0	0	0	0	0	0	1	0
Acidobacterium	2	91	1	1	0	0	0	1	0	0	0	0	0	0	0	0	0	0	0	0
Acidocella	1	2	0	0	0	0	0	0	0	0	0	0	0	0	0	0	0	0	0	0
Acidothermus	2	68	1	1	0	0	0	1	0	0	0	0	0	0	0	1	0	0	0	0
Acidovorax	3	217	1	1	1	0	0	1	0	0	1	0	0	0	0	0	0	0	0	1
Aciduliprofundum	1	11	0	1	0	0	0	0	0	0	0	0	0	0	0	1	0	0	0	0
Acinetobacter	3	188	1	1	1	0	0	1	0	0	1	1	0	0	1	0	0	0	0	0
Actinobacillus	2	112	0	1	1	0	0	0	0	0	0	0	0	0	0	0	0	0	0	0
Actinomadura	1	1	0	0	0	0	0	0	0	0	0	0	0	0	0	0	0	0	0	0
Actinoplanes	1	52	1	1	0	0	0	0	0	0	1	0	1	0	0	0	0	0	1	0
Actinopolyspora	1	2	0	0	0	0	0	0	0	0	0	0	0	0	0	0	0	0	0	0
Actinosynnema	2	122	1	1	0	0	0	1	0	0	1	0	0	0	0	0	0	0	0	0
Advenella	1	30	1	0	0	0	0	1	0	0	0	0	0	0	0	0	0	0	0	0
Aequorivita	1	12	1	1	0	0	0	0	0	0	0	0	0	0	0	0	0	0	0	0
Aerococcus	2	57	1	1	0	0	0	1	0	0	0	0	0	0	0	0	0	0	0	0
Aeromonas	2	127	0	1	0	0	0	1	0	0	0	0	0	0	0	0	0	0	0	0
Aeropyrum	1	13	0	1	0	0	0	0	0	0	0	0	0	0	0	0	0	0	0	0
Afipia	1	54	1	1	1	0	0	1	0	0	0	0	0	0	0	0	0	0	0	0
Aggregatibacter	1	81	0	1	0	0	0	0	0	0	0	0	0	0	0	0	0	0	0	0
Agrobacterium	2	242	1	1	1	1	0	1	0	0	0	1	1	0	1	0	0	0	1	1
Ahrensia	1	2	0	0	0	0	0	0	0	0	0	0	0	0	0	0	0	0	0	0
Akkermansia	2	47	0	1	1	0	0	0	0	0	0	0	0	0	0	0	0	0	0	0
Albidiferax	3	144	1	1	0	0	0	1	0	0	0	0	0	0	0	1	0	0	0	0
Alcaligenes	1	21	0	0	0	0	0	0	0	0	0	0	1	0	0	0	0	0	0	0
Alcanivorax	2	146	0	1	1	0	1	1	0	0	0	1	1	0	1	0	0	0	0	0
Alicycliphilus	3	152	1	1	0	0	0	1	0	0	0	1	0	0	0	0	0	0	0	1
Alicyclobacillus	2	91	1	1	1	0	0	1	0	0	0	1	0	0	0	0	0	0	0	1
Aliivibrio	2	118	0	1	1	0	0	1	0	0	0	0	0	0	0	0	0	0	1	0
Alishewanella	1	1	0	0	0	0	0	0	0	0	0	0	0	0	0	0	0	0	0	0
Alistipes	1	52	0	1	0	0	0	0	0	0	0	0	0	0	0	0	0	0	0	0
Alkalilimnicola	2	97	0	1	0	0	1	1	0	0	0	0	0	0	0	0	0	0	0	0
Alkaliphilus	2	62	1	1	1	0	0	1	0	1	0	0	0	0	0	0	0	0	0	0
Allochromatium	2	84	0	1	0	0	0	0	0	0	0	0	0	0	0	0	0	0	0	0
Alphaproteobacteria	1	3	0	0	0	0	0	0	0	0	0	0	0	0	0	0	0	0	0	0
Alteromonadales	1	12	0	0	0	0	0	0	0	0	0	0	0	0	0	0	0	0	0	0
Alteromonas	1	37	0	1	0	0	1	1	0	0	0	0	0	0	0	0	0	0	0	0
Aminobacter	1	4	0	0	0	0	0	0	0	0	0	0	0	0	0	0	0	0	0	0
Aminobacterium	2	59	0	1	0	0	0	0	0	0	0	0	0	0	0	1	0	0	0	0

Genus	Cluster Number	Protein Counts	K00074	K00088	K00100	K00106	K00114	K00128	K00129	K00132	K00141	K00146	K00148	K00152	K00155	K00169	K00217	K00224	K00274	K00446
**Ammonifex**	2	50	0	1	0	0	0	0	0	0	0	0	0	0	0	0	0	0	0	0
**Amphibacillus**	1	13	0	1	0	0	0	1	0	0	0	0	0	0	0	0	0	0	0	0
**Amphritea**	1	2	0	0	0	0	0	0	0	0	0	0	0	0	0	0	0	0	0	0
**Amycolatopsis**	2	201	1	1	0	0	0	1	0	0	1	0	1	0	0	0	0	0	1	1
**Amycolicicoccus**	1	43	1	1	0	0	0	1	0	0	0	0	0	0	0	0	0	0	1	0
**Anabaena**	2	92	0	1	0	0	0	1	1	0	0	0	0	0	0	0	0	0	0	0
**Anaerobaculum**	1	11	0	1	0	0	0	0	0	0	0	0	0	0	0	1	0	0	0	0
**Anaerococcus**	2	46	1	1	0	0	0	1	0	0	0	0	0	0	0	0	0	0	0	0
**Anaerolinea**	2	66	0	1	0	0	0	0	0	0	0	0	0	0	0	0	0	0	0	0
**Anaeromyxobacter**	3	117	1	1	0	0	0	0	1	0	0	0	1	0	0	0	0	0	0	0
**Anaerostipes**	1	44	1	1	0	0	0	0	0	0	0	0	0	0	0	1	0	0	0	0
**Anaerotruncus**	1	2	0	0	0	0	0	0	0	0	0	0	0	0	0	0	0	0	0	0
**Anaplasma**	1	35	0	1	0	0	0	0	0	0	0	0	0	0	0	0	0	0	0	0
**Ancylobacter**	1	3	0	0	0	0	0	0	0	0	0	0	0	0	0	0	0	0	0	0
**Aneurinibacillus**	1	1	0	0	0	0	0	0	0	0	0	0	0	0	0	0	0	0	0	0
**Angomonas**	1	1	0	1	0	0	0	0	0	0	0	0	0	0	0	0	0	0	0	0
**Anoxybacillus**	2	72	1	1	1	0	0	1	0	0	0	0	0	0	0	0	0	0	0	0
**Aquifex**	2	45	0	1	0	0	0	0	0	0	0	0	0	0	0	1	0	0	0	0
**Aquimarina**	1	2	0	0	0	0	0	0	0	0	0	0	0	0	0	0	0	0	0	0
**Arcanobacterium**	2	45	0	1	0	0	0	0	0	0	0	0	0	0	0	0	0	0	0	0
**Archaeoglobus**	1	14	1	1	0	0	0	0	0	0	0	0	0	0	0	1	0	0	0	0
**Arcobacter**	2	101	1	1	1	0	1	1	0	0	0	0	0	0	0	1	0	0	0	1
**Aromatoleum**	3	133	1	1	0	0	0	1	0	0	1	0	0	0	0	1	0	0	0	0
**Arsenophonus**	1	4	0	0	0	0	0	0	0	0	0	0	0	0	0	0	0	0	0	0
**Arthrobacter**	2	206	1	1	0	0	0	1	0	0	0	1	1	0	0	0	0	0	0	1
**Arthrospira**	2	67	0	1	0	0	0	0	0	0	0	0	0	0	0	0	0	0	0	0
**Aster**	1	10	0	0	0	0	0	0	0	0	0	0	0	0	0	0	0	0	0	0
**Asticcacaulis**	2	103	1	1	0	0	0	1	0	0	0	0	0	0	0	0	0	0	0	0
**Atopobium**	1	28	1	1	0	0	0	0	0	0	0	0	0	0	0	0	0	0	0	0
**Aurantimonas**	1	2	0	0	0	0	0	0	0	0	0	0	0	0	0	0	0	0	0	0
**Avibacterium**	1	17	0	1	0	0	0	0	0	0	0	0	0	0	0	0	0	0	0	0
**Azoarcus**	3	172	1	1	1	0	1	1	0	0	1	1	1	0	1	1	0	0	1	1
**Azorhizobium**	3	149	1	1	0	0	0	1	0	0	0	0	0	0	0	0	0	0	1	0
**Azorhizophilus**	1	4	0	0	0	0	0	0	0	0	0	0	0	0	0	0	0	0	0	0
**Azospira**	1	19	1	1	1	0	0	1	0	0	0	0	0	0	0	0	0	0	0	0
**Azospirillum**	2	198	1	1	0	0	1	1	0	0	1	1	0	0	0	0	0	0	1	0
**Azotobacter**	3	183	1	1	0	0	0	1	0	0	0	0	0	0	0	0	0	0	1	1
**Bacillales**	1	9	0	1	0	0	0	0	0	0	0	0	0	0	0	0	0	0	0	1
**Bacilli**	1	3	0	0	0	0	0	0	0	0	0	0	0	0	0	0	0	0	0	0
**Bacillus**	2	237	1	1	1	0	0	1	0	0	0	1	1	0	0	1	0	0	1	1
**Bacteria**	2	86	1	1	0	0	0	1	0	0	0	0	0	0	0	0	0	0	0	0
**Bacteriovorax**	2	98	1	1	0	0	0	1	0	0	0	0	0	0	0	0	0	0	0	0
**Bacteroidales**	2	59	0	1	0	0	0	0	0	0	0	0	0	0	0	0	0	0	0	0
**Bacteroides**	2	96	0	1	1	0	0	0	0	0	0	0	0	0	0	0	0	0	0	0
**Bacteroidetes**	1	2	0	0	0	0	0	0	0	0	0	0	0	0	0	0	0	0	0	0
**Bartonella**	2	50	0	1	0	0	0	1	0	0	0	0	0	0	0	0	0	0	0	0
**Baumannia**	2	24	0	1	0	0	0	0	0	0	0	0	0	0	0	0	0	0	0	0
**Bdellovibrio**	2	87	1	1	1	0	0	1	0	0	0	0	0	0	1	0	0	0	1	0
**Beijerinckia**	3	123	1	1	0	0	1	1	0	0	0	0	0	0	0	0	0	0	1	0
**Belliella**	1	14	1	1	0	0	0	1	0	0	0	0	0	0	0	0	0	0	0	0
**Beutenbergia**	2	82	1	1	0	0	0	0	0	0	0	0	0	0	0	0	0	0	0	0
**Bifidobacterium**	2	61	1	1	1	0	0	1	0	0	0	0	0	0	0	0	0	0	0	0

Genus	Cluster Number	Protein Counts	K00074	K00088	K00100	K00106	K00114	K00128	K00129	K00132	K00141	K00146	K00148	K00152	K00155	K00169	K00217	K00224	K00274	K00446
Bizionia	1	1	0	0	0	0	0	1	0	0	0	0	0	0	0	0	0	0	0	0
Blastococcus	1	39	1	1	0	0	0	0	0	0	0	0	0	0	0	0	0	0	0	1
Blattabacterium	1	23	0	1	0	0	0	0	0	0	0	0	0	0	0	0	0	0	0	0
Blautia	1	22	0	0	0	0	0	0	0	0	0	0	0	0	0	0	0	0	0	0
Bordetella	3	227	1	1	0	0	0	1	0	0	1	0	0	0	0	0	1	0	1	1
Borrelia	1	32	0	1	0	0	0	0	0	0	0	0	0	0	0	0	0	0	0	0
Brachybacterium	1	18	1	1	0	0	0	1	0	0	0	0	0	0	0	0	0	0	0	0
Brachymonas	1	4	0	0	0	0	0	0	0	0	0	0	0	0	0	0	0	0	0	0
Brachyspira	2	79	1	1	1	0	0	1	0	0	0	0	0	0	0	1	0	0	0	0
Bradyrhizobiaceae	1	20	0	1	1	0	0	0	0	0	0	0	0	0	0	0	0	0	0	0
Bradyrhizobium	3	254	1	1	1	0	1	1	0	0	1	0	0	0	1	0	1	0	1	1
Brenneria	1	20	0	1	0	0	0	0	0	0	0	0	0	0	0	0	0	0	0	0
Brevibacillus	2	122	1	1	1	0	0	1	0	0	0	0	0	0	0	0	0	0	1	0
Brevibacterium	1	4	0	0	0	0	0	0	0	0	0	0	0	0	0	0	0	0	0	0
Brevundimonas	2	105	1	1	0	0	0	1	0	0	0	1	0	0	0	0	0	0	1	0
Brucella	3	138	1	1	1	0	0	1	0	0	0	0	0	0	1	0	0	0	0	1
Buchnera	1	26	0	0	0	0	0	0	0	0	0	0	0	0	0	0	0	0	0	0
Burkholderia	3	424	1	1	1	0	1	1	0	0	1	1	1	1	1	0	1	0	1	1
Burkholderiaceae	3	143	1	1	1	0	1	1	0	0	0	0	0	0	0	0	1	1	0	0
Burkholderiales	1	24	0	0	0	0	0	0	0	0	0	0	0	0	0	0	1	0	0	0
Buttiauxella	1	2	0	0	0	0	0	0	0	0	0	0	0	0	0	0	0	0	0	0
Butyrivibrio	2	67	1	1	0	0	0	1	0	0	0	0	0	0	0	0	0	0	0	0
Caldanaerobacter	2	63	1	1	1	0	0	0	0	0	0	0	0	0	0	0	0	0	0	0
Caldicellulosiruptor	2	75	0	1	0	0	0	0	0	0	0	0	0	0	0	1	0	0	0	0
Caldilinea	1	23	0	1	0	0	0	0	0	0	0	0	0	0	0	0	0	0	0	1
Caldimonas	1	6	0	0	0	0	0	0	0	0	0	0	0	0	0	0	0	0	0	0
Caldisericum	1	10	0	0	0	0	0	0	0	0	0	0	0	0	0	1	0	0	0	0
Caldisphaera	1	7	0	1	0	0	0	0	0	0	0	0	0	0	0	1	0	0	0	0
Calditerrivibrio	2	74	0	1	0	0	0	0	0	0	0	0	0	0	0	0	0	0	0	0
Caldivirga	1	13	0	1	0	0	0	1	0	0	0	0	0	0	0	1	0	0	0	0
Calothrix	1	43	0	1	0	0	0	1	0	0	0	0	0	0	0	0	0	0	1	0
Calyptogena	2	34	0	1	0	0	0	0	0	0	0	0	0	0	0	0	0	0	0	0
Campylobacter	2	80	0	1	1	0	0	0	0	0	0	0	0	0	0	0	0	1	0	0
Candidatus	2	290	1	1	1	0	1	1	0	0	0	1	1	0	0	1	0	0	1	0
Capnocytophaga	1	50	0	1	0	0	0	0	0	0	0	0	0	0	0	0	0	0	0	0
Carbophilus	1	2	0	0	0	0	0	0	0	0	0	0	0	0	0	0	0	0	0	0
Carboxydibrachium	1	14	0	1	0	0	0	0	0	0	0	0	0	0	0	0	0	0	0	0
Carboxydothermus	2	68	1	1	1	0	0	0	0	0	0	0	0	0	0	0	0	0	0	0
Carica	1	2	0	0	0	0	0	0	0	0	0	0	0	0	0	0	0	0	0	0
Carnobacterium	2	71	1	1	0	0	0	1	0	0	0	0	0	0	0	0	0	0	0	0
Catenibacterium	1	2	0	0	0	0	0	0	0	0	0	0	0	0	0	0	0	0	0	0
Catenovulum	1	2	0	0	0	0	0	0	0	0	0	0	0	0	0	0	0	0	0	0
Catenulispora	3	159	1	1	0	0	0	1	0	0	1	0	0	0	0	0	0	0	1	0
Caulobacter	3	168	1	1	0	0	0	1	0	0	1	0	0	0	0	0	0	0	1	0
Cedecea	1	45	0	1	0	0	0	0	0	0	0	0	0	0	0	0	0	0	0	0
Cellulomonas	2	96	0	1	0	0	0	0	0	0	0	0	0	0	0	0	0	0	0	0
Cellulophaga	2	105	1	1	0	0	0	1	0	0	0	0	0	0	0	0	0	0	1	0
Cellulosilyticum	1	14	0	1	1	0	0	1	0	0	0	0	0	0	0	1	0	0	0	0
Cellvibrio	2	85	0	1	0	0	0	0	0	0	0	0	0	0	0	0	0	0	0	0
Cenarchaeum	1	7	0	1	0	0	0	0	0	0	0	0	0	0	0	0	0	0	0	0
Chamaesiphon	1	13	0	1	0	0	0	0	0	0	0	0	0	0	0	0	0	0	1	0
Chelativorans	3	136	1	1	0	0	0	1	0	0	0	0	0	0	1	0	0	0	0	1

Genus	Cluster Number	Protein Counts	K00074	K00088	K00100	K00106	K00114	K00128	K00129	K00132	K00141	K00146	K00148	K00152	K00155	K00169	K00217	K00224	K00274	K00446
Chelatococcus	1	2	0	0	0	0	0	0	0	0	0	0	0	0	0	0	0	0	0	0
Chitinophaga	2	98	1	1	0	0	0	1	0	0	0	0	0	0	0	0	0	0	1	0
Chlamydia	1	36	0	1	0	0	0	0	0	0	0	0	0	0	0	0	0	0	0	0
Chlamydomonas	1	3	0	0	0	0	0	0	0	0	0	0	0	0	0	0	0	0	0	0
Chlamydophila	1	31	0	1	0	0	0	0	0	0	0	0	0	0	0	0	0	0	0	0
Chlorobaculum	2	61	0	1	1	0	0	1	0	0	0	0	0	0	0	0	0	0	0	0
Chlorobium	2	89	0	1	1	0	0	1	1	0	0	0	0	0	0	0	0	0	0	0
Chlorobium/Pelodictyon	1	2	0	0	0	0	0	0	0	0	0	0	0	0	0	0	0	0	0	0
Chloroflexus	2	105	0	1	0	0	0	1	0	0	0	0	0	0	0	0	0	0	1	1
Chlorogloeopsis	1	9	0	1	0	0	0	0	0	0	0	0	0	0	0	0	0	0	0	0
Chloroherpeton	2	59	1	1	0	0	0	0	0	0	0	0	0	0	0	0	0	0	0	0
Chromobacterium	2	117	1	1	0	0	0	1	0	0	0	0	0	0	0	0	0	0	0	0
Chromohalobacter	2	145	0	1	0	0	0	1	0	0	0	0	1	0	0	0	0	0	0	0
Chroococcidiopsis	1	24	0	1	0	0	0	1	0	0	0	0	0	0	0	0	0	0	1	0
Chryseobacterium	2	104	1	0	0	0	1	1	0	0	0	1	0	0	0	0	0	0	0	0
Citreicella	1	13	0	0	0	0	0	0	0	0	0	0	0	0	0	0	0	0	0	1
Citrobacter	2	190	1	1	0	0	0	0	0	0	0	0	0	0	0	0	0	0	0	0
Clavibacter	2	83	1	1	0	0	0	0	0	0	0	0	0	0	0	0	0	0	0	0
Clonorchis	1	1	0	0	0	0	0	0	0	0	0	0	0	0	0	0	0	0	0	0
Clostridiales	2	77	1	1	0	0	0	0	0	0	0	0	0	0	0	1	0	0	0	0
Clostridium	2	191	1	1	1	0	0	1	0	1	0	0	0	0	0	1	0	0	1	0
Cohnella	1	2	0	0	0	0	0	0	0	0	0	0	0	0	0	0	0	0	0	0
Coleofasciculus	1	4	0	0	0	0	0	0	0	0	0	0	0	0	0	0	0	0	0	0
Collimonas	1	35	0	1	0	0	0	1	0	0	0	0	1	0	0	0	0	0	0	0
Colwellia	2	130	0	1	0	0	1	1	0	0	0	0	1	0	1	0	0	0	0	0
Comamonas	2	196	1	1	0	0	1	1	0	0	0	0	0	0	0	0	0	0	1	1
Conexibacter	3	117	1	1	0	0	0	1	0	0	0	0	1	0	0	0	0	0	1	1
Coprobacillus	1	2	0	0	0	0	0	0	0	0	0	0	0	0	0	0	0	0	0	0
Coprococcus	2	76	1	1	0	0	0	0	0	0	0	0	0	0	0	1	0	0	0	0
Coprothermobacter	2	45	0	1	1	0	0	0	0	0	0	0	0	0	0	1	0	0	0	0
Coraliomargarita	2	68	0	1	0	0	0	0	0	0	0	0	0	0	0	0	0	0	0	0
Corallococcus	1	29	1	1	0	0	0	0	0	0	0	0	1	0	0	0	0	0	1	0
Coriobacterium	2	36	1	1	1	0	0	0	0	0	0	0	0	0	0	0	0	0	0	0
Corynebacterineae	1	5	0	0	0	0	0	0	0	0	0	0	0	0	0	0	0	0	0	0
Corynebacterium	2	175	1	1	1	0	0	1	0	0	0	1	1	0	0	0	1	0	0	1
Coxiella	2	54	0	1	0	0	0	0	0	0	0	0	0	0	0	0	0	0	0	0
Crinalium	1	21	0	1	0	0	0	1	0	0	0	0	0	0	0	0	0	0	0	0
Croceibacter	2	80	1	1	0	0	0	1	0	0	0	0	0	0	0	0	0	0	0	0
Crocosphaera	1	6	0	1	0	0	0	1	0	0	0	0	0	0	0	0	0	0	0	0
Cronobacter	2	120	0	1	0	0	0	1	0	0	0	0	0	0	0	0	0	0	0	0
Cryptobacterium	2	39	1	1	1	0	0	0	0	0	0	0	0	0	0	1	0	0	0	0
Cucumis	1	9	0	0	0	0	0	0	0	0	0	0	0	0	0	0	0	0	0	0
Cupriavidus	3	317	1	1	1	0	1	1	0	0	1	1	1	0	1	0	1	1	1	1
Curtobacterium	1	5	0	0	0	0	0	0	0	0	0	0	0	0	0	0	0	0	0	0
Curvibacter	1	27	0	1	0	0	0	1	0	0	0	0	0	0	0	0	0	0	0	0
Cyanobacterium	1	9	0	1	0	0	0	0	0	0	0	0	0	0	0	0	0	0	1	0
Cyanobium	1	10	0	1	0	0	0	0	0	0	0	0	0	0	0	0	0	0	1	0
Cyanothece	1	34	0	1	0	0	0	1	0	0	0	0	0	0	0	0	0	0	1	0
Cyclobacterium	1	19	1	1	0	0	0	1	0	0	0	0	0	0	0	0	0	0	0	0
Cycloclasticus	1	21	0	1	0	0	1	0	0	0	0	0	0	0	0	0	0	0	0	0
Cylindrospermopsis	1	4	0	0	0	0	0	0	0	0	0	0	0	0	0	0	0	0	0	0
Cylindrospermum	1	30	1	1	0	0	0	0	0	0	0	0	0	0	0	0	0	0	0	0

Genus	Cluster Number	Protein Counts	K00074	K00088	K00100	K00106	K00114	K00128	K00129	K00132	K00141	K00146	K00148	K00152	K00155	K00169	K00217	K00224	K00274	K00446
Cytophaga	2	69	1	1	1	0	0	1	0	0	0	0	0	0	0	0	0	0	0	0
Dactylococcopsis	1	8	0	1	0	0	0	0	0	0	0	0	0	0	0	0	0	0	0	0
Dechloromonas	1	40	1	1	0	0	1	1	0	0	0	0	0	0	0	0	0	0	0	1
Dechlorosoma	1	19	1	1	1	0	0	1	0	0	0	0	0	0	0	0	0	0	0	0
Deferribacter	2	73	1	1	0	0	0	0	0	0	0	0	0	0	0	0	0	0	0	0
Dehalobacter	1	9	0	1	0	0	0	0	0	0	0	0	0	0	0	0	0	0	0	0
Dehalococcoides	2	41	0	1	0	0	0	0	0	0	0	0	0	0	0	1	0	0	0	0
Dehalogenimonas	1	37	0	1	0	0	0	0	0	0	0	0	0	0	0	1	0	0	0	0
Deinococcus	2	140	1	1	0	0	0	1	0	0	0	0	0	0	0	0	0	0	1	1
Delftia	3	177	1	1	0	0	1	1	0	0	0	1	0	0	0	0	0	0	0	1
Denitrovibrio	2	67	0	1	0	0	0	0	0	0	0	0	0	0	0	0	0	0	0	0
Desulfarculus	2	92	1	1	0	0	0	0	0	0	0	0	0	0	0	1	0	0	0	0
Desulfatibacillum	3	84	1	1	0	0	0	0	0	0	0	0	0	0	0	0	0	0	1	0
Desulfitobacterium	2	102	1	1	0	0	0	1	0	1	0	0	0	0	0	1	0	0	0	0
Desulfobacca	2	65	1	1	0	0	0	0	0	0	0	0	0	0	0	1	0	0	0	0
Desulfobacterium	2	113	1	1	0	0	0	1	0	0	0	0	0	0	0	0	0	0	1	0
Desulfobacula	1	21	1	1	0	0	0	0	0	0	0	0	0	0	0	1	0	0	1	0
Desulfobulbus	2	88	1	1	1	0	0	0	0	0	0	0	0	0	0	0	0	0	0	0
Desulfocapsa	1	9	0	1	0	0	0	0	0	0	0	0	0	0	0	0	0	0	0	0
Desulfococcus	2	80	1	1	0	0	0	0	0	0	0	0	0	0	0	1	0	0	1	0
Desulfohalobium	2	52	1	1	0	0	0	0	0	0	0	0	0	0	0	0	0	0	0	0
Desulfomicrobium	2	65	0	1	0	0	0	0	0	0	0	0	0	0	0	0	0	0	0	0
Desulfomonile	1	25	1	1	0	0	0	0	0	0	0	0	0	0	0	1	0	0	0	0
Desulforhabdus	1	2	0	0	0	0	0	0	0	0	0	0	0	0	0	0	0	0	0	0
Desulfosporosinus	1	30	1	1	0	0	0	0	0	0	0	0	0	0	0	1	0	0	0	0
Desulfotalea	2	66	0	1	1	0	0	1	0	0	0	0	0	0	0	1	0	0	0	0
Desulfotomaculum	2	98	1	1	0	0	0	0	0	1	0	0	0	0	0	0	0	0	0	0
Desulfovibrio	2	144	0	1	1	0	0	1	0	0	0	0	0	0	1	0	0	0	0	0
Desulfurispirillum	2	78	1	1	1	0	0	0	0	0	0	0	0	0	0	1	0	0	0	0
Desulfurivibrio	2	71	0	1	1	0	0	0	0	0	0	0	0	0	0	0	0	0	0	0
Desulfurobacterium	2	53	0	1	0	0	0	0	0	0	0	0	0	0	0	1	0	0	0	0
Desulfurococcus	1	11	0	0	0	0	0	0	0	0	0	0	0	0	0	1	0	0	0	0
Diaphorobacter	1	16	0	0	0	0	0	0	0	0	0	0	0	0	0	0	0	0	0	1
Dichelobacter	2	45	0	1	0	0	0	0	0	0	0	0	0	0	0	0	0	0	0	0
Dickeya	2	155	0	1	1	0	0	0	0	0	0	0	0	0	0	0	0	0	0	0
Dictyoglomus	2	62	0	1	1	0	0	0	0	0	0	0	0	0	0	1	0	0	0	0
Dinoroseobacter	3	148	1	1	0	0	1	1	0	0	0	0	0	0	0	0	0	0	0	0
Dokdonia	1	3	0	0	0	0	0	0	0	0	0	0	0	0	0	0	0	0	0	0
Dorea	1	7	0	0	0	0	0	0	0	0	0	0	0	0	0	0	0	0	0	0
Dyadobacter	2	83	1	1	0	0	0	1	0	0	0	0	1	0	0	0	0	0	1	0
Echinicola	1	20	1	1	0	0	0	1	0	0	0	0	0	0	0	0	0	0	0	0
Edwardsiella	2	126	0	1	0	0	0	0	0	0	0	0	0	0	0	0	0	0	0	0
Eggerthella	1	51	0	1	1	0	0	1	0	0	0	0	0	0	0	1	0	0	0	0
Ehrlichia	2	37	0	1	1	0	0	0	0	0	0	0	0	0	0	0	0	0	0	0
Eikenella	1	4	0	0	0	0	0	0	0	0	0	0	0	0	0	0	0	0	0	0
Elusimicrobium	2	44	0	1	1	0	0	0	0	0	0	0	0	0	0	0	0	0	0	0
Emticicia	1	21	0	1	0	0	0	1	0	0	0	0	0	0	0	0	0	0	1	0
Enhydrobacter	1	1	0	0	0	0	0	0	0	0	0	0	0	0	0	0	0	0	0	0
Ensifer	1	2	0	0	0	0	0	0	0	0	0	0	0	0	0	0	0	0	0	0
Enterobacter	2	202	1	1	0	0	0	1	0	0	0	1	0	0	0	0	0	0	0	0
Enterobacteriaceae	2	211	1	1	1	0	0	1	0	0	0	1	0	0	0	0	0	0	0	0
Enterococcus	1	187	0	1	1	0	0	1	0	0	0	1	0	0	0	0	0	0	0	0

Genus	Cluster Number	Protein Counts	K00074	K00088	K00100	K00106	K00114	K00128	K00129	K00132	K00141	K00146	K00148	K00152	K00155	K00169	K00217	K00224	K00274	K00446
Enterovibrio	1	15	0	1	0	0	0	0	0	0	0	0	0	0	0	0	0	0	0	0
Erwinia	2	151	0	1	0	0	0	1	0	0	0	0	0	0	0	0	0	0	0	0
Erysipelothrix	1	9	0	1	0	0	0	1	0	0	0	0	0	0	0	0	0	0	0	0
Erysipelotrichaceae	1	4	0	0	0	0	0	0	0	0	0	0	0	0	0	0	0	0	0	0
Erythrobacter	2	80	1	1	0	0	1	0	0	0	0	0	0	0	0	0	0	0	0	0
Escherichia	2	242	1	1	1	0	0	0	0	0	0	1	0	0	0	0	0	0	0	0
Ethanoligenens	2	69	1	1	0	0	0	0	0	0	0	0	0	0	0	0	0	0	0	0
Eubacteriaceae	1	2	0	0	0	0	0	0	0	0	0	0	0	0	0	0	0	0	0	0
Eubacterium	2	103	1	1	1	0	0	1	0	0	0	0	0	0	0	1	0	0	0	0
Eudoraea	1	4	0	0	0	0	0	0	0	0	0	0	0	0	0	0	0	0	0	0
Euryarchaeota	1	1	0	0	0	0	0	0	0	0	0	0	0	0	0	0	0	0	0	0
Exiguobacterium	2	93	1	1	1	0	0	1	0	0	0	0	0	0	0	0	0	0	1	0
Faecalibacterium	1	12	1	1	0	0	0	0	0	0	0	0	0	0	0	0	0	0	0	0
Ferrimonas	2	115	0	1	0	0	0	1	0	0	0	0	0	0	0	0	0	0	0	0
Ferroglobus	1	14	1	1	0	0	0	0	0	0	0	0	0	0	0	1	0	0	0	0
Ferroplasma	1	11	1	1	0	0	0	0	0	0	0	0	0	0	0	0	0	0	0	0
Fervidicoccus	1	7	0	0	0	0	0	0	0	0	0	0	0	0	0	1	0	0	0	0
Fervidobacterium	2	54	0	1	1	0	0	0	0	0	0	0	0	0	0	1	0	0	0	0
Fibrella	1	17	0	1	0	0	0	1	0	0	0	0	0	0	0	0	0	0	1	0
Fibrobacter	2	52	0	1	1	0	0	1	0	0	0	0	0	0	0	0	0	0	0	0
Filifactor	1	9	1	1	0	0	0	0	0	0	0	0	0	0	0	0	0	0	0	0
Finegoldia	2	55	0	1	0	0	0	1	0	0	0	0	0	0	0	1	0	0	0	0
Firmicutes	1	15	0	1	0	0	0	0	0	0	0	0	0	0	0	0	0	0	0	0
Fischerella	1	15	0	1	0	0	0	0	0	0	0	0	0	0	0	0	0	0	0	0
Flavobacteria	1	7	1	0	0	0	0	1	0	0	0	0	0	0	0	0	0	0	0	0
Flavobacteriaceae	2	69	1	1	0	0	0	1	0	0	0	0	0	0	0	0	0	0	0	0
Flavobacteriales	1	3	0	0	0	0	0	1	0	0	0	0	0	0	0	0	0	0	0	0
Flavobacterium	2	103	1	1	0	0	0	1	0	0	0	0	0	0	0	0	0	0	1	0
Flexibacter	1	17	1	1	0	0	0	1	0	0	0	0	0	0	0	0	0	0	0	0
Flexistipes	1	11	0	1	0	0	0	0	0	0	0	0	0	0	0	0	0	1	0	0
Fluoribacter	1	6	0	1	0	0	0	0	0	0	0	0	0	0	0	0	0	0	0	0
Fluviicola	2	69	1	1	0	0	0	1	0	0	0	0	0	0	0	0	0	0	0	0
Formosa	1	2	0	0	0	0	0	0	0	0	0	0	0	0	0	0	0	0	0	0
Francisella	2	88	0	1	1	0	0	1	0	0	0	0	0	0	0	0	0	0	0	0
Frankia	3	202	1	1	1	0	0	1	0	0	0	0	1	0	0	0	0	0	0	0
Frateuria	1	25	0	1	0	0	1	1	0	0	0	0	0	0	0	0	0	0	0	0
Fructobacillus	1	2	0	0	0	0	0	0	0	0	0	0	0	0	0	0	0	0	0	0
Fulvimarina	1	2	0	0	0	0	0	0	0	0	0	0	0	0	0	0	0	0	0	0
Fusobacterium	1	48	1	1	1	0	0	0	0	0	0	0	0	0	0	0	0	0	0	0
Gallibacterium	2	87	0	1	0	0	0	1	0	0	0	0	0	0	0	0	0	0	0	0
Gallionella	2	67	0	1	0	0	0	0	0	0	0	0	0	0	0	1	0	0	0	0
Gammaproteobacteria	1	9	0	0	0	0	0	0	0	0	0	0	0	0	0	0	0	0	0	0
Gardnerella	1	31	0	1	0	0	0	0	0	0	0	0	0	0	0	0	0	0	0	0
Geitlerinema	1	9	0	1	0	0	0	0	0	0	0	0	0	0	0	0	0	0	1	0
Gemmatimonas	2	93	1	1	0	0	0	1	0	0	0	0	0	0	0	0	0	0	0	0
Geobacillus	2	168	1	1	1	0	0	1	0	0	0	1	0	0	0	1	0	0	1	1
Geobacter	2	150	1	1	1	0	0	1	0	0	0	0	0	0	1	0	0	0	0	0
Geodermatophilus	2	136	1	1	0	0	1	0	0	0	1	0	1	0	0	0	0	0	0	0
Geopsychrobacter	1	2	0	0	0	0	0	0	0	0	0	0	0	0	0	0	0	0	0	0
Gillisia	1	11	0	0	0	0	0	0	0	0	0	0	0	0	0	0	0	0	0	0
Gilvimarinus	1	4	0	0	0	0	0	0	0	0	0	0	0	0	0	0	0	0	0	0
Glaciecola	2	117	1	1	0	0	1	1	0	0	0	0	0	0	0	0	0	0	1	0

Genus	Cluster Number	Protein Counts	K00074	K00088	K00100	K00106	K00114	K00128	K00129	K00132	K00141	K00146	K00148	K00152	K00155	K00169	K00217	K00224	K00274	K00446
**Gloeobacter**	2	67	0	1	0	0	0	1	0	0	0	0	0	0	0	0	0	0	0	0
**Gloeocapsa**	1	20	0	1	0	0	0	0	0	0	0	0	0	0	0	0	0	0	1	0
**Gloeothece**	1	1	0	0	0	0	0	0	0	0	0	0	0	0	0	0	0	0	0	0
**Gluconacetobacter**	2	96	0	1	0	0	1	1	0	0	0	0	0	0	0	0	0	0	1	0
**Gluconobacter**	2	76	0	1	1	0	1	1	0	0	0	0	1	0	0	0	0	0	0	0
**Gordonia**	1	158	1	1	0	0	0	1	0	0	1	0	0	0	0	0	0	0	1	1
**Gordonibacter**	1	32	0	0	0	0	0	0	0	0	0	0	0	0	0	1	0	0	0	0
**Gramella**	2	104	1	1	1	0	0	1	0	0	0	0	0	0	1	0	0	0	1	0
**Granulibacter**	2	90	1	1	0	0	1	1	0	0	0	0	0	0	0	0	0	0	0	0
**Granulicella**	2	98	1	1	0	0	0	1	0	0	1	0	1	0	0	0	0	0	1	0
**Grimontia**	1	9	0	0	0	0	0	0	0	0	0	0	0	0	0	0	0	0	0	0
**Haemophilus**	2	117	0	1	1	0	0	0	0	0	0	0	0	0	0	0	0	0	0	0
**Hafnia**	1	29	0	1	0	0	0	0	0	0	0	0	0	0	0	0	0	0	0	0
**Hahella**	3	130	1	1	1	0	0	1	0	0	0	0	0	0	1	0	0	0	0	0
**Halalkalicoccus**	1	18	1	1	0	0	0	1	0	0	0	0	0	0	0	0	0	0	0	0
**Halanaerobium**	2	89	1	1	1	0	0	0	0	0	0	0	0	0	0	1	0	0	0	0
**Haliangium**	2	108	1	1	0	0	0	0	0	0	0	0	0	0	0	0	0	0	1	0
**Haliscomenobacter**	2	107	1	1	0	0	0	1	0	0	0	0	0	0	0	0	0	0	1	0
**Haloarcula**	1	22	1	1	1	0	0	1	0	0	0	0	1	0	1	0	0	0	0	0
**Halobacillus**	1	19	1	1	0	0	0	1	0	0	0	0	0	0	0	0	0	0	0	0
**Halobacteriaceae**	1	1	0	0	0	0	0	0	0	0	0	0	0	0	0	0	0	0	0	0
**Halobacterium**	1	10	1	1	0	0	0	1	0	0	0	0	0	0	0	0	0	0	0	0
**Halobacteroides**	1	14	0	1	0	0	0	0	0	0	0	0	0	0	0	1	0	0	0	0
**Haloferax**	1	20	1	1	0	0	0	1	0	0	0	0	0	0	0	0	0	0	0	0
**Halogeometricum**	1	12	1	1	0	0	0	1	0	0	0	0	0	0	0	0	0	0	0	0
**Halomicrobium**	1	10	1	1	0	0	0	1	0	0	0	0	0	0	0	0	0	0	0	0
**Halomonas**	1	149	1	1	0	0	0	1	0	0	0	0	1	0	0	0	0	0	0	0
**Halopiger**	1	16	1	1	0	0	0	1	0	0	0	0	1	0	0	0	0	0	0	0
**Haloquadratum**	1	19	1	1	1	0	0	1	0	0	0	0	1	0	0	0	0	0	0	0
**Halorhabdus**	1	10	1	1	0	0	0	0	0	0	0	0	0	0	0	1	0	0	0	0
**Halorhodospira**	2	71	0	1	0	0	0	0	0	0	0	0	0	0	0	0	0	0	0	0
**Halorubrum**	1	16	1	1	0	0	0	1	0	0	0	0	0	0	0	0	0	0	0	0
**Haloterrigena**	1	19	1	1	0	0	0	1	0	0	0	0	0	0	0	0	0	0	0	0
**Halothece**	1	9	0	1	0	0	0	0	0	0	0	0	0	0	0	0	0	0	0	0
**Halothermothrix**	2	50	0	1	1	0	0	0	0	0	0	0	0	0	0	1	0	0	0	0
**Halothiobacillus**	2	65	0	1	0	0	0	0	0	0	0	0	0	0	0	0	0	0	0	0
**Halovivax**	1	12	1	1	0	0	0	0	0	0	0	0	1	0	0	0	0	0	0	0
**Helicobacter**	2	79	0	1	0	0	0	0	0	0	0	0	0	0	0	1	0	0	0	0
**Heliobacillus**	1	4	0	1	0	0	0	0	0	0	0	0	0	0	0	0	0	0	0	0
**Heliobacterium**	2	51	1	1	0	0	0	0	0	0	0	0	0	0	0	0	0	0	0	0
**Herbaspirillum**	2	172	1	1	0	0	0	1	0	0	1	0	0	0	0	0	0	0	0	0
**Herminiimonas**	3	89	0	1	1	0	0	0	0	0	0	0	1	0	0	0	0	0	0	0
**Herpetosiphon**	3	90	1	1	0	0	0	1	0	0	0	0	0	0	0	0	0	0	0	0
**Hippea**	2	44	0	1	0	0	0	0	0	0	0	0	0	0	0	1	0	0	0	0
**Hirschia**	2	102	1	1	0	0	0	1	0	0	0	0	0	0	0	0	0	0	0	0
**Histophilus**	2	72	0	1	1	0	0	0	0	0	0	0	0	0	0	0	0	0	0	0
**Hoeflea**	1	16	0	1	1	0	0	0	0	0	0	0	0	0	0	0	0	0	0	0
**Hydrogenivirga**	1	2	0	0	0	0	0	0	0	0	0	0	0	0	0	0	0	0	0	0
**Hydrogenobacter**	2	55	0	1	0	0	1	0	0	0	0	0	0	0	0	1	0	0	0	0
**Hydrogenobaculum**	2	44	0	1	0	0	0	0	0	0	0	0	0	0	0	1	0	0	0	0
**Hydrogenophaga**	1	9	0	0	0	0	0	0	0	0	0	0	0	0	0	0	0	0	0	1
**Hylemonella**	1	4	0	0	0	0	0	0	0	0	0	0	0	0	0	0	0	0	0	0

Genus	Cluster Number	Protein Counts	K00074	K00088	K00100	K00106	K00114	K00128	K00129	K00132	K00141	K00146	K00148	K00152	K00155	K00169	K00217	K00224	K00274	K00446
Hyperthermus	1	6	0	0	0	0	0	0	0	0	0	0	0	0	0	1	0	0	0	0
Hyphomicrobium	2	93	1	1	0	0	1	1	0	0	0	0	0	0	0	0	0	0	1	0
Hyphomonas	3	105	1	1	1	0	1	1	0	0	0	0	0	0	0	0	0	0	0	0
Ideonella	1	18	0	0	0	0	0	0	0	0	0	0	0	0	0	0	0	0	0	0
Idiomarina	2	86	0	1	0	0	0	1	0	0	0	0	0	0	0	0	0	0	0	0
Ignavibacterium	1	14	1	1	0	0	0	0	0	0	0	0	0	0	0	0	0	0	0	0
Ignicoccus	1	5	0	0	0	0	0	0	0	0	0	0	0	0	0	1	0	0	0	0
Ignisphaera	1	8	0	1	0	0	0	0	0	0	0	0	0	0	0	1	0	0	0	0
Ilyobacter	2	85	1	1	1	0	0	0	0	0	0	0	0	0	0	1	0	0	0	0
Intrasporangium	2	114	1	1	0	0	0	1	0	0	0	0	1	0	0	0	0	0	0	1
Isoptericola	1	18	0	1	0	0	0	1	0	0	0	0	0	0	0	0	0	0	1	0
Isosphaera	2	75	1	1	0	0	0	1	0	0	0	1	0	0	0	0	0	0	0	0
Jannaschia	3	151	1	1	0	0	0	1	0	0	0	0	0	0	0	0	0	0	0	1
Janthinobacterium	2	99	0	1	0	0	0	0	0	0	0	0	0	0	0	0	0	0	0	0
Jonesia	2	56	0	1	0	0	0	1	0	0	0	0	0	0	0	0	0	0	0	0
Joostella	1	3	0	0	0	0	0	0	0	0	0	0	0	0	0	0	0	0	0	0
Kaistia	1	2	0	0	0	0	0	0	0	0	0	0	0	0	0	0	0	0	0	0
Kangiella	2	84	0	1	0	0	0	1	0	0	0	0	0	0	0	0	0	0	0	0
Ketogulonicigenium	2	102	0	1	0	0	1	1	0	0	0	0	0	0	0	0	0	0	1	0
Kineococcus	1	24	1	1	0	0	0	1	0	0	0	0	1	0	0	0	0	0	1	0
Kinetoplastibacterium	1	2	0	1	0	0	0	0	0	0	0	0	0	0	0	0	0	0	0	0
Kingella	1	4	0	0	0	0	0	0	0	0	0	0	0	0	0	0	0	0	0	0
Kitasatospora	1	36	1	1	0	0	0	1	0	0	0	0	0	0	0	0	0	0	1	0
Klebsiella	3	222	1	1	0	0	0	1	0	0	0	1	0	0	0	0	0	0	0	0
Kluyvera	1	1	0	0	0	0	0	0	0	0	0	0	0	0	0	0	0	0	0	0
Kocuria	2	87	1	1	0	0	0	1	0	0	0	0	1	0	0	0	0	0	1	0
Kordia	1	5	0	0	0	0	0	0	0	0	0	0	0	0	0	0	0	0	0	0
Kosmotoga	2	61	0	0	0	0	0	0	0	0	0	0	0	0	0	1	0	0	0	0
Kribbella	2	99	1	1	0	0	0	1	0	0	0	0	0	0	0	0	0	0	0	0
Krokinobacter	2	90	1	1	0	0	0	1	0	0	0	0	0	0	0	0	0	0	1	0
Kyrpidia	2	113	1	1	0	0	0	1	0	0	0	0	0	0	0	0	0	0	0	1
Kytococcus	2	70	1	1	0	0	0	1	0	0	0	0	0	0	0	0	0	0	0	0
Labrenzia	1	4	0	0	0	0	0	0	0	0	0	0	0	0	0	0	0	0	0	0
Lachnoanaerobaculum	1	1	0	0	0	0	0	0	0	0	0	0	0	0	0	0	0	0	0	0
Lachnospiraceae	1	51	1	1	0	0	0	0	0	0	0	0	0	0	0	1	0	0	0	0
Lacinutrix	1	20	1	1	0	0	0	1	0	0	0	0	0	0	0	0	0	0	1	0
Lactobacillus	2	119	1	1	1	0	0	0	0	0	0	0	0	0	0	0	0	0	0	0
Lactococcus	2	82	0	1	1	0	0	0	0	0	0	0	0	0	0	0	0	0	0	0
Lamprocystis	1	2	0	0	0	0	0	0	0	0	0	0	0	0	0	0	0	0	0	0
Laribacter	2	80	1	1	0	0	0	0	0	0	0	0	0	0	0	0	0	0	0	0
Lawsonia	2	33	0	1	0	0	0	0	0	0	0	0	0	0	0	0	0	0	0	0
Leadbetterella	2	79	0	1	0	0	0	1	0	0	0	0	0	0	0	0	0	0	0	0
Leeuwenhoekiella	1	2	0	0	0	0	0	0	0	0	0	0	0	0	0	0	0	0	0	0
Legionella	2	117	1	1	1	0	0	1	0	0	0	0	0	0	1	0	0	0	0	0
Leifsonia	2	48	0	1	1	0	0	1	0	0	0	0	0	0	0	0	0	0	0	0
Leptolyngbya	1	10	0	1	0	0	0	0	0	0	0	0	0	0	0	0	0	0	0	0
Leptospira	2	76	0	1	1	0	0	1	0	0	0	0	0	0	0	0	0	0	1	0
Leptospirillum	1	10	1	1	0	0	0	0	0	0	0	0	0	0	0	1	0	0	0	0
Leptothrix	3	186	1	1	0	0	1	1	0	0	0	0	0	1	0	1	1	0	1	1
Leptotrichia	2	43	0	1	0	0	0	0	0	0	0	0	0	0	0	0	0	0	0	0
Leucobacter	1	2	0	0	0	0	0	0	0	0	0	0	0	0	0	0	0	0	0	0
Leuconostoc	2	64	0	1	1	0	0	1	0	0	0	0	0	0	0	0	0	0	0	0

Genus	Cluster Number	Protein Counts	K00074	K00088	K00100	K00106	K00114	K00128	K00129	K00132	K00141	K00146	K00148	K00152	K00155	K00169	K00217	K00224	K00274	K00446
Liberibacter	1	4	0	1	0	0	0	0	0	0	0	0	0	0	0	0	0	0	0	0
Limnohabitans	1	11	0	0	0	0	0	1	0	0	0	0	0	0	0	0	0	0	0	0
Listeria	2	77	0	1	0	0	0	0	0	0	0	0	0	0	0	0	0	0	0	0
Listeriaceae	1	6	0	0	0	0	0	0	0	0	0	0	0	0	0	0	0	0	0	0
Listonella	1	38	0	1	0	0	0	0	0	0	0	0	0	0	0	0	0	0	0	0
Loktanella	1	3	0	0	0	0	0	0	0	0	0	0	0	0	0	0	0	0	0	0
Lysinibacillus	2	97	1	1	1	0	0	1	0	0	0	0	0	0	0	0	0	0	0	0
Macrococcus	2	60	1	1	1	0	0	1	0	0	0	0	0	0	0	0	0	0	0	0
Magnetococcus	2	64	0	1	0	0	0	0	0	0	0	0	0	0	1	0	0	0	0	0
Magnetospirillum	2	113	1	1	0	0	0	0	0	0	0	0	0	0	0	1	0	0	0	0
Mahella	2	58	0	1	1	0	0	0	0	0	0	0	0	0	0	1	0	0	0	0
Mannheimia	2	85	0	1	1	0	0	1	0	0	0	0	0	0	0	0	0	0	0	0
Maribacter	3	87	1	1	0	0	0	1	0	0	0	0	0	0	0	0	0	0	1	0
Maricaulis	3	87	0	1	0	0	0	0	0	0	0	0	0	0	0	0	0	0	1	0
Marichromatium	1	2	0	0	0	0	0	0	0	0	0	0	0	0	0	0	0	0	0	0
Marinithermus	2	77	1	1	0	0	0	1	0	0	0	0	0	0	0	0	0	0	0	1
Marinitoga	1	15	1	1	0	0	0	0	0	0	0	0	0	0	0	1	0	0	0	0
Marinobacter	2	163	1	1	0	0	1	1	0	0	0	0	1	0	0	0	0	0	0	1
Marinomonas	3	192	1	1	0	0	0	1	0	0	1	1	1	0	0	0	0	0	0	0
Maritimibacter	1	7	0	0	0	0	0	0	0	0	0	0	0	0	0	0	0	0	0	0
Marivirga	2	96	1	1	0	0	0	1	0	0	0	0	0	0	0	0	0	0	0	0
Martelella	1	3	0	0	0	0	0	0	0	0	0	0	0	0	0	0	0	0	0	0
Marvinbryantia	1	1	0	0	0	0	0	0	0	0	0	0	0	0	0	0	0	0	0	0
Massilia	1	4	0	0	0	0	0	0	0	0	0	0	0	0	0	0	0	0	0	0
Mastigocladopsis	1	16	0	1	0	0	0	0	0	0	0	0	0	0	0	0	0	0	0	0
Megamonas	1	47	0	0	0	0	0	0	0	0	0	0	0	0	0	0	0	0	0	0
Megasphaera	1	14	1	1	1	0	0	0	0	0	0	0	0	0	0	0	0	0	0	0
Meiothermus	2	110	1	1	0	0	0	1	0	0	0	0	0	0	0	0	0	0	0	1
Melissococcus	2	46	0	1	0	0	0	0	0	0	0	0	0	0	0	0	0	0	0	0
Mesoflavibacter	1	6	0	0	0	0	0	0	0	0	0	0	0	0	0	0	0	0	0	0
Mesoplasma	1	23	0	1	0	0	0	0	0	0	0	0	0	0	0	0	0	0	0	0
Mesorhizobium	2	201	1	1	1	0	0	1	0	0	0	1	0	0	0	0	0	0	1	1
Mesotoga	1	14	0	0	0	0	0	0	0	0	0	0	0	0	0	0	0	0	0	0
Metallosphaera	1	18	1	0	0	0	0	0	0	0	0	0	0	0	0	1	1	0	0	1
Methanobacterium	1	7	0	1	0	0	0	0	0	0	0	0	0	0	0	1	0	0	0	0
Methanobrevibacter	1	9	1	1	0	0	0	0	0	0	0	0	0	0	0	1	0	0	0	0
Methanocaldococcus	1	6	0	1	0	0	0	0	0	0	0	0	0	0	0	1	0	0	0	0
Methanocella	1	15	0	1	1	0	0	0	0	0	0	0	1	0	0	1	0	0	0	0
Methanococcaceae	1	1	0	0	0	0	0	0	0	0	0	0	0	0	0	0	0	0	0	0
Methanococcoides	1	5	0	0	0	0	0	0	0	0	0	0	0	0	0	1	0	0	0	0
Methanococcus	1	9	0	1	0	0	0	0	0	0	0	0	0	0	0	1	0	0	0	0
Methanocorpusculum	1	7	0	1	0	0	0	0	0	0	0	0	0	0	0	1	0	0	0	0
Methanoculleus	1	8	0	1	0	0	0	0	0	0	0	0	0	0	0	1	0	0	0	0
Methanohalobium	1	5	0	0	0	0	0	0	0	0	0	0	0	0	0	1	0	0	0	0
Methanohalophilus	1	5	0	0	0	0	0	0	0	0	0	0	0	0	0	1	0	0	0	0
Methanolobus	1	5	0	0	0	0	0	0	0	0	0	0	0	0	0	1	0	0	0	0
Methanomethylovorans	1	5	0	0	0	0	0	0	0	0	0	0	0	0	0	1	0	0	0	0
Methanoplanus	1	7	0	1	0	0	0	0	0	0	0	0	0	0	0	1	0	0	0	0
Methanopyrus	1	4	0	1	0	0	0	0	0	0	0	0	0	0	0	1	0	0	0	0
Methanoregula	1	8	0	1	0	0	0	0	0	0	0	0	0	0	0	1	0	0	0	0
Methanosaeta	1	9	0	0	0	0	0	0	0	0	0	0	0	0	0	1	0	0	0	0
Methanosalsum	1	7	0	0	0	0	0	1	0	0	0	0	0	0	0	1	0	0	0	0

Genus	Cluster Number	Protein Counts	K00074	K00088	K00100	K00106	K00114	K00128	K00129	K00132	K00141	K00146	K00148	K00152	K00155	K00169	K00217	K00224	K00274	K00446
Methanosarcina	1	18	0	0	1	0	0	1	0	0	0	0	1	0	0	1	0	0	0	0
Methanosphaera	1	7	1	1	0	0	0	0	0	0	0	0	0	0	0	1	0	0	0	0
Methanosphaerula	1	8	0	1	0	0	0	0	0	0	0	0	0	0	0	1	0	0	0	0
Methanospirillum	1	6	0	1	0	0	0	0	0	0	0	0	0	0	0	1	0	0	0	0
Methanothermobacter	1	6	0	1	0	0	0	0	0	0	0	0	0	0	0	1	0	0	0	0
Methanothermococcus	1	5	0	1	0	0	0	0	0	0	0	0	0	0	0	1	0	0	0	0
Methanothermus	1	5	0	1	0	0	0	0	0	0	0	0	0	0	0	1	0	0	0	0
Methanotorris	1	5	0	1	0	0	0	0	0	0	0	0	0	0	0	1	0	0	0	0
Methylacidiphilum	2	48	0	1	0	0	0	0	0	0	0	1	0	0	0	0	0	0	0	0
Methylarcula	1	2	0	0	0	0	0	0	0	0	0	0	0	0	0	0	0	0	0	0
Methylibium	1	38	1	1	1	0	1	1	0	0	0	0	0	0	0	0	0	0	0	1
Methylobacillus	2	61	0	1	1	0	0	1	0	0	0	0	0	0	0	0	0	0	0	0
Methylobacterium	3	236	1	1	0	0	1	1	0	0	0	0	0	0	0	0	0	0	1	0
Methylocella	3	117	1	1	0	0	1	1	0	0	0	1	0	0	0	0	0	0	1	1
Methylococcus	2	80	0	1	0	0	0	0	0	0	0	0	0	0	0	0	0	0	0	0
Methylocystis	1	21	1	1	0	0	0	0	0	0	0	0	0	0	0	0	0	0	1	0
Methyloferula	1	2	0	0	0	0	0	0	0	0	0	0	0	0	0	0	0	0	0	0
Methylomicrobium	1	12	0	1	0	0	0	0	0	0	0	0	0	0	0	0	0	0	0	0
Methylomonas	1	15	0	1	0	0	0	0	0	0	0	0	0	0	0	0	0	0	1	0
Methylophaga	1	7	0	1	0	0	0	0	0	0	0	0	0	0	0	0	0	0	0	0
Methylophilus	1	2	0	0	0	0	0	0	0	0	0	0	0	0	0	0	0	0	0	0
Methylosinus	1	2	0	0	0	0	0	0	0	0	0	0	0	0	0	0	0	0	0	0
Methylotenera	2	72	0	1	0	0	0	1	0	0	0	0	0	0	0	0	0	0	0	0
Methyloversatilis	1	5	0	0	0	0	0	0	0	0	0	0	0	0	0	0	0	0	0	0
Methylovorus	2	66	0	1	0	0	0	1	0	0	0	0	0	0	0	0	0	0	0	0
Micavibrio	1	9	0	1	0	0	0	0	0	0	0	0	0	0	0	0	0	0	0	0
Microbacterium	2	115	1	1	0	0	0	1	0	0	0	0	0	0	0	0	0	0	1	1
Microchaete	1	23	0	1	0	0	0	0	0	0	0	0	0	0	0	0	0	0	0	0
Micrococcus	2	72	1	1	0	0	0	1	0	0	0	0	1	0	0	0	0	0	0	0
Microcoleus	1	17	1	1	0	0	0	0	0	0	0	0	0	0	0	0	0	0	0	0
Microcystis	2	67	0	1	0	0	0	1	0	0	0	0	0	0	0	0	0	0	1	0
Microlunatus	1	25	1	1	0	0	0	1	0	0	0	0	1	0	0	0	0	0	1	0
Micromonospora	2	115	1	1	0	0	0	1	0	0	0	0	1	0	0	0	0	0	1	0
Microvirga	1	6	0	0	0	0	0	0	0	0	0	0	0	0	0	0	0	0	0	0
Mobiluncus	1	41	0	1	0	0	0	0	0	0	0	0	0	0	0	1	0	0	0	0
Modestobacter	1	43	1	1	0	0	0	0	0	0	0	0	1	0	0	0	0	0	0	1
Moorea	1	2	0	0	0	0	0	0	0	0	0	0	0	0	0	0	0	0	0	0
Moorella	2	69	0	1	0	0	0	0	0	0	0	0	0	0	0	1	0	0	0	0
Moraxella	2	66	0	1	0	0	0	1	0	0	0	0	0	0	0	0	0	0	0	0
Morganella	1	29	0	1	0	0	0	0	0	0	0	0	0	0	0	0	0	0	0	0
Moritella	1	2	0	0	0	0	0	0	0	0	0	0	0	0	0	0	0	0	0	0
Muricauda	1	23	1	1	0	0	0	1	0	0	0	0	0	0	0	0	0	0	0	0
Mycobacterium	3	306	1	1	1	0	1	1	0	0	0	1	0	0	1	0	1	0	1	0
Mycoplasma	1	53	0	1	1	0	0	1	0	0	0	0	0	0	0	0	0	0	0	0
Myxococcus	2	110	1	1	0	1	0	1	0	0	0	0	1	0	0	0	0	0	1	0
Nakamurella	2	115	1	1	0	0	0	1	0	0	0	0	1	0	0	0	0	0	0	0
napthalene	1	2	0	0	0	0	0	0	0	0	0	0	0	0	0	0	0	0	0	0
Natranaerobius	2	55	1	1	0	0	0	0	0	0	0	0	0	0	0	0	0	0	0	0
Natrialba	1	16	1	1	0	0	0	1	0	0	0	0	0	0	0	0	0	0	1	0
Natrinema	1	16	1	1	0	0	1	1	0	0	0	0	0	0	0	0	0	0	0	0
Natronobacterium	1	11	1	1	0	0	0	0	0	0	0	0	0	0	0	0	0	0	1	0
Natronococcus	1	21	1	1	0	0	1	1	0	0	0	0	1	0	0	0	0	0	1	0

Genus	Cluster Number	Protein Counts	K00074	K00088	K00100	K00106	K00114	K00128	K00129	K00132	K00141	K00146	K00148	K00152	K00155	K00169	K00217	K00224	K00274	K00446
Natronomonas	1	20	1	1	1	0	0	1	0	0	0	0	0	0	1	0	0	0	0	0
Nautilia	2	48	0	1	0	0	0	0	0	0	0	0	0	0	0	1	0	0	0	0
Neisseria	2	67	0	1	0	0	0	0	0	0	0	0	0	0	0	0	0	0	0	0
Neisseriaceae	1	1	0	0	0	0	0	0	0	0	0	0	0	0	0	0	0	0	0	0
Neorickettsia	1	31	0	1	0	0	0	0	0	0	0	0	0	0	0	0	0	0	0	0
Neptuniibacter	1	2	0	0	0	0	0	0	0	0	0	0	0	0	0	0	0	0	0	0
Niastella	1	21	1	1	0	0	0	0	0	0	0	0	0	0	0	0	0	0	0	0
Nitratifractor	2	54	0	1	0	0	0	0	0	0	0	0	0	0	0	1	0	0	0	0
Nitratireductor	1	19	0	0	0	0	0	0	0	0	0	0	0	0	0	0	0	0	0	0
Nitratiruptor	2	40	0	1	0	0	0	0	0	0	0	0	0	0	0	1	0	0	0	0
Nitrobacter	2	99	1	1	0	0	0	1	0	0	0	0	0	0	0	0	0	0	1	0
Nitrococcus	1	2	0	0	0	0	0	0	0	0	0	0	0	0	0	0	0	0	0	0
Nitrosococcus	2	94	0	1	0	0	0	1	0	0	0	0	0	0	0	0	0	0	0	0
Nitrosomonas	2	72	0	1	0	0	0	0	0	0	0	0	0	0	0	0	0	0	0	0
Nitrosopumilaceae	1	7	0	1	0	0	0	0	0	0	0	0	0	0	0	0	0	0	0	0
Nitrosopumilus	1	11	0	1	0	0	0	0	0	0	0	0	0	0	0	0	0	0	0	0
Nitrosospira	2	74	0	1	0	0	0	0	0	0	0	0	0	0	0	0	0	0	0	0
Nocardia	2	185	1	1	1	0	0	1	0	0	0	0	0	0	1	0	0	0	1	1
Nocardioidaceae	1	2	0	0	0	0	0	0	0	0	0	0	0	0	0	0	0	0	0	0
Nocardioides	2	157	1	1	0	0	0	1	0	0	0	0	0	0	0	1	0	0	1	0
Nocardiopsis	2	102	1	1	0	0	0	1	0	0	0	0	1	0	0	0	0	0	1	0
Nodularia	1	22	0	1	0	0	0	0	0	0	0	0	0	0	0	0	0	0	0	0
Nonlabens	1	19	1	1	0	0	0	1	0	0	0	0	0	0	0	0	0	0	1	0
Nostoc	2	111	1	1	0	0	0	1	0	0	0	0	0	0	0	0	0	1	1	0
Nostocaceae	1	2	0	0	0	0	0	0	0	0	0	0	0	0	0	0	0	0	0	0
Novosphingobium	2	169	1	1	0	0	1	1	0	0	0	1	0	0	0	0	0	0	0	1
Oceanibaculum	1	3	0	0	0	0	0	0	0	0	0	0	0	0	0	0	0	0	0	0
Oceanibulbus	1	12	0	1	0	0	0	0	0	0	0	0	0	0	0	0	0	0	0	0
Oceanicola	1	9	0	0	0	0	0	1	0	0	0	0	0	0	0	0	0	0	0	0
Oceanimonas	1	33	0	1	0	0	0	1	0	0	0	0	1	0	0	0	0	0	0	0
Oceaniovalibus	1	2	0	0	0	0	0	0	0	0	0	0	0	0	0	0	0	0	0	0
Oceanithermus	2	72	1	1	0	0	0	1	0	0	0	0	0	0	0	0	0	0	0	0
Oceanobacillus	2	91	1	1	1	0	0	1	0	0	0	0	0	0	1	0	0	0	0	0
Oceanospirillum	1	2	0	0	0	0	0	0	0	0	0	0	0	0	0	0	0	0	0	0
Ochrobactrum	2	133	1	1	0	0	0	1	0	0	0	0	1	0	1	0	0	0	0	0
Octadecabacter	1	29	1	1	0	0	0	0	0	0	0	0	0	0	0	0	0	0	1	0
Oenococcus	2	41	0	1	0	0	0	0	0	0	0	0	0	0	0	0	0	0	0	0
Oleomonas	1	2	0	0	0	0	0	0	0	0	0	0	0	0	0	0	0	0	0	0
Oligotropha	3	87	1	1	0	0	0	1	0	0	0	0	0	0	0	0	0	0	1	0
Olsenella	2	40	1	1	1	0	0	0	0	0	0	0	0	0	0	0	0	0	0	0
Opitutus	2	67	0	1	0	0	0	1	0	0	0	0	0	0	0	0	0	0	1	0
Orientia	1	21	0	0	0	0	0	0	0	0	0	0	0	0	0	0	0	0	0	0
Ornithobacterium	1	11	0	1	0	0	0	0	0	0	0	0	0	0	0	0	0	0	0	0
Oscillatoria	1	16	0	1	0	0	0	1	0	0	0	0	0	0	0	0	0	0	1	0
Oscillatoriales	1	2	0	0	0	0	0	0	0	0	0	0	0	0	0	0	0	0	0	0
Oscillibacter	1	20	1	1	1	0	0	0	0	0	0	0	0	0	0	1	0	0	0	0
Owenweeksia	1	15	1	1	0	0	0	1	0	0	0	0	0	0	0	0	0	0	0	0
Oxalobacteraceae	1	4	0	0	0	0	0	0	0	0	0	0	0	0	0	0	0	0	0	0
Paenibacillus	2	152	1	1	1	0	0	1	0	0	0	0	0	0	0	1	0	0	1	0
Paludibacter	2	67	1	1	1	0	0	0	0	0	0	0	0	0	0	0	0	0	0	0
Pandoraea	1	18	0	0	0	0	0	0	0	0	0	0	0	0	0	0	0	0	0	0
Pannonibacter	1	3	0	0	0	0	0	0	0	0	0	0	1	0	0	0	0	0	0	0

Genus	Cluster Number	Protein Counts	K00074	K00088	K00100	K00106	K00114	K00128	K00129	K00132	K00141	K00146	K00148	K00152	K00155	K00169	K00217	K00224	K00274	K00446
Pantholops	1	23	0	0	0	0	0	0	0	0	0	0	0	0	0	0	0	0	0	0
Pantoea	2	181	0	1	0	0	0	1	0	0	0	0	1	0	0	0	0	0	0	1
Parabacteroides	2	64	0	1	0	0	0	0	0	0	0	0	0	0	0	0	0	0	0	0
Parachlamydia	1	11	0	0	0	0	0	0	0	0	0	0	0	0	0	0	0	0	1	0
Paracoccus	2	169	1	1	0	0	0	1	0	0	0	0	1	1	0	0	0	0	0	0
Parvibaculum	3	117	1	1	0	0	1	1	0	0	0	0	0	0	0	0	0	0	1	0
Parvularcula	2	72	1	1	0	0	0	0	0	0	0	0	0	0	0	0	0	0	1	0
Pasteurella	2	85	0	1	0	0	0	0	0	0	0	0	0	0	0	0	0	0	0	0
Pasteurellaceae	1	2	0	0	0	0	0	0	0	0	0	0	0	0	0	0	0	0	0	0
Pectobacterium	1	44	0	1	0	0	0	0	0	0	0	0	0	0	0	0	0	0	0	0
Pediococcus	1	43	0	1	0	0	0	0	0	0	0	0	0	0	0	0	0	0	0	0
Pedobacter	2	88	1	1	0	0	0	1	0	0	0	0	0	0	0	0	0	0	0	0
Pelagibaca	1	10	0	0	0	0	0	0	0	0	0	0	0	0	0	0	0	0	0	0
Pelagibacterium	1	39	0	1	0	0	1	1	0	0	0	0	1	0	0	0	0	0	0	1
Pelobacter	2	85	1	1	1	0	0	1	0	0	0	0	0	0	0	1	0	0	0	0
Pelodictyon	2	62	0	1	0	0	0	0	0	0	0	0	0	0	0	0	0	0	0	0
Pelosinus	1	1	0	0	0	0	0	0	0	0	0	0	0	0	0	0	0	0	0	0
Pelotomaculum	2	60	1	1	0	0	0	0	0	0	0	0	0	0	0	0	0	0	0	0
Peptostreptococcus	1	1	0	0	0	0	0	0	0	0	0	0	0	0	0	0	0	0	0	0
Persephonella	2	55	0	1	0	0	0	0	0	0	0	0	0	0	0	1	0	0	0	0
Petrotoga	2	72	0	1	1	0	0	0	0	0	0	0	0	0	0	1	0	0	0	0
Phaeobacter	2	55	1	1	0	0	0	1	0	0	0	0	0	0	0	0	0	0	0	0
Phenylobacterium	3	103	1	1	0	0	1	1	0	0	0	0	0	0	0	0	0	0	0	0
Photobacterium	2	119	0	1	1	0	0	1	0	0	0	0	0	0	0	0	0	0	0	0
Photorhabdus	2	149	1	1	0	0	0	1	0	0	0	1	0	0	0	0	0	0	0	0
Phycisphaera	1	9	0	1	0	0	0	0	0	0	0	0	0	0	0	0	0	0	0	0
Phyllobacterium	1	6	0	0	0	0	0	0	0	0	0	0	0	0	0	0	0	0	0	0
Picrophilus	1	15	1	1	1	0	0	1	0	0	0	0	0	0	0	0	0	0	0	0
Pirellula	2	85	0	1	0	0	0	1	0	0	0	0	0	0	0	0	0	0	0	0
Planctomyces	2	93	0	1	0	0	0	1	0	0	0	0	0	0	0	0	0	0	1	0
Plautia	1	17	0	1	0	0	0	0	0	0	0	0	0	0	0	0	0	0	0	0
Plesiomonas	1	4	0	0	0	0	0	0	0	0	0	0	0	0	0	0	0	0	0	0
Pleurocapsa	1	23	0	1	0	0	0	1	0	0	0	1	0	0	0	0	0	0	1	0
Polaribacter	2	74	1	1	0	0	0	1	0	0	0	0	0	0	0	0	0	0	0	0
Polaromonas	3	238	1	1	1	0	1	1	1	0	1	0	0	1	1	0	1	0	1	0
Polymorphum	3	173	1	1	0	0	0	1	0	0	0	1	0	0	0	0	0	0	0	1
Polynucleobacter	1	19	1	1	0	0	0	0	0	0	0	0	0	0	0	0	0	0	0	0
Ponticaulis	1	1	0	0	0	0	0	0	0	0	0	0	0	0	0	0	0	0	0	0
Porphyromonas	2	63	1	1	0	0	0	0	0	0	0	0	0	0	0	0	0	0	0	0
Prevotella	1	65	0	1	0	0	0	0	0	0	0	0	0	0	0	0	0	0	0	0
Prochlorococcus	2	68	0	1	0	0	0	1	0	0	0	0	0	0	0	0	0	0	0	0
Propionibacterium	1	62	0	1	0	0	0	0	0	0	0	0	0	0	0	0	0	0	1	0
Prosthecochloris	1	16	0	1	0	0	0	1	0	0	0	0	0	0	0	0	0	0	0	0
Proteobacteria	1	19	0	0	0	0	0	0	0	0	0	0	1	0	0	0	0	0	0	0
Proteus	2	113	0	1	0	0	0	0	0	0	0	0	0	0	0	0	0	0	0	0
Providencia	1	38	1	1	0	0	0	0	0	0	0	1	0	0	0	0	0	0	0	0
Pseudaminobacter	1	10	0	0	0	0	0	0	0	0	0	0	0	0	0	0	0	0	0	0
Pseudanabaena	1	11	0	1	0	0	0	0	0	0	0	0	0	0	0	0	0	0	0	0
Pseudoalteromonas	2	188	1	1	1	0	1	1	0	0	0	0	1	0	0	0	0	0	1	0
Pseudochrobactrum	1	7	0	0	0	0	0	1	0	0	0	0	0	0	0	0	0	0	0	0
Pseudogulbenkiania	1	40	1	1	0	0	1	1	0	0	0	1	1	0	0	0	0	0	0	0
pseudomallei	3	131	1	1	1	0	0	1	0	0	1	1	0	0	1	0	0	0	0	0

Genus	Cluster Number	Protein Counts	K00074	K00088	K00100	K00106	K00114	K00128	K00129	K00132	K00141	K00146	K00148	K00152	K00155	K00169	K00217	K00224	K00274	K00446
Pseudomonas	3	381	1	1	1	0	1	1	0	0	1	1	1	1	1	0	1	0	1	1
Pseudonocardia	3	180	1	1	0	0	1	0	0	0	0	1	1	0	0	0	0	0	0	1
Pseudorhodobacter	1	4	0	1	0	0	0	0	0	0	0	0	0	0	0	0	0	0	0	0
Pseudovibrio	1	33	1	1	0	0	0	0	0	0	0	0	0	0	0	0	0	0	0	0
Pseudoxanthomonas	2	124	0	1	0	0	0	1	0	0	1	0	0	0	0	0	0	0	1	1
Psychrobacter	2	124	1	1	0	0	0	1	0	0	0	0	0	0	0	0	0	0	1	0
Psychroflexus	1	22	1	1	0	0	0	1	0	0	0	0	0	0	0	0	0	0	1	0
Psychromonas	2	110	1	1	0	0	0	1	0	0	0	0	0	0	0	0	0	0	1	0
Pusillimonas	3	111	1	1	0	0	0	1	0	0	0	0	0	0	0	0	0	0	0	0
Pyrobaculum	1	15	0	0	1	0	0	1	0	0	0	0	0	0	0	1	0	0	0	0
Pyrococcus	1	10	0	1	0	0	0	0	0	0	0	0	0	0	0	1	0	0	0	0
Pyrolobus	1	4	0	0	0	0	0	0	0	0	0	0	0	0	0	1	0	0	0	0
Rahnella	2	143	1	1	0	0	0	0	0	0	0	0	0	0	0	0	0	0	0	0
Ralstonia	3	332	1	1	1	0	1	1	0	0	1	1	1	1	1	0	1	1	1	1
Ramlibacter	1	35	1	1	0	0	0	1	0	0	0	0	0	0	0	0	0	0	0	0
Raoultella	2	139	1	1	0	0	0	1	0	0	0	1	0	0	0	0	0	0	0	0
Raphidiopsis	1	5	0	0	0	0	0	0	0	0	0	0	0	0	0	0	0	0	0	0
Reinekea	1	2	0	0	0	0	0	0	0	0	0	0	0	0	0	0	0	0	0	0
Renibacterium	2	67	1	1	0	0	0	1	0	0	0	0	0	0	0	0	0	0	0	0
Rhizobiaceae	1	3	0	0	0	0	0	0	0	0	0	0	0	0	0	0	0	0	0	0
Rhizobiales	1	6	0	0	0	0	0	0	0	0	0	0	0	0	0	0	0	0	0	0
Rhizobium	3	257	1	1	1	0	1	1	0	0	0	0	1	0	1	0	1	0	1	1
Rhizobium/Agrobacterium	2	87	1	1	0	0	0	1	0	0	0	0	1	0	0	0	0	0	0	0
Rhodanobacter	1	21	0	1	0	0	0	1	0	0	0	0	0	0	0	0	0	0	0	0
Rhodobacter	3	175	1	1	1	0	1	1	0	0	0	0	0	0	1	0	0	0	0	1
Rhodobacteraceae	1	21	0	0	0	0	0	1	0	0	0	0	0	0	0	0	0	0	0	0
Rhodobacterales	1	34	0	1	0	0	0	1	0	0	0	0	0	0	0	0	0	0	0	0
Rhodococcus	2	292	1	1	1	0	0	1	1	0	1	0	1	0	1	0	1	1	1	1
Rhodocyclaceae	1	7	0	0	0	0	0	0	0	0	0	0	0	0	0	0	0	0	0	0
Rhodoferax	3	144	1	1	0	0	0	1	0	0	0	0	0	0	0	1	0	0	0	0
Rhodomicrobium	2	103	1	1	0	0	1	1	0	0	0	0	0	0	0	0	0	0	0	0
Rhodopirellula	2	87	0	1	1	0	0	1	0	0	0	0	1	0	0	0	0	0	0	0
Rhodopseudomonas	3	246	1	1	1	0	1	1	0	0	0	0	0	0	0	1	1	0	1	1
Rhodospirillum	2	121	1	1	0	0	0	1	0	0	0	0	0	0	0	0	0	0	1	0
Rhodothermus	2	76	1	1	0	0	0	1	0	0	0	0	0	0	0	1	0	0	0	0
Rhodovulum	1	4	0	0	0	0	0	0	0	0	0	0	0	0	0	0	0	0	0	0
Richelia	1	2	0	0	0	0	0	0	0	0	0	0	0	0	0	0	0	0	0	0
Rickettsia	2	43	0	0	0	0	0	0	0	0	0	0	0	0	0	0	0	0	0	0
Rickettsiaceae	1	5	0	0	0	0	0	0	0	0	0	0	0	0	0	0	0	0	0	0
Riemerella	2	61	1	1	0	0	0	1	0	0	0	0	0	0	0	0	0	0	0	0
Rivularia	1	18	0	1	0	0	0	0	0	0	0	0	0	0	0	0	0	0	1	0
Robiginitalea	2	83	1	1	0	0	0	1	0	0	0	0	0	0	0	0	0	0	0	0
Roseburia	2	58	1	1	0	0	0	0	0	0	0	0	0	0	0	1	0	0	0	0
Roseibium	1	3	0	0	0	0	0	0	0	0	0	0	0	0	0	0	0	0	0	0
Roseiflexus	3	100	0	1	0	0	0	1	0	0	0	0	0	0	0	0	0	0	1	1
Roseobacter	2	149	1	1	1	0	0	1	0	0	0	0	0	0	1	0	0	0	1	0
Roseomonas	1	2	0	0	0	0	0	0	0	0	0	0	0	0	0	0	0	0	0	0
Roseovarius	1	15	0	0	0	0	0	0	0	0	0	0	0	0	0	0	0	0	0	0
Rothia	1	44	0	1	0	0	0	0	0	0	0	0	0	0	0	0	0	0	0	0
Rubrivivax	1	24	1	1	0	0	0	1	0	0	0	0	0	0	0	1	0	0	0	0
Rubrobacter	3	117	1	1	1	0	0	1	0	0	0	0	1	0	0	1	0	0	0	1
Ruegeria	2	189	1	1	1	0	1	1	0	0	0	1	0	0	1	0	0	0	0	1

Genus	Cluster Number	Protein Counts	K00074	K00088	K00100	K00106	K00114	K00128	K00129	K00132	K00141	K00146	K00148	K00152	K00155	K00169	K00217	K00224	K00274	K00446
Ruminococcaceae	1	2	0	0	0	0	0	0	0	0	0	0	0	0	0	0	0	0	0	0
Ruminococcus	2	101	1	1	1	0	0	0	0	0	0	0	0	0	0	0	0	0	0	0
Runella	1	26	1	1	0	0	0	1	0	0	0	0	0	0	0	0	0	0	0	1
Saccharomonospora	2	132	1	1	0	0	0	0	0	0	1	0	1	0	0	0	0	0	0	0
Saccharophagus	2	81	0	1	0	0	0	0	0	0	0	0	0	0	0	0	0	0	0	0
Saccharopolyspora	3	194	1	1	1	0	1	1	0	0	1	1	1	0	0	0	0	0	1	0
Saccharothrix	1	32	1	1	0	0	0	0	0	0	0	0	0	0	0	0	0	0	1	0
Sagittula	1	10	0	0	0	0	0	0	0	0	0	0	0	0	0	0	0	0	0	1
Salinibacter	2	77	1	1	1	0	0	1	0	0	0	0	0	0	0	0	0	0	1	0
Salinimonas	1	1	0	0	0	0	0	0	0	0	0	0	0	0	0	0	0	0	0	0
Salinispora	3	127	1	1	0	0	0	1	0	0	0	0	0	0	0	0	0	0	1	0
Salinivibrio	1	2	0	0	0	0	0	0	0	0	0	0	0	0	0	0	0	0	0	0
Salmonella	2	151	0	1	1	0	0	0	0	0	0	1	0	0	0	0	0	0	0	0
Sanguibacter	2	73	0	1	0	0	0	1	0	0	0	0	0	0	0	0	0	0	0	0
Saprospira	1	13	1	1	0	0	0	0	0	0	0	0	0	0	0	0	0	0	0	0
Scardovia	1	1	0	0	0	0	0	0	0	0	0	0	0	0	0	0	0	0	0	0
Scytonema	1	10	0	0	0	0	0	0	0	0	0	0	0	0	0	0	0	0	0	0
Sebaldella	2	69	0	1	1	0	0	0	0	0	0	0	0	0	0	0	0	0	0	0
Segniliparus	2	85	1	1	0	0	0	1	0	0	0	0	0	0	0	0	0	0	0	0
Selenomonas	2	57	0	1	1	0	0	0	0	0	0	0	0	0	0	0	0	0	0	0
Serinicoccus	1	1	0	0	0	0	0	0	0	0	0	0	0	0	0	0	0	0	0	0
Serratia	2	186	1	1	0	0	0	1	0	0	0	1	1	0	0	0	0	0	0	0
Shewanella	3	214	0	1	1	0	1	1	0	0	0	0	0	0	0	0	0	1	0	0
Shigella	2	172	1	1	1	0	0	0	0	0	0	1	0	0	0	0	0	0	0	0
Shinella	1	2	0	0	0	0	0	0	0	0	0	0	0	0	0	0	0	0	0	0
Sideroxydans	2	81	0	1	0	0	0	0	0	0	0	0	0	0	0	0	0	0	0	0
Silicibacter	2	105	1	0	0	0	0	1	0	0	0	0	0	0	1	0	0	0	0	1
Simiduia	1	18	0	1	0	0	0	0	0	0	0	0	0	0	0	0	0	0	1	0
Simkania	1	6	0	1	0	0	0	0	0	0	0	0	0	0	0	0	0	0	0	0
Simonsiella	1	2	0	0	0	0	0	0	0	0	0	0	0	0	0	0	0	0	0	0
Singulisphaera	1	18	0	1	0	1	0	0	0	0	0	0	0	0	0	0	0	0	1	0
Sinorhizobium	3	207	1	1	1	0	1	1	0	0	0	1	1	0	1	0	0	0	1	1
Slackia	2	46	1	1	0	0	0	1	0	0	0	0	0	0	0	0	0	0	0	0
Smaragdicoccus	1	3	0	0	0	0	0	0	0	0	0	0	0	0	0	0	0	0	0	0
Sodalis	2	71	0	1	0	0	0	0	0	0	0	0	0	0	0	0	0	0	0	0
Solibacillus	1	23	1	1	0	0	0	1	0	0	0	0	0	0	0	0	0	0	0	1
Solitalea	1	13	1	1	0	0	0	0	0	0	0	0	0	0	0	0	0	0	1	0
Sorangium	3	115	1	1	0	0	0	1	0	0	0	0	0	0	0	0	0	0	1	0
Sphaerobacter	2	100	1	1	0	0	0	1	0	0	0	0	1	0	0	0	0	0	0	1
Sphaerochaeta	1	42	0	1	0	0	0	1	0	0	0	0	0	0	0	0	0	0	0	0
Sphingobacterium	3	85	1	1	0	0	0	1	0	0	0	0	0	0	0	0	0	0	0	0
Sphingobium	1	179	1	1	0	0	1	1	0	0	0	1	1	0	0	0	0	0	1	1
Sphingomonadaceae	1	15	0	0	0	0	0	1	0	0	0	0	0	0	0	0	0	0	0	0
Sphingomonas	2	220	1	1	0	0	1	1	0	0	0	1	0	0	0	0	0	0	0	0
Sphingopyxis	3	99	1	1	1	0	1	1	0	0	0	0	0	0	0	0	0	0	0	0
Spirochaeta	2	103	0	1	1	0	0	1	0	0	0	0	0	0	0	0	0	0	0	0
Spiroplasma	1	6	0	1	0	0	0	0	0	0	0	0	0	0	0	0	0	0	0	0
Spirosoma	2	107	1	1	0	0	0	1	0	0	0	0	1	0	0	0	0	0	1	1
Stackebrandtia	2	110	1	1	0	0	0	1	0	0	0	0	0	0	0	0	0	0	1	0
Stanieria	1	17	0	1	0	0	0	0	0	0	0	0	0	0	0	0	0	0	0	0
Staphylococcus	2	108	1	1	0	0	0	1	0	0	0	0	0	0	0	0	0	0	0	0
Staphylothermus	1	12	0	0	0	0	0	0	0	0	0	0	0	0	0	1	0	0	0	0

Genus	Cluster Number	Protein Counts	K00074	K00088	K00100	K00106	K00114	K00128	K00129	K00132	K00141	K00146	K00148	K00152	K00155	K00169	K00217	K00224	K00274	K00446
**Stappia**	1	2	0	0	0	0	0	0	0	0	0	0	0	0	0	0	0	0	0	0
**Starkeya**	3	134	1	1	0	0	0	1	0	0	0	0	0	0	0	0	0	0	1	0
**Stenotrophomonas**	3	104	0	1	0	0	0	1	0	0	1	0	0	0	0	0	0	0	0	0
**Stigmatella**	3	129	1	1	0	0	0	1	0	0	0	0	0	0	0	0	0	0	1	0
**Streptobacillus**	1	38	0	1	0	0	0	0	0	0	0	0	0	0	0	0	0	0	0	0
**Streptococcus**	2	121	1	1	1	0	0	0	0	0	0	0	0	0	0	0	0	0	0	0
**Streptomyces**	2	253	1	1	1	0	1	1	0	0	1	1	1	0	0	0	0	0	1	1
**Streptosporangium**	3	148	1	1	0	0	0	1	0	0	0	0	1	0	0	0	0	0	1	0
**Strigomonas**	1	2	0	1	0	0	0	0	0	0	0	0	0	0	0	0	0	0	0	0
**Sulfitobacter**	1	10	0	1	0	0	0	0	0	0	0	0	0	0	0	0	0	0	0	0
**Sulfobacillus**	1	30	1	1	0	0	0	1	0	0	0	0	1	0	0	0	0	0	0	1
**Sulfolobus**	1	23	1	0	0	0	0	1	0	0	0	0	0	0	0	1	1	0	0	1
**Sulfuricurvum**	2	67	0	1	1	0	0	0	0	0	0	0	0	0	0	1	0	0	0	0
**Sulfurihydrogenibium**	2	55	0	1	1	0	0	0	0	0	0	0	0	0	0	1	0	0	0	0
**Sulfurimonas**	2	60	0	1	0	0	0	0	0	0	0	0	0	0	0	1	0	0	0	0
**Sulfurospirillum**	1	48	0	1	1	0	0	1	0	0	0	0	0	0	0	0	0	0	0	0
**Sulfurovum**	2	48	0	1	0	0	0	0	0	0	0	0	0	0	0	1	0	0	0	0
**Symbiobacterium**	2	68	1	1	1	0	0	0	0	0	0	0	0	0	0	1	0	0	0	0
**Synechococcus**	1	34	0	1	1	0	0	1	0	0	0	0	0	0	0	0	0	0	1	0
**Synechocystis**	1	40	0	1	0	0	0	1	0	0	0	0	0	0	0	0	0	0	1	0
**Synergistetes**	1	27	0	1	0	0	0	0	0	0	0	0	0	0	0	0	0	0	0	0
**Syntrophobacter**	2	101	1	1	0	0	0	0	0	0	0	0	0	0	0	1	0	0	0	0
**Syntrophobotulus**	2	71	0	1	0	0	0	1	0	0	0	0	0	0	0	0	0	0	1	0
**Syntrophomonas**	2	58	1	1	1	0	0	0	0	0	0	0	0	0	0	1	0	0	0	0
**Syntrophothermus**	2	60	1	1	0	0	0	0	0	0	0	0	0	0	0	0	0	0	0	0
**Syntrophus**	2	66	1	1	1	0	0	1	0	0	0	0	0	0	0	1	0	1	0	0
**Tannerella**	1	8	0	1	0	0	0	0	0	0	0	0	0	0	0	0	0	0	0	0
**Taylorella**	1	43	0	1	0	0	0	1	0	0	0	0	0	0	0	0	0	0	0	0
**Tepidanaerobacter**	2	55	1	1	0	0	0	0	0	0	0	0	0	0	0	1	0	0	0	0
**Teredinibacter**	2	109	0	1	0	0	0	0	0	0	0	0	0	0	0	0	0	0	1	0
**Terrabacter**	1	1	0	0	0	0	0	0	0	0	0	0	0	0	0	0	0	0	0	0
**Terriglobus**	2	83	1	1	0	0	1	0	0	0	0	0	1	0	0	0	0	0	1	0
**Tetragenococcus**	1	12	0	1	1	0	0	0	0	0	0	0	0	0	0	0	0	0	0	0
**Thalassiobium**	1	11	0	0	0	0	0	0	0	0	0	0	0	0	0	0	0	0	0	1
**Thalassobacter**	1	9	0	0	0	0	0	0	0	0	0	0	0	0	0	0	0	0	0	0
**Thalassolituus**	1	12	0	1	0	0	0	0	0	0	0	0	0	0	0	0	0	0	0	0
**Thalassospira**	1	2	0	0	0	0	0	0	0	0	0	0	0	0	0	0	0	0	0	0
**Thauera**	1	111	1	1	0	0	1	1	0	0	0	1	0	0	0	1	0	0	0	1
**Thermacetogenium**	1	7	0	1	0	0	0	0	0	0	0	0	0	0	0	0	0	0	0	0
**Thermaerobacter**	2	79	1	1	0	0	0	1	0	0	0	1	0	0	0	0	0	0	0	1
**Thermanaerovibrio**	2	58	0	1	0	0	0	0	0	0	0	0	0	0	0	0	0	0	0	0
**Thermincola**	2	62	1	1	0	0	0	0	0	0	0	0	0	0	0	0	0	0	0	0
**Thermoanaerobacter**	2	76	1	1	1	0	0	0	0	0	0	0	0	0	0	0	0	0	0	0
**Thermoanaerobacterium**	2	64	1	1	1	0	0	0	0	0	0	0	0	0	0	0	0	0	0	0
**Thermobacillus**	1	11	1	1	0	0	0	1	0	0	0	0	0	0	0	1	0	0	0	0
**Thermobaculum**	2	62	1	1	0	0	0	1	0	0	0	0	0	0	0	0	0	0	0	0
**Thermobifida**	2	72	1	1	0	0	0	1	0	0	0	0	1	0	0	0	0	0	0	0
**Thermobispora**	2	109	0	1	0	0	0	1	0	0	0	0	0	0	0	0	0	0	0	0
**Thermococcus**	1	13	0	1	0	0	0	0	0	0	0	0	0	0	0	1	0	0	0	0
**Thermocrinis**	2	60	0	1	0	0	0	0	0	0	0	0	0	0	0	1	0	0	0	0
**Thermodesulfatator**	1	10	0	1	0	0	0	0	0	0	0	0	0	0	0	1	0	0	0	0
**Thermodesulfobacterium**	1	9	0	1	1	0	0	0	0	0	0	0	0	0	0	1	0	0	0	0

Genus	Cluster Number	Protein Counts	K00074	K00088	K00100	K00106	K00114	K00128	K00129	K00132	K00141	K00146	K00148	K00152	K00155	K00169	K00217	K00224	K00274	K00446
**Thermodesulfobium**	1	13	0	1	1	0	0	0	0	0	0	0	0	0	0	1	0	0	0	0
**Thermodesulfovibrio**	2	60	0	1	1	0	0	0	0	0	0	0	0	0	0	1	0	0	0	0
**Thermofilum**	1	9	0	1	1	0	0	0	0	0	0	0	0	0	0	1	0	0	0	0
**Thermogladius**	1	7	0	0	0	0	0	0	0	0	0	0	0	0	0	1	0	0	0	0
**Thermomicrobium**	2	92	0	1	0	0	0	1	0	0	0	0	0	0	0	0	0	0	0	1
**Thermomonospora**	2	125	1	1	0	0	0	1	0	0	0	0	0	0	0	0	0	0	1	1
**Thermoplasma**	1	11	1	1	0	0	0	1	0	0	0	0	0	0	1	1	0	0	0	0
**Thermoplasmatales**	1	6	0	1	0	0	0	0	0	0	0	0	0	0	0	1	0	0	0	0
**Thermoproteus**	1	12	0	0	0	0	0	0	0	0	0	0	0	0	0	1	0	0	0	0
**Thermosediminibacter**	2	64	0	1	0	0	0	0	0	0	0	0	0	0	0	1	0	0	1	0
**Thermosipho**	2	70	0	1	1	0	0	0	0	0	0	0	0	0	0	1	0	0	0	0
**Thermosphaera**	1	10	0	0	0	0	0	0	0	0	0	0	0	0	0	1	0	0	0	0
**Thermosynechococcus**	1	6	0	1	0	0	0	0	0	0	0	0	0	0	0	0	0	0	0	0
**Thermotoga**	2	80	0	1	1	0	0	0	0	0	0	0	0	0	0	1	0	0	0	0
**Thermovibrio**	2	58	0	1	0	0	0	0	0	0	0	0	0	0	0	1	0	0	0	0
**Thermovirga**	1	9	0	1	0	0	0	0	0	0	0	0	0	0	0	1	0	0	0	0
**Thermus**	2	107	1	1	1	0	0	1	0	0	0	0	0	0	0	0	0	0	0	1
**Thioalkalimicrobium**	1	4	0	1	0	0	0	0	0	0	0	0	0	0	0	0	0	0	0	0
**Thioalkalivibrio**	2	91	0	1	0	0	0	0	0	0	0	0	0	0	0	1	0	0	0	0
**Thiobaca**	1	2	0	0	0	0	0	0	0	0	0	0	0	0	0	0	0	0	0	0
**Thiobacillus**	2	67	0	1	1	0	0	0	0	0	0	0	0	0	0	0	0	0	0	0
**Thiocapsa**	1	2	0	0	0	0	0	0	0	0	0	0	0	0	0	0	0	0	0	0
**Thiocystis**	1	11	0	1	0	0	0	0	0	0	0	0	0	0	0	0	0	0	0	0
**Thioflavicoccus**	1	13	0	1	0	0	0	0	0	0	0	0	0	0	0	0	0	0	0	0
**Thiomicrospira**	2	50	0	1	0	0	0	0	0	0	0	0	0	0	0	0	0	0	0	0
**Thiomonas**	2	85	1	1	0	0	1	0	0	0	0	0	0	0	0	0	0	0	0	0
**Thiorhodococcus**	1	2	0	0	0	0	0	0	0	0	0	0	0	0	0	0	0	0	0	0
**Thiothrix**	1	2	0	0	0	0	0	1	0	0	0	0	0	0	0	0	0	0	0	0
**Tistrella**	1	42	1	1	0	0	0	0	0	0	0	0	0	0	0	0	0	0	0	0
**Tolumonas**	2	100	0	1	1	0	0	0	0	0	0	0	0	0	0	0	0	0	0	0
**Transposon**	1	2	0	0	0	0	0	0	0	0	0	0	0	0	0	0	0	0	0	0
**Treponema**	1	63	1	1	1	0	0	1	0	0	0	0	0	0	0	0	0	0	0	1
**Trichodesmium**	2	71	0	1	0	0	0	1	0	0	0	0	0	0	0	0	0	0	1	0
**Trichormus**	1	12	0	1	0	0	0	1	0	0	0	0	0	0	0	0	0	0	0	0
**Tropheryma**	2	22	0	1	1	0	0	0	0	0	0	0	0	0	0	0	0	0	0	0
**Truepera**	2	104	1	1	0	0	0	1	0	0	0	0	0	0	0	0	0	0	1	0
**Tsukamurella**	2	123	1	1	0	0	0	1	0	0	0	0	0	0	0	0	0	0	1	0
**Turneriella**	1	16	1	1	0	0	0	0	0	0	0	0	0	0	0	0	0	0	1	0
**Uliginosibacterium**	1	2	0	0	0	0	0	0	0	0	0	0	0	0	0	0	0	0	0	0
**Ureaplasma**	1	24	0	0	0	0	0	0	0	0	0	0	0	0	0	0	0	0	0	0
**Ureibacillus**	1	2	0	0	0	0	0	0	0	0	0	0	0	0	0	0	0	0	0	0
**Variovorax**	3	187	1	1	1	0	1	1	0	0	0	1	0	0	0	0	0	0	0	0
**Veillonella**	1	54	0	1	0	0	0	0	0	0	0	0	0	0	0	0	0	0	0	0
**Verminephrobacter**	3	188	1	1	1	0	1	1	0	0	0	0	1	0	1	0	1	0	1	1
**Verrucosispora**	3	126	1	1	0	0	0	1	0	0	1	0	1	0	0	0	0	0	1	0
**Vibrio**	2	195	0	1	1	0	0	1	0	0	0	0	1	0	0	0	0	0	1	0
**Vibrionales**	1	46	0	1	0	0	0	0	0	0	0	0	0	0	0	0	0	0	0	0
**Vitreoscilla**	1	1	0	0	0	0	0	0	0	0	0	0	0	0	0	0	0	0	0	0
**Vulcanisaeta**	1	14	1	0	0	0	0	1	0	0	0	0	0	0	0	1	0	0	0	0
**Waddlia**	2	68	0	1	0	0	0	0	0	0	0	0	0	0	0	0	0	0	0	0
**Wautersia**	1	2	0	0	0	0	0	0	0	0	0	0	0	0	0	0	0	0	0	0
**Weeksella**	2	65	1	1	0	0	0	1	0	0	0	0	0	0	0	0	0	0	0	0

Genus	Cluster Number	Protein Counts	K00074	K00088	K00100	K00106	K00114	K00128	K00129	K00132	K00141	K00146	K00148	K00152	K00155	K00169	K00217	K00224	K00274	K00446
**Weissella**	1	14	0	0	0	0	0	0	0	0	0	0	0	0	0	0	0	0	0	0
**Wenxinia**	1	2	0	0	0	0	0	0	0	0	0	0	0	0	0	0	0	0	0	0
**Wigglesworthia**	1	29	0	1	0	0	0	0	0	0	0	0	0	0	0	0	0	0	0	0
**Wolbachia**	1	32	0	1	0	0	0	0	0	0	0	0	0	0	0	0	0	0	0	0
**Wolinella**	2	51	0	1	1	0	0	0	0	0	0	0	0	0	0	1	0	1	0	0
**Xanthobacter**	3	176	1	1	0	0	1	1	0	0	0	0	0	0	0	0	0	0	1	0
**Xanthobacteraceae**	1	4	0	0	0	0	0	0	0	0	0	0	0	0	0	0	0	0	0	0
**Xanthomonadaceae**	1	39	0	1	0	0	0	0	0	0	0	0	0	0	0	0	0	0	0	0
**Xanthomonas**	2	162	1	1	1	0	0	1	0	0	1	0	0	0	0	0	0	0	1	0
**Xenorhabdus**	2	127	1	1	0	0	0	0	0	0	0	1	0	0	0	0	0	0	0	0
**Xylanimonas**	2	66	0	1	0	0	0	1	0	0	0	0	0	0	0	0	0	0	1	0
**Xylella**	2	52	0	1	0	0	0	0	0	0	0	0	0	0	0	0	0	0	0	0
**Yersinia**	2	145	0	1	1	0	0	1	0	0	0	0	0	0	0	0	0	0	0	0
**Yokenella**	2	61	0	1	0	0	0	0	0	0	0	0	0	0	0	0	0	0	0	0
**Zobellia**	1	28	1	1	0	0	0	1	0	0	0	0	0	0	0	0	0	0	1	0
**Zunongwangia**	2	98	1	1	0	0	0	1	0	0	0	0	0	0	0	0	0	0	0	0
**Zymomonas**	2	67	0	1	0	0	0	0	0	0	0	0	0	0	0	0	0	0	0	0
**Zymophilus**	1	2	0	0	0	0	0	0	0	0	0	0	0	0	0	0	0	0	0	0

Genus	Cluster Number	Protein Counts	K00448	K00451	K00462	K00480	K00481	K00485	K00492	K00493	K00531	K00539	K00569	K00599	K00622	K00626	K00632	K00680	K00757	K00758
Acaricomes	1	2	0	0	0	0	0	0	0	0	0	0	0	0	0	0	0	0	0	0
Acaryochloris	2	88	0	0	0	1	0	0	0	0	0	0	0	0	0	0	1	0	1	0
Acetivibrio	1	2	0	0	0	0	0	0	0	0	0	0	0	0	0	0	0	0	0	0
Acetobacter	2	85	0	0	0	0	0	0	0	0	0	0	1	0	0	0	0	0	0	0
Acetobacteraceae	1	2	0	0	0	0	0	0	0	0	0	0	0	0	0	0	0	0	0	0
Acetobacterium	1	8	0	0	0	0	0	0	0	0	1	0	0	0	0	0	0	0	0	0
Acetohalobium	2	79	0	0	0	0	0	0	0	0	0	0	0	0	0	1	0	0	0	0
Acholeplasma	2	38	0	0	0	0	0	0	0	0	0	0	0	1	0	1	0	0	0	0
Achromobacter	2	192	0	1	1	1	0	0	1	0	0	0	1	0	0	1	1	0	0	1
Acidaminococcus	1	65	0	0	0	0	0	0	0	0	0	0	0	0	0	1	0	0	1	0
Acidianus	1	9	0	0	0	0	0	0	0	0	0	0	0	0	0	1	0	0	0	0
Acidilobus	1	8	0	0	0	0	0	0	0	0	0	0	0	0	0	1	0	0	1	0
Acidimicrobium	2	58	0	1	0	0	0	0	0	0	0	0	0	0	0	1	1	0	0	0
Acidiphilium	3	144	1	0	0	0	1	0	0	0	0	0	0	0	0	1	1	0	0	0
Acidithiobacillus	2	70	0	0	0	0	0	0	0	0	0	0	0	0	0	0	0	0	0	0
Acidobacterium	2	91	0	1	0	0	0	0	0	0	0	0	0	0	0	1	1	0	0	0
Acidocella	1	2	0	0	0	0	0	0	0	0	0	0	0	0	0	0	0	0	0	0
Acidothermus	2	68	0	0	0	0	0	0	0	0	0	0	0	1	0	1	0	0	0	0
Acidovorax	3	217	0	0	0	1	1	0	1	0	0	0	0	1	0	1	1	0	0	1
Aciduliprofundum	1	11	0	0	0	0	0	0	0	0	0	0	0	0	0	1	0	0	1	1
Acinetobacter	3	188	1	0	1	1	1	0	1	0	0	0	1	1	0	1	1	1	0	0
Actinobacillus	2	112	0	0	0	0	0	0	0	0	0	0	0	0	0	0	1	1	1	0
Actinomadura	1	1	0	0	0	0	0	0	0	0	0	0	0	0	0	0	0	0	0	0
Actinoplanes	1	52	1	1	0	1	1	0	0	0	0	0	1	0	0	1	0	0	0	1
Actinopolyspora	1	2	0	0	0	0	0	0	0	0	0	0	0	0	0	0	0	0	0	0
Actinosynnema	2	122	1	1	0	0	1	0	0	0	0	0	1	1	0	1	1	0	1	1
Advenella	1	30	0	1	0	1	0	0	0	0	0	0	0	0	0	1	0	0	0	0
Aequorivita	1	12	0	1	0	0	0	0	0	0	0	0	0	0	0	1	0	0	1	0
Aerococcus	2	57	0	0	0	0	0	0	0	0	0	0	0	1	0	1	0	0	0	0
Aeromonas	2	127	0	1	0	0	0	0	0	0	0	0	1	0	0	1	1	1	1	1
Aeropyrum	1	13	0	0	0	0	0	0	0	0	0	0	0	1	0	1	1	0	1	0
Afipia	1	54	0	0	0	0	0	0	0	0	0	0	0	0	0	1	0	0	0	0
Aggregatibacter	1	81	0	0	0	0	0	0	0	0	0	0	0	0	0	1	0	0	1	0
Agrobacterium	2	242	1	1	0	1	1	1	1	1	0	0	0	1	0	1	1	1	1	1
Ahrensia	1	2	0	0	0	0	0	0	0	0	0	0	0	0	0	0	0	0	0	0
Akkermansia	2	47	0	0	0	0	0	0	0	0	0	0	0	0	0	0	0	0	0	0
Albidiferax	3	144	0	0	0	1	1	0	0	0	0	0	0	0	0	1	1	1	0	1
Alcaligenes	1	21	0	0	0	0	0	0	0	0	0	0	0	0	0	1	0	0	0	0
Alcanivorax	2	146	0	0	0	0	0	0	1	0	0	0	1	1	0	1	1	1	0	0
Alicycliphilus	3	152	0	0	0	1	0	0	0	0	0	0	0	0	0	1	1	0	0	1
Alicyclobacillus	2	91	0	0	0	0	1	0	0	0	0	0	0	1	0	1	1	0	0	0
Aliivibrio	2	118	0	0	0	0	0	0	0	0	0	0	1	1	0	0	1	1	1	1
Alishewanella	1	1	0	0	0	0	0	0	0	0	0	0	0	0	0	0	0	0	0	0
Alistipes	1	52	0	0	0	0	0	0	0	0	0	0	0	0	0	0	0	0	1	0
Alkalilimnicola	2	97	0	0	0	0	0	0	0	0	0	0	0	0	0	1	1	0	0	0
Alkaliphilus	2	62	0	0	0	0	0	0	0	0	0	0	0	1	0	1	0	0	1	0
Allochromatium	2	84	0	0	0	0	0	0	0	0	0	0	1	0	0	1	0	0	0	0
Alphaproteobacteria	1	3	0	0	0	0	0	0	0	0	0	0	0	0	0	0	0	0	0	1
Alteromonadales	1	12	0	0	0	0	0	0	0	0	0	0	0	0	0	0	1	0	0	0
Alteromonas	1	37	0	1	0	0	0	0	0	0	0	0	1	0	0	1	1	0	0	0
Aminobacter	1	4	0	0	0	0	0	0	0	0	0	0	0	0	0	0	0	0	0	0
Aminobacterium	2	59	0	0	0	0	0	0	0	0	0	0	0	1	0	1	0	0	0	0

Genus	Cluster Number	Protein Counts	K00448	K00451	K00462	K00480	K00481	K00485	K00492	K00493	K00531	K00539	K00569	K00599	K00622	K00626	K00632	K00680	K00757	K00758
Ammonifex	2	50	0	0	0	0	0	0	0	0	0	0	0	0	0	0	0	0	0	0
Amphibacillus	1	13	0	0	0	0	0	0	0	0	0	0	0	0	0	1	0	0	0	0
Amphritea	1	2	0	0	0	0	0	0	0	0	0	0	0	0	0	0	0	0	0	0
Amycolatopsis	2	201	1	1	0	1	1	0	0	0	0	0	0	1	0	1	1	0	1	1
Amycolicicoccus	1	43	0	1	0	0	0	0	0	0	0	0	0	1	0	1	0	0	0	1
Anabaena	2	92	0	0	0	0	0	0	0	1	0	0	0	1	0	0	0	0	0	0
Anaerobaculum	1	11	0	0	0	0	0	0	0	0	0	0	0	0	0	0	0	0	0	0
Anaerococcus	2	46	0	0	0	0	0	0	0	0	0	0	0	0	0	1	0	0	0	0
Anaerolinea	2	66	0	0	0	0	0	0	0	0	0	0	0	0	0	1	0	0	0	0
Anaeromyxobacter	3	117	0	1	0	0	0	0	0	0	0	0	0	1	0	1	1	0	0	1
Anaerostipes	1	44	0	0	0	0	0	0	0	0	0	0	0	0	0	1	0	0	0	0
Anaerotruncus	1	2	0	0	0	0	0	0	0	0	0	0	0	0	0	0	0	0	0	0
Anaplasma	1	35	0	0	0	0	0	0	0	0	0	0	0	1	0	0	0	0	0	0
Ancylobacter	1	3	0	0	0	0	0	0	0	0	0	0	0	0	0	0	0	0	0	0
Aneurinibacillus	1	1	0	0	0	0	0	0	0	0	0	0	0	0	0	0	0	0	0	0
Angomonas	1	1	0	0	0	0	0	0	0	0	0	0	0	0	0	0	0	0	0	0
Anoxybacillus	2	72	0	0	0	0	0	0	0	0	0	0	0	0	0	1	1	0	0	0
Aquifex	2	45	0	0	0	0	0	0	0	0	0	0	0	1	0	0	0	0	0	0
Aquimarina	1	2	0	0	0	0	0	0	0	0	0	0	0	0	0	0	1	0	0	0
Arcanobacterium	2	45	0	0	0	0	0	0	0	0	0	0	0	1	0	0	0	0	0	1
Archaeoglobus	1	14	0	0	0	0	0	0	0	0	0	0	0	1	0	1	1	0	0	1
Arcobacter	2	101	0	0	0	0	0	0	0	0	0	0	1	0	0	1	0	1	0	0
Aromatoleum	3	133	0	0	0	1	0	0	0	0	0	0	0	1	0	1	1	1	0	0
Arsenophonus	1	4	0	0	0	0	0	0	0	0	0	0	0	0	0	0	0	0	0	0
Arthrobacter	2	206	1	0	0	1	1	0	1	0	0	0	0	1	0	1	1	1	0	1
Arthrospira	2	67	0	0	0	0	0	0	0	0	0	0	0	0	0	1	0	0	0	0
Aster	1	10	0	0	0	0	0	0	0	0	0	0	0	0	0	0	0	0	0	0
Asticcacaulis	2	103	0	1	0	0	0	0	0	0	0	0	0	1	0	1	0	0	0	0
Atopobium	1	28	0	0	0	0	0	0	0	0	0	0	0	1	0	0	0	0	0	0
Aurantimonas	1	2	0	0	0	0	0	0	0	0	0	0	0	0	0	0	0	0	0	0
Avibacterium	1	17	0	0	0	0	0	0	0	0	0	0	0	0	0	0	0	0	0	0
Azoarcus	3	172	0	0	1	1	1	0	1	0	0	0	1	1	0	1	1	1	0	0
Azorhizobium	3	149	1	0	1	1	1	1	0	0	0	0	0	1	0	1	1	1	0	1
Azorhizophilus	1	4	0	0	0	0	0	0	0	0	1	0	0	0	0	0	0	0	0	0
Azospira	1	19	0	0	0	0	0	0	0	0	0	0	0	0	0	1	1	0	0	0
Azospirillum	2	198	1	1	0	1	1	0	0	0	1	0	0	0	0	1	1	0	0	1
Azotobacter	3	183	1	1	0	0	1	0	0	0	1	0	1	0	0	1	1	0	0	0
Bacillales	1	9	0	0	0	0	0	0	0	0	0	0	0	0	0	0	0	0	0	0
Bacilli	1	3	0	0	0	0	0	0	0	0	0	0	0	0	0	0	0	0	0	0
Bacillus	2	237	0	1	0	0	1	0	1	0	0	0	0	1	0	1	1	1	0	0
Bacteria	2	86	0	0	0	0	0	0	0	0	0	0	0	0	0	1	0	0	0	0
Bacteriovorax	2	98	0	1	0	0	0	0	0	0	0	0	1	0	0	1	0	0	0	0
Bacteroidales	2	59	0	0	0	0	0	0	0	0	0	0	0	0	0	0	0	0	1	0
Bacteroides	2	96	0	0	0	0	0	0	0	0	0	0	0	1	0	0	0	1	1	0
Bacteroidetes	1	2	0	0	0	0	0	0	0	0	0	0	0	0	0	0	0	0	0	0
Bartonella	2	50	0	0	0	1	0	0	0	0	0	0	0	1	0	0	0	1	0	0
Baumannia	2	24	0	0	0	0	0	0	0	0	0	0	0	0	0	0	0	0	0	0
Bdellovibrio	2	87	0	1	0	0	0	0	0	0	0	0	0	1	0	1	1	1	0	0
Beijerinckia	3	123	0	0	0	1	0	0	0	0	0	0	0	0	0	1	1	0	0	0
Belliella	1	14	0	1	0	0	0	0	0	0	0	0	0	0	0	1	0	0	1	0
Beutenbergia	2	82	0	0	0	0	0	0	0	0	0	0	0	1	0	1	1	0	0	1
Bifidobacterium	2	61	0	0	0	0	0	0	0	0	0	0	0	1	0	0	0	0	0	0

Genus	Cluster Number	Protein Counts	K00448	K00451	K00462	K00480	K00481	K00485	K00492	K00493	K00531	K00539	K00569	K00599	K00622	K00626	K00632	K00680	K00757	K00758
**Bizionia**	1	1	0	0	0	0	0	0	0	0	0	0	0	0	0	0	0	0	0	0
**Blastococcus**	1	39	1	1	0	1	1	0	0	0	0	0	0	0	0	1	0	0	0	1
**Blattabacterium**	1	23	0	0	0	0	0	0	0	0	0	0	0	0	0	0	0	0	0	0
**Blautia**	1	22	0	0	0	0	0	0	0	0	0	0	0	0	0	0	0	0	0	0
**Bordetella**	3	227	1	1	0	1	0	1	0	0	0	0	1	0	0	1	1	0	0	0
**Borrelia**	1	32	0	0	0	0	0	0	0	0	0	0	0	1	0	1	0	0	0	0
**Brachybacterium**	1	18	0	0	0	0	0	0	1	0	0	0	0	1	0	1	0	0	0	1
**Brachymonas**	1	4	0	0	0	0	0	0	0	0	0	0	0	0	0	0	0	0	0	1
**Brachyspira**	2	79	0	0	0	0	0	0	0	0	0	0	0	0	0	1	0	0	1	0
**Bradyrhizobiaceae**	1	20	0	0	0	0	0	0	0	0	0	0	0	0	0	0	0	0	0	0
**Bradyrhizobium**	3	254	1	1	1	1	1	1	1	1	0	0	0	1	0	1	0	1	0	1
**Brenneria**	1	20	0	0	0	0	0	0	0	0	0	0	0	0	0	0	0	0	0	0
**Brevibacillus**	2	122	0	1	0	0	0	0	0	0	0	0	0	0	0	1	1	0	1	0
**Brevibacterium**	1	4	0	0	0	0	0	0	0	0	0	0	0	0	0	0	0	0	0	0
**Brevundimonas**	2	105	0	1	0	0	1	0	0	0	0	0	0	1	0	1	0	0	0	0
**Brucella**	3	138	1	0	1	1	1	0	1	0	0	0	0	1	0	1	1	1	0	0
**Buchnera**	1	26	0	0	0	0	0	0	0	0	0	0	0	0	0	0	0	0	0	0
**Burkholderia**	3	424	1	1	1	1	1	1	1	1	0	0	0	1	0	1	1	1	0	1
**Burkholderiaceae**	3	143	0	0	0	0	0	0	1	0	0	0	1	0	0	1	1	1	0	0
**Burkholderiales**	1	24	0	0	0	1	0	0	0	0	0	0	0	0	0	0	0	0	0	0
**Buttiauxella**	1	2	0	0	0	0	0	0	0	0	0	0	0	0	0	0	0	0	0	0
**Butyrivibrio**	2	67	0	0	0	0	0	0	0	0	0	0	0	1	0	1	0	0	1	0
**Caldanaerobacter**	2	63	0	0	0	0	0	0	0	0	0	0	0	1	0	1	0	1	0	0
**Caldicellulosiruptor**	2	75	0	0	0	0	0	0	0	0	0	0	0	0	0	0	0	0	0	0
**Caldilinea**	1	23	0	0	0	0	0	0	0	0	0	0	0	0	0	1	0	0	0	0
**Caldimonas**	1	6	0	0	0	0	0	0	0	0	0	0	0	0	0	0	0	0	0	0
**Caldisericum**	1	10	0	0	0	0	0	0	0	0	0	0	0	0	0	0	0	0	1	0
**Caldisphaera**	1	7	0	0	0	0	0	0	0	0	0	0	0	0	0	1	0	0	1	0
**Calditerrivibrio**	2	74	0	0	0	0	0	0	0	0	0	0	0	0	0	1	1	0	0	0
**Caldivirga**	1	13	0	0	0	0	0	0	0	0	0	0	0	0	0	1	0	0	1	0
**Calothrix**	1	43	0	0	0	1	0	0	0	0	0	0	0	0	0	0	0	0	1	0
**Calyptogena**	2	34	0	0	0	0	0	0	0	0	0	0	0	0	0	0	0	0	0	0
**Campylobacter**	2	80	0	0	0	0	0	0	0	0	0	0	0	1	0	0	0	1	0	0
**Candidatus**	2	290	1	1	1	1	1	0	0	0	0	0	1	1	0	1	1	0	1	1
**Capnocytophaga**	1	50	0	0	0	0	0	0	0	0	0	0	0	0	0	0	0	1	1	0
**Carbophilus**	1	2	0	0	0	0	0	0	0	0	0	0	0	0	0	0	0	0	0	0
**Carboxydibrachium**	1	14	0	0	0	0	0	0	0	0	0	0	0	1	0	0	0	0	0	0
**Carboxydothermus**	2	68	0	0	0	0	0	0	0	0	0	0	0	0	0	1	1	0	0	0
**Carica**	1	2	0	0	0	0	0	0	0	0	0	0	0	0	0	0	0	0	0	0
**Carnobacterium**	2	71	0	0	0	0	0	0	0	0	0	0	0	0	0	1	1	1	0	0
**Catenibacterium**	1	2	0	0	0	0	0	0	0	0	0	0	0	0	0	0	0	0	0	0
**Catenovulum**	1	2	0	0	0	0	0	0	0	0	0	0	0	0	0	0	0	0	0	0
**Catenulispora**	3	159	1	1	0	0	1	0	0	0	0	0	0	1	0	1	1	0	0	1
**Caulobacter**	3	168	1	1	0	0	1	0	0	0	0	0	0	1	0	1	1	0	0	1
**Cedecea**	1	45	0	0	0	0	0	0	0	0	0	0	0	0	0	0	1	0	1	1
**Cellulomonas**	2	96	0	0	0	1	0	0	0	0	0	0	0	1	0	1	1	0	0	1
**Cellulophaga**	2	105	0	1	0	0	0	0	0	0	0	0	0	0	0	1	1	0	1	0
**Cellulosilyticum**	1	14	0	0	0	0	0	0	0	0	0	0	0	1	0	0	0	0	0	0
**Cellvibrio**	2	85	0	0	0	0	0	0	0	0	0	0	0	0	0	0	1	0	0	0
**Cenarchaeum**	1	7	0	0	0	0	0	0	0	0	0	0	0	0	0	1	0	0	0	0
**Chamaesiphon**	1	13	0	0	0	0	0	0	0	0	0	0	0	0	0	0	0	0	0	0
**Chelativorans**	3	136	1	1	0	1	1	0	0	0	0	0	0	1	0	1	1	0	0	1

Genus	Cluster Number	Protein Counts	K00448	K00451	K00462	K00480	K00481	K00485	K00492	K00493	K00531	K00539	K00569	K00599	K00622	K00626	K00632	K00680	K00757	K00758
**Chelatococcus**	1	2	0	1	0	0	0	0	0	0	0	0	0	0	0	0	0	0	0	0
**Chitinophaga**	2	98	0	1	0	0	1	0	0	0	0	0	0	0	0	1	1	0	1	0
**Chlamydia**	1	36	0	0	0	0	0	0	0	0	0	0	0	1	0	0	0	0	0	0
**Chlamydomonas**	1	3	0	0	0	0	0	0	0	0	0	0	0	0	0	0	0	0	0	0
**Chlamydophila**	1	31	0	0	0	0	0	0	0	0	0	0	0	1	0	0	0	0	0	0
**Chlorobaculum**	2	61	0	0	0	0	0	0	0	0	0	0	0	0	0	0	0	0	0	0
**Chlorobium**	2	89	0	0	0	0	0	0	0	0	0	0	0	0	0	0	0	0	0	0
**Chlorobium/Pelodictyon**	1	2	0	0	0	0	0	0	0	0	0	0	0	0	0	0	0	0	0	0
**Chloroflexus**	2	105	0	1	0	0	0	0	0	0	0	0	0	1	0	1	1	0	0	0
**Chlorogloeopsis**	1	9	0	0	0	0	0	0	0	0	0	0	0	0	0	0	0	0	0	0
**Chloroherpeton**	2	59	0	0	0	0	0	0	0	0	1	0	0	0	0	0	0	0	0	0
**Chromobacterium**	2	117	0	1	0	0	0	0	0	0	0	0	0	1	0	1	1	1	0	1
**Chromohalobacter**	2	145	1	0	0	0	1	1	0	0	0	0	1	0	0	1	1	0	0	1
**Chroococcidiopsis**	1	24	0	1	0	0	0	0	0	0	0	0	0	0	0	0	0	0	1	0
**Chryseobacterium**	2	104	0	0	0	1	1	0	0	0	0	0	0	0	0	1	1	0	0	0
**Citreicella**	1	13	0	0	0	0	0	0	0	0	0	0	0	0	0	1	0	0	0	0
**Citrobacter**	2	190	1	0	0	1	1	0	0	0	0	0	0	0	0	1	1	1	1	1
**Clavibacter**	2	83	0	0	0	0	0	0	0	0	0	0	0	1	0	0	1	1	0	1
**Clonorchis**	1	1	0	0	0	0	0	0	0	0	0	0	0	0	0	0	0	0	0	0
**Clostridiales**	2	77	0	0	0	0	0	0	0	0	0	0	0	0	0	1	0	0	1	0
**Clostridium**	2	191	0	0	0	0	0	0	0	0	1	0	0	1	0	1	0	1	1	1
**Cohnella**	1	2	0	0	0	0	0	0	0	0	0	0	0	0	0	0	0	0	0	0
**Coleofasciculus**	1	4	0	0	0	0	0	0	0	0	0	0	0	0	0	0	0	0	0	0
**Collimonas**	1	35	1	1	0	0	1	0	0	0	0	0	1	0	0	1	1	0	0	0
**Colwellia**	2	130	0	0	0	0	0	0	0	0	0	0	1	0	0	1	1	1	0	1
**Comamonas**	2	196	0	0	0	1	1	0	0	0	0	0	0	0	0	1	1	0	0	1
**Conexibacter**	3	117	0	1	0	1	0	0	0	0	0	0	0	0	0	1	1	0	0	0
**Coprobacillus**	1	2	0	0	0	0	0	0	0	0	0	0	0	0	0	0	0	0	0	0
**Coprococcus**	2	76	0	0	0	0	0	0	0	0	0	0	0	0	0	1	0	0	1	0
**Coprothermobacter**	2	45	0	0	0	0	0	0	0	0	0	0	0	0	0	1	0	0	1	0
**Coraliomargarita**	2	68	0	0	0	0	0	0	0	0	0	0	0	0	0	0	0	0	0	0
**Corallococcus**	1	29	0	1	0	0	0	0	0	0	0	0	0	0	0	1	0	0	0	0
**Coriobacterium**	2	36	0	0	0	0	0	0	0	0	0	0	0	1	0	0	0	0	1	0
**Corynebacterineae**	1	5	0	0	0	0	0	0	0	0	0	0	0	0	0	1	0	0	0	0
**Corynebacterium**	2	175	1	0	0	1	1	1	1	0	0	0	0	1	0	1	1	1	0	1
**Coxiella**	2	54	0	0	0	0	0	0	0	0	0	0	0	1	0	1	0	0	1	0
**Crinalium**	1	21	0	1	0	1	0	0	0	0	0	0	1	0	0	1	0	0	0	0
**Croceibacter**	2	80	0	1	0	0	0	0	0	0	0	0	0	0	0	1	1	0	1	0
**Crocosphaera**	1	6	0	0	0	0	0	0	0	0	0	0	0	0	0	0	0	0	0	0
**Cronobacter**	2	120	0	0	0	0	0	0	0	0	0	0	0	0	0	1	1	0	1	1
**Cryptobacterium**	2	39	0	0	0	0	0	0	0	0	0	0	0	1	0	0	0	0	0	0
**Cucumis**	1	9	0	0	0	0	0	0	0	0	0	0	0	0	0	0	0	0	0	0
**Cupriavidus**	3	317	1	1	1	1	1	0	1	1	0	0	1	1	0	1	1	1	0	1
**Curtobacterium**	1	5	0	0	0	0	0	0	0	0	0	0	0	0	0	0	0	0	0	0
**Curvibacter**	1	27	0	0	0	0	0	0	0	0	0	0	0	0	0	0	1	0	0	0
**Cyanobacterium**	1	9	0	0	0	1	0	0	0	0	0	0	0	0	0	0	0	0	0	0
**Cyanobium**	1	10	0	0	0	0	0	0	0	0	0	0	0	0	0	0	0	0	0	0
**Cyanothece**	1	34	0	1	0	1	0	0	0	0	0	0	0	0	0	1	1	0	1	0
**Cyclobacterium**	1	19	0	0	0	0	0	0	0	0	0	0	0	0	0	1	1	0	1	1
**Cycloclasticus**	1	21	0	0	0	0	0	0	0	0	0	0	1	0	0	1	0	0	0	0
**Cylindrospermopsis**	1	4	0	0	0	0	0	0	0	0	0	0	0	0	0	0	0	0	0	0
**Cylindrospermum**	1	30	0	0	0	0	0	0	0	0	0	0	0	0	0	0	0	0	0	0

Genus	Cluster Number	Protein Counts	K00448	K00451	K00462	K00480	K00481	K00485	K00492	K00493	K00531	K00539	K00569	K00599	K00622	K00626	K00632	K00680	K00757	K00758
**Cytophaga**	2	69	0	0	0	0	0	0	0	0	0	0	0	1	0	1	1	1	0	0
**Dactylococcopsis**	1	8	0	0	0	0	0	0	0	0	0	0	0	0	0	0	0	0	0	0
**Dechloromonas**	1	40	0	0	1	0	0	0	1	0	0	0	1	1	0	1	1	0	0	0
**Dechlorosoma**	1	19	0	0	0	0	0	0	0	0	0	0	0	0	0	1	1	0	0	0
**Deferribacter**	2	73	0	0	0	0	0	0	0	0	0	0	0	0	0	1	1	0	0	0
**Dehalobacter**	1	9	0	0	0	0	0	0	0	0	0	0	0	0	0	0	0	0	0	0
**Dehalococcoides**	2	41	0	0	0	0	0	0	0	0	0	0	0	1	0	0	0	0	0	0
**Dehalogenimonas**	1	37	0	0	0	0	0	0	0	0	0	0	0	0	0	0	0	0	0	0
**Deinococcus**	2	140	1	0	0	0	1	0	0	1	0	0	0	0	0	1	1	0	0	1
**Delftia**	3	177	0	0	0	1	1	0	0	0	0	0	0	0	0	1	1	0	0	0
**Denitrovibrio**	2	67	0	0	0	0	0	0	0	0	0	0	0	0	0	1	0	0	0	0
**Desulfarculus**	2	92	0	0	0	0	0	0	0	0	0	0	0	0	0	1	1	0	0	0
**Desulfatibacillum**	3	84	0	0	0	0	0	0	0	0	0	0	0	0	0	1	1	0	0	0
**Desulfitobacterium**	2	102	0	0	0	0	0	0	0	0	0	0	0	1	0	1	1	0	0	0
**Desulfobacca**	2	65	0	0	0	0	0	0	0	0	0	0	0	0	0	0	0	0	1	0
**Desulfobacterium**	2	113	0	0	0	0	0	0	0	0	0	0	0	0	0	1	1	0	0	0
**Desulfobacula**	1	21	0	0	0	0	0	0	0	0	0	0	0	0	0	1	0	0	0	0
**Desulfobulbus**	2	88	0	0	0	0	0	0	0	0	0	0	0	0	0	1	0	0	0	0
**Desulfocapsa**	1	9	0	0	0	0	0	0	0	0	0	0	0	0	0	0	0	0	0	0
**Desulfococcus**	2	80	0	0	0	0	0	0	0	0	0	0	0	0	0	1	1	0	0	0
**Desulfohalobium**	2	52	0	0	0	0	0	0	0	0	0	0	0	0	0	0	0	0	0	0
**Desulfomicrobium**	2	65	0	0	0	0	0	0	0	0	0	0	0	0	0	0	0	1	1	0
**Desulfomonile**	1	25	0	0	0	0	0	0	0	0	0	0	0	0	0	1	0	0	0	0
**Desulforhabdus**	1	2	0	0	0	0	0	0	0	0	0	0	0	0	0	0	0	0	0	0
**Desulfosporosinus**	1	30	0	0	0	0	0	0	0	0	0	0	0	1	0	1	0	0	0	0
**Desulfotalea**	2	66	0	0	0	0	0	0	0	0	0	0	0	0	0	0	0	0	0	0
**Desulfotomaculum**	2	98	0	0	0	0	0	0	0	0	0	0	0	0	0	1	1	0	0	0
**Desulfovibrio**	2	144	0	0	0	0	0	0	0	0	0	0	0	1	0	0	0	0	0	1
**Desulfurispirillum**	2	78	0	0	0	0	0	0	0	0	0	0	0	0	0	1	1	0	0	0
**Desulfurivibrio**	2	71	0	0	0	0	0	0	0	0	0	0	0	0	0	0	0	0	0	0
**Desulfurobacterium**	2	53	0	0	0	0	0	0	0	0	0	0	0	0	0	0	0	0	0	0
**Desulfurococcus**	1	11	0	0	0	0	0	0	0	0	0	0	0	0	0	1	0	0	1	1
**Diaphorobacter**	1	16	0	0	0	0	0	0	0	0	0	0	0	0	0	0	0	0	0	0
**Dichelobacter**	2	45	0	0	0	0	0	0	0	0	0	0	0	0	0	0	0	0	0	1
**Dickeya**	2	155	0	0	0	0	0	0	0	0	1	0	0	0	0	1	1	1	1	1
**Dictyoglomus**	2	62	0	0	0	0	0	0	0	0	0	0	0	0	0	0	0	0	0	0
**Dinoroseobacter**	3	148	1	1	0	1	1	0	0	0	0	0	0	0	0	1	1	0	0	1
**Dokdonia**	1	3	0	0	0	0	0	0	0	0	0	0	0	0	0	0	1	0	0	0
**Dorea**	1	7	0	0	0	0	0	0	0	0	0	0	0	0	0	0	0	0	0	0
**Dyadobacter**	2	83	0	1	0	0	0	0	0	0	0	0	0	0	0	1	1	0	0	0
**Echinicola**	1	20	0	1	0	0	0	0	0	0	0	0	0	0	0	1	0	0	1	1
**Edwardsiella**	2	126	0	0	0	0	0	0	0	0	0	0	0	0	0	1	0	0	1	1
**Eggerthella**	1	51	0	0	0	0	0	0	0	0	0	0	0	1	0	0	0	0	0	0
**Ehrlichia**	2	37	0	0	0	0	0	0	0	0	0	0	0	1	0	0	0	1	0	0
**Eikenella**	1	4	0	0	0	0	0	0	0	0	0	0	0	0	0	0	0	0	0	0
**Elusimicrobium**	2	44	0	0	0	0	0	0	0	0	0	0	0	0	0	0	0	0	0	0
**Emticicia**	1	21	1	1	0	0	1	0	0	0	0	0	0	0	0	1	0	0	0	0
**Enhydrobacter**	1	1	0	0	0	0	0	0	0	0	0	0	0	0	0	0	0	0	0	0
**Ensifer**	1	2	0	0	0	0	0	0	0	0	0	0	0	0	0	0	0	0	0	0
**Enterobacter**	2	202	1	0	0	1	1	0	0	0	0	0	0	1	0	1	1	1	1	1
**Enterobacteriaceae**	2	211	1	0	0	1	1	0	0	0	0	0	0	0	0	1	1	0	1	1
**Enterococcus**	1	187	1	0	0	0	1	0	0	0	0	0	1	1	0	1	1	1	1	1

Genus	Cluster Number	Protein Counts	K00448	K00451	K00462	K00480	K00481	K00485	K00492	K00493	K00531	K00539	K00569	K00599	K00622	K00626	K00632	K00680	K00757	K00758
Enterovibrio	1	15	0	0	0	0	0	0	0	0	0	0	0	0	0	0	0	0	0	0
Erwinia	2	151	0	0	0	0	1	0	0	0	0	0	0	0	0	1	1	0	1	1
Erysipelothrix	1	9	0	0	0	0	0	0	0	0	0	0	0	0	0	1	0	0	0	0
Erysipelotrichaceae	1	4	0	0	0	0	0	0	0	0	0	0	0	0	0	0	0	0	0	0
Erythrobacter	2	80	0	0	0	0	0	0	0	0	0	0	0	0	0	1	0	0	0	0
Escherichia	2	242	1	0	0	1	1	0	0	0	0	0	0	0	0	1	1	1	1	1
Ethanoligenens	2	69	0	0	0	0	0	0	0	0	1	0	0	0	0	0	0	0	1	0
Eubacteriaceae	1	2	0	0	0	0	0	0	0	0	0	0	0	0	0	0	0	0	0	0
Eubacterium	2	103	0	0	0	0	0	0	0	0	0	0	0	1	0	1	0	0	1	0
Eudoraea	1	4	0	0	0	0	0	0	0	0	0	0	0	0	0	0	0	0	0	0
Euryarchaeota	1	1	0	0	0	0	0	0	0	0	0	0	0	0	0	1	0	0	0	0
Exiguobacterium	2	93	0	0	0	0	0	0	0	0	0	0	0	1	0	1	1	1	0	0
Faecalibacterium	1	12	0	0	0	0	0	0	0	0	0	0	0	0	0	1	0	0	0	0
Ferrimonas	2	115	0	1	0	0	0	0	0	0	0	0	1	0	0	1	1	0	1	1
Ferroglobus	1	14	0	0	0	0	0	0	0	0	0	0	0	0	0	1	0	0	0	1
Ferroplasma	1	11	0	1	0	0	0	0	0	0	0	0	0	0	0	1	0	0	0	0
Fervidicoccus	1	7	0	0	0	0	0	0	0	0	0	0	0	0	0	1	0	0	1	0
Fervidobacterium	2	54	0	0	0	0	0	0	0	0	0	0	0	0	0	1	0	0	0	0
Fibrella	1	17	0	1	0	0	0	0	0	0	0	0	0	0	0	1	0	0	0	0
Fibrobacter	2	52	0	0	0	0	0	0	0	0	0	0	0	0	0	0	0	0	0	0
Filifactor	1	9	0	0	0	0	0	0	0	0	0	0	0	0	0	1	0	0	0	0
Finegoldia	2	55	0	0	0	0	0	0	0	0	0	0	0	0	0	0	0	0	1	0
Firmicutes	1	15	0	0	0	0	0	0	0	0	0	0	0	0	0	0	0	1	0	0
Fischerella	1	15	0	0	0	0	0	0	0	0	0	0	0	0	0	0	0	0	0	0
Flavobacteria	1	7	0	0	0	0	0	0	0	0	0	0	0	0	0	0	0	0	0	0
Flavobacteriaceae	2	69	0	1	0	0	0	0	0	0	0	0	0	0	0	1	1	0	1	0
Flavobacteriales	1	3	0	0	0	0	0	0	0	0	0	0	0	0	0	0	0	0	0	0
Flavobacterium	2	103	0	1	0	0	0	0	0	0	0	0	0	0	0	1	1	1	1	0
Flexibacter	1	17	0	1	0	0	0	0	0	0	0	0	0	0	0	1	0	0	1	0
Flexistipes	1	11	0	0	0	0	0	0	0	0	0	0	0	0	0	1	1	0	0	0
Fluoribacter	1	6	0	0	0	0	0	0	0	0	0	0	0	0	0	0	0	0	0	0
Fluviicola	2	69	0	1	0	0	0	0	0	0	0	0	0	1	0	1	1	0	1	0
Formosa	1	2	0	0	0	0	0	0	0	0	0	0	0	0	0	0	0	0	0	0
Francisella	2	88	0	0	0	0	0	0	0	1	0	0	1	1	0	0	1	1	1	0
Frankia	3	202	1	1	0	1	1	0	0	1	0	0	0	1	0	1	1	1	0	1
Frateuria	1	25	0	0	0	1	0	0	0	0	0	0	0	0	0	1	1	0	0	0
Fructobacillus	1	2	0	0	0	0	0	0	0	0	0	0	0	0	0	0	0	0	0	0
Fulvimarina	1	2	0	0	0	0	0	0	0	0	0	0	0	0	0	0	0	0	0	0
Fusobacterium	1	48	0	0	0	0	0	0	0	0	0	0	0	1	0	1	0	1	0	0
Gallibacterium	2	87	0	0	0	0	0	0	0	0	0	0	0	0	0	0	1	0	1	0
Gallionella	2	67	0	0	0	0	0	0	0	0	0	0	0	0	0	0	1	0	0	0
Gammaproteobacteria	1	9	0	0	0	0	0	0	0	0	0	0	0	0	0	0	0	1	0	0
Gardnerella	1	31	0	0	0	0	0	0	0	0	0	0	0	1	0	1	0	0	0	0
Geitlerinema	1	9	0	0	0	0	0	0	0	0	0	0	0	0	0	0	0	0	0	0
Gemmatimonas	2	93	0	1	0	0	0	0	0	0	0	0	0	1	0	1	1	0	0	0
Geobacillus	2	168	0	0	0	1	0	0	0	0	0	0	0	1	0	1	1	0	0	0
Geobacter	2	150	0	0	0	0	0	0	0	0	0	0	0	0	0	1	1	0	1	0
Geodermatophilus	2	136	1	1	0	0	1	0	0	0	0	0	0	1	0	1	1	0	0	1
Geopsychrobacter	1	2	0	0	0	0	0	0	0	0	0	0	0	0	0	0	0	0	0	0
Gillisia	1	11	0	1	0	0	0	0	0	0	0	0	0	0	0	0	0	0	0	0
Gilvimarinus	1	4	0	0	0	0	0	0	0	0	0	0	0	0	0	0	0	0	0	0
Glaciecola	2	117	0	1	0	1	0	0	0	0	0	0	1	0	0	1	1	0	0	0

Genus	Cluster Number	Protein Counts	K00448	K00451	K00462	K00480	K00481	K00485	K00492	K00493	K00531	K00539	K00569	K00599	K00622	K00626	K00632	K00680	K00757	K00758
**Gloeobacter**	2	67	0	0	0	0	0	0	0	0	0	0	0	1	0	0	0	0	0	0
**Gloeocapsa**	1	20	0	0	0	0	0	0	0	0	0	0	0	0	0	0	0	0	0	0
**Gloeothece**	1	1	0	0	0	0	0	0	0	0	0	0	0	0	0	0	0	0	0	0
**Gluconacetobacter**	2	96	0	0	0	0	0	0	0	0	0	0	1	0	0	1	0	0	1	0
**Gluconobacter**	2	76	0	0	0	0	0	0	0	0	0	0	1	1	0	0	0	1	0	0
**Gordonia**	1	158	1	1	0	1	1	0	0	1	0	0	0	1	0	1	1	0	0	1
**Gordonibacter**	1	32	0	0	0	0	0	0	0	0	0	0	0	0	0	0	0	0	0	0
**Gramella**	2	104	0	1	0	0	0	0	0	0	0	0	1	1	0	1	1	1	0	0
**Granulibacter**	2	90	0	0	0	0	0	1	0	0	0	0	0	1	0	1	0	1	0	0
**Granulicella**	2	98	0	1	0	0	0	0	0	0	0	0	0	0	0	1	1	0	0	0
**Grimontia**	1	9	0	0	0	0	0	0	0	0	0	0	0	0	0	0	0	0	0	0
**Haemophilus**	2	117	0	0	0	0	0	0	1	0	0	0	0	0	0	1	1	0	1	0
**Hafnia**	1	29	0	0	0	0	0	0	0	0	0	0	0	0	0	0	0	0	1	0
**Hahella**	3	130	0	1	0	0	0	0	0	0	0	0	1	0	0	1	1	0	0	1
**Halalkalicoccus**	1	18	0	0	0	0	0	0	0	0	0	0	0	0	0	1	1	0	1	0
**Halanaerobium**	2	89	0	0	0	0	0	0	0	0	0	0	0	0	0	1	0	0	1	0
**Haliangium**	2	108	0	1	0	0	0	0	0	0	0	0	1	0	0	1	1	0	0	0
**Haliscomenobacter**	2	107	1	1	0	0	1	0	0	0	0	0	0	0	0	1	1	0	1	0
**Haloarcula**	1	22	0	0	0	0	1	0	0	0	0	0	0	1	0	1	1	0	1	0
**Halobacillus**	1	19	0	0	0	0	0	0	0	0	0	0	0	0	0	1	1	0	0	0
**Halobacteriaceae**	1	1	0	0	0	0	0	0	0	0	0	0	0	0	0	1	0	0	0	0
**Halobacterium**	1	10	0	0	0	0	0	0	0	0	0	0	0	0	0	0	1	0	1	0
**Halobacteroides**	1	14	0	0	0	0	0	0	0	0	0	0	0	0	0	1	0	0	0	0
**Haloferax**	1	20	0	0	0	0	0	0	0	0	0	0	0	0	0	1	1	0	1	1
**Halogeometricum**	1	12	0	0	0	0	0	0	0	0	0	0	0	0	0	1	1	0	1	1
**Halomicrobium**	1	10	0	0	0	0	0	0	0	0	0	0	0	0	0	1	1	0	1	0
**Halomonas**	1	149	1	1	0	0	1	0	0	0	0	0	1	0	0	1	1	0	0	1
**Halopiger**	1	16	0	0	0	0	0	0	0	0	0	0	0	0	0	1	1	0	1	0
**Haloquadratum**	1	19	0	0	0	0	0	0	0	0	0	0	0	1	0	1	1	1	1	0
**Halorhabdus**	1	10	0	0	0	0	0	0	0	0	0	0	0	0	0	1	0	0	1	0
**Halorhodospira**	2	71	0	0	0	0	0	0	0	0	0	0	0	0	0	1	0	0	0	0
**Halorubrum**	1	16	0	0	0	0	0	0	0	0	0	0	0	0	0	1	1	0	1	0
**Haloterrigena**	1	19	0	0	0	0	0	0	0	0	0	0	0	0	0	1	1	0	1	0
**Halothece**	1	9	0	0	0	0	0	0	0	0	0	0	0	0	0	0	0	0	0	0
**Halothermothrix**	2	50	0	0	0	0	0	0	0	0	0	0	0	0	0	0	0	0	0	0
**Halothiobacillus**	2	65	0	0	0	0	0	0	0	0	0	0	0	0	0	0	0	0	0	0
**Halovivax**	1	12	0	0	0	0	0	0	0	0	0	0	0	0	0	1	0	0	1	0
**Helicobacter**	2	79	0	0	0	0	0	0	0	0	0	0	0	1	0	1	0	1	0	0
**Heliobacillus**	1	4	0	0	0	0	0	0	0	0	0	0	0	0	0	0	0	0	0	0
**Heliobacterium**	2	51	0	0	0	0	0	0	0	0	0	0	0	0	0	1	1	0	0	0
**Herbaspirillum**	2	172	1	1	0	0	1	0	0	0	0	0	1	0	0	1	1	0	0	0
**Herminiimonas**	3	89	0	0	0	0	0	1	0	0	0	0	0	0	0	1	1	0	0	0
**Herpetosiphon**	3	90	0	1	0	0	0	0	0	0	0	0	1	1	0	1	1	0	0	0
**Hippea**	2	44	0	0	0	0	0	0	0	0	0	0	0	0	0	1	0	0	0	0
**Hirschia**	2	102	0	0	0	1	0	0	0	0	0	0	0	0	0	1	0	0	0	0
**Histophilus**	2	72	0	0	0	0	0	0	1	0	0	0	0	0	0	1	0	0	1	0
**Hoeflea**	1	16	0	0	0	0	0	0	0	0	0	0	0	0	0	0	1	0	0	0
**Hydrogenivirga**	1	2	0	0	0	0	0	0	0	0	0	0	0	0	0	0	0	0	0	0
**Hydrogenobacter**	2	55	0	0	0	0	0	0	0	0	0	0	0	0	0	0	0	0	0	0
**Hydrogenobaculum**	2	44	0	0	0	0	0	0	0	0	0	0	0	0	0	0	0	0	0	0
**Hydrogenophaga**	1	9	0	0	0	0	0	0	0	0	0	0	0	0	0	1	0	0	0	1
**Hylemonella**	1	4	0	0	0	0	0	0	0	0	0	0	0	0	0	0	0	0	0	0

Genus	Cluster Number	Protein Counts	K00448	K00451	K00462	K00480	K00481	K00485	K00492	K00493	K00531	K00539	K00569	K00599	K00622	K00626	K00632	K00680	K00757	K00758
**Hyperthermus**	1	6	0	0	0	0	0	0	0	0	0	0	0	0	0	0	1	0	1	1
**Hyphomicrobium**	2	93	0	0	0	1	0	0	0	0	0	0	1	0	0	1	0	0	0	0
**Hyphomonas**	3	105	0	0	0	1	0	0	0	0	0	0	0	0	0	1	0	1	0	0
**Ideonella**	1	18	0	0	0	0	0	0	0	0	0	0	0	0	0	0	0	0	0	0
**Idiomarina**	2	86	0	1	0	0	0	0	0	0	0	0	0	0	0	1	1	0	0	1
**Ignavibacterium**	1	14	0	1	0	0	0	0	0	0	0	0	0	0	0	1	0	0	0	0
**Ignicoccus**	1	5	0	0	0	0	0	0	0	0	0	0	0	0	0	1	0	0	0	0
**Ignisphaera**	1	8	0	0	0	0	0	0	0	0	0	0	0	0	0	1	0	0	0	0
**Ilyobacter**	2	85	0	0	0	0	0	0	0	0	0	0	0	1	0	1	0	0	0	0
**Intrasporangium**	2	114	0	1	0	1	0	0	0	0	0	0	0	1	0	1	1	0	0	1
**Isoptericola**	1	18	0	0	0	0	0	0	0	0	0	0	0	1	0	1	1	0	0	1
**Isosphaera**	2	75	0	0	0	0	0	0	0	0	0	0	0	1	0	1	0	0	0	0
**Jannaschia**	3	151	1	1	0	1	1	0	0	0	0	0	0	0	0	1	0	0	0	1
**Janthinobacterium**	2	99	0	0	0	0	0	0	0	0	0	0	0	0	0	1	1	0	0	0
**Jonesia**	2	56	0	0	0	0	0	0	0	0	0	0	0	1	0	1	1	0	0	1
**Joostella**	1	3	0	0	0	0	0	0	0	0	0	0	0	0	0	0	0	0	0	0
**Kaistia**	1	2	0	0	0	0	0	0	0	0	0	0	0	0	0	0	0	0	0	0
**Kangiella**	2	84	0	1	0	0	0	0	0	0	0	0	1	0	0	1	1	0	0	1
**Ketogulonicigenium**	2	102	1	0	0	1	1	0	0	0	0	0	0	0	0	0	0	0	0	1
**Kineococcus**	1	24	0	0	0	0	0	0	0	0	0	0	0	1	0	1	1	0	0	1
**Kinetoplastibacterium**	1	2	0	0	0	0	0	0	0	0	0	0	0	0	0	0	0	0	0	0
**Kingella**	1	4	0	0	0	0	0	0	0	0	0	0	0	0	0	0	0	0	0	0
**Kitasatospora**	1	36	0	1	0	1	1	0	0	0	0	0	0	0	0	1	0	0	0	1
**Klebsiella**	3	222	1	0	0	1	1	0	0	0	0	0	0	1	0	1	1	1	1	1
**Kluyvera**	1	1	0	0	0	0	0	0	0	0	0	0	0	0	0	0	0	0	0	0
**Kocuria**	2	87	1	0	0	0	0	0	1	0	0	0	0	1	0	1	0	0	0	1
**Kordia**	1	5	0	0	0	0	0	0	0	0	0	0	0	0	0	0	1	0	0	0
**Kosmotoga**	2	61	0	0	0	0	0	0	0	0	0	0	0	0	0	1	0	0	1	0
**Kribbella**	2	99	0	1	0	0	0	0	0	0	0	0	0	1	0	1	1	0	0	1
**Krokinobacter**	2	90	0	1	0	0	0	0	0	0	0	0	0	0	0	1	1	0	1	0
**Kyrpidia**	2	113	0	1	0	0	0	0	0	0	0	0	0	1	0	1	1	0	0	0
**Kytococcus**	2	70	0	1	0	0	0	0	0	0	0	0	0	1	0	1	0	0	0	1
**Labrenzia**	1	4	0	0	0	0	0	0	0	0	0	0	0	0	0	0	0	0	0	0
**Lachnoanaerobaculum**	1	1	0	0	0	0	0	0	0	0	0	0	0	0	0	0	0	0	0	0
**Lachnospiraceae**	1	51	0	0	0	0	0	0	0	0	0	0	0	0	0	1	0	1	0	0
**Lacinutrix**	1	20	0	1	0	0	0	0	0	0	0	0	0	0	0	1	1	0	1	0
**Lactobacillus**	2	119	0	0	0	0	0	0	0	0	0	0	0	1	0	1	0	1	1	0
**Lactococcus**	2	82	0	0	0	0	0	0	0	0	0	0	0	1	0	1	0	1	1	0
**Lamprocystis**	1	2	0	0	0	0	0	0	0	0	0	0	0	0	0	0	0	0	0	0
**Laribacter**	2	80	0	0	0	0	0	0	0	0	0	0	0	0	0	1	1	0	0	0
**Lawsonia**	2	33	0	0	0	0	0	0	0	0	0	0	0	1	0	0	0	0	0	0
**Leadbetterella**	2	79	0	1	0	0	0	0	0	0	0	0	0	0	0	1	1	0	1	0
**Leeuwenhoekiella**	1	2	0	0	0	0	0	0	0	0	0	0	0	0	0	0	0	0	0	0
**Legionella**	2	117	0	1	0	0	0	0	0	0	0	0	1	1	0	1	1	0	1	1
**Leifsonia**	2	48	0	0	0	0	0	0	0	0	0	0	0	1	0	0	1	0	0	1
**Leptolyngbya**	1	10	0	0	0	1	0	0	0	0	0	0	0	0	0	0	0	0	0	0
**Leptospira**	2	76	0	0	0	0	0	0	0	0	0	0	1	1	0	1	1	0	0	0
**Leptospirillum**	1	10	0	0	0	0	0	0	0	0	0	0	0	0	0	0	0	0	0	0
**Leptothrix**	3	186	0	1	0	1	1	1	0	0	0	0	0	0	0	1	1	0	0	1
**Leptotrichia**	2	43	0	0	0	0	0	0	0	0	0	0	0	1	0	0	0	0	0	0
**Leucobacter**	1	2	0	0	0	0	0	0	0	0	0	0	0	0	0	0	0	0	0	0
**Leuconostoc**	2	64	0	0	0	0	0	0	0	0	0	0	0	1	0	1	0	0	0	0

Genus	Cluster Number	Protein Counts	K00448	K00451	K00462	K00480	K00481	K00485	K00492	K00493	K00531	K00539	K00569	K00599	K00622	K00626	K00632	K00680	K00757	K00758
Liberibacter	1	4	0	0	0	1	0	0	0	0	0	0	0	0	0	0	0	0	0	0
Limnohabitans	1	11	0	0	0	0	0	0	0	0	0	0	0	0	0	0	0	0	0	0
Listeria	2	77	0	0	0	0	0	0	0	0	0	0	0	1	0	1	0	1	0	0
Listeriaceae	1	6	0	0	0	0	0	0	0	0	0	0	0	0	0	0	0	0	0	0
Listonella	1	38	0	1	0	0	0	0	0	0	0	0	1	0	0	0	1	0	1	1
Loktanella	1	3	0	0	0	0	0	0	0	0	0	0	0	0	0	1	0	0	0	0
Lysinibacillus	2	97	0	0	0	0	0	0	0	0	0	0	0	1	0	1	1	0	0	0
Macrococcus	2	60	0	0	0	0	0	0	0	0	0	0	0	1	0	0	0	0	0	0
Magnetococcus	2	64	0	0	0	0	0	0	0	0	0	0	1	0	0	1	0	0	0	0
Magnetospirillum	2	113	0	0	0	0	1	0	0	0	0	0	0	0	0	1	0	0	0	0
Mahella	2	58	0	0	0	0	0	0	0	0	0	0	0	0	0	0	0	0	0	0
Mannheimia	2	85	0	0	0	0	0	0	0	0	0	0	0	0	0	0	0	1	1	0
Maribacter	3	87	0	1	0	0	0	0	0	0	0	0	0	0	0	1	1	0	1	0
Maricaulis	3	87	0	0	0	0	0	0	0	0	0	0	0	0	0	1	0	0	0	1
Marichromatium	1	2	0	0	0	0	0	0	0	0	0	0	0	0	0	0	0	0	0	0
Marinithermus	2	77	0	0	0	0	0	0	0	0	0	0	0	0	0	1	1	0	0	0
Marinitoga	1	15	0	0	0	0	0	0	0	0	0	0	0	0	0	1	0	0	1	0
Marinobacter	2	163	1	0	0	0	0	0	0	0	0	0	1	0	0	1	1	0	0	0
Marinomonas	3	192	0	1	0	1	1	0	0	0	0	0	1	0	0	1	1	0	0	0
Maritimibacter	1	7	0	0	0	0	0	0	0	0	0	0	0	0	0	0	0	0	0	0
Marivirga	2	96	0	1	0	0	0	0	0	0	0	0	0	0	0	1	1	0	1	1
Martelella	1	3	0	0	0	0	0	0	0	0	0	0	0	0	0	0	0	0	0	0
Marvinbryantia	1	1	0	0	0	0	0	0	0	0	0	0	0	0	0	0	0	0	0	0
Massilia	1	4	0	0	0	0	0	0	0	0	0	0	0	0	0	0	0	0	0	0
Mastigocladopsis	1	16	0	0	0	0	0	0	0	0	0	0	0	0	0	0	0	0	0	0
Megamonas	1	47	0	0	0	0	0	0	0	0	0	0	0	0	0	0	0	0	0	0
Megasphaera	1	14	0	0	0	0	0	0	0	0	0	0	0	0	0	1	0	0	0	0
Meiothermus	2	110	1	0	0	0	1	0	0	0	0	0	0	0	0	1	1	0	1	0
Melissococcus	2	46	0	0	0	0	0	0	0	0	0	0	0	0	0	0	0	0	0	0
Mesoflavibacter	1	6	0	0	0	0	0	0	0	0	0	0	0	0	0	0	1	0	0	0
Mesoplasma	1	23	0	0	0	0	0	0	0	0	0	0	0	0	0	0	0	0	0	1
Mesorhizobium	2	201	1	1	0	1	1	1	1	1	0	0	0	1	0	1	1	1	0	1
Mesotoga	1	14	0	0	0	0	0	0	0	0	0	0	0	0	0	1	0	0	1	0
Metallosphaera	1	18	0	0	0	0	0	0	0	0	0	0	0	0	0	1	1	0	0	0
Methanobacterium	1	7	0	0	0	0	0	0	0	0	1	0	0	0	0	1	0	0	0	0
Methanobrevibacter	1	9	0	0	0	0	0	0	0	0	0	0	0	0	0	1	0	0	1	0
Methanocaldococcus	1	6	0	0	0	0	0	0	0	0	0	0	0	0	0	1	0	0	0	1
Methanocella	1	15	0	0	0	0	0	0	0	0	0	0	0	1	0	1	0	1	0	1
Methanococcaceae	1	1	0	0	0	0	0	0	0	0	0	0	0	0	0	0	0	0	0	0
Methanococcoides	1	5	0	0	0	0	0	0	0	0	0	0	0	0	0	1	0	0	0	1
Methanococcus	1	9	0	0	0	0	0	0	0	0	0	0	0	1	0	1	0	0	0	1
Methanocorpusculum	1	7	0	0	0	0	0	0	0	0	0	0	0	0	0	1	0	0	0	1
Methanoculleus	1	8	0	0	0	0	0	0	0	0	0	0	0	0	0	1	0	0	0	1
Methanohalobium	1	5	0	0	0	0	0	0	0	0	0	0	0	0	0	1	0	0	0	1
Methanohalophilus	1	5	0	0	0	0	0	0	0	0	0	0	0	0	0	1	0	0	0	1
Methanolobus	1	5	0	0	0	0	0	0	0	0	0	0	0	0	0	1	0	0	0	1
Methanomethylovorans	1	5	0	0	0	0	0	0	0	0	0	0	0	0	0	1	0	0	0	1
Methanoplanus	1	7	0	0	0	0	0	0	0	0	0	0	0	0	0	1	0	0	0	1
Methanopyrus	1	4	0	0	0	0	0	0	0	0	0	0	0	0	0	1	0	0	0	0
Methanoregula	1	8	0	0	0	0	0	0	0	0	0	0	0	0	0	1	0	0	0	1
Methanosaeta	1	9	0	0	0	0	0	0	0	0	0	0	0	0	0	1	0	0	0	1
Methanosalsum	1	7	0	0	0	0	0	0	0	0	0	0	0	0	0	1	0	0	0	1

Genus	Cluster Number	Protein Counts	K00448	K00451	K00462	K00480	K00481	K00485	K00492	K00493	K00531	K00539	K00569	K00599	K00622	K00626	K00632	K00680	K00757	K00758
Methanosarcina	1	18	0	0	0	0	0	0	0	0	1	0	0	1	0	1	0	1	0	1
Methanosphaera	1	7	0	0	0	0	0	0	0	0	0	0	0	0	0	1	0	0	0	0
Methanosphaerula	1	8	0	0	0	0	0	0	0	0	0	0	0	0	0	1	0	0	0	1
Methanospirillum	1	6	0	0	0	0	0	0	0	0	0	0	0	0	0	1	0	0	0	1
Methanothermobacter	1	6	0	0	0	0	0	0	0	0	0	0	0	0	0	1	0	0	0	0
Methanothermococcus	1	5	0	0	0	0	0	0	0	0	0	0	0	0	0	1	0	0	0	1
Methanothermus	1	5	0	0	0	0	0	0	0	0	0	0	0	0	0	1	0	0	0	0
Methanotorris	1	5	0	0	0	0	0	0	0	0	0	0	0	0	0	1	0	0	0	1
Methylacidiphilum	2	48	0	0	0	0	0	0	0	0	0	0	0	0	0	0	0	0	0	0
Methylarcula	1	2	0	0	0	0	0	0	0	0	0	0	0	0	0	0	0	0	0	0
Methylibium	1	38	0	1	0	0	0	0	0	0	0	1	0	1	0	1	1	0	0	0
Methylobacillus	2	61	0	0	0	0	0	0	1	0	0	0	0	1	0	0	0	0	0	0
Methylobacterium	3	236	1	1	0	1	1	0	1	0	0	0	0	1	0	1	0	1	0	1
Methylocella	3	117	0	0	0	1	0	1	0	0	0	0	0	0	0	1	1	0	0	0
Methylococcus	2	80	0	0	0	0	0	0	0	0	0	0	1	0	0	0	0	0	0	1
Methylocystis	1	21	0	1	0	1	0	0	0	0	0	0	0	0	0	1	0	0	0	0
Methyloferula	1	2	0	0	0	0	0	0	0	0	0	0	0	0	0	0	0	0	0	0
Methylomicrobium	1	12	0	0	0	0	0	0	0	0	0	0	1	0	0	0	1	0	0	1
Methylomonas	1	15	0	0	0	0	0	0	0	0	0	0	1	0	0	0	0	0	0	0
Methylophaga	1	7	0	0	0	0	0	0	0	0	0	0	0	0	0	0	0	0	0	0
Methylophilus	1	2	0	0	0	0	0	0	0	0	0	0	0	0	0	0	0	0	0	0
Methylosinus	1	2	0	0	0	0	0	0	0	0	0	0	0	0	0	0	0	0	0	0
Methylotenera	2	72	0	0	0	0	0	0	0	0	0	0	1	0	0	0	0	0	0	0
Methyloversatilis	1	5	0	0	0	0	0	0	0	0	0	0	0	0	0	0	0	0	0	0
Methylovorus	2	66	0	0	0	0	0	0	0	0	0	0	1	0	0	0	0	0	0	0
Micavibrio	1	9	0	1	0	0	0	0	0	0	0	0	0	0	0	1	0	0	0	1
Microbacterium	2	115	1	0	0	0	1	0	0	0	0	0	0	1	0	1	1	0	0	1
Microchaete	1	23	0	0	0	0	0	0	0	0	0	0	0	0	0	0	0	0	0	0
Micrococcus	2	72	0	0	0	0	0	0	0	0	0	0	0	1	0	1	0	0	0	1
Microcoleus	1	17	0	0	0	0	0	0	0	0	0	0	0	0	0	0	0	0	1	0
Microcystis	2	67	0	0	0	0	0	0	0	0	0	0	0	0	0	1	0	0	1	0
Microlunatus	1	25	0	1	0	0	1	0	0	0	0	0	0	1	0	1	1	0	0	0
Micromonospora	2	115	0	1	0	0	0	0	0	0	0	0	0	1	0	1	1	0	0	1
Microvirga	1	6	0	1	0	0	0	0	0	0	0	0	0	0	0	0	0	0	0	0
Mobiluncus	1	41	0	0	0	0	0	0	0	0	0	0	0	1	0	0	0	0	0	0
Modestobacter	1	43	1	1	0	1	1	0	0	0	0	0	0	0	0	1	0	0	0	1
Moorea	1	2	0	0	0	0	0	0	0	0	0	0	0	0	0	0	0	0	0	0
Moorella	2	69	0	0	0	0	0	0	0	0	0	0	0	0	0	1	0	0	0	1
Moraxella	2	66	0	0	0	0	0	0	0	0	0	0	0	0	0	1	1	0	0	0
Morganella	1	29	0	0	0	0	0	0	0	0	0	0	0	0	0	1	0	1	1	1
Moritella	1	2	0	0	0	0	0	0	0	0	0	0	0	0	0	0	0	0	0	0
Muricauda	1	23	0	1	0	0	0	0	0	0	0	0	0	0	0	1	1	0	1	0
Mycobacterium	3	306	1	1	1	1	0	1	1	1	0	0	0	1	1	1	1	1	0	1
Mycoplasma	1	53	0	0	0	0	0	0	0	0	0	0	0	1	0	0	0	1	0	1
Myxococcus	2	110	0	1	0	0	0	1	0	0	0	0	0	1	0	1	1	0	0	0
Nakamurella	2	115	0	1	0	0	1	0	0	0	0	0	0	1	0	1	1	0	0	1
napthalene	1	2	0	0	0	0	0	0	0	0	0	0	0	0	0	0	0	0	0	0
Natranaerobius	2	55	0	0	0	0	0	0	0	0	0	0	0	0	0	1	0	0	0	0
Natrialba	1	16	0	0	0	0	0	0	0	0	0	0	0	0	0	1	1	0	1	1
Natrinema	1	16	0	0	0	0	0	0	0	0	0	0	0	0	0	1	0	0	1	0
Natronobacterium	1	11	0	0	0	0	0	0	0	0	0	0	0	0	0	1	0	0	1	0
Natronococcus	1	21	0	0	0	0	0	0	0	0	0	0	0	0	0	1	0	0	1	0

Genus	Cluster Number	Protein Counts	K00448	K00451	K00462	K00480	K00481	K00485	K00492	K00493	K00531	K00539	K00569	K00599	K00622	K00626	K00632	K00680	K00757	K00758
**Natronomonas**	1	20	0	0	0	0	0	0	0	0	0	0	0	1	0	1	1	0	1	1
**Nautilia**	2	48	0	0	0	0	0	0	0	0	0	0	0	0	0	0	0	0	0	0
**Neisseria**	2	67	0	0	0	0	0	0	0	0	0	0	0	1	0	0	0	0	0	0
**Neisseriaceae**	1	1	0	0	0	0	0	0	0	0	0	0	0	0	0	0	0	0	0	0
**Neorickettsia**	1	31	0	0	0	0	0	0	0	0	0	0	0	1	0	0	0	0	0	0
**Neptuniibacter**	1	2	0	0	0	0	0	0	0	0	0	0	0	0	0	0	0	0	0	0
**Niastella**	1	21	0	1	0	0	0	0	0	0	0	0	0	0	0	1	0	0	1	0
**Nitratifractor**	2	54	0	0	0	0	0	0	0	0	0	0	0	1	0	0	0	0	0	0
**Nitratireductor**	1	19	0	0	0	0	0	0	0	0	0	0	0	0	0	1	0	0	0	0
**Nitratiruptor**	2	40	0	0	0	0	0	0	0	0	0	0	0	0	0	0	0	0	0	0
**Nitrobacter**	2	99	0	1	0	1	0	0	0	0	0	0	0	0	0	1	1	0	0	1
**Nitrococcus**	1	2	0	0	0	0	0	0	0	0	0	0	0	0	0	0	0	0	0	0
**Nitrosococcus**	2	94	0	1	0	0	0	0	0	0	0	0	0	0	0	1	1	0	0	0
**Nitrosomonas**	2	72	0	0	0	0	0	0	0	0	0	0	1	1	0	1	1	0	0	0
**Nitrosopumilaceae**	1	7	0	0	0	0	0	0	0	0	0	0	0	0	0	1	0	0	0	0
**Nitrosopumilus**	1	11	0	0	0	0	0	0	0	0	0	0	0	0	0	1	0	0	0	0
**Nitrosospira**	2	74	0	0	0	0	0	0	0	0	0	0	1	0	0	0	1	0	0	0
**Nocardia**	2	185	1	1	0	0	1	0	1	1	0	0	0	1	0	1	1	1	0	1
**Nocardioidaceae**	1	2	0	0	0	0	0	0	0	0	0	0	0	0	0	0	0	0	0	0
**Nocardioides**	2	157	0	1	0	0	0	0	0	1	0	0	0	1	0	1	1	0	0	1
**Nocardiopsis**	2	102	0	1	0	0	0	0	0	0	0	0	0	1	0	1	1	0	0	1
**Nodularia**	1	22	0	0	0	0	0	0	0	0	0	0	0	0	0	0	0	0	0	0
**Nonlabens**	1	19	0	1	0	0	0	0	0	0	0	0	0	0	0	1	0	0	1	0
**Nostoc**	2	111	0	0	0	1	0	0	0	0	0	0	0	1	0	0	0	0	1	0
**Nostocaceae**	1	2	0	0	0	0	0	0	0	0	0	0	0	0	0	0	0	0	0	0
**Novosphingobium**	2	169	0	0	0	0	1	1	0	0	0	0	0	0	0	1	1	0	0	1
**Oceanibaculum**	1	3	0	0	0	0	0	0	0	0	0	0	0	0	0	0	0	0	0	0
**Oceanibulbus**	1	12	0	0	0	0	0	0	0	0	0	0	0	0	0	1	0	0	0	0
**Oceanicola**	1	9	0	0	0	0	0	0	0	0	0	0	0	0	0	0	0	0	0	0
**Oceanimonas**	1	33	1	1	0	1	1	1	1	0	0	0	1	0	0	1	1	0	1	1
**Oceaniovalibus**	1	2	0	0	0	0	0	0	0	0	0	0	0	0	0	0	0	0	0	0
**Oceanithermus**	2	72	0	0	0	0	0	0	0	0	0	0	0	0	0	1	1	0	0	0
**Oceanobacillus**	2	91	0	0	0	0	0	0	0	0	0	0	0	1	0	1	1	0	0	0
**Oceanospirillum**	1	2	0	0	0	0	0	0	0	0	0	0	0	0	0	0	0	0	0	0
**Ochrobactrum**	2	133	1	0	0	1	1	0	0	0	0	0	0	0	0	1	0	0	0	0
**Octadecabacter**	1	29	0	0	0	1	0	0	0	0	0	0	0	0	0	1	0	0	0	1
**Oenococcus**	2	41	0	0	0	0	0	0	0	0	0	0	0	0	0	1	0	0	0	0
**Oleomonas**	1	2	0	0	0	0	0	0	0	0	0	0	0	0	0	0	0	0	0	0
**Oligotropha**	3	87	0	0	0	1	0	0	0	0	0	0	0	0	0	1	0	0	0	0
**Olsenella**	2	40	0	0	0	0	0	0	0	0	0	0	0	1	0	1	0	0	1	0
**Opitutus**	2	67	0	0	0	0	0	0	0	0	0	0	0	0	0	0	0	0	0	0
**Orientia**	1	21	0	0	0	0	0	0	0	0	0	0	0	0	0	0	0	0	0	0
**Ornithobacterium**	1	11	0	0	0	0	0	0	0	0	0	0	0	0	0	0	0	0	1	0
**Oscillatoria**	1	16	0	0	0	0	0	0	0	0	0	0	0	0	0	0	0	0	0	0
**Oscillatoriales**	1	2	0	0	0	0	0	0	0	0	0	0	0	0	0	0	0	0	0	0
**Oscillibacter**	1	20	0	0	0	0	0	0	0	0	0	0	0	0	0	1	0	0	1	0
**Owenweeksia**	1	15	0	1	0	0	0	0	0	0	0	0	0	0	0	1	0	0	1	0
**Oxalobacteraceae**	1	4	0	0	0	0	0	0	0	0	0	0	0	0	0	0	0	0	0	0
**Paenibacillus**	2	152	0	0	0	0	0	0	0	0	0	0	0	1	0	1	1	1	1	0
**Paludibacter**	2	67	0	0	0	0	0	0	0	0	1	0	0	0	0	0	0	0	1	0
**Pandoraea**	1	18	0	0	0	0	0	0	0	0	0	0	0	0	0	0	0	0	0	0
**Pannonibacter**	1	3	0	0	0	0	0	0	0	0	0	0	0	0	0	0	0	0	0	0

Genus	Cluster Number	Protein Counts	K00448	K00451	K00462	K00480	K00481	K00485	K00492	K00493	K00531	K00539	K00569	K00599	K00622	K00626	K00632	K00680	K00757	K00758
Pantholops	1	23	0	0	0	0	0	0	0	0	0	0	0	0	0	1	1	0	0	0
Pantoea	2	181	1	0	0	0	0	0	0	0	0	0	0	0	0	0	1	0	1	1
Parabacteroides	2	64	0	0	0	0	0	0	0	0	0	0	0	0	0	0	0	0	1	0
Parachlamydia	1	11	0	0	0	0	0	0	0	0	0	0	0	1	0	1	1	0	0	0
Paracoccus	2	169	1	1	0	1	1	0	0	0	0	0	0	0	0	1	1	0	0	1
Parvibaculum	3	117	0	0	0	0	0	0	0	0	0	0	0	0	0	1	1	0	0	1
Parvularcula	2	72	0	0	0	0	0	0	0	0	0	0	0	0	0	1	0	0	0	0
Pasteurella	2	85	0	0	0	0	0	0	0	0	0	0	0	0	0	0	0	1	1	0
Pasteurellaceae	1	2	0	0	0	0	0	0	0	0	0	0	0	0	0	0	0	0	0	0
Pectobacterium	1	44	0	0	0	0	0	0	0	0	0	0	0	0	0	1	1	1	1	1
Pediococcus	1	43	0	0	0	0	0	0	0	0	0	0	0	1	0	1	0	1	0	0
Pedobacter	2	88	0	1	0	0	0	1	0	0	0	0	0	0	0	1	1	1	1	0
Pelagibaca	1	10	0	0	0	0	0	0	0	0	0	0	0	0	0	1	0	0	0	0
Pelagibacterium	1	39	1	0	0	1	1	0	0	0	0	0	0	0	0	1	0	0	1	1
Pelobacter	2	85	0	0	0	0	0	0	0	0	0	0	0	0	0	0	0	1	1	0
Pelodictyon	2	62	0	0	0	0	0	0	0	0	0	0	0	0	0	0	0	0	0	0
Pelosinus	1	1	0	0	0	0	0	0	0	0	0	0	0	0	0	0	0	0	0	0
Pelotomaculum	2	60	0	0	0	0	0	0	0	0	0	0	0	0	0	1	0	0	0	1
Peptostreptococcus	1	1	0	0	0	0	0	0	0	0	0	0	0	0	0	0	0	0	0	0
Persephonella	2	55	0	0	0	0	0	0	0	0	0	0	0	0	0	0	0	0	0	0
Petrotoga	2	72	0	0	0	0	0	0	0	0	0	0	0	0	0	1	0	0	1	0
Phaeobacter	2	55	1	1	0	1	1	0	0	0	0	0	0	0	0	1	0	0	0	1
Phenylobacterium	3	103	0	1	0	0	0	0	0	0	0	0	0	0	0	1	0	0	0	1
Photobacterium	2	119	0	1	0	0	0	0	0	0	0	0	1	0	0	1	1	0	1	0
Photorhabdus	2	149	0	0	0	0	0	0	0	0	0	0	0	0	0	1	1	1	1	1
Phycisphaera	1	9	0	0	0	0	0	0	0	0	0	0	0	0	0	0	0	0	0	0
Phyllobacterium	1	6	0	0	0	0	0	0	0	0	0	0	0	0	0	1	0	0	0	0
Picrophilus	1	15	0	1	0	0	0	0	0	1	0	0	0	1	0	1	0	1	0	0
Pirellula	2	85	0	0	0	0	0	0	0	0	0	0	0	1	0	1	1	0	0	0
Planctomyces	2	93	0	0	0	0	0	0	0	0	0	0	0	1	0	1	0	0	0	0
Plautia	1	17	0	0	0	0	0	0	0	0	0	0	0	0	0	0	0	0	0	0
Plesiomonas	1	4	0	0	0	0	0	0	0	0	0	0	0	0	0	0	0	0	0	0
Pleurocapsa	1	23	0	1	0	0	0	0	0	0	0	0	0	0	0	1	0	0	1	0
Polaribacter	2	74	0	1	0	0	0	0	0	0	0	0	0	0	0	1	0	0	1	0
Polaromonas	3	238	1	1	1	1	1	0	0	0	0	0	0	0	0	1	1	0	0	1
Polymorphum	3	173	1	1	0	1	1	0	0	0	0	0	0	0	0	1	1	1	0	1
Polynucleobacter	1	19	0	0	0	0	0	0	0	0	0	0	0	0	0	1	1	0	0	0
Ponticaulis	1	1	0	0	0	0	0	0	0	0	0	0	0	0	0	0	0	0	0	0
Porphyromonas	2	63	0	0	0	0	0	0	0	0	0	0	0	1	0	0	0	0	1	0
Prevotella	1	65	0	0	0	0	0	0	0	0	0	0	0	1	0	0	0	0	1	0
Prochlorococcus	2	68	0	0	0	0	0	0	0	0	0	0	0	1	0	0	0	0	0	0
Propionibacterium	1	62	0	0	0	0	0	0	0	0	0	0	0	1	0	1	0	1	0	1
Prosthecochloris	1	16	0	0	0	0	0	0	0	0	0	0	0	0	0	0	0	0	0	0
Proteobacteria	1	19	0	0	0	0	0	0	0	0	0	0	0	0	0	0	0	0	0	1
Proteus	2	113	0	0	0	0	0	0	0	0	0	0	0	0	0	0	1	0	1	1
Providencia	1	38	0	0	0	0	0	0	0	0	0	0	0	0	0	0	1	0	1	1
Pseudaminobacter	1	10	0	0	0	0	0	0	0	0	0	0	0	0	0	1	0	0	0	0
Pseudanabaena	1	11	0	0	0	0	0	0	0	0	0	0	0	0	0	0	0	0	0	0
Pseudoalteromonas	2	188	0	1	0	1	1	1	0	0	0	0	1	0	0	1	1	0	1	0
Pseudochrobactrum	1	7	0	0	0	0	0	0	0	0	0	0	0	0	0	1	0	0	0	0
Pseudogulbenkiania	1	40	1	1	0	1	0	0	0	0	0	0	0	0	0	1	1	0	0	1
pseudomallei	3	131	1	1	0	0	0	0	1	0	0	0	0	1	0	1	1	1	0	1

Genus	Cluster Number	Protein Counts	K00448	K00451	K00462	K00480	K00481	K00485	K00492	K00493	K00531	K00539	K00569	K00599	K00622	K00626	K00632	K00680	K00757	K00758
Pseudomonas	3	381	1	1	1	1	1	1	1	1	0	0	1	1	0	1	1	1	0	1
Pseudonocardia	3	180	1	1	0	1	1	0	0	0	0	0	0	1	0	1	1	0	0	0
Pseudorhodobacter	1	4	0	0	0	0	0	0	0	0	0	0	0	0	0	0	0	0	0	0
Pseudovibrio	1	33	1	1	0	1	1	0	0	0	0	0	0	0	0	1	0	0	0	1
Pseudoxanthomonas	2	124	0	1	1	1	0	0	0	0	0	0	1	0	0	1	1	0	0	1
Psychrobacter	2	124	0	0	0	0	0	0	1	0	0	0	1	0	0	1	1	0	0	0
Psychroflexus	1	22	0	1	0	0	0	0	0	0	0	0	0	0	0	1	0	0	1	0
Psychromonas	2	110	0	0	0	0	0	0	0	0	0	0	1	0	0	1	1	0	1	1
Pusillimonas	3	111	0	1	0	0	0	0	1	0	0	0	1	0	0	1	1	0	0	0
Pyrobaculum	1	15	0	0	0	0	0	0	0	0	0	0	0	0	0	1	1	0	1	0
Pyrococcus	1	10	0	0	0	0	0	0	0	0	0	0	0	0	0	1	0	0	0	1
Pyrolobus	1	4	0	0	0	0	0	0	0	0	0	0	0	0	0	1	0	0	0	0
Rahnella	2	143	0	0	0	0	0	0	0	0	0	0	0	1	0	0	1	1	1	1
Ralstonia	3	332	1	1	0	1	1	0	1	1	0	1	1	1	0	1	1	1	0	1
Ramlibacter	1	35	0	0	0	1	1	0	0	0	0	0	0	0	0	1	1	0	0	0
Raoultella	2	139	1	0	0	0	1	0	0	0	0	0	0	0	0	1	1	0	1	1
Raphidiopsis	1	5	0	0	0	0	0	0	0	0	0	0	0	0	0	0	0	0	0	0
Reinekea	1	2	0	0	0	0	0	0	0	0	0	0	0	0	0	0	0	0	0	0
Renibacterium	2	67	0	0	0	0	0	0	0	0	0	0	0	1	0	1	1	0	0	1
Rhizobiaceae	1	3	0	0	0	0	0	0	0	0	0	0	0	0	0	0	0	0	0	0
Rhizobiales	1	6	0	0	0	0	0	0	0	0	0	0	0	0	0	1	0	0	0	0
Rhizobium	3	257	1	1	1	1	1	1	1	1	0	0	0	1	0	1	1	1	0	1
Rhizobium/Agrobacterium	2	87	0	0	0	0	0	0	0	0	0	0	0	0	0	1	0	1	0	0
Rhodanobacter	1	21	0	1	0	0	0	0	0	0	0	0	1	0	0	1	0	0	0	1
Rhodobacter	3	175	0	0	0	1	0	0	1	1	1	0	0	1	0	1	1	1	0	1
Rhodobacteraceae	1	21	0	0	0	0	0	0	0	0	0	0	0	0	0	1	0	0	0	0
Rhodobacterales	1	34	0	1	0	0	0	0	0	0	0	0	0	0	0	1	0	0	0	0
Rhodococcus	2	292	1	1	1	1	1	1	1	1	0	0	0	1	0	1	1	1	0	1
Rhodocyclaceae	1	7	0	0	0	0	0	0	0	0	0	0	0	0	0	0	0	0	0	0
Rhodoferax	3	144	0	0	0	1	1	0	0	0	0	0	0	0	0	1	1	1	0	1
Rhodomicrobium	2	103	0	0	0	1	0	0	0	0	1	0	0	0	0	1	0	1	0	0
Rhodopirellula	2	87	0	0	0	0	0	0	1	0	0	0	0	1	0	0	0	1	0	0
Rhodopseudomonas	3	246	0	1	0	1	1	0	0	1	1	0	0	1	0	1	1	1	0	1
Rhodospirillum	2	121	0	1	0	0	0	0	0	0	1	0	0	0	0	1	1	0	0	1
Rhodothermus	2	76	0	0	0	0	0	0	0	0	0	0	0	0	0	1	1	0	0	0
Rhodovulum	1	4	0	0	0	0	0	0	0	0	0	0	0	0	0	0	0	0	0	0
Richelia	1	2	0	0	0	0	0	0	0	0	0	0	0	0	0	0	0	0	0	0
Rickettsia	2	43	0	0	0	0	0	0	0	0	0	0	0	1	0	1	0	0	0	0
Rickettsiaceae	1	5	0	0	0	0	0	0	0	0	0	0	0	0	0	0	0	0	0	0
Riemerella	2	61	0	1	0	0	0	0	0	0	0	0	0	0	0	1	1	0	1	0
Rivularia	1	18	0	0	0	1	0	0	0	0	0	0	0	0	0	0	0	0	0	0
Robiginitalea	2	83	0	1	0	0	0	0	0	0	0	0	0	0	0	1	1	0	1	1
Roseburia	2	58	0	0	0	0	0	0	0	0	0	0	0	1	0	1	0	0	0	0
Roseibium	1	3	0	0	0	0	0	0	0	0	0	0	0	0	0	0	0	0	0	0
Roseiflexus	3	100	0	0	0	0	0	0	0	0	0	0	0	1	0	1	1	0	1	0
Roseobacter	2	149	1	1	0	1	1	1	1	1	0	0	0	1	0	1	0	1	0	1
Roseomonas	1	2	0	0	0	0	0	0	0	0	0	0	0	0	0	0	0	0	0	0
Roseovarius	1	15	0	0	0	0	0	0	0	0	0	0	0	0	0	1	0	0	0	0
Rothia	1	44	0	0	0	0	0	0	0	0	0	0	0	1	0	0	0	0	0	0
Rubrivivax	1	24	0	0	0	0	0	0	0	0	0	0	0	0	0	1	0	0	0	1
Rubrobacter	3	117	1	0	0	0	1	0	0	0	0	0	0	0	0	1	1	0	0	0
Ruegeria	2	189	1	1	0	1	1	0	0	1	0	0	0	0	0	1	1	1	0	1

Genus	Cluster Number	Protein Counts	K00448	K00451	K00462	K00480	K00481	K00485	K00492	K00493	K00531	K00539	K00569	K00599	K00622	K00626	K00632	K00680	K00757	K00758
Ruminococcaceae	1	2	0	0	0	0	0	0	0	0	0	0	0	0	0	0	0	0	0	0
Ruminococcus	2	101	0	0	0	0	0	0	0	0	0	0	0	1	0	1	0	0	1	0
Runella	1	26	1	1	0	0	1	0	0	0	0	0	0	0	0	1	1	0	0	0
Saccharomonospora	2	132	1	1	0	1	1	0	0	0	0	0	0	1	0	1	0	0	0	1
Saccharophagus	2	81	0	0	0	0	0	0	0	0	0	0	0	0	0	1	1	0	0	0
Saccharopolyspora	3	194	1	1	0	0	1	0	1	1	0	0	0	1	0	1	1	1	0	1
Saccharothrix	1	32	1	1	0	1	1	0	0	0	0	0	0	0	0	1	0	0	1	1
Sagittula	1	10	0	0	0	0	0	0	0	0	0	0	0	0	0	1	0	0	0	0
Salinibacter	2	77	0	0	0	0	0	0	0	0	0	0	0	1	0	1	1	0	0	0
Salinimonas	1	1	0	0	0	0	0	0	0	0	0	0	0	0	0	0	1	0	0	0
Salinispora	3	127	0	1	0	0	0	0	0	0	0	0	0	1	0	1	1	0	0	1
Salinivibrio	1	2	0	0	0	0	0	0	0	0	0	0	0	0	0	0	0	0	0	0
Salmonella	2	151	0	0	0	1	0	0	0	0	0	0	0	1	0	1	1	1	1	1
Sanguibacter	2	73	0	0	0	0	0	0	0	0	0	0	0	1	0	1	1	0	0	0
Saprospira	1	13	0	1	0	0	0	0	0	0	0	0	0	0	0	1	0	0	1	0
Scardovia	1	1	0	0	0	0	0	0	0	0	0	0	0	0	0	0	0	0	0	0
Scytonema	1	10	0	0	0	0	0	0	0	0	0	0	0	0	0	0	0	0	0	0
Sebaldella	2	69	0	0	0	0	0	0	0	0	0	0	0	1	0	0	0	1	1	0
Segniliparus	2	85	0	0	0	1	0	0	0	1	0	0	0	1	0	1	0	0	0	1
Selenomonas	2	57	0	0	0	0	0	0	0	0	0	0	0	0	0	0	0	0	0	0
Serinicoccus	1	1	0	0	0	0	0	0	0	0	0	0	0	0	0	0	0	0	0	0
Serratia	2	186	1	0	0	0	1	0	0	0	0	0	0	0	0	1	1	1	1	1
Shewanella	3	214	0	1	0	0	0	0	1	0	0	0	1	0	0	1	1	0	1	1
Shigella	2	172	0	0	0	1	0	0	0	0	0	0	0	0	0	1	1	1	1	1
Shinella	1	2	0	0	0	0	0	0	0	0	0	0	0	0	0	0	0	0	0	0
Sideroxydans	2	81	0	0	0	0	0	0	0	0	0	0	1	0	0	0	1	0	0	0
Silicibacter	2	105	0	1	0	0	1	0	0	1	0	0	0	0	0	1	1	0	0	0
Simiduia	1	18	0	1	0	0	0	0	0	0	0	0	1	0	0	1	0	0	0	0
Simkania	1	6	0	0	0	0	0	0	0	0	0	0	0	1	0	0	0	0	0	0
Simonsiella	1	2	0	0	0	0	0	0	0	0	0	0	0	0	0	0	0	0	0	0
Singulisphaera	1	18	0	1	0	0	0	0	0	0	0	0	0	0	0	1	0	0	0	0
Sinorhizobium	3	207	1	1	0	1	1	1	1	1	0	0	0	1	0	1	1	1	0	1
Slackia	2	46	0	0	0	0	0	0	0	0	0	0	0	1	0	0	0	0	0	0
Smaragdicoccus	1	3	0	0	0	0	0	0	0	0	0	0	0	0	0	0	0	0	0	0
Sodalis	2	71	0	0	0	1	0	0	0	0	0	0	0	0	0	0	0	0	1	1
Solibacillus	1	23	0	0	0	1	0	0	0	0	0	0	0	0	0	1	0	0	0	0
Solitalea	1	13	0	1	0	0	0	0	0	0	0	0	0	0	0	1	0	0	1	0
Sorangium	3	115	0	1	0	0	0	0	0	0	0	0	0	1	0	1	1	0	0	0
Sphaerobacter	2	100	1	0	0	0	1	0	1	0	0	0	0	0	0	1	1	0	0	0
Sphaerochaeta	1	42	0	0	0	0	0	0	0	0	0	0	0	0	0	1	0	0	1	0
Sphingobacterium	3	85	0	0	0	0	0	0	0	0	0	0	0	0	0	1	1	0	1	0
Sphingobium	1	179	0	1	1	1	1	0	0	0	0	0	0	0	0	1	0	0	0	1
Sphingomonadaceae	1	15	0	0	0	0	0	0	0	0	0	0	0	0	0	1	0	0	0	1
Sphingomonas	2	220	1	1	1	0	0	0	0	0	0	0	0	0	0	1	1	0	0	1
Sphingopyxis	3	99	0	0	0	0	0	0	0	0	0	0	0	0	0	1	1	0	0	1
Spirochaeta	2	103	0	0	0	0	0	0	0	0	0	0	0	0	0	1	0	0	1	0
Spiroplasma	1	6	0	0	0	0	0	0	0	0	0	0	0	0	0	0	0	0	0	0
Spirosoma	2	107	1	1	0	0	1	0	0	0	0	0	1	0	0	1	1	0	0	0
Stackebrandtia	2	110	1	1	0	0	0	0	1	0	0	0	0	1	0	1	1	0	0	1
Stanieria	1	17	0	1	0	0	0	0	0	0	0	0	0	0	0	1	0	0	1	0
Staphylococcus	2	108	0	0	0	0	0	0	1	0	0	0	0	0	0	1	1	1	0	0
Staphylothermus	1	12	0	0	0	0	0	0	0	0	0	0	0	0	0	1	1	0	1	1

Genus	Cluster Number	Protein Counts	K00448	K00451	K00462	K00480	K00481	K00485	K00492	K00493	K00531	K00539	K00569	K00599	K00622	K00626	K00632	K00680	K00757	K00758
Stappia	1	2	0	0	0	0	0	0	0	0	0	0	0	0	0	0	0	0	0	0
Starkeya	3	134	0	0	0	1	0	0	0	0	0	0	1	1	0	1	0	0	0	1
Stenotrophomonas	3	104	1	1	0	0	0	0	0	0	0	0	0	0	0	1	1	0	0	0
Stigmatella	3	129	1	1	0	0	1	0	0	0	0	0	0	0	0	1	1	0	0	0
Streptobacillus	1	38	0	0	0	0	0	0	0	0	0	0	0	1	0	1	0	0	0	0
Streptococcus	2	121	0	0	0	0	0	0	0	0	0	0	0	1	0	1	1	1	1	0
Streptomyces	2	253	1	1	0	1	1	0	1	1	0	0	0	1	0	1	1	1	1	1
Streptosporangium	3	148	1	1	0	1	1	0	0	0	0	0	0	1	0	1	1	0	0	1
Strigomonas	1	2	0	0	0	0	0	0	0	0	0	0	0	0	0	0	0	0	0	0
Sulfitobacter	1	10	0	1	0	0	0	0	0	0	0	0	0	0	0	1	0	0	0	0
Sulfobacillus	1	30	0	0	0	0	0	0	0	0	0	0	0	0	0	1	1	0	0	0
Sulfolobus	1	23	0	1	0	0	0	0	0	0	0	0	0	0	0	1	1	0	0	0
Sulfuricurvum	2	67	0	0	0	0	0	0	0	0	0	0	0	0	0	0	0	0	0	0
Sulfurihydrogenibium	2	55	0	0	0	0	0	0	0	0	0	0	0	0	0	0	0	0	0	0
Sulfurimonas	2	60	0	0	0	0	0	0	0	0	0	0	0	1	0	0	0	0	0	0
Sulfurospirillum	1	48	0	0	0	0	0	0	0	0	0	0	0	0	0	0	0	0	0	0
Sulfurovum	2	48	0	0	0	0	0	0	0	0	0	0	0	0	0	0	0	0	0	0
Symbiobacterium	2	68	0	0	0	0	0	0	0	0	0	0	0	0	0	1	1	0	1	0
Synechococcus	1	34	0	0	0	0	0	0	0	0	0	0	1	1	0	0	0	1	0	0
Synechocystis	1	40	0	0	0	0	0	0	0	0	0	0	0	1	0	1	0	0	0	0
Synergistetes	1	27	0	0	0	0	0	0	0	0	0	0	0	0	0	0	0	0	0	0
Syntrophobacter	2	101	0	0	0	0	0	0	0	0	0	0	0	0	0	1	1	0	0	0
Syntrophobotulus	2	71	0	0	0	0	0	0	0	0	1	0	0	1	0	0	0	0	0	0
Syntrophomonas	2	58	0	0	0	0	0	0	0	0	0	0	0	0	0	1	0	0	0	0
Syntrophothermus	2	60	0	0	0	0	0	0	0	0	0	0	0	0	0	1	0	0	0	0
Syntrophus	2	66	0	0	0	0	0	0	0	0	0	0	0	0	0	1	0	0	0	0
Tannerella	1	8	0	0	0	0	0	0	0	0	0	0	0	0	0	0	0	0	1	0
Taylorella	1	43	0	0	0	0	0	0	0	0	0	0	0	1	0	0	0	0	0	0
Tepidanaerobacter	2	55	0	0	0	0	0	0	0	0	0	0	0	0	0	0	0	0	0	0
Teredinibacter	2	109	0	0	0	0	0	0	0	0	0	0	1	0	0	1	1	0	0	0
Terrabacter	1	1	0	0	0	0	0	0	0	0	0	0	0	0	0	0	0	0	0	0
Terriglobus	2	83	0	0	0	0	1	0	0	0	0	0	0	0	0	0	0	0	0	0
Tetragenococcus	1	12	0	0	0	0	0	0	0	0	0	0	0	0	0	0	0	0	0	0
Thalassiobium	1	11	0	0	0	0	0	0	0	0	0	0	0	0	0	1	0	0	0	0
Thalassobacter	1	9	0	1	0	0	0	0	0	0	0	0	0	0	0	1	0	0	0	0
Thalassolituus	1	12	0	0	0	0	0	0	0	0	0	0	1	0	0	1	0	0	0	1
Thalassospira	1	2	0	0	0	0	0	0	0	0	0	0	0	0	0	0	0	0	0	0
Thauera	1	111	0	0	0	1	0	0	0	0	0	0	0	0	0	1	1	0	0	1
Thermacetogenium	1	7	0	0	0	0	0	0	0	0	0	0	0	0	0	0	0	0	0	0
Thermaerobacter	2	79	0	0	0	0	0	0	0	0	0	0	0	1	0	1	1	0	0	0
Thermanaerovibrio	2	58	0	0	0	0	0	0	0	0	0	0	0	0	0	0	0	0	0	0
Thermincola	2	62	0	0	0	0	0	0	0	0	0	0	0	0	0	0	0	0	0	0
Thermoanaerobacter	2	76	0	0	0	0	0	0	0	0	0	0	0	1	0	1	0	1	0	0
Thermoanaerobacterium	2	64	0	0	0	0	0	0	0	0	0	0	0	0	0	1	0	0	1	0
Thermobacillus	1	11	0	0	0	0	0	0	0	0	0	0	0	0	0	1	0	0	1	0
Thermobaculum	2	62	0	0	0	0	0	0	0	0	0	0	0	0	0	1	0	0	0	0
Thermobifida	2	72	0	0	0	0	0	0	1	0	0	0	0	1	0	1	1	0	0	0
Thermobispora	2	109	1	1	0	0	1	0	0	0	0	0	0	1	0	1	1	0	0	1
Thermococcus	1	13	0	0	0	0	0	0	0	0	0	0	0	0	0	1	0	0	1	1
Thermocrinis	2	60	0	0	0	0	0	0	0	0	0	0	0	0	0	0	0	0	0	0
Thermodesulfatator	1	10	0	0	0	0	0	0	0	0	0	0	0	0	0	0	0	0	0	0
Thermodesulfobacterium	1	9	0	0	0	0	0	0	0	0	0	0	0	0	0	0	0	0	0	0

Genus	Cluster Number	Protein Counts	K00448	K00451	K00462	K00480	K00481	K00485	K00492	K00493	K00531	K00539	K00569	K00599	K00622	K00626	K00632	K00680	K00757	K00758
Thermodesulfobium	1	13	0	0	0	0	0	0	0	0	0	0	0	1	0	0	0	0	0	0
Thermodesulfovibrio	2	60	0	0	0	0	0	0	0	0	0	0	0	0	0	0	0	0	0	0
Thermofilum	1	9	0	0	0	0	0	0	0	0	0	0	0	0	0	1	0	0	1	1
Thermogladius	1	7	0	0	0	0	0	0	0	0	0	0	0	0	0	1	0	0	1	1
Thermomicrobium	2	92	0	0	0	0	0	0	0	0	0	0	0	0	0	1	0	0	0	0
Thermomonospora	2	125	1	1	1	0	1	0	0	0	0	0	0	1	0	1	1	0	0	1
Thermoplasma	1	11	0	0	0	0	0	0	0	0	0	0	0	0	0	1	0	0	0	0
Thermoplasmatales	1	6	0	0	0	0	0	0	0	0	0	0	0	0	0	1	0	0	0	1
Thermoproteus	1	12	0	0	0	0	0	0	0	0	0	0	0	0	0	1	0	0	1	0
Thermosediminibacter	2	64	0	0	0	0	0	0	0	0	0	0	0	0	0	0	0	0	0	0
Thermosipho	2	70	0	0	0	0	0	0	0	0	0	0	0	0	0	1	0	0	0	0
Thermosphaera	1	10	0	0	0	0	0	0	0	0	0	0	0	0	0	1	0	0	1	1
Thermosynechococcus	1	6	0	0	0	0	0	0	0	0	0	0	0	1	0	0	0	0	0	0
Thermotoga	2	80	0	0	0	0	0	0	0	0	0	0	0	1	0	1	0	0	1	0
Thermovibrio	2	58	0	0	0	0	0	0	0	0	0	0	0	0	0	0	0	0	0	0
Thermovirga	1	9	0	0	0	0	0	0	0	0	0	0	0	0	0	0	0	0	0	0
Thermus	2	107	0	0	0	0	1	0	0	0	0	0	0	1	0	1	1	1	0	1
Thioalkalimicrobium	1	4	0	0	0	0	0	0	0	0	0	0	0	0	0	0	0	0	0	0
Thioalkalivibrio	2	91	0	0	0	0	0	0	0	0	0	0	0	0	0	1	0	0	0	0
Thiobaca	1	2	0	0	0	0	0	0	0	0	0	0	0	0	0	0	0	0	0	0
Thiobacillus	2	67	0	0	0	0	0	0	0	0	0	0	0	1	0	0	0	0	0	0
Thiocapsa	1	2	0	0	0	0	0	0	0	0	0	0	0	0	0	0	0	0	0	0
Thiocystis	1	11	0	0	0	0	0	0	0	0	0	0	1	0	0	1	0	0	0	0
Thioflavicoccus	1	13	0	0	0	0	0	0	0	0	0	0	1	0	0	1	0	0	0	1
Thiomicrospira	2	50	0	0	0	0	0	0	0	0	0	0	1	0	0	0	0	0	0	0
Thiomonas	2	85	0	0	0	0	0	0	0	0	0	0	0	0	0	1	1	0	0	0
Thiorhodococcus	1	2	0	0	0	0	0	0	0	0	0	0	0	0	0	0	0	0	0	0
Thiothrix	1	2	0	0	0	0	0	0	0	0	0	0	0	0	0	0	0	0	0	0
Tistrella	1	42	1	1	0	0	1	0	0	0	0	0	1	0	0	1	0	0	0	0
Tolumonas	2	100	0	0	0	0	0	0	0	0	0	0	1	0	0	0	0	0	0	1
Transposon	1	2	0	0	0	0	0	0	0	0	0	0	0	0	0	0	0	0	0	0
Treponema	1	63	0	0	0	0	0	0	0	0	0	0	0	0	0	0	0	0	1	0
Trichodesmium	2	71	0	0	0	1	0	1	0	0	0	0	0	1	0	0	0	0	0	0
Trichormus	1	12	0	0	0	0	0	0	0	0	0	0	0	0	0	0	0	0	0	0
Tropheryma	2	22	0	0	0	0	0	0	0	0	0	0	0	1	0	0	0	0	0	0
Truepera	2	104	0	0	0	0	0	0	0	0	0	0	0	0	0	1	1	0	0	0
Tsukamurella	2	123	1	0	0	1	0	0	0	0	0	0	0	1	0	1	1	0	0	1
Turneriella	1	16	0	0	0	0	0	0	0	0	0	0	0	0	0	1	0	0	0	0
Uliginosibacterium	1	2	0	0	0	0	0	0	0	0	0	0	0	0	0	0	0	0	0	0
Ureaplasma	1	24	0	0	0	0	0	0	0	0	0	0	0	0	0	0	0	0	0	1
Ureibacillus	1	2	0	0	0	0	0	0	0	0	0	0	0	0	0	0	0	0	0	0
Variovorax	3	187	1	1	0	1	1	0	1	0	0	0	0	0	0	1	1	0	0	0
Veillonella	1	54	0	0	0	0	0	0	0	0	0	0	0	0	0	1	0	0	0	0
Verminephrobacter	3	188	0	0	0	1	1	0	0	0	0	0	0	0	0	1	1	0	0	0
Verrucosispora	3	126	1	1	0	0	1	0	0	0	0	0	0	1	0	1	1	0	0	1
Vibrio	2	195	0	1	0	0	1	0	0	0	0	0	1	1	0	1	1	1	1	1
Vibrionales	1	46	0	0	0	0	0	0	0	0	0	0	0	0	0	0	1	0	1	1
Vitreoscilla	1	1	0	0	0	0	0	0	0	0	0	0	0	0	0	0	0	0	0	0
Vulcanisaeta	1	14	0	0	0	0	0	0	0	0	0	0	0	0	0	1	0	0	1	0
Waddlia	2	68	0	0	0	0	0	0	0	0	0	0	0	1	0	1	1	0	0	0
Wautersia	1	2	0	0	0	0	0	0	0	0	0	0	0	0	0	0	0	0	0	0
Weeksella	2	65	0	1	0	0	0	1	0	0	0	0	0	0	0	1	1	0	1	0

Genus	Cluster Number	Protein Counts	K00448	K00451	K00462	K00480	K00481	K00485	K00492	K00493	K00531	K00539	K00569	K00599	K00622	K00626	K00632	K00680	K00757	K00758
**Weissella**	1	14	0	0	0	0	0	0	0	0	0	0	0	1	0	1	0	1	0	0
**Wenxinia**	1	2	0	0	0	0	0	0	0	0	0	0	0	0	0	0	0	0	0	0
**Wigglesworthia**	1	29	0	0	0	0	0	0	0	0	0	0	0	0	0	0	0	0	0	0
**Wolbachia**	1	32	0	0	0	0	0	0	0	0	0	0	0	1	0	0	0	0	0	0
**Wolinella**	2	51	0	0	0	0	0	0	0	0	0	0	0	1	0	0	0	1	0	0
**Xanthobacter**	3	176	1	1	1	1	1	0	0	0	0	0	1	0	0	1	1	0	0	0
**Xanthobacteraceae**	1	4	0	0	0	0	0	0	0	0	0	0	0	0	0	0	0	0	0	0
**Xanthomonadaceae**	1	39	0	1	0	0	0	0	0	0	0	0	0	0	0	0	1	0	0	0
**Xanthomonas**	2	162	1	1	0	0	1	1	0	0	0	0	1	1	0	1	1	0	0	0
**Xenorhabdus**	2	127	0	0	0	0	0	0	0	0	0	0	0	0	0	0	1	0	1	1
**Xylanimonas**	2	66	0	0	0	0	0	0	1	0	0	0	0	1	0	1	1	0	0	1
**Xylella**	2	52	0	0	0	0	0	0	0	0	0	0	0	0	0	0	0	0	0	0
**Yersinia**	2	145	0	0	0	0	0	0	0	0	0	0	0	1	0	0	1	1	1	1
**Yokenella**	2	61	0	0	0	0	0	0	0	0	0	0	0	0	0	1	1	0	1	1
**Zobellia**	1	28	0	1	0	0	0	0	0	0	0	0	0	0	0	1	1	0	1	0
**Zunongwangia**	2	98	0	1	0	0	0	0	0	0	0	0	0	0	0	1	1	0	1	1
**Zymomonas**	2	67	0	0	0	0	0	0	0	0	1	0	0	0	0	0	0	0	0	0
**Zymophilus**	1	2	0	0	0	0	0	0	0	0	0	0	0	0	0	0	0	0	0	0

Genus	Cluster Number	Protein Counts	K00760	K00799	K00857	K00876	K01026	K01031	K01034	K01039	K01041	K01045	K01053	K01055	K01061	K01066	K01075	K01077	K01078	K01101
Acaricomes	1	2	1	0	0	0	0	0	0	0	0	0	0	0	0	0	0	0	0	0
Acaryochloris	2	88	0	1	0	1	0	0	0	0	0	0	0	0	1	0	0	0	0	0
Acetivibrio	1	2	0	0	0	0	0	0	0	0	0	0	0	0	0	0	0	0	0	0
Acetobacter	2	85	0	1	0	0	0	0	0	0	0	0	1	0	1	0	0	0	0	0
Acetobacteraceae	1	2	0	0	0	0	0	0	0	0	0	0	0	0	0	0	0	0	0	0
Acetobacterium	1	8	1	0	0	0	1	0	1	0	0	0	0	0	0	0	0	0	0	0
Acetohalobium	2	79	1	0	0	0	0	0	1	0	0	0	0	0	0	0	0	0	0	0
Acholeplasma	2	38	1	0	1	1	0	0	0	0	0	0	0	0	0	0	0	0	0	0
Achromobacter	2	192	0	1	0	0	0	1	0	1	0	0	1	1	1	0	1	0	0	0
Acidaminococcus	1	65	1	0	0	1	0	0	0	1	0	0	0	0	0	0	0	1	0	0
Acidianus	1	9	0	0	0	0	0	0	0	1	0	0	0	0	0	0	0	0	0	0
Acidilobus	1	8	0	0	0	0	0	0	0	1	0	0	0	0	0	0	0	0	0	0
Acidimicrobium	2	58	1	0	0	0	0	0	0	0	0	0	0	1	1	0	0	0	0	0
Acidiphilium	3	144	0	1	0	0	0	1	0	0	0	0	1	0	1	0	0	0	1	0
Acidithiobacillus	2	70	1	1	0	0	0	0	0	0	0	0	0	0	1	0	0	0	0	0
Acidobacterium	2	91	1	0	0	1	0	0	0	0	0	0	1	0	1	0	0	0	0	0
Acidocella	1	2	0	0	0	0	0	0	0	0	0	0	0	0	0	0	0	0	0	0
Acidothermus	2	68	1	0	0	0	0	0	0	1	0	0	0	0	1	0	0	0	0	0
Acidovorax	3	217	0	1	0	0	1	1	0	0	0	0	1	1	1	0	1	0	0	0
Aciduliprofundum	1	11	0	0	0	0	0	0	0	0	0	0	0	0	0	0	0	0	0	1
Acinetobacter	3	188	1	1	0	0	0	1	0	0	0	0	1	1	1	1	0	0	0	0
Actinobacillus	2	112	1	1	1	1	0	0	0	0	0	0	1	0	0	0	0	1	0	0
Actinomadura	1	1	0	0	0	0	0	0	0	0	0	0	0	0	0	0	0	0	0	0
Actinoplanes	1	52	1	0	1	1	0	1	0	0	0	0	1	1	1	0	0	1	0	0
Actinopolyspora	1	2	0	0	0	0	0	0	0	0	0	0	0	0	0	0	0	0	0	0
Actinosynnema	2	122	1	0	1	1	0	0	0	1	0	0	0	1	1	0	0	1	0	0
Advenella	1	30	0	1	0	0	1	1	0	1	0	0	1	1	1	0	0	0	0	0
Aequorivita	1	12	0	0	1	1	0	0	0	0	0	0	0	0	0	0	0	1	0	0
Aerococcus	2	57	1	0	1	0	1	0	0	0	0	0	0	0	0	0	0	0	0	1
Aeromonas	2	127	1	1	1	1	0	0	0	0	0	0	0	0	0	0	0	1	0	0
Aeropyrum	1	13	0	0	0	0	1	0	0	0	0	0	0	0	0	0	0	0	0	1
Afipia	1	54	0	1	0	0	0	0	0	0	1	0	0	0	0	0	0	0	0	0
Aggregatibacter	1	81	1	1	1	1	0	0	1	0	0	0	0	0	0	0	0	0	0	0
Agrobacterium	2	242	1	1	1	1	0	1	0	0	0	0	1	1	1	0	0	1	1	0
Ahrensia	1	2	0	0	0	0	0	0	0	0	0	0	0	0	0	0	0	0	0	0
Akkermansia	2	47	1	0	0	0	0	0	0	0	0	0	0	0	0	0	0	1	0	0
Albidiferax	3	144	0	1	1	0	1	1	0	0	0	0	0	0	1	0	1	0	0	0
Alcaligenes	1	21	0	0	0	0	0	0	0	0	0	0	0	0	0	0	0	0	0	0
Alcanivorax	2	146	1	1	0	1	0	0	0	0	0	0	1	0	1	1	1	1	0	0
Alicycliphilus	3	152	0	1	0	0	0	1	0	0	0	0	1	1	1	0	0	0	0	0
Alicyclobacillus	2	91	1	0	1	0	0	0	0	0	0	0	0	0	1	0	0	0	0	0
Aliivibrio	2	118	1	1	1	1	0	0	0	0	0	0	0	0	0	0	0	0	0	0
Alishewanella	1	1	0	0	0	0	0	0	0	0	0	0	0	0	0	0	0	0	0	0
Alistipes	1	52	1	0	1	1	0	0	0	0	0	0	0	0	0	0	0	1	0	0
Alkalilimnicola	2	97	1	1	0	0	0	0	0	0	0	0	0	0	0	0	0	1	0	0
Alkaliphilus	2	62	1	0	1	1	1	0	1	0	0	0	0	0	0	0	0	1	0	0
Allochromatium	2	84	1	0	0	0	0	0	0	0	0	0	0	0	0	0	0	0	0	0
Alphaproteobacteria	1	3	0	0	0	0	0	0	0	0	0	0	0	0	0	0	0	0	0	0
Alteromonadales	1	12	0	0	0	1	0	0	0	0	0	0	0	0	0	0	0	0	0	0
Alteromonas	1	37	0	1	1	1	0	0	0	1	0	0	0	1	1	0	0	1	0	0
Aminobacter	1	4	0	0	0	0	0	0	0	0	0	0	0	0	0	0	0	0	0	0
Aminobacterium	2	59	1	0	0	1	0	0	1	0	0	0	0	0	0	0	0	1	0	0

Genus	Cluster Number	Protein Counts	K00760	K00799	K00857	K00876	K01026	K01031	K01034	K01039	K01041	K01045	K01053	K01055	K01061	K01066	K01075	K01077	K01078	K01101
Ammonifex	2	50	1	0	0	0	0	0	0	0	0	0	0	0	0	0	0	0	0	0
Amphibacillus	1	13	1	0	1	0	0	0	0	0	0	0	0	0	0	0	0	0	0	0
Amphritea	1	2	0	0	0	0	0	0	0	0	0	0	0	0	0	0	0	0	0	0
Amycolatopsis	2	201	1	0	1	0	0	0	0	1	1	0	1	1	1	0	0	1	1	0
Amycolicicoccus	1	43	1	0	0	0	0	0	0	0	1	0	0	1	1	0	0	0	0	0
Anabaena	2	92	0	1	0	1	0	0	0	0	0	0	0	0	1	0	0	1	1	0
Anaerobaculum	1	11	1	0	0	1	0	0	0	0	0	0	0	0	0	0	0	0	0	0
Anaerococcus	2	46	1	0	1	1	0	0	0	0	0	0	0	0	0	0	0	0	0	0
Anaerolinea	2	66	1	0	1	1	0	0	0	1	0	0	0	0	0	0	0	0	0	1
Anaeromyxobacter	3	117	1	1	0	1	0	0	0	0	0	0	0	0	0	0	1	1	0	0
Anaerostipes	1	44	0	0	0	1	0	0	0	0	0	0	0	0	0	0	0	0	0	0
Anaerotruncus	1	2	0	0	0	0	0	0	0	0	0	0	0	0	0	0	0	0	0	0
Anaplasma	1	35	0	1	0	0	0	0	0	0	0	0	0	0	0	0	0	0	0	0
Ancylobacter	1	3	0	0	0	0	0	0	0	0	0	0	0	0	0	0	0	0	0	0
Aneurinibacillus	1	1	0	0	0	0	0	0	0	0	0	0	0	0	0	0	0	0	0	0
Angomonas	1	1	0	0	0	0	0	0	0	0	0	0	0	0	0	0	0	0	0	0
Anoxybacillus	2	72	1	0	1	1	0	0	0	0	0	0	0	0	0	0	0	0	0	1
Aquifex	2	45	1	0	0	0	0	0	0	0	0	0	0	0	1	0	0	0	0	0
Aquimarina	1	2	0	0	0	0	0	0	0	0	0	0	0	0	0	0	0	0	0	0
Arcanobacterium	2	45	0	0	1	1	0	0	0	0	0	0	0	0	0	1	0	0	0	0
Archaeoglobus	1	14	1	0	0	0	0	0	0	1	0	0	0	0	0	0	0	0	0	0
Arcobacter	2	101	0	1	0	0	0	0	1	0	0	0	0	0	0	0	0	0	0	0
Aromatoleum	3	133	1	1	0	0	0	0	0	0	0	0	0	0	0	0	0	0	0	0
Arsenophonus	1	4	0	0	0	0	0	0	0	0	0	0	0	0	0	0	0	0	0	0
Arthrobacter	2	206	1	0	1	1	0	1	0	0	0	0	1	1	1	0	0	1	0	0
Arthrospira	2	67	0	0	0	1	0	0	0	0	0	0	0	0	1	0	0	0	0	0
Aster	1	10	0	0	1	1	0	0	0	0	0	0	0	0	0	0	0	0	0	0
Asticcacaulis	2	103	1	1	1	0	0	0	0	0	0	0	1	0	1	0	0	1	0	0
Atopobium	1	28	1	0	0	0	0	0	0	0	0	0	0	0	0	0	0	0	0	0
Aurantimonas	1	2	0	0	0	0	0	0	0	0	0	0	0	0	0	0	0	0	0	0
Avibacterium	1	17	0	0	0	0	0	0	0	0	0	0	0	0	0	0	0	0	0	0
Azoarcus	3	172	1	1	0	0	1	0	1	0	1	0	1	0	1	0	1	1	0	0
Azorhizobium	3	149	1	1	0	0	0	1	0	0	0	0	1	1	1	0	1	1	0	0
Azorhizophilus	1	4	0	0	0	0	0	0	0	0	0	0	0	0	0	0	0	0	0	0
Azospira	1	19	1	1	0	0	0	0	0	1	0	0	0	0	1	0	0	0	0	0
Azospirillum	2	198	0	1	0	0	0	0	1	1	0	0	1	1	1	0	1	1	0	0
Azotobacter	3	183	1	1	0	0	0	0	0	1	0	0	0	1	1	0	1	1	0	0
Bacillales	1	9	1	0	1	1	0	0	0	0	0	0	0	0	0	0	0	0	0	0
Bacilli	1	3	0	0	0	0	0	0	0	0	0	0	0	0	0	0	0	0	0	0
Bacillus	2	237	1	1	1	1	1	0	1	1	1	0	0	0	1	1	0	1	0	1
Bacteria	2	86	0	1	0	0	0	1	0	0	0	0	0	0	1	0	0	0	0	0
Bacteriovorax	2	98	1	1	1	1	0	0	0	0	0	0	0	0	0	0	0	1	0	0
Bacteroidales	2	59	0	0	1	1	0	0	0	0	0	0	0	0	0	0	0	1	0	0
Bacteroides	2	96	1	0	1	1	0	0	0	0	1	0	0	0	0	0	0	1	0	0
Bacteroidetes	1	2	0	0	0	0	0	0	0	0	0	0	0	0	0	0	0	0	0	0
Bartonella	2	50	0	1	0	0	0	0	0	0	0	0	0	0	0	0	0	0	0	0
Baumannia	2	24	0	0	0	0	0	0	0	0	0	0	0	0	0	0	0	0	0	0
Bdellovibrio	2	87	1	1	1	1	0	0	0	0	0	0	0	1	0	1	0	1	0	0
Beijerinckia	3	123	0	1	0	1	0	0	1	0	0	0	1	1	1	0	0	0	0	0
Belliella	1	14	1	0	1	1	0	0	0	0	0	0	1	0	1	0	0	0	0	0
Beutenbergia	2	82	1	0	1	1	0	0	0	0	0	0	1	0	1	0	0	0	0	0
Bifidobacterium	2	61	1	0	0	0	0	0	0	0	0	0	0	0	0	0	0	1	0	0

Genus	Cluster Number	Protein Counts	K00760	K00799	K00857	K00876	K01026	K01031	K01034	K01039	K01041	K01045	K01053	K01055	K01061	K01066	K01075	K01077	K01078	K01101
Bizionia	1	1	0	0	0	0	0	0	0	0	0	0	0	0	0	0	0	0	0	0
Blastococcus	1	39	1	0	1	0	0	1	0	0	0	0	0	1	1	0	0	0	0	0
Blattabacterium	1	23	0	0	0	0	0	0	0	0	0	0	0	0	0	0	0	0	0	0
Blautia	1	22	1	0	0	0	0	0	0	0	0	0	0	0	0	0	0	0	0	0
Bordetella	3	227	0	1	0	0	0	1	0	1	0	0	1	1	1	1	1	0	0	0
Borrelia	1	32	1	0	1	1	0	0	0	0	0	0	0	0	0	0	0	0	0	0
Brachybacterium	1	18	1	0	1	1	0	0	0	0	0	0	0	0	1	0	0	0	0	0
Brachymonas	1	4	0	0	0	0	0	0	0	0	0	0	0	0	0	0	0	0	0	0
Brachyspira	2	79	1	0	1	0	1	0	0	0	0	0	0	0	0	0	0	1	0	0
Bradyrhizobiaceae	1	20	0	1	0	0	0	0	0	0	1	0	0	0	0	0	0	0	0	0
Bradyrhizobium	3	254	0	1	0	1	1	0	1	0	1	0	1	1	1	1	1	1	0	0
Brenneria	1	20	1	0	0	1	0	0	0	0	0	0	0	0	0	0	0	0	0	0
Brevibacillus	2	122	1	0	1	1	0	0	1	0	0	0	0	0	0	0	0	1	0	0
Brevibacterium	1	4	0	0	0	0	0	0	0	0	0	0	0	0	0	0	0	0	0	0
Brevundimonas	2	105	1	1	0	1	0	0	0	0	0	0	0	0	1	0	0	0	0	0
Brucella	3	138	1	1	0	0	0	1	0	0	1	0	0	1	0	1	0	1	0	0
Buchnera	1	26	1	0	0	0	0	0	0	0	0	0	0	0	0	0	0	0	0	0
Burkholderia	3	424	1	1	0	0	1	1	0	1	1	0	1	1	1	1	1	1	1	1
Burkholderiaceae	3	143	0	1	0	0	1	1	0	0	1	0	1	1	1	1	1	0	0	0
Burkholderiales	1	24	0	0	0	0	0	0	0	0	0	0	0	0	1	0	0	0	0	0
Buttiauxella	1	2	0	0	0	0	0	0	0	0	0	0	0	0	0	0	0	0	0	0
Butyrivibrio	2	67	1	0	1	1	0	0	0	0	0	0	0	0	0	0	0	0	0	0
Caldanaerobacter	2	63	1	0	1	1	0	0	1	0	0	0	0	0	0	0	0	0	0	0
Caldicellulosiruptor	2	75	1	0	0	1	0	0	0	0	0	0	0	0	0	0	0	1	0	0
Caldilinea	1	23	1	0	1	1	0	0	0	0	0	0	1	0	1	0	0	1	0	0
Caldimonas	1	6	0	0	0	0	0	0	0	0	0	0	0	0	0	0	0	0	0	0
Caldisericum	1	10	1	0	1	1	0	0	0	0	0	0	0	0	0	0	0	0	0	0
Caldisphaera	1	7	0	0	0	0	0	0	0	1	0	0	0	0	0	0	0	0	0	0
Calditerrivibrio	2	74	1	0	0	0	0	0	0	0	0	0	0	0	0	0	0	1	0	0
Caldivirga	1	13	0	0	0	0	0	0	0	0	0	0	0	0	1	0	0	0	0	0
Calothrix	1	43	0	1	0	0	0	0	0	0	0	0	0	0	1	0	0	1	0	0
Calyptogena	2	34	0	1	0	0	0	0	0	0	0	0	0	0	0	0	0	0	0	0
Campylobacter	2	80	0	0	1	0	0	0	0	0	0	0	0	0	0	0	0	0	0	0
Candidatus	2	290	1	1	1	1	1	0	0	1	0	0	1	1	1	1	0	0	1	0
Capnocytophaga	1	50	0	0	1	1	0	0	0	0	0	0	0	0	0	0	0	1	0	0
Carbophilus	1	2	0	0	0	0	0	0	0	0	0	0	0	0	0	0	0	0	0	0
Carboxydibrachium	1	14	0	0	0	1	0	0	1	0	0	0	0	0	0	0	0	0	0	0
Carboxydothermus	2	68	1	0	1	0	0	0	0	0	0	0	0	0	0	0	0	0	0	0
Carica	1	2	0	0	0	0	0	0	0	0	0	0	0	0	0	0	0	0	0	0
Carnobacterium	2	71	1	0	1	1	1	0	0	0	0	0	0	0	0	0	0	0	0	1
Catenibacterium	1	2	0	0	0	0	0	0	0	0	0	0	0	0	0	0	0	0	0	0
Catenovulum	1	2	0	0	0	0	0	0	0	0	0	0	0	0	0	0	0	0	0	0
Catenulispora	3	159	1	0	1	1	0	1	0	1	1	0	0	0	1	0	0	0	1	0
Caulobacter	3	168	1	1	0	0	0	0	0	0	0	0	1	0	1	0	0	1	1	0
Cedecea	1	45	1	0	0	1	0	0	0	0	0	0	0	0	0	0	0	0	0	0
Cellulomonas	2	96	1	0	1	1	0	0	0	0	0	0	0	0	0	0	0	1	0	0
Cellulophaga	2	105	0	0	1	1	0	0	0	0	0	0	1	0	1	0	0	1	0	0
Cellulosilyticum	1	14	1	0	1	1	0	0	0	0	0	0	0	0	0	0	0	1	0	0
Cellvibrio	2	85	1	0	0	0	0	0	0	0	0	0	1	0	1	0	0	1	0	0
Cenarchaeum	1	7	0	0	0	0	0	0	0	0	0	0	0	0	0	0	0	0	0	0
Chamaesiphon	1	13	0	1	0	0	0	0	0	0	0	0	0	0	1	0	0	0	0	0
Chelativorans	3	136	1	1	0	0	0	0	0	1	0	0	1	1	1	0	0	0	0	0

Genus	Cluster Number	Protein Counts	K00760	K00799	K00857	K00876	K01026	K01031	K01034	K01039	K01041	K01045	K01053	K01055	K01061	K01066	K01075	K01077	K01078	K01101
Chelatococcus	1	2	0	0	0	0	0	0	0	0	0	0	0	0	0	0	0	0	0	0
Chitinophaga	2	98	1	0	1	0	0	0	0	0	0	0	1	0	1	0	0	1	0	0
Chlamydia	1	36	0	0	0	1	0	0	0	0	0	0	0	0	0	0	0	0	0	0
Chlamydomonas	1	3	0	0	0	0	0	0	0	0	0	0	0	0	0	0	0	0	0	0
Chlamydophila	1	31	0	0	0	1	0	0	0	0	0	0	0	0	0	0	0	0	0	0
Chlorobaculum	2	61	1	0	0	1	0	0	0	0	0	0	0	0	0	0	0	0	0	0
Chlorobium	2	89	1	0	0	1	0	0	0	0	0	0	0	0	0	0	0	1	1	0
Chlorobium/Pelodictyon	1	2	0	0	0	0	0	0	0	0	0	0	0	0	0	0	0	0	0	0
Chloroflexus	2	105	1	0	1	1	0	0	0	0	0	0	0	0	1	0	0	1	0	1
Chlorogloeopsis	1	9	0	0	0	0	0	0	0	0	0	0	0	0	0	0	0	0	0	0
Chloroherpeton	2	59	1	0	1	0	0	0	0	0	0	0	0	0	0	0	0	1	0	0
Chromobacterium	2	117	1	1	1	1	0	0	0	0	0	0	0	1	1	1	0	1	1	0
Chromohalobacter	2	145	1	1	0	0	0	0	0	1	0	0	1	0	1	0	1	0	0	0
Chroococcidiopsis	1	24	1	1	0	0	0	0	0	0	0	0	1	0	1	0	0	1	0	0
Chryseobacterium	2	104	0	1	0	0	0	0	0	0	0	0	1	1	1	0	0	0	0	0
Citreicella	1	13	0	0	0	0	0	0	0	0	0	0	0	0	0	0	0	0	0	0
Citrobacter	2	190	1	1	1	1	0	1	1	0	0	0	0	1	1	1	0	1	0	0
Clavibacter	2	83	1	0	1	0	0	0	0	0	0	0	0	0	1	0	0	0	0	0
Clonorchis	1	1	0	0	0	0	0	0	0	0	0	0	0	0	0	0	0	0	0	0
Clostridiales	2	77	1	0	1	1	1	0	0	0	0	0	0	0	0	0	0	0	0	0
Clostridium	2	191	1	0	1	1	1	0	1	1	0	0	0	0	0	1	0	1	0	0
Cohnella	1	2	0	0	0	0	0	0	0	0	0	0	0	0	0	0	0	0	0	0
Coleofasciculus	1	4	0	0	0	0	0	0	0	0	0	0	0	0	0	0	0	0	0	0
Collimonas	1	35	0	1	0	0	0	1	0	0	0	0	0	1	1	0	0	1	0	0
Colwellia	2	130	1	1	1	1	0	1	0	0	0	0	0	0	1	0	0	1	0	0
Comamonas	2	196	0	1	0	0	0	1	0	1	0	0	0	0	1	0	1	0	0	0
Conexibacter	3	117	1	1	0	0	0	1	0	0	1	0	1	0	0	0	0	1	0	0
Coprobacillus	1	2	0	0	0	0	0	0	0	0	0	0	0	0	0	0	0	0	0	0
Coprococcus	2	76	1	0	0	1	1	0	1	0	0	0	0	0	0	0	0	0	0	0
Coprothermobacter	2	45	1	0	1	1	0	0	0	0	0	0	0	0	0	0	0	0	0	0
Coraliomargarita	2	68	1	0	0	0	0	0	0	0	0	0	0	0	0	0	0	1	0	0
Corallococcus	1	29	1	1	1	1	0	0	0	1	0	0	0	0	1	0	0	0	0	0
Coriobacterium	2	36	1	0	0	0	0	0	0	0	0	0	0	0	0	0	0	0	0	0
Corynebacterineae	1	5	0	0	0	0	0	0	0	0	0	0	0	0	0	0	0	0	0	0
Corynebacterium	2	175	1	0	1	0	0	0	0	0	1	0	0	1	0	1	0	1	1	0
Coxiella	2	54	1	0	1	1	0	0	0	0	0	0	0	0	0	0	0	0	0	0
Crinalium	1	21	0	1	0	0	0	0	0	0	0	0	1	0	1	0	0	0	0	0
Croceibacter	2	80	0	0	1	1	0	0	0	0	0	0	0	0	0	0	0	0	0	0
Crocosphaera	1	6	0	1	0	0	0	0	0	0	0	0	0	0	0	0	0	0	0	0
Cronobacter	2	120	1	1	1	1	0	0	0	0	0	0	0	0	1	0	0	0	0	0
Cryptobacterium	2	39	1	0	0	0	0	0	0	0	0	0	0	0	0	0	0	0	0	0
Cucumis	1	9	1	0	0	1	0	0	0	0	0	0	0	0	0	0	0	0	0	0
Cupriavidus	3	317	1	1	0	0	1	1	0	1	1	0	1	1	1	1	1	1	0	0
Curtobacterium	1	5	0	0	0	0	0	0	0	0	0	0	0	0	0	0	0	0	0	0
Curvibacter	1	27	0	0	0	0	0	0	0	0	0	0	0	0	0	0	0	0	0	0
Cyanobacterium	1	9	0	1	0	0	0	0	0	0	0	0	0	0	1	0	0	0	0	0
Cyanobium	1	10	0	1	0	0	0	0	0	0	0	0	0	0	1	0	0	0	0	0
Cyanothece	1	34	0	1	0	1	0	0	0	0	0	0	1	0	1	0	0	1	0	0
Cyclobacterium	1	19	1	0	1	1	0	0	0	0	0	0	1	0	1	0	0	1	0	0
Cycloclasticus	1	21	1	1	0	0	0	0	0	0	0	0	0	0	0	0	0	0	0	0
Cylindrospermopsis	1	4	0	0	0	0	0	0	0	0	0	0	0	0	0	0	0	0	0	0
Cylindrospermum	1	30	0	1	0	0	0	0	0	0	0	0	0	0	1	0	0	1	0	0

Genus	Cluster Number	Protein Counts	K00760	K00799	K00857	K00876	K01026	K01031	K01034	K01039	K01041	K01045	K01053	K01055	K01061	K01066	K01075	K01077	K01078	K01101
Cytophaga	2	69	0	0	0	1	0	0	0	0	0	0	1	0	1	1	0	0	0	0
Dactylococcopsis	1	8	0	1	0	0	0	0	0	0	0	0	0	0	1	0	0	0	0	0
Dechloromonas	1	40	1	1	0	0	1	0	0	0	0	0	0	0	1	0	0	1	0	0
Dechlorosoma	1	19	1	1	0	0	0	0	0	1	0	0	0	0	1	0	0	0	0	0
Deferribacter	2	73	1	0	0	0	0	0	0	0	0	0	0	0	0	0	0	0	0	0
Dehalobacter	1	9	1	0	0	1	0	0	0	0	0	0	0	0	0	0	0	0	0	0
Dehalococcoides	2	41	1	0	0	0	0	0	0	0	0	0	0	0	0	0	0	0	0	0
Dehalogenimonas	1	37	1	0	0	0	0	0	0	0	0	0	0	0	0	0	0	1	0	0
Deinococcus	2	140	1	0	1	1	0	1	0	0	0	0	1	0	0	0	0	1	0	0
Delftia	3	177	0	1	0	0	0	1	0	0	0	0	1	1	1	0	0	1	0	0
Denitrovibrio	2	67	1	0	0	0	0	0	0	0	0	0	0	0	0	0	0	1	0	0
Desulfarculus	2	92	1	0	0	0	0	0	0	0	0	0	0	0	0	0	0	1	0	0
Desulfatibacillum	3	84	1	0	0	0	0	0	0	1	0	0	0	0	0	0	0	0	0	0
Desulfitobacterium	2	102	1	0	1	1	0	0	0	1	0	0	0	0	0	0	0	1	0	0
Desulfobacca	2	65	1	0	0	0	0	0	0	0	0	0	0	0	0	0	0	1	0	0
Desulfobacterium	2	113	1	0	0	0	0	0	0	1	0	0	0	0	1	0	0	1	0	0
Desulfobacula	1	21	1	0	0	0	0	0	0	1	0	0	0	0	0	0	0	0	0	0
Desulfobulbus	2	88	1	0	0	0	0	0	0	1	0	0	0	0	0	0	0	1	0	0
Desulfocapsa	1	9	1	0	0	0	0	0	0	0	0	0	0	0	1	0	0	0	0	0
Desulfococcus	2	80	1	0	0	0	0	0	0	0	0	0	0	0	0	0	0	0	0	0
Desulfohalobium	2	52	1	0	0	0	0	0	0	0	0	0	0	1	0	0	0	1	0	0
Desulfomicrobium	2	65	1	0	0	0	0	0	0	0	0	0	0	0	0	0	0	0	0	0
Desulfomonile	1	25	1	0	0	0	0	0	0	1	0	0	0	0	1	0	0	1	0	0
Desulforhabdus	1	2	0	0	0	0	0	0	0	0	0	0	0	0	0	0	0	0	0	0
Desulfosporosinus	1	30	1	0	1	1	1	0	1	1	0	0	0	0	1	0	0	1	0	0
Desulfotalea	2	66	1	0	0	0	0	0	0	0	0	0	0	0	0	0	0	0	0	0
Desulfotomaculum	2	98	1	0	0	1	1	0	0	1	1	0	0	1	0	0	0	1	0	0
Desulfovibrio	2	144	1	0	0	0	0	0	0	0	0	0	0	0	0	1	0	1	0	0
Desulfurispirillum	2	78	1	1	0	0	0	0	0	0	0	0	0	0	0	0	0	0	0	0
Desulfurivibrio	2	71	1	0	0	0	0	0	0	0	0	0	0	0	0	0	0	0	0	0
Desulfurobacterium	2	53	1	0	0	0	0	0	0	0	0	0	0	0	0	0	0	0	0	1
Desulfurococcus	1	11	0	0	0	0	0	0	0	0	0	0	0	0	0	0	0	0	0	0
Diaphorobacter	1	16	0	0	0	0	0	0	0	0	0	0	0	0	0	0	0	0	0	0
Dichelobacter	2	45	1	1	0	0	0	0	0	0	0	0	0	0	0	0	0	1	0	0
Dickeya	2	155	1	1	1	1	0	0	1	0	0	0	0	0	1	0	0	1	0	0
Dictyoglomus	2	62	1	0	1	1	0	0	0	0	0	0	0	0	0	0	0	1	0	1
Dinoroseobacter	3	148	1	1	1	0	1	0	0	0	0	0	0	1	0	0	0	0	0	0
Dokdonia	1	3	0	0	0	0	0	0	0	0	0	0	0	0	0	0	0	0	0	0
Dorea	1	7	0	0	0	0	0	0	0	0	0	0	0	0	0	0	0	0	0	0
Dyadobacter	2	83	1	0	1	1	0	0	0	0	0	0	1	0	1	0	0	1	0	0
Echinicola	1	20	1	0	1	1	0	0	0	0	0	0	1	1	1	0	0	1	0	0
Edwardsiella	2	126	1	1	1	1	0	0	1	0	0	0	0	0	0	1	0	1	0	0
Eggerthella	1	51	1	0	0	0	0	0	0	0	0	0	0	0	0	1	0	0	0	0
Ehrlichia	2	37	0	1	0	0	0	0	0	0	0	0	0	0	0	0	0	0	0	0
Eikenella	1	4	0	0	0	0	0	0	0	0	0	0	0	0	0	0	0	0	0	0
Elusimicrobium	2	44	1	0	1	0	0	0	0	0	0	0	0	0	0	0	0	1	0	0
Emticicia	1	21	1	0	1	1	0	0	0	0	0	0	1	0	1	0	0	0	0	0
Enhydrobacter	1	1	0	0	0	0	0	0	0	0	0	0	0	0	0	0	0	0	0	0
Ensifer	1	2	0	0	0	0	0	0	0	0	0	0	0	0	0	0	0	0	0	0
Enterobacter	2	202	1	1	1	1	0	1	0	0	0	0	1	1	1	0	0	1	0	0
Enterobacteriaceae	2	211	1	1	1	1	0	1	1	0	0	0	1	1	1	1	0	1	0	0
Enterococcus	1	187	1	1	1	1	0	1	0	0	0	0	1	1	1	0	0	1	0	0

Genus	Cluster Number	Protein Counts	K00760	K00799	K00857	K00876	K01026	K01031	K01034	K01039	K01041	K01045	K01053	K01055	K01061	K01066	K01075	K01077	K01078	K01101
Enterovibrio	1	15	0	0	0	0	0	0	0	0	0	0	0	0	0	0	0	0	0	0
Erwinia	2	151	1	1	1	1	0	0	0	0	0	0	1	0	1	0	0	1	0	0
Erysipelothrix	1	9	1	0	1	1	0	0	0	0	0	0	0	0	0	0	0	0	0	0
Erysipelotrichaceae	1	4	0	0	0	0	0	0	0	0	0	0	0	0	0	0	0	0	0	0
Erythrobacter	2	80	0	1	1	0	0	0	0	0	0	0	0	0	1	0	0	1	0	0
Escherichia	2	242	1	1	1	1	0	1	1	0	1	0	0	1	1	1	0	1	0	0
Ethanoligenens	2	69	1	0	0	1	0	0	0	0	0	0	0	0	0	0	0	0	0	0
Eubacteriaceae	1	2	0	0	0	0	0	0	0	0	0	0	0	0	0	0	0	0	0	0
Eubacterium	2	103	1	0	0	1	1	0	0	0	0	0	1	0	0	0	0	0	0	0
Eudoraea	1	4	0	0	0	0	0	0	0	0	0	0	0	0	0	0	0	0	0	0
Euryarchaeota	1	1	0	0	0	0	0	0	0	0	0	0	0	0	0	0	0	0	0	0
Exiguobacterium	2	93	1	0	1	1	0	0	0	0	0	0	0	0	0	0	0	1	0	1
Faecalibacterium	1	12	1	0	1	1	0	0	0	0	0	0	0	0	0	0	0	1	0	0
Ferrimonas	2	115	1	1	1	1	0	0	0	0	0	0	0	0	0	0	0	1	0	0
Ferroglobus	1	14	0	0	0	0	0	0	0	1	0	0	0	0	0	0	0	0	0	1
Ferroplasma	1	11	0	0	1	0	0	0	0	0	0	0	0	0	0	0	0	0	0	0
Fervidicoccus	1	7	0	0	0	0	0	0	0	0	0	0	0	0	0	0	0	0	0	0
Fervidobacterium	2	54	1	0	1	1	0	0	1	0	0	0	0	0	0	0	0	1	0	0
Fibrella	1	17	1	0	1	1	0	0	0	0	0	0	1	0	1	0	0	1	0	0
Fibrobacter	2	52	1	0	0	0	0	0	0	0	0	0	0	0	0	0	0	0	0	0
Filifactor	1	9	1	0	1	1	0	0	0	1	0	0	0	0	0	0	0	0	0	0
Finegoldia	2	55	1	1	1	1	0	0	0	0	0	0	0	0	0	0	0	0	0	0
Firmicutes	1	15	1	0	1	1	0	0	0	0	0	0	0	0	0	0	0	0	0	0
Fischerella	1	15	0	0	0	0	0	0	0	0	0	0	0	0	0	0	0	0	0	0
Flavobacteria	1	7	0	0	0	0	0	0	0	0	0	0	0	0	0	0	0	0	0	0
Flavobacteriaceae	2	69	1	0	1	1	0	0	0	0	0	0	0	0	0	0	0	0	0	0
Flavobacteriales	1	3	0	0	0	0	0	0	0	0	0	0	0	0	0	0	0	0	0	0
Flavobacterium	2	103	1	0	1	1	0	0	0	0	0	0	0	0	1	0	1	1	0	0
Flexibacter	1	17	1	0	1	1	0	0	0	0	0	0	0	0	1	0	0	0	0	0
Flexistipes	1	11	1	0	0	0	0	0	0	0	0	0	0	0	0	0	0	0	0	0
Fluoribacter	1	6	0	0	0	0	0	0	0	0	0	0	0	0	0	0	0	0	0	0
Fluviicola	2	69	1	0	1	1	0	0	0	0	0	0	0	0	0	0	0	0	0	0
Formosa	1	2	0	0	0	0	0	0	0	0	0	0	0	0	0	0	0	0	0	0
Francisella	2	88	1	0	1	1	0	0	0	0	0	0	0	0	0	1	0	0	1	0
Frankia	3	202	1	0	0	1	0	0	0	0	1	0	1	1	1	0	0	0	1	0
Frateuria	1	25	1	1	1	0	0	0	0	0	0	0	1	0	1	0	0	0	0	0
Fructobacillus	1	2	0	0	0	0	0	0	0	0	0	0	0	0	0	0	0	0	0	0
Fulvimarina	1	2	0	0	0	0	0	0	0	0	0	0	0	0	0	0	0	0	0	0
Fusobacterium	1	48	1	0	0	0	1	0	0	1	0	0	0	0	0	0	0	0	0	1
Gallibacterium	2	87	1	0	1	1	0	0	0	0	0	0	0	0	0	0	0	1	0	0
Gallionella	2	67	1	1	0	0	0	0	0	0	0	0	0	0	0	0	0	0	0	0
Gammaproteobacteria	1	9	0	1	0	0	0	0	0	0	0	0	0	0	0	0	0	0	0	0
Gardnerella	1	31	1	0	0	0	0	0	0	0	0	0	0	0	0	0	0	0	0	0
Geitlerinema	1	9	0	1	0	0	0	0	0	0	0	0	0	0	1	0	0	0	0	0
Gemmatimonas	2	93	1	0	1	1	0	0	0	0	0	0	0	0	1	0	1	0	0	0
Geobacillus	2	168	1	0	1	1	0	0	0	0	1	0	0	0	0	0	0	1	0	1
Geobacter	2	150	1	0	0	0	0	0	0	1	0	0	0	0	0	0	0	1	0	0
Geodermatophilus	2	136	1	0	1	0	1	1	0	0	0	0	0	1	1	0	0	0	0	0
Geopsychrobacter	1	2	0	0	0	0	0	0	0	0	0	0	0	0	0	0	0	0	0	0
Gillisia	1	11	0	0	1	0	0	0	0	0	0	0	0	0	0	0	0	0	0	0
Gilvimarinus	1	4	0	0	0	0	0	0	0	0	0	0	0	0	0	0	0	0	0	0
Glaciecola	2	117	0	1	1	1	0	0	0	0	0	0	1	1	1	0	0	1	0	0

Genus	Cluster Number	Protein Counts	K00760	K00799	K00857	K00876	K01026	K01031	K01034	K01039	K01041	K01045	K01053	K01055	K01061	K01066	K01075	K01077	K01078	K01101
Gloeobacter	2	67	1	1	0	0	0	0	0	0	0	0	0	0	1	0	0	1	0	0
Gloeocapsa	1	20	0	1	0	0	0	0	0	0	0	0	1	0	1	0	0	1	0	0
Gloeothece	1	1	0	0	0	0	0	0	0	0	0	0	0	0	0	0	0	0	0	0
Gluconacetobacter	2	96	0	1	0	0	0	0	0	0	0	0	1	0	1	0	0	0	0	0
Gluconobacter	2	76	0	1	1	0	0	0	0	0	0	0	1	0	1	0	0	0	1	0
Gordonia	1	158	1	0	1	0	0	0	0	0	0	0	1	1	1	0	0	0	0	0
Gordonibacter	1	32	0	0	0	0	0	0	0	0	0	0	0	0	0	0	0	0	0	0
Gramella	2	104	0	0	1	1	0	0	0	0	0	0	0	0	0	1	0	0	0	0
Granulibacter	2	90	0	1	0	0	0	0	0	0	0	0	0	0	1	1	0	0	1	0
Granulicella	2	98	1	0	1	1	0	0	0	0	0	0	0	0	1	0	0	0	0	0
Grimontia	1	9	0	0	0	0	0	0	0	0	0	0	0	0	0	0	0	0	0	0
Haemophilus	2	117	1	1	1	1	0	0	1	0	0	0	1	0	0	0	0	0	0	0
Hafnia	1	29	1	0	0	1	0	0	0	0	0	0	0	0	0	0	0	0	0	0
Hahella	3	130	1	1	0	1	1	0	0	0	0	0	0	0	1	0	0	1	0	0
Halalkalicoccus	1	18	0	0	1	1	0	0	0	0	0	0	0	0	0	0	0	0	0	1
Halanaerobium	2	89	1	0	1	1	1	0	1	0	0	0	0	0	0	0	0	1	0	0
Haliangium	2	108	1	1	0	1	0	0	0	0	0	0	1	0	0	0	0	0	0	0
Haliscomenobacter	2	107	1	0	1	1	0	0	0	0	0	0	1	0	1	0	0	1	0	0
Haloarcula	1	22	0	0	0	0	0	0	0	0	0	0	0	0	0	0	0	0	0	0
Halobacillus	1	19	1	0	1	1	0	0	1	0	0	0	0	0	0	0	0	1	0	0
Halobacteriaceae	1	1	0	0	0	0	0	0	0	0	0	0	0	0	0	0	0	0	0	0
Halobacterium	1	10	0	0	1	1	0	0	0	0	0	0	0	0	0	0	0	1	0	0
Halobacteroides	1	14	1	0	1	1	0	0	0	0	0	0	0	0	0	0	0	1	0	0
Haloferax	1	20	0	0	1	1	1	0	0	0	0	0	0	0	0	0	0	0	0	1
Halogeometricum	1	12	0	0	1	1	0	0	0	0	0	0	0	0	0	0	0	0	0	0
Halomicrobium	1	10	0	0	0	1	0	0	0	0	0	0	0	0	0	0	0	0	0	0
Halomonas	1	149	1	1	0	0	1	0	0	1	0	0	1	0	1	0	1	1	0	0
Halopiger	1	16	0	0	0	1	0	0	0	0	0	0	0	0	0	0	0	1	0	1
Haloquadratum	1	19	0	0	1	0	0	0	0	0	0	0	0	0	0	0	0	0	0	0
Halorhabdus	1	10	0	0	0	0	0	0	0	0	0	0	0	0	0	0	0	0	0	0
Halorhodospira	2	71	1	1	0	0	0	0	0	0	0	0	0	0	0	0	0	1	0	0
Halorubrum	1	16	0	0	1	1	0	0	0	0	0	0	0	0	0	0	0	0	0	1
Haloterrigena	1	19	0	0	0	0	0	0	0	1	0	0	0	0	0	0	0	0	0	0
Halothece	1	9	0	1	0	0	0	0	0	0	0	0	0	0	1	0	0	0	0	0
Halothermothrix	2	50	1	0	1	1	0	0	0	0	0	0	0	0	0	0	0	0	0	0
Halothiobacillus	2	65	1	0	0	0	0	0	0	0	0	0	0	0	0	0	0	0	0	0
Halovivax	1	12	0	0	0	0	0	0	0	0	0	0	0	0	0	0	0	0	0	0
Helicobacter	2	79	0	0	0	0	0	0	0	0	0	0	1	0	0	0	0	0	0	0
Heliobacillus	1	4	0	0	0	0	0	0	0	0	0	0	0	0	0	0	0	0	0	0
Heliobacterium	2	51	1	0	0	1	0	0	0	0	1	0	0	0	0	0	0	1	0	0
Herbaspirillum	2	172	0	1	0	0	0	1	0	0	0	0	1	1	1	0	1	0	0	0
Herminiimonas	3	89	0	1	0	0	0	0	0	0	0	0	0	0	1	0	0	0	0	0
Herpetosiphon	3	90	1	0	1	1	0	0	0	0	0	0	0	0	1	0	0	0	0	1
Hippea	2	44	1	0	0	0	0	0	0	1	0	0	0	0	0	0	0	0	0	0
Hirschia	2	102	1	1	1	1	0	0	0	0	0	0	1	0	1	0	0	1	0	1
Histophilus	2	72	1	0	1	1	0	0	1	0	0	0	1	0	0	0	0	0	0	0
Hoeflea	1	16	1	0	1	0	0	0	0	0	0	0	0	0	0	0	0	0	0	0
Hydrogenivirga	1	2	0	0	0	0	0	0	0	0	0	0	0	0	0	0	0	0	0	0
Hydrogenobacter	2	55	1	0	0	0	0	0	0	0	0	0	0	0	1	0	0	0	0	1
Hydrogenobaculum	2	44	1	0	0	0	0	0	0	0	0	0	0	0	1	0	0	0	0	0
Hydrogenophaga	1	9	0	0	0	0	0	0	0	0	0	0	0	0	0	0	0	0	0	0
Hylemonella	1	4	0	0	0	0	0	0	0	0	0	0	0	0	0	0	0	0	0	0

Genus	Cluster Number	Protein Counts	K00760	K00799	K00857	K00876	K01026	K01031	K01034	K01039	K01041	K01045	K01053	K01055	K01061	K01066	K01075	K01077	K01078	K01101
**Hyperthermus**	1	6	0	0	0	0	0	0	0	0	0	0	0	0	0	0	0	0	0	0
**Hyphomicrobium**	2	93	1	1	0	0	1	0	0	1	0	0	0	0	1	0	0	0	0	0
**Hyphomonas**	3	105	1	1	1	1	0	0	0	0	0	0	1	0	1	1	0	1	0	0
**Ideonella**	1	18	0	0	0	0	0	0	0	0	0	0	0	0	0	0	0	0	0	0
**Idiomarina**	2	86	0	1	1	1	0	0	0	0	0	0	0	0	0	0	0	1	0	0
**Ignavibacterium**	1	14	1	0	1	1	0	0	0	0	0	0	0	0	1	0	0	1	0	0
**Ignicoccus**	1	5	0	0	0	0	0	0	0	0	0	0	0	0	0	0	0	0	0	1
**Ignisphaera**	1	8	0	0	0	0	0	0	0	0	0	0	0	0	0	0	0	0	0	0
**Ilyobacter**	2	85	1	0	1	1	1	0	1	0	0	0	0	0	0	0	0	0	0	0
**Intrasporangium**	2	114	1	0	1	1	0	1	0	0	0	0	0	0	1	0	0	0	0	0
**Isoptericola**	1	18	1	0	1	1	0	0	0	0	0	0	0	0	0	0	0	0	0	0
**Isosphaera**	2	75	1	0	0	0	0	0	0	0	0	0	1	0	1	0	0	0	0	0
**Jannaschia**	3	151	1	1	1	0	0	0	0	0	0	0	0	1	0	0	1	0	0	0
**Janthinobacterium**	2	99	0	1	0	0	0	0	0	0	0	0	0	0	1	0	0	0	0	0
**Jonesia**	2	56	1	0	1	1	0	0	0	0	0	0	0	0	0	0	0	0	0	0
**Joostella**	1	3	0	0	0	0	0	0	0	0	0	0	0	0	0	0	0	0	0	0
**Kaistia**	1	2	0	0	0	0	0	0	0	0	0	0	0	0	0	0	0	0	0	0
**Kangiella**	2	84	1	1	0	0	0	0	0	0	0	0	0	0	0	0	0	0	0	0
**Ketogulonicigenium**	2	102	1	1	1	0	0	1	0	0	0	0	0	1	0	0	0	1	0	0
**Kineococcus**	1	24	1	0	1	1	0	0	0	0	0	0	1	1	1	0	0	1	0	0
**Kinetoplastibacterium**	1	2	0	0	0	0	0	0	0	0	0	0	0	0	0	0	0	0	0	0
**Kingella**	1	4	0	0	0	0	0	0	0	0	0	0	0	0	0	0	0	0	0	0
**Kitasatospora**	1	36	1	0	1	1	0	0	0	0	0	0	0	0	1	0	0	0	0	0
**Klebsiella**	3	222	1	1	1	1	1	1	1	0	0	0	1	1	1	0	0	1	0	0
**Kluyvera**	1	1	0	0	0	0	0	0	0	0	0	0	0	0	0	0	0	0	0	0
**Kocuria**	2	87	1	0	1	0	0	1	0	0	0	0	0	1	0	0	0	0	0	0
**Kordia**	1	5	0	0	0	0	0	0	0	0	0	0	0	0	0	0	0	0	0	0
**Kosmotoga**	2	61	1	0	1	1	0	0	1	0	0	0	0	0	0	0	0	1	0	1
**Kribbella**	2	99	1	0	1	1	0	0	0	0	0	0	1	1	1	0	0	0	0	0
**Krokinobacter**	2	90	0	0	1	1	0	0	0	0	0	0	0	0	1	0	0	1	0	0
**Kyrpidia**	2	113	1	0	0	1	0	0	0	0	0	0	0	0	1	0	0	0	0	1
**Kytococcus**	2	70	1	0	1	1	0	0	0	0	0	0	0	0	0	0	0	0	0	0
**Labrenzia**	1	4	0	0	0	0	0	0	0	0	0	0	0	0	0	0	0	0	0	0
**Lachnoanaerobaculum**	1	1	0	0	0	0	0	0	0	0	0	0	0	0	0	0	0	0	0	0
**Lachnospiraceae**	1	51	0	0	0	1	1	0	0	0	0	0	0	0	0	0	0	0	0	0
**Lacinutrix**	1	20	0	0	1	1	0	0	0	0	0	0	0	0	0	0	0	0	0	0
**Lactobacillus**	2	119	1	1	1	1	0	0	0	0	0	0	0	1	0	1	0	1	0	1
**Lactococcus**	2	82	1	0	1	1	0	0	0	0	0	0	0	0	0	0	0	1	0	1
**Lamprocystis**	1	2	0	0	0	0	0	0	0	0	0	0	0	0	0	0	0	0	0	0
**Laribacter**	2	80	1	1	0	0	0	0	0	0	0	0	0	1	0	0	0	0	0	0
**Lawsonia**	2	33	0	0	0	0	0	0	0	0	0	0	0	0	0	0	0	0	0	0
**Leadbetterella**	2	79	1	0	1	1	0	0	0	0	0	0	1	0	1	0	0	1	0	0
**Leeuwenhoekiella**	1	2	0	0	0	0	0	0	0	0	0	0	0	0	0	0	0	0	0	0
**Legionella**	2	117	1	1	1	1	0	0	0	0	0	0	0	0	1	0	0	0	1	0
**Leifsonia**	2	48	1	0	1	0	0	0	0	0	0	0	0	0	0	0	0	0	0	0
**Leptolyngbya**	1	10	0	1	0	0	0	0	0	0	0	0	0	0	1	0	0	0	0	0
**Leptospira**	2	76	1	1	0	0	0	0	0	0	0	0	0	0	0	0	1	0	0	0
**Leptospirillum**	1	10	1	0	0	0	0	0	0	0	0	0	0	0	1	0	0	0	0	0
**Leptothrix**	3	186	0	1	0	0	0	1	1	0	0	0	1	0	1	0	1	0	0	0
**Leptotrichia**	2	43	1	0	1	1	0	0	0	0	0	0	0	0	0	0	0	0	0	0
**Leucobacter**	1	2	0	0	0	0	0	0	0	0	0	0	0	0	0	0	0	0	0	0
**Leuconostoc**	2	64	1	0	1	1	0	0	0	0	0	0	0	0	0	0	0	0	0	1

Genus	Cluster Number	Protein Counts	K00760	K00799	K00857	K00876	K01026	K01031	K01034	K01039	K01041	K01045	K01053	K01055	K01061	K01066	K01075	K01077	K01078	K01101
Liberibacter	1	4	0	1	0	0	0	0	0	0	0	0	0	0	0	0	0	0	0	0
Limnohabitans	1	11	0	0	0	0	0	0	0	0	0	0	0	0	0	0	0	0	0	0
Listeria	2	77	1	0	1	1	1	0	0	0	0	0	0	0	0	0	0	0	0	1
Listeriaceae	1	6	0	0	0	0	0	0	0	0	0	0	0	0	0	0	0	0	0	0
Listonella	1	38	1	0	1	1	0	0	0	0	0	0	0	0	0	0	0	0	0	0
Loktanella	1	3	0	0	0	0	0	0	0	0	0	0	0	0	0	0	0	0	0	0
Lysinibacillus	2	97	1	0	1	1	0	0	0	0	1	0	0	0	0	0	0	1	0	1
Macrococcus	2	60	1	0	1	1	0	0	0	0	0	0	0	0	0	0	0	1	0	0
Magnetococcus	2	64	1	1	0	0	0	0	0	0	0	0	0	0	0	0	0	0	0	0
Magnetospirillum	2	113	0	1	0	0	0	0	0	0	0	0	0	0	1	0	0	0	0	0
Mahella	2	58	1	0	0	1	0	0	0	0	0	0	0	0	0	0	0	0	0	1
Mannheimia	2	85	1	1	1	1	0	0	0	0	0	0	1	0	0	0	0	0	0	0
Maribacter	3	87	0	0	1	1	0	0	0	0	0	0	1	0	1	0	0	1	0	0
Maricaulis	3	87	0	1	1	1	0	0	0	0	0	0	1	0	1	0	0	0	0	0
Marichromatium	1	2	0	0	0	0	0	0	0	0	0	0	0	0	0	0	0	0	0	0
Marinithermus	2	77	1	0	1	1	0	0	0	0	0	0	0	1	0	0	0	0	0	0
Marinitoga	1	15	1	0	1	1	0	0	1	0	0	0	0	0	0	0	0	0	0	0
Marinobacter	2	163	1	1	0	0	0	1	0	1	0	0	0	1	1	0	0	0	0	0
Marinomonas	3	192	1	1	1	1	1	0	0	1	0	0	1	1	1	0	1	0	0	0
Maritimibacter	1	7	0	0	0	0	0	0	0	0	0	0	0	0	0	0	0	0	0	0
Marivirga	2	96	1	0	1	1	0	0	0	0	0	0	1	0	1	0	0	0	0	0
Martelella	1	3	0	0	0	0	0	0	0	0	0	0	0	0	0	0	0	0	0	0
Marvinbryantia	1	1	0	0	0	0	0	0	0	0	0	0	0	0	0	0	0	0	0	0
Massilia	1	4	0	0	0	0	0	0	0	0	0	0	0	0	0	0	0	0	0	0
Mastigocladopsis	1	16	0	0	0	0	0	0	0	0	0	0	0	0	0	0	0	0	0	0
Megamonas	1	47	1	0	0	0	0	0	0	0	0	0	0	0	0	0	0	0	0	0
Megasphaera	1	14	1	0	0	1	1	0	1	0	0	0	0	0	0	0	0	1	0	0
Meiothermus	2	110	1	0	1	1	0	0	0	0	0	0	0	1	1	0	0	1	0	0
Melissococcus	2	46	1	0	1	1	0	0	0	0	0	0	0	0	0	0	0	0	0	1
Mesoflavibacter	1	6	0	0	0	0	0	0	0	0	0	0	0	0	0	0	0	0	0	0
Mesoplasma	1	23	1	0	1	1	0	0	0	0	0	0	0	0	0	0	0	0	0	0
Mesorhizobium	2	201	1	1	1	0	0	0	0	1	1	0	1	1	1	0	0	0	0	0
Mesotoga	1	14	1	0	1	1	0	0	1	0	0	0	0	0	0	0	0	1	0	0
Metallosphaera	1	18	0	0	0	0	0	0	0	1	0	0	0	0	1	0	0	0	0	1
Methanobacterium	1	7	0	0	0	0	0	0	0	0	0	0	0	0	0	0	0	0	0	0
Methanobrevibacter	1	9	0	0	0	0	0	0	0	0	0	0	0	0	0	0	0	0	0	0
Methanocaldococcus	1	6	0	0	0	0	0	0	0	0	0	0	0	0	0	0	0	0	0	0
Methanocella	1	15	0	0	0	0	0	0	0	0	0	0	0	0	0	0	0	0	0	0
Methanococcaceae	1	1	0	0	0	0	0	0	0	0	0	0	0	0	0	0	0	1	0	0
Methanococcoides	1	5	0	0	0	0	0	0	0	0	0	0	0	0	0	0	0	0	0	0
Methanococcus	1	9	0	0	0	0	0	0	0	0	0	0	0	0	0	0	0	1	0	0
Methanocorpusculum	1	7	0	0	0	0	0	0	0	0	0	0	0	0	0	0	0	0	0	0
Methanoculleus	1	8	0	0	0	0	0	0	0	0	0	0	0	0	1	0	0	0	0	0
Methanohalobium	1	5	0	0	0	0	0	0	0	0	0	0	0	0	0	0	0	0	0	0
Methanohalophilus	1	5	0	0	0	0	0	0	0	0	0	0	0	0	0	0	0	0	0	0
Methanolobus	1	5	0	0	0	0	0	0	0	0	0	0	0	0	0	0	0	0	0	0
Methanomethylovorans	1	5	0	0	0	0	0	0	0	0	0	0	0	0	0	0	0	0	0	0
Methanoplanus	1	7	0	0	0	0	0	0	0	0	0	0	0	0	0	0	0	0	0	0
Methanopyrus	1	4	0	0	0	0	0	0	0	0	0	0	0	0	0	0	0	0	0	0
Methanoregula	1	8	0	0	0	0	0	0	0	0	0	0	0	0	0	0	0	0	0	0
Methanosaeta	1	9	0	0	0	0	0	0	0	0	0	0	0	0	1	0	0	1	0	0
Methanosalsum	1	7	0	0	0	0	0	0	0	0	0	0	0	0	0	0	0	0	0	0

Genus	Cluster Number	Protein Counts	K00760	K00799	K00857	K00876	K01026	K01031	K01034	K01039	K01041	K01045	K01053	K01055	K01061	K01066	K01075	K01077	K01078	K01101
**Methanosarcina**	1	18	0	0	0	0	0	0	0	0	0	0	0	0	1	0	0	1	0	0
**Methanosphaera**	1	7	0	0	0	0	0	0	0	0	0	0	0	0	0	0	0	0	0	0
**Methanosphaerula**	1	8	0	0	0	0	0	0	0	0	0	0	0	0	0	0	0	0	0	0
**Methanospirillum**	1	6	0	0	0	0	0	0	0	0	0	0	0	0	0	0	0	0	0	0
**Methanothermobacter**	1	6	0	0	0	0	0	0	0	0	0	0	0	0	0	0	0	0	0	0
**Methanothermococcus**	1	5	0	0	0	0	0	0	0	0	0	0	0	0	0	0	0	0	0	0
**Methanothermus**	1	5	0	0	0	0	0	0	0	0	0	0	0	0	0	0	0	0	0	0
**Methanotorris**	1	5	0	0	0	0	0	0	0	0	0	0	0	0	0	0	0	0	0	0
**Methylacidiphilum**	2	48	1	0	0	0	0	0	0	0	0	0	0	0	1	0	0	0	0	0
**Methylarcula**	1	2	0	0	0	0	0	0	0	0	0	0	0	0	0	0	0	0	0	0
**Methylibium**	1	38	0	1	0	0	0	0	0	0	0	0	0	0	1	0	0	0	0	0
**Methylobacillus**	2	61	1	1	0	0	0	0	0	0	0	0	0	0	1	1	0	1	0	0
**Methylobacterium**	3	236	1	1	0	1	1	0	1	0	0	0	1	1	1	0	0	1	0	0
**Methylocella**	3	117	0	1	0	0	0	0	1	0	0	0	0	0	1	0	0	0	0	0
**Methylococcus**	2	80	1	1	0	0	0	0	0	0	0	0	0	0	0	0	0	1	0	0
**Methylocystis**	1	21	0	1	0	0	1	0	1	0	0	0	0	0	1	0	0	0	0	0
**Methyloferula**	1	2	0	0	0	0	0	0	0	0	0	0	0	0	0	0	0	0	0	0
**Methylomicrobium**	1	12	1	0	0	0	0	0	0	0	0	0	0	0	0	0	0	1	0	1
**Methylomonas**	1	15	1	1	0	0	0	0	0	0	0	0	0	0	0	0	0	1	0	1
**Methylophaga**	1	7	1	0	0	0	0	0	0	0	0	0	0	0	1	0	0	0	0	0
**Methylophilus**	1	2	0	0	0	0	0	0	0	0	0	0	0	0	0	0	0	0	0	0
**Methylosinus**	1	2	0	0	0	0	0	0	0	0	0	0	0	0	0	0	0	0	0	0
**Methylotenera**	2	72	1	1	0	0	0	0	0	0	0	0	0	0	1	0	0	1	0	0
**Methyloversatilis**	1	5	0	0	0	0	0	0	0	0	0	0	0	0	0	0	0	0	0	0
**Methylovorus**	2	66	1	1	0	0	0	0	0	0	0	0	0	0	1	1	0	1	0	0
**Micavibrio**	1	9	0	1	0	0	0	0	0	0	0	0	0	0	1	0	0	0	0	0
**Microbacterium**	2	115	1	0	1	0	0	1	0	0	0	0	1	1	0	0	0	0	0	0
**Microchaete**	1	23	0	0	0	0	0	0	0	0	0	0	0	0	0	0	0	0	0	0
**Micrococcus**	2	72	1	0	1	1	0	0	0	0	0	0	0	0	1	0	0	0	0	0
**Microcoleus**	1	17	0	1	0	0	0	0	0	0	0	0	1	0	1	0	0	0	0	0
**Microcystis**	2	67	0	1	0	1	0	0	0	0	0	0	0	0	1	0	0	0	0	0
**Microlunatus**	1	25	1	0	0	1	0	0	0	0	0	0	1	0	1	0	0	0	0	0
**Micromonospora**	2	115	1	0	1	1	0	0	0	1	0	0	0	0	1	0	0	0	0	0
**Microvirga**	1	6	0	0	0	0	0	0	0	0	0	0	0	0	0	0	0	0	0	0
**Mobiluncus**	1	41	1	0	0	0	0	0	0	0	0	0	0	0	0	0	0	0	0	0
**Modestobacter**	1	43	1	0	1	0	0	0	0	0	0	0	0	1	1	0	0	1	0	0
**Moorea**	1	2	0	0	0	0	0	0	0	0	0	0	0	0	0	0	0	0	0	0
**Moorella**	2	69	1	0	0	0	1	0	0	0	0	0	0	0	0	0	0	0	0	0
**Moraxella**	2	66	1	1	0	0	0	0	0	0	0	0	0	0	0	0	0	0	0	0
**Morganella**	1	29	1	1	1	1	0	0	1	0	0	0	0	0	0	0	0	1	0	0
**Moritella**	1	2	0	0	0	0	0	0	0	0	0	0	0	0	0	0	0	0	0	0
**Muricauda**	1	23	0	0	1	1	0	0	0	0	0	0	1	0	1	0	0	0	0	0
**Mycobacterium**	3	306	1	0	0	0	0	1	0	1	1	0	1	1	1	1	0	1	1	0
**Mycoplasma**	1	53	1	0	1	1	0	0	0	0	0	0	0	0	0	0	0	0	0	0
**Myxococcus**	2	110	1	1	1	1	0	0	0	1	0	1	0	1	1	0	0	0	0	0
**Nakamurella**	2	115	1	0	1	0	0	1	0	0	0	0	0	0	1	0	0	0	0	0
**napthalene**	1	2	0	0	0	0	0	0	0	0	0	0	0	0	0	0	0	0	0	0
**Natranaerobius**	2	55	1	0	1	0	1	0	1	0	0	0	0	0	0	0	0	0	0	0
**Natrialba**	1	16	0	0	0	1	0	0	0	1	0	0	0	0	0	0	0	0	0	0
**Natrinema**	1	16	0	0	0	1	1	0	0	1	0	0	0	0	0	0	0	0	0	0
**Natronobacterium**	1	11	0	0	0	1	0	0	0	0	0	0	0	0	0	0	0	0	0	0
**Natronococcus**	1	21	0	0	0	1	0	0	0	0	0	0	0	0	0	0	0	1	0	0

Genus	Cluster Number	Protein Counts	K00760	K00799	K00857	K00876	K01026	K01031	K01034	K01039	K01041	K01045	K01053	K01055	K01061	K01066	K01075	K01077	K01078	K01101
Natronomonas	1	20	0	0	0	0	0	0	0	0	0	0	0	0	0	1	0	0	0	0
Nautilia	2	48	0	0	0	0	0	0	0	0	0	0	0	0	0	0	0	0	0	0
Neisseria	2	67	1	0	0	0	0	0	0	0	0	0	0	0	0	0	0	0	0	0
Neisseriaceae	1	1	0	0	0	0	0	0	0	0	0	0	0	0	0	0	0	0	0	0
Neorickettsia	1	31	0	0	0	0	0	0	0	0	0	0	0	0	0	0	0	0	0	0
Neptuniibacter	1	2	0	0	0	0	0	0	0	0	0	0	0	0	0	0	0	0	0	0
Niastella	1	21	1	0	1	0	0	0	0	0	0	0	1	0	1	0	0	1	0	0
Nitratifractor	2	54	0	0	0	0	0	0	0	0	0	0	0	0	0	0	0	0	0	1
Nitratireductor	1	19	0	0	0	0	0	0	0	0	0	0	0	0	0	0	0	0	0	0
Nitratiruptor	2	40	0	0	0	0	0	0	0	0	0	0	0	0	0	0	0	0	0	0
Nitrobacter	2	99	0	1	0	0	0	0	1	0	1	0	0	1	0	0	0	0	0	0
Nitrococcus	1	2	0	0	0	0	0	0	0	0	0	0	0	0	0	0	0	0	0	0
Nitrosococcus	2	94	1	0	0	0	0	0	0	0	0	0	1	0	0	0	0	0	0	0
Nitrosomonas	2	72	1	1	0	0	0	0	0	0	0	0	0	0	0	0	0	0	0	0
Nitrosopumilaceae	1	7	0	0	0	0	0	0	0	0	0	0	0	0	0	0	0	0	0	0
Nitrosopumilus	1	11	0	0	0	0	0	0	0	0	0	0	0	0	1	0	0	0	0	0
Nitrosospira	2	74	1	1	0	0	0	0	0	0	0	0	0	0	0	0	0	0	0	0
Nocardia	2	185	1	0	0	0	0	0	0	1	1	0	1	1	1	0	0	1	0	0
Nocardioidaceae	1	2	0	0	0	0	0	0	0	0	0	0	0	0	0	0	0	0	0	0
Nocardioides	2	157	1	0	1	1	0	1	0	1	1	0	0	0	0	0	0	0	0	0
Nocardiopsis	2	102	1	0	1	0	0	0	0	1	0	0	0	1	0	0	0	0	0	0
Nodularia	1	22	0	1	0	0	0	0	0	0	0	0	0	0	0	0	0	0	0	0
Nonlabens	1	19	0	0	1	1	0	0	0	0	0	0	0	0	1	0	0	0	0	0
Nostoc	2	111	0	1	0	0	0	0	0	0	0	0	1	0	1	0	0	1	0	0
Nostocaceae	1	2	0	0	0	0	0	0	0	0	0	0	0	0	0	0	0	0	0	0
Novosphingobium	2	169	0	1	1	0	0	0	0	1	0	0	1	1	1	0	1	0	0	0
Oceanibaculum	1	3	0	0	0	0	0	0	0	0	0	0	0	0	0	0	0	0	0	0
Oceanibulbus	1	12	1	0	0	0	0	0	0	0	0	0	0	0	0	0	0	0	0	0
Oceanicola	1	9	0	0	0	0	0	0	0	0	0	0	0	0	0	0	0	0	0	0
Oceanimonas	1	33	1	1	0	0	0	0	0	1	0	0	0	1	1	0	0	0	0	0
Oceaniovalibus	1	2	0	0	0	0	0	0	0	0	0	0	0	0	0	0	0	0	0	0
Oceanithermus	2	72	1	0	1	1	0	0	0	0	0	0	0	0	0	0	0	0	0	0
Oceanobacillus	2	91	1	0	1	1	0	0	0	0	0	0	0	0	0	0	0	1	0	0
Oceanospirillum	1	2	0	0	0	0	0	0	0	0	0	0	0	0	0	0	0	0	0	0
Ochrobactrum	2	133	1	1	0	0	0	1	0	0	0	0	1	1	1	0	0	0	0	0
Octadecabacter	1	29	1	1	1	0	0	1	0	0	0	0	0	1	0	0	0	0	0	0
Oenococcus	2	41	1	0	1	1	0	0	0	0	0	0	0	0	0	0	0	0	0	0
Oleomonas	1	2	0	0	0	0	0	0	0	0	0	0	0	0	0	0	0	0	0	0
Oligotropha	3	87	0	1	0	0	0	0	0	1	0	0	0	1	1	0	0	0	0	0
Olsenella	2	40	1	0	1	0	0	0	0	0	0	0	0	0	0	0	0	0	0	0
Opitutus	2	67	1	0	0	1	0	0	0	0	0	0	1	0	0	0	0	0	0	0
Orientia	1	21	0	1	0	0	0	0	0	0	0	0	0	0	0	0	0	0	0	0
Ornithobacterium	1	11	1	0	1	1	0	0	0	0	0	0	0	0	0	0	0	0	0	0
Oscillatoria	1	16	0	1	0	0	0	0	0	0	0	0	1	0	1	0	0	0	0	0
Oscillatoriales	1	2	0	0	0	0	0	0	0	0	0	0	0	0	0	0	0	0	0	0
Oscillibacter	1	20	1	0	1	1	1	0	1	1	0	0	0	0	0	0	0	0	0	0
Owenweeksia	1	15	0	0	1	0	0	0	0	0	0	0	0	0	0	0	0	1	0	0
Oxalobacteraceae	1	4	0	0	0	0	0	0	0	0	0	0	0	0	0	0	0	0	0	0
Paenibacillus	2	152	1	0	1	1	0	0	1	0	0	0	0	1	1	0	0	1	0	1
Paludibacter	2	67	1	0	1	1	0	0	0	0	0	0	0	0	0	0	0	0	0	0
Pandoraea	1	18	0	0	0	0	0	0	0	0	0	0	0	0	0	0	0	0	0	0
Pannonibacter	1	3	0	0	0	0	0	0	0	0	0	0	0	0	0	0	0	0	0	0

Genus	Cluster Number	Protein Counts	K00760	K00799	K00857	K00876	K01026	K01031	K01034	K01039	K01041	K01045	K01053	K01055	K01061	K01066	K01075	K01077	K01078	K01101
Pantholops	1	23	0	1	0	0	0	0	0	0	0	0	0	0	1	0	0	0	0	0
Pantoea	2	181	1	1	1	1	0	1	0	0	0	0	1	0	1	0	0	1	0	0
Parabacteroides	2	64	1	0	1	1	0	0	0	0	0	0	0	0	0	0	0	1	0	0
Parachlamydia	1	11	0	0	0	1	0	0	0	0	0	0	1	0	0	0	0	0	0	0
Paracoccus	2	169	0	1	1	0	0	1	0	1	0	0	0	1	1	0	0	0	0	0
Parvibaculum	3	117	1	1	0	0	0	0	0	0	0	0	1	0	1	0	0	0	0	0
Parvularcula	2	72	1	1	0	0	0	0	0	0	0	0	0	0	0	0	0	1	0	0
Pasteurella	2	85	1	0	1	1	0	0	0	0	0	0	1	0	0	0	0	0	0	0
Pasteurellaceae	1	2	0	0	0	0	0	0	0	0	0	0	0	0	0	0	0	0	0	0
Pectobacterium	1	44	1	1	1	1	0	0	1	0	0	0	1	0	1	1	0	1	0	0
Pediococcus	1	43	1	0	1	1	0	0	0	0	0	0	0	0	0	0	0	0	0	0
Pedobacter	2	88	1	0	1	1	0	0	0	0	0	0	1	0	1	0	0	1	0	0
Pelagibaca	1	10	0	0	0	0	0	0	0	0	0	0	0	0	0	0	0	0	0	0
Pelagibacterium	1	39	0	1	1	0	0	0	0	1	0	0	0	1	0	0	1	1	0	0
Pelobacter	2	85	1	0	0	0	0	0	0	0	0	0	0	0	0	0	0	1	0	0
Pelodictyon	2	62	1	0	0	0	0	0	0	0	0	0	0	0	0	0	0	1	1	0
Pelosinus	1	1	0	0	0	0	0	0	0	0	0	0	0	0	0	0	0	0	0	0
Pelotomaculum	2	60	1	0	0	0	1	0	0	1	0	0	0	0	0	0	0	0	0	0
Peptostreptococcus	1	1	0	0	0	0	0	0	0	0	0	0	0	0	0	0	0	0	0	0
Persephonella	2	55	1	0	0	1	0	0	0	0	0	0	0	0	0	0	0	0	0	1
Petrotoga	2	72	1	0	1	1	0	0	1	0	0	0	0	0	0	0	0	1	0	1
Phaeobacter	2	55	1	1	1	0	0	0	0	0	0	0	0	1	0	0	0	0	0	0
Phenylobacterium	3	103	1	1	0	0	0	0	0	0	0	0	1	0	1	0	0	0	0	0
Photobacterium	2	119	1	1	1	1	0	0	0	0	0	0	0	0	0	0	0	1	0	0
Photorhabdus	2	149	1	1	1	1	0	0	1	1	0	0	0	0	0	0	0	1	0	0
Phycisphaera	1	9	1	0	0	0	0	0	0	0	0	0	1	0	0	0	0	1	0	0
Phyllobacterium	1	6	0	0	0	0	0	0	0	0	0	0	0	0	0	0	0	0	0	0
Picrophilus	1	15	0	0	0	0	0	0	0	0	0	0	0	0	1	1	0	0	0	0
Pirellula	2	85	1	0	0	0	0	0	0	0	0	0	1	0	0	0	0	0	0	0
Planctomyces	2	93	1	0	0	0	0	0	0	0	0	0	1	0	1	0	0	1	0	0
Plautia	1	17	0	0	0	1	0	0	0	0	0	0	0	0	0	0	0	0	0	0
Plesiomonas	1	4	0	0	0	0	0	0	0	0	0	0	0	0	0	0	0	0	0	0
Pleurocapsa	1	23	0	1	0	0	0	0	0	0	0	0	0	0	1	0	0	0	0	0
Polaribacter	2	74	0	0	1	1	0	0	0	0	0	0	0	0	1	0	0	0	0	0
Polaromonas	3	238	0	1	0	0	0	1	0	0	0	0	1	1	1	0	1	0	0	0
Polymorphum	3	173	1	1	1	0	0	0	0	1	0	0	0	1	1	0	0	0	0	0
Polynucleobacter	1	19	0	1	0	0	1	1	0	0	0	0	0	0	1	0	0	1	0	0
Ponticaulis	1	1	0	0	0	0	0	0	0	0	0	0	0	0	0	0	0	0	0	0
Porphyromonas	2	63	1	0	1	1	0	0	1	0	0	0	0	0	0	0	0	1	0	0
Prevotella	1	65	1	0	1	1	0	0	0	0	0	0	0	0	0	0	0	0	0	0
Prochlorococcus	2	68	0	1	0	1	0	0	0	0	0	0	0	0	1	0	0	1	0	0
Propionibacterium	1	62	1	0	1	1	0	0	0	0	1	0	0	1	0	1	0	0	0	0
Prosthecochloris	1	16	1	0	0	1	0	0	0	0	0	0	0	0	0	0	0	1	1	0
Proteobacteria	1	19	0	1	0	0	0	0	0	0	0	0	0	0	0	0	0	0	0	0
Proteus	2	113	1	1	1	1	0	0	0	0	0	0	0	0	0	0	0	1	0	0
Providencia	1	38	1	1	1	1	0	0	0	0	0	0	0	0	0	0	0	1	0	0
Pseudaminobacter	1	10	1	0	0	0	0	0	0	0	0	0	0	0	0	0	0	0	0	0
Pseudanabaena	1	11	0	1	0	1	0	0	0	0	0	0	0	0	1	0	0	0	0	0
Pseudoalteromonas	2	188	0	1	1	1	0	0	0	1	0	0	1	0	1	0	0	1	0	0
Pseudochrobactrum	1	7	0	1	0	0	0	0	0	0	0	0	0	0	0	0	0	0	0	0
Pseudogulbenkiania	1	40	1	1	0	1	0	1	0	0	0	0	0	1	1	0	0	0	0	0
pseudomallei	3	131	1	1	0	0	0	1	0	0	0	0	1	1	1	1	0	0	0	0

Genus	Cluster Number	Protein Counts	K00760	K00799	K00857	K00876	K01026	K01031	K01034	K01039	K01041	K01045	K01053	K01055	K01061	K01066	K01075	K01077	K01078	K01101
Pseudomonas	3	381	1	1	1	0	1	1	0	1	0	0	1	1	1	1	1	1	1	0
Pseudonocardia	3	180	1	0	0	0	0	1	0	1	0	0	1	1	1	0	0	0	0	0
Pseudorhodobacter	1	4	0	0	0	0	0	0	0	0	0	0	0	0	0	0	0	0	0	0
Pseudovibrio	1	33	1	1	1	0	1	0	0	1	0	0	0	0	0	0	1	1	0	0
Pseudoxanthomonas	2	124	1	1	1	0	0	0	0	0	0	0	1	1	1	0	0	1	1	0
Psychrobacter	2	124	1	1	0	0	0	1	0	0	0	0	0	1	0	0	0	0	0	0
Psychroflexus	1	22	1	0	1	1	0	0	0	0	0	0	1	0	1	0	0	0	0	0
Psychromonas	2	110	1	1	1	1	0	0	0	0	0	0	0	0	0	0	0	1	0	0
Pusillimonas	3	111	0	1	0	0	0	1	0	0	0	0	0	0	1	0	0	0	0	0
Pyrobaculum	1	15	0	0	1	0	0	0	0	0	0	0	0	0	0	0	0	0	0	0
Pyrococcus	1	10	0	0	0	0	0	0	0	0	0	0	0	0	0	0	0	1	0	1
Pyrolobus	1	4	0	0	0	0	0	0	0	0	0	0	0	0	0	0	0	0	0	0
Rahnella	2	143	1	1	1	1	0	0	0	0	0	0	0	1	1	0	0	1	0	0
Ralstonia	3	332	1	1	0	0	1	1	1	1	1	0	1	1	1	1	1	1	1	0
Ramlibacter	1	35	0	1	0	0	0	1	0	0	0	0	1	0	1	0	1	0	0	0
Raoultella	2	139	1	1	1	1	0	1	0	0	0	0	1	1	1	0	0	1	0	0
Raphidiopsis	1	5	0	0	0	0	0	0	0	0	0	0	0	0	0	0	0	0	0	0
Reinekea	1	2	0	0	0	0	0	0	0	0	0	0	0	0	0	0	0	0	0	0
Renibacterium	2	67	1	0	1	0	0	0	0	0	0	0	0	0	1	0	0	0	0	0
Rhizobiaceae	1	3	0	0	0	0	0	0	0	0	0	0	0	0	0	0	0	0	0	0
Rhizobiales	1	6	0	0	0	0	0	0	0	0	0	0	0	0	0	0	0	0	0	0
Rhizobium	3	257	1	1	1	0	1	1	0	1	1	0	1	1	1	1	1	1	1	0
Rhizobium/Agrobacterium	2	87	1	1	0	0	0	1	0	0	0	0	1	0	0	0	0	0	1	0
Rhodanobacter	1	21	1	1	1	0	0	0	0	1	0	0	0	0	1	0	0	1	0	0
Rhodobacter	3	175	1	1	1	0	0	0	0	1	0	0	0	1	0	1	0	1	0	0
Rhodobacteraceae	1	21	1	0	0	0	0	0	0	0	0	0	0	0	0	0	0	0	0	0
Rhodobacterales	1	34	1	1	0	0	0	0	0	0	0	0	0	0	0	0	0	0	0	0
Rhodococcus	2	292	1	0	0	0	0	0	0	1	1	0	1	0	1	1	1	0	0	0
Rhodocyclaceae	1	7	0	0	0	0	0	0	0	0	0	0	0	0	0	0	0	0	0	0
Rhodoferax	3	144	0	1	1	0	1	1	0	0	0	0	0	0	1	0	1	0	0	0
Rhodomicrobium	2	103	0	1	0	0	0	0	0	0	0	0	0	0	0	0	0	1	0	0
Rhodopirellula	2	87	1	0	1	0	0	0	0	0	0	0	1	0	1	1	1	1	0	0
Rhodopseudomonas	3	246	0	1	0	0	0	1	1	1	1	0	1	1	1	1	1	1	0	0
Rhodospirillum	2	121	0	1	1	0	0	0	1	0	0	0	1	0	1	0	0	1	1	0
Rhodothermus	2	76	1	0	1	1	0	0	0	0	0	0	0	0	1	0	0	1	0	0
Rhodovulum	1	4	0	0	0	0	0	0	0	0	0	0	0	0	0	0	0	0	0	0
Richelia	1	2	0	0	0	0	0	0	0	0	0	0	0	0	0	0	0	0	0	0
Rickettsia	2	43	0	1	0	0	0	0	0	0	0	0	0	0	0	0	0	0	0	0
Rickettsiaceae	1	5	0	0	0	0	0	0	0	0	0	0	0	0	0	0	0	0	0	0
Riemerella	2	61	1	0	1	1	0	0	0	0	0	0	0	0	0	0	0	1	0	0
Rivularia	1	18	0	1	0	0	0	0	0	0	0	0	1	0	1	0	0	1	0	0
Robiginitalea	2	83	0	0	1	1	0	0	0	0	0	0	1	0	1	0	0	0	0	0
Roseburia	2	58	1	0	1	1	0	0	0	0	0	0	0	0	0	1	0	0	0	0
Roseibium	1	3	0	0	0	0	0	0	0	0	0	0	0	0	0	0	0	0	0	0
Roseiflexus	3	100	0	0	1	0	0	0	0	0	0	0	1	0	1	0	0	1	0	1
Roseobacter	2	149	1	1	1	0	1	0	0	0	0	0	1	1	0	0	0	0	0	0
Roseomonas	1	2	0	0	0	0	0	0	0	0	0	0	0	0	0	0	0	0	0	0
Roseovarius	1	15	0	0	0	0	0	0	0	0	0	0	0	0	0	0	0	0	0	0
Rothia	1	44	1	0	0	0	0	0	0	0	0	0	0	0	0	0	0	0	0	0
Rubrivivax	1	24	0	1	0	1	0	0	1	0	0	0	0	0	1	0	0	1	0	0
Rubrobacter	3	117	1	1	1	0	0	0	0	1	0	0	0	1	0	0	0	1	0	0
Ruegeria	2	189	1	1	1	0	0	0	0	0	0	0	1	1	0	1	1	0	0	0

Genus	Cluster Number	Protein Counts	K00760	K00799	K00857	K00876	K01026	K01031	K01034	K01039	K01041	K01045	K01053	K01055	K01061	K01066	K01075	K01077	K01078	K01101
Ruminococcaceae	1	2	0	0	0	0	0	0	0	0	0	0	0	0	0	0	0	0	0	0
Ruminococcus	2	101	1	0	1	1	1	0	0	0	0	0	0	0	0	0	0	0	0	0
Runella	1	26	1	0	1	1	0	0	0	0	0	0	1	0	1	0	0	1	0	0
Saccharomonospora	2	132	1	0	1	0	0	0	0	1	0	0	0	1	1	0	0	0	0	0
Saccharophagus	2	81	1	1	0	0	0	0	0	0	0	0	0	0	1	0	0	1	0	0
Saccharopolyspora	3	194	1	1	0	1	0	0	0	1	1	0	1	1	1	1	0	0	0	0
Saccharothrix	1	32	1	0	0	1	0	0	0	1	0	0	0	1	1	0	0	0	0	0
Sagittula	1	10	0	0	0	0	0	0	0	0	0	0	0	0	0	0	0	0	0	0
Salinibacter	2	77	1	0	1	1	0	0	0	0	0	0	0	0	1	0	0	1	1	0
Salinimonas	1	1	0	0	0	0	0	0	0	0	0	0	0	0	0	0	0	0	0	0
Salinispora	3	127	1	0	1	1	0	0	0	0	1	0	1	0	1	0	0	1	0	0
Salinivibrio	1	2	0	0	0	0	0	0	0	0	0	0	0	0	0	0	0	0	0	0
Salmonella	2	151	1	1	1	1	0	0	0	0	0	0	0	0	1	1	0	0	0	0
Sanguibacter	2	73	1	0	1	1	0	0	0	0	0	0	0	0	0	0	0	1	0	0
Saprospira	1	13	1	0	1	1	0	0	0	0	0	0	0	0	0	0	0	0	0	0
Scardovia	1	1	0	0	0	0	0	0	0	0	0	0	0	0	0	0	0	0	0	0
Scytonema	1	10	0	0	0	0	0	0	0	0	0	0	0	0	0	0	0	0	0	0
Sebaldella	2	69	1	0	1	1	0	0	0	0	0	0	0	0	0	0	0	1	0	0
Segniliparus	2	85	1	0	0	0	0	0	0	0	0	0	0	0	1	0	0	0	0	0
Selenomonas	2	57	1	0	0	1	0	0	0	0	0	0	0	0	0	0	0	0	0	0
Serinicoccus	1	1	0	0	0	0	0	0	0	0	0	0	0	0	0	0	0	0	0	0
Serratia	2	186	1	1	1	1	0	1	0	0	0	0	1	1	1	1	0	1	0	0
Shewanella	3	214	1	1	1	1	1	1	0	1	0	0	0	0	1	1	0	1	0	0
Shigella	2	172	1	1	1	1	0	0	1	0	0	0	0	0	1	1	0	1	0	0
Shinella	1	2	0	0	0	0	0	0	0	0	0	0	0	0	0	0	0	0	0	0
Sideroxydans	2	81	1	1	0	0	0	0	0	0	0	0	0	0	0	0	0	0	0	0
Silicibacter	2	105	1	1	0	0	0	0	0	0	0	0	0	0	0	0	0	0	0	0
Simiduia	1	18	1	1	0	0	0	0	0	0	0	0	1	0	1	0	0	1	0	0
Simkania	1	6	1	0	1	1	0	0	0	0	0	0	0	0	0	0	0	0	0	0
Simonsiella	1	2	0	0	0	0	0	0	0	0	0	0	0	0	0	0	0	0	0	0
Singulisphaera	1	18	1	0	0	0	0	0	0	0	0	0	1	0	1	0	0	1	0	0
Sinorhizobium	3	207	1	1	1	0	0	1	0	1	1	0	1	1	1	0	0	1	0	0
Slackia	2	46	1	0	0	0	0	0	0	0	0	0	0	0	0	0	0	0	0	0
Smaragdicoccus	1	3	0	0	0	0	0	0	0	0	0	0	0	0	0	0	0	0	0	0
Sodalis	2	71	1	0	1	1	0	0	0	0	0	0	0	0	0	0	0	0	0	0
Solibacillus	1	23	1	0	1	1	0	0	0	0	0	0	0	0	0	0	0	1	0	0
Solitalea	1	13	1	0	1	1	0	0	0	0	0	0	0	0	1	0	0	0	0	0
Sorangium	3	115	1	1	0	0	0	0	0	0	0	0	1	0	1	0	1	0	1	0
Sphaerobacter	2	100	1	0	0	0	0	0	0	1	0	0	0	0	1	0	0	0	0	1
Sphaerochaeta	1	42	1	0	1	1	0	0	0	0	0	0	0	0	0	0	0	1	0	0
Sphingobacterium	3	85	1	0	1	1	0	0	0	0	0	0	1	0	1	0	0	1	0	0
Sphingobium	1	179	0	1	1	0	0	1	0	0	0	0	1	1	1	0	1	1	0	0
Sphingomonadaceae	1	15	0	0	0	0	0	0	0	0	0	0	0	0	0	0	0	0	0	0
Sphingomonas	2	220	0	1	1	0	0	1	0	1	0	0	0	1	1	1	1	0	0	0
Sphingopyxis	3	99	0	1	1	0	0	0	0	0	0	0	1	0	1	0	0	0	0	0
Spirochaeta	2	103	1	0	1	1	0	0	0	0	0	0	0	0	0	0	0	1	0	1
Spiroplasma	1	6	1	0	1	1	0	0	0	0	0	0	0	0	0	0	0	0	0	0
Spirosoma	2	107	1	0	1	1	0	0	0	0	0	0	1	0	1	0	0	0	0	0
Stackebrandtia	2	110	1	0	0	1	0	0	0	1	0	0	1	1	1	0	0	0	0	0
Stanieria	1	17	0	1	0	0	0	0	0	0	0	0	1	0	1	0	0	0	0	0
Staphylococcus	2	108	1	1	1	1	1	0	0	0	0	0	0	0	0	0	0	1	0	1
Staphylothermus	1	12	0	0	0	0	0	0	0	0	0	0	0	0	0	0	0	0	0	1

Genus	Cluster Number	Protein Counts	K00760	K00799	K00857	K00876	K01026	K01031	K01034	K01039	K01041	K01045	K01053	K01055	K01061	K01066	K01075	K01077	K01078	K01101
**Stappia**	1	2	0	0	0	0	0	0	0	0	0	0	0	0	0	0	0	0	0	0
**Starkeya**	3	134	1	1	0	0	0	0	0	1	0	0	1	1	1	0	0	0	0	0
**Stenotrophomonas**	3	104	1	1	1	0	0	0	0	0	0	0	0	0	1	0	0	1	1	0
**Stigmatella**	3	129	1	1	1	1	0	0	0	1	0	0	0	0	1	0	0	0	0	0
**Streptobacillus**	1	38	1	0	1	1	0	0	0	0	0	0	0	0	0	0	0	0	0	0
**Streptococcus**	2	121	1	1	1	1	1	0	1	0	0	0	0	0	0	1	0	1	1	1
**Streptomyces**	2	253	1	0	1	1	0	1	0	1	1	0	1	0	1	0	0	1	0	0
**Streptosporangium**	3	148	1	0	1	0	0	0	0	1	1	0	1	1	1	0	0	0	0	0
**Strigomonas**	1	2	0	0	0	0	0	0	0	0	0	0	0	0	0	0	0	0	0	0
**Sulfitobacter**	1	10	0	0	0	0	0	0	0	0	0	0	0	0	0	0	0	0	0	0
**Sulfobacillus**	1	30	1	0	1	0	0	0	0	1	0	0	0	1	1	0	0	0	0	0
**Sulfolobus**	1	23	0	0	0	0	0	0	0	1	0	0	0	0	1	0	0	0	0	1
**Sulfuricurvum**	2	67	0	0	0	0	0	0	0	0	0	0	0	0	0	0	0	0	0	0
**Sulfurihydrogenibium**	2	55	1	0	0	0	0	0	0	0	0	0	0	0	0	0	0	0	0	0
**Sulfurimonas**	2	60	0	0	0	0	0	0	0	0	0	0	0	0	0	0	0	0	0	0
**Sulfurospirillum**	1	48	0	0	0	0	0	0	0	0	0	0	0	0	0	0	0	0	0	0
**Sulfurovum**	2	48	0	0	0	0	0	0	0	0	0	0	0	0	0	0	0	1	0	0
**Symbiobacterium**	2	68	1	0	1	1	0	0	0	0	0	0	0	1	0	0	0	0	0	0
**Synechococcus**	1	34	0	1	0	1	0	0	0	0	0	0	1	0	1	1	0	1	0	0
**Synechocystis**	1	40	0	1	0	0	0	0	0	0	0	0	0	0	1	0	0	0	0	0
**Synergistetes**	1	27	1	0	0	1	0	0	0	0	0	0	0	0	0	0	0	0	0	0
**Syntrophobacter**	2	101	1	0	0	0	1	0	0	1	1	0	0	1	0	0	0	0	0	0
**Syntrophobotulus**	2	71	1	0	0	0	0	0	0	0	0	0	0	1	0	0	0	1	0	0
**Syntrophomonas**	2	58	1	0	1	1	0	0	0	0	1	0	0	0	0	0	0	0	0	0
**Syntrophothermus**	2	60	1	0	0	0	0	0	0	1	0	0	0	0	0	0	0	0	0	0
**Syntrophus**	2	66	1	0	0	0	1	0	0	0	0	0	0	0	0	0	0	0	0	0
**Tannerella**	1	8	1	0	0	1	0	0	0	0	0	0	0	0	0	0	0	1	0	0
**Taylorella**	1	43	0	1	0	0	0	0	0	0	0	0	0	0	0	0	0	1	0	0
**Tepidanaerobacter**	2	55	1	0	1	1	1	0	0	0	0	0	0	0	0	0	0	1	0	0
**Teredinibacter**	2	109	1	1	0	0	0	0	0	0	0	0	1	0	0	0	1	1	0	0
**Terrabacter**	1	1	0	0	0	0	0	0	0	0	0	0	0	0	0	0	0	0	0	0
**Terriglobus**	2	83	1	0	1	1	0	0	0	0	0	0	1	0	1	0	0	0	0	0
**Tetragenococcus**	1	12	1	0	1	1	0	0	0	0	0	0	0	0	0	0	0	0	0	1
**Thalassiobium**	1	11	0	0	0	0	0	0	0	0	0	0	0	0	0	0	0	0	0	0
**Thalassobacter**	1	9	0	0	0	0	0	0	0	0	0	0	0	0	0	0	0	0	0	0
**Thalassolituus**	1	12	1	1	0	0	0	0	0	0	0	0	0	0	0	0	0	1	0	0
**Thalassospira**	1	2	0	0	0	0	0	0	0	0	0	0	0	0	0	0	0	0	0	0
**Thauera**	1	111	1	1	0	0	1	0	0	0	0	0	0	0	1	0	1	0	0	0
**Thermacetogenium**	1	7	1	0	0	0	0	0	0	0	0	0	0	0	0	0	0	0	0	0
**Thermaerobacter**	2	79	1	0	1	1	0	0	0	0	0	0	0	0	0	0	0	0	0	0
**Thermanaerovibrio**	2	58	1	0	0	1	0	0	0	0	0	0	0	0	0	0	0	1	0	0
**Thermincola**	2	62	1	0	0	0	0	0	0	0	0	0	0	0	0	0	0	0	0	0
**Thermoanaerobacter**	2	76	1	0	1	1	0	0	1	0	0	0	0	0	0	0	0	0	0	0
**Thermoanaerobacterium**	2	64	1	0	1	1	0	0	0	0	0	0	0	0	0	0	0	0	0	0
**Thermobacillus**	1	11	1	0	0	0	0	0	0	0	0	0	0	0	0	0	0	0	0	0
**Thermobaculum**	2	62	1	0	0	0	0	0	0	0	0	0	1	0	1	0	0	1	0	1
**Thermobifida**	2	72	1	0	0	0	0	0	0	0	0	0	0	1	0	0	0	0	0	0
**Thermobispora**	2	109	1	0	0	0	0	0	0	1	0	0	0	0	1	0	0	0	0	0
**Thermococcus**	1	13	0	0	1	0	0	0	0	0	0	0	0	0	0	0	0	0	0	1
**Thermocrinis**	2	60	1	0	0	0	0	0	0	0	0	0	0	0	1	0	0	0	0	1
**Thermodesulfatator**	1	10	1	0	0	0	0	0	0	0	0	0	0	0	0	0	0	1	0	0
**Thermodesulfobacterium**	1	9	1	0	0	0	0	0	0	0	0	0	0	0	0	0	0	0	0	0

Genus	Cluster Number	Protein Counts	K00760	K00799	K00857	K00876	K01026	K01031	K01034	K01039	K01041	K01045	K01053	K01055	K01061	K01066	K01075	K01077	K01078	K01101
Thermodesulfobium	1	13	1	0	0	0	0	0	0	0	0	0	0	0	0	0	0	1	0	0
Thermodesulfovibrio	2	60	1	0	0	0	0	0	0	0	0	0	0	0	0	0	0	1	0	0
Thermofilum	1	9	0	0	0	0	0	0	0	0	0	0	0	0	0	0	0	0	0	0
Thermogladius	1	7	0	0	0	0	0	0	0	0	0	0	0	0	0	0	0	0	0	0
Thermomicrobium	2	92	1	0	0	0	0	1	0	0	0	0	0	0	0	0	0	0	0	1
Thermomonospora	2	125	1	0	0	0	0	0	0	0	1	0	0	0	1	0	0	0	0	0
Thermoplasma	1	11	0	0	1	0	0	0	0	0	0	0	0	0	1	0	0	0	0	0
Thermoplasmatales	1	6	0	0	0	0	0	0	0	0	0	0	0	0	0	0	0	0	0	0
Thermoproteus	1	12	0	0	1	0	0	0	0	0	0	0	0	0	0	0	0	0	0	0
Thermosediminibacter	2	64	1	0	0	1	0	0	0	0	0	0	0	0	0	0	0	0	0	0
Thermosipho	2	70	1	0	1	1	0	0	1	0	0	0	0	0	0	0	0	1	0	0
Thermosphaera	1	10	0	0	0	0	0	0	0	0	0	0	0	0	0	0	0	0	0	0
Thermosynechococcus	1	6	0	1	0	0	0	0	0	0	0	0	0	0	1	0	0	0	0	0
Thermotoga	2	80	1	0	1	1	0	0	1	0	0	0	0	0	0	0	0	1	0	0
Thermovibrio	2	58	1	0	0	0	0	0	0	0	0	0	0	0	0	0	0	0	0	0
Thermovirga	1	9	1	0	0	1	0	0	0	0	0	0	0	0	0	0	0	0	0	0
Thermus	2	107	1	0	1	1	0	0	0	0	0	0	0	1	0	0	0	1	1	0
Thioalkalimicrobium	1	4	0	1	0	0	0	0	0	0	0	0	0	0	0	0	0	0	0	0
Thioalkalivibrio	2	91	1	1	0	0	0	0	0	0	0	0	0	0	1	0	0	0	0	0
Thiobaca	1	2	0	0	0	0	0	0	0	0	0	0	0	0	0	0	0	0	0	0
Thiobacillus	2	67	1	1	0	0	0	0	0	0	0	0	0	0	0	0	0	0	0	0
Thiocapsa	1	2	0	0	0	0	0	0	0	0	0	0	0	0	0	0	0	0	0	0
Thiocystis	1	11	1	0	0	0	0	0	0	0	0	0	0	0	0	0	0	0	0	0
Thioflavicoccus	1	13	1	0	0	0	0	0	0	0	0	0	0	0	0	0	0	1	0	0
Thiomicrospira	2	50	0	1	0	0	0	0	0	0	0	0	0	0	0	0	0	0	0	0
Thiomonas	2	85	0	1	0	0	0	0	0	0	0	0	0	0	1	0	0	0	0	0
Thiorhodococcus	1	2	0	0	0	0	0	0	0	0	0	0	0	0	0	0	0	0	0	0
Thiothrix	1	2	0	0	0	0	0	0	0	0	0	0	0	0	0	0	0	0	0	0
Tistrella	1	42	1	1	0	0	0	1	0	0	0	0	1	1	1	0	0	0	0	0
Tolumonas	2	100	1	1	0	1	0	0	0	0	0	0	0	0	1	0	0	1	0	0
Transposon	1	2	0	0	0	0	0	0	0	0	0	0	0	0	0	0	0	0	0	0
Treponema	1	63	0	0	0	1	0	0	0	0	0	0	0	0	1	0	0	1	0	0
Trichodesmium	2	71	0	1	0	0	0	0	0	0	0	0	0	0	1	0	0	0	0	0
Trichormus	1	12	0	1	0	0	0	0	0	0	0	0	0	0	0	0	0	0	0	0
Tropheryma	2	22	1	0	0	0	0	0	0	0	0	0	0	0	0	0	0	0	0	0
Truepera	2	104	1	0	1	1	0	0	0	0	0	0	0	0	1	0	0	1	0	1
Tsukamurella	2	123	1	0	0	0	1	1	0	0	1	0	0	0	0	0	0	1	0	0
Turneriella	1	16	1	1	1	1	0	0	0	0	0	0	0	0	1	0	0	0	0	0
Uliginosibacterium	1	2	0	0	0	0	0	0	0	0	0	0	0	0	0	0	0	0	0	0
Ureaplasma	1	24	1	0	1	1	0	0	0	0	0	0	0	0	0	0	0	0	0	0
Ureibacillus	1	2	0	0	0	0	0	0	0	0	0	0	0	0	0	0	0	0	0	0
Variovorax	3	187	0	1	0	0	0	1	0	0	0	0	1	1	1	0	1	0	0	0
Veillonella	1	54	1	0	0	0	0	0	0	0	0	0	0	0	0	0	0	0	0	0
Verminephrobacter	3	188	0	1	0	0	0	1	0	1	0	0	1	0	1	0	0	0	0	0
Verrucosispora	3	126	1	0	1	1	0	0	0	1	0	0	0	1	1	0	0	0	0	0
Vibrio	2	195	1	1	1	1	1	0	0	1	0	0	0	1	1	0	0	1	0	0
Vibrionales	1	46	1	0	1	1	0	0	0	0	0	0	0	0	0	0	0	0	0	0
Vitreoscilla	1	1	0	0	0	0	0	0	0	0	0	0	0	0	0	0	0	0	0	0
Vulcanisaeta	1	14	0	0	0	0	0	0	0	1	0	0	0	0	0	0	0	0	0	0
Waddlia	2	68	0	0	1	1	0	0	0	0	0	0	0	0	0	0	0	0	0	0
Wautersia	1	2	0	0	0	0	0	0	0	0	0	0	0	0	0	0	0	0	0	0
Weeksella	2	65	1	0	1	1	0	0	0	0	0	0	0	0	0	0	0	0	0	0

Genus	Cluster Number	Protein Counts	K00760	K00799	K00857	K00876	K01026	K01031	K01034	K01039	K01041	K01045	K01053	K01055	K01061	K01066	K01075	K01077	K01078	K01101
**Weissella**	1	14	1	0	1	1	0	0	0	0	0	0	0	0	0	0	0	0	0	1
**Wenxinia**	1	2	0	0	0	0	0	0	0	0	0	0	0	0	0	0	0	0	0	0
**Wigglesworthia**	1	29	0	0	0	0	0	0	0	0	0	0	0	0	0	0	0	0	0	0
**Wolbachia**	1	32	0	1	0	0	0	0	0	0	0	0	0	0	0	0	0	0	0	0
**Wolinella**	2	51	0	0	0	0	0	0	0	0	0	0	0	0	0	0	0	0	0	0
**Xanthobacter**	3	176	1	1	0	0	0	1	0	1	0	0	1	1	1	0	0	0	0	0
**Xanthobacteraceae**	1	4	0	0	0	0	0	0	0	0	0	0	0	0	0	0	0	0	0	0
**Xanthomonadaceae**	1	39	1	1	0	0	0	0	0	0	0	0	0	0	0	0	0	0	1	0
**Xanthomonas**	2	162	1	1	1	0	0	1	0	1	0	0	1	1	1	1	0	1	1	0
**Xenorhabdus**	2	127	1	1	1	1	0	0	0	0	0	0	0	0	0	0	0	0	0	0
**Xylanimonas**	2	66	1	0	1	1	0	0	0	0	0	0	0	0	0	0	0	0	0	0
**Xylella**	2	52	1	1	0	0	0	0	0	0	0	0	1	0	0	1	0	1	0	0
**Yersinia**	2	145	1	1	1	1	0	0	0	0	0	0	0	0	1	0	0	1	0	0
**Yokenella**	2	61	1	0	0	1	0	0	0	0	0	0	0	0	0	0	0	0	0	0
**Zobellia**	1	28	0	0	1	1	0	0	0	0	0	0	1	0	0	0	0	1	0	0
**Zunongwangia**	2	98	1	0	1	1	0	0	0	0	0	0	1	0	0	0	0	1	0	0
**Zymomonas**	2	67	0	1	1	0	0	0	0	0	0	0	1	1	1	1	0	1	0	0
**Zymophilus**	1	2	0	0	0	0	0	0	0	0	0	0	0	0	0	0	0	0	0	0

Genus	Cluster Number	Protein Counts	K01195	K01253	K01426	K01428	K01457	K01464	K01489	K01500	K01501	K01502	K01512	K01555	K01560	K01561	K01563	K01564	K01576	K01607
Acaricomes	1	2	0	0	0	0	0	0	0	0	0	0	0	0	0	0	0	0	0	0
Acaryochloris	2	88	0	0	1	1	0	0	1	0	0	1	1	0	0	0	0	0	0	0
Acetivibrio	1	2	0	0	0	0	0	0	0	0	0	0	0	0	0	0	0	0	0	0
Acetobacter	2	85	0	0	0	1	1	1	0	0	0	1	0	0	0	0	0	0	1	0
Acetobacteraceae	1	2	0	0	0	0	0	0	0	0	0	0	0	0	0	0	0	0	0	0
Acetobacterium	1	8	0	0	0	0	0	1	0	0	0	0	0	0	0	0	0	0	0	0
Acetohalobium	2	79	0	0	0	0	0	0	1	0	0	0	1	0	0	0	0	0	0	0
Acholeplasma	2	38	0	0	0	0	0	0	1	0	0	0	0	0	0	0	0	0	0	0
Achromobacter	2	192	0	0	1	0	1	1	0	0	1	0	1	1	0	1	0	0	0	1
Acidaminococcus	1	65	0	0	0	0	0	0	1	0	1	0	0	0	0	0	0	0	0	1
Acidianus	1	9	0	0	0	0	0	0	1	0	0	0	1	0	0	0	0	0	1	0
Acidilobus	1	8	0	0	0	0	0	0	1	0	0	0	1	0	0	0	0	0	0	0
Acidimicrobium	2	58	0	0	1	0	0	0	0	0	0	0	0	1	0	0	0	0	0	0
Acidiphilium	3	144	0	0	1	0	1	0	0	0	1	0	1	1	1	1	0	0	0	1
Acidithiobacillus	2	70	0	0	0	0	0	0	1	0	1	0	0	0	0	0	0	0	1	1
Acidobacterium	2	91	0	0	0	0	0	0	1	0	0	0	1	1	0	0	0	0	1	0
Acidocella	1	2	0	0	0	0	0	0	0	0	0	0	0	0	0	0	0	0	0	0
Acidothermus	2	68	0	0	1	0	0	0	0	0	0	0	1	0	0	0	0	0	0	0
Acidovorax	3	217	0	0	1	1	0	1	0	0	1	1	1	1	1	1	0	0	1	1
Aciduliprofundum	1	11	0	0	0	0	0	0	1	0	0	0	1	0	1	0	0	0	0	0
Acinetobacter	3	188	0	0	1	1	1	0	0	1	0	0	0	1	0	0	0	0	0	1
Actinobacillus	2	112	0	0	0	1	0	0	1	0	0	0	1	0	0	0	0	0	0	1
Actinomadura	1	1	0	0	0	0	0	0	0	0	0	0	0	0	0	0	0	0	0	0
Actinoplanes	1	52	0	0	1	1	0	1	1	0	0	0	0	1	1	1	1	0	0	1
Actinopolyspora	1	2	0	0	0	0	0	0	0	0	0	0	0	0	0	0	0	0	0	0
Actinosynnema	2	122	0	0	1	1	0	1	1	0	0	0	1	1	0	0	0	0	0	1
Advenella	1	30	0	0	0	1	0	1	0	0	0	0	0	0	0	0	0	0	0	1
Aequorivita	1	12	0	0	0	0	0	0	1	0	0	0	0	1	0	0	0	0	0	0
Aerococcus	2	57	1	0	1	0	0	0	0	0	0	0	1	0	0	0	0	0	0	0
Aeromonas	2	127	0	0	1	0	0	1	1	0	1	0	1	0	0	0	0	0	0	0
Aeropyrum	1	13	0	0	1	0	0	0	1	0	0	0	1	0	0	0	0	0	0	0
Afipia	1	54	0	0	0	1	0	0	0	0	0	1	0	0	1	0	0	0	0	1
Aggregatibacter	1	81	0	0	0	0	0	0	1	0	0	0	1	0	1	0	0	0	0	0
Agrobacterium	2	242	0	0	1	1	1	1	1	0	0	1	1	0	1	1	1	0	1	1
Ahrensia	1	2	0	0	0	0	0	0	0	0	0	0	0	0	0	0	0	0	0	0
Akkermansia	2	47	0	0	0	0	0	0	0	0	0	0	0	0	0	0	0	0	0	1
Albidiferax	3	144	0	0	0	1	1	0	0	0	1	0	1	0	1	0	0	0	0	1
Alcaligenes	1	21	0	0	0	0	0	0	0	0	0	0	0	0	0	0	0	0	0	0
Alcanivorax	2	146	0	0	1	1	1	0	0	1	0	1	1	0	1	0	1	1	0	0
Alicycliphilus	3	152	0	0	1	0	0	0	0	0	1	0	1	0	0	0	0	0	0	0
Alicyclobacillus	2	91	0	0	1	0	1	0	0	0	0	0	0	0	1	0	0	0	0	0
Aliivibrio	2	118	0	0	0	1	0	0	1	0	0	0	1	0	0	0	0	0	0	0
Alishewanella	1	1	0	0	0	0	0	0	0	0	0	0	0	0	0	0	0	0	0	0
Alistipes	1	52	1	0	0	1	0	0	1	0	0	0	0	0	0	0	0	0	0	1
Alkalilimnicola	2	97	0	0	0	1	1	0	0	1	1	0	1	0	0	0	0	0	0	0
Alkaliphilus	2	62	0	0	1	0	0	1	1	0	0	0	0	0	0	0	0	0	0	0
Allochromatium	2	84	0	0	0	0	0	0	0	0	1	0	1	0	0	0	0	0	0	0
Alphaproteobacteria	1	3	0	0	0	0	0	0	0	0	0	0	0	0	0	0	0	0	0	0
Alteromonadales	1	12	0	0	0	0	0	0	0	0	0	0	0	0	0	0	0	0	0	0
Alteromonas	1	37	0	0	1	1	0	1	1	0	1	0	0	0	1	0	1	0	0	0
Aminobacter	1	4	0	0	0	0	0	0	0	0	0	0	0	0	0	0	0	0	0	0
Aminobacterium	2	59	0	0	0	0	0	1	1	0	0	0	1	0	0	0	0	0	0	0

Genus	Cluster Number	Protein Counts	K01195	K01253	K01426	K01428	K01457	K01464	K01489	K01500	K01501	K01502	K01512	K01555	K01560	K01561	K01563	K01564	K01576	K01607
**Ammonifex**	2	50	0	0	0	0	0	0	1	0	0	0	1	0	0	0	0	0	0	0
**Amphibacillus**	1	13	0	0	1	0	0	0	1	0	0	0	1	0	0	0	0	0	0	1
**Amphritea**	1	2	0	0	0	0	0	0	0	0	0	0	0	0	0	0	0	0	0	0
**Amycolatopsis**	2	201	0	1	1	1	1	1	1	0	0	0	1	1	1	1	1	0	1	1
**Amycolicicoccus**	1	43	0	0	1	1	1	0	1	0	0	0	1	1	1	0	1	0	0	1
**Anabaena**	2	92	0	0	1	1	0	0	0	1	0	0	1	0	0	1	1	0	0	1
**Anaerobaculum**	1	11	0	0	1	0	0	1	1	0	0	0	1	0	0	0	0	0	0	0
**Anaerococcus**	2	46	0	0	0	1	0	1	1	0	0	0	1	0	0	0	0	0	0	0
**Anaerolinea**	2	66	0	0	0	0	0	1	1	0	0	0	1	0	0	0	0	0	0	0
**Anaeromyxobacter**	3	117	0	0	1	1	0	0	1	0	0	0	1	0	1	1	1	0	0	0
**Anaerostipes**	1	44	0	0	0	0	0	1	0	0	0	0	1	0	1	0	0	0	0	0
**Anaerotruncus**	1	2	0	0	0	0	0	0	0	0	0	0	0	0	0	0	0	0	0	0
**Anaplasma**	1	35	0	0	0	0	0	0	0	0	0	0	0	0	0	0	0	0	0	0
**Ancylobacter**	1	3	0	0	0	0	0	0	0	0	0	0	0	0	0	0	0	0	0	0
**Aneurinibacillus**	1	1	0	0	0	0	0	0	0	0	0	0	0	0	0	0	0	0	0	0
**Angomonas**	1	1	0	0	0	0	0	0	0	0	0	0	0	0	0	0	0	0	0	0
**Anoxybacillus**	2	72	0	0	0	0	0	0	1	0	0	0	1	0	0	0	0	0	0	0
**Aquifex**	2	45	0	0	0	0	0	0	0	0	0	0	0	0	0	0	0	0	0	0
**Aquimarina**	1	2	0	0	0	0	0	0	0	0	0	0	0	0	0	0	0	0	0	0
**Arcanobacterium**	2	45	0	0	0	0	0	0	1	0	0	0	0	0	0	0	0	0	0	0
**Archaeoglobus**	1	14	0	0	0	0	0	0	0	0	0	0	1	0	0	0	0	0	0	0
**Arcobacter**	2	101	0	0	0	1	1	0	0	0	0	0	1	0	0	0	0	0	0	1
**Aromatoleum**	3	133	0	0	0	0	0	0	0	0	1	0	0	0	1	0	0	0	0	0
**Arsenophonus**	1	4	0	0	0	0	0	0	0	0	0	0	0	0	0	0	0	0	0	0
**Arthrobacter**	2	206	1	1	1	1	1	1	1	1	1	1	1	0	1	0	0	0	1	1
**Arthrospira**	2	67	0	0	0	1	0	0	0	0	0	0	1	0	0	0	0	0	0	0
**Aster**	1	10	0	0	0	0	0	0	0	0	0	0	0	0	0	0	0	0	0	0
**Asticcacaulis**	2	103	1	0	1	0	1	0	1	0	0	0	0	1	0	0	0	0	0	1
**Atopobium**	1	28	0	0	0	0	0	1	0	0	0	0	0	0	0	0	0	0	0	0
**Aurantimonas**	1	2	0	0	0	0	0	0	0	0	0	0	0	0	0	0	0	0	0	0
**Avibacterium**	1	17	0	0	0	0	0	0	0	0	0	0	0	0	0	0	0	0	0	0
**Azoarcus**	3	172	0	0	1	1	1	1	0	0	1	1	1	0	1	1	0	0	0	1
**Azorhizobium**	3	149	0	0	1	1	1	1	0	0	1	1	1	0	1	1	0	0	0	1
**Azorhizophilus**	1	4	0	0	0	0	0	0	0	0	0	0	0	0	0	0	0	0	0	0
**Azospira**	1	19	0	0	0	0	0	0	0	0	1	0	1	0	1	0	0	0	0	1
**Azospirillum**	2	198	1	0	1	1	1	1	1	0	1	0	1	0	1	1	0	0	0	1
**Azotobacter**	3	183	0	0	0	1	0	0	0	0	0	0	1	1	0	1	0	0	0	1
**Bacillales**	1	9	0	0	0	0	0	0	1	0	0	0	0	0	0	0	0	0	0	0
**Bacilli**	1	3	0	0	0	0	0	0	0	0	0	0	0	0	0	0	0	0	0	0
**Bacillus**	2	237	1	1	1	1	1	1	1	0	1	1	1	0	1	0	0	0	0	1
**Bacteria**	2	86	0	0	1	1	0	0	0	0	1	0	0	0	0	0	1	0	0	0
**Bacteriovorax**	2	98	0	0	0	0	0	0	1	0	0	0	0	0	1	0	1	0	0	0
**Bacteroidales**	2	59	0	0	0	0	0	0	1	0	0	0	0	0	0	0	0	0	0	1
**Bacteroides**	2	96	1	0	0	0	0	0	1	0	0	0	0	0	0	0	0	0	0	1
**Bacteroidetes**	1	2	0	0	0	0	0	0	0	0	0	0	0	0	0	0	0	0	0	0
**Bartonella**	2	50	0	0	0	0	0	0	0	0	0	0	0	0	0	0	0	0	0	0
**Baumannia**	2	24	0	0	0	0	0	0	0	0	0	0	0	0	0	0	0	0	0	0
**Bdellovibrio**	2	87	0	0	0	0	0	0	1	0	0	0	0	0	1	0	1	0	0	0
**Beijerinckia**	3	123	0	0	1	1	0	0	1	0	1	0	1	0	1	1	0	0	0	1
**Belliella**	1	14	0	0	0	0	0	0	1	0	0	0	0	1	0	0	0	0	0	0
**Beutenbergia**	2	82	0	0	1	0	0	1	1	0	0	0	1	0	0	0	0	0	0	1
**Bifidobacterium**	2	61	1	0	1	1	0	0	1	0	0	0	1	0	1	0	0	0	0	1

Genus	Cluster Number	Protein Counts	K01195	K01253	K01426	K01428	K01457	K01464	K01489	K01500	K01501	K01502	K01512	K01555	K01560	K01561	K01563	K01564	K01576	K01607
Bizionia	1	1	0	0	0	0	0	0	0	0	0	0	0	0	0	0	0	0	0	0
Blastococcus	1	39	0	0	1	0	0	0	1	0	0	0	1	1	1	0	1	0	0	1
Blattabacterium	1	23	0	0	0	1	0	0	0	0	0	0	0	0	0	0	0	0	0	0
Blautia	1	22	0	0	0	0	0	0	1	0	0	0	0	0	0	0	0	0	0	0
Bordetella	3	227	0	0	1	1	1	1	0	0	1	0	1	1	1	1	0	0	0	1
Borrelia	1	32	0	0	0	0	0	0	1	0	0	0	1	0	0	0	0	0	0	0
Brachybacterium	1	18	0	0	1	1	0	0	1	0	0	0	1	0	0	0	0	0	0	0
Brachymonas	1	4	0	0	0	0	0	0	0	0	0	0	0	0	0	0	0	0	0	0
Brachyspira	2	79	1	0	0	0	0	1	0	0	0	0	1	0	0	0	0	0	0	1
Bradyrhizobiaceae	1	20	0	0	0	1	0	0	0	0	0	0	0	0	0	0	0	0	0	0
Bradyrhizobium	3	254	0	1	1	1	1	1	1	1	0	1	1	1	1	1	1	1	1	1
Brenneria	1	20	0	0	0	0	0	0	0	0	0	0	0	0	0	0	0	0	0	0
Brevibacillus	2	122	0	0	1	0	0	1	1	0	1	0	0	0	1	0	0	0	0	1
Brevibacterium	1	4	0	0	0	0	0	0	0	0	0	0	0	0	0	0	0	0	0	0
Brevundimonas	2	105	0	0	1	0	0	0	0	0	0	0	0	0	0	0	0	0	1	1
Brucella	3	138	0	0	1	1	0	1	0	0	0	0	0	0	1	0	0	1	0	1
Buchnera	1	26	0	0	0	0	0	0	0	0	0	0	0	0	0	0	0	0	0	0
Burkholderia	3	424	0	1	1	1	1	1	1	0	1	1	1	1	1	1	1	1	1	1
Burkholderiaceae	3	143	0	0	1	0	0	0	0	0	1	1	1	1	1	1	0	0	0	1
Burkholderiales	1	24	0	0	0	0	0	0	0	0	0	0	0	0	0	0	0	0	0	0
Buttiauxella	1	2	0	0	0	0	0	0	0	0	0	0	0	0	0	0	0	0	0	0
Butyrivibrio	2	67	0	0	0	0	0	0	1	0	0	0	1	0	0	0	0	0	0	0
Caldanaerobacter	2	63	0	0	1	0	0	0	1	0	0	0	1	0	0	0	0	0	0	0
Caldicellulosiruptor	2	75	1	0	0	1	0	0	1	0	0	0	0	0	0	0	0	0	0	0
Caldilinea	1	23	0	0	1	1	0	1	1	0	0	0	1	0	0	0	0	0	0	0
Caldimonas	1	6	0	0	0	0	0	0	0	0	0	0	0	0	0	0	0	0	0	0
Caldisericum	1	10	0	0	0	0	0	0	1	0	0	0	1	0	0	0	0	0	0	0
Caldisphaera	1	7	0	0	0	0	0	0	0	0	0	0	1	0	0	0	0	0	0	0
Calditerrivibrio	2	74	0	0	0	0	0	0	0	0	0	0	1	0	0	0	0	0	0	0
Caldivirga	1	13	1	0	0	0	0	0	1	0	0	0	1	0	0	0	0	0	0	1
Calothrix	1	43	0	0	1	1	0	0	0	0	0	0	0	0	0	0	0	0	0	0
Calyptogena	2	34	0	0	0	0	0	0	0	0	0	0	0	0	0	0	0	0	0	0
Campylobacter	2	80	0	0	0	0	0	0	0	0	1	0	0	0	0	0	0	1	0	1
Candidatus	2	290	1	1	1	1	1	1	1	1	1	0	1	1	1	1	1	1	0	1
Capnocytophaga	1	50	0	0	0	0	0	0	1	0	0	0	0	1	0	0	0	0	0	0
Carbophilus	1	2	0	0	0	0	0	0	0	0	0	0	0	0	0	0	0	0	0	0
Carboxydibrachium	1	14	0	0	0	0	0	0	0	0	0	0	0	0	0	0	0	0	0	0
Carboxydothermus	2	68	0	0	0	0	0	0	1	0	0	0	0	0	0	0	0	0	0	0
Carica	1	2	1	0	0	0	0	0	0	0	0	0	0	0	0	0	0	0	0	0
Carnobacterium	2	71	1	0	1	0	0	0	1	0	0	0	1	0	1	1	0	0	0	1
Catenibacterium	1	2	0	0	0	0	0	0	0	0	0	0	0	0	0	0	0	0	0	1
Catenovulum	1	2	0	0	0	0	0	0	0	0	0	0	0	0	0	0	0	0	0	0
Catenulispora	3	159	0	0	1	1	1	0	1	0	0	0	0	1	0	1	1	0	0	1
Caulobacter	3	168	0	0	1	0	1	0	1	0	1	0	0	1	1	1	1	0	0	0
Cedecea	1	45	0	0	0	0	0	0	0	0	0	0	0	0	0	0	0	0	0	0
Cellulomonas	2	96	0	0	0	1	1	1	1	0	0	0	1	0	1	0	1	0	0	0
Cellulophaga	2	105	1	0	1	0	0	0	1	0	1	0	0	1	1	0	0	0	0	0
Cellulosilyticum	1	14	0	0	0	0	1	1	1	0	0	0	0	0	0	0	0	0	0	0
Cellvibrio	2	85	0	0	0	0	1	0	0	0	0	0	0	0	0	0	0	0	0	0
Cenarchaeum	1	7	0	0	0	1	0	1	0	0	0	0	0	0	0	0	0	0	0	0
Chamaesiphon	1	13	0	0	1	1	0	0	1	0	0	1	0	0	0	0	0	0	0	0
Chelativorans	3	136	0	0	1	1	0	1	1	0	1	0	0	0	1	0	0	0	0	1

Genus	Cluster Number	Protein Counts	K01195	K01253	K01426	K01428	K01457	K01464	K01489	K01500	K01501	K01502	K01512	K01555	K01560	K01561	K01563	K01564	K01576	K01607
Chelatococcus	1	2	0	0	0	0	0	0	0	0	0	0	0	0	0	0	0	0	0	0
Chitinophaga	2	98	0	0	0	1	0	1	1	0	0	0	1	0	0	0	0	0	1	0
Chlamydia	1	36	0	0	0	0	0	0	0	1	0	0	0	0	0	0	0	0	0	0
Chlamydomonas	1	3	0	0	0	0	0	0	0	0	0	0	0	0	0	0	0	0	0	0
Chlamydophila	1	31	0	0	0	0	0	0	0	0	0	0	0	0	0	0	0	0	0	0
Chlorobaculum	2	61	0	0	0	0	0	0	0	0	0	0	1	0	1	0	0	0	0	0
Chlorobium	2	89	0	0	0	0	0	0	0	0	1	0	1	0	1	0	0	0	0	0
Chlorobium/Pelodictyon	1	2	0	0	0	0	0	0	0	0	0	0	0	0	0	0	0	0	0	0
Chloroflexus	2	105	0	0	1	0	0	0	1	0	0	0	1	1	1	1	1	0	0	0
Chlorogloeopsis	1	9	0	0	0	1	0	0	0	0	0	0	0	0	0	0	0	0	0	0
Chloroherpeton	2	59	0	0	0	0	0	0	0	0	0	0	1	0	1	0	0	0	0	0
Chromobacterium	2	117	0	0	0	0	0	0	1	0	1	0	0	0	1	0	0	0	1	1
Chromohalobacter	2	145	0	0	0	1	0	0	1	0	0	0	1	0	1	1	0	0	1	0
Chroococcidiopsis	1	24	0	0	1	1	0	0	0	0	0	0	1	1	1	0	0	0	1	0
Chryseobacterium	2	104	0	0	1	0	0	1	0	0	0	0	0	1	0	1	0	0	1	1
Citreicella	1	13	0	0	0	0	0	0	0	0	0	0	0	0	0	0	0	0	0	0
Citrobacter	2	190	0	0	1	1	0	1	1	0	0	0	1	0	0	0	0	0	0	1
Clavibacter	2	83	0	0	1	0	1	0	1	0	0	0	0	0	1	0	0	0	0	1
Clonorchis	1	1	0	0	0	0	0	0	0	0	0	0	0	0	0	0	0	1	0	0
Clostridiales	2	77	0	0	0	0	0	1	1	0	0	0	1	0	1	0	0	0	0	1
Clostridium	2	191	1	0	1	1	1	1	1	0	1	0	1	0	1	0	0	0	1	1
Cohnella	1	2	0	0	0	0	0	0	0	0	0	0	0	0	0	0	0	0	0	0
Coleofasciculus	1	4	0	0	0	0	0	0	0	0	0	0	0	0	0	0	0	0	0	0
Collimonas	1	35	0	0	1	1	0	0	0	0	1	0	0	1	1	0	0	0	0	1
Colwellia	2	130	0	0	0	0	0	1	1	0	0	0	1	0	1	0	1	0	0	1
Comamonas	2	196	0	0	1	1	0	1	0	0	1	0	0	1	0	0	0	0	1	0
Conexibacter	3	117	1	0	1	1	0	1	0	0	0	0	0	0	0	0	1	0	1	0
Coprobacillus	1	2	0	0	0	0	0	0	0	0	0	0	0	0	0	0	0	0	0	0
Coprococcus	2	76	0	0	0	0	0	1	1	0	0	0	1	0	1	0	0	0	0	1
Coprothermobacter	2	45	0	0	0	0	0	0	1	0	0	0	1	0	0	0	0	0	0	0
Coraliomargarita	2	68	0	0	0	1	1	0	1	0	0	0	1	0	0	0	1	0	0	0
Corallococcus	1	29	0	0	1	1	0	0	1	0	0	0	1	0	0	0	1	0	0	0
Coriobacterium	2	36	0	0	0	0	0	0	1	0	0	0	1	0	0	0	0	0	0	0
Corynebacterineae	1	5	0	0	0	0	0	0	0	0	0	0	0	0	0	0	0	0	0	0
Corynebacterium	2	175	1	0	1	1	1	1	1	0	0	0	1	0	0	0	0	0	0	1
Coxiella	2	54	0	0	0	0	0	0	0	0	0	0	1	0	0	0	1	0	0	0
Crinalium	1	21	0	0	1	1	0	0	0	0	0	0	1	1	1	0	0	0	0	0
Croceibacter	2	80	0	0	1	0	0	0	1	0	1	0	0	1	1	0	0	0	0	0
Crocosphaera	1	6	0	0	0	0	0	0	0	0	0	0	0	0	0	0	0	0	0	0
Cronobacter	2	120	0	0	0	0	1	0	1	0	0	0	1	0	0	0	0	0	0	1
Cryptobacterium	2	39	0	0	0	0	0	0	0	0	0	0	0	0	0	0	0	0	0	0
Cucumis	1	9	0	0	0	0	0	0	0	0	0	0	0	0	0	0	0	0	0	0
Cupriavidus	3	317	0	1	1	1	1	1	0	1	1	1	1	1	1	1	0	0	1	1
Curtobacterium	1	5	0	0	0	0	0	0	0	0	0	0	0	0	0	0	0	0	0	0
Curvibacter	1	27	0	0	0	0	0	0	0	0	0	0	0	0	0	0	0	0	0	0
Cyanobacterium	1	9	0	0	1	1	0	0	0	0	0	0	0	0	0	0	0	0	0	1
Cyanobium	1	10	0	0	0	1	0	0	0	0	0	1	1	0	0	0	0	0	0	0
Cyanothece	1	34	0	0	1	1	1	0	1	0	0	0	1	1	0	1	1	0	1	1
Cyclobacterium	1	19	0	0	0	0	0	0	1	0	0	0	0	0	1	0	0	0	0	0
Cycloclasticus	1	21	0	0	0	1	0	0	0	0	0	0	0	0	0	0	0	0	0	1
Cylindrospermopsis	1	4	0	0	0	0	0	0	0	0	0	0	0	0	0	0	0	0	0	0
Cylindrospermum	1	30	0	0	1	1	0	0	0	0	0	0	1	0	0	0	0	0	0	0

Genus	Cluster Number	Protein Counts	K01195	K01253	K01426	K01428	K01457	K01464	K01489	K01500	K01501	K01502	K01512	K01555	K01560	K01561	K01563	K01564	K01576	K01607
**Cytophaga**	2	69	0	0	0	1	0	0	0	0	0	0	0	0	0	0	0	0	0	0
**Dactylococcopsis**	1	8	0	0	0	1	0	0	0	0	0	1	1	0	0	0	0	0	0	0
**Dechloromonas**	1	40	0	0	1	1	1	0	0	0	1	0	1	0	0	1	0	0	0	1
**Dechlorosoma**	1	19	0	0	0	0	0	0	0	0	1	0	1	0	1	0	0	0	0	1
**Deferribacter**	2	73	0	0	0	0	0	0	0	0	0	0	1	0	1	0	0	0	0	0
**Dehalobacter**	1	9	0	0	0	0	0	0	1	0	0	0	0	0	1	0	0	0	0	0
**Dehalococcoides**	2	41	0	0	0	0	0	0	0	0	0	0	1	0	0	0	0	0	0	0
**Dehalogenimonas**	1	37	0	0	0	0	0	0	0	0	0	0	1	0	0	0	0	0	0	0
**Deinococcus**	2	140	0	0	1	1	0	1	1	0	0	0	1	0	1	1	1	0	0	1
**Delftia**	3	177	0	0	1	1	0	1	0	0	1	0	0	1	0	1	0	0	1	1
**Denitrovibrio**	2	67	0	0	0	0	0	0	0	0	0	0	1	0	0	0	0	0	0	0
**Desulfarculus**	2	92	0	0	1	0	0	0	0	0	0	0	1	0	0	0	0	0	0	1
**Desulfatibacillum**	3	84	0	0	1	0	0	0	0	0	0	0	1	0	0	0	1	0	0	1
**Desulfitobacterium**	2	102	0	0	0	0	0	1	1	0	0	0	0	0	1	0	0	0	0	0
**Desulfobacca**	2	65	0	0	0	0	0	0	1	0	0	0	1	0	0	0	0	0	0	0
**Desulfobacterium**	2	113	0	0	0	0	0	0	0	0	0	0	0	0	1	0	1	0	0	1
**Desulfobacula**	1	21	0	0	0	0	0	1	0	0	0	0	0	0	0	0	1	0	0	1
**Desulfobulbus**	2	88	0	0	0	0	0	0	0	0	0	0	1	0	0	0	1	0	0	0
**Desulfocapsa**	1	9	0	0	0	0	0	1	0	0	0	0	0	0	0	0	1	0	0	0
**Desulfococcus**	2	80	0	0	1	0	0	0	0	0	0	0	1	0	0	0	1	0	0	0
**Desulfohalobium**	2	52	0	0	0	0	0	0	0	0	0	0	1	0	0	0	0	0	0	0
**Desulfomicrobium**	2	65	0	0	0	0	0	0	1	0	0	0	0	0	0	0	0	0	0	0
**Desulfomonile**	1	25	0	0	0	0	0	0	1	0	0	1	1	0	0	0	0	0	0	1
**Desulforhabdus**	1	2	0	0	0	0	0	0	0	0	0	0	0	0	0	0	0	0	0	0
**Desulfosporosinus**	1	30	0	0	0	0	0	1	1	0	0	0	1	0	1	0	0	0	0	1
**Desulfotalea**	2	66	0	0	0	0	0	0	0	0	0	0	0	0	0	0	1	0	0	0
**Desulfotomaculum**	2	98	0	0	1	1	0	1	1	0	0	0	1	0	0	0	1	0	0	0
**Desulfovibrio**	2	144	0	0	1	1	0	1	1	0	0	0	1	0	0	0	0	0	1	1
**Desulfurispirillum**	2	78	0	0	0	0	0	0	0	0	0	0	1	0	0	0	0	0	0	0
**Desulfurivibrio**	2	71	0	0	0	0	0	0	0	0	0	0	0	0	0	0	0	0	0	0
**Desulfurobacterium**	2	53	0	0	0	0	0	0	0	0	0	0	0	0	0	0	0	0	0	0
**Desulfurococcus**	1	11	0	0	1	0	0	1	1	0	0	0	1	0	0	0	0	0	0	0
**Diaphorobacter**	1	16	0	0	0	0	0	0	0	0	0	0	0	0	0	0	0	0	0	0
**Dichelobacter**	2	45	0	0	0	0	0	0	0	1	0	0	0	0	0	0	0	0	0	0
**Dickeya**	2	155	0	0	1	0	1	1	1	0	1	0	1	0	0	0	0	0	0	1
**Dictyoglomus**	2	62	1	0	0	0	0	0	1	0	0	0	1	0	0	0	0	0	0	0
**Dinoroseobacter**	3	148	0	0	0	1	0	1	1	0	0	0	0	1	1	1	0	0	0	1
**Dokdonia**	1	3	0	0	0	0	0	0	0	0	0	0	0	0	0	0	0	0	0	0
**Dorea**	1	7	0	0	0	0	0	0	0	0	0	0	0	0	0	0	0	0	0	1
**Dyadobacter**	2	83	1	0	0	0	0	0	0	0	0	0	0	1	0	0	0	0	0	0
**Echinicola**	1	20	0	0	0	0	0	0	1	0	0	0	1	1	0	0	0	0	0	0
**Edwardsiella**	2	126	1	0	0	1	0	1	1	0	0	0	1	0	1	0	0	0	0	0
**Eggerthella**	1	51	0	0	1	0	0	0	0	0	0	0	0	0	0	0	0	0	0	1
**Ehrlichia**	2	37	0	0	0	0	0	0	0	1	0	0	0	0	0	0	0	0	0	0
**Eikenella**	1	4	0	0	0	0	0	0	0	0	0	0	0	0	0	0	0	0	0	0
**Elusimicrobium**	2	44	0	0	0	0	0	0	0	0	0	0	0	0	0	0	0	0	0	0
**Emticicia**	1	21	0	0	0	0	0	0	0	0	0	0	0	1	0	0	0	0	0	0
**Enhydrobacter**	1	1	0	0	0	0	0	0	0	0	0	0	0	0	0	0	0	0	0	0
**Ensifer**	1	2	0	0	0	0	0	0	0	0	0	0	0	0	0	0	0	0	0	0
**Enterobacter**	2	202	0	0	1	1	1	1	1	0	0	0	1	0	0	0	0	0	1	1
**Enterobacteriaceae**	2	211	1	0	1	1	0	1	1	0	0	0	1	0	0	0	0	1	0	1
**Enterococcus**	1	187	0	0	1	1	0	1	1	0	0	0	1	0	1	0	0	0	0	1

Genus	Cluster Number	Protein Counts	K01195	K01253	K01426	K01428	K01457	K01464	K01489	K01500	K01501	K01502	K01512	K01555	K01560	K01561	K01563	K01564	K01576	K01607
Enterovibrio	1	15	0	0	0	0	0	0	0	0	0	0	0	0	0	0	0	0	0	0
Erwinia	2	151	0	0	0	0	1	0	1	0	0	1	1	0	1	1	0	0	0	1
Erysipelothrix	1	9	0	0	0	0	0	0	1	0	0	0	0	0	1	0	0	0	0	0
Erysipelotrichaceae	1	4	0	0	0	0	0	0	0	0	0	0	0	0	0	0	0	0	0	0
Erythrobacter	2	80	0	0	1	0	0	0	1	0	0	0	0	0	1	0	1	0	0	0
Escherichia	2	242	1	0	1	1	0	1	1	0	0	0	1	0	0	0	0	1	0	1
Ethanoligenens	2	69	0	0	0	1	1	1	1	0	0	0	1	0	0	0	0	0	0	1
Eubacteriaceae	1	2	0	0	0	0	0	0	0	0	0	0	0	0	0	0	0	0	0	0
Eubacterium	2	103	1	0	0	0	0	1	1	0	0	0	1	0	1	0	0	0	0	1
Eudoraea	1	4	0	0	0	0	0	0	0	0	0	0	0	0	0	0	0	0	0	0
Euryarchaeota	1	1	0	0	0	0	0	0	0	0	0	0	0	0	0	0	0	0	0	0
Exiguobacterium	2	93	0	0	1	0	0	0	1	0	0	0	1	0	1	0	0	0	0	1
Faecalibacterium	1	12	1	0	0	0	0	0	1	0	0	0	0	0	0	0	0	0	0	0
Ferrimonas	2	115	0	0	0	0	0	0	1	0	0	0	1	1	0	0	0	0	0	0
Ferroglobus	1	14	0	0	1	0	0	0	0	0	0	0	1	0	0	0	0	0	0	0
Ferroplasma	1	11	0	0	0	0	0	1	0	0	0	0	1	0	0	0	0	0	1	0
Fervidicoccus	1	7	0	0	0	0	0	0	1	0	0	0	1	0	0	0	0	0	0	0
Fervidobacterium	2	54	0	0	1	0	0	0	1	0	0	0	1	0	0	0	0	0	0	0
Fibrella	1	17	0	0	1	0	0	0	0	0	0	0	0	1	0	0	0	0	0	0
Fibrobacter	2	52	0	0	0	0	0	0	0	0	0	0	0	0	0	0	0	0	0	0
Filifactor	1	9	0	0	0	0	0	0	1	0	0	0	0	0	0	0	0	0	0	0
Finegoldia	2	55	0	0	0	0	0	0	1	0	0	0	0	0	0	0	0	0	0	0
Firmicutes	1	15	0	0	1	0	0	0	1	0	0	0	0	0	1	0	0	0	0	1
Fischerella	1	15	0	0	1	1	0	0	0	0	0	0	0	0	0	0	0	0	0	0
Flavobacteria	1	7	0	0	0	0	0	0	0	0	0	0	0	0	0	0	0	0	0	0
Flavobacteriaceae	2	69	0	0	0	0	0	0	1	0	0	0	0	1	1	0	0	0	0	0
Flavobacteriales	1	3	0	0	0	0	0	0	0	0	0	0	0	0	0	0	0	0	0	0
Flavobacterium	2	103	1	0	0	1	0	0	1	0	0	1	0	1	1	0	0	0	0	0
Flexibacter	1	17	0	0	1	0	0	0	1	0	0	0	0	1	1	0	0	0	0	0
Flexistipes	1	11	0	0	0	0	0	0	0	0	0	0	1	0	1	0	0	0	0	0
Fluoribacter	1	6	0	0	0	0	0	0	0	0	0	0	0	0	0	0	0	0	0	0
Fluviicola	2	69	0	0	0	0	0	0	1	0	0	0	0	1	0	0	0	0	0	0
Formosa	1	2	0	0	0	0	0	0	0	0	0	0	0	0	0	0	0	0	0	0
Francisella	2	88	0	0	0	1	0	0	1	0	0	0	0	0	0	0	0	0	0	0
Frankia	3	202	1	1	1	1	1	1	1	1	0	0	1	1	1	1	0	0	1	1
Frateuria	1	25	0	0	0	1	0	0	1	0	1	0	0	0	0	0	0	0	0	1
Fructobacillus	1	2	0	0	0	0	0	0	0	0	0	0	0	0	0	0	0	0	0	0
Fulvimarina	1	2	0	0	0	0	0	0	0	0	0	0	0	0	0	0	0	0	0	0
Fusobacterium	1	48	0	0	0	0	0	0	0	0	0	0	0	0	0	0	0	0	0	0
Gallibacterium	2	87	0	0	0	0	0	0	1	0	0	0	1	0	0	0	0	0	0	0
Gallionella	2	67	0	0	0	0	1	0	0	0	1	0	0	0	0	0	0	0	0	0
Gammaproteobacteria	1	9	0	0	0	0	0	0	0	0	0	0	0	0	0	0	0	0	0	0
Gardnerella	1	31	0	0	0	0	0	0	0	0	0	0	0	0	0	0	0	0	0	0
Geitlerinema	1	9	0	0	1	1	0	0	0	0	0	0	1	0	0	0	0	0	0	1
Gemmatimonas	2	93	0	0	1	0	0	0	1	0	0	0	0	1	0	0	1	0	0	0
Geobacillus	2	168	0	0	1	1	0	1	1	0	1	0	1	0	0	0	0	0	0	0
Geobacter	2	150	0	0	0	0	0	0	1	0	0	0	1	0	1	0	1	0	0	1
Geodermatophilus	2	136	0	0	1	1	0	0	1	0	0	0	1	1	1	1	1	0	0	1
Geopsychrobacter	1	2	0	0	0	0	0	0	0	0	0	0	0	0	0	0	0	0	0	0
Gillisia	1	11	0	0	0	0	0	0	0	0	0	0	0	0	0	0	0	0	0	0
Gilvimarinus	1	4	0	0	1	0	0	0	0	0	0	0	0	0	0	0	0	0	0	0
Glaciecola	2	117	0	0	1	1	0	0	1	0	1	0	0	1	1	0	1	0	0	0

Genus	Cluster Number	Protein Counts	K01195	K01253	K01426	K01428	K01457	K01464	K01489	K01500	K01501	K01502	K01512	K01555	K01560	K01561	K01563	K01564	K01576	K01607
**Gloeobacter**	2	67	0	0	1	0	1	0	0	0	0	0	1	0	0	0	0	0	0	0
**Gloeocapsa**	1	20	0	0	1	1	0	1	0	0	0	0	1	0	1	0	0	0	0	0
**Gloeothece**	1	1	0	0	0	0	0	0	0	0	0	0	0	0	0	0	0	0	0	0
**Gluconacetobacter**	2	96	0	0	1	0	0	1	1	0	1	1	0	0	1	1	0	0	1	1
**Gluconobacter**	2	76	0	0	0	0	0	1	1	0	0	1	0	0	1	0	0	0	0	0
**Gordonia**	1	158	0	0	1	1	1	1	1	0	0	0	1	0	1	0	1	0	1	1
**Gordonibacter**	1	32	0	0	1	0	0	0	0	0	0	0	0	0	1	0	0	0	0	0
**Gramella**	2	104	0	0	0	0	0	0	1	1	0	0	0	1	1	0	0	0	0	0
**Granulibacter**	2	90	0	1	1	1	1	0	0	1	1	0	1	0	0	0	0	0	0	1
**Granulicella**	2	98	1	0	0	0	1	1	1	0	0	0	1	1	0	0	0	0	1	1
**Grimontia**	1	9	0	0	0	0	0	0	0	0	0	0	0	0	0	0	0	0	0	0
**Haemophilus**	2	117	1	0	0	1	0	0	1	0	0	0	1	0	0	0	0	0	0	1
**Hafnia**	1	29	0	0	0	0	0	0	0	0	0	0	0	0	0	0	0	0	0	0
**Hahella**	3	130	0	0	1	1	0	0	0	0	1	0	1	0	0	0	1	0	0	0
**Halalkalicoccus**	1	18	0	0	0	1	0	1	1	0	0	0	1	0	1	0	0	0	1	0
**Halanaerobium**	2	89	1	0	1	0	0	1	1	0	1	0	1	0	0	0	0	0	0	0
**Haliangium**	2	108	0	0	1	1	0	1	1	0	0	1	1	0	0	0	1	0	1	0
**Haliscomenobacter**	2	107	1	0	0	0	0	0	1	0	0	0	0	1	1	0	0	0	0	0
**Haloarcula**	1	22	0	0	1	1	0	1	1	0	1	0	1	0	1	0	0	0	0	0
**Halobacillus**	1	19	0	0	1	1	0	1	1	0	0	0	1	0	1	0	0	0	0	0
**Halobacteriaceae**	1	1	0	0	0	0	0	0	0	0	0	0	0	0	0	0	0	0	0	0
**Halobacterium**	1	10	0	0	0	0	0	0	1	0	0	0	0	0	0	0	0	0	0	0
**Halobacteroides**	1	14	0	0	0	0	0	1	1	0	0	0	1	0	0	0	0	0	0	0
**Haloferax**	1	20	0	0	0	1	0	1	1	0	0	0	1	0	1	0	1	0	0	0
**Halogeometricum**	1	12	0	0	0	0	0	0	1	0	0	0	1	0	0	0	0	0	0	0
**Halomicrobium**	1	10	0	0	0	0	0	0	1	0	0	0	1	0	0	0	0	0	0	0
**Halomonas**	1	149	0	0	1	1	0	0	1	0	0	0	1	0	1	0	0	0	1	1
**Halopiger**	1	16	0	0	0	0	0	0	1	0	0	0	1	0	1	0	0	0	1	0
**Haloquadratum**	1	19	0	0	0	1	0	1	1	0	0	0	1	0	1	0	0	0	0	0
**Halorhabdus**	1	10	1	0	0	0	0	0	1	0	0	0	1	0	0	0	0	0	0	0
**Halorhodospira**	2	71	0	0	0	0	1	0	0	0	1	0	1	0	1	0	0	0	0	0
**Halorubrum**	1	16	0	0	1	0	0	1	1	0	0	0	1	0	1	0	0	0	0	0
**Haloterrigena**	1	19	1	0	1	1	0	1	1	0	0	0	1	0	1	0	1	0	1	0
**Halothece**	1	9	0	0	0	1	0	0	0	0	0	0	1	0	0	0	0	0	0	0
**Halothermothrix**	2	50	0	0	0	0	0	0	1	0	0	0	1	0	0	0	0	0	0	0
**Halothiobacillus**	2	65	0	0	1	0	1	0	0	0	0	0	0	0	0	0	0	0	0	1
**Halovivax**	1	12	0	0	1	0	0	0	1	0	0	0	1	0	0	0	0	0	1	0
**Helicobacter**	2	79	0	0	1	1	0	0	0	0	0	0	1	0	0	0	0	1	0	1
**Heliobacillus**	1	4	0	0	0	0	0	0	0	0	0	0	0	0	0	0	0	0	0	0
**Heliobacterium**	2	51	0	0	0	0	0	1	1	0	0	0	0	0	0	0	0	0	0	0
**Herbaspirillum**	2	172	0	0	1	1	1	0	0	0	1	0	0	1	1	0	0	0	1	1
**Herminiimonas**	3	89	0	0	1	0	0	0	0	0	1	0	0	0	1	1	0	0	0	0
**Herpetosiphon**	3	90	0	0	0	1	0	0	1	0	0	0	1	1	1	0	0	0	0	0
**Hippea**	2	44	0	0	0	0	0	0	0	0	0	0	1	0	0	0	0	0	0	0
**Hirschia**	2	102	0	0	0	1	0	0	0	0	0	0	0	0	1	0	0	0	0	1
**Histophilus**	2	72	1	0	0	0	0	0	1	0	0	0	0	0	0	0	0	0	0	1
**Hoeflea**	1	16	0	0	0	0	0	0	0	0	0	0	0	0	0	0	0	0	0	0
**Hydrogenivirga**	1	2	0	0	0	0	0	0	0	0	0	0	0	0	0	0	0	0	0	0
**Hydrogenobacter**	2	55	0	0	0	0	0	0	0	0	0	0	1	0	0	0	0	0	0	0
**Hydrogenobaculum**	2	44	0	0	0	0	0	0	0	0	0	0	1	0	0	0	0	0	0	0
**Hydrogenophaga**	1	9	0	0	0	0	0	0	0	0	0	0	0	0	0	0	0	0	0	0
**Hylemonella**	1	4	0	0	0	0	0	0	0	0	0	0	0	0	0	0	0	0	0	0

Genus	Cluster Number	Protein Counts	K01195	K01253	K01426	K01428	K01457	K01464	K01489	K01500	K01501	K01502	K01512	K01555	K01560	K01561	K01563	K01564	K01576	K01607
Hyperthermus	1	6	0	0	0	0	0	0	1	0	0	0	0	0	0	0	0	0	0	0
Hyphomicrobium	2	93	0	0	1	1	0	0	0	0	0	1	0	0	1	0	0	0	0	0
Hyphomonas	3	105	0	1	1	0	1	0	1	0	0	0	0	0	0	0	1	0	0	1
Ideonella	1	18	0	0	0	0	0	0	0	0	0	0	0	0	0	0	0	0	0	0
Idiomarina	2	86	0	0	0	0	0	0	0	0	0	0	0	0	1	0	0	0	0	0
Ignavibacterium	1	14	1	0	0	0	0	0	1	0	0	0	1	0	0	0	0	0	0	1
Ignicoccus	1	5	0	0	0	0	0	0	0	0	0	0	0	0	0	0	0	0	0	0
Ignisphaera	1	8	1	0	0	0	0	0	0	0	0	0	1	0	0	0	0	0	0	0
Ilyobacter	2	85	0	0	0	0	0	1	1	0	0	0	1	0	1	0	0	0	0	0
Intrasporangium	2	114	0	0	1	0	0	0	1	0	0	0	1	1	1	0	0	0	0	0
Isoptericola	1	18	0	0	1	1	0	0	1	0	0	0	1	0	1	0	0	0	0	0
Isosphaera	2	75	0	0	0	0	0	0	1	0	0	0	0	0	0	0	1	0	0	0
Jannaschia	3	151	0	0	1	1	1	1	1	0	1	0	0	1	1	1	1	0	0	1
Janthinobacterium	2	99	0	0	0	1	1	0	0	0	1	1	0	0	1	1	0	0	0	0
Jonesia	2	56	0	0	0	0	0	0	1	0	0	0	0	0	0	0	0	0	0	0
Joostella	1	3	0	0	0	0	0	0	0	0	0	0	0	0	0	0	0	0	0	0
Kaistia	1	2	0	0	0	0	0	0	0	0	0	0	0	0	0	0	0	0	0	0
Kangiella	2	84	0	0	0	0	0	0	0	0	1	0	0	0	0	0	0	0	0	0
Ketogulonicigenium	2	102	0	0	0	1	0	1	1	0	0	0	0	0	0	0	0	0	0	1
Kineococcus	1	24	0	0	0	1	1	1	1	0	0	0	0	0	0	0	0	0	0	0
Kinetoplastibacterium	1	2	0	0	0	0	0	0	0	0	0	0	0	0	0	0	0	0	0	0
Kingella	1	4	0	0	0	0	0	0	0	0	0	0	0	0	0	0	0	0	0	0
Kitasatospora	1	36	0	0	1	1	0	0	1	0	0	0	1	1	0	0	0	0	1	1
Klebsiella	3	222	0	0	1	1	1	1	1	0	0	0	1	0	0	0	0	0	1	1
Kluyvera	1	1	0	0	0	0	0	0	0	0	0	0	0	0	0	0	0	0	0	0
Kocuria	2	87	0	0	0	1	0	0	1	0	0	0	1	0	0	1	0	0	0	1
Kordia	1	5	0	0	0	0	0	0	0	0	0	0	0	0	0	0	0	0	0	0
Kosmotoga	2	61	0	0	0	0	0	1	1	0	0	0	1	0	0	0	0	0	0	0
Kribbella	2	99	0	0	1	0	0	1	1	0	0	0	0	1	0	1	1	0	0	0
Krokinobacter	2	90	0	0	1	0	0	0	1	0	1	0	0	1	1	0	0	0	0	0
Kyrpidia	2	113	0	0	1	1	0	0	1	0	0	0	1	0	0	0	0	0	0	0
Kytococcus	2	70	0	0	0	0	0	0	1	0	0	0	0	1	0	0	0	0	0	0
Labrenzia	1	4	0	0	0	0	0	0	0	0	0	0	0	0	0	0	0	0	0	0
Lachnoanaerobaculum	1	1	0	0	0	0	0	0	0	0	0	0	0	0	0	0	0	0	0	0
Lachnospiraceae	1	51	0	0	0	0	0	1	0	0	0	0	1	0	0	0	0	0	0	0
Lacinutrix	1	20	0	0	1	0	0	0	1	0	1	0	0	1	1	0	0	0	0	0
Lactobacillus	2	119	1	0	1	0	0	0	1	0	0	0	1	0	1	0	0	0	0	1
Lactococcus	2	82	0	0	1	0	0	0	1	0	0	0	1	0	1	0	0	0	0	1
Lamprocystis	1	2	0	0	0	0	0	0	0	0	0	0	0	0	0	0	0	0	0	0
Laribacter	2	80	0	0	0	1	0	0	0	0	1	0	1	0	0	0	0	0	0	1
Lawsonia	2	33	0	0	0	0	0	0	0	0	0	0	0	0	0	0	0	0	0	0
Leadbetterella	2	79	0	0	0	0	0	0	1	0	0	0	0	1	0	0	0	0	0	0
Leeuwenhoekiella	1	2	0	0	0	0	0	0	0	0	0	0	0	0	0	0	0	0	0	0
Legionella	2	117	0	0	1	0	0	0	1	0	1	0	1	0	1	0	0	0	1	0
Leifsonia	2	48	0	0	1	0	0	0	1	0	0	0	0	0	0	0	0	0	0	0
Leptolyngbya	1	10	0	0	0	1	0	0	0	0	0	1	1	0	0	0	0	0	0	0
Leptospira	2	76	0	0	1	0	0	0	0	0	0	0	1	0	1	0	0	0	0	0
Leptospirillum	1	10	0	0	0	0	0	0	0	0	0	0	1	0	0	0	0	0	0	1
Leptothrix	3	186	0	0	0	1	1	0	0	0	1	0	1	1	1	1	0	0	0	1
Leptotrichia	2	43	0	0	0	0	0	0	1	0	0	0	0	0	0	0	0	0	0	0
Leucobacter	1	2	0	0	0	0	0	0	0	0	0	0	0	0	0	0	0	0	0	0
Leuconostoc	2	64	0	0	0	0	0	0	1	0	0	0	1	0	0	0	0	0	0	1

Genus	Cluster Number	Protein Counts	K01195	K01253	K01426	K01428	K01457	K01464	K01489	K01500	K01501	K01502	K01512	K01555	K01560	K01561	K01563	K01564	K01576	K01607
Liberibacter	1	4	0	0	0	0	0	0	0	0	0	0	0	0	0	0	0	0	0	0
Limnohabitans	1	11	0	0	0	0	0	0	0	0	0	0	0	0	0	0	0	0	0	0
Listeria	2	77	0	0	1	0	0	0	1	0	0	0	1	0	0	0	0	0	0	1
Listeriaceae	1	6	0	0	0	0	0	0	0	0	0	0	0	0	0	0	0	0	0	0
Listonella	1	38	0	0	0	0	0	0	1	0	0	0	1	0	0	0	0	0	0	0
Loktanella	1	3	0	0	0	0	0	0	0	0	0	0	0	0	0	0	0	0	0	0
Lysinibacillus	2	97	0	0	0	1	0	1	1	0	0	0	1	0	1	0	0	0	0	0
Macrococcus	2	60	0	0	0	0	0	0	1	0	0	0	0	0	0	0	0	0	0	0
Magnetococcus	2	64	0	0	0	1	0	0	0	0	1	0	0	0	0	0	0	0	0	0
Magnetospirillum	2	113	0	0	1	0	0	0	0	0	0	0	0	0	1	0	0	0	0	1
Mahella	2	58	0	0	0	0	0	0	1	0	0	0	0	0	0	0	0	0	0	1
Mannheimia	2	85	0	0	0	0	0	0	1	0	0	0	1	0	0	0	0	0	0	1
Maribacter	3	87	0	0	0	0	0	0	1	0	1	0	0	1	1	0	1	0	0	0
Maricaulis	3	87	0	0	0	0	0	0	0	0	1	0	0	0	0	0	0	0	0	0
Marichromatium	1	2	0	0	0	0	0	0	0	0	0	0	0	0	0	0	0	0	0	0
Marinithermus	2	77	0	0	1	0	0	0	1	0	0	0	1	0	1	0	0	0	0	1
Marinitoga	1	15	0	0	0	0	0	1	1	0	0	0	1	0	0	0	0	0	0	0
Marinobacter	2	163	0	0	1	1	0	0	0	0	1	0	1	0	1	0	1	0	0	1
Marinomonas	3	192	0	0	1	1	0	1	1	0	0	0	0	1	1	0	0	0	0	1
Maritimibacter	1	7	0	0	0	0	0	0	0	0	0	0	0	0	0	0	0	0	0	0
Marivirga	2	96	0	0	1	0	0	0	1	0	1	0	0	1	0	0	0	0	0	0
Martelella	1	3	0	0	0	0	0	0	0	0	0	0	0	0	0	0	0	0	0	0
Marvinbryantia	1	1	0	0	0	0	0	0	0	0	0	0	0	0	0	0	0	0	0	0
Massilia	1	4	0	0	0	0	0	0	0	0	0	0	0	0	0	0	0	0	0	0
Mastigocladopsis	1	16	0	0	0	1	0	0	0	0	0	0	0	0	0	0	0	0	0	0
Megamonas	1	47	0	0	0	0	0	0	0	0	0	0	1	0	0	0	0	0	0	1
Megasphaera	1	14	0	0	0	0	0	1	1	0	1	0	0	0	0	0	0	0	0	1
Meiothermus	2	110	0	0	1	0	0	1	1	0	0	0	1	0	0	0	0	0	0	1
Melissococcus	2	46	0	0	0	0	0	0	1	0	0	0	1	0	1	0	0	0	0	1
Mesoflavibacter	1	6	0	0	0	0	0	0	0	0	0	0	0	0	0	0	0	0	0	0
Mesoplasma	1	23	0	0	0	0	0	0	1	0	0	0	0	0	0	0	0	0	0	0
Mesorhizobium	2	201	0	0	1	1	1	1	1	0	0	0	1	0	1	1	1	0	0	1
Mesotoga	1	14	0	0	1	0	0	1	1	0	0	0	1	0	0	0	0	0	0	0
Metallosphaera	1	18	0	0	0	1	0	0	1	0	0	0	1	0	1	1	0	0	1	0
Methanobacterium	1	7	0	0	0	0	0	0	0	0	0	0	0	0	0	0	0	0	0	1
Methanobrevibacter	1	9	0	0	0	0	0	0	0	0	0	0	0	0	0	0	0	0	0	1
Methanocaldococcus	1	6	0	0	0	0	0	0	0	0	0	0	0	0	0	0	0	0	0	0
Methanocella	1	15	0	0	1	0	0	0	0	0	0	0	1	0	0	0	0	0	0	1
Methanococcaceae	1	1	0	0	0	0	0	0	0	0	0	0	0	0	0	0	0	0	0	0
Methanococcoides	1	5	0	0	0	0	0	0	0	0	0	0	1	0	0	0	0	0	0	0
Methanococcus	1	9	0	0	0	0	0	0	0	0	0	0	0	0	0	0	0	0	0	1
Methanocorpusculum	1	7	0	0	0	0	0	0	0	0	0	0	1	0	0	0	0	0	0	0
Methanoculleus	1	8	0	0	0	0	0	0	0	0	0	0	1	0	0	0	0	0	0	0
Methanohalobium	1	5	0	0	0	0	0	0	0	0	0	0	1	0	0	0	0	0	0	0
Methanohalophilus	1	5	0	0	0	0	0	0	0	0	0	0	1	0	0	0	0	0	0	0
Methanolobus	1	5	0	0	0	0	0	0	0	0	0	0	1	0	0	0	0	0	0	0
Methanomethylovorans	1	5	0	0	0	0	0	0	0	0	0	0	1	0	0	0	0	0	0	0
Methanoplanus	1	7	0	0	0	0	0	0	0	0	0	0	1	0	0	0	0	0	0	1
Methanopyrus	1	4	0	0	0	0	0	0	0	0	0	0	0	0	0	0	0	0	0	0
Methanoregula	1	8	0	0	0	0	0	0	0	0	0	0	1	0	0	0	0	0	0	1
Methanosaeta	1	9	0	0	0	0	0	0	0	0	0	0	1	0	0	0	0	0	0	1
Methanosalsum	1	7	0	0	0	0	0	0	0	0	0	0	1	0	0	0	0	0	0	0

Genus	Cluster Number	Protein Counts	K01195	K01253	K01426	K01428	K01457	K01464	K01489	K01500	K01501	K01502	K01512	K01555	K01560	K01561	K01563	K01564	K01576	K01607
Methanosarcina	1	18	0	0	0	0	0	1	0	0	1	0	1	0	0	0	0	0	0	1
Methanosphaera	1	7	0	0	0	0	0	0	0	0	0	0	0	0	0	0	0	0	0	0
Methanosphaerula	1	8	0	0	0	0	0	0	0	0	0	0	1	0	0	0	0	0	0	1
Methanospirillum	1	6	0	0	0	0	0	0	0	0	0	0	1	0	0	0	0	0	0	0
Methanothermobacter	1	6	0	0	0	0	0	0	0	0	0	0	0	0	0	0	0	0	0	1
Methanothermococcus	1	5	0	0	0	0	0	0	0	0	0	0	0	0	0	0	0	0	0	0
Methanothermus	1	5	0	0	0	0	0	0	0	0	0	0	1	0	0	0	0	0	0	0
Methanotorris	1	5	0	0	0	0	0	0	0	0	0	0	0	0	0	0	0	0	0	0
Methylacidiphilum	2	48	0	0	0	0	0	1	0	0	0	0	1	0	0	0	0	0	0	0
Methylarcula	1	2	0	0	0	0	0	0	0	0	0	0	0	0	0	0	0	0	0	0
Methylibium	1	38	0	0	1	1	1	0	1	0	1	1	0	0	1	1	0	0	0	0
Methylobacillus	2	61	0	0	1	1	0	0	0	0	1	0	0	0	0	0	0	0	0	0
Methylobacterium	3	236	0	0	1	1	1	1	1	0	1	1	1	1	1	1	0	0	1	1
Methylocella	3	117	0	0	0	1	1	0	1	0	1	0	1	0	1	1	0	0	1	0
Methylococcus	2	80	0	0	0	0	0	0	0	0	1	0	1	0	0	0	0	0	0	0
Methylocystis	1	21	0	0	1	0	0	0	0	0	0	0	1	0	1	1	0	0	0	1
Methyloferula	1	2	0	0	0	0	0	0	0	0	0	0	0	0	0	0	0	0	0	0
Methylomicrobium	1	12	0	0	0	1	0	0	0	0	1	0	1	0	0	0	0	0	0	0
Methylomonas	1	15	0	0	0	1	0	0	1	0	1	1	1	0	0	0	0	0	0	0
Methylophaga	1	7	0	0	0	1	0	0	0	0	0	0	1	0	0	0	0	0	0	0
Methylophilus	1	2	0	0	0	0	0	0	0	0	0	0	0	0	0	0	0	0	0	0
Methylosinus	1	2	0	0	0	0	0	0	0	0	0	0	0	0	0	0	0	0	0	0
Methylotenera	2	72	0	0	0	1	1	0	0	0	1	0	0	0	0	0	0	0	0	0
Methyloversatilis	1	5	0	0	0	0	0	0	0	0	0	0	0	0	0	0	0	0	0	0
Methylovorus	2	66	0	0	0	1	1	0	0	0	1	0	0	0	0	0	0	0	0	0
Micavibrio	1	9	0	0	0	0	0	0	0	0	0	0	0	0	0	0	0	0	0	0
Microbacterium	2	115	0	0	1	0	0	1	1	0	0	0	1	0	0	0	0	0	0	0
Microchaete	1	23	0	0	0	1	0	0	0	0	0	0	0	0	0	0	0	0	0	0
Micrococcus	2	72	0	0	1	1	0	0	1	0	0	0	0	0	0	0	0	0	0	0
Microcoleus	1	17	0	0	1	1	0	0	0	0	0	0	1	0	1	0	0	0	0	0
Microcystis	2	67	0	0	1	1	0	0	1	0	0	0	0	0	0	1	0	0	0	0
Microlunatus	1	25	0	0	0	1	0	1	0	0	0	0	1	1	1	0	0	0	0	1
Micromonospora	2	115	0	0	1	1	0	0	1	0	0	0	0	1	0	1	0	0	1	0
Microvirga	1	6	0	0	0	0	0	0	0	0	0	0	0	0	0	0	0	0	0	0
Mobiluncus	1	41	0	0	0	0	0	0	0	0	0	0	0	0	0	0	0	0	0	0
Modestobacter	1	43	1	0	1	1	0	1	1	0	0	0	1	1	1	1	1	0	0	1
Moorea	1	2	0	0	0	0	0	0	0	0	0	0	0	0	0	0	0	0	0	0
Moorella	2	69	0	0	0	0	0	1	1	0	0	0	1	0	0	0	0	0	0	0
Moraxella	2	66	0	0	0	0	0	0	0	0	0	0	0	0	0	0	0	0	0	1
Morganella	1	29	0	0	0	1	0	1	1	0	0	0	1	0	0	0	0	0	1	0
Moritella	1	2	0	0	0	0	0	0	0	0	0	0	0	0	0	0	0	0	0	0
Muricauda	1	23	0	0	1	0	0	0	1	0	0	0	0	1	0	0	0	0	0	0
Mycobacterium	3	306	0	1	1	1	1	1	1	0	1	1	1	1	1	1	1	0	1	1
Mycoplasma	1	53	0	0	0	0	0	0	1	0	0	0	0	0	0	0	0	0	0	1
Myxococcus	2	110	0	0	1	1	0	0	1	0	0	0	1	0	0	0	1	0	0	0
Nakamurella	2	115	0	0	1	1	1	0	1	0	0	0	1	1	0	1	0	0	0	0
napthalene	1	2	0	0	0	0	0	0	0	0	0	0	0	0	0	0	0	0	0	0
Natranaerobius	2	55	0	0	1	0	0	0	1	0	1	0	1	0	0	0	0	0	0	0
Natrialba	1	16	0	0	1	0	0	1	1	0	0	0	1	0	0	0	0	0	1	0
Natrinema	1	16	0	0	0	0	0	0	1	0	0	0	1	0	1	0	1	0	1	0
Natronobacterium	1	11	0	0	1	0	0	0	1	0	0	0	1	0	0	0	0	0	0	0
Natronococcus	1	21	0	0	1	1	0	1	1	0	0	0	1	0	1	0	0	0	1	0

Genus	Cluster Number	Protein Counts	K01195	K01253	K01426	K01428	K01457	K01464	K01489	K01500	K01501	K01502	K01512	K01555	K01560	K01561	K01563	K01564	K01576	K01607
Natronomonas	1	20	0	0	1	1	0	1	1	0	0	0	1	0	1	0	0	0	1	0
Nautilia	2	48	0	0	0	0	0	0	0	0	0	0	1	0	0	0	0	0	0	0
Neisseria	2	67	0	0	0	0	0	0	0	0	1	0	0	0	0	0	0	0	0	0
Neisseriaceae	1	1	0	0	0	0	0	0	0	0	0	0	0	0	0	0	0	0	0	0
Neorickettsia	1	31	0	0	0	0	0	0	0	0	0	0	0	0	0	0	0	0	0	0
Neptuniibacter	1	2	0	0	0	0	0	0	0	0	0	0	0	0	0	0	0	0	0	0
Niastella	1	21	1	0	0	1	0	1	1	0	0	0	1	0	0	0	0	0	0	0
Nitratifractor	2	54	0	0	0	0	0	0	0	0	0	0	1	0	0	0	0	0	0	0
Nitratireductor	1	19	0	0	0	0	0	0	0	0	0	0	0	0	0	0	0	0	0	0
Nitratiruptor	2	40	0	0	0	0	0	0	0	0	0	0	0	0	0	0	0	0	0	0
Nitrobacter	2	99	0	0	0	0	0	0	0	0	0	0	1	1	0	0	0	0	0	0
Nitrococcus	1	2	0	0	0	0	0	0	0	0	0	0	0	0	0	0	0	0	0	0
Nitrosococcus	2	94	0	0	0	1	0	0	0	0	1	0	1	0	0	0	0	0	0	0
Nitrosomonas	2	72	0	0	0	1	0	0	0	0	1	0	0	0	0	0	0	0	0	0
Nitrosopumilaceae	1	7	0	0	0	0	0	1	0	0	0	0	1	0	0	0	0	0	0	0
Nitrosopumilus	1	11	0	0	0	1	0	1	1	0	0	0	1	0	0	0	0	0	0	0
Nitrosospira	2	74	0	0	0	1	1	0	0	0	1	0	0	0	0	0	0	0	0	0
Nocardia	2	185	0	0	1	1	1	1	1	0	0	1	1	1	1	0	1	0	1	1
Nocardioidaceae	1	2	0	0	0	0	0	0	0	0	0	0	0	0	0	0	0	0	0	0
Nocardioides	2	157	0	0	1	0	0	1	1	0	0	0	1	1	1	0	0	0	0	0
Nocardiopsis	2	102	0	0	1	1	0	1	1	0	0	0	1	1	1	1	0	0	0	1
Nodularia	1	22	0	0	0	1	0	0	0	0	0	0	0	0	0	0	0	0	0	0
Nonlabens	1	19	0	0	1	0	0	0	1	0	0	0	0	1	1	0	0	0	0	0
Nostoc	2	111	0	0	1	1	0	0	0	0	0	0	1	0	1	1	0	0	1	1
Nostocaceae	1	2	0	0	0	0	0	0	0	0	0	0	0	0	0	0	0	0	0	0
Novosphingobium	2	169	0	0	1	1	1	0	1	0	0	1	0	1	1	0	0	0	1	0
Oceanibaculum	1	3	0	0	0	0	0	0	0	0	0	0	0	0	0	0	0	0	0	0
Oceanibulbus	1	12	0	0	0	0	0	0	0	0	0	0	0	0	0	0	0	0	0	0
Oceanicola	1	9	0	0	0	0	0	0	0	0	0	0	0	0	0	0	0	0	0	0
Oceanimonas	1	33	0	0	0	1	0	1	0	0	0	0	1	0	0	0	0	0	0	0
Oceaniovalibus	1	2	0	0	0	0	0	0	0	0	0	0	0	0	0	0	0	0	0	0
Oceanithermus	2	72	0	0	0	0	0	0	1	0	0	0	1	0	0	0	0	0	0	1
Oceanobacillus	2	91	0	0	1	0	0	0	1	0	0	0	1	0	0	0	0	0	0	0
Oceanospirillum	1	2	0	0	0	0	0	0	0	0	0	0	0	0	0	0	0	0	0	0
Ochrobactrum	2	133	0	0	1	1	0	1	0	0	1	0	0	0	1	1	0	0	0	1
Octadecabacter	1	29	0	0	1	1	0	1	1	0	1	0	0	0	1	1	0	0	0	1
Oenococcus	2	41	0	0	1	0	0	0	1	0	0	0	0	0	0	0	0	0	0	0
Oleomonas	1	2	0	0	0	0	0	0	0	0	0	0	0	0	0	0	0	0	0	0
Oligotropha	3	87	0	0	0	0	0	0	0	0	0	1	1	0	1	0	0	0	0	1
Olsenella	2	40	0	0	0	0	0	0	1	0	0	0	1	0	0	0	0	0	0	0
Opitutus	2	67	1	0	0	1	0	0	1	0	0	0	1	0	0	0	1	0	0	0
Orientia	1	21	0	0	0	0	0	0	0	0	0	0	0	0	0	0	0	0	0	0
Ornithobacterium	1	11	0	0	0	1	0	0	1	0	0	0	0	0	1	0	0	0	0	0
Oscillatoria	1	16	0	0	1	1	0	0	0	0	0	0	1	0	0	0	0	0	0	1
Oscillatoriales	1	2	0	0	0	0	0	0	0	0	0	0	0	0	0	0	0	0	0	0
Oscillibacter	1	20	0	0	0	0	0	1	1	0	0	0	0	0	0	0	0	0	0	0
Owenweeksia	1	15	0	0	0	0	0	0	0	0	0	0	1	1	1	0	1	0	0	0
Oxalobacteraceae	1	4	0	0	0	0	0	0	0	0	0	0	0	0	0	0	0	0	0	0
Paenibacillus	2	152	1	0	1	1	1	1	1	0	0	0	1	0	1	0	1	0	0	1
Paludibacter	2	67	1	0	0	0	0	0	1	0	0	0	0	0	0	0	0	0	0	0
Pandoraea	1	18	0	0	1	0	0	0	0	0	0	0	0	0	0	0	0	0	0	0
Pannonibacter	1	3	0	0	0	0	0	0	0	0	0	0	0	0	0	0	0	0	0	0

Genus	Cluster Number	Protein Counts	K01195	K01253	K01426	K01428	K01457	K01464	K01489	K01500	K01501	K01502	K01512	K01555	K01560	K01561	K01563	K01564	K01576	K01607
**Pantholops**	1	23	0	0	0	0	0	0	0	0	0	0	0	0	0	0	0	0	0	0
**Pantoea**	2	181	1	0	1	0	1	1	1	0	1	1	1	0	1	1	0	0	0	1
**Parabacteroides**	2	64	0	0	0	0	0	0	1	0	0	0	0	0	0	0	0	0	0	1
**Parachlamydia**	1	11	0	0	0	0	0	0	1	0	0	0	1	0	0	0	0	0	0	1
**Paracoccus**	2	169	0	0	1	1	0	1	1	0	0	0	0	1	0	0	0	0	0	1
**Parvibaculum**	3	117	0	0	1	0	1	0	0	0	0	0	0	0	0	0	1	0	0	0
**Parvularcula**	2	72	0	0	0	0	0	0	0	0	0	0	0	0	0	0	1	0	0	0
**Pasteurella**	2	85	0	0	0	1	0	0	1	0	0	0	1	0	0	0	0	0	0	0
**Pasteurellaceae**	1	2	0	0	0	0	0	0	0	0	0	0	0	0	0	0	0	0	0	0
**Pectobacterium**	1	44	0	0	0	0	1	0	1	0	0	0	1	0	1	0	0	0	0	1
**Pediococcus**	1	43	0	0	0	0	0	0	0	0	0	0	1	0	1	0	0	0	0	1
**Pedobacter**	2	88	1	0	0	0	0	0	0	0	0	0	1	0	0	0	0	0	0	0
**Pelagibaca**	1	10	0	0	0	0	0	0	0	0	0	0	0	0	0	0	0	0	0	0
**Pelagibacterium**	1	39	0	0	0	1	1	1	1	0	1	0	0	0	1	0	0	0	1	1
**Pelobacter**	2	85	0	0	0	0	0	0	1	0	0	0	1	0	0	0	1	0	0	0
**Pelodictyon**	2	62	0	0	0	0	0	0	0	0	0	0	1	0	0	0	0	0	0	0
**Pelosinus**	1	1	0	0	0	0	0	0	0	0	0	0	0	0	0	0	0	0	0	0
**Pelotomaculum**	2	60	0	0	0	0	0	0	1	0	0	0	1	0	0	0	0	0	0	0
**Peptostreptococcus**	1	1	0	0	0	0	0	0	1	0	0	0	0	0	0	0	0	0	0	0
**Persephonella**	2	55	0	0	0	0	0	0	0	0	0	0	1	0	0	0	0	0	0	0
**Petrotoga**	2	72	0	0	1	0	0	1	1	0	0	0	1	0	0	0	0	0	0	0
**Phaeobacter**	2	55	0	0	1	1	0	1	1	0	0	0	0	1	1	0	0	0	0	1
**Phenylobacterium**	3	103	0	0	1	0	0	0	0	0	0	0	1	0	0	0	1	0	0	0
**Photobacterium**	2	119	0	0	0	0	0	1	1	0	0	0	1	0	0	0	1	0	0	1
**Photorhabdus**	2	149	0	0	0	1	0	0	1	0	0	1	1	0	0	0	0	0	0	1
**Phycisphaera**	1	9	0	0	0	1	0	1	0	0	0	0	1	0	0	0	0	0	0	0
**Phyllobacterium**	1	6	0	0	0	0	0	0	0	0	0	0	0	0	0	0	0	0	0	0
**Picrophilus**	1	15	0	0	0	0	0	0	0	0	0	0	1	0	0	0	0	0	0	0
**Pirellula**	2	85	0	0	0	0	0	0	1	0	1	0	1	0	0	0	1	0	1	0
**Planctomyces**	2	93	0	0	0	0	0	0	0	0	0	0	1	0	1	0	1	0	0	0
**Plautia**	1	17	0	0	0	0	0	0	0	0	0	0	0	0	0	0	0	0	0	0
**Plesiomonas**	1	4	0	0	0	0	0	0	0	0	0	0	0	0	0	0	0	0	0	0
**Pleurocapsa**	1	23	0	0	1	1	0	0	1	0	0	0	1	1	0	1	0	0	0	0
**Polaribacter**	2	74	0	0	1	0	0	0	1	0	0	0	0	1	0	0	0	0	0	0
**Polaromonas**	3	238	0	0	1	1	1	1	0	0	1	1	1	1	1	1	0	0	1	1
**Polymorphum**	3	173	1	0	0	1	1	1	1	0	0	0	1	0	1	1	0	0	1	1
**Polynucleobacter**	1	19	0	0	1	1	0	0	0	0	1	0	0	0	1	1	0	0	1	1
**Ponticaulis**	1	1	0	0	0	0	0	0	0	0	0	0	0	0	0	0	0	0	0	0
**Porphyromonas**	2	63	0	0	0	0	0	0	1	0	0	0	0	0	0	0	0	0	0	0
**Prevotella**	1	65	1	0	0	0	0	0	1	0	0	0	0	0	0	0	0	0	0	1
**Prochlorococcus**	2	68	0	0	0	1	0	0	0	0	1	0	0	0	0	0	0	0	0	0
**Propionibacterium**	1	62	1	0	1	0	0	0	1	0	0	0	1	0	0	0	0	0	0	1
**Prosthecochloris**	1	16	0	0	0	0	0	0	0	0	0	0	1	0	1	0	0	0	0	0
**Proteobacteria**	1	19	0	0	0	0	0	0	0	0	0	0	0	0	0	0	0	0	0	0
**Proteus**	2	113	0	0	0	1	0	0	1	0	0	0	1	0	0	0	0	0	0	0
**Providencia**	1	38	0	0	1	1	0	0	1	0	0	0	1	0	0	0	0	0	1	0
**Pseudaminobacter**	1	10	0	0	0	1	0	0	0	0	0	0	0	0	0	0	0	0	0	0
**Pseudanabaena**	1	11	0	0	0	1	0	0	0	0	0	0	1	0	0	0	0	0	0	1
**Pseudoalteromonas**	2	188	0	0	1	1	1	0	1	0	1	0	0	1	1	0	1	0	0	0
**Pseudochrobactrum**	1	7	0	0	0	0	0	0	0	0	0	0	0	0	0	0	0	0	0	0
**Pseudogulbenkiania**	1	40	0	0	0	1	0	0	0	0	1	0	1	0	0	0	0	0	0	1
**pseudomallei**	3	131	0	0	1	1	0	0	1	0	0	0	1	0	1	0	0	0	0	1

Genus	Cluster Number	Protein Counts	K01195	K01253	K01426	K01428	K01457	K01464	K01489	K01500	K01501	K01502	K01512	K01555	K01560	K01561	K01563	K01564	K01576	K01607
Pseudomonas	3	381	0	0	1	1	1	1	0	1	1	1	1	1	1	1	1	1	1	1
Pseudonocardia	3	180	0	0	1	1	1	1	0	0	1	1	1	1	1	0	1	0	0	1
Pseudorhodobacter	1	4	0	0	0	0	0	0	0	0	0	0	0	0	0	0	0	0	0	0
Pseudovibrio	1	33	1	0	1	1	0	1	1	0	0	0	1	1	1	0	0	0	0	1
Pseudoxanthomonas	2	124	0	0	1	0	0	0	0	0	0	0	1	0	0	0	1	0	0	0
Psychrobacter	2	124	0	0	1	1	0	0	0	1	1	0	0	1	1	0	1	0	1	1
Psychroflexus	1	22	0	0	1	0	0	0	1	0	0	0	0	1	1	0	0	0	0	0
Psychromonas	2	110	0	0	0	1	1	0	1	0	0	0	1	0	1	1	1	0	0	0
Pusillimonas	3	111	0	0	1	0	0	1	0	0	1	0	1	1	1	1	0	0	0	1
Pyrobaculum	1	15	0	0	0	0	0	0	1	0	0	0	1	0	1	0	0	0	0	0
Pyrococcus	1	10	0	0	1	0	0	0	0	0	0	0	1	0	1	0	0	0	0	0
Pyrolobus	1	4	0	0	0	0	0	0	0	0	0	0	0	0	0	0	0	0	0	0
Rahnella	2	143	0	0	0	0	1	0	1	0	0	0	1	0	0	1	0	0	0	1
Ralstonia	3	332	0	1	1	1	1	1	0	1	1	1	1	1	1	1	1	0	1	1
Ramlibacter	1	35	0	0	0	1	0	0	0	0	1	0	0	0	1	1	0	0	0	1
Raoultella	2	139	0	0	1	1	0	1	1	0	0	0	1	0	0	0	0	0	0	1
Raphidiopsis	1	5	0	0	0	0	0	0	0	0	0	0	0	0	0	0	0	0	0	0
Reinekea	1	2	0	0	0	0	0	0	0	0	0	0	0	0	0	0	0	0	0	0
Renibacterium	2	67	0	0	0	0	0	0	0	0	0	0	0	0	0	0	0	0	0	1
Rhizobiaceae	1	3	0	0	0	0	0	0	0	0	0	0	0	0	0	0	0	0	0	0
Rhizobiales	1	6	0	0	0	0	0	0	0	0	0	0	0	0	0	0	0	0	0	0
Rhizobium	3	257	1	0	1	1	1	1	1	0	1	1	1	0	1	1	1	1	0	1
Rhizobium/Agrobacterium	2	87	0	0	0	1	0	0	0	0	0	0	0	0	1	0	0	0	0	0
Rhodanobacter	1	21	0	0	1	0	0	0	1	0	0	0	1	0	1	0	0	0	0	0
Rhodobacter	3	175	0	0	1	1	1	1	1	0	1	0	0	0	1	1	0	0	0	1
Rhodobacteraceae	1	21	0	0	0	0	0	1	0	0	0	0	0	0	0	0	0	0	0	0
Rhodobacterales	1	34	0	0	0	0	0	1	0	0	0	0	0	1	0	0	0	0	0	0
Rhodococcus	2	292	0	0	1	1	1	0	1	1	1	0	1	1	1	1	0	1	1	1
Rhodocyclaceae	1	7	0	0	0	0	0	0	0	0	0	0	0	0	0	0	0	0	0	0
Rhodoferax	3	144	0	0	0	1	1	0	0	0	1	0	1	0	1	0	0	0	0	1
Rhodomicrobium	2	103	0	0	0	0	0	0	0	0	0	0	0	0	0	0	0	0	0	1
Rhodopirellula	2	87	0	0	0	0	0	0	1	1	0	0	1	0	1	0	1	1	0	0
Rhodopseudomonas	3	246	0	0	1	1	1	0	1	1	1	1	1	1	1	1	0	0	1	1
Rhodospirillum	2	121	0	0	1	1	1	0	1	0	0	0	1	0	0	0	0	0	0	0
Rhodothermus	2	76	1	0	0	0	0	0	0	0	0	0	1	0	0	0	0	0	0	0
Rhodovulum	1	4	0	0	0	0	0	0	0	0	0	0	0	0	0	0	0	0	0	0
Richelia	1	2	0	0	0	0	0	0	0	0	0	0	0	0	0	0	0	0	0	0
Rickettsia	2	43	0	0	0	0	0	0	0	0	0	0	0	0	0	0	0	0	0	0
Rickettsiaceae	1	5	0	0	0	0	0	0	0	0	0	0	0	0	0	0	0	0	0	0
Riemerella	2	61	0	0	0	1	0	0	1	0	0	0	0	1	0	0	0	0	0	0
Rivularia	1	18	0	0	1	1	0	0	0	0	0	0	1	0	0	0	0	0	0	1
Robiginitalea	2	83	0	0	1	0	0	0	1	0	0	0	0	1	0	0	0	0	0	0
Roseburia	2	58	1	0	0	0	0	0	1	0	0	0	1	0	0	0	0	0	0	1
Roseibium	1	3	0	0	0	0	0	0	0	0	0	0	0	0	0	0	0	0	0	0
Roseiflexus	3	100	0	0	1	0	0	0	1	0	0	0	1	0	1	1	0	1	0	0
Roseobacter	2	149	0	0	1	1	1	1	1	0	0	0	0	1	1	1	0	0	0	1
Roseomonas	1	2	0	0	0	0	0	0	0	0	0	0	0	0	0	0	0	0	0	0
Roseovarius	1	15	0	0	1	0	0	0	0	0	0	0	0	0	0	0	0	0	0	0
Rothia	1	44	0	0	0	0	0	0	0	0	0	0	1	0	0	0	0	0	0	1
Rubrivivax	1	24	0	0	1	1	0	0	1	0	0	0	0	1	1	0	0	0	0	1
Rubrobacter	3	117	0	0	1	0	0	1	1	0	1	0	1	0	1	0	0	0	0	1
Ruegeria	2	189	1	0	1	1	0	1	1	0	0	1	0	1	1	0	0	0	0	1

Genus	Cluster Number	Protein Counts	K01195	K01253	K01426	K01428	K01457	K01464	K01489	K01500	K01501	K01502	K01512	K01555	K01560	K01561	K01563	K01564	K01576	K01607
**Ruminococcaceae**	1	2	0	0	0	0	0	0	0	0	0	0	0	0	0	0	0	0	0	0
**Ruminococcus**	2	101	0	0	0	1	0	1	1	0	0	0	1	0	1	0	0	0	0	1
**Runella**	1	26	1	0	0	0	0	0	0	0	1	0	0	1	0	0	0	0	0	0
**Saccharomonospora**	2	132	0	0	1	0	0	0	1	0	0	0	1	1	0	0	1	0	0	1
**Saccharophagus**	2	81	1	0	0	1	1	0	0	0	0	0	1	0	0	0	0	0	0	0
**Saccharopolyspora**	3	194	0	1	1	1	0	1	1	1	0	0	1	1	1	1	0	1	1	1
**Saccharothrix**	1	32	0	0	1	0	0	1	1	0	0	1	1	1	0	1	0	0	1	1
**Sagittula**	1	10	0	0	0	0	0	0	0	0	0	0	0	0	0	0	0	0	0	0
**Salinibacter**	2	77	0	0	1	0	0	0	0	0	0	0	1	1	1	0	1	0	0	0
**Salinimonas**	1	1	0	0	0	0	0	0	0	0	0	0	0	0	0	0	0	0	0	0
**Salinispora**	3	127	0	1	1	0	0	0	1	0	0	0	0	1	0	0	1	0	0	0
**Salinivibrio**	1	2	0	0	0	0	0	0	0	0	0	0	0	0	0	0	0	0	0	0
**Salmonella**	2	151	1	0	0	0	0	0	1	0	0	0	1	0	0	1	0	0	0	0
**Sanguibacter**	2	73	0	0	1	0	1	1	1	0	0	0	0	0	0	0	0	0	0	0
**Saprospira**	1	13	0	0	0	0	0	0	1	0	0	0	0	1	1	0	0	0	0	0
**Scardovia**	1	1	0	0	0	0	0	0	0	0	0	0	0	0	0	0	0	0	0	0
**Scytonema**	1	10	0	0	0	1	0	0	0	0	0	0	0	0	0	0	0	0	0	0
**Sebaldella**	2	69	0	0	0	0	0	1	1	0	0	0	0	0	0	0	0	0	0	1
**Segniliparus**	2	85	0	0	0	1	1	1	1	0	0	0	0	0	0	0	0	0	0	1
**Selenomonas**	2	57	0	0	0	0	0	0	1	0	0	0	1	0	0	0	0	0	0	0
**Serinicoccus**	1	1	0	0	0	0	0	0	0	0	0	0	0	0	0	0	0	0	0	0
**Serratia**	2	186	1	0	1	0	1	0	1	0	0	0	1	0	0	0	0	0	1	1
**Shewanella**	3	214	0	0	1	1	0	1	1	0	1	1	1	0	1	0	1	0	0	1
**Shigella**	2	172	1	0	0	1	0	1	1	0	0	0	1	0	0	0	0	1	0	1
**Shinella**	1	2	0	0	0	0	0	0	0	0	0	0	0	0	0	0	0	0	0	0
**Sideroxydans**	2	81	0	0	0	0	1	0	0	0	1	0	1	0	1	0	0	0	0	0
**Silicibacter**	2	105	1	0	1	1	0	1	1	0	0	0	0	1	1	0	0	0	0	1
**Simiduia**	1	18	0	0	1	0	0	0	0	0	0	0	0	1	0	0	0	0	0	1
**Simkania**	1	6	0	0	0	0	0	0	0	0	0	0	1	0	0	0	0	0	0	0
**Simonsiella**	1	2	0	0	0	0	0	0	0	0	0	0	0	0	0	0	0	0	0	0
**Singulisphaera**	1	18	0	0	0	1	0	0	1	0	0	0	1	0	0	0	1	0	0	0
**Sinorhizobium**	3	207	0	0	1	1	1	1	1	0	1	0	1	0	1	0	0	0	0	1
**Slackia**	2	46	0	0	1	0	0	0	0	0	0	0	0	0	0	0	0	0	0	0
**Smaragdicoccus**	1	3	0	0	0	0	0	0	0	0	0	0	0	0	0	0	0	0	0	0
**Sodalis**	2	71	0	0	0	0	0	0	1	0	0	0	1	0	0	0	0	0	0	0
**Solibacillus**	1	23	0	0	0	0	0	1	1	0	0	0	0	0	1	0	0	0	0	0
**Solitalea**	1	13	0	0	1	0	0	0	0	0	0	0	1	0	0	0	0	0	0	0
**Sorangium**	3	115	0	0	1	1	0	1	1	0	0	1	1	0	1	0	1	0	0	0
**Sphaerobacter**	2	100	0	0	1	0	0	1	0	0	0	0	1	0	1	0	0	0	0	1
**Sphaerochaeta**	1	42	0	0	0	0	0	1	0	0	0	0	0	0	0	0	0	0	0	0
**Sphingobacterium**	3	85	0	0	0	0	0	0	0	0	0	0	1	0	1	0	0	0	0	1
**Sphingobium**	1	179	0	0	0	1	1	0	1	0	0	0	1	1	1	0	1	0	1	1
**Sphingomonadaceae**	1	15	0	0	0	0	0	0	0	0	0	0	0	0	0	0	0	0	0	0
**Sphingomonas**	2	220	0	1	1	1	0	0	1	0	1	0	1	1	1	0	1	0	1	1
**Sphingopyxis**	3	99	0	0	0	0	0	0	0	0	0	0	0	0	0	0	0	0	0	0
**Spirochaeta**	2	103	0	0	0	0	0	1	1	0	0	0	1	0	1	0	0	0	0	1
**Spiroplasma**	1	6	0	0	0	0	0	0	1	0	0	0	0	0	0	0	0	0	0	0
**Spirosoma**	2	107	1	0	0	0	0	0	0	0	1	0	0	1	0	0	0	0	0	0
**Stackebrandtia**	2	110	0	0	1	0	0	0	1	0	0	0	1	1	1	0	0	0	0	1
**Stanieria**	1	17	0	0	0	1	0	0	1	0	0	0	1	1	0	1	0	0	0	0
**Staphylococcus**	2	108	1	0	0	1	0	0	1	0	0	0	1	0	1	0	0	0	0	1
**Staphylothermus**	1	12	0	0	0	0	0	1	1	0	0	0	0	0	0	0	0	1	0	0

Genus	Cluster Number	Protein Counts	K01195	K01253	K01426	K01428	K01457	K01464	K01489	K01500	K01501	K01502	K01512	K01555	K01560	K01561	K01563	K01564	K01576	K01607
Stappia	1	2	0	0	0	0	0	0	0	0	0	0	0	0	0	0	0	0	0	0
Starkeya	3	134	0	0	1	1	1	1	0	0	0	1	1	0	0	1	0	0	0	1
Stenotrophomonas	3	104	0	0	1	0	0	0	0	0	0	0	0	0	0	0	1	0	0	1
Stigmatella	3	129	0	0	1	1	0	1	1	0	0	0	1	0	0	0	0	0	0	0
Streptobacillus	1	38	1	0	0	0	0	0	1	0	0	0	0	0	0	0	0	0	0	0
Streptococcus	2	121	1	0	1	1	0	0	1	0	0	0	1	0	0	0	0	0	0	1
Streptomyces	2	253	1	1	1	1	1	1	1	0	0	0	1	1	1	1	1	0	1	1
Streptosporangium	3	148	0	0	1	1	0	1	1	0	0	0	1	1	0	1	1	0	0	0
Strigomonas	1	2	0	0	0	0	0	0	0	0	0	0	0	0	0	0	0	0	0	0
Sulfitobacter	1	10	0	0	0	0	0	0	0	0	0	0	0	0	0	0	0	0	0	0
Sulfobacillus	1	30	0	0	1	1	0	1	1	0	0	0	1	0	1	0	0	0	0	0
Sulfolobus	1	23	1	0	1	1	0	0	0	0	0	0	1	0	1	1	0	0	1	1
Sulfuricurvum	2	67	0	0	0	0	1	0	0	0	0	0	0	0	0	0	0	0	0	0
Sulfurihydrogenibium	2	55	0	0	0	0	0	0	0	0	0	0	1	0	0	0	0	0	0	0
Sulfurimonas	2	60	0	0	0	0	0	0	0	0	0	0	0	0	0	0	0	0	0	0
Sulfurospirillum	1	48	0	0	0	0	0	0	0	0	0	0	1	0	0	0	0	0	0	0
Sulfurovum	2	48	0	0	0	0	0	0	0	0	0	0	1	0	0	0	0	0	0	0
Symbiobacterium	2	68	0	0	0	0	0	0	1	0	0	0	0	0	0	0	0	0	0	0
Synechococcus	1	34	0	0	1	1	0	0	1	1	1	1	1	0	0	0	1	0	0	1
Synechocystis	1	40	0	0	1	1	0	0	1	0	0	1	0	0	0	0	0	0	0	1
Synergistetes	1	27	0	0	0	0	0	0	0	0	0	0	0	0	0	0	0	0	0	0
Syntrophobacter	2	101	0	0	0	0	0	0	0	0	1	0	1	0	0	0	0	0	1	1
Syntrophobotulus	2	71	0	0	0	1	1	1	1	0	0	0	0	0	0	0	0	0	0	0
Syntrophomonas	2	58	0	0	0	0	0	0	1	0	0	0	0	0	0	0	0	0	0	0
Syntrophothermus	2	60	0	0	0	0	0	0	1	0	0	0	0	0	0	0	0	0	0	0
Syntrophus	2	66	0	0	0	0	0	0	1	0	0	0	1	0	0	0	0	0	0	1
Tannerella	1	8	0	0	0	0	0	0	1	0	0	0	0	0	0	0	0	0	0	0
Taylorella	1	43	0	0	0	0	0	0	0	0	0	0	0	0	0	0	0	0	0	0
Tepidanaerobacter	2	55	0	0	1	0	0	0	1	0	0	0	1	0	0	0	0	0	0	0
Teredinibacter	2	109	0	0	0	1	1	0	0	0	0	1	0	0	0	0	0	0	0	0
Terrabacter	1	1	0	0	0	0	0	0	0	0	0	0	0	0	0	0	0	0	0	0
Terriglobus	2	83	1	0	0	0	0	1	1	0	0	0	1	0	0	0	0	0	0	1
Tetragenococcus	1	12	0	0	1	0	0	1	1	0	0	0	1	0	0	0	0	0	0	1
Thalassiobium	1	11	0	0	0	0	0	1	0	0	0	0	0	0	0	0	0	0	0	0
Thalassobacter	1	9	0	0	0	0	0	0	0	0	0	0	0	1	0	0	0	0	0	0
Thalassolituus	1	12	0	0	1	1	0	0	0	0	0	0	0	0	0	0	0	0	0	0
Thalassospira	1	2	0	0	0	0	0	0	0	0	0	0	0	0	0	0	0	0	0	0
Thauera	1	111	0	0	0	1	0	0	0	0	1	0	1	0	0	0	0	0	0	0
Thermacetogenium	1	7	0	0	0	0	0	1	1	0	0	0	0	0	0	0	0	0	0	0
Thermaerobacter	2	79	0	0	0	0	0	0	0	0	0	0	1	0	0	0	0	0	0	0
Thermanaerovibrio	2	58	0	0	0	0	0	1	1	0	0	0	1	0	0	0	0	0	0	0
Thermincola	2	62	0	0	1	1	0	0	1	0	0	0	0	0	0	0	0	0	0	0
Thermoanaerobacter	2	76	0	0	1	0	0	0	1	0	0	0	1	0	0	0	0	0	0	0
Thermoanaerobacterium	2	64	0	0	1	0	0	0	1	0	0	0	1	0	0	0	0	0	0	0
Thermobacillus	1	11	1	0	0	0	0	0	1	0	0	0	0	0	0	0	0	0	0	0
Thermobaculum	2	62	1	0	0	0	0	0	0	0	0	0	1	0	0	0	0	0	0	0
Thermobifida	2	72	0	0	1	0	0	0	0	0	0	0	1	0	0	0	0	0	0	0
Thermobispora	2	109	0	0	1	1	0	0	1	0	0	0	1	1	0	0	0	0	1	0
Thermococcus	1	13	0	0	1	0	0	0	0	0	0	0	1	0	1	0	0	1	0	0
Thermocrinis	2	60	0	0	0	1	0	0	0	0	0	0	1	0	0	0	0	0	0	0
Thermodesulfatator	1	10	0	0	0	1	0	0	0	0	0	0	1	0	1	0	0	0	0	0
Thermodesulfobacterium	1	9	0	0	0	0	0	0	0	0	0	0	1	0	0	0	0	0	0	0

Genus	Cluster Number	Protein Counts	K01195	K01253	K01426	K01428	K01457	K01464	K01489	K01500	K01501	K01502	K01512	K01555	K01560	K01561	K01563	K01564	K01576	K01607
Thermodesulfobium	1	13	0	0	0	0	0	0	1	0	0	0	1	0	0	0	0	0	0	0
Thermodesulfovibrio	2	60	0	0	0	0	0	0	0	0	0	0	1	0	0	0	0	0	0	0
Thermofilum	1	9	0	0	0	0	0	0	0	0	0	0	1	0	0	0	0	1	0	0
Thermogladius	1	7	0	0	0	0	0	1	1	0	0	0	0	0	0	0	0	0	0	0
Thermomicrobium	2	92	0	0	1	1	0	0	1	0	0	0	1	0	1	0	0	0	0	0
Thermomonospora	2	125	0	0	1	1	0	0	1	0	0	0	1	1	0	0	0	0	0	0
Thermoplasma	1	11	0	0	0	0	0	0	0	0	0	0	1	0	0	0	0	0	1	0
Thermoplasmatales	1	6	0	0	0	0	0	0	0	0	0	0	0	0	0	0	0	0	0	0
Thermoproteus	1	12	0	0	0	0	0	0	1	0	0	0	1	0	1	0	0	0	0	0
Thermosediminibacter	2	64	0	0	0	0	0	0	1	0	0	0	1	0	0	0	0	0	0	0
Thermosipho	2	70	0	0	1	0	0	1	1	0	0	0	0	0	0	0	0	0	0	0
Thermosphaera	1	10	0	0	0	0	0	1	1	0	0	0	0	0	0	0	0	0	0	0
Thermosynechococcus	1	6	0	0	0	1	0	0	0	0	0	0	0	0	0	0	0	0	0	0
Thermotoga	2	80	1	0	1	0	0	0	1	0	0	0	1	0	0	0	0	0	0	0
Thermovibrio	2	58	0	0	0	0	0	0	1	0	0	0	1	0	0	0	0	0	0	0
Thermovirga	1	9	0	0	0	0	0	1	1	0	0	0	1	0	0	0	0	0	0	0
Thermus	2	107	0	0	0	0	0	0	1	0	0	0	1	0	0	0	0	0	1	1
Thioalkalimicrobium	1	4	0	0	0	0	0	0	0	0	0	0	0	0	0	0	0	0	0	0
Thioalkalivibrio	2	91	0	0	1	1	1	0	0	0	1	0	1	0	1	0	0	0	0	1
Thiobaca	1	2	0	0	0	0	0	0	0	0	0	0	0	0	0	0	0	0	0	0
Thiobacillus	2	67	0	0	0	0	0	0	0	0	1	0	1	0	0	0	0	0	0	0
Thiocapsa	1	2	0	0	0	0	0	0	0	0	0	0	0	0	0	0	0	0	0	0
Thiocystis	1	11	0	0	0	1	0	0	0	0	0	0	1	0	0	0	0	0	0	0
Thioflavicoccus	1	13	0	0	0	0	0	0	0	0	0	0	1	0	0	0	0	0	0	0
Thiomicrospira	2	50	0	0	0	0	1	0	0	0	0	0	0	0	0	0	0	0	0	0
Thiomonas	2	85	0	0	1	1	0	1	0	0	1	0	1	0	0	0	0	0	0	1
Thiorhodococcus	1	2	0	0	0	0	0	0	0	0	0	0	0	0	0	0	0	0	0	0
Thiothrix	1	2	0	0	0	0	0	0	0	0	0	0	0	0	0	0	0	0	0	0
Tistrella	1	42	0	0	1	1	0	1	0	0	0	1	1	1	1	1	1	0	1	1
Tolumonas	2	100	1	0	0	1	1	0	1	0	0	0	1	0	0	0	0	0	0	1
Transposon	1	2	1	0	0	0	0	0	0	0	0	0	0	0	0	0	0	0	0	0
Treponema	1	63	0	0	0	0	0	1	1	0	0	0	1	0	0	0	0	0	0	1
Trichodesmium	2	71	0	0	1	1	0	0	0	0	0	0	0	0	0	0	0	0	0	0
Trichormus	1	12	0	0	1	0	0	0	0	0	0	0	0	0	0	0	0	0	0	0
Tropheryma	2	22	0	0	0	0	0	0	0	0	0	0	0	0	0	0	0	0	0	0
Truepera	2	104	0	0	1	0	0	1	1	0	0	0	1	0	0	0	0	0	0	1
Tsukamurella	2	123	0	0	1	1	1	1	1	0	0	0	1	0	0	0	1	0	0	1
Turneriella	1	16	0	0	1	0	0	0	1	0	0	0	1	0	0	0	0	0	0	0
Uliginosibacterium	1	2	0	0	0	0	0	0	0	0	0	0	0	0	0	0	0	0	0	0
Ureaplasma	1	24	0	0	0	1	0	0	1	0	0	0	0	0	0	0	0	0	0	0
Ureibacillus	1	2	0	0	0	0	0	0	0	0	0	0	0	0	0	0	0	0	0	0
Variovorax	3	187	0	0	1	1	1	1	0	0	1	1	1	1	1	1	0	0	0	1
Veillonella	1	54	0	0	0	0	0	1	1	0	0	0	0	0	0	0	0	0	0	1
Verminephrobacter	3	188	0	0	1	1	1	1	0	0	1	0	0	0	1	0	0	0	0	1
Verrucosispora	3	126	0	0	1	1	0	0	1	0	0	0	0	1	0	0	0	0	1	1
Vibrio	2	195	0	0	0	1	0	0	1	0	1	0	1	0	0	0	0	0	0	1
Vibrionales	1	46	0	0	0	0	0	0	0	0	0	0	0	0	0	0	0	0	0	0
Vitreoscilla	1	1	0	0	0	0	0	0	0	0	0	0	0	0	0	0	0	0	0	0
Vulcanisaeta	1	14	0	0	0	0	0	0	1	0	0	0	1	0	0	0	0	0	0	0
Waddlia	2	68	0	0	0	0	0	0	0	0	0	0	1	0	0	0	0	0	0	0
Wautersia	1	2	0	0	0	0	0	0	0	0	0	0	0	0	0	0	0	0	0	0
Weeksella	2	65	0	0	0	0	0	0	1	0	0	0	0	1	0	0	0	0	0	0

Genus	Cluster Number	Protein Counts	K01195	K01253	K01426	K01428	K01457	K01464	K01489	K01500	K01501	K01502	K01512	K01555	K01560	K01561	K01563	K01564	K01576	K01607
**Weissella**	1	14	0	0	0	0	0	0	0	0	0	0	1	0	0	0	0	0	0	1
**Wenxinia**	1	2	0	0	0	0	0	0	0	0	0	0	0	0	0	0	0	0	0	0
**Wigglesworthia**	1	29	0	0	0	0	0	0	0	0	0	0	0	0	0	0	0	0	0	0
**Wolbachia**	1	32	0	0	0	0	0	0	0	0	0	0	0	0	0	0	0	0	0	0
**Wolinella**	2	51	0	0	0	0	1	0	0	0	0	0	0	0	0	0	0	1	0	0
**Xanthobacter**	3	176	0	0	1	1	1	0	1	0	1	0	1	1	1	1	0	0	0	1
**Xanthobacteraceae**	1	4	0	0	1	0	0	0	0	0	0	0	0	0	0	0	0	0	0	0
**Xanthomonadaceae**	1	39	0	0	0	0	0	0	0	0	0	0	0	0	0	0	0	0	0	0
**Xanthomonas**	2	162	0	0	1	0	1	0	0	0	0	0	1	0	1	0	1	0	0	1
**Xenorhabdus**	2	127	0	0	1	0	0	0	1	0	0	1	1	0	0	0	0	0	0	0
**Xylanimonas**	2	66	0	0	1	0	0	0	1	0	0	0	0	0	0	0	0	0	1	0
**Xylella**	2	52	0	0	0	0	0	0	0	0	0	0	0	0	0	0	1	0	0	0
**Yersinia**	2	145	0	0	0	1	0	0	1	0	0	0	1	0	0	0	0	0	0	1
**Yokenella**	2	61	0	0	0	0	0	0	0	0	0	0	0	0	0	0	0	0	0	0
**Zobellia**	1	28	0	0	0	0	0	0	1	0	0	0	0	1	1	0	0	0	0	0
**Zunongwangia**	2	98	1	0	1	0	0	0	1	0	0	0	0	1	1	0	0	0	0	1
**Zymomonas**	2	67	0	0	0	0	0	0	1	0	1	0	0	0	0	0	0	0	0	1
**Zymophilus**	1	2	0	0	0	0	0	0	0	0	0	0	0	0	0	0	0	0	0	0

Genus	Cluster Number	Protein Counts	K01612	K01615	K01617	K01666	K01692	K01721	K01726	K01781	K01800	K01821	K01822	K01856	K01857	K01860	K01913	K01951	K02554	K03268
**Acaricomes**	1	2	0	0	0	0	0	0	0	0	0	0	0	0	0	0	0	0	0	0
**Acaryochloris**	2	88	0	0	0	0	0	1	0	0	0	0	0	0	0	0	0	1	0	0
**Acetivibrio**	1	2	0	0	0	0	0	0	0	0	0	0	0	0	0	0	0	0	0	0
**Acetobacter**	2	85	0	0	0	0	0	0	0	0	0	0	0	0	0	0	0	1	0	0
**Acetobacteraceae**	1	2	0	0	0	0	0	0	0	0	0	0	0	0	0	0	0	0	0	0
**Acetobacterium**	1	8	0	0	0	0	0	0	0	0	0	0	0	0	0	0	0	1	0	0
**Acetohalobium**	2	79	0	0	0	0	0	0	0	0	0	1	0	0	0	0	0	1	0	0
**Acholeplasma**	2	38	0	0	0	0	0	0	0	0	0	0	0	0	0	0	0	0	0	0
**Achromobacter**	2	192	0	0	0	1	1	0	0	0	1	1	0	1	0	0	0	1	1	0
**Acidaminococcus**	1	65	0	1	0	0	0	0	0	0	0	0	0	0	0	0	0	1	0	0
**Acidianus**	1	9	0	0	0	0	0	0	0	0	0	0	0	0	0	0	0	1	0	0
**Acidilobus**	1	8	0	0	0	0	0	0	0	0	0	0	0	0	0	0	0	1	0	0
**Acidimicrobium**	2	58	0	0	0	0	1	0	0	0	0	0	0	0	0	0	0	1	0	0
**Acidiphilium**	3	144	0	0	0	0	1	1	0	0	0	1	0	0	1	0	0	1	0	0
**Acidithiobacillus**	2	70	0	0	0	0	0	0	0	0	0	0	0	0	0	0	0	1	0	0
**Acidobacterium**	2	91	0	0	0	0	0	0	0	0	0	0	0	0	0	0	0	1	0	0
**Acidocella**	1	2	0	0	0	0	0	0	0	0	0	0	0	0	0	0	0	0	0	0
**Acidothermus**	2	68	0	0	0	0	0	0	0	0	0	0	0	0	0	0	0	1	0	0
**Acidovorax**	3	217	0	0	1	1	1	1	0	0	1	1	1	1	0	1	0	1	1	0
**Aciduliprofundum**	1	11	0	0	0	0	0	0	0	0	0	0	0	0	0	0	0	1	0	0
**Acinetobacter**	3	188	0	0	0	0	1	1	0	0	1	1	0	1	1	0	0	1	0	1
**Actinobacillus**	2	112	0	0	0	0	0	0	0	0	0	1	0	0	0	0	0	1	0	0
**Actinomadura**	1	1	0	0	0	0	0	0	0	0	0	0	0	0	0	0	0	0	0	0
**Actinoplanes**	1	52	0	0	0	1	1	0	0	0	0	1	0	0	1	0	0	1	1	0
**Actinopolyspora**	1	2	0	0	0	0	0	0	0	0	0	0	0	0	0	0	0	0	0	0
**Actinosynnema**	2	122	0	0	0	0	1	0	0	0	0	1	0	0	1	0	0	1	0	0
**Advenella**	1	30	0	0	0	0	1	0	0	0	0	0	0	1	1	0	0	1	0	0
**Aequorivita**	1	12	0	0	0	0	0	0	0	0	0	0	0	0	0	0	0	1	0	0
**Aerococcus**	2	57	0	0	0	0	0	0	0	0	0	1	0	0	0	0	0	1	0	0
**Aeromonas**	2	127	0	0	0	0	0	0	0	0	0	0	0	0	0	0	0	1	0	0
**Aeropyrum**	1	13	0	0	0	0	0	0	0	0	0	0	0	0	0	0	0	1	0	0
**Afipia**	1	54	0	0	0	0	0	0	0	0	0	0	0	0	0	0	0	1	0	0
**Aggregatibacter**	1	81	0	0	0	0	0	0	0	0	0	0	0	0	0	0	0	1	0	0
**Agrobacterium**	2	242	0	0	1	1	1	1	0	1	1	1	0	1	1	0	0	1	0	0
**Ahrensia**	1	2	0	0	0	0	0	0	0	0	0	0	0	0	0	0	0	0	0	0
**Akkermansia**	2	47	0	0	0	0	0	0	0	0	0	0	0	0	0	0	0	1	0	0
**Albidiferax**	3	144	0	0	0	1	1	0	0	0	0	1	0	0	1	0	0	1	0	0
**Alcaligenes**	1	21	0	0	0	0	0	0	0	0	0	0	0	0	0	1	0	0	1	0
**Alcanivorax**	2	146	0	0	0	0	1	0	0	0	0	1	0	0	0	0	0	1	0	0
**Alicycliphilus**	3	152	0	0	1	1	1	0	0	0	1	1	0	1	0	0	0	1	1	0
**Alicyclobacillus**	2	91	0	0	1	1	0	0	0	0	0	1	0	0	0	0	0	1	0	0
**Aliivibrio**	2	118	0	0	0	0	0	0	1	0	0	0	0	0	0	0	0	1	0	0
**Alishewanella**	1	1	0	0	0	0	0	0	0	0	0	0	0	0	0	0	0	0	0	0
**Alistipes**	1	52	0	0	0	0	0	0	0	0	0	0	0	0	0	0	0	1	0	0
**Alkalilimnicola**	2	97	0	0	0	1	0	0	0	0	0	0	0	0	0	0	0	1	1	0
**Alkaliphilus**	2	62	0	0	0	0	0	0	0	0	0	0	0	0	0	0	0	1	0	0
**Allochromatium**	2	84	0	0	0	1	0	0	0	0	0	0	0	0	0	0	0	1	0	0
**Alphaproteobacteria**	1	3	0	0	0	0	0	0	0	0	0	0	0	0	0	0	0	0	0	0
**Alteromonadales**	1	12	0	0	0	0	0	0	0	0	0	0	0	0	0	0	0	0	0	0
**Alteromonas**	1	37	0	0	0	0	1	0	0	0	1	0	0	1	0	0	0	1	1	0
**Aminobacter**	1	4	0	0	0	0	0	0	0	0	0	0	0	0	0	0	0	0	0	0
**Aminobacterium**	2	59	0	0	0	1	0	0	0	0	0	1	0	0	0	0	0	1	0	0

Genus	Cluster Number	Protein Counts	K01612	K01615	K01617	K01666	K01692	K01721	K01726	K01781	K01800	K01821	K01822	K01856	K01857	K01860	K01913	K01951	K02554	K03268
**Ammonifex**	2	50	0	0	0	0	0	0	0	0	0	0	0	0	0	0	0	1	0	0
**Amphibacillus**	1	13	0	0	0	0	0	0	0	0	0	1	0	0	0	0	0	1	0	0
**Amphritea**	1	2	0	0	0	0	0	0	0	0	0	0	0	0	0	0	0	0	0	0
**Amycolatopsis**	2	201	0	0	1	1	1	0	0	0	0	1	0	1	1	0	0	1	1	0
**Amycolicicoccus**	1	43	0	0	0	1	1	0	0	0	0	1	1	0	0	0	1	1	0	0
**Anabaena**	2	92	0	0	0	0	0	0	0	0	0	1	0	1	0	0	0	1	0	0
**Anaerobaculum**	1	11	0	0	0	0	0	0	0	0	0	0	0	0	0	0	0	1	0	0
**Anaerococcus**	2	46	0	0	0	0	0	0	0	0	0	0	0	0	0	0	0	1	0	0
**Anaerolinea**	2	66	0	0	0	0	0	0	0	0	0	0	0	0	0	0	0	1	0	0
**Anaeromyxobacter**	3	117	0	0	0	0	0	0	0	0	0	1	0	0	0	0	0	1	0	0
**Anaerostipes**	1	44	0	0	0	0	0	0	0	0	0	0	0	0	0	0	0	0	0	0
**Anaerotruncus**	1	2	0	0	0	0	0	0	0	0	0	0	0	0	0	0	0	0	0	0
**Anaplasma**	1	35	0	0	0	0	0	0	0	0	0	0	0	0	0	0	0	1	0	0
**Ancylobacter**	1	3	0	0	0	0	0	0	0	0	0	0	0	0	0	0	0	0	0	0
**Aneurinibacillus**	1	1	0	0	0	0	0	0	0	0	0	0	0	0	0	0	0	0	0	0
**Angomonas**	1	1	0	0	0	0	0	0	0	0	0	0	0	0	0	0	0	0	0	0
**Anoxybacillus**	2	72	0	0	0	0	0	0	0	0	0	1	0	0	0	0	0	1	0	0
**Aquifex**	2	45	0	0	0	0	0	0	0	0	0	0	0	0	0	0	0	1	0	0
**Aquimarina**	1	2	0	0	0	0	0	0	0	0	0	0	0	0	0	0	0	0	0	0
**Arcanobacterium**	2	45	0	0	0	0	0	0	0	0	0	0	0	0	0	0	0	1	0	0
**Archaeoglobus**	1	14	0	0	0	0	0	0	0	0	0	1	0	0	0	0	0	1	0	0
**Arcobacter**	2	101	0	0	1	1	0	0	0	0	0	1	0	0	0	0	0	1	1	0
**Aromatoleum**	3	133	0	0	0	0	1	0	0	0	0	0	0	0	0	0	0	1	0	0
**Arsenophonus**	1	4	0	0	0	0	0	0	0	0	0	0	0	0	0	0	0	0	0	0
**Arthrobacter**	2	206	0	0	0	1	1	1	0	0	0	1	0	0	1	0	0	1	0	0
**Arthrospira**	2	67	0	0	0	1	0	0	0	0	0	0	0	0	0	0	0	1	0	0
**Aster**	1	10	0	0	0	0	0	0	0	0	0	0	0	0	0	0	0	0	0	0
**Asticcacaulis**	2	103	0	0	0	0	1	0	0	0	0	0	0	0	0	0	0	1	1	0
**Atopobium**	1	28	0	0	0	0	0	0	0	0	0	0	0	0	0	0	0	1	0	0
**Aurantimonas**	1	2	0	0	0	0	0	0	0	0	0	0	0	0	0	0	0	0	0	0
**Avibacterium**	1	17	0	0	0	0	0	0	0	0	0	0	0	0	0	0	0	1	0	0
**Azoarcus**	3	172	0	0	1	1	1	0	0	0	1	1	1	0	1	0	0	1	1	0
**Azorhizobium**	3	149	0	0	0	0	1	1	0	0	0	1	0	1	1	0	0	1	0	0
**Azorhizophilus**	1	4	0	0	0	0	0	0	0	0	0	0	0	0	0	0	0	0	0	0
**Azospira**	1	19	0	0	0	0	1	0	0	0	0	1	0	0	0	0	0	1	0	0
**Azospirillum**	2	198	0	0	0	0	1	1	0	0	1	1	0	1	1	0	0	1	1	0
**Azotobacter**	3	183	0	0	1	1	1	0	0	0	1	1	0	0	1	0	0	1	1	0
**Bacillales**	1	9	0	0	0	0	0	0	0	0	0	1	0	0	0	0	0	0	0	0
**Bacilli**	1	3	0	0	0	0	0	0	0	0	0	0	0	0	0	0	0	0	0	0
**Bacillus**	2	237	0	0	1	1	0	1	1	0	0	1	0	1	1	0	1	1	1	0
**Bacteria**	2	86	0	0	0	0	1	0	0	0	1	1	0	0	0	0	0	1	0	0
**Bacteriovorax**	2	98	0	0	0	0	1	0	0	0	0	0	0	0	0	0	0	1	0	0
**Bacteroidales**	2	59	0	0	0	0	0	0	0	0	0	0	0	0	0	0	0	1	0	0
**Bacteroides**	2	96	0	0	0	1	0	0	0	0	0	0	0	0	0	0	0	1	0	0
**Bacteroidetes**	1	2	0	0	0	0	0	0	0	0	0	0	0	0	0	0	0	0	0	0
**Bartonella**	2	50	0	0	0	1	0	0	0	0	0	1	0	0	0	0	0	1	0	0
**Baumannia**	2	24	0	0	0	0	0	0	0	0	0	0	0	0	0	0	0	1	0	0
**Bdellovibrio**	2	87	0	0	0	0	0	0	0	0	1	1	0	0	0	0	0	1	0	0
**Beijerinckia**	3	123	0	0	0	0	1	0	0	0	1	1	0	0	0	0	0	1	0	0
**Belliella**	1	14	0	0	0	0	0	0	0	0	0	0	0	0	0	0	0	1	0	0
**Beutenbergia**	2	82	0	0	0	0	0	0	0	0	0	0	0	0	0	0	0	1	0	0
**Bifidobacterium**	2	61	0	0	0	0	0	0	0	0	0	0	0	0	0	0	0	1	0	0

Genus	Cluster Number	Protein Counts	K01612	K01615	K01617	K01666	K01692	K01721	K01726	K01781	K01800	K01821	K01822	K01856	K01857	K01860	K01913	K01951	K02554	K03268
**Bizionia**	1	1	0	0	0	0	0	0	0	0	0	0	0	0	0	0	0	0	0	0
**Blastococcus**	1	39	0	0	0	0	1	0	0	0	0	0	0	0	0	0	0	1	0	0
**Blattabacterium**	1	23	0	0	0	0	0	0	0	0	0	0	0	0	0	0	0	1	0	0
**Blautia**	1	22	0	0	0	0	0	0	0	0	0	0	0	0	0	0	0	0	0	0
**Bordetella**	3	227	0	0	0	1	1	1	1	0	1	1	0	1	1	1	1	1	1	1
**Borrelia**	1	32	0	0	0	0	0	0	0	0	0	0	0	0	0	0	0	1	0	0
**Brachybacterium**	1	18	0	0	0	0	0	0	0	0	0	0	0	0	0	0	0	1	0	0
**Brachymonas**	1	4	0	0	0	0	0	0	0	0	0	0	0	0	0	0	0	1	0	0
**Brachyspira**	2	79	0	0	0	1	0	0	0	0	0	0	0	0	0	0	0	1	0	0
**Bradyrhizobiaceae**	1	20	0	0	0	0	0	0	0	0	0	0	0	0	0	0	0	1	0	0
**Bradyrhizobium**	3	254	0	0	1	0	1	1	1	1	1	1	0	1	1	0	1	1	0	0
**Brenneria**	1	20	0	0	0	0	0	0	0	0	0	0	0	0	0	0	0	1	0	0
**Brevibacillus**	2	122	0	0	1	1	1	0	0	0	0	1	0	0	0	0	0	1	0	0
**Brevibacterium**	1	4	0	0	0	0	0	1	0	0	0	0	0	0	0	0	0	0	0	0
**Brevundimonas**	2	105	0	0	0	0	1	0	0	0	1	0	0	0	0	0	0	1	1	0
**Brucella**	3	138	0	0	0	0	1	0	1	0	0	0	0	0	1	0	0	1	0	0
**Buchnera**	1	26	0	0	0	0	0	0	0	0	0	0	0	0	0	0	0	0	0	0
**Burkholderia**	3	424	0	0	1	1	1	1	1	1	1	1	0	1	1	1	1	1	1	1
**Burkholderiaceae**	3	143	0	0	1	1	1	0	1	0	0	1	1	1	1	1	1	1	1	0
**Burkholderiales**	1	24	0	0	0	0	1	0	0	0	0	0	0	0	0	0	0	0	1	0
**Buttiauxella**	1	2	0	0	0	0	0	1	0	0	0	0	0	0	0	0	0	0	0	0
**Butyrivibrio**	2	67	0	0	0	1	0	0	0	0	0	1	0	0	0	0	0	1	0	0
**Caldanaerobacter**	2	63	0	0	0	0	0	0	0	0	0	0	0	0	0	0	0	1	0	0
**Caldicellulosiruptor**	2	75	0	0	0	0	0	0	0	0	0	0	0	0	0	0	0	1	0	0
**Caldilinea**	1	23	0	0	0	1	1	0	0	0	0	0	0	0	0	0	0	1	1	0
**Caldimonas**	1	6	0	0	0	0	0	0	0	0	0	0	0	0	0	0	0	0	0	0
**Caldisericum**	1	10	0	0	0	0	0	0	0	0	0	0	0	0	0	0	0	0	0	0
**Caldisphaera**	1	7	0	0	0	0	0	0	0	0	0	0	0	0	0	0	0	1	0	0
**Calditerrivibrio**	2	74	0	0	0	0	0	0	0	0	0	1	0	0	0	0	0	1	0	0
**Caldivirga**	1	13	0	0	0	0	0	0	0	0	0	0	0	0	0	0	0	1	0	0
**Calothrix**	1	43	0	0	0	0	0	0	0	0	0	1	0	0	0	0	0	1	0	0
**Calyptogena**	2	34	0	0	0	0	0	0	0	0	0	0	0	0	0	0	0	1	0	0
**Campylobacter**	2	80	0	0	0	1	0	0	0	0	0	1	0	0	0	0	0	1	0	0
**Candidatus**	2	290	0	0	0	1	1	1	1	1	0	1	0	1	1	0	0	1	0	0
**Capnocytophaga**	1	50	0	0	0	0	0	0	0	0	0	0	0	0	0	0	0	1	0	0
**Carbophilus**	1	2	0	0	0	0	0	0	0	0	0	0	0	0	0	0	0	0	0	0
**Carboxydibrachium**	1	14	0	0	0	0	0	0	0	0	0	0	0	0	0	0	0	1	0	0
**Carboxydothermus**	2	68	0	0	0	1	0	0	0	0	0	1	0	0	0	0	0	1	0	0
**Carica**	1	2	0	0	0	0	0	0	0	0	0	0	0	0	0	0	0	0	0	0
**Carnobacterium**	2	71	0	0	0	0	0	0	0	0	0	1	0	0	0	0	0	1	0	0
**Catenibacterium**	1	2	0	0	0	0	0	0	0	0	0	0	0	0	0	0	0	0	0	0
**Catenovulum**	1	2	0	0	0	0	0	0	0	0	0	0	0	0	0	0	0	0	0	0
**Catenulispora**	3	159	0	0	0	0	1	0	0	0	0	1	0	0	1	0	0	1	0	0
**Caulobacter**	3	168	0	0	0	0	1	0	0	0	1	1	0	0	1	0	0	1	1	0
**Cedecea**	1	45	0	0	0	0	0	0	0	0	0	0	0	0	0	0	0	1	0	0
**Cellulomonas**	2	96	0	0	0	0	0	0	0	0	0	0	0	0	0	0	0	1	0	0
**Cellulophaga**	2	105	0	0	0	0	0	0	0	0	0	0	0	0	0	0	0	1	0	0
**Cellulosilyticum**	1	14	0	0	0	1	0	0	0	0	0	0	0	0	0	0	0	1	0	0
**Cellvibrio**	2	85	0	0	0	0	0	0	0	0	0	0	0	0	0	0	0	1	0	0
**Cenarchaeum**	1	7	0	0	0	0	0	0	0	0	0	1	0	0	0	0	0	1	0	0
**Chamaesiphon**	1	13	0	0	0	0	0	1	0	0	0	1	0	0	0	0	0	1	0	0
**Chelativorans**	3	136	0	0	0	0	1	0	0	0	1	0	0	0	1	0	0	1	0	0

Genus	Cluster Number	Protein Counts	K01612	K01615	K01617	K01666	K01692	K01721	K01726	K01781	K01800	K01821	K01822	K01856	K01857	K01860	K01913	K01951	K02554	K03268
Chelatococcus	1	2	0	0	0	0	0	0	0	0	0	0	0	0	0	0	0	0	0	0
Chitinophaga	2	98	0	0	0	0	1	0	0	0	0	1	0	0	0	0	0	1	0	0
Chlamydia	1	36	0	0	0	0	0	0	0	0	0	0	0	0	0	0	0	1	0	0
Chlamydomonas	1	3	0	0	0	0	0	0	0	0	0	0	0	0	0	0	0	0	0	0
Chlamydophila	1	31	0	0	0	0	0	0	0	0	0	0	0	0	0	0	0	1	0	0
Chlorobaculum	2	61	0	0	0	0	0	0	0	0	0	0	0	0	0	0	0	1	0	0
Chlorobium	2	89	0	0	0	0	0	0	0	0	0	0	0	0	0	0	0	1	0	0
Chlorobium/Pelodictyon	1	2	0	0	0	0	0	0	0	0	0	0	0	0	0	0	0	0	0	0
Chloroflexus	2	105	0	0	0	1	1	0	0	0	0	1	0	0	0	0	0	1	0	0
Chlorogloeopsis	1	9	0	0	0	0	0	0	0	0	0	0	0	0	0	0	0	0	0	0
Chloroherpeton	2	59	0	0	0	1	0	0	0	0	0	0	0	0	0	0	0	1	0	0
Chromobacterium	2	117	0	0	0	0	0	0	0	0	1	0	0	0	0	0	0	1	0	0
Chromohalobacter	2	145	0	0	0	0	0	0	0	0	0	0	0	0	1	0	0	1	0	0
Chroococcidiopsis	1	24	0	0	0	0	0	0	0	0	0	0	0	0	0	0	0	1	0	0
Chryseobacterium	2	104	0	0	0	0	0	0	0	0	0	0	0	1	0	0	0	1	0	0
Citreicella	1	13	0	0	0	0	0	0	0	0	0	0	0	0	0	0	0	0	0	0
Citrobacter	2	190	0	0	0	1	0	0	0	0	0	0	0	0	1	0	0	1	1	0
Clavibacter	2	83	0	0	0	0	0	0	0	0	0	0	0	0	0	0	0	1	0	0
Clonorchis	1	1	0	0	0	0	0	0	0	0	0	0	0	0	0	0	0	0	0	0
Clostridiales	2	77	0	0	0	0	0	0	0	0	0	0	0	0	0	0	0	1	0	0
Clostridium	2	191	0	0	0	1	0	0	1	0	0	1	0	0	0	0	0	1	0	0
Cohnella	1	2	0	0	0	0	0	0	0	0	0	0	0	0	0	0	0	0	0	0
Coleofasciculus	1	4	0	0	0	0	0	0	0	0	0	0	0	0	0	0	0	0	0	0
Collimonas	1	35	0	0	0	0	1	0	0	0	0	1	0	0	1	0	0	1	0	0
Colwellia	2	130	0	0	0	0	0	0	0	0	1	1	0	0	0	0	0	1	0	0
Comamonas	2	196	0	0	1	1	1	1	0	0	1	1	1	0	0	0	0	1	1	0
Conexibacter	3	117	0	0	0	1	1	0	0	0	0	0	0	1	0	0	0	1	0	0
Coprobacillus	1	2	0	0	0	0	0	0	0	0	0	0	0	0	0	0	0	0	0	0
Coprococcus	2	76	0	0	0	1	0	0	0	0	0	0	0	0	0	0	0	1	0	0
Coprothermobacter	2	45	0	0	0	0	0	0	0	0	0	0	0	0	0	0	0	1	0	0
Coraliomargarita	2	68	0	0	0	0	0	0	0	0	0	0	0	0	0	0	0	1	0	0
Corallococcus	1	29	0	0	0	0	1	0	0	0	0	1	0	0	0	0	0	1	0	0
Coriobacterium	2	36	0	0	0	0	0	0	0	0	0	0	0	0	0	0	0	1	0	0
Corynebacterineae	1	5	0	0	0	0	1	0	0	0	0	0	0	0	0	0	0	0	0	0
Corynebacterium	2	175	0	0	0	1	1	0	0	0	0	1	0	1	1	0	0	1	1	0
Coxiella	2	54	0	0	0	0	0	0	0	0	0	0	0	0	0	0	0	1	0	0
Crinalium	1	21	0	0	0	0	0	0	0	0	0	0	0	0	0	0	0	1	0	0
Croceibacter	2	80	0	0	0	0	0	0	0	0	0	0	0	0	0	0	0	1	0	0
Crocosphaera	1	6	0	0	0	0	0	0	0	0	0	0	0	0	0	0	0	1	0	0
Cronobacter	2	120	0	0	0	0	0	0	0	0	0	1	0	0	0	0	0	1	0	0
Cryptobacterium	2	39	0	0	0	0	0	0	0	0	0	0	0	0	0	0	0	1	0	0
Cucumis	1	9	0	0	0	0	0	0	0	0	0	0	0	0	0	0	0	0	0	0
Cupriavidus	3	317	1	0	1	1	1	0	1	0	1	1	1	1	1	1	1	1	1	0
Curtobacterium	1	5	0	0	0	0	0	1	0	0	0	0	0	0	0	0	0	1	0	0
Curvibacter	1	27	0	0	0	0	0	0	0	0	0	0	0	0	1	0	0	1	0	0
Cyanobacterium	1	9	0	0	0	0	0	0	0	0	0	0	0	0	0	0	0	1	0	0
Cyanobium	1	10	0	0	0	0	0	0	0	0	0	0	0	0	0	0	0	1	0	0
Cyanothece	1	34	0	0	0	0	0	0	0	0	0	1	1	0	0	0	0	1	0	0
Cyclobacterium	1	19	0	0	0	0	0	0	0	0	0	0	0	0	0	0	0	1	0	0
Cycloclasticus	1	21	0	0	0	1	1	0	0	0	0	1	0	0	0	0	0	1	1	0
Cylindrospermopsis	1	4	0	0	0	0	0	0	0	0	0	0	0	0	0	0	0	0	0	0
Cylindrospermum	1	30	0	0	0	0	0	0	0	0	0	0	0	0	0	0	0	1	0	0

Genus	Cluster Number	Protein Counts	K01612	K01615	K01617	K01666	K01692	K01721	K01726	K01781	K01800	K01821	K01822	K01856	K01857	K01860	K01913	K01951	K02554	K03268
Cytophaga	2	69	0	0	0	0	0	0	0	0	0	1	0	0	0	0	0	1	0	0
Dactylococcopsis	1	8	0	0	0	0	0	0	0	0	0	0	0	0	0	0	0	1	0	0
Dechloromonas	1	40	0	0	1	1	1	0	0	0	0	1	0	0	0	0	0	1	1	0
Dechlorosoma	1	19	0	0	0	0	1	0	0	0	0	1	0	0	0	0	0	1	0	0
Deferribacter	2	73	0	0	0	0	0	0	0	0	0	1	0	0	0	0	0	1	0	0
Dehalobacter	1	9	0	0	0	1	0	0	0	0	0	1	0	0	0	0	0	1	0	0
Dehalococcoides	2	41	0	0	0	0	0	0	0	0	0	0	0	0	0	0	0	1	0	0
Dehalogenimonas	1	37	0	0	0	0	0	0	0	0	0	0	0	0	0	0	0	1	0	0
Deinococcus	2	140	0	0	0	0	1	0	0	0	0	0	0	0	1	0	0	1	0	0
Delftia	3	177	0	0	1	1	1	0	0	0	1	1	0	1	1	0	0	1	1	0
Denitrovibrio	2	67	0	0	0	0	0	0	0	0	0	1	0	0	0	0	0	1	0	0
Desulfarculus	2	92	0	0	0	0	1	0	0	0	0	0	0	0	0	0	0	1	0	0
Desulfatibacillum	3	84	0	0	0	0	1	0	0	0	0	0	0	0	0	0	0	1	0	0
Desulfitobacterium	2	102	0	0	0	1	0	0	0	0	0	1	0	0	0	0	0	1	0	0
Desulfobacca	2	65	0	0	0	0	0	0	0	0	0	0	0	0	0	0	0	1	0	0
Desulfobacterium	2	113	0	0	0	0	1	0	0	0	0	1	0	0	0	0	0	1	0	0
Desulfobacula	1	21	0	0	0	0	0	0	0	0	0	0	0	0	0	0	0	1	0	0
Desulfobulbus	2	88	0	0	0	0	0	0	0	0	0	1	0	0	0	0	0	1	0	0
Desulfocapsa	1	9	0	0	0	0	0	0	0	0	0	1	0	0	0	0	0	1	0	0
Desulfococcus	2	80	0	0	0	0	1	0	0	0	0	0	0	0	0	0	0	1	0	0
Desulfohalobium	2	52	0	0	0	0	0	0	0	0	0	0	0	0	0	0	0	1	0	0
Desulfomicrobium	2	65	0	0	0	0	0	0	0	0	0	1	0	0	0	0	0	1	0	0
Desulfomonile	1	25	0	0	0	0	1	0	0	0	0	1	0	0	0	0	0	1	0	0
Desulforhabdus	1	2	0	0	0	0	0	0	0	0	0	0	0	0	0	0	0	0	0	0
Desulfosporosinus	1	30	0	0	0	1	1	0	0	0	0	1	0	0	0	0	0	1	1	0
Desulfotalea	2	66	0	0	0	0	0	0	0	0	0	0	0	0	0	0	0	1	0	0
Desulfotomaculum	2	98	0	0	0	1	0	0	0	0	0	1	0	0	0	0	0	1	1	0
Desulfovibrio	2	144	0	0	0	1	0	0	0	0	0	1	0	0	0	0	0	1	0	0
Desulfurispirillum	2	78	0	0	0	0	0	0	0	0	0	1	0	0	0	0	0	1	0	0
Desulfurivibrio	2	71	0	0	0	0	0	0	0	0	0	1	0	0	0	0	0	1	0	0
Desulfurobacterium	2	53	0	0	0	0	0	0	0	0	0	0	0	0	0	0	0	1	0	0
Desulfurococcus	1	11	0	0	0	0	0	0	0	0	0	0	0	0	0	0	0	1	0	0
Diaphorobacter	1	16	0	0	1	1	0	0	0	0	0	0	0	0	0	0	0	0	1	0
Dichelobacter	2	45	0	0	0	0	0	0	0	0	0	0	0	0	0	0	0	1	0	0
Dickeya	2	155	0	0	0	0	0	0	0	0	0	1	0	0	1	0	0	1	0	0
Dictyoglomus	2	62	0	0	0	0	0	0	0	0	0	0	0	0	0	0	0	1	0	0
Dinoroseobacter	3	148	0	0	0	0	1	1	0	0	0	0	0	0	1	0	0	1	0	0
Dokdonia	1	3	0	0	0	0	0	0	0	0	0	0	0	0	0	0	0	0	0	0
Dorea	1	7	0	0	0	0	0	0	0	0	0	0	0	0	0	0	0	0	0	0
Dyadobacter	2	83	0	0	0	0	0	0	0	0	0	0	0	0	0	0	0	1	0	0
Echinicola	1	20	0	0	0	0	0	0	0	0	0	0	0	0	0	0	0	1	0	0
Edwardsiella	2	126	0	0	0	0	0	0	0	0	0	0	0	0	0	0	0	1	0	0
Eggerthella	1	51	0	0	0	0	0	0	0	0	0	0	0	0	0	0	0	1	0	0
Ehrlichia	2	37	0	0	0	0	0	0	0	0	0	0	0	0	0	0	0	1	0	0
Eikenella	1	4	0	0	0	0	0	0	0	0	0	0	0	0	0	0	0	1	0	0
Elusimicrobium	2	44	0	0	0	0	0	0	0	0	0	0	0	0	0	0	0	1	0	0
Emticicia	1	21	0	0	0	0	1	0	0	0	0	0	0	0	1	0	0	1	0	0
Enhydrobacter	1	1	0	0	0	0	0	0	0	0	0	0	0	0	0	0	0	0	0	0
Ensifer	1	2	0	0	0	0	0	0	0	0	0	0	0	0	0	0	0	0	0	0
Enterobacter	2	202	0	0	0	0	1	0	0	0	0	1	0	1	1	0	0	1	0	0
Enterobacteriaceae	2	211	0	0	0	1	1	0	0	0	0	1	0	1	1	0	0	1	1	0
Enterococcus	1	187	0	0	0	1	1	0	0	0	0	1	0	1	0	0	0	1	1	0

Genus	Cluster Number	Protein Counts	K01612	K01615	K01617	K01666	K01692	K01721	K01726	K01781	K01800	K01821	K01822	K01856	K01857	K01860	K01913	K01951	K02554	K03268
Enterovibrio	1	15	0	0	0	0	0	0	0	0	0	0	0	0	0	0	0	1	0	0
Erwinia	2	151	0	0	0	1	0	0	0	0	0	1	0	0	0	0	0	1	0	0
Erysipelothrix	1	9	0	0	0	0	0	0	0	0	0	0	0	0	0	0	0	1	0	0
Erysipelotrichaceae	1	4	0	0	0	0	0	0	0	0	0	0	0	0	0	0	0	0	0	0
Erythrobacter	2	80	0	0	0	0	1	0	0	0	1	0	0	0	0	0	0	1	0	0
Escherichia	2	242	0	0	0	1	1	0	0	0	0	1	0	1	1	0	0	1	1	0
Ethanoligenens	2	69	0	0	0	0	0	0	0	0	0	0	0	0	0	0	0	1	0	0
Eubacteriaceae	1	2	0	0	0	0	0	0	0	0	0	0	0	0	0	0	0	0	0	0
Eubacterium	2	103	0	0	0	1	0	0	0	0	0	0	0	0	0	0	0	1	0	0
Eudoraea	1	4	0	0	0	0	0	0	0	0	0	0	0	0	0	0	0	0	0	0
Euryarchaeota	1	1	0	0	0	0	0	0	0	0	0	0	0	0	0	0	0	0	0	0
Exiguobacterium	2	93	0	0	0	0	1	0	0	0	0	1	0	0	0	0	0	1	0	0
Faecalibacterium	1	12	0	0	0	0	0	0	0	0	0	0	0	0	0	0	0	1	0	0
Ferrimonas	2	115	0	0	0	0	0	0	0	0	1	0	0	0	0	0	0	1	0	0
Ferroglobus	1	14	0	0	0	0	0	0	0	0	0	1	0	0	0	0	0	1	0	0
Ferroplasma	1	11	0	0	0	0	1	0	0	0	0	0	0	0	0	0	0	1	0	0
Fervidicoccus	1	7	0	0	0	0	0	0	0	0	0	0	0	0	0	0	0	1	0	0
Fervidobacterium	2	54	0	0	0	0	0	0	0	0	0	0	0	0	0	0	0	1	0	0
Fibrella	1	17	0	0	0	0	1	0	0	0	0	0	0	0	0	0	0	1	0	0
Fibrobacter	2	52	0	0	0	1	0	0	0	0	0	0	0	0	0	0	0	1	0	0
Filifactor	1	9	0	0	0	0	0	0	0	0	0	0	0	0	0	0	0	1	0	0
Finegoldia	2	55	0	0	0	0	0	0	0	0	0	0	0	0	0	0	0	1	0	0
Firmicutes	1	15	0	0	0	0	0	0	0	0	0	1	0	0	0	0	0	1	0	0
Fischerella	1	15	0	0	0	0	0	0	0	0	0	0	0	0	0	0	0	1	0	0
Flavobacteria	1	7	0	0	0	0	0	0	0	0	0	0	0	0	0	0	0	0	0	0
Flavobacteriaceae	2	69	0	0	0	0	0	0	0	0	0	0	0	0	0	0	0	1	0	0
Flavobacteriales	1	3	0	0	0	0	0	0	0	0	0	0	0	0	0	0	0	0	0	0
Flavobacterium	2	103	0	0	0	0	0	0	0	0	0	1	0	0	0	0	0	1	0	0
Flexibacter	1	17	0	0	0	0	0	0	0	0	0	0	0	0	0	0	0	1	0	0
Flexistipes	1	11	0	0	0	0	0	0	0	0	0	1	0	0	0	0	0	1	0	0
Fluoribacter	1	6	0	0	0	0	0	0	0	0	0	0	0	0	0	0	0	0	0	0
Fluviicola	2	69	0	0	0	0	0	0	0	0	0	0	0	0	0	0	0	1	0	0
Formosa	1	2	0	0	0	0	0	0	0	0	0	0	0	0	0	0	0	0	0	0
Francisella	2	88	0	0	0	0	0	0	0	0	0	0	0	0	0	0	0	1	0	0
Frankia	3	202	0	0	1	1	1	0	1	0	0	1	1	0	1	0	1	1	1	0
Frateuria	1	25	0	0	0	0	0	0	0	0	0	0	0	1	0	0	0	1	0	0
Fructobacillus	1	2	0	0	0	0	0	0	0	0	0	0	0	0	0	0	0	0	0	0
Fulvimarina	1	2	0	0	0	0	0	0	0	0	0	0	0	0	0	0	0	0	0	0
Fusobacterium	1	48	0	1	0	0	0	0	1	0	0	0	0	0	0	0	0	1	0	0
Gallibacterium	2	87	0	0	0	0	0	0	0	0	0	0	0	0	0	0	0	1	0	0
Gallionella	2	67	0	0	0	0	0	0	0	0	0	1	0	0	0	0	0	1	0	0
Gammaproteobacteria	1	9	0	0	0	0	0	0	0	0	0	0	0	0	0	0	0	0	0	0
Gardnerella	1	31	0	0	0	0	0	0	0	0	0	0	0	0	0	0	0	1	0	0
Geitlerinema	1	9	0	0	0	0	0	0	0	0	0	0	0	0	0	0	0	1	0	0
Gemmatimonas	2	93	0	0	0	0	0	0	0	0	0	0	0	0	0	0	0	1	0	0
Geobacillus	2	168	0	0	1	1	1	0	0	0	0	1	0	0	0	0	0	1	1	0
Geobacter	2	150	0	0	0	1	0	0	0	0	0	1	0	0	0	0	0	1	0	0
Geodermatophilus	2	136	0	0	0	0	1	1	0	0	0	0	0	0	1	0	0	1	0	0
Geopsychrobacter	1	2	0	0	0	0	0	0	0	0	0	0	0	0	0	0	0	0	0	0
Gillisia	1	11	0	0	0	0	0	0	0	0	0	0	0	0	0	0	0	0	0	0
Gilvimarinus	1	4	0	0	0	0	0	0	0	0	0	0	0	0	0	0	0	0	0	0
Glaciecola	2	117	0	0	0	0	1	0	0	0	1	0	0	0	0	0	0	1	0	0

Genus	Cluster Number	Protein Counts	K01612	K01615	K01617	K01666	K01692	K01721	K01726	K01781	K01800	K01821	K01822	K01856	K01857	K01860	K01913	K01951	K02554	K03268
Gloeobacter	2	67	0	0	0	0	0	0	0	0	0	0	0	0	0	0	0	1	0	0
Gloeocapsa	1	20	0	0	0	0	0	1	0	0	0	0	0	0	0	0	0	1	0	0
Gloeothece	1	1	0	0	0	0	0	0	0	0	0	0	0	0	0	0	0	0	0	0
Gluconacetobacter	2	96	0	0	0	0	1	0	0	0	0	1	0	0	1	0	0	1	1	0
Gluconobacter	2	76	0	0	0	0	0	0	0	0	0	0	0	0	0	0	0	1	0	0
Gordonia	1	158	0	0	1	1	1	1	0	0	0	1	1	0	1	0	0	1	0	0
Gordonibacter	1	32	0	0	0	0	0	0	0	0	0	0	0	0	0	0	0	1	0	0
Gramella	2	104	0	0	0	0	0	0	1	0	0	0	0	0	0	0	0	1	0	0
Granulibacter	2	90	0	0	0	0	1	0	0	0	0	0	0	0	0	0	0	1	0	0
Granulicella	2	98	0	0	0	0	0	0	0	0	0	0	0	0	0	0	0	1	0	0
Grimontia	1	9	0	0	0	0	0	0	0	0	0	0	0	0	0	0	0	0	0	0
Haemophilus	2	117	0	0	0	0	0	0	0	0	0	1	0	0	0	0	0	1	0	0
Hafnia	1	29	0	0	0	0	0	0	0	0	0	0	0	0	0	0	0	1	0	0
Hahella	3	130	0	0	0	0	1	0	0	0	0	1	0	0	0	0	0	1	0	0
Halalkalicoccus	1	18	0	0	0	0	0	0	0	0	0	0	0	0	0	0	0	1	0	0
Halanaerobium	2	89	0	0	0	0	0	0	0	0	0	0	0	0	0	0	0	1	0	0
Haliangium	2	108	0	0	0	1	1	0	0	0	0	0	0	0	0	0	0	1	0	0
Haliscomenobacter	2	107	0	0	0	0	0	0	0	0	0	0	0	0	1	0	0	1	0	0
Haloarcula	1	22	0	0	0	0	0	0	1	0	0	0	0	0	0	0	0	1	0	0
Halobacillus	1	19	0	0	0	0	0	0	0	0	0	0	0	0	0	0	0	1	0	0
Halobacteriaceae	1	1	0	0	0	0	0	0	0	0	0	0	0	0	0	0	0	0	0	0
Halobacterium	1	10	0	0	0	0	0	0	0	0	0	0	0	0	0	0	0	1	0	0
Halobacteroides	1	14	0	0	0	0	0	0	0	0	0	1	0	0	0	0	0	1	0	0
Haloferax	1	20	0	0	0	0	0	0	0	0	0	0	0	0	0	0	0	1	0	0
Halogeometricum	1	12	0	0	0	0	0	0	0	0	0	0	0	0	0	0	0	1	0	0
Halomicrobium	1	10	0	0	0	0	0	0	0	0	0	0	0	0	0	0	0	1	0	0
Halomonas	1	149	0	0	0	0	0	0	0	0	0	0	0	0	1	0	0	1	0	0
Halopiger	1	16	0	0	0	0	0	0	0	0	0	0	0	0	0	0	0	1	0	0
Haloquadratum	1	19	0	0	0	0	0	0	0	0	0	0	0	0	0	0	0	1	0	0
Halorhabdus	1	10	0	0	0	0	0	0	0	0	0	0	0	0	0	0	0	1	0	0
Halorhodospira	2	71	0	0	0	0	0	0	0	0	0	0	0	0	0	0	0	1	0	0
Halorubrum	1	16	0	0	0	0	0	0	0	0	0	0	0	0	0	0	0	1	0	0
Haloterrigena	1	19	0	0	0	0	0	0	0	0	0	0	0	0	0	0	0	1	0	0
Halothece	1	9	0	0	0	0	0	0	0	0	0	1	0	0	0	0	0	1	0	0
Halothermothrix	2	50	0	0	0	0	0	0	0	0	0	0	0	0	0	0	0	1	0	0
Halothiobacillus	2	65	0	0	0	0	0	0	0	0	0	1	0	0	0	0	0	1	0	0
Halovivax	1	12	0	0	0	0	0	0	0	0	0	0	0	0	0	0	0	1	0	0
Helicobacter	2	79	0	0	0	0	0	0	0	0	0	1	0	0	0	0	0	1	0	0
Heliobacillus	1	4	0	0	0	0	0	0	0	0	0	0	0	0	0	0	0	0	0	0
Heliobacterium	2	51	0	0	0	0	0	0	0	0	0	1	0	0	0	0	0	1	0	0
Herbaspirillum	2	172	0	0	0	0	1	1	0	0	0	0	0	1	1	0	0	1	1	0
Herminiimonas	3	89	0	0	0	0	1	0	0	0	0	1	0	0	0	0	0	1	0	0
Herpetosiphon	3	90	0	0	0	0	0	0	0	0	0	0	0	0	0	0	0	1	0	0
Hippea	2	44	0	0	0	0	0	0	0	0	0	0	0	0	0	0	0	1	0	0
Hirschia	2	102	0	0	0	0	0	0	0	0	1	1	0	0	0	0	0	1	1	0
Histophilus	2	72	0	0	0	0	0	0	0	0	0	0	0	0	0	0	0	1	0	0
Hoeflea	1	16	0	0	0	0	0	0	0	0	0	0	0	0	0	0	0	0	0	0
Hydrogenivirga	1	2	0	0	0	0	0	0	0	0	0	0	0	0	0	0	0	0	0	0
Hydrogenobacter	2	55	0	0	0	0	0	0	0	0	0	0	0	0	0	0	0	1	0	0
Hydrogenobaculum	2	44	0	0	0	0	0	0	0	0	0	0	0	0	0	0	0	1	0	0
Hydrogenophaga	1	9	0	0	0	0	0	0	0	0	0	0	0	0	0	0	0	1	0	0
Hylemonella	1	4	0	0	0	0	0	0	0	0	0	0	0	0	0	0	0	1	0	0

Genus	Cluster Number	Protein Counts	K01612	K01615	K01617	K01666	K01692	K01721	K01726	K01781	K01800	K01821	K01822	K01856	K01857	K01860	K01913	K01951	K02554	K03268
Hyperthermus	1	6	0	0	0	0	0	0	0	0	0	0	0	0	0	0	0	0	0	0
Hyphomicrobium	2	93	0	0	0	0	1	0	0	0	0	1	0	0	0	0	0	1	0	0
Hyphomonas	3	105	0	0	0	0	1	0	0	0	0	0	1	0	0	0	0	1	0	0
Ideonella	1	18	0	0	0	1	0	0	0	0	0	0	0	0	0	0	0	0	0	0
Idiomarina	2	86	0	0	0	0	0	0	0	0	1	0	0	0	0	0	0	1	0	0
Ignavibacterium	1	14	0	0	0	0	0	0	0	0	0	0	0	0	0	0	0	1	0	0
Ignicoccus	1	5	0	0	0	0	0	0	0	0	0	0	0	0	0	0	0	1	0	0
Ignisphaera	1	8	0	0	0	0	0	0	0	0	0	1	0	0	0	0	0	1	0	0
Ilyobacter	2	85	0	0	0	0	0	0	0	0	0	0	0	0	0	0	0	1	0	0
Intrasporangium	2	114	0	0	1	1	1	0	0	0	0	1	0	0	0	0	0	1	0	0
Isoptericola	1	18	0	0	0	0	0	0	0	0	0	0	0	0	1	0	0	1	0	0
Isosphaera	2	75	0	0	0	0	0	0	0	0	0	0	0	0	0	0	0	1	0	0
Jannaschia	3	151	0	0	0	0	1	0	0	0	1	0	0	1	0	0	0	1	0	0
Janthinobacterium	2	99	0	0	0	0	1	0	0	1	0	1	0	0	0	0	0	1	0	0
Jonesia	2	56	0	0	0	0	0	0	0	0	0	0	0	0	0	0	0	1	0	0
Joostella	1	3	0	0	0	0	0	0	0	0	0	0	0	0	0	0	0	0	0	0
Kaistia	1	2	0	0	0	0	0	0	0	0	0	0	0	0	0	0	0	0	0	0
Kangiella	2	84	0	0	0	0	0	0	0	0	1	0	0	0	0	0	0	1	0	0
Ketogulonicigenium	2	102	0	0	0	0	0	0	0	0	0	0	0	0	1	0	0	1	0	0
Kineococcus	1	24	0	0	0	0	0	0	0	0	0	0	0	0	0	0	0	1	0	0
Kinetoplastibacterium	1	2	0	0	0	0	0	0	0	0	0	0	0	0	0	0	0	1	0	0
Kingella	1	4	0	0	0	0	0	0	0	0	0	0	0	0	0	0	0	1	0	0
Kitasatospora	1	36	0	0	0	1	1	0	0	0	0	0	0	0	1	0	0	1	1	0
Klebsiella	3	222	0	0	0	1	1	1	0	0	0	1	0	1	1	0	0	1	1	0
Kluyvera	1	1	0	0	0	0	0	0	0	0	0	0	0	0	0	0	0	0	0	0
Kocuria	2	87	0	0	0	0	1	0	0	0	0	0	0	0	1	0	0	1	0	0
Kordia	1	5	0	0	0	0	0	0	0	0	0	0	0	0	0	0	0	0	0	0
Kosmotoga	2	61	0	0	0	0	0	0	0	0	0	0	0	0	0	0	0	1	0	0
Kribbella	2	99	0	0	0	0	0	0	0	0	0	1	0	0	0	0	0	1	0	0
Krokinobacter	2	90	0	0	0	0	0	0	0	0	0	0	0	0	0	0	0	1	0	0
Kyrpidia	2	113	0	0	1	1	1	0	0	0	0	1	0	0	0	0	0	1	0	0
Kytococcus	2	70	0	0	0	0	1	0	0	0	0	0	0	0	0	0	0	1	0	0
Labrenzia	1	4	0	0	0	0	0	0	0	0	0	0	0	0	0	0	0	0	0	0
Lachnoanaerobaculum	1	1	0	0	0	0	0	0	0	0	0	0	0	0	0	0	0	0	0	0
Lachnospiraceae	1	51	0	0	0	0	0	0	0	0	0	0	0	0	0	0	0	0	0	0
Lacinutrix	1	20	0	0	0	0	0	0	0	0	0	0	0	0	0	0	0	1	0	0
Lactobacillus	2	119	0	0	0	0	0	0	0	0	0	1	0	0	1	0	0	1	0	0
Lactococcus	2	82	0	0	0	0	0	0	0	0	0	1	0	0	0	0	0	1	0	0
Lamprocystis	1	2	0	0	0	0	0	0	0	0	0	0	0	0	0	0	0	0	0	0
Laribacter	2	80	0	0	0	0	0	0	0	0	0	1	0	0	0	0	0	1	0	0
Lawsonia	2	33	0	0	0	0	0	0	0	0	0	0	0	0	0	0	0	1	0	0
Leadbetterella	2	79	0	0	0	0	0	0	0	0	0	0	0	0	0	0	0	1	0	0
Leeuwenhoekiella	1	2	0	0	0	0	0	0	0	0	0	0	0	0	0	0	0	0	0	0
Legionella	2	117	0	0	0	1	0	0	1	0	1	0	0	0	0	0	0	1	0	0
Leifsonia	2	48	0	0	0	0	0	0	0	0	0	0	0	0	0	0	0	1	0	0
Leptolyngbya	1	10	0	0	0	0	0	0	0	0	0	0	0	0	0	0	0	1	0	0
Leptospira	2	76	0	0	0	0	1	0	0	0	0	1	0	0	0	0	0	1	0	0
Leptospirillum	1	10	0	0	0	0	0	0	0	0	0	0	0	0	0	0	0	1	0	0
Leptothrix	3	186	0	0	1	1	1	0	0	0	1	1	0	0	1	0	0	1	1	0
Leptotrichia	2	43	0	0	0	0	0	0	0	0	0	0	0	0	0	0	0	1	0	0
Leucobacter	1	2	0	0	0	0	0	0	0	0	0	0	0	0	0	0	0	0	0	0
Leuconostoc	2	64	0	0	0	0	0	0	0	0	0	1	0	0	0	0	0	1	0	0

Genus	Cluster Number	Protein Counts	K01612	K01615	K01617	K01666	K01692	K01721	K01726	K01781	K01800	K01821	K01822	K01856	K01857	K01860	K01913	K01951	K02554	K03268
Liberibacter	1	4	0	0	0	0	0	0	0	0	0	0	0	0	0	0	0	1	0	0
Limnohabitans	1	11	0	0	0	0	0	0	0	0	0	0	0	0	0	0	0	1	0	0
Listeria	2	77	0	0	0	0	0	0	0	0	0	1	0	0	0	0	0	1	0	0
Listeriaceae	1	6	0	0	0	0	0	0	0	0	0	0	0	0	0	0	0	1	0	0
Listonella	1	38	0	0	0	0	0	0	0	0	1	0	0	0	0	0	0	1	0	0
Loktanella	1	3	0	0	0	0	0	0	0	0	0	0	0	0	0	0	0	0	0	0
Lysinibacillus	2	97	0	0	0	0	0	0	0	0	0	1	0	0	0	0	0	1	0	0
Macrococcus	2	60	0	0	0	0	0	0	0	0	0	0	0	0	0	0	0	1	0	0
Magnetococcus	2	64	0	0	0	0	0	0	0	0	0	0	0	0	0	0	0	1	0	0
Magnetospirillum	2	113	0	0	0	0	1	0	0	0	0	0	0	0	0	0	0	1	0	0
Mahella	2	58	0	0	0	0	0	0	0	0	0	1	0	0	0	0	0	1	0	0
Mannheimia	2	85	0	0	0	0	0	0	0	0	0	0	0	0	0	0	0	1	0	0
Maribacter	3	87	0	0	0	0	0	0	0	0	0	0	0	0	0	0	0	1	0	0
Maricaulis	3	87	0	0	0	0	1	0	0	0	0	0	0	0	0	0	0	1	1	0
Marichromatium	1	2	0	0	0	0	0	0	0	0	0	0	0	0	0	0	0	0	0	0
Marinithermus	2	77	0	0	0	0	1	0	0	0	0	0	0	0	0	0	0	1	0	0
Marinitoga	1	15	0	0	0	0	0	0	0	0	0	0	0	0	0	0	0	1	0	0
Marinobacter	2	163	0	0	1	1	1	0	0	0	0	1	0	1	1	0	0	1	1	0
Marinomonas	3	192	0	0	0	1	1	0	0	0	1	1	0	1	0	0	0	1	0	0
Maritimibacter	1	7	0	0	0	0	0	0	0	0	0	0	0	0	0	0	0	0	0	0
Marivirga	2	96	0	0	0	0	1	0	0	0	0	0	0	0	0	0	0	1	0	0
Martelella	1	3	0	0	0	0	0	0	0	0	0	0	0	0	0	0	0	0	0	0
Marvinbryantia	1	1	0	0	0	0	0	0	0	0	0	0	0	0	0	0	0	0	0	0
Massilia	1	4	0	0	0	1	0	0	0	0	0	0	0	0	0	0	0	0	0	0
Mastigocladopsis	1	16	0	0	0	0	0	0	0	0	0	0	0	0	0	0	0	0	0	0
Megamonas	1	47	0	0	0	0	0	0	0	0	0	0	0	0	0	0	0	1	0	0
Megasphaera	1	14	0	0	0	0	0	0	0	0	0	0	0	0	0	0	0	0	0	0
Meiothermus	2	110	0	0	0	0	1	0	0	0	0	0	0	0	1	0	0	1	0	0
Melissococcus	2	46	0	0	0	0	0	0	0	0	0	0	0	0	0	0	0	1	0	0
Mesoflavibacter	1	6	0	0	0	0	0	0	0	0	0	0	0	0	0	0	0	0	0	0
Mesoplasma	1	23	0	0	0	0	0	0	0	0	0	0	0	0	0	0	0	1	0	0
Mesorhizobium	2	201	0	0	0	0	1	1	0	0	0	1	1	0	1	0	0	1	0	0
Mesotoga	1	14	0	0	0	0	0	0	0	0	0	0	0	0	0	0	0	1	0	0
Metallosphaera	1	18	0	0	0	0	0	0	0	0	0	0	0	0	0	0	0	1	0	0
Methanobacterium	1	7	0	0	0	0	0	0	0	0	0	1	0	0	0	0	0	1	0	0
Methanobrevibacter	1	9	0	0	0	0	0	0	0	0	0	1	0	0	0	0	0	1	0	0
Methanocaldococcus	1	6	0	0	0	0	0	0	0	0	0	1	0	0	0	0	0	1	0	0
Methanocella	1	15	1	0	0	0	0	0	0	0	0	1	0	0	0	0	0	1	0	0
Methanococcaceae	1	1	0	0	0	0	0	0	0	0	0	0	0	0	0	0	0	0	0	0
Methanococcoides	1	5	0	0	0	0	0	0	0	0	0	0	0	0	0	0	0	1	0	0
Methanococcus	1	9	0	0	0	0	0	0	0	0	0	1	0	0	0	0	0	1	0	0
Methanocorpusculum	1	7	0	0	0	1	0	0	0	0	0	0	0	0	0	0	0	1	0	0
Methanoculleus	1	8	0	0	0	0	0	0	0	0	0	1	0	0	0	0	0	1	0	0
Methanohalobium	1	5	0	0	0	0	0	0	0	0	0	0	0	0	0	0	0	1	0	0
Methanohalophilus	1	5	0	0	0	0	0	0	0	0	0	0	0	0	0	0	0	1	0	0
Methanolobus	1	5	0	0	0	0	0	0	0	0	0	0	0	0	0	0	0	1	0	0
Methanomethylovorans	1	5	0	0	0	0	0	0	0	0	0	0	0	0	0	0	0	1	0	0
Methanoplanus	1	7	0	0	0	0	0	0	0	0	0	0	0	0	0	0	0	1	0	0
Methanopyrus	1	4	0	0	0	0	0	0	0	0	0	0	0	0	0	0	0	1	0	0
Methanoregula	1	8	0	0	0	0	0	0	0	0	0	1	0	0	0	0	0	1	0	0
Methanosaeta	1	9	0	0	0	0	0	0	0	0	0	0	0	0	0	0	0	1	0	0
Methanosalsum	1	7	0	0	0	0	0	0	0	0	0	0	0	0	0	0	0	1	0	0

Genus	Cluster Number	Protein Counts	K01612	K01615	K01617	K01666	K01692	K01721	K01726	K01781	K01800	K01821	K01822	K01856	K01857	K01860	K01913	K01951	K02554	K03268
**Methanosarcina**	1	18	1	0	0	0	0	0	0	0	0	0	0	0	0	0	0	1	0	0
**Methanosphaera**	1	7	0	0	0	0	0	0	0	0	0	1	0	0	0	0	0	1	0	0
**Methanosphaerula**	1	8	0	0	0	0	0	0	0	0	0	1	0	0	0	0	0	1	0	0
**Methanospirillum**	1	6	0	0	0	0	0	0	0	0	0	0	0	0	0	0	0	1	0	0
**Methanothermobacter**	1	6	0	0	0	0	0	0	0	0	0	1	0	0	0	0	0	1	0	0
**Methanothermococcus**	1	5	0	0	0	0	0	0	0	0	0	0	0	0	0	0	0	1	0	0
**Methanothermus**	1	5	0	0	0	0	0	0	0	0	0	0	0	0	0	0	0	1	0	0
**Methanotorris**	1	5	0	0	0	0	0	0	0	0	0	0	0	0	0	0	0	1	0	0
**Methylacidiphilum**	2	48	0	0	0	0	0	0	0	0	0	0	0	0	0	0	0	1	0	0
**Methylarcula**	1	2	0	0	0	0	0	0	0	0	0	0	0	0	0	0	0	0	0	0
**Methylibium**	1	38	0	0	1	1	1	0	0	0	0	1	0	0	0	0	0	1	1	0
**Methylobacillus**	2	61	0	0	0	0	0	0	0	0	0	1	0	0	0	0	0	1	0	0
**Methylobacterium**	3	236	0	0	1	0	1	1	0	0	1	1	0	1	1	0	0	1	0	0
**Methylocella**	3	117	0	0	1	1	1	1	0	0	0	1	0	0	0	0	0	1	0	0
**Methylococcus**	2	80	0	0	0	0	0	0	0	0	0	0	0	0	0	0	0	1	0	0
**Methylocystis**	1	21	0	0	0	0	1	0	0	0	0	0	0	0	0	0	0	1	0	0
**Methyloferula**	1	2	0	0	0	0	0	0	0	0	0	0	0	0	0	0	0	0	0	0
**Methylomicrobium**	1	12	0	0	0	0	0	0	0	0	0	0	0	0	0	0	0	1	0	0
**Methylomonas**	1	15	0	0	0	0	0	0	0	0	0	1	0	0	0	0	0	1	0	0
**Methylophaga**	1	7	0	0	0	1	0	0	0	0	0	0	0	0	0	0	0	1	0	0
**Methylophilus**	1	2	0	0	0	0	0	0	0	0	0	0	0	0	0	0	0	0	0	0
**Methylosinus**	1	2	0	0	0	0	0	0	0	0	0	0	0	0	0	0	0	0	0	0
**Methylotenera**	2	72	0	0	0	0	0	0	0	0	0	1	0	0	0	0	0	1	0	0
**Methyloversatilis**	1	5	0	0	0	0	0	0	0	0	0	0	0	0	0	0	0	0	0	0
**Methylovorus**	2	66	0	0	0	0	0	0	0	0	0	1	0	0	0	0	0	1	0	0
**Micavibrio**	1	9	0	0	0	0	1	0	0	0	0	1	0	0	0	0	0	1	0	0
**Microbacterium**	2	115	0	0	0	0	1	1	0	0	0	0	0	0	1	0	0	1	0	0
**Microchaete**	1	23	0	0	0	0	0	0	0	0	0	0	0	0	0	0	0	1	0	0
**Micrococcus**	2	72	0	0	0	0	1	0	0	0	0	0	0	0	0	0	0	1	0	0
**Microcoleus**	1	17	0	0	0	0	0	0	0	0	0	0	0	0	0	0	0	1	0	0
**Microcystis**	2	67	0	0	0	0	0	0	0	0	0	0	0	0	0	0	0	1	0	0
**Microlunatus**	1	25	0	0	0	0	0	0	0	0	0	0	0	1	0	0	0	1	0	0
**Micromonospora**	2	115	0	0	1	1	1	0	0	0	0	0	0	0	0	0	0	1	0	0
**Microvirga**	1	6	0	0	0	0	0	0	0	0	0	0	0	0	0	0	0	0	0	0
**Mobiluncus**	1	41	0	0	0	0	0	0	0	0	0	0	0	0	0	0	0	1	0	0
**Modestobacter**	1	43	0	0	0	0	1	1	0	0	0	0	0	0	1	0	0	1	0	0
**Moorea**	1	2	0	0	0	0	0	0	0	0	0	0	0	0	0	0	0	0	0	0
**Moorella**	2	69	0	0	1	1	0	0	0	0	0	0	0	0	0	0	0	1	0	0
**Moraxella**	2	66	0	0	0	0	0	0	0	0	1	0	0	0	0	0	0	1	0	0
**Morganella**	1	29	0	0	0	0	0	0	0	0	0	1	0	0	0	0	0	1	0	0
**Moritella**	1	2	0	0	0	0	0	0	0	0	0	0	0	0	0	0	0	1	0	0
**Muricauda**	1	23	0	0	0	0	0	0	0	0	0	0	0	0	0	0	0	1	0	0
**Mycobacterium**	3	306	0	0	1	1	1	1	0	0	0	1	1	1	1	0	1	1	1	0
**Mycoplasma**	1	53	0	0	0	0	0	0	0	0	0	0	0	0	0	0	0	1	0	0
**Myxococcus**	2	110	0	0	1	0	1	0	0	0	0	0	0	0	0	0	0	1	0	0
**Nakamurella**	2	115	0	0	1	1	1	0	0	0	0	0	0	0	0	0	0	1	0	0
**napthalene**	1	2	0	0	0	0	0	0	0	0	0	0	0	0	0	0	0	0	0	0
**Natranaerobius**	2	55	0	0	0	0	0	0	0	0	0	0	0	0	0	0	0	1	0	0
**Natrialba**	1	16	0	0	0	0	0	0	0	0	0	0	0	0	0	0	0	1	0	0
**Natrinema**	1	16	0	0	0	0	0	0	0	0	0	0	0	0	0	0	0	1	0	0
**Natronobacterium**	1	11	0	0	0	0	0	0	0	0	0	0	0	0	0	0	0	1	0	0
**Natronococcus**	1	21	0	0	0	0	0	0	0	0	0	0	0	0	0	0	0	1	0	0

Genus	Cluster Number	Protein Counts	K01612	K01615	K01617	K01666	K01692	K01721	K01726	K01781	K01800	K01821	K01822	K01856	K01857	K01860	K01913	K01951	K02554	K03268
**Natronomonas**	1	20	0	0	0	0	0	0	0	0	0	0	0	0	0	0	0	1	0	0
**Nautilia**	2	48	0	0	0	0	0	0	0	0	0	0	0	0	0	0	0	1	0	0
**Neisseria**	2	67	0	0	0	0	0	0	0	0	0	1	0	0	0	0	0	1	0	0
**Neisseriaceae**	1	1	0	0	0	0	0	0	0	0	0	0	0	0	0	0	0	0	0	0
**Neorickettsia**	1	31	0	0	0	0	0	0	0	0	0	0	0	0	0	0	0	1	0	0
**Neptuniibacter**	1	2	0	0	0	0	0	0	0	0	0	0	0	0	0	0	0	0	0	0
**Niastella**	1	21	0	0	0	0	0	0	0	0	0	1	0	0	0	0	0	1	0	0
**Nitratifractor**	2	54	0	0	0	0	0	0	0	0	0	0	0	0	0	0	0	1	0	0
**Nitratireductor**	1	19	0	0	0	0	0	0	0	0	0	0	0	0	0	0	0	1	0	0
**Nitratiruptor**	2	40	0	0	0	0	0	0	0	0	0	0	0	0	0	0	0	1	0	0
**Nitrobacter**	2	99	0	0	0	0	1	0	0	0	0	0	0	0	0	0	0	1	0	0
**Nitrococcus**	1	2	0	0	0	0	0	0	0	0	0	0	0	0	0	0	0	0	0	0
**Nitrosococcus**	2	94	0	0	0	0	0	0	0	0	1	0	0	0	0	0	0	1	0	0
**Nitrosomonas**	2	72	0	0	0	0	0	0	0	0	0	1	0	0	0	0	0	1	0	0
**Nitrosopumilaceae**	1	7	0	0	0	0	0	0	0	0	0	1	0	0	0	0	0	1	0	0
**Nitrosopumilus**	1	11	0	0	0	1	0	0	0	0	0	1	0	0	0	0	0	1	0	0
**Nitrosospira**	2	74	0	0	0	0	0	0	0	0	0	1	0	0	0	0	0	1	0	0
**Nocardia**	2	185	0	0	1	1	1	0	0	0	0	1	1	0	1	0	1	1	1	0
**Nocardioidaceae**	1	2	0	0	0	0	0	0	0	0	0	0	0	0	0	0	0	0	0	0
**Nocardioides**	2	157	0	0	1	1	1	0	0	0	0	0	1	0	0	0	0	1	0	0
**Nocardiopsis**	2	102	0	0	0	1	1	0	0	0	0	1	0	0	0	0	0	1	0	0
**Nodularia**	1	22	0	0	0	0	0	0	0	0	0	0	0	0	0	0	0	1	0	0
**Nonlabens**	1	19	0	0	0	1	0	0	0	0	0	0	0	0	0	0	0	1	0	0
**Nostoc**	2	111	0	0	0	0	0	0	0	0	0	1	0	0	0	0	0	1	0	0
**Nostocaceae**	1	2	0	0	0	0	0	0	0	0	0	0	0	0	0	0	0	0	0	0
**Novosphingobium**	2	169	0	0	0	1	1	0	0	0	1	1	1	1	0	0	0	1	1	0
**Oceanibaculum**	1	3	0	0	0	0	0	0	0	0	0	0	0	0	0	0	0	0	0	0
**Oceanibulbus**	1	12	0	0	0	0	0	0	0	0	0	0	0	0	0	0	0	0	0	0
**Oceanicola**	1	9	0	0	0	0	0	0	0	0	0	0	0	0	0	0	0	0	0	0
**Oceanimonas**	1	33	0	0	0	0	0	0	0	0	0	1	0	1	1	0	0	1	0	0
**Oceaniovalibus**	1	2	0	0	0	0	0	0	0	0	0	0	0	0	0	0	0	0	0	0
**Oceanithermus**	2	72	0	0	0	0	0	0	0	0	0	0	0	0	0	0	0	1	0	0
**Oceanobacillus**	2	91	0	0	0	0	0	0	0	0	0	0	1	0	0	0	0	1	0	0
**Oceanospirillum**	1	2	0	0	0	0	0	0	0	0	0	0	0	0	0	0	0	0	0	0
**Ochrobactrum**	2	133	0	0	0	0	1	0	0	0	0	1	0	0	1	0	0	1	0	0
**Octadecabacter**	1	29	0	0	0	1	0	1	0	0	0	0	0	0	0	0	0	1	0	0
**Oenococcus**	2	41	0	0	0	0	0	0	0	0	0	1	0	0	0	0	0	1	0	0
**Oleomonas**	1	2	0	0	0	0	0	0	0	0	0	0	0	0	0	0	0	0	0	0
**Oligotropha**	3	87	0	0	0	0	0	0	0	0	0	1	0	0	0	0	0	1	1	0
**Olsenella**	2	40	0	0	0	0	0	0	0	0	0	0	0	0	0	0	0	1	0	0
**Opitutus**	2	67	0	0	0	0	0	0	0	0	0	0	0	0	0	0	0	1	0	0
**Orientia**	1	21	0	0	0	0	0	0	0	0	0	0	0	0	0	0	0	0	0	0
**Ornithobacterium**	1	11	0	0	0	0	0	0	0	0	0	0	0	0	0	0	0	1	0	0
**Oscillatoria**	1	16	0	0	0	0	0	0	0	0	0	0	0	0	0	0	0	1	0	0
**Oscillatoriales**	1	2	0	0	0	0	0	0	0	0	0	0	0	0	0	0	0	0	0	0
**Oscillibacter**	1	20	0	0	0	0	0	0	0	0	0	0	0	0	0	0	0	1	0	0
**Owenweeksia**	1	15	0	0	0	0	0	0	0	0	0	0	0	0	0	0	0	1	0	0
**Oxalobacteraceae**	1	4	0	0	0	0	0	0	0	0	0	0	0	0	0	0	0	0	0	0
**Paenibacillus**	2	152	0	0	0	1	0	1	0	0	0	1	0	1	0	0	0	1	0	0
**Paludibacter**	2	67	0	0	0	1	0	0	0	0	0	0	0	0	0	0	0	1	0	0
**Pandoraea**	1	18	0	0	0	1	0	0	0	0	0	0	0	0	0	1	0	0	0	1
**Pannonibacter**	1	3	0	0	0	0	0	0	0	0	0	0	0	0	0	0	0	0	0	0

Genus	Cluster Number	Protein Counts	K01612	K01615	K01617	K01666	K01692	K01721	K01726	K01781	K01800	K01821	K01822	K01856	K01857	K01860	K01913	K01951	K02554	K03268
**Pantholops**	1	23	0	0	0	0	0	0	0	0	0	0	0	0	0	0	0	0	0	0
**Pantoea**	2	181	0	0	0	0	0	1	0	0	0	1	0	0	1	0	0	1	0	0
**Parabacteroides**	2	64	0	0	0	0	0	0	0	0	0	0	0	0	0	0	0	1	0	0
**Parachlamydia**	1	11	0	0	0	0	0	0	0	0	0	0	0	0	0	0	0	1	0	0
**Paracoccus**	2	169	0	0	0	0	0	0	0	0	1	1	0	1	0	0	0	1	0	0
**Parvibaculum**	3	117	0	0	0	0	1	0	0	0	0	0	0	0	0	0	0	1	0	0
**Parvularcula**	2	72	0	0	0	0	1	0	0	0	0	0	0	0	0	0	0	1	0	0
**Pasteurella**	2	85	0	0	0	0	0	0	0	0	0	0	0	0	0	0	0	1	0	0
**Pasteurellaceae**	1	2	0	0	0	0	0	0	0	0	0	0	0	0	0	0	0	0	0	0
**Pectobacterium**	1	44	0	0	0	1	0	0	0	0	0	1	0	0	1	0	0	1	0	0
**Pediococcus**	1	43	0	0	0	0	0	0	0	0	0	1	0	0	0	0	0	1	0	0
**Pedobacter**	2	88	0	0	0	0	0	0	0	0	0	0	0	0	0	0	0	1	0	0
**Pelagibaca**	1	10	0	0	0	0	0	0	0	0	0	0	0	0	0	0	0	0	0	0
**Pelagibacterium**	1	39	0	0	0	0	1	0	0	0	0	0	0	0	1	0	0	1	0	0
**Pelobacter**	2	85	0	0	0	0	0	0	0	0	0	1	0	0	0	0	0	1	0	0
**Pelodictyon**	2	62	0	0	0	0	0	0	0	0	0	0	0	0	0	0	0	1	0	0
**Pelosinus**	1	1	0	0	0	0	0	0	0	0	0	0	0	0	0	0	0	0	0	0
**Pelotomaculum**	2	60	0	0	0	1	0	0	0	0	0	1	0	0	0	0	0	1	0	0
**Peptostreptococcus**	1	1	0	0	0	0	0	0	0	0	0	0	0	0	0	0	0	0	0	0
**Persephonella**	2	55	0	0	0	0	0	0	0	0	0	1	0	0	0	0	0	1	0	0
**Petrotoga**	2	72	0	0	0	0	0	0	0	0	0	0	0	0	0	0	0	1	0	0
**Phaeobacter**	2	55	0	0	0	0	0	1	0	0	0	1	0	0	1	0	0	1	0	0
**Phenylobacterium**	3	103	0	0	0	0	1	0	0	0	0	0	0	0	0	0	0	1	1	0
**Photobacterium**	2	119	0	0	0	0	0	0	0	0	1	0	0	0	0	0	0	1	0	0
**Photorhabdus**	2	149	0	0	0	1	0	0	0	0	0	1	0	0	0	0	0	1	1	0
**Phycisphaera**	1	9	0	0	0	0	0	0	0	0	0	1	0	0	0	0	0	1	0	0
**Phyllobacterium**	1	6	0	0	0	0	0	0	0	0	0	0	0	0	0	0	0	0	0	0
**Picrophilus**	1	15	0	0	0	0	0	0	0	0	0	0	0	0	0	0	0	1	0	0
**Pirellula**	2	85	0	0	0	0	0	0	0	0	0	0	0	0	0	0	0	1	0	0
**Planctomyces**	2	93	0	0	0	0	0	0	0	0	0	1	0	0	0	0	0	1	0	0
**Plautia**	1	17	0	0	0	0	0	0	0	0	0	0	0	0	0	0	0	1	0	0
**Plesiomonas**	1	4	0	0	0	0	0	0	0	0	0	0	0	0	0	0	0	0	0	0
**Pleurocapsa**	1	23	0	0	0	0	0	0	0	0	0	1	0	0	0	0	0	1	0	0
**Polaribacter**	2	74	0	0	0	1	1	0	0	0	0	0	0	0	0	0	0	1	0	0
**Polaromonas**	3	238	0	0	1	1	1	0	0	1	1	1	0	1	1	0	0	1	1	0
**Polymorphum**	3	173	0	0	0	1	1	1	0	0	0	0	0	0	1	0	0	1	0	0
**Polynucleobacter**	1	19	0	0	0	0	1	0	0	0	1	0	0	0	0	0	0	1	0	0
**Ponticaulis**	1	1	0	0	0	0	0	0	0	0	0	0	0	0	0	0	0	0	0	0
**Porphyromonas**	2	63	0	0	0	0	0	0	0	0	0	0	0	0	0	0	0	1	0	0
**Prevotella**	1	65	0	0	0	0	0	0	0	0	0	0	0	0	0	0	0	1	0	0
**Prochlorococcus**	2	68	0	0	0	0	0	0	0	0	0	0	0	0	0	0	0	1	0	0
**Propionibacterium**	1	62	0	0	0	0	1	0	0	0	0	0	0	0	1	0	0	1	0	0
**Prosthecochloris**	1	16	0	0	0	0	0	0	0	0	0	0	0	0	0	0	0	1	0	0
**Proteobacteria**	1	19	0	0	0	1	0	0	0	0	0	0	0	0	0	1	0	0	0	0
**Proteus**	2	113	0	0	0	0	0	0	0	0	0	1	0	0	0	0	0	1	0	0
**Providencia**	1	38	0	0	0	0	1	0	0	0	0	0	0	0	0	0	0	1	0	0
**Pseudaminobacter**	1	10	0	0	0	0	0	0	0	0	0	0	0	0	0	0	0	0	0	0
**Pseudanabaena**	1	11	0	0	0	0	0	1	0	0	0	1	0	0	0	0	0	1	0	0
**Pseudoalteromonas**	2	188	0	0	1	1	1	0	0	0	0	0	0	0	0	0	0	1	0	0
**Pseudochrobactrum**	1	7	0	0	0	0	0	0	0	0	0	0	0	0	0	0	0	0	0	0
**Pseudogulbenkiania**	1	40	0	0	0	0	0	0	0	0	1	1	0	1	1	0	0	1	0	0
**pseudomallei**	3	131	0	0	0	0	1	0	0	0	0	1	0	0	1	0	0	1	0	0

Genus	Cluster Number	Protein Counts	K01612	K01615	K01617	K01666	K01692	K01721	K01726	K01781	K01800	K01821	K01822	K01856	K01857	K01860	K01913	K01951	K02554	K03268
Pseudomonas	3	381	0	0	1	1	1	1	1	0	1	1	0	1	1	1	1	1	1	1
Pseudonocardia	3	180	0	0	1	1	1	1	0	1	0	1	0	1	1	0	0	1	0	0
Pseudorhodobacter	1	4	0	0	0	0	0	0	0	0	0	0	0	0	0	0	0	0	0	0
Pseudovibrio	1	33	0	0	0	0	1	0	0	0	0	1	0	0	1	0	0	1	0	0
Pseudoxanthomonas	2	124	0	0	1	1	0	0	0	0	1	1	0	0	0	0	0	1	1	0
Psychrobacter	2	124	0	0	0	0	0	0	0	0	1	0	0	1	0	0	0	1	0	0
Psychroflexus	1	22	0	0	0	1	0	0	0	0	0	0	0	0	0	0	0	1	0	0
Psychromonas	2	110	0	0	0	0	1	0	0	0	0	0	0	0	0	0	0	1	0	0
Pusillimonas	3	111	0	0	0	1	1	0	0	0	0	0	0	0	1	0	0	1	0	0
Pyrobaculum	1	15	0	0	0	0	0	0	0	0	0	0	0	0	0	0	0	1	0	0
Pyrococcus	1	10	0	0	0	0	0	0	0	0	0	0	0	0	0	0	0	1	0	0
Pyrolobus	1	4	0	0	0	0	0	0	0	0	0	0	0	0	0	0	0	1	0	0
Rahnella	2	143	0	0	0	0	1	1	0	0	0	1	0	0	0	0	0	1	0	0
Ralstonia	3	332	1	0	1	1	1	0	1	0	1	1	1	1	1	1	1	1	1	1
Ramlibacter	1	35	0	0	0	0	1	0	0	1	1	1	0	0	0	0	0	1	0	0
Raoultella	2	139	0	0	0	1	1	0	0	0	0	1	0	1	1	0	0	1	1	0
Raphidiopsis	1	5	0	0	0	0	0	1	0	0	0	0	0	0	0	0	0	0	0	0
Reinekea	1	2	0	0	0	0	0	0	0	0	0	0	0	0	0	0	0	0	0	0
Renibacterium	2	67	0	0	0	0	1	0	0	0	0	0	0	0	0	0	0	1	0	0
Rhizobiaceae	1	3	0	0	0	0	0	0	0	0	0	0	0	0	0	0	0	0	0	0
Rhizobiales	1	6	0	0	0	0	0	0	0	0	0	0	0	0	0	0	0	0	0	0
Rhizobium	3	257	0	0	1	1	1	1	0	0	1	1	0	0	1	0	0	1	0	0
Rhizobium/Agrobacterium	2	87	0	0	1	1	1	0	0	0	0	0	0	0	0	0	0	1	0	0
Rhodanobacter	1	21	0	0	0	0	0	0	0	0	0	0	0	0	0	0	0	1	0	0
Rhodobacter	3	175	0	0	0	0	0	0	0	0	0	0	0	0	1	0	0	1	0	0
Rhodobacteraceae	1	21	0	0	0	0	0	0	0	0	0	0	0	0	0	0	0	1	0	0
Rhodobacterales	1	34	0	0	0	0	0	0	0	0	0	0	0	0	0	0	0	1	0	0
Rhodococcus	2	292	0	0	1	1	1	1	1	0	0	1	1	1	1	0	1	1	1	0
Rhodocyclaceae	1	7	0	0	0	0	0	0	0	0	0	0	0	0	0	0	0	0	0	0
Rhodoferax	3	144	0	0	0	1	1	0	0	0	0	1	0	0	1	0	0	1	0	0
Rhodomicrobium	2	103	0	0	0	0	1	1	0	0	0	1	0	0	0	0	0	1	0	0
Rhodopirellula	2	87	0	0	0	0	0	0	0	0	0	1	0	1	0	0	0	1	0	0
Rhodopseudomonas	3	246	0	0	0	0	1	1	1	0	1	1	0	0	0	0	1	1	0	0
Rhodospirillum	2	121	0	0	0	0	1	0	0	0	0	0	0	0	0	0	0	1	0	0
Rhodothermus	2	76	0	0	0	0	0	0	0	0	0	0	0	0	0	0	0	1	0	0
Rhodovulum	1	4	0	0	0	0	0	0	0	0	0	0	0	0	0	0	0	0	0	0
Richelia	1	2	0	0	0	0	0	0	0	0	0	0	0	0	0	0	0	0	0	0
Rickettsia	2	43	0	0	0	0	0	0	0	0	0	0	0	0	0	0	0	0	0	0
Rickettsiaceae	1	5	0	0	0	0	0	0	0	0	0	0	0	0	0	0	0	0	0	0
Riemerella	2	61	0	0	0	0	0	0	0	0	0	0	0	0	0	0	0	1	0	0
Rivularia	1	18	0	0	0	0	0	0	0	0	0	1	0	0	0	0	0	1	0	0
Robiginitalea	2	83	0	0	0	0	0	0	0	0	0	0	0	0	0	0	0	1	0	0
Roseburia	2	58	0	0	0	0	0	0	0	0	0	0	0	0	0	0	0	1	0	0
Roseibium	1	3	0	0	0	0	0	0	0	0	0	0	0	0	0	0	0	0	0	0
Roseiflexus	3	100	0	0	0	1	0	0	0	0	0	1	0	0	0	0	0	1	0	0
Roseobacter	2	149	0	0	0	0	0	1	0	0	1	1	0	0	1	0	0	1	0	0
Roseomonas	1	2	0	0	0	0	0	0	0	0	0	0	0	0	0	0	0	0	0	0
Roseovarius	1	15	0	0	0	0	0	0	0	0	0	0	0	0	0	0	0	0	0	0
Rothia	1	44	0	0	0	0	0	0	0	0	0	0	0	0	0	0	0	1	0	0
Rubrivivax	1	24	0	0	0	0	1	0	0	0	0	0	0	0	0	0	0	1	0	0
Rubrobacter	3	117	0	0	0	0	0	1	0	0	0	0	0	1	1	0	0	1	0	0
Ruegeria	2	189	0	0	0	0	1	1	1	0	1	1	0	1	1	0	0	1	0	0

Genus	Cluster Number	Protein Counts	K01612	K01615	K01617	K01666	K01692	K01721	K01726	K01781	K01800	K01821	K01822	K01856	K01857	K01860	K01913	K01951	K02554	K03268
**Ruminococcaceae**	1	2	0	0	0	0	0	0	0	0	0	0	0	0	0	0	0	0	0	0
**Ruminococcus**	2	101	0	0	0	1	0	0	0	0	0	0	0	0	0	0	0	1	0	0
**Runella**	1	26	0	0	0	0	1	0	0	0	0	0	0	0	1	0	0	1	0	0
**Saccharomonospora**	2	132	0	0	0	0	1	0	0	0	0	0	0	0	1	0	0	1	0	0
**Saccharophagus**	2	81	0	0	0	0	0	0	0	0	0	0	0	0	0	0	0	1	0	0
**Saccharopolyspora**	3	194	0	0	0	0	1	0	0	0	0	0	1	1	1	0	1	1	0	0
**Saccharothrix**	1	32	0	0	0	0	1	0	0	0	0	0	0	0	1	0	0	0	0	0
**Sagittula**	1	10	0	0	0	0	0	0	0	0	0	0	0	0	0	0	0	0	0	0
**Salinibacter**	2	77	0	0	0	0	0	0	0	0	0	0	0	0	0	0	0	1	0	0
**Salinimonas**	1	1	0	0	0	0	0	0	0	0	0	0	0	0	0	0	0	0	0	0
**Salinispora**	3	127	0	0	1	1	1	0	0	0	0	1	1	0	1	0	0	1	0	0
**Salinivibrio**	1	2	0	0	0	0	0	0	0	0	0	0	0	0	0	0	0	0	0	0
**Salmonella**	2	151	0	0	0	0	0	0	0	0	0	0	0	0	0	0	0	1	0	0
**Sanguibacter**	2	73	0	0	0	0	0	0	0	0	0	0	0	0	0	0	0	1	0	0
**Saprospira**	1	13	0	0	0	0	0	0	0	0	0	0	0	0	0	0	0	1	0	0
**Scardovia**	1	1	0	0	0	0	0	0	0	0	0	0	0	0	0	0	0	0	0	0
**Scytonema**	1	10	0	0	0	0	0	0	0	0	0	0	0	0	0	0	0	0	0	0
**Sebaldella**	2	69	0	0	0	0	0	0	0	0	0	1	0	0	0	0	0	1	0	0
**Segniliparus**	2	85	0	0	0	0	1	0	0	0	0	1	0	0	0	0	0	1	0	0
**Selenomonas**	2	57	0	0	0	1	0	0	0	0	0	0	0	0	0	0	0	1	0	0
**Serinicoccus**	1	1	0	0	0	0	0	0	0	0	0	0	0	0	0	0	0	0	0	0
**Serratia**	2	186	0	0	0	1	1	1	0	0	0	1	0	1	1	0	0	1	0	0
**Shewanella**	3	214	0	0	1	1	1	0	1	0	0	1	1	0	0	0	0	1	0	0
**Shigella**	2	172	0	0	0	1	1	0	0	0	0	1	0	0	0	0	0	1	1	0
**Shinella**	1	2	0	0	0	0	0	0	0	0	0	0	0	0	0	0	0	0	0	0
**Sideroxydans**	2	81	0	0	0	0	0	0	0	0	0	1	0	0	0	0	0	1	0	0
**Silicibacter**	2	105	0	0	0	0	0	0	0	0	0	0	0	0	0	0	0	1	0	0
**Simiduia**	1	18	0	0	0	0	1	0	0	0	0	0	0	0	0	0	0	1	0	0
**Simkania**	1	6	0	0	0	0	0	0	0	0	0	0	0	0	0	0	0	0	0	0
**Simonsiella**	1	2	0	0	0	0	0	0	0	0	0	0	0	0	0	0	0	1	0	0
**Singulisphaera**	1	18	0	0	0	0	0	0	0	0	0	0	0	1	0	0	0	1	0	0
**Sinorhizobium**	3	207	0	0	0	0	1	1	0	0	1	1	0	1	1	0	0	1	0	0
**Slackia**	2	46	0	0	0	0	0	0	0	0	0	0	0	0	0	0	0	1	0	0
**Smaragdicoccus**	1	3	0	0	0	0	0	0	0	0	0	0	0	0	0	0	0	0	0	0
**Sodalis**	2	71	0	0	0	0	0	0	0	0	0	0	0	0	0	0	0	1	0	0
**Solibacillus**	1	23	0	0	0	1	0	0	0	0	0	1	0	0	0	0	0	1	1	0
**Solitalea**	1	13	0	0	0	0	0	0	0	0	0	0	0	0	0	0	0	1	0	0
**Sorangium**	3	115	0	0	0	0	0	0	0	0	0	0	0	0	0	0	0	1	0	0
**Sphaerobacter**	2	100	0	0	0	0	1	0	0	0	0	0	0	0	1	0	0	1	0	0
**Sphaerochaeta**	1	42	0	0	0	0	0	0	0	0	0	0	0	0	0	0	0	1	0	0
**Sphingobacterium**	3	85	0	0	0	0	0	0	0	0	0	0	0	0	0	0	0	1	0	0
**Sphingobium**	1	179	0	0	1	1	1	0	0	0	0	1	0	1	0	1	0	1	1	0
**Sphingomonadaceae**	1	15	0	0	0	0	0	0	0	0	0	0	0	0	0	0	0	0	0	0
**Sphingomonas**	2	220	0	0	1	1	1	0	0	0	1	1	1	1	1	0	0	1	1	0
**Sphingopyxis**	3	99	0	0	0	0	1	0	0	0	1	1	0	0	0	0	0	1	1	0
**Spirochaeta**	2	103	0	0	0	1	0	0	0	0	0	0	0	0	0	0	0	1	0	0
**Spiroplasma**	1	6	0	0	0	0	0	0	0	0	0	0	0	0	0	0	0	1	0	0
**Spirosoma**	2	107	0	0	0	0	1	0	0	0	0	0	0	0	1	0	0	1	0	0
**Stackebrandtia**	2	110	0	0	0	0	1	0	0	0	0	0	0	0	1	0	0	1	0	0
**Stanieria**	1	17	0	0	0	0	0	0	0	0	0	0	0	0	0	0	0	1	0	0
**Staphylococcus**	2	108	0	0	0	0	0	0	0	0	0	1	0	0	0	0	0	1	0	0
**Staphylothermus**	1	12	0	0	0	0	0	0	0	0	0	0	0	0	0	0	0	1	0	0

Genus	Cluster Number	Protein Counts	K01612	K01615	K01617	K01666	K01692	K01721	K01726	K01781	K01800	K01821	K01822	K01856	K01857	K01860	K01913	K01951	K02554	K03268
Stappia	1	2	0	0	0	0	0	0	0	0	0	0	0	0	0	0	0	0	0	0
Starkeya	3	134	0	0	0	0	1	0	0	0	0	1	0	0	0	0	0	1	0	0
Stenotrophomonas	3	104	0	0	0	0	0	0	0	0	1	0	0	0	0	0	0	1	0	0
Stigmatella	3	129	0	0	1	0	1	0	0	0	0	0	0	0	1	0	0	1	0	0
Streptobacillus	1	38	0	0	0	0	0	0	0	0	0	0	0	0	0	0	0	1	0	0
Streptococcus	2	121	0	0	0	0	0	0	0	0	0	1	0	0	0	0	0	1	0	0
Streptomyces	2	253	0	0	1	1	1	1	0	0	0	1	1	1	1	0	1	1	1	0
Streptosporangium	3	148	0	0	1	1	1	0	0	0	0	0	0	0	1	0	0	1	1	0
Strigomonas	1	2	0	0	0	0	0	0	0	0	0	0	0	0	0	0	0	1	0	0
Sulfitobacter	1	10	0	0	0	0	0	0	0	0	0	0	0	0	0	0	0	0	0	0
Sulfobacillus	1	30	0	0	1	1	1	0	0	0	0	1	0	0	0	0	0	1	0	0
Sulfolobus	1	23	0	0	0	0	0	0	0	0	0	0	0	0	0	0	0	1	0	0
Sulfuricurvum	2	67	0	0	0	0	0	0	0	0	0	0	0	0	0	0	0	1	0	0
Sulfurihydrogenibium	2	55	0	0	0	0	0	0	0	0	0	1	0	0	0	0	0	1	0	0
Sulfurimonas	2	60	0	0	0	0	0	0	0	0	0	0	0	0	0	0	0	1	0	0
Sulfurospirillum	1	48	0	0	0	0	0	0	0	0	0	1	0	0	0	0	0	1	0	0
Sulfurovum	2	48	0	0	0	1	0	0	0	0	0	0	0	0	0	0	0	1	0	0
Symbiobacterium	2	68	0	0	0	0	1	0	0	0	0	1	0	0	0	0	0	1	0	0
Synechococcus	1	34	0	0	0	1	0	1	0	0	0	0	0	0	0	0	0	1	0	0
Synechocystis	1	40	0	0	0	0	0	0	0	0	0	0	0	0	0	0	0	1	0	0
Synergistetes	1	27	0	0	0	0	0	0	0	0	0	0	0	0	0	0	0	0	0	0
Syntrophobacter	2	101	0	0	0	0	0	0	0	0	0	1	0	0	0	0	0	1	0	0
Syntrophobotulus	2	71	0	0	0	0	0	0	0	0	0	1	0	0	0	0	0	1	0	0
Syntrophomonas	2	58	0	0	0	0	0	0	0	0	0	0	0	0	0	0	0	1	0	0
Syntrophothermus	2	60	0	0	0	0	0	0	0	0	0	0	0	0	0	0	0	1	0	0
Syntrophus	2	66	0	0	0	0	0	0	0	0	0	0	0	0	0	0	0	1	0	0
Tannerella	1	8	0	0	0	0	0	0	0	0	0	0	0	0	0	0	0	1	0	0
Taylorella	1	43	0	0	0	0	0	0	0	0	0	0	0	0	0	0	0	1	1	0
Tepidanaerobacter	2	55	0	0	0	0	0	0	0	0	0	0	0	0	0	0	0	1	0	0
Teredinibacter	2	109	0	0	0	1	0	0	0	0	0	0	0	0	0	0	0	1	1	0
Terrabacter	1	1	0	0	0	0	0	0	0	0	0	0	0	0	0	0	0	0	0	0
Terriglobus	2	83	0	0	0	0	0	0	0	0	0	1	0	0	0	0	0	1	0	0
Tetragenococcus	1	12	0	0	0	0	0	0	0	0	0	0	0	0	0	0	0	1	0	0
Thalassiobium	1	11	0	0	0	0	0	0	0	0	0	0	0	0	0	0	0	0	0	0
Thalassobacter	1	9	0	0	0	0	0	0	0	0	0	0	0	0	0	0	0	0	0	0
Thalassolituus	1	12	0	0	0	0	0	0	0	0	0	0	0	0	0	0	0	1	0	0
Thalassospira	1	2	0	0	0	0	0	0	0	0	0	0	0	0	0	0	0	0	0	0
Thauera	1	111	0	0	1	1	1	0	0	0	0	1	0	0	0	0	0	1	1	0
Thermacetogenium	1	7	0	0	0	0	0	0	0	0	0	0	0	0	0	0	0	1	0	0
Thermaerobacter	2	79	0	0	0	1	1	0	0	0	0	1	0	0	0	0	0	1	0	0
Thermanaerovibrio	2	58	0	0	0	0	0	0	0	0	0	1	0	0	0	0	0	1	0	0
Thermincola	2	62	0	0	0	0	0	0	0	0	0	0	0	0	0	0	0	1	0	0
Thermoanaerobacter	2	76	0	0	0	0	0	0	0	0	0	1	0	0	0	0	0	1	0	0
Thermoanaerobacterium	2	64	0	0	0	0	0	0	0	0	0	0	0	0	0	0	0	1	0	0
Thermobacillus	1	11	0	0	0	1	0	0	0	0	0	0	0	0	0	0	0	1	0	0
Thermobaculum	2	62	0	0	0	0	0	0	0	0	0	0	0	0	0	0	0	1	0	0
Thermobifida	2	72	0	0	0	0	1	0	0	0	0	0	0	0	0	0	0	1	0	0
Thermobispora	2	109	0	0	0	0	1	0	0	0	0	0	0	0	1	0	0	1	0	0
Thermococcus	1	13	0	0	0	0	0	0	0	0	0	1	0	0	0	0	0	1	0	0
Thermocrinis	2	60	0	0	0	0	0	0	0	0	0	0	0	0	0	0	0	1	0	0
Thermodesulfatator	1	10	0	0	0	0	0	0	0	0	0	0	0	0	0	0	0	1	0	0
Thermodesulfobacterium	1	9	0	0	0	0	0	0	0	0	0	0	0	0	0	0	0	1	0	0

Genus	Cluster Number	Protein Counts	K01612	K01615	K01617	K01666	K01692	K01721	K01726	K01781	K01800	K01821	K01822	K01856	K01857	K01860	K01913	K01951	K02554	K03268
Thermodesulfobium	1	13	0	0	0	0	0	0	0	0	0	1	0	0	0	0	0	1	0	0
Thermodesulfovibrio	2	60	0	0	0	0	0	0	0	0	0	0	0	0	0	0	0	1	0	0
Thermofilum	1	9	0	0	0	0	0	0	0	0	0	0	0	0	0	0	0	0	0	0
Thermogladius	1	7	0	0	0	0	0	0	0	0	0	0	0	0	0	0	0	0	0	0
Thermomicrobium	2	92	0	0	0	1	1	0	0	0	0	1	0	0	0	0	0	1	0	0
Thermomonospora	2	125	0	0	1	1	1	0	0	0	0	1	0	0	1	0	0	1	0	0
Thermoplasma	1	11	0	0	0	0	0	0	0	0	0	0	0	0	0	0	0	1	0	0
Thermoplasmatales	1	6	0	0	0	1	0	0	0	0	0	0	0	0	0	0	0	1	0	0
Thermoproteus	1	12	0	0	0	0	0	0	0	0	0	0	0	0	0	0	0	1	0	0
Thermosediminibacter	2	64	0	0	0	1	0	0	0	0	0	0	0	0	0	0	0	1	0	0
Thermosipho	2	70	0	0	0	0	0	0	0	0	0	0	0	0	0	0	0	1	0	0
Thermosphaera	1	10	0	0	0	0	0	0	0	0	0	1	0	0	0	0	0	1	0	0
Thermosynechococcus	1	6	0	0	0	0	0	0	0	0	0	0	0	0	0	0	0	1	0	0
Thermotoga	2	80	0	0	0	0	0	0	0	0	0	0	0	0	0	0	0	1	0	0
Thermovibrio	2	58	0	0	0	0	0	0	0	0	0	0	0	0	0	0	0	1	0	0
Thermovirga	1	9	0	0	0	0	0	0	0	0	0	1	0	0	0	0	0	1	0	0
Thermus	2	107	0	0	0	1	1	0	0	0	0	1	0	1	0	0	0	1	1	0
Thioalkalimicrobium	1	4	0	0	0	1	0	0	0	0	0	0	0	0	0	0	0	1	0	0
Thioalkalivibrio	2	91	0	0	0	0	0	0	0	0	0	1	0	0	0	0	0	1	0	0
Thiobaca	1	2	0	0	0	0	0	0	0	0	0	0	0	0	0	0	0	0	0	0
Thiobacillus	2	67	0	0	0	0	0	0	0	0	0	1	0	0	0	0	0	1	0	0
Thiocapsa	1	2	0	0	0	0	0	0	0	0	0	0	0	0	0	0	0	0	0	0
Thiocystis	1	11	0	0	0	1	0	0	0	0	0	0	0	0	0	0	0	1	0	0
Thioflavicoccus	1	13	0	0	0	1	0	0	0	0	0	0	0	0	0	0	0	1	0	0
Thiomicrospira	2	50	0	0	0	0	0	0	0	0	0	0	0	0	0	0	0	1	0	0
Thiomonas	2	85	0	0	0	0	1	0	0	0	0	1	0	0	0	0	0	1	0	0
Thiorhodococcus	1	2	0	0	0	0	0	0	0	0	0	0	0	0	0	0	0	0	0	0
Thiothrix	1	2	0	0	0	0	0	0	0	0	0	0	0	0	0	0	0	0	0	0
Tistrella	1	42	0	0	0	1	1	1	0	0	0	1	0	1	1	0	0	1	0	0
Tolumonas	2	100	0	0	0	0	0	0	0	0	0	1	0	0	0	0	0	1	0	0
Transposon	1	2	0	0	0	0	0	0	0	0	0	0	0	0	0	0	0	0	0	0
Treponema	1	63	0	0	0	1	0	0	0	0	0	1	0	0	0	0	0	1	1	0
Trichodesmium	2	71	0	0	0	0	0	0	0	0	0	0	0	0	0	0	0	1	0	0
Trichormus	1	12	0	0	0	0	0	0	0	0	0	0	0	0	0	0	0	1	0	0
Tropheryma	2	22	0	0	0	0	0	0	0	0	0	0	0	0	0	0	0	1	0	0
Truepera	2	104	0	0	0	0	0	0	0	0	0	0	0	0	0	0	0	1	0	0
Tsukamurella	2	123	0	0	0	1	1	0	0	0	0	0	0	0	1	0	0	1	0	0
Turneriella	1	16	0	0	0	0	1	0	0	0	0	1	0	0	0	0	0	1	0	0
Uliginosibacterium	1	2	0	0	0	0	0	0	0	0	0	0	0	0	0	0	0	0	0	0
Ureaplasma	1	24	0	0	0	0	0	0	0	0	0	0	0	0	0	0	0	0	0	0
Ureibacillus	1	2	0	0	0	0	0	0	0	0	0	0	0	0	0	0	0	0	0	0
Variovorax	3	187	0	0	0	0	1	1	0	0	0	1	0	1	1	0	0	1	0	0
Veillonella	1	54	0	1	0	0	0	0	0	0	0	1	0	0	0	0	0	1	0	0
Verminephrobacter	3	188	0	0	1	1	1	0	0	0	0	1	0	0	1	0	0	1	1	0
Verrucosispora	3	126	0	0	1	1	1	0	0	0	0	0	0	0	1	0	0	1	0	0
Vibrio	2	195	0	0	0	1	0	0	1	0	1	0	0	1	1	0	0	1	1	0
Vibrionales	1	46	0	0	0	0	0	0	0	0	0	0	0	0	0	0	0	1	0	0
Vitreoscilla	1	1	0	0	0	0	0	0	0	0	0	0	0	0	0	0	0	0	0	0
Vulcanisaeta	1	14	0	0	0	0	1	0	0	0	0	0	0	0	0	0	0	1	0	0
Waddlia	2	68	0	0	0	0	0	0	0	0	0	0	0	0	0	0	0	1	0	0
Wautersia	1	2	0	0	0	0	0	0	0	0	0	0	0	0	0	0	0	0	0	0
Weeksella	2	65	0	0	0	0	0	0	0	0	0	0	0	0	0	0	0	1	0	0

Genus	Cluster Number	Protein Counts	K01612	K01615	K01617	K01666	K01692	K01721	K01726	K01781	K01800	K01821	K01822	K01856	K01857	K01860	K01913	K01951	K02554	K03268
**Weissella**	1	14	0	0	0	0	0	0	0	0	0	1	0	0	0	0	0	1	0	0
**Wenxinia**	1	2	0	0	0	0	0	0	0	0	0	0	0	0	0	0	0	0	0	0
**Wigglesworthia**	1	29	0	0	0	0	0	0	0	0	0	0	0	0	0	0	0	1	0	0
**Wolbachia**	1	32	0	0	0	0	0	0	0	0	0	0	0	0	0	0	0	1	0	0
**Wolinella**	2	51	0	0	0	0	0	0	0	0	0	1	0	0	0	0	0	1	0	0
**Xanthobacter**	3	176	0	0	0	1	1	0	0	0	0	1	1	1	1	0	0	1	1	0
**Xanthobacteraceae**	1	4	0	0	0	0	0	0	0	0	0	0	0	0	0	0	0	0	0	0
**Xanthomonadaceae**	1	39	0	0	0	0	0	0	0	0	0	0	0	0	0	0	0	1	0	0
**Xanthomonas**	2	162	0	0	0	0	0	0	0	0	1	0	0	0	1	0	0	1	1	0
**Xenorhabdus**	2	127	0	0	0	1	1	0	0	0	0	1	0	0	0	0	0	1	0	0
**Xylanimonas**	2	66	0	0	0	0	0	0	0	0	0	0	0	0	0	0	0	1	0	0
**Xylella**	2	52	0	0	0	0	0	0	0	0	0	1	0	0	0	0	0	1	0	0
**Yersinia**	2	145	0	0	0	0	0	0	1	0	0	1	0	0	0	0	0	1	0	0
**Yokenella**	2	61	0	0	0	0	0	0	0	0	0	0	0	0	0	0	0	1	0	0
**Zobellia**	1	28	0	0	0	0	0	0	0	0	0	0	0	0	0	0	0	1	0	0
**Zunongwangia**	2	98	0	0	0	0	0	0	0	0	0	0	0	0	0	0	0	1	0	0
**Zymomonas**	2	67	0	0	0	0	0	0	0	1	0	0	0	0	0	0	0	1	0	0
**Zymophilus**	1	2	0	0	0	0	0	0	0	0	0	0	0	0	0	0	0	0	0	0

Genus	Cluster Number	Protein Counts	K03333	K03379	K03380	K03381	K03382	K03383	K03384	K03391	K03464	K03518	K03862	K04098	K04099	K04100	K04102	K04105	K04107	K04110
**Acaricomes**	1	2	0	0	0	0	0	0	0	0	0	0	0	0	0	0	0	0	0	0
**Acaryochloris**	2	88	0	0	0	0	0	0	0	0	0	0	0	0	0	0	0	0	0	0
**Acetivibrio**	1	2	0	0	0	0	0	0	0	0	0	0	0	0	0	0	0	0	0	0
**Acetobacter**	2	85	0	0	0	0	1	0	0	0	0	0	1	0	0	0	0	0	0	0
**Acetobacteraceae**	1	2	0	0	0	0	0	0	0	0	0	0	0	0	0	0	0	0	0	0
**Acetobacterium**	1	8	0	0	0	0	0	0	0	0	0	1	0	0	0	0	0	0	0	0
**Acetohalobium**	2	79	0	0	0	0	0	0	0	0	0	1	0	0	0	0	0	0	0	0
**Acholeplasma**	2	38	0	0	0	0	0	0	0	0	0	0	0	0	0	0	0	0	0	0
**Achromobacter**	2	192	0	0	0	1	0	0	0	0	1	1	0	0	0	0	0	0	0	1
**Acidaminococcus**	1	65	0	0	0	0	0	0	0	0	0	0	0	0	0	0	0	0	0	0
**Acidianus**	1	9	0	0	0	0	0	0	0	0	0	1	0	0	0	0	0	0	0	0
**Acidilobus**	1	8	0	0	0	0	0	0	0	0	0	0	0	0	0	0	0	0	0	0
**Acidimicrobium**	2	58	0	0	0	0	0	0	0	0	0	1	0	0	0	0	0	0	0	0
**Acidiphilium**	3	144	0	0	0	0	0	0	0	0	0	1	0	0	0	0	0	0	0	0
**Acidithiobacillus**	2	70	0	0	0	0	0	1	0	0	0	1	0	0	0	0	0	0	0	0
**Acidobacterium**	2	91	0	0	0	0	0	0	0	0	0	0	0	0	0	0	0	0	0	0
**Acidocella**	1	2	0	0	0	0	0	0	0	0	0	0	0	0	0	0	0	0	0	0
**Acidothermus**	2	68	0	0	0	0	0	0	0	0	0	1	0	0	0	0	0	0	0	0
**Acidovorax**	3	217	0	1	0	1	1	0	0	0	1	1	1	1	1	1	0	0	0	1
**Aciduliprofundum**	1	11	0	0	0	0	0	0	0	0	0	0	0	0	0	0	0	0	0	0
**Acinetobacter**	3	188	1	1	0	1	1	0	0	0	1	0	1	0	0	0	0	0	0	0
**Actinobacillus**	2	112	0	0	0	0	0	0	0	0	0	0	0	0	0	0	0	0	0	0
**Actinomadura**	1	1	0	0	0	0	0	0	0	0	0	0	0	0	0	0	0	0	0	0
**Actinoplanes**	1	52	1	0	0	0	1	0	0	0	0	1	0	0	0	0	0	0	0	0
**Actinopolyspora**	1	2	0	0	0	0	0	0	0	0	0	0	0	0	0	0	0	0	0	0
**Actinosynnema**	2	122	1	0	0	0	0	0	0	0	0	0	0	0	0	0	0	0	0	0
**Advenella**	1	30	0	0	0	0	0	0	0	0	1	1	1	0	0	0	0	0	0	1
**Aequorivita**	1	12	0	0	0	0	0	0	0	0	0	0	0	0	0	0	0	0	0	0
**Aerococcus**	2	57	0	0	0	0	0	0	0	0	0	0	0	0	0	0	0	0	0	0
**Aeromonas**	2	127	0	0	0	0	0	0	0	0	0	0	0	0	0	0	0	0	0	0
**Aeropyrum**	1	13	0	0	0	0	0	0	0	0	0	1	0	0	0	0	0	0	0	0
**Afipia**	1	54	0	0	0	0	0	0	0	0	0	1	0	0	0	0	0	0	0	0
**Aggregatibacter**	1	81	0	0	0	0	0	0	0	0	0	0	0	0	0	0	0	0	0	0
**Agrobacterium**	2	242	1	0	1	1	0	1	0	0	1	1	1	0	0	1	1	0	0	0
**Ahrensia**	1	2	0	0	0	0	0	0	0	0	0	0	0	0	0	0	0	0	0	0
**Akkermansia**	2	47	0	0	0	0	0	0	0	0	0	0	0	0	0	0	0	0	0	0
**Albidiferax**	3	144	1	0	0	0	0	0	0	0	0	0	1	0	0	1	0	0	0	1
**Alcaligenes**	1	21	0	0	0	0	0	0	0	0	0	0	0	0	0	0	0	0	0	0
**Alcanivorax**	2	146	1	0	0	0	1	1	0	1	0	0	1	0	0	0	0	0	0	0
**Alicycliphilus**	3	152	0	0	0	1	0	0	0	0	1	1	0	0	0	0	0	0	0	0
**Alicyclobacillus**	2	91	0	0	0	0	0	0	0	0	0	0	0	0	0	0	0	0	0	0
**Aliivibrio**	2	118	0	0	0	0	0	0	0	0	0	0	0	0	0	0	0	0	0	0
**Alishewanella**	1	1	0	0	0	0	0	0	0	0	0	0	0	0	0	0	0	0	0	0
**Alistipes**	1	52	0	0	0	0	0	0	0	0	0	0	0	0	0	0	0	0	0	0
**Alkalilimnicola**	2	97	0	0	0	0	0	0	0	0	0	1	0	0	0	0	0	0	0	0
**Alkaliphilus**	2	62	0	0	0	0	0	0	0	0	0	1	0	0	0	0	0	0	0	0
**Allochromatium**	2	84	0	0	0	0	0	0	0	0	0	0	0	0	0	0	0	0	0	0
**Alphaproteobacteria**	1	3	0	0	0	0	0	0	0	0	0	0	0	0	0	0	0	0	0	0
**Alteromonadales**	1	12	0	0	0	0	0	0	0	0	0	0	0	0	0	0	0	0	0	0
**Alteromonas**	1	37	0	0	0	1	1	0	0	0	1	0	0	0	0	1	0	0	0	0
**Aminobacter**	1	4	0	0	0	0	1	0	0	0	0	0	0	0	0	0	0	0	0	0
**Aminobacterium**	2	59	0	0	0	0	0	0	0	0	0	0	0	0	0	0	0	0	0	0

Genus	Cluster Number	Protein Counts	K03333	K03379	K03380	K03381	K03382	K03383	K03384	K03391	K03464	K03518	K03862	K04098	K04099	K04100	K04102	K04105	K04107	K04110
**Ammonifex**	2	50	0	0	0	0	0	0	0	0	0	0	0	0	0	0	0	0	0	0
**Amphibacillus**	1	13	0	0	0	0	0	0	0	0	0	0	0	0	0	0	0	0	0	0
**Amphritea**	1	2	0	0	0	0	0	0	0	0	0	0	0	0	0	0	0	0	0	0
**Amycolatopsis**	2	201	1	0	0	1	1	0	0	0	1	1	1	0	0	0	1	0	0	1
**Amycolicicoccus**	1	43	1	0	0	0	1	0	0	0	0	0	1	0	0	0	0	0	0	0
**Anabaena**	2	92	0	0	0	0	0	0	0	0	1	0	0	0	0	0	0	0	0	0
**Anaerobaculum**	1	11	0	0	0	0	0	0	0	0	0	1	0	0	0	0	1	0	0	0
**Anaerococcus**	2	46	0	0	0	0	0	0	0	0	0	0	0	0	0	0	0	0	0	0
**Anaerolinea**	2	66	0	0	0	0	1	0	0	0	0	1	0	0	0	0	0	0	0	0
**Anaeromyxobacter**	3	117	1	0	0	0	0	0	0	0	0	0	0	0	0	0	0	0	0	0
**Anaerostipes**	1	44	0	0	0	0	0	0	0	0	0	1	0	0	0	0	0	0	0	0
**Anaerotruncus**	1	2	0	0	0	0	0	0	0	0	0	0	0	0	0	0	0	0	0	0
**Anaplasma**	1	35	0	0	0	0	0	0	0	0	0	0	0	0	0	0	0	0	0	0
**Ancylobacter**	1	3	0	0	0	0	0	0	0	0	0	0	0	0	0	0	0	0	0	0
**Aneurinibacillus**	1	1	0	0	0	0	0	0	0	0	0	0	0	0	0	0	0	0	0	0
**Angomonas**	1	1	0	0	0	0	0	0	0	0	0	0	0	0	0	0	0	0	0	0
**Anoxybacillus**	2	72	0	0	0	0	0	0	0	0	0	0	0	0	0	0	0	0	0	0
**Aquifex**	2	45	0	0	0	0	0	0	0	0	0	0	0	0	0	0	0	0	0	0
**Aquimarina**	1	2	0	0	0	0	0	0	0	0	0	0	0	0	0	0	0	0	0	0
**Arcanobacterium**	2	45	0	0	0	0	0	0	0	0	0	0	0	0	0	0	0	0	0	0
**Archaeoglobus**	1	14	0	0	0	0	0	0	0	0	0	0	0	0	0	0	0	0	0	0
**Arcobacter**	2	101	0	0	0	0	0	0	0	0	0	0	0	0	0	0	0	0	0	0
**Aromatoleum**	3	133	0	0	0	0	0	0	0	0	0	0	0	0	0	0	0	0	1	1
**Arsenophonus**	1	4	0	0	0	0	0	0	0	0	0	0	0	0	0	0	0	0	0	0
**Arthrobacter**	2	206	1	0	1	1	1	0	0	0	1	1	1	1	1	0	0	0	0	0
**Arthrospira**	2	67	0	0	0	0	0	0	0	0	0	0	0	0	0	0	0	0	0	0
**Aster**	1	10	0	0	0	0	0	0	0	0	0	0	0	0	0	0	0	0	0	0
**Asticcacaulis**	2	103	0	0	0	0	0	0	0	0	0	0	0	0	1	0	0	0	0	0
**Atopobium**	1	28	0	0	0	0	0	0	0	0	0	0	0	0	0	0	0	0	0	0
**Aurantimonas**	1	2	0	0	0	0	0	0	0	0	0	0	0	0	0	0	0	0	0	0
**Avibacterium**	1	17	0	0	0	0	0	0	0	0	0	0	0	0	0	0	0	0	0	0
**Azoarcus**	3	172	0	0	0	0	0	0	1	0	0	0	0	0	0	1	0	0	0	1
**Azorhizobium**	3	149	0	0	0	0	0	1	0	0	1	1	0	1	0	0	0	0	0	0
**Azorhizophilus**	1	4	0	0	0	0	0	0	0	0	0	0	0	0	0	0	0	0	0	0
**Azospira**	1	19	0	0	0	0	0	0	0	0	0	0	0	0	0	0	0	0	0	0
**Azospirillum**	2	198	0	0	0	1	0	0	0	0	1	1	1	0	0	1	0	0	0	0
**Azotobacter**	3	183	0	0	0	0	0	0	0	0	0	0	0	0	1	1	0	0	0	0
**Bacillales**	1	9	0	0	0	0	0	0	0	0	0	0	0	0	0	0	0	0	0	0
**Bacilli**	1	3	0	0	0	0	0	0	0	0	0	0	0	0	0	0	0	0	0	0
**Bacillus**	2	237	0	0	0	0	0	1	0	0	0	1	1	0	0	0	0	0	0	0
**Bacteria**	2	86	0	0	0	0	1	0	0	0	1	0	0	0	0	0	0	0	0	0
**Bacteriovorax**	2	98	1	0	0	0	0	0	0	0	0	0	0	0	0	0	0	0	0	0
**Bacteroidales**	2	59	0	0	0	0	0	0	0	0	0	0	0	0	0	0	0	0	0	0
**Bacteroides**	2	96	0	0	0	0	0	0	0	0	0	0	0	0	0	0	0	0	0	0
**Bacteroidetes**	1	2	0	0	0	0	0	0	0	0	0	0	0	0	0	0	0	0	0	0
**Bartonella**	2	50	0	0	0	0	0	0	0	0	0	0	0	0	0	0	0	0	0	0
**Baumannia**	2	24	0	0	0	0	0	0	0	0	0	0	0	0	0	0	0	0	0	0
**Bdellovibrio**	2	87	1	0	0	0	0	0	0	0	0	0	0	0	0	0	0	0	0	0
**Beijerinckia**	3	123	0	0	0	0	0	0	0	0	0	0	0	0	0	0	0	0	0	0
**Belliella**	1	14	0	0	0	0	0	0	0	0	0	0	0	0	0	0	0	0	0	0
**Beutenbergia**	2	82	0	0	0	0	1	0	0	0	0	1	0	0	0	0	0	0	0	0
**Bifidobacterium**	2	61	0	0	0	0	0	0	0	0	0	0	0	0	0	0	0	0	0	0

Genus	Cluster Number	Protein Counts	K03333	K03379	K03380	K03381	K03382	K03383	K03384	K03391	K03464	K03518	K03862	K04098	K04099	K04100	K04102	K04105	K04107	K04110
Bizionia	1	1	0	0	0	0	0	0	0	0	0	0	0	0	0	0	0	0	0	0
Blastococcus	1	39	0	0	1	1	0	1	0	0	1	1	0	0	0	1	0	0	0	1
Blattabacterium	1	23	0	0	0	0	0	0	0	0	0	0	0	0	0	0	0	0	0	0
Blautia	1	22	0	0	0	0	0	0	0	0	0	1	0	0	0	0	0	0	0	0
Bordetella	3	227	0	0	0	1	0	0	1	0	1	1	0	0	0	0	1	0	0	1
Borrelia	1	32	0	0	0	0	0	0	0	0	0	0	0	0	0	0	0	0	0	0
Brachybacterium	1	18	0	0	0	0	0	0	0	0	0	0	0	0	0	0	0	0	0	0
Brachymonas	1	4	0	0	0	0	0	0	0	0	0	0	0	0	0	0	0	0	0	0
Brachyspira	2	79	0	0	0	0	1	0	0	0	0	1	0	0	0	0	0	0	0	0
Bradyrhizobiaceae	1	20	0	0	0	0	0	0	0	0	0	1	0	0	0	0	0	0	0	0
Bradyrhizobium	3	254	0	0	1	1	1	1	1	0	0	1	1	0	1	1	1	1	0	1
Brenneria	1	20	0	0	0	0	0	0	0	0	0	0	0	0	0	0	0	0	0	0
Brevibacillus	2	122	0	0	0	0	0	0	0	0	0	0	0	0	0	0	0	0	0	0
Brevibacterium	1	4	0	0	0	0	0	0	0	0	0	0	0	0	0	0	0	0	0	0
Brevundimonas	2	105	0	0	0	0	0	0	0	0	0	0	0	0	0	1	0	0	0	0
Brucella	3	138	0	0	0	0	0	0	0	0	0	0	0	0	0	0	0	0	0	0
Buchnera	1	26	0	0	0	0	0	0	0	0	0	0	0	0	0	0	0	0	0	0
Burkholderia	3	424	1	1	1	1	1	0	0	1	1	1	1	1	1	1	1	0	0	1
Burkholderiaceae	3	143	0	0	0	0	0	0	0	0	1	1	0	0	0	0	0	0	0	0
Burkholderiales	1	24	0	0	0	0	0	0	0	0	0	0	0	0	0	0	0	0	0	0
Buttiauxella	1	2	0	0	0	0	0	0	0	0	0	0	0	0	0	0	0	0	0	0
Butyrivibrio	2	67	0	0	0	0	0	0	0	0	0	0	0	0	0	0	0	0	0	0
Caldanaerobacter	2	63	0	0	0	0	0	0	0	0	0	0	0	0	0	0	0	0	0	0
Caldicellulosiruptor	2	75	0	0	0	0	0	0	0	0	0	0	0	0	0	0	0	0	0	0
Caldilinea	1	23	0	0	0	0	1	0	0	0	0	1	0	0	0	0	0	0	0	0
Caldimonas	1	6	0	0	0	0	0	0	0	0	0	0	0	0	0	0	0	0	0	0
Caldisericum	1	10	0	0	0	0	1	0	0	0	0	1	0	0	0	0	0	0	0	0
Caldisphaera	1	7	0	0	0	0	0	0	0	0	0	0	0	0	0	0	0	0	0	0
Calditerrivibrio	2	74	0	0	0	0	0	0	0	0	0	0	0	0	0	0	0	0	0	0
Caldivirga	1	13	0	0	0	0	0	0	0	0	0	0	0	0	0	0	0	0	0	0
Calothrix	1	43	1	0	0	0	0	0	0	0	0	0	0	0	0	0	0	0	0	0
Calyptogena	2	34	0	0	0	0	0	0	0	0	0	0	0	0	0	0	0	0	0	0
Campylobacter	2	80	0	0	0	0	0	0	0	0	0	0	0	0	0	0	0	0	0	0
Candidatus	2	290	1	1	1	0	1	0	0	0	0	1	1	0	0	1	0	0	0	0
Capnocytophaga	1	50	0	0	0	0	0	0	0	0	0	0	0	0	0	0	0	0	0	0
Carbophilus	1	2	0	0	0	0	0	0	0	0	0	0	0	0	0	0	0	0	0	0
Carboxydibrachium	1	14	0	0	0	0	0	0	0	0	0	0	0	0	0	0	0	0	0	0
Carboxydothermus	2	68	0	0	0	0	0	0	0	0	0	1	0	0	0	0	0	0	0	0
Carica	1	2	0	0	0	0	0	0	0	0	0	0	0	0	0	0	0	0	0	0
Carnobacterium	2	71	0	0	0	0	0	0	0	0	0	0	0	0	0	0	0	0	0	0
Catenibacterium	1	2	0	0	0	0	0	0	0	0	0	0	0	0	0	0	0	0	0	0
Catenovulum	1	2	0	0	0	0	0	0	0	0	0	0	0	0	0	0	0	0	0	0
Catenulispora	3	159	1	0	0	0	1	1	0	0	0	1	1	1	0	0	1	0	0	0
Caulobacter	3	168	0	0	0	0	0	0	0	0	0	0	1	0	0	0	0	0	0	0
Cedecea	1	45	0	0	0	0	0	0	0	0	0	0	0	0	0	0	0	0	0	0
Cellulomonas	2	96	1	0	1	0	0	0	0	0	0	0	0	0	0	0	0	0	0	0
Cellulophaga	2	105	0	0	0	0	0	0	0	0	0	0	0	0	0	0	0	0	0	0
Cellulosilyticum	1	14	0	0	0	0	0	0	0	0	0	0	0	0	0	0	0	0	0	0
Cellvibrio	2	85	1	0	0	0	1	0	0	0	0	0	0	0	0	0	0	0	0	0
Cenarchaeum	1	7	0	0	0	0	0	0	0	0	0	0	0	0	0	0	0	0	0	0
Chamaesiphon	1	13	0	0	0	0	0	0	0	0	0	0	0	0	0	0	0	0	0	0
Chelativorans	3	136	0	0	0	0	0	0	0	0	0	1	0	0	0	0	1	0	0	0

Genus	Cluster Number	Protein Counts	K03333	K03379	K03380	K03381	K03382	K03383	K03384	K03391	K03464	K03518	K03862	K04098	K04099	K04100	K04102	K04105	K04107	K04110
**Chelatococcus**	1	2	0	0	0	0	0	0	0	0	0	0	0	0	0	0	0	0	0	0
**Chitinophaga**	2	98	0	0	0	0	0	0	0	0	0	0	0	0	0	0	0	0	0	0
**Chlamydia**	1	36	0	0	0	0	0	0	0	0	0	0	0	0	0	0	0	0	0	0
**Chlamydomonas**	1	3	0	0	0	0	0	0	0	0	0	0	0	0	0	0	0	0	0	0
**Chlamydophila**	1	31	0	0	0	0	0	0	0	0	0	0	0	0	0	0	0	0	0	0
**Chlorobaculum**	2	61	0	0	0	0	0	0	0	0	0	0	0	0	0	0	0	0	0	0
**Chlorobium**	2	89	0	0	0	0	0	0	0	0	0	0	0	0	0	0	0	0	0	0
**Chlorobium/Pelodictyon**	1	2	0	0	0	0	0	0	0	0	0	0	0	0	0	0	0	0	0	0
**Chloroflexus**	2	105	0	0	0	0	0	0	0	0	0	1	0	0	0	0	0	0	0	0
**Chlorogloeopsis**	1	9	0	0	0	0	0	0	0	0	0	0	0	0	0	0	0	0	0	0
**Chloroherpeton**	2	59	0	0	0	0	0	0	0	0	0	0	0	0	0	0	0	0	0	0
**Chromobacterium**	2	117	1	0	0	0	0	0	0	0	0	0	0	0	0	0	0	0	0	0
**Chromohalobacter**	2	145	0	0	0	0	0	0	0	0	0	0	1	0	1	1	0	0	0	0
**Chroococcidiopsis**	1	24	1	0	0	0	1	0	0	0	0	1	0	0	0	0	0	0	0	0
**Chryseobacterium**	2	104	0	0	0	1	0	0	0	0	0	1	1	0	0	1	0	0	0	1
**Citreicella**	1	13	0	0	0	0	0	0	0	0	0	0	0	0	0	0	0	0	0	0
**Citrobacter**	2	190	0	0	0	0	0	0	0	0	0	1	0	0	1	0	0	0	0	0
**Clavibacter**	2	83	0	0	0	0	0	0	0	0	0	0	0	0	0	0	0	0	0	0
**Clonorchis**	1	1	0	0	0	0	0	0	0	0	0	0	0	0	0	0	0	0	0	0
**Clostridiales**	2	77	0	0	0	0	0	0	0	0	0	1	0	0	0	0	0	0	0	0
**Clostridium**	2	191	0	0	0	0	1	0	0	0	0	1	0	0	0	0	0	0	0	0
**Cohnella**	1	2	0	0	0	0	0	0	0	0	0	0	0	0	0	0	0	0	0	0
**Coleofasciculus**	1	4	0	0	0	0	0	0	0	0	0	0	0	0	0	0	0	0	0	0
**Collimonas**	1	35	0	0	0	0	1	0	0	0	0	0	1	0	0	0	0	0	0	0
**Colwellia**	2	130	0	0	0	0	0	0	0	0	0	0	0	0	0	0	0	0	0	0
**Comamonas**	2	196	0	0	1	0	1	0	0	0	0	1	1	0	0	1	1	0	0	1
**Conexibacter**	3	117	1	0	0	0	1	0	0	0	1	1	0	0	0	0	0	0	0	0
**Coprobacillus**	1	2	0	0	0	0	0	0	0	0	0	0	0	0	0	0	0	0	0	0
**Coprococcus**	2	76	0	0	0	0	0	0	0	0	0	1	0	0	0	0	0	0	0	0
**Coprothermobacter**	2	45	0	0	0	0	0	0	0	0	0	0	0	0	0	0	0	0	0	0
**Coraliomargarita**	2	68	0	0	0	0	0	0	0	0	0	0	0	0	0	0	1	0	0	0
**Corallococcus**	1	29	1	0	0	0	0	0	0	0	0	0	0	0	0	0	0	0	0	0
**Coriobacterium**	2	36	0	0	0	0	0	0	0	0	0	0	0	0	0	0	0	0	0	0
**Corynebacterineae**	1	5	0	0	0	0	0	0	0	0	0	0	0	0	0	0	0	0	0	0
**Corynebacterium**	2	175	1	0	1	1	1	0	0	0	1	0	1	1	0	0	0	0	0	0
**Coxiella**	2	54	0	0	0	0	0	0	0	0	0	0	0	0	0	0	0	0	0	0
**Crinalium**	1	21	0	0	0	0	0	0	0	0	0	0	0	0	0	0	0	0	0	0
**Croceibacter**	2	80	0	0	0	0	0	0	0	0	0	0	0	0	0	0	0	0	0	0
**Crocosphaera**	1	6	0	0	0	0	0	0	0	0	1	0	0	0	0	0	0	0	0	0
**Cronobacter**	2	120	0	0	0	0	0	0	0	0	0	0	0	0	0	0	0	0	0	0
**Cryptobacterium**	2	39	0	0	0	0	0	0	0	0	0	0	0	0	0	0	0	0	0	0
**Cucumis**	1	9	0	0	0	0	0	0	0	0	0	0	0	0	0	0	0	0	0	0
**Cupriavidus**	3	317	1	1	0	1	1	0	0	0	1	1	1	1	1	0	1	0	0	1
**Curtobacterium**	1	5	0	0	0	0	0	0	0	0	0	0	0	0	0	0	0	0	0	0
**Curvibacter**	1	27	0	0	0	0	0	0	0	0	0	0	0	0	0	0	0	0	0	0
**Cyanobacterium**	1	9	0	0	0	0	0	0	0	0	0	0	0	0	0	0	0	0	0	0
**Cyanobium**	1	10	0	0	0	0	0	0	0	0	0	0	0	0	0	0	0	0	0	0
**Cyanothece**	1	34	0	0	0	0	0	0	0	0	1	1	0	0	0	0	0	0	0	0
**Cyclobacterium**	1	19	1	0	0	0	0	0	0	0	0	0	0	0	0	0	0	0	0	0
**Cycloclasticus**	1	21	0	0	0	0	0	0	0	0	0	0	0	0	0	0	0	0	0	0
**Cylindrospermopsis**	1	4	0	0	0	0	0	0	0	0	0	0	0	0	0	0	0	0	0	0
**Cylindrospermum**	1	30	0	0	0	0	0	0	0	0	0	0	0	0	0	0	0	0	0	0

Genus	Cluster Number	Protein Counts	K03333	K03379	K03380	K03381	K03382	K03383	K03384	K03391	K03464	K03518	K03862	K04098	K04099	K04100	K04102	K04105	K04107	K04110
Cytophaga	2	69	0	0	0	0	0	0	0	0	0	0	0	0	0	0	0	0	0	0
Dactylococcopsis	1	8	0	0	0	0	1	0	0	0	0	0	0	0	0	0	0	0	0	0
Dechloromonas	1	40	0	0	0	0	0	0	0	0	0	0	0	0	0	1	0	0	0	0
Dechlorosoma	1	19	0	0	0	0	0	0	0	0	0	0	0	0	0	0	0	0	0	0
Deferribacter	2	73	0	0	0	0	0	0	0	0	0	0	0	0	0	0	0	0	0	0
Dehalobacter	1	9	0	0	0	0	0	0	0	0	0	0	0	0	0	0	0	0	0	0
Dehalococcoides	2	41	0	0	0	0	0	0	0	0	0	0	0	0	0	0	0	0	0	0
Dehalogenimonas	1	37	0	0	0	0	0	0	0	0	0	0	0	0	0	0	0	0	0	0
Deinococcus	2	140	0	0	0	0	0	0	0	0	0	1	0	0	0	0	0	0	0	0
Delftia	3	177	0	0	0	1	1	0	0	0	1	1	1	0	0	1	1	0	0	1
Denitrovibrio	2	67	0	0	0	0	0	0	0	0	0	1	0	0	0	0	0	0	0	0
Desulfarculus	2	92	0	0	0	0	0	0	0	0	0	1	0	0	0	0	0	0	0	0
Desulfatibacillum	3	84	1	0	0	0	0	0	0	0	0	0	0	0	0	0	0	0	0	0
Desulfitobacterium	2	102	0	0	0	0	0	0	0	0	0	1	0	0	0	0	0	0	0	0
Desulfobacca	2	65	0	0	0	0	0	0	0	0	0	0	0	0	0	0	0	0	0	0
Desulfobacterium	2	113	0	0	0	0	0	0	0	0	0	1	0	0	0	0	0	0	0	0
Desulfobacula	1	21	0	0	0	0	0	0	0	0	0	1	0	0	0	0	0	0	0	1
Desulfobulbus	2	88	0	0	0	0	0	0	0	0	0	1	0	0	0	0	0	0	0	1
Desulfocapsa	1	9	0	0	0	0	0	0	0	0	0	0	0	0	0	0	0	0	0	0
Desulfococcus	2	80	0	0	0	0	0	0	0	0	0	0	0	0	0	0	0	0	0	0
Desulfohalobium	2	52	0	0	0	0	0	0	0	0	0	0	0	0	0	0	0	0	0	0
Desulfomicrobium	2	65	0	0	0	0	0	0	0	0	0	0	0	0	0	0	0	0	0	0
Desulfomonile	1	25	0	0	0	0	0	0	0	0	0	1	0	0	0	0	0	0	0	1
Desulforhabdus	1	2	0	0	0	0	0	0	0	0	0	0	0	0	0	0	0	0	0	0
Desulfosporosinus	1	30	0	0	0	0	1	0	0	0	1	1	0	0	0	0	0	0	0	0
Desulfotalea	2	66	0	0	0	0	0	0	0	0	0	1	0	0	0	0	0	0	0	0
Desulfotomaculum	2	98	0	0	0	0	0	0	0	0	0	1	0	0	0	0	0	0	0	1
Desulfovibrio	2	144	0	0	0	0	0	0	0	0	0	1	0	0	0	0	0	0	0	1
Desulfurispirillum	2	78	0	0	0	0	0	0	0	0	0	0	0	0	0	0	0	0	0	0
Desulfurivibrio	2	71	0	0	0	0	0	0	0	0	0	0	0	0	0	0	0	0	0	0
Desulfurobacterium	2	53	0	0	0	0	0	0	0	0	0	0	0	0	0	0	0	0	0	0
Desulfurococcus	1	11	0	0	0	0	0	0	0	0	0	1	0	0	0	0	0	0	0	0
Diaphorobacter	1	16	0	0	0	0	0	0	0	0	0	0	0	0	0	0	0	0	0	0
Dichelobacter	2	45	0	0	0	0	0	0	0	0	0	0	0	0	0	0	0	0	0	0
Dickeya	2	155	0	0	0	0	0	0	0	0	0	0	1	0	0	0	0	0	0	0
Dictyoglomus	2	62	0	0	0	0	0	0	0	0	0	0	0	0	0	0	0	0	0	0
Dinoroseobacter	3	148	0	0	0	1	1	0	0	0	0	1	0	0	0	0	1	0	0	0
Dokdonia	1	3	0	0	0	0	0	0	0	0	0	0	0	0	0	0	0	0	0	0
Dorea	1	7	0	0	0	0	0	0	0	0	0	0	0	0	0	0	0	0	0	0
Dyadobacter	2	83	0	0	0	0	0	0	0	0	0	0	0	0	0	0	0	0	0	0
Echinicola	1	20	0	0	0	0	0	0	0	0	0	0	0	0	0	0	0	0	0	0
Edwardsiella	2	126	0	0	0	0	1	0	0	0	0	0	0	0	0	0	0	0	0	0
Eggerthella	1	51	0	0	0	0	0	0	0	0	0	0	0	0	0	0	0	0	0	0
Ehrlichia	2	37	0	0	0	0	0	0	0	0	0	0	0	0	0	0	0	0	0	0
Eikenella	1	4	0	0	0	0	0	0	0	0	0	0	0	0	0	0	0	0	0	0
Elusimicrobium	2	44	0	0	0	0	0	0	0	0	0	0	0	0	0	0	0	0	0	0
Emticicia	1	21	0	0	0	0	0	0	0	0	1	0	0	0	0	0	0	0	0	0
Enhydrobacter	1	1	0	0	0	0	0	0	0	0	0	0	0	0	0	0	0	0	0	0
Ensifer	1	2	0	0	0	0	0	0	0	0	0	0	0	0	0	0	0	0	0	0
Enterobacter	2	202	0	0	0	1	0	0	0	0	1	1	1	0	0	0	0	0	0	0
Enterobacteriaceae	2	211	0	0	0	1	0	0	0	0	1	1	0	0	1	0	0	0	0	0
Enterococcus	1	187	0	0	0	1	1	0	0	0	1	0	1	0	1	0	0	0	0	0

Genus	Cluster Number	Protein Counts	K03333	K03379	K03380	K03381	K03382	K03383	K03384	K03391	K03464	K03518	K03862	K04098	K04099	K04100	K04102	K04105	K04107	K04110
Enterovibrio	1	15	0	0	0	0	0	0	0	0	0	0	0	0	0	0	0	0	0	0
Erwinia	2	151	0	0	0	0	1	0	0	0	0	0	1	0	1	0	1	0	0	0
Erysipelothrix	1	9	0	0	0	0	0	0	0	0	0	0	0	0	0	0	0	0	0	0
Erysipelotrichaceae	1	4	0	0	0	0	0	0	0	0	0	0	0	0	0	0	0	0	0	0
Erythrobacter	2	80	0	0	0	0	0	0	0	0	0	0	0	0	0	0	0	0	0	0
Escherichia	2	242	0	0	0	1	1	0	0	0	1	0	0	0	1	0	0	0	0	0
Ethanoligenens	2	69	0	0	0	0	0	0	0	0	0	0	0	0	0	0	0	0	0	0
Eubacteriaceae	1	2	0	0	0	0	0	0	0	0	0	0	0	0	0	0	0	0	0	0
Eubacterium	2	103	0	0	0	0	0	0	0	0	0	1	0	0	0	0	0	0	0	0
Eudoraea	1	4	0	0	0	0	0	0	0	0	0	0	0	0	0	0	0	0	0	0
Euryarchaeota	1	1	0	0	0	0	0	0	0	0	0	0	0	0	0	0	0	0	0	0
Exiguobacterium	2	93	0	0	0	0	0	0	0	0	0	0	0	0	0	0	0	0	0	0
Faecalibacterium	1	12	0	0	0	0	0	0	0	0	0	1	0	0	0	0	0	0	0	0
Ferrimonas	2	115	0	0	0	0	0	0	0	0	0	0	0	0	0	0	0	0	0	0
Ferroglobus	1	14	0	0	0	0	0	0	0	0	0	0	0	0	0	0	0	0	0	0
Ferroplasma	1	11	0	0	0	0	0	0	0	0	0	1	0	0	0	0	0	0	0	0
Fervidicoccus	1	7	0	0	0	0	0	0	0	0	0	1	0	0	0	0	0	0	0	0
Fervidobacterium	2	54	0	0	0	0	0	0	0	0	0	0	0	0	0	0	0	0	0	0
Fibrella	1	17	0	0	0	0	0	0	0	0	0	1	0	0	0	0	0	0	0	0
Fibrobacter	2	52	0	0	0	0	0	0	0	0	0	0	0	0	0	0	0	0	0	0
Filifactor	1	9	0	0	0	0	0	0	0	0	0	0	0	0	0	0	0	0	0	0
Finegoldia	2	55	0	0	0	0	0	0	0	0	0	1	0	0	0	0	0	0	0	0
Firmicutes	1	15	0	0	0	0	0	0	0	0	0	0	0	0	0	0	0	0	0	0
Fischerella	1	15	0	0	0	0	0	0	0	0	0	0	0	0	0	0	0	0	0	0
Flavobacteria	1	7	0	0	0	0	0	0	0	0	0	0	0	0	0	0	0	0	0	0
Flavobacteriaceae	2	69	0	0	0	0	0	0	0	0	0	0	0	0	0	0	0	0	0	0
Flavobacteriales	1	3	0	0	0	0	0	0	0	0	0	0	0	0	0	0	0	0	0	0
Flavobacterium	2	103	0	0	0	0	0	0	0	0	0	0	0	0	0	0	0	0	0	0
Flexibacter	1	17	1	0	0	0	0	0	0	0	0	0	0	0	0	0	0	0	0	0
Flexistipes	1	11	0	0	0	0	0	0	0	0	0	0	0	0	0	0	0	0	0	0
Fluoribacter	1	6	0	0	0	0	0	0	0	0	0	0	0	0	0	0	0	0	0	0
Fluviicola	2	69	0	0	0	0	0	0	0	0	0	0	0	0	0	0	0	0	0	0
Formosa	1	2	0	0	0	0	0	0	0	0	0	0	0	0	0	0	0	0	0	0
Francisella	2	88	0	0	0	0	0	0	0	0	0	0	0	0	0	0	0	0	0	0
Frankia	3	202	1	1	0	0	1	1	0	1	0	1	1	0	0	0	0	0	0	0
Frateuria	1	25	0	0	0	1	0	0	0	0	1	0	1	0	0	0	0	0	0	0
Fructobacillus	1	2	0	0	0	0	0	0	0	0	0	0	0	0	0	0	0	0	0	0
Fulvimarina	1	2	0	0	0	0	0	0	0	0	0	0	0	0	0	0	0	0	0	0
Fusobacterium	1	48	0	0	0	0	0	0	0	0	0	0	0	0	0	0	0	0	0	0
Gallibacterium	2	87	0	0	0	0	0	0	0	0	0	0	0	0	0	0	0	0	0	0
Gallionella	2	67	0	0	0	0	0	0	0	0	0	0	0	0	0	0	0	0	0	0
Gammaproteobacteria	1	9	0	0	0	0	0	0	0	0	0	0	0	0	0	0	0	0	0	0
Gardnerella	1	31	0	0	0	0	0	0	0	0	0	0	0	0	0	0	0	0	0	0
Geitlerinema	1	9	0	0	0	0	0	0	0	0	0	0	0	0	0	0	0	0	0	0
Gemmatimonas	2	93	0	0	0	0	0	0	0	0	0	0	0	0	0	0	0	0	0	0
Geobacillus	2	168	0	0	0	0	1	0	0	0	0	1	0	0	0	0	0	0	0	1
Geobacter	2	150	0	0	0	0	0	0	0	0	0	1	0	0	0	0	0	0	0	1
Geodermatophilus	2	136	0	0	0	1	1	1	0	0	1	1	1	0	0	0	0	0	0	0
Geopsychrobacter	1	2	0	0	0	0	0	0	0	0	0	0	0	0	0	0	0	0	0	0
Gillisia	1	11	0	0	0	0	0	0	0	0	0	0	0	0	0	0	0	0	0	0
Gilvimarinus	1	4	0	0	0	0	0	0	0	0	0	0	0	0	0	0	0	0	0	0
Glaciecola	2	117	0	0	0	1	1	0	0	0	0	0	0	0	0	0	0	0	0	0

Genus	Cluster Number	Protein Counts	K03333	K03379	K03380	K03381	K03382	K03383	K03384	K03391	K03464	K03518	K03862	K04098	K04099	K04100	K04102	K04105	K04107	K04110
Gloeobacter	2	67	0	0	0	0	0	0	0	0	0	0	0	0	0	0	0	0	0	0
Gloeocapsa	1	20	0	0	0	0	1	1	0	0	0	0	0	0	0	0	0	0	0	1
Gloeothece	1	1	0	0	0	0	0	0	0	0	0	0	0	0	0	0	0	0	0	0
Gluconacetobacter	2	96	0	0	0	0	1	0	0	0	0	0	1	0	0	0	0	0	0	0
Gluconobacter	2	76	0	0	0	0	1	0	0	0	0	0	1	0	0	0	0	0	0	0
Gordonia	1	158	1	0	1	1	1	1	0	0	1	1	1	0	0	0	0	0	0	0
Gordonibacter	1	32	0	0	0	0	0	0	0	0	0	0	0	0	0	0	0	0	0	0
Gramella	2	104	0	0	0	0	0	0	0	0	0	0	0	0	0	0	0	0	0	0
Granulibacter	2	90	0	0	0	0	0	0	0	0	0	0	1	0	0	0	0	0	0	0
Granulicella	2	98	0	0	0	0	0	0	0	0	0	0	1	0	0	0	1	0	0	0
Grimontia	1	9	0	0	0	0	0	0	0	0	0	0	0	0	0	0	0	0	0	0
Haemophilus	2	117	0	0	0	0	0	0	0	0	0	0	0	0	0	0	0	0	0	0
Hafnia	1	29	0	0	0	0	0	0	0	0	0	0	0	0	0	0	0	0	0	0
Hahella	3	130	1	0	0	0	1	0	0	0	0	0	0	0	0	0	0	0	0	0
Halalkalicoccus	1	18	0	0	0	0	0	0	0	0	0	1	0	0	0	0	0	0	0	0
Halanaerobium	2	89	0	0	0	0	1	0	0	0	0	1	0	0	0	0	0	0	0	0
Haliangium	2	108	1	0	0	0	0	0	0	0	0	1	0	0	0	0	0	0	0	0
Haliscomenobacter	2	107	0	0	0	0	0	0	0	0	0	1	0	0	0	0	0	0	0	0
Haloarcula	1	22	0	0	0	0	0	0	0	0	0	1	0	0	0	0	0	0	0	0
Halobacillus	1	19	0	0	0	0	0	0	0	0	0	0	0	0	0	0	0	0	0	0
Halobacteriaceae	1	1	0	0	0	0	0	0	0	0	0	0	0	0	0	0	0	0	0	0
Halobacterium	1	10	0	0	0	0	0	0	0	0	0	0	0	0	0	0	0	0	0	0
Halobacteroides	1	14	0	0	0	0	1	0	0	0	0	1	0	0	0	0	0	0	0	0
Haloferax	1	20	0	0	0	0	0	0	0	0	0	1	0	0	0	0	0	0	0	0
Halogeometricum	1	12	0	0	0	0	0	0	0	0	0	0	0	0	0	0	0	0	0	0
Halomicrobium	1	10	0	0	0	0	0	0	0	0	0	0	0	0	0	0	0	0	0	0
Halomonas	1	149	0	0	0	0	1	0	0	0	0	0	1	0	0	0	0	0	0	0
Halopiger	1	16	0	0	0	0	0	0	0	0	0	0	0	0	0	0	0	0	0	0
Haloquadratum	1	19	0	0	0	0	0	0	0	0	0	1	0	0	0	0	0	0	0	0
Halorhabdus	1	10	0	0	0	0	0	0	0	0	0	0	0	0	0	0	1	0	0	0
Halorhodospira	2	71	0	0	0	0	0	0	0	0	0	0	0	0	0	0	0	0	0	0
Halorubrum	1	16	0	0	0	0	0	0	0	0	0	0	0	0	0	0	0	0	0	0
Haloterrigena	1	19	0	0	0	0	0	0	0	0	0	1	0	0	0	0	0	0	0	0
Halothece	1	9	0	0	0	0	0	0	0	0	0	0	0	0	0	0	0	0	0	0
Halothermothrix	2	50	0	0	0	0	0	0	0	0	0	0	0	0	0	0	0	0	0	0
Halothiobacillus	2	65	0	0	0	0	1	0	0	0	0	0	0	0	0	0	0	0	0	0
Halovivax	1	12	0	0	0	0	0	0	0	0	0	0	0	0	0	0	0	0	0	0
Helicobacter	2	79	0	0	0	0	0	0	0	0	0	0	0	0	0	0	0	0	0	0
Heliobacillus	1	4	0	0	0	0	0	0	0	0	0	0	0	0	0	0	0	0	0	0
Heliobacterium	2	51	0	0	0	0	0	0	0	0	0	0	0	0	0	0	0	0	0	0
Herbaspirillum	2	172	0	0	0	1	1	0	0	0	1	0	1	1	0	0	0	0	0	0
Herminiimonas	3	89	0	0	0	0	0	0	0	0	0	0	0	0	0	0	1	0	0	0
Herpetosiphon	3	90	0	0	0	0	0	0	0	0	0	0	0	0	0	0	0	0	0	0
Hippea	2	44	0	0	0	0	0	0	0	0	0	0	0	0	0	0	0	0	0	0
Hirschia	2	102	0	0	0	0	0	0	0	0	0	0	0	0	0	1	1	0	0	0
Histophilus	2	72	0	0	0	0	0	0	0	0	0	0	0	0	0	0	0	0	0	0
Hoeflea	1	16	0	0	0	0	0	0	0	0	0	0	0	0	0	0	0	0	0	0
Hydrogenivirga	1	2	0	0	0	0	0	0	0	0	0	0	0	0	0	0	0	0	0	0
Hydrogenobacter	2	55	0	0	0	0	0	0	0	0	0	1	0	0	0	0	0	0	0	0
Hydrogenobaculum	2	44	0	0	0	0	0	0	0	0	0	0	0	0	0	0	0	0	0	0
Hydrogenophaga	1	9	0	0	0	0	0	0	0	0	0	0	0	0	0	0	0	0	0	0
Hylemonella	1	4	0	0	0	0	0	0	0	0	0	0	0	0	0	0	0	0	0	0

Genus	Cluster Number	Protein Counts	K03333	K03379	K03380	K03381	K03382	K03383	K03384	K03391	K03464	K03518	K03862	K04098	K04099	K04100	K04102	K04105	K04107	K04110
Hyperthermus	1	6	0	0	0	0	0	0	0	0	0	0	0	0	0	0	0	0	0	0
Hyphomicrobium	2	93	0	0	0	0	0	0	0	0	0	1	0	0	0	0	0	0	0	0
Hyphomonas	3	105	0	0	0	0	0	0	0	0	0	0	0	0	0	0	0	0	0	0
Ideonella	1	18	0	0	0	0	0	0	0	0	0	0	0	0	0	0	0	0	0	0
Idiomarina	2	86	0	0	0	0	0	0	0	0	0	0	0	0	0	0	0	0	0	0
Ignavibacterium	1	14	0	0	0	0	0	0	0	0	0	0	0	0	0	0	0	0	0	0
Ignicoccus	1	5	0	0	0	0	0	0	0	0	0	0	0	0	0	0	0	0	0	0
Ignisphaera	1	8	0	0	0	0	0	0	0	0	0	0	0	0	0	0	0	0	0	0
Ilyobacter	2	85	0	0	0	0	0	0	0	0	0	1	0	0	0	0	0	0	0	0
Intrasporangium	2	114	1	0	0	0	0	1	0	0	0	1	0	0	0	0	0	0	0	0
Isoptericola	1	18	0	0	1	0	0	0	0	0	0	0	0	0	0	0	0	0	0	0
Isosphaera	2	75	0	0	0	0	0	0	0	0	0	0	0	0	0	0	0	0	0	0
Jannaschia	3	151	0	0	0	0	1	0	0	0	0	1	1	0	0	0	0	1	0	0
Janthinobacterium	2	99	0	0	0	0	1	0	0	0	0	1	1	0	0	0	0	0	0	0
Jonesia	2	56	0	0	0	0	0	0	0	0	0	0	0	0	0	0	0	0	0	0
Joostella	1	3	0	0	0	0	0	0	0	0	0	0	0	0	0	0	0	0	0	0
Kaistia	1	2	0	0	0	0	0	0	0	0	0	0	0	0	0	0	0	0	0	0
Kangiella	2	84	0	0	0	0	0	0	0	0	0	0	0	0	0	0	0	0	0	0
Ketogulonicigenium	2	102	0	0	0	0	0	0	0	0	0	0	0	0	0	0	0	0	0	0
Kineococcus	1	24	0	0	0	0	1	0	0	0	0	0	0	0	0	0	0	0	0	0
Kinetoplastibacterium	1	2	0	0	0	0	0	0	0	0	0	0	0	0	0	0	0	0	0	0
Kingella	1	4	0	0	0	0	0	0	0	0	0	0	0	0	0	0	0	0	0	0
Kitasatospora	1	36	1	0	0	0	1	0	0	0	0	1	1	0	1	0	0	0	0	0
Klebsiella	3	222	0	0	0	1	1	0	0	0	1	1	1	0	1	0	0	0	0	0
Kluyvera	1	1	0	0	0	0	0	0	0	0	0	0	0	0	0	0	0	0	0	0
Kocuria	2	87	0	0	1	0	0	0	0	0	0	0	0	0	0	0	0	0	0	0
Kordia	1	5	0	0	0	0	0	0	0	0	0	0	0	0	0	0	0	0	0	0
Kosmotoga	2	61	0	0	0	0	1	0	0	0	0	1	0	0	0	0	0	0	0	0
Kribbella	2	99	1	0	0	0	0	0	0	0	0	0	0	0	0	0	0	0	0	0
Krokinobacter	2	90	1	0	0	0	0	0	0	0	0	0	0	0	0	0	0	0	0	0
Kyrpidia	2	113	0	0	0	0	1	0	0	0	0	1	0	0	0	0	0	0	0	0
Kytococcus	2	70	0	0	0	0	0	0	0	0	0	0	0	0	0	0	0	0	0	0
Labrenzia	1	4	0	0	0	0	0	0	0	0	0	0	0	0	0	0	0	0	0	0
Lachnoanaerobaculum	1	1	0	0	0	0	0	0	0	0	0	0	0	0	0	0	0	0	0	0
Lachnospiraceae	1	51	0	0	0	0	0	0	0	0	0	1	0	0	0	0	0	0	0	0
Lacinutrix	1	20	0	0	0	0	0	0	0	0	0	0	0	0	0	0	0	0	0	0
Lactobacillus	2	119	0	0	0	0	0	0	0	0	0	0	0	0	0	0	0	0	0	0
Lactococcus	2	82	0	0	0	0	0	0	0	0	0	0	0	0	0	0	0	0	0	0
Lamprocystis	1	2	0	0	0	0	0	0	0	0	0	0	0	0	0	0	0	0	0	0
Laribacter	2	80	0	0	0	0	0	0	0	0	0	0	0	0	0	0	0	0	0	0
Lawsonia	2	33	0	0	0	0	0	0	0	0	0	0	0	0	0	0	0	0	0	0
Leadbetterella	2	79	0	0	0	0	0	0	0	0	0	0	0	0	0	0	0	0	0	0
Leeuwenhoekiella	1	2	0	0	0	0	0	0	0	0	0	0	0	0	0	0	0	0	0	0
Legionella	2	117	0	0	0	0	0	0	0	0	0	0	0	0	0	0	0	0	0	0
Leifsonia	2	48	0	0	0	0	0	0	0	0	0	0	0	0	0	0	0	0	0	0
Leptolyngbya	1	10	0	0	0	0	0	0	0	0	0	0	0	0	0	0	0	0	0	0
Leptospira	2	76	1	0	0	0	0	0	0	0	0	0	0	0	0	0	0	0	0	0
Leptospirillum	1	10	0	0	0	0	0	0	0	0	0	0	0	0	0	0	0	0	0	0
Leptothrix	3	186	1	0	0	0	1	0	0	0	0	0	1	1	0	1	0	0	0	1
Leptotrichia	2	43	0	0	0	0	0	0	0	0	0	0	0	0	0	0	0	0	0	0
Leucobacter	1	2	0	0	0	0	0	0	0	0	0	0	0	0	1	0	0	0	0	0
Leuconostoc	2	64	0	0	0	0	0	0	0	0	0	0	0	0	0	0	0	0	0	0

Genus	Cluster Number	Protein Counts	K03333	K03379	K03380	K03381	K03382	K03383	K03384	K03391	K03464	K03518	K03862	K04098	K04099	K04100	K04102	K04105	K04107	K04110
Liberibacter	1	4	0	0	0	0	0	0	0	0	0	0	0	0	0	0	0	0	0	0
Limnohabitans	1	11	0	0	0	0	0	0	0	0	0	0	0	0	0	1	0	0	0	0
Listeria	2	77	0	0	0	0	0	0	0	0	0	0	0	0	0	0	0	0	0	0
Listeriaceae	1	6	0	0	0	0	0	0	0	0	0	0	0	0	0	0	0	0	0	0
Listonella	1	38	0	0	0	0	0	0	0	0	0	0	0	0	0	0	0	0	0	0
Loktanella	1	3	0	0	0	0	0	0	0	0	0	0	0	0	0	0	0	0	0	0
Lysinibacillus	2	97	0	0	0	0	0	0	0	0	0	0	0	0	0	0	0	0	0	0
Macrococcus	2	60	0	0	0	0	0	0	0	0	0	0	0	0	0	0	0	0	0	0
Magnetococcus	2	64	0	0	0	0	0	0	0	0	0	0	0	0	0	0	0	0	0	0
Magnetospirillum	2	113	0	0	0	0	0	0	0	0	0	1	0	0	0	1	0	1	1	0
Mahella	2	58	0	0	0	0	0	0	0	0	0	0	0	0	0	0	0	0	0	0
Mannheimia	2	85	0	0	0	0	0	0	0	0	0	0	0	0	0	0	0	0	0	0
Maribacter	3	87	0	0	0	0	0	0	0	0	0	0	0	0	0	0	0	0	0	0
Maricaulis	3	87	0	0	0	0	0	0	0	0	0	0	0	0	0	0	0	0	0	0
Marichromatium	1	2	0	0	0	0	0	0	0	0	0	0	0	0	0	0	0	0	0	0
Marinithermus	2	77	0	0	0	0	0	0	0	0	0	0	0	0	0	0	0	0	0	0
Marinitoga	1	15	0	0	0	0	1	0	0	0	0	1	0	0	0	0	0	0	0	0
Marinobacter	2	163	0	1	0	1	0	0	0	0	1	0	0	0	0	0	1	0	0	0
Marinomonas	3	192	0	0	1	1	1	0	0	0	1	0	1	0	0	1	0	0	0	0
Maritimibacter	1	7	0	0	0	0	0	0	0	0	0	0	0	0	0	0	0	0	0	0
Marivirga	2	96	1	0	0	0	0	0	0	0	0	0	0	0	0	0	0	0	0	0
Martelella	1	3	0	0	0	0	0	0	0	0	0	0	0	0	0	0	0	0	0	0
Marvinbryantia	1	1	0	0	0	0	0	0	0	0	0	0	0	0	0	0	0	0	0	0
Massilia	1	4	0	0	0	0	0	0	0	0	0	0	0	0	0	0	0	0	0	0
Mastigocladopsis	1	16	0	0	0	0	0	0	0	0	0	0	0	0	0	0	0	0	0	0
Megamonas	1	47	0	0	0	0	0	0	0	0	0	1	0	0	0	0	0	0	0	0
Megasphaera	1	14	0	0	0	0	0	0	0	0	0	1	0	0	0	0	0	0	0	0
Meiothermus	2	110	0	0	0	0	0	0	0	0	0	1	0	0	0	0	0	0	0	0
Melissococcus	2	46	0	0	0	0	0	0	0	0	0	0	0	0	0	0	0	0	0	0
Mesoflavibacter	1	6	0	0	0	0	0	0	0	0	0	0	0	0	0	0	0	0	0	0
Mesoplasma	1	23	0	0	0	0	0	0	0	0	0	0	0	0	0	0	0	0	0	0
Mesorhizobium	2	201	0	0	0	1	0	0	0	0	0	1	1	0	0	0	0	0	0	0
Mesotoga	1	14	0	0	0	0	1	0	0	0	0	1	0	0	0	0	0	0	0	0
Metallosphaera	1	18	0	0	0	0	0	0	0	0	0	1	0	0	0	0	0	0	0	0
Methanobacterium	1	7	0	0	0	0	0	0	0	0	0	0	0	0	0	0	0	0	0	0
Methanobrevibacter	1	9	0	0	0	0	0	0	0	0	0	0	0	0	0	0	0	0	0	0
Methanocaldococcus	1	6	0	0	0	0	0	0	0	0	0	0	0	0	0	0	0	0	0	0
Methanocella	1	15	0	0	0	0	0	0	0	0	0	0	0	0	0	0	0	0	0	0
Methanococcaceae	1	1	0	0	0	0	0	0	0	0	0	0	0	0	0	0	0	0	0	0
Methanococcoides	1	5	0	0	0	0	0	0	0	0	0	0	0	0	0	0	0	0	0	0
Methanococcus	1	9	0	0	0	0	0	0	0	0	0	0	0	0	0	0	0	0	0	0
Methanocorpusculum	1	7	0	0	0	0	0	0	0	0	0	0	0	0	0	0	0	0	0	0
Methanoculleus	1	8	0	0	0	0	0	0	0	0	0	0	0	0	0	0	0	0	0	0
Methanohalobium	1	5	0	0	0	0	0	0	0	0	0	0	0	0	0	0	0	0	0	0
Methanohalophilus	1	5	0	0	0	0	0	0	0	0	0	0	0	0	0	0	0	0	0	0
Methanolobus	1	5	0	0	0	0	0	0	0	0	0	0	0	0	0	0	0	0	0	0
Methanomethylovorans	1	5	0	0	0	0	0	0	0	0	0	0	0	0	0	0	0	0	0	0
Methanoplanus	1	7	0	0	0	0	0	0	0	0	0	0	0	0	0	0	0	0	0	0
Methanopyrus	1	4	0	0	0	0	0	0	0	0	0	0	0	0	0	0	0	0	0	0
Methanoregula	1	8	0	0	0	0	0	0	0	0	0	0	0	0	0	0	0	0	0	0
Methanosaeta	1	9	0	0	0	0	0	0	0	0	0	0	0	0	0	0	0	0	0	0
Methanosalsum	1	7	0	0	0	0	0	0	0	0	0	0	0	0	0	0	0	0	0	0

Genus	Cluster Number	Protein Counts	K03333	K03379	K03380	K03381	K03382	K03383	K03384	K03391	K03464	K03518	K03862	K04098	K04099	K04100	K04102	K04105	K04107	K04110
Methanosarcina	1	18	0	0	0	0	0	0	0	0	0	0	0	0	0	0	0	0	0	0
Methanosphaera	1	7	0	0	0	0	0	0	0	0	0	0	0	0	0	0	0	0	0	0
Methanosphaerula	1	8	0	0	0	0	0	0	0	0	0	0	0	0	0	0	0	0	0	0
Methanospirillum	1	6	0	0	0	0	0	0	0	0	0	0	0	0	0	0	0	0	0	0
Methanothermobacter	1	6	0	0	0	0	0	0	0	0	0	0	0	0	0	0	0	0	0	0
Methanothermococcus	1	5	0	0	0	0	0	0	0	0	0	0	0	0	0	0	0	0	0	0
Methanothermus	1	5	0	0	0	0	0	0	0	0	0	0	0	0	0	0	0	0	0	0
Methanotorris	1	5	0	0	0	0	0	0	0	0	0	0	0	0	0	0	0	0	0	0
Methylacidiphilum	2	48	0	0	0	0	0	0	0	0	0	0	1	0	0	0	0	0	0	0
Methylarcula	1	2	0	0	0	0	0	0	0	0	0	0	0	0	0	0	0	0	0	0
Methylibium	1	38	0	1	0	0	1	0	0	0	0	0	1	0	0	0	0	0	0	0
Methylobacillus	2	61	0	0	0	0	0	0	0	0	0	0	0	0	0	0	0	0	0	0
Methylobacterium	3	236	0	0	1	1	1	1	0	0	1	1	1	1	0	0	0	0	0	0
Methylocella	3	117	0	0	0	0	0	0	0	0	0	0	0	0	0	0	0	0	0	0
Methylococcus	2	80	0	0	0	0	0	0	0	0	0	0	0	0	0	0	0	0	0	0
Methylocystis	1	21	0	0	0	0	0	0	0	0	0	0	0	0	0	0	0	0	0	0
Methyloferula	1	2	0	0	0	0	0	0	0	0	0	0	0	0	0	0	0	0	0	0
Methylomicrobium	1	12	0	0	0	0	0	0	0	0	0	0	0	0	0	0	0	0	0	0
Methylomonas	1	15	0	0	0	0	0	0	0	0	0	0	0	0	0	0	0	0	0	0
Methylophaga	1	7	0	0	0	0	0	0	0	0	0	0	0	0	0	0	0	0	0	0
Methylophilus	1	2	0	0	0	0	0	0	0	0	0	0	0	0	0	0	0	0	0	0
Methylosinus	1	2	0	0	0	0	0	0	0	0	0	0	0	0	0	0	0	0	0	0
Methylotenera	2	72	0	0	0	0	0	0	0	0	0	0	0	0	0	0	0	0	0	0
Methyloversatilis	1	5	0	0	0	0	0	0	0	0	0	0	0	0	0	0	0	0	0	0
Methylovorus	2	66	0	0	0	0	0	0	0	0	0	0	0	0	0	0	0	0	0	0
Micavibrio	1	9	0	0	0	0	0	0	0	0	0	0	0	0	0	0	0	0	0	0
Microbacterium	2	115	0	0	1	0	1	0	0	0	0	0	1	0	1	0	0	0	0	0
Microchaete	1	23	0	0	0	0	0	0	0	0	0	0	0	0	0	0	0	0	0	0
Micrococcus	2	72	0	0	1	0	0	0	0	0	0	0	0	0	0	0	0	0	0	0
Microcoleus	1	17	0	0	0	0	0	0	0	0	0	0	0	0	0	0	0	0	0	1
Microcystis	2	67	0	0	0	0	0	0	0	0	0	0	0	0	0	0	0	0	0	0
Microlunatus	1	25	0	0	0	0	0	0	0	0	0	0	0	0	0	0	0	0	0	0
Micromonospora	2	115	1	0	0	0	0	0	0	0	0	1	0	0	0	0	0	0	0	0
Microvirga	1	6	0	0	0	0	0	0	0	0	0	0	0	0	0	0	0	0	0	0
Mobiluncus	1	41	0	0	0	0	0	0	0	0	0	0	0	0	0	0	0	0	0	0
Modestobacter	1	43	0	0	1	0	1	1	0	0	1	1	0	0	0	1	0	0	0	0
Moorea	1	2	0	0	0	0	0	0	0	0	0	0	0	0	0	0	0	0	0	0
Moorella	2	69	0	0	0	0	0	1	0	0	0	1	0	0	0	0	0	0	0	0
Moraxella	2	66	0	0	0	0	0	0	0	0	0	0	0	0	0	0	0	0	0	0
Morganella	1	29	0	0	0	0	0	0	0	0	0	0	0	0	0	0	0	0	0	0
Moritella	1	2	0	0	0	0	0	0	0	0	0	0	0	0	0	0	0	0	0	0
Muricauda	1	23	0	0	0	0	0	0	0	0	0	0	0	0	0	0	0	0	0	0
Mycobacterium	3	306	1	1	0	1	1	0	0	1	1	1	1	1	0	0	1	0	0	0
Mycoplasma	1	53	0	0	0	0	0	0	0	0	0	0	0	0	0	0	0	0	0	0
Myxococcus	2	110	1	0	0	0	0	0	0	0	0	0	1	0	0	0	0	0	0	0
Nakamurella	2	115	1	0	0	0	1	1	0	0	0	1	0	0	0	0	0	0	0	0
napthalene	1	2	0	0	0	0	0	0	0	0	0	0	0	0	0	0	0	0	0	0
Natranaerobius	2	55	0	0	0	0	0	0	0	0	0	1	0	0	0	0	0	0	0	0
Natrialba	1	16	0	0	0	0	0	0	0	0	0	0	0	0	0	0	0	0	0	0
Natrinema	1	16	0	0	0	0	0	0	0	0	0	0	0	0	0	0	0	0	0	0
Natronobacterium	1	11	0	0	0	0	0	0	0	0	0	0	0	0	0	0	0	0	0	0
Natronococcus	1	21	0	0	0	0	0	0	0	0	0	1	0	0	0	0	0	0	0	0

Genus	Cluster Number	Protein Counts	K03333	K03379	K03380	K03381	K03382	K03383	K03384	K03391	K03464	K03518	K03862	K04098	K04099	K04100	K04102	K04105	K04107	K04110
**Natronomonas**	1	20	0	0	0	0	0	0	0	0	0	1	0	0	0	0	0	0	0	0
**Nautilia**	2	48	0	0	0	0	0	0	0	0	0	0	0	0	0	0	0	0	0	0
**Neisseria**	2	67	0	0	0	0	0	0	0	0	0	0	0	0	0	0	0	0	0	0
**Neisseriaceae**	1	1	0	0	0	0	0	0	0	0	0	0	0	0	0	0	0	0	0	0
**Neorickettsia**	1	31	0	0	0	0	0	0	0	0	0	0	0	0	0	0	0	0	0	0
**Neptuniibacter**	1	2	0	0	0	0	0	0	0	0	0	0	0	0	0	0	0	0	0	0
**Niastella**	1	21	0	0	0	0	0	0	0	0	0	1	0	0	0	0	0	0	0	0
**Nitratifractor**	2	54	0	0	0	0	0	0	0	0	0	0	0	0	0	0	0	0	0	0
**Nitratireductor**	1	19	0	0	0	0	0	0	0	0	0	0	0	0	0	0	0	0	0	0
**Nitratiruptor**	2	40	0	0	0	0	0	0	0	0	0	0	0	0	0	0	0	0	0	0
**Nitrobacter**	2	99	0	0	0	0	0	0	0	0	0	1	0	0	0	0	0	0	0	0
**Nitrococcus**	1	2	0	0	0	0	0	0	0	0	0	0	0	0	0	0	0	0	0	0
**Nitrosococcus**	2	94	0	0	0	0	0	0	0	0	0	0	0	0	0	0	0	0	0	0
**Nitrosomonas**	2	72	0	0	0	0	0	0	0	0	0	0	0	0	0	0	0	0	0	0
**Nitrosopumilaceae**	1	7	0	0	0	0	0	0	0	0	0	0	0	0	0	0	0	0	0	0
**Nitrosopumilus**	1	11	0	0	0	0	0	0	0	0	0	0	0	0	0	0	0	0	0	0
**Nitrosospira**	2	74	0	0	0	0	0	0	0	0	0	0	0	0	0	0	0	0	0	0
**Nocardia**	2	185	1	0	1	1	1	0	0	0	1	1	0	0	0	0	0	0	0	0
**Nocardioidaceae**	1	2	0	0	0	0	0	0	0	0	0	0	0	0	0	0	0	0	0	0
**Nocardioides**	2	157	1	0	0	1	1	1	0	0	1	1	0	0	0	0	0	0	0	0
**Nocardiopsis**	2	102	1	0	0	0	1	0	0	0	0	1	0	0	0	0	0	0	0	0
**Nodularia**	1	22	0	0	0	0	0	0	0	0	0	0	0	0	0	0	0	0	0	0
**Nonlabens**	1	19	0	0	0	0	0	0	0	0	0	0	0	0	0	0	0	0	0	0
**Nostoc**	2	111	1	0	0	0	1	0	0	0	1	0	0	0	0	0	0	0	0	0
**Nostocaceae**	1	2	0	0	0	0	0	0	0	0	0	0	0	0	0	0	0	0	0	0
**Novosphingobium**	2	169	0	1	0	1	0	0	0	0	1	1	1	0	0	1	0	0	0	0
**Oceanibaculum**	1	3	0	0	0	0	0	0	0	0	0	0	0	0	0	0	0	0	0	0
**Oceanibulbus**	1	12	0	0	0	0	0	0	0	0	0	0	1	0	0	0	0	0	0	0
**Oceanicola**	1	9	0	0	0	0	0	0	0	0	0	0	0	0	0	0	0	0	0	0
**Oceanimonas**	1	33	0	0	0	1	1	0	0	0	1	0	0	0	0	0	0	0	0	0
**Oceaniovalibus**	1	2	0	0	0	0	0	0	0	0	0	0	0	0	0	0	0	0	0	0
**Oceanithermus**	2	72	0	0	0	0	0	0	0	0	0	0	0	0	0	0	0	0	0	0
**Oceanobacillus**	2	91	0	0	0	0	0	0	0	0	0	0	0	0	0	0	0	0	0	0
**Oceanospirillum**	1	2	0	0	0	0	0	0	0	0	0	0	0	0	0	0	0	0	0	0
**Ochrobactrum**	2	133	0	0	0	0	0	0	0	0	0	0	0	0	0	0	0	0	0	0
**Octadecabacter**	1	29	0	0	0	1	1	0	0	0	0	1	1	0	0	0	0	0	0	0
**Oenococcus**	2	41	0	0	0	0	0	0	0	0	0	0	0	0	0	0	0	0	0	0
**Oleomonas**	1	2	0	0	0	0	0	0	0	0	0	0	0	0	0	0	0	0	0	0
**Oligotropha**	3	87	0	0	0	0	0	0	0	0	0	1	0	0	0	0	0	0	0	0
**Olsenella**	2	40	0	0	0	0	0	0	0	0	0	0	0	0	0	0	0	0	0	0
**Opitutus**	2	67	0	0	0	0	0	0	0	0	0	0	0	0	0	0	0	0	0	0
**Orientia**	1	21	0	0	0	0	0	0	0	0	0	0	0	0	0	0	0	0	0	0
**Ornithobacterium**	1	11	1	0	0	0	0	0	0	0	0	0	0	0	0	0	0	0	0	0
**Oscillatoria**	1	16	0	0	0	0	0	0	0	0	0	0	0	0	0	0	0	0	0	0
**Oscillatoriales**	1	2	0	0	0	0	0	0	0	0	0	0	0	0	0	0	0	0	0	0
**Oscillibacter**	1	20	0	0	0	0	1	0	0	0	0	1	0	0	0	0	0	0	0	0
**Owenweeksia**	1	15	1	0	0	0	0	0	0	0	0	0	0	0	0	0	0	0	0	0
**Oxalobacteraceae**	1	4	0	0	0	0	0	0	0	0	0	0	0	0	0	0	0	0	0	0
**Paenibacillus**	2	152	0	0	0	0	0	1	0	0	0	1	1	0	0	0	0	0	0	0
**Paludibacter**	2	67	0	0	0	0	0	0	0	0	0	0	0	0	0	0	0	0	0	0
**Pandoraea**	1	18	0	0	0	0	0	0	0	0	0	0	0	0	0	0	1	0	0	0
**Pannonibacter**	1	3	0	0	0	0	0	0	0	0	0	0	0	0	0	0	0	0	0	0

Genus	Cluster Number	Protein Counts	K03333	K03379	K03380	K03381	K03382	K03383	K03384	K03391	K03464	K03518	K03862	K04098	K04099	K04100	K04102	K04105	K04107	K04110
**Pantholops**	1	23	0	0	0	0	0	0	0	0	0	0	0	0	0	0	0	0	0	0
**Pantoea**	2	181	0	0	0	0	1	0	0	0	0	0	1	0	0	0	1	0	0	0
**Parabacteroides**	2	64	0	0	0	0	0	0	0	0	0	0	0	0	0	0	0	0	0	0
**Parachlamydia**	1	11	0	0	0	0	0	0	0	0	0	0	0	0	0	0	0	0	0	0
**Paracoccus**	2	169	0	0	1	1	1	0	0	0	1	0	0	0	0	0	0	0	0	0
**Parvibaculum**	3	117	0	1	0	0	0	0	0	0	0	1	0	0	0	0	0	0	0	0
**Parvularcula**	2	72	1	0	0	0	1	0	0	0	0	0	0	0	0	0	0	0	0	0
**Pasteurella**	2	85	0	0	0	0	0	0	0	0	0	0	0	0	0	0	0	0	0	0
**Pasteurellaceae**	1	2	0	0	0	0	0	0	0	0	0	0	0	0	0	0	0	0	0	0
**Pectobacterium**	1	44	0	0	0	0	0	0	0	0	0	0	0	0	0	0	0	0	0	0
**Pediococcus**	1	43	0	0	0	0	0	0	0	0	0	0	0	0	0	0	0	0	0	0
**Pedobacter**	2	88	0	0	0	0	0	0	0	0	0	0	0	0	0	0	0	0	0	0
**Pelagibaca**	1	10	0	0	0	0	0	0	0	0	0	0	0	0	0	0	0	0	0	0
**Pelagibacterium**	1	39	0	0	1	1	0	0	0	0	0	0	0	1	0	0	1	0	0	0
**Pelobacter**	2	85	0	0	0	0	0	0	0	0	0	1	0	0	0	0	0	0	0	0
**Pelodictyon**	2	62	0	0	0	0	0	0	0	0	0	0	0	0	0	0	0	0	0	0
**Pelosinus**	1	1	0	0	0	0	0	0	0	0	0	0	0	0	0	0	0	0	0	0
**Pelotomaculum**	2	60	0	0	0	0	0	0	0	0	0	1	0	0	0	0	0	0	0	0
**Peptostreptococcus**	1	1	0	0	0	0	0	0	0	0	0	0	0	0	0	0	0	0	0	0
**Persephonella**	2	55	0	0	0	0	0	0	0	0	0	0	0	0	0	0	0	0	0	0
**Petrotoga**	2	72	0	0	0	0	1	0	0	0	0	1	0	0	0	0	0	0	0	0
**Phaeobacter**	2	55	0	0	0	0	1	0	0	0	0	1	0	0	0	0	0	0	0	0
**Phenylobacterium**	3	103	0	0	0	0	0	0	0	0	0	0	0	0	0	0	0	0	0	0
**Photobacterium**	2	119	0	0	0	0	1	0	0	0	0	1	0	0	0	0	0	0	0	0
**Photorhabdus**	2	149	0	0	0	0	0	0	0	0	0	0	0	0	0	0	0	0	0	0
**Phycisphaera**	1	9	0	0	0	0	0	0	0	0	0	0	0	0	0	0	0	0	0	0
**Phyllobacterium**	1	6	0	0	0	0	0	0	0	0	0	0	0	0	0	0	0	0	0	0
**Picrophilus**	1	15	0	0	0	0	0	0	0	0	0	1	0	0	0	0	0	0	0	0
**Pirellula**	2	85	0	0	0	0	0	0	0	0	0	1	0	0	0	0	0	0	0	0
**Planctomyces**	2	93	0	0	0	0	0	0	0	0	0	0	0	0	0	0	0	0	0	0
**Plautia**	1	17	0	0	0	0	0	0	0	0	0	0	0	0	0	0	0	0	0	0
**Plesiomonas**	1	4	0	0	0	0	0	0	0	0	0	0	0	0	0	0	0	0	0	0
**Pleurocapsa**	1	23	0	0	0	0	0	0	0	0	0	0	0	0	0	0	0	0	0	0
**Polaribacter**	2	74	0	0	0	0	0	0	0	0	0	0	0	0	0	0	0	0	0	0
**Polaromonas**	3	238	0	0	0	1	0	0	0	0	1	1	1	1	0	1	1	0	0	1
**Polymorphum**	3	173	0	0	1	1	1	0	0	0	0	1	1	0	1	0	1	0	0	0
**Polynucleobacter**	1	19	0	0	0	0	0	0	0	0	0	0	0	0	0	0	0	0	0	0
**Ponticaulis**	1	1	0	0	0	0	0	0	0	0	0	0	0	0	0	0	0	0	0	0
**Porphyromonas**	2	63	0	0	0	0	0	0	0	0	0	0	0	0	0	0	0	0	0	0
**Prevotella**	1	65	0	0	0	0	0	0	0	0	0	0	0	0	0	0	0	0	0	0
**Prochlorococcus**	2	68	0	0	0	0	0	0	0	0	0	0	0	0	0	0	0	0	0	0
**Propionibacterium**	1	62	0	0	0	0	0	0	0	0	0	0	0	0	0	0	0	0	0	0
**Prosthecochloris**	1	16	0	0	0	0	0	0	0	0	0	0	0	0	0	0	0	0	0	0
**Proteobacteria**	1	19	0	0	0	0	0	0	0	0	0	0	0	0	0	0	0	0	0	0
**Proteus**	2	113	0	0	0	0	0	0	0	0	0	0	0	0	0	0	0	0	0	0
**Providencia**	1	38	0	0	0	0	0	0	0	0	0	0	0	0	0	0	0	0	0	0
**Pseudaminobacter**	1	10	0	0	0	0	0	0	0	0	0	0	0	0	0	0	0	0	0	0
**Pseudanabaena**	1	11	0	0	0	0	0	0	0	0	0	0	0	0	0	0	0	0	0	0
**Pseudoalteromonas**	2	188	0	0	0	0	1	0	0	0	0	0	1	0	0	1	0	0	0	0
**Pseudochrobactrum**	1	7	0	0	0	0	0	0	0	0	0	0	0	0	0	0	0	0	0	0
**Pseudogulbenkiania**	1	40	0	0	0	1	0	0	0	0	1	0	0	0	1	0	0	0	0	0
**pseudomallei**	3	131	0	0	0	0	0	0	0	0	1	0	0	0	0	0	0	0	0	0

Genus	Cluster Number	Protein Counts	K03333	K03379	K03380	K03381	K03382	K03383	K03384	K03391	K03464	K03518	K03862	K04098	K04099	K04100	K04102	K04105	K04107	K04110
Pseudomonas	3	381	1	1	0	1	1	0	0	0	1	1	1	0	1	1	0	0	0	0
Pseudonocardia	3	180	0	0	0	1	1	1	0	0	1	1	1	1	0	1	0	0	0	1
Pseudorhodobacter	1	4	0	0	0	0	0	0	0	0	0	0	0	0	0	0	0	0	0	0
Pseudovibrio	1	33	0	0	0	0	0	0	0	0	0	1	0	0	0	0	0	0	0	0
Pseudoxanthomonas	2	124	0	0	0	0	0	0	0	0	0	0	1	0	0	0	0	0	0	0
Psychrobacter	2	124	1	0	0	1	1	0	0	0	1	0	0	0	0	0	0	0	0	0
Psychroflexus	1	22	1	0	0	0	0	0	0	0	0	0	0	0	0	0	0	0	0	0
Psychromonas	2	110	0	0	0	0	0	0	0	0	0	0	0	0	0	0	0	0	0	0
Pusillimonas	3	111	0	0	0	0	0	0	0	0	0	0	1	0	0	0	0	0	0	0
Pyrobaculum	1	15	0	0	0	0	0	0	0	0	0	1	0	0	0	0	0	0	0	0
Pyrococcus	1	10	0	0	0	0	0	0	0	0	0	0	0	0	0	0	0	0	0	0
Pyrolobus	1	4	0	0	0	0	0	0	0	0	0	0	0	0	0	0	0	0	0	0
Rahnella	2	143	0	0	0	0	1	0	0	0	0	0	0	0	1	0	1	0	0	0
Ralstonia	3	332	1	1	0	1	1	0	0	0	1	1	1	1	1	0	1	0	0	1
Ramlibacter	1	35	0	0	0	0	0	0	0	0	0	1	0	0	0	1	0	0	0	1
Raoultella	2	139	0	0	0	1	1	0	0	0	1	1	1	0	1	0	0	0	0	0
Raphidiopsis	1	5	0	0	0	0	0	0	0	0	0	0	0	0	0	0	0	0	0	0
Reinekea	1	2	0	0	0	0	0	0	0	0	0	0	0	0	0	0	0	0	0	0
Renibacterium	2	67	0	0	1	0	0	0	0	0	0	0	0	0	0	0	0	0	0	0
Rhizobiaceae	1	3	0	0	0	0	0	0	0	0	0	0	0	0	0	0	0	0	0	0
Rhizobiales	1	6	0	0	0	0	0	0	0	0	0	1	0	0	0	0	0	0	0	0
Rhizobium	3	257	0	0	1	1	1	1	0	0	0	1	0	0	0	0	1	0	0	0
Rhizobium/Agrobacterium	2	87	0	0	1	1	0	0	0	0	0	0	0	0	0	0	0	0	0	0
Rhodanobacter	1	21	0	0	0	0	0	0	0	0	0	0	0	0	0	0	0	0	0	0
Rhodobacter	3	175	0	0	0	0	1	0	0	0	0	1	0	0	0	0	0	0	0	0
Rhodobacteraceae	1	21	0	0	0	0	0	0	0	0	0	0	0	0	0	0	0	0	0	0
Rhodobacterales	1	34	0	0	0	0	0	0	0	0	0	1	0	0	0	0	0	0	0	0
Rhodococcus	2	292	1	1	0	1	1	1	1	1	1	1	1	1	0	0	1	0	0	0
Rhodocyclaceae	1	7	0	0	0	0	0	0	0	0	0	0	0	0	0	0	0	0	0	0
Rhodoferax	3	144	1	0	0	0	0	0	0	0	0	0	1	0	0	1	0	0	0	1
Rhodomicrobium	2	103	0	0	0	0	0	0	0	0	0	0	0	0	0	0	0	0	0	1
Rhodopirellula	2	87	0	0	0	0	0	0	0	0	0	0	0	0	0	0	0	0	0	0
Rhodopseudomonas	3	246	0	0	0	0	0	0	0	0	0	1	1	0	0	1	0	1	1	1
Rhodospirillum	2	121	0	0	0	0	0	0	0	0	0	1	0	0	0	0	0	0	0	1
Rhodothermus	2	76	0	0	0	0	0	0	0	0	0	1	0	0	0	0	0	0	0	0
Rhodovulum	1	4	0	0	0	0	0	0	0	0	0	0	0	0	0	0	0	0	0	0
Richelia	1	2	0	0	0	0	0	0	0	0	0	0	0	0	0	0	0	0	0	0
Rickettsia	2	43	0	0	0	0	0	0	0	0	0	0	0	0	0	0	0	0	0	0
Rickettsiaceae	1	5	0	0	0	0	0	0	0	0	0	0	0	0	0	0	0	0	0	0
Riemerella	2	61	0	0	0	0	0	0	0	0	0	0	0	0	0	0	0	0	0	0
Rivularia	1	18	1	0	0	0	1	0	0	0	0	0	0	0	0	0	0	0	0	0
Robiginitalea	2	83	0	0	0	0	0	0	0	0	0	0	0	0	0	0	0	0	0	0
Roseburia	2	58	0	0	0	0	0	0	0	0	0	1	0	0	0	0	0	0	0	0
Roseibium	1	3	0	0	0	0	0	0	0	0	0	0	0	0	0	0	0	0	0	0
Roseiflexus	3	100	0	0	0	0	0	0	0	0	0	1	0	0	0	0	0	0	0	0
Roseobacter	2	149	0	0	0	0	1	0	0	0	0	1	1	0	0	0	0	0	0	0
Roseomonas	1	2	0	0	0	0	0	0	0	0	0	0	0	0	0	0	0	0	0	0
Roseovarius	1	15	0	0	0	0	0	0	0	0	0	0	0	0	0	0	0	0	0	0
Rothia	1	44	0	0	0	0	0	0	0	0	0	0	0	0	0	0	0	0	0	0
Rubrivivax	1	24	0	0	0	0	1	0	0	0	0	1	0	0	0	0	0	0	0	0
Rubrobacter	3	117	0	0	1	0	1	0	0	0	0	1	0	0	0	0	0	0	0	0
Ruegeria	2	189	0	0	0	0	1	0	0	0	0	1	0	0	0	0	0	1	0	0

Genus	Cluster Number	Protein Counts	K03333	K03379	K03380	K03381	K03382	K03383	K03384	K03391	K03464	K03518	K03862	K04098	K04099	K04100	K04102	K04105	K04107	K04110
**Ruminococcaceae**	1	2	0	0	0	0	0	0	0	0	0	0	0	0	0	0	0	0	0	0
**Ruminococcus**	2	101	0	0	0	0	0	0	0	0	0	1	0	0	0	0	0	0	0	0
**Runella**	1	26	0	0	0	0	0	0	0	0	1	0	0	0	0	0	0	0	0	0
**Saccharomonospora**	2	132	1	0	0	1	0	1	0	0	1	1	1	0	0	1	0	0	0	0
**Saccharophagus**	2	81	0	0	0	0	0	0	0	0	0	0	0	0	0	0	0	0	0	0
**Saccharopolyspora**	3	194	1	1	0	1	1	0	0	1	1	1	1	0	0	0	0	0	0	0
**Saccharothrix**	1	32	1	0	0	1	1	0	0	0	0	1	0	0	0	0	0	0	0	0
**Sagittula**	1	10	0	0	0	0	0	0	0	0	0	0	0	0	0	0	0	0	0	0
**Salinibacter**	2	77	0	0	0	0	0	0	0	0	0	0	0	0	0	0	0	0	0	0
**Salinimonas**	1	1	0	0	0	0	0	0	0	0	0	0	0	0	0	0	0	0	0	0
**Salinispora**	3	127	1	0	0	0	0	0	0	0	0	0	0	0	0	0	0	0	0	0
**Salinivibrio**	1	2	0	0	0	0	0	0	0	0	0	0	0	0	0	0	0	0	0	0
**Salmonella**	2	151	0	0	0	0	0	0	0	0	0	0	0	0	0	0	0	0	0	0
**Sanguibacter**	2	73	0	0	0	0	1	0	0	0	0	0	0	0	0	0	0	0	0	0
**Saprospira**	1	13	0	0	0	0	0	0	0	0	0	0	0	0	0	0	0	0	0	0
**Scardovia**	1	1	0	0	0	0	0	0	0	0	0	0	0	0	0	0	0	0	0	0
**Scytonema**	1	10	0	0	0	0	0	0	0	0	0	0	0	0	0	0	0	0	0	0
**Sebaldella**	2	69	0	0	0	0	1	0	0	0	0	1	0	0	0	0	0	0	0	0
**Segniliparus**	2	85	1	0	0	0	0	0	0	0	0	0	0	0	0	0	0	0	0	0
**Selenomonas**	2	57	0	0	0	0	0	0	0	0	0	0	0	0	0	0	0	0	0	0
**Serinicoccus**	1	1	0	0	0	0	0	0	0	0	0	0	0	0	0	0	0	0	0	0
**Serratia**	2	186	0	0	0	1	0	0	0	0	1	0	0	0	1	0	0	0	0	0
**Shewanella**	3	214	0	0	0	0	0	0	0	0	0	1	0	0	0	0	0	0	0	0
**Shigella**	2	172	0	0	0	0	0	0	0	0	0	0	0	0	0	0	0	0	0	0
**Shinella**	1	2	0	0	0	0	0	0	0	0	0	0	0	0	0	0	0	0	0	0
**Sideroxydans**	2	81	1	0	0	0	0	0	0	0	0	0	0	0	0	0	0	0	0	0
**Silicibacter**	2	105	0	0	0	0	1	0	0	0	0	1	0	0	0	0	0	0	0	0
**Simiduia**	1	18	0	0	0	0	0	0	0	0	0	0	0	0	0	0	0	0	0	0
**Simkania**	1	6	0	0	0	0	0	0	0	0	0	0	0	0	0	0	0	0	0	0
**Simonsiella**	1	2	0	0	0	0	0	0	0	0	0	0	0	0	0	0	0	0	0	0
**Singulisphaera**	1	18	0	0	0	1	0	0	0	0	0	0	0	0	0	0	0	0	0	0
**Sinorhizobium**	3	207	0	0	0	1	0	0	0	0	1	1	0	0	0	0	0	0	0	0
**Slackia**	2	46	0	0	0	0	0	0	0	0	0	0	0	0	0	0	0	0	0	0
**Smaragdicoccus**	1	3	0	0	0	0	0	0	0	0	0	0	0	0	0	0	0	0	0	0
**Sodalis**	2	71	0	0	0	0	0	0	0	0	0	0	0	0	0	0	0	0	0	0
**Solibacillus**	1	23	0	0	0	0	0	0	0	0	0	0	0	0	0	0	0	0	0	0
**Solitalea**	1	13	0	0	0	0	0	0	0	0	0	0	0	0	0	0	0	0	0	0
**Sorangium**	3	115	1	1	0	0	0	0	0	0	0	1	0	0	0	0	0	0	0	0
**Sphaerobacter**	2	100	0	0	0	0	0	0	0	0	0	1	0	0	0	0	0	0	0	0
**Sphaerochaeta**	1	42	0	0	0	0	1	0	0	0	0	1	0	0	0	0	0	0	0	0
**Sphingobacterium**	3	85	0	0	0	0	0	0	0	0	0	0	0	0	0	0	0	0	0	0
**Sphingobium**	1	179	0	0	0	1	0	0	0	0	1	0	1	1	1	1	0	0	0	0
**Sphingomonadaceae**	1	15	0	0	0	0	0	0	0	0	0	0	1	0	0	0	0	0	0	0
**Sphingomonas**	2	220	0	0	0	1	0	0	0	0	1	1	1	1	1	1	0	0	0	1
**Sphingopyxis**	3	99	0	0	0	0	0	0	0	0	0	0	0	0	0	0	0	0	0	0
**Spirochaeta**	2	103	0	0	0	0	1	0	0	0	0	1	0	0	0	0	0	0	0	0
**Spiroplasma**	1	6	0	0	0	0	0	0	0	0	0	0	0	0	0	0	0	0	0	0
**Spirosoma**	2	107	0	0	0	0	0	0	0	0	0	0	0	0	0	0	0	0	0	0
**Stackebrandtia**	2	110	1	0	0	0	1	0	0	0	0	1	0	0	0	0	0	0	0	0
**Stanieria**	1	17	0	0	0	0	0	0	0	0	0	0	0	0	0	0	0	0	0	0
**Staphylococcus**	2	108	0	0	0	0	0	0	0	0	0	0	0	0	0	0	0	0	0	0
**Staphylothermus**	1	12	0	0	0	0	0	0	0	0	0	1	0	0	0	0	0	0	0	0

Genus	Cluster Number	Protein Counts	K03333	K03379	K03380	K03381	K03382	K03383	K03384	K03391	K03464	K03518	K03862	K04098	K04099	K04100	K04102	K04105	K04107	K04110
Stappia	1	2	0	0	0	0	0	0	0	0	0	0	0	0	0	0	0	0	0	0
Starkeya	3	134	0	0	0	0	1	0	0	0	0	1	0	0	0	0	0	0	0	0
Stenotrophomonas	3	104	0	0	0	1	0	0	0	0	0	0	0	0	0	0	0	0	0	0
Stigmatella	3	129	1	0	0	0	0	0	0	0	0	1	1	0	0	0	0	0	0	0
Streptobacillus	1	38	0	0	0	0	0	0	0	0	0	0	0	0	0	0	0	0	0	0
Streptococcus	2	121	0	0	0	0	0	0	0	0	0	0	0	0	0	0	0	0	0	0
Streptomyces	2	253	1	0	0	1	1	0	0	0	1	1	1	1	0	0	1	0	0	1
Streptosporangium	3	148	1	0	0	0	1	1	0	0	0	1	0	0	0	1	0	0	0	0
Strigomonas	1	2	0	0	0	0	0	0	0	0	0	0	0	0	0	0	0	0	0	0
Sulfitobacter	1	10	0	0	0	0	0	0	0	0	0	0	0	0	0	0	0	0	0	0
Sulfobacillus	1	30	0	0	0	0	0	1	0	0	0	1	0	0	0	0	0	0	0	0
Sulfolobus	1	23	0	0	0	0	0	0	0	0	0	1	0	0	0	0	0	0	0	0
Sulfuricurvum	2	67	0	0	0	0	0	0	0	0	0	0	0	0	0	0	0	0	0	0
Sulfurihydrogenibium	2	55	0	0	0	0	0	0	0	0	0	0	0	0	0	0	0	0	0	0
Sulfurimonas	2	60	0	0	0	0	0	0	0	0	0	0	0	0	0	0	0	0	0	0
Sulfurospirillum	1	48	0	0	0	0	0	0	0	0	0	0	0	0	0	0	0	0	0	0
Sulfurovum	2	48	0	0	0	0	0	0	0	0	0	0	0	0	0	0	0	0	0	0
Symbiobacterium	2	68	0	0	0	0	0	0	0	0	0	0	0	0	0	0	0	0	0	0
Synechococcus	1	34	1	0	0	0	1	0	0	0	0	0	0	0	0	0	0	0	0	0
Synechocystis	1	40	0	0	0	0	0	0	0	0	0	0	0	0	0	0	0	0	0	0
Synergistetes	1	27	0	0	0	0	0	0	0	0	0	1	0	0	0	0	0	0	0	0
Syntrophobacter	2	101	0	0	0	0	0	0	0	0	0	1	0	0	0	0	0	0	0	0
Syntrophobotulus	2	71	0	0	0	0	0	0	0	0	0	0	0	0	0	0	0	0	0	0
Syntrophomonas	2	58	0	0	0	0	0	0	0	0	0	1	0	0	0	0	0	0	0	0
Syntrophothermus	2	60	0	0	0	0	0	0	0	0	0	0	0	0	0	0	0	0	0	0
Syntrophus	2	66	0	0	0	0	0	0	0	0	0	0	0	0	0	0	0	0	0	1
Tannerella	1	8	0	0	0	0	0	0	0	0	0	0	0	0	0	0	0	0	0	0
Taylorella	1	43	0	0	0	0	0	0	0	0	0	0	0	0	0	0	0	0	0	0
Tepidanaerobacter	2	55	0	0	0	0	0	0	0	0	0	0	0	0	0	0	0	0	0	0
Teredinibacter	2	109	0	0	0	0	1	0	0	0	0	0	0	0	0	0	0	0	0	0
Terrabacter	1	1	0	0	0	0	0	0	0	0	0	0	0	0	0	0	0	0	0	0
Terriglobus	2	83	0	0	0	0	0	0	0	0	0	1	1	0	0	0	0	0	0	0
Tetragenococcus	1	12	0	0	0	0	0	0	0	0	0	0	0	0	0	0	0	0	0	0
Thalassiobium	1	11	0	0	0	0	0	0	0	0	0	0	1	0	0	0	0	0	0	0
Thalassobacter	1	9	0	0	0	0	0	0	0	0	0	0	1	0	0	0	0	0	0	0
Thalassolituus	1	12	1	0	0	0	1	0	0	0	0	0	0	0	0	0	0	0	0	0
Thalassospira	1	2	0	0	0	0	0	0	0	0	0	0	0	0	0	0	0	0	0	0
Thauera	1	111	0	0	0	0	0	0	0	0	0	0	0	0	0	1	0	0	1	1
Thermacetogenium	1	7	0	0	0	0	0	0	0	0	0	0	0	0	0	0	0	0	0	0
Thermaerobacter	2	79	0	0	0	0	0	0	0	0	0	1	0	0	0	0	0	0	0	0
Thermanaerovibrio	2	58	0	0	0	0	0	0	0	0	0	1	0	0	0	0	0	0	0	0
Thermincola	2	62	0	0	0	0	0	0	0	0	0	0	0	0	0	0	0	0	0	0
Thermoanaerobacter	2	76	0	0	0	0	0	0	0	0	0	0	0	0	0	0	0	0	0	0
Thermoanaerobacterium	2	64	0	0	0	0	0	0	0	0	0	0	0	0	0	0	0	0	0	0
Thermobacillus	1	11	0	0	0	0	0	0	0	0	0	0	0	0	0	0	0	0	0	0
Thermobaculum	2	62	0	0	0	0	0	0	0	0	0	1	0	0	0	0	0	0	0	0
Thermobifida	2	72	1	0	0	0	0	0	0	0	0	1	0	0	0	0	0	0	0	0
Thermobispora	2	109	1	0	0	0	1	1	0	0	0	1	0	0	0	0	0	0	0	0
Thermococcus	1	13	0	0	0	0	0	0	0	0	0	0	0	0	0	0	0	0	0	0
Thermocrinis	2	60	0	0	0	0	0	0	0	0	0	0	0	0	0	0	0	0	0	0
Thermodesulfatator	1	10	0	0	0	0	0	0	0	0	0	0	0	0	0	0	0	0	0	0
Thermodesulfobacterium	1	9	0	0	0	0	0	0	0	0	0	0	0	0	0	0	0	0	0	0

Genus	Cluster Number	Protein Counts	K03333	K03379	K03380	K03381	K03382	K03383	K03384	K03391	K03464	K03518	K03862	K04098	K04099	K04100	K04102	K04105	K04107	K04110
Thermodesulfobium	1	13	0	0	0	0	0	0	0	0	0	0	0	0	0	0	0	0	0	0
Thermodesulfovibrio	2	60	0	0	0	0	0	0	0	0	0	0	0	0	0	0	0	0	0	0
Thermofilum	1	9	0	0	0	0	0	0	0	0	0	0	0	0	0	0	0	0	0	0
Thermogladius	1	7	0	0	0	0	0	0	0	0	0	1	0	0	0	0	0	0	0	0
Thermomicrobium	2	92	0	0	0	0	0	0	0	0	0	1	0	0	0	0	0	0	0	0
Thermomonospora	2	125	1	0	0	0	0	0	0	0	0	1	0	0	0	0	0	0	0	0
Thermoplasma	1	11	0	0	0	0	0	0	0	0	0	0	0	0	0	0	0	0	0	0
Thermoplasmatales	1	6	0	0	0	0	0	0	0	0	0	0	0	0	0	0	0	0	0	0
Thermoproteus	1	12	0	0	0	0	0	0	0	0	0	1	0	0	0	0	0	0	0	0
Thermosediminibacter	2	64	0	0	0	0	0	0	0	0	0	0	0	0	0	0	0	0	0	0
Thermosipho	2	70	0	0	0	0	1	0	0	0	0	1	0	0	0	0	0	0	0	0
Thermosphaera	1	10	0	0	0	0	0	0	0	0	0	1	0	0	0	0	0	0	0	0
Thermosynechococcus	1	6	0	0	0	0	0	0	0	0	0	0	0	0	0	0	0	0	0	0
Thermotoga	2	80	0	0	0	0	0	0	0	0	0	0	0	0	0	0	0	0	0	0
Thermovibrio	2	58	0	0	0	0	0	0	0	0	0	0	0	0	0	0	0	0	0	0
Thermovirga	1	9	0	0	0	0	0	0	0	0	0	0	0	0	0	0	0	0	0	0
Thermus	2	107	0	0	0	0	0	0	0	0	0	0	0	0	0	0	0	0	0	0
Thioalkalimicrobium	1	4	0	0	0	0	0	0	0	0	0	0	0	0	0	0	0	0	0	0
Thioalkalivibrio	2	91	0	0	0	0	0	0	0	0	0	0	0	0	0	0	0	0	0	0
Thiobaca	1	2	0	0	0	0	0	0	0	0	0	0	0	0	0	0	0	0	0	0
Thiobacillus	2	67	0	0	0	0	0	0	0	0	0	0	0	0	0	0	0	0	0	0
Thiocapsa	1	2	0	0	0	0	0	0	0	0	0	0	0	0	0	0	0	0	0	0
Thiocystis	1	11	0	0	0	0	0	0	0	0	0	0	0	0	0	0	0	0	0	0
Thioflavicoccus	1	13	0	0	0	0	0	0	0	0	0	0	0	0	0	0	0	0	0	0
Thiomicrospira	2	50	0	0	0	0	0	0	0	0	0	0	0	0	0	0	0	0	0	0
Thiomonas	2	85	0	0	0	0	1	0	0	0	0	0	0	0	0	1	0	0	0	1
Thiorhodococcus	1	2	0	0	0	0	0	0	0	0	0	0	0	0	0	0	0	0	0	0
Thiothrix	1	2	0	0	0	0	0	0	0	0	0	0	0	0	0	0	0	0	0	0
Tistrella	1	42	0	0	0	0	0	0	0	0	0	1	0	0	0	1	0	0	0	0
Tolumonas	2	100	0	0	0	0	0	0	0	0	0	0	0	0	0	0	0	0	0	0
Transposon	1	2	0	0	0	0	0	0	0	0	0	0	0	0	0	0	0	0	0	0
Treponema	1	63	0	0	0	0	0	0	0	0	0	1	0	0	0	0	0	0	0	0
Trichodesmium	2	71	0	0	0	0	0	0	0	0	0	0	0	0	0	0	0	0	0	0
Trichormus	1	12	0	0	0	0	0	0	0	0	0	0	0	0	0	0	0	0	0	0
Tropheryma	2	22	0	0	0	0	0	0	0	0	0	0	0	0	0	0	0	0	0	0
Truepera	2	104	0	0	0	0	0	0	0	0	0	0	0	0	0	0	0	0	0	0
Tsukamurella	2	123	1	0	0	0	0	0	0	0	0	0	0	0	0	0	0	0	0	0
Turneriella	1	16	1	0	0	0	0	0	0	0	0	0	0	0	0	0	0	0	0	0
Uliginosibacterium	1	2	0	0	0	0	0	0	0	0	0	0	0	0	0	0	0	0	0	0
Ureaplasma	1	24	0	0	0	0	0	0	0	0	0	0	0	0	0	0	0	0	0	0
Ureibacillus	1	2	0	0	0	0	0	0	0	0	0	0	0	0	0	0	0	0	0	0
Variovorax	3	187	0	0	0	0	1	0	0	0	1	1	1	1	1	0	0	0	0	1
Veillonella	1	54	0	0	0	0	0	0	0	0	0	1	0	0	0	0	0	0	0	0
Verminephrobacter	3	188	0	0	0	0	0	0	0	0	0	1	1	1	1	1	1	0	0	1
Verrucosispora	3	126	1	0	0	0	0	0	0	0	0	0	1	0	0	0	0	0	0	0
Vibrio	2	195	0	0	0	1	1	0	0	0	1	0	0	0	0	1	0	0	0	0
Vibrionales	1	46	0	0	0	0	0	0	0	0	0	0	0	0	0	0	0	0	0	0
Vitreoscilla	1	1	0	0	0	0	0	0	0	0	0	0	0	0	0	0	0	0	0	0
Vulcanisaeta	1	14	0	0	0	0	0	0	0	0	0	1	0	0	0	0	0	0	0	0
Waddlia	2	68	0	0	0	0	0	0	0	0	0	0	0	0	0	0	0	0	0	0
Wautersia	1	2	0	0	0	0	0	0	0	0	0	0	0	0	0	0	0	0	0	0
Weeksella	2	65	1	0	0	0	0	0	0	0	0	0	0	0	0	0	0	0	0	0

Genus	Cluster Number	Protein Counts	K03333	K03379	K03380	K03381	K03382	K03383	K03384	K03391	K03464	K03518	K03862	K04098	K04099	K04100	K04102	K04105	K04107	K04110
**Weissella**	1	14	0	0	0	0	0	0	0	0	0	0	0	0	0	0	0	0	0	0
**Wenxinia**	1	2	0	0	0	0	0	0	0	0	0	0	0	0	0	0	0	0	0	0
**Wigglesworthia**	1	29	0	0	0	0	0	0	0	0	0	0	0	0	0	0	0	0	0	0
**Wolbachia**	1	32	0	0	0	0	0	0	0	0	0	0	0	0	0	0	0	0	0	0
**Wolinella**	2	51	0	0	0	0	0	0	0	0	0	0	0	0	0	0	0	0	0	0
**Xanthobacter**	3	176	0	0	0	1	1	0	0	0	1	1	0	0	0	0	0	0	0	0
**Xanthobacteraceae**	1	4	0	0	0	0	0	0	0	0	0	0	0	0	0	0	0	0	0	0
**Xanthomonadaceae**	1	39	0	0	0	0	0	0	0	0	0	0	0	0	0	0	0	0	0	0
**Xanthomonas**	2	162	0	0	0	0	0	0	0	0	0	0	1	0	0	1	0	0	0	0
**Xenorhabdus**	2	127	0	0	0	0	0	0	0	0	0	0	0	0	0	0	0	0	0	0
**Xylanimonas**	2	66	1	0	0	0	0	0	0	0	0	0	0	0	0	0	0	0	0	0
**Xylella**	2	52	0	0	0	0	0	0	0	0	0	0	0	0	0	0	0	0	0	0
**Yersinia**	2	145	0	0	0	0	0	0	0	0	0	0	0	0	0	0	0	0	0	0
**Yokenella**	2	61	0	0	0	0	0	0	0	0	0	0	0	0	0	0	0	0	0	0
**Zobellia**	1	28	0	0	0	0	0	0	0	0	0	0	0	0	0	0	0	0	0	0
**Zunongwangia**	2	98	0	0	0	0	0	0	0	0	0	0	0	0	0	0	0	0	0	0
**Zymomonas**	2	67	0	0	0	0	0	0	0	0	0	0	0	0	0	0	0	0	0	0
**Zymophilus**	1	2	0	0	0	0	0	0	0	0	0	0	0	0	0	0	0	0	0	0

Genus	Cluster Number	Protein Counts	K04112	K04116	K04117	K04118	K05296	K05394	K05549	K05599	K05783	K05898	K05913	K06281	K06446	K06912	K07408	K07535	K07536	K07537
Acaricomes	1	2	0	0	0	0	0	0	0	0	0	0	0	0	0	0	0	0	0	0
Acaryochloris	2	88	0	0	0	0	0	0	0	0	0	0	0	0	0	0	0	0	0	0
Acetivibrio	1	2	0	0	0	0	0	0	0	0	0	0	0	0	0	0	0	0	0	0
Acetobacter	2	85	0	0	0	0	0	0	0	0	0	0	0	0	0	0	0	0	0	0
Acetobacteraceae	1	2	0	0	0	0	0	0	0	0	0	0	0	0	0	0	0	0	0	0
Acetobacterium	1	8	0	0	0	0	0	0	0	0	0	0	0	0	0	0	0	0	0	0
Acetohalobium	2	79	0	0	0	0	0	0	0	0	0	0	0	1	0	0	0	0	0	0
Acholeplasma	2	38	0	0	0	0	0	0	0	0	0	0	0	0	0	0	0	0	0	0
Achromobacter	2	192	0	0	0	0	0	0	0	0	1	0	0	0	1	0	0	1	1	0
Acidaminococcus	1	65	0	0	0	0	0	0	0	0	0	0	0	0	0	0	0	0	0	0
Acidianus	1	9	0	0	0	0	0	0	0	0	0	0	0	0	0	0	0	0	0	0
Acidilobus	1	8	0	0	0	0	0	0	0	0	0	0	0	0	0	0	0	0	0	0
Acidimicrobium	2	58	0	0	0	0	0	0	0	0	0	0	0	0	0	0	0	0	0	0
Acidiphilium	3	144	0	0	0	0	0	0	0	0	0	0	0	1	1	0	0	0	0	0
Acidithiobacillus	2	70	0	0	0	0	0	0	0	0	0	0	0	1	0	0	0	0	0	0
Acidobacterium	2	91	0	0	0	0	0	0	0	0	0	0	0	1	0	0	0	0	0	0
Acidocella	1	2	0	0	0	0	0	0	0	0	0	0	0	0	0	0	0	0	0	0
Acidothermus	2	68	0	0	0	0	0	0	0	0	0	0	0	0	0	0	0	0	0	0
Acidovorax	3	217	0	0	1	0	0	0	0	0	0	0	0	0	1	0	0	0	1	0
Aciduliprofundum	1	11	0	0	0	0	0	0	0	0	0	0	0	0	0	0	0	0	0	0
Acinetobacter	3	188	0	0	0	0	0	0	1	1	1	0	0	0	1	0	0	0	0	0
Actinobacillus	2	112	0	0	0	0	0	0	0	0	0	0	0	1	0	0	0	0	0	0
Actinomadura	1	1	0	0	0	0	0	0	0	0	0	0	0	0	0	0	0	0	0	0
Actinoplanes	1	52	0	0	0	0	0	0	0	0	0	1	0	0	0	0	0	0	0	0
Actinopolyspora	1	2	0	0	0	0	0	0	0	0	0	0	0	0	0	0	0	0	0	0
Actinosynnema	2	122	0	0	0	0	0	0	0	0	0	0	0	0	0	0	0	0	0	0
Advenella	1	30	0	0	0	0	0	0	0	0	0	0	0	0	1	1	0	0	1	0
Aequorivita	1	12	0	0	0	0	0	0	0	0	0	0	0	0	0	0	0	0	0	0
Aerococcus	2	57	0	0	0	0	0	0	0	0	0	0	0	0	0	0	0	0	0	0
Aeromonas	2	127	0	0	0	0	0	0	0	0	0	0	0	1	0	0	0	0	0	0
Aeropyrum	1	13	0	0	0	0	0	0	0	0	0	0	0	0	0	0	0	0	0	0
Afipia	1	54	0	0	0	0	0	0	1	0	0	0	0	0	0	0	0	0	0	0
Aggregatibacter	1	81	0	0	0	0	0	0	0	0	0	0	0	1	0	0	0	0	0	0
Agrobacterium	2	242	0	0	0	0	0	0	1	0	1	0	0	0	0	0	0	0	0	0
Ahrensia	1	2	0	0	0	0	0	0	0	0	0	0	0	0	0	0	0	0	0	0
Akkermansia	2	47	0	0	0	0	0	0	0	0	0	0	0	1	0	0	0	0	0	0
Albidiferax	3	144	0	0	0	0	0	0	0	0	0	0	0	1	0	0	0	0	0	0
Alcaligenes	1	21	0	0	0	0	0	0	0	0	0	0	0	0	0	0	0	0	0	0
Alcanivorax	2	146	0	0	0	0	0	0	0	0	0	0	0	0	1	0	0	0	0	0
Alicycliphilus	3	152	0	1	1	1	0	0	0	0	1	0	0	0	1	0	0	1	1	0
Alicyclobacillus	2	91	0	1	1	0	0	0	0	0	0	0	0	0	1	0	0	1	0	0
Aliivibrio	2	118	0	0	0	0	0	0	0	0	0	0	0	0	0	0	0	0	0	0
Alishewanella	1	1	0	0	0	0	0	0	0	0	0	0	0	0	0	0	0	0	0	0
Alistipes	1	52	0	0	0	0	0	0	0	0	0	0	0	0	0	0	0	0	0	0
Alkalilimnicola	2	97	0	0	0	0	0	0	0	0	0	0	0	1	0	0	0	0	0	0
Alkaliphilus	2	62	0	0	0	0	0	0	0	0	0	0	0	0	0	0	0	0	0	0
Allochromatium	2	84	0	0	0	0	0	0	0	0	0	0	0	1	0	0	0	0	0	0
Alphaproteobacteria	1	3	0	0	0	0	0	0	0	0	0	0	0	0	0	0	0	0	0	0
Alteromonadales	1	12	0	0	0	0	0	0	0	0	0	0	0	0	0	0	0	0	0	0
Alteromonas	1	37	0	0	0	0	0	0	1	0	1	0	0	1	0	0	0	0	0	0
Aminobacter	1	4	0	0	0	0	0	0	0	0	0	0	0	0	0	0	0	0	0	0
Aminobacterium	2	59	0	0	0	0	0	0	0	0	0	0	0	0	0	0	0	0	0	0

Genus	Cluster Number	Protein Counts	K04112	K04116	K04117	K04118	K05296	K05394	K05549	K05599	K05783	K05898	K05913	K06281	K06446	K06912	K07408	K07535	K07536	K07537
**Ammonifex**	2	50	0	0	0	0	0	0	0	0	0	0	0	1	0	0	0	0	0	0
**Amphibacillus**	1	13	0	0	0	0	0	0	0	0	0	0	0	0	0	0	0	0	0	0
**Amphritea**	1	2	0	0	0	0	0	0	0	0	0	0	0	0	0	0	0	0	0	0
**Amycolatopsis**	2	201	0	0	0	0	0	0	0	0	0	1	0	1	1	0	0	1	0	0
**Amycolicicoccus**	1	43	0	0	0	0	0	0	0	0	0	1	0	1	0	0	0	0	0	0
**Anabaena**	2	92	0	0	0	0	0	0	0	0	0	0	0	1	0	0	0	0	0	0
**Anaerobaculum**	1	11	0	0	0	0	0	0	0	0	0	0	0	0	0	0	0	0	0	0
**Anaerococcus**	2	46	0	0	0	0	0	0	0	0	0	0	0	0	0	0	0	0	0	0
**Anaerolinea**	2	66	0	0	0	0	0	0	0	0	0	0	0	0	0	0	0	0	0	0
**Anaeromyxobacter**	3	117	0	0	0	0	0	0	0	0	0	0	0	1	0	0	0	0	0	0
**Anaerostipes**	1	44	0	0	0	0	0	0	0	0	0	0	0	0	0	0	0	0	0	0
**Anaerotruncus**	1	2	0	0	0	0	0	0	0	0	0	0	0	0	0	0	0	0	0	0
**Anaplasma**	1	35	0	0	0	0	0	0	0	0	0	0	0	0	0	0	0	0	0	0
**Ancylobacter**	1	3	0	0	0	0	0	0	0	0	0	0	0	1	0	0	0	0	0	0
**Aneurinibacillus**	1	1	0	0	0	0	0	0	0	0	0	0	0	0	0	0	0	0	0	0
**Angomonas**	1	1	0	0	0	0	0	0	0	0	0	0	0	0	0	0	0	0	0	0
**Anoxybacillus**	2	72	0	0	0	0	0	0	0	0	0	0	0	0	0	0	0	0	0	0
**Aquifex**	2	45	0	0	0	0	0	0	0	0	0	0	0	1	0	0	0	0	0	0
**Aquimarina**	1	2	0	0	0	0	0	0	0	0	0	0	0	0	0	0	0	0	0	0
**Arcanobacterium**	2	45	0	0	0	0	0	0	0	0	0	0	0	0	0	0	0	0	0	0
**Archaeoglobus**	1	14	0	0	0	0	0	0	0	0	0	0	0	1	0	0	0	0	0	0
**Arcobacter**	2	101	0	0	0	0	0	0	0	0	0	0	0	1	0	0	0	0	0	0
**Aromatoleum**	3	133	1	1	1	1	0	1	0	0	0	0	0	0	1	0	0	1	1	1
**Arsenophonus**	1	4	0	0	0	0	0	0	0	0	0	0	0	0	0	0	0	0	0	0
**Arthrobacter**	2	206	0	0	0	0	0	0	1	0	1	0	0	0	0	0	0	0	0	0
**Arthrospira**	2	67	0	0	0	0	0	0	0	0	0	0	0	0	0	0	0	0	0	0
**Aster**	1	10	0	0	0	0	0	0	0	0	0	0	0	0	0	0	0	0	0	0
**Asticcacaulis**	2	103	0	0	0	0	1	0	0	0	0	0	0	0	0	0	0	0	0	0
**Atopobium**	1	28	0	0	0	0	0	0	0	0	0	0	0	0	0	0	0	0	0	0
**Aurantimonas**	1	2	0	0	0	0	0	0	0	0	0	0	0	0	0	0	0	0	0	0
**Avibacterium**	1	17	0	0	0	0	0	0	0	0	0	0	0	0	0	0	0	0	0	0
**Azoarcus**	3	172	0	1	0	0	0	0	0	0	0	0	0	1	1	0	0	0	0	0
**Azorhizobium**	3	149	0	0	0	0	0	0	0	0	0	0	0	1	0	0	0	0	0	0
**Azorhizophilus**	1	4	0	0	0	0	0	0	0	0	0	0	0	0	0	0	0	0	0	0
**Azospira**	1	19	0	0	0	0	0	0	0	0	0	0	0	1	0	0	0	0	0	0
**Azospirillum**	2	198	0	0	0	0	0	0	1	0	1	0	0	1	1	0	0	0	0	0
**Azotobacter**	3	183	0	0	0	0	0	0	1	0	1	0	0	1	0	0	0	0	0	0
**Bacillales**	1	9	0	0	0	0	0	0	0	0	0	0	0	0	0	0	0	0	0	0
**Bacilli**	1	3	0	0	0	0	0	0	0	0	0	0	0	0	0	0	0	0	0	0
**Bacillus**	2	237	0	0	0	0	0	0	1	0	0	1	0	1	0	0	0	0	0	0
**Bacteria**	2	86	0	0	0	0	0	0	0	0	0	0	0	1	1	0	0	0	0	0
**Bacteriovorax**	2	98	0	0	0	0	0	0	0	0	0	0	0	0	0	0	0	0	0	0
**Bacteroidales**	2	59	0	0	0	0	0	0	0	0	0	0	0	1	0	0	0	0	0	0
**Bacteroides**	2	96	0	0	0	0	0	0	0	0	0	0	0	1	0	0	0	0	0	0
**Bacteroidetes**	1	2	0	0	0	0	0	0	0	0	0	0	0	0	0	0	0	0	0	0
**Bartonella**	2	50	0	0	0	0	0	0	0	0	0	0	0	0	0	0	0	0	0	0
**Baumannia**	2	24	0	0	0	0	0	0	0	0	0	0	0	0	0	0	0	0	0	0
**Bdellovibrio**	2	87	0	0	0	0	0	0	0	0	0	0	0	0	0	0	0	0	0	0
**Beijerinckia**	3	123	0	0	0	0	0	0	0	0	0	0	0	1	0	0	0	0	0	0
**Belliella**	1	14	0	0	0	0	0	0	0	0	0	0	0	0	0	0	0	0	0	0
**Beutenbergia**	2	82	0	0	0	0	0	0	0	0	0	0	0	0	0	0	0	0	0	0
**Bifidobacterium**	2	61	0	0	0	0	0	0	0	0	0	0	0	0	0	0	0	0	0	0

Genus	Cluster Number	Protein Counts	K04112	K04116	K04117	K04118	K05296	K05394	K05549	K05599	K05783	K05898	K05913	K06281	K06446	K06912	K07408	K07535	K07536	K07537
Bizionia	1	1	0	0	0	0	0	0	0	0	0	0	0	0	0	0	0	0	0	0
Blastococcus	1	39	0	0	0	0	0	0	0	0	0	0	0	1	1	0	0	0	0	0
Blattabacterium	1	23	0	0	0	0	0	0	0	0	0	0	0	0	0	0	0	0	0	0
Blautia	1	22	0	0	0	0	0	0	0	0	0	0	0	0	0	0	0	0	0	0
Bordetella	3	227	0	0	0	0	0	0	1	0	1	1	0	0	1	1	0	1	0	0
Borrelia	1	32	0	0	0	0	0	0	0	0	0	0	0	0	0	0	0	0	0	0
Brachybacterium	1	18	0	0	1	0	0	0	0	0	0	0	0	0	0	0	0	1	0	0
Brachymonas	1	4	0	0	0	0	0	0	0	0	0	0	0	0	0	0	0	0	0	0
Brachyspira	2	79	0	0	0	0	0	0	0	0	0	0	0	0	0	0	0	0	0	0
Bradyrhizobiaceae	1	20	0	0	0	0	0	0	1	0	0	0	0	0	0	0	0	0	0	0
Bradyrhizobium	3	254	0	0	0	0	0	0	0	0	0	0	0	1	0	1	0	0	1	0
Brenneria	1	20	0	0	0	0	0	0	0	0	0	0	0	0	0	0	0	0	0	0
Brevibacillus	2	122	0	0	0	0	0	0	0	0	0	0	0	0	0	0	0	0	0	0
Brevibacterium	1	4	0	0	0	0	0	0	0	0	0	0	0	0	0	0	0	0	0	0
Brevundimonas	2	105	0	0	0	0	0	0	0	0	0	0	0	0	0	0	0	0	0	0
Brucella	3	138	0	0	0	0	0	0	0	0	0	0	0	0	1	0	0	0	0	0
Buchnera	1	26	0	0	0	0	0	0	0	0	0	0	0	0	0	0	0	0	0	0
Burkholderia	3	424	0	0	0	0	1	1	1	1	1	1	1	1	1	1	0	1	1	0
Burkholderiaceae	3	143	0	0	0	0	1	0	1	0	0	0	0	0	1	0	0	0	1	0
Burkholderiales	1	24	0	0	0	0	0	0	0	0	0	0	0	0	0	0	0	0	0	0
Buttiauxella	1	2	0	0	0	0	0	0	0	0	0	0	0	0	0	0	0	0	0	0
Butyrivibrio	2	67	0	0	0	0	0	0	0	0	0	0	0	0	0	0	0	0	0	0
Caldanaerobacter	2	63	0	0	0	0	0	0	0	0	0	0	0	0	0	0	0	0	0	0
Caldicellulosiruptor	2	75	0	0	0	0	0	0	0	0	0	0	0	0	0	0	0	0	0	0
Caldilinea	1	23	0	0	0	0	0	0	0	0	0	0	0	0	0	0	0	0	0	0
Caldimonas	1	6	0	0	0	0	0	0	0	0	0	0	0	0	0	0	0	0	0	0
Caldisericum	1	10	0	0	0	0	0	0	0	0	0	0	0	0	0	0	0	0	0	0
Caldisphaera	1	7	0	0	0	0	0	0	0	0	0	0	0	0	0	0	0	0	0	0
Calditerrivibrio	2	74	0	0	0	0	0	0	0	0	0	0	0	1	0	0	0	0	0	0
Caldivirga	1	13	0	0	0	0	0	0	0	0	0	0	0	0	0	0	0	0	0	0
Calothrix	1	43	0	0	0	0	0	0	0	0	0	0	0	1	0	0	0	0	0	0
Calyptogena	2	34	0	0	0	0	0	0	0	0	0	0	0	0	0	0	0	0	0	0
Campylobacter	2	80	0	0	0	0	0	0	0	0	0	0	0	0	0	0	0	0	0	0
Candidatus	2	290	0	0	0	0	0	0	0	0	0	0	0	1	1	0	0	0	0	1
Capnocytophaga	1	50	0	0	0	0	0	0	0	0	0	0	0	0	0	0	0	0	0	0
Carbophilus	1	2	0	0	0	0	0	0	0	0	0	0	0	0	0	0	0	0	0	0
Carboxydibrachium	1	14	0	0	0	0	0	0	0	0	0	0	0	0	0	0	0	0	0	0
Carboxydothermus	2	68	0	0	0	0	0	0	0	0	0	0	0	0	1	0	0	0	0	0
Carica	1	2	0	0	0	0	0	0	0	0	0	0	0	0	0	0	0	0	0	0
Carnobacterium	2	71	0	0	0	0	0	0	0	0	0	0	0	0	0	0	0	0	0	0
Catenibacterium	1	2	0	0	0	0	0	0	0	0	0	0	0	0	0	0	0	0	0	0
Catenovulum	1	2	0	0	0	0	0	0	0	0	0	0	0	0	0	0	0	0	0	0
Catenulispora	3	159	0	0	0	0	0	0	0	0	0	1	0	0	0	0	0	0	0	0
Caulobacter	3	168	0	0	0	0	0	0	0	0	0	0	0	0	1	0	0	0	0	0
Cedecea	1	45	0	0	0	0	0	0	0	0	0	0	0	0	0	0	0	0	0	0
Cellulomonas	2	96	0	0	0	0	0	0	0	0	0	0	0	0	0	0	0	0	0	0
Cellulophaga	2	105	0	0	0	0	0	0	0	0	0	0	0	0	0	0	0	0	0	0
Cellulosilyticum	1	14	0	0	0	0	0	0	0	0	0	0	0	0	0	0	0	0	0	0
Cellvibrio	2	85	0	0	0	0	0	0	0	0	0	0	0	0	0	0	0	0	0	0
Cenarchaeum	1	7	0	0	0	0	0	0	0	0	0	0	0	0	0	0	0	0	0	0
Chamaesiphon	1	13	0	0	0	0	0	0	0	0	0	0	0	0	0	0	0	0	0	0
Chelativorans	3	136	0	0	0	0	0	0	0	0	0	0	0	0	0	0	0	0	0	0

Genus	Cluster Number	Protein Counts	K04112	K04116	K04117	K04118	K05296	K05394	K05549	K05599	K05783	K05898	K05913	K06281	K06446	K06912	K07408	K07535	K07536	K07537
Chelatococcus	1	2	0	0	0	0	0	0	0	0	0	0	0	0	0	0	0	0	0	0
Chitinophaga	2	98	0	0	0	0	0	0	0	0	0	0	0	0	0	0	0	0	0	0
Chlamydia	1	36	0	0	0	0	0	0	0	0	0	0	0	0	0	0	0	0	0	0
Chlamydomonas	1	3	0	0	0	0	0	0	0	0	0	0	0	0	0	0	0	0	0	0
Chlamydophila	1	31	0	0	0	0	0	0	0	0	0	0	0	0	0	0	0	0	0	0
Chlorobaculum	2	61	0	0	0	0	0	0	0	0	0	0	0	1	0	0	0	0	0	0
Chlorobium	2	89	0	0	0	0	0	0	0	0	0	0	0	1	0	0	0	0	0	0
Chlorobium/Pelodictyon	1	2	0	0	0	0	0	0	0	0	0	0	0	0	0	0	0	0	0	0
Chloroflexus	2	105	0	0	0	0	0	0	0	0	0	1	0	1	0	0	0	0	0	0
Chlorogloeopsis	1	9	0	0	0	0	0	0	0	0	0	0	0	0	0	0	0	0	0	0
Chloroherpeton	2	59	0	0	0	0	0	0	0	0	0	0	0	1	0	0	0	0	0	0
Chromobacterium	2	117	0	0	0	0	0	1	0	0	0	0	0	0	0	0	0	0	0	0
Chromohalobacter	2	145	0	0	0	0	0	0	0	0	0	0	0	0	0	0	0	0	0	0
Chroococcidiopsis	1	24	0	0	0	0	0	0	0	0	0	0	0	0	0	0	0	0	0	0
Chryseobacterium	2	104	0	0	0	0	0	0	0	0	0	0	0	0	1	0	0	0	0	0
Citreicella	1	13	0	0	0	0	0	0	0	0	0	0	0	0	0	0	0	0	0	0
Citrobacter	2	190	0	0	0	0	0	0	0	0	0	0	0	1	0	0	0	0	0	0
Clavibacter	2	83	0	0	0	0	0	0	0	0	0	0	0	0	0	0	0	0	0	0
Clonorchis	1	1	0	0	0	0	0	0	0	0	0	0	0	0	0	0	0	0	0	0
Clostridiales	2	77	0	0	0	0	0	0	0	0	0	0	0	0	0	0	0	0	0	0
Clostridium	2	191	0	0	0	0	0	0	0	0	0	0	0	1	0	0	0	0	0	0
Cohnella	1	2	0	0	0	0	0	0	0	0	0	0	0	0	0	0	0	0	0	0
Coleofasciculus	1	4	0	0	0	0	0	0	0	0	0	0	0	0	0	0	0	0	0	0
Collimonas	1	35	0	0	0	0	0	0	0	0	0	0	0	0	0	0	0	0	0	0
Colwellia	2	130	0	0	0	0	0	0	0	0	0	0	0	0	0	0	0	0	0	0
Comamonas	2	196	0	0	0	0	1	0	0	0	0	1	0	0	1	0	0	0	0	0
Conexibacter	3	117	0	1	0	0	0	0	0	0	0	0	0	1	0	0	0	0	0	0
Coprobacillus	1	2	0	0	0	0	0	0	0	0	0	0	0	0	0	0	0	0	0	0
Coprococcus	2	76	0	0	0	0	0	0	0	0	0	0	0	0	0	0	0	0	0	0
Coprothermobacter	2	45	0	0	0	0	0	0	0	0	0	0	0	0	0	0	0	0	0	0
Coraliomargarita	2	68	0	0	0	0	0	0	0	0	0	0	0	0	0	0	0	0	0	0
Corallococcus	1	29	0	1	0	0	0	0	0	0	0	0	0	0	0	0	0	0	0	0
Coriobacterium	2	36	0	0	0	0	0	0	0	0	0	0	0	0	0	0	0	0	0	0
Corynebacterineae	1	5	0	0	0	0	0	0	0	0	0	0	0	0	0	0	0	0	0	0
Corynebacterium	2	175	0	0	1	0	0	0	1	0	1	1	0	1	0	0	0	1	0	0
Coxiella	2	54	0	0	0	0	0	0	0	0	0	0	0	0	0	0	0	0	0	0
Crinalium	1	21	0	0	0	0	0	0	0	0	0	0	0	0	0	0	0	0	0	0
Croceibacter	2	80	0	0	0	0	0	0	0	0	0	0	0	0	0	0	0	0	0	0
Crocosphaera	1	6	0	0	0	0	0	0	0	0	0	0	0	1	0	0	0	0	0	0
Cronobacter	2	120	0	0	0	0	0	0	0	0	0	0	0	0	0	0	0	0	0	0
Cryptobacterium	2	39	0	0	0	0	0	0	0	0	0	0	0	1	1	0	0	0	0	0
Cucumis	1	9	0	0	0	0	0	0	0	0	0	0	0	0	0	0	0	0	0	0
Cupriavidus	3	317	0	1	1	1	1	0	1	0	1	1	0	1	1	1	0	1	1	0
Curtobacterium	1	5	0	0	0	0	0	0	0	0	0	0	0	0	0	0	0	0	0	0
Curvibacter	1	27	0	0	0	0	0	0	0	0	0	0	0	1	0	0	0	0	1	0
Cyanobacterium	1	9	0	0	0	0	0	0	0	0	0	0	0	0	0	0	0	0	0	0
Cyanobium	1	10	0	0	0	0	0	0	0	0	0	0	0	0	0	0	0	0	0	0
Cyanothece	1	34	0	0	0	0	0	0	0	0	0	0	0	1	0	0	0	0	0	0
Cyclobacterium	1	19	0	0	0	0	0	0	0	0	0	0	0	0	0	0	0	0	0	0
Cycloclasticus	1	21	0	0	0	0	0	0	1	0	1	0	0	0	0	0	0	0	0	0
Cylindrospermopsis	1	4	0	0	0	0	0	0	0	0	0	0	0	0	0	0	0	0	0	0
Cylindrospermum	1	30	0	0	0	0	0	0	0	0	0	0	0	0	0	0	0	0	0	0

Genus	Cluster Number	Protein Counts	K04112	K04116	K04117	K04118	K05296	K05394	K05549	K05599	K05783	K05898	K05913	K06281	K06446	K06912	K07408	K07535	K07536	K07537
Cytophaga	2	69	0	0	0	0	0	0	0	0	0	0	0	0	0	0	0	0	0	0
Dactylococcopsis	1	8	0	0	0	0	0	0	0	0	0	0	0	0	0	0	0	0	0	0
Dechloromonas	1	40	0	0	0	0	0	1	1	0	1	0	0	1	0	0	0	0	0	0
Dechlorosoma	1	19	0	0	0	0	0	0	0	0	0	0	0	1	0	0	0	0	0	0
Deferribacter	2	73	0	0	0	0	0	0	0	0	0	0	0	1	0	0	0	0	0	0
Dehalobacter	1	9	0	0	0	0	0	0	0	0	0	0	0	1	0	0	0	0	0	0
Dehalococcoides	2	41	0	0	0	0	0	0	0	0	0	0	0	1	0	0	0	0	0	0
Dehalogenimonas	1	37	0	0	0	0	0	0	0	0	0	0	0	1	0	0	0	0	0	0
Deinococcus	2	140	0	0	0	0	0	0	0	0	0	0	0	0	1	0	0	0	0	0
Delftia	3	177	0	0	0	0	0	0	0	0	0	0	0	0	1	0	0	0	0	0
Denitrovibrio	2	67	0	0	0	0	0	0	0	0	0	0	0	1	0	0	0	0	0	0
Desulfarculus	2	92	0	0	0	0	0	0	0	0	0	0	0	1	1	0	0	0	0	0
Desulfatibacillum	3	84	0	0	0	0	0	0	0	0	0	0	0	1	0	0	0	0	0	0
Desulfitobacterium	2	102	0	0	0	0	0	0	0	0	0	0	0	1	0	0	0	0	0	0
Desulfobacca	2	65	0	0	0	0	0	0	0	0	0	0	0	0	0	0	0	0	0	0
Desulfobacterium	2	113	0	0	0	0	0	0	0	0	0	0	0	1	0	0	0	0	0	0
Desulfobacula	1	21	0	0	0	0	0	0	0	0	0	0	0	0	0	0	0	0	0	1
Desulfobulbus	2	88	0	0	0	0	0	0	0	0	0	0	0	1	0	0	0	0	0	0
Desulfocapsa	1	9	0	0	0	0	0	0	0	0	0	0	0	1	0	0	0	0	0	0
Desulfococcus	2	80	0	0	0	0	0	0	0	0	0	0	0	0	0	0	0	0	0	0
Desulfohalobium	2	52	0	0	0	0	0	0	0	0	0	0	0	1	0	0	0	0	0	0
Desulfomicrobium	2	65	0	0	0	0	0	0	0	0	0	0	0	1	0	0	0	0	0	0
Desulfomonile	1	25	0	0	0	0	0	0	0	0	0	0	0	1	0	0	0	0	0	1
Desulforhabdus	1	2	0	0	0	0	0	0	0	0	0	0	0	0	0	0	0	0	0	0
Desulfosporosinus	1	30	0	0	0	0	0	0	0	0	0	0	0	1	1	0	0	0	0	0
Desulfotalea	2	66	0	0	0	0	0	0	0	0	0	0	0	1	0	0	0	0	0	0
Desulfotomaculum	2	98	0	0	0	0	0	0	0	0	0	0	0	1	1	0	0	0	0	0
Desulfovibrio	2	144	0	0	0	0	0	0	0	0	0	0	0	1	0	0	0	0	0	0
Desulfurispirillum	2	78	0	0	0	0	0	0	0	0	0	0	0	1	0	0	0	0	0	0
Desulfurivibrio	2	71	0	0	0	0	0	0	0	0	0	0	0	1	0	0	0	0	0	0
Desulfurobacterium	2	53	0	0	0	0	0	0	0	0	0	0	0	1	0	0	0	0	0	0
Desulfurococcus	1	11	0	0	0	0	0	0	0	0	0	0	0	0	0	0	0	0	0	0
Diaphorobacter	1	16	0	0	0	0	0	0	0	0	0	0	0	0	0	0	0	0	0	0
Dichelobacter	2	45	0	0	0	0	0	0	0	0	0	0	0	0	0	0	0	0	0	0
Dickeya	2	155	0	0	0	0	0	0	0	0	0	0	0	1	0	0	0	0	0	0
Dictyoglomus	2	62	0	0	0	0	0	0	0	0	0	0	0	0	0	0	0	0	0	0
Dinoroseobacter	3	148	0	0	0	0	0	0	0	0	0	0	0	0	0	0	0	0	0	0
Dokdonia	1	3	0	0	0	0	0	0	0	0	0	0	0	0	0	0	0	0	0	0
Dorea	1	7	0	0	0	0	0	0	0	0	0	0	0	0	0	0	0	0	0	0
Dyadobacter	2	83	0	0	0	0	0	0	0	0	0	0	0	0	0	0	0	0	0	0
Echinicola	1	20	0	0	0	0	0	0	0	0	0	0	0	0	0	0	0	0	0	0
Edwardsiella	2	126	0	0	0	0	0	0	0	0	0	0	0	1	0	0	0	0	0	0
Eggerthella	1	51	0	0	0	0	0	0	0	0	0	0	0	1	1	0	0	0	0	0
Ehrlichia	2	37	0	0	0	0	0	0	0	0	0	0	0	0	0	0	0	0	0	0
Eikenella	1	4	0	0	0	0	0	0	0	0	0	0	0	0	0	0	0	0	0	0
Elusimicrobium	2	44	0	0	0	0	0	0	0	0	0	0	0	0	0	0	0	0	0	0
Emticicia	1	21	0	0	0	0	0	0	0	0	0	0	0	1	0	0	0	0	0	0
Enhydrobacter	1	1	0	0	0	0	0	0	0	0	0	0	0	0	0	0	0	0	0	0
Ensifer	1	2	0	0	0	0	0	0	0	0	0	0	0	0	0	0	0	0	0	0
Enterobacter	2	202	0	0	0	0	0	0	1	0	1	0	0	1	0	0	0	0	0	0
Enterobacteriaceae	2	211	0	0	0	0	0	0	1	0	1	0	0	1	0	0	0	0	0	0
Enterococcus	1	187	0	0	0	0	0	0	0	0	1	0	0	0	0	0	0	0	0	0

Genus	Cluster Number	Protein Counts	K04112	K04116	K04117	K04118	K05296	K05394	K05549	K05599	K05783	K05898	K05913	K06281	K06446	K06912	K07408	K07535	K07536	K07537
Enterovibrio	1	15	0	0	0	0	0	0	0	0	0	0	0	0	0	0	0	0	0	0
Erwinia	2	151	0	0	0	0	0	0	0	0	0	0	0	0	0	0	0	0	0	0
Erysipelothrix	1	9	0	0	0	0	0	0	0	0	0	0	0	0	0	0	0	0	0	0
Erysipelotrichaceae	1	4	0	0	0	0	0	0	0	0	0	0	0	0	0	0	0	0	0	0
Erythrobacter	2	80	0	0	0	0	0	0	0	0	0	0	0	0	1	0	0	0	0	0
Escherichia	2	242	0	0	0	0	0	0	1	0	1	0	0	1	0	0	0	0	0	0
Ethanoligenens	2	69	0	0	0	0	0	0	0	0	0	0	0	0	0	0	0	0	0	0
Eubacteriaceae	1	2	0	0	0	0	0	0	0	0	0	0	0	0	0	0	0	0	0	0
Eubacterium	2	103	0	0	0	0	0	0	0	0	0	0	0	0	0	0	0	0	0	0
Eudoraea	1	4	0	0	0	0	0	0	0	0	0	0	0	0	0	0	0	0	0	0
Euryarchaeota	1	1	0	0	0	0	0	0	0	0	0	0	0	0	0	0	0	0	0	0
Exiguobacterium	2	93	0	0	0	0	0	0	0	0	0	0	0	0	0	0	0	0	0	0
Faecalibacterium	1	12	0	0	0	0	0	0	0	0	0	0	0	0	0	0	0	0	0	0
Ferrimonas	2	115	0	0	0	0	0	0	0	0	0	0	0	0	0	0	0	0	0	0
Ferroglobus	1	14	1	0	0	0	0	0	0	0	0	0	0	1	0	0	0	1	0	0
Ferroplasma	1	11	0	0	0	0	0	0	0	0	0	0	0	0	0	0	0	0	0	0
Fervidicoccus	1	7	0	0	0	0	0	0	0	0	0	0	0	0	0	0	0	0	0	0
Fervidobacterium	2	54	0	0	0	0	0	0	0	0	0	0	0	0	0	0	0	0	0	0
Fibrella	1	17	0	0	0	0	0	0	0	0	0	0	0	0	0	0	0	0	0	0
Fibrobacter	2	52	0	0	0	0	0	0	0	0	0	0	0	0	0	0	0	0	0	0
Filifactor	1	9	0	0	0	0	0	0	0	0	0	0	0	0	0	0	0	0	0	0
Finegoldia	2	55	0	0	0	0	0	0	0	0	0	0	0	0	0	0	0	0	0	0
Firmicutes	1	15	0	0	0	0	0	0	0	0	0	0	0	0	0	0	0	0	0	0
Fischerella	1	15	0	0	0	0	0	0	0	0	0	0	0	0	0	0	0	0	0	0
Flavobacteria	1	7	0	0	0	0	0	0	0	0	0	0	0	0	0	0	0	0	0	0
Flavobacteriaceae	2	69	0	0	0	0	0	0	0	0	0	0	0	0	0	0	0	0	0	0
Flavobacteriales	1	3	0	0	0	0	0	0	0	0	0	0	0	0	0	0	0	0	0	0
Flavobacterium	2	103	0	0	0	0	0	0	0	0	0	0	0	1	0	0	0	0	0	0
Flexibacter	1	17	0	0	0	0	0	0	0	0	0	0	0	0	0	0	0	0	0	0
Flexistipes	1	11	0	0	0	0	0	0	0	0	0	0	0	1	0	0	0	0	0	0
Fluoribacter	1	6	0	0	0	0	0	0	0	0	0	0	0	0	0	0	0	0	0	0
Fluviicola	2	69	0	0	0	0	0	0	0	0	0	0	0	0	0	0	0	0	0	0
Formosa	1	2	0	0	0	0	0	0	0	0	0	0	0	0	0	0	0	0	0	0
Francisella	2	88	0	0	0	0	0	0	0	0	0	0	0	0	0	0	0	0	0	0
Frankia	3	202	0	1	0	0	0	0	1	0	0	0	0	1	1	0	0	1	0	0
Frateuria	1	25	0	0	0	0	0	0	0	0	0	0	0	0	0	0	0	0	0	0
Fructobacillus	1	2	0	0	0	0	0	0	0	0	0	0	0	0	0	0	0	0	0	0
Fulvimarina	1	2	0	0	0	0	0	0	0	0	0	0	0	0	0	0	0	0	0	0
Fusobacterium	1	48	0	0	0	0	0	0	0	0	0	0	0	0	0	0	0	0	0	0
Gallibacterium	2	87	0	0	0	0	0	0	0	0	0	0	0	0	0	0	0	0	0	0
Gallionella	2	67	0	0	0	0	0	0	0	0	0	0	0	0	0	0	0	0	0	0
Gammaproteobacteria	1	9	0	0	0	0	0	0	0	0	0	0	0	0	0	0	0	0	0	0
Gardnerella	1	31	0	0	0	0	0	0	0	0	0	0	0	0	0	0	0	0	0	0
Geitlerinema	1	9	0	0	0	0	0	0	0	0	0	0	0	0	0	0	0	0	0	0
Gemmatimonas	2	93	0	0	0	0	0	0	0	0	0	0	0	1	0	0	0	0	0	0
Geobacillus	2	168	0	1	0	0	0	0	0	0	0	0	0	1	1	0	0	0	0	0
Geobacter	2	150	1	0	0	0	1	0	0	0	0	0	0	1	0	0	0	0	0	1
Geodermatophilus	2	136	0	0	0	0	0	0	1	0	0	0	0	0	1	0	0	1	0	0
Geopsychrobacter	1	2	0	0	0	0	0	0	0	0	0	0	0	0	0	0	0	0	0	0
Gillisia	1	11	0	0	0	0	0	0	0	0	0	0	0	0	0	0	0	0	0	0
Gilvimarinus	1	4	0	0	0	0	0	0	0	0	0	0	0	0	0	0	0	0	0	0
Glaciecola	2	117	0	0	0	0	0	0	0	0	0	0	0	1	1	0	0	0	0	0

Genus	Cluster Number	Protein Counts	K04112	K04116	K04117	K04118	K05296	K05394	K05549	K05599	K05783	K05898	K05913	K06281	K06446	K06912	K07408	K07535	K07536	K07537
**Gloeobacter**	2	67	0	0	0	0	0	0	0	0	0	0	0	0	0	0	0	0	0	0
**Gloeocapsa**	1	20	0	0	0	0	0	0	0	0	0	0	0	0	0	0	0	0	0	0
**Gloeothece**	1	1	0	0	0	0	0	0	0	0	0	0	0	1	0	0	0	0	0	0
**Gluconacetobacter**	2	96	0	0	0	0	0	0	0	0	0	0	0	0	0	0	0	0	0	0
**Gluconobacter**	2	76	0	0	0	0	0	0	0	0	0	0	0	0	0	0	0	0	0	0
**Gordonia**	1	158	0	0	0	0	0	0	1	0	0	1	0	1	1	0	0	1	0	0
**Gordonibacter**	1	32	0	0	0	0	0	0	0	0	0	0	0	1	0	0	0	0	0	0
**Gramella**	2	104	0	0	0	0	0	0	0	0	0	0	0	0	0	0	0	0	0	0
**Granulibacter**	2	90	0	0	0	0	0	0	0	0	0	0	0	0	0	0	0	0	0	0
**Granulicella**	2	98	0	0	0	0	0	0	0	0	0	0	0	1	0	0	0	0	0	0
**Grimontia**	1	9	0	0	0	0	0	0	0	0	0	0	0	0	0	0	0	0	0	0
**Haemophilus**	2	117	0	0	0	0	0	0	0	0	0	0	0	1	0	0	0	0	0	0
**Hafnia**	1	29	0	0	0	0	0	0	0	0	0	0	0	0	0	0	0	0	0	0
**Hahella**	3	130	0	0	0	0	0	0	0	0	0	0	0	0	0	0	0	0	0	0
**Halalkalicoccus**	1	18	0	0	0	0	0	0	0	0	0	0	0	0	0	0	0	0	0	0
**Halanaerobium**	2	89	0	0	0	0	0	0	0	0	0	0	0	0	0	0	0	0	0	0
**Haliangium**	2	108	0	0	0	0	0	0	0	0	0	0	0	0	0	0	0	0	0	0
**Haliscomenobacter**	2	107	0	0	0	0	0	0	0	0	0	0	0	1	0	0	0	0	0	0
**Haloarcula**	1	22	0	0	0	0	0	0	0	0	0	0	0	0	0	0	0	0	0	0
**Halobacillus**	1	19	0	0	0	0	0	0	0	0	0	0	0	0	1	0	0	0	0	0
**Halobacteriaceae**	1	1	0	0	0	0	0	0	0	0	0	0	0	0	0	0	0	0	0	0
**Halobacterium**	1	10	0	0	0	0	0	0	0	0	0	0	0	0	0	0	0	0	0	0
**Halobacteroides**	1	14	0	0	0	0	0	0	0	0	0	0	0	0	0	0	0	0	0	0
**Haloferax**	1	20	0	0	0	0	0	0	0	0	0	0	0	0	0	0	0	0	0	0
**Halogeometricum**	1	12	0	0	0	0	0	0	0	0	0	0	0	0	0	0	0	0	0	0
**Halomicrobium**	1	10	0	0	0	0	0	0	0	0	0	0	0	0	0	0	0	0	0	0
**Halomonas**	1	149	0	0	0	0	0	0	0	0	0	0	0	0	0	0	0	0	0	0
**Halopiger**	1	16	0	0	0	0	0	0	0	0	0	0	0	0	0	0	0	0	0	0
**Haloquadratum**	1	19	0	0	0	0	0	0	0	0	0	0	0	0	0	0	0	0	0	0
**Halorhabdus**	1	10	0	0	0	0	0	0	0	0	0	0	0	0	0	0	0	0	0	0
**Halorhodospira**	2	71	0	0	0	0	0	0	0	0	0	0	0	0	0	0	0	0	0	0
**Halorubrum**	1	16	0	0	0	0	0	0	0	0	0	0	0	0	0	0	0	0	0	0
**Haloterrigena**	1	19	0	0	0	0	0	0	0	0	0	0	0	0	0	0	0	0	0	0
**Halothece**	1	9	0	0	0	0	0	0	0	0	0	0	0	0	0	0	0	0	0	0
**Halothermothrix**	2	50	0	0	0	0	0	0	0	0	0	0	0	1	0	0	0	0	0	0
**Halothiobacillus**	2	65	0	0	0	0	0	0	0	0	0	0	0	0	0	0	0	0	0	0
**Halovivax**	1	12	0	0	0	0	0	0	0	0	0	0	0	0	0	0	0	0	0	0
**Helicobacter**	2	79	0	0	0	0	0	0	0	0	0	0	0	0	0	0	0	0	0	0
**Heliobacillus**	1	4	0	0	0	0	0	0	0	0	0	0	0	0	0	0	0	0	0	0
**Heliobacterium**	2	51	0	0	0	0	0	0	0	0	0	0	0	0	0	0	0	0	0	0
**Herbaspirillum**	2	172	0	0	0	0	0	0	1	0	1	0	0	0	0	0	0	0	0	0
**Herminiimonas**	3	89	0	0	0	0	0	0	0	0	0	0	0	0	0	0	0	0	0	0
**Herpetosiphon**	3	90	0	0	1	0	0	0	0	0	0	0	0	0	0	0	0	0	0	0
**Hippea**	2	44	0	0	0	0	0	0	0	0	0	0	0	0	0	0	0	0	0	0
**Hirschia**	2	102	0	0	0	0	0	0	0	0	0	0	0	0	0	0	0	0	0	0
**Histophilus**	2	72	0	0	0	0	0	0	0	0	0	0	0	0	0	0	0	0	0	0
**Hoeflea**	1	16	0	0	0	0	0	0	0	0	0	0	0	0	0	0	0	0	0	0
**Hydrogenivirga**	1	2	0	0	0	0	0	0	0	0	0	0	0	0	0	0	0	0	0	0
**Hydrogenobacter**	2	55	0	0	0	0	0	0	0	0	0	0	0	1	0	0	0	0	0	0
**Hydrogenobaculum**	2	44	0	0	0	0	0	0	0	0	0	0	0	1	0	0	0	0	0	0
**Hydrogenophaga**	1	9	0	0	0	0	0	0	0	0	0	0	0	0	0	0	0	0	0	0
**Hylemonella**	1	4	0	0	0	0	0	0	0	0	0	0	0	0	0	0	0	0	0	0

Genus	Cluster Number	Protein Counts	K04112	K04116	K04117	K04118	K05296	K05394	K05549	K05599	K05783	K05898	K05913	K06281	K06446	K06912	K07408	K07535	K07536	K07537
Hyperthermus	1	6	0	0	0	0	0	0	0	0	0	0	0	1	0	0	0	0	0	0
Hyphomicrobium	2	93	0	0	0	0	0	0	0	0	0	0	0	1	0	0	0	0	0	0
Hyphomonas	3	105	0	0	0	0	0	0	0	0	0	0	0	0	1	0	0	0	0	0
Ideonella	1	18	0	0	0	0	0	0	0	0	0	0	0	0	0	0	0	0	0	0
Idiomarina	2	86	0	0	0	0	0	0	0	0	0	0	0	0	0	0	0	0	0	0
Ignavibacterium	1	14	0	0	0	0	0	0	0	0	0	0	0	0	0	0	0	0	0	0
Ignicoccus	1	5	0	0	0	0	0	0	0	0	0	0	0	1	0	0	0	0	0	0
Ignisphaera	1	8	0	0	0	0	0	0	0	0	0	0	0	0	0	0	0	0	0	0
Ilyobacter	2	85	0	0	0	0	0	0	0	0	0	0	0	0	0	0	0	0	0	0
Intrasporangium	2	114	0	1	1	0	0	0	0	0	0	0	0	0	0	0	0	1	0	0
Isoptericola	1	18	0	0	0	0	0	0	0	0	0	0	0	0	0	0	0	0	0	0
Isosphaera	2	75	0	0	0	0	0	0	0	0	0	0	0	0	0	0	0	0	0	0
Jannaschia	3	151	0	0	0	0	0	0	0	0	0	0	0	0	0	0	0	0	0	0
Janthinobacterium	2	99	0	0	0	0	0	0	0	0	0	0	0	0	0	0	0	0	0	0
Jonesia	2	56	0	0	0	0	0	0	0	0	0	0	0	0	0	0	0	0	0	0
Joostella	1	3	0	0	0	0	0	0	0	0	0	0	0	0	0	0	0	0	0	0
Kaistia	1	2	0	0	0	0	0	0	0	0	0	0	0	0	0	0	0	0	0	0
Kangiella	2	84	0	0	0	0	0	0	0	0	0	0	0	0	0	0	0	0	0	0
Ketogulonicigenium	2	102	0	0	0	0	0	0	0	0	0	0	0	0	0	0	0	0	0	0
Kineococcus	1	24	0	0	0	0	0	0	0	0	0	0	0	0	0	0	0	0	0	0
Kinetoplastibacterium	1	2	0	0	0	0	0	0	0	0	0	0	0	0	0	0	0	0	0	0
Kingella	1	4	0	0	0	0	0	0	0	0	0	0	0	0	0	0	0	0	0	0
Kitasatospora	1	36	0	0	0	0	0	0	0	0	0	0	0	0	0	0	0	0	0	0
Klebsiella	3	222	0	0	0	0	0	0	1	0	1	0	0	0	0	0	0	0	0	0
Kluyvera	1	1	0	0	0	0	0	0	0	0	0	0	0	0	0	0	0	0	0	0
Kocuria	2	87	0	0	0	0	0	0	0	0	0	0	0	0	0	0	0	0	0	0
Kordia	1	5	0	0	0	0	0	0	0	0	0	0	0	0	0	0	0	0	0	0
Kosmotoga	2	61	0	0	0	0	0	0	0	0	0	0	0	0	0	0	0	0	0	0
Kribbella	2	99	0	0	0	0	0	0	0	0	0	0	0	0	0	0	0	0	0	0
Krokinobacter	2	90	0	0	0	0	0	0	0	0	0	0	0	0	0	0	0	0	0	0
Kyrpidia	2	113	0	0	0	0	0	0	0	0	0	0	0	1	1	0	0	1	0	0
Kytococcus	2	70	0	0	0	0	0	0	0	0	0	0	0	0	0	0	0	0	0	0
Labrenzia	1	4	0	0	0	0	0	0	0	0	0	0	0	0	0	0	0	0	0	0
Lachnoanaerobaculum	1	1	0	0	0	0	0	0	0	0	0	0	0	0	0	0	0	0	0	0
Lachnospiraceae	1	51	0	0	0	0	0	0	0	0	0	0	0	0	0	0	0	0	0	0
Lacinutrix	1	20	0	0	0	0	0	0	0	0	0	0	0	0	0	0	0	0	0	0
Lactobacillus	2	119	0	0	0	0	0	0	0	0	0	0	0	0	0	0	0	0	0	0
Lactococcus	2	82	0	0	0	0	0	0	0	0	0	0	0	0	0	0	0	0	0	0
Lamprocystis	1	2	0	0	0	0	0	0	0	0	0	0	0	0	0	0	0	0	0	0
Laribacter	2	80	0	0	0	0	0	0	0	0	0	0	0	0	1	0	0	0	0	0
Lawsonia	2	33	0	0	0	0	0	0	0	0	0	0	0	1	0	0	0	0	0	0
Leadbetterella	2	79	0	0	0	0	0	0	0	0	0	0	0	0	0	0	0	0	0	0
Leeuwenhoekiella	1	2	0	0	0	0	0	0	0	0	0	0	0	0	0	0	0	0	0	0
Legionella	2	117	0	0	0	0	0	0	0	0	0	0	0	0	0	0	0	0	0	0
Leifsonia	2	48	0	0	0	0	0	0	0	0	0	0	0	0	0	0	0	0	0	0
Leptolyngbya	1	10	0	0	0	0	0	0	0	0	0	0	0	0	0	0	0	0	0	0
Leptospira	2	76	0	0	0	0	0	0	0	0	0	0	0	0	0	0	0	0	0	0
Leptospirillum	1	10	0	0	0	0	0	0	0	0	0	0	0	1	0	0	0	0	0	0
Leptothrix	3	186	0	1	1	0	0	0	0	0	0	0	0	1	1	0	0	1	1	0
Leptotrichia	2	43	0	0	0	0	0	0	0	0	0	0	0	0	0	0	0	0	0	0
Leucobacter	1	2	0	0	0	0	0	0	0	0	0	0	0	0	0	0	0	0	0	0
Leuconostoc	2	64	0	0	0	0	0	0	0	0	0	0	0	0	0	0	0	0	0	0

Genus	Cluster Number	Protein Counts	K04112	K04116	K04117	K04118	K05296	K05394	K05549	K05599	K05783	K05898	K05913	K06281	K06446	K06912	K07408	K07535	K07536	K07537
Liberibacter	1	4	0	0	0	0	0	0	0	0	0	0	0	0	0	0	0	0	0	0
Limnohabitans	1	11	0	0	0	0	0	0	0	0	0	0	0	0	0	0	0	0	0	0
Listeria	2	77	0	0	0	0	0	0	0	0	0	0	0	0	0	0	0	0	0	0
Listeriaceae	1	6	0	0	0	0	0	0	0	0	0	0	0	0	0	0	0	0	0	0
Listonella	1	38	0	0	0	0	0	0	0	0	0	0	0	0	0	0	0	0	0	0
Loktanella	1	3	0	0	0	0	0	0	0	0	0	0	0	0	0	0	0	0	0	0
Lysinibacillus	2	97	0	0	0	0	0	0	0	0	0	1	0	0	0	0	0	0	0	0
Macrococcus	2	60	0	0	0	0	0	0	0	0	0	0	0	0	0	0	0	0	0	0
Magnetococcus	2	64	0	0	0	0	0	0	0	0	0	0	0	1	0	0	0	0	0	0
Magnetospirillum	2	113	1	0	0	0	0	0	0	0	0	0	0	1	1	0	0	1	0	1
Mahella	2	58	0	0	0	0	0	0	0	0	0	0	0	0	0	0	0	0	0	0
Mannheimia	2	85	0	0	0	0	0	0	0	0	0	0	0	1	0	0	0	0	0	0
Maribacter	3	87	0	0	0	0	0	0	0	0	0	0	0	0	0	0	0	0	0	0
Maricaulis	3	87	0	0	0	0	0	0	0	0	0	0	0	0	0	0	0	0	0	0
Marichromatium	1	2	0	0	0	0	0	0	0	0	0	0	0	0	0	0	0	0	0	0
Marinithermus	2	77	0	0	0	0	0	0	0	0	0	0	0	0	0	0	0	0	0	0
Marinitoga	1	15	0	0	0	0	0	0	0	0	0	0	0	0	0	0	0	0	0	0
Marinobacter	2	163	0	1	0	0	0	0	1	0	1	0	0	0	1	0	0	0	1	0
Marinomonas	3	192	0	0	0	0	0	0	1	0	1	0	0	0	0	0	1	0	0	0
Maritimibacter	1	7	0	0	0	0	0	0	0	0	0	0	0	0	0	0	0	0	0	0
Marivirga	2	96	0	0	0	0	0	0	0	0	0	0	0	0	0	0	0	0	0	0
Martelella	1	3	0	0	0	0	0	0	0	0	0	0	0	0	0	0	0	0	0	0
Marvinbryantia	1	1	0	0	0	0	0	0	0	0	0	0	0	0	0	0	0	0	0	0
Massilia	1	4	0	0	0	0	0	0	0	0	0	0	0	0	0	0	0	0	0	0
Mastigocladopsis	1	16	0	0	0	0	0	0	0	0	0	0	0	0	0	0	0	0	0	0
Megamonas	1	47	0	0	0	0	0	0	0	0	0	0	0	1	0	0	0	0	0	0
Megasphaera	1	14	0	0	0	0	0	0	0	0	0	0	0	0	0	0	0	0	0	0
Meiothermus	2	110	0	0	0	0	0	0	0	0	0	0	0	0	0	0	0	0	0	0
Melissococcus	2	46	0	0	0	0	0	0	0	0	0	0	0	0	0	0	0	0	0	0
Mesoflavibacter	1	6	0	0	0	0	0	0	0	0	0	0	0	0	0	0	0	0	0	0
Mesoplasma	1	23	0	0	0	0	0	0	0	0	0	0	0	0	0	0	0	0	0	0
Mesorhizobium	2	201	0	0	0	0	0	0	0	0	0	0	0	1	0	0	0	0	0	0
Mesotoga	1	14	0	0	0	0	0	0	0	0	0	0	0	0	0	0	0	0	0	0
Metallosphaera	1	18	0	0	0	0	0	0	0	0	0	0	0	0	0	0	0	0	0	0
Methanobacterium	1	7	0	0	0	0	0	0	0	0	0	0	0	0	0	0	0	0	0	0
Methanobrevibacter	1	9	0	0	0	0	0	0	0	0	0	0	0	0	0	0	0	0	0	0
Methanocaldococcus	1	6	0	0	0	0	0	0	0	0	0	0	0	0	0	0	0	0	0	0
Methanocella	1	15	0	0	0	0	0	0	0	0	0	0	0	1	0	0	0	0	0	0
Methanococcaceae	1	1	0	0	0	0	0	0	0	0	0	0	0	0	0	0	0	0	0	0
Methanococcoides	1	5	0	0	0	0	0	0	0	0	0	0	0	0	0	0	0	0	0	0
Methanococcus	1	9	0	0	0	0	0	0	0	0	0	0	0	0	0	0	0	0	0	0
Methanocorpusculum	1	7	0	0	0	0	0	0	0	0	0	0	0	0	0	0	0	0	0	0
Methanoculleus	1	8	0	0	0	0	0	0	0	0	0	0	0	0	0	0	0	0	0	0
Methanohalobium	1	5	0	0	0	0	0	0	0	0	0	0	0	0	0	0	0	0	0	0
Methanohalophilus	1	5	0	0	0	0	0	0	0	0	0	0	0	0	0	0	0	0	0	0
Methanolobus	1	5	0	0	0	0	0	0	0	0	0	0	0	0	0	0	0	0	0	0
Methanomethylovorans	1	5	0	0	0	0	0	0	0	0	0	0	0	0	0	0	0	0	0	0
Methanoplanus	1	7	0	0	0	0	0	0	0	0	0	0	0	0	0	0	0	0	0	0
Methanopyrus	1	4	0	0	0	0	0	0	0	0	0	0	0	0	0	0	0	0	0	0
Methanoregula	1	8	0	0	0	0	0	0	0	0	0	0	0	0	0	0	0	0	0	0
Methanosaeta	1	9	0	0	0	0	0	0	0	0	0	0	0	0	0	0	0	0	0	0
Methanosalsum	1	7	0	0	0	0	0	0	0	0	0	0	0	1	0	0	0	0	0	0

Genus	Cluster Number	Protein Counts	K04112	K04116	K04117	K04118	K05296	K05394	K05549	K05599	K05783	K05898	K05913	K06281	K06446	K06912	K07408	K07535	K07536	K07537
**Methanosarcina**	1	18	0	0	0	0	0	0	0	0	0	0	0	0	0	0	0	0	0	0
**Methanosphaera**	1	7	0	0	0	0	0	0	0	0	0	0	0	0	0	0	0	0	0	0
**Methanosphaerula**	1	8	0	0	0	0	0	0	0	0	0	0	0	0	0	0	0	0	0	0
**Methanospirillum**	1	6	0	0	0	0	0	0	0	0	0	0	0	0	0	0	0	0	0	0
**Methanothermobacter**	1	6	0	0	0	0	0	0	0	0	0	0	0	0	0	0	0	0	0	0
**Methanothermococcus**	1	5	0	0	0	0	0	0	0	0	0	0	0	0	0	0	0	0	0	0
**Methanothermus**	1	5	0	0	0	0	0	0	0	0	0	0	0	0	0	0	0	0	0	0
**Methanotorris**	1	5	0	0	0	0	0	0	0	0	0	0	0	0	0	0	0	0	0	0
**Methylacidiphilum**	2	48	0	0	0	0	0	0	0	0	0	0	0	1	0	0	0	0	0	0
**Methylarcula**	1	2	0	0	0	0	0	0	0	0	0	0	0	0	0	0	0	0	0	0
**Methylibium**	1	38	0	0	0	0	0	0	0	0	0	0	0	1	0	0	0	0	0	0
**Methylobacillus**	2	61	0	0	0	0	0	1	0	0	0	0	0	0	0	0	0	0	0	0
**Methylobacterium**	3	236	0	0	0	0	0	0	1	0	1	0	0	0	0	0	0	0	0	0
**Methylocella**	3	117	0	0	0	0	0	0	0	0	0	0	0	1	0	0	0	0	0	0
**Methylococcus**	2	80	0	0	0	0	0	0	0	0	0	0	0	1	0	0	0	0	0	0
**Methylocystis**	1	21	0	0	0	0	0	0	0	0	0	0	0	1	0	0	0	0	0	0
**Methyloferula**	1	2	0	0	0	0	0	0	0	0	0	0	0	0	0	0	0	0	0	0
**Methylomicrobium**	1	12	0	0	0	0	0	0	0	0	0	0	0	1	0	0	0	0	0	0
**Methylomonas**	1	15	0	0	0	0	0	0	0	0	0	0	0	1	0	0	0	0	0	0
**Methylophaga**	1	7	0	0	0	0	0	0	0	0	0	0	0	0	0	0	0	0	0	0
**Methylophilus**	1	2	0	0	0	0	0	0	0	0	0	0	0	0	0	0	0	0	0	0
**Methylosinus**	1	2	0	0	0	0	0	0	0	0	0	0	0	0	0	0	0	0	0	0
**Methylotenera**	2	72	0	0	0	0	0	0	0	0	0	0	0	0	0	0	0	0	0	0
**Methyloversatilis**	1	5	0	0	0	0	0	0	0	0	0	0	0	1	0	0	0	0	0	0
**Methylovorus**	2	66	0	0	0	0	0	0	0	0	0	0	0	0	0	0	0	0	0	0
**Micavibrio**	1	9	0	0	0	0	0	0	0	0	0	0	0	0	0	0	0	0	0	0
**Microbacterium**	2	115	0	0	0	0	0	0	0	0	0	0	0	0	0	0	0	0	0	0
**Microchaete**	1	23	0	0	0	0	0	0	0	0	0	0	0	1	0	0	0	0	0	0
**Micrococcus**	2	72	0	0	0	0	0	0	0	0	0	0	0	0	0	0	0	0	0	0
**Microcoleus**	1	17	0	0	0	0	0	0	0	0	0	0	0	0	0	0	0	0	0	0
**Microcystis**	2	67	0	0	0	0	0	0	0	0	0	0	0	0	0	0	0	0	0	0
**Microlunatus**	1	25	0	0	0	0	0	0	0	0	0	0	0	0	0	0	0	0	0	0
**Micromonospora**	2	115	0	0	0	0	0	0	0	0	0	0	0	0	0	0	0	0	0	0
**Microvirga**	1	6	0	0	0	0	0	0	0	0	0	0	0	0	0	0	0	0	0	0
**Mobiluncus**	1	41	0	0	0	0	0	0	0	0	0	0	0	1	0	0	0	0	0	0
**Modestobacter**	1	43	0	0	0	0	0	0	0	0	0	0	0	0	1	0	0	0	0	0
**Moorea**	1	2	0	0	0	0	0	0	0	0	0	0	0	0	0	0	0	0	0	0
**Moorella**	2	69	0	0	0	0	0	0	0	0	0	0	0	0	0	0	0	1	0	0
**Moraxella**	2	66	0	0	0	0	0	0	0	0	0	0	0	0	0	0	0	0	0	0
**Morganella**	1	29	0	0	0	0	0	0	0	0	0	0	0	1	0	0	0	0	0	0
**Moritella**	1	2	0	0	0	0	0	0	0	0	0	0	0	0	0	0	0	0	0	0
**Muricauda**	1	23	0	0	0	0	0	0	0	0	0	0	0	0	0	0	0	0	0	0
**Mycobacterium**	3	306	0	1	0	0	0	0	1	0	0	1	0	1	1	1	0	1	0	0
**Mycoplasma**	1	53	0	0	0	0	0	0	0	0	0	0	0	0	0	0	0	0	0	0
**Myxococcus**	2	110	0	1	0	0	0	0	0	0	0	1	0	0	0	0	0	0	0	0
**Nakamurella**	2	115	0	1	1	0	0	0	0	0	0	0	0	0	0	0	0	1	0	0
**napthalene**	1	2	0	0	0	0	0	0	0	0	0	0	0	0	0	0	0	0	0	0
**Natranaerobius**	2	55	0	0	0	0	0	0	0	0	0	0	0	0	0	0	0	0	0	0
**Natrialba**	1	16	0	0	0	0	0	0	0	0	0	0	0	0	0	0	0	0	0	0
**Natrinema**	1	16	0	0	0	0	0	0	0	0	0	0	0	0	0	0	0	0	0	0
**Natronobacterium**	1	11	0	0	0	0	0	0	0	0	0	0	0	1	0	0	0	0	0	0
**Natronococcus**	1	21	0	0	0	0	0	0	0	0	0	0	0	0	0	0	0	0	0	0

Genus	Cluster Number	Protein Counts	K04112	K04116	K04117	K04118	K05296	K05394	K05549	K05599	K05783	K05898	K05913	K06281	K06446	K06912	K07408	K07535	K07536	K07537
Natronomonas	1	20	0	0	0	0	0	0	0	0	0	0	0	0	0	0	0	0	0	0
Nautilia	2	48	0	0	0	0	0	0	0	0	0	0	0	0	0	0	0	0	0	0
Neisseria	2	67	0	0	0	0	0	0	0	0	0	0	0	0	0	0	0	0	0	0
Neisseriaceae	1	1	0	0	0	0	0	0	0	0	0	0	0	0	0	0	0	0	0	0
Neorickettsia	1	31	0	0	0	0	0	0	0	0	0	0	0	0	0	0	0	0	0	0
Neptuniibacter	1	2	0	0	0	0	0	0	0	0	0	0	0	0	0	0	0	0	0	0
Niastella	1	21	0	0	0	0	0	0	0	0	0	0	0	1	0	0	0	0	0	0
Nitratifractor	2	54	0	0	0	0	0	0	0	0	0	0	0	0	0	0	0	0	0	0
Nitratireductor	1	19	0	0	0	0	0	0	0	0	0	0	0	0	0	0	0	0	0	0
Nitratiruptor	2	40	0	0	0	0	0	0	0	0	0	0	0	0	0	0	0	0	0	0
Nitrobacter	2	99	0	0	0	0	0	0	0	0	0	0	0	0	0	0	0	0	0	0
Nitrococcus	1	2	0	0	0	0	0	0	0	0	0	0	0	0	0	0	0	0	0	0
Nitrosococcus	2	94	0	0	0	0	0	0	0	0	0	0	0	0	0	0	0	0	0	0
Nitrosomonas	2	72	0	0	0	0	0	0	0	0	0	0	0	0	0	0	0	0	0	0
Nitrosopumilaceae	1	7	0	0	0	0	0	0	0	0	0	0	0	0	0	0	0	0	0	0
Nitrosopumilus	1	11	0	0	0	0	0	0	0	0	0	0	0	0	0	0	0	0	0	0
Nitrosospira	2	74	0	0	0	0	0	0	0	0	0	0	0	0	0	0	0	0	0	0
Nocardia	2	185	0	0	0	0	0	0	0	0	0	1	0	0	0	0	0	0	0	0
Nocardioidaceae	1	2	0	0	0	0	0	0	0	0	0	0	0	0	0	0	0	0	0	0
Nocardioides	2	157	0	1	1	0	0	0	0	0	0	1	0	0	0	0	0	1	0	0
Nocardiopsis	2	102	0	0	0	0	0	0	0	0	0	0	0	0	0	0	0	0	0	0
Nodularia	1	22	0	0	0	0	0	0	0	0	0	0	0	1	0	0	0	0	0	0
Nonlabens	1	19	0	0	0	0	0	0	0	0	0	0	0	0	0	0	0	0	0	0
Nostoc	2	111	0	0	0	0	0	0	0	0	0	0	0	1	0	0	0	0	0	0
Nostocaceae	1	2	0	0	0	0	0	0	0	0	0	0	0	0	0	0	0	0	0	0
Novosphingobium	2	169	0	0	0	0	0	0	1	0	1	1	0	0	1	0	0	0	1	0
Oceanibaculum	1	3	0	0	0	0	0	0	0	0	0	0	0	0	0	0	0	0	0	0
Oceanibulbus	1	12	0	0	0	0	0	0	0	0	0	0	0	0	0	0	0	0	0	0
Oceanicola	1	9	0	0	0	0	0	0	0	0	0	0	0	0	0	0	0	0	0	0
Oceanimonas	1	33	0	0	0	0	0	0	1	0	1	0	0	0	0	0	0	0	0	0
Oceaniovalibus	1	2	0	0	0	0	0	0	0	0	0	0	0	0	0	0	0	0	0	0
Oceanithermus	2	72	0	0	0	0	0	0	0	0	0	0	0	0	0	0	0	0	0	0
Oceanobacillus	2	91	0	0	0	0	0	0	0	0	0	0	0	0	1	0	0	0	0	0
Oceanospirillum	1	2	0	0	0	0	0	0	0	0	0	0	0	0	0	0	0	0	0	0
Ochrobactrum	2	133	0	0	0	0	0	0	0	0	0	0	0	0	0	0	0	0	0	0
Octadecabacter	1	29	0	0	0	0	0	0	0	0	0	0	0	0	0	0	0	0	0	0
Oenococcus	2	41	0	0	0	0	0	0	0	0	0	0	0	0	0	0	0	0	0	0
Oleomonas	1	2	0	0	0	0	0	0	0	0	0	0	0	0	0	0	0	0	0	0
Oligotropha	3	87	0	0	0	0	0	0	0	0	0	0	0	1	0	0	0	0	0	0
Olsenella	2	40	0	0	0	0	0	0	0	0	0	0	0	0	0	0	0	0	0	0
Opitutus	2	67	0	0	0	0	0	0	0	0	0	0	0	0	0	0	0	0	0	0
Orientia	1	21	0	0	0	0	0	0	0	0	0	0	0	0	0	0	0	0	0	0
Ornithobacterium	1	11	0	0	0	0	0	0	0	0	0	0	0	0	0	0	0	0	0	0
Oscillatoria	1	16	0	0	0	0	0	0	0	0	0	0	0	0	0	0	0	0	0	0
Oscillatoriales	1	2	0	0	0	0	0	0	0	0	0	0	0	0	0	0	0	0	0	0
Oscillibacter	1	20	0	0	0	0	0	0	0	0	0	0	0	1	0	0	0	0	0	0
Owenweeksia	1	15	0	0	0	0	0	0	0	0	0	0	0	0	0	0	0	0	0	0
Oxalobacteraceae	1	4	0	0	0	0	0	0	0	0	0	0	0	0	0	0	0	0	0	0
Paenibacillus	2	152	0	0	0	0	0	0	0	0	0	0	0	0	0	0	0	0	0	0
Paludibacter	2	67	0	0	0	0	0	0	0	0	0	0	0	0	0	0	0	0	0	0
Pandoraea	1	18	0	0	0	0	0	0	0	0	0	0	0	0	0	0	0	0	1	0
Pannonibacter	1	3	0	0	0	0	0	0	0	0	0	0	0	0	0	0	0	0	0	0

Genus	Cluster Number	Protein Counts	K04112	K04116	K04117	K04118	K05296	K05394	K05549	K05599	K05783	K05898	K05913	K06281	K06446	K06912	K07408	K07535	K07536	K07537
**Pantholops**	1	23	0	0	0	0	0	0	0	0	0	0	0	0	1	0	0	0	0	0
**Pantoea**	2	181	0	0	0	0	0	0	0	0	0	0	0	0	0	0	0	0	0	0
**Parabacteroides**	2	64	0	0	0	0	0	0	0	0	0	0	0	1	0	0	0	0	0	0
**Parachlamydia**	1	11	0	0	0	0	0	0	0	0	0	0	0	0	0	0	0	0	0	0
**Paracoccus**	2	169	0	0	0	0	0	0	1	0	1	0	0	1	1	0	0	0	0	0
**Parvibaculum**	3	117	0	1	1	0	0	0	1	0	0	1	0	0	1	0	0	1	1	0
**Parvularcula**	2	72	0	0	0	0	0	0	0	0	0	0	0	0	0	0	0	0	0	0
**Pasteurella**	2	85	0	0	0	0	0	0	0	0	0	0	0	0	0	0	0	0	0	0
**Pasteurellaceae**	1	2	0	0	0	0	0	0	0	0	0	0	0	0	0	0	0	0	0	0
**Pectobacterium**	1	44	0	0	0	0	0	0	0	0	0	0	0	1	0	0	0	0	0	0
**Pediococcus**	1	43	0	0	0	0	0	0	0	0	0	0	0	0	0	0	0	0	0	0
**Pedobacter**	2	88	0	0	0	0	0	0	0	0	0	0	0	0	0	0	0	0	0	0
**Pelagibaca**	1	10	0	0	0	0	0	0	0	0	0	0	0	0	0	0	0	0	0	0
**Pelagibacterium**	1	39	0	0	0	0	0	0	0	0	0	0	0	0	0	0	0	0	0	0
**Pelobacter**	2	85	0	0	0	0	0	0	0	0	0	0	0	0	0	0	0	0	0	0
**Pelodictyon**	2	62	0	0	0	0	0	0	0	0	0	0	0	1	0	0	0	0	0	0
**Pelosinus**	1	1	0	0	0	0	0	0	0	0	0	0	0	0	0	0	0	0	0	0
**Pelotomaculum**	2	60	0	0	0	0	0	0	0	0	0	0	0	0	0	0	0	0	0	0
**Peptostreptococcus**	1	1	0	0	0	0	0	0	0	0	0	0	0	0	0	0	0	0	0	0
**Persephonella**	2	55	0	0	0	0	0	0	0	0	0	0	0	0	0	0	0	0	0	0
**Petrotoga**	2	72	0	0	0	0	0	0	0	0	0	0	0	0	0	0	0	0	0	0
**Phaeobacter**	2	55	0	0	0	0	0	0	0	0	0	0	0	0	0	0	0	0	0	0
**Phenylobacterium**	3	103	0	0	0	0	0	0	0	0	0	0	0	0	1	0	0	0	0	0
**Photobacterium**	2	119	0	0	0	0	0	0	0	0	0	0	0	0	0	0	0	0	0	0
**Photorhabdus**	2	149	0	0	0	0	0	0	1	0	0	0	0	0	0	0	0	0	0	0
**Phycisphaera**	1	9	0	0	0	0	0	0	0	0	0	0	0	0	0	0	0	0	0	0
**Phyllobacterium**	1	6	0	0	0	0	0	0	0	0	0	0	0	0	0	0	0	0	0	0
**Picrophilus**	1	15	0	0	0	0	0	0	0	0	0	0	0	0	0	0	0	0	0	0
**Pirellula**	2	85	0	0	0	0	0	0	0	0	0	0	0	0	0	0	0	0	0	0
**Planctomyces**	2	93	0	0	0	0	0	0	0	0	0	0	0	0	0	0	0	0	0	0
**Plautia**	1	17	0	0	0	0	0	0	0	0	0	0	0	0	0	0	0	0	0	0
**Plesiomonas**	1	4	0	0	0	0	0	0	0	0	0	0	0	0	0	0	0	0	0	0
**Pleurocapsa**	1	23	0	0	0	0	0	0	0	0	0	0	0	0	0	0	0	0	0	0
**Polaribacter**	2	74	0	0	0	0	0	0	0	0	0	0	0	0	0	0	0	0	0	0
**Polaromonas**	3	238	0	1	1	0	0	0	1	0	1	0	0	1	1	0	0	1	1	0
**Polymorphum**	3	173	0	1	1	0	0	0	0	0	0	0	0	0	1	0	0	1	1	0
**Polynucleobacter**	1	19	0	0	0	0	0	0	0	0	0	0	0	0	0	0	0	0	0	0
**Ponticaulis**	1	1	0	0	0	0	0	0	0	0	0	0	0	0	0	0	0	0	0	0
**Porphyromonas**	2	63	0	0	0	0	0	0	0	0	0	0	0	0	0	0	0	0	0	0
**Prevotella**	1	65	0	0	0	0	0	0	0	0	0	0	0	0	0	0	0	0	0	0
**Prochlorococcus**	2	68	0	0	0	0	0	0	0	0	0	0	0	0	0	0	0	0	0	0
**Propionibacterium**	1	62	0	0	0	0	0	0	0	0	0	0	0	0	0	0	0	0	0	0
**Prosthecochloris**	1	16	0	0	0	0	0	0	0	0	0	0	0	1	0	0	0	0	0	0
**Proteobacteria**	1	19	0	0	0	0	0	0	0	0	0	0	0	0	0	0	0	0	0	0
**Proteus**	2	113	0	0	0	0	0	0	0	0	0	0	0	1	0	0	0	0	0	0
**Providencia**	1	38	0	0	0	0	0	0	0	0	0	0	0	0	0	0	0	0	0	0
**Pseudaminobacter**	1	10	0	0	0	0	0	0	0	0	0	0	0	0	0	0	0	0	0	0
**Pseudanabaena**	1	11	0	0	0	0	0	0	0	0	0	0	0	0	0	0	0	0	0	0
**Pseudoalteromonas**	2	188	0	0	0	0	0	0	0	0	0	1	0	0	0	0	0	0	0	0
**Pseudochrobactrum**	1	7	0	0	0	0	0	0	0	0	0	0	0	0	0	0	0	0	0	0
**Pseudogulbenkiania**	1	40	0	0	0	0	0	0	1	1	1	0	0	0	0	0	0	0	0	0
**pseudomallei**	3	131	0	0	0	0	0	0	0	0	0	0	1	0	0	0	0	0	0	0

Genus	Cluster Number	Protein Counts	K04112	K04116	K04117	K04118	K05296	K05394	K05549	K05599	K05783	K05898	K05913	K06281	K06446	K06912	K07408	K07535	K07536	K07537
**Pseudomonas**	3	381	0	0	0	0	0	0	1	1	1	1	1	0	1	1	0	0	0	0
**Pseudonocardia**	3	180	0	1	1	0	0	0	1	0	0	0	0	1	1	0	0	1	0	0
**Pseudorhodobacter**	1	4	0	0	0	0	0	0	0	0	0	0	0	0	0	0	0	0	0	0
**Pseudovibrio**	1	33	0	0	0	0	0	0	0	0	0	0	0	0	0	0	0	0	0	0
**Pseudoxanthomonas**	2	124	0	0	0	0	0	0	1	0	1	0	0	0	0	0	0	0	0	0
**Psychrobacter**	2	124	0	0	0	0	0	0	1	0	1	0	0	0	1	0	0	0	0	0
**Psychroflexus**	1	22	0	0	0	0	0	0	0	0	0	0	0	0	0	0	0	0	0	0
**Psychromonas**	2	110	0	0	0	0	0	0	0	0	0	0	0	0	0	0	0	0	0	0
**Pusillimonas**	3	111	0	0	0	0	0	0	0	0	0	0	0	0	1	0	0	0	0	0
**Pyrobaculum**	1	15	0	0	0	0	0	0	0	0	0	0	0	1	0	0	0	0	0	0
**Pyrococcus**	1	10	0	0	0	0	0	0	0	0	0	0	0	0	0	0	0	0	0	0
**Pyrolobus**	1	4	0	0	0	0	0	0	0	0	0	0	0	1	0	0	0	0	0	0
**Rahnella**	2	143	0	0	0	0	0	0	0	0	0	0	0	0	0	0	0	0	0	0
**Ralstonia**	3	332	0	1	1	1	1	0	1	0	1	1	0	0	1	1	0	1	1	0
**Ramlibacter**	1	35	0	0	0	0	0	0	0	0	0	0	0	0	0	0	0	0	0	0
**Raoultella**	2	139	0	0	0	0	0	0	1	0	1	0	0	0	0	0	0	0	0	0
**Raphidiopsis**	1	5	0	0	0	0	0	0	0	0	0	0	0	0	0	0	0	0	0	0
**Reinekea**	1	2	0	0	0	0	0	0	0	0	0	0	0	0	0	0	0	0	0	0
**Renibacterium**	2	67	0	0	0	0	0	0	0	0	0	0	0	0	0	0	0	0	0	0
**Rhizobiaceae**	1	3	0	0	0	0	0	0	0	0	0	0	0	0	0	0	0	0	0	0
**Rhizobiales**	1	6	0	0	0	0	0	0	0	0	0	0	0	0	0	0	0	0	0	0
**Rhizobium**	3	257	0	0	0	0	0	0	0	0	0	0	0	1	0	0	0	0	0	0
**Rhizobium/Agrobacterium**	2	87	0	0	0	0	0	0	0	0	0	0	0	0	0	0	0	0	0	0
**Rhodanobacter**	1	21	0	0	0	0	0	0	0	0	0	0	0	0	0	0	0	0	0	0
**Rhodobacter**	3	175	0	0	0	0	0	0	0	0	0	0	0	1	1	0	0	0	0	0
**Rhodobacteraceae**	1	21	0	0	0	0	0	0	0	0	0	0	0	0	0	0	0	0	0	0
**Rhodobacterales**	1	34	0	0	0	0	0	0	0	0	0	0	0	0	0	0	0	0	0	0
**Rhodococcus**	2	292	0	1	1	0	0	1	1	0	1	1	0	1	1	0	0	1	0	0
**Rhodocyclaceae**	1	7	0	0	0	0	0	0	0	0	0	0	0	1	0	0	0	0	0	0
**Rhodoferax**	3	144	0	0	0	0	0	0	0	0	0	0	0	1	0	0	0	0	0	0
**Rhodomicrobium**	2	103	1	0	0	0	0	0	0	0	0	0	0	1	0	0	0	0	0	1
**Rhodopirellula**	2	87	0	0	0	0	0	0	0	0	0	0	0	0	0	0	0	0	0	0
**Rhodopseudomonas**	3	246	1	1	1	0	0	0	1	0	0	0	0	1	0	0	0	1	1	0
**Rhodospirillum**	2	121	0	0	0	0	0	0	0	0	0	0	0	1	0	0	0	0	0	0
**Rhodothermus**	2	76	0	0	0	0	0	0	0	0	0	0	0	0	0	0	0	0	0	0
**Rhodovulum**	1	4	0	0	0	0	0	0	0	0	0	0	0	0	0	0	0	0	0	0
**Richelia**	1	2	0	0	0	0	0	0	0	0	0	0	0	0	0	0	0	0	0	0
**Rickettsia**	2	43	0	0	0	0	0	0	0	0	0	0	0	0	0	0	0	0	0	0
**Rickettsiaceae**	1	5	0	0	0	0	0	0	0	0	0	0	0	0	0	0	0	0	0	0
**Riemerella**	2	61	0	0	0	0	0	0	0	0	0	0	0	0	0	0	0	0	0	0
**Rivularia**	1	18	0	0	0	0	0	0	0	0	0	0	0	1	0	0	0	0	0	0
**Robiginitalea**	2	83	0	0	0	0	0	0	0	0	0	0	0	0	0	0	0	0	0	0
**Roseburia**	2	58	0	0	0	0	0	0	0	0	0	0	0	0	0	0	0	0	0	0
**Roseibium**	1	3	0	0	0	0	0	0	0	0	0	0	0	0	0	0	0	0	0	0
**Roseiflexus**	3	100	0	0	0	0	0	0	0	0	0	0	0	1	0	0	0	0	0	0
**Roseobacter**	2	149	0	0	0	0	0	0	0	0	0	0	0	0	0	0	0	0	0	0
**Roseomonas**	1	2	0	0	0	0	0	0	0	0	0	0	0	0	0	0	0	0	0	0
**Roseovarius**	1	15	0	0	0	0	0	0	0	0	0	0	0	0	0	0	0	0	0	0
**Rothia**	1	44	0	0	0	0	0	0	0	0	0	0	0	0	0	0	0	0	0	0
**Rubrivivax**	1	24	0	0	0	0	0	0	0	0	0	0	0	1	0	0	0	0	0	0
**Rubrobacter**	3	117	0	0	0	0	0	0	0	0	0	0	0	0	0	0	0	0	0	0
**Ruegeria**	2	189	0	0	0	0	0	0	0	0	0	0	0	0	0	1	0	0	0	0

Genus	Cluster Number	Protein Counts	K04112	K04116	K04117	K04118	K05296	K05394	K05549	K05599	K05783	K05898	K05913	K06281	K06446	K06912	K07408	K07535	K07536	K07537
Ruminococcaceae	1	2	0	0	0	0	0	0	0	0	0	0	0	0	0	0	0	0	0	0
Ruminococcus	2	101	0	0	0	0	0	0	0	0	0	0	0	0	0	0	0	0	0	0
Runella	1	26	0	0	0	0	0	0	0	0	0	0	0	1	0	0	0	0	0	0
Saccharomonospora	2	132	0	0	0	0	0	0	1	0	0	0	0	1	1	0	0	0	0	0
Saccharophagus	2	81	0	0	0	0	0	0	0	0	0	0	0	0	0	0	0	0	0	0
Saccharopolyspora	3	194	0	0	0	0	0	0	1	0	0	0	0	1	0	0	0	1	0	0
Saccharothrix	1	32	0	0	0	0	0	0	0	0	0	0	0	0	0	0	0	0	0	0
Sagittula	1	10	0	0	0	0	0	0	0	0	0	0	0	0	0	0	0	0	0	0
Salinibacter	2	77	0	0	0	0	0	0	0	0	0	0	0	0	0	0	0	0	0	0
Salinimonas	1	1	0	0	0	0	0	0	0	0	0	0	0	0	0	0	0	0	0	0
Salinispora	3	127	0	0	0	0	0	0	0	0	0	1	0	0	0	0	0	0	0	0
Salinivibrio	1	2	0	0	0	0	0	0	0	0	0	0	0	0	0	0	0	0	0	0
Salmonella	2	151	0	0	0	0	0	0	0	0	0	0	0	1	0	0	0	0	0	0
Sanguibacter	2	73	0	0	0	0	0	0	0	0	0	0	0	0	0	0	0	0	0	0
Saprospira	1	13	0	0	0	0	0	0	0	0	0	0	0	0	0	0	0	0	0	0
Scardovia	1	1	0	0	0	0	0	0	0	0	0	0	0	0	0	0	0	0	0	0
Scytonema	1	10	0	0	0	0	0	0	0	0	0	0	0	0	0	0	0	0	0	0
Sebaldella	2	69	0	0	0	0	0	0	0	0	0	0	0	0	0	0	0	0	0	0
Segniliparus	2	85	0	0	0	0	0	0	0	0	0	0	0	0	0	0	0	0	0	0
Selenomonas	2	57	0	0	0	0	0	0	0	0	0	0	0	1	0	0	0	0	0	0
Serinicoccus	1	1	0	0	0	0	0	0	0	0	0	0	0	0	0	0	0	0	0	0
Serratia	2	186	0	0	0	0	0	0	1	0	1	0	0	0	0	0	0	0	0	0
Shewanella	3	214	0	0	0	0	0	0	0	0	0	1	0	0	0	0	0	0	0	0
Shigella	2	172	0	0	0	0	0	0	0	0	0	0	0	1	0	0	0	0	0	0
Shinella	1	2	0	0	0	0	0	0	0	0	0	0	0	0	0	0	0	0	0	0
Sideroxydans	2	81	0	0	0	0	0	0	0	0	0	0	0	1	0	0	0	0	0	0
Silicibacter	2	105	0	0	0	0	0	0	0	0	0	0	0	0	0	0	0	0	0	0
Simiduia	1	18	0	0	0	0	0	0	0	0	0	0	0	0	0	0	0	0	0	0
Simkania	1	6	0	0	0	0	0	0	0	0	0	0	0	0	0	0	0	0	0	0
Simonsiella	1	2	0	0	0	0	0	0	0	0	0	0	0	0	0	0	0	0	0	0
Singulisphaera	1	18	0	0	0	0	0	0	0	0	0	0	0	1	0	0	0	0	0	0
Sinorhizobium	3	207	0	0	0	0	0	0	1	0	1	0	0	0	0	0	0	0	0	0
Slackia	2	46	0	0	0	0	0	0	0	0	0	0	0	1	1	0	0	0	0	0
Smaragdicoccus	1	3	0	0	0	0	0	0	0	0	0	0	0	1	0	0	0	0	0	0
Sodalis	2	71	0	0	0	0	0	0	0	0	0	0	0	0	0	0	0	0	0	0
Solibacillus	1	23	0	0	0	0	0	0	0	0	0	0	0	0	1	0	0	0	0	0
Solitalea	1	13	0	0	0	0	0	0	0	0	0	0	0	0	0	0	0	0	0	0
Sorangium	3	115	0	0	0	0	0	0	0	0	0	0	0	0	0	0	0	0	0	0
Sphaerobacter	2	100	0	0	0	0	0	0	0	0	0	0	0	1	0	0	0	0	0	0
Sphaerochaeta	1	42	0	0	0	0	0	0	0	0	0	0	0	0	0	0	0	0	0	0
Sphingobacterium	3	85	0	0	0	0	0	0	0	0	0	0	0	0	0	0	0	0	0	0
Sphingobium	1	179	0	0	0	0	0	0	1	0	0	0	0	0	1	1	0	0	0	0
Sphingomonadaceae	1	15	0	0	0	0	0	0	0	0	0	0	0	0	0	0	0	0	0	0
Sphingomonas	2	220	0	0	0	0	0	0	1	0	1	1	0	0	0	0	0	1	0	0
Sphingopyxis	3	99	0	0	0	0	0	0	0	0	0	0	0	0	1	0	0	0	0	0
Spirochaeta	2	103	0	0	0	0	0	0	0	0	0	0	0	0	0	0	0	0	0	0
Spiroplasma	1	6	0	0	0	0	0	0	0	0	0	0	0	0	0	0	0	0	0	0
Spirosoma	2	107	0	0	0	0	0	0	0	0	0	0	0	1	0	0	0	0	0	0
Stackebrandtia	2	110	0	0	0	0	0	0	0	0	0	0	0	0	0	0	0	0	0	0
Stanieria	1	17	0	0	0	0	0	0	0	0	0	0	0	0	0	0	0	0	0	0
Staphylococcus	2	108	0	0	0	0	0	0	0	0	0	0	0	0	0	0	0	0	0	0
Staphylothermus	1	12	0	0	0	0	0	0	0	0	0	0	0	0	0	0	0	0	0	0

Genus	Cluster Number	Protein Counts	K04112	K04116	K04117	K04118	K05296	K05394	K05549	K05599	K05783	K05898	K05913	K06281	K06446	K06912	K07408	K07535	K07536	K07537
Stappia	1	2	0	0	0	0	0	0	0	0	0	0	0	0	0	0	0	0	0	0
Starkeya	3	134	0	0	0	0	0	0	0	0	0	0	0	0	0	0	0	0	0	0
Stenotrophomonas	3	104	0	0	0	0	0	0	0	0	0	0	0	0	0	0	0	0	0	0
Stigmatella	3	129	0	0	0	0	0	0	0	0	0	0	0	0	0	0	0	0	0	0
Streptobacillus	1	38	0	0	0	0	0	0	0	0	0	0	0	0	0	0	0	0	0	0
Streptococcus	2	121	0	0	0	0	0	0	0	0	0	0	0	0	0	0	0	0	0	0
Streptomyces	2	253	0	1	0	0	0	0	0	0	0	1	0	1	0	0	0	0	0	0
Streptosporangium	3	148	0	0	0	0	0	0	0	0	0	0	0	1	0	0	0	1	0	0
Strigomonas	1	2	0	0	0	0	0	0	0	0	0	0	0	0	0	0	0	0	0	0
Sulfitobacter	1	10	0	0	0	0	0	0	0	0	0	0	0	0	0	0	0	0	0	0
Sulfobacillus	1	30	0	1	0	0	0	0	0	0	0	0	0	1	0	0	0	1	0	0
Sulfolobus	1	23	0	0	0	0	0	0	0	0	0	0	0	1	0	0	0	0	0	0
Sulfuricurvum	2	67	0	0	0	0	0	0	0	0	0	0	0	0	0	0	0	0	0	0
Sulfurihydrogenibium	2	55	0	0	0	0	0	0	0	0	0	0	0	0	0	0	0	0	0	0
Sulfurimonas	2	60	0	0	0	0	0	0	0	0	0	0	0	0	0	0	0	0	0	0
Sulfurospirillum	1	48	0	0	0	0	0	0	0	0	0	0	0	0	0	0	0	0	0	0
Sulfurovum	2	48	0	0	0	0	0	0	0	0	0	0	0	0	0	0	0	0	0	0
Symbiobacterium	2	68	0	0	0	0	0	0	0	0	0	0	0	0	0	0	0	0	0	0
Synechococcus	1	34	0	0	0	0	0	0	0	0	0	0	0	0	0	0	0	0	0	0
Synechocystis	1	40	0	0	0	0	0	0	0	0	0	0	0	0	0	0	0	0	0	0
Synergistetes	1	27	0	0	0	0	0	0	0	0	0	0	0	0	0	0	0	0	0	0
Syntrophobacter	2	101	0	0	0	0	0	0	0	0	0	0	0	0	0	0	0	0	0	0
Syntrophobotulus	2	71	0	0	0	0	0	0	0	0	0	0	0	0	0	0	0	0	0	0
Syntrophomonas	2	58	0	0	0	0	0	0	0	0	0	0	0	0	0	0	0	0	0	0
Syntrophothermus	2	60	1	0	0	0	0	0	0	0	0	0	0	1	0	0	0	0	0	0
Syntrophus	2	66	0	0	0	0	0	0	0	0	0	0	0	0	0	0	0	0	0	1
Tannerella	1	8	0	0	0	0	0	0	0	0	0	0	0	0	0	0	0	0	0	0
Taylorella	1	43	0	0	0	0	0	0	0	0	0	0	0	0	0	0	0	0	0	0
Tepidanaerobacter	2	55	0	0	0	0	0	0	0	0	0	0	0	0	0	0	0	0	0	0
Teredinibacter	2	109	0	0	0	0	0	0	0	0	0	0	0	0	0	0	0	0	0	0
Terrabacter	1	1	0	0	0	0	0	0	0	0	0	0	0	0	0	0	0	0	0	0
Terriglobus	2	83	0	0	0	0	0	0	0	0	0	0	0	0	0	0	0	0	0	0
Tetragenococcus	1	12	0	0	0	0	0	0	0	0	0	0	0	0	0	0	0	0	0	0
Thalassiobium	1	11	0	0	0	0	0	0	0	0	0	0	0	0	0	0	0	0	0	0
Thalassobacter	1	9	0	0	0	0	0	0	0	0	0	0	0	0	0	0	0	0	0	0
Thalassolituus	1	12	0	0	0	0	0	0	0	0	0	0	0	0	0	0	0	0	0	0
Thalassospira	1	2	0	0	0	0	0	0	0	0	0	0	0	0	0	0	0	0	0	0
Thauera	1	111	1	0	0	0	0	0	1	0	1	0	0	0	0	0	0	0	0	1
Thermacetogenium	1	7	0	0	0	0	0	0	0	0	0	0	0	0	0	0	0	0	0	0
Thermaerobacter	2	79	0	0	0	0	0	0	0	0	0	0	0	0	0	0	0	0	0	0
Thermanaerovibrio	2	58	0	0	0	0	0	0	0	0	0	0	0	0	0	0	0	0	0	0
Thermincola	2	62	0	0	0	0	0	0	0	0	0	0	0	1	0	0	0	0	0	0
Thermoanaerobacter	2	76	0	0	0	0	0	0	0	0	0	0	0	0	0	0	0	0	0	0
Thermoanaerobacterium	2	64	0	0	0	0	0	0	0	0	0	0	0	0	0	0	0	0	0	0
Thermobacillus	1	11	0	0	0	0	0	0	0	0	0	0	0	0	0	0	0	0	0	0
Thermobaculum	2	62	0	0	0	0	0	0	0	0	0	0	0	0	0	0	0	0	0	0
Thermobifida	2	72	0	0	0	0	0	0	0	0	0	0	0	0	0	0	0	0	0	0
Thermobispora	2	109	0	0	0	0	0	0	0	0	0	0	0	1	0	0	0	0	0	0
Thermococcus	1	13	0	0	0	0	0	0	0	0	0	0	0	0	0	0	0	0	0	0
Thermocrinis	2	60	0	0	0	0	0	0	0	0	0	0	0	1	0	0	0	0	0	0
Thermodesulfatator	1	10	0	0	0	0	0	0	0	0	0	0	0	1	0	0	0	0	0	0
Thermodesulfobacterium	1	9	0	0	0	0	0	0	0	0	0	0	0	1	0	0	0	0	0	0

Genus	Cluster Number	Protein Counts	K04112	K04116	K04117	K04118	K05296	K05394	K05549	K05599	K05783	K05898	K05913	K06281	K06446	K06912	K07408	K07535	K07536	K07537
Thermodesulfobium	1	13	0	0	0	0	0	0	0	0	0	0	0	1	0	0	0	0	0	0
Thermodesulfovibrio	2	60	0	0	0	0	0	0	0	0	0	0	0	1	0	0	0	0	0	0
Thermofilum	1	9	0	0	0	0	0	0	0	0	0	0	0	1	0	0	0	0	0	0
Thermogladius	1	7	0	0	0	0	0	0	0	0	0	0	0	0	0	0	0	0	0	0
Thermomicrobium	2	92	0	0	0	0	0	0	0	0	0	1	0	1	1	0	0	0	0	0
Thermomonospora	2	125	0	0	0	0	0	0	0	0	0	1	0	1	1	0	0	0	0	0
Thermoplasma	1	11	0	0	0	0	0	0	0	0	0	0	0	0	0	0	0	0	0	0
Thermoplasmatales	1	6	0	0	0	0	0	0	0	0	0	0	0	0	0	0	0	0	0	0
Thermoproteus	1	12	0	0	0	0	0	0	0	0	0	0	0	1	0	0	0	0	0	0
Thermosediminibacter	2	64	0	0	0	0	0	0	0	0	0	0	0	0	0	0	0	0	0	0
Thermosipho	2	70	0	0	0	0	0	0	0	0	0	0	0	0	0	0	0	0	0	0
Thermosphaera	1	10	0	0	0	0	0	0	0	0	0	0	0	0	0	0	0	0	0	0
Thermosynechococcus	1	6	0	0	0	0	0	0	0	0	0	0	0	0	0	0	0	0	0	0
Thermotoga	2	80	0	0	0	0	0	0	0	0	0	0	0	0	0	0	0	0	0	0
Thermovibrio	2	58	0	0	0	0	0	0	0	0	0	0	0	1	0	0	0	0	0	0
Thermovirga	1	9	0	0	0	0	0	0	0	0	0	0	0	0	0	0	0	0	0	0
Thermus	2	107	0	0	0	0	0	0	1	0	0	0	0	1	0	0	0	0	0	0
Thioalkalimicrobium	1	4	0	0	0	0	0	0	0	0	0	0	0	0	0	0	0	0	0	0
Thioalkalivibrio	2	91	0	0	0	0	0	0	0	0	0	0	0	1	0	0	0	0	0	0
Thiobaca	1	2	0	0	0	0	0	0	0	0	0	0	0	0	0	0	0	0	0	0
Thiobacillus	2	67	0	0	0	0	0	1	0	0	0	0	0	1	0	0	0	0	0	0
Thiocapsa	1	2	0	0	0	0	0	0	0	0	0	0	0	0	0	0	0	0	0	0
Thiocystis	1	11	0	0	0	0	0	0	0	0	0	0	0	1	0	0	0	0	0	0
Thioflavicoccus	1	13	0	0	0	0	0	0	0	0	0	0	0	1	0	0	0	0	0	0
Thiomicrospira	2	50	0	0	0	0	0	0	0	0	0	0	0	1	0	0	0	0	0	0
Thiomonas	2	85	0	0	0	0	0	0	0	0	0	0	0	1	0	0	0	0	0	0
Thiorhodococcus	1	2	0	0	0	0	0	0	0	0	0	0	0	0	0	0	0	0	0	0
Thiothrix	1	2	0	0	0	0	0	0	0	0	0	0	0	0	0	0	0	0	0	0
Tistrella	1	42	0	0	0	0	0	0	0	0	0	0	0	0	1	0	0	0	0	0
Tolumonas	2	100	0	0	0	0	0	0	0	0	0	0	0	0	0	0	0	0	0	0
Transposon	1	2	0	0	0	0	0	0	0	0	0	0	0	0	0	0	0	0	0	0
Treponema	1	63	0	0	0	0	0	0	0	0	0	0	0	0	0	0	0	0	0	0
Trichodesmium	2	71	0	0	0	0	0	0	0	0	0	0	0	0	0	0	0	0	0	0
Trichormus	1	12	0	0	0	0	0	0	0	0	0	0	0	1	0	0	0	0	0	0
Tropheryma	2	22	0	0	0	0	0	0	0	0	0	0	0	0	0	0	0	0	0	0
Truepera	2	104	0	0	0	0	0	0	0	0	0	0	0	0	0	0	0	0	0	0
Tsukamurella	2	123	0	0	0	0	0	0	0	0	0	1	0	0	0	0	0	0	0	0
Turneriella	1	16	0	0	0	0	0	0	0	0	0	0	0	0	0	0	0	0	0	0
Uliginosibacterium	1	2	0	0	0	0	0	0	0	0	0	0	0	0	0	0	0	0	0	0
Ureaplasma	1	24	0	0	0	0	0	0	0	0	0	0	0	0	0	0	0	0	0	0
Ureibacillus	1	2	0	0	0	0	0	0	0	0	0	0	0	0	0	0	0	0	0	0
Variovorax	3	187	0	0	0	0	0	0	0	0	0	0	0	1	1	1	0	0	0	0
Veillonella	1	54	0	0	0	0	0	0	0	0	0	0	0	1	0	0	0	0	0	0
Verminephrobacter	3	188	0	1	1	0	0	0	0	0	0	0	0	0	1	0	0	1	1	0
Verrucosispora	3	126	0	0	0	0	0	0	1	0	0	0	0	0	0	0	0	0	0	0
Vibrio	2	195	0	0	0	0	0	0	1	0	1	0	0	0	0	0	0	0	0	0
Vibrionales	1	46	0	0	0	0	0	0	0	0	0	0	0	0	0	0	0	0	0	0
Vitreoscilla	1	1	0	0	0	0	0	0	0	0	0	0	0	0	0	0	0	0	0	0
Vulcanisaeta	1	14	0	0	0	0	0	0	0	0	0	0	0	1	0	0	0	0	0	0
Waddlia	2	68	0	0	0	0	0	0	0	0	0	0	0	0	0	0	0	0	0	0
Wautersia	1	2	0	0	0	0	0	0	0	0	0	0	0	0	0	0	0	0	0	0
Weeksella	2	65	0	0	0	0	0	0	0	0	0	0	0	0	0	0	0	0	0	0

Genus	Cluster Number	Protein Counts	K04112	K04116	K04117	K04118	K05296	K05394	K05549	K05599	K05783	K05898	K05913	K06281	K06446	K06912	K07408	K07535	K07536	K07537
**Weissella**	1	14	0	0	0	0	0	0	0	0	0	0	0	0	0	0	0	0	0	0
**Wenxinia**	1	2	0	0	0	0	0	0	0	0	0	0	0	0	0	0	0	0	0	0
**Wigglesworthia**	1	29	0	0	0	0	0	0	0	0	0	0	0	0	0	0	0	0	0	0
**Wolbachia**	1	32	0	0	0	0	0	0	0	0	0	0	0	0	0	0	0	0	0	0
**Wolinella**	2	51	0	0	0	0	0	0	0	0	0	0	0	0	0	0	0	0	0	0
**Xanthobacter**	3	176	0	1	1	0	0	0	1	0	1	0	0	1	1	0	0	1	1	0
**Xanthobacteraceae**	1	4	0	0	0	0	0	0	0	0	0	0	0	0	0	0	0	0	0	0
**Xanthomonadaceae**	1	39	0	0	0	0	0	0	0	0	0	0	0	0	0	0	0	0	0	0
**Xanthomonas**	2	162	0	0	0	0	0	0	0	0	0	0	0	0	0	0	0	0	0	0
**Xenorhabdus**	2	127	0	0	0	0	0	0	0	0	0	0	0	0	0	0	0	0	0	0
**Xylanimonas**	2	66	0	0	0	0	0	0	0	0	0	0	0	0	0	0	0	0	0	0
**Xylella**	2	52	0	0	0	0	0	0	0	0	0	0	0	0	0	0	0	0	0	0
**Yersinia**	2	145	0	0	0	0	0	0	0	0	0	0	0	1	0	0	0	0	0	0
**Yokenella**	2	61	0	0	0	0	0	0	0	0	0	0	0	0	0	0	0	0	0	0
**Zobellia**	1	28	0	0	0	0	0	0	0	0	0	0	0	1	0	0	0	0	0	0
**Zunongwangia**	2	98	0	0	0	0	0	0	0	0	0	0	0	0	0	0	0	0	0	0
**Zymomonas**	2	67	0	0	0	0	0	0	0	0	0	0	0	0	0	0	0	0	0	0
**Zymophilus**	1	2	0	0	0	0	0	0	0	0	0	0	0	0	0	0	0	0	0	0

Genus	Cluster Number	Protein Counts	K07538	K07540	K07543	K07545	K07546	K07547	K07549	K07823	K07824	K08295	K08686	K08689	K08690	K08710	K09461	K10215	K10216	K10217
Acaricomes	1	2	0	0	0	0	0	0	0	0	0	0	0	0	0	0	0	0	0	0
Acaryochloris	2	88	0	0	0	0	0	0	0	0	0	0	0	0	0	0	0	0	0	0
Acetivibrio	1	2	0	0	0	0	0	0	0	0	0	0	0	0	0	0	0	0	0	0
Acetobacter	2	85	0	0	0	0	0	0	0	0	0	0	0	0	0	0	0	0	0	0
Acetobacteraceae	1	2	0	0	0	0	0	0	0	0	0	0	0	0	0	0	0	0	0	0
Acetobacterium	1	8	0	0	0	0	0	0	0	0	0	0	0	0	0	0	0	0	0	0
Acetohalobium	2	79	0	0	0	0	0	0	0	0	0	0	0	0	0	0	0	0	0	0
Acholeplasma	2	38	0	0	0	0	0	0	0	0	0	0	0	0	0	0	0	0	0	0
Achromobacter	2	192	0	0	1	0	1	0	0	0	0	1	0	1	0	0	1	0	0	1
Acidaminococcus	1	65	0	0	0	0	0	0	0	0	0	0	0	0	0	0	0	0	0	0
Acidianus	1	9	0	0	0	0	0	0	0	0	0	0	0	0	0	0	0	0	0	0
Acidilobus	1	8	0	0	0	0	0	0	0	0	0	0	0	0	0	0	0	0	0	0
Acidimicrobium	2	58	0	0	0	0	0	0	0	0	0	0	0	0	0	0	0	0	0	0
Acidiphilium	3	144	0	0	0	0	0	0	0	0	0	0	0	0	0	0	0	0	0	0
Acidithiobacillus	2	70	0	0	0	0	0	0	0	0	0	0	0	0	0	0	0	0	0	0
Acidobacterium	2	91	0	0	0	0	0	0	0	0	0	0	0	0	0	0	0	0	0	0
Acidocella	1	2	0	0	0	0	0	0	0	0	0	0	0	0	0	0	0	0	0	0
Acidothermus	2	68	0	0	0	0	0	0	0	0	0	0	0	0	0	0	0	0	0	0
Acidovorax	3	217	0	0	0	0	0	0	0	0	0	1	0	1	0	0	1	0	1	1
Aciduliprofundum	1	11	0	0	0	0	0	0	0	0	0	0	0	0	0	0	0	0	0	0
Acinetobacter	3	188	0	0	0	0	0	0	0	1	0	0	0	0	0	1	0	0	0	0
Actinobacillus	2	112	0	0	0	0	0	0	0	0	0	0	0	0	0	0	0	0	0	0
Actinomadura	1	1	0	0	0	0	0	0	0	0	0	0	0	0	0	0	0	0	0	0
Actinoplanes	1	52	0	0	0	0	0	0	0	0	0	0	0	0	0	0	0	0	0	0
Actinopolyspora	1	2	0	0	0	0	0	0	0	0	0	0	0	0	0	0	0	0	0	0
Actinosynnema	2	122	0	0	0	0	0	0	0	0	0	0	0	0	0	0	0	0	0	0
Advenella	1	30	0	0	0	0	0	0	0	0	0	1	0	0	0	0	1	0	0	0
Aequorivita	1	12	0	0	0	0	0	0	0	0	0	0	0	0	0	0	0	0	0	1
Aerococcus	2	57	0	0	0	0	0	0	0	0	0	0	0	0	0	0	0	0	0	0
Aeromonas	2	127	0	0	0	0	0	0	0	0	0	0	0	0	0	0	0	0	0	0
Aeropyrum	1	13	0	0	0	0	0	0	0	0	0	0	0	0	0	0	0	0	0	0
Afipia	1	54	0	0	0	0	0	0	0	0	0	0	0	0	0	0	0	0	0	0
Aggregatibacter	1	81	0	0	0	0	0	0	0	0	0	0	0	0	0	0	0	0	0	0
Agrobacterium	2	242	0	0	0	0	0	0	0	0	0	0	0	0	0	0	0	0	0	1
Ahrensia	1	2	0	0	0	0	0	0	0	0	0	0	0	0	0	0	0	0	0	0
Akkermansia	2	47	0	0	0	0	0	0	0	0	0	0	0	0	0	0	0	0	0	0
Albidiferax	3	144	0	0	0	0	0	0	0	0	0	0	0	0	0	0	0	0	0	0
Alcaligenes	1	21	0	0	0	0	0	0	0	0	0	0	0	0	0	0	0	0	0	1
Alcanivorax	2	146	0	0	0	0	0	0	0	0	0	0	0	0	0	0	0	0	0	0
Alicycliphilus	3	152	0	0	0	0	0	0	0	0	0	0	0	0	0	0	0	0	1	1
Alicyclobacillus	2	91	0	0	0	0	0	0	0	0	0	0	0	0	0	0	0	0	0	1
Aliivibrio	2	118	0	0	0	0	0	0	0	0	0	0	0	0	0	0	0	0	0	0
Alishewanella	1	1	0	0	0	0	0	0	0	0	0	0	0	0	0	0	0	0	0	0
Alistipes	1	52	0	0	0	0	0	0	0	0	0	0	0	0	0	0	0	0	0	0
Alkalilimnicola	2	97	0	0	0	0	0	0	0	0	0	0	0	0	0	0	0	0	0	0
Alkaliphilus	2	62	0	0	0	0	0	0	0	0	0	0	0	0	0	0	0	0	0	0
Allochromatium	2	84	0	0	0	0	0	0	0	0	0	0	0	0	0	0	0	0	0	0
Alphaproteobacteria	1	3	0	0	0	0	0	0	0	0	0	0	0	0	0	0	0	0	0	0
Alteromonadales	1	12	0	0	0	0	0	0	0	0	0	0	0	0	0	0	0	0	0	0
Alteromonas	1	37	0	0	0	0	0	0	0	0	0	0	0	0	0	0	0	0	0	0
Aminobacter	1	4	0	0	0	0	0	0	0	0	0	0	0	0	0	1	0	0	0	0
Aminobacterium	2	59	0	0	0	0	0	0	0	0	0	0	0	0	0	0	0	0	0	0

Genus	Cluster Number	Protein Counts	K07538	K07540	K07543	K07545	K07546	K07547	K07549	K07823	K07824	K08295	K08686	K08689	K08690	K08710	K09461	K10215	K10216	K10217
**Ammonifex**	2	50	0	0	0	0	0	0	0	0	0	0	0	0	0	0	0	0	0	0
**Amphibacillus**	1	13	0	0	0	0	0	0	0	0	0	0	0	0	0	0	0	0	0	0
**Amphritea**	1	2	0	0	0	0	0	0	0	0	0	0	0	0	0	0	0	0	0	0
**Amycolatopsis**	2	201	0	0	0	0	0	0	0	0	0	1	0	0	0	0	1	0	0	0
**Amycolicicoccus**	1	43	0	0	0	0	0	0	0	0	0	0	0	0	0	0	0	0	0	0
**Anabaena**	2	92	0	0	0	0	0	0	0	0	0	0	0	0	0	0	0	0	0	0
**Anaerobaculum**	1	11	0	0	0	0	0	0	0	0	0	0	0	0	0	0	0	0	0	0
**Anaerococcus**	2	46	0	0	0	0	0	0	0	0	0	0	0	0	0	0	0	0	0	0
**Anaerolinea**	2	66	0	0	0	0	0	0	0	0	0	0	0	0	0	0	0	0	0	0
**Anaeromyxobacter**	3	117	0	0	0	0	0	0	0	0	0	0	0	0	0	0	0	0	0	0
**Anaerostipes**	1	44	0	0	0	0	0	0	0	0	0	0	0	0	0	0	0	0	0	0
**Anaerotruncus**	1	2	0	0	0	0	0	0	0	0	0	0	0	0	0	0	0	0	0	0
**Anaplasma**	1	35	0	0	0	0	0	0	0	0	0	0	0	0	0	0	0	0	0	0
**Ancylobacter**	1	3	0	0	0	0	0	0	0	0	0	0	0	0	0	0	0	0	0	0
**Aneurinibacillus**	1	1	0	0	0	0	0	0	0	0	0	0	0	0	0	0	0	0	0	0
**Angomonas**	1	1	0	0	0	0	0	0	0	0	0	0	0	0	0	0	0	0	0	0
**Anoxybacillus**	2	72	0	0	0	0	0	0	0	0	0	0	0	0	0	0	0	0	0	0
**Aquifex**	2	45	0	0	0	0	0	0	0	0	0	0	0	0	0	0	0	0	0	0
**Aquimarina**	1	2	0	0	0	0	0	0	0	0	0	0	0	0	0	0	0	0	0	0
**Arcanobacterium**	2	45	0	0	0	0	0	0	0	0	0	0	0	0	0	0	0	0	0	0
**Archaeoglobus**	1	14	0	0	0	0	0	0	0	0	0	0	0	0	0	0	0	0	0	0
**Arcobacter**	2	101	0	0	0	0	0	0	0	0	0	0	0	0	0	0	0	0	0	1
**Aromatoleum**	3	133	1	1	1	1	1	0	1	0	0	0	0	0	0	0	1	0	0	0
**Arsenophonus**	1	4	0	0	0	0	0	0	0	0	0	0	0	0	0	0	0	0	0	0
**Arthrobacter**	2	206	0	0	0	0	0	0	0	0	0	1	0	0	0	1	1	0	0	0
**Arthrospira**	2	67	0	0	0	0	0	0	0	0	0	0	0	0	0	0	0	0	0	0
**Aster**	1	10	0	0	0	0	0	0	0	0	0	0	0	0	0	0	0	0	0	0
**Asticcacaulis**	2	103	0	0	0	0	0	0	0	0	0	0	0	0	0	0	0	0	0	0
**Atopobium**	1	28	0	0	0	0	0	0	0	0	0	0	0	0	0	0	0	0	0	0
**Aurantimonas**	1	2	0	0	0	0	0	0	0	0	0	0	0	0	0	0	0	0	0	0
**Avibacterium**	1	17	0	0	0	0	0	0	0	0	0	0	0	0	0	0	0	0	0	0
**Azoarcus**	3	172	0	0	0	0	0	0	0	0	0	1	0	0	0	0	1	0	0	1
**Azorhizobium**	3	149	0	0	0	0	0	0	0	0	0	1	0	0	0	0	1	0	0	0
**Azorhizophilus**	1	4	0	0	0	0	0	0	0	0	0	0	0	0	0	0	0	0	0	0
**Azospira**	1	19	0	0	0	0	0	0	0	0	0	0	0	0	0	0	0	0	0	0
**Azospirillum**	2	198	0	0	0	0	0	0	0	0	0	1	0	0	0	0	0	0	0	0
**Azotobacter**	3	183	0	0	0	0	0	0	0	1	0	0	0	0	0	0	0	0	0	1
**Bacillales**	1	9	0	0	0	0	0	0	0	0	0	0	0	0	0	0	0	0	0	0
**Bacilli**	1	3	0	0	0	0	0	0	0	0	0	0	0	0	0	0	0	0	0	0
**Bacillus**	2	237	0	0	0	0	0	0	0	0	0	0	0	0	0	0	0	0	0	1
**Bacteria**	2	86	0	0	0	0	0	0	0	0	0	0	0	0	0	0	1	0	0	0
**Bacteriovorax**	2	98	0	0	0	0	0	0	0	0	0	0	0	0	0	0	0	0	0	0
**Bacteroidales**	2	59	0	0	0	0	0	0	0	0	0	0	0	0	0	0	0	0	0	0
**Bacteroides**	2	96	0	0	0	0	0	0	0	0	0	0	0	0	0	0	0	0	0	0
**Bacteroidetes**	1	2	0	0	0	0	0	0	0	0	0	0	0	0	0	0	0	0	0	0
**Bartonella**	2	50	0	0	0	0	0	0	0	0	0	0	0	0	0	0	0	0	0	0
**Baumannia**	2	24	0	0	0	0	0	0	0	0	0	0	0	0	0	0	0	0	0	0
**Bdellovibrio**	2	87	0	0	0	0	0	0	0	0	0	0	0	0	0	0	0	0	0	0
**Beijerinckia**	3	123	0	0	0	0	0	0	0	0	0	0	0	0	0	0	0	0	0	0
**Belliella**	1	14	0	0	0	0	0	0	0	0	0	0	0	0	0	0	0	0	0	0
**Beutenbergia**	2	82	0	0	0	0	0	0	0	0	0	0	0	0	0	0	0	0	0	0
**Bifidobacterium**	2	61	0	0	0	0	0	0	0	0	0	0	0	0	0	0	0	0	0	0

Genus	Cluster Number	Protein Counts	K07538	K07540	K07543	K07545	K07546	K07547	K07549	K07823	K07824	K08295	K08686	K08689	K08690	K08710	K09461	K10215	K10216	K10217
Bizionia	1	1	0	0	0	0	0	0	0	0	0	0	0	0	0	0	0	0	0	0
Blastococcus	1	39	0	0	0	0	0	0	0	0	0	1	0	0	0	0	1	0	0	0
Blattabacterium	1	23	0	0	0	0	0	0	0	0	0	0	0	0	0	0	0	0	0	0
Blautia	1	22	0	0	0	0	0	0	0	0	0	0	0	0	0	0	0	0	0	0
Bordetella	3	227	0	0	1	0	0	0	0	0	0	1	0	0	1	0	1	0	0	1
Borrelia	1	32	0	0	0	0	0	0	0	0	0	0	0	0	0	0	0	0	0	0
Brachybacterium	1	18	0	0	0	0	0	0	0	0	0	0	0	0	0	0	0	0	0	0
Brachymonas	1	4	0	0	0	0	0	0	0	0	0	0	0	0	0	0	0	0	0	0
Brachyspira	2	79	0	0	0	0	0	0	0	0	0	0	0	0	0	0	0	0	0	0
Bradyrhizobiaceae	1	20	0	0	0	0	0	0	0	0	0	0	0	0	0	0	0	0	0	0
Bradyrhizobium	3	254	0	0	0	0	0	0	0	1	0	1	0	0	0	0	1	0	0	0
Brenneria	1	20	0	0	0	0	0	0	0	0	0	0	0	0	0	0	0	0	0	0
Brevibacillus	2	122	0	0	0	0	0	0	0	0	0	0	0	0	0	0	0	0	0	1
Brevibacterium	1	4	0	0	0	0	0	0	0	0	0	0	0	0	0	0	0	0	0	0
Brevundimonas	2	105	0	0	0	0	0	0	0	0	0	0	0	0	0	0	0	0	0	0
Brucella	3	138	0	0	0	0	0	0	0	0	0	0	0	0	0	0	0	0	0	0
Buchnera	1	26	0	0	0	0	0	0	0	0	0	0	0	0	0	0	0	0	0	0
Burkholderia	3	424	0	0	1	0	1	0	0	0	1	1	1	1	1	1	1	0	1	1
Burkholderiaceae	3	143	0	0	0	0	0	0	0	0	0	0	0	0	0	0	1	0	1	1
Burkholderiales	1	24	0	0	0	0	0	0	0	0	0	0	0	0	0	0	0	0	0	0
Buttiauxella	1	2	0	0	0	0	0	0	0	0	0	0	0	0	0	0	0	0	0	0
Butyrivibrio	2	67	0	0	0	0	0	0	0	0	0	0	0	0	0	0	0	0	0	0
Caldanaerobacter	2	63	0	0	0	0	0	0	0	0	0	0	0	0	0	0	0	0	0	0
Caldicellulosiruptor	2	75	0	0	0	0	0	0	0	0	0	0	0	0	0	0	0	0	0	0
Caldilinea	1	23	0	0	0	0	0	0	0	0	0	0	0	0	0	0	0	0	0	0
Caldimonas	1	6	0	0	0	0	0	0	0	0	0	0	0	0	0	0	0	0	0	0
Caldisericum	1	10	0	0	0	0	0	0	0	0	0	0	0	0	0	0	0	0	0	0
Caldisphaera	1	7	0	0	0	0	0	0	0	0	0	0	0	0	0	0	0	0	0	0
Calditerrivibrio	2	74	0	0	0	0	0	0	0	0	0	0	0	0	0	0	0	0	0	0
Caldivirga	1	13	0	0	0	0	0	0	0	0	0	0	0	0	0	0	0	0	0	0
Calothrix	1	43	0	0	0	0	0	0	0	0	0	0	0	0	0	0	0	0	0	0
Calyptogena	2	34	0	0	0	0	0	0	0	0	0	0	0	0	0	0	0	0	0	0
Campylobacter	2	80	0	0	0	0	0	0	0	0	0	0	0	0	0	0	0	0	0	0
Candidatus	2	290	0	0	0	0	0	0	0	1	0	1	0	0	0	0	0	0	0	1
Capnocytophaga	1	50	0	0	0	0	0	0	0	0	0	0	0	0	0	0	0	0	0	0
Carbophilus	1	2	0	0	0	0	0	0	0	0	0	0	0	0	0	0	0	0	0	0
Carboxydibrachium	1	14	0	0	0	0	0	0	0	0	0	0	0	0	0	0	0	0	0	0
Carboxydothermus	2	68	0	0	0	0	0	0	0	0	0	0	0	0	0	0	0	0	0	0
Carica	1	2	0	0	0	0	0	0	0	0	0	0	0	0	0	0	0	0	0	0
Carnobacterium	2	71	0	0	0	0	0	0	0	0	0	0	0	0	0	0	0	0	0	0
Catenibacterium	1	2	0	0	0	0	0	0	0	0	0	0	0	0	0	0	0	0	0	0
Catenovulum	1	2	0	0	0	0	0	0	0	0	0	0	0	0	0	0	0	0	0	0
Catenulispora	3	159	0	0	0	0	0	0	0	0	0	1	0	0	0	0	1	0	0	0
Caulobacter	3	168	0	0	0	0	0	0	0	1	0	0	0	0	0	0	0	0	0	0
Cedecea	1	45	0	0	0	0	0	0	0	0	0	0	0	0	0	0	0	0	0	0
Cellulomonas	2	96	0	0	0	0	0	0	0	0	0	0	0	0	0	0	0	0	0	0
Cellulophaga	2	105	0	0	0	0	0	0	0	0	0	0	0	0	0	0	0	0	0	0
Cellulosilyticum	1	14	0	0	0	0	0	0	0	0	0	0	0	0	0	0	0	0	0	0
Cellvibrio	2	85	0	0	0	0	0	0	0	0	0	0	0	0	0	0	0	0	0	0
Cenarchaeum	1	7	0	0	0	0	0	0	0	0	0	0	0	0	0	0	0	0	0	0
Chamaesiphon	1	13	0	0	0	0	0	0	0	0	0	0	0	0	0	0	0	0	0	0
Chelativorans	3	136	0	0	0	0	0	0	0	0	0	0	0	0	0	0	0	0	0	0

Genus	Cluster Number	Protein Counts	K07538	K07540	K07543	K07545	K07546	K07547	K07549	K07823	K07824	K08295	K08686	K08689	K08690	K08710	K09461	K10215	K10216	K10217
Chelatococcus	1	2	0	0	0	0	0	0	0	0	0	0	0	0	0	0	0	0	0	0
Chitinophaga	2	98	0	0	0	0	0	0	0	0	0	0	0	0	0	0	0	0	0	0
Chlamydia	1	36	0	0	0	0	0	0	0	0	0	0	0	0	0	0	0	0	0	0
Chlamydomonas	1	3	0	0	0	0	0	0	0	0	0	0	0	0	0	0	0	0	0	0
Chlamydophila	1	31	0	0	0	0	0	0	0	0	0	0	0	0	0	0	0	0	0	0
Chlorobaculum	2	61	0	0	0	0	0	0	0	0	0	0	0	0	0	0	0	0	0	0
Chlorobium	2	89	0	0	0	0	0	0	0	0	0	0	0	0	0	0	0	0	0	0
Chlorobium/Pelodictyon	1	2	0	0	0	0	0	0	0	0	0	0	0	0	0	0	0	0	0	0
Chloroflexus	2	105	0	0	0	0	0	0	0	0	0	0	0	0	0	0	0	0	0	1
Chlorogloeopsis	1	9	0	0	0	0	0	0	0	0	0	0	0	0	0	0	0	0	0	0
Chloroherpeton	2	59	0	0	0	0	0	0	0	0	0	0	0	0	0	0	0	0	0	0
Chromobacterium	2	117	0	0	0	0	0	0	0	0	0	0	0	0	0	0	0	0	0	0
Chromohalobacter	2	145	0	0	0	0	0	0	0	0	0	0	0	0	0	0	0	0	0	0
Chroococcidiopsis	1	24	0	0	0	0	0	0	0	0	0	0	0	0	0	0	0	0	0	0
Chryseobacterium	2	104	0	0	0	0	0	0	0	0	0	1	0	0	0	0	0	0	0	0
Citreicella	1	13	0	0	0	0	0	0	0	0	0	0	0	0	0	0	0	0	0	0
Citrobacter	2	190	0	0	0	0	0	0	0	0	0	0	0	0	0	0	0	0	0	0
Clavibacter	2	83	0	0	0	0	0	0	0	0	0	0	0	0	0	0	0	0	0	0
Clonorchis	1	1	0	0	0	0	0	0	0	0	0	0	0	0	0	0	0	0	0	0
Clostridiales	2	77	0	0	0	0	0	0	0	0	0	0	0	0	0	0	0	0	0	0
Clostridium	2	191	0	0	0	0	0	0	0	0	0	0	0	0	0	0	0	0	0	0
Cohnella	1	2	0	0	0	0	0	0	0	0	0	0	0	0	0	0	0	0	0	0
Coleofasciculus	1	4	0	0	0	0	0	0	0	0	0	0	0	0	0	0	0	0	0	0
Collimonas	1	35	0	0	0	0	0	0	0	0	0	0	0	0	0	0	0	0	0	0
Colwellia	2	130	0	0	0	0	0	0	0	0	0	0	0	0	0	0	0	0	0	0
Comamonas	2	196	0	0	0	0	0	0	0	0	0	1	0	1	0	1	1	0	1	1
Conexibacter	3	117	0	0	0	0	0	0	0	0	0	0	0	0	0	0	1	0	0	0
Coprobacillus	1	2	0	0	0	0	0	0	0	0	0	0	0	0	0	0	0	0	0	0
Coprococcus	2	76	0	0	0	0	0	0	0	0	0	0	0	0	0	0	0	0	0	0
Coprothermobacter	2	45	0	0	0	0	0	0	0	0	0	0	0	0	0	0	0	0	0	0
Coraliomargarita	2	68	0	0	0	0	0	0	0	0	0	0	0	0	0	0	0	0	0	0
Corallococcus	1	29	0	0	0	0	0	0	0	0	0	0	0	0	0	0	0	0	0	1
Coriobacterium	2	36	0	0	0	0	0	0	0	0	0	0	0	0	0	0	0	0	0	0
Corynebacterineae	1	5	0	0	0	0	0	0	0	0	0	0	0	0	0	0	0	0	0	0
Corynebacterium	2	175	0	0	0	0	0	0	0	0	0	0	0	0	0	0	0	1	0	0
Coxiella	2	54	0	0	0	0	0	0	0	0	0	0	0	0	0	0	0	0	0	0
Crinalium	1	21	0	0	0	0	0	0	0	0	0	0	0	0	0	0	0	0	0	0
Croceibacter	2	80	0	0	0	0	0	0	0	0	0	0	0	0	0	0	0	0	0	1
Crocosphaera	1	6	0	0	0	0	0	0	0	0	0	0	0	0	0	0	0	0	0	0
Cronobacter	2	120	0	0	0	0	0	0	0	0	0	0	0	0	0	0	0	0	0	0
Cryptobacterium	2	39	0	0	0	0	0	0	0	0	0	0	0	0	0	0	0	0	0	0
Cucumis	1	9	0	0	0	0	0	0	0	0	0	0	0	0	0	0	0	0	0	0
Cupriavidus	3	317	0	0	0	0	1	0	0	0	0	1	0	1	1	0	1	0	1	1
Curtobacterium	1	5	0	0	0	0	0	0	0	0	0	0	0	0	0	0	0	0	0	0
Curvibacter	1	27	0	0	0	0	0	0	0	0	0	0	0	0	0	0	0	0	0	0
Cyanobacterium	1	9	0	0	0	0	0	0	0	0	0	0	0	0	0	0	0	0	0	0
Cyanobium	1	10	0	0	0	0	0	0	0	0	0	0	0	0	0	0	0	0	0	0
Cyanothece	1	34	0	0	0	0	0	0	0	0	0	0	0	0	0	0	0	0	0	0
Cyclobacterium	1	19	0	0	0	0	0	0	0	0	0	0	0	0	0	0	0	0	0	0
Cycloclasticus	1	21	0	0	0	0	0	0	0	0	0	0	0	1	0	0	0	0	0	1
Cylindrospermopsis	1	4	0	0	0	0	0	0	0	0	0	0	0	0	0	0	0	0	0	0
Cylindrospermum	1	30	0	0	0	0	0	0	0	0	0	0	0	0	0	0	0	0	0	0

Genus	Cluster Number	Protein Counts	K07538	K07540	K07543	K07545	K07546	K07547	K07549	K07823	K07824	K08295	K08686	K08689	K08690	K08710	K09461	K10215	K10216	K10217
Cytophaga	2	69	0	0	0	0	0	0	0	0	0	0	0	0	0	0	0	0	0	0
Dactylococcopsis	1	8	0	0	0	0	0	0	0	0	0	0	0	0	0	0	0	0	0	0
Dechloromonas	1	40	0	0	0	0	0	0	0	0	0	0	0	0	0	0	0	0	1	1
Dechlorosoma	1	19	0	0	0	0	0	0	0	0	0	0	0	0	0	0	0	0	0	0
Deferribacter	2	73	0	0	0	0	0	0	0	0	0	0	0	0	0	0	0	0	0	0
Dehalobacter	1	9	0	0	0	0	0	0	0	0	0	0	0	0	0	0	0	0	0	0
Dehalococcoides	2	41	0	0	0	0	0	0	0	0	0	0	0	0	0	0	0	0	0	0
Dehalogenimonas	1	37	0	0	0	0	0	0	0	0	0	0	0	0	0	0	0	0	0	0
Deinococcus	2	140	0	0	0	0	0	0	0	0	0	0	0	0	0	0	0	0	0	0
Delftia	3	177	0	0	0	0	0	0	0	0	0	1	0	0	0	0	1	0	1	1
Denitrovibrio	2	67	0	0	0	0	0	0	0	0	0	0	0	0	0	0	0	0	0	0
Desulfarculus	2	92	0	0	0	0	0	0	0	0	0	0	0	0	0	0	0	0	0	0
Desulfatibacillum	3	84	0	1	0	0	0	0	0	0	0	0	0	0	0	0	0	0	0	0
Desulfitobacterium	2	102	0	0	0	0	0	0	0	0	0	0	0	0	0	0	0	0	0	0
Desulfobacca	2	65	0	0	0	0	0	0	0	0	0	1	0	0	0	0	0	0	0	0
Desulfobacterium	2	113	0	0	0	0	0	0	0	0	0	0	0	0	0	0	0	0	0	0
Desulfobacula	1	21	1	1	1	1	1	0	1	0	0	0	0	0	0	0	0	0	0	0
Desulfobulbus	2	88	0	0	0	0	0	0	0	0	0	0	0	0	0	0	0	0	0	0
Desulfocapsa	1	9	0	0	0	0	0	0	0	0	0	0	0	0	0	0	0	0	0	0
Desulfococcus	2	80	0	0	0	0	0	0	0	0	0	0	0	0	0	0	0	0	0	0
Desulfohalobium	2	52	0	0	0	0	0	0	0	0	0	0	0	0	0	0	0	0	0	0
Desulfomicrobium	2	65	0	0	0	0	0	0	0	0	0	0	0	0	0	0	0	0	0	0
Desulfomonile	1	25	1	0	0	0	0	0	0	0	0	0	0	0	0	0	0	0	0	0
Desulforhabdus	1	2	0	0	0	0	0	0	0	0	0	0	0	0	0	0	0	0	0	0
Desulfosporosinus	1	30	0	0	0	0	0	0	0	0	0	0	0	0	0	0	0	0	0	0
Desulfotalea	2	66	0	0	0	0	0	0	0	0	0	0	0	0	0	0	0	0	0	0
Desulfotomaculum	2	98	0	0	0	0	0	0	0	0	0	1	0	0	0	0	0	0	0	0
Desulfovibrio	2	144	0	0	0	0	0	0	0	0	0	0	0	0	0	0	0	0	0	0
Desulfurispirillum	2	78	0	0	0	0	0	0	0	0	0	0	0	0	0	0	0	0	0	0
Desulfurivibrio	2	71	0	0	0	0	0	0	0	0	0	0	0	0	0	0	0	0	0	0
Desulfurobacterium	2	53	0	0	0	0	0	0	0	0	0	0	0	0	0	0	0	0	0	0
Desulfurococcus	1	11	0	0	0	0	0	0	0	0	0	0	0	0	0	0	0	0	0	0
Diaphorobacter	1	16	0	0	0	0	0	0	0	0	0	0	0	0	0	0	0	0	1	1
Dichelobacter	2	45	0	0	0	0	0	0	0	0	0	0	0	0	0	0	0	0	0	0
Dickeya	2	155	0	0	0	0	0	0	0	0	0	0	0	0	0	0	0	0	0	0
Dictyoglomus	2	62	0	0	0	0	0	0	0	0	0	0	0	0	0	0	0	0	0	0
Dinoroseobacter	3	148	0	0	0	0	0	0	0	0	0	0	0	0	0	0	0	0	0	0
Dokdonia	1	3	0	0	0	0	0	0	0	0	0	0	0	0	0	0	0	0	0	0
Dorea	1	7	0	0	0	0	0	0	0	0	0	0	0	0	0	0	0	0	0	0
Dyadobacter	2	83	0	0	0	0	0	0	0	0	0	0	0	0	0	0	0	0	0	0
Echinicola	1	20	0	0	0	0	0	0	0	0	0	0	0	0	0	0	0	0	0	0
Edwardsiella	2	126	0	0	0	0	0	0	0	0	0	0	0	0	0	0	0	0	0	0
Eggerthella	1	51	0	0	0	0	0	0	0	0	0	0	0	0	0	0	0	0	0	0
Ehrlichia	2	37	0	0	0	0	0	0	0	0	0	0	0	0	0	0	0	0	0	0
Eikenella	1	4	0	0	0	0	0	0	0	0	0	0	0	0	0	0	0	0	0	0
Elusimicrobium	2	44	0	0	0	0	0	0	0	0	0	0	0	0	0	0	0	0	0	0
Emticicia	1	21	0	0	0	0	0	0	0	0	0	0	0	0	0	0	0	0	0	0
Enhydrobacter	1	1	0	0	0	0	0	0	0	0	0	0	0	0	0	0	0	0	0	0
Ensifer	1	2	0	0	0	0	0	0	0	0	0	0	0	0	0	0	0	0	0	0
Enterobacter	2	202	0	0	0	0	0	0	0	0	0	0	0	0	0	0	0	0	0	0
Enterobacteriaceae	2	211	0	0	0	0	0	0	0	0	0	0	0	0	0	0	0	0	0	0
Enterococcus	1	187	0	0	0	0	0	0	0	0	0	0	0	0	0	0	0	0	0	0

Genus	Cluster Number	Protein Counts	K07538	K07540	K07543	K07545	K07546	K07547	K07549	K07823	K07824	K08295	K08686	K08689	K08690	K08710	K09461	K10215	K10216	K10217
Enterovibrio	1	15	0	0	0	0	0	0	0	0	0	0	0	0	0	0	0	0	0	0
Erwinia	2	151	0	0	0	0	0	0	0	0	0	0	0	0	0	0	0	0	0	0
Erysipelothrix	1	9	0	0	0	0	0	0	0	0	0	0	0	0	0	0	0	0	0	0
Erysipelotrichaceae	1	4	0	0	0	0	0	0	0	0	0	0	0	0	0	0	0	0	0	0
Erythrobacter	2	80	0	0	0	0	0	0	0	0	0	0	0	0	0	0	0	0	0	0
Escherichia	2	242	0	0	0	0	0	0	0	0	0	0	0	0	0	0	0	0	0	0
Ethanoligenens	2	69	0	0	0	0	0	0	0	0	0	0	0	0	0	0	0	0	0	0
Eubacteriaceae	1	2	0	0	0	0	0	0	0	0	0	0	0	0	0	0	0	0	0	0
Eubacterium	2	103	0	0	0	0	0	0	0	0	0	0	0	0	0	0	0	0	0	0
Eudoraea	1	4	0	0	0	0	0	0	0	0	0	0	0	0	0	0	0	0	0	0
Euryarchaeota	1	1	0	0	0	0	0	0	0	0	0	0	0	0	0	0	0	0	0	0
Exiguobacterium	2	93	0	0	0	0	0	0	0	0	0	0	0	0	0	0	0	0	0	0
Faecalibacterium	1	12	0	0	0	0	0	0	0	0	0	0	0	0	0	0	0	0	0	0
Ferrimonas	2	115	0	0	0	0	0	0	0	0	0	0	0	0	0	0	0	0	0	0
Ferroglobus	1	14	0	0	0	0	0	0	0	0	0	0	0	0	0	0	0	0	0	0
Ferroplasma	1	11	0	0	0	0	0	0	0	0	0	0	0	0	0	0	0	0	0	0
Fervidicoccus	1	7	0	0	0	0	0	0	0	0	0	0	0	0	0	0	0	0	0	0
Fervidobacterium	2	54	0	0	0	0	0	0	0	0	0	0	0	0	0	0	0	0	0	0
Fibrella	1	17	0	0	0	0	0	0	0	0	0	0	0	0	0	0	0	0	0	0
Fibrobacter	2	52	0	0	0	0	0	0	0	0	0	0	0	0	0	0	0	0	0	0
Filifactor	1	9	0	0	0	0	0	0	0	0	0	0	0	0	0	0	0	0	0	0
Finegoldia	2	55	0	0	0	0	0	0	0	0	0	0	0	0	0	0	0	0	0	0
Firmicutes	1	15	0	0	0	0	0	0	0	0	0	0	0	0	0	0	0	0	0	0
Fischerella	1	15	0	0	0	0	0	0	0	0	0	0	0	0	0	0	0	0	0	0
Flavobacteria	1	7	0	0	0	0	0	0	0	0	0	0	0	0	0	0	0	0	0	0
Flavobacteriaceae	2	69	0	0	0	0	0	0	0	0	0	0	0	0	0	0	0	0	0	0
Flavobacteriales	1	3	0	0	0	0	0	0	0	0	0	0	0	0	0	0	0	0	0	0
Flavobacterium	2	103	0	0	0	0	0	0	0	0	0	1	0	0	0	0	1	0	0	0
Flexibacter	1	17	0	0	0	0	0	0	0	0	0	0	0	0	0	0	0	0	0	1
Flexistipes	1	11	0	0	0	0	0	0	0	0	0	0	0	0	0	0	0	0	0	0
Fluoribacter	1	6	0	0	0	0	0	0	0	0	0	0	0	0	0	0	0	0	0	0
Fluviicola	2	69	0	0	0	0	0	0	0	0	0	0	0	0	0	0	0	0	0	1
Formosa	1	2	0	0	0	0	0	0	0	0	0	0	0	0	0	0	0	0	0	0
Francisella	2	88	0	0	0	0	0	0	0	0	0	0	0	0	0	0	0	0	0	0
Frankia	3	202	0	0	0	0	0	0	0	0	0	0	0	0	0	0	0	0	0	1
Frateuria	1	25	0	0	0	0	0	0	0	0	0	0	0	0	0	0	0	0	0	0
Fructobacillus	1	2	0	0	0	0	0	0	0	0	0	0	0	0	0	0	0	0	0	0
Fulvimarina	1	2	0	0	0	0	0	0	0	0	0	0	0	0	0	0	0	0	0	0
Fusobacterium	1	48	0	0	0	0	0	0	0	0	0	0	0	0	0	0	0	0	0	0
Gallibacterium	2	87	0	0	0	0	0	0	0	0	0	0	0	0	0	0	0	0	0	0
Gallionella	2	67	0	0	0	0	0	0	0	0	0	0	0	0	0	0	0	0	0	0
Gammaproteobacteria	1	9	0	0	0	0	0	0	0	0	0	0	0	0	0	0	0	0	0	0
Gardnerella	1	31	0	0	0	0	0	0	0	0	0	0	0	0	0	0	0	0	0	0
Geitlerinema	1	9	0	0	0	0	0	0	0	0	0	0	0	0	0	0	0	0	0	0
Gemmatimonas	2	93	0	0	0	0	0	0	0	0	0	1	0	0	0	0	1	0	0	0
Geobacillus	2	168	0	0	0	0	0	0	0	0	0	0	0	0	0	0	0	0	0	1
Geobacter	2	150	1	1	1	1	1	1	1	0	0	0	0	0	0	0	0	0	0	0
Geodermatophilus	2	136	0	0	0	0	0	0	0	0	0	0	0	0	0	0	0	0	0	0
Geopsychrobacter	1	2	0	0	0	0	0	0	0	0	0	0	0	0	0	0	0	0	0	0
Gillisia	1	11	0	0	0	0	0	0	0	0	0	0	0	0	0	0	0	0	0	0
Gilvimarinus	1	4	0	0	0	0	0	0	0	0	0	0	0	0	0	0	0	0	0	0
Glaciecola	2	117	0	0	0	0	0	0	0	0	0	0	0	0	0	0	0	0	0	0

Genus	Cluster Number	Protein Counts	K07538	K07540	K07543	K07545	K07546	K07547	K07549	K07823	K07824	K08295	K08686	K08689	K08690	K08710	K09461	K10215	K10216	K10217
Gloeobacter	2	67	0	0	0	0	0	0	0	0	0	0	0	0	0	0	0	0	0	0
Gloeocapsa	1	20	0	0	0	0	0	0	0	0	0	0	0	0	0	0	0	0	0	0
Gloeothece	1	1	0	0	0	0	0	0	0	0	0	0	0	0	0	0	0	0	0	0
Gluconacetobacter	2	96	0	0	0	0	0	0	0	0	0	0	0	0	0	0	0	0	0	0
Gluconobacter	2	76	0	0	0	0	0	0	0	0	0	0	0	0	0	0	0	0	0	0
Gordonia	1	158	0	0	0	0	0	0	0	0	0	0	0	0	0	0	0	0	0	1
Gordonibacter	1	32	0	0	0	0	0	0	0	0	0	0	0	0	0	0	0	0	0	0
Gramella	2	104	0	0	0	0	0	0	0	0	0	0	0	0	0	0	0	0	0	0
Granulibacter	2	90	0	0	0	0	0	0	0	0	0	0	0	0	0	0	0	0	0	0
Granulicella	2	98	0	0	0	0	0	0	0	0	0	0	0	0	0	0	0	0	0	0
Grimontia	1	9	0	0	0	0	0	0	0	0	0	0	0	0	0	0	0	0	0	0
Haemophilus	2	117	0	0	0	0	0	0	0	0	0	0	0	0	0	0	0	0	0	0
Hafnia	1	29	0	0	0	0	0	0	0	0	0	0	0	0	0	0	0	0	0	0
Hahella	3	130	0	0	0	0	0	0	0	0	0	0	0	0	0	0	0	0	0	0
Halalkalicoccus	1	18	0	0	0	0	0	0	0	0	0	0	0	0	0	0	0	0	0	0
Halanaerobium	2	89	0	0	0	0	0	0	0	0	0	0	0	0	0	0	0	0	0	0
Haliangium	2	108	0	0	0	0	0	0	0	0	0	0	0	0	0	0	1	0	0	0
Haliscomenobacter	2	107	0	0	0	0	0	0	0	0	0	0	0	0	0	0	0	0	0	0
Haloarcula	1	22	0	0	0	0	0	0	0	0	0	0	0	0	0	0	0	0	0	0
Halobacillus	1	19	0	0	0	0	0	0	0	0	0	0	0	0	0	0	0	0	0	0
Halobacteriaceae	1	1	0	0	0	0	0	0	0	0	0	0	0	0	0	0	0	0	0	0
Halobacterium	1	10	0	0	0	0	0	0	0	0	0	0	0	0	0	0	0	0	0	0
Halobacteroides	1	14	0	0	0	0	0	0	0	0	0	0	0	0	0	0	0	0	0	0
Haloferax	1	20	0	0	0	0	0	0	0	0	0	0	0	0	0	0	0	0	0	0
Halogeometricum	1	12	0	0	0	0	0	0	0	0	0	0	0	0	0	0	0	0	0	0
Halomicrobium	1	10	0	0	0	0	0	0	0	0	0	0	0	0	0	0	0	0	0	0
Halomonas	1	149	0	0	0	0	0	0	0	0	0	0	0	0	0	0	0	0	0	0
Halopiger	1	16	0	0	0	0	0	0	0	0	0	0	0	0	0	0	0	0	0	0
Haloquadratum	1	19	0	0	0	0	0	0	0	0	0	0	0	0	0	0	0	0	0	0
Halorhabdus	1	10	0	0	0	0	0	0	0	0	0	0	0	0	0	0	0	0	0	0
Halorhodospira	2	71	0	0	0	0	0	0	0	0	0	0	0	0	0	0	0	0	0	0
Halorubrum	1	16	0	0	0	0	0	0	0	0	0	0	0	0	0	0	0	0	0	0
Haloterrigena	1	19	0	0	0	0	0	0	0	0	0	0	0	0	0	0	0	0	0	0
Halothece	1	9	0	0	0	0	0	0	0	0	0	0	0	0	0	0	0	0	0	0
Halothermothrix	2	50	0	0	0	0	0	0	0	0	0	0	0	0	0	0	0	0	0	0
Halothiobacillus	2	65	0	0	0	0	0	0	0	0	0	0	0	0	0	0	0	0	0	0
Halovivax	1	12	0	0	0	0	0	0	0	0	0	1	0	0	0	0	1	0	0	0
Helicobacter	2	79	0	0	0	0	0	0	0	0	0	0	0	0	0	0	0	0	0	0
Heliobacillus	1	4	0	0	0	0	0	0	0	0	0	0	0	0	0	0	0	0	0	0
Heliobacterium	2	51	0	0	0	0	0	0	0	0	0	0	0	0	0	0	0	0	0	0
Herbaspirillum	2	172	0	0	0	0	0	0	0	0	0	0	0	0	0	1	0	0	0	1
Herminiimonas	3	89	0	0	0	0	0	0	0	0	0	0	0	0	0	0	0	0	0	0
Herpetosiphon	3	90	0	0	0	0	0	0	0	0	0	0	0	0	0	0	0	0	0	0
Hippea	2	44	0	0	0	0	0	0	0	0	0	0	0	0	0	0	0	0	0	0
Hirschia	2	102	0	0	0	0	0	0	0	0	0	0	0	0	0	0	0	0	0	0
Histophilus	2	72	0	0	0	0	0	0	0	0	0	0	0	0	0	0	0	0	0	0
Hoeflea	1	16	0	0	0	0	0	0	0	0	0	0	0	0	0	0	0	0	0	0
Hydrogenivirga	1	2	0	0	0	0	0	0	0	0	0	0	0	0	0	0	0	0	0	0
Hydrogenobacter	2	55	0	0	0	0	0	0	0	0	0	0	0	0	0	0	0	0	0	0
Hydrogenobaculum	2	44	0	0	0	0	0	0	0	0	0	0	0	0	0	0	0	0	0	0
Hydrogenophaga	1	9	0	0	0	0	0	0	0	0	0	0	0	0	0	0	0	0	0	0
Hylemonella	1	4	0	0	0	0	0	0	0	0	0	0	0	0	0	0	0	0	0	0

Genus	Cluster Number	Protein Counts	K07538	K07540	K07543	K07545	K07546	K07547	K07549	K07823	K07824	K08295	K08686	K08689	K08690	K08710	K09461	K10215	K10216	K10217
Hyperthermus	1	6	0	0	0	0	0	0	0	0	0	0	0	0	0	0	0	0	0	0
Hyphomicrobium	2	93	0	0	0	0	0	0	0	0	0	0	0	0	0	0	0	0	0	0
Hyphomonas	3	105	0	0	0	0	0	0	0	0	0	0	0	0	0	0	0	0	0	0
Ideonella	1	18	0	0	0	0	0	0	0	0	0	0	0	0	0	0	0	0	0	1
Idiomarina	2	86	0	0	0	0	0	0	0	0	0	0	0	0	0	0	0	0	0	0
Ignavibacterium	1	14	0	0	0	0	0	0	0	0	0	0	0	0	0	0	0	0	0	0
Ignicoccus	1	5	0	0	0	0	0	0	0	0	0	0	0	0	0	0	0	0	0	0
Ignisphaera	1	8	0	0	0	0	0	0	0	0	0	0	0	0	0	0	0	0	0	0
Ilyobacter	2	85	0	0	0	0	0	0	0	0	0	0	0	0	0	0	0	0	0	0
Intrasporangium	2	114	0	0	0	0	0	0	0	0	0	0	0	0	0	0	0	0	0	0
Isoptericola	1	18	0	0	0	0	0	0	0	0	0	0	0	0	0	0	0	0	0	0
Isosphaera	2	75	0	0	0	0	0	0	0	0	0	0	0	0	0	0	0	0	0	0
Jannaschia	3	151	0	0	0	0	0	0	0	0	0	1	0	0	0	0	1	0	0	0
Janthinobacterium	2	99	0	0	0	0	0	0	0	0	0	0	0	0	0	0	0	0	0	0
Jonesia	2	56	0	0	0	0	0	0	0	0	0	0	0	0	0	0	0	0	0	0
Joostella	1	3	0	0	0	0	0	0	0	0	0	0	0	0	0	0	0	0	0	0
Kaistia	1	2	0	0	0	0	0	0	0	0	0	0	0	0	0	0	0	0	0	0
Kangiella	2	84	0	0	0	0	0	0	0	0	0	0	0	0	0	0	0	0	0	1
Ketogulonicigenium	2	102	0	0	0	0	0	0	0	0	0	0	0	0	0	0	0	0	0	0
Kineococcus	1	24	0	0	0	0	0	0	0	0	0	0	0	0	0	0	0	0	0	0
Kinetoplastibacterium	1	2	0	0	0	0	0	0	0	0	0	0	0	0	0	0	0	0	0	0
Kingella	1	4	0	0	0	0	0	0	0	0	0	0	0	0	0	0	0	0	0	0
Kitasatospora	1	36	0	0	0	0	0	0	0	0	0	0	0	0	0	0	0	0	0	0
Klebsiella	3	222	0	0	0	0	0	0	0	0	0	0	0	0	0	0	0	0	0	0
Kluyvera	1	1	0	0	0	0	0	0	0	0	0	0	0	0	0	0	0	0	0	0
Kocuria	2	87	0	0	0	0	0	0	0	0	0	0	0	0	0	0	0	1	0	0
Kordia	1	5	0	0	0	0	0	0	0	0	0	0	0	0	0	0	0	0	0	0
Kosmotoga	2	61	0	0	0	0	0	0	0	0	0	0	0	0	0	0	0	0	0	0
Kribbella	2	99	0	0	0	0	0	0	0	0	0	0	0	0	0	0	0	0	0	1
Krokinobacter	2	90	0	0	0	0	0	0	0	0	0	0	0	0	0	0	0	0	0	1
Kyrpidia	2	113	0	0	0	0	0	0	0	0	0	0	0	0	0	0	0	0	0	1
Kytococcus	2	70	0	0	0	0	0	0	0	0	0	0	0	0	0	0	0	0	0	0
Labrenzia	1	4	0	0	0	0	0	0	0	0	0	0	0	0	0	0	0	0	0	0
Lachnoanaerobaculum	1	1	0	0	0	0	0	0	0	0	0	0	0	0	0	0	0	0	0	0
Lachnospiraceae	1	51	0	0	0	0	0	0	0	0	0	0	0	0	0	0	0	0	0	0
Lacinutrix	1	20	0	0	0	0	0	0	0	0	0	0	0	0	0	0	0	0	0	1
Lactobacillus	2	119	0	0	0	0	0	0	0	0	0	0	0	0	0	0	0	0	0	0
Lactococcus	2	82	0	0	0	0	0	0	0	0	0	0	0	0	0	0	0	0	0	0
Lamprocystis	1	2	0	0	0	0	0	0	0	0	0	0	0	0	0	0	0	0	0	0
Laribacter	2	80	0	0	0	0	0	0	0	0	0	0	0	0	0	0	0	0	0	0
Lawsonia	2	33	0	0	0	0	0	0	0	0	0	0	0	0	0	0	0	0	0	0
Leadbetterella	2	79	0	0	0	0	0	0	0	0	0	0	0	0	0	0	0	0	0	0
Leeuwenhoekiella	1	2	0	0	0	0	0	0	0	0	0	0	0	0	0	0	0	0	0	0
Legionella	2	117	0	0	0	0	0	0	0	0	0	0	0	0	0	0	0	0	0	0
Leifsonia	2	48	0	0	0	0	0	0	0	0	0	0	0	0	0	0	0	0	0	0
Leptolyngbya	1	10	0	0	0	0	0	0	0	0	0	0	0	0	0	0	0	0	0	0
Leptospira	2	76	0	0	0	0	0	0	0	0	0	0	0	0	0	0	0	0	0	0
Leptospirillum	1	10	0	0	0	0	0	0	0	0	0	0	0	0	0	0	0	0	0	0
Leptothrix	3	186	0	0	0	0	0	0	0	0	0	0	0	0	0	0	0	0	1	1
Leptotrichia	2	43	0	0	0	0	0	0	0	0	0	0	0	0	0	0	0	0	0	0
Leucobacter	1	2	0	0	0	0	0	0	0	0	0	0	0	0	0	0	0	0	0	0
Leuconostoc	2	64	0	0	0	0	0	0	0	0	0	0	0	0	0	0	0	0	0	0

Genus	Cluster Number	Protein Counts	K07538	K07540	K07543	K07545	K07546	K07547	K07549	K07823	K07824	K08295	K08686	K08689	K08690	K08710	K09461	K10215	K10216	K10217
**Liberibacter**	1	4	0	0	0	0	0	0	0	0	0	0	0	0	0	0	0	0	0	0
**Limnohabitans**	1	11	0	0	0	0	0	0	0	0	0	0	0	0	0	0	0	0	0	0
**Listeria**	2	77	0	0	0	0	0	0	0	0	0	0	0	0	0	0	0	0	0	0
**Listeriaceae**	1	6	0	0	0	0	0	0	0	0	0	0	0	0	0	0	0	0	0	0
**Listonella**	1	38	0	0	0	0	0	0	0	0	0	0	0	0	0	0	0	0	0	0
**Loktanella**	1	3	0	0	0	0	0	0	0	0	0	0	0	0	0	0	0	0	0	0
**Lysinibacillus**	2	97	0	0	0	0	0	0	0	0	0	0	0	0	0	0	0	0	0	0
**Macrococcus**	2	60	0	0	0	0	0	0	0	0	0	0	0	0	0	0	0	0	0	0
**Magnetococcus**	2	64	0	0	0	0	0	0	0	0	0	0	0	0	0	0	0	0	0	0
**Magnetospirillum**	2	113	1	0	0	0	0	0	0	0	0	1	0	0	0	0	0	0	0	0
**Mahella**	2	58	0	0	0	0	0	0	0	0	0	0	0	0	0	0	0	0	0	0
**Mannheimia**	2	85	0	0	0	0	0	0	0	0	0	0	0	0	0	0	0	0	0	0
**Maribacter**	3	87	0	0	0	0	0	0	0	0	0	0	0	0	0	0	0	0	0	0
**Maricaulis**	3	87	0	0	0	0	0	0	0	0	0	1	0	0	0	0	1	0	0	0
**Marichromatium**	1	2	0	0	0	0	0	0	0	0	0	0	0	0	0	0	0	0	0	0
**Marinithermus**	2	77	0	0	0	0	0	0	0	0	0	0	0	0	0	0	0	0	0	0
**Marinitoga**	1	15	0	0	0	0	0	0	0	0	0	0	0	0	0	0	0	0	0	0
**Marinobacter**	2	163	0	0	0	0	0	0	0	0	0	0	0	0	0	0	0	0	1	1
**Marinomonas**	3	192	0	0	0	0	0	0	0	0	0	0	0	0	0	0	0	0	0	0
**Maritimibacter**	1	7	0	0	0	0	0	0	0	0	0	0	0	0	0	0	0	0	0	0
**Marivirga**	2	96	0	0	0	0	0	0	0	0	0	0	0	0	0	0	0	0	0	1
**Martelella**	1	3	0	0	0	0	0	0	0	0	0	0	0	0	0	0	0	0	0	0
**Marvinbryantia**	1	1	0	0	0	0	0	0	0	0	0	0	0	0	0	0	0	0	0	0
**Massilia**	1	4	0	0	0	0	0	0	0	0	0	0	0	0	0	0	0	0	0	0
**Mastigocladopsis**	1	16	0	0	0	0	0	0	0	0	0	0	0	0	0	0	0	0	0	0
**Megamonas**	1	47	0	0	0	0	0	0	0	0	0	0	0	0	0	0	0	0	0	0
**Megasphaera**	1	14	0	0	0	0	0	0	0	0	0	0	0	0	0	0	0	0	0	0
**Meiothermus**	2	110	0	0	0	0	0	0	0	0	0	1	0	0	0	0	1	0	0	0
**Melissococcus**	2	46	0	0	0	0	0	0	0	0	0	0	0	0	0	0	0	0	0	0
**Mesoflavibacter**	1	6	0	0	0	0	0	0	0	0	0	0	0	0	0	0	0	0	0	0
**Mesoplasma**	1	23	0	0	0	0	0	0	0	0	0	0	0	0	0	0	0	0	0	0
**Mesorhizobium**	2	201	0	0	0	0	0	0	0	1	0	1	0	0	0	0	1	0	0	0
**Mesotoga**	1	14	0	0	0	0	0	0	0	0	0	0	0	0	0	0	0	0	0	0
**Metallosphaera**	1	18	0	0	0	0	0	0	0	0	0	0	0	0	0	0	0	0	0	0
**Methanobacterium**	1	7	0	0	0	0	0	0	0	0	0	0	0	0	0	0	0	0	0	0
**Methanobrevibacter**	1	9	0	0	0	0	0	0	0	0	0	0	0	0	0	0	0	0	0	0
**Methanocaldococcus**	1	6	0	0	0	0	0	0	0	0	0	0	0	0	0	0	0	0	0	0
**Methanocella**	1	15	0	0	0	0	0	0	0	0	0	0	0	0	0	0	0	0	0	0
**Methanococcaceae**	1	1	0	0	0	0	0	0	0	0	0	0	0	0	0	0	0	0	0	0
**Methanococcoides**	1	5	0	0	0	0	0	0	0	0	0	0	0	0	0	0	0	0	0	0
**Methanococcus**	1	9	0	0	0	0	0	0	0	0	0	0	0	0	0	0	0	0	0	0
**Methanocorpusculum**	1	7	0	0	0	0	0	0	0	0	0	0	0	0	0	0	0	0	0	0
**Methanoculleus**	1	8	0	0	0	0	0	0	0	0	0	0	0	0	0	0	0	0	0	0
**Methanohalobium**	1	5	0	0	0	0	0	0	0	0	0	0	0	0	0	0	0	0	0	0
**Methanohalophilus**	1	5	0	0	0	0	0	0	0	0	0	0	0	0	0	0	0	0	0	0
**Methanolobus**	1	5	0	0	0	0	0	0	0	0	0	0	0	0	0	0	0	0	0	0
**Methanomethylovorans**	1	5	0	0	0	0	0	0	0	0	0	0	0	0	0	0	0	0	0	0
**Methanoplanus**	1	7	0	0	0	0	0	0	0	0	0	0	0	0	0	0	0	0	0	0
**Methanopyrus**	1	4	0	0	0	0	0	0	0	0	0	0	0	0	0	0	0	0	0	0
**Methanoregula**	1	8	0	0	0	0	0	0	0	0	0	0	0	0	0	0	0	0	0	0
**Methanosaeta**	1	9	0	0	0	0	0	0	0	0	0	0	0	0	0	0	0	0	0	0
**Methanosalsum**	1	7	0	0	0	0	0	0	0	0	0	0	0	0	0	0	0	0	0	0

Genus	Cluster Number	Protein Counts	K07538	K07540	K07543	K07545	K07546	K07547	K07549	K07823	K07824	K08295	K08686	K08689	K08690	K08710	K09461	K10215	K10216	K10217
**Methanosarcina**	1	18	0	0	0	0	0	0	0	0	0	0	0	0	0	0	0	0	0	0
**Methanosphaera**	1	7	0	0	0	0	0	0	0	0	0	0	0	0	0	0	0	0	0	0
**Methanosphaerula**	1	8	0	0	0	0	0	0	0	0	0	0	0	0	0	0	0	0	0	0
**Methanospirillum**	1	6	0	0	0	0	0	0	0	0	0	0	0	0	0	0	0	0	0	0
**Methanothermobacter**	1	6	0	0	0	0	0	0	0	0	0	0	0	0	0	0	0	0	0	0
**Methanothermococcus**	1	5	0	0	0	0	0	0	0	0	0	0	0	0	0	0	0	0	0	0
**Methanothermus**	1	5	0	0	0	0	0	0	0	0	0	0	0	0	0	0	0	0	0	0
**Methanotorris**	1	5	0	0	0	0	0	0	0	0	0	0	0	0	0	0	0	0	0	0
**Methylacidiphilum**	2	48	0	0	0	0	0	0	0	0	0	0	0	0	0	0	0	0	0	0
**Methylarcula**	1	2	0	0	0	0	0	0	0	0	0	0	0	0	0	0	0	0	0	0
**Methylibium**	1	38	0	0	0	0	0	0	0	0	0	0	0	0	0	0	0	0	1	1
**Methylobacillus**	2	61	0	0	0	0	0	0	0	0	0	0	0	0	0	0	0	0	0	0
**Methylobacterium**	3	236	0	0	0	0	0	0	0	1	0	1	0	0	0	0	1	0	0	0
**Methylocella**	3	117	0	0	0	0	0	0	0	0	0	0	0	0	0	0	0	0	0	1
**Methylococcus**	2	80	0	0	0	0	0	0	0	0	0	0	0	0	0	0	0	0	0	0
**Methylocystis**	1	21	0	0	0	0	0	0	0	0	0	0	0	0	0	0	0	0	0	0
**Methyloferula**	1	2	0	0	0	0	0	0	0	0	0	0	0	0	0	0	0	0	0	0
**Methylomicrobium**	1	12	0	0	0	0	0	0	0	0	0	0	0	0	0	0	0	0	0	0
**Methylomonas**	1	15	0	0	0	0	0	0	0	0	0	0	0	0	0	0	0	0	0	0
**Methylophaga**	1	7	0	0	0	0	0	0	0	0	0	0	0	0	0	0	0	0	0	0
**Methylophilus**	1	2	0	0	0	0	0	0	0	0	0	0	0	0	0	0	0	0	0	0
**Methylosinus**	1	2	0	0	0	0	0	0	0	0	0	0	0	0	0	0	0	0	0	0
**Methylotenera**	2	72	0	0	0	0	0	0	0	0	0	0	0	0	0	0	0	0	0	0
**Methyloversatilis**	1	5	0	0	0	0	0	0	0	0	0	0	0	0	0	0	0	0	0	0
**Methylovorus**	2	66	0	0	0	0	0	0	0	0	0	0	0	0	0	0	0	0	0	0
**Micavibrio**	1	9	0	0	0	0	0	0	0	0	0	0	0	0	0	0	0	0	0	0
**Microbacterium**	2	115	0	0	0	0	0	0	0	0	0	0	0	0	0	0	1	0	0	0
**Microchaete**	1	23	0	0	0	0	0	0	0	0	0	0	0	0	0	0	0	0	0	0
**Micrococcus**	2	72	0	0	0	0	0	0	0	0	0	0	0	0	0	0	0	0	0	0
**Microcoleus**	1	17	0	0	0	0	0	0	0	0	0	0	0	0	0	0	0	0	0	0
**Microcystis**	2	67	0	0	0	0	0	0	0	0	0	0	0	0	0	0	0	0	0	0
**Microlunatus**	1	25	0	0	0	0	0	0	0	0	0	0	0	0	0	0	0	0	0	0
**Micromonospora**	2	115	0	0	0	0	0	0	0	0	0	0	0	0	0	0	0	0	0	1
**Microvirga**	1	6	0	0	0	0	0	0	0	0	0	0	0	0	0	0	0	0	0	0
**Mobiluncus**	1	41	0	0	0	0	0	0	0	0	0	0	0	0	0	0	0	0	0	0
**Modestobacter**	1	43	0	0	0	0	0	0	0	0	0	0	0	0	0	0	0	0	0	0
**Moorea**	1	2	0	0	0	0	0	0	0	0	0	0	0	0	0	0	0	0	0	0
**Moorella**	2	69	0	0	0	0	0	0	0	0	0	0	0	0	0	0	0	0	0	0
**Moraxella**	2	66	0	0	0	0	0	0	0	0	0	0	0	0	0	0	0	0	0	0
**Morganella**	1	29	0	0	0	0	0	0	0	0	0	0	0	0	0	0	0	0	0	0
**Moritella**	1	2	0	0	0	0	0	0	0	0	0	0	0	0	0	0	0	0	0	0
**Muricauda**	1	23	0	0	0	0	0	0	0	0	0	0	0	0	0	0	0	0	0	0
**Mycobacterium**	3	306	0	0	0	0	0	0	0	0	0	0	0	0	0	0	1	1	0	1
**Mycoplasma**	1	53	0	0	0	0	0	0	0	0	0	0	0	0	0	0	0	0	0	0
**Myxococcus**	2	110	0	0	0	0	0	0	0	0	0	0	0	0	0	0	0	0	0	1
**Nakamurella**	2	115	0	0	0	0	0	0	0	0	0	0	0	0	0	0	0	0	0	0
**napthalene**	1	2	0	0	0	0	0	0	0	0	0	0	0	0	0	0	0	0	0	0
**Natranaerobius**	2	55	0	0	0	0	0	0	0	0	0	0	0	0	0	0	0	0	0	0
**Natrialba**	1	16	0	0	0	0	0	0	0	0	0	0	0	0	0	0	0	0	0	0
**Natrinema**	1	16	0	0	0	0	0	0	0	0	0	0	0	0	0	0	0	0	0	0
**Natronobacterium**	1	11	0	0	0	0	0	0	0	0	0	0	0	0	0	0	0	0	0	0
**Natronococcus**	1	21	0	0	0	0	0	0	0	0	0	1	0	0	0	0	0	0	0	0

Genus	Cluster Number	Protein Counts	K07538	K07540	K07543	K07545	K07546	K07547	K07549	K07823	K07824	K08295	K08686	K08689	K08690	K08710	K09461	K10215	K10216	K10217
**Natronomonas**	1	20	0	0	0	0	0	0	0	0	0	0	0	0	0	0	0	0	0	0
**Nautilia**	2	48	0	0	0	0	0	0	0	0	0	0	0	0	0	0	0	0	0	0
**Neisseria**	2	67	0	0	0	0	0	0	0	0	0	0	0	0	0	0	0	0	0	0
**Neisseriaceae**	1	1	0	0	0	0	0	0	0	0	0	0	0	0	0	0	0	0	0	0
**Neorickettsia**	1	31	0	0	0	0	0	0	0	0	0	0	0	0	0	0	0	0	0	0
**Neptuniibacter**	1	2	0	0	0	0	0	0	0	0	0	0	0	0	0	0	0	0	0	0
**Niastella**	1	21	0	0	0	0	0	0	0	0	0	0	0	0	0	0	0	0	0	0
**Nitratifractor**	2	54	0	0	0	0	0	0	0	0	0	0	0	0	0	0	0	0	0	0
**Nitratireductor**	1	19	0	0	0	0	0	0	0	0	0	0	0	0	0	0	0	0	0	0
**Nitratiruptor**	2	40	0	0	0	0	0	0	0	0	0	0	0	0	0	0	0	0	0	0
**Nitrobacter**	2	99	0	0	0	0	0	0	0	0	0	0	0	0	0	0	0	0	0	0
**Nitrococcus**	1	2	0	0	0	0	0	0	0	0	0	0	0	0	0	0	0	0	0	0
**Nitrosococcus**	2	94	0	0	0	0	0	0	0	0	0	0	0	0	0	0	0	0	0	0
**Nitrosomonas**	2	72	0	0	0	0	0	0	0	0	0	0	0	0	0	0	0	0	0	0
**Nitrosopumilaceae**	1	7	0	0	0	0	0	0	0	0	0	0	0	0	0	0	0	0	0	0
**Nitrosopumilus**	1	11	0	0	0	0	0	0	0	0	0	0	0	0	0	0	0	0	0	0
**Nitrosospira**	2	74	0	0	0	0	0	0	0	0	0	0	0	0	0	0	0	0	0	0
**Nocardia**	2	185	0	0	0	0	0	0	0	0	0	1	0	0	0	0	1	0	0	1
**Nocardioidaceae**	1	2	0	0	0	0	0	0	0	0	0	0	0	0	0	0	0	0	0	0
**Nocardioides**	2	157	0	0	0	0	0	0	0	0	0	0	0	0	0	1	0	0	0	0
**Nocardiopsis**	2	102	0	0	0	0	0	0	0	0	0	1	0	0	0	0	1	0	0	0
**Nodularia**	1	22	0	0	0	0	0	0	0	0	0	0	0	0	0	0	0	0	0	0
**Nonlabens**	1	19	0	0	0	0	0	0	0	0	0	0	0	0	0	0	0	0	0	1
**Nostoc**	2	111	0	0	0	0	0	0	0	0	0	0	0	0	0	0	0	0	0	0
**Nostocaceae**	1	2	0	0	0	0	0	0	0	0	0	0	0	0	0	0	0	0	0	0
**Novosphingobium**	2	169	0	0	0	0	0	0	0	1	0	0	0	0	0	0	0	0	0	1
**Oceanibaculum**	1	3	0	0	0	0	0	0	0	0	0	0	0	0	0	0	0	0	0	0
**Oceanibulbus**	1	12	0	0	0	0	0	0	0	0	0	0	0	0	0	0	0	0	0	0
**Oceanicola**	1	9	0	0	0	0	0	0	0	0	0	0	0	0	0	0	0	0	0	0
**Oceanimonas**	1	33	0	0	0	0	0	0	0	0	0	0	0	0	0	0	0	0	0	0
**Oceaniovalibus**	1	2	0	0	0	0	0	0	0	0	0	0	0	0	0	0	0	0	0	0
**Oceanithermus**	2	72	0	0	0	0	0	0	0	0	0	0	0	0	0	0	0	0	0	0
**Oceanobacillus**	2	91	0	0	0	0	0	0	0	0	0	0	0	0	0	0	0	0	0	0
**Oceanospirillum**	1	2	0	0	0	0	0	0	0	0	0	0	0	0	0	0	0	0	0	0
**Ochrobactrum**	2	133	0	0	0	0	0	0	0	0	0	1	0	0	0	0	1	0	0	0
**Octadecabacter**	1	29	0	0	0	0	0	0	0	0	0	0	0	0	0	0	0	0	0	0
**Oenococcus**	2	41	0	0	0	0	0	0	0	0	0	0	0	0	0	0	0	0	0	0
**Oleomonas**	1	2	0	0	0	0	0	0	0	0	0	0	0	0	0	0	0	0	0	0
**Oligotropha**	3	87	0	0	0	0	0	0	0	0	0	0	0	0	0	0	0	0	0	0
**Olsenella**	2	40	0	0	0	0	0	0	0	0	0	0	0	0	0	0	0	0	0	0
**Opitutus**	2	67	0	0	0	0	0	0	0	0	0	0	0	0	0	0	0	0	0	0
**Orientia**	1	21	0	0	0	0	0	0	0	0	0	0	0	0	0	0	0	0	0	0
**Ornithobacterium**	1	11	0	0	0	0	0	0	0	0	0	0	0	0	0	0	0	0	0	0
**Oscillatoria**	1	16	0	0	0	0	0	0	0	0	0	0	0	0	0	0	0	0	0	0
**Oscillatoriales**	1	2	0	0	0	0	0	0	0	0	0	0	0	0	0	0	0	0	0	0
**Oscillibacter**	1	20	0	0	0	0	0	0	0	0	0	0	0	0	0	0	0	0	0	0
**Owenweeksia**	1	15	0	0	0	0	0	0	0	0	0	0	0	0	0	0	0	0	0	1
**Oxalobacteraceae**	1	4	0	0	0	0	0	0	0	0	0	0	0	0	0	0	0	0	0	0
**Paenibacillus**	2	152	0	0	0	0	0	0	0	0	0	0	0	0	0	0	0	0	0	0
**Paludibacter**	2	67	0	0	0	0	0	0	0	0	0	0	0	0	0	0	0	0	0	0
**Pandoraea**	1	18	0	0	0	0	0	0	0	0	0	0	0	0	0	0	0	0	0	0
**Pannonibacter**	1	3	0	0	0	0	0	0	0	0	0	0	0	0	0	0	0	0	0	0

Genus	Cluster Number	Protein Counts	K07538	K07540	K07543	K07545	K07546	K07547	K07549	K07823	K07824	K08295	K08686	K08689	K08690	K08710	K09461	K10215	K10216	K10217
**Pantholops**	1	23	0	0	0	0	0	0	0	0	0	0	0	0	0	0	0	0	0	0
**Pantoea**	2	181	0	0	0	0	0	0	0	0	0	0	0	0	0	0	0	0	0	0
**Parabacteroides**	2	64	0	0	0	0	0	0	0	0	0	0	0	0	0	0	0	0	0	0
**Parachlamydia**	1	11	0	0	0	0	0	0	0	0	0	0	0	0	0	0	0	0	0	0
**Paracoccus**	2	169	0	0	0	0	0	0	0	0	0	1	0	0	0	1	1	0	0	0
**Parvibaculum**	3	117	0	0	0	0	0	0	0	0	0	0	0	0	0	0	0	0	0	0
**Parvularcula**	2	72	0	0	0	0	0	0	0	0	0	0	0	0	0	0	0	0	0	0
**Pasteurella**	2	85	0	0	0	0	0	0	0	0	0	0	0	0	0	0	0	0	0	0
**Pasteurellaceae**	1	2	0	0	0	0	0	0	0	0	0	0	0	0	0	0	0	0	0	0
**Pectobacterium**	1	44	0	0	0	0	0	0	0	0	0	0	0	0	0	0	0	0	0	0
**Pediococcus**	1	43	0	0	0	0	0	0	0	0	0	0	0	0	0	0	0	0	0	0
**Pedobacter**	2	88	0	0	0	0	0	0	0	0	0	0	0	0	0	0	0	0	0	0
**Pelagibaca**	1	10	0	0	0	0	0	0	0	0	0	0	0	0	0	0	0	0	0	0
**Pelagibacterium**	1	39	0	0	0	0	0	0	0	0	0	0	0	0	0	0	0	0	0	0
**Pelobacter**	2	85	0	0	0	0	0	0	0	0	0	0	0	0	0	0	0	0	0	0
**Pelodictyon**	2	62	0	0	0	0	0	0	0	0	0	0	0	0	0	0	0	0	0	0
**Pelosinus**	1	1	0	0	0	0	0	0	0	0	0	0	0	0	0	0	0	0	0	0
**Pelotomaculum**	2	60	0	0	0	0	0	0	0	0	0	0	0	0	0	0	0	0	0	0
**Peptostreptococcus**	1	1	0	0	0	0	0	0	0	0	0	0	0	0	0	0	0	0	0	0
**Persephonella**	2	55	0	0	0	0	0	0	0	0	0	0	0	0	0	0	0	0	0	0
**Petrotoga**	2	72	0	0	0	0	0	0	0	0	0	0	0	0	0	0	0	0	0	0
**Phaeobacter**	2	55	0	0	0	0	0	0	0	0	0	1	0	0	0	0	1	0	0	0
**Phenylobacterium**	3	103	0	0	0	0	0	0	0	0	0	0	0	0	0	0	0	0	0	0
**Photobacterium**	2	119	0	0	0	0	0	0	0	0	0	0	0	0	0	0	0	0	0	0
**Photorhabdus**	2	149	0	0	0	0	0	0	0	0	0	0	0	0	0	0	0	0	0	0
**Phycisphaera**	1	9	0	0	0	0	0	0	0	0	0	0	0	0	0	0	0	0	0	0
**Phyllobacterium**	1	6	0	0	0	0	0	0	0	0	0	0	0	0	0	0	0	0	0	0
**Picrophilus**	1	15	0	0	0	0	0	0	0	0	0	0	0	0	0	0	0	0	0	0
**Pirellula**	2	85	0	0	0	0	0	0	0	0	0	0	0	0	0	0	0	0	0	0
**Planctomyces**	2	93	0	0	0	0	0	0	0	0	0	0	0	0	0	0	0	0	0	0
**Plautia**	1	17	0	0	0	0	0	0	0	0	0	0	0	0	0	0	0	0	0	0
**Plesiomonas**	1	4	0	0	0	0	0	0	0	0	0	0	0	0	0	0	0	0	0	0
**Pleurocapsa**	1	23	0	0	0	0	0	0	0	0	0	0	0	0	0	0	0	0	0	0
**Polaribacter**	2	74	0	0	0	0	0	0	0	0	0	0	0	0	0	0	0	0	0	1
**Polaromonas**	3	238	0	0	0	0	0	0	0	0	0	1	0	1	1	0	1	0	0	1
**Polymorphum**	3	173	0	0	0	0	0	0	0	0	0	1	0	1	0	0	1	0	0	0
**Polynucleobacter**	1	19	0	0	0	0	0	0	0	0	0	0	0	0	0	0	0	0	0	0
**Ponticaulis**	1	1	0	0	0	0	0	0	0	0	0	0	0	0	0	0	0	0	0	0
**Porphyromonas**	2	63	0	0	0	0	0	0	0	0	0	0	0	0	0	0	0	0	0	0
**Prevotella**	1	65	0	0	0	0	0	0	0	0	0	0	0	0	0	0	0	0	0	0
**Prochlorococcus**	2	68	0	0	0	0	0	0	0	0	0	0	0	0	0	0	0	0	0	0
**Propionibacterium**	1	62	0	0	0	0	0	0	0	0	0	0	0	0	0	0	0	0	0	0
**Prosthecochloris**	1	16	0	0	0	0	0	0	0	0	0	0	0	0	0	0	0	0	0	0
**Proteobacteria**	1	19	0	0	0	0	0	0	0	0	0	0	0	0	0	0	0	0	0	0
**Proteus**	2	113	0	0	0	0	0	0	0	0	0	0	0	0	0	0	0	0	0	0
**Providencia**	1	38	0	0	0	0	0	0	0	0	0	0	0	0	0	0	0	0	0	0
**Pseudaminobacter**	1	10	0	0	0	0	0	0	0	0	0	0	0	0	0	0	0	0	0	0
**Pseudanabaena**	1	11	0	0	0	0	0	0	0	0	0	0	0	0	0	0	0	0	0	0
**Pseudoalteromonas**	2	188	0	0	0	0	0	0	0	0	0	0	0	0	0	0	0	0	0	0
**Pseudochrobactrum**	1	7	0	0	0	0	0	0	0	0	0	0	0	0	0	0	0	0	0	0
**Pseudogulbenkiania**	1	40	0	0	0	0	0	0	0	0	0	0	0	0	0	0	0	0	0	0
**pseudomallei**	3	131	0	0	0	0	0	0	0	0	0	0	0	0	0	0	0	0	0	0

Genus	Cluster Number	Protein Counts	K07538	K07540	K07543	K07545	K07546	K07547	K07549	K07823	K07824	K08295	K08686	K08689	K08690	K08710	K09461	K10215	K10216	K10217
Pseudomonas	3	381	0	0	0	0	0	0	0	1	0	0	0	1	1	1	0	0	1	1
Pseudonocardia	3	180	0	0	0	0	0	0	0	0	0	1	0	0	0	0	1	0	0	1
Pseudorhodobacter	1	4	0	0	0	0	0	0	0	0	0	0	0	0	0	0	0	0	0	0
Pseudovibrio	1	33	0	0	0	0	0	0	0	0	0	1	0	0	0	0	1	0	0	0
Pseudoxanthomonas	2	124	0	0	0	0	0	0	0	0	0	0	0	1	1	0	0	0	0	1
Psychrobacter	2	124	0	0	0	0	0	0	0	0	0	0	0	0	0	0	0	0	0	0
Psychroflexus	1	22	0	0	0	0	0	0	0	0	0	0	0	0	0	0	0	0	0	0
Psychromonas	2	110	0	0	0	0	0	0	0	0	0	0	0	0	0	0	0	0	0	0
Pusillimonas	3	111	0	0	0	0	0	0	0	0	0	1	0	0	0	0	1	0	0	1
Pyrobaculum	1	15	0	0	0	0	0	0	0	0	0	0	0	0	0	0	0	0	0	0
Pyrococcus	1	10	0	0	0	0	0	0	0	0	0	0	0	0	0	0	0	0	0	0
Pyrolobus	1	4	0	0	0	0	0	0	0	0	0	0	0	0	0	0	0	0	0	0
Rahnella	2	143	0	0	0	0	0	0	0	0	0	0	0	0	0	0	0	0	0	0
Ralstonia	3	332	0	0	0	0	0	0	0	0	0	1	0	0	0	0	1	0	1	1
Ramlibacter	1	35	0	0	0	0	0	0	0	0	0	1	0	0	0	0	1	0	0	0
Raoultella	2	139	0	0	0	0	0	0	0	0	0	0	0	0	0	0	0	0	0	0
Raphidiopsis	1	5	0	0	0	0	0	0	0	0	0	0	0	0	0	0	0	0	0	0
Reinekea	1	2	0	0	0	0	0	0	0	0	0	0	0	0	0	0	0	0	0	0
Renibacterium	2	67	0	0	0	0	0	0	0	0	0	0	0	0	0	0	0	0	0	0
Rhizobiaceae	1	3	0	0	0	0	0	0	0	0	0	0	0	0	0	0	0	0	0	0
Rhizobiales	1	6	0	0	0	0	0	0	0	0	0	0	0	0	0	0	0	0	0	0
Rhizobium	3	257	0	0	0	0	0	0	0	0	0	1	0	0	0	0	1	0	0	1
Rhizobium/Agrobacterium	2	87	0	0	0	0	0	0	0	0	0	0	0	0	0	0	0	0	0	1
Rhodanobacter	1	21	0	0	0	0	0	0	0	1	0	0	0	0	0	0	0	0	0	1
Rhodobacter	3	175	0	0	0	0	0	0	0	0	0	0	0	0	0	0	0	0	0	0
Rhodobacteraceae	1	21	0	0	0	0	0	0	0	0	0	0	0	0	0	0	0	0	0	0
Rhodobacterales	1	34	0	0	0	0	0	0	0	0	0	0	0	0	0	0	0	0	0	0
Rhodococcus	2	292	0	0	0	0	0	0	0	0	0	0	0	1	1	0	0	0	0	1
Rhodocyclaceae	1	7	0	0	0	0	0	0	0	0	0	0	0	0	0	0	0	0	0	0
Rhodoferax	3	144	0	0	0	0	0	0	0	0	0	0	0	0	0	0	0	0	0	0
Rhodomicrobium	2	103	1	0	0	0	0	0	0	0	0	0	0	0	0	0	0	0	0	0
Rhodopirellula	2	87	0	0	0	0	0	0	0	0	0	0	0	0	0	0	0	0	0	0
Rhodopseudomonas	3	246	0	0	0	0	0	0	0	1	0	1	0	0	0	0	1	0	0	0
Rhodospirillum	2	121	0	0	0	0	0	0	0	0	0	0	0	0	0	0	0	0	0	0
Rhodothermus	2	76	0	0	0	0	0	0	0	0	0	0	0	0	0	0	0	0	0	0
Rhodovulum	1	4	0	0	0	0	0	0	0	0	0	0	0	0	0	0	0	0	0	0
Richelia	1	2	0	0	0	0	0	0	0	0	0	0	0	0	0	0	0	0	0	0
Rickettsia	2	43	0	0	0	0	0	0	0	0	0	0	0	0	0	0	0	0	0	0
Rickettsiaceae	1	5	0	0	0	0	0	0	0	0	0	0	0	0	0	0	0	0	0	0
Riemerella	2	61	0	0	0	0	0	0	0	0	0	0	0	0	0	0	0	0	0	0
Rivularia	1	18	0	0	0	0	0	0	0	0	0	0	0	0	0	0	0	0	0	0
Robiginitalea	2	83	0	0	0	0	0	0	0	0	0	0	0	0	0	0	0	0	0	0
Roseburia	2	58	0	0	0	0	0	0	0	0	0	0	0	0	0	0	0	0	0	0
Roseibium	1	3	0	0	0	0	0	0	0	0	0	0	0	0	0	0	0	0	0	0
Roseiflexus	3	100	0	0	0	0	0	0	0	0	0	0	0	0	0	0	0	0	0	1
Roseobacter	2	149	0	0	0	0	0	0	0	0	0	0	0	0	0	0	0	0	0	0
Roseomonas	1	2	0	0	0	0	0	0	0	0	0	0	0	0	0	0	0	0	0	0
Roseovarius	1	15	0	0	0	0	0	0	0	0	0	0	0	0	0	0	0	0	0	0
Rothia	1	44	0	0	0	0	0	0	0	0	0	0	0	0	0	0	0	0	0	0
Rubrivivax	1	24	0	0	0	0	0	0	0	0	0	0	0	0	0	0	0	0	0	0
Rubrobacter	3	117	0	0	0	0	0	0	0	0	0	1	0	0	0	0	1	0	0	0
Ruegeria	2	189	0	0	0	0	0	0	0	0	0	1	0	0	0	0	1	0	0	0

Genus	Cluster Number	Protein Counts	K07538	K07540	K07543	K07545	K07546	K07547	K07549	K07823	K07824	K08295	K08686	K08689	K08690	K08710	K09461	K10215	K10216	K10217
Ruminococcaceae	1	2	0	0	0	0	0	0	0	0	0	0	0	0	0	0	0	0	0	0
Ruminococcus	2	101	0	0	0	0	0	0	0	0	0	0	0	0	0	0	0	0	0	0
Runella	1	26	0	0	0	0	0	0	0	0	0	0	0	0	0	0	0	0	0	0
Saccharomonospora	2	132	0	0	0	0	0	0	0	0	0	0	0	0	0	0	0	0	0	0
Saccharophagus	2	81	0	0	0	0	0	0	0	0	0	0	0	0	0	0	0	0	0	0
Saccharopolyspora	3	194	0	0	0	0	0	0	0	0	0	1	0	0	0	0	1	0	0	0
Saccharothrix	1	32	0	0	0	0	0	0	0	0	0	1	0	0	0	0	1	0	0	0
Sagittula	1	10	0	0	0	0	0	0	0	0	0	0	0	0	0	0	0	0	0	0
Salinibacter	2	77	0	0	0	0	0	0	0	0	0	0	0	0	0	0	0	0	0	0
Salinimonas	1	1	0	0	0	0	0	0	0	0	0	0	0	0	0	0	0	0	0	0
Salinispora	3	127	0	0	0	0	0	0	0	0	0	0	0	0	0	0	0	0	0	1
Salinivibrio	1	2	0	0	0	0	0	0	0	0	0	0	0	0	0	0	0	0	0	0
Salmonella	2	151	0	0	0	0	0	0	0	0	0	0	0	0	0	0	0	0	0	0
Sanguibacter	2	73	0	0	0	0	0	0	0	0	0	0	0	0	0	0	0	0	0	0
Saprospira	1	13	0	0	0	0	0	0	0	0	0	0	0	0	0	0	0	0	0	0
Scardovia	1	1	0	0	0	0	0	0	0	0	0	0	0	0	0	0	0	0	0	0
Scytonema	1	10	0	0	0	0	0	0	0	0	0	0	0	0	0	0	0	0	0	0
Sebaldella	2	69	0	0	0	0	0	0	0	0	0	0	0	0	0	0	0	0	0	0
Segniliparus	2	85	0	0	0	0	0	0	0	0	0	0	0	0	0	0	0	0	0	0
Selenomonas	2	57	0	0	0	0	0	0	0	0	0	0	0	0	0	0	0	0	0	0
Serinicoccus	1	1	0	0	0	0	0	0	0	0	0	0	0	0	0	0	0	0	0	0
Serratia	2	186	0	0	0	0	0	0	0	0	0	0	0	0	0	0	0	0	0	0
Shewanella	3	214	0	0	0	0	0	0	0	0	0	0	0	0	0	0	0	0	0	1
Shigella	2	172	0	0	0	0	0	0	0	0	0	0	0	0	0	0	0	0	0	0
Shinella	1	2	0	0	0	0	0	0	0	0	0	0	0	0	0	0	0	0	0	0
Sideroxydans	2	81	0	0	0	0	0	0	0	0	0	0	0	0	0	0	0	0	0	0
Silicibacter	2	105	0	0	0	0	0	0	0	0	0	1	0	0	0	0	1	0	0	0
Simiduia	1	18	0	0	0	0	0	0	0	0	0	0	0	0	0	0	0	0	0	0
Simkania	1	6	0	0	0	0	0	0	0	0	0	0	0	0	0	0	0	0	0	0
Simonsiella	1	2	0	0	0	0	0	0	0	0	0	0	0	0	0	0	0	0	0	0
Singulisphaera	1	18	0	0	0	0	0	0	0	0	0	0	0	0	0	0	0	0	0	0
Sinorhizobium	3	207	0	0	0	0	0	0	0	0	0	0	0	0	0	0	0	0	0	0
Slackia	2	46	0	0	0	0	0	0	0	0	0	0	0	0	0	0	0	0	0	0
Smaragdicoccus	1	3	0	0	0	0	0	0	0	0	0	0	0	0	0	0	0	0	0	0
Sodalis	2	71	0	0	0	0	0	0	0	0	0	0	0	0	0	0	0	0	0	0
Solibacillus	1	23	0	0	0	0	0	0	0	0	0	0	0	0	0	0	0	0	0	0
Solitalea	1	13	0	0	0	0	0	0	0	0	0	0	0	0	0	0	0	0	0	0
Sorangium	3	115	0	0	0	0	0	0	0	0	0	0	0	0	0	0	1	0	0	0
Sphaerobacter	2	100	0	0	0	0	0	0	0	0	0	0	0	0	0	0	0	0	0	0
Sphaerochaeta	1	42	0	0	0	0	0	0	0	0	0	0	0	0	0	0	0	0	0	0
Sphingobacterium	3	85	0	0	0	0	0	0	0	0	0	0	0	0	0	0	0	0	0	0
Sphingobium	1	179	0	0	0	0	0	0	0	1	0	0	0	0	0	0	0	0	0	1
Sphingomonadaceae	1	15	0	0	0	0	0	0	0	0	0	0	0	0	0	0	0	0	0	0
Sphingomonas	2	220	0	0	0	0	0	0	0	1	0	0	0	0	0	0	1	0	0	1
Sphingopyxis	3	99	0	0	0	0	0	0	0	0	0	0	0	0	0	0	0	0	0	0
Spirochaeta	2	103	0	0	0	0	0	0	0	0	0	0	0	0	0	0	0	0	0	0
Spiroplasma	1	6	0	0	0	0	0	0	0	0	0	0	0	0	0	0	0	0	0	0
Spirosoma	2	107	0	0	0	0	0	0	0	0	0	0	0	0	0	0	0	0	0	0
Stackebrandtia	2	110	0	0	0	0	0	0	0	0	0	1	0	0	0	0	1	0	0	0
Stanieria	1	17	0	0	0	0	0	0	0	0	0	0	0	0	0	0	0	0	0	0
Staphylococcus	2	108	0	0	0	0	0	0	0	0	0	0	0	0	0	0	0	0	0	0
Staphylothermus	1	12	0	0	0	0	0	0	0	0	0	0	0	0	0	0	0	0	0	0

Genus	Cluster Number	Protein Counts	K07538	K07540	K07543	K07545	K07546	K07547	K07549	K07823	K07824	K08295	K08686	K08689	K08690	K08710	K09461	K10215	K10216	K10217
Stappia	1	2	0	0	0	0	0	0	0	0	0	0	0	0	0	0	0	0	0	0
Starkeya	3	134	0	0	0	0	0	0	0	0	0	0	0	0	0	0	0	0	0	0
Stenotrophomonas	3	104	0	0	0	0	0	0	0	0	0	0	0	0	0	0	0	0	0	0
Stigmatella	3	129	0	0	0	0	0	0	0	1	0	0	0	0	0	0	0	0	0	1
Streptobacillus	1	38	0	0	0	0	0	0	0	0	0	0	0	0	0	0	0	0	0	0
Streptococcus	2	121	0	0	0	0	0	0	0	0	0	0	0	0	0	0	0	0	0	0
Streptomyces	2	253	0	0	0	0	0	0	0	0	0	1	0	0	0	0	1	0	0	1
Streptosporangium	3	148	0	0	0	0	0	0	0	0	0	0	0	0	0	0	0	0	0	1
Strigomonas	1	2	0	0	0	0	0	0	0	0	0	0	0	0	0	0	0	0	0	0
Sulfitobacter	1	10	0	0	0	0	0	0	0	0	0	0	0	0	0	0	0	0	0	0
Sulfobacillus	1	30	0	0	0	0	0	0	0	0	0	0	0	0	0	0	0	0	0	1
Sulfolobus	1	23	0	0	0	0	0	0	0	0	0	0	0	0	0	0	0	0	0	0
Sulfuricurvum	2	67	0	0	0	0	0	0	0	0	0	0	0	0	0	0	0	0	0	0
Sulfurihydrogenibium	2	55	0	0	0	0	0	0	0	0	0	0	0	0	0	0	0	0	0	0
Sulfurimonas	2	60	0	0	0	0	0	0	0	0	0	0	0	0	0	0	0	0	0	0
Sulfurospirillum	1	48	0	0	0	0	0	0	0	0	0	0	0	0	0	0	0	0	0	0
Sulfurovum	2	48	0	0	0	0	0	0	0	0	0	0	0	0	0	0	0	0	0	0
Symbiobacterium	2	68	0	0	0	0	0	0	0	0	0	0	0	0	0	0	0	0	0	0
Synechococcus	1	34	0	0	0	0	0	0	0	0	0	0	0	0	0	0	0	0	0	0
Synechocystis	1	40	0	0	0	0	0	0	0	0	0	0	0	0	0	0	0	0	0	0
Synergistetes	1	27	0	0	0	0	0	0	0	0	0	0	0	0	0	0	0	0	0	0
Syntrophobacter	2	101	0	0	0	0	0	0	0	0	0	0	0	0	0	0	0	0	0	0
Syntrophobotulus	2	71	0	0	0	0	0	0	0	0	0	0	0	0	0	0	0	0	0	0
Syntrophomonas	2	58	0	0	0	0	0	0	0	0	0	0	0	0	0	0	0	0	0	0
Syntrophothermus	2	60	0	0	0	0	0	0	0	0	0	0	0	0	0	0	0	0	0	0
Syntrophus	2	66	1	0	0	0	0	0	0	0	0	1	0	0	0	0	0	0	0	0
Tannerella	1	8	0	0	0	0	0	0	0	0	0	0	0	0	0	0	0	0	0	0
Taylorella	1	43	0	0	0	0	0	0	0	0	0	0	0	0	0	0	0	0	0	0
Tepidanaerobacter	2	55	0	0	0	0	0	0	0	0	0	0	0	0	0	0	0	0	0	0
Teredinibacter	2	109	0	0	0	0	0	0	0	0	0	0	0	0	0	0	0	0	0	0
Terrabacter	1	1	0	0	0	0	0	0	0	0	0	0	0	0	0	0	0	0	0	0
Terriglobus	2	83	0	0	0	0	0	0	0	0	0	0	0	0	0	0	0	0	0	0
Tetragenococcus	1	12	0	0	0	0	0	0	0	0	0	0	0	0	0	0	0	0	0	0
Thalassiobium	1	11	0	0	0	0	0	0	0	0	0	0	0	0	0	0	0	0	0	0
Thalassobacter	1	9	0	0	0	0	0	0	0	0	0	0	0	0	0	0	0	0	0	0
Thalassolituus	1	12	0	0	0	0	0	0	0	0	0	0	0	0	0	0	0	0	0	0
Thalassospira	1	2	0	0	0	0	0	0	0	0	0	0	0	0	0	0	0	0	0	0
Thauera	1	111	1	0	0	0	0	0	0	0	0	0	0	0	0	0	0	0	1	1
Thermacetogenium	1	7	0	0	0	0	0	0	0	0	0	0	0	0	0	0	0	0	0	0
Thermaerobacter	2	79	0	0	0	0	0	0	0	0	0	0	0	0	0	0	0	0	0	1
Thermanaerovibrio	2	58	0	0	0	0	0	0	0	0	0	0	0	0	0	0	0	0	0	0
Thermincola	2	62	0	0	0	0	0	0	0	0	0	0	0	0	0	0	0	0	0	0
Thermoanaerobacter	2	76	0	0	0	0	0	0	0	0	0	0	0	0	0	0	0	0	0	0
Thermoanaerobacterium	2	64	0	0	0	0	0	0	0	0	0	0	0	0	0	0	0	0	0	0
Thermobacillus	1	11	0	0	0	0	0	0	0	0	0	0	0	0	0	0	0	0	0	0
Thermobaculum	2	62	0	0	0	0	0	0	0	0	0	0	0	0	0	0	0	0	0	0
Thermobifida	2	72	0	0	0	0	0	0	0	0	0	0	0	0	0	0	0	0	0	0
Thermobispora	2	109	0	0	0	0	0	0	0	0	0	0	0	0	0	0	0	0	0	0
Thermococcus	1	13	0	0	0	0	0	0	0	0	0	0	0	0	0	0	0	0	0	0
Thermocrinis	2	60	0	0	0	0	0	0	0	0	0	0	0	0	0	0	0	0	0	0
Thermodesulfatator	1	10	0	0	0	0	0	0	0	0	0	0	0	0	0	0	0	0	0	0
Thermodesulfobacterium	1	9	0	0	0	0	0	0	0	0	0	0	0	0	0	0	0	0	0	0

Genus	Cluster Number	Protein Counts	K07538	K07540	K07543	K07545	K07546	K07547	K07549	K07823	K07824	K08295	K08686	K08689	K08690	K08710	K09461	K10215	K10216	K10217
Thermodesulfobium	1	13	0	0	0	0	0	0	0	0	0	0	0	0	0	0	0	0	0	0
Thermodesulfovibrio	2	60	0	0	0	0	0	0	0	0	0	0	0	0	0	0	0	0	0	0
Thermofilum	1	9	0	0	0	0	0	0	0	0	0	0	0	0	0	0	0	0	0	0
Thermogladius	1	7	0	0	0	0	0	0	0	0	0	0	0	0	0	0	0	0	0	0
Thermomicrobium	2	92	0	0	0	0	0	0	0	0	0	0	0	0	0	0	0	0	0	0
Thermomonospora	2	125	0	0	0	0	0	0	0	0	0	0	0	0	0	0	0	0	0	1
Thermoplasma	1	11	0	0	0	0	0	0	0	0	0	0	0	0	0	0	0	0	0	0
Thermoplasmatales	1	6	0	0	0	0	0	0	0	0	0	0	0	0	0	0	0	0	0	0
Thermoproteus	1	12	0	0	0	0	0	0	0	0	0	0	0	0	0	0	0	0	0	0
Thermosediminibacter	2	64	0	0	0	0	0	0	0	0	0	0	0	0	0	0	0	0	0	0
Thermosipho	2	70	0	0	0	0	0	0	0	0	0	0	0	0	0	0	0	0	0	0
Thermosphaera	1	10	0	0	0	0	0	0	0	0	0	0	0	0	0	0	0	0	0	0
Thermosynechococcus	1	6	0	0	0	0	0	0	0	0	0	0	0	0	0	0	0	0	0	0
Thermotoga	2	80	0	0	0	0	0	0	0	0	0	0	0	0	0	0	0	0	0	0
Thermovibrio	2	58	0	0	0	0	0	0	0	0	0	0	0	0	0	0	0	0	0	0
Thermovirga	1	9	0	0	0	0	0	0	0	0	0	0	0	0	0	0	0	0	0	0
Thermus	2	107	0	0	0	0	0	0	0	0	0	0	0	0	0	0	0	0	0	1
Thioalkalimicrobium	1	4	0	0	0	0	0	0	0	0	0	0	0	0	0	0	0	0	0	0
Thioalkalivibrio	2	91	0	0	0	0	0	0	0	0	0	0	0	0	0	0	0	0	0	0
Thiobaca	1	2	0	0	0	0	0	0	0	0	0	0	0	0	0	0	0	0	0	0
Thiobacillus	2	67	0	0	0	0	0	0	0	0	0	0	0	0	0	0	0	0	0	0
Thiocapsa	1	2	0	0	0	0	0	0	0	0	0	0	0	0	0	0	0	0	0	0
Thiocystis	1	11	0	0	0	0	0	0	0	0	0	0	0	0	0	0	0	0	0	0
Thioflavicoccus	1	13	0	0	0	0	0	0	0	0	0	0	0	0	0	0	0	0	0	0
Thiomicrospira	2	50	0	0	0	0	0	0	0	0	0	0	0	0	0	0	0	0	0	0
Thiomonas	2	85	0	0	0	0	0	0	0	0	0	0	0	0	0	0	0	0	0	0
Thiorhodococcus	1	2	0	0	0	0	0	0	0	0	0	0	0	0	0	0	0	0	0	0
Thiothrix	1	2	0	0	0	0	0	0	0	0	0	0	0	0	0	0	0	0	0	0
Tistrella	1	42	0	0	0	0	0	0	0	0	0	0	0	0	0	0	0	0	0	1
Tolumonas	2	100	0	0	0	0	0	0	0	0	0	0	0	0	0	0	0	0	0	0
Transposon	1	2	0	0	0	0	0	0	0	0	0	0	0	0	0	0	0	0	0	0
Treponema	1	63	0	0	0	0	0	0	0	0	0	0	0	0	0	0	0	0	0	0
Trichodesmium	2	71	0	0	0	0	0	0	0	0	0	0	0	0	0	0	0	0	0	0
Trichormus	1	12	0	0	0	0	0	0	0	0	0	0	0	0	0	0	0	0	0	0
Tropheryma	2	22	0	0	0	0	0	0	0	0	0	0	0	0	0	0	0	0	0	0
Truepera	2	104	0	0	0	0	0	0	0	0	0	0	0	0	0	0	0	0	0	0
Tsukamurella	2	123	0	0	0	0	0	0	0	0	0	0	0	0	0	0	0	0	0	0
Turneriella	1	16	0	0	0	0	0	0	0	0	0	0	0	0	0	0	0	0	0	0
Uliginosibacterium	1	2	0	0	0	0	0	0	0	0	0	0	0	0	0	0	0	0	0	0
Ureaplasma	1	24	0	0	0	0	0	0	0	0	0	0	0	0	0	0	0	0	0	0
Ureibacillus	1	2	0	0	0	0	0	0	0	0	0	0	0	0	0	0	0	0	0	0
Variovorax	3	187	0	0	0	0	1	0	0	0	0	1	0	0	0	0	1	0	0	0
Veillonella	1	54	0	0	0	0	0	0	0	0	0	0	0	0	0	0	0	0	0	0
Verminephrobacter	3	188	0	0	1	1	1	0	0	0	0	0	0	0	0	1	1	0	1	1
Verrucosispora	3	126	0	0	0	0	0	0	0	0	0	0	0	0	0	0	0	0	0	1
Vibrio	2	195	0	0	0	0	0	0	0	0	0	0	0	0	0	0	0	0	0	0
Vibrionales	1	46	0	0	0	0	0	0	0	0	0	0	0	0	0	0	0	0	0	0
Vitreoscilla	1	1	0	0	0	0	0	0	0	0	0	0	0	0	0	0	0	0	0	0
Vulcanisaeta	1	14	0	0	0	0	0	0	0	0	0	0	0	0	0	0	0	0	0	0
Waddlia	2	68	0	0	0	0	0	0	0	0	0	0	0	0	0	0	0	0	0	0
Wautersia	1	2	0	0	0	0	0	0	0	0	0	0	0	0	0	0	0	0	0	0
Weeksella	2	65	0	0	0	0	0	0	0	0	0	0	0	0	0	0	0	0	0	0

Genus	Cluster Number	Protein Counts	K07538	K07540	K07543	K07545	K07546	K07547	K07549	K07823	K07824	K08295	K08686	K08689	K08690	K08710	K09461	K10215	K10216	K10217
**Weissella**	1	14	0	0	0	0	0	0	0	0	0	0	0	0	0	0	0	0	0	0
**Wenxinia**	1	2	0	0	0	0	0	0	0	0	0	0	0	0	0	0	0	0	0	0
**Wigglesworthia**	1	29	0	0	0	0	0	0	0	0	0	0	0	0	0	0	0	0	0	0
**Wolbachia**	1	32	0	0	0	0	0	0	0	0	0	0	0	0	0	0	0	0	0	0
**Wolinella**	2	51	0	0	0	0	0	0	0	0	0	0	0	0	0	0	0	0	0	0
**Xanthobacter**	3	176	0	0	0	0	0	0	0	0	0	0	0	0	0	0	0	0	0	0
**Xanthobacteraceae**	1	4	0	0	0	0	0	0	0	0	0	0	0	0	0	0	0	0	0	0
**Xanthomonadaceae**	1	39	0	0	0	0	0	0	0	0	0	0	0	0	0	0	0	0	0	0
**Xanthomonas**	2	162	0	0	0	0	0	0	0	1	0	0	0	0	0	0	0	0	0	0
**Xenorhabdus**	2	127	0	0	0	0	0	0	0	0	0	0	0	0	0	0	0	0	0	0
**Xylanimonas**	2	66	0	0	0	0	0	0	0	0	0	0	0	0	0	0	1	0	0	0
**Xylella**	2	52	0	0	0	0	0	0	0	0	0	0	0	0	0	0	0	0	0	0
**Yersinia**	2	145	0	0	0	0	0	0	0	0	0	0	0	0	0	0	0	0	0	0
**Yokenella**	2	61	0	0	0	0	0	0	0	0	0	0	0	0	0	0	0	0	0	0
**Zobellia**	1	28	0	0	0	0	0	0	0	0	0	0	0	0	0	0	0	0	0	0
**Zunongwangia**	2	98	0	0	0	0	0	0	0	0	0	0	0	0	0	0	0	0	0	0
**Zymomonas**	2	67	0	0	0	0	0	0	0	0	0	0	0	0	0	0	0	0	0	0
**Zymophilus**	1	2	0	0	0	0	0	0	0	0	0	0	0	0	0	0	0	0	0	0

Genus	Cluster Number	Protein Counts	K10218	K10219	K10220	K10221	K10222	K10616	K10617	K10618	K10619	K10621	K10622	K10623	K10676	K10700	K10701	K10702	K11180	K11946
Acaricomes	1	2	0	0	0	0	0	0	0	0	0	0	0	0	0	0	0	0	0	0
Acaryochloris	2	88	0	0	0	0	0	0	0	0	0	0	0	0	0	0	0	0	0	0
Acetivibrio	1	2	0	0	0	0	0	0	0	0	0	0	0	0	0	0	0	0	0	0
Acetobacter	2	85	0	0	0	0	0	0	0	0	0	0	0	0	0	0	0	0	0	0
Acetobacteraceae	1	2	0	0	0	0	0	0	0	0	0	0	0	0	0	0	0	0	0	0
Acetobacterium	1	8	0	0	0	0	0	0	0	0	0	0	0	0	0	0	0	0	0	0
Acetohalobium	2	79	0	0	0	0	0	0	0	0	0	0	0	0	0	0	0	0	0	0
Acholeplasma	2	38	0	0	0	0	0	0	0	0	0	0	0	0	0	0	0	0	0	0
Achromobacter	2	192	0	0	0	0	1	0	0	0	0	1	1	0	1	0	0	0	0	0
Acidaminococcus	1	65	0	0	0	0	0	0	0	0	0	0	0	0	0	0	0	0	0	0
Acidianus	1	9	0	0	0	0	0	0	0	0	0	0	0	0	0	0	0	0	0	0
Acidilobus	1	8	0	0	0	0	0	0	0	0	0	0	0	0	0	0	0	0	0	0
Acidimicrobium	2	58	0	0	0	0	0	0	0	0	0	0	0	0	0	0	0	0	0	0
Acidiphilium	3	144	0	0	1	0	0	0	0	0	0	0	0	0	0	0	0	0	0	0
Acidithiobacillus	2	70	0	0	0	0	0	0	0	0	0	0	0	0	0	0	0	0	0	0
Acidobacterium	2	91	0	0	0	0	0	0	0	0	0	0	0	0	0	0	0	0	0	0
Acidocella	1	2	0	0	0	0	0	0	0	0	0	0	0	0	0	0	0	0	0	0
Acidothermus	2	68	0	0	0	0	0	0	0	0	0	0	0	0	0	0	0	0	0	0
Acidovorax	3	217	1	1	1	1	1	0	0	0	0	0	0	0	0	0	0	0	0	0
Aciduliprofundum	1	11	0	0	0	0	0	0	0	0	0	0	0	0	0	0	0	0	0	0
Acinetobacter	3	188	0	0	0	0	0	0	0	0	0	0	0	0	1	0	0	0	0	0
Actinobacillus	2	112	0	0	0	0	0	0	0	0	0	0	0	0	0	0	0	0	0	0
Actinomadura	1	1	0	0	0	0	0	0	0	0	0	0	0	0	0	0	0	0	0	0
Actinoplanes	1	52	1	0	1	0	0	0	0	0	0	0	0	0	0	0	0	0	0	0
Actinopolyspora	1	2	0	0	0	0	0	0	0	0	0	0	0	0	0	0	0	0	0	0
Actinosynnema	2	122	0	0	0	0	0	0	0	0	0	0	0	0	0	0	0	0	0	0
Advenella	1	30	0	0	1	0	0	0	0	0	0	0	0	0	0	0	0	0	0	0
Aequorivita	1	12	0	0	0	0	0	0	0	0	0	0	0	0	0	0	0	0	0	0
Aerococcus	2	57	0	0	0	0	0	0	0	0	0	0	0	0	0	0	0	0	0	0
Aeromonas	2	127	0	0	0	0	0	0	0	0	0	0	0	0	0	0	0	0	0	0
Aeropyrum	1	13	0	0	0	0	0	0	0	0	0	0	0	0	0	0	0	0	0	0
Afipia	1	54	0	0	0	0	0	0	0	0	0	0	0	0	0	0	0	0	0	0
Aggregatibacter	1	81	0	0	0	0	0	0	0	0	0	0	0	0	0	0	0	0	0	0
Agrobacterium	2	242	1	0	0	1	0	0	0	0	0	1	1	0	1	0	0	0	0	0
Ahrensia	1	2	0	0	0	0	0	0	0	0	0	0	0	0	0	0	0	0	0	0
Akkermansia	2	47	0	0	0	0	0	0	0	0	0	0	0	0	0	0	0	0	0	0
Albidiferax	3	144	1	1	1	1	0	0	0	0	0	0	0	0	0	0	0	0	0	0
Alcaligenes	1	21	1	0	0	0	0	0	0	0	0	0	0	0	1	0	0	0	0	0
Alcanivorax	2	146	0	0	0	0	0	0	0	0	0	0	0	0	0	0	0	0	0	0
Alicycliphilus	3	152	1	0	0	1	0	0	0	0	0	0	0	0	1	0	0	0	0	0
Alicyclobacillus	2	91	1	0	0	0	0	0	0	0	0	0	0	0	0	0	0	0	0	0
Aliivibrio	2	118	0	0	0	0	0	0	0	0	0	0	0	0	0	0	0	0	0	0
Alishewanella	1	1	0	0	0	0	0	0	0	0	0	0	0	0	0	0	0	0	0	0
Alistipes	1	52	0	0	0	0	0	0	0	0	0	0	0	0	0	0	0	0	0	0
Alkalilimnicola	2	97	0	0	0	0	0	0	0	0	0	0	0	0	0	0	0	0	1	0
Alkaliphilus	2	62	0	0	0	0	0	0	0	0	0	0	0	0	0	0	0	0	0	0
Allochromatium	2	84	0	0	0	0	0	0	0	0	0	0	0	0	0	0	0	0	1	0
Alphaproteobacteria	1	3	0	0	0	0	0	0	0	0	0	0	0	0	0	0	0	0	0	0
Alteromonadales	1	12	0	0	0	0	0	0	0	0	0	0	0	0	0	0	0	0	0	0
Alteromonas	1	37	0	0	0	0	0	0	0	0	0	0	0	0	0	0	0	0	0	0
Aminobacter	1	4	0	0	0	0	0	0	0	0	0	0	0	0	0	0	0	0	0	0
Aminobacterium	2	59	0	0	0	0	0	0	0	0	0	0	0	0	0	0	0	0	0	0

Genus	Cluster Number	Protein Counts	K10218	K10219	K10220	K10221	K10222	K10616	K10617	K10618	K10619	K10621	K10622	K10623	K10676	K10700	K10701	K10702	K11180	K11946
**Ammonifex**	2	50	0	0	0	0	0	0	0	0	0	0	0	0	0	0	0	0	1	0
**Amphibacillus**	1	13	0	0	0	0	0	0	0	0	0	0	0	0	0	0	0	0	0	0
**Amphritea**	1	2	0	0	0	0	0	0	0	0	0	0	0	0	0	0	0	0	0	0
**Amycolatopsis**	2	201	0	0	0	0	0	0	0	0	0	0	0	0	1	0	0	0	0	0
**Amycolicicoccus**	1	43	0	0	0	0	0	0	0	0	0	0	0	0	0	0	0	0	0	0
**Anabaena**	2	92	0	0	0	0	0	0	0	0	0	0	0	0	0	0	0	0	0	0
**Anaerobaculum**	1	11	0	0	0	0	0	0	0	0	0	0	0	0	0	0	0	0	0	0
**Anaerococcus**	2	46	0	0	0	0	0	0	0	0	0	0	0	0	0	0	0	0	0	0
**Anaerolinea**	2	66	0	0	0	0	0	0	0	0	0	0	0	0	0	0	0	0	0	0
**Anaeromyxobacter**	3	117	0	0	0	0	0	0	0	0	0	0	0	0	0	0	0	0	0	0
**Anaerostipes**	1	44	0	0	0	0	0	0	0	0	0	0	0	0	0	0	0	0	0	0
**Anaerotruncus**	1	2	0	0	0	0	0	0	0	0	0	0	0	0	0	0	0	0	0	0
**Anaplasma**	1	35	0	0	0	0	0	0	0	0	0	0	0	0	0	0	0	0	0	0
**Ancylobacter**	1	3	0	0	0	0	0	0	0	0	0	0	0	0	0	0	0	0	0	0
**Aneurinibacillus**	1	1	0	0	0	0	0	0	0	0	0	0	0	0	0	0	0	0	0	0
**Angomonas**	1	1	0	0	0	0	0	0	0	0	0	0	0	0	0	0	0	0	0	0
**Anoxybacillus**	2	72	0	0	0	0	0	0	0	0	0	0	0	0	0	0	0	0	0	0
**Aquifex**	2	45	0	0	0	0	0	0	0	0	0	0	0	0	0	0	0	0	0	0
**Aquimarina**	1	2	0	0	0	0	0	0	0	0	0	0	0	0	0	0	0	0	0	0
**Arcanobacterium**	2	45	0	0	0	0	0	0	0	0	0	0	0	0	0	0	0	0	0	0
**Archaeoglobus**	1	14	0	0	0	0	0	0	0	0	0	0	0	0	0	0	0	0	1	0
**Arcobacter**	2	101	0	0	0	0	0	0	0	0	0	0	0	0	0	0	0	0	0	0
**Aromatoleum**	3	133	0	0	0	0	0	0	0	0	0	0	0	0	0	1	1	0	0	0
**Arsenophonus**	1	4	0	0	0	0	0	0	0	0	0	0	0	0	0	0	0	0	0	0
**Arthrobacter**	2	206	1	1	1	1	0	0	0	0	0	0	0	0	1	0	0	0	0	0
**Arthrospira**	2	67	0	0	0	0	0	0	0	0	0	0	0	0	0	0	0	0	0	0
**Aster**	1	10	0	0	0	0	0	0	0	0	0	0	0	0	0	0	0	0	0	0
**Asticcacaulis**	2	103	1	0	1	0	0	0	0	0	0	0	0	0	0	0	0	0	0	0
**Atopobium**	1	28	0	0	0	0	0	0	0	0	0	0	0	0	0	0	0	0	0	0
**Aurantimonas**	1	2	0	0	0	0	0	0	0	0	0	0	0	0	0	0	0	0	0	0
**Avibacterium**	1	17	0	0	0	0	0	0	0	0	0	0	0	0	0	0	0	0	0	0
**Azoarcus**	3	172	1	1	1	1	0	0	0	0	0	0	0	0	0	0	0	0	0	0
**Azorhizobium**	3	149	1	0	0	0	0	0	0	0	0	0	0	0	0	0	0	0	0	0
**Azorhizophilus**	1	4	0	0	0	0	0	0	0	0	0	0	0	0	0	0	0	0	0	0
**Azospira**	1	19	0	0	0	0	0	0	0	0	0	0	0	0	0	0	0	0	0	0
**Azospirillum**	2	198	1	1	0	1	0	0	0	0	0	0	0	0	0	0	0	0	0	0
**Azotobacter**	3	183	1	0	0	0	0	0	0	0	0	0	0	0	0	0	0	0	0	0
**Bacillales**	1	9	0	0	0	0	0	0	0	0	0	0	0	0	0	0	0	0	0	0
**Bacilli**	1	3	0	0	0	0	0	0	0	0	0	0	0	0	0	0	0	0	0	0
**Bacillus**	2	237	0	0	0	0	0	0	0	0	0	0	0	0	0	0	0	0	0	0
**Bacteria**	2	86	0	0	0	0	0	0	0	0	0	0	0	0	0	0	0	0	0	0
**Bacteriovorax**	2	98	0	0	0	0	0	0	0	0	0	0	0	0	0	0	0	0	0	0
**Bacteroidales**	2	59	0	0	0	0	0	0	0	0	0	0	0	0	0	0	0	0	0	0
**Bacteroides**	2	96	0	0	0	0	0	0	0	0	0	0	0	0	0	0	0	0	0	0
**Bacteroidetes**	1	2	0	0	0	0	0	0	0	0	0	0	0	0	0	0	0	0	0	0
**Bartonella**	2	50	0	0	0	0	0	0	0	0	0	0	0	0	0	0	0	0	0	0
**Baumannia**	2	24	0	0	0	0	0	0	0	0	0	0	0	0	0	0	0	0	0	0
**Bdellovibrio**	2	87	0	0	0	0	0	0	0	0	0	0	0	0	0	0	0	0	0	0
**Beijerinckia**	3	123	0	0	0	0	0	0	0	0	0	0	0	0	0	0	0	0	0	0
**Belliella**	1	14	0	0	0	0	0	0	0	0	0	0	0	0	0	0	0	0	0	0
**Beutenbergia**	2	82	0	0	0	0	0	0	0	0	0	0	0	0	0	0	0	0	0	0
**Bifidobacterium**	2	61	0	0	0	0	0	0	0	0	0	0	0	0	0	0	0	0	0	0

Genus	Cluster Number	Protein Counts	K10218	K10219	K10220	K10221	K10222	K10616	K10617	K10618	K10619	K10621	K10622	K10623	K10676	K10700	K10701	K10702	K11180	K11946
Bizionia	1	1	0	0	0	0	0	0	0	0	0	0	0	0	0	0	0	0	0	0
Blastococcus	1	39	1	0	1	0	0	0	0	0	0	0	0	0	0	0	0	0	0	0
Blattabacterium	1	23	0	0	0	0	0	0	0	0	0	0	0	0	0	0	0	0	0	0
Blautia	1	22	0	0	0	0	0	0	0	0	0	0	0	0	0	0	0	0	0	0
Bordetella	3	227	1	0	0	0	0	0	0	0	0	0	0	0	1	0	0	0	0	0
Borrelia	1	32	0	0	0	0	0	0	0	0	0	0	0	0	0	0	0	0	0	0
Brachybacterium	1	18	0	0	0	0	0	0	0	0	0	0	0	0	0	0	0	0	0	0
Brachymonas	1	4	0	0	0	0	0	0	0	0	0	0	0	0	0	0	0	0	0	0
Brachyspira	2	79	0	0	0	0	0	0	0	0	0	0	0	0	0	0	0	0	0	0
Bradyrhizobiaceae	1	20	0	0	0	0	0	0	0	0	0	0	0	0	0	0	0	0	0	0
Bradyrhizobium	3	254	1	1	1	1	0	0	0	0	0	0	1	0	1	0	0	0	0	0
Brenneria	1	20	0	0	0	0	0	0	0	0	0	0	0	0	0	0	0	0	0	0
Brevibacillus	2	122	0	0	0	0	0	0	0	0	0	0	0	0	0	0	0	0	0	0
Brevibacterium	1	4	0	0	0	0	0	0	0	0	0	0	0	0	0	0	0	0	0	0
Brevundimonas	2	105	1	1	1	1	0	0	0	0	0	0	0	0	0	0	0	0	0	0
Brucella	3	138	0	0	0	0	0	0	0	0	0	0	0	0	0	0	0	0	0	0
Buchnera	1	26	0	0	0	0	0	0	0	0	0	0	0	0	0	0	0	0	0	0
Burkholderia	3	424	1	1	1	1	1	1	1	1	1	1	1	1	1	0	0	0	0	0
Burkholderiaceae	3	143	0	0	0	0	0	0	0	0	0	0	0	0	0	0	0	0	0	0
Burkholderiales	1	24	0	0	0	0	0	0	0	0	0	0	0	0	0	0	0	0	0	0
Buttiauxella	1	2	0	0	0	0	0	0	0	0	0	0	0	0	0	0	0	0	0	0
Butyrivibrio	2	67	0	0	0	0	0	0	0	0	0	0	0	0	0	0	0	0	0	0
Caldanaerobacter	2	63	0	0	0	0	0	0	0	0	0	0	0	0	0	0	0	0	0	0
Caldicellulosiruptor	2	75	0	0	0	0	0	0	0	0	0	0	0	0	0	0	0	0	0	0
Caldilinea	1	23	0	0	0	0	0	0	0	0	0	0	0	0	0	0	0	0	0	0
Caldimonas	1	6	0	0	0	0	0	0	0	0	0	0	0	0	0	0	0	0	0	0
Caldisericum	1	10	0	0	0	0	0	0	0	0	0	0	0	0	0	0	0	0	0	0
Caldisphaera	1	7	0	0	0	0	0	0	0	0	0	0	0	0	0	0	0	0	0	0
Calditerrivibrio	2	74	0	0	0	0	0	0	0	0	0	0	0	0	0	0	0	0	0	0
Caldivirga	1	13	0	0	0	0	0	0	0	0	0	0	0	0	0	0	0	0	1	0
Calothrix	1	43	0	0	0	0	0	0	0	0	0	0	0	0	0	0	0	0	0	0
Calyptogena	2	34	0	0	0	0	0	0	0	0	0	0	0	0	0	0	0	0	1	0
Campylobacter	2	80	0	0	0	0	0	0	0	0	0	0	0	0	0	0	0	0	0	0
Candidatus	2	290	1	1	1	0	0	0	0	0	0	0	0	0	0	0	0	0	1	0
Capnocytophaga	1	50	0	0	0	0	0	0	0	0	0	0	0	0	0	0	0	0	0	0
Carbophilus	1	2	0	0	0	0	0	0	0	0	0	0	0	0	0	0	0	0	0	0
Carboxydibrachium	1	14	0	0	0	0	0	0	0	0	0	0	0	0	0	0	0	0	0	0
Carboxydothermus	2	68	0	0	0	0	0	0	0	0	0	0	0	0	0	0	0	0	1	0
Carica	1	2	0	0	0	0	0	0	0	0	0	0	0	0	0	0	0	0	0	0
Carnobacterium	2	71	0	0	0	0	0	0	0	0	0	0	0	0	0	0	0	0	0	0
Catenibacterium	1	2	0	0	0	0	0	0	0	0	0	0	0	0	0	0	0	0	0	0
Catenovulum	1	2	0	0	0	0	0	0	0	0	0	0	0	0	0	0	0	0	0	0
Catenulispora	3	159	0	0	1	0	0	0	0	0	0	0	0	0	0	0	0	0	0	0
Caulobacter	3	168	0	0	0	0	0	0	0	0	0	0	0	0	0	0	0	0	0	0
Cedecea	1	45	0	0	0	0	0	0	0	0	0	0	0	0	0	0	0	0	0	0
Cellulomonas	2	96	0	0	0	0	0	0	0	0	0	0	0	0	0	0	0	0	0	0
Cellulophaga	2	105	0	0	0	0	0	0	0	0	0	0	0	0	0	0	0	0	0	0
Cellulosilyticum	1	14	0	0	0	0	0	0	0	0	0	0	0	0	0	0	0	0	0	0
Cellvibrio	2	85	0	0	0	0	0	0	0	0	0	0	0	0	0	0	0	0	0	0
Cenarchaeum	1	7	0	0	0	0	0	0	0	0	0	0	0	0	0	0	0	0	0	0
Chamaesiphon	1	13	0	0	0	0	0	0	0	0	0	0	0	0	0	0	0	0	0	0
Chelativorans	3	136	0	0	0	0	0	0	0	0	0	0	0	0	0	0	0	0	0	0

Genus	Cluster Number	Protein Counts	K10218	K10219	K10220	K10221	K10222	K10616	K10617	K10618	K10619	K10621	K10622	K10623	K10676	K10700	K10701	K10702	K11180	K11946
Chelatococcus	1	2	0	0	0	0	0	0	0	0	0	0	0	0	0	0	0	0	0	0
Chitinophaga	2	98	0	0	0	0	0	0	0	0	0	0	0	0	0	0	0	0	0	0
Chlamydia	1	36	0	0	0	0	0	0	0	0	0	0	0	0	0	0	0	0	0	0
Chlamydomonas	1	3	0	0	0	0	0	0	0	0	0	0	0	0	0	0	0	0	0	0
Chlamydophila	1	31	0	0	0	0	0	0	0	0	0	0	0	0	0	0	0	0	0	0
Chlorobaculum	2	61	0	0	0	0	0	0	0	0	0	0	0	0	0	0	0	0	1	0
Chlorobium	2	89	0	0	0	0	0	0	0	0	0	0	0	0	0	0	0	0	1	0
Chlorobium/Pelodictyon	1	2	0	0	0	0	0	0	0	0	0	0	0	0	0	0	0	0	0	0
Chloroflexus	2	105	0	0	0	0	0	0	0	0	0	0	0	0	0	0	0	0	0	0
Chlorogloeopsis	1	9	0	0	0	0	0	0	0	0	0	0	0	0	0	0	0	0	0	0
Chloroherpeton	2	59	0	0	0	0	0	0	0	0	0	0	0	0	0	0	0	0	0	0
Chromobacterium	2	117	0	0	0	0	0	0	0	0	0	0	0	0	0	0	0	0	0	0
Chromohalobacter	2	145	1	1	0	0	0	0	0	0	0	0	0	0	0	0	0	0	0	0
Chroococcidiopsis	1	24	0	0	0	0	0	0	0	0	0	0	0	0	0	0	0	0	0	0
Chryseobacterium	2	104	1	1	1	1	0	0	0	0	0	0	0	0	0	0	0	0	0	0
Citreicella	1	13	0	0	0	0	0	0	0	0	0	0	0	0	0	0	0	0	0	0
Citrobacter	2	190	1	0	0	0	0	0	0	0	0	0	0	0	0	0	0	0	0	0
Clavibacter	2	83	0	0	0	0	0	0	0	0	0	0	0	0	0	0	0	0	0	0
Clonorchis	1	1	0	0	0	0	0	0	0	0	0	0	0	0	0	0	0	0	0	0
Clostridiales	2	77	0	0	0	0	0	0	0	0	0	0	0	0	0	0	0	0	0	0
Clostridium	2	191	0	0	0	0	0	0	0	0	0	0	0	0	0	0	0	0	0	0
Cohnella	1	2	0	0	0	0	0	0	0	0	0	0	0	0	0	0	0	0	0	0
Coleofasciculus	1	4	0	0	0	0	0	0	0	0	0	0	0	0	0	0	0	0	0	0
Collimonas	1	35	0	0	0	0	0	0	0	0	0	0	0	0	0	0	0	0	0	0
Colwellia	2	130	0	0	0	0	0	0	0	0	0	0	0	0	0	0	0	0	0	0
Comamonas	2	196	1	1	1	1	1	0	0	0	0	0	0	0	0	0	0	0	0	0
Conexibacter	3	117	1	0	0	0	0	0	0	0	0	0	1	0	0	0	0	0	0	0
Coprobacillus	1	2	0	0	0	0	0	0	0	0	0	0	0	0	0	0	0	0	0	0
Coprococcus	2	76	0	0	0	0	0	0	0	0	0	0	0	0	0	0	0	0	0	0
Coprothermobacter	2	45	0	0	0	0	0	0	0	0	0	0	0	0	0	0	0	0	0	0
Coraliomargarita	2	68	0	0	0	0	0	0	0	0	0	0	0	0	0	0	0	0	0	0
Corallococcus	1	29	0	0	0	0	0	0	0	0	0	0	0	0	0	0	0	0	0	0
Coriobacterium	2	36	0	0	0	0	0	0	0	0	0	0	0	0	0	0	0	0	0	0
Corynebacterineae	1	5	0	0	0	0	0	0	0	0	0	0	0	0	0	0	0	0	0	0
Corynebacterium	2	175	0	0	0	0	0	0	0	0	0	0	0	0	1	0	0	0	0	0
Coxiella	2	54	0	0	0	0	0	0	0	0	0	0	0	0	0	0	0	0	0	0
Crinalium	1	21	0	0	0	0	0	0	0	0	0	0	0	0	0	0	0	0	0	0
Croceibacter	2	80	0	0	0	0	0	0	0	0	0	0	0	0	0	0	0	0	0	0
Crocosphaera	1	6	0	0	0	0	0	0	0	0	0	0	0	0	0	0	0	0	0	0
Cronobacter	2	120	0	0	0	0	0	0	0	0	0	0	0	0	0	0	0	0	0	0
Cryptobacterium	2	39	0	0	0	0	0	0	0	0	0	0	0	0	0	0	0	0	0	0
Cucumis	1	9	0	0	0	0	0	0	0	0	0	0	0	0	0	0	0	0	0	0
Cupriavidus	3	317	1	0	1	0	1	0	0	0	0	1	1	0	1	0	0	0	0	0
Curtobacterium	1	5	0	0	0	0	0	0	0	0	0	0	0	0	0	0	0	0	0	0
Curvibacter	1	27	1	1	1	1	0	0	0	0	0	0	0	0	0	0	0	0	0	0
Cyanobacterium	1	9	0	0	0	0	0	0	0	0	0	0	0	0	0	0	0	0	0	0
Cyanobium	1	10	0	0	0	0	0	0	0	0	0	0	0	0	0	0	0	0	0	0
Cyanothece	1	34	0	0	0	0	0	0	0	0	0	0	0	0	0	0	0	0	0	0
Cyclobacterium	1	19	0	0	0	0	0	0	0	0	0	0	0	0	0	0	0	0	0	0
Cycloclasticus	1	21	0	0	0	0	1	0	0	0	0	1	0	0	0	0	0	0	0	0
Cylindrospermopsis	1	4	0	0	0	0	0	0	0	0	0	0	0	0	0	0	0	0	0	0
Cylindrospermum	1	30	0	0	0	0	0	0	0	0	0	0	0	0	0	0	0	0	0	0

Genus	Cluster Number	Protein Counts	K10218	K10219	K10220	K10221	K10222	K10616	K10617	K10618	K10619	K10621	K10622	K10623	K10676	K10700	K10701	K10702	K11180	K11946
Cytophaga	2	69	0	0	0	0	0	0	0	0	0	0	0	0	0	0	0	0	0	0
Dactylococcopsis	1	8	0	0	0	0	0	0	0	0	0	0	0	0	0	0	0	0	0	0
Dechloromonas	1	40	0	0	0	0	0	0	0	0	0	0	0	0	1	0	0	0	0	0
Dechlorosoma	1	19	0	0	0	0	0	0	0	0	0	0	0	0	0	0	0	0	0	0
Deferribacter	2	73	0	0	0	0	0	0	0	0	0	0	0	0	0	0	0	0	0	0
Dehalobacter	1	9	0	0	0	0	0	0	0	0	0	0	0	0	0	0	0	0	0	0
Dehalococcoides	2	41	0	0	0	0	0	0	0	0	0	0	0	0	0	0	0	0	0	0
Dehalogenimonas	1	37	0	0	0	0	0	0	0	0	0	0	0	0	0	0	0	0	0	0
Deinococcus	2	140	0	1	0	0	0	0	0	0	0	0	0	0	0	0	0	0	0	0
Delftia	3	177	1	1	1	1	1	0	0	0	0	0	0	0	0	0	0	0	0	0
Denitrovibrio	2	67	0	0	0	0	0	0	0	0	0	0	0	0	0	0	0	0	0	0
Desulfarculus	2	92	0	0	0	0	0	0	0	0	0	0	0	0	0	0	0	0	1	0
Desulfatibacillum	3	84	0	0	0	0	0	0	0	0	0	0	0	0	0	0	0	0	1	0
Desulfitobacterium	2	102	0	0	0	0	0	0	0	0	0	0	0	0	0	0	0	0	1	0
Desulfobacca	2	65	0	0	0	0	0	0	0	0	0	0	0	0	0	0	0	0	1	0
Desulfobacterium	2	113	0	0	0	0	0	0	0	0	0	0	0	0	0	0	0	0	1	0
Desulfobacula	1	21	0	0	0	0	0	0	0	0	0	0	0	0	0	0	0	0	1	0
Desulfobulbus	2	88	0	0	0	0	0	0	0	0	0	0	0	0	0	0	0	0	1	0
Desulfocapsa	1	9	0	0	0	0	0	0	0	0	0	0	0	0	0	0	0	0	1	0
Desulfococcus	2	80	0	0	0	0	0	0	0	0	0	0	0	0	0	0	0	0	1	0
Desulfohalobium	2	52	0	0	0	0	0	0	0	0	0	0	0	0	0	0	0	0	1	0
Desulfomicrobium	2	65	0	0	0	0	0	0	0	0	0	0	0	0	0	0	0	0	1	0
Desulfomonile	1	25	1	0	0	0	0	0	0	0	0	0	0	0	0	0	0	0	1	0
Desulforhabdus	1	2	0	0	0	0	0	0	0	0	0	0	0	0	0	0	0	0	1	0
Desulfosporosinus	1	30	0	0	0	0	0	0	0	0	0	0	0	0	0	0	0	0	1	0
Desulfotalea	2	66	0	0	0	0	0	0	0	0	0	0	0	0	0	0	0	0	1	0
Desulfotomaculum	2	98	0	0	0	0	0	0	0	0	0	0	0	0	0	0	0	0	1	0
Desulfovibrio	2	144	0	0	0	0	0	0	0	0	0	0	0	0	0	0	0	0	1	0
Desulfurispirillum	2	78	0	0	0	0	0	0	0	0	0	0	0	0	0	0	0	0	0	0
Desulfurivibrio	2	71	0	0	0	0	0	0	0	0	0	0	0	0	0	0	0	0	1	0
Desulfurobacterium	2	53	0	0	0	0	0	0	0	0	0	0	0	0	0	0	0	0	0	0
Desulfurococcus	1	11	0	0	0	0	0	0	0	0	0	0	0	0	0	0	0	0	0	0
Diaphorobacter	1	16	0	0	0	0	0	0	0	0	0	0	0	0	0	0	0	0	0	0
Dichelobacter	2	45	0	0	0	0	0	0	0	0	0	0	0	0	0	0	0	0	0	0
Dickeya	2	155	0	0	0	0	0	0	0	0	0	0	0	0	0	0	0	0	0	0
Dictyoglomus	2	62	0	0	0	0	0	0	0	0	0	0	0	0	0	0	0	0	0	0
Dinoroseobacter	3	148	0	0	0	0	0	0	0	0	0	0	0	0	0	0	0	0	0	0
Dokdonia	1	3	0	0	0	0	0	0	0	0	0	0	0	0	0	0	0	0	0	0
Dorea	1	7	0	0	0	0	0	0	0	0	0	0	0	0	0	0	0	0	0	0
Dyadobacter	2	83	0	0	0	0	0	0	0	0	0	0	0	0	0	0	0	0	0	0
Echinicola	1	20	0	0	0	0	0	0	0	0	0	0	0	0	0	0	0	0	0	0
Edwardsiella	2	126	0	0	0	0	0	0	0	0	0	0	0	0	0	0	0	0	0	0
Eggerthella	1	51	0	0	0	0	0	0	0	0	0	0	0	0	0	0	0	0	0	0
Ehrlichia	2	37	0	0	0	0	0	0	0	0	0	0	0	0	0	0	0	0	0	0
Eikenella	1	4	0	0	0	0	0	0	0	0	0	0	0	0	0	0	0	0	0	0
Elusimicrobium	2	44	0	0	0	0	0	0	0	0	0	0	0	0	0	0	0	0	0	0
Emticicia	1	21	1	0	0	0	0	0	0	0	0	0	0	0	0	0	0	0	0	0
Enhydrobacter	1	1	0	0	0	0	0	0	0	0	0	0	0	0	0	0	0	0	0	0
Ensifer	1	2	0	0	0	0	0	0	0	0	0	0	0	0	0	0	0	0	0	0
Enterobacter	2	202	0	0	0	0	0	0	0	0	0	0	0	0	0	0	0	0	0	0
Enterobacteriaceae	2	211	0	0	0	0	0	0	0	0	0	0	0	0	0	0	0	0	0	0
Enterococcus	1	187	1	0	0	0	0	0	0	0	0	0	0	0	0	0	0	0	0	0

Genus	Cluster Number	Protein Counts	K10218	K10219	K10220	K10221	K10222	K10616	K10617	K10618	K10619	K10621	K10622	K10623	K10676	K10700	K10701	K10702	K11180	K11946
Enterovibrio	1	15	0	0	0	0	0	0	0	0	0	0	0	0	0	0	0	0	0	0
Erwinia	2	151	1	0	0	0	0	0	0	0	0	0	0	0	0	0	0	0	0	0
Erysipelothrix	1	9	0	0	0	0	0	0	0	0	0	0	0	0	0	0	0	0	0	0
Erysipelotrichaceae	1	4	0	0	0	0	0	0	0	0	0	0	0	0	0	0	0	0	0	0
Erythrobacter	2	80	0	0	0	0	0	0	0	0	0	0	0	0	0	0	0	0	0	0
Escherichia	2	242	1	0	0	0	0	0	0	0	0	0	0	0	0	0	0	0	0	0
Ethanoligenens	2	69	0	0	0	0	0	0	0	0	0	0	0	0	0	0	0	0	0	0
Eubacteriaceae	1	2	0	0	0	0	0	0	0	0	0	0	0	0	0	0	0	0	0	0
Eubacterium	2	103	0	0	0	0	0	0	0	0	0	0	0	0	0	0	0	0	0	0
Eudoraea	1	4	0	0	0	0	0	0	0	0	0	0	0	0	0	0	0	0	0	0
Euryarchaeota	1	1	0	0	0	0	0	0	0	0	0	0	0	0	0	0	0	0	0	0
Exiguobacterium	2	93	0	0	0	0	0	0	0	0	0	0	0	0	0	0	0	0	0	0
Faecalibacterium	1	12	0	0	0	0	0	0	0	0	0	0	0	0	0	0	0	0	0	0
Ferrimonas	2	115	0	0	0	0	0	0	0	0	0	0	0	0	0	0	0	0	0	0
Ferroglobus	1	14	0	0	0	0	0	0	0	0	0	0	0	0	0	0	0	0	0	0
Ferroplasma	1	11	0	0	0	0	0	0	0	0	0	0	0	0	0	0	0	0	0	0
Fervidicoccus	1	7	0	0	0	0	0	0	0	0	0	0	0	0	0	0	0	0	0	0
Fervidobacterium	2	54	0	0	0	0	0	0	0	0	0	0	0	0	0	0	0	0	0	0
Fibrella	1	17	0	0	0	0	0	0	0	0	0	0	0	0	0	0	0	0	0	0
Fibrobacter	2	52	0	0	0	0	0	0	0	0	0	0	0	0	0	0	0	0	0	0
Filifactor	1	9	0	0	0	0	0	0	0	0	0	0	0	0	0	0	0	0	0	0
Finegoldia	2	55	0	0	0	0	0	0	0	0	0	0	0	0	0	0	0	0	0	0
Firmicutes	1	15	0	0	0	0	0	0	0	0	0	0	0	0	0	0	0	0	0	0
Fischerella	1	15	0	0	0	0	0	0	0	0	0	0	0	0	0	0	0	0	0	0
Flavobacteria	1	7	0	0	0	0	0	0	0	0	0	0	0	0	0	0	0	0	0	0
Flavobacteriaceae	2	69	0	0	0	0	0	0	0	0	0	0	0	0	0	0	0	0	0	0
Flavobacteriales	1	3	0	0	0	0	0	0	0	0	0	0	0	0	0	0	0	0	0	0
Flavobacterium	2	103	0	0	0	0	0	0	0	0	0	0	0	0	0	0	0	0	0	0
Flexibacter	1	17	0	0	0	0	0	0	0	0	0	0	0	0	0	0	0	0	0	0
Flexistipes	1	11	0	0	0	0	0	0	0	0	0	0	0	0	0	0	0	0	0	0
Fluoribacter	1	6	0	0	0	0	0	0	0	0	0	0	0	0	0	0	0	0	0	0
Fluviicola	2	69	0	0	0	0	0	0	0	0	0	0	0	0	0	0	0	0	0	0
Formosa	1	2	0	0	0	0	0	0	0	0	0	0	0	0	0	0	0	0	0	0
Francisella	2	88	0	0	0	0	0	0	0	0	0	0	0	0	0	0	0	0	0	0
Frankia	3	202	0	0	0	0	0	0	0	0	0	0	0	0	0	0	0	0	0	0
Frateuria	1	25	0	0	0	0	0	0	0	0	0	0	0	0	0	0	0	0	0	0
Fructobacillus	1	2	0	0	0	0	0	0	0	0	0	0	0	0	0	0	0	0	0	0
Fulvimarina	1	2	0	0	0	0	0	0	0	0	0	0	0	0	0	0	0	0	0	0
Fusobacterium	1	48	0	0	0	0	0	0	0	0	0	0	0	0	0	0	0	0	0	0
Gallibacterium	2	87	0	0	0	0	0	0	0	0	0	0	0	0	0	0	0	0	0	0
Gallionella	2	67	0	0	0	0	0	0	0	0	0	0	0	0	0	0	0	0	0	0
Gammaproteobacteria	1	9	0	0	0	0	0	0	0	0	0	0	0	0	0	0	0	0	0	0
Gardnerella	1	31	0	0	0	0	0	0	0	0	0	0	0	0	0	0	0	0	0	0
Geitlerinema	1	9	0	0	0	0	0	0	0	0	0	0	0	0	0	0	0	0	0	0
Gemmatimonas	2	93	0	0	0	0	0	0	0	0	0	0	0	0	0	0	0	0	0	0
Geobacillus	2	168	1	0	0	0	0	0	0	0	0	0	0	0	0	0	0	0	0	0
Geobacter	2	150	0	0	0	0	0	0	0	0	0	0	0	0	0	0	0	0	0	0
Geodermatophilus	2	136	0	0	0	0	0	0	0	0	0	0	0	0	0	0	0	0	0	0
Geopsychrobacter	1	2	0	0	0	0	0	0	0	0	0	0	0	0	0	0	0	0	0	0
Gillisia	1	11	0	0	0	0	0	0	0	0	0	0	0	0	0	0	0	0	0	0
Gilvimarinus	1	4	0	0	0	0	0	0	0	0	0	0	0	0	0	0	0	0	0	0
Glaciecola	2	117	0	1	1	0	0	0	0	0	0	0	0	0	0	0	0	0	0	0

Genus	Cluster Number	Protein Counts	K10218	K10219	K10220	K10221	K10222	K10616	K10617	K10618	K10619	K10621	K10622	K10623	K10676	K10700	K10701	K10702	K11180	K11946
Gloeobacter	2	67	0	0	0	0	0	0	0	0	0	0	0	0	0	0	0	0	0	0
Gloeocapsa	1	20	0	0	0	0	0	0	0	0	0	0	0	0	0	0	0	0	0	0
Gloeothece	1	1	0	0	0	0	0	0	0	0	0	0	0	0	0	0	0	0	0	0
Gluconacetobacter	2	96	0	0	0	0	0	0	0	0	0	0	0	0	0	0	0	0	0	0
Gluconobacter	2	76	0	0	0	0	0	0	0	0	0	0	0	0	0	0	0	0	0	0
Gordonia	1	158	1	0	1	0	0	0	0	0	0	0	0	0	0	0	0	0	0	0
Gordonibacter	1	32	0	0	0	0	0	0	0	0	0	0	0	0	0	0	0	0	1	0
Gramella	2	104	0	0	0	0	0	0	0	0	0	0	0	0	0	0	0	0	0	0
Granulibacter	2	90	0	0	0	0	0	0	0	0	0	0	0	0	0	0	0	0	0	0
Granulicella	2	98	0	0	0	0	0	0	0	0	0	0	0	0	0	0	0	0	0	0
Grimontia	1	9	0	0	0	0	0	0	0	0	0	0	0	0	0	0	0	0	0	0
Haemophilus	2	117	0	0	0	0	0	0	0	0	0	0	0	0	0	0	0	0	0	0
Hafnia	1	29	0	0	0	0	0	0	0	0	0	0	0	0	0	0	0	0	0	0
Hahella	3	130	0	0	0	0	0	0	0	0	0	0	0	0	0	0	0	0	0	0
Halalkalicoccus	1	18	0	0	0	0	0	0	0	0	0	0	0	0	0	0	0	0	0	0
Halanaerobium	2	89	0	0	0	0	0	0	0	0	0	0	0	0	0	0	0	0	0	0
Haliangium	2	108	0	0	0	0	0	0	0	0	0	0	0	0	0	0	0	0	0	0
Haliscomenobacter	2	107	0	0	0	0	0	0	0	0	0	0	0	0	0	0	0	0	0	0
Haloarcula	1	22	0	0	0	0	0	0	0	0	0	0	0	0	0	0	0	0	0	0
Halobacillus	1	19	0	0	0	0	0	0	0	0	0	0	0	0	0	0	0	0	0	0
Halobacteriaceae	1	1	0	0	0	0	0	0	0	0	0	0	0	0	0	0	0	0	0	0
Halobacterium	1	10	0	0	0	0	0	0	0	0	0	0	0	0	0	0	0	0	0	0
Halobacteroides	1	14	0	0	0	0	0	0	0	0	0	0	0	0	0	0	0	0	0	0
Haloferax	1	20	0	0	0	0	0	0	0	0	0	0	0	0	0	0	0	0	0	0
Halogeometricum	1	12	0	0	0	0	0	0	0	0	0	0	0	0	0	0	0	0	0	0
Halomicrobium	1	10	0	0	0	0	0	0	0	0	0	0	0	0	0	0	0	0	0	0
Halomonas	1	149	0	0	0	0	0	0	0	0	0	0	0	0	0	0	0	0	0	0
Halopiger	1	16	0	0	0	0	0	0	0	0	0	0	0	0	0	0	0	0	0	0
Haloquadratum	1	19	0	0	0	0	0	0	0	0	0	0	0	0	0	0	0	0	0	0
Halorhabdus	1	10	0	0	0	0	0	0	0	0	0	0	0	0	0	0	0	0	0	0
Halorhodospira	2	71	0	0	0	0	0	0	0	0	0	0	0	0	0	0	0	0	1	0
Halorubrum	1	16	0	0	0	0	0	0	0	0	0	0	0	0	0	0	0	0	0	0
Haloterrigena	1	19	0	0	0	0	0	0	0	0	0	0	0	0	0	0	0	0	0	0
Halothece	1	9	0	0	0	0	0	0	0	0	0	0	0	0	0	0	0	0	0	0
Halothermothrix	2	50	0	0	0	0	0	0	0	0	0	0	0	0	0	0	0	0	0	0
Halothiobacillus	2	65	0	0	0	0	0	0	0	0	0	0	0	0	0	0	0	0	0	0
Halovivax	1	12	0	0	0	0	0	0	0	0	0	0	0	0	0	0	0	0	0	0
Helicobacter	2	79	0	0	0	0	0	0	0	0	0	0	0	0	0	0	0	0	0	0
Heliobacillus	1	4	0	0	0	0	0	0	0	0	0	0	0	0	0	0	0	0	0	0
Heliobacterium	2	51	0	0	0	0	0	0	0	0	0	0	0	0	0	0	0	0	0	0
Herbaspirillum	2	172	1	0	0	0	0	0	0	0	0	0	0	0	0	0	0	0	0	0
Herminiimonas	3	89	0	0	0	0	0	0	0	0	0	0	0	0	0	0	0	0	0	0
Herpetosiphon	3	90	0	0	0	0	0	0	0	0	0	0	0	0	0	0	0	0	0	0
Hippea	2	44	0	0	0	0	0	0	0	0	0	0	0	0	0	0	0	0	0	0
Hirschia	2	102	0	0	1	0	0	0	0	0	0	0	0	0	0	0	0	0	0	0
Histophilus	2	72	0	0	0	0	0	0	0	0	0	0	0	0	0	0	0	0	0	0
Hoeflea	1	16	0	0	0	0	0	0	0	0	0	0	0	0	0	0	0	0	0	0
Hydrogenivirga	1	2	0	0	0	0	0	0	0	0	0	0	0	0	0	0	0	0	0	0
Hydrogenobacter	2	55	0	0	0	0	0	0	0	0	0	0	0	0	0	0	0	0	0	0
Hydrogenobaculum	2	44	0	0	0	0	0	0	0	0	0	0	0	0	0	0	0	0	0	0
Hydrogenophaga	1	9	1	0	0	0	0	0	0	0	0	0	0	0	0	0	0	0	0	0
Hylemonella	1	4	0	0	0	0	0	0	0	0	0	0	0	0	0	0	0	0	0	0

Genus	Cluster Number	Protein Counts	K10218	K10219	K10220	K10221	K10222	K10616	K10617	K10618	K10619	K10621	K10622	K10623	K10676	K10700	K10701	K10702	K11180	K11946
Hyperthermus	1	6	0	0	0	0	0	0	0	0	0	0	0	0	0	0	0	0	0	0
Hyphomicrobium	2	93	0	0	0	0	0	0	0	0	0	0	0	0	0	0	0	0	0	0
Hyphomonas	3	105	0	0	0	0	0	0	0	0	0	0	0	0	0	0	0	0	0	0
Ideonella	1	18	0	0	1	0	0	0	0	0	0	0	0	0	0	0	0	0	0	0
Idiomarina	2	86	0	0	0	0	0	0	0	0	0	0	0	0	0	0	0	0	0	0
Ignavibacterium	1	14	0	0	0	0	0	0	0	0	0	0	0	0	0	0	0	0	0	0
Ignicoccus	1	5	0	0	0	0	0	0	0	0	0	0	0	0	0	0	0	0	0	0
Ignisphaera	1	8	0	0	0	0	0	0	0	0	0	0	0	0	0	0	0	0	0	0
Ilyobacter	2	85	0	0	0	0	0	0	0	0	0	0	0	0	0	0	0	0	0	0
Intrasporangium	2	114	0	0	0	0	0	0	0	0	0	0	0	0	0	0	0	0	0	0
Isoptericola	1	18	0	0	0	0	0	0	0	0	0	0	0	0	0	0	0	0	0	0
Isosphaera	2	75	0	0	0	0	0	0	0	0	0	0	0	0	0	0	0	0	0	0
Jannaschia	3	151	0	0	0	0	0	0	0	0	0	0	0	0	0	0	0	0	0	0
Janthinobacterium	2	99	0	0	0	0	0	0	0	0	0	0	0	0	0	0	0	0	0	0
Jonesia	2	56	0	0	0	0	0	0	0	0	0	0	0	0	0	0	0	0	0	0
Joostella	1	3	0	0	0	0	0	0	0	0	0	0	0	0	0	0	0	0	0	0
Kaistia	1	2	0	0	0	0	0	0	0	0	0	0	0	0	0	0	0	0	0	0
Kangiella	2	84	0	0	0	0	0	0	0	0	0	0	0	0	0	0	0	0	0	0
Ketogulonicigenium	2	102	1	0	0	0	0	0	0	0	0	0	0	0	0	0	0	0	0	0
Kineococcus	1	24	0	0	0	0	0	0	0	0	0	0	0	0	0	0	0	0	0	0
Kinetoplastibacterium	1	2	0	0	0	0	0	0	0	0	0	0	0	0	0	0	0	0	0	0
Kingella	1	4	0	0	0	0	0	0	0	0	0	0	0	0	0	0	0	0	0	0
Kitasatospora	1	36	1	1	1	1	0	0	0	0	0	0	0	0	0	0	0	0	0	0
Klebsiella	3	222	1	0	0	0	0	0	0	0	0	0	0	0	0	0	0	0	0	0
Kluyvera	1	1	0	0	0	0	0	0	0	0	0	0	0	0	0	0	0	0	0	0
Kocuria	2	87	0	0	0	0	0	0	0	0	0	0	0	0	0	0	0	0	0	0
Kordia	1	5	0	0	0	0	0	0	0	0	0	0	0	0	0	0	0	0	0	0
Kosmotoga	2	61	0	0	0	0	0	0	0	0	0	0	0	0	0	0	0	0	0	0
Kribbella	2	99	0	0	0	0	0	0	0	0	0	0	0	0	0	0	0	0	0	0
Krokinobacter	2	90	0	0	0	0	0	0	0	0	0	0	0	0	0	0	0	0	0	0
Kyrpidia	2	113	0	0	0	0	0	0	0	0	0	0	0	0	0	0	0	0	0	0
Kytococcus	2	70	0	0	0	0	0	0	0	0	0	0	0	0	0	0	0	0	0	0
Labrenzia	1	4	0	0	0	0	0	0	0	0	0	0	0	0	0	0	0	0	0	0
Lachnoanaerobaculum	1	1	0	0	0	0	0	0	0	0	0	0	0	0	0	0	0	0	0	0
Lachnospiraceae	1	51	0	0	0	0	0	0	0	0	0	0	0	0	0	0	0	0	0	0
Lacinutrix	1	20	0	0	0	0	0	0	0	0	0	0	0	0	0	0	0	0	0	0
Lactobacillus	2	119	0	0	0	0	0	0	0	0	0	0	0	0	0	0	0	0	0	0
Lactococcus	2	82	0	0	0	0	0	0	0	0	0	0	0	0	0	0	0	0	0	0
Lamprocystis	1	2	0	0	0	0	0	0	0	0	0	0	0	0	0	0	0	0	1	0
Laribacter	2	80	0	0	0	0	0	0	0	0	0	0	0	0	0	0	0	0	0	0
Lawsonia	2	33	0	0	0	0	0	0	0	0	0	0	0	0	0	0	0	0	0	0
Leadbetterella	2	79	0	0	0	0	0	0	0	0	0	0	0	0	0	0	0	0	0	0
Leeuwenhoekiella	1	2	0	0	0	0	0	0	0	0	0	0	0	0	0	0	0	0	0	0
Legionella	2	117	0	0	0	0	0	0	0	0	0	0	0	0	0	0	0	0	0	0
Leifsonia	2	48	0	0	0	0	0	0	0	0	0	0	0	0	0	0	0	0	0	0
Leptolyngbya	1	10	0	0	0	0	0	0	0	0	0	0	0	0	0	0	0	0	0	0
Leptospira	2	76	0	0	0	0	0	0	0	0	0	0	0	0	0	0	0	0	0	0
Leptospirillum	1	10	0	0	0	0	0	0	0	0	0	0	0	0	0	0	0	0	0	0
Leptothrix	3	186	1	1	1	1	0	0	0	0	0	0	0	0	0	0	0	0	0	0
Leptotrichia	2	43	0	0	0	0	0	0	0	0	0	0	0	0	0	0	0	0	0	0
Leucobacter	1	2	0	0	0	0	0	0	0	0	0	0	0	0	0	0	0	0	0	0
Leuconostoc	2	64	0	0	0	0	0	0	0	0	0	0	0	0	0	0	0	0	0	0

Genus	Cluster Number	Protein Counts	K10218	K10219	K10220	K10221	K10222	K10616	K10617	K10618	K10619	K10621	K10622	K10623	K10676	K10700	K10701	K10702	K11180	K11946
**Liberibacter**	1	4	0	0	0	0	0	0	0	0	0	0	0	0	0	0	0	0	0	0
**Limnohabitans**	1	11	1	0	0	0	0	0	0	0	0	0	0	0	0	0	0	0	0	0
**Listeria**	2	77	0	0	0	0	0	0	0	0	0	0	0	0	0	0	0	0	0	0
**Listeriaceae**	1	6	0	0	0	0	0	0	0	0	0	0	0	0	0	0	0	0	0	0
**Listonella**	1	38	0	0	0	0	0	0	0	0	0	0	0	0	0	0	0	0	0	0
**Loktanella**	1	3	0	0	0	0	0	0	0	0	0	0	0	0	0	0	0	0	0	0
**Lysinibacillus**	2	97	0	0	0	0	0	0	0	0	0	0	0	0	0	0	0	0	0	0
**Macrococcus**	2	60	0	0	0	0	0	0	0	0	0	0	0	0	0	0	0	0	0	0
**Magnetococcus**	2	64	0	0	0	0	0	0	0	0	0	0	0	0	0	0	0	0	1	0
**Magnetospirillum**	2	113	1	1	1	1	0	0	0	0	0	0	0	0	0	0	0	0	1	0
**Mahella**	2	58	0	0	0	0	0	0	0	0	0	0	0	0	0	0	0	0	0	0
**Mannheimia**	2	85	0	0	0	0	0	0	0	0	0	0	0	0	0	0	0	0	0	0
**Maribacter**	3	87	0	0	0	0	0	0	0	0	0	0	0	0	0	0	0	0	0	0
**Maricaulis**	3	87	0	0	0	0	0	0	0	0	0	0	0	0	0	0	0	0	0	0
**Marichromatium**	1	2	0	0	0	0	0	0	0	0	0	0	0	0	0	0	0	0	1	0
**Marinithermus**	2	77	0	0	0	0	0	0	0	0	0	0	0	0	0	0	0	0	0	0
**Marinitoga**	1	15	0	0	0	0	0	0	0	0	0	0	0	0	0	0	0	0	0	0
**Marinobacter**	2	163	1	0	1	0	0	0	0	0	0	0	0	0	0	0	0	0	0	0
**Marinomonas**	3	192	1	1	1	1	0	0	0	0	0	0	0	0	0	0	0	0	0	0
**Maritimibacter**	1	7	0	0	0	0	0	0	0	0	0	0	0	0	0	0	0	0	0	0
**Marivirga**	2	96	0	0	0	0	0	0	0	0	0	0	0	0	0	0	0	0	0	0
**Martelella**	1	3	0	0	0	0	0	0	0	0	0	0	0	0	0	0	0	0	0	0
**Marvinbryantia**	1	1	0	0	0	0	0	0	0	0	0	0	0	0	0	0	0	0	0	0
**Massilia**	1	4	0	0	0	0	0	0	0	0	0	0	0	0	0	0	0	0	0	0
**Mastigocladopsis**	1	16	0	0	0	0	0	0	0	0	0	0	0	0	0	0	0	0	0	0
**Megamonas**	1	47	0	0	0	0	0	0	0	0	0	0	0	0	0	0	0	0	0	0
**Megasphaera**	1	14	0	0	0	0	0	0	0	0	0	0	0	0	0	0	0	0	0	0
**Meiothermus**	2	110	1	0	0	0	0	0	0	0	0	0	0	0	0	0	0	0	0	0
**Melissococcus**	2	46	0	0	0	0	0	0	0	0	0	0	0	0	0	0	0	0	0	0
**Mesoflavibacter**	1	6	0	0	0	0	0	0	0	0	0	0	0	0	0	0	0	0	0	0
**Mesoplasma**	1	23	0	0	0	0	0	0	0	0	0	0	0	0	0	0	0	0	0	0
**Mesorhizobium**	2	201	0	0	0	0	0	0	0	0	0	0	0	0	1	0	0	0	0	0
**Mesotoga**	1	14	0	0	0	0	0	0	0	0	0	0	0	0	0	0	0	0	0	0
**Metallosphaera**	1	18	1	0	0	0	0	0	0	0	0	0	0	0	0	0	0	0	0	0
**Methanobacterium**	1	7	0	0	0	0	0	0	0	0	0	0	0	0	0	0	0	0	0	0
**Methanobrevibacter**	1	9	0	0	0	0	0	0	0	0	0	0	0	0	0	0	0	0	0	0
**Methanocaldococcus**	1	6	0	0	0	0	0	0	0	0	0	0	0	0	0	0	0	0	0	0
**Methanocella**	1	15	0	0	0	0	0	0	0	0	0	0	0	0	0	0	0	0	0	0
**Methanococcaceae**	1	1	0	0	0	0	0	0	0	0	0	0	0	0	0	0	0	0	0	0
**Methanococcoides**	1	5	0	0	0	0	0	0	0	0	0	0	0	0	0	0	0	0	0	0
**Methanococcus**	1	9	0	0	0	0	0	0	0	0	0	0	0	0	0	0	0	0	0	0
**Methanocorpusculum**	1	7	0	0	0	0	0	0	0	0	0	0	0	0	0	0	0	0	0	0
**Methanoculleus**	1	8	0	0	0	0	0	0	0	0	0	0	0	0	0	0	0	0	0	0
**Methanohalobium**	1	5	0	0	0	0	0	0	0	0	0	0	0	0	0	0	0	0	0	0
**Methanohalophilus**	1	5	0	0	0	0	0	0	0	0	0	0	0	0	0	0	0	0	0	0
**Methanolobus**	1	5	0	0	0	0	0	0	0	0	0	0	0	0	0	0	0	0	0	0
**Methanomethylovorans**	1	5	0	0	0	0	0	0	0	0	0	0	0	0	0	0	0	0	0	0
**Methanoplanus**	1	7	0	0	0	0	0	0	0	0	0	0	0	0	0	0	0	0	0	0
**Methanopyrus**	1	4	0	0	0	0	0	0	0	0	0	0	0	0	0	0	0	0	0	0
**Methanoregula**	1	8	0	0	0	0	0	0	0	0	0	0	0	0	0	0	0	0	0	0
**Methanosaeta**	1	9	0	0	0	0	0	0	0	0	0	0	0	0	0	0	0	0	0	0
**Methanosalsum**	1	7	0	0	0	0	0	0	0	0	0	0	0	0	0	0	0	0	0	0

Genus	Cluster Number	Protein Counts	K10218	K10219	K10220	K10221	K10222	K10616	K10617	K10618	K10619	K10621	K10622	K10623	K10676	K10700	K10701	K10702	K11180	K11946
Methanosarcina	1	18	0	0	0	0	0	0	0	0	0	0	0	0	0	0	0	0	0	0
Methanosphaera	1	7	0	0	0	0	0	0	0	0	0	0	0	0	0	0	0	0	0	0
Methanosphaerula	1	8	0	0	0	0	0	0	0	0	0	0	0	0	0	0	0	0	0	0
Methanospirillum	1	6	0	0	0	0	0	0	0	0	0	0	0	0	0	0	0	0	0	0
Methanothermobacter	1	6	0	0	0	0	0	0	0	0	0	0	0	0	0	0	0	0	0	0
Methanothermococcus	1	5	0	0	0	0	0	0	0	0	0	0	0	0	0	0	0	0	0	0
Methanothermus	1	5	0	0	0	0	0	0	0	0	0	0	0	0	0	0	0	0	0	0
Methanotorris	1	5	0	0	0	0	0	0	0	0	0	0	0	0	0	0	0	0	0	0
Methylacidiphilum	2	48	0	0	0	0	0	0	0	0	0	0	0	0	0	0	0	0	0	0
Methylarcula	1	2	0	0	0	0	0	0	0	0	0	0	0	0	0	0	0	0	0	0
Methylibium	1	38	0	0	0	0	0	0	0	0	0	0	0	0	0	0	0	0	0	0
Methylobacillus	2	61	0	0	0	0	0	0	0	0	0	0	0	0	0	0	0	0	0	0
Methylobacterium	3	236	0	0	1	0	0	0	0	0	0	0	0	0	0	0	0	0	0	0
Methylocella	3	117	0	0	0	0	0	0	0	0	0	0	0	0	1	0	0	0	0	0
Methylococcus	2	80	0	0	0	0	0	0	0	0	0	0	0	0	0	0	0	0	0	0
Methylocystis	1	21	0	0	0	0	0	0	0	0	0	0	0	0	0	0	0	0	0	0
Methyloferula	1	2	0	0	0	0	0	0	0	0	0	0	0	0	0	0	0	0	0	0
Methylomicrobium	1	12	0	0	0	0	0	0	0	0	0	0	0	0	0	0	0	0	0	0
Methylomonas	1	15	0	0	0	0	0	0	0	0	0	0	0	0	0	0	0	0	0	0
Methylophaga	1	7	0	0	0	0	0	0	0	0	0	0	0	0	0	0	0	0	0	0
Methylophilus	1	2	0	0	0	0	0	0	0	0	0	0	0	0	0	0	0	0	0	0
Methylosinus	1	2	0	0	0	0	0	0	0	0	0	0	0	0	0	0	0	0	0	0
Methylotenera	2	72	0	0	0	0	0	0	0	0	0	0	0	0	0	0	0	0	0	0
Methyloversatilis	1	5	0	0	0	0	0	0	0	0	0	0	0	0	0	0	0	0	0	0
Methylovorus	2	66	0	0	0	0	0	0	0	0	0	0	0	0	0	0	0	0	0	0
Micavibrio	1	9	0	0	0	0	0	0	0	0	0	0	0	0	0	0	0	0	0	0
Microbacterium	2	115	1	0	0	0	0	0	0	0	0	0	0	0	0	0	0	0	0	0
Microchaete	1	23	0	0	0	0	0	0	0	0	0	0	0	0	0	0	0	0	0	0
Micrococcus	2	72	0	0	0	0	0	0	0	0	0	0	0	0	0	0	0	0	0	0
Microcoleus	1	17	0	0	0	0	0	0	0	0	0	0	0	0	0	0	0	0	0	0
Microcystis	2	67	0	0	0	0	0	0	0	0	0	0	0	0	0	0	0	0	0	0
Microlunatus	1	25	1	0	0	0	0	0	0	0	0	0	0	0	0	0	0	0	0	0
Micromonospora	2	115	1	0	0	0	0	0	0	0	0	0	0	0	0	0	0	0	0	0
Microvirga	1	6	0	0	0	0	0	0	0	0	0	0	0	0	0	0	0	0	0	0
Mobiluncus	1	41	0	0	0	0	0	0	0	0	0	0	0	0	0	0	0	0	0	0
Modestobacter	1	43	1	0	1	0	0	0	0	0	0	0	0	0	0	0	0	0	0	0
Moorea	1	2	0	0	0	0	0	0	0	0	0	0	0	0	0	0	0	0	0	0
Moorella	2	69	0	0	0	0	0	0	0	0	0	0	0	0	0	0	0	0	1	0
Moraxella	2	66	0	0	0	0	0	0	0	0	0	0	0	0	0	0	0	0	0	0
Morganella	1	29	0	0	0	0	0	0	0	0	0	0	0	0	0	0	0	0	0	0
Moritella	1	2	0	0	0	0	0	0	0	0	0	0	0	0	0	0	0	0	0	0
Muricauda	1	23	0	0	0	0	0	0	0	0	0	0	0	0	0	0	0	0	0	0
Mycobacterium	3	306	1	0	1	0	0	0	0	0	0	1	0	0	1	0	0	0	0	1
Mycoplasma	1	53	0	0	0	0	0	0	0	0	0	0	0	0	0	0	0	0	0	0
Myxococcus	2	110	0	0	0	0	0	0	0	0	0	0	0	0	0	0	0	0	0	0
Nakamurella	2	115	0	0	0	0	0	0	0	0	0	0	0	0	1	0	0	0	0	0
napthalene	1	2	0	0	0	0	0	0	0	0	0	0	0	0	0	0	0	0	0	0
Natranaerobius	2	55	0	0	0	0	0	0	0	0	0	0	0	0	0	0	0	0	0	0
Natrialba	1	16	0	0	0	0	0	0	0	0	0	0	0	0	0	0	0	0	0	0
Natrinema	1	16	0	0	0	0	0	0	0	0	0	0	0	0	0	0	0	0	0	0
Natronobacterium	1	11	0	0	0	0	0	0	0	0	0	0	0	0	0	0	0	0	0	0
Natronococcus	1	21	0	0	0	0	0	0	0	0	0	0	0	0	0	0	0	0	0	0

Genus	Cluster Number	Protein Counts	K10218	K10219	K10220	K10221	K10222	K10616	K10617	K10618	K10619	K10621	K10622	K10623	K10676	K10700	K10701	K10702	K11180	K11946
Natronomonas	1	20	0	0	0	0	0	0	0	0	0	0	0	0	0	0	0	0	0	0
Nautilia	2	48	0	0	0	0	0	0	0	0	0	0	0	0	0	0	0	0	0	0
Neisseria	2	67	0	0	0	0	0	0	0	0	0	0	0	0	0	0	0	0	0	0
Neisseriaceae	1	1	0	0	0	0	0	0	0	0	0	0	0	0	0	0	0	0	0	0
Neorickettsia	1	31	0	0	0	0	0	0	0	0	0	0	0	0	0	0	0	0	0	0
Neptuniibacter	1	2	0	0	0	0	0	0	0	0	0	0	0	0	0	0	0	0	0	0
Niastella	1	21	0	0	0	0	0	0	0	0	0	0	0	0	0	0	0	0	0	0
Nitratifractor	2	54	0	0	0	0	0	0	0	0	0	0	0	0	0	0	0	0	0	0
Nitratireductor	1	19	0	0	0	0	0	0	0	0	0	0	0	0	0	0	0	0	0	0
Nitratiruptor	2	40	0	0	0	0	0	0	0	0	0	0	0	0	0	0	0	0	0	0
Nitrobacter	2	99	0	0	0	0	0	0	0	0	0	0	0	0	0	0	0	0	0	0
Nitrococcus	1	2	0	0	0	0	0	0	0	0	0	0	0	0	0	0	0	0	0	0
Nitrosococcus	2	94	0	0	0	0	0	0	0	0	0	0	0	0	0	0	0	0	0	0
Nitrosomonas	2	72	0	0	0	0	0	0	0	0	0	0	0	0	0	0	0	0	0	0
Nitrosopumilaceae	1	7	0	0	0	0	0	0	0	0	0	0	0	0	0	0	0	0	0	0
Nitrosopumilus	1	11	0	0	0	0	0	0	0	0	0	0	0	0	0	0	0	0	0	0
Nitrosospira	2	74	0	0	0	0	0	0	0	0	0	0	0	0	0	0	0	0	0	0
Nocardia	2	185	0	0	0	0	0	0	0	0	0	0	0	0	0	0	0	0	0	0
Nocardioidaceae	1	2	0	0	0	0	0	0	0	0	0	0	0	0	0	0	0	0	0	0
Nocardioides	2	157	0	0	0	0	0	0	0	0	0	0	1	0	1	0	0	0	0	0
Nocardiopsis	2	102	0	0	0	0	0	0	0	0	0	0	0	0	0	0	0	0	0	0
Nodularia	1	22	0	0	0	0	0	0	0	0	0	0	0	0	0	0	0	0	0	0
Nonlabens	1	19	0	0	0	0	0	0	0	0	0	0	0	0	0	0	0	0	0	0
Nostoc	2	111	0	0	0	0	0	0	0	0	0	0	0	0	0	0	0	0	0	0
Nostocaceae	1	2	0	0	0	0	0	0	0	0	0	0	0	0	0	0	0	0	0	0
Novosphingobium	2	169	1	1	1	1	0	0	0	0	0	0	0	0	0	0	0	0	0	0
Oceanibaculum	1	3	0	0	0	0	0	0	0	0	0	0	0	0	0	0	0	0	0	0
Oceanibulbus	1	12	0	0	0	0	0	0	0	0	0	0	0	0	0	0	0	0	0	0
Oceanicola	1	9	0	0	0	0	0	0	0	0	0	0	0	0	0	0	0	0	0	0
Oceanimonas	1	33	0	0	0	0	0	0	0	0	0	0	0	0	0	0	0	0	0	0
Oceaniovalibus	1	2	0	0	0	0	0	0	0	0	0	0	0	0	0	0	0	0	0	0
Oceanithermus	2	72	0	0	0	0	0	0	0	0	0	0	0	0	0	0	0	0	0	0
Oceanobacillus	2	91	0	0	0	0	0	0	0	0	0	0	0	0	0	0	0	0	0	0
Oceanospirillum	1	2	0	0	0	0	0	0	0	0	0	0	0	0	0	0	0	0	0	0
Ochrobactrum	2	133	0	0	0	0	0	0	0	0	0	0	0	0	0	0	0	0	0	0
Octadecabacter	1	29	0	0	0	0	0	0	0	0	0	0	0	0	0	0	0	0	0	0
Oenococcus	2	41	0	0	0	0	0	0	0	0	0	0	0	0	0	0	0	0	0	0
Oleomonas	1	2	0	0	0	0	0	0	0	0	0	0	0	0	0	0	0	0	0	0
Oligotropha	3	87	0	0	0	0	0	0	0	0	0	0	0	0	0	0	0	0	0	0
Olsenella	2	40	0	0	0	0	0	0	0	0	0	0	0	0	0	0	0	0	0	0
Opitutus	2	67	0	0	0	0	0	0	0	0	0	0	0	0	0	0	0	0	0	0
Orientia	1	21	0	0	0	0	0	0	0	0	0	0	0	0	0	0	0	0	0	0
Ornithobacterium	1	11	0	0	0	0	0	0	0	0	0	0	0	0	0	0	0	0	0	0
Oscillatoria	1	16	0	0	0	0	0	0	0	0	0	0	0	0	0	0	0	0	0	0
Oscillatoriales	1	2	0	0	0	0	0	0	0	0	0	0	0	0	0	0	0	0	0	0
Oscillibacter	1	20	0	0	0	0	0	0	0	0	0	0	0	0	0	0	0	0	0	0
Owenweeksia	1	15	0	0	0	0	0	0	0	0	0	0	0	0	0	0	0	0	0	0
Oxalobacteraceae	1	4	0	0	0	0	0	0	0	0	0	0	0	0	0	0	0	0	0	0
Paenibacillus	2	152	0	0	0	0	0	0	0	0	0	0	0	0	0	0	0	0	0	0
Paludibacter	2	67	0	0	0	0	0	0	0	0	0	0	0	0	0	0	0	0	0	0
Pandoraea	1	18	0	0	0	0	0	0	0	0	0	0	0	0	0	0	0	0	0	0
Pannonibacter	1	3	0	0	0	0	0	0	0	0	0	0	0	0	0	0	0	0	0	0

Genus	Cluster Number	Protein Counts	K10218	K10219	K10220	K10221	K10222	K10616	K10617	K10618	K10619	K10621	K10622	K10623	K10676	K10700	K10701	K10702	K11180	K11946
Pantholops	1	23	0	0	1	0	0	0	0	0	0	0	0	0	0	0	0	0	0	0
Pantoea	2	181	1	0	0	0	0	0	0	0	0	0	0	0	0	0	0	0	0	0
Parabacteroides	2	64	0	0	0	0	0	0	0	0	0	0	0	0	0	0	0	0	0	0
Parachlamydia	1	11	0	0	0	0	0	0	0	0	0	0	0	0	0	0	0	0	0	0
Paracoccus	2	169	0	0	1	0	0	0	0	0	0	0	0	0	0	0	0	0	0	0
Parvibaculum	3	117	0	0	0	0	0	0	0	0	0	0	0	0	0	0	0	0	0	0
Parvularcula	2	72	0	0	0	0	0	0	0	0	0	0	0	0	0	0	0	0	0	0
Pasteurella	2	85	0	0	0	0	0	0	0	0	0	0	0	0	0	0	0	0	0	0
Pasteurellaceae	1	2	0	0	0	0	0	0	0	0	0	0	0	0	0	0	0	0	0	0
Pectobacterium	1	44	0	0	0	0	0	0	0	0	0	0	0	0	0	0	0	0	0	0
Pediococcus	1	43	0	0	0	0	0	0	0	0	0	0	0	0	0	0	0	0	0	0
Pedobacter	2	88	0	0	0	0	0	0	0	0	0	0	0	0	0	0	0	0	0	0
Pelagibaca	1	10	0	0	0	0	0	0	0	0	0	0	0	0	0	0	0	0	0	0
Pelagibacterium	1	39	1	0	0	1	0	0	0	0	0	0	0	0	1	0	0	0	0	0
Pelobacter	2	85	0	0	0	0	0	0	0	0	0	0	0	0	0	0	0	0	0	0
Pelodictyon	2	62	0	0	0	0	0	0	0	0	0	0	0	0	0	0	0	0	1	0
Pelosinus	1	1	0	0	0	0	0	0	0	0	0	0	0	0	0	0	0	0	0	0
Pelotomaculum	2	60	0	0	0	0	0	0	0	0	0	0	0	0	0	0	0	0	0	0
Peptostreptococcus	1	1	0	0	0	0	0	0	0	0	0	0	0	0	0	0	0	0	0	0
Persephonella	2	55	0	0	0	0	0	0	0	0	0	0	0	0	0	0	0	0	0	0
Petrotoga	2	72	0	0	0	0	0	0	0	0	0	0	0	0	0	0	0	0	0	0
Phaeobacter	2	55	1	0	0	0	0	0	0	0	0	0	0	0	0	0	0	0	0	0
Phenylobacterium	3	103	0	0	0	0	0	0	0	0	0	0	0	0	0	0	0	0	0	0
Photobacterium	2	119	0	0	0	0	0	0	0	0	0	0	0	0	0	0	0	0	0	0
Photorhabdus	2	149	0	0	0	0	0	0	0	0	0	0	0	0	0	0	0	0	0	0
Phycisphaera	1	9	0	0	0	0	0	0	0	0	0	0	0	0	0	0	0	0	0	0
Phyllobacterium	1	6	0	0	0	0	0	0	0	0	0	0	0	0	0	0	0	0	0	0
Picrophilus	1	15	0	0	0	0	0	0	0	0	0	0	0	0	0	0	0	0	0	0
Pirellula	2	85	0	0	0	0	0	0	0	0	0	0	0	0	0	0	0	0	0	0
Planctomyces	2	93	0	0	0	0	0	0	0	0	0	0	0	0	0	0	0	0	0	0
Plautia	1	17	0	0	0	0	0	0	0	0	0	0	0	0	0	0	0	0	0	0
Plesiomonas	1	4	0	0	0	0	0	0	0	0	0	0	0	0	0	0	0	0	0	0
Pleurocapsa	1	23	0	0	0	0	0	0	0	0	0	0	0	0	0	0	0	0	0	0
Polaribacter	2	74	0	0	0	0	0	0	0	0	0	0	0	0	0	0	0	0	0	0
Polaromonas	3	238	1	1	1	1	1	0	0	0	0	0	0	0	0	0	0	0	0	0
Polymorphum	3	173	1	0	0	1	0	0	0	0	0	0	0	0	0	0	0	0	0	0
Polynucleobacter	1	19	0	0	0	0	0	0	0	0	0	0	0	0	0	0	0	0	0	0
Ponticaulis	1	1	0	0	0	0	0	0	0	0	0	0	0	0	0	0	0	0	0	0
Porphyromonas	2	63	0	0	0	0	0	0	0	0	0	0	0	0	0	0	0	0	0	0
Prevotella	1	65	0	0	0	0	0	0	0	0	0	0	0	0	0	0	0	0	0	0
Prochlorococcus	2	68	0	0	0	0	0	0	0	0	0	0	0	0	0	0	0	0	0	0
Propionibacterium	1	62	0	0	0	0	0	0	0	0	0	0	0	0	0	0	0	0	0	0
Prosthecochloris	1	16	0	0	0	0	0	0	0	0	0	0	0	0	0	0	0	0	1	0
Proteobacteria	1	19	0	0	0	0	0	0	0	0	0	0	0	0	0	0	0	0	0	0
Proteus	2	113	0	0	0	0	0	0	0	0	0	0	0	0	0	0	0	0	0	0
Providencia	1	38	0	0	0	0	0	0	0	0	0	0	0	0	0	0	0	0	0	0
Pseudaminobacter	1	10	0	0	0	0	0	0	0	0	0	0	0	0	0	0	0	0	0	0
Pseudanabaena	1	11	0	0	0	0	0	0	0	0	0	0	0	0	0	0	0	0	0	0
Pseudoalteromonas	2	188	1	1	1	1	0	0	0	0	0	0	0	0	0	0	0	0	0	0
Pseudochrobactrum	1	7	0	0	0	0	0	0	0	0	0	0	0	0	0	0	0	0	0	0
Pseudogulbenkiania	1	40	1	0	1	0	0	0	0	0	0	0	0	0	0	0	0	0	0	0
pseudomallei	3	131	0	0	0	0	0	0	0	0	0	0	0	0	0	0	0	0	0	0

Genus	Cluster Number	Protein Counts	K10218	K10219	K10220	K10221	K10222	K10616	K10617	K10618	K10619	K10621	K10622	K10623	K10676	K10700	K10701	K10702	K11180	K11946
Pseudomonas	3	381	1	1	1	1	1	1	1	1	1	1	1	1	1	0	0	1	0	0
Pseudonocardia	3	180	1	0	0	0	0	0	0	0	0	0	0	0	0	0	0	0	0	0
Pseudorhodobacter	1	4	0	0	0	0	0	0	0	0	0	0	0	0	0	0	0	0	0	0
Pseudovibrio	1	33	0	0	0	0	0	0	0	0	0	0	0	0	0	0	0	0	0	0
Pseudoxanthomonas	2	124	0	0	0	0	1	0	0	0	0	0	0	0	0	0	0	0	0	0
Psychrobacter	2	124	0	0	0	0	0	0	0	0	0	0	0	0	0	0	0	0	0	0
Psychroflexus	1	22	0	0	0	0	0	0	0	0	0	0	0	0	0	0	0	0	0	0
Psychromonas	2	110	0	0	0	0	0	0	0	0	0	0	0	0	0	0	0	0	0	0
Pusillimonas	3	111	0	0	0	0	0	0	0	0	0	0	0	0	0	0	0	0	0	0
Pyrobaculum	1	15	0	0	0	0	0	0	0	0	0	0	0	0	0	0	0	0	1	0
Pyrococcus	1	10	0	0	0	0	0	0	0	0	0	0	0	0	0	0	0	0	0	0
Pyrolobus	1	4	0	0	0	0	0	0	0	0	0	0	0	0	0	0	0	0	0	0
Rahnella	2	143	1	0	0	0	0	0	0	0	0	0	0	0	0	0	0	0	0	0
Ralstonia	3	332	1	0	1	0	1	0	0	0	0	1	1	0	1	0	0	1	0	0
Ramlibacter	1	35	1	1	1	1	0	0	0	0	0	0	0	0	0	0	0	0	0	0
Raoultella	2	139	1	0	0	0	0	0	0	0	0	0	0	0	0	0	0	0	0	0
Raphidiopsis	1	5	0	0	0	0	0	0	0	0	0	0	0	0	0	0	0	0	0	0
Reinekea	1	2	0	0	0	0	0	0	0	0	0	0	0	0	0	0	0	0	0	0
Renibacterium	2	67	0	0	0	0	0	0	0	0	0	0	0	0	0	0	0	0	0	0
Rhizobiaceae	1	3	0	0	0	0	0	0	0	0	0	0	0	0	0	0	0	0	0	0
Rhizobiales	1	6	0	0	0	0	0	0	0	0	0	0	0	0	0	0	0	0	0	0
Rhizobium	3	257	1	0	1	1	0	0	0	0	0	1	1	0	1	0	0	0	0	0
Rhizobium/Agrobacterium	2	87	0	0	0	0	0	0	0	0	0	1	1	0	0	0	0	0	0	0
Rhodanobacter	1	21	0	0	0	0	0	0	0	0	0	0	0	0	0	0	0	0	0	0
Rhodobacter	3	175	1	0	0	0	0	0	0	0	0	0	0	0	0	0	0	0	0	0
Rhodobacteraceae	1	21	0	0	0	0	0	0	0	0	0	0	0	0	0	0	0	0	0	0
Rhodobacterales	1	34	0	0	0	0	0	0	0	0	0	0	0	0	0	0	0	0	0	0
Rhodococcus	2	292	1	0	1	0	1	0	0	0	0	0	0	0	1	0	0	1	0	0
Rhodocyclaceae	1	7	0	0	0	0	0	0	0	0	0	0	0	0	0	0	0	0	0	0
Rhodoferax	3	144	1	1	1	1	0	0	0	0	0	0	0	0	0	0	0	0	0	0
Rhodomicrobium	2	103	0	0	0	0	0	0	0	0	0	0	0	0	0	0	0	0	1	0
Rhodopirellula	2	87	0	0	0	0	0	0	0	0	0	0	0	0	0	0	0	0	0	0
Rhodopseudomonas	3	246	1	1	1	1	0	0	0	0	0	0	0	0	0	0	0	0	0	0
Rhodospirillum	2	121	0	0	0	0	0	0	0	0	0	0	0	0	0	0	0	0	0	0
Rhodothermus	2	76	0	0	0	0	0	0	0	0	0	0	0	0	0	0	0	0	0	0
Rhodovulum	1	4	0	0	0	0	0	0	0	0	0	0	0	0	0	0	0	0	0	0
Richelia	1	2	0	0	0	0	0	0	0	0	0	0	0	0	0	0	0	0	0	0
Rickettsia	2	43	0	0	0	0	0	0	0	0	0	0	0	0	0	0	0	0	0	0
Rickettsiaceae	1	5	0	0	0	0	0	0	0	0	0	0	0	0	0	0	0	0	0	0
Riemerella	2	61	0	0	0	0	0	0	0	0	0	0	0	0	0	0	0	0	0	0
Rivularia	1	18	0	0	0	0	0	0	0	0	0	0	0	0	0	0	0	0	0	0
Robiginitalea	2	83	0	0	0	0	0	0	0	0	0	0	0	0	0	0	0	0	0	0
Roseburia	2	58	0	0	0	0	0	0	0	0	0	0	0	0	0	0	0	0	0	0
Roseibium	1	3	0	0	0	0	0	0	0	0	0	0	0	0	0	0	0	0	0	0
Roseiflexus	3	100	0	0	0	0	0	0	0	0	0	0	0	0	0	0	0	0	0	0
Roseobacter	2	149	0	0	0	0	0	0	0	0	0	0	0	0	0	0	0	0	0	0
Roseomonas	1	2	0	0	0	0	0	0	0	0	0	0	0	0	0	0	0	0	0	0
Roseovarius	1	15	0	0	0	0	0	0	0	0	0	0	0	0	0	0	0	0	0	0
Rothia	1	44	0	0	0	0	0	0	0	0	0	0	0	0	0	0	0	0	0	0
Rubrivivax	1	24	0	0	0	0	0	0	0	0	0	0	0	0	0	0	0	0	0	0
Rubrobacter	3	117	1	0	0	0	0	0	0	0	0	0	0	0	0	0	0	0	0	0
Ruegeria	2	189	0	0	0	0	0	0	0	0	0	0	0	0	0	0	0	0	0	0

Genus	Cluster Number	Protein Counts	K10218	K10219	K10220	K10221	K10222	K10616	K10617	K10618	K10619	K10621	K10622	K10623	K10676	K10700	K10701	K10702	K11180	K11946
Ruminococcaceae	1	2	0	0	0	0	0	0	0	0	0	0	0	0	0	0	0	0	0	0
Ruminococcus	2	101	0	0	0	0	0	0	0	0	0	0	0	0	0	0	0	0	0	0
Runella	1	26	1	0	0	0	0	0	0	0	0	0	0	0	0	0	0	0	0	0
Saccharomonospora	2	132	1	0	0	0	0	0	0	0	0	0	0	0	0	0	0	0	0	0
Saccharophagus	2	81	0	0	0	0	0	0	0	0	0	0	0	0	0	0	0	0	0	0
Saccharopolyspora	3	194	0	0	0	0	0	0	0	0	0	0	0	0	0	0	0	0	0	0
Saccharothrix	1	32	0	0	0	0	0	0	0	0	0	0	0	0	0	0	0	0	0	0
Sagittula	1	10	0	0	0	0	0	0	0	0	0	0	0	0	0	0	0	0	0	0
Salinibacter	2	77	0	0	0	0	0	0	0	0	0	0	0	0	0	0	0	0	0	0
Salinimonas	1	1	0	0	0	0	0	0	0	0	0	0	0	0	0	0	0	0	0	0
Salinispora	3	127	0	0	0	0	0	0	0	0	0	0	0	0	0	0	0	0	0	0
Salinivibrio	1	2	0	0	0	0	0	0	0	0	0	0	0	0	0	0	0	0	0	0
Salmonella	2	151	0	0	0	0	0	0	0	0	0	0	0	0	0	0	0	0	0	0
Sanguibacter	2	73	0	0	0	0	0	0	0	0	0	0	0	0	0	0	0	0	0	0
Saprospira	1	13	0	0	0	0	0	0	0	0	0	0	0	0	0	0	0	0	0	0
Scardovia	1	1	0	0	0	0	0	0	0	0	0	0	0	0	0	0	0	0	0	0
Scytonema	1	10	0	0	0	0	0	0	0	0	0	0	0	0	0	0	0	0	0	0
Sebaldella	2	69	0	0	0	0	0	0	0	0	0	0	0	0	0	0	0	0	0	0
Segniliparus	2	85	0	0	0	0	0	0	0	0	0	0	0	0	0	0	0	0	0	0
Selenomonas	2	57	0	0	0	0	0	0	0	0	0	0	0	0	0	0	0	0	0	0
Serinicoccus	1	1	0	0	0	0	0	0	0	0	0	0	0	0	0	0	0	0	0	0
Serratia	2	186	1	0	0	0	0	0	0	0	0	0	0	0	0	0	0	0	0	0
Shewanella	3	214	0	0	0	0	0	0	0	0	0	0	0	0	0	0	0	0	0	0
Shigella	2	172	0	0	0	0	0	0	0	0	0	0	0	0	0	0	0	0	0	0
Shinella	1	2	0	0	0	0	0	0	0	0	0	0	0	0	0	0	0	0	0	0
Sideroxydans	2	81	0	0	0	0	0	0	0	0	0	0	0	0	0	0	0	0	1	0
Silicibacter	2	105	0	0	0	0	0	0	0	0	0	0	0	0	0	0	0	0	0	0
Simiduia	1	18	0	0	0	0	0	0	0	0	0	0	0	0	0	0	0	0	0	0
Simkania	1	6	0	0	0	0	0	0	0	0	0	0	0	0	0	0	0	0	0	0
Simonsiella	1	2	0	0	0	0	0	0	0	0	0	0	0	0	0	0	0	0	0	0
Singulisphaera	1	18	0	0	0	0	0	0	0	0	0	0	0	0	0	0	0	0	0	0
Sinorhizobium	3	207	1	0	1	0	0	0	0	0	0	0	0	0	0	0	0	0	0	0
Slackia	2	46	0	0	0	0	0	0	0	0	0	0	0	0	0	0	0	0	0	0
Smaragdicoccus	1	3	0	0	0	0	0	0	0	0	0	0	0	0	0	0	0	0	0	0
Sodalis	2	71	0	0	0	0	0	0	0	0	0	0	0	0	0	0	0	0	0	0
Solibacillus	1	23	0	0	0	0	0	0	0	0	0	0	0	0	0	0	0	0	0	0
Solitalea	1	13	0	0	0	0	0	0	0	0	0	0	0	0	0	0	0	0	0	0
Sorangium	3	115	0	0	0	0	0	0	0	0	0	0	0	0	0	0	0	0	0	0
Sphaerobacter	2	100	1	0	0	0	0	0	0	0	0	0	0	0	0	0	0	0	0	0
Sphaerochaeta	1	42	0	0	0	0	0	0	0	0	0	0	0	0	0	0	0	0	0	0
Sphingobacterium	3	85	0	0	0	0	0	0	0	0	0	0	0	0	0	0	0	0	0	0
Sphingobium	1	179	1	1	1	1	0	0	0	0	0	1	0	0	1	0	0	0	0	0
Sphingomonadaceae	1	15	0	0	0	0	0	0	0	0	0	0	0	0	0	0	0	0	0	0
Sphingomonas	2	220	1	1	1	1	0	0	0	0	0	1	0	0	1	0	0	0	0	0
Sphingopyxis	3	99	0	0	0	0	0	0	0	0	0	0	0	0	0	0	0	0	0	0
Spirochaeta	2	103	0	0	0	0	0	0	0	0	0	0	0	0	0	0	0	0	0	0
Spiroplasma	1	6	0	0	0	0	0	0	0	0	0	0	0	0	0	0	0	0	0	0
Spirosoma	2	107	1	0	0	0	0	0	0	0	0	0	0	0	0	0	0	0	0	0
Stackebrandtia	2	110	0	0	0	0	0	0	0	0	0	0	0	0	0	0	0	0	0	0
Stanieria	1	17	0	0	0	0	0	0	0	0	0	0	0	0	0	0	0	0	0	0
Staphylococcus	2	108	0	0	0	0	0	0	0	0	0	0	0	0	0	0	0	0	0	0
Staphylothermus	1	12	0	0	0	0	0	0	0	0	0	0	0	0	0	0	0	0	0	0

Genus	Cluster Number	Protein Counts	K10218	K10219	K10220	K10221	K10222	K10616	K10617	K10618	K10619	K10621	K10622	K10623	K10676	K10700	K10701	K10702	K11180	K11946
**Stappia**	1	2	0	0	0	0	0	0	0	0	0	0	0	0	0	0	0	0	0	0
**Starkeya**	3	134	0	0	0	0	0	0	0	0	0	0	0	0	0	0	0	0	0	0
**Stenotrophomonas**	3	104	0	0	0	0	0	0	0	0	0	0	0	0	0	0	0	0	0	0
**Stigmatella**	3	129	0	0	0	0	0	0	0	0	0	0	0	0	0	0	0	0	0	0
**Streptobacillus**	1	38	0	0	0	0	0	0	0	0	0	0	0	0	0	0	0	0	0	0
**Streptococcus**	2	121	0	0	0	0	0	0	0	0	0	0	0	0	0	0	0	0	0	0
**Streptomyces**	2	253	1	0	1	1	0	0	0	0	0	1	1	0	1	0	0	0	0	0
**Streptosporangium**	3	148	1	0	0	0	0	0	0	0	0	0	0	0	0	0	0	0	0	0
**Strigomonas**	1	2	0	0	0	0	0	0	0	0	0	0	0	0	0	0	0	0	0	0
**Sulfitobacter**	1	10	0	0	0	0	0	0	0	0	0	0	0	0	0	0	0	0	0	0
**Sulfobacillus**	1	30	0	0	0	0	0	0	0	0	0	0	0	0	0	0	0	0	0	0
**Sulfolobus**	1	23	0	0	0	0	0	0	0	0	0	0	0	0	0	0	0	0	0	0
**Sulfuricurvum**	2	67	0	0	0	0	0	0	0	0	0	0	0	0	0	0	0	0	0	0
**Sulfurihydrogenibium**	2	55	0	0	0	0	0	0	0	0	0	0	0	0	0	0	0	0	0	0
**Sulfurimonas**	2	60	0	0	0	0	0	0	0	0	0	0	0	0	0	0	0	0	0	0
**Sulfurospirillum**	1	48	0	0	0	0	0	0	0	0	0	0	0	0	0	0	0	0	0	0
**Sulfurovum**	2	48	0	0	0	0	0	0	0	0	0	0	0	0	0	0	0	0	0	0
**Symbiobacterium**	2	68	0	0	0	0	0	0	0	0	0	0	0	0	0	0	0	0	0	0
**Synechococcus**	1	34	0	0	0	0	0	0	0	0	0	0	0	0	0	0	0	0	0	0
**Synechocystis**	1	40	0	0	0	0	0	0	0	0	0	0	0	0	0	0	0	0	0	0
**Synergistetes**	1	27	0	0	0	0	0	0	0	0	0	0	0	0	0	0	0	0	0	0
**Syntrophobacter**	2	101	0	0	0	0	0	0	0	0	0	0	0	0	0	0	0	0	1	0
**Syntrophobotulus**	2	71	0	0	0	0	0	0	0	0	0	0	0	0	0	0	0	0	0	0
**Syntrophomonas**	2	58	0	0	0	0	0	0	0	0	0	0	0	0	0	0	0	0	0	0
**Syntrophothermus**	2	60	0	0	0	0	0	0	0	0	0	0	0	0	0	0	0	0	0	0
**Syntrophus**	2	66	0	0	0	0	0	0	0	0	0	0	0	0	0	0	0	0	0	0
**Tannerella**	1	8	0	0	0	0	0	0	0	0	0	0	0	0	0	0	0	0	0	0
**Taylorella**	1	43	0	0	0	0	0	0	0	0	0	0	0	0	0	0	0	0	0	0
**Tepidanaerobacter**	2	55	0	0	0	0	0	0	0	0	0	0	0	0	0	0	0	0	0	0
**Teredinibacter**	2	109	0	0	0	0	0	0	0	0	0	0	0	0	0	0	0	0	0	0
**Terrabacter**	1	1	0	0	0	0	0	0	0	0	0	0	0	0	0	0	0	0	0	0
**Terriglobus**	2	83	0	0	0	0	0	0	0	0	0	0	0	0	0	0	0	0	0	0
**Tetragenococcus**	1	12	0	0	0	0	0	0	0	0	0	0	0	0	0	0	0	0	0	0
**Thalassiobium**	1	11	0	0	0	0	0	0	0	0	0	0	0	0	0	0	0	0	0	0
**Thalassobacter**	1	9	0	0	0	0	0	0	0	0	0	0	0	0	0	0	0	0	0	0
**Thalassolituus**	1	12	0	0	0	0	0	0	0	0	0	0	0	0	0	0	0	0	0	0
**Thalassospira**	1	2	0	0	0	0	0	0	0	0	0	0	0	0	0	0	0	0	0	0
**Thauera**	1	111	1	1	1	1	0	0	0	0	0	0	0	0	0	0	0	0	0	0
**Thermacetogenium**	1	7	1	0	0	0	0	0	0	0	0	0	0	0	0	0	0	0	0	0
**Thermaerobacter**	2	79	0	0	0	0	0	0	0	0	0	0	0	0	0	0	0	0	0	0
**Thermanaerovibrio**	2	58	0	0	0	0	0	0	0	0	0	0	0	0	0	0	0	0	0	0
**Thermincola**	2	62	0	0	0	0	0	0	0	0	0	0	0	0	0	0	0	0	0	0
**Thermoanaerobacter**	2	76	0	0	0	0	0	0	0	0	0	0	0	0	0	0	0	0	0	0
**Thermoanaerobacterium**	2	64	0	0	0	0	0	0	0	0	0	0	0	0	0	0	0	0	0	0
**Thermobacillus**	1	11	0	0	0	0	0	0	0	0	0	0	0	0	0	0	0	0	0	0
**Thermobaculum**	2	62	0	0	0	0	0	0	0	0	0	0	0	0	0	0	0	0	0	0
**Thermobifida**	2	72	0	0	0	0	0	0	0	0	0	0	0	0	0	0	0	0	0	0
**Thermobispora**	2	109	0	0	0	0	0	0	0	0	0	0	0	0	0	0	0	0	0	0
**Thermococcus**	1	13	0	0	0	0	0	0	0	0	0	0	0	0	0	0	0	0	0	0
**Thermocrinis**	2	60	0	0	0	0	0	0	0	0	0	0	0	0	0	0	0	0	0	0
**Thermodesulfatator**	1	10	0	0	0	0	0	0	0	0	0	0	0	0	0	0	0	0	1	0
**Thermodesulfobacterium**	1	9	0	0	0	0	0	0	0	0	0	0	0	0	0	0	0	0	1	0

Genus	Cluster Number	Protein Counts	K10218	K10219	K10220	K10221	K10222	K10616	K10617	K10618	K10619	K10621	K10622	K10623	K10676	K10700	K10701	K10702	K11180	K11946
**Thermodesulfobium**	1	13	0	0	0	0	0	0	0	0	0	0	0	0	0	0	0	0	1	0
**Thermodesulfovibrio**	2	60	0	0	0	0	0	0	0	0	0	0	0	0	0	0	0	0	1	0
**Thermofilum**	1	9	0	0	0	0	0	0	0	0	0	0	0	0	0	0	0	0	0	0
**Thermogladius**	1	7	0	0	0	0	0	0	0	0	0	0	0	0	0	0	0	0	0	0
**Thermomicrobium**	2	92	1	0	0	0	0	0	0	0	0	0	0	0	0	0	0	0	0	0
**Thermomonospora**	2	125	0	0	0	0	0	0	0	0	0	0	0	0	0	0	0	1	0	0
**Thermoplasma**	1	11	0	0	0	0	0	0	0	0	0	0	0	0	0	0	0	0	0	0
**Thermoplasmatales**	1	6	0	0	0	0	0	0	0	0	0	0	0	0	0	0	0	0	0	0
**Thermoproteus**	1	12	0	0	0	0	0	0	0	0	0	0	0	0	0	0	0	0	1	0
**Thermosediminibacter**	2	64	0	0	0	0	0	0	0	0	0	0	0	0	0	0	0	0	0	0
**Thermosipho**	2	70	0	0	0	0	0	0	0	0	0	0	0	0	0	0	0	0	0	0
**Thermosphaera**	1	10	0	0	0	0	0	0	0	0	0	0	0	0	0	0	0	0	0	0
**Thermosynechococcus**	1	6	0	0	0	0	0	0	0	0	0	0	0	0	0	0	0	0	0	0
**Thermotoga**	2	80	0	0	0	0	0	0	0	0	0	0	0	0	0	0	0	0	0	0
**Thermovibrio**	2	58	0	0	0	0	0	0	0	0	0	0	0	0	0	0	0	0	0	0
**Thermovirga**	1	9	0	0	0	0	0	0	0	0	0	0	0	0	0	0	0	0	0	0
**Thermus**	2	107	1	0	0	0	0	0	0	0	0	0	0	0	0	0	0	0	0	0
**Thioalkalimicrobium**	1	4	0	0	0	0	0	0	0	0	0	0	0	0	0	0	0	0	0	0
**Thioalkalivibrio**	2	91	0	0	0	0	0	0	0	0	0	0	0	0	0	0	0	0	1	0
**Thiobaca**	1	2	0	0	0	0	0	0	0	0	0	0	0	0	0	0	0	0	1	0
**Thiobacillus**	2	67	0	0	0	0	0	0	0	0	0	0	0	0	0	0	0	0	1	0
**Thiocapsa**	1	2	0	0	0	0	0	0	0	0	0	0	0	0	0	0	0	0	1	0
**Thiocystis**	1	11	0	0	0	0	0	0	0	0	0	0	0	0	0	0	0	0	1	0
**Thioflavicoccus**	1	13	0	0	0	0	0	0	0	0	0	0	0	0	0	0	0	0	1	0
**Thiomicrospira**	2	50	0	0	0	0	0	0	0	0	0	0	0	0	0	0	0	0	0	0
**Thiomonas**	2	85	0	0	0	0	0	0	0	0	0	0	0	0	0	0	0	0	0	0
**Thiorhodococcus**	1	2	0	0	0	0	0	0	0	0	0	0	0	0	0	0	0	0	1	0
**Thiothrix**	1	2	0	0	0	0	0	0	0	0	0	0	0	0	0	0	0	0	0	0
**Tistrella**	1	42	0	0	0	0	0	0	0	0	0	1	1	0	0	0	0	0	0	0
**Tolumonas**	2	100	0	0	0	0	0	0	0	0	0	0	0	0	0	0	0	0	0	0
**Transposon**	1	2	0	0	0	0	0	0	0	0	0	0	0	0	0	0	0	0	0	0
**Treponema**	1	63	0	0	0	0	0	0	0	0	0	0	0	0	0	0	0	0	0	0
**Trichodesmium**	2	71	0	0	0	0	0	0	0	0	0	0	0	0	0	0	0	0	0	0
**Trichormus**	1	12	0	0	0	0	0	0	0	0	0	0	0	0	0	0	0	0	0	0
**Tropheryma**	2	22	0	0	0	0	0	0	0	0	0	0	0	0	0	0	0	0	0	0
**Truepera**	2	104	0	0	0	0	0	0	0	0	0	0	0	0	0	0	0	0	0	0
**Tsukamurella**	2	123	0	0	0	0	0	0	0	0	0	0	0	0	0	0	0	0	0	0
**Turneriella**	1	16	0	0	0	0	0	0	0	0	0	0	0	0	0	0	0	0	0	0
**Uliginosibacterium**	1	2	0	0	0	0	0	0	0	0	0	0	0	0	0	0	0	0	0	0
**Ureaplasma**	1	24	0	0	0	0	0	0	0	0	0	0	0	0	0	0	0	0	0	0
**Ureibacillus**	1	2	0	0	0	0	0	0	0	0	0	0	0	0	0	0	0	0	0	0
**Variovorax**	3	187	1	0	1	1	0	0	0	0	0	0	0	0	0	0	0	0	0	0
**Veillonella**	1	54	0	0	0	0	0	0	0	0	0	0	0	0	0	0	0	0	0	0
**Verminephrobacter**	3	188	0	1	1	1	0	0	0	0	0	0	0	0	0	0	0	0	0	0
**Verrucosispora**	3	126	0	0	0	0	0	0	0	0	0	0	0	0	0	0	0	0	0	0
**Vibrio**	2	195	1	1	1	1	0	0	0	0	0	0	0	0	0	0	0	0	0	0
**Vibrionales**	1	46	0	0	0	0	0	0	0	0	0	0	0	0	0	0	0	0	0	0
**Vitreoscilla**	1	1	0	0	0	0	0	0	0	0	0	0	0	0	0	0	0	0	0	0
**Vulcanisaeta**	1	14	0	0	0	0	0	0	0	0	0	0	0	0	0	0	0	0	1	0
**Waddlia**	2	68	0	0	0	0	0	0	0	0	0	0	0	0	0	0	0	0	0	0
**Wautersia**	1	2	0	0	0	0	0	0	0	0	0	0	0	0	0	0	0	0	0	0
**Weeksella**	2	65	0	0	0	0	0	0	0	0	0	0	0	0	0	0	0	0	0	0

Genus	Cluster Number	Protein Counts	K10218	K10219	K10220	K10221	K10222	K10616	K10617	K10618	K10619	K10621	K10622	K10623	K10676	K10700	K10701	K10702	K11180	K11946
**Weissella**	1	14	0	0	0	0	0	0	0	0	0	0	0	0	0	0	0	0	0	0
**Wenxinia**	1	2	0	0	0	0	0	0	0	0	0	0	0	0	0	0	0	0	0	0
**Wigglesworthia**	1	29	0	0	0	0	0	0	0	0	0	0	0	0	0	0	0	0	0	0
**Wolbachia**	1	32	0	0	0	0	0	0	0	0	0	0	0	0	0	0	0	0	0	0
**Wolinella**	2	51	0	0	0	0	0	0	0	0	0	0	0	0	0	0	0	0	0	0
**Xanthobacter**	3	176	0	0	0	0	0	0	0	0	0	0	0	0	0	0	0	0	0	0
**Xanthobacteraceae**	1	4	0	0	0	0	0	0	0	0	0	0	0	0	0	0	0	0	0	0
**Xanthomonadaceae**	1	39	0	0	0	0	0	0	0	0	0	0	0	0	0	0	0	0	0	0
**Xanthomonas**	2	162	1	0	1	0	0	0	0	0	0	0	0	0	0	0	0	0	0	0
**Xenorhabdus**	2	127	0	0	0	0	0	0	0	0	0	0	0	0	0	0	0	0	0	0
**Xylanimonas**	2	66	0	0	0	0	0	0	0	0	0	0	0	0	0	0	0	0	0	0
**Xylella**	2	52	0	0	0	0	0	0	0	0	0	0	0	0	0	0	0	0	0	0
**Yersinia**	2	145	1	0	0	0	0	0	0	0	0	0	0	0	0	0	0	0	0	0
**Yokenella**	2	61	0	0	0	0	0	0	0	0	0	0	0	0	0	0	0	0	0	0
**Zobellia**	1	28	0	0	0	0	0	0	0	0	0	0	0	0	0	0	0	0	0	0
**Zunongwangia**	2	98	0	0	0	0	0	0	0	0	0	0	0	0	0	0	0	0	0	0
**Zymomonas**	2	67	0	0	0	0	0	0	0	0	0	0	0	0	0	0	0	0	0	0
**Zymophilus**	1	2	0	0	0	0	0	0	0	0	0	0	0	0	0	0	0	0	0	0

Genus	Cluster Number	Protein Counts	K11947	K11948	K11949	K13421	K13953	K14519	K14520	K14579	K14582	K14583	K14584	K14746	K14747	K14748	K14751	K15054	K15058	K15060
Acaricomes	1	2	0	0	0	0	0	0	0	0	0	0	0	0	0	0	0	0	0	0
Acaryochloris	2	88	0	0	0	0	1	0	0	0	0	0	0	0	0	0	0	0	0	0
Acetivibrio	1	2	0	0	0	0	0	0	0	0	0	0	0	0	0	0	0	0	0	0
Acetobacter	2	85	0	0	0	0	1	0	0	0	0	0	0	0	0	0	0	0	0	0
Acetobacteraceae	1	2	0	0	0	0	0	0	0	0	0	0	0	0	0	0	0	0	0	0
Acetobacterium	1	8	0	0	0	0	0	0	0	0	0	0	0	0	0	0	0	0	0	0
Acetohalobium	2	79	0	0	0	0	0	0	0	0	0	0	0	0	0	0	0	0	0	0
Acholeplasma	2	38	0	0	0	0	0	0	0	0	0	0	0	0	0	0	0	0	0	0
Achromobacter	2	192	0	0	0	0	1	0	1	0	0	0	0	0	0	0	0	0	1	0
Acidaminococcus	1	65	0	0	0	0	0	0	0	0	0	0	0	0	0	0	0	0	0	0
Acidianus	1	9	0	0	0	0	0	0	0	0	0	0	0	0	0	0	0	0	0	0
Acidilobus	1	8	0	0	0	0	0	0	0	0	0	0	0	0	0	0	0	0	0	0
Acidimicrobium	2	58	0	0	0	0	0	0	0	0	0	0	0	0	0	0	0	0	0	0
Acidiphilium	3	144	0	0	0	0	1	1	0	0	0	0	0	0	0	0	0	0	0	0
Acidithiobacillus	2	70	0	0	0	0	1	0	0	0	0	0	0	0	0	0	0	0	0	0
Acidobacterium	2	91	0	0	0	0	1	1	0	0	0	0	0	0	0	0	0	0	0	0
Acidocella	1	2	0	0	0	0	0	0	0	0	0	0	0	0	0	0	0	0	0	0
Acidothermus	2	68	0	0	0	0	1	0	0	0	0	0	0	0	0	0	0	0	0	0
Acidovorax	3	217	0	0	0	0	1	1	0	1	0	0	0	0	0	0	0	0	0	0
Aciduliprofundum	1	11	0	0	0	0	1	0	0	0	0	0	0	0	0	0	0	0	0	0
Acinetobacter	3	188	0	0	0	0	1	1	0	0	0	0	0	0	0	0	0	0	0	0
Actinobacillus	2	112	0	0	0	0	1	0	0	0	0	0	0	0	0	0	0	0	0	0
Actinomadura	1	1	0	0	0	0	0	0	0	0	0	0	0	0	0	0	0	0	0	0
Actinoplanes	1	52	0	0	0	0	1	1	0	0	0	0	0	0	0	0	0	0	0	0
Actinopolyspora	1	2	0	0	0	0	0	0	0	0	0	0	0	0	0	0	0	0	0	0
Actinosynnema	2	122	0	0	0	0	1	1	0	0	0	0	0	0	0	0	0	0	0	0
Advenella	1	30	0	0	0	0	1	0	0	0	0	0	0	0	0	0	0	0	0	0
Aequorivita	1	12	0	0	0	0	0	0	0	0	0	0	0	0	0	0	0	0	0	0
Aerococcus	2	57	0	0	0	0	0	0	0	0	0	0	0	0	0	0	0	0	0	0
Aeromonas	2	127	0	0	0	0	0	0	0	0	0	0	0	0	0	0	0	0	0	0
Aeropyrum	1	13	0	0	0	0	1	0	0	0	0	0	0	0	0	0	0	0	0	0
Afipia	1	54	0	0	0	0	0	0	0	0	0	0	0	0	0	0	0	0	0	0
Aggregatibacter	1	81	0	0	0	0	0	0	0	0	0	0	0	0	0	0	0	0	0	0
Agrobacterium	2	242	0	0	0	0	1	1	0	0	0	0	0	0	0	0	0	1	0	0
Ahrensia	1	2	0	0	0	0	0	0	0	0	0	0	0	0	0	0	0	0	0	0
Akkermansia	2	47	0	0	0	0	0	0	0	0	0	0	0	0	0	0	0	0	0	0
Albidiferax	3	144	0	0	0	0	0	0	0	0	0	0	0	0	0	0	0	0	0	0
Alcaligenes	1	21	0	0	0	0	0	0	0	0	0	0	0	0	0	0	0	0	0	0
Alcanivorax	2	146	0	0	0	0	1	0	0	0	0	0	0	0	0	0	0	0	0	0
Alicycliphilus	3	152	0	0	0	0	1	0	0	0	0	0	0	0	0	0	0	0	0	0
Alicyclobacillus	2	91	0	0	0	0	1	0	0	0	0	0	0	0	0	0	0	0	0	0
Aliivibrio	2	118	0	0	0	0	0	0	0	0	0	0	0	0	0	0	0	0	0	0
Alishewanella	1	1	0	0	0	0	0	0	0	0	0	0	0	0	0	0	0	0	0	0
Alistipes	1	52	0	0	0	0	0	0	0	0	0	0	0	0	0	0	0	0	0	0
Alkalilimnicola	2	97	0	0	0	0	1	0	0	0	0	0	0	0	0	0	0	0	0	1
Alkaliphilus	2	62	0	0	0	0	0	0	0	0	0	0	0	0	0	0	0	0	0	0
Allochromatium	2	84	0	0	0	0	0	0	0	0	0	0	0	0	0	0	0	0	0	0
Alphaproteobacteria	1	3	0	0	0	0	0	0	0	0	0	0	0	0	0	0	0	0	0	0
Alteromonadales	1	12	0	0	0	0	0	0	0	0	0	0	0	0	0	0	0	0	0	0
Alteromonas	1	37	0	0	0	0	0	0	0	0	0	0	0	0	0	0	0	0	0	0
Aminobacter	1	4	0	0	0	0	0	0	0	0	0	0	0	0	0	0	0	0	0	0
Aminobacterium	2	59	0	0	0	0	0	0	0	0	0	0	0	0	0	0	0	0	0	0

Genus	Cluster Number	Protein Counts	K11947	K11948	K11949	K13421	K13953	K14519	K14520	K14579	K14582	K14583	K14584	K14746	K14747	K14748	K14751	K15054	K15058	K15060
**Ammonifex**	2	50	0	0	0	0	1	0	0	0	0	0	0	0	0	0	0	0	0	0
**Amphibacillus**	1	13	0	0	0	0	1	0	0	0	0	0	0	0	0	0	0	0	0	0
**Amphritea**	1	2	0	0	0	0	0	0	0	0	0	0	0	0	0	0	0	0	0	0
**Amycolatopsis**	2	201	0	0	0	0	1	1	0	0	0	0	0	0	0	0	0	0	0	0
**Amycolicicoccus**	1	43	0	0	0	0	1	0	0	0	0	0	0	0	0	0	0	0	0	0
**Anabaena**	2	92	0	0	0	1	1	0	0	0	0	0	0	0	0	0	0	0	0	0
**Anaerobaculum**	1	11	0	0	0	0	0	0	0	0	0	0	0	0	0	0	0	0	0	0
**Anaerococcus**	2	46	0	0	0	0	0	0	0	0	0	0	0	0	0	0	0	0	0	0
**Anaerolinea**	2	66	0	0	0	0	1	0	0	0	0	0	0	0	0	0	0	0	0	0
**Anaeromyxobacter**	3	117	0	0	0	0	1	0	0	0	0	0	0	0	0	0	0	0	0	0
**Anaerostipes**	1	44	0	0	0	0	0	0	0	0	0	0	0	0	0	0	0	0	0	0
**Anaerotruncus**	1	2	0	0	0	0	0	0	0	0	0	0	0	0	0	0	0	0	0	0
**Anaplasma**	1	35	0	0	0	0	0	0	0	0	0	0	0	0	0	0	0	0	0	0
**Ancylobacter**	1	3	0	0	0	0	0	0	0	0	0	0	0	0	0	0	0	0	0	0
**Aneurinibacillus**	1	1	0	0	0	0	0	0	0	0	0	0	0	0	0	0	0	0	0	0
**Angomonas**	1	1	0	0	0	0	0	0	0	0	0	0	0	0	0	0	0	0	0	0
**Anoxybacillus**	2	72	0	0	0	0	1	0	0	0	0	0	0	0	0	0	0	0	0	0
**Aquifex**	2	45	0	0	0	0	0	0	0	0	0	0	0	0	0	0	0	0	0	0
**Aquimarina**	1	2	0	0	0	0	0	0	0	0	0	0	0	0	0	0	0	0	0	0
**Arcanobacterium**	2	45	0	0	0	0	0	0	0	0	0	0	0	0	0	0	0	0	0	0
**Archaeoglobus**	1	14	0	0	0	0	0	0	0	0	0	0	0	0	0	0	0	0	0	0
**Arcobacter**	2	101	0	0	0	0	0	0	0	0	0	0	0	0	0	0	0	0	0	0
**Aromatoleum**	3	133	0	0	0	0	0	0	0	0	0	0	0	1	1	0	0	0	0	0
**Arsenophonus**	1	4	0	0	0	0	0	0	0	0	0	0	0	0	0	0	0	0	0	0
**Arthrobacter**	2	206	0	0	0	0	1	1	0	0	0	0	0	0	0	0	0	0	0	0
**Arthrospira**	2	67	0	0	0	1	0	0	0	0	0	0	0	0	0	0	0	0	0	0
**Aster**	1	10	0	0	0	0	0	0	0	0	0	0	0	0	0	0	0	0	0	0
**Asticcacaulis**	2	103	0	0	0	0	1	0	0	0	0	0	0	0	0	0	0	0	0	0
**Atopobium**	1	28	0	0	0	0	0	0	0	0	0	0	0	0	0	0	0	0	0	0
**Aurantimonas**	1	2	0	0	0	0	0	0	0	0	0	0	0	0	0	0	0	0	0	0
**Avibacterium**	1	17	0	0	0	0	0	0	0	0	0	0	0	0	0	0	0	0	0	0
**Azoarcus**	3	172	0	0	0	0	1	0	0	0	0	0	0	0	0	0	0	1	0	0
**Azorhizobium**	3	149	0	0	0	0	1	0	0	0	0	0	0	0	0	0	0	0	0	0
**Azorhizophilus**	1	4	0	0	0	0	0	0	0	0	0	0	0	0	0	0	0	0	0	0
**Azospira**	1	19	0	0	0	0	1	0	0	0	0	0	0	0	0	0	0	0	0	0
**Azospirillum**	2	198	0	0	0	0	1	1	0	0	0	0	0	0	0	0	0	0	0	0
**Azotobacter**	3	183	0	0	0	0	1	1	0	0	0	0	0	0	0	1	0	0	0	0
**Bacillales**	1	9	0	0	0	0	0	0	0	0	0	0	0	0	0	0	0	0	0	0
**Bacilli**	1	3	0	0	0	0	0	0	0	0	0	0	0	0	0	0	0	0	0	0
**Bacillus**	2	237	0	0	0	0	1	0	0	0	0	0	0	0	0	0	0	0	0	0
**Bacteria**	2	86	0	0	0	0	1	0	0	0	0	0	0	0	0	0	0	0	0	0
**Bacteriovorax**	2	98	0	0	0	0	0	0	0	0	0	0	0	0	0	0	0	0	0	0
**Bacteroidales**	2	59	0	0	0	0	0	0	0	0	0	0	0	0	0	0	0	0	0	0
**Bacteroides**	2	96	0	0	0	0	0	0	0	0	0	0	0	0	0	0	0	0	0	0
**Bacteroidetes**	1	2	0	0	0	0	0	0	0	0	0	0	0	0	0	0	0	0	0	0
**Bartonella**	2	50	0	0	0	0	0	0	0	0	0	0	0	0	0	0	0	0	0	0
**Baumannia**	2	24	0	0	0	0	0	0	0	0	0	0	0	0	0	0	0	0	0	0
**Bdellovibrio**	2	87	0	0	0	0	1	0	0	0	0	0	0	0	0	0	0	0	0	0
**Beijerinckia**	3	123	0	0	0	0	1	1	0	0	0	0	0	0	0	0	0	0	0	0
**Belliella**	1	14	0	0	0	0	0	0	0	0	0	0	0	0	0	0	0	0	0	0
**Beutenbergia**	2	82	0	0	0	0	0	1	0	0	0	0	0	0	0	0	0	0	0	0
**Bifidobacterium**	2	61	0	0	0	0	1	0	0	0	0	0	0	0	0	0	0	0	0	0

Genus	Cluster Number	Protein Counts	K11947	K11948	K11949	K13421	K13953	K14519	K14520	K14579	K14582	K14583	K14584	K14746	K14747	K14748	K14751	K15054	K15058	K15060
Bizionia	1	1	0	0	0	0	0	0	0	0	0	0	0	0	0	0	0	0	0	0
Blastococcus	1	39	0	0	0	0	0	1	0	0	0	0	0	0	0	0	0	0	0	0
Blattabacterium	1	23	0	0	0	1	0	0	0	0	0	0	0	0	0	0	0	0	0	0
Blautia	1	22	0	0	0	0	0	0	0	0	0	0	0	0	0	0	0	0	0	0
Bordetella	3	227	0	0	0	0	1	1	0	0	0	0	0	0	0	0	0	1	1	0
Borrelia	1	32	0	0	0	0	0	0	0	0	0	0	0	0	0	0	0	0	0	0
Brachybacterium	1	18	0	0	0	0	0	0	0	0	0	0	0	0	0	0	0	0	0	0
Brachymonas	1	4	0	0	0	0	0	0	0	0	0	0	0	0	0	0	0	0	0	0
Brachyspira	2	79	0	0	0	0	0	0	0	0	0	0	0	0	0	0	0	0	0	0
Bradyrhizobiaceae	1	20	0	0	0	0	0	0	0	0	0	0	0	0	0	0	0	0	0	0
Bradyrhizobium	3	254	0	0	0	0	1	0	1	0	0	0	0	0	0	0	0	0	0	0
Brenneria	1	20	0	0	0	0	0	0	0	0	0	0	0	0	0	0	0	0	0	0
Brevibacillus	2	122	0	0	0	0	1	0	0	0	0	0	0	0	0	0	0	0	0	0
Brevibacterium	1	4	0	0	0	0	0	0	0	0	0	0	0	0	0	0	0	0	0	0
Brevundimonas	2	105	0	0	0	0	0	0	0	0	0	0	0	0	0	0	0	0	0	0
Brucella	3	138	0	0	0	0	1	0	0	0	0	0	0	0	0	0	0	0	0	0
Buchnera	1	26	0	0	0	0	0	0	0	0	0	0	0	0	0	0	0	0	0	0
Burkholderia	3	424	0	0	0	0	1	1	1	1	1	1	0	0	0	0	0	1	1	1
Burkholderiaceae	3	143	0	0	0	0	0	0	0	0	0	0	0	0	0	0	0	1	1	0
Burkholderiales	1	24	0	0	0	0	0	0	0	0	0	0	0	0	0	0	0	0	0	0
Buttiauxella	1	2	0	0	0	0	0	0	0	0	0	0	0	0	0	0	0	0	0	0
Butyrivibrio	2	67	0	0	0	0	0	0	0	0	0	0	0	0	0	0	0	0	0	0
Caldanaerobacter	2	63	0	0	0	0	0	0	0	0	0	0	0	0	0	0	0	0	0	0
Caldicellulosiruptor	2	75	0	0	0	0	1	0	0	0	0	0	0	0	0	0	0	0	0	0
Caldilinea	1	23	0	0	0	1	1	0	0	0	0	0	0	0	0	0	0	0	0	0
Caldimonas	1	6	0	0	0	0	0	0	0	0	0	0	0	0	0	0	0	0	0	0
Caldisericum	1	10	0	0	0	0	1	0	0	0	0	0	0	0	0	0	0	0	0	0
Caldisphaera	1	7	0	0	0	0	0	0	0	0	0	0	0	0	0	0	0	0	0	0
Calditerrivibrio	2	74	0	0	0	0	0	0	0	0	0	0	0	0	0	0	0	0	0	0
Caldivirga	1	13	0	0	0	0	1	0	0	0	0	0	0	0	0	0	0	0	0	0
Calothrix	1	43	0	0	0	1	0	0	0	0	0	0	0	0	0	0	0	0	0	0
Calyptogena	2	34	0	0	0	0	0	0	0	0	0	0	0	0	0	0	0	0	0	0
Campylobacter	2	80	0	0	0	0	0	0	0	0	0	0	0	0	0	0	0	0	0	0
Candidatus	2	290	0	0	0	0	1	0	0	0	0	0	0	0	0	0	0	0	0	0
Capnocytophaga	1	50	0	0	0	0	0	0	0	0	0	0	0	0	0	0	0	0	0	0
Carbophilus	1	2	0	0	0	0	0	0	0	0	0	0	0	0	0	0	0	0	0	0
Carboxydibrachium	1	14	0	0	0	0	0	0	0	0	0	0	0	0	0	0	0	0	0	0
Carboxydothermus	2	68	0	0	0	0	0	0	0	0	0	0	0	0	0	0	0	0	0	0
Carica	1	2	0	0	0	0	0	0	0	0	0	0	0	0	0	0	0	0	0	0
Carnobacterium	2	71	0	0	0	0	0	0	0	0	0	0	0	0	0	0	0	0	0	0
Catenibacterium	1	2	0	0	0	0	0	0	0	0	0	0	0	0	0	0	0	0	0	0
Catenovulum	1	2	0	0	0	0	0	0	0	0	0	0	0	0	0	0	0	0	0	0
Catenulispora	3	159	0	0	0	0	1	1	0	0	0	0	0	0	0	0	0	0	0	0
Caulobacter	3	168	0	0	0	0	1	1	0	0	0	0	0	0	0	0	0	0	0	0
Cedecea	1	45	0	0	0	0	0	0	0	0	0	0	0	0	0	0	0	0	0	0
Cellulomonas	2	96	0	0	0	0	0	0	0	0	0	0	0	0	0	0	0	0	0	0
Cellulophaga	2	105	0	0	0	0	0	1	0	0	0	0	0	0	0	0	0	0	0	0
Cellulosilyticum	1	14	0	0	0	0	0	0	0	0	0	0	0	0	0	0	0	0	0	0
Cellvibrio	2	85	0	0	0	0	0	0	0	0	0	0	0	0	0	0	0	0	0	0
Cenarchaeum	1	7	0	0	0	0	1	0	0	0	0	0	0	0	0	0	0	0	0	0
Chamaesiphon	1	13	0	0	0	0	0	0	0	0	0	0	0	0	0	0	0	0	0	0
Chelativorans	3	136	0	0	0	0	1	1	0	0	0	0	0	0	0	0	0	0	0	0

Genus	Cluster Number	Protein Counts	K11947	K11948	K11949	K13421	K13953	K14519	K14520	K14579	K14582	K14583	K14584	K14746	K14747	K14748	K14751	K15054	K15058	K15060
Chelatococcus	1	2	0	0	0	0	0	0	0	0	0	0	0	0	0	0	0	0	0	0
Chitinophaga	2	98	0	0	0	0	0	1	0	0	0	0	0	0	0	0	0	0	0	0
Chlamydia	1	36	0	0	0	0	0	0	0	0	0	0	0	0	0	0	0	0	0	0
Chlamydomonas	1	3	0	0	0	0	0	0	0	0	0	0	0	0	0	0	0	0	0	0
Chlamydophila	1	31	0	0	0	0	0	0	0	0	0	0	0	0	0	0	0	0	0	0
Chlorobaculum	2	61	0	0	0	0	1	0	0	0	0	0	0	0	0	0	0	0	0	0
Chlorobium	2	89	0	0	0	0	1	0	0	0	0	0	0	0	0	0	0	0	0	0
Chlorobium/Pelodictyon	1	2	0	0	0	0	0	0	0	0	0	0	0	0	0	0	0	0	0	0
Chloroflexus	2	105	0	0	0	0	1	0	0	0	0	0	0	0	0	0	0	0	0	0
Chlorogloeopsis	1	9	0	0	0	0	0	0	0	0	0	0	0	0	0	0	0	0	0	0
Chloroherpeton	2	59	0	0	0	0	0	0	0	0	0	0	0	0	0	0	0	0	0	0
Chromobacterium	2	117	0	0	0	0	0	1	0	0	0	0	0	0	0	0	0	0	0	0
Chromohalobacter	2	145	0	0	0	0	1	1	0	0	0	0	0	0	0	0	0	1	0	0
Chroococcidiopsis	1	24	0	0	0	1	1	0	0	0	0	0	0	0	0	0	0	0	0	0
Chryseobacterium	2	104	0	0	0	0	0	0	0	0	0	0	0	0	0	0	0	0	0	0
Citreicella	1	13	0	0	0	0	0	0	0	0	0	0	0	0	0	0	0	0	0	0
Citrobacter	2	190	0	0	0	0	0	1	0	0	0	0	0	0	0	0	0	0	0	0
Clavibacter	2	83	0	0	0	0	1	1	0	0	0	0	0	0	0	0	0	0	0	0
Clonorchis	1	1	0	0	0	0	0	0	0	0	0	0	0	0	0	0	0	0	0	0
Clostridiales	2	77	0	0	0	0	0	0	0	0	0	0	0	0	0	0	0	0	0	0
Clostridium	2	191	0	0	0	0	1	0	0	0	0	0	0	0	0	0	0	0	0	0
Cohnella	1	2	0	0	0	0	0	0	0	0	0	0	0	0	0	0	0	0	0	0
Coleofasciculus	1	4	0	0	0	0	0	0	0	0	0	0	0	0	0	0	0	0	0	0
Collimonas	1	35	0	0	0	0	0	1	0	0	0	0	0	0	0	0	0	0	0	0
Colwellia	2	130	0	0	0	0	1	1	0	0	0	0	0	0	0	0	0	0	0	0
Comamonas	2	196	0	0	0	0	1	1	0	1	1	0	0	0	0	0	0	0	0	0
Conexibacter	3	117	0	0	0	0	1	1	0	0	0	0	0	0	0	1	0	0	0	0
Coprobacillus	1	2	0	0	0	0	0	0	0	0	0	0	0	0	0	0	0	0	0	0
Coprococcus	2	76	0	0	0	0	0	0	0	0	0	0	0	0	0	0	0	0	0	0
Coprothermobacter	2	45	0	0	0	0	0	0	0	0	0	0	0	0	0	0	0	0	0	0
Coraliomargarita	2	68	0	0	0	0	0	0	0	0	0	0	0	0	0	0	0	0	0	0
Corallococcus	1	29	0	0	0	0	1	0	0	0	0	0	0	0	0	0	0	0	0	0
Coriobacterium	2	36	0	0	0	0	0	0	0	0	0	0	0	0	0	0	0	0	0	0
Corynebacterineae	1	5	0	0	0	0	0	0	0	0	0	0	0	0	0	0	0	0	0	0
Corynebacterium	2	175	0	0	0	0	1	1	0	0	0	0	0	0	0	0	0	0	0	0
Coxiella	2	54	0	0	0	0	0	0	0	0	0	0	0	0	0	0	0	0	0	0
Crinalium	1	21	0	0	0	1	0	0	0	0	0	0	0	0	0	0	0	0	0	0
Croceibacter	2	80	0	0	0	0	0	0	0	0	0	0	0	0	0	0	0	0	0	0
Crocosphaera	1	6	0	0	0	0	0	0	0	0	0	0	0	0	0	0	0	0	0	0
Cronobacter	2	120	0	0	0	0	1	0	0	0	0	0	0	0	0	0	0	0	0	0
Cryptobacterium	2	39	0	0	0	0	0	0	0	0	0	0	0	0	0	0	0	0	0	0
Cucumis	1	9	0	0	0	0	0	0	0	0	0	0	0	0	0	0	0	0	0	0
Cupriavidus	3	317	0	0	0	0	1	1	0	0	0	0	0	0	0	0	0	1	0	0
Curtobacterium	1	5	0	0	0	0	0	0	0	0	0	0	0	0	0	0	0	0	0	0
Curvibacter	1	27	0	0	0	0	0	0	0	0	0	0	0	0	0	0	0	0	0	0
Cyanobacterium	1	9	0	0	0	0	0	0	0	0	0	0	0	0	0	0	0	0	0	0
Cyanobium	1	10	0	0	0	1	1	0	0	0	0	0	0	0	0	0	0	0	0	0
Cyanothece	1	34	0	0	0	1	1	0	0	0	0	0	0	0	0	0	0	0	0	0
Cyclobacterium	1	19	0	0	0	0	0	1	0	0	0	0	0	0	0	0	0	0	0	0
Cycloclasticus	1	21	0	0	0	0	0	0	0	0	0	0	0	0	0	1	0	0	0	0
Cylindrospermopsis	1	4	0	0	0	0	0	0	0	0	0	0	0	0	0	0	0	0	0	0
Cylindrospermum	1	30	0	0	0	1	1	0	0	0	0	0	0	0	0	0	0	0	0	0

Genus	Cluster Number	Protein Counts	K11947	K11948	K11949	K13421	K13953	K14519	K14520	K14579	K14582	K14583	K14584	K14746	K14747	K14748	K14751	K15054	K15058	K15060
Cytophaga	2	69	0	0	0	0	0	0	0	0	0	0	0	0	0	0	0	0	0	0
Dactylococcopsis	1	8	0	0	0	0	0	0	0	0	0	0	0	0	0	0	0	0	0	0
Dechloromonas	1	40	0	0	0	0	0	0	0	0	0	0	0	0	0	1	0	0	0	0
Dechlorosoma	1	19	0	0	0	0	1	0	0	0	0	0	0	0	0	0	0	0	0	0
Deferribacter	2	73	0	0	0	0	1	0	0	0	0	0	0	0	0	0	0	0	0	0
Dehalobacter	1	9	0	0	0	0	0	0	0	0	0	0	0	0	0	0	0	0	0	0
Dehalococcoides	2	41	0	0	0	0	0	0	0	0	0	0	0	0	0	0	0	0	0	0
Dehalogenimonas	1	37	0	0	0	0	0	0	0	0	0	0	0	0	0	0	0	0	0	0
Deinococcus	2	140	0	0	0	0	0	1	0	0	0	0	0	0	0	0	0	0	0	0
Delftia	3	177	0	0	0	0	1	0	0	1	0	0	0	0	0	0	0	0	0	0
Denitrovibrio	2	67	0	0	0	0	0	0	0	0	0	0	0	0	0	0	0	0	0	0
Desulfarculus	2	92	0	0	0	0	0	0	0	0	0	0	0	0	0	0	0	0	0	0
Desulfatibacillum	3	84	0	0	0	0	0	0	0	0	0	0	0	0	0	0	0	0	0	0
Desulfitobacterium	2	102	0	0	0	0	1	0	0	0	0	0	0	0	0	0	0	0	0	0
Desulfobacca	2	65	0	0	0	0	1	0	0	0	0	0	0	0	0	0	0	0	0	0
Desulfobacterium	2	113	0	0	0	0	0	0	0	0	0	0	0	0	0	0	0	0	0	0
Desulfobacula	1	21	0	0	0	0	0	0	0	0	0	0	0	0	0	0	0	0	0	0
Desulfobulbus	2	88	0	0	0	0	0	0	0	0	0	0	0	0	0	0	0	0	0	0
Desulfocapsa	1	9	0	0	0	0	0	0	0	0	0	0	0	0	0	0	0	0	0	0
Desulfococcus	2	80	0	0	0	0	0	0	0	0	0	0	0	0	0	0	0	0	0	0
Desulfohalobium	2	52	0	0	0	0	0	0	0	0	0	0	0	0	0	0	0	0	0	0
Desulfomicrobium	2	65	0	0	0	0	0	0	0	0	0	0	0	0	0	0	0	0	0	0
Desulfomonile	1	25	0	0	0	0	1	0	0	0	0	0	0	0	0	0	0	0	0	0
Desulforhabdus	1	2	0	0	0	0	0	0	0	0	0	0	0	0	0	0	0	0	0	0
Desulfosporosinus	1	30	0	0	0	0	0	0	0	0	0	0	0	0	0	0	0	0	0	0
Desulfotalea	2	66	0	0	0	0	0	0	0	0	0	0	0	0	0	0	0	0	0	0
Desulfotomaculum	2	98	0	0	0	0	0	0	0	0	0	0	0	0	0	0	0	0	0	0
Desulfovibrio	2	144	0	0	0	0	0	0	0	0	0	0	0	0	0	0	0	0	0	0
Desulfurispirillum	2	78	0	0	0	0	0	0	0	0	0	0	0	0	0	0	0	0	0	0
Desulfurivibrio	2	71	0	0	0	0	0	0	0	0	0	0	0	0	0	0	0	0	0	0
Desulfurobacterium	2	53	0	0	0	0	0	0	0	0	0	0	0	0	0	0	0	0	0	0
Desulfurococcus	1	11	0	0	0	0	1	0	0	0	0	0	0	0	0	0	0	0	0	0
Diaphorobacter	1	16	0	0	0	0	0	0	0	0	0	0	0	0	0	0	0	0	0	0
Dichelobacter	2	45	0	0	0	0	0	0	0	0	0	0	0	0	0	0	0	0	0	0
Dickeya	2	155	0	0	0	0	0	0	0	0	0	0	0	0	0	0	0	0	0	0
Dictyoglomus	2	62	0	0	0	0	1	0	0	0	0	0	0	0	0	0	0	0	0	0
Dinoroseobacter	3	148	0	0	0	0	1	1	0	0	0	0	0	0	0	0	0	0	0	0
Dokdonia	1	3	0	0	0	0	0	0	0	0	0	0	0	0	0	0	0	0	0	0
Dorea	1	7	0	0	0	0	0	0	0	0	0	0	0	0	0	0	0	0	0	0
Dyadobacter	2	83	0	0	0	0	0	1	0	0	0	0	0	0	0	0	0	0	0	0
Echinicola	1	20	0	0	0	0	1	1	0	0	0	0	0	0	0	0	0	0	0	0
Edwardsiella	2	126	0	0	0	0	0	0	0	0	0	0	0	0	0	0	0	0	0	0
Eggerthella	1	51	0	0	0	0	0	0	0	0	0	0	0	0	0	0	0	0	0	0
Ehrlichia	2	37	0	0	0	0	0	0	0	0	0	0	0	0	0	0	0	0	0	0
Eikenella	1	4	0	0	0	0	0	0	0	0	0	0	0	0	0	0	0	0	0	0
Elusimicrobium	2	44	0	0	0	0	0	0	0	0	0	0	0	0	0	0	0	0	0	0
Emticicia	1	21	0	0	0	0	0	1	0	0	0	0	0	0	0	0	0	0	0	0
Enhydrobacter	1	1	0	0	0	0	0	0	0	0	0	0	0	0	0	0	0	0	0	0
Ensifer	1	2	0	0	0	0	0	0	0	0	0	0	0	0	0	0	0	0	0	0
Enterobacter	2	202	0	0	0	0	1	1	0	0	0	0	0	0	0	0	0	0	0	0
Enterobacteriaceae	2	211	0	0	0	0	1	1	0	0	0	0	0	0	0	0	0	0	0	0
Enterococcus	1	187	0	0	0	0	1	0	0	0	0	0	0	0	0	0	0	0	0	0

Genus	Cluster Number	Protein Counts	K11947	K11948	K11949	K13421	K13953	K14519	K14520	K14579	K14582	K14583	K14584	K14746	K14747	K14748	K14751	K15054	K15058	K15060
Enterovibrio	1	15	0	0	0	0	0	0	0	0	0	0	0	0	0	0	0	0	0	0
Erwinia	2	151	0	0	0	0	1	1	0	0	0	0	0	0	0	0	0	0	0	0
Erysipelothrix	1	9	0	0	0	0	0	0	0	0	0	0	0	0	0	0	0	0	0	0
Erysipelotrichaceae	1	4	0	0	0	0	0	0	0	0	0	0	0	0	0	0	0	0	0	0
Erythrobacter	2	80	0	0	0	0	0	0	0	0	0	0	0	0	0	0	0	0	0	0
Escherichia	2	242	0	0	0	0	1	0	0	0	0	0	0	0	0	0	0	0	0	0
Ethanoligenens	2	69	0	0	0	0	0	0	0	0	0	0	0	0	0	0	0	0	0	0
Eubacteriaceae	1	2	0	0	0	0	0	0	0	0	0	0	0	0	0	0	0	0	0	0
Eubacterium	2	103	0	0	0	0	0	0	0	0	0	0	0	0	0	0	0	0	0	0
Eudoraea	1	4	0	0	0	0	0	0	0	0	0	0	0	0	0	0	0	0	0	0
Euryarchaeota	1	1	0	0	0	0	0	0	0	0	0	0	0	0	0	0	0	0	0	0
Exiguobacterium	2	93	0	0	0	0	1	0	0	0	0	0	0	0	0	0	0	0	0	0
Faecalibacterium	1	12	0	0	0	0	0	0	0	0	0	0	0	0	0	0	0	0	0	0
Ferrimonas	2	115	0	0	0	0	0	1	0	0	0	0	0	0	0	0	0	0	0	0
Ferroglobus	1	14	0	0	0	0	0	0	0	0	0	0	0	0	0	0	0	0	0	0
Ferroplasma	1	11	0	0	0	0	0	0	0	0	0	0	0	0	0	0	0	0	0	0
Fervidicoccus	1	7	0	0	0	0	0	0	0	0	0	0	0	0	0	0	0	0	0	0
Fervidobacterium	2	54	0	0	0	0	0	0	0	0	0	0	0	0	0	0	0	0	0	0
Fibrella	1	17	0	0	0	0	0	1	0	0	0	0	0	0	0	0	0	0	0	0
Fibrobacter	2	52	0	0	0	0	0	0	0	0	0	0	0	0	0	0	0	0	0	0
Filifactor	1	9	0	0	0	0	0	0	0	0	0	0	0	0	0	0	0	0	0	0
Finegoldia	2	55	0	0	0	0	0	0	0	0	0	0	0	0	0	0	0	0	0	0
Firmicutes	1	15	0	0	0	0	0	0	0	0	0	0	0	0	0	0	0	0	0	0
Fischerella	1	15	0	0	0	0	0	0	0	0	0	0	0	0	0	0	0	0	0	0
Flavobacteria	1	7	0	0	0	0	0	0	0	0	0	0	0	0	0	0	0	0	0	0
Flavobacteriaceae	2	69	0	0	0	1	0	0	0	0	0	0	0	0	0	0	0	0	0	0
Flavobacteriales	1	3	0	0	0	0	0	0	0	0	0	0	0	0	0	0	0	0	0	0
Flavobacterium	2	103	0	0	0	0	1	1	0	0	0	0	0	0	0	0	0	0	0	0
Flexibacter	1	17	0	0	0	0	0	0	0	0	0	0	0	0	0	0	0	0	0	0
Flexistipes	1	11	0	0	0	0	1	0	0	0	0	0	0	0	0	0	0	0	0	0
Fluoribacter	1	6	0	0	0	0	0	0	0	0	0	0	0	0	0	0	0	0	0	0
Fluviicola	2	69	0	0	0	0	0	1	0	0	0	0	0	0	0	0	0	0	0	0
Formosa	1	2	0	0	0	0	0	0	0	0	0	0	0	0	0	0	0	0	0	0
Francisella	2	88	0	0	0	0	0	0	0	0	0	0	0	0	0	0	0	0	0	0
Frankia	3	202	0	0	0	0	1	1	1	0	0	0	0	0	0	0	0	0	0	0
Frateuria	1	25	0	0	0	0	1	1	0	0	0	0	0	0	0	0	0	0	0	0
Fructobacillus	1	2	0	0	0	0	0	0	0	0	0	0	0	0	0	0	0	0	0	0
Fulvimarina	1	2	0	0	0	0	0	0	0	0	0	0	0	0	0	0	0	0	0	0
Fusobacterium	1	48	0	0	0	0	0	0	0	0	0	0	0	0	0	0	0	0	0	0
Gallibacterium	2	87	0	0	0	0	0	0	0	0	0	0	0	0	0	0	0	0	0	0
Gallionella	2	67	0	0	0	0	0	0	0	0	0	0	0	0	0	0	0	0	0	0
Gammaproteobacteria	1	9	0	0	0	0	0	0	0	0	0	0	0	0	0	0	0	0	0	0
Gardnerella	1	31	0	0	0	0	1	0	0	0	0	0	0	0	0	0	0	0	0	0
Geitlerinema	1	9	0	0	0	0	0	0	0	0	0	0	0	0	0	0	0	0	0	0
Gemmatimonas	2	93	0	0	0	0	1	0	0	0	0	0	0	0	0	0	0	0	0	0
Geobacillus	2	168	0	0	0	0	1	0	0	0	0	0	0	0	0	0	0	0	0	0
Geobacter	2	150	0	0	0	0	1	0	0	0	0	0	0	0	0	0	0	0	0	0
Geodermatophilus	2	136	0	0	0	0	1	1	0	0	0	0	0	0	0	0	0	0	0	0
Geopsychrobacter	1	2	0	0	0	0	0	0	0	0	0	0	0	0	0	0	0	0	0	0
Gillisia	1	11	0	0	0	0	0	0	0	0	0	0	0	0	0	0	0	0	0	0
Gilvimarinus	1	4	0	0	0	0	0	0	0	0	0	0	0	0	0	0	0	0	0	0
Glaciecola	2	117	0	0	0	0	0	0	1	0	0	0	0	0	0	0	0	0	0	0

Genus	Cluster Number	Protein Counts	K11947	K11948	K11949	K13421	K13953	K14519	K14520	K14579	K14582	K14583	K14584	K14746	K14747	K14748	K14751	K15054	K15058	K15060
Gloeobacter	2	67	0	0	0	1	0	0	0	0	0	0	0	0	0	0	0	0	0	0
Gloeocapsa	1	20	0	0	0	1	1	0	0	0	0	0	0	0	0	0	0	0	0	0
Gloeothece	1	1	0	0	0	0	0	0	0	0	0	0	0	0	0	0	0	0	0	0
Gluconacetobacter	2	96	0	0	0	0	1	0	0	0	0	0	0	0	0	0	0	0	0	0
Gluconobacter	2	76	0	0	0	0	1	0	0	0	0	0	0	0	0	0	0	0	0	0
Gordonia	1	158	0	0	0	0	1	1	1	0	0	0	0	0	0	0	0	0	0	0
Gordonibacter	1	32	0	0	0	0	0	0	0	0	0	0	0	0	0	0	0	0	0	0
Gramella	2	104	0	0	0	0	1	0	0	0	0	0	0	0	0	0	0	0	0	0
Granulibacter	2	90	0	0	0	0	0	0	0	0	0	0	0	0	0	0	0	0	0	0
Granulicella	2	98	0	0	0	0	1	1	0	0	0	0	0	0	0	0	0	0	0	0
Grimontia	1	9	0	0	0	0	0	0	0	0	0	0	0	0	0	0	0	0	0	0
Haemophilus	2	117	0	0	0	0	0	0	0	0	0	0	0	0	0	0	0	0	0	0
Hafnia	1	29	0	0	0	0	0	0	0	0	0	0	0	0	0	0	0	0	0	0
Hahella	3	130	0	0	0	0	0	0	0	0	0	0	0	0	0	0	0	0	0	0
Halalkalicoccus	1	18	0	0	0	0	1	0	0	0	0	0	0	0	0	0	0	0	0	0
Halanaerobium	2	89	0	0	0	0	0	0	0	0	0	0	0	0	0	0	0	0	0	0
Haliangium	2	108	0	0	0	0	0	0	0	0	0	0	0	0	0	0	0	0	0	0
Haliscomenobacter	2	107	0	0	0	0	0	1	0	0	0	0	0	0	0	0	0	0	0	0
Haloarcula	1	22	0	0	0	0	1	0	0	0	0	0	0	0	0	0	0	0	0	0
Halobacillus	1	19	0	0	0	0	1	0	0	0	0	0	0	0	0	0	0	0	0	0
Halobacteriaceae	1	1	0	0	0	0	0	0	0	0	0	0	0	0	0	0	0	0	0	0
Halobacterium	1	10	0	0	0	0	0	0	0	0	0	0	0	0	0	0	0	0	0	0
Halobacteroides	1	14	0	0	0	0	0	0	0	0	0	0	0	0	0	0	0	0	0	0
Haloferax	1	20	0	0	0	0	0	0	0	0	0	0	0	0	0	0	0	0	0	0
Halogeometricum	1	12	0	0	0	0	0	0	0	0	0	0	0	0	0	0	0	0	0	0
Halomicrobium	1	10	0	0	0	0	0	0	0	0	0	0	0	0	0	0	0	0	0	0
Halomonas	1	149	0	0	0	0	1	1	0	0	0	0	0	0	0	0	0	1	0	0
Halopiger	1	16	0	0	0	0	1	0	0	0	0	0	0	0	0	0	0	0	0	0
Haloquadratum	1	19	0	0	0	0	1	0	0	0	0	0	0	0	0	0	0	0	0	0
Halorhabdus	1	10	0	0	0	0	0	0	0	0	0	0	0	0	0	0	0	0	0	0
Halorhodospira	2	71	0	0	0	0	0	0	0	0	0	0	0	0	0	0	0	0	0	0
Halorubrum	1	16	0	0	0	0	1	0	0	0	0	0	0	0	0	0	0	0	0	0
Haloterrigena	1	19	0	0	0	0	1	0	0	0	0	0	0	0	0	0	0	0	0	0
Halothece	1	9	0	0	0	1	1	0	0	0	0	0	0	0	0	0	0	0	0	0
Halothermothrix	2	50	0	0	0	0	0	0	0	0	0	0	0	0	0	0	0	0	0	0
Halothiobacillus	2	65	0	0	0	0	0	0	0	0	0	0	0	0	0	0	0	0	0	0
Halovivax	1	12	0	0	0	0	0	0	0	0	0	0	0	0	0	0	0	0	0	0
Helicobacter	2	79	0	0	0	0	0	0	0	0	0	0	0	0	0	0	0	0	0	0
Heliobacillus	1	4	0	0	0	0	0	0	0	0	0	0	0	0	0	0	0	0	0	0
Heliobacterium	2	51	0	0	0	0	0	0	0	0	0	0	0	0	0	0	0	0	0	0
Herbaspirillum	2	172	0	0	0	0	1	1	0	0	0	0	0	0	0	0	0	1	0	0
Herminiimonas	3	89	0	0	0	0	1	0	0	0	0	0	0	0	0	0	0	0	0	0
Herpetosiphon	3	90	0	0	0	0	0	1	0	0	0	0	0	0	0	0	0	0	0	0
Hippea	2	44	0	0	0	0	0	0	0	0	0	0	0	0	0	0	0	0	0	0
Hirschia	2	102	0	0	0	0	0	1	0	0	0	0	0	0	0	0	0	0	0	0
Histophilus	2	72	0	0	0	0	0	0	0	0	0	0	0	0	0	0	0	0	0	0
Hoeflea	1	16	0	0	0	0	0	0	0	0	0	0	0	0	0	0	0	0	0	0
Hydrogenivirga	1	2	0	0	0	0	0	0	0	0	0	0	0	0	0	0	0	0	0	0
Hydrogenobacter	2	55	0	0	0	0	0	0	0	0	0	0	0	0	0	0	0	0	0	0
Hydrogenobaculum	2	44	0	0	0	0	1	0	0	0	0	0	0	0	0	0	0	0	0	0
Hydrogenophaga	1	9	0	0	0	0	0	0	0	0	0	0	0	0	0	0	0	0	0	0
Hylemonella	1	4	0	0	0	0	0	0	0	0	0	0	0	0	0	0	0	0	0	0

Genus	Cluster Number	Protein Counts	K11947	K11948	K11949	K13421	K13953	K14519	K14520	K14579	K14582	K14583	K14584	K14746	K14747	K14748	K14751	K15054	K15058	K15060
**Hyperthermus**	1	6	0	0	0	0	0	0	0	0	0	0	0	0	0	0	0	0	0	0
**Hyphomicrobium**	2	93	0	0	0	0	1	0	0	0	0	0	0	0	0	0	0	0	0	0
**Hyphomonas**	3	105	0	0	0	0	0	0	0	0	0	0	0	0	0	0	0	0	0	0
**Ideonella**	1	18	0	0	0	0	0	0	0	0	0	0	0	0	0	0	0	0	0	0
**Idiomarina**	2	86	0	0	0	0	0	0	0	0	0	0	0	0	0	0	0	0	0	0
**Ignavibacterium**	1	14	0	0	0	0	0	0	0	0	0	0	0	0	0	0	0	0	0	0
**Ignicoccus**	1	5	0	0	0	0	0	0	0	0	0	0	0	0	0	0	0	0	0	0
**Ignisphaera**	1	8	0	0	0	0	1	0	0	0	0	0	0	0	0	0	0	0	0	0
**Ilyobacter**	2	85	0	0	0	0	0	0	0	0	0	0	0	0	0	0	0	0	0	0
**Intrasporangium**	2	114	0	0	0	0	1	1	0	0	0	0	0	0	0	0	0	0	0	0
**Isoptericola**	1	18	0	0	0	0	0	0	0	0	0	0	0	0	0	0	0	0	0	0
**Isosphaera**	2	75	0	0	0	0	1	0	0	0	0	0	0	0	0	0	0	0	0	0
**Jannaschia**	3	151	0	0	0	0	0	0	0	0	0	0	0	0	0	0	0	0	0	0
**Janthinobacterium**	2	99	0	0	0	0	0	0	0	0	0	0	0	0	0	0	0	0	0	0
**Jonesia**	2	56	0	0	0	0	0	0	0	0	0	0	0	0	0	0	0	0	0	0
**Joostella**	1	3	0	0	0	0	0	0	0	0	0	0	0	0	0	0	0	0	0	0
**Kaistia**	1	2	0	0	0	0	0	0	0	0	0	0	0	0	0	0	0	0	0	0
**Kangiella**	2	84	0	0	0	0	0	0	0	0	0	0	0	0	0	0	0	0	0	0
**Ketogulonicigenium**	2	102	0	0	0	0	0	1	0	0	0	0	0	0	0	0	0	0	0	0
**Kineococcus**	1	24	0	0	0	0	1	0	0	0	0	0	0	0	0	0	0	0	0	0
**Kinetoplastibacterium**	1	2	0	0	0	0	0	0	0	0	0	0	0	0	0	0	0	0	0	0
**Kingella**	1	4	0	0	0	0	0	0	0	0	0	0	0	0	0	0	0	0	0	0
**Kitasatospora**	1	36	0	0	0	0	1	0	0	0	0	0	0	0	0	0	0	0	0	0
**Klebsiella**	3	222	0	0	0	0	1	1	0	0	0	0	0	0	0	0	0	0	0	0
**Kluyvera**	1	1	0	0	0	0	0	0	0	0	0	0	0	0	0	0	0	0	0	0
**Kocuria**	2	87	0	0	0	0	1	0	0	0	0	0	0	0	0	0	0	0	0	0
**Kordia**	1	5	0	0	0	0	0	0	0	0	0	0	0	0	0	0	0	0	0	0
**Kosmotoga**	2	61	0	0	0	0	0	0	0	0	0	0	0	0	0	0	0	0	0	0
**Kribbella**	2	99	0	0	0	0	0	1	0	0	0	0	0	0	0	0	0	0	0	0
**Krokinobacter**	2	90	0	0	0	0	0	1	0	0	0	0	0	0	0	0	0	0	0	0
**Kyrpidia**	2	113	0	0	0	0	1	0	0	0	0	0	0	0	0	0	0	0	0	0
**Kytococcus**	2	70	0	0	0	0	0	0	0	0	0	0	0	0	0	0	0	0	0	0
**Labrenzia**	1	4	0	0	0	0	0	0	0	0	0	0	0	0	0	0	0	0	0	0
**Lachnoanaerobaculum**	1	1	0	0	0	0	0	0	0	0	0	0	0	0	0	0	0	0	0	0
**Lachnospiraceae**	1	51	0	0	0	0	0	0	0	0	0	0	0	0	0	0	0	0	0	0
**Lacinutrix**	1	20	0	0	0	0	0	0	0	0	0	0	0	0	0	0	0	0	0	0
**Lactobacillus**	2	119	0	0	0	0	1	0	0	0	0	0	0	0	0	0	0	0	0	0
**Lactococcus**	2	82	0	0	0	0	1	0	0	0	0	0	0	0	0	0	0	0	0	0
**Lamprocystis**	1	2	0	0	0	0	0	0	0	0	0	0	0	0	0	0	0	0	0	0
**Laribacter**	2	80	0	0	0	0	0	0	0	0	0	0	0	0	0	0	0	0	0	0
**Lawsonia**	2	33	0	0	0	0	0	0	0	0	0	0	0	0	0	0	0	0	0	0
**Leadbetterella**	2	79	0	0	0	0	0	1	0	0	0	0	0	0	0	0	0	0	0	0
**Leeuwenhoekiella**	1	2	0	0	0	0	0	0	0	0	0	0	0	0	0	0	0	0	0	0
**Legionella**	2	117	0	0	0	0	1	0	0	0	0	0	0	0	0	0	0	0	0	0
**Leifsonia**	2	48	0	0	0	0	1	0	0	0	0	0	0	0	0	0	0	0	0	0
**Leptolyngbya**	1	10	0	0	0	1	0	0	0	0	0	0	0	0	0	0	0	0	0	0
**Leptospira**	2	76	0	0	0	0	1	0	0	0	0	0	0	0	0	0	0	0	0	0
**Leptospirillum**	1	10	0	0	0	0	1	0	0	0	0	0	0	0	0	0	0	0	0	0
**Leptothrix**	3	186	0	0	0	0	0	1	0	0	0	0	0	0	0	0	0	0	0	0
**Leptotrichia**	2	43	0	0	0	0	1	0	0	0	0	0	0	0	0	0	0	0	0	0
**Leucobacter**	1	2	0	0	0	0	0	0	0	0	0	0	0	0	0	0	0	0	0	0
**Leuconostoc**	2	64	0	0	0	0	1	0	0	0	0	0	0	0	0	0	0	0	0	0

Genus	Cluster Number	Protein Counts	K11947	K11948	K11949	K13421	K13953	K14519	K14520	K14579	K14582	K14583	K14584	K14746	K14747	K14748	K14751	K15054	K15058	K15060
Liberibacter	1	4	0	0	0	0	0	0	0	0	0	0	0	0	0	0	0	0	0	0
Limnohabitans	1	11	0	0	0	0	0	0	0	0	0	0	0	0	0	0	0	0	0	0
Listeria	2	77	0	0	0	0	0	0	0	0	0	0	0	0	0	0	0	0	0	0
Listeriaceae	1	6	0	0	0	0	0	0	0	0	0	0	0	0	0	0	0	0	0	0
Listonella	1	38	0	0	0	0	0	0	0	0	0	0	0	0	0	0	0	0	0	0
Loktanella	1	3	0	0	0	0	0	0	0	0	0	0	0	0	0	0	0	0	0	0
Lysinibacillus	2	97	0	0	0	0	0	0	0	0	0	0	0	0	0	0	0	0	0	0
Macrococcus	2	60	0	0	0	0	0	0	0	0	0	0	0	0	0	0	0	0	0	0
Magnetococcus	2	64	0	0	0	0	0	0	0	0	0	0	0	0	0	0	0	0	0	0
Magnetospirillum	2	113	0	0	0	0	0	0	0	0	0	0	0	0	0	0	0	0	0	0
Mahella	2	58	0	0	0	0	0	0	0	0	0	0	0	0	0	0	0	0	0	0
Mannheimia	2	85	0	0	0	0	0	0	0	0	0	0	0	0	0	0	0	0	0	0
Maribacter	3	87	0	0	0	0	0	1	0	0	0	0	0	0	0	0	0	0	0	0
Maricaulis	3	87	0	0	0	0	0	0	0	0	0	0	0	0	0	0	0	0	0	0
Marichromatium	1	2	0	0	0	0	0	0	0	0	0	0	0	0	0	0	0	0	0	0
Marinithermus	2	77	0	0	0	0	0	0	0	0	0	0	0	0	0	0	0	0	0	0
Marinitoga	1	15	0	0	0	0	0	0	0	0	0	0	0	0	0	0	0	0	0	0
Marinobacter	2	163	0	0	0	0	0	0	0	0	0	0	0	0	0	0	0	1	0	0
Marinomonas	3	192	0	0	0	0	1	1	0	0	0	0	0	0	0	0	0	1	0	0
Maritimibacter	1	7	0	0	0	0	0	0	0	0	0	0	0	0	0	0	0	0	0	0
Marivirga	2	96	0	0	0	0	0	0	0	0	0	0	0	0	0	0	0	0	0	0
Martelella	1	3	0	0	0	0	0	0	0	0	0	0	0	0	0	0	0	0	0	0
Marvinbryantia	1	1	0	0	0	0	0	0	0	0	0	0	0	0	0	0	0	0	0	0
Massilia	1	4	0	0	0	0	0	0	0	0	0	0	0	0	0	0	0	0	0	0
Mastigocladopsis	1	16	0	0	0	0	0	0	0	0	0	0	0	0	0	0	0	0	0	0
Megamonas	1	47	0	0	0	0	0	0	0	0	0	0	0	0	0	0	0	0	0	0
Megasphaera	1	14	0	0	0	0	0	0	0	0	0	0	0	0	0	0	0	0	0	0
Meiothermus	2	110	0	0	0	0	1	0	0	0	0	0	0	0	0	0	0	0	0	0
Melissococcus	2	46	0	0	0	0	1	0	0	0	0	0	0	0	0	0	0	0	0	0
Mesoflavibacter	1	6	0	0	0	0	0	0	0	0	0	0	0	0	0	0	0	0	0	0
Mesoplasma	1	23	0	0	0	0	0	0	0	0	0	0	0	0	0	0	0	0	0	0
Mesorhizobium	2	201	0	0	0	0	0	0	0	0	0	0	0	0	0	0	0	1	0	0
Mesotoga	1	14	0	0	0	0	0	0	0	0	0	0	0	0	0	0	0	0	0	0
Metallosphaera	1	18	0	0	0	0	0	0	0	0	0	0	0	0	0	0	0	0	0	0
Methanobacterium	1	7	0	0	0	0	0	0	0	0	0	0	0	0	0	0	0	0	0	0
Methanobrevibacter	1	9	0	0	0	0	1	0	0	0	0	0	0	0	0	0	0	0	0	0
Methanocaldococcus	1	6	0	0	0	0	0	0	0	0	0	0	0	0	0	0	0	0	0	0
Methanocella	1	15	0	0	0	0	0	0	0	0	0	0	0	0	0	0	0	0	0	0
Methanococcaceae	1	1	0	0	0	0	0	0	0	0	0	0	0	0	0	0	0	0	0	0
Methanococcoides	1	5	0	0	0	0	0	0	0	0	0	0	0	0	0	0	0	0	0	0
Methanococcus	1	9	0	0	0	0	0	0	0	0	0	0	0	0	0	0	0	0	0	0
Methanocorpusculum	1	7	0	0	0	0	0	0	0	0	0	0	0	0	0	0	0	0	0	0
Methanoculleus	1	8	0	0	0	0	0	0	0	0	0	0	0	0	0	0	0	0	0	0
Methanohalobium	1	5	0	0	0	0	0	0	0	0	0	0	0	0	0	0	0	0	0	0
Methanohalophilus	1	5	0	0	0	0	0	0	0	0	0	0	0	0	0	0	0	0	0	0
Methanolobus	1	5	0	0	0	0	0	0	0	0	0	0	0	0	0	0	0	0	0	0
Methanomethylovorans	1	5	0	0	0	0	0	0	0	0	0	0	0	0	0	0	0	0	0	0
Methanoplanus	1	7	0	0	0	0	0	0	0	0	0	0	0	0	0	0	0	0	0	0
Methanopyrus	1	4	0	0	0	0	0	0	0	0	0	0	0	0	0	0	0	0	0	0
Methanoregula	1	8	0	0	0	0	0	0	0	0	0	0	0	0	0	0	0	0	0	0
Methanosaeta	1	9	0	0	0	0	1	0	0	0	0	0	0	0	0	0	0	0	0	0
Methanosalsum	1	7	0	0	0	0	0	0	0	0	0	0	0	0	0	0	0	0	0	0

Genus	Cluster Number	Protein Counts	K11947	K11948	K11949	K13421	K13953	K14519	K14520	K14579	K14582	K14583	K14584	K14746	K14747	K14748	K14751	K15054	K15058	K15060
Methanosarcina	1	18	0	0	0	0	0	0	0	0	0	0	0	0	0	0	0	0	0	0
Methanosphaera	1	7	0	0	0	0	0	0	0	0	0	0	0	0	0	0	0	0	0	0
Methanosphaerula	1	8	0	0	0	0	0	0	0	0	0	0	0	0	0	0	0	0	0	0
Methanospirillum	1	6	0	0	0	0	0	0	0	0	0	0	0	0	0	0	0	0	0	0
Methanothermobacter	1	6	0	0	0	0	0	0	0	0	0	0	0	0	0	0	0	0	0	0
Methanothermococcus	1	5	0	0	0	0	0	0	0	0	0	0	0	0	0	0	0	0	0	0
Methanothermus	1	5	0	0	0	0	0	0	0	0	0	0	0	0	0	0	0	0	0	0
Methanotorris	1	5	0	0	0	0	0	0	0	0	0	0	0	0	0	0	0	0	0	0
Methylacidiphilum	2	48	0	0	0	0	1	0	0	0	0	0	0	0	0	0	0	0	0	0
Methylarcula	1	2	0	0	0	0	0	0	0	0	0	0	0	0	0	0	0	0	0	0
Methylibium	1	38	0	0	0	0	1	0	0	0	0	0	0	0	0	0	0	0	0	0
Methylobacillus	2	61	0	0	0	0	0	0	0	0	0	0	0	0	0	0	0	0	0	0
Methylobacterium	3	236	0	0	0	0	1	1	0	0	0	0	0	0	0	0	0	0	0	0
Methylocella	3	117	0	0	0	0	1	0	0	0	0	0	0	0	0	0	0	0	0	0
Methylococcus	2	80	0	0	0	0	1	0	0	0	0	0	0	0	0	0	0	0	0	0
Methylocystis	1	21	0	0	0	0	1	0	0	0	0	0	0	0	0	0	0	0	0	0
Methyloferula	1	2	0	0	0	0	0	0	0	0	0	0	0	0	0	0	0	0	0	0
Methylomicrobium	1	12	0	0	0	0	0	0	0	0	0	0	0	0	0	0	0	0	0	0
Methylomonas	1	15	0	0	0	0	0	0	0	0	0	0	0	0	0	0	0	0	0	0
Methylophaga	1	7	0	0	0	0	0	0	0	0	0	0	0	0	0	0	0	0	0	0
Methylophilus	1	2	0	0	0	0	0	0	0	0	0	0	0	0	0	0	0	0	0	0
Methylosinus	1	2	0	0	0	0	0	0	0	0	0	0	0	0	0	0	0	0	0	0
Methylotenera	2	72	0	0	0	0	0	0	0	0	0	0	0	0	0	0	0	0	0	0
Methyloversatilis	1	5	0	0	0	0	0	0	0	0	0	0	0	0	0	0	0	0	0	0
Methylovorus	2	66	0	0	0	0	0	0	0	0	0	0	0	0	0	0	0	0	0	0
Micavibrio	1	9	0	0	0	0	0	0	0	0	0	0	0	0	0	0	0	0	0	0
Microbacterium	2	115	0	0	0	0	1	0	0	0	0	0	0	0	0	0	0	0	0	0
Microchaete	1	23	0	0	0	0	0	0	0	0	0	0	0	0	0	0	0	0	0	0
Micrococcus	2	72	0	0	0	0	1	0	0	0	0	0	0	0	0	0	0	0	0	0
Microcoleus	1	17	0	0	0	1	0	0	0	0	0	0	0	0	0	0	0	0	0	0
Microcystis	2	67	0	0	0	1	0	0	0	0	0	0	0	0	0	0	0	0	0	0
Microlunatus	1	25	0	0	0	0	1	1	0	0	0	0	0	0	0	0	0	0	0	0
Micromonospora	2	115	0	0	0	0	1	0	0	0	0	0	0	0	0	0	0	0	0	0
Microvirga	1	6	0	0	0	0	0	0	0	0	0	0	0	0	0	0	0	0	0	0
Mobiluncus	1	41	0	0	0	0	0	0	0	0	0	0	0	0	0	0	0	0	0	0
Modestobacter	1	43	0	0	0	0	1	0	0	0	0	0	0	0	0	0	0	0	0	0
Moorea	1	2	0	0	0	0	0	0	0	0	0	0	0	0	0	0	0	0	0	0
Moorella	2	69	0	0	0	0	0	0	0	0	0	0	0	0	0	0	0	0	0	0
Moraxella	2	66	0	0	0	0	0	0	0	0	0	0	0	0	0	0	0	0	0	0
Morganella	1	29	0	0	0	0	1	0	0	0	0	0	0	0	0	0	0	0	0	0
Moritella	1	2	0	0	0	0	0	0	0	0	0	0	0	0	0	0	0	0	0	0
Muricauda	1	23	0	0	0	0	0	1	0	0	0	0	0	0	0	0	0	0	0	0
Mycobacterium	3	306	1	1	1	0	1	1	1	0	0	0	0	0	0	0	0	0	0	0
Mycoplasma	1	53	0	0	0	0	1	0	0	0	0	0	0	0	0	0	0	0	0	0
Myxococcus	2	110	0	0	0	0	1	1	0	0	0	0	0	0	0	0	0	0	0	0
Nakamurella	2	115	0	0	0	0	1	0	0	0	0	0	0	0	0	0	0	0	0	0
napthalene	1	2	0	0	0	0	0	0	0	1	0	0	0	0	0	0	0	0	0	0
Natranaerobius	2	55	0	0	0	0	0	0	0	0	0	0	0	0	0	0	0	0	0	0
Natrialba	1	16	0	0	0	0	0	0	0	0	0	0	0	0	0	0	0	0	0	0
Natrinema	1	16	0	0	0	0	1	0	0	0	0	0	0	0	0	0	0	0	0	0
Natronobacterium	1	11	0	0	0	0	0	0	0	0	0	0	0	0	0	0	0	0	0	0
Natronococcus	1	21	0	0	0	0	1	0	0	0	0	0	0	0	0	0	0	0	0	0

Genus	Cluster Number	Protein Counts	K11947	K11948	K11949	K13421	K13953	K14519	K14520	K14579	K14582	K14583	K14584	K14746	K14747	K14748	K14751	K15054	K15058	K15060
**Natronomonas**	1	20	0	0	0	0	0	0	0	0	0	0	0	0	0	0	0	0	0	0
**Nautilia**	2	48	0	0	0	0	0	0	0	0	0	0	0	0	0	0	0	0	0	0
**Neisseria**	2	67	0	0	0	0	1	0	0	0	0	0	0	0	0	0	0	0	0	0
**Neisseriaceae**	1	1	0	0	0	0	0	0	0	0	0	0	0	0	0	0	0	0	0	0
**Neorickettsia**	1	31	0	0	0	0	0	0	0	0	0	0	0	0	0	0	0	0	0	0
**Neptuniibacter**	1	2	0	0	0	0	0	0	0	0	0	0	0	0	0	0	0	0	0	0
**Niastella**	1	21	0	0	0	0	1	1	0	0	0	0	0	0	0	0	0	0	0	0
**Nitratifractor**	2	54	0	0	0	0	0	0	0	0	0	0	0	0	0	0	0	0	0	0
**Nitratireductor**	1	19	0	0	0	0	0	0	0	0	0	0	0	0	0	0	0	0	0	0
**Nitratiruptor**	2	40	0	0	0	0	0	0	0	0	0	0	0	0	0	0	0	0	0	0
**Nitrobacter**	2	99	0	0	0	0	1	0	0	0	0	0	0	0	0	0	0	0	0	0
**Nitrococcus**	1	2	0	0	0	0	0	0	0	0	0	0	0	0	0	0	0	0	0	0
**Nitrosococcus**	2	94	0	0	0	0	0	0	0	0	0	0	0	0	0	0	0	0	0	0
**Nitrosomonas**	2	72	0	0	0	0	0	0	0	0	0	0	0	0	0	0	0	0	0	0
**Nitrosopumilaceae**	1	7	0	0	0	0	1	0	0	0	0	0	0	0	0	0	0	0	0	0
**Nitrosopumilus**	1	11	0	0	0	0	1	0	0	0	0	0	0	0	0	0	0	0	0	0
**Nitrosospira**	2	74	0	0	0	0	1	0	0	0	0	0	0	0	0	0	0	0	0	0
**Nocardia**	2	185	0	0	0	0	1	1	0	0	0	0	0	0	0	0	0	0	0	0
**Nocardioidaceae**	1	2	0	0	0	0	0	0	0	0	0	0	0	0	0	0	0	0	0	0
**Nocardioides**	2	157	0	0	0	0	1	1	0	0	0	0	0	0	0	0	0	0	0	0
**Nocardiopsis**	2	102	0	0	0	0	1	1	0	0	0	0	0	0	0	0	0	0	0	0
**Nodularia**	1	22	0	0	0	0	0	0	0	0	0	0	0	0	0	0	0	0	0	0
**Nonlabens**	1	19	0	0	0	0	1	0	0	0	0	0	0	0	0	0	0	0	0	0
**Nostoc**	2	111	0	0	0	1	1	0	0	0	0	0	0	0	0	0	0	0	0	0
**Nostocaceae**	1	2	0	0	0	0	0	0	0	0	0	0	0	0	0	0	0	0	0	0
**Novosphingobium**	2	169	0	0	0	0	1	1	1	1	0	0	0	0	0	1	1	0	0	0
**Oceanibaculum**	1	3	0	0	0	0	0	0	0	0	0	0	0	0	0	0	0	0	0	0
**Oceanibulbus**	1	12	0	0	0	0	0	0	0	0	0	0	0	0	0	0	0	0	0	0
**Oceanicola**	1	9	0	0	0	0	0	0	0	0	0	0	0	0	0	0	0	0	0	0
**Oceanimonas**	1	33	0	0	0	0	0	0	0	0	0	0	0	0	0	0	0	0	0	0
**Oceaniovalibus**	1	2	0	0	0	0	0	0	0	0	0	0	0	0	0	0	0	0	0	0
**Oceanithermus**	2	72	0	0	0	0	0	0	0	0	0	0	0	0	0	0	0	0	0	0
**Oceanobacillus**	2	91	0	0	0	0	0	0	0	0	0	0	0	0	0	0	0	0	0	0
**Oceanospirillum**	1	2	0	0	0	0	0	0	0	0	0	0	0	0	0	0	0	0	0	0
**Ochrobactrum**	2	133	0	0	0	0	1	0	0	0	0	0	0	0	0	0	0	0	0	0
**Octadecabacter**	1	29	0	0	0	0	0	1	0	0	0	0	0	0	0	0	0	0	0	0
**Oenococcus**	2	41	0	0	0	0	1	0	0	0	0	0	0	0	0	0	0	0	0	0
**Oleomonas**	1	2	0	0	0	0	0	0	0	0	0	0	0	0	0	0	0	0	0	0
**Oligotropha**	3	87	0	0	0	0	1	0	0	0	0	0	0	0	0	0	0	0	0	0
**Olsenella**	2	40	0	0	0	0	0	0	0	0	0	0	0	0	0	0	0	0	0	0
**Opitutus**	2	67	0	0	0	0	0	1	0	0	0	0	0	0	0	0	0	0	0	0
**Orientia**	1	21	0	0	0	0	0	0	0	0	0	0	0	0	0	0	0	0	0	0
**Ornithobacterium**	1	11	0	0	0	1	0	0	0	0	0	0	0	0	0	0	0	0	0	0
**Oscillatoria**	1	16	0	0	0	1	0	0	0	0	0	0	0	0	0	0	0	0	0	0
**Oscillatoriales**	1	2	0	0	0	0	0	0	0	0	0	0	0	0	0	0	0	0	0	0
**Oscillibacter**	1	20	0	0	0	0	0	0	0	0	0	0	0	0	0	0	0	0	0	0
**Owenweeksia**	1	15	0	0	0	0	0	0	0	0	0	0	0	0	0	0	0	0	0	0
**Oxalobacteraceae**	1	4	0	0	0	0	0	0	0	0	0	0	0	0	0	0	0	0	0	0
**Paenibacillus**	2	152	0	0	0	0	1	0	0	0	0	0	0	0	0	0	0	0	0	0
**Paludibacter**	2	67	0	0	0	0	0	0	0	0	0	0	0	0	0	0	0	0	0	0
**Pandoraea**	1	18	0	0	0	0	0	0	0	0	0	0	0	0	0	0	0	0	0	0
**Pannonibacter**	1	3	0	0	0	0	0	0	0	0	0	0	0	0	0	0	0	0	0	0

Genus	Cluster Number	Protein Counts	K11947	K11948	K11949	K13421	K13953	K14519	K14520	K14579	K14582	K14583	K14584	K14746	K14747	K14748	K14751	K15054	K15058	K15060
Pantholops	1	23	0	0	0	0	0	0	0	0	0	0	0	0	0	0	0	0	0	0
Pantoea	2	181	0	0	0	0	1	1	0	0	0	0	0	0	0	0	0	1	0	0
Parabacteroides	2	64	0	0	0	0	0	0	0	0	0	0	0	0	0	0	0	0	0	0
Parachlamydia	1	11	0	0	0	0	1	0	0	0	0	0	0	0	0	0	0	0	0	0
Paracoccus	2	169	0	0	0	0	1	1	0	0	0	0	0	0	0	0	0	1	0	0
Parvibaculum	3	117	0	0	0	0	1	0	0	0	0	1	0	0	0	0	0	0	0	0
Parvularcula	2	72	0	0	0	0	0	0	0	0	0	0	0	0	0	0	0	0	0	0
Pasteurella	2	85	0	0	0	0	0	0	0	0	0	0	0	0	0	0	0	0	0	0
Pasteurellaceae	1	2	0	0	0	0	0	0	0	0	0	0	0	0	0	0	0	0	0	0
Pectobacterium	1	44	0	0	0	0	1	0	0	0	0	0	0	0	0	0	0	0	0	0
Pediococcus	1	43	0	0	0	0	0	0	0	0	0	0	0	0	0	0	0	0	0	0
Pedobacter	2	88	0	0	0	0	0	0	0	0	0	0	0	0	0	0	0	0	0	0
Pelagibaca	1	10	0	0	0	0	0	0	0	0	0	0	0	0	0	0	0	0	0	0
Pelagibacterium	1	39	0	0	0	0	0	1	0	0	0	0	0	0	0	0	0	0	0	0
Pelobacter	2	85	0	0	0	0	0	0	0	0	0	0	0	0	0	0	0	0	0	0
Pelodictyon	2	62	0	0	0	0	0	0	0	0	0	0	0	0	0	0	0	0	0	0
Pelosinus	1	1	0	0	0	0	0	0	0	0	0	0	0	0	0	0	0	0	0	0
Pelotomaculum	2	60	0	0	0	0	0	0	0	0	0	0	0	0	0	0	0	0	0	0
Peptostreptococcus	1	1	0	0	0	0	0	0	0	0	0	0	0	0	0	0	0	0	0	0
Persephonella	2	55	0	0	0	0	0	0	0	0	0	0	0	0	0	0	0	0	0	0
Petrotoga	2	72	0	0	0	0	0	0	0	0	0	0	0	0	0	0	0	0	0	0
Phaeobacter	2	55	0	0	0	0	0	0	0	0	0	0	0	0	0	0	0	0	0	0
Phenylobacterium	3	103	0	0	0	0	1	0	1	0	0	0	0	0	0	0	0	0	0	0
Photobacterium	2	119	0	0	0	0	0	0	0	0	0	0	0	0	0	0	0	0	0	0
Photorhabdus	2	149	0	0	0	0	0	1	0	0	0	0	0	0	0	0	0	1	0	0
Phycisphaera	1	9	0	0	0	0	0	0	0	0	0	0	0	0	0	0	0	0	0	0
Phyllobacterium	1	6	0	0	0	0	0	0	0	0	0	0	0	0	0	0	0	0	0	0
Picrophilus	1	15	0	0	0	0	1	0	0	0	0	0	0	0	0	0	0	0	0	0
Pirellula	2	85	0	0	0	0	0	1	0	0	0	0	0	0	0	0	0	0	0	0
Planctomyces	2	93	0	0	0	0	0	1	0	0	0	0	0	0	0	0	0	0	0	0
Plautia	1	17	0	0	0	0	0	0	0	0	0	0	0	0	0	0	0	0	0	0
Plesiomonas	1	4	0	0	0	0	0	0	0	0	0	0	0	0	0	0	0	0	0	0
Pleurocapsa	1	23	0	0	0	1	1	0	0	0	0	0	0	0	0	0	0	0	0	0
Polaribacter	2	74	0	0	0	0	0	0	0	0	0	0	0	0	0	0	0	0	0	0
Polaromonas	3	238	0	0	0	0	1	1	0	1	1	1	1	0	0	0	0	1	0	0
Polymorphum	3	173	0	0	0	0	0	0	0	0	0	0	0	0	0	0	0	0	0	0
Polynucleobacter	1	19	0	0	0	0	0	0	0	0	0	0	0	0	0	0	0	0	0	0
Ponticaulis	1	1	0	0	0	0	0	0	0	0	0	0	0	0	0	0	0	0	0	0
Porphyromonas	2	63	0	0	0	0	0	0	0	0	0	0	0	0	0	0	0	0	0	0
Prevotella	1	65	0	0	0	0	0	0	0	0	0	0	0	0	0	0	0	0	0	0
Prochlorococcus	2	68	0	0	0	0	0	0	0	0	0	0	0	0	0	0	0	0	0	0
Propionibacterium	1	62	0	0	0	0	1	0	0	0	0	0	0	0	0	0	0	0	0	0
Prosthecochloris	1	16	0	0	0	0	0	0	0	0	0	0	0	0	0	0	0	0	0	0
Proteobacteria	1	19	0	0	0	0	0	0	0	0	0	0	0	0	0	0	0	0	0	0
Proteus	2	113	0	0	0	0	0	0	0	0	0	0	0	0	0	0	0	0	0	0
Providencia	1	38	0	0	0	0	0	0	0	0	0	0	0	0	0	0	0	0	0	0
Pseudaminobacter	1	10	0	0	0	0	0	0	0	0	0	0	0	0	0	0	0	0	0	0
Pseudanabaena	1	11	0	0	0	1	0	0	0	0	0	0	0	0	0	0	0	0	0	0
Pseudoalteromonas	2	188	0	0	0	0	1	0	0	0	0	0	0	0	0	0	0	0	0	0
Pseudochrobactrum	1	7	0	0	0	0	0	0	0	0	0	0	0	0	0	0	0	0	0	0
Pseudogulbenkiania	1	40	0	0	0	0	0	1	0	0	0	0	0	0	0	0	0	0	0	0
pseudomallei	3	131	0	0	0	0	1	0	0	0	0	0	0	0	0	0	0	0	0	0

Genus	Cluster Number	Protein Counts	K11947	K11948	K11949	K13421	K13953	K14519	K14520	K14579	K14582	K14583	K14584	K14746	K14747	K14748	K14751	K15054	K15058	K15060
**Pseudomonas**	3	381	0	0	0	0	1	1	0	1	1	1	1	0	0	0	0	1	1	0
**Pseudonocardia**	3	180	0	0	0	0	1	1	1	0	0	0	0	0	0	0	0	0	0	1
**Pseudorhodobacter**	1	4	0	0	0	0	0	0	0	0	0	0	0	0	0	0	0	0	0	0
**Pseudovibrio**	1	33	0	0	0	0	1	1	0	0	0	0	0	0	0	0	0	0	0	0
**Pseudoxanthomonas**	2	124	0	0	0	0	1	1	0	0	0	0	0	0	0	0	0	0	0	0
**Psychrobacter**	2	124	0	0	0	0	0	0	0	0	0	0	0	0	0	0	0	0	0	0
**Psychroflexus**	1	22	0	0	0	0	0	1	0	0	0	0	0	0	0	0	0	0	0	0
**Psychromonas**	2	110	0	0	0	0	0	0	0	0	0	0	0	0	0	0	0	0	0	0
**Pusillimonas**	3	111	0	0	0	0	0	0	0	0	0	0	0	0	0	0	0	0	0	0
**Pyrobaculum**	1	15	0	0	0	0	1	0	0	0	0	0	0	0	0	0	0	0	0	0
**Pyrococcus**	1	10	0	0	0	0	0	0	0	0	0	0	0	0	0	0	0	0	0	0
**Pyrolobus**	1	4	0	0	0	0	0	0	0	0	0	0	0	0	0	0	0	0	0	0
**Rahnella**	2	143	0	0	0	0	1	0	0	1	0	0	0	0	0	0	0	1	0	0
**Ralstonia**	3	332	0	0	0	0	1	1	0	1	1	1	1	0	0	0	0	1	0	0
**Ramlibacter**	1	35	0	0	0	0	0	0	0	0	0	0	0	0	0	0	0	0	0	0
**Raoultella**	2	139	0	0	0	0	1	1	0	0	0	0	0	0	0	0	0	0	0	0
**Raphidiopsis**	1	5	0	0	0	0	0	0	0	0	0	0	0	0	0	0	0	0	0	0
**Reinekea**	1	2	0	0	0	0	0	0	0	0	0	0	0	0	0	0	0	0	0	0
**Renibacterium**	2	67	0	0	0	0	0	1	0	0	0	0	0	0	0	0	0	0	0	0
**Rhizobiaceae**	1	3	0	0	0	0	0	0	0	0	0	0	0	0	0	0	0	0	0	0
**Rhizobiales**	1	6	0	0	0	0	0	0	0	0	0	0	0	0	0	0	0	0	0	0
**Rhizobium**	3	257	0	0	0	0	1	1	1	0	0	0	0	0	0	0	0	0	0	0
**Rhizobium/Agrobacterium**	2	87	0	0	0	0	0	0	0	0	0	0	0	0	0	0	0	0	0	0
**Rhodanobacter**	1	21	0	0	0	0	1	0	0	0	0	0	0	0	0	0	0	0	0	0
**Rhodobacter**	3	175	0	0	0	0	1	1	0	0	0	0	0	0	0	0	0	0	0	0
**Rhodobacteraceae**	1	21	0	0	0	0	0	0	0	0	0	0	0	0	0	0	0	0	0	0
**Rhodobacterales**	1	34	0	0	0	0	0	0	0	0	0	0	0	0	0	0	0	0	0	0
**Rhodococcus**	2	292	0	0	1	0	1	1	1	0	0	0	0	0	0	1	1	0	0	0
**Rhodocyclaceae**	1	7	0	0	0	0	0	0	0	0	0	0	0	0	0	0	0	0	0	0
**Rhodoferax**	3	144	0	0	0	0	0	0	0	0	0	0	0	0	0	0	0	0	0	0
**Rhodomicrobium**	2	103	0	0	0	0	0	0	0	0	0	0	0	0	0	0	0	0	0	0
**Rhodopirellula**	2	87	0	0	0	0	0	1	0	0	0	0	0	0	0	0	0	0	0	0
**Rhodopseudomonas**	3	246	0	0	0	0	1	0	0	0	0	0	0	0	0	0	0	0	0	0
**Rhodospirillum**	2	121	0	0	0	0	0	0	0	0	0	0	0	0	0	0	0	0	0	0
**Rhodothermus**	2	76	0	0	0	0	1	0	0	0	0	0	0	0	0	0	0	0	0	0
**Rhodovulum**	1	4	0	0	0	0	0	0	0	0	0	0	0	0	0	0	0	0	0	0
**Richelia**	1	2	0	0	0	0	0	0	0	0	0	0	0	0	0	0	0	0	0	0
**Rickettsia**	2	43	0	0	0	0	0	0	0	0	0	0	0	0	0	0	0	0	0	0
**Rickettsiaceae**	1	5	0	0	0	0	0	0	0	0	0	0	0	0	0	0	0	0	0	0
**Riemerella**	2	61	0	0	0	1	0	0	0	0	0	0	0	0	0	0	0	0	0	0
**Rivularia**	1	18	0	0	0	0	0	0	0	0	0	0	0	0	0	0	0	0	0	0
**Robiginitalea**	2	83	0	0	0	0	0	1	0	0	0	0	0	0	0	0	0	0	0	0
**Roseburia**	2	58	0	0	0	0	0	0	0	0	0	0	0	0	0	0	0	0	0	0
**Roseibium**	1	3	0	0	0	0	0	0	0	0	0	0	0	0	0	0	0	0	0	0
**Roseiflexus**	3	100	0	0	0	0	1	0	0	0	0	0	0	0	0	0	0	0	0	0
**Roseobacter**	2	149	0	0	0	0	0	0	0	0	0	0	0	0	0	0	0	0	0	0
**Roseomonas**	1	2	0	0	0	0	0	0	0	0	0	0	0	0	0	0	0	0	0	0
**Roseovarius**	1	15	0	0	0	0	0	0	0	0	0	0	0	0	0	0	0	0	0	0
**Rothia**	1	44	0	0	0	0	1	0	0	0	0	0	0	0	0	0	0	0	0	0
**Rubrivivax**	1	24	0	0	0	0	0	0	0	0	0	0	0	0	0	0	0	0	0	0
**Rubrobacter**	3	117	0	0	0	0	1	0	0	0	0	0	0	0	0	0	0	0	0	0
**Ruegeria**	2	189	0	0	0	0	0	1	0	0	0	0	0	0	0	0	0	0	0	0

Genus	Cluster Number	Protein Counts	K11947	K11948	K11949	K13421	K13953	K14519	K14520	K14579	K14582	K14583	K14584	K14746	K14747	K14748	K14751	K15054	K15058	K15060
**Ruminococcaceae**	1	2	0	0	0	0	0	0	0	0	0	0	0	0	0	0	0	0	0	0
**Ruminococcus**	2	101	0	0	0	0	0	0	0	0	0	0	0	0	0	0	0	0	0	0
**Runella**	1	26	0	0	0	0	0	1	0	0	0	0	0	0	0	0	0	0	0	0
**Saccharomonospora**	2	132	0	0	0	0	1	0	0	0	0	0	0	0	0	0	0	0	0	0
**Saccharophagus**	2	81	0	0	0	0	0	0	0	0	0	0	0	0	0	0	0	0	0	0
**Saccharopolyspora**	3	194	0	0	0	0	1	1	0	0	0	0	0	0	0	0	0	0	0	0
**Saccharothrix**	1	32	0	0	0	0	0	0	0	0	0	0	0	0	0	0	0	0	0	0
**Sagittula**	1	10	0	0	0	0	0	0	0	0	0	0	0	0	0	0	0	0	0	0
**Salinibacter**	2	77	0	0	0	0	1	0	0	0	0	0	0	0	0	0	0	0	0	0
**Salinimonas**	1	1	0	0	0	0	0	0	0	0	0	0	0	0	0	0	0	0	0	0
**Salinispora**	3	127	0	0	0	0	1	0	0	0	0	0	0	0	0	0	0	0	0	0
**Salinivibrio**	1	2	0	0	0	0	0	0	0	0	0	0	0	0	0	0	0	0	0	0
**Salmonella**	2	151	0	0	0	0	1	0	0	0	0	0	0	0	0	0	0	0	0	0
**Sanguibacter**	2	73	0	0	0	0	1	0	0	0	0	0	0	0	0	0	0	0	0	0
**Saprospira**	1	13	0	0	0	1	0	0	0	0	0	0	0	0	0	0	0	0	0	0
**Scardovia**	1	1	0	0	0	0	0	0	0	0	0	0	0	0	0	0	0	0	0	0
**Scytonema**	1	10	0	0	0	0	0	0	0	0	0	0	0	0	0	0	0	0	0	0
**Sebaldella**	2	69	0	0	0	0	0	0	0	0	0	0	0	0	0	0	0	0	0	0
**Segniliparus**	2	85	0	0	0	0	0	0	0	0	0	0	0	0	0	0	0	0	0	0
**Selenomonas**	2	57	0	0	0	0	0	0	0	0	0	0	0	0	0	0	0	0	0	0
**Serinicoccus**	1	1	0	0	0	0	0	0	0	0	0	0	0	0	0	0	0	0	0	0
**Serratia**	2	186	0	0	0	0	1	1	0	0	0	0	0	0	0	0	0	1	0	0
**Shewanella**	3	214	0	0	0	0	0	1	0	0	0	0	0	0	0	0	0	0	0	0
**Shigella**	2	172	0	0	0	0	1	0	0	0	0	0	0	0	0	0	0	0	0	0
**Shinella**	1	2	0	0	0	0	0	0	0	0	0	0	0	0	0	0	0	0	0	0
**Sideroxydans**	2	81	0	0	0	0	0	0	0	0	0	0	0	0	0	0	0	0	0	0
**Silicibacter**	2	105	0	0	0	0	0	1	0	0	0	0	0	0	0	0	0	0	0	0
**Simiduia**	1	18	0	0	0	0	0	1	0	0	0	0	0	0	0	0	0	0	0	0
**Simkania**	1	6	0	0	0	0	0	0	0	0	0	0	0	0	0	0	0	0	0	0
**Simonsiella**	1	2	0	0	0	0	0	0	0	0	0	0	0	0	0	0	0	0	0	0
**Singulisphaera**	1	18	0	0	0	0	1	0	0	0	0	0	0	0	0	0	0	0	0	0
**Sinorhizobium**	3	207	0	0	0	0	1	1	1	0	0	0	0	0	0	0	0	0	0	0
**Slackia**	2	46	0	0	0	0	0	0	0	0	0	0	0	0	0	0	0	0	0	0
**Smaragdicoccus**	1	3	0	0	0	0	0	0	0	0	0	0	0	0	0	0	0	0	0	0
**Sodalis**	2	71	0	0	0	0	0	0	0	0	0	0	0	0	0	0	0	0	0	0
**Solibacillus**	1	23	0	0	0	0	1	0	0	0	0	0	0	0	0	0	0	0	0	0
**Solitalea**	1	13	0	0	0	0	0	0	0	0	0	0	0	0	0	0	0	0	0	0
**Sorangium**	3	115	0	0	0	0	1	1	0	0	0	0	0	0	0	0	0	0	0	0
**Sphaerobacter**	2	100	0	0	0	0	0	0	0	0	0	0	0	0	0	0	0	0	0	0
**Sphaerochaeta**	1	42	0	0	0	0	0	0	0	0	0	0	0	0	0	0	0	0	0	0
**Sphingobacterium**	3	85	0	0	0	0	0	1	0	0	0	0	0	0	0	0	0	0	0	0
**Sphingobium**	1	179	0	0	0	0	1	1	1	0	0	0	0	0	0	0	0	0	0	1
**Sphingomonadaceae**	1	15	0	0	0	0	1	0	1	0	0	0	0	0	0	0	0	0	0	0
**Sphingomonas**	2	220	0	0	0	0	1	1	1	0	0	0	0	0	0	0	0	1	0	1
**Sphingopyxis**	3	99	0	0	0	0	1	0	0	0	0	0	0	0	0	0	0	0	0	0
**Spirochaeta**	2	103	0	0	1	1	0	0	0	0	0	0	0	0	0	0	0	0	0	0
**Spiroplasma**	1	6	0	0	0	0	0	0	0	0	0	0	0	0	0	0	0	0	0	0
**Spirosoma**	2	107	0	0	0	0	1	1	0	0	0	0	0	0	0	0	0	0	0	0
**Stackebrandtia**	2	110	0	0	0	0	0	1	0	0	0	0	0	0	0	0	0	0	0	0
**Stanieria**	1	17	0	0	0	1	1	0	0	0	0	0	0	0	0	0	0	0	0	0
**Staphylococcus**	2	108	0	0	0	0	1	0	0	0	0	0	0	0	0	0	0	0	0	0
**Staphylothermus**	1	12	0	0	0	0	1	0	0	0	0	0	0	0	0	0	0	0	0	0

Genus	Cluster Number	Protein Counts	K11947	K11948	K11949	K13421	K13953	K14519	K14520	K14579	K14582	K14583	K14584	K14746	K14747	K14748	K14751	K15054	K15058	K15060
Stappia	1	2	0	0	0	0	0	0	0	0	0	0	0	0	0	0	0	0	0	0
Starkeya	3	134	0	0	0	0	1	0	0	0	0	0	0	0	0	0	0	1	0	0
Stenotrophomonas	3	104	0	0	0	0	1	0	0	0	0	0	0	0	0	0	0	0	0	0
Stigmatella	3	129	0	0	0	0	1	0	0	0	0	0	0	0	0	0	0	0	0	0
Streptobacillus	1	38	0	0	0	0	1	0	0	0	0	0	0	0	0	0	0	0	0	0
Streptococcus	2	121	0	0	0	0	1	0	0	0	0	0	0	0	0	0	0	0	0	0
Streptomyces	2	253	0	0	0	0	1	1	0	0	0	0	0	0	0	0	0	0	0	0
Streptosporangium	3	148	0	0	0	0	1	1	0	0	0	0	0	0	0	0	0	0	0	0
Strigomonas	1	2	0	0	0	0	0	0	0	0	0	0	0	0	0	0	0	0	0	0
Sulfitobacter	1	10	0	0	0	0	0	0	0	0	0	0	0	0	0	0	0	0	0	0
Sulfobacillus	1	30	0	0	0	0	1	0	0	0	0	0	0	0	0	0	0	0	0	0
Sulfolobus	1	23	0	0	0	0	1	0	0	0	0	0	0	0	0	0	0	0	0	0
Sulfuricurvum	2	67	0	0	0	0	0	0	0	0	0	0	0	0	0	0	0	0	0	0
Sulfurihydrogenibium	2	55	0	0	0	0	0	0	0	0	0	0	0	0	0	0	0	0	0	0
Sulfurimonas	2	60	0	0	0	0	0	0	0	0	0	0	0	0	0	0	0	0	0	0
Sulfurospirillum	1	48	0	0	0	0	0	0	0	0	0	0	0	0	0	0	0	0	0	0
Sulfurovum	2	48	0	0	0	0	0	0	0	0	0	0	0	0	0	0	0	0	0	0
Symbiobacterium	2	68	0	0	0	0	0	0	0	0	0	0	0	0	0	0	0	0	0	0
Synechococcus	1	34	0	0	0	1	0	0	0	0	0	0	0	0	0	0	0	0	0	0
Synechocystis	1	40	0	0	0	0	0	0	0	0	0	0	0	0	0	0	0	0	0	0
Synergistetes	1	27	0	0	0	0	0	0	0	0	0	0	0	0	0	0	0	0	0	0
Syntrophobacter	2	101	0	0	0	0	1	0	0	0	0	0	0	0	0	0	0	0	0	0
Syntrophobotulus	2	71	0	0	0	0	0	0	0	0	0	0	0	0	0	0	0	0	0	0
Syntrophomonas	2	58	0	0	0	0	0	0	0	0	0	0	0	0	0	0	0	0	0	0
Syntrophothermus	2	60	0	0	0	0	0	0	0	0	0	0	0	0	0	0	0	0	0	0
Syntrophus	2	66	0	0	0	0	1	0	0	0	0	0	0	0	0	0	0	0	0	0
Tannerella	1	8	0	0	0	0	0	0	0	0	0	0	0	0	0	0	0	0	0	0
Taylorella	1	43	0	0	0	0	0	0	0	0	0	0	0	0	0	0	0	0	0	0
Tepidanaerobacter	2	55	0	0	0	0	0	0	0	0	0	0	0	0	0	0	0	0	0	0
Teredinibacter	2	109	0	0	0	0	0	1	0	0	0	0	0	0	0	0	0	0	0	0
Terrabacter	1	1	0	0	0	0	0	0	0	0	0	0	0	0	0	0	0	0	0	0
Terriglobus	2	83	0	0	0	0	1	1	0	0	0	0	0	0	0	0	0	0	0	0
Tetragenococcus	1	12	0	0	0	0	0	0	0	0	0	0	0	0	0	0	0	0	0	0
Thalassiobium	1	11	0	0	0	0	0	0	0	0	0	0	0	0	0	0	0	0	0	0
Thalassobacter	1	9	0	0	0	0	0	0	0	0	0	0	0	0	0	0	0	0	0	0
Thalassolituus	1	12	0	0	0	0	0	0	0	0	0	0	0	0	0	0	0	0	0	0
Thalassospira	1	2	0	0	0	0	0	0	0	0	0	0	0	0	0	0	0	0	0	0
Thauera	1	111	0	0	0	0	1	0	0	0	0	0	0	0	0	0	0	1	0	0
Thermacetogenium	1	7	0	0	0	0	0	0	0	0	0	0	0	0	0	0	0	0	0	0
Thermaerobacter	2	79	0	0	0	0	1	0	0	0	0	0	0	0	0	0	0	0	0	0
Thermanaerovibrio	2	58	0	0	0	0	0	0	0	0	0	0	0	0	0	0	0	0	0	0
Thermincola	2	62	0	0	0	0	0	0	0	0	0	0	0	0	0	0	0	0	0	0
Thermoanaerobacter	2	76	0	0	0	0	0	0	0	0	0	0	0	0	0	0	0	0	0	0
Thermoanaerobacterium	2	64	0	0	0	0	0	0	0	0	0	0	0	0	0	0	0	0	0	0
Thermobacillus	1	11	0	0	0	0	0	0	0	0	0	0	0	0	0	0	0	0	0	0
Thermobaculum	2	62	0	0	0	0	1	0	0	0	0	0	0	0	0	0	0	0	0	0
Thermobifida	2	72	0	0	0	0	1	1	0	0	0	0	0	0	0	0	0	0	0	0
Thermobispora	2	109	0	0	0	0	1	1	0	0	0	0	0	0	0	0	0	0	0	0
Thermococcus	1	13	0	0	0	0	0	0	0	0	0	0	0	0	0	0	0	0	0	0
Thermocrinis	2	60	0	0	0	0	0	0	0	0	0	0	0	0	0	0	0	0	0	0
Thermodesulfatator	1	10	0	0	0	0	0	0	0	0	0	0	0	0	0	0	0	0	0	0
Thermodesulfobacterium	1	9	0	0	0	0	1	0	0	0	0	0	0	0	0	0	0	0	0	0

Genus	Cluster Number	Protein Counts	K11947	K11948	K11949	K13421	K13953	K14519	K14520	K14579	K14582	K14583	K14584	K14746	K14747	K14748	K14751	K15054	K15058	K15060
Thermodesulfobium	1	13	0	0	0	0	1	0	0	0	0	0	0	0	0	0	0	0	0	0
Thermodesulfovibrio	2	60	0	0	0	0	1	0	0	0	0	0	0	0	0	0	0	0	0	0
Thermofilum	1	9	0	0	0	0	0	0	0	0	0	0	0	0	0	0	0	0	0	0
Thermogladius	1	7	0	0	0	0	0	0	0	0	0	0	0	0	0	0	0	0	0	0
Thermomicrobium	2	92	0	0	0	0	1	0	0	0	0	0	0	0	0	0	0	0	0	0
Thermomonospora	2	125	0	0	0	0	1	0	0	0	0	0	0	0	0	0	0	0	0	0
Thermoplasma	1	11	0	0	0	0	0	0	0	0	0	0	0	0	0	0	0	0	0	0
Thermoplasmatales	1	6	0	0	0	0	0	0	0	0	0	0	0	0	0	0	0	0	0	0
Thermoproteus	1	12	0	0	0	0	1	0	0	0	0	0	0	0	0	0	0	0	0	0
Thermosediminibacter	2	64	0	0	0	0	0	0	0	0	0	0	0	0	0	0	0	0	0	0
Thermosipho	2	70	0	0	0	0	0	0	0	0	0	0	0	0	0	0	0	0	0	0
Thermosphaera	1	10	0	0	0	0	1	0	0	0	0	0	0	0	0	0	0	0	0	0
Thermosynechococcus	1	6	0	0	0	0	0	0	0	0	0	0	0	0	0	0	0	0	0	0
Thermotoga	2	80	0	0	0	0	0	0	0	0	0	0	0	0	0	0	0	0	0	0
Thermovibrio	2	58	0	0	0	0	0	0	0	0	0	0	0	0	0	0	0	0	0	0
Thermovirga	1	9	0	0	0	0	0	0	0	0	0	0	0	0	0	0	0	0	0	0
Thermus	2	107	0	0	0	0	1	0	0	0	0	0	0	0	0	0	0	0	0	0
Thioalkalimicrobium	1	4	0	0	0	0	0	0	0	0	0	0	0	0	0	0	0	0	0	0
Thioalkalivibrio	2	91	0	0	0	0	0	0	0	0	0	0	0	0	0	0	0	0	0	0
Thiobaca	1	2	0	0	0	0	0	0	0	0	0	0	0	0	0	0	0	0	0	0
Thiobacillus	2	67	0	0	0	0	0	0	0	0	0	0	0	0	0	0	0	0	0	0
Thiocapsa	1	2	0	0	0	0	0	0	0	0	0	0	0	0	0	0	0	0	0	0
Thiocystis	1	11	0	0	0	0	0	0	0	0	0	0	0	0	0	0	0	0	0	0
Thioflavicoccus	1	13	0	0	0	0	1	0	0	0	0	0	0	0	0	0	0	0	0	0
Thiomicrospira	2	50	0	0	0	0	0	0	0	0	0	0	0	0	0	0	0	0	0	0
Thiomonas	2	85	0	0	0	0	0	0	0	0	0	0	0	0	0	0	0	0	0	0
Thiorhodococcus	1	2	0	0	0	0	0	0	0	0	0	0	0	0	0	0	0	0	0	0
Thiothrix	1	2	0	0	0	0	0	0	0	0	0	0	0	0	0	0	0	0	0	0
Tistrella	1	42	0	0	0	0	1	1	1	0	0	0	0	0	0	0	0	0	0	0
Tolumonas	2	100	0	0	0	0	0	0	0	0	0	0	0	0	0	0	0	0	0	0
Transposon	1	2	0	0	0	0	0	0	0	0	0	0	0	0	0	0	0	0	0	0
Treponema	1	63	0	0	0	0	0	0	0	0	0	0	0	0	0	0	0	0	0	0
Trichodesmium	2	71	0	0	0	1	0	0	0	0	0	0	0	0	0	0	0	0	0	0
Trichormus	1	12	0	0	0	0	0	0	0	0	0	0	0	0	0	0	0	0	0	0
Tropheryma	2	22	0	0	0	0	0	0	0	0	0	0	0	0	0	0	0	0	0	0
Truepera	2	104	0	0	0	0	1	0	0	0	0	0	0	0	0	0	0	0	0	0
Tsukamurella	2	123	0	0	0	0	0	0	0	0	0	0	0	0	0	0	0	0	0	0
Turneriella	1	16	0	0	0	0	0	0	0	0	0	0	0	0	0	0	0	0	0	0
Uliginosibacterium	1	2	0	0	0	0	0	0	0	0	0	0	0	0	0	0	0	0	0	0
Ureaplasma	1	24	0	0	0	0	0	0	0	0	0	0	0	0	0	0	0	0	0	0
Ureibacillus	1	2	0	0	0	0	0	0	0	0	0	0	0	0	0	0	0	0	0	0
Variovorax	3	187	0	0	0	0	0	0	0	0	0	0	0	0	0	0	0	1	0	1
Veillonella	1	54	0	0	0	0	0	0	0	0	0	0	0	0	0	0	0	0	0	0
Verminephrobacter	3	188	0	0	0	0	1	0	0	0	0	0	0	0	0	0	0	0	0	0
Verrucosispora	3	126	0	0	0	0	1	0	0	0	0	0	0	0	0	0	0	0	1	0
Vibrio	2	195	0	0	0	0	0	1	0	0	0	0	0	0	0	0	0	0	0	0
Vibrionales	1	46	0	0	0	0	0	0	0	0	0	0	0	0	0	0	0	0	0	0
Vitreoscilla	1	1	0	0	0	0	0	0	0	0	0	0	0	0	0	0	0	0	0	0
Vulcanisaeta	1	14	0	0	0	0	1	0	0	0	0	0	0	0	0	0	0	0	0	0
Waddlia	2	68	0	0	0	1	0	0	0	0	0	0	0	0	0	0	0	0	0	0
Wautersia	1	2	0	0	0	0	0	0	0	0	0	0	0	0	0	0	0	0	0	0
Weeksella	2	65	0	0	0	1	0	0	0	0	0	0	0	0	0	0	0	0	0	0

Genus	Cluster Number	Protein Counts	K11947	K11948	K11949	K13421	K13953	K14519	K14520	K14579	K14582	K14583	K14584	K14746	K14747	K14748	K14751	K15054	K15058	K15060
Weissella	1	14	0	0	0	0	1	0	0	0	0	0	0	0	0	0	0	0	0	0
Wenxinia	1	2	0	0	0	0	0	0	0	0	0	0	0	0	0	0	0	0	0	0
Wigglesworthia	1	29	0	0	0	0	0	0	0	0	0	0	0	0	0	0	0	0	0	0
Wolbachia	1	32	0	0	0	0	0	0	0	0	0	0	0	0	0	0	0	0	0	0
Wolinella	2	51	0	0	0	0	0	0	0	0	0	0	0	0	0	0	0	0	0	0
Xanthobacter	3	176	0	0	0	0	1	1	0	0	0	0	0	0	0	0	0	0	0	0
Xanthobacteraceae	1	4	0	0	0	0	0	0	0	0	0	0	0	0	0	0	0	0	0	0
Xanthomonadaceae	1	39	0	0	0	0	0	0	0	0	0	0	0	0	0	0	0	0	0	0
Xanthomonas	2	162	0	0	0	0	1	1	0	0	0	0	0	0	0	0	0	0	0	0
Xenorhabdus	2	127	0	0	0	0	0	0	0	0	0	0	0	0	0	0	0	0	0	0
Xylanimonas	2	66	0	0	0	0	0	0	0	0	0	0	0	0	0	0	0	0	0	0
Xylella	2	52	0	0	0	0	0	0	0	0	0	0	0	0	0	0	0	0	0	0
Yersinia	2	145	0	0	0	0	0	0	0	0	0	0	0	0	0	0	0	0	0	0
Yokenella	2	61	0	0	0	0	0	0	0	0	0	0	0	0	0	0	0	0	0	0
Zobellia	1	28	0	0	0	0	0	1	0	0	0	0	0	0	0	0	0	0	0	0
Zunongwangia	2	98	0	0	0	0	1	1	0	0	0	0	0	0	0	0	0	0	0	0
Zymomonas	2	67	0	0	0	0	1	0	0	0	0	0	0	0	0	0	0	0	0	0
Zymophilus	1	2	0	0	0	0	0	0	0	0	0	0	0	0	0	0	0	0	0	0

Genus	Cluster Number	Protein Counts	K15061	K15062	K15063	K15064	K15065	K15066	K15236	K15237	K15238	K15242	K15247	K15253	K15749	K15751	K15754	K15756	K15757	K15981
Acaricomes	1	2	0	0	0	0	0	0	0	0	0	0	0	0	0	0	0	0	0	0
Acaryochloris	2	88	0	0	0	0	0	0	0	0	0	0	0	0	0	0	0	0	0	0
Acetivibrio	1	2	0	0	0	0	0	0	0	0	0	0	0	0	0	0	0	0	0	0
Acetobacter	2	85	0	0	0	0	0	0	0	0	0	0	0	0	0	0	0	0	0	0
Acetobacteraceae	1	2	0	0	0	0	0	0	0	0	0	0	0	0	0	0	0	0	0	0
Acetobacterium	1	8	0	0	0	0	0	0	0	0	0	0	0	0	0	0	0	0	0	0
Acetohalobium	2	79	0	0	0	0	0	0	0	0	0	0	0	0	0	0	0	0	0	0
Acholeplasma	2	38	0	0	0	0	0	0	0	0	0	0	0	0	0	0	0	0	0	0
Achromobacter	2	192	0	0	0	0	0	0	0	0	0	0	0	1	0	0	0	0	0	0
Acidaminococcus	1	65	0	0	0	0	0	0	0	0	0	0	0	0	0	0	0	0	0	0
Acidianus	1	9	0	0	0	0	0	0	0	0	0	0	0	0	0	0	0	0	0	0
Acidilobus	1	8	0	0	0	0	0	0	0	0	0	0	0	0	0	0	0	0	0	0
Acidimicrobium	2	58	0	0	0	0	0	0	0	0	0	0	0	0	0	0	0	0	0	0
Acidiphilium	3	144	0	0	0	0	0	0	0	0	0	0	0	0	0	0	0	0	0	0
Acidithiobacillus	2	70	0	0	0	0	0	0	0	0	0	0	0	0	0	0	0	0	0	0
Acidobacterium	2	91	0	0	0	0	0	0	0	0	0	0	0	0	0	0	0	0	0	0
Acidocella	1	2	0	0	0	0	0	0	0	0	0	0	0	0	0	0	0	0	0	0
Acidothermus	2	68	0	0	0	0	0	0	0	0	0	0	0	0	0	0	0	0	0	0
Acidovorax	3	217	0	0	0	0	0	0	0	0	0	0	0	0	0	0	0	0	0	0
Aciduliprofundum	1	11	0	0	0	0	0	0	0	0	0	0	0	0	0	0	0	0	0	0
Acinetobacter	3	188	0	0	0	0	0	0	0	0	0	0	0	0	0	0	0	0	0	0
Actinobacillus	2	112	0	0	0	0	0	0	0	0	0	0	0	0	0	0	0	0	0	0
Actinomadura	1	1	0	0	0	0	0	0	0	0	0	0	0	0	0	0	0	0	0	0
Actinoplanes	1	52	0	0	0	0	0	0	0	0	0	0	0	0	0	0	0	0	0	1
Actinopolyspora	1	2	0	0	0	0	0	0	0	0	0	0	0	0	0	0	0	0	0	0
Actinosynnema	2	122	0	0	0	0	0	0	0	0	0	0	0	0	0	0	0	0	0	0
Advenella	1	30	0	0	0	0	0	0	0	0	0	0	0	0	0	0	0	0	0	0
Aequorivita	1	12	0	0	0	0	0	0	0	0	0	0	0	0	0	0	0	0	0	0
Aerococcus	2	57	0	0	0	0	0	0	0	0	0	0	0	0	0	0	0	0	0	0
Aeromonas	2	127	0	0	0	0	0	0	0	0	0	0	0	0	0	0	0	0	0	0
Aeropyrum	1	13	0	0	0	0	0	0	0	0	0	0	0	0	0	0	0	0	0	0
Afipia	1	54	0	0	0	0	0	0	0	0	0	0	0	0	0	0	0	0	0	0
Aggregatibacter	1	81	0	0	0	0	0	0	0	0	0	0	0	0	0	0	0	0	0	0
Agrobacterium	2	242	0	0	0	0	0	1	0	0	0	0	0	0	0	0	0	0	0	0
Ahrensia	1	2	0	0	0	0	0	0	0	0	0	0	0	0	0	0	0	0	0	0
Akkermansia	2	47	0	0	0	0	0	0	0	0	0	0	0	0	0	0	0	0	0	0
Albidiferax	3	144	0	0	0	0	0	0	0	0	0	0	0	0	0	0	0	0	0	0
Alcaligenes	1	21	0	0	0	0	0	0	0	0	0	0	0	0	0	0	0	0	0	0
Alcanivorax	2	146	0	0	0	0	0	0	0	0	0	0	0	1	0	0	0	0	0	0
Alicycliphilus	3	152	0	0	0	0	0	0	0	0	0	0	0	0	0	0	0	0	0	0
Alicyclobacillus	2	91	0	0	0	0	0	0	0	0	0	0	0	0	0	0	0	0	0	0
Aliivibrio	2	118	0	0	0	0	0	0	0	0	0	0	0	0	0	0	0	0	0	0
Alishewanella	1	1	0	0	0	0	0	0	0	0	0	0	0	0	0	0	0	0	0	0
Alistipes	1	52	0	0	0	0	0	0	0	0	0	0	0	0	0	0	0	0	0	0
Alkalilimnicola	2	97	0	0	0	0	0	0	0	0	0	0	0	0	0	0	0	0	0	0
Alkaliphilus	2	62	0	0	0	0	0	0	0	0	0	0	0	0	0	0	0	0	0	0
Allochromatium	2	84	0	0	0	0	0	0	0	0	0	0	0	0	0	0	0	0	0	0
Alphaproteobacteria	1	3	0	0	0	0	0	0	0	0	0	0	0	0	0	0	0	0	0	0
Alteromonadales	1	12	0	0	0	0	0	0	0	0	0	0	0	0	0	0	0	0	0	0
Alteromonas	1	37	0	0	0	0	0	0	0	0	0	0	0	0	0	0	0	0	0	0
Aminobacter	1	4	0	0	0	0	0	0	0	0	0	0	0	0	0	0	0	0	0	0
Aminobacterium	2	59	0	0	0	0	0	0	0	0	0	0	0	0	0	0	0	0	0	0

Genus	Cluster Number	Protein Counts	K15061	K15062	K15063	K15064	K15065	K15066	K15236	K15237	K15238	K15242	K15247	K15253	K15749	K15751	K15754	K15756	K15757	K15981
**Ammonifex**	2	50	0	0	0	0	0	0	0	0	0	0	0	0	0	0	0	0	0	0
**Amphibacillus**	1	13	0	0	0	0	0	0	0	0	0	0	0	0	0	0	0	0	0	0
**Amphritea**	1	2	0	0	0	0	0	0	0	0	0	0	0	0	0	0	0	0	0	0
**Amycolatopsis**	2	201	0	0	0	0	0	0	0	0	0	0	0	0	0	0	0	0	0	1
**Amycolicicoccus**	1	43	0	0	0	0	0	0	0	0	0	0	0	0	0	0	0	0	0	1
**Anabaena**	2	92	0	0	0	0	0	0	0	0	0	0	0	0	0	0	0	0	0	0
**Anaerobaculum**	1	11	0	0	0	0	0	0	0	0	0	0	0	0	0	0	0	0	0	0
**Anaerococcus**	2	46	0	0	0	0	0	0	0	0	0	0	0	0	0	0	0	0	0	0
**Anaerolinea**	2	66	0	0	0	0	0	0	0	0	0	0	0	0	0	0	0	0	0	0
**Anaeromyxobacter**	3	117	0	0	0	0	0	0	0	0	0	0	0	0	0	0	0	0	0	0
**Anaerostipes**	1	44	0	0	0	0	0	0	0	0	0	0	0	0	0	0	0	0	0	0
**Anaerotruncus**	1	2	0	0	0	0	0	0	0	0	0	0	0	0	0	0	0	0	0	0
**Anaplasma**	1	35	0	0	0	0	0	0	0	0	0	0	0	0	0	0	0	0	0	0
**Ancylobacter**	1	3	0	0	0	0	0	0	0	0	0	0	0	0	0	0	0	0	0	0
**Aneurinibacillus**	1	1	0	0	0	0	0	0	0	0	0	0	0	0	0	0	0	0	0	0
**Angomonas**	1	1	0	0	0	0	0	0	0	0	0	0	0	0	0	0	0	0	0	0
**Anoxybacillus**	2	72	0	0	0	0	0	0	0	0	0	0	0	0	0	0	0	0	0	0
**Aquifex**	2	45	0	0	0	0	0	0	0	0	0	0	0	0	0	0	0	0	0	0
**Aquimarina**	1	2	0	0	0	0	0	0	0	0	0	0	0	0	0	0	0	0	0	0
**Arcanobacterium**	2	45	0	0	0	0	0	0	0	0	0	0	0	0	0	0	0	0	0	0
**Archaeoglobus**	1	14	0	0	0	0	0	0	0	0	0	0	0	0	0	0	0	0	0	0
**Arcobacter**	2	101	0	0	0	0	0	0	0	0	0	0	0	0	0	0	0	0	0	0
**Aromatoleum**	3	133	0	0	0	0	0	0	0	0	0	0	0	0	0	0	0	0	0	0
**Arsenophonus**	1	4	0	0	0	0	0	0	0	0	0	0	0	0	0	0	0	0	0	0
**Arthrobacter**	2	206	0	0	0	0	0	0	0	0	0	0	0	0	0	0	0	0	0	0
**Arthrospira**	2	67	0	0	0	0	0	0	0	0	0	0	0	0	0	0	0	0	0	0
**Aster**	1	10	0	0	0	0	0	0	0	0	0	0	0	0	0	0	0	0	0	0
**Asticcacaulis**	2	103	0	0	0	0	0	0	0	0	0	0	0	0	0	0	0	0	0	0
**Atopobium**	1	28	0	0	0	0	0	0	0	0	0	0	0	0	0	0	0	0	0	0
**Aurantimonas**	1	2	0	0	0	0	0	0	0	0	0	0	0	0	0	0	0	0	0	0
**Avibacterium**	1	17	0	0	0	0	0	0	0	0	0	0	0	0	0	0	0	0	0	0
**Azoarcus**	3	172	0	0	0	0	0	0	0	0	0	0	0	0	0	0	0	0	0	0
**Azorhizobium**	3	149	0	0	0	0	0	0	0	0	0	0	0	0	0	0	0	0	0	0
**Azorhizophilus**	1	4	0	0	0	0	0	0	0	0	0	0	0	0	0	0	0	0	0	0
**Azospira**	1	19	0	0	0	0	0	0	0	0	0	0	0	0	0	0	0	0	0	0
**Azospirillum**	2	198	0	0	0	0	0	0	0	0	0	0	0	0	0	0	0	0	0	0
**Azotobacter**	3	183	0	0	0	0	0	0	0	0	0	0	0	0	0	0	0	0	0	0
**Bacillales**	1	9	0	0	0	0	0	0	0	0	0	0	0	0	0	0	0	0	0	0
**Bacilli**	1	3	0	0	0	0	0	0	0	0	0	0	0	0	0	0	0	0	0	0
**Bacillus**	2	237	0	0	0	0	0	0	0	0	0	0	0	0	0	0	0	0	0	0
**Bacteria**	2	86	0	0	0	0	0	0	0	0	0	0	0	0	0	0	0	0	0	0
**Bacteriovorax**	2	98	0	0	0	0	0	0	0	0	0	0	0	0	0	0	0	0	0	0
**Bacteroidales**	2	59	0	0	0	0	0	0	0	0	0	0	0	0	0	0	0	0	0	0
**Bacteroides**	2	96	0	0	0	0	0	0	0	0	0	0	0	0	0	0	0	0	0	0
**Bacteroidetes**	1	2	0	0	0	0	0	0	0	0	0	0	0	0	0	0	0	0	0	0
**Bartonella**	2	50	0	0	0	0	0	0	0	0	0	0	0	0	0	0	0	0	0	0
**Baumannia**	2	24	0	0	0	0	0	0	0	0	0	0	0	0	0	0	0	0	0	0
**Bdellovibrio**	2	87	0	0	0	0	0	0	0	0	0	0	0	0	0	0	0	0	0	0
**Beijerinckia**	3	123	0	0	0	0	0	0	0	0	0	0	0	0	0	0	0	0	0	0
**Belliella**	1	14	0	0	0	0	0	0	0	0	0	0	0	0	0	0	0	0	0	0
**Beutenbergia**	2	82	0	0	0	0	0	0	0	0	0	0	0	0	0	0	0	0	0	0
**Bifidobacterium**	2	61	0	0	0	0	0	0	0	0	0	0	0	0	0	0	0	0	0	0

Genus	Cluster Number	Protein Counts	K15061	K15062	K15063	K15064	K15065	K15066	K15236	K15237	K15238	K15242	K15247	K15253	K15749	K15751	K15754	K15756	K15757	K15981
Bizionia	1	1	0	0	0	0	0	0	0	0	0	0	0	0	0	0	0	0	0	0
Blastococcus	1	39	0	0	0	0	0	1	0	0	0	0	0	0	0	0	0	0	0	0
Blattabacterium	1	23	0	0	0	0	0	0	0	0	0	0	0	0	0	0	0	0	0	0
Blautia	1	22	0	0	0	0	0	0	0	0	0	0	0	0	0	0	0	0	0	0
Bordetella	3	227	0	0	0	0	0	0	0	0	0	0	0	1	0	0	0	0	0	0
Borrelia	1	32	0	0	0	0	0	0	0	0	0	0	0	0	0	0	0	0	0	0
Brachybacterium	1	18	0	0	0	0	0	0	0	0	0	0	0	0	0	0	0	0	0	0
Brachymonas	1	4	0	0	0	0	0	0	0	0	0	0	0	0	0	0	0	0	0	0
Brachyspira	2	79	0	0	0	0	0	0	0	0	0	0	0	0	0	0	0	0	0	0
Bradyrhizobiaceae	1	20	0	0	0	0	0	0	0	0	0	0	0	0	0	0	0	0	0	0
Bradyrhizobium	3	254	0	0	0	0	0	0	0	0	0	0	0	0	0	0	0	0	0	0
Brenneria	1	20	0	0	0	0	0	0	0	0	0	0	0	0	0	0	0	0	0	0
Brevibacillus	2	122	0	0	0	0	0	0	0	0	0	0	0	0	0	0	0	0	0	0
Brevibacterium	1	4	0	0	0	0	0	0	0	0	0	0	0	0	0	0	0	0	0	0
Brevundimonas	2	105	0	0	0	1	0	0	0	0	0	0	0	0	0	0	0	0	0	0
Brucella	3	138	0	0	0	0	0	0	0	0	0	0	0	0	0	0	0	0	0	0
Buchnera	1	26	0	0	0	0	0	0	0	0	0	0	0	0	0	0	0	0	0	0
Burkholderia	3	424	0	0	0	0	1	0	0	0	0	0	1	1	0	0	0	0	0	0
Burkholderiaceae	3	143	0	0	0	0	0	0	0	0	0	0	0	0	0	0	0	0	0	0
Burkholderiales	1	24	0	0	0	0	0	0	0	0	0	0	0	1	0	0	0	0	0	0
Buttiauxella	1	2	0	0	0	0	0	0	0	0	0	0	0	0	0	0	0	0	0	0
Butyrivibrio	2	67	0	0	0	0	0	0	0	0	0	0	0	0	0	0	0	0	0	0
Caldanaerobacter	2	63	0	0	0	0	0	0	0	0	0	0	0	0	0	0	0	0	0	0
Caldicellulosiruptor	2	75	0	0	0	0	0	0	0	0	0	0	0	0	0	0	0	0	0	0
Caldilinea	1	23	0	0	0	0	0	0	0	0	0	0	0	0	0	0	0	0	0	0
Caldimonas	1	6	0	0	0	0	0	0	0	0	0	0	0	0	0	0	0	0	0	0
Caldisericum	1	10	0	0	0	0	0	0	0	0	0	0	0	0	0	0	0	0	0	0
Caldisphaera	1	7	0	0	0	0	0	0	0	0	0	0	0	0	0	0	0	0	0	0
Calditerrivibrio	2	74	0	0	0	0	0	0	0	0	0	0	0	0	0	0	0	0	0	0
Caldivirga	1	13	0	0	0	0	0	0	0	0	0	0	0	0	0	0	0	0	0	0
Calothrix	1	43	0	0	0	0	0	0	0	0	0	0	0	0	0	0	0	0	0	0
Calyptogena	2	34	0	0	0	0	0	0	0	0	0	0	0	0	0	0	0	0	0	0
Campylobacter	2	80	0	0	0	0	0	0	0	0	0	0	0	0	0	0	0	0	0	0
Candidatus	2	290	0	0	0	0	0	0	0	0	0	0	0	0	0	0	0	0	0	0
Capnocytophaga	1	50	0	0	0	0	0	0	0	0	0	0	0	0	0	0	0	0	0	0
Carbophilus	1	2	0	0	0	0	0	0	0	0	0	0	0	0	0	0	0	0	0	0
Carboxydibrachium	1	14	0	0	0	0	0	0	0	0	0	0	0	0	0	0	0	0	0	0
Carboxydothermus	2	68	0	0	0	0	0	0	0	0	0	0	0	0	0	0	0	0	0	0
Carica	1	2	0	0	0	0	0	0	0	0	0	0	0	0	0	0	0	0	0	0
Carnobacterium	2	71	0	0	0	0	0	0	0	0	0	0	0	0	0	0	0	0	0	0
Catenibacterium	1	2	0	0	0	0	0	0	0	0	0	0	0	0	0	0	0	0	0	0
Catenovulum	1	2	0	0	0	0	0	0	0	0	0	0	0	0	0	0	0	0	0	0
Catenulispora	3	159	0	0	0	0	0	0	0	0	0	0	0	0	0	0	0	0	0	1
Caulobacter	3	168	0	0	0	0	0	0	0	0	0	0	0	0	0	0	0	0	0	0
Cedecea	1	45	0	0	0	0	0	0	0	0	0	0	0	0	0	0	0	0	0	0
Cellulomonas	2	96	0	0	0	0	0	0	0	0	0	0	0	0	0	0	0	0	0	0
Cellulophaga	2	105	0	0	0	0	0	0	0	0	0	0	0	0	0	0	0	0	0	0
Cellulosilyticum	1	14	0	0	0	0	0	0	0	0	0	0	0	0	0	0	0	0	0	0
Cellvibrio	2	85	0	0	0	0	0	0	0	0	0	0	0	0	0	0	0	0	0	0
Cenarchaeum	1	7	0	0	0	0	0	0	0	0	0	0	0	0	0	0	0	0	0	0
Chamaesiphon	1	13	0	0	0	0	0	0	0	0	0	0	0	0	0	0	0	0	0	0
Chelativorans	3	136	0	0	0	0	0	0	0	0	0	0	0	0	0	0	0	0	0	0

Genus	Cluster Number	Protein Counts	K15061	K15062	K15063	K15064	K15065	K15066	K15236	K15237	K15238	K15242	K15247	K15253	K15749	K15751	K15754	K15756	K15757	K15981
Chelatococcus	1	2	0	0	0	0	0	0	0	0	0	0	0	0	0	0	0	0	0	0
Chitinophaga	2	98	0	0	0	0	0	0	0	0	0	0	0	0	0	0	0	0	0	0
Chlamydia	1	36	0	0	0	0	0	0	0	0	0	0	0	0	0	0	0	0	0	0
Chlamydomonas	1	3	0	0	0	0	0	0	0	0	0	0	0	0	0	0	0	0	0	0
Chlamydophila	1	31	0	0	0	0	0	0	0	0	0	0	0	0	0	0	0	0	0	0
Chlorobaculum	2	61	0	0	0	0	0	0	0	0	0	0	0	0	0	0	0	0	0	0
Chlorobium	2	89	0	0	0	0	0	0	0	0	0	0	0	0	0	0	0	0	0	0
Chlorobium/Pelodictyon	1	2	0	0	0	0	0	0	0	0	0	0	0	0	0	0	0	0	0	0
Chloroflexus	2	105	0	0	0	0	0	0	0	0	0	0	0	0	0	0	0	0	0	0
Chlorogloeopsis	1	9	0	0	0	0	0	0	0	0	0	0	0	0	0	0	0	0	0	0
Chloroherpeton	2	59	0	0	0	0	0	0	0	0	0	0	0	0	0	0	0	0	0	0
Chromobacterium	2	117	0	0	0	0	0	0	0	0	0	0	0	0	0	0	0	0	0	0
Chromohalobacter	2	145	0	0	0	0	0	0	0	0	0	0	0	0	0	0	0	0	0	0
Chroococcidiopsis	1	24	0	0	0	0	0	0	0	0	0	0	0	0	0	0	0	0	0	0
Chryseobacterium	2	104	0	0	0	0	0	0	0	0	0	0	0	0	0	0	0	0	0	0
Citreicella	1	13	0	0	0	0	0	0	0	0	0	0	0	0	0	0	0	0	0	0
Citrobacter	2	190	0	0	0	0	0	0	0	0	0	0	0	0	0	0	0	0	0	0
Clavibacter	2	83	0	0	0	0	0	0	0	0	0	0	0	0	0	0	0	0	0	0
Clonorchis	1	1	0	0	0	0	0	0	0	0	0	0	0	0	0	0	0	0	0	0
Clostridiales	2	77	0	0	0	0	0	0	0	0	0	0	0	0	0	0	0	0	0	0
Clostridium	2	191	0	0	0	0	0	0	0	0	0	0	0	0	0	0	0	0	0	0
Cohnella	1	2	0	0	0	0	0	0	0	0	0	0	0	0	0	0	0	0	0	0
Coleofasciculus	1	4	0	0	0	0	0	0	0	0	0	0	0	0	0	0	0	0	0	0
Collimonas	1	35	0	0	0	0	0	0	0	0	0	0	0	0	0	0	0	0	0	0
Colwellia	2	130	0	0	0	0	0	0	0	0	0	0	0	0	0	0	0	0	0	0
Comamonas	2	196	0	0	0	0	0	0	0	0	0	0	0	0	0	0	0	0	0	0
Conexibacter	3	117	0	0	0	0	0	0	0	0	0	0	0	0	0	0	0	0	0	0
Coprobacillus	1	2	0	0	0	0	0	0	0	0	0	0	0	0	0	0	0	0	0	0
Coprococcus	2	76	0	0	0	0	0	0	0	0	0	0	0	0	0	0	0	0	0	0
Coprothermobacter	2	45	0	0	0	0	0	0	0	0	0	0	0	0	0	0	0	0	0	0
Coraliomargarita	2	68	0	0	0	0	0	0	0	0	0	0	0	0	0	0	0	0	0	0
Corallococcus	1	29	0	0	0	0	0	0	0	0	0	0	0	0	0	0	0	0	0	0
Coriobacterium	2	36	0	0	0	0	0	0	0	0	0	0	0	0	0	0	0	0	0	0
Corynebacterineae	1	5	0	0	0	0	0	0	0	0	0	0	0	0	0	0	0	0	0	0
Corynebacterium	2	175	0	0	0	0	0	0	0	0	0	0	0	0	0	0	0	0	0	0
Coxiella	2	54	0	0	0	0	0	0	0	0	0	0	0	0	0	0	0	0	0	0
Crinalium	1	21	0	0	0	0	0	0	0	0	0	0	0	0	0	0	0	0	0	0
Croceibacter	2	80	0	0	0	0	0	0	0	0	0	0	0	0	0	0	0	0	0	0
Crocosphaera	1	6	0	0	0	0	0	0	0	0	0	0	0	0	0	0	0	0	0	0
Cronobacter	2	120	0	0	0	0	0	0	0	0	0	0	0	0	0	0	0	0	0	0
Cryptobacterium	2	39	0	0	0	0	0	0	0	0	0	0	0	0	0	0	0	0	0	0
Cucumis	1	9	0	0	0	0	0	0	0	0	0	0	0	0	0	0	0	0	0	0
Cupriavidus	3	317	0	0	0	0	1	0	0	0	0	0	1	1	0	0	0	0	0	0
Curtobacterium	1	5	0	0	0	0	0	0	0	0	0	0	0	0	0	0	0	0	0	0
Curvibacter	1	27	0	0	0	0	0	0	0	0	0	0	0	0	0	0	0	0	0	0
Cyanobacterium	1	9	0	0	0	0	0	0	0	0	0	0	0	0	0	0	0	0	0	0
Cyanobium	1	10	0	0	0	0	0	0	0	0	0	0	0	0	0	0	0	0	0	0
Cyanothece	1	34	0	0	0	0	0	0	0	0	0	0	0	0	0	0	0	0	0	0
Cyclobacterium	1	19	0	0	0	0	0	0	0	0	0	0	0	0	0	0	0	0	0	0
Cycloclasticus	1	21	0	0	0	0	0	0	0	0	0	0	0	0	0	0	0	0	0	0
Cylindrospermopsis	1	4	0	0	0	0	0	0	0	0	0	0	0	0	0	0	0	0	0	0
Cylindrospermum	1	30	0	0	0	0	0	0	0	0	0	0	0	0	0	0	0	0	0	0

Genus	Cluster Number	Protein Counts	K15061	K15062	K15063	K15064	K15065	K15066	K15236	K15237	K15238	K15242	K15247	K15253	K15749	K15751	K15754	K15756	K15757	K15981
**Cytophaga**	2	69	0	0	0	0	0	0	0	0	0	0	0	0	0	0	0	0	0	0
**Dactylococcopsis**	1	8	0	0	0	0	0	0	0	0	0	0	0	0	0	0	0	0	0	0
**Dechloromonas**	1	40	0	0	0	0	0	0	0	0	0	0	0	0	0	0	0	0	0	0
**Dechlorosoma**	1	19	0	0	0	0	0	0	0	0	0	0	0	0	0	0	0	0	0	0
**Deferribacter**	2	73	0	0	0	0	0	0	0	0	0	0	0	0	0	0	0	0	0	0
**Dehalobacter**	1	9	0	0	0	0	0	0	0	0	0	0	0	0	0	0	0	0	0	0
**Dehalococcoides**	2	41	0	0	0	0	0	0	0	0	0	0	0	0	0	0	0	0	0	0
**Dehalogenimonas**	1	37	0	0	0	0	0	0	0	0	0	0	0	0	0	0	0	0	0	0
**Deinococcus**	2	140	0	0	0	0	0	0	0	0	0	0	0	0	0	0	0	0	0	0
**Delftia**	3	177	0	0	0	0	0	0	0	0	0	0	0	1	0	0	0	0	0	0
**Denitrovibrio**	2	67	0	0	0	0	0	0	0	0	0	0	0	0	0	0	0	0	0	0
**Desulfarculus**	2	92	0	0	0	0	0	0	0	0	0	0	0	0	0	0	0	0	0	0
**Desulfatibacillum**	3	84	0	0	0	0	0	0	0	0	0	0	0	0	0	0	0	0	0	0
**Desulfitobacterium**	2	102	0	0	0	0	0	0	0	0	0	0	0	0	0	0	0	0	0	0
**Desulfobacca**	2	65	0	0	0	0	0	0	0	0	0	0	0	0	0	0	0	0	0	0
**Desulfobacterium**	2	113	0	0	0	0	0	0	0	0	0	0	0	0	0	0	0	0	0	0
**Desulfobacula**	1	21	0	0	0	0	0	0	0	0	0	0	0	0	0	0	0	0	0	0
**Desulfobulbus**	2	88	0	0	0	0	0	0	0	0	0	0	0	0	0	0	0	0	0	0
**Desulfocapsa**	1	9	0	0	0	0	0	0	0	0	0	0	0	0	0	0	0	0	0	0
**Desulfococcus**	2	80	0	0	0	0	0	0	0	0	0	0	0	0	0	0	0	0	0	0
**Desulfohalobium**	2	52	0	0	0	0	0	0	0	0	0	0	0	0	0	0	0	0	0	0
**Desulfomicrobium**	2	65	0	0	0	0	0	0	0	0	0	0	0	0	0	0	0	0	0	0
**Desulfomonile**	1	25	0	0	0	0	0	0	0	0	0	0	0	0	0	0	0	0	0	0
**Desulforhabdus**	1	2	0	0	0	0	0	0	0	0	0	0	0	0	0	0	0	0	0	0
**Desulfosporosinus**	1	30	0	0	0	0	0	0	0	0	0	0	0	0	0	0	0	0	0	0
**Desulfotalea**	2	66	0	0	0	0	0	0	0	0	0	0	0	0	0	0	0	0	0	0
**Desulfotomaculum**	2	98	0	0	0	0	0	0	0	0	0	0	0	0	0	0	0	0	0	0
**Desulfovibrio**	2	144	0	0	0	0	0	0	0	0	0	0	0	0	0	0	0	0	0	0
**Desulfurispirillum**	2	78	0	0	0	0	0	0	0	0	0	0	0	0	0	0	0	0	0	0
**Desulfurivibrio**	2	71	0	0	0	0	0	0	0	0	0	0	0	0	0	0	0	0	0	0
**Desulfurobacterium**	2	53	0	0	0	0	0	0	0	0	0	0	0	0	0	0	0	0	0	0
**Desulfurococcus**	1	11	0	0	0	0	0	0	0	0	0	0	0	0	0	0	0	0	0	0
**Diaphorobacter**	1	16	0	0	0	0	0	0	0	0	0	0	0	0	0	0	0	0	0	0
**Dichelobacter**	2	45	0	0	0	0	0	0	0	0	0	0	0	0	0	0	0	0	0	0
**Dickeya**	2	155	0	0	0	0	0	0	0	0	0	0	0	0	0	0	0	0	0	0
**Dictyoglomus**	2	62	0	0	0	0	0	0	0	0	0	0	0	0	0	0	0	0	0	0
**Dinoroseobacter**	3	148	0	0	0	0	0	0	0	0	0	0	0	0	0	0	0	0	0	0
**Dokdonia**	1	3	0	0	0	0	0	0	0	0	0	0	0	0	0	0	0	0	0	0
**Dorea**	1	7	0	0	0	0	0	0	0	0	0	0	0	0	0	0	0	0	0	0
**Dyadobacter**	2	83	0	0	0	0	0	0	0	0	0	0	0	0	0	0	0	0	0	0
**Echinicola**	1	20	0	0	0	0	0	0	0	0	0	0	0	0	0	0	0	0	0	0
**Edwardsiella**	2	126	0	0	0	0	0	0	0	0	0	0	0	0	0	0	0	0	0	0
**Eggerthella**	1	51	0	0	0	0	0	0	0	0	0	0	0	0	0	0	0	0	0	0
**Ehrlichia**	2	37	0	0	0	0	0	0	0	0	0	0	0	0	0	0	0	0	0	0
**Eikenella**	1	4	0	0	0	0	0	0	0	0	0	0	0	0	0	0	0	0	0	0
**Elusimicrobium**	2	44	0	0	0	0	0	0	0	0	0	0	0	0	0	0	0	0	0	0
**Emticicia**	1	21	0	0	0	0	0	0	0	0	0	0	0	0	0	0	0	0	0	0
**Enhydrobacter**	1	1	0	0	0	0	0	0	0	0	0	0	0	0	0	0	0	0	0	0
**Ensifer**	1	2	0	0	0	0	0	0	0	0	0	0	0	0	0	0	0	0	0	0
**Enterobacter**	2	202	0	0	0	0	0	0	0	0	0	0	0	0	0	0	0	0	0	0
**Enterobacteriaceae**	2	211	0	0	0	0	0	0	0	0	0	0	0	0	0	0	0	0	0	0
**Enterococcus**	1	187	0	0	0	0	0	0	0	0	0	0	0	0	0	0	0	0	0	0

Genus	Cluster Number	Protein Counts	K15061	K15062	K15063	K15064	K15065	K15066	K15236	K15237	K15238	K15242	K15247	K15253	K15749	K15751	K15754	K15756	K15757	K15981
Enterovibrio	1	15	0	0	0	0	0	0	0	0	0	0	0	0	0	0	0	0	0	0
Erwinia	2	151	0	0	0	0	0	0	0	0	0	0	0	0	0	0	0	0	0	0
Erysipelothrix	1	9	0	0	0	0	0	0	0	0	0	0	0	0	0	0	0	0	0	0
Erysipelotrichaceae	1	4	0	0	0	0	0	0	0	0	0	0	0	0	0	0	0	0	0	0
Erythrobacter	2	80	0	0	0	0	0	0	0	0	0	0	0	0	0	0	0	0	0	0
Escherichia	2	242	0	0	0	0	0	0	0	0	0	0	0	0	0	0	0	0	0	0
Ethanoligenens	2	69	0	0	0	0	0	0	0	0	0	0	0	0	0	0	0	0	0	0
Eubacteriaceae	1	2	0	0	0	0	0	0	0	0	0	0	0	0	0	0	0	0	0	0
Eubacterium	2	103	0	0	0	0	0	0	0	0	0	0	0	0	0	0	0	0	0	0
Eudoraea	1	4	0	0	0	0	0	0	0	0	0	0	0	0	0	0	0	0	0	0
Euryarchaeota	1	1	0	0	0	0	0	0	0	0	0	0	0	0	0	0	0	0	0	0
Exiguobacterium	2	93	0	0	0	0	0	0	0	0	0	0	0	0	0	0	0	0	0	0
Faecalibacterium	1	12	0	0	0	0	0	0	0	0	0	0	0	0	0	0	0	0	0	0
Ferrimonas	2	115	0	0	0	0	0	0	0	0	0	0	0	0	0	0	0	0	0	0
Ferroglobus	1	14	0	0	0	0	0	0	0	0	0	0	0	0	0	0	0	0	0	0
Ferroplasma	1	11	0	0	0	0	0	0	0	0	0	0	0	0	0	0	0	0	0	0
Fervidicoccus	1	7	0	0	0	0	0	0	0	0	0	0	0	0	0	0	0	0	0	0
Fervidobacterium	2	54	0	0	0	0	0	0	0	0	0	0	0	0	0	0	0	0	0	0
Fibrella	1	17	0	0	0	0	0	0	0	0	0	0	0	0	0	0	0	0	0	0
Fibrobacter	2	52	0	0	0	0	0	0	0	0	0	0	0	0	0	0	0	0	0	0
Filifactor	1	9	0	0	0	0	0	0	0	0	0	0	0	0	0	0	0	0	0	0
Finegoldia	2	55	0	0	0	0	0	0	0	0	0	0	0	0	0	0	0	0	0	0
Firmicutes	1	15	0	0	0	0	0	0	0	0	0	0	0	0	0	0	0	0	0	0
Fischerella	1	15	0	0	0	0	0	0	0	0	0	0	0	0	0	0	0	0	0	0
Flavobacteria	1	7	0	0	0	0	0	0	0	0	0	0	0	0	0	0	0	0	0	0
Flavobacteriaceae	2	69	0	0	0	0	0	0	0	0	0	0	0	0	0	0	0	0	0	0
Flavobacteriales	1	3	0	0	0	0	0	0	0	0	0	0	0	0	0	0	0	0	0	0
Flavobacterium	2	103	0	0	0	0	0	0	0	0	0	0	0	0	0	0	0	0	0	0
Flexibacter	1	17	0	0	0	0	0	0	0	0	0	0	0	0	0	0	0	0	0	0
Flexistipes	1	11	0	0	0	0	0	0	0	0	0	0	0	0	0	0	0	0	0	0
Fluoribacter	1	6	0	0	0	0	0	0	0	0	0	0	0	0	0	0	0	0	0	0
Fluviicola	2	69	0	0	0	0	0	0	0	0	0	0	0	0	0	0	0	0	0	0
Formosa	1	2	0	0	0	0	0	0	0	0	0	0	0	0	0	0	0	0	0	0
Francisella	2	88	0	0	0	0	0	0	0	0	0	0	0	0	0	0	0	0	0	0
Frankia	3	202	0	0	0	0	0	0	0	0	0	0	0	0	0	0	0	0	0	1
Frateuria	1	25	0	0	0	0	0	0	0	0	0	0	0	0	0	0	0	0	0	0
Fructobacillus	1	2	0	0	0	0	0	0	0	0	0	0	0	0	0	0	0	0	0	0
Fulvimarina	1	2	0	0	0	0	0	0	0	0	0	0	0	0	0	0	0	0	0	0
Fusobacterium	1	48	0	0	0	0	0	0	0	0	0	0	0	0	0	0	0	0	0	0
Gallibacterium	2	87	0	0	0	0	0	0	0	0	0	0	0	0	0	0	0	0	0	0
Gallionella	2	67	0	0	0	0	0	0	0	0	0	0	0	0	0	0	0	0	0	0
Gammaproteobacteria	1	9	0	0	0	0	0	0	0	0	0	0	0	0	0	0	0	0	0	0
Gardnerella	1	31	0	0	0	0	0	0	0	0	0	0	0	0	0	0	0	0	0	0
Geitlerinema	1	9	0	0	0	0	0	0	0	0	0	0	0	0	0	0	0	0	0	0
Gemmatimonas	2	93	0	0	0	0	0	0	0	0	0	0	0	0	0	0	0	0	0	0
Geobacillus	2	168	0	0	0	0	0	0	0	0	0	0	0	0	0	0	0	0	0	0
Geobacter	2	150	0	0	0	0	0	0	0	0	0	0	0	0	0	0	0	0	0	0
Geodermatophilus	2	136	0	0	0	0	0	0	0	0	0	0	0	0	0	0	0	0	0	0
Geopsychrobacter	1	2	0	0	0	0	0	0	0	0	0	0	0	0	0	0	0	0	0	0
Gillisia	1	11	0	0	0	0	0	0	0	0	0	0	0	0	0	0	0	0	0	0
Gilvimarinus	1	4	0	0	0	0	0	0	0	0	0	0	0	0	0	0	0	0	0	0
Glaciecola	2	117	0	0	0	0	0	0	0	0	0	0	0	0	0	0	0	0	0	0

Genus	Cluster Number	Protein Counts	K15061	K15062	K15063	K15064	K15065	K15066	K15236	K15237	K15238	K15242	K15247	K15253	K15749	K15751	K15754	K15756	K15757	K15981
**Gloeobacter**	2	67	0	0	0	0	0	0	0	0	0	0	0	0	0	0	0	0	0	0
**Gloeocapsa**	1	20	0	0	0	0	0	0	0	0	0	0	0	0	0	0	0	0	0	0
**Gloeothece**	1	1	0	0	0	0	0	0	0	0	0	0	0	0	0	0	0	0	0	0
**Gluconacetobacter**	2	96	0	0	0	0	0	0	0	0	0	0	0	0	0	0	0	0	0	0
**Gluconobacter**	2	76	0	0	0	0	0	0	0	0	0	0	0	0	0	0	0	0	0	0
**Gordonia**	1	158	0	0	0	0	0	0	0	0	0	0	0	0	0	0	0	0	0	1
**Gordonibacter**	1	32	0	0	0	0	0	0	0	0	0	0	0	0	0	0	0	0	0	0
**Gramella**	2	104	0	0	0	0	0	0	0	0	0	0	0	0	0	0	0	0	0	0
**Granulibacter**	2	90	0	0	0	0	0	0	0	0	0	0	0	0	0	0	0	0	0	0
**Granulicella**	2	98	0	0	0	0	0	0	0	0	0	0	0	0	0	0	0	0	0	0
**Grimontia**	1	9	0	0	0	0	0	0	0	0	0	0	0	0	0	0	0	0	0	0
**Haemophilus**	2	117	0	0	0	0	0	0	0	0	0	0	0	0	0	0	0	0	0	0
**Hafnia**	1	29	0	0	0	0	0	0	0	0	0	0	0	0	0	0	0	0	0	0
**Hahella**	3	130	0	0	0	0	0	0	0	0	0	0	0	0	0	0	0	0	0	0
**Halalkalicoccus**	1	18	0	0	0	0	0	0	0	0	0	0	0	0	0	0	0	0	0	0
**Halanaerobium**	2	89	0	0	0	0	0	0	0	0	0	0	0	0	0	0	0	0	0	0
**Haliangium**	2	108	0	0	0	0	0	0	0	0	0	0	0	0	0	0	0	0	0	0
**Haliscomenobacter**	2	107	0	0	0	0	0	0	0	0	0	0	0	0	0	0	0	0	0	0
**Haloarcula**	1	22	0	0	0	0	0	0	0	0	0	0	0	0	0	0	0	0	0	0
**Halobacillus**	1	19	0	0	0	0	0	0	0	0	0	0	0	0	0	0	0	0	0	0
**Halobacteriaceae**	1	1	0	0	0	0	0	0	0	0	0	0	0	0	0	0	0	0	0	0
**Halobacterium**	1	10	0	0	0	0	0	0	0	0	0	0	0	0	0	0	0	0	0	0
**Halobacteroides**	1	14	0	0	0	0	0	0	0	0	0	0	0	0	0	0	0	0	0	0
**Haloferax**	1	20	0	0	0	0	0	0	0	0	0	0	0	0	0	0	0	0	0	0
**Halogeometricum**	1	12	0	0	0	0	0	0	0	0	0	0	0	0	0	0	0	0	0	0
**Halomicrobium**	1	10	0	0	0	0	0	0	0	0	0	0	0	0	0	0	0	0	0	0
**Halomonas**	1	149	0	0	0	0	0	0	0	0	0	0	0	0	0	0	0	0	0	0
**Halopiger**	1	16	0	0	0	0	0	0	0	0	0	0	0	0	0	0	0	0	0	0
**Haloquadratum**	1	19	0	0	0	0	0	0	0	0	0	0	0	0	0	0	0	0	0	0
**Halorhabdus**	1	10	0	0	0	0	0	0	0	0	0	0	0	0	0	0	0	0	0	0
**Halorhodospira**	2	71	0	0	0	0	0	0	0	0	0	0	0	0	0	0	0	0	0	0
**Halorubrum**	1	16	0	0	0	0	0	0	0	0	0	0	0	0	0	0	0	0	0	0
**Haloterrigena**	1	19	0	0	0	0	0	0	0	0	0	0	0	0	0	0	0	0	0	0
**Halothece**	1	9	0	0	0	0	0	0	0	0	0	0	0	0	0	0	0	0	0	0
**Halothermothrix**	2	50	0	0	0	0	0	0	0	0	0	0	0	0	0	0	0	0	0	0
**Halothiobacillus**	2	65	0	0	0	0	0	0	0	0	0	0	0	0	0	0	0	0	0	0
**Halovivax**	1	12	0	0	0	0	0	0	0	0	0	0	0	0	0	0	0	0	0	0
**Helicobacter**	2	79	0	0	0	0	0	0	0	0	0	0	0	0	0	0	0	0	0	0
**Heliobacillus**	1	4	0	0	0	0	0	0	0	0	0	0	0	0	0	0	0	0	0	0
**Heliobacterium**	2	51	0	0	0	0	0	0	0	0	0	0	0	0	0	0	0	0	0	0
**Herbaspirillum**	2	172	0	0	0	0	0	0	0	0	0	0	0	0	0	0	0	0	0	0
**Herminiimonas**	3	89	0	0	0	0	0	0	0	0	0	0	0	0	0	0	0	0	0	0
**Herpetosiphon**	3	90	0	0	0	0	0	0	0	0	0	0	0	0	0	0	0	0	0	0
**Hippea**	2	44	0	0	0	0	0	0	0	0	0	0	0	0	0	0	0	0	0	0
**Hirschia**	2	102	0	0	0	0	0	0	0	0	0	0	0	0	0	0	0	0	0	0
**Histophilus**	2	72	0	0	0	0	0	0	0	0	0	0	0	0	0	0	0	0	0	0
**Hoeflea**	1	16	0	0	0	0	0	0	0	0	0	0	0	0	0	0	0	0	0	0
**Hydrogenivirga**	1	2	0	0	0	0	0	0	0	0	0	0	0	0	0	0	0	0	0	0
**Hydrogenobacter**	2	55	0	0	0	0	0	0	0	0	0	0	0	0	0	0	0	0	0	0
**Hydrogenobaculum**	2	44	0	0	0	0	0	0	0	0	0	0	0	0	0	0	0	0	0	0
**Hydrogenophaga**	1	9	0	0	0	0	0	0	0	0	0	0	0	0	0	0	0	0	0	0
**Hylemonella**	1	4	0	0	0	0	0	0	0	0	0	0	0	0	0	0	0	0	0	0

Genus	Cluster Number	Protein Counts	K15061	K15062	K15063	K15064	K15065	K15066	K15236	K15237	K15238	K15242	K15247	K15253	K15749	K15751	K15754	K15756	K15757	K15981
Hyperthermus	1	6	0	0	0	0	0	0	0	0	0	0	0	0	0	0	0	0	0	0
Hyphomicrobium	2	93	0	0	0	0	0	0	0	0	0	0	0	0	0	0	0	0	0	0
Hyphomonas	3	105	0	0	0	0	0	0	0	0	0	0	0	0	0	0	0	0	0	0
Ideonella	1	18	0	0	0	0	0	0	0	0	0	0	0	0	0	0	0	0	0	0
Idiomarina	2	86	0	0	0	0	0	0	0	0	0	0	0	0	0	0	0	0	0	0
Ignavibacterium	1	14	0	0	0	0	0	0	0	0	0	0	0	0	0	0	0	0	0	0
Ignicoccus	1	5	0	0	0	0	0	0	0	0	0	0	0	0	0	0	0	0	0	0
Ignisphaera	1	8	0	0	0	0	0	0	0	0	0	0	0	0	0	0	0	0	0	0
Ilyobacter	2	85	0	0	0	0	0	0	0	0	0	0	0	0	0	0	0	0	0	0
Intrasporangium	2	114	0	0	0	0	0	0	0	0	0	0	0	0	0	0	0	0	0	0
Isoptericola	1	18	0	0	0	0	0	0	0	0	0	0	0	0	0	0	0	0	0	0
Isosphaera	2	75	0	0	0	0	0	0	0	0	0	0	0	0	0	0	0	0	0	0
Jannaschia	3	151	0	0	0	0	0	0	0	0	0	0	0	0	0	0	0	0	0	0
Janthinobacterium	2	99	0	0	0	0	0	0	0	0	0	0	0	0	0	0	0	0	0	0
Jonesia	2	56	0	0	0	0	0	0	0	0	0	0	0	0	0	0	0	0	0	0
Joostella	1	3	0	0	0	0	0	0	0	0	0	0	0	0	0	0	0	0	0	0
Kaistia	1	2	0	0	0	0	0	0	0	0	0	0	0	0	0	0	0	0	0	0
Kangiella	2	84	0	0	0	0	0	0	0	0	0	0	0	0	0	0	0	0	0	0
Ketogulonicigenium	2	102	0	0	0	0	0	0	0	0	0	0	0	0	0	0	0	0	0	0
Kineococcus	1	24	0	0	0	0	0	0	0	0	0	0	0	0	0	0	0	0	0	0
Kinetoplastibacterium	1	2	0	0	0	0	0	0	0	0	0	0	0	0	0	0	0	0	0	0
Kingella	1	4	0	0	0	0	0	0	0	0	0	0	0	0	0	0	0	0	0	0
Kitasatospora	1	36	0	0	0	0	0	0	0	0	0	0	0	0	0	0	0	0	0	0
Klebsiella	3	222	0	0	0	0	0	0	0	0	0	0	0	0	0	0	0	0	0	0
Kluyvera	1	1	0	0	0	0	0	0	0	0	0	0	0	0	0	0	0	0	0	0
Kocuria	2	87	0	0	0	0	0	0	0	0	0	0	0	0	0	0	0	0	0	0
Kordia	1	5	0	0	0	0	0	0	0	0	0	0	0	0	0	0	0	0	0	0
Kosmotoga	2	61	0	0	0	0	0	0	0	0	0	0	0	0	0	0	0	0	0	0
Kribbella	2	99	0	0	0	0	0	0	0	0	0	0	0	0	0	0	0	0	0	0
Krokinobacter	2	90	0	0	0	0	0	0	0	0	0	0	0	0	0	0	0	0	0	0
Kyrpidia	2	113	0	0	0	0	0	0	0	0	0	0	0	0	0	0	0	0	0	0
Kytococcus	2	70	0	0	0	0	0	0	0	0	0	0	0	0	0	0	0	0	0	0
Labrenzia	1	4	0	0	0	0	0	0	0	0	0	0	0	0	0	0	0	0	0	0
Lachnoanaerobaculum	1	1	0	0	0	0	0	0	0	0	0	0	0	0	0	0	0	0	0	0
Lachnospiraceae	1	51	0	0	0	0	0	0	0	0	0	0	0	0	0	0	0	0	0	0
Lacinutrix	1	20	0	0	0	0	0	0	0	0	0	0	0	0	0	0	0	0	0	0
Lactobacillus	2	119	0	0	0	0	0	0	0	0	0	0	0	0	0	0	0	0	0	0
Lactococcus	2	82	0	0	0	0	0	0	0	0	0	0	0	0	0	0	0	0	0	0
Lamprocystis	1	2	0	0	0	0	0	0	0	0	0	0	0	0	0	0	0	0	0	0
Laribacter	2	80	0	0	0	0	0	0	0	0	0	0	0	0	0	0	0	0	0	0
Lawsonia	2	33	0	0	0	0	0	0	0	0	0	0	0	0	0	0	0	0	0	0
Leadbetterella	2	79	0	0	0	0	0	0	0	0	0	0	0	0	0	0	0	0	0	0
Leeuwenhoekiella	1	2	0	0	0	0	0	0	0	0	0	0	0	0	0	0	0	0	0	0
Legionella	2	117	0	0	0	0	0	0	0	0	0	0	0	0	0	0	0	0	0	0
Leifsonia	2	48	0	0	0	0	0	0	0	0	0	0	0	0	0	0	0	0	0	0
Leptolyngbya	1	10	0	0	0	0	0	0	0	0	0	0	0	0	0	0	0	0	0	0
Leptospira	2	76	0	0	0	0	0	0	0	0	0	0	0	0	0	0	0	0	0	0
Leptospirillum	1	10	0	0	0	0	0	0	0	0	0	0	0	0	0	0	0	0	0	0
Leptothrix	3	186	0	0	0	0	0	0	0	0	0	0	0	0	0	0	0	0	0	0
Leptotrichia	2	43	0	0	0	0	0	0	0	0	0	0	0	0	0	0	0	0	0	0
Leucobacter	1	2	0	0	0	0	0	0	0	0	0	0	0	0	0	0	0	0	0	0
Leuconostoc	2	64	0	0	0	0	0	0	0	0	0	0	0	0	0	0	0	0	0	0

Genus	Cluster Number	Protein Counts	K15061	K15062	K15063	K15064	K15065	K15066	K15236	K15237	K15238	K15242	K15247	K15253	K15749	K15751	K15754	K15756	K15757	K15981
Liberibacter	1	4	0	0	0	0	0	0	0	0	0	0	0	0	0	0	0	0	0	0
Limnohabitans	1	11	0	0	0	0	0	0	0	0	0	0	0	0	0	0	0	0	0	0
Listeria	2	77	0	0	0	0	0	0	0	0	0	0	0	0	0	0	0	0	0	0
Listeriaceae	1	6	0	0	0	0	0	0	0	0	0	0	0	0	0	0	0	0	0	0
Listonella	1	38	0	0	0	0	0	0	0	0	0	0	0	0	0	0	0	0	0	0
Loktanella	1	3	0	0	0	0	0	0	0	0	0	0	0	0	0	0	0	0	0	0
Lysinibacillus	2	97	0	0	0	0	0	0	0	0	0	0	0	0	0	0	0	0	0	0
Macrococcus	2	60	0	0	0	0	0	0	0	0	0	0	0	0	0	0	0	0	0	0
Magnetococcus	2	64	0	0	0	0	0	0	0	0	0	0	0	0	0	0	0	0	0	0
Magnetospirillum	2	113	0	0	0	0	0	0	0	0	0	0	0	0	0	0	0	0	0	0
Mahella	2	58	0	0	0	0	0	0	0	0	0	0	0	0	0	0	0	0	0	0
Mannheimia	2	85	0	0	0	0	0	0	0	0	0	0	0	0	0	0	0	0	0	0
Maribacter	3	87	0	0	0	0	0	0	0	0	0	0	0	0	0	0	0	0	0	0
Maricaulis	3	87	0	0	0	0	0	0	0	0	0	0	0	0	0	0	0	0	0	0
Marichromatium	1	2	0	0	0	0	0	0	0	0	0	0	0	0	0	0	0	0	0	0
Marinithermus	2	77	0	0	0	0	0	0	0	0	0	0	0	0	0	0	0	0	0	0
Marinitoga	1	15	0	0	0	0	0	0	0	0	0	0	0	0	0	0	0	0	0	0
Marinobacter	2	163	0	0	0	0	0	0	0	0	0	0	0	0	0	0	0	0	0	0
Marinomonas	3	192	0	0	0	0	0	0	0	0	0	0	0	0	0	0	0	0	0	0
Maritimibacter	1	7	0	0	0	0	0	0	0	0	0	0	0	0	0	0	0	0	0	0
Marivirga	2	96	0	0	0	0	0	0	0	0	0	0	0	0	0	0	0	0	0	0
Martelella	1	3	0	0	0	0	0	0	0	0	0	0	0	0	0	0	0	0	0	0
Marvinbryantia	1	1	0	0	0	0	0	0	0	0	0	0	0	0	0	0	0	0	0	0
Massilia	1	4	0	0	0	0	0	0	0	0	0	0	0	0	0	0	0	0	0	0
Mastigocladopsis	1	16	0	0	0	0	0	0	0	0	0	0	0	0	0	0	0	0	0	0
Megamonas	1	47	0	0	0	0	0	0	0	0	0	0	0	0	0	0	0	0	0	0
Megasphaera	1	14	0	0	0	0	0	0	0	0	0	0	0	0	0	0	0	0	0	0
Meiothermus	2	110	0	0	0	0	0	0	0	0	0	0	0	0	0	0	0	0	0	0
Melissococcus	2	46	0	0	0	0	0	0	0	0	0	0	0	0	0	0	0	0	0	0
Mesoflavibacter	1	6	0	0	0	0	0	0	0	0	0	0	0	0	0	0	0	0	0	0
Mesoplasma	1	23	0	0	0	0	0	0	0	0	0	0	0	0	0	0	0	0	0	0
Mesorhizobium	2	201	0	0	0	0	0	0	0	0	0	0	0	0	0	0	0	0	0	0
Mesotoga	1	14	0	0	0	0	0	0	0	0	0	0	0	0	0	0	0	0	0	0
Metallosphaera	1	18	0	0	0	0	0	0	0	0	0	0	0	0	0	0	0	0	0	0
Methanobacterium	1	7	0	0	0	0	0	0	0	0	0	0	0	0	0	0	0	0	0	0
Methanobrevibacter	1	9	0	0	0	0	0	0	0	0	0	0	0	0	0	0	0	0	0	0
Methanocaldococcus	1	6	0	0	0	0	0	0	0	0	0	0	0	0	0	0	0	0	0	0
Methanocella	1	15	0	0	0	0	0	0	0	0	0	0	0	0	0	0	0	0	0	0
Methanococcaceae	1	1	0	0	0	0	0	0	0	0	0	0	0	0	0	0	0	0	0	0
Methanococcoides	1	5	0	0	0	0	0	0	0	0	0	0	0	0	0	0	0	0	0	0
Methanococcus	1	9	0	0	0	0	0	0	0	0	0	0	0	0	0	0	0	0	0	0
Methanocorpusculum	1	7	0	0	0	0	0	0	0	0	0	0	0	0	0	0	0	0	0	0
Methanoculleus	1	8	0	0	0	0	0	0	0	0	0	0	0	0	0	0	0	0	0	0
Methanohalobium	1	5	0	0	0	0	0	0	0	0	0	0	0	0	0	0	0	0	0	0
Methanohalophilus	1	5	0	0	0	0	0	0	0	0	0	0	0	0	0	0	0	0	0	0
Methanolobus	1	5	0	0	0	0	0	0	0	0	0	0	0	0	0	0	0	0	0	0
Methanomethylovorans	1	5	0	0	0	0	0	0	0	0	0	0	0	0	0	0	0	0	0	0
Methanoplanus	1	7	0	0	0	0	0	0	0	0	0	0	0	0	0	0	0	0	0	0
Methanopyrus	1	4	0	0	0	0	0	0	0	0	0	0	0	0	0	0	0	0	0	0
Methanoregula	1	8	0	0	0	0	0	0	0	0	0	0	0	0	0	0	0	0	0	0
Methanosaeta	1	9	0	0	0	0	0	0	0	0	0	0	0	0	0	0	0	0	0	0
Methanosalsum	1	7	0	0	0	0	0	0	0	0	0	0	0	0	0	0	0	0	0	0

Genus	Cluster Number	Protein Counts	K15061	K15062	K15063	K15064	K15065	K15066	K15236	K15237	K15238	K15242	K15247	K15253	K15749	K15751	K15754	K15756	K15757	K15981
**Methanosarcina**	1	18	0	0	0	0	0	0	0	0	0	0	0	0	0	0	0	0	0	0
**Methanosphaera**	1	7	0	0	0	0	0	0	0	0	0	0	0	0	0	0	0	0	0	0
**Methanosphaerula**	1	8	0	0	0	0	0	0	0	0	0	0	0	0	0	0	0	0	0	0
**Methanospirillum**	1	6	0	0	0	0	0	0	0	0	0	0	0	0	0	0	0	0	0	0
**Methanothermobacter**	1	6	0	0	0	0	0	0	0	0	0	0	0	0	0	0	0	0	0	0
**Methanothermococcus**	1	5	0	0	0	0	0	0	0	0	0	0	0	0	0	0	0	0	0	0
**Methanothermus**	1	5	0	0	0	0	0	0	0	0	0	0	0	0	0	0	0	0	0	0
**Methanotorris**	1	5	0	0	0	0	0	0	0	0	0	0	0	0	0	0	0	0	0	0
**Methylacidiphilum**	2	48	0	0	0	0	0	0	0	0	0	0	0	0	0	0	0	0	0	0
**Methylarcula**	1	2	0	0	0	0	0	0	0	0	0	0	0	0	0	0	0	0	0	0
**Methylibium**	1	38	0	0	0	0	0	0	0	0	0	0	0	0	0	0	0	0	0	0
**Methylobacillus**	2	61	0	0	0	0	0	0	0	0	0	0	0	0	0	0	0	0	0	0
**Methylobacterium**	3	236	0	0	0	0	0	0	0	0	0	0	0	0	0	0	0	0	0	0
**Methylocella**	3	117	0	0	0	0	0	0	0	0	0	0	0	0	0	0	0	0	0	0
**Methylococcus**	2	80	0	0	0	0	0	0	0	0	0	0	0	0	0	0	0	0	0	0
**Methylocystis**	1	21	0	0	0	0	0	0	0	0	0	0	0	0	0	0	0	0	0	0
**Methyloferula**	1	2	0	0	0	0	0	0	0	0	0	0	0	0	0	0	0	0	0	0
**Methylomicrobium**	1	12	0	0	0	0	0	0	0	0	0	0	0	0	0	0	0	0	0	0
**Methylomonas**	1	15	0	0	0	0	0	0	0	0	0	0	0	0	0	0	0	0	0	0
**Methylophaga**	1	7	0	0	0	0	0	0	0	0	0	0	0	0	0	0	0	0	0	0
**Methylophilus**	1	2	0	0	0	0	0	0	0	0	0	0	0	0	0	0	0	0	0	0
**Methylosinus**	1	2	0	0	0	0	0	0	0	0	0	0	0	0	0	0	0	0	0	0
**Methylotenera**	2	72	0	0	0	0	0	0	0	0	0	0	0	0	0	0	0	0	0	0
**Methyloversatilis**	1	5	0	0	0	0	0	0	0	0	0	0	0	0	0	0	0	0	0	0
**Methylovorus**	2	66	0	0	0	0	0	0	0	0	0	0	0	0	0	0	0	0	0	0
**Micavibrio**	1	9	0	0	0	0	0	0	0	0	0	0	0	0	0	0	0	0	0	0
**Microbacterium**	2	115	0	0	0	0	0	0	0	0	0	0	0	0	0	0	0	0	0	0
**Microchaete**	1	23	0	0	0	0	0	0	0	0	0	0	0	0	0	0	0	0	0	0
**Micrococcus**	2	72	0	0	0	0	0	0	0	0	0	0	0	0	0	0	0	0	0	0
**Microcoleus**	1	17	0	0	0	0	0	0	0	0	0	0	0	0	0	0	0	0	0	0
**Microcystis**	2	67	0	0	0	0	0	0	0	0	0	0	0	0	0	0	0	0	0	0
**Microlunatus**	1	25	0	0	0	0	0	0	0	0	0	0	0	0	0	0	0	0	0	0
**Micromonospora**	2	115	0	0	0	0	0	0	0	0	0	0	0	0	0	0	0	0	0	0
**Microvirga**	1	6	0	0	0	0	0	0	0	0	0	0	0	0	0	0	0	0	0	0
**Mobiluncus**	1	41	0	0	0	0	0	0	0	0	0	0	0	0	0	0	0	0	0	0
**Modestobacter**	1	43	0	0	0	0	0	1	0	0	0	0	0	0	0	0	0	0	0	0
**Moorea**	1	2	0	0	0	0	0	0	0	0	0	0	0	0	0	0	0	0	0	0
**Moorella**	2	69	0	0	0	0	0	0	0	0	0	0	0	0	0	0	0	0	0	0
**Moraxella**	2	66	0	0	0	0	0	0	0	0	0	0	0	0	0	0	0	0	0	0
**Morganella**	1	29	0	0	0	0	0	0	0	0	0	0	0	0	0	0	0	0	0	0
**Moritella**	1	2	0	0	0	0	0	0	0	0	0	0	0	0	0	0	0	0	0	0
**Muricauda**	1	23	0	0	0	0	0	0	0	0	0	0	0	0	0	0	0	0	0	0
**Mycobacterium**	3	306	0	0	0	0	0	0	0	0	0	0	0	0	0	0	0	0	0	1
**Mycoplasma**	1	53	0	0	0	0	0	0	0	0	0	0	0	0	0	0	0	0	0	0
**Myxococcus**	2	110	0	0	0	0	0	0	0	0	0	0	0	0	0	0	0	0	0	0
**Nakamurella**	2	115	0	0	0	0	0	0	0	0	0	0	0	0	0	0	0	0	0	0
**napthalene**	1	2	0	0	0	0	0	0	0	0	0	0	0	0	0	0	0	0	0	0
**Natranaerobius**	2	55	0	0	0	0	0	0	0	0	0	0	0	0	0	0	0	0	0	0
**Natrialba**	1	16	0	0	0	0	0	0	0	0	0	0	0	0	0	0	0	0	0	0
**Natrinema**	1	16	0	0	0	0	0	0	0	0	0	0	0	0	0	0	0	0	0	0
**Natronobacterium**	1	11	0	0	0	0	0	0	0	0	0	0	0	0	0	0	0	0	0	0
**Natronococcus**	1	21	0	0	0	0	0	0	0	0	0	0	0	0	0	0	0	0	0	0

Genus	Cluster Number	Protein Counts	K15061	K15062	K15063	K15064	K15065	K15066	K15236	K15237	K15238	K15242	K15247	K15253	K15749	K15751	K15754	K15756	K15757	K15981
**Natronomonas**	1	20	0	0	0	0	0	0	0	0	0	0	0	0	0	0	0	0	0	0
**Nautilia**	2	48	0	0	0	0	0	0	0	0	0	0	0	0	0	0	0	0	0	0
**Neisseria**	2	67	0	0	0	0	0	0	0	0	0	0	0	0	0	0	0	0	0	0
**Neisseriaceae**	1	1	0	0	0	0	0	0	0	0	0	0	0	0	0	0	0	0	0	0
**Neorickettsia**	1	31	0	0	0	0	0	0	0	0	0	0	0	0	0	0	0	0	0	0
**Neptuniibacter**	1	2	0	0	0	0	0	0	0	0	0	0	0	0	0	0	0	0	0	0
**Niastella**	1	21	0	0	0	0	0	0	0	0	0	0	0	0	0	0	0	0	0	0
**Nitratifractor**	2	54	0	0	0	0	0	0	0	0	0	0	0	0	0	0	0	0	0	0
**Nitratireductor**	1	19	0	0	0	0	0	0	0	0	0	0	0	0	0	0	0	0	0	0
**Nitratiruptor**	2	40	0	0	0	0	0	0	0	0	0	0	0	0	0	0	0	0	0	0
**Nitrobacter**	2	99	0	0	0	0	0	0	0	0	0	0	0	0	0	0	0	0	0	0
**Nitrococcus**	1	2	0	0	0	0	0	0	0	0	0	0	0	0	0	0	0	0	0	0
**Nitrosococcus**	2	94	0	0	0	0	0	0	0	0	0	0	0	0	0	0	0	0	0	0
**Nitrosomonas**	2	72	0	0	0	0	0	0	0	0	0	0	0	0	0	0	0	0	0	0
**Nitrosopumilaceae**	1	7	0	0	0	0	0	0	0	0	0	0	0	0	0	0	0	0	0	0
**Nitrosopumilus**	1	11	0	0	0	0	0	0	0	0	0	0	0	0	0	0	0	0	0	0
**Nitrosospira**	2	74	0	0	0	0	0	0	0	0	0	0	0	0	0	0	0	0	0	0
**Nocardia**	2	185	0	0	0	0	0	0	0	0	0	0	0	0	0	0	0	0	0	1
**Nocardioidaceae**	1	2	0	0	0	0	0	0	0	0	0	0	0	0	0	0	0	0	0	0
**Nocardioides**	2	157	0	0	0	0	0	0	0	0	0	0	0	0	0	0	0	0	0	1
**Nocardiopsis**	2	102	0	0	0	0	0	0	0	0	0	0	0	0	0	0	0	0	0	0
**Nodularia**	1	22	0	0	0	0	0	0	0	0	0	0	0	0	0	0	0	0	0	0
**Nonlabens**	1	19	0	0	0	0	0	0	0	0	0	0	0	0	0	0	0	0	0	0
**Nostoc**	2	111	0	0	0	0	0	0	0	0	0	0	0	0	0	0	0	0	0	0
**Nostocaceae**	1	2	0	0	0	0	0	0	0	0	0	0	0	0	0	0	0	0	0	0
**Novosphingobium**	2	169	0	0	0	1	0	1	0	0	0	0	0	0	0	0	0	0	0	0
**Oceanibaculum**	1	3	0	0	0	0	0	0	0	0	0	0	0	0	0	0	0	0	0	0
**Oceanibulbus**	1	12	0	0	0	0	0	0	0	0	0	0	0	0	0	0	0	0	0	0
**Oceanicola**	1	9	0	0	0	0	0	0	0	0	0	0	0	0	0	0	0	0	0	0
**Oceanimonas**	1	33	0	0	0	0	0	0	0	0	0	0	0	0	0	0	0	0	0	0
**Oceaniovalibus**	1	2	0	0	0	0	0	0	0	0	0	0	0	0	0	0	0	0	0	0
**Oceanithermus**	2	72	0	0	0	0	0	0	0	0	0	0	0	0	0	0	0	0	0	0
**Oceanobacillus**	2	91	0	0	0	0	0	0	0	0	0	0	0	0	0	0	0	0	0	0
**Oceanospirillum**	1	2	0	0	0	0	0	0	0	0	0	0	0	0	0	0	0	0	0	0
**Ochrobactrum**	2	133	0	0	0	0	0	0	0	0	0	0	0	0	0	0	0	0	0	0
**Octadecabacter**	1	29	0	0	0	0	0	0	0	0	0	0	0	0	0	0	0	0	0	0
**Oenococcus**	2	41	0	0	0	0	0	0	0	0	0	0	0	0	0	0	0	0	0	0
**Oleomonas**	1	2	0	0	0	0	0	0	0	0	0	0	0	0	0	0	0	0	0	0
**Oligotropha**	3	87	0	0	0	0	0	0	0	0	0	0	0	0	0	0	0	0	0	0
**Olsenella**	2	40	0	0	0	0	0	0	0	0	0	0	0	0	0	0	0	0	0	0
**Opitutus**	2	67	0	0	0	0	0	0	0	0	0	0	0	0	0	0	0	0	0	0
**Orientia**	1	21	0	0	0	0	0	0	0	0	0	0	0	0	0	0	0	0	0	0
**Ornithobacterium**	1	11	0	0	0	0	0	0	0	0	0	0	0	0	0	0	0	0	0	0
**Oscillatoria**	1	16	0	0	0	0	0	0	0	0	0	0	0	0	0	0	0	0	0	0
**Oscillatoriales**	1	2	0	0	0	0	0	0	0	0	0	0	0	0	0	0	0	0	0	0
**Oscillibacter**	1	20	0	0	0	0	0	0	0	0	0	0	0	0	0	0	0	0	0	0
**Owenweeksia**	1	15	0	0	0	0	0	0	0	0	0	0	0	0	0	0	0	0	0	0
**Oxalobacteraceae**	1	4	0	0	0	0	0	0	0	0	0	0	0	0	0	0	0	0	0	0
**Paenibacillus**	2	152	0	0	0	0	0	0	0	0	0	0	0	0	0	0	0	0	0	0
**Paludibacter**	2	67	0	0	0	0	0	0	0	0	0	0	0	0	0	0	0	0	0	0
**Pandoraea**	1	18	0	0	0	0	0	0	0	0	0	0	0	0	0	0	0	0	0	0
**Pannonibacter**	1	3	0	0	0	0	0	0	0	0	0	0	0	0	0	0	0	0	0	0

Genus	Cluster Number	Protein Counts	K15061	K15062	K15063	K15064	K15065	K15066	K15236	K15237	K15238	K15242	K15247	K15253	K15749	K15751	K15754	K15756	K15757	K15981
Pantholops	1	23	0	0	0	0	0	0	0	0	0	0	0	0	0	0	0	0	0	0
Pantoea	2	181	0	0	0	0	0	0	0	0	0	0	0	0	0	0	0	0	0	0
Parabacteroides	2	64	0	0	0	0	0	0	0	0	0	0	0	0	0	0	0	0	0	0
Parachlamydia	1	11	0	0	0	0	0	0	0	0	0	0	0	0	0	0	0	0	0	0
Paracoccus	2	169	0	0	0	0	0	0	0	0	0	0	0	0	0	0	0	0	0	0
Parvibaculum	3	117	0	0	0	0	0	0	0	0	0	0	0	0	0	0	0	0	0	0
Parvularcula	2	72	0	0	0	0	0	0	0	0	0	0	0	0	0	0	0	0	0	0
Pasteurella	2	85	0	0	0	0	0	0	0	0	0	0	0	0	0	0	0	0	0	0
Pasteurellaceae	1	2	0	0	0	0	0	0	0	0	0	0	0	0	0	0	0	0	0	0
Pectobacterium	1	44	0	0	0	0	0	0	0	0	0	0	0	0	0	0	0	0	0	0
Pediococcus	1	43	0	0	0	0	0	0	0	0	0	0	0	0	0	0	0	0	0	0
Pedobacter	2	88	0	0	0	0	0	0	0	0	0	0	0	0	0	0	0	0	0	0
Pelagibaca	1	10	0	0	0	0	0	0	0	0	0	0	0	0	0	0	0	0	0	0
Pelagibacterium	1	39	0	0	0	0	0	0	0	0	0	0	0	0	0	0	0	0	0	0
Pelobacter	2	85	0	0	0	0	0	0	0	0	0	0	0	0	0	0	0	0	0	0
Pelodictyon	2	62	0	0	0	0	0	0	0	0	0	0	0	0	0	0	0	0	0	0
Pelosinus	1	1	0	0	0	0	0	0	0	0	0	0	0	0	0	0	0	0	0	0
Pelotomaculum	2	60	0	0	0	0	0	0	0	0	0	0	0	0	0	0	0	0	0	0
Peptostreptococcus	1	1	0	0	0	0	0	0	0	0	0	0	0	0	0	0	0	0	0	0
Persephonella	2	55	0	0	0	0	0	0	0	0	0	0	0	0	0	0	0	0	0	0
Petrotoga	2	72	0	0	0	0	0	0	0	0	0	0	0	0	0	0	0	0	0	0
Phaeobacter	2	55	0	0	0	0	0	0	0	0	0	0	0	0	0	0	0	0	0	0
Phenylobacterium	3	103	0	0	0	0	0	0	0	0	0	0	0	0	0	0	0	0	0	0
Photobacterium	2	119	0	0	0	0	0	0	0	0	0	0	0	0	0	0	0	0	0	0
Photorhabdus	2	149	0	0	0	0	0	0	0	0	0	0	0	0	0	0	0	0	0	0
Phycisphaera	1	9	0	0	0	0	0	0	0	0	0	0	0	0	0	0	0	0	0	0
Phyllobacterium	1	6	0	0	0	0	0	0	0	0	0	0	0	0	0	0	0	0	0	0
Picrophilus	1	15	0	0	0	0	0	0	0	0	0	0	0	0	0	0	0	0	0	0
Pirellula	2	85	0	0	0	0	0	0	0	0	0	0	0	0	0	0	0	0	0	0
Planctomyces	2	93	0	0	0	0	0	0	0	0	0	0	0	0	0	0	0	0	0	0
Plautia	1	17	0	0	0	0	0	0	0	0	0	0	0	0	0	0	0	0	0	0
Plesiomonas	1	4	0	0	0	0	0	0	0	0	0	0	0	0	0	0	0	0	0	0
Pleurocapsa	1	23	0	0	0	0	0	0	0	0	0	0	0	0	0	0	0	0	0	0
Polaribacter	2	74	0	0	0	0	0	0	0	0	0	0	0	0	0	0	0	0	0	0
Polaromonas	3	238	0	0	0	0	0	0	0	0	0	0	0	0	0	0	0	0	0	0
Polymorphum	3	173	0	0	0	0	0	0	0	0	0	0	0	0	0	0	0	0	0	0
Polynucleobacter	1	19	0	0	0	0	0	0	0	0	0	0	0	0	0	0	0	0	0	0
Ponticaulis	1	1	0	0	0	0	0	0	0	0	0	0	0	0	0	0	0	0	0	0
Porphyromonas	2	63	0	0	0	0	0	0	0	0	0	0	0	0	0	0	0	0	0	0
Prevotella	1	65	0	0	0	0	0	0	0	0	0	0	0	0	0	0	0	0	0	0
Prochlorococcus	2	68	0	0	0	0	0	0	0	0	0	0	0	0	0	0	0	0	0	0
Propionibacterium	1	62	0	0	0	0	0	0	0	0	0	0	0	0	0	0	0	0	0	0
Prosthecochloris	1	16	0	0	0	0	0	0	0	0	0	0	0	0	0	0	0	0	0	0
Proteobacteria	1	19	0	0	0	0	0	0	0	0	0	0	0	0	0	0	0	0	0	0
Proteus	2	113	0	0	0	0	0	0	0	0	0	0	0	0	0	0	0	0	0	0
Providencia	1	38	0	0	0	0	0	0	0	0	0	0	0	0	0	0	0	0	0	0
Pseudaminobacter	1	10	0	0	0	0	0	0	0	0	0	0	0	0	0	0	0	0	0	0
Pseudanabaena	1	11	0	0	0	0	0	0	0	0	0	0	0	0	0	0	0	0	0	0
Pseudoalteromonas	2	188	0	0	0	0	0	0	0	0	0	0	0	0	0	0	0	0	0	0
Pseudochrobactrum	1	7	0	0	0	0	0	0	0	0	0	0	0	0	0	0	0	0	0	0
Pseudogulbenkiania	1	40	0	0	0	0	0	0	0	0	0	0	0	0	0	0	0	0	0	0
pseudomallei	3	131	0	0	0	0	0	0	0	0	0	0	0	0	0	0	0	0	0	0

Genus	Cluster Number	Protein Counts	K15061	K15062	K15063	K15064	K15065	K15066	K15236	K15237	K15238	K15242	K15247	K15253	K15749	K15751	K15754	K15756	K15757	K15981
**Pseudomonas**	3	381	0	0	0	0	0	0	0	0	0	0	0	1	0	0	0	0	0	0
**Pseudonocardia**	3	180	0	0	0	0	0	1	0	0	0	0	0	0	0	0	0	0	0	0
**Pseudorhodobacter**	1	4	0	0	0	0	0	0	0	0	0	0	0	0	0	0	0	0	0	0
**Pseudovibrio**	1	33	0	0	0	0	0	0	0	0	0	0	0	0	0	0	0	0	0	0
**Pseudoxanthomonas**	2	124	0	0	0	0	0	0	0	0	0	0	0	0	0	1	1	1	1	0
**Psychrobacter**	2	124	0	0	0	0	0	0	0	0	0	0	0	0	0	0	0	0	0	0
**Psychroflexus**	1	22	0	0	0	0	0	0	0	0	0	0	0	0	0	0	0	0	0	0
**Psychromonas**	2	110	0	0	0	0	0	0	0	0	0	0	0	0	0	0	0	0	0	0
**Pusillimonas**	3	111	0	0	0	0	0	0	0	0	0	0	0	0	0	0	0	0	0	0
**Pyrobaculum**	1	15	0	0	0	0	0	0	0	0	0	0	0	0	0	0	0	0	0	0
**Pyrococcus**	1	10	0	0	0	0	0	0	0	0	0	0	0	0	0	0	0	0	0	0
**Pyrolobus**	1	4	0	0	0	0	0	0	0	0	0	0	0	0	0	0	0	0	0	0
**Rahnella**	2	143	0	0	0	0	0	0	0	0	0	0	0	0	0	0	0	0	0	0
**Ralstonia**	3	332	0	0	0	0	1	0	0	0	0	0	1	1	0	0	0	0	0	0
**Ramlibacter**	1	35	0	0	0	0	0	0	0	0	0	0	0	0	0	0	0	0	0	0
**Raoultella**	2	139	0	0	0	0	0	0	0	0	0	0	0	0	0	0	0	0	0	0
**Raphidiopsis**	1	5	0	0	0	0	0	0	0	0	0	0	0	0	0	0	0	0	0	0
**Reinekea**	1	2	0	0	0	0	0	0	0	0	0	0	0	0	0	0	0	0	0	0
**Renibacterium**	2	67	0	0	0	0	0	0	0	0	0	0	0	0	0	0	0	0	0	0
**Rhizobiaceae**	1	3	0	0	0	0	0	0	0	0	0	0	0	0	0	0	0	0	0	0
**Rhizobiales**	1	6	0	0	0	0	0	0	0	0	0	0	0	0	0	0	0	0	0	0
**Rhizobium**	3	257	0	0	0	0	0	1	0	0	0	0	0	0	0	0	0	0	0	0
**Rhizobium/Agrobacterium**	2	87	0	0	0	0	0	0	0	0	0	0	0	0	0	0	0	0	0	0
**Rhodanobacter**	1	21	0	0	0	0	0	0	1	0	0	0	0	0	0	0	0	0	0	0
**Rhodobacter**	3	175	0	0	0	0	0	0	0	0	0	0	0	0	0	0	0	0	0	0
**Rhodobacteraceae**	1	21	0	0	0	0	0	0	0	0	0	0	0	0	0	0	0	0	0	0
**Rhodobacterales**	1	34	0	0	0	0	0	0	0	0	0	0	0	0	0	0	0	0	0	0
**Rhodococcus**	2	292	0	0	0	0	0	0	0	0	0	0	0	0	0	0	0	0	0	1
**Rhodocyclaceae**	1	7	0	0	0	0	0	0	0	0	0	0	0	0	0	0	0	0	0	0
**Rhodoferax**	3	144	0	0	0	0	0	0	0	0	0	0	0	0	0	0	0	0	0	0
**Rhodomicrobium**	2	103	0	0	0	0	0	0	0	0	0	0	0	0	0	0	0	0	0	0
**Rhodopirellula**	2	87	0	0	0	0	0	0	0	0	0	0	0	0	0	0	0	0	0	0
**Rhodopseudomonas**	3	246	0	0	0	0	0	0	0	0	0	0	0	0	0	0	0	0	0	0
**Rhodospirillum**	2	121	0	0	0	0	0	0	0	0	0	0	0	0	0	0	0	0	0	0
**Rhodothermus**	2	76	0	0	0	0	0	0	0	0	0	0	0	0	0	0	0	0	0	0
**Rhodovulum**	1	4	0	0	0	0	0	0	0	0	0	0	0	0	0	0	0	0	0	0
**Richelia**	1	2	0	0	0	0	0	0	0	0	0	0	0	0	0	0	0	0	0	0
**Rickettsia**	2	43	0	0	0	0	0	0	0	0	0	0	0	0	0	0	0	0	0	0
**Rickettsiaceae**	1	5	0	0	0	0	0	0	0	0	0	0	0	0	0	0	0	0	0	0
**Riemerella**	2	61	0	0	0	0	0	0	0	0	0	0	0	0	0	0	0	0	0	0
**Rivularia**	1	18	0	0	0	0	0	0	0	0	0	0	0	0	0	0	0	0	0	0
**Robiginitalea**	2	83	0	0	0	0	0	0	0	0	0	0	0	0	0	0	0	0	0	0
**Roseburia**	2	58	0	0	0	0	0	0	0	0	0	0	0	0	0	0	0	0	0	0
**Roseibium**	1	3	0	0	0	0	0	0	0	0	0	0	0	0	0	0	0	0	0	0
**Roseiflexus**	3	100	0	0	0	0	0	0	0	0	0	0	0	0	0	0	0	0	0	0
**Roseobacter**	2	149	0	0	0	0	0	0	0	0	0	0	0	0	0	0	0	0	0	0
**Roseomonas**	1	2	0	0	0	0	0	0	0	0	0	0	0	0	0	0	0	0	0	0
**Roseovarius**	1	15	0	0	0	0	0	0	0	0	0	0	0	0	0	0	0	0	0	0
**Rothia**	1	44	0	0	0	0	0	0	0	0	0	0	0	0	0	0	0	0	0	0
**Rubrivivax**	1	24	0	0	0	0	0	0	0	0	0	0	0	0	0	0	0	0	0	0
**Rubrobacter**	3	117	0	0	0	0	0	0	0	0	0	0	0	0	0	0	0	0	0	0
**Ruegeria**	2	189	0	0	0	0	0	0	0	0	0	0	0	0	0	0	0	0	0	0

Genus	Cluster Number	Protein Counts	K15061	K15062	K15063	K15064	K15065	K15066	K15236	K15237	K15238	K15242	K15247	K15253	K15749	K15751	K15754	K15756	K15757	K15981
Ruminococcaceae	1	2	0	0	0	0	0	0	0	0	0	0	0	0	0	0	0	0	0	0
Ruminococcus	2	101	0	0	0	0	0	0	0	0	0	0	0	0	0	0	0	0	0	0
Runella	1	26	0	0	0	0	0	0	0	0	0	0	0	0	0	0	0	0	0	0
Saccharomonospora	2	132	0	0	0	0	0	1	0	0	0	0	0	0	0	0	0	0	0	0
Saccharophagus	2	81	0	0	0	0	0	0	0	0	0	0	0	0	0	0	0	0	0	0
Saccharopolyspora	3	194	0	0	0	0	0	0	0	0	0	0	0	0	0	0	0	0	0	0
Saccharothrix	1	32	0	0	0	0	0	0	0	0	0	0	0	0	0	0	0	0	0	0
Sagittula	1	10	0	0	0	0	0	0	0	0	0	0	0	0	0	0	0	0	0	0
Salinibacter	2	77	0	0	0	0	0	0	0	0	0	0	0	0	0	0	0	0	0	0
Salinimonas	1	1	0	0	0	0	0	0	0	0	0	0	0	0	0	0	0	0	0	0
Salinispora	3	127	0	0	0	0	0	0	0	0	0	0	0	0	0	0	0	0	0	1
Salinivibrio	1	2	0	0	0	0	0	0	0	0	0	0	0	0	0	0	0	0	0	0
Salmonella	2	151	0	0	0	0	0	0	0	0	0	0	0	0	0	0	0	0	0	0
Sanguibacter	2	73	0	0	0	0	0	0	0	0	0	0	0	0	0	0	0	0	0	0
Saprospira	1	13	0	0	0	0	0	0	0	0	0	0	0	0	0	0	0	0	0	0
Scardovia	1	1	0	0	0	0	0	0	0	0	0	0	0	0	0	0	0	0	0	0
Scytonema	1	10	0	0	0	0	0	0	0	0	0	0	0	0	0	0	0	0	0	0
Sebaldella	2	69	0	0	0	0	0	0	0	0	0	0	0	0	0	0	0	0	0	0
Segniliparus	2	85	0	0	0	0	0	0	0	0	0	0	0	0	0	0	0	0	0	0
Selenomonas	2	57	0	0	0	0	0	0	0	0	0	0	0	0	0	0	0	0	0	0
Serinicoccus	1	1	0	0	0	0	0	0	0	0	0	0	0	0	0	0	0	0	0	0
Serratia	2	186	0	0	0	0	0	0	0	0	0	0	0	0	0	0	0	0	0	0
Shewanella	3	214	0	0	0	0	0	0	0	0	0	0	0	0	0	0	0	0	0	0
Shigella	2	172	0	0	0	0	0	0	0	0	0	0	0	0	0	0	0	0	0	0
Shinella	1	2	0	0	0	0	0	0	0	0	0	0	0	0	0	0	0	0	0	0
Sideroxydans	2	81	0	0	0	0	0	0	0	0	0	0	0	0	0	0	0	0	0	0
Silicibacter	2	105	0	0	0	0	0	0	0	0	0	0	0	0	0	0	0	0	0	0
Simiduia	1	18	0	0	0	0	0	0	0	0	0	0	0	0	0	0	0	0	0	0
Simkania	1	6	0	0	0	0	0	0	0	0	0	0	0	0	0	0	0	0	0	0
Simonsiella	1	2	0	0	0	0	0	0	0	0	0	0	0	0	0	0	0	0	0	0
Singulisphaera	1	18	0	0	0	0	0	0	0	0	0	0	0	0	0	0	0	0	0	0
Sinorhizobium	3	207	0	0	0	0	0	0	0	0	0	0	0	0	0	0	0	0	0	0
Slackia	2	46	0	0	0	0	0	0	0	0	0	0	0	0	0	0	0	0	0	0
Smaragdicoccus	1	3	0	0	0	0	0	0	0	0	0	0	0	0	0	0	0	0	0	0
Sodalis	2	71	0	0	0	0	0	0	0	0	0	0	0	0	0	0	0	0	0	0
Solibacillus	1	23	0	0	0	0	0	0	0	0	0	0	0	0	0	0	0	0	0	0
Solitalea	1	13	0	0	0	0	0	0	0	0	0	0	0	0	0	0	0	0	0	0
Sorangium	3	115	0	0	0	0	0	0	0	0	0	0	0	0	0	0	0	0	0	0
Sphaerobacter	2	100	0	0	0	0	0	1	0	0	0	0	0	0	0	0	0	0	0	0
Sphaerochaeta	1	42	0	0	0	0	0	0	0	0	0	0	0	0	0	0	0	0	0	0
Sphingobacterium	3	85	0	0	0	0	0	0	0	0	0	0	0	0	0	0	0	0	0	0
Sphingobium	1	179	1	1	1	1	1	1	1	1	1	1	0	1	0	0	0	0	0	0
Sphingomonadaceae	1	15	0	0	0	0	0	0	0	1	1	0	0	0	0	0	0	0	0	0
Sphingomonas	2	220	1	1	1	1	1	0	1	1	1	1	0	0	1	0	0	0	0	0
Sphingopyxis	3	99	0	0	0	0	0	0	0	0	0	0	0	0	0	0	0	0	0	0
Spirochaeta	2	103	0	0	0	0	0	0	0	0	0	0	0	0	0	0	0	0	0	0
Spiroplasma	1	6	0	0	0	0	0	0	0	0	0	0	0	0	0	0	0	0	0	0
Spirosoma	2	107	0	0	0	0	0	0	0	0	0	0	0	0	0	0	0	0	0	0
Stackebrandtia	2	110	0	0	0	0	0	0	0	0	0	0	0	0	0	0	0	0	0	0
Stanieria	1	17	0	0	0	0	0	0	0	0	0	0	0	0	0	0	0	0	0	0
Staphylococcus	2	108	0	0	0	0	0	0	0	0	0	0	0	0	0	0	0	0	0	0
Staphylothermus	1	12	0	0	0	0	0	0	0	0	0	0	0	0	0	0	0	0	0	0

Genus	Cluster Number	Protein Counts	K15061	K15062	K15063	K15064	K15065	K15066	K15236	K15237	K15238	K15242	K15247	K15253	K15749	K15751	K15754	K15756	K15757	K15981
**Stappia**	1	2	0	0	0	0	0	0	0	0	0	0	0	0	0	0	0	0	0	0
**Starkeya**	3	134	0	0	0	0	0	0	0	0	0	0	0	0	0	0	0	0	0	0
**Stenotrophomonas**	3	104	0	0	0	0	0	0	0	0	0	0	0	0	0	0	0	0	0	0
**Stigmatella**	3	129	0	0	0	0	0	0	0	0	0	0	0	0	0	0	0	0	0	0
**Streptobacillus**	1	38	0	0	0	0	0	0	0	0	0	0	0	0	0	0	0	0	0	0
**Streptococcus**	2	121	0	0	0	0	0	0	0	0	0	0	0	0	0	0	0	0	0	0
**Streptomyces**	2	253	0	0	0	0	0	0	0	0	0	0	0	0	0	0	0	0	0	1
**Streptosporangium**	3	148	0	0	0	0	0	0	0	0	0	0	0	0	0	0	0	0	0	1
**Strigomonas**	1	2	0	0	0	0	0	0	0	0	0	0	0	0	0	0	0	0	0	0
**Sulfitobacter**	1	10	0	0	0	0	0	0	0	0	0	0	0	0	0	0	0	0	0	0
**Sulfobacillus**	1	30	0	0	0	0	0	0	0	0	0	0	0	0	0	0	0	0	0	0
**Sulfolobus**	1	23	0	0	0	0	0	0	0	0	0	0	0	0	0	0	0	0	0	0
**Sulfuricurvum**	2	67	0	0	0	0	0	0	0	0	0	0	0	0	0	0	0	0	0	0
**Sulfurihydrogenibium**	2	55	0	0	0	0	0	0	0	0	0	0	0	0	0	0	0	0	0	0
**Sulfurimonas**	2	60	0	0	0	0	0	0	0	0	0	0	0	0	0	0	0	0	0	0
**Sulfurospirillum**	1	48	0	0	0	0	0	0	0	0	0	0	0	0	0	0	0	0	0	0
**Sulfurovum**	2	48	0	0	0	0	0	0	0	0	0	0	0	0	0	0	0	0	0	0
**Symbiobacterium**	2	68	0	0	0	0	0	0	0	0	0	0	0	0	0	0	0	0	0	0
**Synechococcus**	1	34	0	0	0	0	0	0	0	0	0	0	0	0	0	0	0	0	0	0
**Synechocystis**	1	40	0	0	0	0	0	0	0	0	0	0	0	0	0	0	0	0	0	0
**Synergistetes**	1	27	0	0	0	0	0	0	0	0	0	0	0	0	0	0	0	0	0	0
**Syntrophobacter**	2	101	0	0	0	0	0	0	0	0	0	0	0	0	0	0	0	0	0	0
**Syntrophobotulus**	2	71	0	0	0	0	0	0	0	0	0	0	0	0	0	0	0	0	0	0
**Syntrophomonas**	2	58	0	0	0	0	0	0	0	0	0	0	0	0	0	0	0	0	0	0
**Syntrophothermus**	2	60	0	0	0	0	0	0	0	0	0	0	0	0	0	0	0	0	0	0
**Syntrophus**	2	66	0	0	0	0	0	0	0	0	0	0	0	0	0	0	0	0	0	0
**Tannerella**	1	8	0	0	0	0	0	0	0	0	0	0	0	0	0	0	0	0	0	0
**Taylorella**	1	43	0	0	0	0	0	0	0	0	0	0	0	0	0	0	0	0	0	0
**Tepidanaerobacter**	2	55	0	0	0	0	0	0	0	0	0	0	0	0	0	0	0	0	0	0
**Teredinibacter**	2	109	0	0	0	0	0	0	0	0	0	0	0	0	0	0	0	0	0	0
**Terrabacter**	1	1	0	0	0	0	0	0	0	0	0	0	0	0	0	0	0	0	0	0
**Terriglobus**	2	83	0	0	0	0	0	0	0	0	0	0	0	0	0	0	0	0	0	0
**Tetragenococcus**	1	12	0	0	0	0	0	0	0	0	0	0	0	0	0	0	0	0	0	0
**Thalassiobium**	1	11	0	0	0	0	0	0	0	0	0	0	0	0	0	0	0	0	0	0
**Thalassobacter**	1	9	0	0	0	0	0	0	0	0	0	0	0	0	0	0	0	0	0	0
**Thalassolituus**	1	12	0	0	0	0	0	0	0	0	0	0	0	0	0	0	0	0	0	0
**Thalassospira**	1	2	0	0	0	0	0	0	0	0	0	0	0	0	0	0	0	0	0	0
**Thauera**	1	111	0	0	0	0	0	0	0	0	0	0	0	0	0	0	0	0	0	0
**Thermacetogenium**	1	7	0	0	0	0	0	0	0	0	0	0	0	0	0	0	0	0	0	0
**Thermaerobacter**	2	79	0	0	0	0	0	0	0	0	0	0	0	0	0	0	0	0	0	0
**Thermanaerovibrio**	2	58	0	0	0	0	0	0	0	0	0	0	0	0	0	0	0	0	0	0
**Thermincola**	2	62	0	0	0	0	0	0	0	0	0	0	0	0	0	0	0	0	0	0
**Thermoanaerobacter**	2	76	0	0	0	0	0	0	0	0	0	0	0	0	0	0	0	0	0	0
**Thermoanaerobacterium**	2	64	0	0	0	0	0	0	0	0	0	0	0	0	0	0	0	0	0	0
**Thermobacillus**	1	11	0	0	0	0	0	0	0	0	0	0	0	0	0	0	0	0	0	0
**Thermobaculum**	2	62	0	0	0	0	0	0	0	0	0	0	0	0	0	0	0	0	0	0
**Thermobifida**	2	72	0	0	0	0	0	0	0	0	0	0	0	0	0	0	0	0	0	0
**Thermobispora**	2	109	0	0	0	0	0	0	0	0	0	0	0	0	0	0	0	0	0	0
**Thermococcus**	1	13	0	0	0	0	0	0	0	0	0	0	0	0	0	0	0	0	0	0
**Thermocrinis**	2	60	0	0	0	0	0	0	0	0	0	0	0	0	0	0	0	0	0	0
**Thermodesulfatator**	1	10	0	0	0	0	0	0	0	0	0	0	0	0	0	0	0	0	0	0
**Thermodesulfobacterium**	1	9	0	0	0	0	0	0	0	0	0	0	0	0	0	0	0	0	0	0

Genus	Cluster Number	Protein Counts	K15061	K15062	K15063	K15064	K15065	K15066	K15236	K15237	K15238	K15242	K15247	K15253	K15749	K15751	K15754	K15756	K15757	K15981
Thermodesulfobium	1	13	0	0	0	0	0	0	0	0	0	0	0	0	0	0	0	0	0	0
Thermodesulfovibrio	2	60	0	0	0	0	0	0	0	0	0	0	0	0	0	0	0	0	0	0
Thermofilum	1	9	0	0	0	0	0	0	0	0	0	0	0	0	0	0	0	0	0	0
Thermogladius	1	7	0	0	0	0	0	0	0	0	0	0	0	0	0	0	0	0	0	0
Thermomicrobium	2	92	0	0	0	0	0	0	0	0	0	0	0	0	0	0	0	0	0	0
Thermomonospora	2	125	0	0	0	0	0	0	0	0	0	0	0	0	0	0	0	0	0	1
Thermoplasma	1	11	0	0	0	0	0	0	0	0	0	0	0	0	0	0	0	0	0	0
Thermoplasmatales	1	6	0	0	0	0	0	0	0	0	0	0	0	0	0	0	0	0	0	0
Thermoproteus	1	12	0	0	0	0	0	0	0	0	0	0	0	0	0	0	0	0	0	0
Thermosediminibacter	2	64	0	0	0	0	0	0	0	0	0	0	0	0	0	0	0	0	0	0
Thermosipho	2	70	0	0	0	0	0	0	0	0	0	0	0	0	0	0	0	0	0	0
Thermosphaera	1	10	0	0	0	0	0	0	0	0	0	0	0	0	0	0	0	0	0	0
Thermosynechococcus	1	6	0	0	0	0	0	0	0	0	0	0	0	0	0	0	0	0	0	0
Thermotoga	2	80	0	0	0	0	0	0	0	0	0	0	0	0	0	0	0	0	0	0
Thermovibrio	2	58	0	0	0	0	0	0	0	0	0	0	0	0	0	0	0	0	0	0
Thermovirga	1	9	0	0	0	0	0	0	0	0	0	0	0	0	0	0	0	0	0	0
Thermus	2	107	0	0	0	0	0	0	0	0	0	0	0	0	0	0	0	0	0	0
Thioalkalimicrobium	1	4	0	0	0	0	0	0	0	0	0	0	0	0	0	0	0	0	0	0
Thioalkalivibrio	2	91	0	0	0	0	0	0	0	0	0	0	0	0	0	0	0	0	0	0
Thiobaca	1	2	0	0	0	0	0	0	0	0	0	0	0	0	0	0	0	0	0	0
Thiobacillus	2	67	0	0	0	0	0	0	0	0	0	0	0	0	0	0	0	0	0	0
Thiocapsa	1	2	0	0	0	0	0	0	0	0	0	0	0	0	0	0	0	0	0	0
Thiocystis	1	11	0	0	0	0	0	0	0	0	0	0	0	0	0	0	0	0	0	0
Thioflavicoccus	1	13	0	0	0	0	0	0	0	0	0	0	0	0	0	0	0	0	0	0
Thiomicrospira	2	50	0	0	0	0	0	0	0	0	0	0	0	0	0	0	0	0	0	0
Thiomonas	2	85	0	0	0	0	0	0	0	0	0	0	0	0	0	0	0	0	0	0
Thiorhodococcus	1	2	0	0	0	0	0	0	0	0	0	0	0	0	0	0	0	0	0	0
Thiothrix	1	2	0	0	0	0	0	0	0	0	0	0	0	0	0	0	0	0	0	0
Tistrella	1	42	0	0	0	0	0	0	0	0	0	0	0	0	0	0	0	0	0	0
Tolumonas	2	100	0	0	0	0	0	0	0	0	0	0	0	0	0	0	0	0	0	0
Transposon	1	2	0	0	0	0	0	0	0	0	0	0	0	0	0	0	0	0	0	0
Treponema	1	63	0	0	0	0	0	0	0	0	0	0	0	0	0	0	0	0	0	0
Trichodesmium	2	71	0	0	0	0	0	0	0	0	0	0	0	0	0	0	0	0	0	0
Trichormus	1	12	0	0	0	0	0	0	0	0	0	0	0	0	0	0	0	0	0	0
Tropheryma	2	22	0	0	0	0	0	0	0	0	0	0	0	0	0	0	0	0	0	0
Truepera	2	104	0	0	0	0	0	0	0	0	0	0	0	0	0	0	0	0	0	0
Tsukamurella	2	123	0	0	0	0	0	0	0	0	0	0	0	0	0	0	0	0	0	1
Turneriella	1	16	0	0	0	0	0	0	0	0	0	0	0	0	0	0	0	0	0	0
Uliginosibacterium	1	2	0	0	0	0	0	0	0	0	0	0	0	0	0	0	0	0	0	0
Ureaplasma	1	24	0	0	0	0	0	0	0	0	0	0	0	0	0	0	0	0	0	0
Ureibacillus	1	2	0	0	0	0	0	0	0	0	0	0	0	0	0	0	0	0	0	0
Variovorax	3	187	0	0	0	0	0	0	0	0	0	0	0	0	0	0	0	0	0	0
Veillonella	1	54	0	0	0	0	0	0	0	0	0	0	0	0	0	0	0	0	0	0
Verminephrobacter	3	188	0	0	0	0	0	0	0	0	0	0	0	0	0	0	0	0	0	0
Verrucosispora	3	126	0	0	0	0	0	0	0	0	0	0	0	0	0	0	0	0	0	0
Vibrio	2	195	0	0	0	0	0	0	0	0	0	0	0	0	0	0	0	0	0	0
Vibrionales	1	46	0	0	0	0	0	0	0	0	0	0	0	0	0	0	0	0	0	0
Vitreoscilla	1	1	0	0	0	0	0	0	0	0	0	0	0	0	0	0	0	0	0	0
Vulcanisaeta	1	14	0	0	0	0	0	0	0	0	0	0	0	0	0	0	0	0	0	0
Waddlia	2	68	0	0	0	0	0	0	0	0	0	0	0	0	0	0	0	0	0	0
Wautersia	1	2	0	0	0	0	0	0	0	0	0	0	0	0	0	0	0	0	0	0
Weeksella	2	65	0	0	0	0	0	0	0	0	0	0	0	0	0	0	0	0	0	0

Genus	Cluster Number	Protein Counts	K15061	K15062	K15063	K15064	K15065	K15066	K15236	K15237	K15238	K15242	K15247	K15253	K15749	K15751	K15754	K15756	K15757	K15981
Weissella	1	14	0	0	0	0	0	0	0	0	0	0	0	0	0	0	0	0	0	0
Wenxinia	1	2	0	0	0	0	0	0	0	0	0	0	0	0	0	0	0	0	0	0
Wigglesworthia	1	29	0	0	0	0	0	0	0	0	0	0	0	0	0	0	0	0	0	0
Wolbachia	1	32	0	0	0	0	0	0	0	0	0	0	0	0	0	0	0	0	0	0
Wolinella	2	51	0	0	0	0	0	0	0	0	0	0	0	0	0	0	0	0	0	0
Xanthobacter	3	176	0	0	0	0	0	0	0	0	0	0	0	0	0	0	0	0	0	0
Xanthobacteraceae	1	4	0	0	0	0	0	0	0	0	0	0	0	0	0	0	0	0	0	0
Xanthomonadaceae	1	39	0	0	0	0	0	0	0	0	0	0	0	0	0	0	0	0	0	0
Xanthomonas	2	162	0	0	0	0	0	0	0	0	0	0	0	0	0	0	0	0	0	0
Xenorhabdus	2	127	0	0	0	0	0	0	0	0	0	0	0	0	0	0	0	0	0	0
Xylanimonas	2	66	0	0	0	0	0	0	0	0	0	0	0	0	0	0	0	0	0	0
Xylella	2	52	0	0	0	0	0	0	0	0	0	0	0	0	0	0	0	0	0	0
Yersinia	2	145	0	0	0	0	0	0	0	0	0	0	0	0	0	0	0	0	0	0
Yokenella	2	61	0	0	0	0	0	0	0	0	0	0	0	0	0	0	0	0	0	0
Zobellia	1	28	0	0	0	0	0	0	0	0	0	0	0	0	0	0	0	0	0	0
Zunongwangia	2	98	0	0	0	0	0	0	0	0	0	0	0	0	0	0	0	0	0	0
Zymomonas	2	67	0	0	0	0	0	0	0	0	0	0	0	0	0	0	0	0	0	0
Zymophilus	1	2	0	0	0	0	0	0	0	0	0	0	0	0	0	0	0	0	0	0

Genus	Cluster Number	Protein Counts	K15982	K16045	K16047	K16049	K16050	K16051	K16173	K16249	K16269	K16270	K16514	K16873	K16874	K16876	K16880	K17067	K17070
Acaricomes	1	2	0	0	0	0	0	0	0	0	0	0	0	0	0	0	0	0	0
Acaryochloris	2	88	0	0	0	0	0	0	0	0	0	0	0	0	0	0	0	0	0
Acetivibrio	1	2	0	0	0	0	0	0	0	0	0	0	0	0	0	0	0	0	0
Acetobacter	2	85	0	0	0	0	0	0	0	0	0	0	0	0	0	0	0	0	0
Acetobacteraceae	1	2	0	0	0	0	0	0	0	0	0	0	0	0	0	0	0	0	0
Acetobacterium	1	8	0	0	0	0	0	0	0	0	0	0	0	0	0	0	0	0	0
Acetohalobium	2	79	0	0	0	0	0	0	0	0	0	0	0	0	0	0	0	0	0
Acholeplasma	2	38	0	0	0	0	0	0	0	0	0	0	0	0	0	0	0	0	0
Achromobacter	2	192	0	0	0	0	0	0	0	0	0	0	0	0	0	0	0	0	0
Acidaminococcus	1	65	0	0	0	0	0	0	0	0	0	0	0	0	0	0	0	0	0
Acidianus	1	9	0	0	0	0	0	0	0	0	0	0	0	0	0	0	0	0	0
Acidilobus	1	8	0	0	0	0	0	0	0	0	0	0	0	0	0	0	0	0	0
Acidimicrobium	2	58	0	0	0	0	0	0	0	0	0	0	0	0	0	0	0	0	0
Acidiphilium	3	144	0	0	0	0	0	0	0	0	0	0	0	0	0	1	1	0	0
Acidithiobacillus	2	70	0	0	0	0	0	0	0	0	0	0	0	0	0	0	0	0	0
Acidobacterium	2	91	0	0	0	0	0	0	0	0	0	0	0	0	0	0	0	0	0
Acidocella	1	2	0	0	0	0	0	0	0	0	0	0	0	0	0	0	0	0	0
Acidothermus	2	68	0	0	0	0	0	0	0	0	0	0	0	0	0	0	0	0	0
Acidovorax	3	217	0	0	0	0	0	0	0	1	0	0	0	0	1	1	1	0	0
Aciduliprofundum	1	11	0	0	0	0	0	0	0	0	0	0	0	0	0	0	0	0	0
Acinetobacter	3	188	0	0	0	0	0	0	0	1	0	0	0	0	0	0	0	0	0
Actinobacillus	2	112	0	0	0	0	0	0	0	0	0	0	0	0	0	0	0	0	0
Actinomadura	1	1	0	0	0	0	0	0	0	0	0	0	0	0	0	0	0	0	0
Actinoplanes	1	52	1	0	1	1	1	1	0	0	0	0	1	0	0	0	0	0	0
Actinopolyspora	1	2	0	0	0	0	0	0	0	0	0	0	0	0	0	0	0	0	0
Actinosynnema	2	122	0	0	0	0	0	0	0	0	0	0	0	0	0	0	0	0	0
Advenella	1	30	0	0	0	0	0	0	0	0	0	0	0	0	0	0	0	0	0
Aequorivita	1	12	0	0	0	0	0	0	0	0	0	0	0	0	0	0	0	0	0
Aerococcus	2	57	0	0	0	0	0	0	0	0	0	0	0	0	0	0	0	0	0
Aeromonas	2	127	0	0	0	0	0	0	0	0	0	0	0	0	0	0	0	0	0
Aeropyrum	1	13	0	0	0	0	0	0	0	0	0	0	0	0	0	0	0	0	0
Afipia	1	54	0	0	0	0	0	0	0	0	0	0	0	0	0	0	1	0	0
Aggregatibacter	1	81	0	0	0	0	0	0	0	0	0	0	0	0	0	0	0	0	0
Agrobacterium	2	242	0	0	1	0	0	0	0	0	0	0	1	1	0	0	0	0	0
Ahrensia	1	2	0	0	0	0	0	0	0	0	0	0	0	0	0	0	0	0	0
Akkermansia	2	47	0	0	0	0	0	0	0	0	0	0	0	0	0	0	0	0	0
Albidiferax	3	144	0	0	0	0	0	0	0	0	0	0	0	0	0	0	0	0	0
Alcaligenes	1	21	0	0	0	0	0	0	0	0	0	0	0	0	0	0	0	0	0
Alcanivorax	2	146	0	0	0	0	0	0	0	0	0	0	0	0	0	1	1	0	0
Alicycliphilus	3	152	0	0	0	0	0	0	0	1	0	0	0	0	0	0	0	0	0
Alicyclobacillus	2	91	0	0	0	0	0	0	0	0	0	0	0	0	1	1	0	0	0
Aliivibrio	2	118	0	0	0	0	0	0	0	0	0	0	0	0	0	0	0	0	0
Alishewanella	1	1	0	0	0	0	0	0	0	0	0	0	0	0	0	0	0	0	0
Alistipes	1	52	0	0	0	0	0	0	0	0	0	0	0	0	0	0	0	0	0
Alkalilimnicola	2	97	0	0	0	0	0	0	0	0	0	0	0	0	0	0	0	0	0
Alkaliphilus	2	62	0	0	0	0	0	0	0	0	0	0	0	0	0	0	0	0	0
Allochromatium	2	84	0	0	0	0	0	0	0	0	0	0	0	0	0	0	0	0	0
Alphaproteobacteria	1	3	0	0	0	0	0	0	0	0	0	0	0	0	0	0	0	0	0
Alteromonadales	1	12	0	0	0	0	0	0	0	0	0	0	0	0	0	0	0	0	0
Alteromonas	1	37	0	0	0	0	0	0	0	0	0	0	0	0	0	0	0	0	0
Aminobacter	1	4	0	0	0	0	0	0	0	0	0	0	0	0	0	0	0	0	0
Aminobacterium	2	59	0	0	0	0	0	0	0	0	0	0	0	0	0	0	0	0	0

Genus	Cluster Number	Protein Counts	K15982	K16045	K16047	K16049	K16050	K16051	K16173	K16249	K16269	K16270	K16514	K16873	K16874	K16876	K16880	K17067	K17070
**Ammonifex**	2	50	0	0	0	0	0	0	0	0	0	0	0	0	0	0	0	0	0
**Amphibacillus**	1	13	0	0	0	0	0	0	0	0	0	0	0	0	0	0	0	0	0
**Amphritea**	1	2	0	0	0	0	0	0	0	0	0	0	0	0	0	0	0	0	0
**Amycolatopsis**	2	201	1	0	1	1	1	1	0	0	0	0	0	0	0	0	0	0	0
**Amycolicicoccus**	1	43	1	0	1	1	1	0	0	0	0	0	0	0	0	0	0	1	0
**Anabaena**	2	92	0	0	0	0	0	0	0	0	0	0	0	0	0	0	0	0	0
**Anaerobaculum**	1	11	0	0	0	0	0	0	0	0	0	0	0	0	0	0	0	0	0
**Anaerococcus**	2	46	0	0	0	0	0	0	0	0	0	0	0	0	0	0	0	0	0
**Anaerolinea**	2	66	0	0	0	0	0	0	0	0	0	0	0	0	0	0	0	0	0
**Anaeromyxobacter**	3	117	0	0	0	0	0	0	0	0	0	0	0	0	0	0	0	0	0
**Anaerostipes**	1	44	0	0	0	0	0	0	0	0	0	0	0	0	0	0	0	0	0
**Anaerotruncus**	1	2	0	0	0	0	0	0	0	0	0	0	0	0	0	0	0	0	0
**Anaplasma**	1	35	0	0	0	0	0	0	0	0	0	0	0	0	0	0	0	0	0
**Ancylobacter**	1	3	0	0	0	0	0	0	0	0	0	0	0	0	0	0	0	0	0
**Aneurinibacillus**	1	1	0	0	0	0	0	0	0	0	0	0	0	0	0	0	0	0	0
**Angomonas**	1	1	0	0	0	0	0	0	0	0	0	0	0	0	0	0	0	0	0
**Anoxybacillus**	2	72	0	0	0	0	0	0	1	0	0	0	0	0	0	0	0	0	0
**Aquifex**	2	45	0	0	0	0	0	0	0	0	0	0	0	0	0	0	0	0	0
**Aquimarina**	1	2	0	0	0	0	0	0	0	0	0	0	0	0	0	0	0	0	0
**Arcanobacterium**	2	45	0	0	0	0	0	0	0	0	0	0	0	0	0	0	0	0	0
**Archaeoglobus**	1	14	0	0	0	0	0	0	0	0	0	0	0	0	0	0	0	0	0
**Arcobacter**	2	101	0	0	0	0	0	0	0	1	0	0	0	0	0	0	0	0	0
**Aromatoleum**	3	133	0	0	0	0	0	0	0	0	0	0	0	0	0	0	0	0	0
**Arsenophonus**	1	4	0	0	0	0	0	0	0	0	0	0	0	0	0	0	0	0	0
**Arthrobacter**	2	206	0	0	0	0	0	0	0	0	0	0	1	0	0	0	0	0	0
**Arthrospira**	2	67	0	0	0	0	0	0	0	0	0	0	0	0	0	0	0	0	0
**Aster**	1	10	0	0	0	0	0	0	0	0	0	0	0	0	0	0	0	0	0
**Asticcacaulis**	2	103	0	0	0	0	0	0	0	0	0	0	1	0	0	0	0	0	0
**Atopobium**	1	28	0	0	0	0	0	0	0	0	0	0	0	0	0	0	0	0	0
**Aurantimonas**	1	2	0	0	0	0	0	0	0	0	0	0	0	0	0	0	0	0	0
**Avibacterium**	1	17	0	0	0	0	0	0	0	0	0	0	0	0	0	0	0	0	0
**Azoarcus**	3	172	0	0	0	0	0	0	0	1	0	0	1	0	0	0	0	0	0
**Azorhizobium**	3	149	0	0	0	0	0	0	0	0	0	0	0	0	0	0	0	0	0
**Azorhizophilus**	1	4	0	0	0	0	0	0	0	0	0	0	0	0	0	0	0	0	0
**Azospira**	1	19	0	0	0	0	0	0	0	0	0	0	0	0	0	0	0	0	0
**Azospirillum**	2	198	0	0	0	0	0	0	0	0	0	0	1	0	1	1	1	0	0
**Azotobacter**	3	183	0	0	1	0	0	0	0	1	0	0	1	0	0	0	0	0	0
**Bacillales**	1	9	0	0	0	0	0	0	0	0	0	0	0	0	0	0	0	0	0
**Bacilli**	1	3	0	0	0	0	0	0	0	0	0	0	0	0	0	0	0	0	0
**Bacillus**	2	237	0	0	0	0	0	0	1	0	0	0	0	0	0	0	0	0	0
**Bacteria**	2	86	0	0	0	0	0	0	0	0	0	0	0	0	0	0	0	0	0
**Bacteriovorax**	2	98	0	0	0	0	0	0	0	0	0	0	0	0	0	0	0	0	0
**Bacteroidales**	2	59	0	0	0	0	0	0	0	0	0	0	0	0	0	0	0	0	0
**Bacteroides**	2	96	0	0	0	0	0	0	0	0	0	0	0	0	0	0	0	0	0
**Bacteroidetes**	1	2	0	0	0	0	0	0	0	0	0	0	0	0	0	0	0	0	0
**Bartonella**	2	50	0	0	0	0	0	0	0	0	0	0	0	0	0	0	0	0	0
**Baumannia**	2	24	0	0	0	0	0	0	0	0	0	0	0	0	0	0	0	0	0
**Bdellovibrio**	2	87	0	0	0	0	0	0	0	0	0	0	0	0	0	0	0	0	0
**Beijerinckia**	3	123	0	0	0	0	0	0	0	0	0	0	0	0	0	0	0	0	0
**Belliella**	1	14	0	0	0	0	0	0	0	0	0	0	0	0	0	0	0	0	0
**Beutenbergia**	2	82	0	0	0	0	0	0	0	0	0	0	0	0	0	0	0	0	0
**Bifidobacterium**	2	61	0	0	0	0	0	0	0	0	0	0	0	0	0	0	0	0	0

Genus	Cluster Number	Protein Counts	K15982	K16045	K16047	K16049	K16050	K16051	K16173	K16249	K16269	K16270	K16514	K16873	K16874	K16876	K16880	K17067	K17070
Bizionia	1	1	0	0	0	0	0	0	0	0	0	0	0	0	0	0	0	0	0
Blastococcus	1	39	0	0	0	0	0	0	0	0	0	0	1	0	0	0	0	0	0
Blattabacterium	1	23	0	0	0	0	0	0	0	0	0	0	0	0	0	0	0	0	0
Blautia	1	22	0	0	0	0	0	0	0	0	0	0	0	0	0	0	0	0	0
Bordetella	3	227	0	0	0	0	1	0	0	0	0	0	0	0	0	0	0	0	0
Borrelia	1	32	0	0	0	0	0	0	0	0	0	0	0	0	0	0	0	0	0
Brachybacterium	1	18	0	0	0	0	0	0	0	0	0	0	0	0	0	0	0	0	0
Brachymonas	1	4	0	0	0	0	0	0	0	0	0	0	0	0	0	0	0	0	0
Brachyspira	2	79	0	0	0	0	0	0	0	0	0	0	0	0	0	0	0	0	0
Bradyrhizobiaceae	1	20	0	0	0	0	0	0	0	0	0	0	0	0	0	0	0	0	0
Bradyrhizobium	3	254	0	0	1	0	0	0	0	0	0	0	0	1	1	1	1	0	0
Brenneria	1	20	0	0	0	0	0	0	0	0	0	0	0	0	0	0	0	0	0
Brevibacillus	2	122	0	0	0	0	0	0	0	0	0	0	0	0	0	0	0	0	0
Brevibacterium	1	4	0	0	0	0	0	0	0	0	0	0	0	0	0	0	0	0	0
Brevundimonas	2	105	0	0	0	0	0	0	0	0	0	0	1	0	0	0	0	0	0
Brucella	3	138	0	0	0	0	0	0	0	0	0	0	0	0	0	0	0	0	0
Buchnera	1	26	0	0	0	0	0	0	0	0	0	0	0	0	0	0	0	0	0
Burkholderia	3	424	1	0	1	1	1	0	0	1	0	0	1	1	1	1	1	0	0
Burkholderiaceae	3	143	0	0	0	0	0	0	0	1	0	0	0	0	0	0	0	0	0
Burkholderiales	1	24	0	0	0	0	0	0	0	0	0	0	0	0	0	0	0	0	0
Buttiauxella	1	2	0	0	0	0	0	0	0	0	0	0	0	0	0	0	0	0	0
Butyrivibrio	2	67	0	0	0	0	0	0	0	0	0	0	0	0	0	0	0	0	0
Caldanaerobacter	2	63	0	0	0	0	0	0	0	0	0	0	0	0	0	0	0	0	0
Caldicellulosiruptor	2	75	0	0	0	0	0	0	0	0	0	0	0	0	0	0	0	0	0
Caldilinea	1	23	0	0	0	0	0	0	0	0	0	0	0	0	0	0	0	0	0
Caldimonas	1	6	0	0	0	0	0	0	0	0	0	0	0	0	0	0	0	0	0
Caldisericum	1	10	0	0	0	0	0	0	0	0	0	0	0	0	0	0	0	0	0
Caldisphaera	1	7	0	0	0	0	0	0	0	0	0	0	0	0	0	0	0	0	0
Calditerrivibrio	2	74	0	0	0	0	0	0	0	0	0	0	0	0	0	0	0	0	0
Caldivirga	1	13	0	0	0	0	0	0	0	0	0	0	0	0	0	0	0	0	0
Calothrix	1	43	0	0	0	0	0	0	0	0	0	0	0	0	0	0	0	0	0
Calyptogena	2	34	0	0	0	0	0	0	0	0	0	0	0	0	0	0	0	0	0
Campylobacter	2	80	0	0	0	0	0	0	0	0	0	0	0	0	0	0	0	0	0
Candidatus	2	290	0	0	0	0	0	0	0	0	0	0	1	0	0	0	0	0	0
Capnocytophaga	1	50	0	0	0	0	0	0	0	0	0	0	0	0	0	0	0	0	0
Carbophilus	1	2	0	0	0	0	0	0	0	0	0	0	0	0	0	0	0	0	0
Carboxydibrachium	1	14	0	0	0	0	0	0	0	0	0	0	0	0	0	0	0	0	0
Carboxydothermus	2	68	0	0	0	0	0	0	1	0	0	0	0	0	1	0	0	0	0
Carica	1	2	0	0	0	0	0	0	0	0	0	0	0	0	0	0	0	0	0
Carnobacterium	2	71	0	0	0	0	0	0	0	0	0	0	0	0	0	0	0	0	0
Catenibacterium	1	2	0	0	0	0	0	0	0	0	0	0	0	0	0	0	0	0	0
Catenovulum	1	2	0	0	0	0	0	0	0	0	0	0	0	0	0	0	0	0	0
Catenulispora	3	159	0	0	0	0	0	0	0	0	0	0	0	0	0	0	0	0	0
Caulobacter	3	168	0	0	1	0	0	0	0	0	0	0	0	0	0	0	0	0	0
Cedecea	1	45	0	0	0	0	0	0	0	0	0	0	0	0	0	0	0	0	0
Cellulomonas	2	96	0	0	0	0	0	0	0	0	0	0	0	0	0	0	0	0	0
Cellulophaga	2	105	0	0	0	0	0	0	0	0	0	0	0	0	0	0	0	0	0
Cellulosilyticum	1	14	0	0	0	0	0	0	0	0	0	0	0	0	0	0	0	0	0
Cellvibrio	2	85	0	0	0	0	0	0	0	0	0	0	0	0	0	0	0	0	0
Cenarchaeum	1	7	0	0	0	0	0	0	0	0	0	0	0	0	0	0	0	0	0
Chamaesiphon	1	13	0	0	0	0	0	0	0	0	0	0	0	0	0	0	0	0	0
Chelativorans	3	136	0	0	0	0	0	0	0	0	0	0	0	0	0	0	0	0	0

Genus	Cluster Number	Protein Counts	K15982	K16045	K16047	K16049	K16050	K16051	K16173	K16249	K16269	K16270	K16514	K16873	K16874	K16876	K16880	K17067	K17070
Chelatococcus	1	2	0	0	0	0	0	0	0	0	0	0	0	0	0	0	0	0	0
Chitinophaga	2	98	0	0	0	0	0	0	0	0	0	0	0	0	0	0	0	0	0
Chlamydia	1	36	0	0	0	0	0	0	0	0	0	0	0	0	0	0	0	0	0
Chlamydomonas	1	3	0	0	0	0	0	0	0	0	0	0	0	0	0	0	0	0	0
Chlamydophila	1	31	0	0	0	0	0	0	0	0	0	0	0	0	0	0	0	0	0
Chlorobaculum	2	61	0	0	0	0	0	0	0	0	0	0	0	0	0	0	0	0	0
Chlorobium	2	89	0	0	0	0	0	0	0	0	0	0	0	0	0	0	0	0	0
Chlorobium/Pelodictyon	1	2	0	0	0	0	0	0	0	0	0	0	0	0	0	0	0	0	0
Chloroflexus	2	105	0	0	0	0	0	0	0	0	0	0	0	0	0	0	0	0	0
Chlorogloeopsis	1	9	0	0	0	0	0	0	0	0	0	0	0	0	0	0	0	0	0
Chloroherpeton	2	59	0	0	0	0	0	0	0	0	0	0	0	0	0	0	0	0	0
Chromobacterium	2	117	0	0	0	0	0	0	0	0	0	0	0	0	0	0	0	0	0
Chromohalobacter	2	145	0	0	0	0	0	0	0	0	0	0	1	0	0	1	0	0	0
Chroococcidiopsis	1	24	0	0	0	0	0	0	0	0	0	0	0	0	0	0	0	0	0
Chryseobacterium	2	104	0	0	0	0	0	0	0	0	0	0	1	0	0	0	0	0	0
Citreicella	1	13	0	0	0	0	0	0	0	0	0	0	0	0	0	0	0	0	0
Citrobacter	2	190	0	0	0	0	0	0	0	0	0	0	1	0	0	0	0	0	0
Clavibacter	2	83	0	0	0	0	0	0	0	0	0	0	0	0	0	0	0	0	0
Clonorchis	1	1	0	0	0	0	0	0	0	0	0	0	0	0	0	0	0	0	0
Clostridiales	2	77	0	0	0	0	0	0	0	0	0	0	0	0	0	0	0	0	0
Clostridium	2	191	0	0	0	0	0	0	0	0	0	0	0	0	0	0	0	0	0
Cohnella	1	2	0	0	0	0	0	0	0	0	0	0	0	0	0	0	0	0	0
Coleofasciculus	1	4	0	0	0	0	0	0	0	0	0	0	0	0	0	0	0	0	0
Collimonas	1	35	0	0	0	0	0	0	0	0	0	0	0	0	0	0	0	0	0
Colwellia	2	130	0	0	0	0	0	0	0	0	0	0	0	0	0	0	0	0	0
Comamonas	2	196	1	0	1	1	1	1	0	1	0	0	0	0	0	0	0	0	0
Conexibacter	3	117	0	0	0	0	0	0	0	0	0	0	0	0	0	0	0	0	0
Coprobacillus	1	2	0	0	0	0	0	0	0	0	0	0	0	0	0	0	0	0	0
Coprococcus	2	76	0	0	0	0	0	0	0	0	0	0	0	0	0	0	0	0	0
Coprothermobacter	2	45	0	0	0	0	0	0	0	0	0	0	0	0	0	0	0	0	0
Coraliomargarita	2	68	0	0	0	0	0	0	0	0	0	0	0	0	0	0	0	0	0
Corallococcus	1	29	0	0	0	0	0	0	0	0	0	0	0	0	0	0	0	0	0
Coriobacterium	2	36	0	0	0	0	0	0	0	0	0	0	0	0	0	0	0	0	0
Corynebacterineae	1	5	0	0	0	0	0	0	0	0	0	0	0	0	0	0	0	0	0
Corynebacterium	2	175	0	0	0	0	0	0	0	0	0	0	0	0	0	0	0	0	0
Coxiella	2	54	0	0	0	0	0	0	0	0	0	0	0	0	0	0	0	0	0
Crinalium	1	21	0	0	0	0	0	0	0	0	0	0	0	0	0	0	0	0	0
Croceibacter	2	80	0	0	0	0	0	0	0	0	0	0	0	0	0	0	0	0	0
Crocosphaera	1	6	0	0	0	0	0	0	0	0	0	0	0	0	0	0	0	0	0
Cronobacter	2	120	0	0	0	0	0	0	0	0	0	0	0	0	0	0	0	0	0
Cryptobacterium	2	39	0	0	0	0	0	0	0	0	0	0	0	0	0	0	0	0	0
Cucumis	1	9	0	0	0	0	0	0	0	0	0	0	0	0	0	0	0	0	0
Cupriavidus	3	317	1	0	1	1	1	1	0	1	0	0	0	0	0	1	1	0	0
Curtobacterium	1	5	0	0	0	0	0	0	0	0	0	0	0	0	0	0	0	0	0
Curvibacter	1	27	0	0	0	0	0	0	0	0	0	0	0	0	0	0	0	0	0
Cyanobacterium	1	9	0	0	0	0	0	0	0	0	0	0	0	0	0	0	0	0	0
Cyanobium	1	10	0	0	0	0	0	0	0	0	0	0	0	0	0	0	0	0	0
Cyanothece	1	34	0	0	0	0	0	0	0	0	0	0	0	0	0	0	0	0	0
Cyclobacterium	1	19	0	0	0	0	0	0	0	0	0	0	0	0	0	0	0	0	0
Cycloclasticus	1	21	0	0	0	0	0	0	0	0	0	0	0	0	0	0	0	0	0
Cylindrospermopsis	1	4	0	0	0	0	0	0	0	0	0	0	0	0	0	0	0	0	0
Cylindrospermum	1	30	0	0	0	0	0	0	0	0	0	0	0	0	0	0	0	0	0

Genus	Cluster Number	Protein Counts	K15982	K16045	K16047	K16049	K16050	K16051	K16173	K16249	K16269	K16270	K16514	K16873	K16874	K16876	K16880	K17067	K17070
Cytophaga	2	69	0	0	0	0	0	0	0	0	0	0	0	0	0	0	0	0	0
Dactylococcopsis	1	8	0	0	0	0	0	0	0	0	0	0	0	0	0	0	0	0	0
Dechloromonas	1	40	0	0	1	0	0	0	0	1	0	0	0	0	0	0	0	0	0
Dechlorosoma	1	19	0	0	0	0	0	0	0	0	0	0	0	0	0	0	0	0	0
Deferribacter	2	73	0	0	0	0	0	0	0	0	0	0	0	0	0	0	0	0	0
Dehalobacter	1	9	0	0	0	0	0	0	0	0	0	0	0	0	0	0	0	0	0
Dehalococcoides	2	41	0	0	0	0	0	0	0	0	0	0	0	0	0	0	0	0	0
Dehalogenimonas	1	37	0	0	0	0	0	0	0	0	0	0	0	0	0	0	0	0	0
Deinococcus	2	140	0	0	0	0	0	0	0	0	0	0	0	0	0	0	0	0	0
Delftia	3	177	0	0	0	0	0	0	0	0	0	0	1	0	0	0	0	0	0
Denitrovibrio	2	67	0	0	0	0	0	0	0	0	0	0	0	0	0	0	0	0	0
Desulfarculus	2	92	0	0	0	0	0	0	1	0	0	0	0	0	0	0	0	0	0
Desulfatibacillum	3	84	0	0	0	0	0	0	1	0	0	0	0	0	0	0	0	0	0
Desulfitobacterium	2	102	0	0	0	0	0	0	0	0	0	0	0	0	0	0	0	0	0
Desulfobacca	2	65	0	0	0	0	0	0	0	0	0	0	0	0	0	0	0	0	0
Desulfobacterium	2	113	0	0	0	0	0	0	1	0	0	0	0	0	0	0	0	0	0
Desulfobacula	1	21	0	0	0	0	0	0	0	0	0	0	0	0	0	0	0	0	0
Desulfobulbus	2	88	0	0	0	0	0	0	0	0	0	0	0	0	0	0	0	0	0
Desulfocapsa	1	9	0	0	0	0	0	0	0	0	0	0	0	0	0	0	0	0	0
Desulfococcus	2	80	0	0	0	0	0	0	0	0	0	0	0	0	0	0	0	0	0
Desulfohalobium	2	52	0	0	0	0	0	0	0	0	0	0	0	0	0	0	0	0	0
Desulfomicrobium	2	65	0	0	0	0	0	0	0	0	0	0	0	0	0	0	0	0	0
Desulfomonile	1	25	0	0	0	0	0	0	1	0	0	0	0	0	0	0	0	0	0
Desulforhabdus	1	2	0	0	0	0	0	0	0	0	0	0	0	0	0	0	0	0	0
Desulfosporosinus	1	30	0	0	0	0	0	0	1	0	0	0	0	0	0	0	0	0	0
Desulfotalea	2	66	0	0	0	0	0	0	0	0	0	0	0	0	0	0	0	0	0
Desulfotomaculum	2	98	0	0	0	0	0	0	1	0	0	0	0	0	1	0	0	1	0
Desulfovibrio	2	144	0	0	0	0	0	0	0	0	0	0	0	0	0	0	0	0	0
Desulfurispirillum	2	78	0	0	0	0	0	0	0	0	0	0	0	0	0	0	0	0	0
Desulfurivibrio	2	71	0	0	0	0	0	0	0	0	0	0	0	0	0	0	0	0	0
Desulfurobacterium	2	53	0	0	0	0	0	0	0	0	0	0	0	0	0	0	0	0	0
Desulfurococcus	1	11	0	0	0	0	0	0	0	0	0	0	0	0	0	0	0	0	0
Diaphorobacter	1	16	0	0	0	0	0	0	0	0	0	0	0	0	0	0	0	0	0
Dichelobacter	2	45	0	0	0	0	0	0	0	0	0	0	0	0	0	0	0	0	0
Dickeya	2	155	0	0	0	0	0	0	0	0	0	0	0	0	0	0	0	0	0
Dictyoglomus	2	62	0	0	0	0	0	0	0	0	0	0	0	0	0	0	0	0	0
Dinoroseobacter	3	148	0	0	0	0	0	0	0	0	0	0	0	0	1	1	1	0	0
Dokdonia	1	3	0	0	0	0	0	0	0	0	0	0	0	0	0	0	0	0	0
Dorea	1	7	0	0	0	0	0	0	0	0	0	0	0	0	0	0	0	0	0
Dyadobacter	2	83	0	0	0	0	0	0	0	0	0	0	0	0	0	0	0	0	0
Echinicola	1	20	0	0	0	0	0	0	0	0	0	0	0	0	0	0	0	0	0
Edwardsiella	2	126	0	0	0	0	0	0	0	0	0	0	0	0	0	0	0	0	0
Eggerthella	1	51	0	0	0	0	0	0	0	0	0	0	0	0	0	0	0	0	0
Ehrlichia	2	37	0	0	0	0	0	0	0	0	0	0	0	0	0	0	0	0	0
Eikenella	1	4	0	0	0	0	0	0	0	0	0	0	0	0	0	0	0	0	0
Elusimicrobium	2	44	0	0	0	0	0	0	0	0	0	0	0	0	0	0	0	0	0
Emticicia	1	21	0	0	0	0	0	0	0	0	0	0	1	0	0	0	0	0	0
Enhydrobacter	1	1	0	0	0	0	0	0	0	0	0	0	0	0	0	0	0	0	0
Ensifer	1	2	0	0	0	0	0	0	0	0	0	0	0	0	0	0	0	0	0
Enterobacter	2	202	0	0	0	0	0	0	0	0	0	0	0	0	0	0	0	0	0
Enterobacteriaceae	2	211	0	0	0	0	0	0	0	0	0	0	1	0	0	0	0	0	0
Enterococcus	1	187	0	0	0	0	0	0	0	0	0	0	1	0	0	0	0	0	0

Genus	Cluster Number	Protein Counts	K15982	K16045	K16047	K16049	K16050	K16051	K16173	K16249	K16269	K16270	K16514	K16873	K16874	K16876	K16880	K17067	K17070
Enterovibrio	1	15	0	0	0	0	0	0	0	0	0	0	0	0	0	0	0	0	0
Erwinia	2	151	0	0	0	0	0	0	0	0	0	0	1	0	0	0	0	0	0
Erysipelothrix	1	9	0	0	0	0	0	0	0	0	0	0	0	0	0	0	0	0	0
Erysipelotrichaceae	1	4	0	0	0	0	0	0	0	0	0	0	0	0	0	0	0	0	0
Erythrobacter	2	80	0	0	0	0	0	0	0	0	0	0	0	0	0	0	0	0	0
Escherichia	2	242	0	0	0	0	0	0	0	0	0	0	1	0	0	0	0	0	0
Ethanoligenens	2	69	0	0	0	0	0	0	0	0	0	0	0	0	0	0	0	0	0
Eubacteriaceae	1	2	0	0	0	0	0	0	0	0	0	0	0	0	0	0	0	0	0
Eubacterium	2	103	0	0	0	0	0	0	0	0	0	0	0	0	0	0	0	0	0
Eudoraea	1	4	0	0	0	0	0	0	0	0	0	0	0	0	0	0	0	0	0
Euryarchaeota	1	1	0	0	0	0	0	0	0	0	0	0	0	0	0	0	0	0	0
Exiguobacterium	2	93	0	0	0	0	0	0	0	0	0	0	0	0	0	0	0	0	0
Faecalibacterium	1	12	0	0	0	0	0	0	0	0	0	0	0	0	0	0	0	0	0
Ferrimonas	2	115	0	0	0	0	0	0	0	0	0	0	0	0	0	0	0	0	0
Ferroglobus	1	14	0	0	0	0	0	0	0	0	0	0	0	0	0	0	0	0	0
Ferroplasma	1	11	0	0	0	0	0	0	0	0	0	0	0	0	0	0	0	0	0
Fervidicoccus	1	7	0	0	0	0	0	0	0	0	0	0	0	0	0	0	0	0	0
Fervidobacterium	2	54	0	0	0	0	0	0	0	0	0	0	0	0	0	0	0	0	0
Fibrella	1	17	0	0	0	0	0	0	0	0	0	0	0	0	0	0	0	0	0
Fibrobacter	2	52	0	0	0	0	0	0	0	0	0	0	0	0	0	0	0	0	0
Filifactor	1	9	0	0	0	0	0	0	0	0	0	0	0	0	0	0	0	0	0
Finegoldia	2	55	0	0	0	0	0	0	0	0	0	0	0	0	0	0	0	0	0
Firmicutes	1	15	0	0	0	0	0	0	0	0	0	0	0	0	0	0	0	0	0
Fischerella	1	15	0	0	0	0	0	0	0	0	0	0	0	0	0	0	0	0	0
Flavobacteria	1	7	0	0	0	0	0	0	0	0	0	0	0	0	0	0	0	0	0
Flavobacteriaceae	2	69	0	0	0	0	0	0	0	0	0	0	0	0	0	0	0	0	0
Flavobacteriales	1	3	0	0	0	0	0	0	0	0	0	0	0	0	0	0	0	0	0
Flavobacterium	2	103	0	0	0	0	0	0	0	0	0	0	0	0	0	0	0	0	0
Flexibacter	1	17	0	0	0	0	0	0	0	0	0	0	0	0	0	0	0	0	0
Flexistipes	1	11	0	0	0	0	0	0	0	0	0	0	0	0	0	0	0	0	0
Fluoribacter	1	6	0	0	0	0	0	0	0	0	0	0	0	0	0	0	0	0	0
Fluviicola	2	69	0	0	0	0	0	0	0	0	0	0	0	0	0	0	0	0	0
Formosa	1	2	0	0	0	0	0	0	0	0	0	0	0	0	0	0	0	0	0
Francisella	2	88	0	0	0	0	0	0	0	0	0	0	0	0	0	0	0	0	0
Frankia	3	202	0	0	0	0	0	1	0	0	0	0	0	0	0	0	0	0	0
Frateuria	1	25	0	0	0	0	0	0	0	0	0	0	0	0	0	0	0	0	0
Fructobacillus	1	2	0	0	0	0	0	0	0	0	0	0	0	0	0	0	0	0	0
Fulvimarina	1	2	0	0	0	0	0	0	0	0	0	0	0	0	0	0	0	0	0
Fusobacterium	1	48	0	0	0	0	0	0	0	0	0	0	0	0	0	0	0	0	0
Gallibacterium	2	87	0	0	0	0	0	0	0	0	0	0	0	0	0	0	0	0	0
Gallionella	2	67	0	0	0	0	0	0	0	0	0	0	0	0	0	0	0	0	0
Gammaproteobacteria	1	9	0	0	0	0	0	0	0	0	0	0	0	0	0	0	0	0	0
Gardnerella	1	31	0	0	0	0	0	0	0	0	0	0	0	0	0	0	0	0	0
Geitlerinema	1	9	0	0	0	0	0	0	0	0	0	0	0	0	0	0	0	0	0
Gemmatimonas	2	93	0	0	0	0	0	0	0	0	0	0	0	0	0	0	0	0	0
Geobacillus	2	168	0	0	0	0	0	0	1	0	0	0	0	0	1	1	1	0	0
Geobacter	2	150	0	0	0	0	0	0	1	0	0	0	0	0	0	0	0	1	0
Geodermatophilus	2	136	0	0	0	0	0	0	0	0	0	0	0	0	0	0	0	0	0
Geopsychrobacter	1	2	0	0	0	0	0	0	0	0	0	0	0	0	0	0	0	1	0
Gillisia	1	11	0	0	0	0	0	0	0	0	0	0	0	0	0	0	0	0	0
Gilvimarinus	1	4	0	0	0	0	0	0	0	0	0	0	0	0	0	0	0	0	0
Glaciecola	2	117	0	0	0	0	0	0	0	0	0	0	0	0	0	0	0	0	0

Genus	Cluster Number	Protein Counts	K15982	K16045	K16047	K16049	K16050	K16051	K16173	K16249	K16269	K16270	K16514	K16873	K16874	K16876	K16880	K17067	K17070
Gloeobacter	2	67	0	0	0	0	0	0	0	0	0	0	0	0	0	0	0	0	0
Gloeocapsa	1	20	0	0	0	0	0	0	0	0	0	0	0	0	0	0	0	0	0
Gloeothece	1	1	0	0	0	0	0	0	0	0	0	0	0	0	0	0	0	0	0
Gluconacetobacter	2	96	0	0	0	0	0	0	0	0	0	0	0	0	0	0	0	0	0
Gluconobacter	2	76	0	0	0	0	0	0	0	0	0	0	0	0	0	0	0	0	0
Gordonia	1	158	1	0	1	1	0	0	0	0	0	0	0	0	0	0	0	1	0
Gordonibacter	1	32	0	0	0	0	0	0	0	0	0	0	0	0	0	0	0	0	0
Gramella	2	104	0	0	0	0	0	0	0	0	0	0	0	0	0	0	0	0	0
Granulibacter	2	90	0	0	0	0	0	0	0	0	0	0	0	0	0	0	0	0	0
Granulicella	2	98	0	0	0	0	0	0	0	0	0	0	0	0	0	0	0	0	0
Grimontia	1	9	0	0	0	0	0	0	0	0	0	0	0	0	0	0	0	0	0
Haemophilus	2	117	0	0	0	0	0	0	0	0	0	0	0	0	0	0	0	0	0
Hafnia	1	29	0	0	0	0	0	0	0	0	0	0	0	0	0	0	0	0	0
Hahella	3	130	0	0	0	0	0	0	0	0	0	0	0	0	0	0	0	0	0
Halalkalicoccus	1	18	0	0	0	0	0	0	0	0	0	0	0	0	0	0	0	0	0
Halanaerobium	2	89	0	0	0	0	0	0	0	0	0	0	0	0	0	0	0	0	0
Haliangium	2	108	0	0	0	0	0	0	0	0	0	0	0	0	0	0	0	0	0
Haliscomenobacter	2	107	0	0	0	0	0	0	0	0	0	0	0	0	0	0	0	0	0
Haloarcula	1	22	0	0	0	0	0	0	0	0	0	0	0	0	0	0	0	0	0
Halobacillus	1	19	0	0	0	0	0	0	0	0	0	0	0	0	0	0	0	0	0
Halobacteriaceae	1	1	0	0	0	0	0	0	0	0	0	0	0	0	0	0	0	0	0
Halobacterium	1	10	0	0	0	0	0	0	0	0	0	0	0	0	0	0	0	0	0
Halobacteroides	1	14	0	0	0	0	0	0	0	0	0	0	0	0	0	0	0	0	0
Haloferax	1	20	0	0	0	0	0	0	0	0	0	0	0	0	0	0	0	0	0
Halogeometricum	1	12	0	0	0	0	0	0	0	0	0	0	0	0	0	0	0	0	0
Halomicrobium	1	10	0	0	0	0	0	0	0	0	0	0	0	0	0	0	0	0	0
Halomonas	1	149	0	0	0	0	0	0	0	0	0	0	0	0	0	0	0	0	0
Halopiger	1	16	0	0	0	0	0	0	0	0	0	0	0	0	0	0	0	0	0
Haloquadratum	1	19	0	0	0	0	0	0	0	0	0	0	0	0	0	0	0	0	0
Halorhabdus	1	10	0	0	0	0	0	0	0	0	0	0	0	0	0	0	0	0	0
Halorhodospira	2	71	0	0	0	0	0	0	0	0	0	0	0	0	0	0	0	0	0
Halorubrum	1	16	0	0	0	0	0	0	0	0	0	0	0	0	0	0	0	0	0
Haloterrigena	1	19	0	0	0	0	0	0	0	0	0	0	0	0	0	0	0	0	0
Halothece	1	9	0	0	0	0	0	0	0	0	0	0	0	0	0	0	0	0	0
Halothermothrix	2	50	0	0	0	0	0	0	0	0	0	0	0	0	0	0	0	0	0
Halothiobacillus	2	65	0	0	0	0	0	0	0	0	0	0	0	0	0	0	0	0	0
Halovivax	1	12	0	0	0	0	0	0	0	0	0	0	0	0	0	0	0	0	0
Helicobacter	2	79	0	0	0	0	0	0	0	0	0	0	0	0	0	0	0	0	0
Heliobacillus	1	4	0	0	0	0	0	0	0	0	0	0	0	0	0	0	0	0	0
Heliobacterium	2	51	0	0	0	0	0	0	0	0	0	0	0	0	0	0	0	0	0
Herbaspirillum	2	172	0	0	0	0	0	0	0	0	0	0	0	0	0	0	0	0	0
Herminiimonas	3	89	0	0	0	0	0	0	0	0	0	0	0	0	0	0	0	0	0
Herpetosiphon	3	90	0	0	0	0	0	0	0	0	0	0	0	0	0	0	0	0	0
Hippea	2	44	0	0	0	0	0	0	0	0	0	0	0	0	0	0	0	0	0
Hirschia	2	102	0	0	0	0	0	0	0	0	0	0	0	0	0	0	0	0	0
Histophilus	2	72	0	0	0	0	0	0	0	0	0	0	0	0	0	0	0	0	0
Hoeflea	1	16	0	0	0	0	0	0	0	0	0	0	0	0	0	0	0	0	0
Hydrogenivirga	1	2	0	0	0	0	0	0	0	0	0	0	0	0	0	0	0	0	0
Hydrogenobacter	2	55	0	0	0	0	0	0	0	0	0	0	0	0	0	0	0	0	0
Hydrogenobaculum	2	44	0	0	0	0	0	0	0	0	0	0	0	0	0	0	0	0	0
Hydrogenophaga	1	9	0	0	0	0	0	0	0	0	0	0	0	0	0	0	0	0	0
Hylemonella	1	4	0	0	0	0	0	0	0	0	0	0	0	0	0	0	0	0	0

Genus	Cluster Number	Protein Counts	K15982	K16045	K16047	K16049	K16050	K16051	K16173	K16249	K16269	K16270	K16514	K16873	K16874	K16876	K16880	K17067	K17070
**Hyperthermus**	1	6	0	0	0	0	0	0	0	0	0	0	0	0	0	0	0	0	0
**Hyphomicrobium**	2	93	0	0	0	0	0	0	0	0	0	0	0	0	0	0	0	0	1
**Hyphomonas**	3	105	0	0	0	0	0	0	0	0	0	0	0	0	0	0	0	0	0
**Ideonella**	1	18	0	0	0	0	0	0	0	0	0	0	0	0	0	0	0	0	0
**Idiomarina**	2	86	0	0	0	0	0	0	0	0	0	0	0	0	0	0	0	0	0
**Ignavibacterium**	1	14	0	0	0	0	0	0	0	0	0	0	0	0	0	0	0	0	0
**Ignicoccus**	1	5	0	0	0	0	0	0	0	0	0	0	0	0	0	0	0	0	0
**Ignisphaera**	1	8	0	0	0	0	0	0	0	0	0	0	0	0	0	0	0	0	0
**Ilyobacter**	2	85	0	0	0	0	0	0	0	0	0	0	0	0	0	0	0	0	0
**Intrasporangium**	2	114	0	0	0	0	0	0	0	0	0	0	0	0	0	0	0	0	0
**Isoptericola**	1	18	0	0	0	0	0	0	0	0	0	0	0	0	0	0	0	0	0
**Isosphaera**	2	75	0	0	0	0	0	0	0	0	0	0	0	0	0	0	0	0	0
**Jannaschia**	3	151	0	0	0	0	0	0	0	0	0	0	0	0	0	0	0	0	0
**Janthinobacterium**	2	99	0	0	0	0	0	0	0	0	0	0	0	0	0	0	0	0	0
**Jonesia**	2	56	0	0	0	0	0	0	0	0	0	0	0	0	0	0	0	0	0
**Joostella**	1	3	0	0	0	0	0	0	0	0	0	0	0	0	0	0	0	0	0
**Kaistia**	1	2	0	0	0	0	0	0	0	0	0	0	0	0	0	0	0	0	0
**Kangiella**	2	84	0	0	0	0	0	0	0	0	0	0	0	0	0	0	0	0	0
**Ketogulonicigenium**	2	102	0	0	0	0	0	0	0	0	0	0	0	0	0	0	0	0	0
**Kineococcus**	1	24	0	0	0	0	0	0	0	0	0	0	0	0	0	0	0	0	0
**Kinetoplastibacterium**	1	2	0	0	0	0	0	0	0	0	0	0	0	0	0	0	0	0	0
**Kingella**	1	4	0	0	0	0	0	0	0	0	0	0	0	0	0	0	0	0	0
**Kitasatospora**	1	36	0	0	0	0	0	0	0	0	0	0	0	0	0	0	0	0	0
**Klebsiella**	3	222	0	0	0	0	0	0	0	0	0	0	1	0	0	0	0	0	0
**Kluyvera**	1	1	0	0	0	0	0	0	0	0	0	0	0	0	0	0	0	0	0
**Kocuria**	2	87	0	0	0	0	0	0	0	0	0	0	0	0	0	0	0	0	0
**Kordia**	1	5	0	0	0	0	0	0	0	0	0	0	0	0	0	0	0	0	0
**Kosmotoga**	2	61	0	0	0	0	0	0	0	0	0	0	0	0	0	0	0	0	0
**Kribbella**	2	99	0	0	0	0	0	0	0	0	0	0	0	0	0	0	0	0	0
**Krokinobacter**	2	90	0	0	0	0	0	0	0	0	0	0	0	0	0	0	0	0	0
**Kyrpidia**	2	113	0	0	0	0	0	0	0	0	0	0	0	0	0	0	0	0	0
**Kytococcus**	2	70	0	0	0	0	0	0	0	0	0	0	0	0	0	0	0	0	0
**Labrenzia**	1	4	0	0	0	0	0	0	0	0	0	0	0	0	0	0	0	0	0
**Lachnoanaerobaculum**	1	1	0	0	0	0	0	0	0	0	0	0	0	0	0	0	0	0	0
**Lachnospiraceae**	1	51	0	0	0	0	0	0	0	0	0	0	0	0	0	0	0	0	0
**Lacinutrix**	1	20	0	0	0	0	0	0	0	0	0	0	0	0	0	0	0	0	0
**Lactobacillus**	2	119	0	0	0	0	0	0	0	0	0	0	0	0	0	0	0	0	0
**Lactococcus**	2	82	0	0	0	0	0	0	0	0	0	0	0	0	0	0	0	0	0
**Lamprocystis**	1	2	0	0	0	0	0	0	0	0	0	0	0	0	0	0	0	0	0
**Laribacter**	2	80	0	0	0	0	0	0	0	0	0	0	0	0	0	0	0	0	0
**Lawsonia**	2	33	0	0	0	0	0	0	0	0	0	0	0	0	0	0	0	0	0
**Leadbetterella**	2	79	0	0	0	0	0	0	0	0	0	0	0	0	0	0	0	0	0
**Leeuwenhoekiella**	1	2	0	0	0	0	0	0	0	0	0	0	0	0	0	0	0	0	0
**Legionella**	2	117	0	0	0	0	0	0	0	0	0	0	0	0	0	0	0	0	0
**Leifsonia**	2	48	0	0	0	0	0	0	0	0	0	0	0	0	0	0	0	0	0
**Leptolyngbya**	1	10	0	0	0	0	0	0	0	0	0	0	0	0	0	0	0	0	0
**Leptospira**	2	76	0	0	0	0	0	0	0	0	0	0	0	0	0	0	0	0	0
**Leptospirillum**	1	10	0	0	0	0	0	0	0	0	0	0	0	0	0	0	0	0	0
**Leptothrix**	3	186	0	0	0	0	0	0	0	1	0	0	0	0	0	0	0	0	0
**Leptotrichia**	2	43	0	0	0	0	0	0	0	0	0	0	0	0	0	0	0	0	0
**Leucobacter**	1	2	0	0	0	0	0	0	0	0	0	0	0	0	0	0	0	0	0
**Leuconostoc**	2	64	0	0	0	0	0	0	0	0	0	0	0	0	0	0	0	0	0

Genus	Cluster Number	Protein Counts	K15982	K16045	K16047	K16049	K16050	K16051	K16173	K16249	K16269	K16270	K16514	K16873	K16874	K16876	K16880	K17067	K17070
Liberibacter	1	4	0	0	0	0	0	0	0	0	0	0	0	0	0	0	0	0	0
Limnohabitans	1	11	0	0	0	0	0	0	0	0	0	0	0	0	0	0	0	0	0
Listeria	2	77	0	0	0	0	0	0	0	0	0	0	0	0	0	0	0	0	0
Listeriaceae	1	6	0	0	0	0	0	0	0	0	0	0	0	0	0	0	0	0	0
Listonella	1	38	0	0	0	0	0	0	0	0	0	0	0	0	0	0	0	0	0
Loktanella	1	3	0	0	0	0	0	0	0	0	0	0	0	0	0	0	0	0	0
Lysinibacillus	2	97	0	0	0	0	0	0	0	0	0	0	0	0	0	0	0	0	0
Macrococcus	2	60	0	0	0	0	0	0	0	0	0	0	0	0	0	0	0	0	0
Magnetococcus	2	64	0	0	0	0	0	0	0	0	0	0	0	0	0	0	0	0	0
Magnetospirillum	2	113	0	0	0	0	0	0	0	0	0	0	1	0	0	0	0	0	0
Mahella	2	58	0	0	0	0	0	0	0	0	0	0	0	0	0	0	0	0	0
Mannheimia	2	85	0	0	0	0	0	0	0	0	0	0	0	0	0	0	0	0	0
Maribacter	3	87	0	0	0	0	0	0	0	0	0	0	0	0	0	0	0	0	0
Maricaulis	3	87	0	0	0	0	0	0	0	0	0	0	0	0	0	0	0	0	0
Marichromatium	1	2	0	0	0	0	0	0	0	0	0	0	0	0	0	0	0	0	0
Marinithermus	2	77	0	0	0	0	0	0	0	0	0	0	0	0	0	0	0	0	0
Marinitoga	1	15	0	0	0	0	0	0	0	0	0	0	0	0	0	0	0	0	0
Marinobacter	2	163	0	0	0	0	0	0	0	1	0	0	0	0	0	0	0	0	0
Marinomonas	3	192	0	0	0	0	0	0	0	0	0	0	1	0	0	0	0	0	0
Maritimibacter	1	7	0	0	0	0	0	0	0	0	0	0	0	0	0	0	0	0	0
Marivirga	2	96	0	0	0	0	0	0	0	0	0	0	0	0	0	0	0	0	0
Martelella	1	3	0	0	0	0	0	0	0	0	0	0	0	0	0	0	0	0	0
Marvinbryantia	1	1	0	0	0	0	0	0	0	0	0	0	0	0	0	0	0	0	0
Massilia	1	4	0	0	0	0	0	0	0	0	0	0	0	0	0	0	0	0	0
Mastigocladopsis	1	16	0	0	0	0	0	0	0	0	0	0	0	0	0	0	0	0	0
Megamonas	1	47	0	0	0	0	0	0	0	0	0	0	0	0	0	0	0	0	0
Megasphaera	1	14	0	0	0	0	0	0	0	0	0	0	0	0	0	0	0	0	0
Meiothermus	2	110	0	0	0	0	0	0	0	0	0	0	0	0	0	0	0	0	0
Melissococcus	2	46	0	0	0	0	0	0	0	0	0	0	0	0	0	0	0	0	0
Mesoflavibacter	1	6	0	0	0	0	0	0	0	0	0	0	0	0	0	0	0	0	0
Mesoplasma	1	23	0	0	0	0	0	0	0	0	0	0	0	0	0	0	0	0	0
Mesorhizobium	2	201	0	0	0	0	0	0	0	0	0	0	0	0	0	0	1	0	0
Mesotoga	1	14	0	0	0	0	0	0	0	0	0	0	0	0	0	0	0	0	0
Metallosphaera	1	18	0	0	0	0	0	0	0	0	0	0	0	0	0	0	0	0	0
Methanobacterium	1	7	0	0	0	0	0	0	0	0	0	0	0	0	0	0	0	0	0
Methanobrevibacter	1	9	0	0	0	0	0	0	0	0	0	0	0	0	0	0	0	0	0
Methanocaldococcus	1	6	0	0	0	0	0	0	0	0	0	0	0	0	0	0	0	0	0
Methanocella	1	15	0	0	0	0	0	0	0	0	0	0	0	0	0	0	0	0	0
Methanococcaceae	1	1	0	0	0	0	0	0	0	0	0	0	0	0	0	0	0	0	0
Methanococcoides	1	5	0	0	0	0	0	0	0	0	0	0	0	0	0	0	0	0	0
Methanococcus	1	9	0	0	0	0	0	0	0	0	0	0	0	0	0	0	0	0	0
Methanocorpusculum	1	7	0	0	0	0	0	0	0	0	0	0	0	0	0	0	0	0	0
Methanoculleus	1	8	0	0	0	0	0	0	0	0	0	0	0	0	0	0	0	0	0
Methanohalobium	1	5	0	0	0	0	0	0	0	0	0	0	0	0	0	0	0	0	0
Methanohalophilus	1	5	0	0	0	0	0	0	0	0	0	0	0	0	0	0	0	0	0
Methanolobus	1	5	0	0	0	0	0	0	0	0	0	0	0	0	0	0	0	0	0
Methanomethylovorans	1	5	0	0	0	0	0	0	0	0	0	0	0	0	0	0	0	0	0
Methanoplanus	1	7	0	0	0	0	0	0	0	0	0	0	0	0	0	0	0	0	0
Methanopyrus	1	4	0	0	0	0	0	0	0	0	0	0	0	0	0	0	0	0	0
Methanoregula	1	8	0	0	0	0	0	0	0	0	0	0	0	0	0	0	0	0	0
Methanosaeta	1	9	0	0	0	0	0	0	0	0	0	0	0	0	0	0	0	0	0
Methanosalsum	1	7	0	0	0	0	0	0	0	0	0	0	0	0	0	0	0	0	0

Genus	Cluster Number	Protein Counts	K15982	K16045	K16047	K16049	K16050	K16051	K16173	K16249	K16269	K16270	K16514	K16873	K16874	K16876	K16880	K17067	K17070
**Methanosarcina**	1	18	0	0	0	0	0	0	0	0	0	0	0	0	0	0	0	0	0
**Methanosphaera**	1	7	0	0	0	0	0	0	0	0	0	0	0	0	0	0	0	0	0
**Methanosphaerula**	1	8	0	0	0	0	0	0	0	0	0	0	0	0	0	0	0	0	0
**Methanospirillum**	1	6	0	0	0	0	0	0	0	0	0	0	0	0	0	0	0	0	0
**Methanothermobacter**	1	6	0	0	0	0	0	0	0	0	0	0	0	0	0	0	0	0	0
**Methanothermococcus**	1	5	0	0	0	0	0	0	0	0	0	0	0	0	0	0	0	0	0
**Methanothermus**	1	5	0	0	0	0	0	0	0	0	0	0	0	0	0	0	0	0	0
**Methanotorris**	1	5	0	0	0	0	0	0	0	0	0	0	0	0	0	0	0	0	0
**Methylacidiphilum**	2	48	0	0	0	0	0	0	0	0	0	0	0	0	0	0	0	0	0
**Methylarcula**	1	2	0	0	0	0	0	0	0	0	0	0	0	0	0	0	0	0	0
**Methylibium**	1	38	0	0	0	0	0	0	0	1	0	0	0	0	0	0	0	0	0
**Methylobacillus**	2	61	0	0	0	0	0	0	0	0	0	0	0	0	0	0	0	0	0
**Methylobacterium**	3	236	0	0	0	0	0	0	0	0	0	0	0	1	1	1	1	0	1
**Methylocella**	3	117	0	0	0	0	0	0	0	0	0	0	0	0	0	0	0	0	0
**Methylococcus**	2	80	0	0	0	0	0	0	0	0	0	0	0	0	0	0	0	0	0
**Methylocystis**	1	21	0	0	0	0	0	0	0	0	0	0	0	0	0	0	0	0	0
**Methyloferula**	1	2	0	0	0	0	0	0	0	0	0	0	0	0	0	0	0	0	0
**Methylomicrobium**	1	12	0	0	0	0	0	0	0	0	0	0	0	0	0	0	0	0	0
**Methylomonas**	1	15	0	0	0	0	0	0	0	0	0	0	0	0	0	0	0	0	0
**Methylophaga**	1	7	0	0	0	0	0	0	0	0	0	0	0	0	0	0	0	0	0
**Methylophilus**	1	2	0	0	0	0	0	0	0	0	0	0	0	0	0	0	0	0	0
**Methylosinus**	1	2	0	0	0	0	0	0	0	0	0	0	0	0	0	0	0	0	0
**Methylotenera**	2	72	0	0	0	0	0	0	0	0	0	0	0	0	0	0	0	0	0
**Methyloversatilis**	1	5	0	0	0	0	0	0	0	0	0	0	0	0	0	0	0	0	0
**Methylovorus**	2	66	0	0	0	0	0	0	0	0	0	0	0	0	0	0	0	0	0
**Micavibrio**	1	9	0	0	0	0	0	0	0	0	0	0	0	0	0	0	0	0	0
**Microbacterium**	2	115	0	0	0	0	0	0	0	0	0	0	0	0	0	0	0	0	0
**Microchaete**	1	23	0	0	0	0	0	0	0	0	0	0	0	0	0	0	0	0	0
**Micrococcus**	2	72	0	0	0	0	0	0	0	0	0	0	0	0	0	0	0	0	0
**Microcoleus**	1	17	0	0	0	0	0	0	0	0	0	0	0	0	0	0	0	0	0
**Microcystis**	2	67	0	0	0	0	0	0	0	0	0	0	0	0	0	0	0	0	0
**Microlunatus**	1	25	0	0	0	0	0	0	0	0	0	0	0	0	0	0	0	0	0
**Micromonospora**	2	115	0	0	0	0	0	0	0	0	0	0	0	0	0	0	0	0	0
**Microvirga**	1	6	0	0	0	0	0	0	0	0	0	0	0	0	0	0	0	0	0
**Mobiluncus**	1	41	0	0	0	0	0	0	0	0	0	0	0	0	0	0	0	0	0
**Modestobacter**	1	43	0	0	0	0	0	0	0	0	0	0	1	0	0	0	0	0	0
**Moorea**	1	2	0	0	0	0	0	0	0	0	0	0	0	0	0	0	0	0	0
**Moorella**	2	69	0	0	0	0	0	0	0	0	0	0	0	0	0	0	0	0	0
**Moraxella**	2	66	0	0	0	0	0	0	0	0	0	0	0	0	0	0	0	0	0
**Morganella**	1	29	0	0	0	0	0	0	0	0	0	0	0	0	0	0	0	0	0
**Moritella**	1	2	0	0	0	0	0	0	0	0	0	0	0	0	0	0	0	0	0
**Muricauda**	1	23	0	0	0	0	0	0	0	0	0	0	0	0	0	0	0	0	0
**Mycobacterium**	3	306	1	1	1	1	1	1	0	0	0	0	0	0	0	0	0	1	0
**Mycoplasma**	1	53	0	0	0	0	0	0	0	0	0	0	0	0	0	0	0	0	0
**Myxococcus**	2	110	0	0	0	0	0	0	0	0	0	0	0	0	0	0	0	0	0
**Nakamurella**	2	115	0	0	0	0	0	0	0	0	0	0	0	0	0	0	0	1	0
**napthalene**	1	2	0	0	0	0	0	0	0	0	0	0	0	0	0	0	0	0	0
**Natranaerobius**	2	55	0	0	0	0	0	0	0	0	0	0	0	0	0	0	0	0	0
**Natrialba**	1	16	0	0	0	0	0	0	0	0	0	0	0	0	0	0	0	0	0
**Natrinema**	1	16	0	0	0	0	0	0	0	0	0	0	0	0	0	0	0	0	0
**Natronobacterium**	1	11	0	0	0	0	0	0	0	0	0	0	0	0	0	0	0	0	0
**Natronococcus**	1	21	0	0	0	0	0	0	0	0	0	0	0	0	0	0	0	0	0

Genus	Cluster Number	Protein Counts	K15982	K16045	K16047	K16049	K16050	K16051	K16173	K16249	K16269	K16270	K16514	K16873	K16874	K16876	K16880	K17067	K17070
Natronomonas	1	20	0	0	0	0	0	0	0	0	0	0	0	0	0	0	0	0	0
Nautilia	2	48	0	0	0	0	0	0	0	0	0	0	0	0	0	0	0	0	0
Neisseria	2	67	0	0	0	0	0	0	0	0	0	0	0	0	0	0	0	0	0
Neisseriaceae	1	1	0	0	0	0	0	0	0	0	0	0	0	0	0	0	0	0	0
Neorickettsia	1	31	0	0	0	0	0	0	0	0	0	0	0	0	0	0	0	0	0
Neptuniibacter	1	2	0	0	0	0	0	0	0	0	0	0	0	0	0	0	0	0	0
Niastella	1	21	0	0	0	0	0	0	0	0	0	0	0	0	0	0	0	0	0
Nitratifractor	2	54	0	0	0	0	0	0	0	0	0	0	0	0	0	0	0	0	0
Nitratireductor	1	19	0	0	0	0	0	0	0	0	0	0	0	0	0	0	0	0	0
Nitratiruptor	2	40	0	0	0	0	0	0	0	0	0	0	0	0	0	0	0	0	0
Nitrobacter	2	99	0	0	1	0	0	0	0	0	0	0	0	0	0	0	0	0	0
Nitrococcus	1	2	0	0	0	0	0	0	0	0	0	0	0	0	0	0	0	0	0
Nitrosococcus	2	94	0	0	0	0	0	0	0	0	0	0	0	0	0	0	0	0	0
Nitrosomonas	2	72	0	0	0	0	0	0	0	0	0	0	0	0	0	0	0	0	0
Nitrosopumilaceae	1	7	0	0	0	0	0	0	0	0	0	0	0	0	0	0	0	0	0
Nitrosopumilus	1	11	0	0	0	0	0	0	0	0	0	0	0	0	0	0	0	0	0
Nitrosospira	2	74	0	0	0	0	0	0	0	0	0	0	0	0	0	0	0	0	0
Nocardia	2	185	1	0	1	1	1	1	0	0	0	0	0	0	0	0	0	1	0
Nocardioidaceae	1	2	0	0	0	0	0	0	0	0	0	0	0	0	0	0	0	0	0
Nocardioides	2	157	1	0	1	0	1	0	0	0	0	0	0	0	0	0	0	0	0
Nocardiopsis	2	102	0	0	0	0	0	0	0	0	0	0	0	0	0	0	0	0	0
Nodularia	1	22	0	0	0	0	0	0	0	0	0	0	0	0	0	0	0	0	0
Nonlabens	1	19	0	0	0	0	0	0	0	0	0	0	0	0	0	0	0	0	0
Nostoc	2	111	0	0	0	0	0	0	0	0	0	0	0	0	0	0	0	0	0
Nostocaceae	1	2	0	0	0	0	0	0	0	0	0	0	0	0	0	0	0	0	0
Novosphingobium	2	169	1	0	1	1	0	1	0	0	0	0	1	0	0	0	0	0	0
Oceanibaculum	1	3	0	0	0	0	0	0	0	0	0	0	0	0	0	0	0	0	0
Oceanibulbus	1	12	0	0	0	0	0	0	0	0	0	0	0	0	0	0	0	0	0
Oceanicola	1	9	0	0	0	0	0	0	0	0	0	0	0	0	0	0	0	0	0
Oceanimonas	1	33	0	0	0	0	0	0	0	0	0	0	0	0	0	0	0	0	0
Oceaniovalibus	1	2	0	0	0	0	0	0	0	0	0	0	0	0	0	0	0	0	0
Oceanithermus	2	72	0	0	0	0	0	0	0	0	0	0	0	0	0	0	0	0	0
Oceanobacillus	2	91	0	0	0	0	0	0	0	0	0	0	0	0	0	0	0	0	0
Oceanospirillum	1	2	0	0	0	0	0	0	0	0	0	0	0	0	0	0	0	0	0
Ochrobactrum	2	133	0	0	0	0	0	0	0	0	0	0	0	0	0	0	0	0	0
Octadecabacter	1	29	0	0	0	0	0	0	0	0	0	0	0	0	0	0	0	0	0
Oenococcus	2	41	0	0	0	0	0	0	0	0	0	0	0	0	0	0	0	0	0
Oleomonas	1	2	0	0	0	0	0	0	0	0	0	0	0	0	0	0	0	0	0
Oligotropha	3	87	0	0	0	0	0	0	0	0	0	0	0	0	0	0	0	0	0
Olsenella	2	40	0	0	0	0	0	0	0	0	0	0	0	0	0	0	0	0	0
Opitutus	2	67	0	0	0	0	0	0	0	0	0	0	0	0	0	0	0	0	0
Orientia	1	21	0	0	0	0	0	0	0	0	0	0	0	0	0	0	0	0	0
Ornithobacterium	1	11	0	0	0	0	0	0	0	0	0	0	0	0	0	0	0	0	0
Oscillatoria	1	16	0	0	0	0	0	0	0	0	0	0	0	0	0	0	0	0	0
Oscillatoriales	1	2	0	0	0	0	0	0	0	0	0	0	0	0	0	0	0	0	0
Oscillibacter	1	20	0	0	0	0	0	0	0	0	0	0	0	0	0	0	0	0	0
Owenweeksia	1	15	0	0	0	0	0	0	0	0	0	0	0	0	0	0	0	0	0
Oxalobacteraceae	1	4	0	0	0	0	0	0	0	0	0	0	0	0	0	0	0	0	0
Paenibacillus	2	152	0	0	0	0	0	0	0	0	0	0	0	0	0	0	0	0	0
Paludibacter	2	67	0	0	0	0	0	0	0	0	0	0	0	0	0	0	0	0	0
Pandoraea	1	18	0	0	0	0	0	0	0	0	1	0	0	0	0	0	0	0	0
Pannonibacter	1	3	0	0	0	0	0	0	0	0	0	0	0	0	0	0	0	0	0

Genus	Cluster Number	Protein Counts	K15982	K16045	K16047	K16049	K16050	K16051	K16173	K16249	K16269	K16270	K16514	K16873	K16874	K16876	K16880	K17067	K17070
**Pantholops**	1	23	0	0	0	0	0	0	0	0	0	0	0	0	0	0	0	0	0
**Pantoea**	2	181	0	0	0	0	0	0	0	0	0	0	1	0	0	0	0	0	0
**Parabacteroides**	2	64	0	0	0	0	0	0	0	0	0	0	0	0	0	0	0	0	0
**Parachlamydia**	1	11	0	0	0	0	0	0	0	0	0	0	0	0	0	0	0	0	0
**Paracoccus**	2	169	0	0	0	0	0	0	0	0	0	0	0	0	0	0	0	0	0
**Parvibaculum**	3	117	0	0	0	0	0	0	0	0	0	0	0	0	0	0	0	0	0
**Parvularcula**	2	72	0	0	0	0	0	0	0	0	0	0	0	0	0	0	0	0	0
**Pasteurella**	2	85	0	0	0	0	0	0	0	0	0	0	0	0	0	0	0	0	0
**Pasteurellaceae**	1	2	0	0	0	0	0	0	0	0	0	0	0	0	0	0	0	0	0
**Pectobacterium**	1	44	0	0	0	0	0	0	0	0	0	0	0	0	0	0	0	0	0
**Pediococcus**	1	43	0	0	0	0	0	0	0	0	0	0	0	0	0	0	0	0	0
**Pedobacter**	2	88	0	0	0	0	0	0	0	0	0	0	0	0	0	0	0	0	0
**Pelagibaca**	1	10	0	0	0	0	0	0	0	0	0	0	0	0	0	0	0	0	0
**Pelagibacterium**	1	39	0	0	0	0	0	0	0	0	0	0	1	0	0	0	0	0	0
**Pelobacter**	2	85	0	0	0	0	0	0	0	0	0	0	0	0	0	0	0	0	0
**Pelodictyon**	2	62	0	0	0	0	0	0	0	0	0	0	0	0	0	0	0	0	0
**Pelosinus**	1	1	0	0	0	0	0	0	0	0	0	0	0	0	0	0	0	0	0
**Pelotomaculum**	2	60	0	0	0	0	0	0	0	0	0	0	0	0	1	0	0	0	0
**Peptostreptococcus**	1	1	0	0	0	0	0	0	0	0	0	0	0	0	0	0	0	0	0
**Persephonella**	2	55	0	0	0	0	0	0	0	0	0	0	0	0	0	0	0	0	0
**Petrotoga**	2	72	0	0	0	0	0	0	0	0	0	0	0	0	0	0	0	0	0
**Phaeobacter**	2	55	0	0	0	0	0	0	0	0	0	0	0	0	0	0	0	0	0
**Phenylobacterium**	3	103	0	0	0	0	0	0	0	0	0	0	0	0	0	0	0	0	0
**Photobacterium**	2	119	0	0	0	0	0	0	0	0	0	0	0	0	0	0	0	0	0
**Photorhabdus**	2	149	0	0	0	0	0	0	0	0	0	0	0	0	0	0	0	0	0
**Phycisphaera**	1	9	0	0	0	0	0	0	0	0	0	0	0	0	0	0	0	0	0
**Phyllobacterium**	1	6	0	0	0	0	0	0	0	0	0	0	0	0	0	0	0	0	0
**Picrophilus**	1	15	0	0	0	0	0	0	0	0	0	0	0	0	0	0	0	0	0
**Pirellula**	2	85	0	0	0	0	0	0	0	0	0	0	0	0	0	0	0	0	0
**Planctomyces**	2	93	0	0	0	0	0	0	0	0	0	0	0	0	0	0	0	0	0
**Plautia**	1	17	0	0	0	0	0	0	0	0	0	0	0	0	0	0	0	0	0
**Plesiomonas**	1	4	0	0	0	0	0	0	0	0	0	0	0	0	0	0	0	0	0
**Pleurocapsa**	1	23	0	0	0	0	0	0	0	0	0	0	0	0	0	0	0	0	0
**Polaribacter**	2	74	0	0	0	0	0	0	0	0	0	0	0	0	0	0	0	0	0
**Polaromonas**	3	238	0	0	0	0	0	0	0	0	0	0	1	0	0	0	0	0	0
**Polymorphum**	3	173	0	0	0	0	0	0	0	0	0	0	1	0	0	0	0	0	0
**Polynucleobacter**	1	19	0	0	0	0	0	0	0	0	0	0	0	0	0	0	0	0	0
**Ponticaulis**	1	1	0	0	0	0	0	0	0	0	0	0	0	0	0	0	0	0	0
**Porphyromonas**	2	63	0	0	0	0	0	0	0	0	0	0	0	0	0	0	0	0	0
**Prevotella**	1	65	0	0	0	0	0	0	0	0	0	0	0	0	0	0	0	0	0
**Prochlorococcus**	2	68	0	0	0	0	0	0	0	0	0	0	0	0	0	0	0	0	0
**Propionibacterium**	1	62	0	0	0	0	0	0	0	0	0	0	0	0	0	0	0	0	0
**Prosthecochloris**	1	16	0	0	0	0	0	0	0	0	0	0	0	0	0	0	0	0	0
**Proteobacteria**	1	19	0	0	0	0	0	0	0	0	0	0	0	0	0	0	0	0	0
**Proteus**	2	113	0	0	0	0	0	0	0	0	0	0	0	0	0	0	0	0	0
**Providencia**	1	38	0	0	0	0	0	0	0	0	0	0	0	0	0	0	0	0	0
**Pseudaminobacter**	1	10	0	0	0	0	0	0	0	0	0	0	0	0	0	0	0	0	0
**Pseudanabaena**	1	11	0	0	0	0	0	0	0	0	0	0	0	0	0	0	0	0	0
**Pseudoalteromonas**	2	188	1	0	1	1	1	0	0	0	0	0	1	0	0	0	0	0	0
**Pseudochrobactrum**	1	7	0	0	0	0	0	0	0	0	0	0	0	0	0	0	0	0	0
**Pseudogulbenkiania**	1	40	0	0	0	0	0	0	0	0	0	0	1	0	0	0	0	0	0
**pseudomallei**	3	131	0	0	0	0	0	0	0	0	0	0	0	0	0	0	0	0	0

Genus	Cluster Number	Protein Counts	K15982	K16045	K16047	K16049	K16050	K16051	K16173	K16249	K16269	K16270	K16514	K16873	K16874	K16876	K16880	K17067	K17070
Pseudomonas	3	381	0	0	1	1	1	1	0	1	1	1	1	0	0	1	1	0	0
Pseudonocardia	3	180	0	0	0	0	0	0	0	0	0	0	0	0	0	1	1	0	0
Pseudorhodobacter	1	4	0	0	0	0	0	0	0	0	0	0	0	0	0	0	0	0	0
Pseudovibrio	1	33	0	0	0	0	0	0	0	0	0	0	0	0	0	0	0	0	0
Pseudoxanthomonas	2	124	0	0	0	0	0	0	0	1	0	0	0	0	0	0	0	0	0
Psychrobacter	2	124	0	0	0	0	0	0	0	0	0	0	0	0	0	0	0	0	0
Psychroflexus	1	22	0	0	0	0	0	0	0	0	0	0	0	0	0	0	0	0	0
Psychromonas	2	110	0	0	0	0	0	0	0	0	0	0	0	0	0	0	0	0	0
Pusillimonas	3	111	0	0	0	0	0	0	0	0	0	0	0	0	0	0	0	0	0
Pyrobaculum	1	15	0	0	0	0	0	0	0	0	0	0	0	0	0	0	0	0	0
Pyrococcus	1	10	0	0	0	0	0	0	0	0	0	0	0	0	0	0	0	0	0
Pyrolobus	1	4	0	0	0	0	0	0	0	0	0	0	0	0	0	0	0	0	0
Rahnella	2	143	0	0	0	0	0	0	0	0	0	0	1	0	0	0	0	0	0
Ralstonia	3	332	1	0	1	1	1	1	0	1	1	0	1	0	0	1	1	0	0
Ramlibacter	1	35	0	0	0	0	0	0	0	0	0	0	0	0	0	0	0	0	0
Raoultella	2	139	0	0	0	0	0	0	0	0	0	0	1	0	0	0	0	0	0
Raphidiopsis	1	5	0	0	0	0	0	0	0	0	0	0	0	0	0	0	0	0	0
Reinekea	1	2	0	0	0	0	0	0	0	0	0	0	0	0	0	0	0	0	0
Renibacterium	2	67	0	0	0	0	0	0	0	0	0	0	0	0	0	0	0	0	0
Rhizobiaceae	1	3	0	0	0	0	0	0	0	0	0	0	0	0	0	0	0	0	0
Rhizobiales	1	6	0	0	0	0	0	0	0	0	0	0	0	0	0	0	0	0	0
Rhizobium	3	257	0	0	0	0	0	0	0	0	0	0	1	1	0	0	0	0	0
Rhizobium/Agrobacterium	2	87	0	0	0	0	0	0	0	0	0	0	0	0	0	0	0	0	0
Rhodanobacter	1	21	0	0	0	0	0	0	0	0	0	0	0	0	0	0	0	0	0
Rhodobacter	3	175	0	0	0	0	0	0	0	0	0	0	0	0	0	0	0	0	0
Rhodobacteraceae	1	21	0	0	0	0	0	0	0	0	0	0	0	0	0	0	0	0	0
Rhodobacterales	1	34	0	0	0	0	0	0	0	0	0	0	0	0	0	0	0	0	0
Rhodococcus	2	292	1	0	1	1	1	1	0	0	0	0	0	0	0	0	0	1	0
Rhodocyclaceae	1	7	0	0	0	0	0	0	0	0	0	0	0	0	0	0	0	0	0
Rhodoferax	3	144	0	0	0	0	0	0	0	0	0	0	0	0	0	0	0	0	0
Rhodomicrobium	2	103	0	0	0	0	0	0	0	0	0	0	0	0	0	0	0	0	0
Rhodopirellula	2	87	0	0	0	0	0	0	0	0	0	0	0	0	0	0	0	0	0
Rhodopseudomonas	3	246	0	0	0	0	0	0	0	0	0	0	1	0	1	1	1	0	0
Rhodospirillum	2	121	0	0	0	0	0	0	0	0	0	0	0	0	0	0	0	0	0
Rhodothermus	2	76	0	0	0	0	0	0	0	0	0	0	0	0	0	0	0	0	0
Rhodovulum	1	4	0	0	0	0	0	0	0	0	0	0	0	0	0	0	0	0	0
Richelia	1	2	0	0	0	0	0	0	0	0	0	0	0	0	0	0	0	0	0
Rickettsia	2	43	0	0	0	0	0	0	0	0	0	0	0	0	0	0	0	0	0
Rickettsiaceae	1	5	0	0	0	0	0	0	0	0	0	0	0	0	0	0	0	0	0
Riemerella	2	61	0	0	0	0	0	0	0	0	0	0	0	0	0	0	0	0	0
Rivularia	1	18	0	0	0	0	0	0	0	0	0	0	0	0	0	0	0	0	0
Robiginitalea	2	83	0	0	0	0	0	0	0	0	0	0	0	0	0	0	0	0	0
Roseburia	2	58	0	0	0	0	0	0	0	0	0	0	0	0	0	0	0	0	0
Roseibium	1	3	0	0	0	0	0	0	0	0	0	0	0	0	0	0	0	0	0
Roseiflexus	3	100	0	0	0	0	0	0	0	0	0	0	0	0	0	0	0	0	0
Roseobacter	2	149	0	0	0	0	0	0	0	0	0	0	0	0	0	0	0	0	0
Roseomonas	1	2	0	0	0	0	0	0	0	0	0	0	0	0	0	0	0	0	0
Roseovarius	1	15	0	0	0	0	0	0	0	0	0	0	0	0	0	0	0	0	0
Rothia	1	44	0	0	0	0	0	0	0	0	0	0	0	0	0	0	0	0	0
Rubrivivax	1	24	0	0	0	0	0	0	0	0	0	0	0	0	0	0	0	0	0
Rubrobacter	3	117	0	0	0	0	0	0	0	0	0	0	0	0	0	0	0	0	0
Ruegeria	2	189	0	0	0	0	0	0	0	0	0	0	0	0	0	0	0	0	0

Genus	Cluster Number	Protein Counts	K15982	K16045	K16047	K16049	K16050	K16051	K16173	K16249	K16269	K16270	K16514	K16873	K16874	K16876	K16880	K17067	K17070
Ruminococcaceae	1	2	0	0	0	0	0	0	0	0	0	0	0	0	0	0	0	0	0
Ruminococcus	2	101	0	0	0	0	0	0	0	0	0	0	0	0	0	0	0	0	0
Runella	1	26	0	0	0	0	0	0	0	0	0	0	1	0	0	0	0	0	0
Saccharomonospora	2	132	0	0	0	0	0	0	0	0	0	0	0	0	0	0	0	0	0
Saccharophagus	2	81	0	0	0	0	0	0	0	0	0	0	0	0	0	0	0	0	0
Saccharopolyspora	3	194	0	0	0	0	0	0	0	0	0	0	0	0	0	0	0	1	0
Saccharothrix	1	32	0	0	0	0	0	0	0	0	0	0	0	0	0	0	0	0	0
Sagittula	1	10	0	0	0	0	0	0	0	0	0	0	0	0	0	0	0	0	0
Salinibacter	2	77	0	0	0	0	0	0	0	0	0	0	0	0	0	0	0	0	0
Salinimonas	1	1	0	0	0	0	0	0	0	0	0	0	0	0	0	0	0	0	0
Salinispora	3	127	1	0	1	1	1	1	0	0	0	0	0	0	0	0	0	0	0
Salinivibrio	1	2	0	0	0	0	0	0	0	0	0	0	0	0	0	0	0	0	0
Salmonella	2	151	0	0	0	0	0	0	0	0	0	0	0	0	0	0	0	0	0
Sanguibacter	2	73	0	0	0	0	0	0	0	0	0	0	0	0	0	0	0	0	0
Saprospira	1	13	0	0	0	0	0	0	0	0	0	0	0	0	0	0	0	0	0
Scardovia	1	1	0	0	0	0	0	0	0	0	0	0	0	0	0	0	0	0	0
Scytonema	1	10	0	0	0	0	0	0	0	0	0	0	0	0	0	0	0	0	0
Sebaldella	2	69	0	0	0	0	0	0	0	0	0	0	0	0	0	0	0	0	0
Segniliparus	2	85	0	0	0	0	0	0	0	0	0	0	0	0	0	0	0	0	0
Selenomonas	2	57	0	0	0	0	0	0	0	0	0	0	0	0	0	0	0	0	0
Serinicoccus	1	1	0	0	0	0	0	0	0	0	0	0	0	0	0	0	0	0	0
Serratia	2	186	0	0	0	0	0	0	0	0	0	0	1	0	0	0	0	0	0
Shewanella	3	214	1	0	1	1	1	0	0	0	0	0	0	0	0	0	0	0	0
Shigella	2	172	0	0	0	0	0	0	0	0	0	0	0	0	0	0	0	0	0
Shinella	1	2	0	0	0	0	0	0	0	0	0	0	0	0	0	0	0	0	0
Sideroxydans	2	81	0	0	0	0	0	0	0	0	0	0	0	0	0	0	0	0	0
Silicibacter	2	105	0	0	0	0	0	0	0	0	0	0	0	0	0	0	0	0	0
Simiduia	1	18	0	0	0	0	0	0	0	0	0	0	0	0	0	0	0	0	0
Simkania	1	6	0	0	0	0	0	0	0	0	0	0	0	0	0	0	0	0	0
Simonsiella	1	2	0	0	0	0	0	0	0	0	0	0	0	0	0	0	0	0	0
Singulisphaera	1	18	0	0	0	0	0	0	0	0	0	0	0	0	0	0	0	0	0
Sinorhizobium	3	207	0	0	0	0	0	0	0	0	0	0	1	0	0	0	0	0	0
Slackia	2	46	0	0	0	0	0	0	0	0	0	0	0	0	0	0	0	0	0
Smaragdicoccus	1	3	0	0	0	0	0	0	0	0	0	0	0	0	0	0	0	0	0
Sodalis	2	71	0	0	0	0	0	0	0	0	0	0	0	0	0	0	0	0	0
Solibacillus	1	23	0	0	0	0	0	0	0	0	0	0	0	0	0	0	0	0	0
Solitalea	1	13	0	0	0	0	0	0	0	0	0	0	0	0	0	0	0	0	0
Sorangium	3	115	0	0	0	0	0	0	0	0	0	0	0	0	0	0	0	0	0
Sphaerobacter	2	100	0	0	0	0	0	0	0	0	0	0	0	0	0	0	0	0	0
Sphaerochaeta	1	42	0	0	0	0	0	0	0	0	0	0	0	0	0	0	0	0	0
Sphingobacterium	3	85	0	0	0	0	0	0	0	0	0	0	0	0	0	0	0	0	0
Sphingobium	1	179	0	0	1	1	0	0	0	1	0	0	1	0	0	0	0	0	0
Sphingomonadaceae	1	15	0	0	0	0	0	0	0	0	0	0	0	0	0	0	0	0	0
Sphingomonas	2	220	1	0	1	1	0	1	0	0	0	0	1	0	0	0	0	0	0
Sphingopyxis	3	99	0	0	0	0	0	0	0	0	0	0	0	0	0	0	0	0	0
Spirochaeta	2	103	0	0	0	0	0	0	0	0	0	0	0	0	0	0	0	0	0
Spiroplasma	1	6	0	0	0	0	0	0	0	0	0	0	0	0	0	0	0	0	0
Spirosoma	2	107	0	0	0	0	0	0	0	0	0	0	1	0	0	0	0	0	0
Stackebrandtia	2	110	0	0	0	0	0	0	0	0	0	0	0	0	0	0	0	0	0
Stanieria	1	17	0	0	0	0	0	0	0	0	0	0	0	0	0	0	0	0	0
Staphylococcus	2	108	0	0	0	0	0	0	0	0	0	0	0	0	0	0	0	0	0
Staphylothermus	1	12	0	0	0	0	0	0	0	0	0	0	0	0	0	0	0	0	0

Genus	Cluster Number	Protein Counts	K15982	K16045	K16047	K16049	K16050	K16051	K16173	K16249	K16269	K16270	K16514	K16873	K16874	K16876	K16880	K17067	K17070
Stappia	1	2	0	0	0	0	0	0	0	0	0	0	0	0	0	0	0	0	0
Starkeya	3	134	0	0	0	0	0	0	0	0	0	0	0	0	0	0	0	0	0
Stenotrophomonas	3	104	0	0	0	0	0	0	0	0	0	0	0	0	0	0	0	0	0
Stigmatella	3	129	0	0	1	0	0	0	0	0	0	0	0	0	0	0	0	0	0
Streptobacillus	1	38	0	0	0	0	0	0	0	0	0	0	0	0	0	0	0	0	0
Streptococcus	2	121	0	0	0	0	0	0	0	0	0	0	0	0	0	0	0	0	0
Streptomyces	2	253	0	0	1	0	0	0	0	0	0	0	1	0	0	1	1	1	0
Streptosporangium	3	148	0	0	1	0	0	0	0	0	0	0	0	0	0	0	0	0	0
Strigomonas	1	2	0	0	0	0	0	0	0	0	0	0	0	0	0	0	0	0	0
Sulfitobacter	1	10	0	0	0	0	0	0	0	0	0	0	0	0	0	0	0	0	0
Sulfobacillus	1	30	0	0	0	0	0	0	0	0	0	0	0	0	0	0	0	0	0
Sulfolobus	1	23	0	0	0	0	0	0	0	0	0	0	0	0	0	0	0	0	0
Sulfuricurvum	2	67	0	0	0	0	0	0	0	0	0	0	0	0	0	0	0	0	0
Sulfurihydrogenibium	2	55	0	0	0	0	0	0	0	0	0	0	0	0	0	0	0	0	0
Sulfurimonas	2	60	0	0	0	0	0	0	0	0	0	0	0	0	0	0	0	0	0
Sulfurospirillum	1	48	0	0	0	0	0	0	0	0	0	0	0	0	0	0	0	0	0
Sulfurovum	2	48	0	0	0	0	0	0	0	0	0	0	0	0	0	0	0	0	0
Symbiobacterium	2	68	0	0	0	0	0	0	0	0	0	0	0	0	0	0	0	0	0
Synechococcus	1	34	0	0	0	0	1	0	0	0	0	0	0	0	0	0	0	0	0
Synechocystis	1	40	0	0	0	0	0	0	0	0	0	0	0	0	0	0	0	0	0
Synergistetes	1	27	0	0	0	0	0	0	0	0	0	0	0	0	0	0	0	0	0
Syntrophobacter	2	101	0	0	0	0	0	0	0	0	0	0	0	0	0	0	0	0	0
Syntrophobotulus	2	71	0	0	0	0	0	0	0	0	0	0	0	0	0	0	0	0	0
Syntrophomonas	2	58	0	0	0	0	0	0	0	0	0	0	0	0	0	0	0	0	0
Syntrophothermus	2	60	0	0	0	0	0	0	0	0	0	0	0	0	0	0	0	0	0
Syntrophus	2	66	0	0	0	0	0	0	1	0	0	0	0	0	0	0	0	0	0
Tannerella	1	8	0	0	0	0	0	0	0	0	0	0	0	0	0	0	0	0	0
Taylorella	1	43	0	0	0	0	0	0	0	0	0	0	0	0	0	0	0	0	0
Tepidanaerobacter	2	55	0	0	0	0	0	0	0	0	0	0	0	0	0	0	0	0	0
Teredinibacter	2	109	0	0	0	0	0	0	0	0	0	0	0	0	0	0	0	0	0
Terrabacter	1	1	0	0	0	0	0	0	0	0	0	0	0	0	0	0	0	0	0
Terriglobus	2	83	0	0	0	0	0	0	0	0	0	0	0	0	0	0	0	0	0
Tetragenococcus	1	12	0	0	0	0	0	0	0	0	0	0	0	0	0	0	0	0	0
Thalassiobium	1	11	0	0	0	0	0	0	0	0	0	0	0	0	0	0	0	0	0
Thalassobacter	1	9	0	0	0	0	0	0	0	0	0	0	0	0	0	0	0	0	0
Thalassolituus	1	12	0	0	0	0	0	0	0	0	0	0	0	0	0	0	0	0	0
Thalassospira	1	2	0	0	0	0	0	0	0	0	0	0	0	0	0	0	0	0	0
Thauera	1	111	0	0	0	0	0	0	0	1	0	0	1	0	0	0	0	0	0
Thermacetogenium	1	7	0	0	0	0	0	0	0	0	0	0	0	0	0	0	0	0	0
Thermaerobacter	2	79	0	0	0	0	0	0	0	0	0	0	0	0	0	0	0	0	0
Thermanaerovibrio	2	58	0	0	0	0	0	0	0	0	0	0	0	0	0	0	0	0	0
Thermincola	2	62	0	0	0	0	0	0	0	0	0	0	0	0	0	0	0	0	0
Thermoanaerobacter	2	76	0	0	0	0	0	0	0	0	0	0	0	0	0	0	0	0	0
Thermoanaerobacterium	2	64	0	0	0	0	0	0	0	0	0	0	0	0	0	0	0	0	0
Thermobacillus	1	11	0	0	0	0	0	0	0	0	0	0	0	0	0	0	0	0	0
Thermobaculum	2	62	0	0	0	0	0	0	0	0	0	0	0	0	0	0	0	0	0
Thermobifida	2	72	0	0	0	0	0	0	0	0	0	0	0	0	0	0	0	0	0
Thermobispora	2	109	0	0	1	0	0	0	0	0	0	0	0	0	0	0	0	0	0
Thermococcus	1	13	0	0	0	0	0	0	0	0	0	0	0	0	0	0	0	0	0
Thermocrinis	2	60	0	0	0	0	0	0	0	0	0	0	0	0	0	0	0	0	0
Thermodesulfatator	1	10	0	0	0	0	0	0	0	0	0	0	0	0	0	0	0	0	0
Thermodesulfobacterium	1	9	0	0	0	0	0	0	0	0	0	0	0	0	0	0	0	0	0

Genus	Cluster Number	Protein Counts	K15982	K16045	K16047	K16049	K16050	K16051	K16173	K16249	K16269	K16270	K16514	K16873	K16874	K16876	K16880	K17067	K17070
Thermodesulfobium	1	13	0	0	0	0	0	0	0	0	0	0	0	0	0	0	0	0	0
Thermodesulfovibrio	2	60	0	0	0	0	0	0	0	0	0	0	0	0	0	0	0	0	0
Thermofilum	1	9	0	0	0	0	0	0	0	0	0	0	0	0	0	0	0	0	0
Thermogladius	1	7	0	0	0	0	0	0	0	0	0	0	0	0	0	0	0	0	0
Thermomicrobium	2	92	0	0	0	0	0	0	0	0	0	0	0	0	0	0	0	0	0
Thermomonospora	2	125	1	0	1	0	0	1	0	0	0	0	0	0	0	0	0	0	0
Thermoplasma	1	11	0	0	0	0	0	0	0	0	0	0	0	0	0	0	0	0	0
Thermoplasmatales	1	6	0	0	0	0	0	0	0	0	0	0	0	0	0	0	0	0	0
Thermoproteus	1	12	0	0	0	0	0	0	0	0	0	0	0	0	0	0	0	0	0
Thermosediminibacter	2	64	0	0	0	0	0	0	0	0	0	0	0	0	0	0	0	0	0
Thermosipho	2	70	0	0	0	0	0	0	0	0	0	0	0	0	0	0	0	0	0
Thermosphaera	1	10	0	0	0	0	0	0	0	0	0	0	0	0	0	0	0	0	0
Thermosynechococcus	1	6	0	0	0	0	0	0	0	0	0	0	0	0	0	0	0	0	0
Thermotoga	2	80	0	0	0	0	0	0	0	0	0	0	0	0	0	0	0	0	0
Thermovibrio	2	58	0	0	0	0	0	0	0	0	0	0	0	0	0	0	0	0	0
Thermovirga	1	9	0	0	0	0	0	0	0	0	0	0	0	0	0	0	0	0	0
Thermus	2	107	0	0	0	0	0	0	0	0	0	0	0	0	0	0	0	0	0
Thioalkalimicrobium	1	4	0	0	0	0	0	0	0	0	0	0	0	0	0	0	0	0	0
Thioalkalivibrio	2	91	0	0	0	0	0	0	0	0	0	0	0	0	0	0	0	0	0
Thiobaca	1	2	0	0	0	0	0	0	0	0	0	0	0	0	0	0	0	0	0
Thiobacillus	2	67	0	0	0	0	0	0	0	0	0	0	0	0	0	0	0	0	0
Thiocapsa	1	2	0	0	0	0	0	0	0	0	0	0	0	0	0	0	0	0	0
Thiocystis	1	11	0	0	0	0	0	0	0	0	0	0	0	0	0	0	0	0	0
Thioflavicoccus	1	13	0	0	0	0	0	0	0	0	0	0	0	0	0	0	0	0	0
Thiomicrospira	2	50	0	0	0	0	0	0	0	0	0	0	0	0	0	0	0	0	0
Thiomonas	2	85	0	0	0	0	0	0	0	0	0	0	0	0	0	0	0	0	0
Thiorhodococcus	1	2	0	0	0	0	0	0	0	0	0	0	0	0	0	0	0	0	0
Thiothrix	1	2	0	0	0	0	0	0	0	0	0	0	0	0	0	0	0	0	0
Tistrella	1	42	0	0	0	0	0	0	0	0	0	0	0	0	0	0	0	0	0
Tolumonas	2	100	0	0	0	0	0	0	0	0	0	0	0	0	0	0	0	0	0
Transposon	1	2	0	0	0	0	0	0	0	0	0	0	0	0	0	0	0	0	0
Treponema	1	63	0	0	0	0	0	0	0	0	0	0	0	0	0	0	0	0	0
Trichodesmium	2	71	0	0	0	0	0	0	0	0	0	0	0	0	0	0	0	0	0
Trichormus	1	12	0	0	0	0	0	0	0	0	0	0	0	0	0	0	0	0	0
Tropheryma	2	22	0	0	0	0	0	0	0	0	0	0	0	0	0	0	0	0	0
Truepera	2	104	0	0	0	0	0	0	0	0	0	0	0	0	0	0	0	0	0
Tsukamurella	2	123	1	0	1	1	1	1	0	0	0	0	0	0	0	0	0	0	0
Turneriella	1	16	0	0	0	0	0	0	0	0	0	0	0	0	0	0	0	0	0
Uliginosibacterium	1	2	0	0	0	0	0	0	0	0	0	0	0	0	0	0	0	0	0
Ureaplasma	1	24	0	0	0	0	0	0	0	0	0	0	0	0	0	0	0	0	0
Ureibacillus	1	2	0	0	0	0	0	0	0	0	0	0	0	0	0	0	0	0	0
Variovorax	3	187	0	0	0	0	0	0	0	0	0	0	0	0	0	0	0	0	0
Veillonella	1	54	0	0	0	0	0	0	0	0	0	0	0	0	0	0	0	0	0
Verminephrobacter	3	188	0	0	0	0	0	0	0	1	0	0	0	0	0	0	0	0	0
Verrucosispora	3	126	0	0	0	0	0	0	0	0	0	0	0	0	0	0	0	0	0
Vibrio	2	195	0	0	0	0	0	0	0	0	0	0	1	0	0	0	0	0	0
Vibrionales	1	46	0	0	0	0	0	0	0	0	0	0	0	0	0	0	0	0	0
Vitreoscilla	1	1	0	0	0	0	0	0	0	0	0	0	0	0	0	0	0	0	0
Vulcanisaeta	1	14	0	0	0	0	0	0	0	0	0	0	0	0	0	0	0	0	0
Waddlia	2	68	0	0	0	0	0	0	0	0	0	0	0	0	0	0	0	0	0
Wautersia	1	2	0	0	0	0	0	0	0	0	0	0	0	0	0	0	0	0	0
Weeksella	2	65	0	0	0	0	0	0	0	0	0	0	0	0	0	0	0	0	0

Genus	Cluster Number	Protein Counts	K15982	K16045	K16047	K16049	K16050	K16051	K16173	K16249	K16269	K16270	K16514	K16873	K16874	K16876	K16880	K17067	K17070
**Weissella**	1	14	0	0	0	0	0	0	0	0	0	0	0	0	0	0	0	0	0
**Wenxinia**	1	2	0	0	0	0	0	0	0	0	0	0	0	0	0	0	0	0	0
**Wigglesworthia**	1	29	0	0	0	0	0	0	0	0	0	0	0	0	0	0	0	0	0
**Wolbachia**	1	32	0	0	0	0	0	0	0	0	0	0	0	0	0	0	0	0	0
**Wolinella**	2	51	0	0	0	0	0	0	0	0	0	0	0	0	0	0	0	0	0
**Xanthobacter**	3	176	0	0	1	0	0	0	0	0	0	0	0	0	0	0	0	0	0
**Xanthobacteraceae**	1	4	0	0	0	0	0	0	0	0	0	0	0	0	0	0	0	0	0
**Xanthomonadaceae**	1	39	0	0	0	0	0	0	0	0	0	0	0	0	0	0	0	0	0
**Xanthomonas**	2	162	0	0	0	0	0	0	0	0	0	0	1	0	0	0	0	0	0
**Xenorhabdus**	2	127	0	0	0	0	0	0	0	0	0	0	0	0	0	0	0	0	0
**Xylanimonas**	2	66	0	0	0	0	0	0	0	0	0	0	0	0	0	0	0	0	0
**Xylella**	2	52	0	0	0	0	0	0	0	0	0	0	0	0	0	0	0	0	0
**Yersinia**	2	145	0	0	0	0	0	0	0	0	0	0	0	0	0	0	0	0	0
**Yokenella**	2	61	0	0	0	0	0	0	0	0	0	0	0	0	0	0	0	0	0
**Zobellia**	1	28	0	0	0	0	0	0	0	0	0	0	0	0	0	0	0	0	0
**Zunongwangia**	2	98	0	0	0	0	0	0	0	0	0	0	0	0	0	0	0	0	0
**Zymomonas**	2	67	0	0	0	0	0	0	0	0	0	0	0	0	0	0	0	0	0
**Zymophilus**	1	2	0	0	0	0	0	0	0	0	0	0	0	0	0	0	0	0	0
