# Supplementary material for: Xenobiotic Metabolism and Gut Microbiomes
Source: PLoS One. 2016 Oct 3;11(10):e0163099. doi: 10.1371/journal.pone.0163099 (PMC5047465; doi:10.1371/journal.pone.0163099)
Supplement: S5 Table — (PDF) [file pone.0163099.s024.pdf]

EC/KO	AM-AD-10	AM-AD-11	AM-AD-12	AM-AD-13	AM-AD-14	AM-AD-15	AM-AD-16	AM-AD-17	AM-AD-18	AM-AD-19	AM-AD-20	AM-AD-21	AM-AD-22	AM-AD-23	AM-AD-24	AM-AD-25	AM-AD-26	AM-AD-27
**1.1.1.-**	67	71	79	52	93	79	86	37	104	60	84	92	78	46	69	33	42	84
**1.1.1.1**	55	65	70	41	63	53	75	31	64	38	61	56	50	28	36	11	19	36
**1.1.1.157**	2	3	3	2	5	1	2	0	1	4	1	5	1	2	2	1	0	2
**1.1.1.205**	40	39	40	22	35	30	39	18	38	25	34	33	38	17	25	11	11	25
**1.1.1.35**	2	4	5	3	8	5	5	2	3	3	6	4	4	1	0	0	1	3
**1.12.99.6**	0	0	1	2	1	1	2	2	2	2	1	2	3	1	3	3	1	3
**1.13.11.-**	0	0	0	0	0	0	0	0	0	0	0	0	0	0	0	0	0	0
**1.13.11.1**	0	0	0	0	0	0	0	0	0	0	0	0	0	0	0	0	0	0
**1.13.11.2**	0	0	0	0	0	0	0	0	0	0	0	0	0	0	0	0	0	0
**1.13.11.3**	0	0	0	0	0	0	0	0	0	0	0	0	0	0	0	0	0	0
**1.13.11.39**	0	0	0	0	0	0	0	0	0	0	0	0	0	0	0	0	0	0
**1.13.11.5**	0	0	0	0	0	0	0	0	0	1	0	0	0	0	0	1	0	0
**1.13.11.8**	0	0	0	0	0	0	0	0	0	0	0	0	0	0	0	0	0	0
**1.14.12.10**	0	0	0	0	0	0	0	0	0	0	0	0	0	0	0	0	0	0
**1.14.12.13**	0	0	0	0	0	0	0	0	0	0	0	0	0	0	0	0	0	0
**1.14.12.18**	0	0	0	0	0	0	0	0	0	0	0	0	0	0	0	0	0	0
**1.14.13.-**	1	2	1	1	1	3	1	0	0	1	1	0	1	0	0	0	0	0
**1.14.13.1**	0	0	0	0	0	0	0	0	0	0	0	0	0	0	0	0	0	0
**1.14.13.2**	0	0	0	0	0	0	0	0	0	0	0	0	0	0	0	0	0	0
**1.14.13.50**	0	0	0	0	0	0	0	0	0	0	0	0	0	0	0	0	0	0
**1.14.13.7**	2	0	0	0	0	0	0	1	0	0	0	0	1	0	0	0	0	0
**1.14.13.8**	0	0	0	0	0	0	0	0	0	0	0	0	0	0	0	0	0	0
**1.14.13.82**	0	0	0	0	0	0	0	0	0	0	0	0	0	0	1	0	0	0
**1.14.99.-**	0	0	0	0	0	0	0	0	0	0	0	0	0	0	0	0	0	0
**1.17.99.1**	0	0	0	0	0	0	0	0	0	0	0	0	0	0	0	0	0	0
**1.18.6.1**	0	0	1	1	1	0	0	0	0	0	0	0	1	0	0	0	0	0
**1.2.1.-**	0	0	0	0	0	0	0	0	0	0	0	0	0	0	0	0	0	0
**1.2.1.10**	8	9	5	5	7	9	9	4	10	3	12	6	4	4	3	0	3	7
**1.2.1.3**	10	9	14	6	20	12	11	3	15	11	12	18	16	7	7	4	2	14
**1.2.1.39**	0	0	0	0	0	0	0	0	0	0	0	0	0	0	0	0	0	0
**1.2.7.1**	20	20	21	18	26	17	24	11	19	18	13	27	25	17	15	9	8	24
**1.2.99.2**	7	6	8	1	9	10	7	2	9	1	10	3	4	2	1	0	0	4
**1.3.1.-**	0	0	0	0	1	0	0	0	0	0	0	1	0	0	1	1	0	0
**1.3.1.2**	0	1	3	1	0	0	3	2	2	1	0	1	0	0	1	1	1	2
**1.3.1.25**	0	0	0	0	0	0	0	0	0	0	0	0	0	0	0	0	0	0
**1.3.99.-**	0	0	0	0	0	1	0	0	0	0	0	0	0	1	0	0	0	0
**1.6.5.-**	68	54	75	63	79	63	82	35	66	44	48	80	77	53	73	24	54	74
**1.7.1.-**	16	16	15	12	17	10	18	8	12	12	12	13	20	8	12	6	10	16
**1.8.99.3**	0	0	0	1	0	0	0	0	0	0	1	1	2	0	2	0	0	1
**2.1.1.-**	430	452	469	291	484	405	479	185	425	251	427	494	458	306	251	121	192	390
**2.3.1.-**	278	245	266	176	285	201	253	128	262	184	184	295	292	189	188	117	120	249
**2.3.1.16**	2	1	1	1	2	3	4	1	5	2	1	3	3	2	0	0	0	0
**2.3.1.5**	0	0	0	0	0	0	0	0	0	0	0	0	0	0	0	0	0	0
**2.3.1.9**	0	1	1	2	2	1	2	2	2	1	3	2	0	1	1	0	1	2
**2.4.2.10**	17	23	23	20	28	25	24	10	29	18	14	24	23	19	6	5	8	19
**2.4.2.3**	17	16	14	7	16	14	19	3	20	12	11	15	17	10	6	2	5	15
**2.4.2.4**	15	13	8	3	5	4	10	2	5	1	9	9	9	4	2	0	1	9
**2.4.2.8**	28	25	19	14	21	22	19	9	26	12	24	23	22	12	11	3	9	20
**2.5.1.-**	27	30	28	28	28	25	41	20	27	21	21	36	38	19	25	22	22	26

EC/KO	AM-AD-10	AM-AD-11	AM-AD-12	AM-AD-13	AM-AD-14	AM-AD-15	AM-AD-16	AM-AD-17	AM-AD-18	AM-AD-19	AM-AD-20	AM-AD-21	AM-AD-22	AM-AD-23	AM-AD-24	AM-AD-25	AM-AD-26	AM-AD-27
**2.5.1.18**	1	1	0	0	2	0	2	0	5	3	2	0	2	0	0	0	0	2
**2.6.1.-**	71	42	55	26	64	39	59	27	57	40	42	57	53	46	41	19	28	51
**2.7.1.21**	17	8	9	9	10	8	8	3	10	9	6	10	20	6	9	8	8	12
**2.7.1.48**	60	56	57	41	56	35	64	21	43	40	46	58	62	31	41	17	25	54
**2.7.4.-**	33	26	29	22	32	22	30	10	20	18	23	21	25	21	18	8	13	31
**2.8.3.-**	2	2	1	0	4	4	1	1	3	0	0	2	3	0	1	0	0	3
**2.8.3.1**	0	2	0	0	0	1	2	1	3	0	0	1	0	0	0	0	0	0
**2.8.3.12**	2	1	0	0	0	1	2	0	0	1	0	2	0	0	0	2	2	0
**2.8.3.6**	2	0	2	1	1	2	0	0	1	0	0	0	0	0	3	1	1	0
**2.8.3.8**	3	1	3	1	2	4	2	0	7	2	3	5	2	2	3	0	1	2
**3.1.1.-**	11	7	9	4	9	6	9	1	4	7	7	12	3	10	8	2	2	9
**3.1.1.1**	8	4	13	7	8	6	13	4	14	10	7	9	14	9	9	6	3	10
**3.1.1.17**	2	1	3	0	3	2	3	0	7	1	2	4	1	1	3	3	2	4
**3.1.1.2**	0	1	0	0	2	0	1	0	1	0	0	1	0	1	0	0	0	0
**3.1.1.24**	5	5	6	1	6	7	3	3	2	0	2	3	1	2	1	1	2	3
**3.1.1.45**	0	0	0	0	0	0	0	0	0	0	0	0	0	0	0	0	0	0
**3.1.2.-**	1	1	2	1	0	0	0	0	0	0	3	0	0	0	1	0	0	1
**3.1.2.23**	9	7	7	9	9	3	6	6	3	8	2	13	12	6	12	10	8	8
**3.1.3.1**	18	16	30	19	14	14	21	18	18	30	12	26	35	15	26	21	17	30
**3.1.3.2**	3	3	7	3	3	4	7	2	4	3	6	9	6	3	4	5	5	7
**3.1.3.41**	0	0	0	0	0	0	0	0	0	0	1	0	0	0	0	0	0	0
**3.2.1.31**	3	7	6	4	10	3	8	5	5	4	5	2	9	5	1	0	1	3
**3.3.2.9**	0	0	0	0	0	0	0	0	0	0	0	0	0	0	0	0	0	0
**3.5.1.-**	42	36	35	25	56	32	41	13	37	28	45	47	35	30	21	5	14	44
**3.5.1.4**	5	5	4	2	1	3	2	0	3	1	4	1	2	1	2	1	1	2
**3.5.1.5**	6	1	3	1	4	6	4	2	3	1	6	1	4	3	2	1	0	2
**3.5.1.54**	2	4	6	1	7	1	8	3	4	7	7	5	4	5	2	1	1	4
**3.5.1.6**	2	1	4	2	1	3	4	2	2	3	4	0	2	1	1	1	0	2
**3.5.2.-**	0	0	0	0	0	0	0	0	0	0	0	0	0	0	0	0	0	0
**3.5.2.2**	0	2	3	0	4	2	5	0	4	1	2	1	2	2	2	0	1	2
**3.5.4.-**	45	42	34	28	42	37	32	17	36	22	27	43	48	28	29	19	22	43
**3.5.4.5**	33	20	26	18	34	18	28	10	29	13	20	28	30	14	15	9	15	27
**3.5.5.1**	0	0	0	2	1	1	0	0	3	1	0	3	1	0	0	1	0	2
**3.5.5.7**	0	0	0	0	0	0	0	0	0	0	0	0	0	0	0	0	0	0
**3.5.99.3**	0	0	0	0	0	0	0	0	0	0	0	0	0	0	0	0	0	0
**3.6.1.7**	5	7	10	5	10	11	15	2	6	3	15	8	8	9	2	0	1	5
**3.7.1.-**	1	1	1	0	1	1	0	1	4	0	1	2	0	1	1	0	0	0
**3.7.1.2**	4	4	9	5	2	3	10	6	4	4	4	5	8	6	6	3	5	7
**3.8.1.2**	9	13	11	9	7	7	14	4	10	6	9	10	9	5	10	3	8	9
**3.8.1.3**	0	0	0	0	0	0	0	0	0	0	0	0	0	0	1	0	0	0
**3.8.1.5**	2	1	0	0	1	0	1	0	1	0	0	0	1	0	0	0	0	0
**4.1.1.-**	14	15	16	14	14	22	15	10	24	18	12	18	16	15	14	14	8	10
**4.1.1.44**	53	53	43	28	49	43	41	20	42	26	36	49	53	33	43	16	24	50
**4.1.1.55**	0	0	0	0	0	0	0	0	0	0	0	0	0	0	0	0	0	0
**4.1.1.7**	0	0	0	0	0	0	0	0	0	0	0	0	0	0	0	0	0	0
**4.1.1.70**	0	1	3	0	0	0	1	0	1	2	0	0	1	0	0	0	0	1
**4.1.1.77**	0	0	0	0	0	0	0	0	0	0	0	0	0	0	0	0	0	0
**4.1.2.-**	3	10	2	4	13	16	7	3	9	10	5	7	5	2	6	2	1	6
**4.1.3.-**	25	28	39	14	38	25	40	13	34	19	26	35	27	29	17	8	11	27

EC/KO	AM-AD-10	AM-AD-11	AM-AD-12	AM-AD-13	AM-AD-14	AM-AD-15	AM-AD-16	AM-AD-17	AM-AD-18	AM-AD-19	AM-AD-20	AM-AD-21	AM-AD-22	AM-AD-23	AM-AD-24	AM-AD-25	AM-AD-26	AM-AD-27
**4.1.3.39**	1	0	0	0	2	0	0	0	0	0	2	1	2	0	2	0	1	0
**4.1.99.-**	8	6	1	4	6	3	3	1	3	1	5	6	3	4	1	0	0	3
**4.2.1.-**	111	76	105	61	88	65	106	51	89	78	64	85	80	50	76	35	42	78
**4.2.1.17**	7	6	10	6	15	6	8	4	9	3	9	11	4	9	4	3	3	5
**4.2.1.80**	0	0	0	0	0	0	0	0	0	1	0	0	0	0	0	0	0	0
**4.2.1.83**	1	2	2	1	2	0	1	0	0	0	1	0	0	1	0	0	0	2
**4.2.1.84**	0	1	1	0	1	0	1	0	0	1	1	2	0	2	0	0	0	1
**5.1.2.2**	0	0	0	0	0	0	0	0	0	0	0	0	0	0	0	0	0	0
**5.2.1.2**	0	0	0	0	0	0	0	0	0	0	0	0	0	0	0	0	0	0
**5.3.3.4**	0	0	0	0	0	0	0	0	0	0	0	0	0	0	0	0	0	0
**5.3.99.-**	8	8	5	3	6	10	5	2	10	2	2	3	4	2	1	1	2	3
**5.4.99.-**	0	1	0	1	0	1	0	1	0	0	0	0	1	0	1	1	1	1
**5.5.1.1**	4	1	7	2	5	3	4	3	6	7	5	1	0	4	7	3	3	4
**5.5.1.2**	0	0	0	0	0	0	0	0	0	0	0	0	0	0	0	0	0	0
**6.2.1.-**	0	0	0	0	0	0	0	0	0	0	0	0	0	0	0	0	0	0
**6.3.5.2**	26	32	30	19	30	29	31	17	44	23	30	28	32	20	15	15	19	28
**K00002**	2	3	3	0	4	4	3	1	4	1	5	2	3	0	4	0	0	3
**K00055**	0	0	0	0	1	0	0	0	0	0	0	0	0	0	0	0	0	0
**K00074**	4	9	13	6	18	6	8	7	12	10	8	13	8	4	1	2	2	8
**K00088**	24	27	35	18	37	28	39	12	35	17	23	28	27	14	19	7	7	18
**K00100**	77	72	85	49	91	65	85	37	106	64	66	100	85	53	60	33	37	66
**K00128**	2	2	1	3	4	3	1	1	6	0	3	3	2	1	1	0	0	1
**K00129**	0	0	0	0	0	0	0	0	0	0	0	0	0	0	0	0	0	0
**K00132**	3	0	0	1	1	1	1	0	2	0	0	1	0	0	0	0	1	1
**K00141**	0	0	0	0	0	0	0	0	0	0	0	0	0	0	0	0	0	0
**K00146**	0	0	0	0	0	0	0	0	0	0	0	0	0	0	0	0	0	0
**K00148**	0	0	0	0	0	0	0	0	0	0	0	0	0	0	0	0	0	0
**K00155**	0	0	1	0	0	0	0	0	0	1	0	0	1	1	1	0	0	1
**K00169**	0	9	4	5	6	3	4	1	6	2	3	3	3	6	3	0	0	5
**K00224**	0	0	2	1	0	0	2	1	0	1	1	1	1	0	1	0	0	1
**K00274**	0	0	0	0	0	0	0	0	0	0	0	0	0	0	0	0	0	0
**K00446**	0	0	0	0	0	0	0	0	0	0	0	0	0	0	0	0	0	0
**K00448**	0	0	0	0	0	0	0	0	0	0	0	0	0	0	0	0	0	0
**K00462**	2	0	4	1	3	1	3	1	1	1	1	0	0	0	2	1	3	2
**K00480**	0	0	0	0	0	0	0	0	0	0	0	0	0	0	0	0	0	0
**K00481**	0	0	0	0	0	0	0	0	0	0	0	0	0	0	0	0	0	0
**K00539**	0	0	3	1	0	0	4	1	0	1	1	0	1	1	1	1	0	1
**K00599**	58	70	74	41	70	54	85	30	67	49	66	79	88	54	38	15	23	69
**K00626**	2	2	4	3	2	6	6	4	12	6	3	6	5	4	1	0	2	2
**K00632**	0	0	0	0	0	0	0	0	1	0	0	0	0	0	0	0	0	0
**K00680**	76	51	66	52	59	59	67	31	63	55	47	75	71	47	43	24	37	66
**K00757**	9	11	22	15	13	12	22	9	29	18	8	26	23	7	14	11	8	14
**K00758**	6	3	3	1	5	6	5	4	6	0	3	6	4	2	1	0	3	6
**K00760**	47	38	36	21	42	31	34	15	42	19	34	37	33	20	19	12	16	37
**K00799**	0	0	0	0	0	0	0	0	0	0	0	0	0	0	0	0	0	0
**K00857**	20	12	16	13	17	12	14	8	18	15	12	16	25	13	12	11	12	21
**K00876**	52	48	43	39	48	30	52	21	43	35	29	51	52	27	34	18	23	46
**K01026**	0	0	2	1	0	0	1	0	2	1	1	2	1	1	0	0	0	0
**K01031**	0	0	0	0	0	0	0	0	0	0	0	0	0	0	0	0	0	0

EC/KO	AM-AD-10	AM-AD-11	AM-AD-12	AM-AD-13	AM-AD-14	AM-AD-15	AM-AD-16	AM-AD-17	AM-AD-18	AM-AD-19	AM-AD-20	AM-AD-21	AM-AD-22	AM-AD-23	AM-AD-24	AM-AD-25	AM-AD-26	AM-AD-27
K01034	3	1	3	1	2	2	3	1	2	1	1	1	2	0	3	1	2	3
K01039	1	2	1	0	1	0	2	1	0	1	0	2	0	1	0	0	2	1
K01041	6	5	9	6	4	4	10	4	7	7	2	9	10	3	2	4	6	4
K01053	0	0	0	0	0	0	0	0	1	0	0	0	0	0	0	0	0	0
K01055	0	0	0	0	0	0	0	0	0	0	0	0	0	0	0	0	0	0
K01061	0	0	0	0	0	0	0	0	0	0	0	0	0	0	0	0	0	0
K01066	12	5	15	9	11	7	11	5	13	8	8	12	13	6	9	4	8	16
K01075	0	0	2	1	0	0	2	2	0	0	0	1	1	2	1	1	0	0
K01077	12	9	18	11	4	6	13	9	11	21	7	11	19	10	17	12	10	18
K01101	9	4	2	3	8	10	1	0	10	3	2	4	8	4	3	0	3	1
K01195	11	12	16	12	14	7	17	9	11	10	11	14	12	8	9	4	6	13
K01426	1	0	0	0	2	2	0	0	1	0	0	3	2	1	2	0	1	1
K01428	3	0	2	0	1	2	2	0	0	2	3	2	3	1	0	1	1	1
K01457	0	0	0	0	0	0	0	0	0	0	1	0	0	1	0	0	0	0
K01464	0	3	5	1	3	3	5	0	8	1	2	2	2	1	3	0	1	3
K01489	32	32	32	18	35	23	29	13	32	25	23	32	37	17	22	10	16	27
K01500	0	0	0	0	0	0	0	0	0	0	0	0	0	0	0	0	0	0
K01501	0	1	2	3	2	2	3	1	4	3	0	3	3	0	0	0	0	1
K01502	0	0	0	0	0	0	0	0	0	0	0	0	0	0	0	0	0	0
K01512	7	8	14	8	14	16	21	3	12	5	25	11	10	15	5	1	2	7
K01560	6	8	15	6	11	10	17	4	11	6	9	9	8	7	2	1	2	6
K01561	1	1	0	0	1	1	0	0	1	0	0	0	1	0	0	0	0	0
K01563	0	0	0	0	0	0	0	0	0	0	0	0	0	0	0	0	0	0
K01564	2	1	3	1	2	3	1	1	1	1	0	4	5	3	2	3	1	3
K01607	30	28	13	8	22	21	15	13	20	12	11	21	24	13	23	9	13	25
K01612	0	0	0	0	1	1	0	0	0	0	0	0	0	0	0	0	0	0
K01615	16	20	30	11	16	25	26	14	23	19	11	25	22	9	13	7	16	18
K01617	0	0	0	0	0	0	0	0	0	0	0	0	0	0	0	0	0	0
K01666	10	13	12	7	11	11	12	4	7	4	11	9	7	9	7	0	3	7
K01692	0	0	1	0	1	0	1	1	0	0	0	0	0	0	0	0	0	0
K01721	0	0	0	0	0	0	0	0	0	0	0	0	0	0	0	0	0	0
K01726	10	1	9	11	9	3	11	3	17	13	6	10	12	2	12	13	5	14
K01781	2	1	0	2	0	0	1	0	1	1	0	1	0	1	2	0	0	1
K01821	2	1	5	1	1	2	2	2	2	1	1	1	3	0	2	1	1	2
K01856	0	0	0	0	0	0	0	0	0	0	0	0	0	0	0	0	0	0
K01857	0	0	1	1	0	0	1	0	0	0	1	0	0	0	0	0	0	1
K01913	0	0	0	0	1	0	0	0	1	0	1	0	0	1	0	0	1	1
K01951	21	21	24	19	26	24	25	16	32	22	20	24	23	14	11	10	15	17
K02554	0	0	0	0	0	0	0	0	0	0	0	0	0	0	0	0	0	0
K03381	0	0	0	0	0	0	0	0	0	0	0	0	0	0	0	0	0	0
K03382	0	2	1	0	2	0	1	0	1	0	0	2	0	0	0	0	0	0
K03464	0	0	0	0	0	0	0	0	0	0	0	0	0	0	0	0	0	0
K03518	10	17	15	6	18	12	16	6	18	10	22	8	16	5	6	2	5	8
K03862	0	0	0	0	0	0	0	0	0	0	0	0	0	0	0	0	0	0
K04099	0	0	0	0	0	0	0	0	0	0	0	0	0	0	0	0	0	0
K04100	0	0	0	0	0	0	0	0	0	0	0	0	0	0	0	0	0	0
K04102	0	0	0	0	0	0	0	0	0	0	0	0	0	0	0	0	0	0
K04116	0	0	0	0	0	0	0	0	0	0	0	0	0	0	0	0	0	0
K05394	0	1	2	1	2	0	1	1	1	1	1	0	0	1	1	1	0	1

EC/KO	AM-AD-10	AM-AD-11	AM-AD-12	AM-AD-13	AM-AD-14	AM-AD-15	AM-AD-16	AM-AD-17	AM-AD-18	AM-AD-19	AM-AD-20	AM-AD-21	AM-AD-22	AM-AD-23	AM-AD-24	AM-AD-25	AM-AD-26	AM-AD-27
**K05549**	0	0	0	0	0	0	0	0	0	0	0	0	0	0	0	0	0	0
**K05783**	0	0	0	0	0	0	0	0	0	0	0	0	0	0	0	0	0	0
**K05797**	0	0	0	0	0	0	0	0	0	0	0	0	0	0	0	0	0	0
**K06281**	0	0	1	2	2	1	1	2	2	2	1	2	2	1	3	3	1	1
**K06446**	3	10	6	2	8	5	8	3	10	9	3	4	4	2	5	0	2	6
**K06912**	0	0	0	0	0	0	0	0	0	0	0	0	0	0	0	0	0	0
**K07535**	1	0	0	1	0	0	0	0	0	0	0	0	0	0	0	0	0	0
**K07536**	3	3	3	2	0	2	3	1	2	3	2	3	4	2	4	0	2	3
**K08689**	0	0	0	0	0	0	0	0	0	0	0	0	0	0	0	0	0	0
**K08710**	0	0	0	0	0	0	0	0	0	0	0	0	0	0	0	0	0	0
**K09461**	0	0	0	0	0	0	0	0	0	0	0	0	0	0	0	0	0	0
**K10217**	0	0	0	0	0	0	0	0	0	0	0	0	0	0	0	0	0	0
**K10218**	2	0	0	0	0	1	1	0	0	0	0	0	0	0	0	0	0	0
**K10220**	0	0	0	0	0	0	0	0	0	0	0	0	0	0	0	0	0	0
**K11180**	0	0	0	0	0	0	0	0	0	0	0	0	0	0	0	0	0	0
**K13953**	0	0	0	0	1	0	0	1	0	0	0	0	0	0	0	0	0	0
**K14333**	0	0	0	0	0	0	0	0	0	0	0	0	0	0	0	0	0	0
**K14519**	0	0	0	0	0	0	0	0	0	0	0	0	0	0	0	0	0	0
**K15054**	0	0	0	0	0	0	0	0	0	0	0	0	0	0	0	0	0	0
**K16173**	0	0	2	0	1	1	0	0	2	0	0	0	0	0	0	0	0	0
**K16514**	0	0	0	0	0	0	0	0	0	1	0	0	0	0	0	0	0	0
**K16874**	0	0	0	0	0	0	0	0	0	0	0	0	0	0	0	0	0	0

EC/KO	AM-AD-28	AM-AD-29	AM-AD-3	AM-AD-30	AM-AD-31	AM-AD-32	AM-AD-33	AM-AD-34	AM-AD-35	AM-AD-36	AM-AD-37	AM-AD-38	AM-AD-39	AM-AD-4	AM-AD-40	AM-AD-41	AM-AD-42	AM-AD-43	AM-AD-44
**1.1.1.-**	47	55	75	34	78	54	54	64	39	41	55	71	28	57	41	91	36	72	81
**1.1.1.1**	34	25	47	23	47	38	41	32	34	26	37	44	10	52	20	63	19	51	52
**1.1.1.157**	0	0	1	3	1	1	3	2	0	1	3	0	0	3	0	1	0	2	2
**1.1.1.205**	12	11	23	14	29	26	23	22	18	20	21	18	12	31	11	40	14	31	29
**1.1.1.35**	1	1	3	0	4	5	1	1	0	1	3	3	1	2	1	5	0	4	3
**1.12.99.6**	0	0	3	3	5	3	2	4	3	1	3	0	3	3	3	1	1	2	3
**1.13.11.-**	0	0	0	0	0	0	0	0	0	0	0	0	0	0	0	0	0	0	0
**1.13.11.1**	0	0	0	0	0	0	0	0	0	0	0	0	0	0	0	0	0	0	0
**1.13.11.2**	0	0	0	0	0	0	0	0	0	0	0	0	0	0	0	0	0	0	0
**1.13.11.3**	0	0	0	0	0	0	0	0	0	0	0	0	0	0	0	0	0	0	0
**1.13.11.39**	0	0	0	0	0	0	0	0	0	0	0	0	0	0	0	0	0	0	0
**1.13.11.5**	0	0	0	0	1	0	1	0	0	0	0	0	0	0	0	0	0	0	0
**1.13.11.8**	0	0	0	0	0	0	0	0	0	0	0	0	0	0	0	0	0	0	0
**1.14.12.10**	0	0	0	0	0	3	0	0	0	0	0	0	0	0	0	1	0	0	0
**1.14.12.13**	0	0	0	0	0	0	0	0	0	0	0	0	0	0	0	0	0	0	0
**1.14.12.18**	0	0	0	0	0	0	0	0	0	0	0	0	0	0	0	0	0	0	0
**1.14.13.-**	0	0	0	0	1	2	0	0	0	1	0	0	0	0	0	1	0	1	1
**1.14.13.1**	0	0	0	0	0	0	0	0	0	0	0	0	0	0	0	0	0	0	0
**1.14.13.2**	0	0	0	0	0	0	0	0	0	0	0	0	0	0	0	0	0	0	0
**1.14.13.50**	0	0	0	0	0	0	0	0	0	0	0	0	0	0	0	0	0	0	0
**1.14.13.7**	0	1	0	0	0	0	0	2	0	0	0	0	0	0	0	0	0	0	0
**1.14.13.8**	0	0	0	0	0	0	0	0	0	0	0	0	0	0	0	0	0	0	0
**1.14.13.82**	0	0	0	0	0	0	0	0	0	0	0	0	0	0	0	0	0	0	0
**1.14.99.-**	0	0	0	0	0	0	0	0	0	0	0	0	0	0	0	0	0	0	0
**1.17.99.1**	0	0	0	0	0	0	0	0	0	0	0	0	0	0	0	0	0	0	0
**1.18.6.1**	0	0	0	1	0	0	2	0	0	0	0	0	0	0	0	0	0	1	0
**1.2.1.-**	0	0	0	0	0	1	0	0	0	0	0	0	0	0	0	0	0	0	0
**1.2.1.10**	3	3	8	1	5	12	7	0	2	4	3	2	0	7	2	8	3	4	9
**1.2.1.3**	7	7	9	4	14	8	13	9	6	7	12	6	2	13	3	14	2	16	11
**1.2.1.39**	0	0	0	0	0	0	0	0	0	0	0	0	0	0	0	0	0	0	0
**1.2.7.1**	6	5	18	12	10	12	16	15	11	8	8	11	5	17	5	24	9	18	11
**1.2.99.2**	1	1	6	1	6	7	3	3	0	3	3	1	1	3	1	6	1	4	6
**1.3.1.-**	1	1	1	0	1	0	0	1	0	0	0	0	0	0	0	0	0	0	0
**1.3.1.2**	0	0	2	1	1	3	0	1	2	1	1	0	1	2	1	1	1	0	2
**1.3.1.25**	0	0	0	0	0	0	0	0	0	0	0	0	0	0	0	0	0	0	0
**1.3.99.-**	0	0	0	0	0	0	0	0	0	0	0	0	0	0	0	0	0	0	0
**1.6.5.-**	31	29	63	32	55	52	61	57	52	32	49	31	27	59	37	83	33	72	50
**1.7.1.-**	8	6	17	12	10	5	13	15	10	5	8	8	5	12	6	19	5	10	10
**1.8.99.3**	0	0	0	0	2	1	1	0	0	0	0	0	0	2	0	0	1	0	1
**2.1.1.-**	199	158	481	181	363	281	299	250	195	211	307	302	113	330	164	473	155	405	306
**2.3.1.-**	130	120	293	125	252	182	191	199	174	110	182	195	87	204	108	277	94	263	236
**2.3.1.16**	0	1	1	0	0	0	4	2	1	2	1	0	1	1	0	3	1	3	2
**2.3.1.5**	0	0	0	0	0	1	0	0	0	0	0	0	0	0	0	0	0	0	0
**2.3.1.9**	0	0	2	0	1	2	0	2	1	1	1	1	0	0	0	0	1	1	3
**2.4.2.10**	5	9	21	8	21	19	13	18	11	12	16	12	5	24	8	29	11	19	18
**2.4.2.3**	5	6	18	3	12	6	8	8	7	8	6	11	3	6	4	13	4	15	16
**2.4.2.4**	0	0	11	1	3	4	6	0	1	2	1	0	0	5	0	9	2	5	3
**2.4.2.8**	13	8	16	10	22	16	20	11	15	10	12	20	7	16	5	29	5	27	14
**2.5.1.-**	12	12	45	18	24	23	27	30	29	12	27	21	10	24	16	40	16	36	21

EC/KO	AM-AD-28	AM-AD-29	AM-AD-3	AM-AD-30	AM-AD-31	AM-AD-32	AM-AD-33	AM-AD-34	AM-AD-35	AM-AD-36	AM-AD-37	AM-AD-38	AM-AD-39	AM-AD-4	AM-AD-40	AM-AD-41	AM-AD-42	AM-AD-43	AM-AD-44
**2.5.1.18**	1	1	1	0	1	10	1	2	0	0	0	0	0	1	0	0	0	0	4
**2.6.1.-**	29	21	56	29	51	37	48	39	46	27	42	28	24	50	30	53	26	42	45
**2.7.1.21**	4	4	25	8	14	8	9	10	12	3	9	7	2	11	4	9	4	12	9
**2.7.1.48**	18	19	64	24	51	39	40	36	30	21	41	41	10	35	21	61	21	49	33
**2.7.4.-**	18	12	39	18	20	22	24	18	18	12	23	19	10	19	12	30	10	21	16
**2.8.3.-**	2	1	2	0	3	1	5	2	2	2	2	0	2	3	1	3	2	2	3
**2.8.3.1**	0	1	0	0	1	0	1	1	1	3	0	1	0	0	0	1	0	0	1
**2.8.3.12**	0	0	0	0	1	0	1	0	0	0	1	1	0	1	0	0	1	0	0
**2.8.3.6**	1	2	0	0	0	0	1	1	1	2	0	1	0	1	0	0	0	1	2
**2.8.3.8**	0	0	2	1	6	2	4	1	1	0	5	1	0	1	0	0	0	2	8
**3.1.1.-**	5	2	7	3	12	6	12	6	3	6	8	9	2	5	4	10	2	4	3
**3.1.1.1**	4	6	7	10	11	9	11	8	5	4	8	7	4	12	7	12	3	11	10
**3.1.1.17**	1	1	2	3	2	1	2	3	3	1	2	2	4	3	1	3	2	4	2
**3.1.1.2**	1	1	0	1	0	2	1	0	0	0	0	0	0	0	0	0	0	0	0
**3.1.1.24**	1	1	8	1	5	2	3	1	1	0	4	1	0	3	2	3	3	6	1
**3.1.1.45**	0	0	0	0	0	1	0	0	0	0	0	0	0	0	0	0	0	0	0
**3.1.2.-**	1	0	0	0	1	2	0	1	1	1	4	0	0	1	1	0	0	0	0
**3.1.2.23**	3	5	10	7	12	6	10	8	8	2	8	8	4	11	8	11	6	11	6
**3.1.3.1**	13	5	28	18	29	18	26	28	29	6	14	12	12	22	11	29	19	29	21
**3.1.3.2**	2	3	6	3	4	3	5	10	6	1	3	3	2	3	2	9	3	8	5
**3.1.3.41**	0	1	0	0	1	0	0	0	0	0	0	0	0	1	0	0	0	0	0
**3.2.1.31**	2	1	4	2	9	3	8	7	1	5	4	5	2	1	2	9	3	4	2
**3.3.2.9**	0	0	0	0	0	0	0	0	0	0	0	0	0	0	0	0	0	0	0
**3.5.1.-**	15	14	48	16	27	27	34	20	15	19	20	23	8	32	12	43	18	31	23
**3.5.1.4**	3	0	1	1	4	2	2	4	2	2	3	4	1	1	3	4	0	1	1
**3.5.1.5**	0	3	5	1	5	2	1	2	0	3	3	6	0	3	4	2	1	2	3
**3.5.1.54**	2	3	4	0	4	7	0	0	1	2	6	4	0	2	4	2	1	3	3
**3.5.1.6**	1	1	3	0	1	1	1	1	0	2	1	1	2	3	0	2	3	1	3
**3.5.2.-**	0	0	0	0	0	0	0	0	0	0	0	0	0	0	0	0	0	0	0
**3.5.2.2**	0	0	0	1	3	3	0	1	0	1	4	0	1	0	0	1	0	2	2
**3.5.4.-**	17	17	71	22	35	24	26	27	33	16	32	30	9	33	21	46	15	36	18
**3.5.4.5**	10	10	29	10	22	20	25	21	11	7	14	24	5	12	10	30	12	28	17
**3.5.5.1**	1	1	2	1	2	1	1	2	0	1	3	0	2	1	1	0	1	2	1
**3.5.5.7**	0	0	0	0	0	0	0	0	0	0	0	0	0	0	0	0	0	0	0
**3.5.99.3**	0	0	0	0	0	0	0	0	0	0	0	0	0	0	0	0	0	0	0
**3.6.1.7**	2	5	7	3	6	7	3	4	0	4	7	9	1	6	6	7	0	6	3
**3.7.1.-**	1	1	2	3	0	3	0	2	0	1	2	0	0	1	1	1	0	1	1
**3.7.1.2**	3	3	2	6	5	4	4	10	8	2	6	1	2	6	4	11	3	6	3
**3.8.1.2**	6	3	9	10	8	6	8	9	11	2	5	8	6	8	2	14	5	7	8
**3.8.1.3**	0	0	0	0	0	0	0	0	0	0	0	0	0	0	0	0	0	0	0
**3.8.1.5**	0	0	0	0	3	0	0	1	0	0	1	1	0	1	2	0	0	0	0
**4.1.1.-**	6	8	13	7	9	13	5	19	11	12	9	8	8	15	10	22	7	16	13
**4.1.1.44**	27	27	74	20	48	24	36	32	35	23	38	34	20	45	17	58	22	53	36
**4.1.1.55**	0	0	0	0	0	0	0	0	0	0	0	0	0	0	0	0	0	0	0
**4.1.1.7**	0	0	0	0	0	0	0	0	0	0	0	0	0	0	0	0	0	0	0
**4.1.1.70**	1	1	0	0	0	1	1	0	0	0	2	1	0	1	0	0	0	0	0
**4.1.1.77**	0	0	0	0	0	0	0	0	0	0	0	0	0	0	0	0	0	0	0
**4.1.2.-**	6	7	6	2	7	7	9	3	4	3	4	10	1	8	2	11	2	7	6
**4.1.3.-**	16	12	22	19	27	18	28	20	11	12	17	23	5	16	11	39	10	27	18

EC/KO	AM-AD-28	AM-AD-29	AM-AD-3	AM-AD-30	AM-AD-31	AM-AD-32	AM-AD-33	AM-AD-34	AM-AD-35	AM-AD-36	AM-AD-37	AM-AD-38	AM-AD-39	AM-AD-4	AM-AD-40	AM-AD-41	AM-AD-42	AM-AD-43	AM-AD-44
4.1.3.39	0	0	0	0	2	1	0	1	4	0	0	0	0	0	0	0	0	0	0
4.1.99.-	1	2	1	1	0	3	1	1	0	1	1	1	0	4	0	5	2	3	1
4.2.1.-	36	40	86	48	78	65	57	69	67	52	54	61	26	68	35	92	36	82	61
4.2.1.17	5	3	5	7	7	8	2	8	3	3	5	4	2	6	4	8	3	5	5
4.2.1.80	0	0	0	0	0	1	0	0	0	0	0	0	0	0	1	0	0	0	0
4.2.1.83	0	0	0	0	0	1	2	1	0	0	0	0	0	1	0	1	0	1	0
4.2.1.84	0	0	0	0	0	0	1	1	0	0	0	0	0	0	0	1	0	2	0
5.1.2.2	0	0	0	0	0	0	0	0	0	0	0	0	0	0	0	0	0	0	0
5.2.1.2	0	0	0	0	0	0	0	0	0	0	0	0	0	0	0	0	0	0	0
5.3.3.4	0	0	0	0	1	1	0	0	0	0	0	0	0	0	0	0	0	0	0
5.3.99.-	1	4	5	2	6	2	4	2	5	5	6	4	4	2	3	5	0	4	5
5.4.99.-	0	0	0	0	2	1	1	0	1	0	0	1	1	1	0	0	1	1	0
5.5.1.1	2	3	3	3	4	3	3	3	6	2	3	3	1	2	2	6	3	3	4
5.5.1.2	0	0	0	0	0	0	0	0	0	0	0	0	0	0	0	0	0	0	0
6.2.1.-	0	0	0	0	0	0	0	0	0	0	0	0	0	0	0	0	0	0	0
6.3.5.2	13	15	19	13	22	22	23	22	15	13	20	18	10	31	9	38	14	24	24
K00002	1	2	2	0	2	1	3	0	1	1	4	1	0	1	1	2	1	0	3
K00055	0	0	0	0	0	0	0	0	0	0	0	0	0	0	0	0	0	0	0
K00074	5	2	5	2	6	7	3	7	0	3	3	6	3	4	3	11	2	10	11
K00088	14	8	20	8	13	17	19	16	16	15	16	8	9	24	9	31	9	22	21
K00100	35	36	81	44	83	55	76	64	44	46	60	62	21	74	30	99	38	88	62
K00128	0	0	0	0	2	3	2	0	1	0	5	1	1	1	1	2	0	4	5
K00129	0	0	0	0	0	0	0	0	0	0	0	0	0	0	0	0	0	0	0
K00132	0	0	0	0	0	0	0	0	0	1	1	0	0	1	0	2	0	1	1
K00141	0	0	0	0	0	0	0	0	0	0	0	0	0	0	0	0	0	0	0
K00146	0	0	0	0	0	0	0	0	0	0	0	0	0	0	0	0	0	0	0
K00148	0	0	0	0	0	0	0	0	0	0	0	0	0	0	0	0	0	0	0
K00155	0	0	0	1	0	0	1	0	0	0	0	0	0	0	0	1	0	0	0
K00169	0	0	0	1	4	1	3	2	0	0	0	1	1	3	0	8	1	3	1
K00224	0	0	1	1	1	0	1	1	0	0	1	0	0	2	0	1	1	0	0
K00274	0	0	0	0	0	0	0	0	0	0	0	0	0	0	0	0	0	0	0
K00446	0	0	0	0	0	0	0	0	0	0	0	0	0	0	0	0	0	0	0
K00448	0	0	0	0	0	0	0	0	0	0	0	0	0	0	0	0	0	0	0
K00462	1	0	0	1	1	1	2	3	1	1	3	2	2	1	0	2	0	1	1
K00480	0	0	0	0	0	0	0	0	0	0	0	0	0	0	0	0	0	0	0
K00481	0	0	0	0	0	0	0	0	0	0	0	0	0	0	0	0	0	0	0
K00539	0	0	0	1	1	0	0	1	1	1	3	0	1	1	0	0	2	1	0
K00599	25	31	66	30	55	52	55	46	31	37	44	55	20	41	20	72	23	58	43
K00626	2	2	3	1	0	4	4	4	2	5	3	2	1	3	1	5	3	5	4
K00632	0	0	0	0	0	0	0	0	0	0	0	0	0	0	0	0	0	0	0
K00680	31	37	82	44	67	45	52	51	52	20	45	44	28	62	35	79	33	68	61
K00757	7	6	15	10	12	10	10	22	14	10	12	13	6	15	6	22	7	21	17
K00758	0	1	5	0	3	3	2	1	2	1	1	6	0	5	2	1	2	5	1
K00760	21	15	35	18	37	26	33	21	20	16	23	28	9	30	10	40	11	43	25
K00799	0	0	0	0	0	3	0	0	0	0	0	1	0	0	0	0	0	0	0
K00857	7	8	32	9	20	14	14	13	13	5	15	14	4	13	7	17	7	21	15
K00876	24	15	50	22	37	35	37	30	26	18	34	31	9	38	15	56	18	46	29
K01026	0	1	3	0	1	1	0	0	0	0	2	1	0	0	1	0	0	0	3
K01031	0	0	0	0	0	0	0	0	0	0	0	0	0	0	0	0	0	0	0

EC/KO	AM-AD-28	AM-AD-29	AM-AD-3	AM-AD-30	AM-AD-31	AM-AD-32	AM-AD-33	AM-AD-34	AM-AD-35	AM-AD-36	AM-AD-37	AM-AD-38	AM-AD-39	AM-AD-4	AM-AD-40	AM-AD-41	AM-AD-42	AM-AD-43	AM-AD-44
K01034	1	0	4	0	2	2	2	1	1	1	3	3	0	2	0	1	1	4	3
K01039	0	1	0	0	0	0	0	0	0	0	1	0	1	2	1	0	1	0	0
K01041	1	1	5	1	3	3	7	11	4	3	2	1	4	6	4	6	4	7	7
K01053	0	0	0	0	1	0	0	0	0	0	0	0	0	0	1	0	0	0	0
K01055	0	0	0	0	0	0	0	0	0	0	0	0	0	0	0	0	0	0	0
K01061	0	0	0	0	0	2	0	0	0	0	0	0	0	0	0	0	0	0	1
K01066	4	4	8	8	10	7	9	7	11	4	9	8	3	11	7	8	5	12	11
K01075	1	0	0	1	1	1	1	2	1	0	2	0	1	0	0	1	0	0	1
K01077	7	5	16	10	20	12	14	16	16	5	8	5	3	12	10	16	11	15	12
K01101	3	3	7	1	5	3	7	3	3	3	7	1	1	9	2	6	1	9	6
K01195	3	6	9	7	11	13	13	8	11	4	8	10	4	13	5	17	6	11	8
K01426	0	0	1	1	1	1	2	2	0	1	1	0	1	2	1	2	0	2	0
K01428	0	0	0	2	0	1	0	1	0	1	1	1	0	2	1	2	1	2	1
K01457	0	0	0	0	0	0	0	0	0	0	0	0	0	0	0	0	0	0	0
K01464	1	0	0	1	1	5	0	1	0	1	5	0	2	0	0	1	0	2	2
K01489	15	13	44	15	29	23	34	27	15	12	22	31	10	19	13	31	13	37	18
K01500	0	0	1	0	0	0	0	0	0	0	0	0	0	1	0	0	0	0	0
K01501	1	2	2	0	2	0	3	2	0	1	4	0	2	1	0	0	0	3	0
K01502	0	0	0	0	0	0	0	0	0	0	0	0	0	0	0	0	0	0	0
K01512	5	7	12	6	11	8	5	7	2	7	10	13	3	10	9	10	1	7	6
K01560	4	2	9	1	10	6	4	5	1	2	12	8	3	6	2	5	1	11	7
K01561	0	1	0	0	0	0	1	1	0	2	0	0	1	1	0	1	1	0	1
K01563	0	0	1	0	0	0	0	0	1	0	0	0	0	0	0	0	0	0	0
K01564	0	1	1	0	4	2	2	4	1	1	0	1	1	4	0	4	1	2	0
K01607	17	16	34	11	24	12	11	12	21	6	17	21	9	22	12	27	12	25	19
K01612	0	0	0	0	0	0	0	0	0	0	0	0	0	0	0	0	0	0	0
K01615	10	9	10	7	9	12	12	10	11	7	12	8	2	20	15	26	7	13	16
K01617	0	0	0	0	0	0	0	0	0	0	0	0	0	0	0	0	0	0	0
K01666	5	2	6	4	8	8	7	2	4	4	3	5	0	5	2	10	2	2	5
K01692	0	0	0	0	0	1	0	0	1	0	0	0	0	1	0	0	0	0	0
K01721	0	0	0	0	0	0	0	0	0	0	0	0	0	0	0	0	0	0	0
K01726	9	7	22	11	13	9	8	17	8	7	7	6	5	8	4	9	3	12	8
K01781	1	1	0	1	0	1	0	0	0	2	0	0	0	1	0	1	0	0	1
K01821	5	3	3	0	2	2	1	2	2	1	2	1	2	3	1	2	1	2	2
K01856	0	0	0	0	0	0	0	0	0	0	0	0	0	0	0	0	0	0	0
K01857	0	0	0	0	0	0	1	0	0	0	1	0	0	1	0	1	0	0	0
K01913	1	0	0	0	0	0	0	0	0	0	0	1	0	0	0	0	0	0	0
K01951	10	5	14	10	10	20	13	16	14	8	14	10	8	23	8	26	11	19	18
K02554	0	0	0	0	0	0	0	0	0	0	0	0	0	0	0	0	0	0	1
K03381	0	0	0	0	0	0	0	0	0	0	0	0	0	0	0	0	0	0	0
K03382	0	1	1	0	0	1	0	0	0	0	2	2	1	0	0	1	0	2	1
K03464	0	0	0	0	0	1	0	0	0	0	0	0	0	0	0	0	0	0	0
K03518	9	4	12	2	16	12	7	11	1	13	12	30	4	6	6	13	1	12	13
K03862	0	0	0	0	0	0	0	0	0	0	0	0	0	0	0	0	0	0	0
K04099	0	0	0	0	0	0	0	0	0	0	0	0	0	0	0	0	0	0	0
K04100	0	0	0	0	0	0	0	0	0	0	0	0	0	0	0	0	0	0	0
K04102	0	0	0	0	0	0	0	0	0	0	0	0	0	0	0	0	0	0	0
K04116	0	0	0	0	0	0	0	0	0	0	0	0	0	0	0	0	0	0	0
K05394	0	0	1	1	1	1	1	1	0	0	1	0	0	1	3	0	1	2	0

EC/KO	AM-AD-28	AM-AD-29	AM-AD-3	AM-AD-30	AM-AD-31	AM-AD-32	AM-AD-33	AM-AD-34	AM-AD-35	AM-AD-36	AM-AD-37	AM-AD-38	AM-AD-39	AM-AD-4	AM-AD-40	AM-AD-41	AM-AD-42	AM-AD-43	AM-AD-44
**K05549**	0	0	0	0	0	0	0	0	0	0	0	0	0	0	0	0	0	0	0
**K05783**	0	0	0	0	0	0	0	0	0	0	0	0	0	0	0	0	0	0	0
**K05797**	0	0	0	0	0	0	0	0	0	0	0	0	0	0	0	0	0	0	0
**K06281**	0	0	2	3	4	2	3	3	3	2	2	0	2	4	2	1	2	2	3
**K06446**	6	4	4	1	4	6	5	6	2	4	1	3	2	6	2	7	1	5	2
**K06912**	0	0	0	0	0	0	0	0	0	0	0	0	0	0	0	0	0	0	0
**K07535**	0	0	0	1	1	0	0	0	0	0	0	0	0	0	0	0	0	0	0
**K07536**	1	1	7	1	3	3	3	3	3	1	5	0	3	3	2	3	3	1	4
**K08689**	0	0	0	0	0	0	0	0	0	0	0	0	0	0	0	0	0	0	0
**K08710**	0	0	0	0	1	0	0	0	0	0	0	0	0	0	0	0	0	0	0
**K09461**	0	0	0	0	1	0	0	0	0	0	1	0	0	0	0	0	0	0	0
**K10217**	0	0	0	0	0	1	0	0	0	0	0	0	0	0	0	0	0	0	0
**K10218**	0	0	0	0	0	0	0	0	0	1	0	0	0	1	0	0	0	0	0
**K10220**	0	0	0	0	0	0	0	0	0	0	0	0	0	0	0	0	0	0	0
**K11180**	0	0	0	0	1	0	0	0	0	0	0	0	0	0	0	0	0	0	0
**K13953**	0	0	0	0	1	1	0	0	0	0	0	0	0	0	1	0	0	0	0
**K14333**	0	0	0	0	0	0	0	0	0	0	0	0	0	0	0	0	0	0	0
**K14519**	0	0	0	0	0	0	0	0	0	0	0	0	0	0	0	0	0	0	0
**K15054**	0	0	0	0	0	1	0	0	0	0	0	0	0	0	0	0	0	0	0
**K16173**	0	0	0	0	0	0	1	1	0	0	0	0	0	0	0	0	0	0	0
**K16514**	0	1	0	0	0	1	0	1	0	1	0	0	0	0	0	0	0	0	1
**K16874**	0	0	0	0	0	0	0	0	0	0	0	0	0	0	0	0	0	0	1

EC/KO	AM-AD-45	AM-AD-46	AM-AD-47	AM-AD-48	AM-AD-49	AM-AD-5	AM-AD-50	AM-AD-51	AM-AD-52	AM-AD-53	AM-AD-54	AM-AD-55	AM-AD-56	AM-AD-57	AM-AD-58	AM-AD-59	AM-AD-6	AM-AD-60	AM-AD-61
**1.1.1.-**	42	41	43	114	95	49	86	66	85	56	83	34	67	41	25	44	20	85	32
**1.1.1.1**	25	15	17	54	59	25	56	30	59	26	65	19	51	23	21	28	6	61	20
**1.1.1.157**	2	2	0	5	1	1	3	1	2	2	1	1	2	2	1	2	0	5	0
**1.1.1.205**	22	14	14	38	36	21	38	23	34	16	39	6	33	10	12	13	5	43	8
**1.1.1.35**	3	2	1	7	5	1	5	2	6	0	5	2	1	2	0	1	0	5	0
**1.12.99.6**	3	3	3	2	5	1	3	3	5	0	4	0	2	1	2	0	0	4	0
**1.13.11.-**	0	0	0	0	0	0	0	0	0	0	0	0	0	0	0	0	0	0	0
**1.13.11.1**	0	0	0	0	0	0	0	0	0	0	0	0	0	0	0	0	0	0	0
**1.13.11.2**	0	0	0	0	0	0	0	0	0	0	0	0	0	0	0	0	0	0	0
**1.13.11.3**	0	0	0	0	0	0	0	0	0	0	0	0	0	0	0	0	0	0	0
**1.13.11.39**	0	0	0	0	0	0	0	0	0	0	0	0	0	0	0	0	0	0	0
**1.13.11.5**	0	0	0	0	0	1	0	0	0	0	0	0	0	0	0	0	0	0	0
**1.13.11.8**	0	0	0	0	0	0	0	0	0	0	0	0	0	0	0	0	0	0	0
**1.14.12.10**	0	1	0	0	0	0	0	0	0	0	0	1	1	1	0	0	0	1	0
**1.14.12.13**	0	0	0	0	0	0	0	0	0	0	0	0	0	0	0	0	0	0	0
**1.14.12.18**	0	0	0	0	0	0	0	0	0	0	0	0	0	0	0	0	0	0	0
**1.14.13.-**	0	0	1	0	1	0	1	0	0	0	0	0	0	0	1	0	0	1	0
**1.14.13.1**	0	0	0	0	0	0	0	0	0	0	0	0	0	0	0	0	0	0	0
**1.14.13.2**	0	0	0	0	0	0	0	0	0	0	0	0	0	0	0	0	0	0	0
**1.14.13.50**	0	0	0	0	0	0	0	0	0	0	0	0	0	0	0	0	0	0	0
**1.14.13.7**	0	0	0	1	0	0	0	0	0	0	0	1	0	0	1	1	0	0	0
**1.14.13.8**	0	0	0	0	0	0	0	0	0	0	0	0	0	0	0	0	0	0	0
**1.14.13.82**	0	0	0	0	0	0	0	0	0	0	0	0	0	0	0	0	0	0	0
**1.14.99.-**	0	0	0	0	0	0	0	0	0	0	0	0	0	0	0	0	0	0	0
**1.17.99.1**	0	0	0	0	0	0	0	0	0	0	0	0	0	0	0	0	0	0	0
**1.18.6.1**	0	0	0	0	0	0	0	0	0	0	0	0	2	0	0	0	0	0	0
**1.2.1.-**	0	0	0	0	0	0	0	0	0	0	0	0	0	0	0	0	0	0	0
**1.2.1.10**	4	5	4	10	11	3	12	2	4	1	10	2	2	2	2	5	1	11	0
**1.2.1.3**	4	6	5	17	17	8	15	10	16	7	9	6	10	6	3	10	2	13	0
**1.2.1.39**	0	0	0	0	0	0	0	0	0	0	0	0	0	0	0	0	0	0	0
**1.2.7.1**	5	10	6	23	15	8	19	15	15	11	37	7	17	5	5	5	6	30	3
**1.2.99.2**	1	2	0	5	9	0	8	3	5	3	6	2	2	3	2	3	0	9	1
**1.3.1.-**	0	0	1	1	1	0	1	1	0	1	0	0	1	1	0	0	0	1	0
**1.3.1.2**	2	1	1	2	1	2	0	0	2	1	2	0	1	2	1	0	0	2	0
**1.3.1.25**	0	0	0	0	0	0	0	0	0	0	0	0	0	0	0	0	0	0	0
**1.3.99.-**	0	0	0	0	0	0	2	0	0	0	0	0	0	0	0	0	0	0	0
**1.6.5.-**	49	46	58	92	76	44	71	51	56	39	80	35	76	50	33	39	21	95	33
**1.7.1.-**	11	10	9	22	15	8	8	14	11	6	24	5	12	9	4	6	3	21	7
**1.8.99.3**	0	0	0	2	0	0	2	0	0	0	0	1	1	0	0	0	0	1	0
**2.1.1.-**	193	162	211	538	441	211	480	335	466	228	509	137	423	177	126	193	79	549	145
**2.3.1.-**	157	135	142	311	282	134	268	236	248	150	289	117	227	143	92	131	71	318	96
**2.3.1.16**	0	1	0	5	2	2	3	3	1	0	2	3	6	0	0	0	0	1	1
**2.3.1.5**	0	0	0	0	0	0	0	0	0	0	0	0	0	0	0	0	0	0	0
**2.3.1.9**	3	0	0	2	1	2	1	0	1	1	1	0	2	0	1	0	0	2	0
**2.4.2.10**	13	11	10	25	22	10	22	18	22	14	30	6	20	6	5	14	4	25	10
**2.4.2.3**	1	5	3	14	15	6	22	6	20	10	12	1	9	1	0	3	1	24	2
**2.4.2.4**	1	0	3	8	5	3	11	2	17	4	15	2	6	4	1	2	0	15	3
**2.4.2.8**	8	12	13	26	27	12	27	15	25	10	24	7	21	13	8	9	5	38	8
**2.5.1.-**	21	21	24	38	32	16	37	25	34	20	50	17	34	24	17	15	8	46	10

EC/KO	AM-AD-45	AM-AD-46	AM-AD-47	AM-AD-48	AM-AD-49	AM-AD-5	AM-AD-50	AM-AD-51	AM-AD-52	AM-AD-53	AM-AD-54	AM-AD-55	AM-AD-56	AM-AD-57	AM-AD-58	AM-AD-59	AM-AD-6	AM-AD-60	AM-AD-61
**2.5.1.18**	1	0	0	0	3	0	2	1	2	0	0	0	0	0	0	0	0	4	0
**2.6.1.-**	32	27	28	63	41	27	60	51	55	20	81	32	56	27	15	33	19	79	17
**2.7.1.21**	7	5	10	17	12	5	14	10	12	7	26	5	12	7	5	6	3	12	7
**2.7.1.48**	23	26	26	65	47	23	53	38	48	31	72	23	50	30	16	20	13	62	19
**2.7.4.-**	17	15	17	31	31	11	32	21	27	17	36	10	26	15	4	14	5	42	16
**2.8.3.-**	1	0	1	5	1	1	2	3	4	1	0	1	3	0	1	1	0	4	0
**2.8.3.1**	0	0	0	1	1	0	0	0	0	0	0	0	0	0	0	0	1	0	0
**2.8.3.12**	0	2	0	1	0	0	1	1	1	2	0	0	2	0	0	0	0	2	2
**2.8.3.6**	3	2	2	4	0	0	0	1	2	0	1	0	0	0	0	1	0	1	1
**2.8.3.8**	0	3	1	5	1	0	3	8	4	1	3	0	0	1	1	0	0	1	0
**3.1.1.-**	6	1	3	12	5	5	8	11	6	4	8	7	7	5	4	5	1	9	3
**3.1.1.1**	7	4	8	14	12	5	9	10	14	7	15	3	8	9	6	7	4	10	3
**3.1.1.17**	1	3	5	2	4	3	3	1	2	2	3	3	2	4	3	3	1	3	0
**3.1.1.2**	0	1	0	0	0	0	0	0	0	1	1	0	0	0	0	0	0	0	0
**3.1.1.24**	0	1	3	12	4	6	6	4	5	4	9	0	4	1	2	4	0	4	1
**3.1.1.45**	0	0	0	0	0	0	0	0	0	0	0	0	0	0	0	0	0	0	0
**3.1.2.-**	1	0	0	0	0	2	2	2	1	1	1	1	2	0	0	0	0	1	0
**3.1.2.23**	8	5	9	16	13	4	8	12	11	10	14	7	10	12	4	6	6	8	7
**3.1.3.1**	16	19	21	29	27	20	20	18	26	13	34	16	31	21	19	14	14	35	12
**3.1.3.2**	5	6	5	6	7	4	2	6	2	6	8	6	8	5	2	4	7	6	0
**3.1.3.41**	0	0	0	0	0	0	0	0	1	0	0	0	0	0	0	0	0	0	0
**3.2.1.31**	4	1	3	7	4	2	8	3	2	4	5	1	6	3	4	0	0	6	4
**3.3.2.9**	0	0	0	0	0	0	0	0	0	0	0	0	0	0	0	0	0	0	0
**3.5.1.-**	14	16	18	55	39	22	58	26	44	21	50	11	38	11	11	19	4	50	10
**3.5.1.4**	2	0	1	2	4	2	6	2	4	2	2	0	2	1	0	1	0	3	2
**3.5.1.5**	1	2	2	8	2	2	3	2	4	0	3	0	6	5	0	2	1	3	2
**3.5.1.54**	0	1	2	9	1	0	7	4	5	4	1	0	4	0	0	1	0	4	2
**3.5.1.6**	1	1	0	2	0	1	1	3	3	1	3	0	1	0	2	0	0	3	0
**3.5.2.-**	0	0	0	0	0	0	0	0	0	0	0	0	1	0	0	0	0	0	0
**3.5.2.2**	0	0	1	5	1	0	1	1	1	0	3	0	3	0	0	1	0	2	0
**3.5.4.-**	20	19	23	68	44	19	45	26	49	22	49	16	46	27	13	21	9	58	17
**3.5.4.5**	12	16	11	39	21	12	30	20	30	10	36	10	27	16	6	12	6	45	11
**3.5.5.1**	0	0	1	3	4	1	2	1	1	1	1	1	2	1	0	1	1	1	1
**3.5.5.7**	0	0	0	0	0	0	0	0	0	0	0	0	0	0	0	0	0	0	0
**3.5.99.3**	0	0	1	0	0	0	1	1	0	0	1	0	0	0	0	0	0	0	0
**3.6.1.7**	0	1	3	13	5	2	10	8	10	5	8	3	4	2	1	2	0	8	1
**3.7.1.-**	0	2	1	2	1	0	3	2	1	1	1	0	2	0	1	0	2	0	1
**3.7.1.2**	7	3	6	5	7	3	6	5	7	4	5	3	7	11	5	4	5	7	3
**3.8.1.2**	7	6	3	10	10	7	11	6	10	5	11	5	12	10	5	4	6	6	5
**3.8.1.3**	0	0	0	0	0	0	0	0	0	0	0	0	0	0	0	0	0	0	0
**3.8.1.5**	0	1	0	1	0	0	1	0	1	0	0	0	0	0	0	0	0	1	1
**4.1.1.-**	11	11	10	15	13	7	14	18	19	11	14	9	17	10	7	5	3	21	4
**4.1.1.44**	30	21	28	76	53	32	41	54	60	28	61	24	46	33	22	23	15	57	30
**4.1.1.55**	0	0	0	0	0	0	0	0	0	0	0	0	0	0	0	0	0	0	0
**4.1.1.7**	0	0	0	0	0	0	0	0	0	0	0	0	0	0	0	0	0	0	0
**4.1.1.70**	0	0	0	0	0	0	1	2	1	0	0	0	0	0	0	0	0	0	0
**4.1.1.77**	0	0	0	1	0	0	0	0	0	0	0	0	0	0	0	0	0	0	0
**4.1.2.-**	4	3	6	8	10	1	5	8	9	2	9	4	6	1	3	5	1	14	6
**4.1.3.-**	17	14	18	31	28	18	29	25	26	13	27	8	28	14	7	15	6	40	12

EC/KO	AM-AD-45	AM-AD-46	AM-AD-47	AM-AD-48	AM-AD-49	AM-AD-5	AM-AD-50	AM-AD-51	AM-AD-52	AM-AD-53	AM-AD-54	AM-AD-55	AM-AD-56	AM-AD-57	AM-AD-58	AM-AD-59	AM-AD-6	AM-AD-60	AM-AD-61
4.1.3.39	0	0	0	0	0	0	2	0	1	0	0	1	2	1	1	0	0	1	0
4.1.99.-	1	0	1	4	3	1	3	1	1	5	3	0	3	0	0	0	0	8	1
4.2.1.-	52	40	45	111	74	55	86	70	80	41	77	39	97	49	30	42	20	111	35
4.2.1.17	0	2	5	11	4	4	7	7	8	4	6	1	5	2	2	6	0	10	5
4.2.1.80	0	0	0	0	0	0	0	0	0	0	0	0	0	0	0	0	0	0	0
4.2.1.83	0	0	0	2	1	1	0	1	0	1	1	0	1	0	0	0	0	1	0
4.2.1.84	0	1	0	1	0	2	0	0	0	1	1	0	0	0	0	1	0	0	1
5.1.2.2	0	0	0	0	0	0	0	0	0	0	0	0	0	0	0	0	0	0	0
5.2.1.2	0	0	0	0	0	0	0	0	0	0	0	0	0	0	0	0	0	0	0
5.3.3.4	0	0	0	0	0	0	0	1	0	0	0	0	0	0	0	0	0	0	0
5.3.99.-	2	4	1	7	8	1	9	3	4	2	5	1	4	2	0	3	1	5	5
5.4.99.-	0	0	1	1	1	1	0	1	1	0	0	0	0	1	0	1	0	0	0
5.5.1.1	6	2	4	4	4	3	4	3	5	3	8	3	5	5	4	2	3	8	4
5.5.1.2	0	0	0	0	0	0	0	0	0	0	0	0	0	0	0	0	0	0	0
6.2.1.-	0	0	0	0	0	0	0	0	0	0	0	0	0	0	0	0	0	0	0
6.3.5.2	18	19	22	38	46	22	32	18	24	15	36	9	29	12	12	15	10	38	10
K00002	1	0	0	1	5	2	4	2	3	1	2	0	2	3	1	1	0	2	1
K00055	0	0	0	0	0	0	1	0	0	0	0	1	0	0	0	0	0	0	0
K00074	7	5	4	9	6	2	7	3	5	3	6	3	4	3	0	2	0	9	0
K00088	13	10	13	21	27	10	24	19	23	16	31	8	23	11	13	12	4	32	13
K00100	49	32	44	92	88	43	91	78	70	35	106	51	76	56	29	49	22	105	22
K00128	1	0	1	2	4	2	3	2	4	2	3	1	1	0	1	3	0	4	0
K00129	0	0	0	0	0	0	0	0	0	0	0	0	0	0	0	0	0	0	0
K00132	0	0	0	1	1	2	0	2	1	0	2	0	3	0	0	2	0	2	0
K00141	0	0	0	0	0	0	0	0	0	0	0	0	0	0	0	0	0	0	0
K00146	0	0	0	0	0	0	0	0	0	0	0	0	0	0	0	0	0	0	0
K00148	0	0	0	0	0	0	0	0	0	0	0	0	0	0	0	0	0	0	0
K00155	0	0	0	0	0	0	0	0	0	0	3	0	0	0	1	0	0	0	0
K00169	0	0	0	3	3	0	3	3	2	2	6	0	2	0	0	0	1	9	0
K00224	0	1	1	0	1	0	1	1	1	0	1	0	1	0	1	0	0	0	0
K00274	0	0	0	0	0	0	0	0	0	0	0	0	0	0	0	0	0	0	0
K00446	0	0	0	0	0	0	0	0	0	0	0	0	0	0	0	0	0	0	0
K00448	0	0	0	0	0	0	0	0	0	0	0	0	0	0	0	0	0	0	0
K00462	2	1	0	0	1	2	2	2	3	2	2	2	2	2	0	2	0	2	2
K00480	0	0	0	0	0	0	0	0	0	0	0	0	0	0	0	0	0	0	0
K00481	0	0	0	0	0	0	0	0	0	0	0	0	0	0	0	0	0	0	0
K00539	1	0	0	1	0	1	1	1	1	0	1	0	2	1	1	0	0	0	0
K00599	34	20	23	87	49	37	72	57	66	36	86	16	77	40	15	36	17	84	28
K00626	2	1	0	8	3	4	4	3	2	1	3	3	9	0	1	0	0	3	1
K00632	0	0	0	0	0	0	0	0	0	0	0	0	0	0	0	0	0	0	0
K00680	37	25	33	82	71	37	70	65	61	36	79	28	60	48	22	35	23	74	33
K00757	6	10	9	20	23	10	18	13	19	15	15	7	16	12	5	9	3	22	5
K00758	0	2	2	3	6	2	12	3	3	2	8	0	4	2	1	3	0	6	1
K00760	15	19	19	43	38	20	36	31	42	17	38	10	34	16	10	20	8	51	18
K00799	0	0	0	1	0	0	0	0	1	0	1	0	1	1	0	0	0	0	0
K00857	9	9	18	30	20	7	22	17	21	11	32	8	17	12	5	9	5	20	9
K00876	20	20	26	52	51	22	48	31	45	30	56	21	48	28	15	21	11	57	17
K01026	1	0	0	2	0	0	2	1	2	0	1	1	0	0	0	0	0	1	0
K01031	0	0	0	0	0	0	0	1	0	0	0	0	0	0	0	0	0	0	0

EC/KO	AM-AD-45	AM-AD-46	AM-AD-47	AM-AD-48	AM-AD-49	AM-AD-5	AM-AD-50	AM-AD-51	AM-AD-52	AM-AD-53	AM-AD-54	AM-AD-55	AM-AD-56	AM-AD-57	AM-AD-58	AM-AD-59	AM-AD-6	AM-AD-60	AM-AD-61
K01034	0	0	2	3	2	1	5	3	3	1	2	0	3	1	0	2	0	4	1
K01039	0	0	0	0	0	0	0	1	0	0	1	0	1	0	0	0	0	0	1
K01041	3	5	4	10	8	2	5	3	4	4	16	3	5	6	5	3	1	11	1
K01053	0	0	0	1	0	0	0	0	1	0	0	1	0	0	0	0	0	0	0
K01055	0	0	0	0	0	0	0	0	0	0	0	0	0	0	0	0	0	0	0
K01061	0	0	0	0	0	0	0	0	0	0	0	0	0	0	0	0	0	0	0
K01066	5	2	7	11	13	4	10	15	17	5	16	3	10	6	4	6	3	11	3
K01075	0	0	0	0	1	1	0	1	2	1	2	0	2	1	1	0	0	0	0
K01077	7	10	10	12	16	6	17	13	15	11	17	8	23	10	12	7	6	19	7
K01101	1	2	0	5	15	1	8	9	7	1	5	0	4	0	2	2	1	8	2
K01195	10	4	5	11	12	6	13	4	10	6	21	7	15	8	5	8	3	13	5
K01426	0	1	0	1	2	0	0	2	1	2	4	0	0	0	1	0	0	3	0
K01428	0	0	1	3	2	2	2	3	1	2	2	0	1	1	0	2	0	2	0
K01457	0	0	0	0	0	0	0	0	0	0	1	0	0	0	0	0	0	0	0
K01464	0	0	1	4	2	0	2	1	1	0	2	1	1	0	0	1	1	2	0
K01489	14	19	16	41	31	13	35	30	36	15	39	11	32	17	7	13	7	40	15
K01500	0	0	0	1	0	0	0	0	0	0	0	0	1	0	0	0	0	0	0
K01501	0	0	1	5	5	1	3	4	2	1	3	0	2	0	0	1	1	3	2
K01502	0	0	0	0	0	0	0	0	0	0	0	0	0	0	0	0	0	0	0
K01512	3	2	7	17	7	5	17	13	15	8	11	3	10	2	1	4	0	13	4
K01560	1	3	5	11	13	3	6	8	7	2	10	0	7	0	0	4	1	7	7
K01561	0	0	0	0	0	0	0	1	1	0	0	1	0	0	0	0	0	1	0
K01563	0	0	0	0	0	0	0	0	0	0	1	0	0	0	0	0	0	0	0
K01564	1	1	2	3	4	1	3	1	0	2	1	1	4	3	1	2	1	4	1
K01607	16	11	13	41	27	13	22	23	30	12	34	12	22	18	11	14	7	27	14
K01612	0	0	0	0	0	0	0	0	0	0	0	0	0	0	0	0	0	0	0
K01615	13	9	8	14	20	11	20	15	20	10	26	4	22	9	12	16	3	28	5
K01617	0	0	0	0	0	0	0	0	0	0	0	0	0	0	0	0	0	0	0
K01666	4	1	5	5	4	6	13	9	7	4	9	2	9	2	3	3	2	10	3
K01692	0	0	0	0	0	0	1	0	1	1	1	0	0	1	0	0	0	0	0
K01721	0	0	0	0	0	0	0	0	0	0	0	0	0	0	0	0	0	0	0
K01726	7	7	10	18	15	7	11	13	4	10	22	11	14	10	10	5	5	16	4
K01781	0	0	0	1	0	0	1	1	0	0	0	0	0	0	0	0	0	1	0
K01821	1	2	1	4	4	2	2	4	4	6	4	2	1	0	1	1	1	2	2
K01856	0	0	0	0	0	0	0	0	0	0	0	0	0	0	0	0	0	0	0
K01857	0	0	0	0	0	0	1	0	1	0	1	0	0	0	0	0	0	1	0
K01913	0	0	0	1	0	0	0	1	1	0	0	0	1	0	0	0	0	0	0
K01951	14	14	14	13	34	13	16	8	17	12	25	8	19	9	11	11	6	30	7
K02554	0	0	0	0	0	0	0	0	0	0	0	0	0	0	0	0	0	0	0
K03381	0	0	0	0	0	0	0	0	0	0	0	0	0	0	0	0	0	0	0
K03382	0	0	1	1	3	0	2	1	1	2	1	1	0	0	0	0	0	0	1
K03464	0	0	0	0	0	0	0	0	0	0	0	0	0	0	0	0	0	0	0
K03518	2	9	5	24	15	0	15	6	9	12	8	6	10	2	3	1	2	19	5
K03862	0	0	0	0	0	0	0	0	0	0	0	0	0	0	0	0	0	0	0
K04099	0	0	0	0	0	0	0	0	0	0	0	0	0	0	0	0	0	0	0
K04100	0	0	0	0	0	0	0	0	0	0	0	0	0	0	0	0	0	0	0
K04102	0	0	0	0	1	0	0	0	0	0	0	0	0	0	0	0	0	0	0
K04116	0	0	0	0	0	0	0	0	0	0	0	0	0	0	0	0	0	0	0
K05394	1	0	0	0	3	0	2	1	1	1	0	0	2	1	1	1	0	1	1

EC/KO	AM-AD-45	AM-AD-46	AM-AD-47	AM-AD-48	AM-AD-49	AM-AD-5	AM-AD-50	AM-AD-51	AM-AD-52	AM-AD-53	AM-AD-54	AM-AD-55	AM-AD-56	AM-AD-57	AM-AD-58	AM-AD-59	AM-AD-6	AM-AD-60	AM-AD-61
K05549	0	0	0	0	0	0	0	0	0	0	0	0	0	0	0	0	0	0	0
K05783	0	0	0	0	0	0	0	0	0	0	0	0	0	0	0	0	0	0	0
K05797	0	0	0	0	0	0	0	0	0	0	0	0	0	0	0	0	0	0	0
K06281	2	2	3	1	4	1	3	3	3	0	3	0	1	1	2	0	0	3	0
K06446	3	1	2	4	4	2	5	4	2	2	2	3	4	4	2	1	1	4	5
K06912	0	0	0	0	0	0	0	0	0	0	0	0	0	0	0	0	0	0	0
K07535	0	0	0	1	0	0	0	0	0	0	0	0	0	0	0	0	0	1	0
K07536	3	3	2	7	4	2	2	2	1	3	5	3	2	3	1	1	0	5	3
K08689	0	0	0	0	0	0	0	0	0	0	0	0	0	0	0	0	0	0	0
K08710	0	0	0	0	0	0	0	0	0	0	0	0	0	0	0	0	0	0	0
K09461	0	0	0	0	0	0	0	0	1	0	0	0	0	0	0	0	0	0	0
K10217	0	0	0	0	0	0	0	0	0	0	0	0	0	0	0	0	0	0	0
K10218	0	0	1	1	0	0	0	0	0	0	0	0	0	0	1	0	1	2	0
K10220	0	0	0	0	0	0	0	0	0	0	0	0	0	0	0	0	0	0	0
K11180	0	0	0	0	0	0	0	0	0	0	0	0	0	0	0	0	0	0	0
K13953	0	1	0	0	1	0	2	0	0	0	0	0	0	0	0	0	0	0	0
K14333	0	0	0	0	0	0	0	0	0	0	0	0	0	0	0	0	0	0	0
K14519	0	0	0	0	0	0	0	0	0	0	0	0	0	0	0	0	0	0	0
K15054	0	0	0	0	0	0	0	0	0	0	0	0	0	0	0	0	0	0	0
K16173	0	1	0	2	0	0	3	1	0	0	0	1	0	0	0	0	0	0	0
K16514	0	0	0	0	0	0	0	0	0	0	0	0	0	0	0	0	0	0	0
K16874	0	0	0	1	0	0	1	0	0	0	0	0	0	0	0	0	0	0	0

EC/KO	AM-AD-62	AM-AD-63	AM-AD-64	AM-AD-65	AM-AD-66	AM-AD-67	AM-AD-68	AM-AD-69	AM-AD-7	AM-AD-70	AM-AD-71	AM-AD-72	AM-AD-73	AM-AD-74	AM-AD-75	AM-AD-76	AM-AD-77	AM-AD-78
**1.1.1.-**	69	82	23	41	21	48	38	41	57	61	27	99	81	44	101	73	74	77
**1.1.1.1**	47	52	12	11	21	25	21	30	23	32	12	62	41	18	49	40	33	61
**1.1.1.157**	1	1	0	0	3	2	1	0	1	2	2	2	2	2	2	1	2	3
**1.1.1.205**	30	32	7	17	11	20	15	16	23	26	13	39	28	13	39	28	28	38
**1.1.1.35**	4	6	1	1	0	3	1	1	5	2	2	8	0	0	5	1	4	4
**1.12.99.6**	5	3	0	5	0	3	1	0	0	5	1	2	1	3	1	5	3	2
**1.13.11.-**	0	0	0	0	0	0	0	0	0	0	0	0	0	0	0	0	0	0
**1.13.11.1**	0	0	0	0	0	0	0	0	0	0	0	0	0	0	0	0	0	0
**1.13.11.2**	0	0	0	0	0	0	0	0	0	0	0	0	0	0	0	0	0	0
**1.13.11.3**	0	0	0	0	0	0	0	0	0	0	0	0	0	0	0	0	0	0
**1.13.11.39**	0	0	0	0	0	0	0	0	0	0	0	0	0	0	0	0	0	0
**1.13.11.5**	0	1	0	1	0	0	0	0	0	0	0	0	0	1	0	0	0	0
**1.13.11.8**	0	0	0	0	0	0	0	0	0	0	0	0	0	0	0	0	0	0
**1.14.12.10**	1	0	0	0	0	1	0	0	0	1	0	0	0	0	1	0	0	0
**1.14.12.13**	0	0	0	0	0	0	0	0	0	0	0	0	0	0	0	0	0	0
**1.14.12.18**	0	0	0	0	0	0	0	0	0	0	0	0	0	0	0	0	0	0
**1.14.13.-**	0	0	0	1	0	0	0	1	0	1	0	0	0	0	1	0	1	0
**1.14.13.1**	0	0	0	0	0	0	0	0	0	0	0	0	0	0	0	0	0	0
**1.14.13.2**	0	0	0	0	0	0	0	0	0	0	0	0	0	0	0	0	0	0
**1.14.13.50**	0	0	0	0	0	0	0	0	0	0	0	0	0	0	0	0	0	0
**1.14.13.7**	1	0	0	0	0	0	1	2	2	0	0	0	1	0	0	0	0	0
**1.14.13.8**	0	0	0	0	0	0	0	0	0	0	0	0	0	0	0	0	0	0
**1.14.13.82**	0	0	0	0	0	0	0	0	0	0	0	0	0	0	0	0	0	0
**1.14.99.-**	0	0	0	0	0	0	0	0	0	0	0	0	0	0	0	0	0	0
**1.17.99.1**	0	0	0	0	0	0	0	0	0	0	0	0	0	0	0	0	0	0
**1.18.6.1**	0	2	0	0	0	0	0	0	0	0	0	1	2	0	0	0	0	0
**1.2.1.-**	0	0	0	0	0	0	0	0	0	0	0	0	0	0	0	0	0	0
**1.2.1.10**	5	4	0	2	2	2	4	3	6	8	0	5	7	0	8	7	12	9
**1.2.1.3**	12	13	5	8	4	8	7	4	5	4	6	16	9	4	14	13	12	15
**1.2.1.39**	0	0	0	0	0	0	0	0	0	0	0	0	0	0	0	1	0	0
**1.2.7.1**	12	28	6	7	5	14	7	11	12	9	5	20	27	9	18	11	16	24
**1.2.99.2**	4	3	0	1	2	3	1	1	6	1	2	4	3	1	10	0	3	4
**1.3.1.-**	1	1	0	0	0	0	0	0	1	0	1	0	0	0	0	1	0	1
**1.3.1.2**	1	3	0	0	1	2	2	0	0	1	1	1	0	1	1	1	1	1
**1.3.1.25**	0	0	0	0	0	0	0	0	0	0	0	0	0	0	0	0	0	0
**1.3.99.-**	0	0	0	0	0	0	1	0	0	0	0	0	0	0	0	0	0	0
**1.6.5.-**	81	85	10	40	21	44	44	54	75	52	30	99	74	42	75	56	72	64
**1.7.1.-**	15	10	6	9	4	7	8	9	20	9	3	18	15	10	12	12	10	12
**1.8.99.3**	0	1	0	0	0	0	0	0	1	0	0	1	0	1	1	1	1	0
**2.1.1.-**	349	498	93	176	113	220	154	240	306	276	115	573	388	151	429	344	375	441
**2.3.1.-**	263	309	71	174	73	160	137	153	215	183	71	312	262	138	220	197	254	242
**2.3.1.16**	1	0	0	3	1	2	0	2	0	0	0	4	1	0	2	3	3	7
**2.3.1.5**	0	0	0	0	0	0	0	0	0	0	0	0	0	0	0	0	0	0
**2.3.1.9**	0	4	1	0	0	1	0	0	0	0	0	2	1	1	2	0	0	3
**2.4.2.10**	18	25	4	12	7	14	7	10	14	15	6	25	15	9	25	16	15	27
**2.4.2.3**	9	14	3	8	3	4	7	3	7	4	6	16	8	4	19	9	8	11
**2.4.2.4**	4	11	0	1	2	2	0	3	4	6	1	4	8	2	7	3	2	9
**2.4.2.8**	19	24	5	11	7	16	8	11	22	14	6	41	21	9	21	19	21	28
**2.5.1.-**	31	38	9	20	3	19	19	25	29	30	11	38	38	20	34	26	29	20

EC/KO	AM-AD-62	AM-AD-63	AM-AD-64	AM-AD-65	AM-AD-66	AM-AD-67	AM-AD-68	AM-AD-69	AM-AD-7	AM-AD-70	AM-AD-71	AM-AD-72	AM-AD-73	AM-AD-74	AM-AD-75	AM-AD-76	AM-AD-77	AM-AD-78
**2.5.1.18**	0	2	0	0	1	0	4	0	0	0	0	2	0	0	6	1	0	1
**2.6.1.-**	55	75	14	40	16	39	27	28	42	26	20	68	65	33	65	40	56	41
**2.7.1.21**	13	15	2	8	6	9	8	5	13	13	4	24	8	11	11	9	12	10
**2.7.1.48**	46	63	9	29	17	32	22	27	47	35	23	65	45	25	57	42	43	47
**2.7.4.-**	26	31	8	14	5	13	7	15	21	14	9	45	26	14	24	14	26	22
**2.8.3.-**	2	2	1	3	0	2	1	2	2	0	1	2	1	0	2	1	3	2
**2.8.3.1**	0	0	0	1	0	1	1	0	0	0	0	1	1	0	0	0	0	0
**2.8.3.12**	2	0	0	1	1	2	0	0	0	0	1	1	2	0	1	0	0	1
**2.8.3.6**	0	3	0	2	0	2	0	0	1	2	0	1	0	1	2	0	0	0
**2.8.3.8**	4	5	1	0	0	3	0	1	1	3	0	1	1	1	2	2	5	4
**3.1.1.-**	11	11	7	5	2	4	4	3	6	6	4	10	8	4	9	2	9	9
**3.1.1.1**	6	11	4	5	4	11	6	5	6	7	4	12	13	8	11	6	14	8
**3.1.1.17**	3	3	0	2	0	2	2	0	1	5	2	0	3	2	2	3	2	4
**3.1.1.2**	0	0	0	0	0	0	0	0	0	0	0	0	0	0	1	1	0	0
**3.1.1.24**	4	6	1	2	1	5	0	2	4	1	0	5	4	0	1	3	1	3
**3.1.1.45**	0	0	0	0	0	0	0	0	0	0	0	0	0	0	0	0	0	0
**3.1.2.-**	1	0	1	2	0	0	0	0	0	1	0	0	0	1	1	0	1	0
**3.1.2.23**	13	13	3	12	3	8	7	6	7	6	2	8	14	12	9	5	13	8
**3.1.3.1**	27	34	9	19	11	24	15	27	25	20	10	21	38	25	26	24	29	28
**3.1.3.2**	4	6	3	3	2	4	5	7	8	5	2	5	9	7	4	5	5	8
**3.1.3.41**	0	0	0	0	0	0	0	0	0	0	0	0	0	0	0	0	0	0
**3.2.1.31**	4	3	4	4	5	1	2	2	5	4	3	6	4	1	7	3	6	6
**3.3.2.9**	0	0	0	0	0	0	0	0	0	0	0	0	0	0	0	0	0	0
**3.5.1.-**	34	41	9	15	9	22	14	20	33	20	12	44	40	15	36	22	34	43
**3.5.1.4**	3	3	2	2	2	3	2	2	1	1	1	5	5	0	6	2	5	2
**3.5.1.5**	3	3	0	2	0	2	2	3	0	2	3	2	3	1	2	3	2	1
**3.5.1.54**	4	5	1	0	1	1	2	1	2	1	0	5	1	0	7	5	3	1
**3.5.1.6**	1	4	2	1	1	1	1	0	0	3	0	4	2	1	1	1	0	1
**3.5.2.-**	0	0	0	0	0	0	0	0	0	0	0	0	0	0	0	0	0	0
**3.5.2.2**	1	2	0	1	0	2	1	1	1	0	0	2	2	0	4	0	0	2
**3.5.4.-**	39	57	5	32	11	26	18	24	41	32	9	57	39	22	49	27	36	32
**3.5.4.5**	17	33	3	14	8	12	12	11	25	10	13	29	28	10	18	30	21	18
**3.5.5.1**	1	2	0	0	0	0	1	0	0	4	1	3	0	1	1	1	0	1
**3.5.5.7**	0	0	0	0	0	0	0	0	0	0	0	0	0	0	0	0	0	0
**3.5.99.3**	0	0	1	0	0	0	0	0	0	0	0	0	0	0	0	0	0	0
**3.6.1.7**	2	7	2	2	2	2	0	1	3	2	4	8	5	1	16	8	2	7
**3.7.1.-**	1	3	0	0	0	2	0	0	0	2	0	2	1	0	4	2	1	3
**3.7.1.2**	9	9	3	6	3	3	4	8	6	1	2	6	10	6	7	5	6	3
**3.8.1.2**	7	12	3	9	3	7	7	5	6	8	4	14	11	7	11	2	8	8
**3.8.1.3**	0	0	0	0	0	0	0	0	0	0	0	1	0	0	0	0	0	0
**3.8.1.5**	0	0	1	0	0	0	0	0	0	0	0	1	0	0	1	1	0	1
**4.1.1.-**	14	18	6	15	5	9	7	13	9	17	6	23	18	8	24	11	24	6
**4.1.1.44**	50	66	8	31	29	39	42	25	40	32	21	45	45	22	37	29	44	45
**4.1.1.55**	0	0	0	0	0	0	0	0	0	0	0	0	0	0	0	0	0	0
**4.1.1.7**	0	0	0	0	0	0	0	0	0	0	0	0	0	0	0	0	0	0
**4.1.1.70**	0	0	0	0	0	0	0	0	0	0	0	0	1	0	0	1	1	1
**4.1.1.77**	0	0	0	0	0	0	0	0	0	0	0	0	0	0	0	0	0	0
**4.1.2.-**	6	8	1	3	0	3	3	3	4	2	4	12	10	3	9	8	4	6
**4.1.3.-**	28	26	5	14	11	10	12	17	14	20	12	34	26	14	20	28	17	34

EC/KO	AM-AD-62	AM-AD-63	AM-AD-64	AM-AD-65	AM-AD-66	AM-AD-67	AM-AD-68	AM-AD-69	AM-AD-7	AM-AD-70	AM-AD-71	AM-AD-72	AM-AD-73	AM-AD-74	AM-AD-75	AM-AD-76	AM-AD-77	AM-AD-78	
4.1.3.39	2	0	0	1	0	0	0	2	1	0	1	0	2	0	0	1	2	1	
4.1.99.-	1	1	1	0	1	0	0	3	2	3	2	3	1	0	2	1	0	5	
4.2.1.-	87	97	20	49	28	51	43	58	49	65	31	102	77	60	100	67	79	79	
4.2.1.17	7	13	1	2	0	6	3	4	4	3	4	10	4	2	9	6	7	9	
4.2.1.80	0	0	0	0	0	0	0	0	0	0	0	0	0	1	0	0	0	0	
4.2.1.83	1	0	0	0	0	0	0	1	1	0	0	0	1	0	0	0	0	2	
4.2.1.84	1	0	0	0	0	0	0	0	1	1	0	1	0	0	0	0	0	1	
5.1.2.2	0	0	0	0	0	0	0	0	0	0	0	0	0	0	0	0	0	0	
5.2.1.2	0	0	0	0	0	0	0	0	0	0	0	0	0	0	0	0	0	0	
5.3.3.4	1	0	0	0	0	0	0	0	0	0	0	0	0	0	2	0	0	1	
5.3.99.-	5	9	0	2	0	5	1	4	4	5	3	12	4	3	6	5	4	3	
5.4.99.-	1	1	0	0	0	1	1	1	0	0	0	2	2	1	0	0	0	0	
5.5.1.1	4	4	3	2	0	3	4	0	6	5	1	8	4	5	7	4	4	3	
5.5.1.2	0	0	0	0	0	0	0	0	0	0	0	0	0	0	0	0	0	0	
6.2.1.-	0	0	0	0	0	0	0	0	0	0	0	0	0	0	0	0	0	0	
6.3.5.2	28	35	8	11	8	15	8	16	18	36	12	33	32	10	26	31	33	30	
K00002	2	6	1	0	1	1	0	1	1	0	2	4	5	1	4	3	2	2	
K00055	0	0	1	0	0	0	0	0	0	0	0	0	0	0	0	0	0	0	
K00074	8	8	1	1	2	2	3	2	6	5	3	11	4	2	8	4	11	7	
K00088	20	31	5	11	4	13	12	21	13	20	5	29	16	11	25	24	21	28	
K00100	77	100	25	41	21	54	39	41	48	55	24	95	80	37	89	72	93	79	
K00128	3	3	0	3	1	0	1	1	2	1	1	6	3	1	0	4	5	1	
K00129	0	0	0	0	0	0	0	0	0	0	0	0	0	0	0	0	0	0	
K00132	2	0	0	1	0	0	0	1	0	0	0	1	0	0	0	0	0	0	
K00141	0	0	0	0	0	0	0	0	0	0	0	0	0	0	0	0	0	0	
K00146	0	0	0	0	0	0	0	0	0	0	0	0	0	0	0	0	0	0	
K00148	0	0	0	0	0	0	0	0	0	0	0	0	0	0	0	0	0	0	
K00155	1	1	1	0	0	1	1	1	0	0	0	0	0	1	1	0	1	2	
K00169	1	7	2	0	1	1	1	3	2	4	0	2	3	0	3	1	2	7	
K00224	1	2	1	0	0	2	2	1	0	0	0	2	0	0	0	2	1	2	
K00274	0	0	0	0	0	0	0	0	0	0	0	0	0	0	0	0	0	0	
K00446	0	0	0	0	0	0	0	0	0	0	0	0	0	0	0	0	0	0	
K00448	0	0	0	0	0	0	0	0	0	0	0	0	0	0	0	0	0	0	
K00462	1	1	2	1	0	0	0	0	2	1	1	2	3	1	2	3	3	1	
K00480	0	0	0	0	0	0	0	0	0	0	0	0	0	0	0	0	0	0	
K00481	0	0	0	0	0	0	0	0	0	0	0	0	0	0	0	0	0	0	
K00539	0	6	1	0	0	1	1	0	0	0	0	3	0	0	2	1	0	1	
K00599	57	61	16	30	18	36	20	34	56	32	14	81	52	24	79	38	59	76	
K00626	3	3	1	3	1	5	0	2	0	3	1	6	2	2	4	5	4	9	
K00632	0	0	0	0	0	0	0	0	0	0	0	0	0	0	0	0	0	0	
K00680	67	77	11	33	17	59	41	31	41	53	12	83	70	40	56	43	60	47	
K00757	17	22	8	13	4	10	6	11	12	13	9	24	23	10	27	14	19	22	
K00758	6	13	1	2	1	1	0	4	5	1	0	7	2	3	4	6	3	2	
K00760	33	40	8	17	14	27	16	17	33	23	12	52	27	17	28	26	33	36	
K00799	0	2	0	0	0	0	0	0	0	0	0	1	0	1	3	0	0	1	
K00857	20	21	4	13	7	12	13	11	18	19	5	30	17	14	17	17	20	16	
K00876	38	44	9	27	15	24	20	33	46	33	18	64	47	23	47	36	43	48	
K01026	1	2	0	0	0	1	0	0	0	3	0	1	1	0	2	0	1	1	
K01031	0	0	0	0	0	0	0	0	0	0	0	0	0	0	0	0	0	0	

EC/KO	AM-AD-62	AM-AD-63	AM-AD-64	AM-AD-65	AM-AD-66	AM-AD-67	AM-AD-68	AM-AD-69	AM-AD-7	AM-AD-70	AM-AD-71	AM-AD-72	AM-AD-73	AM-AD-74	AM-AD-75	AM-AD-76	AM-AD-77	AM-AD-78
K01034	2	5	0	2	1	1	1	1	2	1	1	4	4	0	3	1	1	3
K01039	0	0	0	0	1	2	1	1	0	0	1	0	1	1	1	0	1	0
K01041	6	5	1	3	2	4	2	8	5	3	2	10	11	1	7	8	10	9
K01053	0	0	0	0	0	0	0	0	0	0	0	0	1	0	0	0	0	0
K01055	0	0	0	0	0	0	0	0	0	0	0	0	0	0	0	0	0	0
K01061	0	0	0	0	0	0	0	0	0	0	0	0	0	0	0	1	0	0
K01066	10	16	1	3	2	7	3	3	7	7	4	12	16	9	11	10	9	10
K01075	2	7	1	0	0	3	1	1	1	0	0	3	0	1	3	1	0	2
K01077	17	16	4	8	5	14	7	13	15	11	5	10	22	13	13	16	18	14
K01101	3	6	1	3	1	2	3	3	2	2	0	13	5	2	8	7	9	3
K01195	17	12	3	10	5	5	8	6	6	9	5	13	10	7	14	13	18	10
K01426	1	1	1	2	0	2	1	1	1	0	0	2	1	0	3	0	2	2
K01428	2	2	0	0	1	2	0	1	0	3	0	0	1	1	2	0	2	3
K01457	0	0	0	0	0	0	0	0	0	0	0	2	2	0	0	0	0	0
K01464	0	3	0	1	0	2	1	1	1	1	0	4	5	0	7	2	0	2
K01489	25	43	3	17	9	15	14	16	30	18	14	40	34	14	25	32	29	24
K01500	1	0	0	0	0	0	0	0	0	0	0	0	0	0	0	0	0	0
K01501	0	2	0	0	0	1	0	1	0	4	0	4	1	2	2	2	0	2
K01502	0	0	0	0	0	0	0	0	0	0	0	0	0	0	0	0	0	0
K01512	6	10	3	4	4	3	2	2	7	6	6	14	5	5	21	10	4	12
K01560	7	7	0	2	2	4	3	3	7	6	1	13	7	1	10	6	5	12
K01561	0	2	0	1	0	0	0	0	0	0	0	0	0	0	1	0	1	0
K01563	0	0	0	0	0	0	0	0	0	0	0	0	0	0	0	0	0	0
K01564	3	5	0	3	1	3	1	2	2	5	0	1	1	1	3	1	4	4
K01607	23	36	5	18	11	16	19	11	23	16	10	18	25	10	19	16	23	23
K01612	0	0	0	0	0	0	0	0	0	1	0	0	0	0	0	0	0	0
K01615	15	15	5	6	6	9	9	12	15	21	7	25	27	12	15	20	21	26
K01617	0	0	0	0	0	0	0	0	0	0	0	0	0	0	0	0	0	0
K01666	10	5	1	3	2	2	1	6	7	1	4	8	5	0	6	6	10	12
K01692	0	3	0	0	0	0	0	0	0	0	0	1	0	1	1	0	0	1
K01721	0	0	0	0	0	0	0	0	0	0	0	0	0	0	0	0	0	0
K01726	17	13	5	9	4	9	10	9	17	12	3	17	15	8	7	8	13	8
K01781	1	2	2	1	0	0	2	0	0	1	0	1	0	0	3	0	1	0
K01821	2	4	1	1	1	0	0	1	2	2	0	3	2	1	3	0	4	2
K01856	0	0	0	0	0	0	0	0	0	0	0	0	0	0	0	0	0	0
K01857	0	1	0	0	0	0	2	0	0	0	0	0	0	0	0	1	0	1
K01913	0	0	0	0	0	0	0	0	0	0	0	0	0	0	0	0	1	0
K01951	18	21	5	10	4	12	6	10	11	29	5	21	21	9	22	21	17	20
K02554	0	0	0	0	0	0	0	0	0	0	0	0	0	0	0	0	0	0
K03381	0	0	0	0	0	0	0	0	0	0	0	0	0	0	0	0	0	0
K03382	0	1	1	0	0	1	0	0	0	0	0	2	2	0	0	1	0	1
K03464	0	0	0	0	0	0	0	0	0	0	0	0	0	0	0	0	0	0
K03518	11	13	4	3	6	5	2	4	9	10	4	13	10	1	25	12	11	9
K03862	0	0	0	0	0	0	0	0	0	0	0	0	0	0	0	0	0	0
K04099	0	0	0	0	0	0	0	0	0	0	0	0	0	0	0	0	0	0
K04100	0	0	0	0	0	0	0	0	0	0	0	0	0	1	0	0	0	0
K04102	0	0	0	0	0	0	0	0	0	0	0	0	0	0	0	0	0	0
K04116	0	0	0	0	0	0	0	0	0	0	0	0	0	0	0	0	0	0
K05394	1	1	0	0	0	3	2	0	0	1	1	2	2	0	1	3	2	1

EC/KO	AM-AD-62	AM-AD-63	AM-AD-64	AM-AD-65	AM-AD-66	AM-AD-67	AM-AD-68	AM-AD-69	AM-AD-7	AM-AD-70	AM-AD-71	AM-AD-72	AM-AD-73	AM-AD-74	AM-AD-75	AM-AD-76	AM-AD-77	AM-AD-78	
**K05549**	0	0	0	0	0	0	0	0	0	0	0	0	0	0	0	0	0	0	
**K05783**	0	0	0	0	0	0	0	0	0	0	0	0	0	0	0	0	0	0	
**K05797**	0	0	0	0	0	0	0	0	0	0	0	0	0	0	0	0	0	0	
**K06281**	4	3	1	4	0	2	2	0	0	4	1	3	1	2	2	5	4	2	
**K06446**	3	5	2	2	2	1	2	3	1	6	2	4	3	1	4	2	6	5	
**K06912**	0	0	0	0	0	0	0	0	0	0	0	0	0	0	0	0	0	0	
**K07535**	0	0	0	0	0	0	0	0	0	0	0	0	1	0	0	0	0	0	
**K07536**	7	2	2	4	1	1	3	2	5	4	1	6	1	0	5	1	0	2	
**K08689**	0	0	0	0	0	0	0	0	0	0	0	0	0	0	0	0	0	0	
**K08710**	1	0	0	0	0	0	0	0	0	0	0	0	0	0	0	0	0	0	
**K09461**	0	0	0	0	0	0	0	0	0	0	0	0	0	0	0	0	0	0	
**K10217**	0	0	0	0	0	0	0	0	0	0	0	0	0	0	0	0	0	0	
**K10218**	0	2	1	1	0	1	1	0	0	1	0	1	0	0	1	0	0	0	
**K10220**	0	0	0	0	0	0	0	0	0	0	0	0	0	0	0	0	0	0	
**K11180**	0	0	0	0	0	0	0	0	0	0	0	0	0	0	0	0	0	0	
**K13953**	0	0	0	0	0	0	0	0	0	0	0	0	0	0	0	0	0	0	
**K14333**	0	0	0	0	0	0	0	0	0	0	0	0	0	0	0	0	0	0	
**K14519**	0	0	0	0	0	0	0	0	0	0	0	0	0	0	0	0	0	0	
**K15054**	0	0	0	0	0	0	0	0	0	0	0	0	0	0	0	0	0	0	
**K16173**	1	1	0	0	0	0	0	0	0	0	0	0	0	1	2	0	1	0	
**K16514**	0	0	0	0	0	0	0	0	0	0	0	1	0	0	0	0	0	0	
**K16874**	0	0	0	0	0	0	0	0	0	0	0	1	1	0	0	0	0	0	

EC/KO	AM-AD-79	AM-AD-8	AM-AD-80	AM-AD-81	AM-AD-82	AM-AD-83	AM-AD-84	AM-AD-85	AM-AD-86	AM-AD-87	AM-AD-88	AM-AD-89	AM-AD-9	AM-AD-90	AM-AD-91	AM-AD-92	CH-DLF001	CH-DLF002
**1.1.1.-**	50	19	102	66	56	55	56	23	39	71	37	59	17	49	22	88	27	26
**1.1.1.1**	43	16	50	39	30	25	26	11	21	32	19	40	7	28	10	45	10	15
**1.1.1.157**	1	0	1	3	3	3	2	0	1	0	0	2	0	1	0	3	0	0
**1.1.1.205**	21	10	41	23	18	20	25	10	12	28	8	25	8	17	12	25	9	10
**1.1.1.35**	2	0	7	2	5	1	4	0	3	2	0	5	0	2	0	3	1	0
**1.12.99.6**	2	3	3	2	0	0	3	0	1	4	1	1	1	4	1	3	3	0
**1.13.11.-**	0	0	0	0	0	0	0	0	0	0	0	0	0	0	0	0	0	0
**1.13.11.1**	0	0	0	0	0	0	0	0	0	0	0	0	0	0	0	0	0	0
**1.13.11.2**	0	0	0	0	0	0	0	0	0	0	0	0	0	0	0	0	0	0
**1.13.11.3**	0	0	0	0	0	0	0	0	0	0	0	0	0	0	0	0	0	0
**1.13.11.39**	0	0	0	0	0	0	0	0	0	0	0	0	0	0	0	0	0	0
**1.13.11.5**	0	0	0	0	0	0	2	0	0	0	0	0	0	0	0	0	0	0
**1.13.11.8**	0	0	0	0	0	0	0	0	0	0	0	0	0	0	0	0	0	0
**1.14.12.10**	1	0	0	0	0	0	0	0	0	0	0	0	0	0	0	0	0	0
**1.14.12.13**	0	0	0	0	0	0	0	0	0	0	0	0	0	0	0	0	0	0
**1.14.12.18**	0	0	0	0	0	0	0	0	0	0	0	0	0	0	0	0	0	0
**1.14.13.-**	1	0	0	0	0	0	0	0	0	0	0	0	0	0	0	0	0	0
**1.14.13.1**	0	0	0	0	0	0	0	0	0	0	0	0	0	0	0	0	0	0
**1.14.13.2**	0	0	0	0	0	0	0	0	0	0	0	0	0	0	0	0	0	0
**1.14.13.50**	0	0	0	0	0	0	0	0	0	0	0	0	0	0	0	0	0	0
**1.14.13.7**	0	0	0	0	0	1	0	0	0	0	0	0	0	0	0	0	0	0
**1.14.13.8**	0	0	0	0	0	0	0	0	0	0	0	0	0	0	0	0	0	0
**1.14.13.82**	0	0	0	0	0	0	1	0	0	0	0	0	0	0	0	0	0	0
**1.14.99.-**	0	0	0	0	0	0	0	0	0	0	0	0	0	0	0	0	0	0
**1.17.99.1**	0	0	0	0	0	0	0	0	0	0	0	0	0	0	0	0	0	0
**1.18.6.1**	0	0	0	0	1	0	0	0	0	0	0	0	0	0	0	2	0	0
**1.2.1.-**	0	0	0	0	0	0	0	0	0	0	0	0	0	0	0	0	0	0
**1.2.1.10**	6	3	13	5	6	1	5	2	7	1	0	5	0	2	1	11	1	3
**1.2.1.3**	11	4	20	6	8	5	10	7	3	9	2	6	5	8	4	11	3	2
**1.2.1.39**	0	0	0	0	0	0	0	0	0	0	0	0	0	0	0	0	0	0
**1.2.7.1**	9	5	28	10	8	5	14	1	5	6	5	17	4	7	4	18	4	3
**1.2.99.2**	2	1	6	2	1	0	1	3	2	1	1	3	1	1	0	5	0	2
**1.3.1.-**	0	0	0	0	0	1	1	0	0	1	0	0	0	0	1	0	0	0
**1.3.1.2**	2	0	3	1	0	1	2	0	1	2	1	0	0	1	1	1	0	0
**1.3.1.25**	0	0	0	0	0	0	0	0	0	0	0	0	0	0	0	0	0	0
**1.3.99.-**	0	0	1	0	0	0	0	0	0	0	0	0	0	0	0	1	0	0
**1.6.5.-**	39	16	84	52	33	51	64	10	41	55	44	42	17	43	24	77	18	19
**1.7.1.-**	10	5	18	7	4	6	11	2	5	11	8	6	5	10	4	13	6	7
**1.8.99.3**	0	0	0	0	0	0	1	0	0	1	0	0	0	0	0	0	0	0
**2.1.1.-**	259	101	502	267	234	225	258	72	150	271	133	304	71	179	84	403	62	102
**2.3.1.-**	141	61	311	173	122	164	211	64	81	212	98	186	51	147	62	264	70	88
**2.3.1.16**	1	1	1	2	0	2	4	0	0	0	0	2	0	0	0	1	1	0
**2.3.1.5**	0	0	0	0	0	0	0	0	0	0	0	0	0	0	0	0	0	0
**2.3.1.9**	0	0	2	0	3	0	2	1	3	1	0	1	0	0	0	2	0	2
**2.4.2.10**	15	4	20	14	16	13	15	2	7	14	6	15	5	11	5	22	3	5
**2.4.2.3**	7	3	19	8	14	3	6	3	3	4	1	8	2	5	2	10	0	2
**2.4.2.4**	1	2	8	1	2	2	0	0	3	0	1	10	0	0	0	5	0	1
**2.4.2.8**	11	6	28	13	11	11	13	2	9	17	10	13	4	10	4	26	4	3
**2.5.1.-**	18	10	40	20	13	25	26	3	10	32	15	25	7	16	10	23	12	9

EC/KO	AM-AD-79	AM-AD-8	AM-AD-80	AM-AD-81	AM-AD-82	AM-AD-83	AM-AD-84	AM-AD-85	AM-AD-86	AM-AD-87	AM-AD-88	AM-AD-89	AM-AD-9	AM-AD-90	AM-AD-91	AM-AD-92	CH-DLF001	CH-DLF002
**2.5.1.18**	2	0	2	1	1	0	2	0	1	0	0	0	0	0	0	2	0	0
**2.6.1.-**	47	14	67	26	23	28	36	10	19	39	20	27	8	23	12	49	17	23
**2.7.1.21**	5	1	14	8	3	9	8	2	5	10	5	8	2	6	1	13	2	4
**2.7.1.48**	35	13	64	28	17	33	35	10	15	40	15	31	7	28	13	50	9	12
**2.7.4.-**	15	8	34	16	15	16	12	6	7	18	6	23	5	11	5	27	5	7
**2.8.3.-**	1	2	1	2	2	0	4	0	1	2	0	1	0	0	1	2	2	0
**2.8.3.1**	0	0	0	0	1	1	0	0	1	0	0	0	0	0	0	0	0	0
**2.8.3.12**	0	0	1	0	1	0	2	0	2	0	0	0	0	0	0	0	0	0
**2.8.3.6**	0	0	1	1	1	0	1	0	0	1	0	0	0	0	1	0	1	0
**2.8.3.8**	1	0	4	1	3	1	1	1	0	1	0	1	1	2	1	4	0	1
**3.1.1.-**	3	8	10	3	6	4	4	2	3	6	4	8	3	3	1	9	3	1
**3.1.1.1**	5	2	9	9	5	7	8	0	8	10	6	10	3	6	5	8	4	3
**3.1.1.17**	2	0	1	1	1	1	3	0	2	3	2	0	0	2	2	2	1	2
**3.1.1.2**	0	1	2	0	0	0	0	0	0	1	0	0	0	0	0	0	1	0
**3.1.1.24**	2	0	5	3	2	2	3	0	2	3	1	1	0	0	1	2	0	0
**3.1.1.45**	0	0	0	0	0	0	0	0	0	0	0	0	0	0	0	0	0	0
**3.1.2.-**	0	0	1	0	2	1	1	0	1	0	0	0	0	0	0	1	0	0
**3.1.2.23**	6	2	12	6	3	8	11	2	3	13	4	5	3	9	5	8	2	3
**3.1.3.1**	17	6	22	23	6	16	29	6	11	28	17	16	6	20	17	19	15	11
**3.1.3.2**	2	1	7	4	3	8	6	4	2	9	3	5	4	3	5	4	3	3
**3.1.3.41**	0	0	0	0	0	0	0	0	0	0	0	0	0	0	0	0	0	0
**3.2.1.31**	4	1	6	0	6	1	1	0	4	2	1	7	0	1	0	9	0	2
**3.3.2.9**	0	0	0	0	0	0	0	0	0	0	0	0	0	0	0	0	0	0
**3.5.1.-**	23	7	48	13	14	16	19	5	14	21	11	32	7	15	6	41	3	6
**3.5.1.4**	4	1	3	1	1	2	0	0	0	2	1	5	1	2	1	4	0	0
**3.5.1.5**	2	0	5	1	2	1	5	0	0	1	1	1	1	3	2	6	0	0
**3.5.1.54**	4	0	3	3	1	2	0	0	0	5	1	3	0	0	0	4	0	0
**3.5.1.6**	1	1	2	0	3	1	2	0	1	3	0	1	1	0	0	1	0	0
**3.5.2.-**	0	0	0	0	0	0	0	0	0	0	0	0	0	0	0	0	0	0
**3.5.2.2**	1	1	2	0	0	2	1	1	0	0	0	1	0	0	0	0	0	0
**3.5.4.-**	20	11	47	22	14	19	18	6	12	34	9	35	11	25	5	36	7	5
**3.5.4.5**	14	7	30	13	9	18	19	2	11	17	8	19	5	9	6	15	5	4
**3.5.5.1**	0	1	1	1	1	0	0	0	1	2	0	1	0	0	1	2	1	0
**3.5.5.7**	0	0	0	0	0	0	0	0	0	0	0	0	0	0	0	0	0	0
**3.5.99.3**	0	1	0	0	0	0	0	0	0	0	0	0	0	0	0	1	0	0
**3.6.1.7**	4	2	9	5	7	4	1	3	3	2	2	6	2	2	2	5	1	0
**3.7.1.-**	0	1	1	1	1	1	2	0	0	1	0	1	0	2	0	3	1	0
**3.7.1.2**	2	3	10	5	3	4	7	2	4	8	6	5	4	6	2	6	4	2
**3.8.1.2**	5	2	15	5	2	7	9	3	5	12	5	6	3	9	5	9	5	4
**3.8.1.3**	0	0	0	0	0	0	0	0	0	0	0	0	0	0	0	1	0	0
**3.8.1.5**	0	0	0	0	0	0	0	0	0	0	0	0	0	0	0	0	0	0
**4.1.1.-**	12	7	30	11	7	14	13	2	4	16	9	7	5	12	7	13	7	5
**4.1.1.44**	24	13	54	26	22	24	39	8	11	40	13	28	7	30	10	49	14	8
**4.1.1.55**	0	0	0	0	0	0	0	0	0	0	0	0	0	0	0	0	0	0
**4.1.1.7**	0	0	0	0	0	0	0	0	0	0	0	0	0	0	0	0	0	0
**4.1.1.70**	1	0	0	1	0	1	0	0	0	1	0	0	0	0	0	0	0	0
**4.1.1.77**	0	0	0	0	0	0	0	0	0	0	0	0	0	0	0	0	0	0
**4.1.2.-**	8	3	5	1	5	3	8	3	4	7	0	8	0	4	1	11	3	3
**4.1.3.-**	21	4	29	20	16	16	17	6	11	17	6	11	6	15	7	30	6	6

EC/KO	AM-AD-79	AM-AD-8	AM-AD-80	AM-AD-81	AM-AD-82	AM-AD-83	AM-AD-84	AM-AD-85	AM-AD-86	AM-AD-87	AM-AD-88	AM-AD-89	AM-AD-9	AM-AD-90	AM-AD-91	AM-AD-92	CH-DLF001	CH-DLF002
**4.1.3.39**	0	0	0	0	0	0	3	0	0	0	0	0	0	0	1	0	0	1
**4.1.99.-**	2	0	3	2	2	1	2	0	2	0	2	1	0	2	0	2	0	0
**4.2.1.-**	58	18	103	67	40	49	88	18	42	62	39	65	15	44	27	83	25	24
**4.2.1.17**	3	2	8	7	8	1	4	1	2	4	2	6	2	2	1	5	2	3
**4.2.1.80**	0	0	0	0	0	0	0	0	0	0	0	0	0	0	0	0	0	0
**4.2.1.83**	1	0	3	0	0	0	0	0	0	0	0	1	0	1	0	1	0	0
**4.2.1.84**	0	0	0	0	0	0	0	0	0	0	0	0	0	0	1	2	0	0
**5.1.2.2**	0	0	0	0	0	0	0	0	0	0	0	0	0	0	0	0	0	0
**5.2.1.2**	0	0	0	0	0	0	0	0	0	0	0	0	0	0	0	0	0	0
**5.3.3.4**	0	0	0	0	0	0	0	0	0	0	0	0	0	0	0	0	0	0
**5.3.99.-**	0	1	5	0	4	4	4	1	1	3	3	2	0	3	0	8	3	0
**5.4.99.-**	0	0	1	0	0	1	1	0	0	0	0	0	0	1	0	2	0	0
**5.5.1.1**	2	4	6	4	3	8	6	1	2	6	4	4	1	4	2	5	3	3
**5.5.1.2**	0	0	0	0	0	0	0	0	0	0	0	0	0	0	0	0	0	0
**6.2.1.-**	0	0	0	0	0	0	0	0	0	0	0	0	0	0	0	0	0	0
**6.3.5.2**	25	12	39	25	14	14	19	5	14	22	13	17	5	15	7	29	9	9
**K00002**	2	1	2	1	0	0	4	0	0	3	0	2	0	0	1	0	0	0
**K00055**	0	0	0	0	0	0	0	0	0	0	0	0	0	0	0	0	0	0
**K00074**	6	0	9	6	12	3	7	0	6	4	0	9	0	5	0	10	1	1
**K00088**	10	9	33	22	16	16	20	6	11	23	6	18	5	7	9	20	10	9
**K00100**	57	22	99	58	45	46	56	27	31	73	36	55	18	50	18	84	11	18
**K00128**	2	1	3	0	2	0	0	2	1	1	0	1	0	2	0	1	0	0
**K00129**	0	0	0	0	0	0	0	0	0	0	0	0	0	0	0	0	0	0
**K00132**	0	0	0	0	0	0	0	0	0	0	0	2	0	0	0	0	0	0
**K00141**	0	0	0	0	0	0	0	0	0	0	0	0	0	0	0	0	0	0
**K00146**	0	0	0	0	0	0	0	0	0	0	0	0	0	0	0	0	0	0
**K00148**	0	0	0	0	0	0	0	0	0	0	0	0	0	0	0	0	0	0
**K00155**	1	0	1	0	0	0	0	1	0	0	0	0	1	1	0	0	0	0
**K00169**	3	0	2	1	2	0	3	1	1	1	1	2	1	0	0	3	0	1
**K00224**	1	1	1	0	1	0	1	1	0	1	0	1	0	1	0	2	0	0
**K00274**	0	0	0	0	0	0	0	0	0	0	0	0	0	0	0	0	0	0
**K00446**	0	0	0	0	0	0	0	0	0	0	0	0	0	0	0	0	0	0
**K00448**	0	0	0	0	0	0	0	0	0	0	0	0	0	0	0	0	0	0
**K00462**	1	2	1	0	1	0	1	0	0	2	2	2	2	1	1	0	1	1
**K00480**	0	0	0	0	0	0	0	0	0	0	0	0	0	0	0	0	0	0
**K00481**	0	0	0	0	0	0	0	0	0	0	0	0	0	0	0	0	0	0
**K00539**	0	1	1	1	1	0	0	0	0	1	0	1	1	1	0	2	0	0
**K00599**	51	14	88	35	30	38	39	10	33	41	18	40	12	28	10	69	10	10
**K00626**	4	1	3	4	4	3	6	1	3	2	0	5	0	0	0	6	1	3
**K00632**	0	0	0	0	0	0	0	0	0	0	0	0	0	0	0	0	0	0
**K00680**	41	17	73	48	33	41	49	10	27	46	27	53	13	45	16	63	25	17
**K00757**	11	4	29	15	11	13	14	4	9	18	5	15	3	12	10	15	3	4
**K00758**	3	0	4	1	3	1	3	0	0	0	0	3	0	0	0	7	0	0
**K00760**	21	6	44	20	19	19	22	5	13	28	16	23	7	22	6	41	4	6
**K00799**	0	0	0	0	0	0	0	0	0	0	0	1	0	0	0	0	0	0
**K00857**	8	1	22	13	7	13	14	3	7	16	7	15	4	9	2	16	3	5
**K00876**	25	8	58	30	22	28	32	5	15	37	15	32	7	19	12	40	9	12
**K01026**	0	0	1	2	2	1	0	0	0	1	0	1	0	0	0	2	0	0
**K01031**	0	0	0	0	0	0	0	0	0	0	0	0	0	0	0	0	0	0

EC/KO	AM-AD-79	AM-AD-8	AM-AD-80	AM-AD-81	AM-AD-82	AM-AD-83	AM-AD-84	AM-AD-85	AM-AD-86	AM-AD-87	AM-AD-88	AM-AD-89	AM-AD-9	AM-AD-90	AM-AD-91	AM-AD-92	CH-DLF001	CH-DLF002
K01034	0	0	2	1	1	0	2	0	0	1	1	2	0	2	0	1	0	1
K01039	0	0	0	0	0	0	0	0	0	0	0	1	0	0	0	2	0	0
K01041	4	2	9	5	2	5	9	2	3	5	7	8	2	6	6	2	5	4
K01053	0	0	0	0	0	0	0	0	0	0	0	0	0	0	0	0	0	0
K01055	0	0	0	0	0	0	0	0	0	0	0	0	0	0	0	0	0	0
K01061	0	0	0	0	0	0	0	0	0	0	0	0	0	0	0	0	0	0
K01066	5	2	14	6	3	7	10	1	3	12	3	11	2	5	3	9	5	3
K01075	0	2	1	0	2	0	1	0	0	1	0	1	1	1	0	1	0	1
K01077	8	2	15	14	5	12	18	4	7	20	10	12	2	6	10	11	7	7
K01101	2	2	7	3	3	2	6	0	0	4	0	3	1	1	0	5	0	0
K01195	9	4	15	6	10	10	12	1	6	10	6	8	2	5	4	13	4	3
K01426	1	1	1	1	0	2	1	1	0	0	0	1	1	0	0	1	0	0
K01428	1	1	4	2	0	2	0	0	0	1	0	3	0	1	0	4	1	0
K01457	0	0	0	0	0	0	0	0	0	0	0	0	0	0	0	0	0	0
K01464	1	1	1	1	2	4	1	1	0	1	0	2	0	0	0	0	0	0
K01489	15	7	39	18	13	20	19	2	12	26	10	23	5	10	6	23	7	5
K01500	0	0	0	0	0	0	0	0	0	0	0	0	0	0	0	0	0	0
K01501	1	1	1	3	2	1	1	0	1	1	0	2	0	0	0	1	0	0
K01502	0	0	0	0	0	0	0	0	0	0	0	0	0	0	0	0	0	0
K01512	7	2	14	7	11	6	2	4	6	4	4	8	2	8	3	12	1	1
K01560	3	1	10	4	9	4	2	0	3	4	0	6	0	1	0	8	1	1
K01561	0	1	0	0	0	0	1	0	0	0	0	0	0	0	0	0	0	0
K01563	0	0	0	0	0	0	0	0	0	0	0	0	0	0	0	0	0	0
K01564	1	2	3	2	1	4	1	0	0	5	1	2	0	3	0	5	1	0
K01607	10	4	30	17	6	14	20	6	5	20	9	20	4	16	8	27	4	3
K01612	0	0	0	0	0	0	0	0	0	0	0	0	0	0	0	0	0	0
K01615	12	4	26	18	15	20	13	1	13	13	10	18	6	8	10	16	9	7
K01617	0	0	0	0	0	0	0	0	0	0	0	0	0	0	0	0	0	0
K01666	4	0	13	5	5	3	6	0	5	3	1	4	1	5	1	9	1	4
K01692	0	0	0	0	0	0	0	0	0	0	0	1	0	0	0	0	0	0
K01721	0	0	0	0	0	0	0	0	0	0	0	0	0	0	0	0	0	0
K01726	9	3	11	9	4	11	15	3	3	11	5	8	5	7	5	14	7	5
K01781	1	0	0	2	2	0	1	0	0	0	0	1	1	1	0	0	0	1
K01821	0	1	1	1	3	4	2	0	0	1	0	2	0	2	0	2	0	0
K01856	0	0	0	0	0	0	0	0	0	0	0	0	0	0	0	0	0	0
K01857	0	1	1	0	0	0	0	1	0	1	0	0	0	1	0	0	0	0
K01913	0	0	0	1	0	0	1	0	0	0	0	1	0	0	0	1	0	0
K01951	16	9	28	23	14	12	16	4	8	16	10	14	4	12	7	13	7	7
K02554	0	0	0	0	0	0	0	0	0	0	0	0	0	0	0	0	0	0
K03381	0	0	0	0	0	0	0	0	0	0	0	0	0	0	0	0	0	0
K03382	0	1	3	0	0	1	0	0	0	1	0	2	0	0	0	1	0	0
K03464	0	0	0	0	0	0	0	0	0	0	0	0	0	0	0	0	0	0
K03518	7	4	12	11	13	8	4	2	6	5	2	12	0	2	1	13	1	3
K03862	0	0	0	0	0	0	0	0	0	0	0	0	0	0	0	0	0	0
K04099	0	0	0	0	0	0	0	0	0	0	0	0	0	0	0	0	0	0
K04100	0	0	0	0	0	0	0	0	0	0	0	0	0	0	0	0	0	0
K04102	0	0	0	0	0	0	0	0	0	0	0	0	0	0	0	0	0	0
K04116	0	0	0	0	0	0	0	0	0	0	0	0	0	0	0	0	0	0
K05394	1	1	1	1	1	0	0	1	0	1	0	0	1	1	1	0	0	0

EC/KO	AM-AD-79	AM-AD-8	AM-AD-80	AM-AD-81	AM-AD-82	AM-AD-83	AM-AD-84	AM-AD-85	AM-AD-86	AM-AD-87	AM-AD-88	AM-AD-89	AM-AD-9	AM-AD-90	AM-AD-91	AM-AD-92	CH-DLF001	CH-DLF002
**K05549**	0	0	0	0	0	0	0	0	0	0	0	0	0	0	0	0	0	0
**K05783**	0	0	0	0	0	0	0	0	0	0	0	0	0	0	0	0	0	0
**K05797**	0	0	0	0	0	0	0	0	0	0	0	0	0	0	0	0	0	0
**K06281**	3	2	3	2	1	0	3	1	1	3	1	1	1	3	1	3	1	0
**K06446**	7	0	5	4	2	6	5	0	1	4	3	4	0	0	2	8	0	2
**K06912**	0	0	0	0	0	0	0	0	0	0	0	0	0	0	0	0	0	0
**K07535**	0	0	0	0	0	0	1	0	0	0	0	0	0	0	0	0	0	0
**K07536**	3	1	4	1	2	3	4	0	2	4	1	1	1	5	2	3	1	2
**K08689**	0	0	0	0	0	0	0	0	0	0	0	0	0	0	0	0	0	0
**K08710**	1	0	0	0	0	0	0	0	0	0	0	0	0	0	0	0	0	0
**K09461**	0	0	0	0	0	0	0	0	0	0	0	0	0	0	0	0	0	0
**K10217**	0	0	0	0	0	0	0	0	0	0	0	0	0	0	0	0	0	0
**K10218**	0	0	0	1	1	0	0	0	1	0	0	0	0	0	0	0	0	0
**K10220**	0	0	0	0	0	0	0	0	0	0	0	0	0	0	0	0	0	0
**K11180**	0	0	0	0	0	0	0	0	0	0	0	0	0	0	0	0	0	0
**K13953**	0	0	0	0	1	1	0	0	0	0	0	0	0	0	0	0	0	0
**K14333**	0	0	0	0	0	0	0	0	0	0	0	0	0	0	0	0	0	0
**K14519**	0	0	0	0	0	0	0	0	0	0	0	0	0	0	0	0	0	0
**K15054**	0	0	0	0	0	0	0	0	0	0	0	0	0	0	0	0	0	0
**K16173**	0	0	0	0	0	0	0	0	0	1	0	0	0	0	0	1	0	0
**K16514**	1	1	0	0	0	0	0	0	0	0	0	0	0	0	0	0	0	0
**K16874**	0	0	0	0	0	0	0	0	0	0	0	0	0	0	0	0	0	0

EC/KO	CH-DLF003	CH-DLF004	CH-DLF005	CH-DLF006	CH-DLF007	CH-DLF008	CH-DLF009	CH-DLF010	CH-DLF012	CH-DLF013	CH-DLF014	CH-DLM001	CH-DLM002	CH-DLM003	CH-DLM004	CH-DLM005
**1.1.1.-**	14	39	24	37	26	47	24	30	37	38	49	59	35	26	27	37
**1.1.1.1**	20	24	42	22	12	30	16	31	36	28	41	46	27	13	12	22
**1.1.1.157**	0	0	1	1	0	0	0	2	0	1	0	1	0	2	0	1
**1.1.1.205**	12	16	19	10	9	9	7	17	11	12	17	33	20	17	14	12
**1.1.1.35**	0	0	1	0	1	1	4	2	0	2	2	3	1	0	0	1
**1.12.99.6**	0	0	1	4	1	3	2	0	2	0	0	4	1	1	1	3
**1.13.11.-**	0	0	0	0	0	0	0	0	0	0	0	0	0	0	0	0
**1.13.11.1**	0	0	0	0	0	0	0	0	0	0	0	0	0	0	0	0
**1.13.11.2**	0	0	0	0	0	0	0	0	0	0	0	0	0	0	0	0
**1.13.11.3**	0	0	0	0	0	0	0	2	0	0	0	0	0	0	0	0
**1.13.11.39**	0	0	0	0	0	0	0	0	1	0	0	0	0	0	0	0
**1.13.11.5**	0	0	0	0	0	0	0	0	0	0	0	0	0	0	0	0
**1.13.11.8**	0	0	0	0	0	0	0	1	0	0	0	0	0	0	0	0
**1.14.12.10**	0	0	0	0	0	1	1	1	1	1	0	0	0	0	0	1
**1.14.12.13**	0	0	0	0	0	0	0	0	0	0	0	0	0	0	0	0
**1.14.12.18**	0	0	0	0	0	0	0	0	0	0	0	0	0	0	0	0
**1.14.13.-**	0	0	0	0	0	3	1	5	3	0	0	0	0	0	0	4
**1.14.13.1**	0	0	0	0	0	0	0	0	0	0	0	0	0	0	0	0
**1.14.13.2**	0	0	0	0	0	0	0	1	0	0	0	0	0	0	0	0
**1.14.13.50**	0	0	0	0	0	0	0	0	0	0	0	0	0	0	0	0
**1.14.13.7**	0	1	0	0	1	0	0	0	0	0	0	0	0	0	0	0
**1.14.13.8**	0	0	0	0	0	0	0	0	0	0	0	0	0	0	0	0
**1.14.13.82**	0	0	0	0	0	0	0	0	0	0	0	0	0	0	0	0
**1.14.99.-**	0	0	0	0	0	0	1	0	0	0	0	0	0	0	0	0
**1.17.99.1**	0	0	0	0	0	0	0	0	0	0	0	0	0	0	0	0
**1.18.6.1**	0	0	0	0	0	0	0	0	0	0	0	0	0	0	0	0
**1.2.1.-**	0	0	0	0	0	0	1	0	0	0	0	0	0	0	0	0
**1.2.1.10**	2	2	11	2	3	4	1	6	8	6	9	4	7	0	2	4
**1.2.1.3**	1	4	5	5	7	8	6	8	7	4	12	7	5	1	3	7
**1.2.1.39**	0	0	0	0	0	1	0	0	0	0	0	0	0	0	0	1
**1.2.7.1**	8	7	9	4	3	6	5	10	0	5	1	17	10	5	4	5
**1.2.99.2**	0	1	2	2	1	5	0	3	7	1	3	3	1	1	0	3
**1.3.1.-**	0	0	0	0	0	0	1	0	1	0	0	0	0	1	0	0
**1.3.1.2**	0	0	2	1	0	2	1	0	0	0	0	1	0	1	1	1
**1.3.1.25**	0	0	0	0	0	0	0	0	0	0	0	0	0	0	0	0
**1.3.99.-**	0	0	0	0	0	0	1	0	2	0	0	1	0	0	0	1
**1.6.5.-**	21	33	29	34	15	18	14	32	8	37	13	66	42	28	29	32
**1.7.1.-**	6	6	8	5	6	6	1	8	4	7	9	8	17	6	5	12
**1.8.99.3**	0	0	0	1	0	0	0	0	0	0	0	0	0	0	0	1
**2.1.1.-**	101	125	205	133	90	106	108	189	147	165	151	222	188	108	96	130
**2.3.1.-**	62	97	98	104	84	106	57	109	67	100	71	166	141	84	62	98
**2.3.1.16**	1	2	0	1	0	3	0	2	1	0	2	1	2	0	0	2
**2.3.1.5**	0	0	0	1	0	1	0	0	0	0	0	0	0	0	0	1
**2.3.1.9**	1	1	0	1	0	3	1	0	2	0	2	0	1	0	0	1
**2.4.2.10**	6	7	13	11	4	4	10	12	9	7	8	19	8	5	5	9
**2.4.2.3**	3	5	5	5	3	5	3	8	7	3	4	5	5	3	3	4
**2.4.2.4**	3	1	3	4	0	0	0	6	2	1	2	2	2	0	0	1
**2.4.2.8**	8	11	12	6	6	7	5	12	7	10	7	11	10	11	6	6
**2.5.1.-**	13	14	15	13	11	11	13	16	8	19	5	30	19	14	12	15

EC/KO	CH-DLF003	CH-DLF004	CH-DLF005	CH-DLF006	CH-DLF007	CH-DLF008	CH-DLF009	CH-DLF010	CH-DLF012	CH-DLF013	CH-DLF014	CH-DLM001	CH-DLM002	CH-DLM003	CH-DLM004	CH-DLM005
**2.5.1.18**	0	0	1	4	1	6	2	8	5	1	5	1	1	0	0	5
**2.6.1.-**	14	23	16	15	18	18	9	28	9	15	15	34	32	13	16	19
**2.7.1.21**	3	6	4	5	2	3	1	4	3	5	4	7	2	8	4	7
**2.7.1.48**	13	19	22	13	11	12	6	13	12	20	13	26	24	14	11	12
**2.7.4.-**	10	10	11	8	6	6	5	8	8	13	6	22	13	5	4	9
**2.8.3.-**	0	2	1	5	2	3	6	1	2	0	1	3	3	0	1	3
**2.8.3.1**	0	0	1	1	1	0	0	0	0	0	0	1	0	0	0	1
**2.8.3.12**	0	0	0	3	0	0	0	0	0	0	1	1	1	0	0	0
**2.8.3.6**	0	0	0	1	0	0	0	1	0	0	0	0	1	0	0	1
**2.8.3.8**	0	1	2	3	2	3	1	0	3	2	4	0	1	1	0	3
**3.1.1.-**	3	3	2	5	3	6	2	3	4	2	5	8	5	1	6	7
**3.1.1.1**	2	3	5	2	3	3	2	5	2	2	2	8	10	4	5	3
**3.1.1.17**	0	3	0	2	1	0	0	3	0	2	0	0	0	1	2	1
**3.1.1.2**	0	0	0	1	0	1	0	0	1	0	0	0	0	0	0	1
**3.1.1.24**	0	0	2	3	1	1	0	3	3	2	2	2	0	0	0	2
**3.1.1.45**	0	0	0	1	0	1	0	1	0	0	0	1	0	0	0	1
**3.1.2.-**	0	1	0	2	0	2	2	1	2	1	1	3	0	0	1	2
**3.1.2.23**	2	3	3	2	2	4	3	3	0	2	1	7	5	2	2	5
**3.1.3.1**	8	15	11	9	12	10	8	9	3	11	8	21	15	15	15	7
**3.1.3.2**	3	7	4	3	5	4	2	5	3	5	3	7	4	7	3	5
**3.1.3.41**	0	0	0	0	0	0	1	0	0	0	0	1	0	0	0	0
**3.2.1.31**	0	1	2	1	1	2	2	0	4	2	0	3	5	0	0	1
**3.3.2.9**	0	0	0	0	0	0	0	0	0	0	0	0	0	0	0	0
**3.5.1.-**	19	9	17	9	6	7	7	13	10	10	8	15	17	6	9	13
**3.5.1.4**	0	2	2	3	1	1	0	3	3	1	3	5	3	1	0	2
**3.5.1.5**	0	0	1	0	0	0	0	2	0	0	2	1	0	2	0	0
**3.5.1.54**	3	0	1	4	0	1	2	2	3	4	0	2	0	0	0	0
**3.5.1.6**	0	0	2	1	1	1	0	1	0	0	3	2	1	0	1	0
**3.5.2.-**	0	0	0	0	0	0	0	0	0	0	0	0	0	0	0	0
**3.5.2.2**	0	1	0	1	1	2	0	1	1	1	3	2	2	0	0	0
**3.5.4.-**	15	9	20	9	10	9	12	15	7	13	9	24	20	11	9	17
**3.5.4.5**	7	8	9	5	4	5	5	9	3	9	3	10	6	6	5	7
**3.5.5.1**	0	1	0	0	2	2	0	1	0	1	2	1	0	1	1	1
**3.5.5.7**	0	0	0	0	0	0	0	0	0	0	1	0	0	0	0	0
**3.5.99.3**	0	0	0	0	1	1	0	0	0	1	0	0	0	0	0	0
**3.6.1.7**	1	1	5	1	0	2	2	2	4	5	4	3	2	1	0	3
**3.7.1.-**	1	0	2	1	0	1	0	2	3	3	2	1	1	0	0	2
**3.7.1.2**	0	2	1	3	2	3	4	3	1	4	1	10	4	5	4	3
**3.8.1.2**	3	4	1	5	6	5	2	5	5	5	3	8	7	6	6	6
**3.8.1.3**	0	0	0	0	0	0	0	0	0	0	0	0	0	0	0	0
**3.8.1.5**	0	1	0	1	1	1	3	1	1	1	1	1	0	0	0	0
**4.1.1.-**	4	7	5	10	5	10	4	9	3	10	6	27	8	8	6	12
**4.1.1.44**	9	13	15	16	11	9	11	13	10	11	11	27	16	10	15	15
**4.1.1.55**	0	0	0	0	0	0	0	0	0	0	0	0	0	0	0	0
**4.1.1.7**	0	0	0	0	0	0	0	0	0	0	0	0	0	0	0	0
**4.1.1.70**	0	0	0	0	0	0	0	1	0	0	0	0	0	0	0	0
**4.1.1.77**	0	0	0	0	0	0	0	0	0	0	0	0	0	0	0	0
**4.1.2.-**	6	2	6	5	1	9	3	3	8	7	3	5	5	0	2	5
**4.1.3.-**	9	12	11	6	5	7	4	15	9	9	7	15	16	5	9	6

EC/KO	CH-DLF003	CH-DLF004	CH-DLF005	CH-DLF006	CH-DLF007	CH-DLF008	CH-DLF009	CH-DLF010	CH-DLF012	CH-DLF013	CH-DLF014	CH-DLM001	CH-DLM002	CH-DLM003	CH-DLM004	CH-DLM005
**4.1.3.39**	0	1	0	0	0	0	0	1	0	0	0	0	0	0	1	1
**4.1.99.-**	0	1	4	0	1	0	1	3	0	0	1	0	2	0	0	0
**4.2.1.-**	13	30	33	31	25	32	21	28	22	28	21	53	38	38	27	25
**4.2.1.17**	3	2	6	2	1	3	4	2	3	2	2	3	3	3	3	4
**4.2.1.80**	0	1	0	0	0	0	0	0	0	0	0	0	0	0	0	0
**4.2.1.83**	1	1	1	0	0	0	0	0	0	0	0	0	1	0	0	0
**4.2.1.84**	1	0	0	0	0	0	0	0	0	0	0	0	1	0	0	0
**5.1.2.2**	0	0	0	0	0	0	0	0	0	0	0	0	0	0	0	0
**5.2.1.2**	0	0	0	0	0	0	0	0	0	0	0	0	0	0	0	0
**5.3.3.4**	0	0	0	0	0	0	0	0	0	0	0	0	0	0	0	0
**5.3.99.-**	0	1	0	6	4	3	2	1	2	2	2	1	1	1	1	2
**5.4.99.-**	0	1	0	2	0	0	1	0	0	1	0	0	0	1	0	1
**5.5.1.1**	2	3	3	1	2	5	2	1	0	5	1	6	2	2	4	3
**5.5.1.2**	0	0	0	0	0	0	0	0	0	0	0	0	0	0	0	0
**6.2.1.-**	0	0	0	0	0	0	0	0	0	0	0	0	0	0	0	0
**6.3.5.2**	6	14	16	14	13	13	10	11	14	15	10	25	20	9	10	17
**K00002**	0	0	0	0	0	0	0	2	0	0	2	1	0	0	0	0
**K00055**	0	0	0	0	1	1	0	1	0	0	1	0	0	0	0	0
**K00074**	3	1	2	6	1	1	4	7	2	7	5	9	3	1	0	4
**K00088**	7	14	15	11	10	12	7	12	12	9	15	30	20	7	9	12
**K00100**	21	28	33	31	20	31	16	28	27	31	33	69	30	22	24	31
**K00128**	0	1	0	0	1	0	3	0	3	0	2	2	2	0	0	1
**K00129**	0	0	0	0	0	0	0	0	0	0	0	0	0	0	0	0
**K00132**	0	0	0	0	0	0	0	0	0	0	1	1	0	0	0	1
**K00141**	0	0	0	0	0	0	0	0	0	0	0	0	0	0	0	0
**K00146**	0	0	0	0	0	1	0	1	0	0	0	0	0	0	0	1
**K00148**	0	0	0	0	0	0	0	0	0	0	0	0	0	0	0	0
**K00155**	0	0	0	1	0	0	0	0	1	0	0	0	0	1	0	0
**K00169**	0	1	3	0	1	3	1	2	0	0	1	2	2	0	0	0
**K00224**	0	0	0	0	0	0	0	0	0	0	0	1	0	0	0	0
**K00274**	0	0	0	0	0	0	0	0	0	0	0	0	0	0	0	0
**K00446**	0	0	0	0	0	0	0	0	0	0	0	0	0	0	0	0
**K00448**	0	0	0	0	0	0	0	1	0	0	0	0	0	0	0	0
**K00462**	1	0	0	0	2	0	0	0	0	1	1	3	0	1	1	1
**K00480**	0	0	0	0	0	0	0	0	0	0	0	0	0	0	0	0
**K00481**	0	0	0	0	0	0	0	1	0	0	0	0	0	0	0	0
**K00539**	0	0	0	0	0	0	0	0	0	0	0	1	0	0	1	0
**K00599**	16	27	30	20	18	20	18	24	17	19	23	36	31	17	13	15
**K00626**	3	4	1	4	1	5	3	2	3	2	7	5	5	0	0	4
**K00632**	0	0	0	0	0	0	0	0	0	0	0	0	0	0	0	0
**K00680**	18	30	24	22	15	19	22	16	20	29	13	41	29	25	17	27
**K00757**	7	10	5	7	6	8	5	6	3	10	3	16	8	7	8	6
**K00758**	4	0	3	2	0	1	1	2	1	0	3	2	2	0	0	3
**K00760**	4	12	18	12	6	7	8	15	10	13	12	16	13	12	7	8
**K00799**	0	0	0	2	1	2	2	4	2	0	1	0	0	0	0	2
**K00857**	7	7	8	9	2	3	4	6	6	10	6	12	4	11	4	9
**K00876**	15	18	25	12	10	10	8	17	9	22	16	32	31	14	11	15
**K01026**	0	1	1	1	1	3	0	0	0	2	6	0	1	1	0	2
**K01031**	0	0	0	0	0	0	0	0	0	0	0	0	0	0	0	0

EC/KO	CH-DLF003	CH-DLF004	CH-DLF005	CH-DLF006	CH-DLF007	CH-DLF008	CH-DLF009	CH-DLF010	CH-DLF012	CH-DLF013	CH-DLF014	CH-DLM001	CH-DLM002	CH-DLM003	CH-DLM004	CH-DLM005
K01034	0	0	1	1	0	1	1	1	1	2	0	2	0	1	0	1
K01039	0	0	0	1	0	0	0	0	0	0	1	1	0	0	0	0
K01041	3	8	3	5	5	3	2	3	2	5	2	16	4	4	5	6
K01053	0	0	0	0	0	0	0	1	0	0	0	0	0	0	0	0
K01055	0	0	0	0	0	0	0	0	0	0	0	0	0	0	0	0
K01061	0	0	0	1	0	1	0	1	1	0	0	1	0	0	0	1
K01066	2	1	5	2	1	4	3	3	3	4	4	9	4	2	2	3
K01075	0	0	0	1	0	1	0	1	1	0	0	1	0	0	1	1
K01077	1	6	3	6	5	5	5	4	3	7	4	10	5	9	7	4
K01101	1	1	1	4	1	2	2	1	5	4	3	5	2	1	1	3
K01195	2	1	3	3	4	5	2	2	2	4	3	9	7	0	3	3
K01426	0	1	0	1	1	2	1	0	0	0	2	2	1	0	0	0
K01428	0	0	1	0	0	0	0	0	1	0	2	0	0	2	0	0
K01457	0	0	0	0	0	0	0	0	0	0	0	0	0	0	0	0
K01464	0	1	0	2	2	6	1	3	2	1	6	3	1	1	0	2
K01489	10	10	11	8	5	5	6	15	2	11	5	14	8	8	5	10
K01500	0	0	0	0	0	0	0	0	0	1	0	0	0	0	0	0
K01501	0	1	0	1	1	2	1	2	0	2	4	2	0	0	0	1
K01502	0	0	0	0	0	0	0	0	0	0	1	0	0	0	0	0
K01512	2	2	6	4	0	3	2	4	6	8	6	6	3	1	0	4
K01560	1	2	0	0	2	0	4	5	2	5	2	5	3	1	0	1
K01561	0	0	0	1	0	1	0	0	0	0	0	2	1	0	0	0
K01563	0	0	0	0	0	0	0	0	0	0	0	0	0	0	0	0
K01564	0	1	1	5	0	0	1	0	1	1	1	3	2	2	0	3
K01607	4	7	6	6	4	4	3	10	6	7	3	12	6	4	5	9
K01612	0	0	0	0	0	0	0	0	0	0	0	0	0	0	0	0
K01615	7	11	10	10	7	8	5	17	4	13	5	21	8	9	9	9
K01617	0	0	0	0	0	0	0	0	0	0	0	0	0	0	0	0
K01666	2	2	8	0	2	0	0	3	0	2	3	2	5	0	1	1
K01692	0	0	0	0	0	0	0	0	0	0	0	0	0	0	0	1
K01721	0	0	0	0	0	0	0	0	0	0	0	0	0	0	0	0
K01726	4	6	2	5	6	8	1	2	4	6	3	9	8	1	5	5
K01781	0	0	0	0	1	3	0	0	0	0	1	0	0	0	1	0
K01821	0	2	0	1	0	1	0	0	1	1	3	1	1	1	1	2
K01856	0	0	0	0	0	0	0	0	0	0	0	0	0	0	0	0
K01857	0	0	0	0	0	0	0	0	0	0	1	1	0	0	0	0
K01913	0	0	0	0	0	0	0	0	0	0	0	0	1	0	1	0
K01951	6	12	15	13	8	9	9	10	11	15	14	22	17	8	7	8
K02554	0	0	0	0	0	0	0	1	0	0	0	0	0	0	0	0
K03381	0	0	0	0	0	0	0	0	0	0	0	0	0	0	0	0
K03382	0	0	0	0	1	2	2	2	0	1	0	0	0	0	0	0
K03464	0	0	0	0	0	0	0	0	0	0	0	0	0	0	0	0
K03518	1	2	4	3	9	11	3	9	6	6	10	4	5	2	3	4
K03862	0	0	0	0	0	0	0	0	0	0	0	0	0	0	0	0
K04099	0	0	0	0	0	0	0	0	0	0	0	0	0	0	0	0
K04100	0	0	0	0	0	0	0	1	0	0	0	0	0	0	0	0
K04102	0	0	0	0	0	0	0	0	0	0	0	0	0	0	0	0
K04116	0	0	0	0	0	0	0	0	0	0	0	0	0	0	0	0
K05394	1	0	0	0	0	0	0	3	0	0	2	0	0	0	1	0

EC/KO	CH-DLF003	CH-DLF004	CH-DLF005	CH-DLF006	CH-DLF007	CH-DLF008	CH-DLF009	CH-DLF010	CH-DLF012	CH-DLF013	CH-DLF014	CH-DLM001	CH-DLM002	CH-DLM003	CH-DLM004	CH-DLM005
**K05549**	0	0	0	0	0	0	0	1	0	0	0	0	0	0	0	0
**K05783**	0	0	0	0	0	0	0	0	0	0	0	0	0	0	0	0
**K05797**	0	0	0	0	0	0	0	0	0	0	0	0	0	0	0	0
**K06281**	0	0	1	3	1	2	2	0	1	0	1	4	1	1	1	1
**K06446**	3	5	4	5	0	2	4	2	1	3	6	12	2	1	3	5
**K06912**	0	0	0	0	0	0	0	0	0	0	0	0	0	0	0	0
**K07535**	0	0	0	0	0	0	0	0	0	0	0	0	0	0	0	0
**K07536**	1	2	2	4	1	3	2	1	2	3	0	2	0	2	1	2
**K08689**	0	0	0	0	0	0	0	0	0	0	0	0	0	0	0	0
**K08710**	0	0	0	0	0	1	0	0	0	0	1	0	0	0	1	0
**K09461**	0	0	0	0	0	0	1	0	0	0	0	0	0	0	0	0
**K10217**	0	0	0	0	0	0	0	0	0	0	0	0	0	0	0	1
**K10218**	0	0	0	1	0	0	0	2	0	0	0	3	1	0	0	1
**K10220**	0	0	0	0	0	0	0	0	0	0	0	0	0	0	0	0
**K11180**	0	0	0	0	0	0	0	0	0	0	0	0	0	0	0	0
**K13953**	0	0	0	0	0	1	1	0	1	0	2	1	0	1	0	1
**K14333**	0	0	0	0	0	0	0	1	0	0	0	0	0	0	0	0
**K14519**	0	0	0	0	0	0	0	0	0	0	0	0	0	0	0	0
**K15054**	0	0	0	0	0	0	0	0	0	0	0	0	0	0	0	1
**K16173**	0	1	2	1	0	0	0	0	0	0	3	1	0	0	0	1
**K16514**	0	0	0	1	0	1	0	0	0	0	1	0	0	0	0	1
**K16874**	0	0	0	0	0	0	0	0	0	0	0	1	0	0	0	0

EC/KO	CH-DLM006	CH-DLM007	CH-DLM008	CH-DLM009	CH-DLM010	CH-DLM011	CH-DLM012	CH-DLM014	CH-DLM015	CH-DLM016	CH-DLM017	CH-DLM018	CH-DLM019	CH-DLM020	CH-DLM021	CH-DLM022
**1.1.1.-**	22	20	25	26	53	26	34	54	38	40	33	31	37	49	33	54
**1.1.1.1**	16	18	27	31	36	21	41	29	21	25	30	32	37	23	27	49
**1.1.1.157**	0	0	0	0	1	0	0	1	0	1	1	2	2	0	0	1
**1.1.1.205**	16	10	16	16	13	15	13	28	22	15	12	14	24	18	13	33
**1.1.1.35**	1	0	4	1	3	0	2	2	1	3	0	2	2	0	1	6
**1.12.99.6**	0	0	0	7	0	2	3	1	3	1	2	1	1	1	0	6
**1.13.11.-**	0	0	0	0	0	0	0	0	0	0	0	0	0	0	0	0
**1.13.11.1**	0	0	0	0	0	0	0	0	0	0	0	0	0	0	0	0
**1.13.11.2**	0	0	0	0	0	0	0	0	0	0	0	0	0	0	0	0
**1.13.11.3**	0	0	0	0	0	0	0	0	0	0	0	0	0	0	0	0
**1.13.11.39**	0	0	0	0	0	0	0	0	0	0	0	0	0	0	0	0
**1.13.11.5**	0	0	0	0	0	1	0	0	0	0	0	0	0	0	0	1
**1.13.11.8**	0	0	0	0	1	0	0	0	0	0	0	0	0	0	0	0
**1.14.12.10**	0	0	0	0	0	0	1	0	0	0	0	0	0	0	1	0
**1.14.12.13**	0	0	0	0	0	0	0	0	0	0	0	0	0	0	0	0
**1.14.12.18**	0	0	0	0	0	0	0	0	0	0	0	0	0	0	0	0
**1.14.13.-**	0	0	0	0	2	0	3	0	0	0	0	0	2	0	0	1
**1.14.13.1**	0	0	0	0	1	0	0	0	0	0	0	0	0	0	0	0
**1.14.13.2**	0	0	0	0	1	0	0	0	0	0	0	0	0	0	0	0
**1.14.13.50**	0	0	0	0	0	0	0	0	0	0	0	0	0	0	0	0
**1.14.13.7**	1	0	0	0	0	0	0	1	0	0	0	0	0	0	1	0
**1.14.13.8**	0	0	0	0	0	0	0	0	0	0	0	0	0	0	0	0
**1.14.13.82**	0	0	0	0	0	0	0	0	0	0	0	0	0	0	0	0
**1.14.99.-**	0	0	0	0	0	0	0	0	0	0	0	0	0	0	0	0
**1.17.99.1**	0	0	0	0	0	0	0	0	0	0	0	0	0	0	0	0
**1.18.6.1**	0	0	0	0	1	0	0	0	0	0	0	0	0	0	0	0
**1.2.1.-**	0	0	0	0	1	0	3	0	0	0	0	0	0	0	0	1
**1.2.1.10**	6	1	2	4	6	0	4	4	7	6	4	7	10	2	1	16
**1.2.1.3**	3	3	1	8	6	3	9	3	8	3	3	9	6	5	5	10
**1.2.1.39**	0	0	0	0	0	0	1	0	0	0	0	0	0	0	0	0
**1.2.7.1**	7	4	7	5	5	3	0	9	6	4	5	3	13	5	10	23
**1.2.99.2**	1	0	2	5	2	2	6	2	3	3	2	2	6	1	2	4
**1.3.1.-**	0	1	0	0	1	1	0	3	0	1	1	0	0	1	0	0
**1.3.1.2**	1	1	2	0	0	0	1	1	0	1	0	0	0	1	1	2
**1.3.1.25**	0	0	0	0	0	0	0	0	0	0	0	0	0	0	0	0
**1.3.99.-**	0	0	0	0	0	0	1	0	0	0	0	0	0	1	0	1
**1.6.5.-**	32	33	35	12	32	28	15	68	37	38	33	20	38	37	34	56
**1.7.1.-**	9	8	4	7	6	7	3	16	6	11	6	7	9	8	4	7
**1.8.99.3**	0	0	0	0	0	0	0	0	0	0	0	0	0	0	0	0
**2.1.1.-**	135	128	151	140	163	106	135	209	149	151	179	177	255	140	156	353
**2.3.1.-**	75	97	93	66	115	78	73	177	118	119	84	89	135	119	91	193
**2.3.1.16**	2	1	4	0	2	0	1	2	1	2	1	1	1	2	1	3
**2.3.1.5**	0	0	0	1	0	0	1	0	0	0	0	0	0	0	0	0
**2.3.1.9**	0	1	0	1	2	0	5	0	1	1	0	1	1	2	1	4
**2.4.2.10**	9	8	13	8	8	5	9	11	9	11	8	16	11	4	9	21
**2.4.2.3**	6	4	5	2	6	2	5	4	7	6	2	3	7	1	4	15
**2.4.2.4**	2	0	1	3	2	1	3	0	2	1	2	3	4	1	2	11
**2.4.2.8**	9	5	5	8	11	4	8	8	9	11	8	6	14	8	6	26
**2.5.1.-**	16	12	11	10	13	12	15	29	16	15	16	13	19	19	12	18

EC/KO	CH-DLM006	CH-DLM007	CH-DLM008	CH-DLM009	CH-DLM010	CH-DLM011	CH-DLM012	CH-DLM014	CH-DLM015	CH-DLM016	CH-DLM017	CH-DLM018	CH-DLM019	CH-DLM020	CH-DLM021	CH-DLM022
**2.5.1.18**	0	0	0	3	4	4	4	0	0	1	1	1	1	0	1	2
**2.6.1.-**	21	21	26	10	29	21	23	42	35	21	25	16	23	23	13	30
**2.7.1.21**	5	4	3	5	7	5	3	12	7	7	4	4	8	5	4	6
**2.7.1.48**	18	16	14	10	20	15	9	39	18	19	12	14	25	16	17	31
**2.7.4.-**	10	9	13	4	12	9	10	14	9	8	14	11	15	9	11	25
**2.8.3.-**	2	0	0	1	1	3	1	1	4	2	1	0	1	3	1	4
**2.8.3.1**	0	0	0	1	0	0	0	0	0	0	0	1	0	0	0	1
**2.8.3.12**	0	0	1	0	0	0	0	0	0	0	0	1	0	0	0	0
**2.8.3.6**	0	0	1	0	0	1	0	0	0	0	0	0	0	0	0	2
**2.8.3.8**	0	1	4	2	1	0	1	1	1	0	1	2	2	0	1	4
**3.1.1.-**	3	0	5	7	9	5	4	3	8	3	0	3	3	3	3	11
**3.1.1.1**	2	0	3	3	2	9	2	8	5	8	4	7	3	9	4	4
**3.1.1.17**	0	0	1	0	2	1	0	2	2	4	3	0	1	4	1	3
**3.1.1.2**	0	1	0	1	1	1	1	0	0	0	0	0	0	0	0	2
**3.1.1.24**	0	1	2	1	0	1	1	5	1	0	0	2	7	0	0	4
**3.1.1.45**	0	0	0	0	1	0	0	0	0	0	0	0	0	0	0	1
**3.1.2.-**	0	0	0	0	0	1	2	0	0	0	0	2	1	0	0	0
**3.1.2.23**	1	6	1	0	6	3	2	9	4	3	6	3	4	5	4	5
**3.1.3.1**	8	14	9	4	13	14	4	30	15	21	17	11	10	25	17	15
**3.1.3.2**	5	5	5	3	7	9	3	9	7	6	2	2	4	8	4	3
**3.1.3.41**	0	0	0	0	0	0	0	0	0	0	0	1	0	0	0	0
**3.2.1.31**	4	0	2	1	5	2	1	4	2	3	5	4	2	1	1	2
**3.3.2.9**	0	0	0	0	1	0	0	0	0	0	0	0	0	0	0	0
**3.5.1.-**	14	9	10	7	15	9	7	13	11	13	9	16	24	5	10	31
**3.5.1.4**	1	1	1	3	1	1	1	1	3	3	1	3	4	1	1	2
**3.5.1.5**	0	2	0	1	1	0	0	2	0	1	0	0	1	2	0	0
**3.5.1.54**	0	0	3	4	1	0	5	0	0	0	1	1	5	0	0	3
**3.5.1.6**	1	0	1	3	3	0	0	0	0	1	0	0	1	0	0	1
**3.5.2.-**	0	0	0	0	0	0	0	0	0	0	0	0	0	0	0	0
**3.5.2.2**	0	0	0	1	0	0	3	1	0	1	1	2	4	0	0	2
**3.5.4.-**	11	10	10	7	13	9	11	20	8	12	19	15	17	8	12	32
**3.5.4.5**	4	7	7	4	11	6	7	14	10	5	6	4	10	8	6	21
**3.5.5.1**	0	0	0	0	2	0	1	2	1	0	0	1	1	0	0	1
**3.5.5.7**	0	0	0	0	0	0	0	0	0	0	0	0	0	0	0	0
**3.5.99.3**	0	0	0	0	0	0	0	0	0	0	0	0	1	0	0	0
**3.6.1.7**	1	1	3	1	3	1	7	0	1	1	0	1	1	2	2	5
**3.7.1.-**	0	0	0	1	2	0	0	0	1	0	1	1	0	0	0	2
**3.7.1.2**	0	5	3	3	4	3	0	7	4	6	2	0	4	4	3	3
**3.8.1.2**	2	12	4	4	7	6	7	8	8	6	4	1	3	9	6	5
**3.8.1.3**	0	0	0	0	0	0	0	0	0	0	0	0	0	0	0	1
**3.8.1.5**	0	0	0	0	2	0	2	2	0	0	0	1	2	0	0	1
**4.1.1.-**	6	5	7	4	9	11	7	16	10	17	9	7	7	10	12	10
**4.1.1.44**	8	8	6	12	14	11	10	23	21	11	14	19	19	15	12	29
**4.1.1.55**	0	0	0	0	0	0	0	0	0	0	0	0	0	0	0	0
**4.1.1.7**	0	0	0	0	0	0	0	0	0	0	0	0	0	0	0	0
**4.1.1.70**	0	0	0	0	0	0	0	0	0	0	0	0	1	0	0	0
**4.1.1.77**	0	0	0	0	0	0	0	0	0	0	0	0	0	0	0	0
**4.1.2.-**	0	1	4	4	5	2	10	0	4	2	3	5	10	4	1	9
**4.1.3.-**	10	9	13	8	5	6	4	16	15	7	13	11	17	11	11	16

EC/KO	CH-DLM006	CH-DLM007	CH-DLM008	CH-DLM009	CH-DLM010	CH-DLM011	CH-DLM012	CH-DLM014	CH-DLM015	CH-DLM016	CH-DLM017	CH-DLM018	CH-DLM019	CH-DLM020	CH-DLM021	CH-DLM022
**4.1.3.39**	0	1	0	0	0	0	0	2	2	0	0	0	1	0	0	0
**4.1.99.-**	1	0	1	1	1	0	0	1	0	0	0	0	0	0	0	4
**4.2.1.-**	29	27	25	18	28	29	25	63	40	38	30	20	38	46	33	60
**4.2.1.17**	3	4	4	3	7	7	4	3	5	2	2	5	3	1	5	7
**4.2.1.80**	0	0	0	1	0	0	0	0	1	0	0	0	0	0	0	0
**4.2.1.83**	0	0	0	0	0	0	0	0	1	0	0	0	0	0	1	0
**4.2.1.84**	0	0	1	0	0	0	0	0	1	0	0	0	0	0	0	0
**5.1.2.2**	0	0	0	0	0	0	0	0	0	0	0	0	0	0	0	0
**5.2.1.2**	0	0	0	0	0	0	0	0	0	0	0	0	0	0	0	0
**5.3.3.4**	0	0	0	0	1	0	0	0	0	0	0	0	0	0	0	0
**5.3.99.-**	0	2	2	0	1	1	6	2	3	1	2	2	1	1	0	8
**5.4.99.-**	1	0	0	0	0	0	0	1	0	1	1	0	1	0	1	0
**5.5.1.1**	1	3	1	1	3	1	0	6	5	4	3	2	7	8	6	6
**5.5.1.2**	0	0	0	0	1	0	0	0	0	0	0	0	0	0	0	0
**6.2.1.-**	0	0	0	0	0	0	0	0	0	0	0	0	0	0	0	0
**6.3.5.2**	15	11	11	7	13	9	10	24	18	14	16	17	19	14	14	26
**K00002**	0	0	0	2	1	0	1	0	0	2	1	0	0	0	0	4
**K00055**	0	0	0	1	1	0	0	0	0	0	0	0	0	0	0	0
**K00074**	1	0	5	1	1	0	3	2	3	4	3	9	9	0	3	14
**K00088**	14	13	13	11	10	12	10	21	18	15	12	13	21	20	13	29
**K00100**	23	20	20	33	33	23	29	50	32	30	34	31	46	35	32	54
**K00128**	2	0	0	2	1	0	5	0	2	0	0	2	2	1	1	3
**K00129**	0	0	0	0	0	0	0	0	0	0	0	0	0	0	0	0
**K00132**	0	0	0	0	0	0	0	0	0	0	0	0	2	0	0	2
**K00141**	0	0	0	0	1	0	0	0	0	0	0	0	0	0	0	0
**K00146**	0	0	0	0	2	0	1	0	0	0	0	0	0	0	0	0
**K00148**	0	0	0	0	0	0	0	0	0	0	0	0	0	0	0	0
**K00155**	0	0	0	2	0	0	0	0	0	0	0	0	0	0	0	1
**K00169**	0	0	0	1	1	0	0	1	1	0	1	1	2	0	0	6
**K00224**	0	0	0	1	0	0	0	0	0	0	0	0	0	0	0	0
**K00274**	0	0	0	0	0	0	0	0	0	0	0	0	0	0	0	0
**K00446**	0	0	0	0	0	0	0	0	0	0	0	0	0	0	0	0
**K00448**	0	0	0	0	0	0	0	0	0	0	0	0	0	0	0	0
**K00462**	1	1	0	2	1	0	0	0	1	0	0	2	0	0	0	0
**K00480**	0	0	0	0	0	0	0	0	0	0	0	0	0	0	0	0
**K00481**	0	0	0	0	1	0	0	0	0	0	0	0	0	0	0	0
**K00539**	0	0	0	0	1	0	0	0	0	0	0	0	0	0	0	1
**K00599**	33	23	27	20	26	13	19	40	37	32	20	28	31	21	26	46
**K00626**	4	2	9	1	5	0	4	3	2	3	3	6	4	4	5	8
**K00632**	0	0	0	0	0	0	1	0	0	0	0	0	0	0	0	0
**K00680**	16	17	15	17	21	26	21	47	31	26	29	29	31	26	25	54
**K00757**	9	7	5	1	9	7	3	19	9	13	3	5	6	6	9	8
**K00758**	1	0	1	1	1	3	1	1	1	1	3	1	6	0	0	5
**K00760**	12	9	18	8	12	8	9	15	10	13	14	13	26	10	12	38
**K00799**	0	0	0	3	2	3	1	0	0	0	0	0	0	0	0	1
**K00857**	7	6	6	6	10	8	3	18	7	8	10	7	17	7	7	11
**K00876**	17	10	13	10	19	19	7	35	13	17	17	13	31	17	17	29
**K01026**	0	0	1	0	2	0	0	1	1	0	1	3	0	0	1	0
**K01031**	0	0	0	0	0	0	0	0	0	0	0	0	0	0	0	0

EC/KO	CH-DLM006	CH-DLM007	CH-DLM008	CH-DLM009	CH-DLM010	CH-DLM011	CH-DLM012	CH-DLM014	CH-DLM015	CH-DLM016	CH-DLM017	CH-DLM018	CH-DLM019	CH-DLM020	CH-DLM021	CH-DLM022
K01034	0	1	0	1	0	0	0	1	1	0	1	1	3	0	1	2
K01039	0	0	1	0	0	0	0	0	0	1	0	0	1	0	0	0
K01041	7	6	4	1	4	9	2	17	5	10	5	7	3	8	7	7
K01053	0	0	0	0	1	0	0	0	0	0	0	0	0	0	0	1
K01055	0	0	0	0	0	0	0	0	0	0	0	0	0	0	0	0
K01061	0	0	0	1	1	1	0	0	0	0	0	0	0	1	0	1
K01066	0	2	5	3	3	6	4	7	6	5	6	7	5	5	4	4
K01075	0	0	0	1	0	1	1	0	0	1	0	0	0	0	1	0
K01077	3	6	7	4	10	11	2	17	8	11	8	6	5	17	12	13
K01101	2	2	2	1	1	3	2	1	1	1	1	4	3	2	3	4
K01195	6	3	2	4	7	5	1	9	6	2	7	5	7	8	6	3
K01426	1	0	0	2	1	1	1	1	1	2	0	1	2	1	0	1
K01428	0	0	0	2	1	0	1	0	0	0	1	0	1	1	0	1
K01457	0	0	0	0	0	0	0	0	0	0	0	0	0	0	0	0
K01464	0	0	1	2	1	0	4	1	0	1	1	3	7	0	0	3
K01489	8	8	10	4	12	8	8	15	11	7	10	7	14	10	10	16
K01500	0	0	0	0	0	0	0	0	0	0	0	0	0	0	0	0
K01501	0	0	0	0	2	0	1	1	0	1	0	1	1	0	0	3
K01502	0	0	0	0	0	0	0	0	0	0	0	0	0	0	0	0
K01512	2	2	4	2	3	1	8	0	1	1	1	3	1	2	2	5
K01560	1	2	1	0	3	2	0	1	0	3	2	6	4	0	3	4
K01561	1	0	0	1	1	1	0	0	1	0	0	0	0	0	1	0
K01563	0	0	0	0	0	0	0	0	0	0	0	0	0	0	0	0
K01564	1	1	0	2	1	0	1	2	1	2	2	0	0	2	1	2
K01607	5	6	4	2	7	7	7	17	11	9	9	7	7	10	7	17
K01612	0	0	0	0	0	0	0	0	0	0	0	0	0	0	0	0
K01615	8	5	9	5	10	16	3	27	8	15	11	8	25	13	13	20
K01617	0	0	0	0	0	0	0	0	0	0	0	0	0	0	0	0
K01666	4	2	5	1	0	1	1	5	4	1	2	4	6	1	0	3
K01692	0	0	0	0	0	0	0	0	0	0	0	0	0	0	0	0
K01721	0	0	0	0	0	0	0	0	0	0	0	0	0	0	0	0
K01726	4	6	3	4	9	11	3	16	6	9	4	6	4	7	4	8
K01781	0	0	0	0	0	1	0	0	0	0	0	0	0	0	0	0
K01821	0	1	0	1	2	2	1	3	1	2	1	1	1	0	0	2
K01856	0	0	0	0	0	0	0	0	0	0	0	0	0	0	0	0
K01857	0	0	0	0	1	0	0	0	0	0	0	0	1	0	0	0
K01913	0	1	0	0	0	0	0	0	0	0	0	1	0	0	0	0
K01951	13	9	12	10	11	7	10	21	13	14	14	12	23	13	18	28
K02554	0	0	0	1	0	0	0	0	1	0	0	0	0	0	0	0
K03381	0	0	0	0	0	0	0	0	0	0	0	0	0	0	0	0
K03382	0	0	0	0	1	0	0	0	0	0	0	1	4	0	0	2
K03464	0	0	0	0	0	0	0	0	0	0	0	0	0	0	0	0
K03518	3	2	5	6	6	2	8	3	1	4	5	8	13	2	5	10
K03862	0	0	0	0	0	0	0	0	0	0	0	0	0	0	0	0
K04099	0	0	0	0	1	0	0	0	0	0	0	0	0	0	0	0
K04100	0	0	0	0	0	0	0	0	0	0	0	0	0	0	0	0
K04102	0	0	0	0	0	0	0	0	0	0	0	0	0	0	0	0
K04116	0	0	0	0	0	0	0	0	0	0	0	0	0	0	0	0
K05394	0	0	0	0	0	0	0	0	1	0	0	0	0	0	0	1

EC/KO	CH-DLM006	CH-DLM007	CH-DLM008	CH-DLM009	CH-DLM010	CH-DLM011	CH-DLM012	CH-DLM014	CH-DLM015	CH-DLM016	CH-DLM017	CH-DLM018	CH-DLM019	CH-DLM020	CH-DLM021	CH-DLM022
**K05549**	0	0	0	0	1	0	0	0	0	0	0	0	0	0	0	0
**K05783**	0	0	0	0	0	0	0	0	0	0	0	0	0	0	0	0
**K05797**	0	0	0	0	0	0	0	0	0	0	0	0	0	0	0	0
**K06281**	0	0	0	4	0	2	2	1	2	1	2	1	0	1	0	5
**K06446**	2	2	4	0	6	3	0	3	3	3	4	5	5	0	3	7
**K06912**	0	0	0	0	0	0	0	0	0	0	0	0	0	0	0	0
**K07535**	0	0	0	0	0	0	0	0	0	0	0	1	1	0	0	1
**K07536**	4	2	2	4	2	0	3	6	1	1	3	0	2	2	1	0
**K08689**	0	0	0	0	0	0	0	0	0	0	0	0	0	0	0	0
**K08710**	0	0	0	0	0	0	1	0	0	0	0	0	0	0	0	0
**K09461**	0	0	0	0	0	0	1	0	0	0	0	0	0	0	0	0
**K10217**	0	0	0	0	0	0	0	0	0	0	0	0	0	0	0	0
**K10218**	0	0	0	0	0	0	0	0	1	1	0	0	1	0	1	0
**K10220**	0	0	0	0	0	0	0	0	0	0	0	0	0	0	0	0
**K11180**	0	0	0	0	0	0	0	1	1	1	0	0	1	0	0	0
**K13953**	0	0	0	0	0	0	2	0	0	0	0	0	0	0	0	1
**K14333**	0	0	0	0	0	0	0	0	0	0	0	0	0	0	0	0
**K14519**	0	0	0	0	1	0	0	0	0	0	0	0	0	0	0	0
**K15054**	0	0	0	0	1	0	0	0	0	0	0	0	0	0	0	0
**K16173**	0	0	1	0	0	0	0	1	0	0	0	1	1	0	1	0
**K16514**	0	0	0	1	0	0	1	1	0	0	0	0	0	0	0	0
**K16874**	0	0	0	0	0	0	0	0	0	0	0	0	0	0	0	0

EC/KO	CH-DLM023	CH-DLM024	CH-DLM027	CH-DLM028	CH-DOF002	CH-DOF003	CH-DOF004	CH-DOF006	CH-DOF007	CH-DOF008	CH-DOF009	CH-DOF010	CH-DOF011	CH-DOF012	CH-DOF013	CH-DOF014
**1.1.1.-**	40	26	40	38	20	54	60	43	35	37	33	36	13	36	30	26
**1.1.1.1**	36	22	37	35	11	38	29	34	17	41	23	19	7	28	13	28
**1.1.1.157**	1	0	0	0	0	0	1	0	0	2	1	1	0	1	0	0
**1.1.1.205**	22	9	16	14	8	22	18	16	9	26	13	6	8	14	10	15
**1.1.1.35**	1	1	0	4	1	1	1	0	0	6	0	1	0	1	1	0
**1.12.99.6**	0	1	1	3	2	1	4	5	2	0	0	2	1	2	2	0
**1.13.11.-**	0	0	0	0	0	0	0	0	0	0	0	0	0	0	0	0
**1.13.11.1**	0	0	0	0	0	0	0	0	0	0	0	0	0	0	0	0
**1.13.11.2**	0	0	0	0	0	0	0	0	0	0	0	0	0	0	0	0
**1.13.11.3**	0	0	0	1	0	0	0	0	0	0	0	0	0	0	0	0
**1.13.11.39**	0	0	0	0	0	0	0	0	0	0	0	0	0	0	0	0
**1.13.11.5**	0	0	0	0	0	0	0	0	0	0	0	0	0	0	0	0
**1.13.11.8**	0	0	0	1	0	0	0	0	0	0	0	0	0	0	0	0
**1.14.12.10**	0	1	0	2	0	0	1	1	0	0	1	1	0	1	0	0
**1.14.12.13**	0	0	0	0	0	0	0	0	0	0	0	0	0	0	0	0
**1.14.12.18**	0	0	0	0	0	0	0	0	0	0	0	0	0	0	0	0
**1.14.13.-**	0	1	0	11	0	0	3	3	0	2	0	2	0	0	0	0
**1.14.13.1**	0	0	0	0	0	0	0	0	0	0	0	0	0	0	0	0
**1.14.13.2**	0	0	0	1	0	0	0	0	0	0	0	0	0	0	0	0
**1.14.13.50**	0	0	0	0	0	0	0	0	0	0	0	0	0	0	0	0
**1.14.13.7**	0	0	0	0	0	0	0	1	0	0	0	0	0	0	0	0
**1.14.13.8**	0	0	0	0	0	0	0	0	0	0	0	0	0	0	0	0
**1.14.13.82**	0	0	0	2	0	0	0	0	0	0	0	0	0	0	0	0
**1.14.99.-**	0	0	0	0	0	0	1	0	0	0	0	0	0	0	0	0
**1.17.99.1**	0	0	0	0	0	0	0	0	0	0	0	0	0	0	0	0
**1.18.6.1**	0	0	0	1	0	0	0	0	0	0	0	0	0	0	0	0
**1.2.1.-**	0	1	0	5	0	0	2	0	0	0	0	2	0	2	0	0
**1.2.1.10**	2	3	7	4	2	5	7	5	1	11	3	2	1	8	2	6
**1.2.1.3**	5	5	5	12	4	6	12	9	5	17	2	9	1	8	3	3
**1.2.1.39**	0	0	0	1	0	0	0	1	0	0	0	1	0	0	0	0
**1.2.7.1**	7	1	2	6	6	7	7	7	4	5	7	3	3	1	6	2
**1.2.99.2**	6	6	2	3	0	3	5	7	1	10	0	5	0	4	0	0
**1.3.1.-**	1	0	0	0	0	0	2	0	0	0	0	0	1	0	0	1
**1.3.1.2**	0	0	1	1	1	1	2	2	1	1	0	0	0	2	0	0
**1.3.1.25**	0	0	0	1	0	0	0	0	0	0	0	0	0	0	0	0
**1.3.99.-**	0	1	0	0	0	0	2	1	1	0	0	0	0	0	0	0
**1.6.5.-**	40	7	15	34	21	52	32	19	23	21	31	16	16	11	25	14
**1.7.1.-**	4	1	5	7	2	11	7	2	6	8	12	7	5	5	8	6
**1.8.99.3**	0	0	0	0	0	0	0	1	0	1	0	0	0	0	0	0
**2.1.1.-**	204	75	139	223	117	212	183	128	89	216	130	98	58	121	95	113
**2.3.1.-**	107	56	63	129	65	166	99	106	71	101	98	69	31	71	85	72
**2.3.1.16**	3	0	2	4	1	1	2	2	0	1	0	1	0	1	1	1
**2.3.1.5**	0	0	0	2	0	0	1	0	0	1	0	1	0	1	0	0
**2.3.1.9**	0	2	2	9	0	0	3	2	0	2	0	3	0	6	0	0
**2.4.2.10**	12	5	8	15	1	9	10	8	3	12	6	5	2	6	6	7
**2.4.2.3**	4	7	7	9	2	5	7	3	3	9	3	1	3	7	2	5
**2.4.2.4**	3	1	0	4	2	4	1	1	0	4	2	0	1	0	1	0
**2.4.2.8**	8	5	9	8	5	12	9	10	5	16	9	6	3	14	7	2
**2.5.1.-**	14	3	14	14	8	20	17	13	10	15	19	7	8	19	13	12

EC/KO	CH-DLM023	CH-DLM024	CH-DLM027	CH-DLM028	CH-DOF002	CH-DOF003	CH-DOF004	CH-DOF006	CH-DOF007	CH-DOF008	CH-DOF009	CH-DOF010	CH-DOF011	CH-DOF012	CH-DOF013	CH-DOF014
**2.5.1.18**	1	3	2	8	0	0	3	3	5	7	0	5	0	5	0	0
**2.6.1.-**	21	10	20	33	18	29	28	19	15	25	16	18	7	24	22	14
**2.7.1.21**	5	1	4	7	7	4	4	3	3	6	4	1	2	9	6	3
**2.7.1.48**	21	9	12	17	12	26	20	17	9	18	18	13	7	12	11	11
**2.7.4.-**	12	1	6	11	6	16	10	6	3	9	10	6	5	6	8	7
**2.8.3.-**	1	0	2	2	3	3	3	0	3	0	0	5	1	2	3	0
**2.8.3.1**	0	2	0	0	0	0	0	0	1	0	2	0	0	0	0	0
**2.8.3.12**	1	0	0	0	0	1	0	1	0	0	0	1	0	0	0	1
**2.8.3.6**	0	0	0	1	1	0	0	0	0	0	0	1	0	0	0	0
**2.8.3.8**	1	3	0	1	0	1	2	3	3	5	2	1	0	1	0	0
**3.1.1.-**	5	5	5	5	3	12	7	7	6	4	3	5	2	6	1	3
**3.1.1.1**	3	2	4	2	4	9	2	6	5	8	8	7	2	5	6	5
**3.1.1.17**	3	0	1	3	3	4	2	2	1	0	1	1	0	1	3	1
**3.1.1.2**	1	1	1	2	0	0	2	1	1	2	0	0	1	4	0	0
**3.1.1.24**	3	1	1	6	1	2	3	1	1	5	1	2	0	1	1	0
**3.1.1.45**	0	0	0	0	0	0	0	0	0	1	0	1	0	1	0	0
**3.1.2.-**	0	1	2	2	2	0	2	2	0	3	2	2	0	2	0	1
**3.1.2.23**	1	0	1	2	2	5	3	1	3	0	6	3	2	1	3	2
**3.1.3.1**	12	3	11	9	5	24	21	14	15	3	14	8	7	9	9	11
**3.1.3.2**	4	1	2	6	2	5	8	8	6	2	7	2	5	3	5	4
**3.1.3.41**	0	0	0	0	0	0	0	0	0	0	1	0	0	0	0	0
**3.2.1.31**	4	3	1	3	1	3	4	1	1	1	1	1	0	2	2	2
**3.3.2.9**	0	0	0	0	0	0	0	0	0	0	0	0	0	0	0	0
**3.5.1.-**	18	9	9	12	9	15	14	7	3	28	9	9	2	8	4	3
**3.5.1.4**	2	4	1	2	1	2	1	1	1	1	1	2	1	2	2	0
**3.5.1.5**	0	0	0	1	0	0	2	2	0	2	0	0	0	0	0	1
**3.5.1.54**	3	3	0	5	0	1	3	2	0	2	0	3	0	5	0	1
**3.5.1.6**	3	0	0	1	0	1	1	0	1	0	0	0	0	0	2	0
**3.5.2.-**	0	0	0	0	0	0	0	0	0	0	0	0	0	0	0	0
**3.5.2.2**	1	3	2	1	0	0	0	4	0	4	0	1	0	2	0	0
**3.5.4.-**	14	4	8	16	11	15	11	10	9	15	15	7	10	10	13	14
**3.5.4.5**	9	1	3	7	7	14	12	5	6	9	6	2	3	4	9	6
**3.5.5.1**	1	1	1	0	1	2	1	1	0	0	0	1	0	0	1	1
**3.5.5.7**	0	0	0	0	0	0	0	0	0	0	0	0	0	0	0	0
**3.5.99.3**	0	2	0	0	0	0	0	0	0	0	0	0	0	0	0	0
**3.6.1.7**	4	2	2	4	0	4	5	1	0	6	2	2	1	4	1	1
**3.7.1.-**	3	2	0	2	0	2	0	1	0	5	0	0	1	0	0	2
**3.7.1.2**	4	0	0	5	4	4	3	2	4	1	4	4	1	3	4	2
**3.8.1.2**	5	1	3	5	3	6	8	5	5	5	4	6	3	4	1	6
**3.8.1.3**	0	0	0	0	0	0	0	0	0	0	0	0	0	0	0	0
**3.8.1.5**	0	1	3	2	0	0	0	1	0	3	0	0	0	3	0	1
**4.1.1.-**	9	7	4	14	6	14	12	11	10	13	7	10	4	8	10	3
**4.1.1.44**	19	1	9	9	12	18	11	13	12	11	17	10	5	13	15	9
**4.1.1.55**	0	0	0	0	0	0	0	0	0	0	0	0	0	0	0	0
**4.1.1.7**	0	0	0	0	0	0	0	0	0	0	0	0	0	0	0	0
**4.1.1.70**	0	1	0	1	0	0	0	0	0	0	0	0	0	0	0	0
**4.1.1.77**	0	0	0	0	0	0	0	0	0	0	0	0	0	0	0	0
**4.1.2.-**	7	5	1	4	1	5	7	15	3	10	4	2	2	7	2	1
**4.1.3.-**	15	4	8	18	3	18	14	11	4	9	9	3	2	6	12	8

EC/KO	CH-DLM023	CH-DLM024	CH-DLM027	CH-DLM028	CH-DOF002	CH-DOF003	CH-DOF004	CH-DOF006	CH-DOF007	CH-DOF008	CH-DOF009	CH-DOF010	CH-DOF011	CH-DOF012	CH-DOF013	CH-DOF014
4.1.3.39	1	1	0	0	0	1	0	1	0	0	0	1	1	1	0	1
4.1.99.-	1	3	0	0	1	1	2	1	0	1	0	0	0	0	0	0
4.2.1.-	46	18	19	36	18	53	42	34	33	27	31	22	10	22	32	27
4.2.1.17	4	1	3	8	1	5	6	1	3	3	1	3	0	3	1	5
4.2.1.80	0	0	0	0	0	0	0	1	1	0	0	0	0	0	0	0
4.2.1.83	0	0	0	1	0	0	0	0	0	0	0	0	0	0	0	0
4.2.1.84	0	0	0	1	0	0	0	0	0	0	0	1	0	0	0	1
5.1.2.2	0	0	0	0	0	0	0	0	0	0	0	0	0	0	0	0
5.2.1.2	0	0	0	0	0	0	0	0	0	0	0	0	0	0	0	0
5.3.3.4	0	0	0	1	0	0	0	0	0	0	0	0	0	0	0	0
5.3.99.-	4	6	2	1	0	4	3	2	0	2	1	2	1	2	1	0
5.4.99.-	0	0	0	0	1	1	0	1	0	0	2	0	0	0	1	0
5.5.1.1	4	1	3	4	2	5	4	4	3	2	4	1	2	0	4	2
5.5.1.2	0	0	0	0	0	0	0	0	0	0	0	0	0	0	0	0
6.2.1.-	0	0	0	1	0	0	0	0	0	0	0	0	0	0	0	0
6.3.5.2	21	11	15	16	10	29	17	12	8	20	12	12	4	10	13	10
K00002	0	1	0	1	0	1	1	0	0	3	1	0	0	2	0	0
K00055	0	1	0	0	0	0	0	0	0	0	0	0	0	0	0	0
K00074	3	1	2	3	3	3	3	1	0	9	5	3	0	3	1	1
K00088	17	8	13	14	7	21	19	12	12	19	9	8	6	16	8	11
K00100	42	26	33	44	22	45	42	36	27	33	31	17	15	41	24	22
K00128	0	1	0	4	0	2	3	0	0	4	0	2	0	6	1	2
K00129	0	0	0	0	0	0	0	0	0	0	0	0	0	0	0	0
K00132	0	1	0	1	0	0	0	0	0	0	0	0	0	1	0	0
K00141	0	0	0	0	0	0	0	0	0	0	0	0	0	0	0	0
K00146	0	0	0	2	0	0	0	0	0	0	0	1	0	0	0	0
K00148	0	0	0	0	0	0	0	0	0	0	0	0	0	0	0	0
K00155	0	1	0	1	0	1	2	0	0	0	0	1	0	1	0	0
K00169	0	0	1	0	0	0	2	0	0	0	0	0	0	0	0	0
K00224	0	0	1	0	0	1	1	0	0	0	0	0	0	0	0	0
K00274	0	0	0	0	0	0	0	0	0	0	0	0	0	0	0	0
K00446	0	0	0	0	0	0	0	0	0	0	0	0	0	0	0	0
K00448	0	0	0	0	0	0	0	0	0	0	0	0	0	0	0	0
K00462	1	0	1	0	0	2	0	2	1	0	0	0	0	0	0	1
K00480	0	0	0	0	0	0	0	0	0	0	0	0	0	0	0	0
K00481	0	0	0	1	0	0	0	0	0	0	0	0	0	0	0	0
K00539	1	0	0	0	0	2	1	0	0	0	0	0	0	0	0	0
K00599	42	13	32	35	10	33	23	13	9	40	26	17	7	17	19	18
K00626	5	1	5	15	2	3	4	3	0	4	3	3	1	5	1	1
K00632	0	1	0	1	0	0	1	1	0	0	0	0	0	1	0	0
K00680	34	9	18	27	12	41	29	32	25	20	17	19	9	22	26	18
K00757	8	7	7	6	5	12	8	6	9	6	9	4	3	4	6	4
K00758	2	3	2	4	0	2	4	1	1	1	1	1	0	2	0	0
K00760	14	5	14	15	7	14	13	8	7	18	13	6	2	16	7	5
K00799	0	1	0	4	0	0	1	0	1	4	0	2	0	1	0	0
K00857	6	1	6	11	10	6	9	3	3	8	7	2	3	9	7	4
K00876	25	8	17	21	13	24	22	15	9	21	16	10	8	12	13	7
K01026	0	3	3	0	0	0	0	1	0	2	1	0	0	0	0	0
K01031	0	0	0	0	0	0	0	0	0	0	0	0	0	0	0	0

EC/KO	CH-DLM023	CH-DLM024	CH-DLM027	CH-DLM028	CH-DOF002	CH-DOF003	CH-DOF004	CH-DOF006	CH-DOF007	CH-DOF008	CH-DOF009	CH-DOF010	CH-DOF011	CH-DOF012	CH-DOF013	CH-DOF014
K01034	0	1	0	0	1	0	1	2	0	0	1	0	0	0	0	0
K01039	1	0	0	1	1	1	0	0	0	0	0	1	0	0	0	0
K01041	4	1	5	5	4	6	9	6	6	2	7	6	4	2	7	6
K01053	0	0	0	0	0	0	0	0	0	0	0	0	0	0	0	0
K01055	0	0	0	0	0	0	0	0	0	0	0	0	0	0	0	0
K01061	0	0	0	0	0	0	0	0	1	1	0	1	0	1	0	0
K01066	1	6	2	5	1	7	2	4	4	9	7	5	1	5	3	3
K01075	0	1	1	1	0	1	3	2	1	1	0	1	0	1	0	0
K01077	9	2	7	7	3	10	15	8	8	4	10	6	3	6	4	8
K01101	4	4	3	3	2	2	1	0	3	2	1	3	0	2	1	1
K01195	7	5	2	10	1	9	8	6	3	4	4	6	0	5	6	4
K01426	1	3	1	1	0	1	1	2	1	0	0	1	0	1	1	1
K01428	2	0	0	0	0	0	1	0	0	2	0	1	0	0	0	1
K01457	0	0	0	1	0	0	0	0	0	0	0	0	0	0	0	0
K01464	1	7	3	2	0	0	1	8	0	5	2	2	0	2	0	0
K01489	15	2	6	16	9	16	13	6	6	12	8	3	5	4	9	8
K01500	0	0	0	0	0	0	0	0	0	0	0	0	0	0	0	0
K01501	4	3	1	0	0	2	2	1	0	0	1	2	0	0	0	1
K01502	0	0	0	0	0	0	0	0	0	0	0	0	0	0	0	0
K01512	8	3	3	7	3	4	5	1	0	7	4	3	1	8	1	2
K01560	4	1	4	3	0	4	7	2	0	7	3	0	0	0	1	2
K01561	1	0	1	0	1	1	1	0	1	0	0	1	0	0	1	0
K01563	0	0	0	0	0	0	0	0	0	0	0	0	0	0	0	0
K01564	2	0	1	1	1	4	1	0	2	0	1	2	0	0	1	0
K01607	8	3	3	11	8	7	6	8	7	3	8	3	3	3	5	4
K01612	0	0	0	0	0	0	0	0	0	0	0	0	0	0	0	0
K01615	13	6	7	14	5	16	14	5	8	9	15	4	2	6	11	8
K01617	0	0	0	0	0	0	0	0	0	0	0	0	0	0	0	0
K01666	4	1	2	4	1	5	4	1	0	2	0	1	1	1	2	4
K01692	0	0	0	1	0	1	1	0	0	0	0	0	0	0	0	0
K01721	0	0	0	0	0	0	0	0	0	0	0	0	0	0	0	0
K01726	9	2	5	3	1	10	6	4	5	3	6	2	3	5	8	3
K01781	0	1	0	0	0	0	2	1	0	0	0	0	0	0	0	0
K01821	0	0	3	3	2	1	1	1	0	2	0	3	0	3	2	0
K01856	0	0	0	0	0	0	0	0	0	0	0	0	0	0	0	0
K01857	0	0	2	0	0	0	0	1	0	0	0	0	0	1	0	0
K01913	0	0	0	0	0	0	0	0	0	0	0	0	0	0	0	0
K01951	19	8	12	18	7	21	12	15	8	19	10	8	3	8	9	8
K02554	0	0	0	1	0	0	0	0	1	0	0	0	0	0	0	0
K03381	0	0	0	0	0	0	0	0	0	0	0	0	0	0	0	0
K03382	0	3	0	1	0	0	0	0	0	0	1	0	0	0	0	0
K03464	0	0	0	0	0	0	0	0	0	0	0	0	0	0	0	0
K03518	7	15	9	7	1	5	10	8	0	11	4	2	2	9	1	3
K03862	0	0	0	1	0	0	0	0	0	0	0	0	0	0	0	0
K04099	0	0	0	1	0	0	0	0	0	0	0	0	0	0	0	0
K04100	0	0	0	0	0	0	0	0	0	0	0	0	0	0	0	0
K04102	0	0	0	0	0	0	0	0	0	0	0	0	0	0	0	0
K04116	0	0	0	0	0	0	0	0	0	0	0	0	0	0	0	0
K05394	0	0	1	0	0	0	0	0	0	0	0	0	0	0	1	0

EC/KO	CH-DLM023	CH-DLM024	CH-DLM027	CH-DLM028	CH-DOF002	CH-DOF003	CH-DOF004	CH-DOF006	CH-DOF007	CH-DOF008	CH-DOF009	CH-DOF010	CH-DOF011	CH-DOF012	CH-DOF013	CH-DOF014
K05549	0	0	0	1	0	0	0	0	0	0	0	0	0	0	0	0
K05783	0	0	0	1	0	0	0	0	0	0	0	0	0	0	0	0
K05797	0	0	0	0	0	0	0	0	0	0	0	0	0	0	0	0
K06281	2	1	1	3	1	0	3	3	2	0	0	2	1	2	1	0
K06446	6	4	3	4	3	5	3	2	1	4	2	3	1	0	1	1
K06912	0	0	0	0	0	0	0	0	0	0	0	0	0	0	0	0
K07535	0	0	0	0	0	0	0	1	0	0	0	0	0	0	0	0
K07536	1	1	1	2	1	1	0	2	0	1	2	0	0	1	2	2
K08689	0	0	0	0	0	0	0	0	0	0	0	0	0	0	0	0
K08710	0	1	0	0	0	0	0	0	0	0	0	1	0	0	0	0
K09461	0	0	1	0	0	0	0	0	0	1	1	0	0	1	0	1
K10217	0	0	0	0	0	0	1	0	0	0	0	0	0	0	0	0
K10218	0	1	0	1	1	0	0	0	0	0	0	1	0	0	0	1
K10220	0	0	0	0	0	0	0	0	0	0	0	0	0	0	0	0
K11180	0	0	0	0	0	0	0	1	0	0	0	0	0	0	0	0
K13953	0	1	4	2	0	1	1	1	0	0	0	1	0	3	0	0
K14333	0	0	0	0	0	0	0	0	0	0	0	0	0	0	0	0
K14519	0	0	0	1	0	0	0	0	0	0	0	0	0	0	0	0
K15054	0	0	0	0	0	0	0	0	0	0	0	0	0	0	0	0
K16173	0	0	0	0	0	0	0	0	0	1	1	1	0	0	0	0
K16514	0	1	0	0	0	0	1	0	0	0	0	1	0	0	0	0
K16874	0	0	0	0	0	0	0	0	0	0	0	0	0	0	0	0

EC/KO	CH-DOM001	CH-DOM003	CH-DOM005	CH-DOM008	CH-DOM010	CH-DOM012	CH-DOM013	CH-DOM014	CH-DOM015	CH-DOM016	CH-DOM017	CH-DOM018	CH-DOM019	CH-DOM020	CH-DOM021
**1.1.1.-**	34	22	40	41	55	26	45	13	24	53	33	38	40	22	26
**1.1.1.1**	18	21	20	18	37	12	36	9	18	35	21	32	31	17	13
**1.1.1.157**	0	0	1	0	1	1	0	0	1	1	1	0	0	0	1
**1.1.1.205**	14	13	11	13	24	10	18	10	15	29	15	18	22	14	11
**1.1.1.35**	3	0	2	0	0	2	0	1	2	3	1	2	0	1	0
**1.12.99.6**	2	2	1	4	3	0	3	0	0	5	0	1	2	1	1
**1.13.11.-**	0	0	0	0	0	0	0	0	0	0	0	0	0	0	0
**1.13.11.1**	0	0	0	0	0	0	0	0	0	0	0	0	0	0	0
**1.13.11.2**	0	0	0	0	0	0	0	0	0	0	0	0	0	0	0
**1.13.11.3**	0	0	0	0	0	0	0	0	0	0	0	0	0	0	0
**1.13.11.39**	0	0	0	0	0	0	0	0	0	0	0	0	0	0	0
**1.13.11.5**	0	0	0	0	0	0	0	1	0	0	1	0	1	0	0
**1.13.11.8**	0	0	0	0	0	0	0	0	0	0	0	0	0	0	0
**1.14.12.10**	0	0	0	0	0	1	0	0	0	1	0	0	0	0	0
**1.14.12.13**	0	0	0	0	0	0	0	0	0	0	0	0	0	0	0
**1.14.12.18**	1	0	0	0	0	0	0	0	0	0	0	0	0	0	0
**1.14.13.-**	2	2	3	1	0	4	0	0	0	2	0	0	0	0	0
**1.14.13.1**	0	0	0	0	0	0	0	0	0	0	0	0	0	0	0
**1.14.13.2**	0	0	0	0	0	0	0	0	0	0	0	0	0	0	0
**1.14.13.50**	0	0	0	0	0	0	0	0	0	0	0	0	0	0	0
**1.14.13.7**	0	0	0	0	0	0	0	0	0	0	0	0	1	0	0
**1.14.13.8**	0	0	0	0	0	0	0	0	0	0	0	0	0	0	0
**1.14.13.82**	0	0	1	0	0	0	0	0	0	0	0	0	0	0	0
**1.14.99.-**	0	0	0	0	0	0	0	0	0	0	0	0	0	0	0
**1.17.99.1**	0	0	0	0	0	1	0	0	0	0	0	0	0	0	0
**1.18.6.1**	0	0	0	0	0	0	0	0	0	0	0	0	0	0	0
**1.2.1.-**	0	2	1	1	0	0	0	0	0	0	0	0	0	0	0
**1.2.1.10**	3	2	4	2	6	3	4	2	3	6	3	5	4	2	0
**1.2.1.3**	5	3	7	8	5	6	6	1	4	5	2	3	5	0	1
**1.2.1.39**	1	0	0	0	0	0	0	0	0	0	0	0	0	0	0
**1.2.7.1**	4	4	7	10	13	5	13	5	6	9	6	3	9	2	2
**1.2.99.2**	3	1	2	5	1	2	1	0	1	6	2	0	0	1	1
**1.3.1.-**	0	0	0	2	0	0	0	0	1	2	0	1	0	0	1
**1.3.1.2**	1	1	1	2	1	0	0	0	1	1	0	0	2	0	0
**1.3.1.25**	0	0	0	0	0	0	0	0	0	0	0	0	0	0	0
**1.3.99.-**	0	0	0	0	0	0	0	0	0	1	0	0	0	0	0
**1.6.5.-**	37	10	31	21	48	23	37	21	25	33	35	31	51	15	40
**1.7.1.-**	5	5	6	7	10	10	9	4	3	13	5	6	5	4	6
**1.8.99.3**	0	0	0	0	0	1	1	0	0	1	0	1	0	0	0
**2.1.1.-**	141	103	108	116	197	144	224	100	120	204	117	174	202	90	94
**2.3.1.-**	124	38	100	103	113	94	120	67	92	116	94	89	130	62	80
**2.3.1.16**	1	0	2	0	4	3	3	0	1	2	1	0	2	0	0
**2.3.1.5**	0	1	1	1	0	0	0	0	0	0	0	0	0	0	0
**2.3.1.9**	2	3	1	3	1	2	0	0	1	1	1	0	0	0	0
**2.4.2.10**	9	8	3	6	15	11	15	7	6	15	9	14	13	4	7
**2.4.2.3**	3	5	4	1	11	4	9	0	4	5	5	7	5	0	1
**2.4.2.4**	1	1	2	1	0	1	4	2	2	3	2	2	1	2	1
**2.4.2.8**	12	7	9	5	8	8	11	5	4	12	5	7	8	4	6
**2.5.1.-**	18	7	12	18	17	17	16	8	7	22	9	12	20	6	11

EC/KO	CH-DOM001	CH-DOM003	CH-DOM005	CH-DOM008	CH-DOM010	CH-DOM012	CH-DOM013	CH-DOM014	CH-DOM015	CH-DOM016	CH-DOM017	CH-DOM018	CH-DOM019	CH-DOM020	CH-DOM021
**2.5.1.18**	1	2	5	5	0	4	0	0	0	9	1	1	1	0	0
**2.6.1.-**	23	15	14	26	28	12	27	9	22	32	27	14	38	16	15
**2.7.1.21**	5	0	6	3	8	3	6	2	3	7	6	2	6	3	4
**2.7.1.48**	22	10	14	15	25	14	24	7	19	25	17	12	20	10	15
**2.7.4.-**	15	8	10	5	18	7	14	4	5	12	8	10	12	6	8
**2.8.3.-**	2	3	1	1	2	0	4	0	1	2	0	1	2	1	3
**2.8.3.1**	0	0	0	0	0	0	0	0	0	0	0	0	0	0	0
**2.8.3.12**	2	0	0	0	1	0	1	0	1	0	0	1	1	0	0
**2.8.3.6**	0	0	0	0	0	1	0	1	0	1	0	0	1	0	1
**2.8.3.8**	2	2	1	0	0	1	3	0	0	1	0	0	1	0	0
**3.1.1.-**	3	2	6	5	6	7	5	1	2	13	4	2	6	1	2
**3.1.1.1**	5	2	4	4	3	3	2	1	5	4	7	5	4	7	5
**3.1.1.17**	3	0	5	1	3	0	2	0	1	3	2	2	2	2	1
**3.1.1.2**	1	0	1	1	0	0	0	0	0	0	0	0	0	0	0
**3.1.1.24**	0	1	1	1	4	1	0	1	2	4	2	4	2	1	0
**3.1.1.45**	1	0	0	1	0	0	0	0	0	1	0	0	0	0	0
**3.1.2.-**	1	1	2	2	0	0	1	0	0	1	0	1	0	0	1
**3.1.2.23**	3	0	4	4	6	2	4	3	0	4	3	1	2	3	7
**3.1.3.1**	18	2	13	17	23	6	19	5	13	21	14	9	16	12	13
**3.1.3.2**	4	2	5	5	8	3	4	2	5	8	4	4	7	6	7
**3.1.3.41**	0	1	0	0	0	0	0	0	0	0	0	0	0	0	0
**3.2.1.31**	1	3	2	2	4	0	0	0	6	6	2	1	1	1	1
**3.3.2.9**	0	0	1	0	0	0	0	0	0	0	0	0	0	0	0
**3.5.1.-**	16	9	7	9	21	10	21	10	11	17	9	9	13	5	7
**3.5.1.4**	2	3	0	0	4	2	2	0	1	4	1	2	2	0	1
**3.5.1.5**	1	0	1	0	0	2	2	0	1	1	0	2	1	0	1
**3.5.1.54**	2	2	1	2	0	4	2	3	2	2	0	1	1	1	0
**3.5.1.6**	1	0	1	2	1	4	1	0	1	3	0	1	3	0	0
**3.5.2.-**	0	0	0	0	0	0	0	0	0	0	0	0	0	0	0
**3.5.2.2**	1	0	1	1	3	0	0	0	0	0	0	0	1	0	0
**3.5.4.-**	17	11	10	13	18	17	20	11	9	17	10	9	16	8	13
**3.5.4.5**	7	1	4	5	11	8	7	7	6	10	8	3	12	5	8
**3.5.5.1**	0	0	1	0	2	0	1	1	0	1	1	0	0	0	0
**3.5.5.7**	0	0	0	0	0	0	0	0	0	0	0	0	0	0	0
**3.5.99.3**	0	0	0	0	1	0	0	0	0	0	0	0	0	0	0
**3.6.1.7**	0	4	1	2	3	0	3	0	1	5	0	7	1	3	1
**3.7.1.-**	0	1	2	0	1	1	2	0	1	2	1	0	1	1	1
**3.7.1.2**	5	0	2	4	8	3	5	2	2	4	4	3	3	3	3
**3.8.1.2**	10	3	6	7	9	5	5	6	7	5	4	5	5	4	4
**3.8.1.3**	0	0	0	0	0	0	0	0	0	0	0	0	0	0	0
**3.8.1.5**	1	1	1	0	2	0	0	0	0	1	1	0	0	0	0
**4.1.1.-**	11	9	12	9	14	4	11	2	9	15	4	12	11	5	10
**4.1.1.44**	13	5	10	8	22	16	22	16	12	21	11	9	17	6	11
**4.1.1.55**	0	0	0	0	0	0	0	0	0	0	0	1	0	0	0
**4.1.1.7**	0	0	0	0	0	0	0	0	0	0	0	0	0	0	0
**4.1.1.70**	0	0	0	1	0	1	0	0	0	0	0	0	0	0	0
**4.1.1.77**	0	0	0	0	0	0	0	0	0	0	0	0	0	0	0
**4.1.2.-**	5	7	2	6	2	3	3	2	4	8	4	3	3	4	1
**4.1.3.-**	7	6	9	10	14	7	10	4	10	18	15	17	14	7	6

EC/KO	CH-DOM001	CH-DOM003	CH-DOM005	CH-DOM008	CH-DOM010	CH-DOM012	CH-DOM013	CH-DOM014	CH-DOM015	CH-DOM016	CH-DOM017	CH-DOM018	CH-DOM019	CH-DOM020	CH-DOM021
**4.1.3.39**	0	0	0	0	0	0	0	0	0	2	1	0	1	0	0
**4.1.99.-**	0	0	2	1	0	0	2	0	1	2	0	0	1	0	0
**4.2.1.-**	49	9	42	38	48	23	43	10	31	51	33	31	43	15	24
**4.2.1.17**	1	1	5	1	4	7	4	4	2	7	5	4	5	2	2
**4.2.1.80**	0	0	1	0	0	0	0	0	0	0	0	0	0	0	0
**4.2.1.83**	0	0	0	0	1	0	0	0	0	1	1	0	0	0	0
**4.2.1.84**	0	0	0	0	0	0	0	0	0	1	0	0	0	0	0
**5.1.2.2**	0	0	0	0	0	0	0	0	0	0	0	0	0	0	0
**5.2.1.2**	0	0	0	0	0	0	0	0	0	0	0	0	0	0	0
**5.3.3.4**	0	0	0	0	0	0	0	0	0	0	0	0	0	0	0
**5.3.99.-**	2	1	5	2	2	1	3	1	1	0	0	1	1	0	1
**5.4.99.-**	1	0	0	0	1	0	0	0	0	0	0	0	0	0	1
**5.5.1.1**	4	0	3	5	5	2	2	1	3	6	3	3	4	1	3
**5.5.1.2**	0	0	0	0	0	0	0	0	0	0	0	0	0	0	0
**6.2.1.-**	0	0	0	0	0	1	0	0	0	0	0	0	0	0	0
**6.3.5.2**	14	11	12	14	27	14	14	7	13	25	11	19	21	9	9
**K00002**	0	1	0	0	0	2	1	0	1	1	0	1	2	0	0
**K00055**	0	0	1	1	0	0	0	0	0	0	0	0	0	0	0
**K00074**	4	1	2	0	1	5	4	1	4	2	4	6	2	2	2
**K00088**	16	9	10	12	21	7	16	7	7	23	10	16	20	7	9
**K00100**	37	19	30	36	64	29	41	11	22	50	25	34	38	19	22
**K00128**	0	3	1	1	0	1	0	0	0	0	0	1	0	0	0
**K00129**	0	0	0	0	0	0	0	0	0	0	0	0	0	0	0
**K00132**	0	0	0	0	0	0	0	0	0	0	0	0	0	0	0
**K00141**	0	0	0	0	0	0	0	0	0	0	0	0	0	0	0
**K00146**	0	0	2	0	0	0	0	0	0	0	0	0	0	0	0
**K00148**	0	0	0	0	0	0	0	0	0	0	0	0	0	0	0
**K00155**	1	0	1	0	0	0	0	0	0	0	0	1	0	0	0
**K00169**	0	1	1	1	1	2	1	0	1	4	1	0	2	0	0
**K00224**	1	0	0	0	0	0	1	0	0	1	0	2	0	0	0
**K00274**	0	0	0	0	0	1	0	0	0	0	0	0	0	0	0
**K00446**	0	0	0	0	0	0	0	0	0	0	0	0	0	0	0
**K00448**	0	0	0	0	0	0	0	0	0	0	0	0	0	0	0
**K00462**	0	0	1	1	0	2	2	0	0	2	1	3	0	0	0
**K00480**	0	0	0	0	0	1	0	0	0	0	0	0	0	0	0
**K00481**	0	0	0	0	0	0	0	0	0	0	0	0	0	0	0
**K00539**	0	0	0	0	0	0	0	0	0	1	0	1	0	0	0
**K00599**	23	19	23	14	37	10	31	10	16	31	27	31	33	14	17
**K00626**	4	6	2	4	5	3	7	1	4	4	2	1	5	0	0
**K00632**	0	0	0	1	0	1	0	0	0	0	0	0	0	0	0
**K00680**	31	16	28	20	37	20	31	20	18	36	36	24	36	19	21
**K00757**	11	6	9	7	17	6	13	4	5	10	6	9	9	4	8
**K00758**	2	2	1	1	1	1	2	0	3	1	0	1	3	0	0
**K00760**	15	7	8	6	12	12	19	9	7	15	7	13	15	7	10
**K00799**	0	1	3	2	0	4	0	0	0	2	0	0	0	0	0
**K00857**	12	1	6	3	10	5	12	6	7	7	6	7	11	4	6
**K00876**	29	9	13	15	26	14	24	9	16	25	12	15	20	12	15
**K01026**	0	1	2	1	1	0	3	0	0	1	0	0	0	0	0
**K01031**	0	0	0	0	0	1	0	0	0	0	0	0	0	0	0

EC/KO	CH-DOM001	CH-DOM003	CH-DOM005	CH-DOM008	CH-DOM010	CH-DOM012	CH-DOM013	CH-DOM014	CH-DOM015	CH-DOM016	CH-DOM017	CH-DOM018	CH-DOM019	CH-DOM020	CH-DOM021
K01034	3	0	1	0	1	2	2	1	0	1	0	0	1	0	0
K01039	1	0	0	1	0	0	0	0	1	0	0	1	1	0	0
K01041	6	2	4	7	13	2	5	0	6	5	6	4	11	6	6
K01053	0	0	1	0	0	0	0	0	0	0	0	0	0	0	0
K01055	0	0	0	0	0	0	0	0	0	0	0	0	0	0	0
K01061	1	0	1	1	0	0	0	0	0	1	0	0	0	0	0
K01066	2	2	6	9	3	1	3	1	2	3	5	4	5	5	5
K01075	1	0	1	1	0	0	0	0	0	1	0	0	0	0	0
K01077	8	2	7	13	13	5	13	4	8	12	7	7	9	6	7
K01101	2	3	1	1	2	6	2	1	2	2	0	1	2	0	1
K01195	5	3	4	6	5	2	4	2	9	7	4	4	5	3	2
K01426	1	0	1	0	3	1	1	0	1	1	1	0	1	0	0
K01428	1	0	0	0	1	1	2	0	0	1	0	1	0	1	2
K01457	0	0	0	0	0	0	0	0	0	0	0	0	0	0	0
K01464	2	1	1	1	3	1	1	1	1	3	1	0	1	0	0
K01489	12	4	6	5	14	9	14	7	8	11	9	6	16	6	9
K01500	0	0	0	0	0	0	0	0	0	0	0	0	0	0	0
K01501	0	0	1	1	1	2	4	1	0	2	1	1	0	0	0
K01502	0	0	0	0	0	0	0	0	0	0	0	0	0	0	0
K01512	1	4	1	2	3	1	6	0	1	6	0	10	2	4	2
K01560	4	1	0	0	3	4	4	1	2	1	1	3	4	1	0
K01561	0	0	0	0	0	0	1	0	1	1	0	1	1	0	1
K01563	0	0	0	0	0	0	0	0	0	0	0	0	0	0	0
K01564	3	1	0	0	3	0	3	0	1	2	1	0	1	0	3
K01607	9	3	6	5	9	8	6	8	4	9	6	4	11	3	5
K01612	0	0	0	0	0	0	0	0	0	0	0	0	0	0	0
K01615	15	4	12	11	20	9	11	2	10	14	12	9	18	12	8
K01617	0	0	0	0	0	0	0	0	0	0	0	0	0	0	0
K01666	0	0	1	0	1	1	4	0	1	5	3	4	5	1	0
K01692	0	0	1	0	0	1	0	0	0	0	0	0	0	0	0
K01721	0	0	0	0	0	0	0	0	0	0	0	0	0	0	0
K01726	8	4	4	9	13	3	5	1	5	7	8	2	5	3	4
K01781	1	0	1	0	1	1	0	0	0	2	0	0	0	0	0
K01821	2	1	0	0	1	1	3	1	1	2	2	1	1	1	1
K01856	0	0	0	0	0	0	0	0	0	0	0	0	0	0	0
K01857	0	0	0	0	0	0	0	0	0	0	0	0	0	0	0
K01913	0	0	0	0	0	1	1	0	0	0	0	0	0	0	0
K01951	14	8	8	12	25	13	15	6	13	16	9	15	20	6	8
K02554	0	0	0	0	0	0	0	0	0	0	0	0	0	0	0
K03381	0	0	0	0	0	0	0	0	0	0	0	0	0	0	0
K03382	0	0	1	0	1	2	0	1	0	0	0	0	2	0	0
K03464	0	0	0	0	0	0	0	0	0	0	0	0	0	0	0
K03518	11	6	11	7	7	6	10	4	7	9	2	9	6	3	1
K03862	0	0	0	0	0	0	0	0	0	0	0	0	0	0	0
K04099	0	0	0	0	0	0	0	0	0	0	0	0	0	0	0
K04100	0	0	0	0	0	0	0	0	0	0	0	0	0	0	0
K04102	0	0	0	0	0	0	0	0	0	0	0	1	0	0	0
K04116	0	0	0	0	0	0	0	0	0	0	0	0	0	0	0
K05394	0	0	0	0	0	2	1	0	0	2	0	2	0	0	0

EC/KO	CH-DOM001	CH-DOM003	CH-DOM005	CH-DOM008	CH-DOM010	CH-DOM012	CH-DOM013	CH-DOM014	CH-DOM015	CH-DOM016	CH-DOM017	CH-DOM018	CH-DOM019	CH-DOM020	CH-DOM021
**K05549**	0	0	0	0	0	0	0	0	0	0	0	0	0	0	0
**K05783**	0	0	0	0	0	0	0	0	0	0	0	0	0	0	0
**K05797**	0	0	0	0	0	1	0	0	0	0	0	0	0	0	0
**K06281**	1	2	1	3	2	0	2	0	0	5	0	1	2	1	1
**K06446**	5	5	1	3	6	4	5	2	4	7	5	5	5	3	5
**K06912**	0	0	0	0	0	1	0	0	0	0	0	0	0	0	0
**K07535**	0	0	1	0	1	0	0	0	0	0	0	0	0	0	0
**K07536**	1	1	2	2	1	1	2	0	0	5	0	2	1	1	1
**K08689**	1	0	0	0	0	0	0	0	0	0	0	0	0	0	0
**K08710**	0	0	1	1	0	0	0	0	0	1	0	0	0	0	0
**K09461**	0	0	0	0	0	0	0	0	0	0	0	0	0	0	0
**K10217**	0	0	1	0	0	0	0	0	0	0	0	0	0	0	0
**K10218**	1	0	1	0	1	0	0	0	1	0	0	0	0	0	1
**K10220**	0	0	0	0	0	1	0	0	0	0	0	0	0	0	0
**K11180**	0	0	0	0	1	0	0	0	0	1	0	0	0	0	0
**K13953**	0	1	0	0	0	0	0	0	0	0	0	0	1	0	0
**K14333**	0	0	0	0	0	0	0	0	0	0	0	0	0	0	0
**K14519**	0	0	0	0	0	0	0	0	0	0	0	0	0	0	0
**K15054**	0	0	0	0	0	0	0	0	0	0	0	0	0	0	0
**K16173**	0	0	0	0	1	0	2	0	0	1	0	0	0	0	0
**K16514**	0	0	0	1	0	0	1	0	0	1	0	0	0	0	0
**K16874**	0	0	0	0	0	0	0	0	0	0	0	0	0	0	0

EC/KO	CH-DOM022	CH-DOM023	CH-DOM024	CH-DOM025	CH-DOM026	CH-NLF001	CH-NLF002	CH-NLF005	CH-NLF006	CH-NLF007	CH-NLF008	CH-NLF009	CH-NLF010	CH-NLF011	CH-NLF012	CH-NLF013
**1.1.1.-**	63	18	18	34	44	35	27	24	17	42	42	31	24	13	42	42
**1.1.1.1**	43	14	11	16	40	17	16	22	13	67	26	21	12	23	43	36
**1.1.1.157**	1	0	0	0	0	0	0	0	1	1	0	1	0	0	0	2
**1.1.1.205**	26	8	16	11	18	11	13	14	10	34	22	14	8	14	23	24
**1.1.1.35**	1	0	1	0	0	1	0	2	0	3	0	0	1	1	1	2
**1.12.99.6**	4	1	1	0	0	1	1	0	0	1	3	0	1	0	0	2
**1.13.11.-**	0	0	0	0	0	0	0	0	0	0	0	0	0	0	0	0
**1.13.11.1**	0	0	0	1	0	0	0	0	0	0	0	0	0	0	0	0
**1.13.11.2**	0	0	0	0	0	0	0	0	0	0	0	0	0	0	0	0
**1.13.11.3**	0	0	0	0	0	0	0	0	0	0	0	0	0	0	0	0
**1.13.11.39**	0	0	0	0	0	0	0	0	0	0	0	0	0	0	0	0
**1.13.11.5**	0	0	0	0	0	0	0	0	0	0	0	0	0	0	0	0
**1.13.11.8**	0	0	0	1	0	0	0	0	0	0	0	0	0	0	0	0
**1.14.12.10**	0	0	0	2	0	0	0	1	0	0	0	1	0	0	0	0
**1.14.12.13**	0	0	0	0	0	0	0	0	0	0	0	0	0	0	0	0
**1.14.12.18**	0	0	0	0	0	0	0	0	0	0	0	0	0	0	0	0
**1.14.13.-**	0	0	0	3	0	0	0	0	0	0	3	0	0	0	0	0
**1.14.13.1**	0	0	0	0	0	0	0	0	0	0	0	0	0	0	0	0
**1.14.13.2**	0	0	0	1	0	0	0	0	0	0	0	0	0	0	0	0
**1.14.13.50**	0	0	0	0	0	0	0	0	0	0	0	0	0	0	0	0
**1.14.13.7**	0	0	0	0	0	0	0	0	0	0	0	0	0	0	1	0
**1.14.13.8**	0	0	0	0	0	0	0	0	0	0	0	0	0	0	0	0
**1.14.13.82**	0	0	0	1	0	0	0	0	0	0	0	0	0	0	0	0
**1.14.99.-**	0	0	0	0	0	0	0	0	0	0	0	0	0	0	0	0
**1.17.99.1**	0	0	0	0	0	0	0	0	0	0	0	0	0	0	0	0
**1.18.6.1**	0	0	0	0	0	0	0	1	0	0	0	0	0	0	0	0
**1.2.1.-**	0	0	0	2	0	0	0	0	0	0	0	0	0	0	0	0
**1.2.1.10**	3	0	4	3	5	1	4	10	0	6	0	0	2	2	8	5
**1.2.1.3**	6	1	2	6	5	1	2	3	0	4	9	4	2	1	5	8
**1.2.1.39**	0	0	0	1	0	0	0	0	0	0	0	0	0	0	0	0
**1.2.7.1**	10	4	6	5	4	3	2	7	3	11	10	6	2	9	8	15
**1.2.99.2**	4	1	1	3	5	0	1	3	0	5	4	0	1	1	0	4
**1.3.1.-**	0	2	3	0	0	0	0	1	0	0	0	2	1	1	0	0
**1.3.1.2**	0	0	0	0	0	0	0	0	0	1	0	1	0	0	2	0
**1.3.1.25**	0	0	0	0	0	0	0	0	0	0	0	0	0	0	0	0
**1.3.99.-**	0	0	0	0	0	0	0	0	0	0	0	0	0	0	0	0
**1.6.5.-**	40	21	24	33	27	28	18	30	16	47	35	39	16	20	35	32
**1.7.1.-**	9	5	4	5	7	3	5	8	6	8	7	8	7	4	10	9
**1.8.99.3**	1	0	0	1	0	0	0	0	0	0	0	0	0	0	0	0
**2.1.1.-**	231	76	96	110	186	91	92	166	76	233	133	127	78	122	183	230
**2.3.1.-**	112	58	72	101	97	81	68	105	39	128	100	115	66	74	106	130
**2.3.1.16**	1	0	0	1	1	1	1	2	0	5	0	0	0	3	2	3
**2.3.1.5**	0	0	0	0	0	0	0	0	0	0	1	0	0	0	0	0
**2.3.1.9**	3	0	0	0	1	1	0	0	0	0	2	0	1	0	1	1
**2.4.2.10**	13	4	7	8	13	9	5	11	6	14	6	7	4	6	12	12
**2.4.2.3**	8	3	4	1	6	5	4	3	1	13	2	5	4	1	8	6
**2.4.2.4**	4	1	1	0	0	1	1	3	0	2	1	0	1	2	3	2
**2.4.2.8**	11	4	5	7	10	5	3	8	6	12	5	10	3	8	6	10
**2.5.1.-**	16	9	12	13	13	11	10	14	13	22	20	20	12	11	17	18

EC/KO	CH-DOM022	CH-DOM023	CH-DOM024	CH-DOM025	CH-DOM026	CH-NLF001	CH-NLF002	CH-NLF005	CH-NLF006	CH-NLF007	CH-NLF008	CH-NLF009	CH-NLF010	CH-NLF011	CH-NLF012	CH-NLF013
**2.5.1.18**	1	0	0	7	1	0	1	1	0	1	4	1	0	0	0	1
**2.6.1.-**	33	12	16	18	22	18	14	26	15	35	22	29	21	19	23	28
**2.7.1.21**	3	3	5	4	3	7	4	7	3	6	4	8	6	3	4	5
**2.7.1.48**	23	11	15	17	23	15	8	20	12	27	20	15	10	15	19	24
**2.7.4.-**	13	4	4	6	13	8	6	12	6	23	8	11	3	10	14	10
**2.8.3.-**	2	1	0	1	1	1	1	0	0	2	4	0	1	0	1	1
**2.8.3.1**	0	0	0	0	0	1	0	0	0	0	2	0	0	0	0	0
**2.8.3.12**	1	0	0	0	0	0	0	0	0	2	0	0	0	0	0	0
**2.8.3.6**	0	0	1	1	0	1	0	0	0	0	1	0	0	0	0	0
**2.8.3.8**	2	0	0	1	0	0	0	0	0	0	1	0	0	0	1	1
**3.1.1.-**	5	2	3	5	6	3	4	2	2	2	5	3	3	0	5	5
**3.1.1.1**	7	3	2	3	10	5	4	2	1	3	7	5	4	3	4	6
**3.1.1.17**	3	0	2	7	3	2	2	1	0	1	1	1	1	2	2	2
**3.1.1.2**	1	0	0	0	0	0	0	0	0	2	1	0	1	0	0	1
**3.1.1.24**	1	0	0	0	3	2	0	1	0	4	0	2	1	1	2	3
**3.1.1.45**	0	0	0	1	0	0	0	0	0	0	1	0	0	0	0	0
**3.1.2.-**	0	0	0	1	0	0	0	0	0	0	3	1	0	0	0	0
**3.1.2.23**	3	2	4	5	3	4	2	1	2	4	6	6	3	5	2	1
**3.1.3.1**	22	10	13	8	13	7	11	10	8	12	18	10	7	15	17	20
**3.1.3.2**	11	4	4	5	4	3	3	5	4	6	5	5	3	5	4	5
**3.1.3.41**	0	0	0	0	0	0	0	0	0	0	0	0	0	0	0	0
**3.2.1.31**	1	2	1	1	1	3	4	3	0	3	1	0	2	2	3	7
**3.3.2.9**	0	0	0	0	0	0	0	0	0	0	0	0	0	0	0	0
**3.5.1.-**	14	5	4	10	13	9	4	19	3	20	10	9	6	6	17	21
**3.5.1.4**	2	1	1	0	2	2	1	2	0	0	1	0	2	0	2	4
**3.5.1.5**	0	0	1	1	2	0	0	1	0	2	1	0	0	0	2	0
**3.5.1.54**	2	0	0	1	1	1	0	0	0	2	1	0	0	1	1	0
**3.5.1.6**	4	0	0	0	0	1	1	3	0	2	1	0	0	0	4	0
**3.5.2.-**	0	0	0	0	0	0	0	0	0	0	0	0	0	0	0	0
**3.5.2.2**	3	0	0	0	3	0	0	0	0	1	1	0	0	1	1	0
**3.5.4.-**	21	9	7	10	16	10	8	7	7	18	17	12	7	10	13	19
**3.5.4.5**	9	3	5	6	7	4	6	9	3	14	7	13	5	5	8	14
**3.5.5.1**	1	0	0	0	0	0	0	0	0	1	1	0	0	0	0	1
**3.5.5.7**	0	0	0	0	0	0	0	0	0	0	0	0	0	0	0	0
**3.5.99.3**	1	0	0	0	0	0	0	0	0	0	0	0	0	0	0	0
**3.6.1.7**	3	0	0	1	6	1	1	1	0	6	2	3	1	1	3	5
**3.7.1.-**	1	1	0	4	0	1	1	0	0	1	0	0	1	1	2	0
**3.7.1.2**	7	3	3	7	0	3	2	2	2	2	7	4	1	3	4	5
**3.8.1.2**	6	3	4	4	8	4	4	3	2	11	9	8	3	2	6	6
**3.8.1.3**	0	0	0	0	0	0	0	0	0	0	0	0	0	0	0	0
**3.8.1.5**	0	0	0	1	0	1	0	0	0	0	0	0	0	0	0	1
**4.1.1.-**	11	7	6	12	4	3	1	6	3	8	11	4	4	4	12	5
**4.1.1.44**	21	7	9	17	12	15	9	8	7	16	13	14	9	9	16	20
**4.1.1.55**	0	0	0	0	0	0	0	0	0	0	0	0	0	0	0	0
**4.1.1.7**	0	0	0	0	0	0	0	0	0	0	0	0	0	0	0	0
**4.1.1.70**	0	0	0	0	0	0	0	0	0	0	0	0	0	0	0	0
**4.1.1.77**	0	0	0	0	0	0	0	0	0	0	0	0	0	0	0	0
**4.1.2.-**	1	1	1	4	7	1	3	2	1	6	6	2	0	2	7	2
**4.1.3.-**	13	8	6	11	16	5	9	12	4	23	7	13	7	11	16	20

EC/KO	CH-DOM022	CH-DOM023	CH-DOM024	CH-DOM025	CH-DOM026	CH-NLF001	CH-NLF002	CH-NLF005	CH-NLF006	CH-NLF007	CH-NLF008	CH-NLF009	CH-NLF010	CH-NLF011	CH-NLF012	CH-NLF013
4.1.3.39	0	0	0	0	0	1	0	1	0	1	0	0	1	1	0	0
4.1.99.-	2	0	1	0	1	2	0	1	0	1	0	0	0	1	2	4
4.2.1.-	53	12	19	32	32	25	33	32	13	53	42	42	22	20	41	33
4.2.1.17	4	0	1	4	3	4	2	1	0	5	2	5	3	1	3	3
4.2.1.80	0	0	0	0	0	0	0	0	0	0	0	0	0	0	0	0
4.2.1.83	1	0	0	0	0	0	1	1	0	1	0	0	1	1	1	0
4.2.1.84	1	0	0	0	0	0	1	0	1	0	0	0	0	0	0	0
5.1.2.2	1	0	0	0	0	0	0	0	0	0	0	0	0	0	0	0
5.2.1.2	0	0	0	0	0	0	0	0	0	0	0	0	0	0	0	0
5.3.3.4	0	0	0	0	0	0	0	0	0	0	0	0	0	0	0	0
5.3.99.-	5	0	1	2	3	1	0	0	0	0	2	3	0	0	0	1
5.4.99.-	0	1	0	1	0	0	0	1	2	0	1	2	0	0	1	0
5.5.1.1	4	2	2	4	2	3	3	2	1	2	5	3	2	1	3	2
5.5.1.2	0	0	0	0	0	0	0	0	0	0	0	0	0	0	0	0
6.2.1.-	0	0	0	0	0	0	0	0	0	0	0	0	0	0	0	0
6.3.5.2	23	8	10	12	18	8	11	13	6	21	16	12	9	12	20	24
K00002	1	0	0	0	2	0	0	0	0	0	0	0	0	1	0	2
K00055	1	0	0	1	0	0	0	1	0	0	0	0	0	0	0	0
K00074	4	0	2	2	1	2	1	3	2	8	0	1	1	2	3	6
K00088	24	7	8	10	16	9	11	12	5	22	18	10	6	11	20	18
K00100	61	18	19	32	33	21	20	24	18	45	42	38	16	26	36	28
K00128	0	0	0	1	0	0	0	1	0	1	1	0	0	0	1	0
K00129	0	0	0	0	0	0	0	0	0	0	0	0	0	0	0	0
K00132	0	0	0	0	0	0	0	0	0	0	0	0	1	0	0	0
K00141	0	0	0	0	0	0	0	0	0	0	0	0	0	0	0	0
K00146	0	0	0	0	0	0	0	0	0	0	0	0	0	0	0	0
K00148	0	0	0	0	0	0	0	0	0	0	0	0	0	0	0	0
K00155	1	0	0	0	0	0	0	0	0	0	1	0	0	0	0	0
K00169	0	0	0	2	0	0	0	1	0	1	1	0	0	3	1	4
K00224	1	0	0	0	0	0	0	0	0	0	1	1	0	0	0	1
K00274	0	0	0	0	0	0	0	0	0	0	0	0	0	0	0	0
K00446	0	0	0	0	0	0	0	0	0	0	0	0	0	0	0	0
K00448	0	0	0	0	0	0	0	0	0	0	0	0	0	0	0	0
K00462	1	0	0	0	1	1	1	0	0	0	2	0	1	0	1	2
K00480	0	0	0	0	0	0	0	0	0	0	0	0	0	0	0	0
K00481	0	0	0	1	0	0	0	0	0	0	0	0	0	0	0	0
K00539	1	0	0	0	0	0	0	0	0	1	1	0	0	0	0	1
K00599	38	7	13	16	41	18	17	32	13	41	20	26	16	12	33	40
K00626	5	1	0	2	4	4	1	3	1	6	2	0	1	3	4	4
K00632	0	0	0	0	0	0	0	0	0	0	1	0	0	0	0	0
K00680	28	16	17	29	24	19	18	13	11	45	21	28	17	20	32	27
K00757	13	5	5	7	7	4	6	5	3	11	8	5	6	4	9	4
K00758	1	0	0	1	3	2	1	1	0	1	2	0	1	0	0	1
K00760	12	6	10	12	15	6	6	10	5	22	8	12	4	10	10	14
K00799	0	0	0	4	0	0	0	1	0	0	1	0	0	0	0	0
K00857	6	3	6	6	5	7	4	9	5	10	6	11	6	3	6	6
K00876	22	13	13	16	20	13	8	16	11	26	20	16	9	13	21	21
K01026	2	0	0	0	1	0	0	0	0	0	0	0	0	0	1	0
K01031	0	0	0	0	0	0	0	0	0	0	0	0	0	0	0	0

EC/KO	CH-DOM022	CH-DOM023	CH-DOM024	CH-DOM025	CH-DOM026	CH-NLF001	CH-NLF002	CH-NLF005	CH-NLF006	CH-NLF007	CH-NLF008	CH-NLF009	CH-NLF010	CH-NLF011	CH-NLF012	CH-NLF013
**K01034**	0	1	0	2	0	0	0	1	0	0	2	1	0	0	0	0
**K01039**	1	0	0	1	0	0	0	0	0	0	0	0	0	0	0	0
**K01041**	7	5	7	4	3	3	4	7	4	10	8	5	5	7	5	6
**K01053**	0	0	0	1	0	0	0	0	0	0	0	0	0	0	0	0
**K01055**	0	0	0	0	0	0	0	0	0	0	0	0	0	0	0	0
**K01061**	0	0	0	2	0	0	0	0	0	0	1	0	0	0	0	0
**K01066**	4	2	3	3	8	2	4	1	1	3	6	4	1	2	2	4
**K01075**	1	0	0	1	0	0	0	1	0	1	1	0	0	0	0	2
**K01077**	9	6	8	7	7	5	4	3	0	5	13	9	5	6	10	9
**K01101**	0	0	1	2	3	1	2	0	0	1	2	0	0	1	3	0
**K01195**	5	1	2	4	7	6	7	4	0	9	4	6	5	5	5	9
**K01426**	1	1	0	0	0	0	0	0	0	0	2	0	0	0	1	0
**K01428**	0	0	1	0	1	0	0	1	0	1	0	0	1	0	1	0
**K01457**	0	0	0	0	0	0	0	0	0	0	0	0	0	0	0	0
**K01464**	3	0	0	0	4	0	0	0	0	1	2	0	0	1	1	0
**K01489**	12	5	6	9	8	5	8	11	6	17	9	15	6	6	10	15
**K01500**	0	0	0	0	0	0	0	0	0	0	0	0	0	0	0	0
**K01501**	1	0	0	0	0	0	0	0	0	0	1	0	0	0	0	1
**K01502**	0	0	0	0	0	0	0	0	0	0	0	0	0	0	0	0
**K01512**	6	0	0	1	7	2	1	2	2	8	2	4	1	2	5	5
**K01560**	0	0	0	2	4	2	2	0	0	3	1	1	1	2	1	2
**K01561**	1	0	0	0	0	1	0	0	0	0	1	0	0	0	1	0
**K01563**	0	0	0	0	0	0	0	0	0	0	0	0	0	0	0	0
**K01564**	0	2	0	1	0	2	1	0	0	0	4	1	0	0	2	0
**K01607**	10	4	5	6	7	4	5	6	4	12	4	5	5	2	8	10
**K01612**	0	0	0	0	0	0	0	0	0	0	0	0	0	0	0	0
**K01615**	11	12	11	12	8	5	8	10	5	21	14	16	7	11	13	17
**K01617**	0	0	0	0	0	0	0	0	0	0	0	0	0	0	0	0
**K01666**	1	2	5	1	5	4	3	5	1	11	0	3	1	6	5	11
**K01692**	0	0	0	0	0	0	0	0	0	0	0	0	0	0	0	0
**K01721**	0	0	0	0	0	0	0	0	0	0	0	0	0	0	0	0
**K01726**	8	5	6	3	4	5	8	4	3	5	8	7	5	4	2	3
**K01781**	0	0	0	0	0	0	0	0	0	0	0	0	0	0	0	1
**K01821**	4	1	0	2	0	1	1	1	1	0	1	3	0	0	2	2
**K01856**	0	0	0	0	0	0	0	0	0	0	0	0	0	0	0	0
**K01857**	0	0	0	0	0	0	0	0	0	0	1	0	0	0	0	0
**K01913**	0	0	0	0	0	0	0	0	0	1	0	0	0	0	1	0
**K01951**	22	6	7	12	14	7	9	11	4	18	16	8	4	9	18	16
**K02554**	0	0	0	0	0	0	0	0	0	0	0	0	0	0	0	0
**K03381**	0	0	0	1	0	0	0	0	0	0	0	0	0	0	0	0
**K03382**	2	0	0	0	0	0	0	0	0	0	0	0	0	0	0	0
**K03464**	0	0	0	0	0	0	0	0	0	0	0	0	0	0	0	0
**K03518**	17	2	3	4	12	4	4	3	1	6	4	2	3	2	5	7
**K03862**	0	0	0	0	0	0	0	0	0	0	0	0	0	0	0	0
**K04099**	0	0	0	1	0	0	0	0	0	0	0	0	0	0	0	0
**K04100**	0	0	0	0	0	0	0	0	0	0	0	0	0	0	0	0
**K04102**	0	0	0	0	0	0	0	0	0	0	0	0	0	0	0	0
**K04116**	0	0	0	0	0	0	0	0	0	0	0	0	0	0	0	0
**K05394**	1	0	0	0	0	0	0	0	0	0	0	0	0	0	0	1

EC/KO	CH-DOM022	CH-DOM023	CH-DOM024	CH-DOM025	CH-DOM026	CH-NLF001	CH-NLF002	CH-NLF005	CH-NLF006	CH-NLF007	CH-NLF008	CH-NLF009	CH-NLF010	CH-NLF011	CH-NLF012	CH-NLF013
K05549	0	0	0	1	0	0	0	0	0	0	0	0	0	0	0	0
K05783	0	0	0	0	0	0	0	0	0	0	0	0	0	0	0	0
K05797	0	0	0	0	0	0	0	0	0	0	0	0	0	0	0	0
K06281	4	1	1	0	0	1	1	0	0	1	4	0	1	0	0	1
K06446	3	1	1	3	1	6	5	4	1	3	3	2	0	2	7	4
K06912	0	0	0	0	0	0	0	0	0	0	0	0	0	0	0	0
K07535	0	0	0	0	0	0	0	0	0	0	0	0	0	0	0	0
K07536	6	2	1	1	2	1	2	1	1	2	3	2	1	1	0	1
K08689	0	0	0	0	0	0	0	0	0	0	0	0	0	0	0	0
K08710	0	0	0	0	0	0	0	0	0	0	0	0	0	0	0	0
K09461	0	0	0	0	0	0	0	0	0	0	0	0	0	0	0	0
K10217	0	0	0	0	0	0	0	0	0	0	0	0	0	0	0	0
K10218	2	0	0	1	0	0	0	0	0	0	0	0	0	0	0	0
K10220	0	0	0	0	0	0	0	0	0	0	0	0	0	0	0	0
K11180	1	0	0	0	0	0	0	0	0	0	0	0	0	0	0	0
K13953	1	0	0	1	0	0	0	0	0	0	1	0	0	0	0	0
K14333	0	0	0	1	0	0	0	0	0	0	0	0	0	0	0	0
K14519	0	0	0	0	0	0	0	0	0	0	0	0	0	0	0	0
K15054	0	0	0	1	0	0	0	0	0	0	0	0	0	0	0	0
K16173	1	0	0	0	0	0	0	0	0	0	0	0	0	0	1	0
K16514	0	0	0	0	0	0	0	0	0	0	1	0	0	0	0	0
K16874	0	0	0	0	0	0	0	0	0	0	0	0	0	0	0	0

EC/KO	CH-NLF014	CH-NLF015	CH-NLM001	CH-NLM002	CH-NLM003	CH-NLM004	CH-NLM005	CH-NLM006	CH-NLM007	CH-NLM008	CH-NLM009	CH-NLM010	CH-NLM015	CH-NLM016	CH-NLM017	CH-NLM021
**1.1.1.-**	24	43	20	31	11	36	16	57	34	25	17	44	31	5	40	52
**1.1.1.1**	11	24	21	25	14	42	8	41	25	20	9	35	19	7	29	50
**1.1.1.157**	0	0	0	0	0	2	0	0	0	0	0	2	0	0	1	0
**1.1.1.205**	8	16	10	11	4	21	5	19	17	12	12	26	13	3	19	29
**1.1.1.35**	0	1	0	0	1	0	0	2	2	0	1	2	2	0	0	2
**1.12.99.6**	0	1	0	2	0	0	0	7	2	0	0	2	2	0	2	1
**1.13.11.-**	0	0	0	0	0	0	0	0	0	0	0	0	0	0	0	0
**1.13.11.1**	0	0	0	0	0	0	0	0	0	0	0	0	0	0	0	0
**1.13.11.2**	0	0	0	0	0	0	0	0	0	0	0	0	0	0	0	0
**1.13.11.3**	0	0	0	0	0	0	0	0	0	0	0	0	0	0	0	0
**1.13.11.39**	0	0	0	0	0	0	0	0	0	0	0	0	0	0	0	0
**1.13.11.5**	0	0	0	0	0	0	0	0	0	0	0	0	0	0	0	1
**1.13.11.8**	0	0	0	0	0	0	0	2	0	0	0	0	0	0	0	0
**1.14.12.10**	0	0	0	1	1	0	0	3	0	0	0	0	1	0	0	0
**1.14.12.13**	0	0	0	0	0	0	0	1	0	0	0	0	0	0	0	0
**1.14.12.18**	0	0	0	0	0	0	0	0	0	0	0	0	0	0	0	0
**1.14.13.-**	0	0	0	3	0	0	0	2	0	0	0	0	2	0	0	0
**1.14.13.1**	0	0	0	0	0	0	0	0	0	0	0	0	0	0	0	0
**1.14.13.2**	0	0	0	0	0	0	0	0	0	0	0	0	0	0	0	0
**1.14.13.50**	0	0	0	0	0	0	0	0	0	0	0	0	0	0	0	0
**1.14.13.7**	1	0	0	0	0	1	0	1	0	1	0	0	0	0	0	0
**1.14.13.8**	0	0	0	0	0	0	0	0	0	0	0	0	0	0	0	0
**1.14.13.82**	0	0	0	0	0	0	0	0	0	0	0	0	0	0	0	0
**1.14.99.-**	0	0	0	0	0	0	0	0	0	0	0	0	0	0	0	0
**1.17.99.1**	0	0	0	0	0	0	0	0	0	0	0	0	0	0	0	0
**1.18.6.1**	0	0	0	0	0	0	0	0	0	0	0	0	1	0	0	0
**1.2.1.-**	0	0	0	1	0	0	0	1	0	0	0	0	0	0	0	0
**1.2.1.10**	0	3	1	3	0	6	2	4	3	5	2	5	2	1	6	6
**1.2.1.3**	2	3	3	4	2	9	0	4	5	5	1	6	4	1	7	5
**1.2.1.39**	0	0	0	1	0	0	0	0	0	0	0	0	0	0	0	0
**1.2.7.1**	5	8	7	5	2	9	3	7	4	4	2	10	5	4	7	16
**1.2.99.2**	0	2	4	4	3	4	0	2	3	2	0	1	4	1	4	2
**1.3.1.-**	0	0	0	1	0	0	1	1	1	0	0	0	0	1	0	0
**1.3.1.2**	0	0	0	1	0	0	0	1	1	0	0	1	0	0	0	0
**1.3.1.25**	0	0	0	0	0	0	0	0	0	0	0	0	0	0	0	0
**1.3.99.-**	0	0	0	0	0	0	0	0	0	0	0	0	0	0	0	0
**1.6.5.-**	27	29	15	19	8	33	15	63	36	21	11	44	19	12	38	50
**1.7.1.-**	9	7	6	5	2	9	5	16	6	5	6	10	6	3	8	5
**1.8.99.3**	0	0	1	0	0	1	0	0	1	0	0	0	0	0	0	0
**2.1.1.-**	82	132	96	107	48	195	69	236	150	111	75	200	101	47	160	256
**2.3.1.-**	82	96	62	64	45	93	68	171	78	64	39	139	70	21	113	157
**2.3.1.16**	0	4	3	2	0	2	0	0	1	0	0	2	1	0	1	4
**2.3.1.5**	0	0	0	0	0	0	0	0	0	0	0	0	1	0	0	0
**2.3.1.9**	0	1	0	3	2	1	0	2	0	1	1	1	2	1	0	1
**2.4.2.10**	6	7	3	7	3	13	2	12	9	3	4	14	4	2	6	21
**2.4.2.3**	2	2	3	4	0	11	3	5	4	3	4	5	1	0	7	14
**2.4.2.4**	0	2	1	0	1	2	0	1	1	0	0	3	3	1	0	4
**2.4.2.8**	4	5	7	6	1	13	6	14	7	4	4	11	5	4	14	11
**2.5.1.-**	9	11	13	11	6	14	11	40	15	8	11	19	10	5	15	20

EC/KO	CH-NLF014	CH-NLF015	CH-NLM001	CH-NLM002	CH-NLM003	CH-NLM004	CH-NLM005	CH-NLM006	CH-NLM007	CH-NLM008	CH-NLM009	CH-NLM010	CH-NLM015	CH-NLM016	CH-NLM017	CH-NLM021
**2.5.1.18**	0	1	2	4	6	1	0	10	2	0	0	0	5	0	0	0
**2.6.1.-**	15	27	21	12	5	20	13	40	15	12	13	32	14	7	29	33
**2.7.1.21**	5	3	6	5	2	2	1	13	5	2	1	4	2	0	5	7
**2.7.1.48**	13	19	11	12	7	15	9	29	15	13	10	28	11	3	20	23
**2.7.4.-**	6	10	7	7	2	14	6	15	8	8	7	10	5	4	13	17
**2.8.3.-**	1	1	2	1	1	1	0	5	2	0	0	2	1	0	3	1
**2.8.3.1**	0	0	0	0	0	0	0	1	0	0	0	0	0	1	0	0
**2.8.3.12**	0	1	0	0	0	0	0	0	0	0	0	0	0	0	2	1
**2.8.3.6**	1	0	0	0	0	0	0	1	0	0	0	0	1	0	0	0
**2.8.3.8**	0	1	1	2	3	3	1	3	0	0	0	1	2	0	2	1
**3.1.1.-**	2	4	4	8	2	3	2	11	6	1	0	3	5	1	6	2
**3.1.1.1**	6	4	4	1	4	1	3	6	6	4	0	9	2	0	8	4
**3.1.1.17**	1	2	3	0	2	0	1	0	1	0	0	1	0	0	3	1
**3.1.1.2**	0	0	0	1	1	0	0	2	0	0	0	0	1	0	0	0
**3.1.1.24**	0	0	2	2	1	2	0	3	2	0	1	0	2	1	1	3
**3.1.1.45**	0	1	0	0	0	0	0	2	0	0	0	0	1	0	0	0
**3.1.2.-**	0	1	1	1	2	0	1	2	3	0	0	1	2	0	0	0
**3.1.2.23**	5	4	2	1	1	0	4	10	2	1	3	5	2	1	6	5
**3.1.3.1**	11	17	12	9	5	3	4	23	18	10	8	18	10	2	19	17
**3.1.3.2**	5	6	5	8	4	6	3	10	7	4	4	5	6	1	5	6
**3.1.3.41**	0	0	0	0	0	0	0	0	0	0	0	0	0	0	0	0
**3.2.1.31**	1	1	2	0	1	1	1	4	1	1	2	3	1	0	1	5
**3.3.2.9**	0	0	0	0	0	0	0	0	0	0	0	0	0	0	0	0
**3.5.1.-**	3	8	10	8	1	24	6	21	14	7	9	14	6	5	11	21
**3.5.1.4**	1	1	1	2	0	2	1	4	1	0	1	2	1	1	1	3
**3.5.1.5**	0	0	0	0	1	2	0	1	0	0	0	2	0	0	1	1
**3.5.1.54**	0	2	0	2	1	3	0	3	2	1	1	1	1	0	2	5
**3.5.1.6**	1	1	0	2	0	2	1	0	1	0	0	2	1	0	0	1
**3.5.2.-**	0	0	0	1	0	1	0	0	0	0	0	0	0	0	0	0
**3.5.2.2**	0	0	0	1	0	3	0	2	2	0	1	0	0	0	0	1
**3.5.4.-**	8	7	10	11	4	20	6	22	11	10	9	14	9	3	16	13
**3.5.4.5**	6	8	6	4	3	6	3	10	4	4	4	10	5	1	7	14
**3.5.5.1**	0	1	0	1	1	1	0	1	3	0	0	0	0	0	1	2
**3.5.5.7**	0	0	0	0	0	0	0	0	0	0	0	0	0	0	0	0
**3.5.99.3**	0	0	0	0	0	0	0	0	0	0	0	0	0	0	0	0
**3.6.1.7**	0	0	1	3	1	7	0	2	3	3	2	3	2	0	2	4
**3.7.1.-**	0	0	0	1	1	0	0	2	0	0	0	0	2	1	0	0
**3.7.1.2**	5	2	2	2	2	0	2	11	2	1	1	6	3	0	4	4
**3.8.1.2**	6	6	2	3	3	5	2	9	8	2	2	6	3	1	9	4
**3.8.1.3**	0	0	0	0	0	0	0	0	0	0	0	0	0	0	0	0
**3.8.1.5**	0	0	0	0	0	0	0	2	1	0	0	0	0	0	1	0
**4.1.1.-**	7	13	4	12	4	5	5	20	5	5	4	10	6	5	9	10
**4.1.1.44**	10	12	6	8	6	16	11	28	12	12	10	19	10	3	20	20
**4.1.1.55**	0	0	0	0	0	0	0	0	0	0	0	0	0	0	0	0
**4.1.1.7**	0	0	0	0	0	0	0	1	0	0	0	0	0	0	0	0
**4.1.1.70**	0	0	0	0	0	0	0	0	0	0	0	0	0	0	0	1
**4.1.1.77**	0	0	0	0	0	0	0	0	0	0	0	0	0	0	0	0
**4.1.2.-**	1	6	3	7	3	1	1	7	7	1	1	5	5	1	3	6
**4.1.3.-**	6	11	10	7	2	12	7	21	11	7	3	18	8	3	9	19

EC/KO	CH-NLF014	CH-NLF015	CH-NLM001	CH-NLM002	CH-NLM003	CH-NLM004	CH-NLM005	CH-NLM006	CH-NLM007	CH-NLM008	CH-NLM009	CH-NLM010	CH-NLM015	CH-NLM016	CH-NLM017	CH-NLM021
**4.1.3.39**	1	0	0	1	0	1	1	1	0	0	0	1	0	0	0	0
**4.1.99.-**	0	1	0	0	0	2	0	2	0	0	1	1	1	0	0	2
**4.2.1.-**	16	38	19	15	16	24	17	50	35	19	18	57	21	7	50	56
**4.2.1.17**	3	5	1	5	2	3	3	9	3	6	5	3	3	1	4	7
**4.2.1.80**	0	0	0	0	0	0	0	0	0	0	0	0	0	0	0	0
**4.2.1.83**	0	0	1	0	0	1	1	0	0	0	0	1	0	0	0	1
**4.2.1.84**	0	0	0	0	0	1	0	0	0	0	1	0	0	0	0	1
**5.1.2.2**	0	0	0	0	0	0	0	0	0	0	0	0	0	0	0	0
**5.2.1.2**	0	0	0	0	0	0	0	0	0	0	0	0	0	0	0	0
**5.3.3.4**	0	0	1	0	0	0	0	0	0	0	0	1	0	0	1	0
**5.3.99.-**	0	3	1	0	0	3	1	0	3	0	0	0	2	0	3	2
**5.4.99.-**	0	0	0	1	0	1	0	1	0	0	0	0	1	0	0	0
**5.5.1.1**	5	5	1	0	1	0	2	4	3	2	1	6	0	0	7	3
**5.5.1.2**	0	0	0	0	0	0	0	0	0	0	0	0	0	0	0	0
**6.2.1.-**	0	0	0	0	0	0	0	0	0	0	0	0	0	0	0	0
**6.3.5.2**	7	18	11	14	4	15	8	19	14	8	9	18	7	3	14	25
**K00002**	0	0	0	0	0	0	0	0	3	0	0	0	0	0	1	1
**K00055**	0	0	0	0	0	0	0	0	0	0	0	0	0	0	0	0
**K00074**	0	1	3	0	0	6	2	0	2	0	3	6	2	0	1	5
**K00088**	7	13	8	11	3	13	5	14	13	9	6	22	9	3	13	21
**K00100**	21	38	19	24	12	32	18	68	35	17	10	51	23	10	40	49
**K00128**	0	0	0	1	1	4	0	4	1	0	0	2	1	0	1	2
**K00129**	0	0	0	0	0	0	0	0	0	0	0	0	0	0	0	0
**K00132**	0	0	0	0	0	0	0	0	0	0	0	0	0	0	0	0
**K00141**	0	0	0	0	0	0	0	0	0	0	0	0	0	0	0	0
**K00146**	0	0	0	1	0	0	0	0	0	0	0	0	1	0	0	0
**K00148**	0	0	0	0	0	0	0	0	0	0	0	0	0	0	0	0
**K00155**	0	0	0	2	0	0	0	1	0	0	0	1	0	0	0	1
**K00169**	0	2	0	1	0	2	0	1	1	0	0	3	0	0	0	6
**K00224**	0	0	0	1	0	0	0	0	1	0	0	1	0	0	0	1
**K00274**	0	0	0	0	0	0	0	0	0	0	0	0	0	0	0	0
**K00446**	0	0	0	0	0	0	0	1	0	0	0	0	0	0	0	0
**K00448**	0	0	0	0	0	0	0	0	0	0	0	0	0	0	0	0
**K00462**	1	1	0	1	0	0	2	2	2	0	0	1	1	0	0	3
**K00480**	0	0	0	0	0	0	0	0	0	0	0	0	0	0	0	0
**K00481**	0	0	0	0	0	0	0	0	0	0	0	0	0	0	0	0
**K00539**	0	0	0	1	0	0	0	0	1	0	0	1	0	0	0	0
**K00599**	16	19	17	18	5	30	10	35	27	21	16	42	14	9	22	53
**K00626**	0	6	3	4	1	5	0	2	2	2	1	6	4	1	1	12
**K00632**	0	0	0	0	0	0	0	0	0	0	0	0	0	0	0	0
**K00680**	15	29	17	18	8	26	12	49	21	20	7	34	24	5	32	30
**K00757**	7	6	6	8	1	10	5	16	7	6	5	12	6	3	6	13
**K00758**	0	0	0	0	2	1	0	3	0	1	0	1	3	0	0	3
**K00760**	5	7	6	5	2	15	8	19	11	6	7	16	9	2	14	20
**K00799**	0	0	2	2	2	0	0	6	0	0	0	0	1	0	1	0
**K00857**	5	6	6	6	2	5	1	16	7	3	1	11	3	0	5	10
**K00876**	11	17	12	14	7	16	12	30	17	16	11	30	10	3	15	32
**K01026**	0	1	0	1	1	2	0	0	0	0	0	0	0	0	0	0
**K01031**	0	0	0	0	0	0	0	0	0	0	0	0	0	0	0	0

EC/KO	CH-NLF014	CH-NLF015	CH-NLM001	CH-NLM002	CH-NLM003	CH-NLM004	CH-NLM005	CH-NLM006	CH-NLM007	CH-NLM008	CH-NLM009	CH-NLM010	CH-NLM015	CH-NLM016	CH-NLM017	CH-NLM021
K01034	0	2	1	0	1	0	0	2	0	0	0	0	1	0	2	0
K01039	0	0	0	1	0	0	0	0	1	0	0	0	0	0	0	1
K01041	5	5	5	7	1	1	2	14	3	4	2	9	5	2	9	7
K01053	0	0	0	0	0	0	0	0	0	0	0	0	0	0	0	0
K01055	0	0	0	0	0	0	0	0	0	0	0	0	0	0	0	0
K01061	0	1	1	0	1	0	0	3	0	0	0	0	1	0	1	0
K01066	4	3	2	3	1	1	1	8	2	3	0	7	2	0	6	4
K01075	0	0	1	2	1	0	1	3	2	0	0	0	1	0	1	0
K01077	6	9	4	5	3	0	3	12	11	4	2	12	5	0	9	11
K01101	1	3	1	2	1	2	0	2	2	0	0	2	1	1	2	4
K01195	3	3	3	0	2	3	2	8	7	2	1	13	2	0	6	10
K01426	0	2	0	1	0	0	0	1	1	0	0	1	0	0	1	1
K01428	0	0	0	0	0	1	0	0	1	0	0	2	0	0	0	1
K01457	0	1	0	0	0	1	0	0	0	0	0	0	0	0	0	0
K01464	0	1	0	1	1	2	0	3	4	0	1	1	0	0	1	1
K01489	8	8	7	5	3	7	4	15	5	5	6	14	8	2	8	17
K01500	1	0	0	0	0	0	0	0	0	0	0	0	0	0	0	0
K01501	0	1	0	1	0	1	0	0	4	0	0	0	0	0	0	3
K01502	0	0	0	0	0	0	0	0	0	0	0	0	0	0	0	0
K01512	0	0	1	5	1	10	2	4	4	4	2	4	2	0	2	6
K01560	1	2	1	1	0	4	0	1	1	1	3	2	1	1	2	2
K01561	0	1	0	2	0	0	0	0	1	0	0	1	0	0	0	1
K01563	0	0	0	0	0	0	0	0	0	0	0	0	0	0	0	0
K01564	1	1	2	2	0	0	0	0	1	0	0	3	2	0	1	2
K01607	8	9	2	3	4	5	5	15	7	4	4	14	6	2	10	9
K01612	0	0	0	0	0	0	0	0	0	0	0	0	0	0	0	0
K01615	8	12	8	7	1	4	7	19	11	5	2	16	8	0	14	23
K01617	0	0	0	0	0	0	0	0	0	0	0	0	0	0	0	0
K01666	1	0	3	1	0	7	2	4	1	1	0	6	1	0	0	7
K01692	0	0	0	0	0	0	0	0	0	0	0	0	1	0	0	0
K01721	0	0	0	0	0	0	0	0	0	0	0	0	0	0	0	0
K01726	6	9	3	3	3	2	5	17	6	3	1	3	4	3	11	4
K01781	0	0	0	1	0	0	0	0	3	0	0	0	0	0	0	0
K01821	0	1	0	2	2	2	2	5	1	0	0	1	1	0	1	0
K01856	0	0	0	0	0	0	0	0	0	0	0	0	0	0	0	0
K01857	0	0	0	1	0	0	0	0	1	0	0	0	0	0	0	2
K01913	0	0	0	0	0	0	0	1	0	0	0	0	0	0	0	0
K01951	5	16	10	13	4	14	7	15	11	7	7	18	7	3	13	27
K02554	0	0	0	0	0	0	0	0	0	0	0	0	0	0	0	0
K03381	0	0	0	0	0	0	0	0	0	0	0	0	0	0	0	0
K03382	0	0	0	0	0	0	0	0	0	0	0	0	0	0	0	1
K03464	0	0	0	0	0	0	0	0	0	0	0	0	0	0	0	0
K03518	0	4	2	6	0	11	0	5	10	5	2	4	0	0	4	8
K03862	0	0	0	0	0	0	0	0	0	0	0	0	0	0	0	0
K04099	0	0	0	0	0	0	0	1	0	0	0	0	0	0	0	0
K04100	0	0	0	0	0	0	0	0	0	0	0	0	0	0	0	0
K04102	0	0	0	0	0	0	0	0	0	0	0	0	0	0	0	0
K04116	0	0	0	0	0	0	0	1	0	0	0	0	0	0	0	0
K05394	0	0	0	1	0	0	0	0	1	0	0	1	0	0	0	0

EC/KO	CH-NLF014	CH-NLF015	CH-NLM001	CH-NLM002	CH-NLM003	CH-NLM004	CH-NLM005	CH-NLM006	CH-NLM007	CH-NLM008	CH-NLM009	CH-NLM010	CH-NLM015	CH-NLM016	CH-NLM017	CH-NLM021
**K05549**	0	0	0	0	0	0	0	0	0	0	0	0	0	0	0	0
**K05783**	0	0	0	0	0	0	0	0	0	0	0	0	0	0	0	0
**K05797**	0	0	0	0	0	0	0	0	0	0	0	0	0	0	0	0
**K06281**	0	1	0	1	0	0	0	4	2	0	0	2	0	0	1	1
**K06446**	0	4	1	7	0	2	1	7	6	2	2	1	4	0	0	4
**K06912**	0	0	0	0	0	0	0	0	0	0	0	0	0	0	0	0
**K07535**	0	0	0	0	0	0	0	0	0	0	0	0	0	0	0	0
**K07536**	3	0	1	3	1	1	0	4	1	2	2	0	0	1	0	3
**K08689**	0	0	0	0	0	0	0	0	0	0	0	0	0	0	0	0
**K08710**	0	0	0	0	0	0	0	0	0	0	0	0	0	0	0	0
**K09461**	0	0	0	0	0	0	0	0	0	0	0	0	0	0	0	0
**K10217**	0	0	0	0	0	0	0	0	0	0	0	0	0	0	0	0
**K10218**	0	1	0	1	0	0	0	0	1	0	0	1	0	0	0	0
**K10220**	0	0	0	0	0	0	0	0	0	0	0	0	0	0	0	0
**K11180**	0	0	0	0	0	0	0	0	0	0	0	0	0	0	0	0
**K13953**	0	0	1	0	0	1	0	0	0	0	0	0	0	0	0	0
**K14333**	0	0	0	0	0	0	0	1	0	0	0	0	0	0	0	0
**K14519**	0	0	0	0	0	0	0	0	0	0	0	0	0	0	0	0
**K15054**	0	1	0	0	1	0	0	1	1	0	0	0	0	0	0	0
**K16173**	0	1	0	0	0	0	0	0	0	0	0	0	0	0	0	0
**K16514**	0	0	1	1	1	0	0	1	1	0	0	0	1	0	0	0
**K16874**	0	0	0	0	0	0	0	0	0	0	0	0	0	0	0	0

EC/KO	CH-NLM022	CH-NLM023	CH-NLM024	CH-NLM025	CH-NLM026	CH-NLM027	CH-NLM028	CH-NLM029	CH-NLM031	CH-NLM032	CH-NOF001	CH-NOF002	CH-NOF004	CH-NOF005	CH-NOF006	CH-NOF007
**1.1.1.-**	38	64	32	10	44	73	41	39	38	53	18	27	30	55	38	31
**1.1.1.1**	40	54	21	20	54	64	47	39	42	52	12	27	26	52	27	21
**1.1.1.157**	0	1	0	0	1	0	0	0	1	0	1	1	0	1	0	1
**1.1.1.205**	26	22	17	14	33	23	20	23	21	20	6	18	17	27	11	13
**1.1.1.35**	0	2	0	0	0	5	2	1	2	2	1	3	1	3	1	0
**1.12.99.6**	2	4	5	0	0	3	0	0	0	3	1	1	0	0	3	1
**1.13.11.-**	0	0	0	0	0	0	0	0	0	0	0	0	0	0	0	0
**1.13.11.1**	0	0	0	0	0	0	0	0	0	0	0	0	0	0	0	0
**1.13.11.2**	0	0	0	0	0	0	0	0	0	0	0	0	0	0	0	0
**1.13.11.3**	0	0	0	0	0	0	0	0	0	0	0	0	0	0	0	0
**1.13.11.39**	0	0	0	0	0	4	0	0	0	0	0	0	0	0	0	0
**1.13.11.5**	0	1	0	0	1	0	0	0	0	0	0	0	0	0	0	0
**1.13.11.8**	0	0	0	0	0	0	0	0	0	0	0	0	0	0	0	0
**1.14.12.10**	1	1	1	0	0	0	0	0	0	0	0	1	1	0	1	0
**1.14.12.13**	0	0	0	0	0	0	0	0	0	0	0	0	0	0	0	0
**1.14.12.18**	0	0	0	0	0	1	0	0	0	0	0	0	0	0	0	0
**1.14.13.-**	1	1	3	0	0	5	0	0	0	0	0	2	0	0	3	0
**1.14.13.1**	0	0	0	0	0	0	0	0	0	0	0	0	0	0	0	0
**1.14.13.2**	0	0	0	0	0	0	0	0	0	0	0	0	0	0	0	0
**1.14.13.50**	0	0	0	0	0	0	0	0	0	0	0	0	0	0	0	0
**1.14.13.7**	0	0	0	0	0	0	0	0	0	0	0	0	0	0	0	0
**1.14.13.8**	0	0	0	0	0	0	0	0	0	0	0	0	0	0	0	0
**1.14.13.82**	0	0	0	0	0	1	0	0	0	0	0	0	0	0	0	0
**1.14.99.-**	0	0	0	0	0	0	0	0	0	0	0	0	0	0	0	0
**1.17.99.1**	0	0	0	0	0	0	0	0	0	0	0	0	0	0	0	0
**1.18.6.1**	0	0	0	0	0	0	0	0	1	0	0	0	0	0	0	0
**1.2.1.-**	0	0	2	0	0	2	0	0	0	0	0	1	0	0	0	0
**1.2.1.10**	9	6	1	4	6	5	4	9	4	5	3	11	4	7	2	0
**1.2.1.3**	4	11	6	0	5	17	6	4	5	11	2	6	4	7	5	2
**1.2.1.39**	0	0	1	0	0	0	0	0	0	0	0	0	0	0	0	0
**1.2.7.1**	6	11	7	7	20	0	4	8	11	12	5	4	10	11	5	9
**1.2.99.2**	3	6	2	2	6	8	5	3	3	3	2	1	0	5	3	0
**1.3.1.-**	1	1	0	0	1	0	0	0	0	1	0	1	0	1	1	1
**1.3.1.2**	1	1	1	0	0	0	1	0	0	0	0	0	1	0	0	0
**1.3.1.25**	0	0	0	0	0	0	0	0	0	0	0	0	0	0	0	0
**1.3.99.-**	2	0	1	0	0	0	0	0	0	0	0	0	0	0	1	0
**1.6.5.-**	38	47	26	22	50	21	26	47	39	40	28	16	29	42	30	22
**1.7.1.-**	9	12	6	8	8	10	6	6	7	8	8	5	8	10	4	6
**1.8.99.3**	0	0	0	1	0	0	0	0	0	0	0	1	0	0	1	0
**2.1.1.-**	203	222	119	91	245	268	182	236	199	297	96	126	157	252	120	146
**2.3.1.-**	122	162	76	41	143	133	90	125	111	149	76	69	103	141	106	94
**2.3.1.16**	3	3	2	1	5	1	0	5	1	2	1	0	3	5	0	0
**2.3.1.5**	1	0	1	0	0	1	0	0	0	0	0	0	0	0	0	0
**2.3.1.9**	2	3	4	0	0	2	1	0	0	1	0	3	1	1	1	2
**2.4.2.10**	13	16	9	9	11	15	14	14	14	17	4	9	6	20	4	10
**2.4.2.3**	3	4	2	3	8	26	8	8	5	8	4	4	6	6	3	5
**2.4.2.4**	1	1	1	1	8	7	3	4	3	7	0	1	1	2	0	0
**2.4.2.8**	9	14	5	7	10	17	7	10	10	15	8	7	7	14	7	10
**2.5.1.-**	16	24	18	13	19	16	14	18	20	16	16	9	14	17	13	17

EC/KO	CH-NLM022	CH-NLM023	CH-NLM024	CH-NLM025	CH-NLM026	CH-NLM027	CH-NLM028	CH-NLM029	CH-NLM031	CH-NLM032	CH-NOF001	CH-NOF002	CH-NOF004	CH-NOF005	CH-NOF006	CH-NOF007
**2.5.1.18**	6	5	6	1	0	8	1	1	1	3	0	6	0	0	5	0
**2.6.1.-**	24	44	16	13	33	23	26	32	25	31	19	16	20	41	20	20
**2.7.1.21**	5	10	3	3	7	8	8	6	5	6	3	3	3	8	7	4
**2.7.1.48**	21	27	13	10	23	27	20	23	20	24	12	13	16	28	15	13
**2.7.4.-**	9	20	9	7	21	16	11	11	16	17	9	9	5	15	9	12
**2.8.3.-**	0	3	4	1	0	0	0	2	2	0	2	1	0	3	1	0
**2.8.3.1**	1	2	0	0	0	0	0	0	0	1	0	2	0	0	0	0
**2.8.3.12**	0	1	0	0	1	0	0	0	0	0	0	1	0	0	0	0
**2.8.3.6**	0	0	0	0	0	0	1	1	0	0	1	1	0	0	0	1
**2.8.3.8**	2	1	2	0	2	4	1	0	0	2	0	1	0	0	1	1
**3.1.1.-**	7	10	6	1	1	7	4	3	3	9	4	3	4	6	7	1
**3.1.1.1**	5	9	7	1	7	4	1	3	9	3	1	4	3	6	7	2
**3.1.1.17**	0	5	1	0	4	1	0	1	1	2	0	1	3	2	2	4
**3.1.1.2**	1	1	1	0	1	3	1	1	0	1	0	1	0	0	1	1
**3.1.1.24**	2	1	1	0	4	2	0	2	3	2	0	2	0	0	2	3
**3.1.1.45**	1	1	1	0	0	0	0	0	0	0	0	1	0	0	1	0
**3.1.2.-**	2	2	2	0	0	4	0	0	0	1	0	1	0	1	1	0
**3.1.2.23**	3	6	3	1	5	0	2	3	4	2	6	0	6	4	5	2
**3.1.3.1**	11	22	10	8	15	3	10	9	21	12	12	12	10	20	19	15
**3.1.3.2**	8	8	5	3	9	5	3	3	7	5	3	3	7	5	6	5
**3.1.3.41**	0	0	0	0	0	0	0	0	0	0	0	0	0	0	0	0
**3.2.1.31**	6	5	1	2	1	2	3	6	2	4	1	3	1	3	1	6
**3.3.2.9**	0	0	0	0	0	1	0	0	0	0	0	0	0	0	0	0
**3.5.1.-**	17	17	7	9	21	31	14	28	9	24	10	9	13	20	9	6
**3.5.1.4**	0	3	1	2	0	2	1	1	2	3	3	0	0	1	1	0
**3.5.1.5**	2	2	0	1	2	2	0	0	2	2	2	2	2	0	0	1
**3.5.1.54**	2	0	1	1	0	5	3	1	1	4	0	2	0	6	3	1
**3.5.1.6**	2	2	1	0	1	2	1	2	1	1	1	1	0	1	0	0
**3.5.2.-**	0	0	0	0	0	0	0	0	0	0	0	0	0	1	0	0
**3.5.2.2**	1	0	0	0	1	2	1	1	1	3	0	0	0	0	0	0
**3.5.4.-**	12	19	10	11	19	21	11	17	16	21	7	7	12	21	8	12
**3.5.4.5**	8	9	5	4	15	5	4	9	6	10	8	6	7	12	7	7
**3.5.5.1**	0	1	1	1	0	0	0	0	2	0	0	0	0	1	1	1
**3.5.5.7**	0	0	0	0	0	0	0	0	0	0	0	0	0	0	0	0
**3.5.99.3**	0	0	0	0	0	0	0	0	0	0	0	0	0	0	0	0
**3.6.1.7**	4	4	1	1	6	9	7	2	3	10	0	1	2	7	2	7
**3.7.1.-**	3	1	2	1	1	4	0	2	1	1	1	1	0	0	2	0
**3.7.1.2**	5	7	2	0	3	0	3	3	2	2	2	2	2	7	7	3
**3.8.1.2**	4	8	3	1	8	9	4	4	6	8	2	3	8	9	8	6
**3.8.1.3**	0	0	0	0	0	0	0	0	0	0	0	0	0	0	0	0
**3.8.1.5**	0	1	0	0	0	2	0	1	0	0	0	0	0	1	0	0
**4.1.1.-**	11	19	11	2	6	11	7	13	7	11	10	5	9	8	10	5
**4.1.1.44**	13	25	13	6	10	15	10	15	17	15	3	6	12	15	14	15
**4.1.1.55**	0	0	0	0	0	0	0	0	0	0	0	0	0	0	0	0
**4.1.1.7**	0	0	0	0	0	0	0	0	0	0	0	0	0	0	0	0
**4.1.1.70**	0	0	0	0	0	0	0	0	0	0	0	1	0	0	0	0
**4.1.1.77**	0	0	0	0	0	0	0	0	0	0	0	0	0	1	0	0
**4.1.2.-**	7	5	6	2	6	7	8	6	3	9	2	3	0	10	2	2
**4.1.3.-**	21	22	7	8	19	14	16	19	20	13	8	9	13	12	8	12

EC/KO	CH-NLM022	CH-NLM023	CH-NLM024	CH-NLM025	CH-NLM026	CH-NLM027	CH-NLM028	CH-NLM029	CH-NLM031	CH-NLM032	CH-NOF001	CH-NOF002	CH-NOF004	CH-NOF005	CH-NOF006	CH-NOF007
4.1.3.39	2	1	2	0	0	0	1	0	0	0	0	0	0	0	1	0
4.1.99.-	1	2	0	0	4	0	0	5	1	3	0	0	0	0	0	0
4.2.1.-	42	71	20	16	35	31	32	48	37	47	18	28	27	59	41	37
4.2.1.17	5	5	3	2	5	5	3	6	3	5	1	5	1	9	2	3
4.2.1.80	0	1	0	0	0	0	0	0	0	0	0	0	1	0	0	0
4.2.1.83	1	0	0	1	0	0	1	1	0	0	0	0	0	0	0	0
4.2.1.84	0	0	0	0	0	0	1	0	0	0	0	0	0	0	0	0
5.1.2.2	0	0	0	0	0	0	0	0	0	0	0	0	0	0	0	0
5.2.1.2	0	0	0	0	0	0	0	0	0	0	0	0	0	0	0	0
5.3.3.4	0	0	0	0	0	0	0	0	0	0	0	0	0	0	0	0
5.3.99.-	1	2	2	1	3	7	2	4	4	5	4	1	0	4	0	1
5.4.99.-	1	0	0	0	0	0	0	1	0	0	1	0	0	0	0	0
5.5.1.1	2	6	5	0	4	0	5	3	3	5	2	2	3	6	5	2
5.5.1.2	0	0	0	0	0	0	0	0	0	0	0	0	0	0	0	0
6.2.1.-	0	0	0	0	0	1	0	0	0	0	0	0	0	0	0	0
6.3.5.2	17	15	12	7	21	14	11	28	17	18	9	9	10	17	12	11
K00002	0	0	0	0	0	2	1	1	0	1	0	1	0	2	1	1
K00055	0	0	0	0	0	1	0	0	0	0	0	0	0	0	0	0
K00074	3	5	1	0	6	5	3	2	5	6	3	5	2	7	1	1
K00088	19	18	13	8	23	21	16	21	17	17	10	12	12	26	13	14
K00100	36	70	34	19	39	54	37	42	30	66	18	21	24	49	33	28
K00128	4	3	0	0	0	4	1	2	1	2	1	3	2	2	1	0
K00129	0	0	0	0	0	0	0	0	0	0	0	0	0	0	0	0
K00132	0	0	0	0	0	0	1	0	0	0	0	0	0	0	0	0
K00141	0	0	0	0	0	0	0	0	0	0	0	0	0	0	0	0
K00146	0	0	1	0	0	2	0	0	0	0	0	0	0	0	0	0
K00148	0	0	0	0	0	0	0	0	0	0	0	0	0	0	0	0
K00155	0	1	1	0	0	0	0	0	0	0	0	0	0	0	1	0
K00169	1	3	2	1	3	0	1	2	1	2	1	0	1	1	0	2
K00224	0	1	1	0	0	0	0	0	0	0	0	0	0	0	0	0
K00274	0	0	0	0	0	0	0	0	0	0	0	0	0	0	0	0
K00446	0	0	0	0	0	0	0	0	0	0	0	0	0	0	0	0
K00448	0	0	0	0	0	0	0	0	0	0	0	0	0	0	0	0
K00462	0	2	0	1	0	0	0	0	0	0	1	0	1	1	1	2
K00480	0	0	0	0	0	1	0	0	0	0	0	0	0	0	0	0
K00481	0	0	0	0	0	1	0	0	0	0	0	0	0	0	0	0
K00539	0	1	0	0	0	0	0	0	0	0	0	0	0	0	0	1
K00599	42	43	17	18	37	47	40	59	31	61	12	19	32	47	18	26
K00626	5	4	4	1	8	6	4	9	3	5	1	5	4	9	1	4
K00632	1	2	1	0	0	0	0	0	0	0	0	0	0	0	0	0
K00680	30	40	32	13	29	33	20	25	27	38	17	14	21	43	29	27
K00757	4	9	7	3	11	10	11	5	12	9	6	5	9	11	8	8
K00758	2	2	1	1	2	1	1	5	1	2	1	1	0	2	2	2
K00760	16	17	4	6	16	21	10	20	16	19	10	9	10	19	8	12
K00799	2	2	1	0	0	3	0	0	0	1	0	1	0	0	1	0
K00857	8	11	4	5	12	11	10	13	10	11	5	4	5	12	7	6
K00876	25	24	13	10	27	23	20	28	24	27	14	12	16	30	14	12
K01026	0	0	1	0	1	2	1	0	0	0	0	2	0	0	0	0
K01031	0	0	0	0	0	1	0	0	0	0	0	0	0	0	0	0

EC/KO	CH-NLM022	CH-NLM023	CH-NLM024	CH-NLM025	CH-NLM026	CH-NLM027	CH-NLM028	CH-NLM029	CH-NLM031	CH-NLM032	CH-NOF001	CH-NOF002	CH-NOF004	CH-NOF005	CH-NOF006	CH-NOF007
K01034	2	1	0	0	1	3	0	0	1	1	1	1	1	1	1	0
K01039	0	1	0	0	1	0	0	1	0	0	0	1	0	1	0	0
K01041	7	7	7	1	6	0	5	6	7	6	4	3	5	4	8	6
K01053	0	0	0	0	0	0	0	0	0	0	0	0	0	0	0	0
K01055	0	0	0	0	0	1	0	0	0	0	0	0	0	0	0	0
K01061	1	1	2	0	0	0	0	0	0	0	0	2	0	0	1	0
K01066	5	8	5	0	9	9	3	3	8	2	0	5	3	7	4	2
K01075	1	1	2	1	0	1	0	0	0	1	0	1	0	0	1	1
K01077	6	16	6	3	8	3	7	6	9	8	3	5	6	11	11	7
K01101	0	4	1	0	2	4	2	2	2	3	3	0	1	3	2	1
K01195	9	11	4	1	6	4	5	13	7	10	2	6	3	7	6	5
K01426	0	0	2	0	0	4	0	1	0	0	1	0	0	1	0	0
K01428	1	0	0	1	1	3	1	1	1	1	0	1	1	0	0	0
K01457	0	0	0	0	0	0	0	0	0	0	0	0	0	0	0	0
K01464	1	2	2	0	0	5	1	1	2	3	0	1	0	1	0	0
K01489	11	9	5	7	14	10	6	11	11	17	10	7	9	17	8	11
K01500	0	0	0	1	0	0	0	0	0	0	0	0	0	0	0	0
K01501	0	1	2	1	0	3	0	0	4	1	0	0	0	1	0	0
K01502	0	0	0	0	0	0	0	0	0	0	0	0	0	0	0	0
K01512	9	4	1	1	7	13	8	4	3	15	1	2	2	8	2	7
K01560	6	1	1	3	3	2	5	3	4	7	0	1	1	3	1	4
K01561	0	2	1	0	0	0	0	1	0	0	0	0	0	1	0	1
K01563	0	0	0	0	0	0	0	0	0	0	0	0	0	0	0	0
K01564	1	4	3	0	1	1	0	1	1	0	2	0	0	0	2	2
K01607	6	12	9	3	4	10	5	11	12	8	2	5	6	11	10	6
K01612	0	0	0	0	0	0	0	0	0	0	0	0	0	0	0	0
K01615	14	13	13	7	19	7	12	22	14	18	6	11	12	21	11	12
K01617	0	0	0	0	0	0	0	0	0	0	0	0	0	0	0	0
K01666	10	3	2	3	5	0	4	12	2	2	1	3	5	4	1	2
K01692	0	0	1	0	0	0	0	0	0	0	0	0	0	0	0	0
K01721	0	0	0	0	0	0	0	0	0	0	0	0	0	0	0	0
K01726	5	11	8	5	9	8	3	4	9	3	4	3	2	11	12	7
K01781	0	1	0	0	0	0	0	0	0	0	0	1	0	0	0	0
K01821	2	2	1	0	2	5	0	0	2	4	0	0	0	1	1	1
K01856	0	0	0	0	0	0	0	0	0	0	0	0	0	0	0	0
K01857	0	1	0	0	0	0	0	0	0	0	0	0	0	0	0	1
K01913	1	1	0	0	0	0	0	0	0	0	0	0	0	1	0	0
K01951	17	13	11	6	20	24	11	24	18	21	9	10	10	18	9	10
K02554	0	1	0	0	0	0	0	0	0	0	0	1	0	0	0	0
K03381	0	0	0	0	0	0	0	0	0	0	0	0	0	0	0	0
K03382	0	1	0	0	0	0	0	1	1	0	0	0	0	0	0	0
K03464	0	0	0	0	0	0	0	0	0	0	0	0	0	0	0	0
K03518	4	8	2	2	4	20	13	8	7	17	3	6	1	13	0	3
K03862	0	0	0	0	0	1	0	0	0	0	0	0	0	0	0	0
K04099	0	0	0	0	0	0	0	0	0	0	0	0	0	0	0	0
K04100	0	0	0	0	0	0	0	0	0	0	0	0	0	0	0	0
K04102	0	0	0	0	0	0	0	0	0	0	0	0	0	0	0	0
K04116	0	0	0	0	0	0	0	0	0	0	0	0	0	0	0	0
K05394	0	0	0	1	0	0	0	0	0	0	0	0	0	0	0	0

EC/KO	CH-NLM022	CH-NLM023	CH-NLM024	CH-NLM025	CH-NLM026	CH-NLM027	CH-NLM028	CH-NLM029	CH-NLM031	CH-NLM032	CH-NOF001	CH-NOF002	CH-NOF004	CH-NOF005	CH-NOF006	CH-NOF007
K05549	0	0	0	0	0	1	0	0	0	0	0	0	0	0	0	0
K05783	0	0	0	0	0	1	0	0	0	0	0	0	0	0	0	0
K05797	0	0	0	0	0	0	0	0	0	0	0	0	0	0	0	0
K06281	1	3	2	0	0	0	0	0	0	2	0	1	0	0	1	1
K06446	2	5	3	0	3	3	3	5	3	8	1	3	2	7	1	2
K06912	0	0	0	0	0	0	0	0	0	0	0	0	0	0	0	0
K07535	0	0	0	0	0	0	0	0	0	0	0	0	0	0	0	0
K07536	2	4	2	1	1	2	1	0	3	4	1	1	2	1	2	1
K08689	0	0	0	0	0	0	0	0	0	0	0	0	0	0	0	0
K08710	0	0	1	0	0	1	0	0	0	0	0	0	0	0	0	0
K09461	0	0	0	0	0	1	0	0	0	0	0	0	0	0	0	0
K10217	0	0	0	0	0	0	0	0	0	0	0	0	0	0	0	0
K10218	0	0	1	0	1	1	0	0	0	0	0	0	0	1	0	1
K10220	0	0	0	0	0	0	0	0	0	0	0	0	0	0	0	0
K11180	0	0	0	0	1	0	0	0	0	0	0	0	0	0	0	0
K13953	1	1	1	0	0	5	0	1	0	1	0	0	0	1	0	0
K14333	0	0	0	0	0	1	0	0	0	0	0	0	0	0	0	0
K14519	0	0	0	0	0	0	0	0	0	0	0	0	0	0	0	0
K15054	1	1	1	0	0	0	0	0	0	0	0	1	0	0	0	0
K16173	0	1	1	1	0	0	1	0	0	1	0	0	0	1	0	0
K16514	0	1	1	0	0	1	0	1	0	0	0	1	0	0	1	0
K16874	0	0	0	0	0	0	0	0	0	0	0	0	0	0	0	0

EC/KO	CH-NOF008	CH-NOF009	CH-NOF010	CH-NOF011	CH-NOF012	CH-NOF013	CH-NOF014	CH-NOM001	CH-NOM002	CH-NOM004	CH-NOM005	CH-NOM007	CH-NOM008	CH-NOM009	CH-NOM010	CH-NOM012
**1.1.1.-**	47	36	52	30	32	48	27	18	44	40	48	15	25	67	36	25
**1.1.1.1**	33	17	30	15	24	39	31	25	50	20	39	20	15	72	30	24
**1.1.1.157**	1	1	0	0	0	0	0	1	1	0	0	2	0	0	0	0
**1.1.1.205**	25	15	22	10	12	23	17	13	26	14	15	16	16	32	15	16
**1.1.1.35**	6	1	0	0	1	0	2	0	3	0	1	2	0	5	1	1
**1.12.99.6**	1	2	1	0	0	0	0	1	1	2	2	0	2	0	0	0
**1.13.11.-**	0	0	0	0	0	0	0	0	0	0	0	0	0	0	0	0
**1.13.11.1**	0	0	0	0	0	0	0	0	0	0	0	0	0	0	0	0
**1.13.11.2**	0	0	0	0	0	0	0	0	0	0	0	0	0	0	0	0
**1.13.11.3**	0	0	0	0	0	0	0	0	0	0	0	0	0	0	0	0
**1.13.11.39**	0	0	0	0	0	0	0	0	0	0	0	0	0	0	0	0
**1.13.11.5**	0	0	0	0	0	0	0	0	0	0	0	0	0	0	0	0
**1.13.11.8**	0	0	0	0	0	0	0	0	0	0	0	0	0	0	0	0
**1.14.12.10**	0	0	0	0	1	0	0	0	0	0	1	0	0	0	1	0
**1.14.12.13**	0	0	0	0	0	0	0	0	0	0	0	0	0	0	0	0
**1.14.12.18**	0	0	0	0	0	0	0	0	0	0	0	0	0	0	0	0
**1.14.13.-**	0	0	0	0	0	0	0	0	1	1	2	0	0	0	0	0
**1.14.13.1**	0	0	0	0	0	0	0	0	0	0	0	0	0	0	0	0
**1.14.13.2**	0	0	0	0	0	0	0	0	0	0	0	0	0	0	0	0
**1.14.13.50**	0	0	0	0	0	0	0	0	0	0	0	0	0	0	0	0
**1.14.13.7**	0	0	0	0	0	0	0	0	1	0	0	0	0	0	0	0
**1.14.13.8**	0	0	0	0	0	0	0	0	0	0	0	0	0	0	0	0
**1.14.13.82**	0	0	0	0	0	0	0	0	0	0	0	0	0	0	0	0
**1.14.99.-**	0	0	0	0	0	0	0	0	0	0	0	0	0	0	0	0
**1.17.99.1**	0	0	0	0	0	0	0	0	0	0	0	0	0	0	0	0
**1.18.6.1**	0	0	0	0	1	0	0	0	0	0	0	0	0	0	0	0
**1.2.1.-**	0	0	0	0	0	0	0	0	1	0	1	0	0	0	0	0
**1.2.1.10**	7	1	1	0	3	2	3	2	11	5	9	4	1	12	4	3
**1.2.1.3**	4	3	3	3	6	2	5	4	12	5	7	1	2	9	5	5
**1.2.1.39**	0	0	0	0	0	0	0	0	0	0	1	0	0	0	0	0
**1.2.7.1**	17	9	12	7	11	7	7	2	7	3	3	9	2	6	10	5
**1.2.99.2**	3	0	2	0	0	1	4	1	5	2	6	1	0	7	1	1
**1.3.1.-**	0	1	2	0	2	2	1	0	0	0	1	1	2	0	1	1
**1.3.1.2**	1	2	1	0	0	0	0	0	1	1	0	0	0	0	0	0
**1.3.1.25**	0	0	0	0	0	0	0	0	0	0	0	0	0	0	0	0
**1.3.99.-**	0	0	1	0	0	0	0	0	0	1	1	0	0	0	0	0
**1.6.5.-**	63	27	51	28	27	50	33	13	29	23	34	22	38	35	38	35
**1.7.1.-**	8	5	13	4	10	10	8	5	9	4	7	5	6	11	8	10
**1.8.99.3**	0	0	0	0	0	0	0	0	0	1	0	0	0	0	0	0
**2.1.1.-**	268	102	192	92	146	171	199	89	217	126	227	149	113	298	153	136
**2.3.1.-**	169	89	123	94	105	153	122	46	107	87	99	93	89	121	112	102
**2.3.1.16**	5	2	1	1	2	3	5	1	1	1	1	1	1	2	3	2
**2.3.1.5**	0	0	0	0	0	0	0	0	0	1	1	0	0	0	0	0
**2.3.1.9**	1	1	2	0	0	0	1	0	3	2	2	1	0	1	0	0
**2.4.2.10**	12	7	11	2	9	8	11	7	13	10	13	10	7	15	12	13
**2.4.2.3**	10	2	3	2	4	5	7	3	11	6	10	8	1	15	4	3
**2.4.2.4**	5	0	0	0	3	2	4	1	5	1	2	4	0	5	4	3
**2.4.2.8**	13	7	9	5	7	10	13	6	19	6	12	9	5	15	8	9
**2.5.1.-**	21	17	25	13	15	25	12	6	13	14	12	10	16	13	17	16

EC/KO	CH-NOF008	CH-NOF009	CH-NOF010	CH-NOF011	CH-NOF012	CH-NOF013	CH-NOF014	CH-NOM001	CH-NOM002	CH-NOM004	CH-NOM005	CH-NOM007	CH-NOM008	CH-NOM009	CH-NOM010	CH-NOM012
**2.5.1.18**	0	0	4	0	1	1	4	1	0	3	6	1	0	4	0	0
**2.6.1.-**	32	17	30	23	22	34	33	8	33	23	24	14	18	32	23	14
**2.7.1.21**	6	5	5	5	5	8	2	1	2	3	2	5	4	3	8	7
**2.7.1.48**	31	10	23	15	14	25	33	5	19	15	19	19	11	32	24	17
**2.7.4.-**	17	7	12	7	9	11	8	5	13	7	9	9	8	16	10	8
**2.8.3.-**	1	2	1	1	2	1	2	1	1	3	0	0	2	1	2	2
**2.8.3.1**	0	0	0	0	0	0	0	0	0	0	0	0	0	0	0	0
**2.8.3.12**	0	0	0	1	1	0	0	0	1	0	0	0	0	0	0	0
**2.8.3.6**	0	0	0	0	0	1	0	0	0	1	0	0	0	0	1	0
**2.8.3.8**	1	0	0	0	1	0	2	1	2	2	2	0	0	9	0	0
**3.1.1.-**	3	4	6	2	3	5	3	3	9	6	5	3	4	3	2	5
**3.1.1.1**	3	7	8	5	7	8	3	3	8	2	6	2	5	3	4	3
**3.1.1.17**	2	3	2	2	3	1	1	0	2	1	1	0	3	0	2	0
**3.1.1.2**	0	0	1	0	0	0	3	0	0	1	1	1	0	0	0	0
**3.1.1.24**	2	0	4	0	1	2	2	1	2	4	3	2	0	4	2	1
**3.1.1.45**	0	0	1	0	0	0	0	0	0	1	1	0	0	0	0	0
**3.1.2.-**	1	0	0	1	0	1	0	0	1	3	2	0	0	0	1	0
**3.1.2.23**	3	3	7	3	4	5	4	1	2	1	1	2	4	0	7	5
**3.1.3.1**	15	16	21	15	12	18	6	6	14	15	8	11	18	10	16	5
**3.1.3.2**	6	7	7	4	5	8	3	1	2	5	4	5	7	4	8	2
**3.1.3.41**	0	0	0	0	0	0	0	0	0	0	0	0	0	0	0	0
**3.2.1.31**	3	0	1	0	2	1	2	5	8	1	11	0	4	6	3	2
**3.3.2.9**	0	0	0	0	0	0	0	0	0	0	0	0	0	0	0	0
**3.5.1.-**	22	4	16	7	8	16	20	7	20	11	12	15	5	22	13	15
**3.5.1.4**	6	2	3	1	1	1	1	2	1	5	1	4	1	2	1	1
**3.5.1.5**	0	1	1	0	0	0	1	0	2	0	1	0	0	2	0	0
**3.5.1.54**	1	0	0	1	0	0	1	0	4	3	4	0	1	3	2	1
**3.5.1.6**	1	2	1	0	0	0	0	0	2	1	0	0	1	3	1	1
**3.5.2.-**	0	0	0	0	0	0	0	0	0	0	0	0	0	0	0	0
**3.5.2.2**	1	2	1	0	0	0	1	0	4	1	1	1	0	4	0	0
**3.5.4.-**	21	7	19	11	12	14	17	10	13	8	15	15	9	21	17	12
**3.5.4.5**	13	5	13	7	12	7	10	4	9	7	6	9	8	13	7	8
**3.5.5.1**	0	1	0	0	0	0	0	0	0	2	0	0	1	0	0	0
**3.5.5.7**	0	0	0	0	0	0	0	0	0	0	0	0	0	0	0	0
**3.5.99.3**	0	0	0	0	0	0	0	0	1	0	0	0	0	0	0	0
**3.6.1.7**	3	0	2	2	1	0	3	1	8	2	4	1	1	12	1	0
**3.7.1.-**	1	0	0	1	0	0	1	2	1	0	2	2	1	5	0	1
**3.7.1.2**	6	5	7	6	3	4	2	1	2	4	1	2	5	2	6	2
**3.8.1.2**	6	4	10	5	3	5	7	1	11	7	6	4	5	7	8	6
**3.8.1.3**	0	0	0	0	0	0	0	0	0	1	0	0	0	0	0	0
**3.8.1.5**	0	1	0	0	0	0	0	0	1	0	0	0	0	0	0	0
**4.1.1.-**	13	8	11	8	8	11	5	4	8	11	8	5	8	7	9	6
**4.1.1.44**	14	16	19	12	12	21	15	10	18	9	10	10	14	20	14	12
**4.1.1.55**	0	0	0	0	0	0	0	0	0	0	0	0	0	0	0	0
**4.1.1.7**	0	0	0	0	0	0	0	0	0	0	0	0	0	0	0	0
**4.1.1.70**	1	1	0	0	0	0	0	0	0	2	0	0	0	0	0	0
**4.1.1.77**	0	0	0	0	0	0	0	0	0	0	0	0	0	0	0	0
**4.1.2.-**	7	2	4	3	6	2	1	5	6	5	5	3	1	11	3	2
**4.1.3.-**	27	6	15	9	14	14	19	5	17	12	20	7	8	18	15	10

EC/KO	CH-NOF008	CH-NOF009	CH-NOF010	CH-NOF011	CH-NOF012	CH-NOF013	CH-NOF014	CH-NOM001	CH-NOM002	CH-NOM004	CH-NOM005	CH-NOM007	CH-NOM008	CH-NOM009	CH-NOM010	CH-NOM012
4.1.3.39	0	1	1	1	0	1	0	0	0	0	0	0	0	0	0	0
4.1.99.-	4	1	2	0	2	1	0	1	0	1	3	3	0	1	3	0
4.2.1.-	57	36	52	30	28	48	30	14	40	43	33	29	39	38	33	25
4.2.1.17	1	4	8	2	3	6	0	4	5	3	4	1	2	6	2	2
4.2.1.80	0	0	0	0	0	1	0	0	0	1	0	0	0	0	1	0
4.2.1.83	0	0	0	0	0	0	0	0	0	0	0	2	0	0	0	0
4.2.1.84	0	0	0	0	0	1	2	0	0	0	1	0	0	0	0	0
5.1.2.2	0	0	0	0	0	0	0	0	0	0	0	0	0	0	0	0
5.2.1.2	0	0	0	0	0	0	0	0	0	0	0	0	0	0	0	0
5.3.3.4	0	0	0	0	0	0	0	0	0	0	0	0	0	0	0	0
5.3.99.-	0	1	2	0	1	0	2	0	5	2	5	1	1	8	0	4
5.4.99.-	1	1	0	0	1	0	0	0	0	0	0	0	0	0	1	1
5.5.1.1	2	4	5	5	2	6	3	2	2	4	2	2	4	3	6	0
5.5.1.2	0	0	0	0	0	0	0	0	0	0	0	0	0	0	0	0
6.2.1.-	0	0	0	0	0	0	0	0	0	0	0	0	0	0	0	0
6.3.5.2	24	8	17	7	12	14	18	7	21	13	21	15	14	22	12	12
K00002	2	0	0	0	1	0	1	1	2	0	2	0	0	2	0	0
K00055	0	0	0	0	0	0	0	0	0	0	0	0	0	0	0	0
K00074	11	5	0	3	1	3	3	4	6	0	6	4	0	4	5	1
K00088	25	15	21	10	9	19	17	10	18	15	12	10	14	26	14	13
K00100	53	24	47	26	31	46	28	18	42	38	41	21	31	49	27	18
K00128	1	0	0	0	1	0	1	1	4	1	2	0	0	2	1	1
K00129	0	0	0	0	0	0	0	0	0	0	0	0	0	0	0	0
K00132	1	1	0	0	0	0	0	0	0	1	0	0	0	0	0	0
K00141	0	0	0	0	0	0	0	0	0	0	0	0	0	0	0	0
K00146	0	0	0	0	0	0	0	0	0	0	1	0	0	0	0	0
K00148	0	0	0	0	0	0	0	0	0	0	0	0	0	0	0	0
K00155	0	0	0	0	0	0	0	0	0	0	1	0	1	0	0	0
K00169	5	1	1	0	1	2	1	0	4	1	0	2	0	3	2	0
K00224	0	1	1	0	0	0	0	0	0	1	0	0	0	0	0	0
K00274	0	0	0	0	0	0	0	0	0	0	0	0	0	0	0	0
K00446	0	0	0	0	0	0	0	0	0	0	0	0	0	0	0	0
K00448	0	0	0	0	0	0	0	0	0	0	0	0	0	0	0	0
K00462	0	2	0	1	1	1	0	1	1	1	1	0	0	0	0	0
K00480	0	0	0	0	0	0	0	0	0	0	0	0	0	0	0	0
K00481	0	0	0	0	0	0	0	0	0	0	0	0	0	0	0	0
K00539	0	1	1	0	0	0	0	0	0	0	0	0	0	0	0	0
K00599	41	19	31	15	21	43	38	19	42	23	39	25	16	57	30	20
K00626	9	3	6	2	3	5	6	2	7	3	3	2	1	5	5	2
K00632	0	0	0	0	0	0	0	0	0	0	1	0	0	0	0	0
K00680	36	28	39	21	23	44	25	15	21	26	34	14	25	22	20	27
K00757	12	8	9	6	5	12	9	6	9	8	6	4	6	12	7	5
K00758	3	0	1	0	2	1	0	0	1	3	1	2	2	2	0	0
K00760	20	7	16	6	10	12	17	7	23	5	17	10	9	20	14	11
K00799	0	0	1	0	0	0	0	0	0	1	4	0	0	0	0	0
K00857	14	5	9	8	7	12	5	3	3	3	5	8	6	9	11	8
K00876	31	9	24	14	18	23	23	9	19	13	17	18	14	29	26	16
K01026	0	2	1	0	0	0	0	1	1	3	0	0	0	4	0	0
K01031	0	0	0	0	0	0	0	0	0	0	0	0	0	0	0	0

EC/KO	CH-NOF008	CH-NOF009	CH-NOF010	CH-NOF011	CH-NOF012	CH-NOF013	CH-NOF014	CH-NOM001	CH-NOM002	CH-NOM004	CH-NOM005	CH-NOM007	CH-NOM008	CH-NOM009	CH-NOM010	CH-NOM012
K01034	3	1	0	1	0	0	1	0	0	0	1	0	0	0	0	0
K01039	0	0	0	0	0	0	0	0	0	0	0	0	0	0	0	0
K01041	7	8	8	8	4	7	4	3	4	5	5	4	7	5	5	3
K01053	0	0	0	0	0	0	0	0	0	0	0	0	0	0	0	0
K01055	0	0	0	0	0	0	0	0	0	0	0	0	0	0	0	0
K01061	0	0	1	0	0	0	0	0	0	1	1	0	0	0	0	0
K01066	2	2	5	5	5	7	3	2	6	3	7	2	2	3	4	2
K01075	0	0	0	0	0	0	0	0	0	1	2	0	0	0	0	0
K01077	11	11	11	8	9	9	2	2	7	9	4	4	11	5	10	1
K01101	5	2	1	2	2	0	3	0	4	1	0	0	2	1	1	3
K01195	6	3	7	7	2	8	5	3	6	4	10	2	6	6	10	3
K01426	1	2	0	1	0	1	1	0	1	2	0	0	1	0	1	1
K01428	0	0	1	0	0	0	1	0	3	0	0	0	0	2	0	0
K01457	0	0	0	0	0	0	0	0	0	0	0	0	0	0	0	0
K01464	2	2	1	0	0	0	1	1	5	5	2	1	0	4	0	0
K01489	22	7	14	12	15	10	12	4	9	8	9	7	12	14	13	11
K01500	0	0	0	0	0	0	1	0	0	0	0	0	0	0	0	0
K01501	2	2	0	1	0	0	0	0	1	2	1	0	0	3	0	0
K01502	0	0	0	0	0	0	0	0	0	0	0	0	0	0	0	0
K01512	4	1	4	2	1	1	3	1	8	3	6	1	1	16	4	0
K01560	6	1	2	1	0	3	4	2	3	0	3	2	0	8	3	1
K01561	1	1	0	1	0	1	0	0	0	0	0	0	1	0	1	1
K01563	0	0	0	0	0	0	0	0	0	0	0	0	0	0	0	0
K01564	1	1	1	0	2	0	1	1	1	0	1	0	1	0	1	0
K01607	9	7	11	6	9	8	7	3	10	5	3	7	5	6	6	5
K01612	0	0	0	0	0	0	0	0	0	0	0	0	0	0	0	0
K01615	27	10	23	12	8	12	15	7	13	9	12	5	10	18	19	10
K01617	0	0	0	0	0	0	0	0	0	0	0	0	0	0	0	0
K01666	7	1	3	1	3	5	5	4	6	3	6	1	2	4	4	3
K01692	0	0	0	0	0	0	0	0	0	0	0	0	0	0	0	0
K01721	0	0	0	0	0	0	0	0	0	0	0	0	0	0	0	0
K01726	6	6	11	4	3	8	5	1	6	10	3	3	9	6	5	6
K01781	0	1	1	0	0	0	0	0	1	0	1	0	1	0	0	0
K01821	0	2	1	1	2	0	2	0	1	1	1	2	1	0	1	1
K01856	0	0	0	0	0	0	0	0	0	0	0	0	0	0	0	0
K01857	0	0	1	0	1	0	0	1	0	0	0	0	0	0	0	0
K01913	0	0	0	0	0	0	0	0	1	0	0	0	0	1	0	0
K01951	22	8	16	8	11	16	16	7	21	10	14	11	10	19	15	7
K02554	0	0	0	0	0	0	0	0	0	1	0	0	0	0	0	0
K03381	0	0	0	0	0	0	0	0	0	0	0	0	0	0	0	0
K03382	2	0	0	0	0	0	0	0	1	0	0	0	0	0	0	0
K03464	0	0	0	0	0	0	0	0	0	0	0	0	0	0	0	0
K03518	8	1	5	3	1	3	6	3	17	6	14	1	1	23	2	0
K03862	0	0	0	0	0	0	0	0	0	0	0	0	0	0	0	0
K04099	0	0	0	0	0	0	0	0	0	0	0	0	0	0	0	0
K04100	0	0	0	0	0	0	0	0	0	0	0	0	0	0	0	0
K04102	0	0	0	0	0	0	0	0	0	0	0	0	0	0	0	0
K04116	0	0	0	0	0	0	0	0	0	0	0	0	0	0	0	0
K05394	0	0	2	0	1	0	0	0	0	0	0	0	0	0	0	0

EC/KO	CH-NOF008	CH-NOF009	CH-NOF010	CH-NOF011	CH-NOF012	CH-NOF013	CH-NOF014	CH-NOM001	CH-NOM002	CH-NOM004	CH-NOM005	CH-NOM007	CH-NOM008	CH-NOM009	CH-NOM010	CH-NOM012
**K05549**	0	0	0	0	0	0	0	0	0	0	0	0	0	0	0	0
**K05783**	0	0	0	0	0	0	0	0	0	0	0	0	0	0	0	0
**K05797**	0	0	0	0	0	0	0	0	0	0	0	0	0	0	0	0
**K06281**	1	2	1	0	0	0	0	1	1	1	0	0	1	0	0	0
**K06446**	5	2	2	2	5	3	1	3	7	3	5	2	3	6	4	2
**K06912**	0	0	0	0	0	0	0	0	0	0	0	0	0	0	0	0
**K07535**	0	0	0	0	0	0	0	0	0	0	0	0	0	0	0	0
**K07536**	2	1	3	0	1	3	0	1	1	0	1	0	2	0	2	0
**K08689**	0	0	0	0	0	0	0	0	0	0	0	0	0	0	0	0
**K08710**	0	0	0	0	0	0	0	0	0	0	0	0	0	0	0	0
**K09461**	0	0	0	0	0	0	0	0	1	0	0	0	0	0	0	0
**K10217**	0	0	0	0	0	0	0	0	0	0	0	0	0	0	0	0
**K10218**	0	0	0	0	0	0	0	0	1	1	0	0	0	0	0	0
**K10220**	0	0	0	0	0	0	0	0	0	0	0	0	0	0	0	0
**K11180**	0	0	0	0	0	0	0	0	0	0	0	0	0	0	0	0
**K13953**	0	0	2	0	0	0	0	0	0	0	1	0	0	0	0	1
**K14333**	0	0	0	0	0	0	0	0	0	0	1	0	0	0	0	0
**K14519**	0	0	0	0	0	0	0	0	0	0	0	0	0	0	0	0
**K15054**	0	0	0	0	0	0	0	0	0	1	0	0	0	0	0	0
**K16173**	0	1	0	0	0	0	0	0	1	0	0	0	0	1	0	0
**K16514**	0	0	0	0	0	0	0	0	1	0	1	0	0	0	0	0
**K16874**	0	0	0	0	0	0	0	0	0	0	0	0	0	0	0	0

EC/KO	CH-NOM013	CH-NOM014	CH-NOM015	CH-NOM016	CH-NOM017	CH-NOM018	CH-NOM019	CH-NOM020	CH-NOM022	CH-NOM023	CH-NOM025	CH-NOM026	CH-NOM027	CH-NOM028	CH-NOM029	DA-AD-1
**1.1.1.-**	22	41	38	39	25	47	33	43	29	33	33	46	32	55	30	39
**1.1.1.1**	18	24	19	29	26	42	26	28	25	18	24	47	25	44	38	23
**1.1.1.157**	0	0	0	2	1	2	0	0	0	0	0	1	0	1	0	1
**1.1.1.205**	13	20	15	17	13	22	13	18	14	13	13	20	17	22	16	18
**1.1.1.35**	1	1	1	0	0	3	0	3	1	1	1	3	2	4	2	0
**1.12.99.6**	1	1	0	0	1	0	0	0	1	1	2	1	1	3	2	1
**1.13.11.-**	0	0	0	0	0	0	0	1	0	0	0	0	0	0	0	0
**1.13.11.1**	0	0	0	0	0	0	0	0	0	0	0	0	0	0	0	0
**1.13.11.2**	0	0	0	0	0	0	0	0	0	0	0	0	0	0	0	0
**1.13.11.3**	0	0	0	0	0	0	0	0	0	0	0	0	0	0	0	0
**1.13.11.39**	0	0	0	0	0	0	0	0	0	0	0	0	0	0	0	0
**1.13.11.5**	0	0	0	0	0	0	0	0	0	0	0	0	0	0	0	0
**1.13.11.8**	0	0	0	0	0	0	0	1	0	0	0	1	0	0	0	0
**1.14.12.10**	0	0	0	0	0	0	0	1	0	0	1	1	0	1	1	0
**1.14.12.13**	0	0	0	0	0	0	0	0	0	0	0	0	0	0	0	0
**1.14.12.18**	0	0	0	0	0	0	0	0	0	0	0	0	0	0	0	0
**1.14.13.-**	0	0	0	0	0	0	0	0	0	0	2	6	2	3	2	0
**1.14.13.1**	0	0	0	0	0	0	0	0	0	0	0	0	0	0	0	0
**1.14.13.2**	0	0	0	0	0	0	0	1	0	0	0	1	0	0	0	0
**1.14.13.50**	0	0	0	0	0	0	0	0	0	0	0	0	0	0	0	0
**1.14.13.7**	0	0	0	0	0	0	0	0	0	0	0	0	0	1	0	0
**1.14.13.8**	0	0	0	0	0	0	0	0	0	0	0	0	0	0	0	0
**1.14.13.82**	0	0	0	0	0	0	0	1	0	0	0	0	0	0	0	0
**1.14.99.-**	0	0	0	0	0	0	0	0	0	0	0	1	0	0	0	0
**1.17.99.1**	0	0	0	0	0	0	0	0	0	0	0	0	0	0	0	0
**1.18.6.1**	0	0	0	0	0	0	0	0	1	0	0	0	0	0	0	0
**1.2.1.-**	0	0	0	0	0	0	0	2	0	0	2	2	0	2	1	0
**1.2.1.10**	1	6	0	1	3	8	3	3	2	3	4	4	3	5	5	0
**1.2.1.3**	3	4	1	3	3	6	5	6	2	3	3	12	9	11	7	4
**1.2.1.39**	0	0	0	0	0	0	0	0	0	0	0	1	0	0	0	0
**1.2.7.1**	8	6	6	7	8	9	7	4	5	5	3	3	2	5	0	10
**1.2.99.2**	2	0	0	1	2	4	3	3	0	1	2	9	3	5	5	2
**1.3.1.-**	0	0	0	0	0	0	0	1	2	0	0	0	0	0	0	0
**1.3.1.2**	0	0	0	0	0	0	0	0	0	0	1	1	0	2	1	0
**1.3.1.25**	0	0	0	0	0	0	0	0	0	0	0	0	0	0	0	0
**1.3.99.-**	0	0	0	0	0	0	0	0	0	0	0	0	0	0	0	0
**1.6.5.-**	30	25	35	36	25	41	36	30	26	29	21	27	12	32	23	49
**1.7.1.-**	7	5	4	9	8	9	7	6	6	8	6	12	4	5	7	12
**1.8.99.3**	0	0	0	0	0	0	0	1	0	0	0	2	0	0	0	0
**2.1.1.-**	126	134	129	163	128	235	155	145	128	108	141	204	142	174	154	186
**2.3.1.-**	86	88	91	113	59	130	114	106	92	105	90	117	65	120	101	99
**2.3.1.16**	3	1	1	2	2	2	2	0	2	1	0	1	2	2	0	1
**2.3.1.5**	0	0	0	0	0	0	0	0	0	0	1	1	0	1	1	0
**2.3.1.9**	0	1	0	1	0	0	0	3	1	1	3	3	0	1	2	0
**2.4.2.10**	11	8	8	11	12	16	9	11	5	5	6	13	8	10	9	13
**2.4.2.3**	5	12	2	4	4	7	4	3	6	2	1	9	6	3	5	7
**2.4.2.4**	3	0	1	2	3	4	4	5	0	0	3	4	4	2	2	0
**2.4.2.8**	7	10	4	9	6	10	7	7	6	7	6	11	8	14	8	12
**2.5.1.-**	14	12	17	17	7	19	11	15	16	12	17	12	5	14	10	23

EC/KO	CH-NOM013	CH-NOM014	CH-NOM015	CH-NOM016	CH-NOM017	CH-NOM018	CH-NOM019	CH-NOM020	CH-NOM022	CH-NOM023	CH-NOM025	CH-NOM026	CH-NOM027	CH-NOM028	CH-NOM029	DA-AD-1
**2.5.1.18**	0	0	0	0	0	1	1	7	0	0	3	7	0	3	4	0
**2.6.1.-**	15	20	26	23	18	26	19	20	18	15	26	24	13	30	15	21
**2.7.1.21**	6	6	4	10	6	2	5	3	4	1	2	5	1	5	4	11
**2.7.1.48**	12	14	15	22	11	21	10	14	16	16	12	21	9	23	14	28
**2.7.4.-**	7	8	11	13	9	13	10	10	7	6	7	11	5	8	8	17
**2.8.3.-**	3	2	2	0	2	0	1	2	1	1	2	1	1	2	2	0
**2.8.3.1**	0	0	0	0	0	0	0	0	0	0	0	0	1	2	0	0
**2.8.3.12**	0	2	0	0	0	1	1	0	0	0	0	0	0	0	1	2
**2.8.3.6**	0	0	1	0	0	0	0	0	0	0	0	1	0	0	0	1
**2.8.3.8**	1	1	0	0	0	2	1	1	1	2	1	2	3	3	1	1
**3.1.1.-**	2	6	1	4	3	3	6	4	5	3	2	7	6	8	5	1
**3.1.1.1**	1	5	3	6	1	5	1	2	2	6	5	2	1	6	3	4
**3.1.1.17**	1	3	3	3	0	0	1	1	0	2	2	2	0	2	0	0
**3.1.1.2**	0	0	0	0	0	0	0	0	0	0	1	3	0	1	1	1
**3.1.1.24**	1	1	0	3	1	3	2	2	0	0	1	1	2	1	2	3
**3.1.1.45**	0	0	0	0	0	0	0	2	0	0	0	0	0	0	0	0
**3.1.2.-**	0	0	1	0	0	2	0	1	0	0	1	2	1	2	2	0
**3.1.2.23**	4	2	3	7	0	4	1	2	3	3	3	1	0	2	3	0
**3.1.3.1**	13	18	12	18	7	14	7	14	16	16	14	10	6	18	15	8
**3.1.3.2**	4	6	6	6	2	7	2	7	7	5	6	4	1	5	6	3
**3.1.3.41**	0	0	0	0	0	0	0	0	0	0	0	0	0	0	0	0
**3.2.1.31**	2	1	1	1	4	3	3	6	3	1	6	6	0	1	3	4
**3.3.2.9**	0	0	0	0	0	0	0	0	0	0	0	1	0	0	0	0
**3.5.1.-**	12	9	11	13	14	18	14	11	8	5	8	24	15	15	9	20
**3.5.1.4**	1	4	2	2	2	2	1	3	1	2	0	2	3	2	1	2
**3.5.1.5**	1	1	0	1	0	1	0	1	1	1	0	3	0	0	0	1
**3.5.1.54**	0	1	0	1	1	6	3	2	1	0	2	5	3	2	4	1
**3.5.1.6**	1	1	0	0	0	1	1	0	0	1	0	3	2	1	0	1
**3.5.2.-**	0	0	0	0	0	0	0	0	0	0	0	0	0	0	0	0
**3.5.2.2**	0	2	0	0	1	1	2	0	0	0	0	4	2	3	1	0
**3.5.4.-**	9	8	12	14	14	13	16	11	14	10	9	16	14	18	14	31
**3.5.4.5**	8	4	7	9	5	10	9	4	8	6	5	10	5	6	7	11
**3.5.5.1**	0	2	0	0	0	0	0	1	1	1	1	1	0	1	2	1
**3.5.5.7**	0	0	0	0	0	0	0	0	0	0	0	1	0	0	0	0
**3.5.99.3**	0	1	0	0	0	0	0	0	0	0	0	0	0	0	0	0
**3.6.1.7**	3	2	1	1	1	4	1	2	1	1	2	5	4	4	4	1
**3.7.1.-**	1	0	0	0	1	2	1	1	0	0	0	3	2	0	1	1
**3.7.1.2**	2	3	5	4	0	3	4	5	2	5	2	1	1	5	2	2
**3.8.1.2**	4	5	6	5	3	7	5	3	6	7	6	7	5	7	7	1
**3.8.1.3**	0	0	0	0	0	0	0	0	0	0	0	0	0	0	0	0
**3.8.1.5**	0	0	0	0	0	0	0	0	1	1	0	1	2	1	0	0
**4.1.1.-**	6	9	5	7	5	8	4	12	8	6	7	13	4	12	8	6
**4.1.1.44**	10	16	10	21	10	16	14	14	11	9	8	13	8	17	12	18
**4.1.1.55**	0	0	0	0	0	0	0	0	0	0	0	0	0	0	0	0
**4.1.1.7**	0	0	0	0	0	0	0	0	0	0	0	0	0	0	0	0
**4.1.1.70**	0	1	0	0	0	0	0	0	0	0	0	0	0	0	0	0
**4.1.1.77**	0	0	0	0	0	0	0	0	0	0	0	0	0	0	0	0
**4.1.2.-**	7	4	1	2	8	7	4	2	2	1	6	7	3	5	7	5
**4.1.3.-**	8	13	5	6	14	21	11	12	8	8	12	11	11	8	11	9

EC/KO	CH-NOM013	CH-NOM014	CH-NOM015	CH-NOM016	CH-NOM017	CH-NOM018	CH-NOM019	CH-NOM020	CH-NOM022	CH-NOM023	CH-NOM025	CH-NOM026	CH-NOM027	CH-NOM028	CH-NOM029	DA-AD-1
**4.1.3.39**	1	0	0	0	0	0	2	1	0	1	1	0	0	0	3	0
**4.1.99.-**	1	1	1	1	0	3	0	0	0	1	1	2	0	1	1	1
**4.2.1.-**	27	38	40	39	16	41	24	32	26	35	33	30	20	36	39	38
**4.2.1.17**	1	3	2	2	4	3	1	0	4	4	2	4	6	10	6	9
**4.2.1.80**	0	0	0	0	0	0	0	1	0	0	0	0	0	0	0	0
**4.2.1.83**	0	1	0	0	0	2	0	1	1	0	0	1	0	0	1	0
**4.2.1.84**	0	1	0	0	0	1	0	1	1	0	0	0	0	0	0	0
**5.1.2.2**	0	0	0	0	0	0	0	0	0	0	0	0	0	0	1	0
**5.2.1.2**	0	0	0	0	0	0	0	0	0	0	0	0	0	0	0	0
**5.3.3.4**	0	0	0	0	0	0	0	0	0	0	0	0	0	0	0	0
**5.3.99.-**	1	1	3	2	3	1	2	2	2	0	1	2	3	7	3	1
**5.4.99.-**	1	0	1	0	0	0	1	1	0	0	0	0	0	0	0	1
**5.5.1.1**	0	4	5	3	2	3	3	4	1	4	6	1	1	2	5	0
**5.5.1.2**	0	0	0	0	0	0	0	0	0	0	0	0	0	0	0	0
**6.2.1.-**	0	0	0	0	0	0	0	0	0	0	0	1	0	0	0	0
**6.3.5.2**	14	19	13	7	10	21	18	15	12	16	10	17	12	13	12	11
**K00002**	0	0	0	0	0	0	0	0	0	0	0	1	2	3	0	1
**K00055**	0	0	0	0	0	0	0	0	0	0	0	1	0	0	0	0
**K00074**	2	1	3	4	1	7	5	8	1	2	3	5	3	6	2	2
**K00088**	11	16	13	11	13	18	12	15	11	11	13	8	12	19	14	16
**K00100**	20	38	35	27	24	44	34	32	26	21	19	35	21	46	26	21
**K00128**	0	0	0	1	0	0	0	3	0	0	2	4	1	3	2	0
**K00129**	0	0	0	0	0	0	0	0	0	0	0	0	0	0	0	0
**K00132**	0	1	0	0	0	0	0	0	0	1	0	0	0	1	0	0
**K00141**	0	0	0	0	0	0	0	0	0	0	0	1	0	0	0	0
**K00146**	0	0	0	0	0	0	0	0	0	0	0	3	0	0	0	0
**K00148**	0	0	0	0	0	0	0	0	0	0	0	0	0	0	0	0
**K00155**	0	0	0	0	0	0	0	0	0	0	1	0	0	1	0	0
**K00169**	1	1	0	2	0	5	2	1	1	0	0	1	0	0	0	0
**K00224**	0	0	0	0	0	1	2	1	0	0	0	1	0	0	0	1
**K00274**	0	0	0	0	0	0	0	0	0	0	0	0	0	0	0	0
**K00446**	0	0	0	0	0	0	0	0	0	0	0	0	0	0	0	0
**K00448**	0	0	0	0	0	0	0	0	0	0	0	1	0	0	0	0
**K00462**	0	0	1	0	1	1	0	0	0	1	1	0	0	0	1	0
**K00480**	0	0	0	0	0	0	0	0	0	0	0	0	0	0	0	0
**K00481**	0	0	0	0	0	0	0	0	0	0	0	1	0	0	0	0
**K00539**	0	1	0	0	0	1	1	0	0	0	0	0	0	0	0	0
**K00599**	20	19	29	34	20	48	18	20	17	22	23	42	24	26	25	25
**K00626**	4	3	3	4	4	4	4	2	3	3	3	3	4	4	4	1
**K00632**	0	0	0	0	0	0	0	1	0	0	0	0	0	0	0	0
**K00680**	22	26	21	27	16	23	25	15	25	25	22	37	13	28	30	21
**K00757**	6	11	11	10	6	10	7	9	10	7	3	9	4	10	4	8
**K00758**	1	0	2	1	1	1	1	2	0	0	1	1	1	1	2	0
**K00760**	11	10	8	14	8	18	14	10	9	8	9	16	11	15	13	21
**K00799**	0	0	0	1	0	0	0	4	0	0	1	4	1	2	2	0
**K00857**	6	6	8	11	8	4	10	6	4	1	5	7	2	7	5	16
**K00876**	13	13	19	19	17	21	16	22	14	15	14	16	11	18	14	22
**K01026**	0	2	0	0	0	1	0	0	0	0	1	2	4	0	0	1
**K01031**	0	0	0	0	0	0	0	0	0	0	0	1	0	0	0	0

EC/KO	CH-NOM013	CH-NOM014	CH-NOM015	CH-NOM016	CH-NOM017	CH-NOM018	CH-NOM019	CH-NOM020	CH-NOM022	CH-NOM023	CH-NOM025	CH-NOM026	CH-NOM027	CH-NOM028	CH-NOM029	DA-AD-1
K01034	0	0	0	0	0	0	1	1	1	1	0	1	1	2	0	3
K01039	0	2	0	0	0	0	1	0	0	0	0	0	0	0	0	2
K01041	4	6	6	8	3	7	4	8	6	4	4	4	1	7	4	3
K01053	0	0	0	0	0	0	0	1	0	0	0	1	0	0	0	0
K01055	0	0	0	0	0	0	0	0	0	0	0	0	0	0	0	0
K01061	0	0	0	0	0	0	0	2	0	0	0	0	0	0	0	0
K01066	1	4	4	3	0	3	2	3	0	4	5	2	2	5	6	2
K01075	0	0	0	0	0	2	0	2	0	0	0	2	0	0	1	0
K01077	3	8	9	10	4	6	4	11	6	10	8	3	2	10	8	3
K01101	3	2	2	1	1	2	4	0	2	0	0	0	2	0	1	2
K01195	2	3	7	5	3	8	5	4	2	4	6	5	2	4	6	3
K01426	1	2	0	0	0	0	2	0	1	1	0	1	0	1	0	0
K01428	1	0	0	1	0	0	0	0	0	1	0	2	1	1	0	0
K01457	0	0	0	0	0	0	0	0	0	0	0	0	1	0	0	0
K01464	0	2	0	0	1	1	6	0	0	0	1	4	5	6	2	0
K01489	10	6	10	13	8	12	16	11	11	8	9	12	7	9	10	18
K01500	0	0	0	0	0	0	0	0	0	0	0	0	0	0	0	0
K01501	0	1	0	0	0	0	1	1	1	0	0	3	1	0	0	1
K01502	0	0	0	0	0	0	0	0	0	0	0	1	0	0	0	0
K01512	4	2	2	4	3	6	4	2	1	1	4	5	5	4	6	3
K01560	1	1	2	1	4	2	8	4	2	0	4	7	1	2	4	1
K01561	1	0	0	0	0	1	0	0	0	1	0	0	0	0	0	0
K01563	0	0	0	0	0	0	0	0	0	0	0	0	0	0	0	0
K01564	1	1	2	0	0	1	1	1	0	1	0	1	0	0	0	1
K01607	6	5	7	11	5	6	9	10	3	5	5	8	2	10	10	10
K01612	0	0	0	0	0	0	0	0	0	0	0	0	0	0	0	0
K01615	11	14	16	14	15	17	12	14	9	9	11	10	6	9	9	4
K01617	0	0	0	0	0	0	0	0	0	0	0	0	0	0	0	0
K01666	3	2	1	2	3	5	3	3	0	2	2	4	4	1	8	1
K01692	0	0	0	0	0	0	0	0	0	0	0	0	0	0	0	0
K01721	0	0	0	0	0	0	0	0	0	0	0	0	0	0	0	0
K01726	5	3	6	8	4	6	4	6	6	9	5	4	4	8	7	3
K01781	0	1	0	0	3	1	0	0	0	0	0	0	3	2	0	0
K01821	0	1	2	1	1	1	1	2	1	0	0	3	0	0	0	0
K01856	0	0	0	0	0	0	0	0	0	0	0	0	0	0	0	0
K01857	0	0	0	0	1	1	1	0	0	0	0	0	0	0	0	0
K01913	0	1	0	0	0	0	0	0	0	0	0	0	1	0	0	0
K01951	7	14	16	8	11	18	15	16	10	14	12	14	9	10	10	7
K02554	0	0	0	0	0	0	0	1	0	0	0	0	0	0	0	0
K03381	0	0	0	0	0	0	0	0	0	0	0	0	0	0	0	0
K03382	0	1	0	0	0	0	2	0	0	0	0	0	0	0	0	1
K03464	0	0	0	0	0	0	0	0	0	0	0	0	0	0	0	0
K03518	4	6	3	3	10	8	8	3	4	5	3	10	13	19	7	13
K03862	0	0	0	0	0	0	0	1	0	0	0	1	0	0	0	0
K04099	0	0	0	0	0	0	0	1	0	0	0	1	0	0	0	0
K04100	0	0	0	0	0	0	0	0	0	0	0	0	0	0	0	0
K04102	0	0	0	0	0	0	0	0	0	0	0	0	0	0	0	0
K04116	0	0	0	0	0	0	0	0	0	0	0	0	0	0	0	0
K05394	0	0	0	0	2	0	1	0	0	0	0	0	0	0	0	1

EC/KO	CH-NOM013	CH-NOM014	CH-NOM015	CH-NOM016	CH-NOM017	CH-NOM018	CH-NOM019	CH-NOM020	CH-NOM022	CH-NOM023	CH-NOM025	CH-NOM026	CH-NOM027	CH-NOM028	CH-NOM029	DA-AD-1
**K05549**	0	0	0	0	0	0	0	0	0	0	0	1	0	0	0	0
**K05783**	0	0	0	0	0	0	0	0	0	0	0	0	0	0	0	0
**K05797**	0	0	0	0	0	0	0	0	0	0	0	0	0	0	0	0
**K06281**	1	1	0	0	1	0	0	0	1	1	2	2	1	3	2	1
**K06446**	1	3	4	4	0	2	1	4	2	2	1	4	4	2	0	8
**K06912**	0	0	0	0	0	0	0	0	0	0	0	0	0	0	0	0
**K07535**	0	0	0	0	0	0	0	0	0	0	0	0	0	0	0	0
**K07536**	2	2	3	3	1	0	2	2	2	1	1	3	0	4	2	3
**K08689**	0	0	0	0	0	0	0	0	0	0	0	0	0	0	0	0
**K08710**	0	0	0	0	0	0	0	0	0	0	0	0	0	0	0	0
**K09461**	0	0	0	0	0	0	0	0	0	0	0	1	1	1	0	0
**K10217**	0	0	0	0	0	0	0	0	0	0	0	0	0	0	0	0
**K10218**	0	0	0	0	0	0	0	0	0	1	0	0	0	0	0	0
**K10220**	0	0	0	0	0	0	0	0	0	0	0	0	0	0	0	0
**K11180**	0	0	0	0	0	0	0	0	0	0	0	0	0	0	0	1
**K13953**	0	0	0	0	0	0	0	1	0	0	1	3	0	1	1	0
**K14333**	0	0	0	0	0	0	0	1	0	0	0	0	0	0	0	0
**K14519**	0	0	0	0	0	0	0	0	0	0	0	0	0	0	0	0
**K15054**	0	0	0	0	0	1	0	0	0	0	0	2	0	0	1	0
**K16173**	0	0	0	0	0	1	0	1	1	0	0	0	1	0	1	0
**K16514**	0	0	0	0	0	0	0	1	0	0	0	1	1	1	1	0
**K16874**	0	0	0	0	0	0	0	0	0	0	0	0	0	1	0	0

EC/KO	DA-AD-10	DA-AD-11	DA-AD-12	DA-AD-13	DA-AD-14	DA-AD-15	DA-AD-16	DA-AD-17	DA-AD-18	DA-AD-19	DA-AD-2	DA-AD-20	DA-AD-21	DA-AD-22	DA-AD-23	DA-AD-24	DA-AD-25	DA-AD-26	DA-AD-27
**1.1.1.-**	12	29	51	17	71	63	21	71	21	9	34	23	73	28	17	10	31	78	35
**1.1.1.1**	8	20	31	7	48	47	13	42	7	4	18	12	40	30	7	12	11	46	35
**1.1.1.157**	0	0	1	0	2	0	0	1	0	0	1	0	1	0	0	1	0	0	0
**1.1.1.205**	6	4	30	6	35	27	9	34	10	4	9	7	24	8	4	6	11	26	12
**1.1.1.35**	1	3	2	2	3	3	0	2	1	0	2	0	2	2	0	0	1	3	1
**1.12.99.6**	0	0	1	0	5	3	0	6	0	0	1	0	3	0	1	0	2	7	1
**1.13.11.-**	0	0	0	0	0	0	0	0	0	0	0	0	0	0	0	0	0	0	0
**1.13.11.1**	0	0	0	0	0	0	0	0	0	0	0	0	0	0	0	0	0	0	0
**1.13.11.2**	0	0	0	0	0	0	0	0	0	0	0	0	0	0	0	0	0	0	0
**1.13.11.3**	0	1	0	0	0	0	0	0	0	0	0	0	0	0	0	0	0	0	0
**1.13.11.39**	0	0	0	0	0	0	0	0	0	0	0	0	0	0	0	0	0	0	0
**1.13.11.5**	0	1	0	0	0	0	0	0	0	0	0	0	0	0	0	0	0	0	0
**1.13.11.8**	0	1	0	0	0	0	0	0	0	0	0	0	0	0	0	0	0	0	0
**1.14.12.10**	0	0	0	0	0	0	0	1	0	0	0	0	0	0	1	0	0	1	0
**1.14.12.13**	0	0	0	0	0	0	0	0	0	0	0	0	0	0	0	0	0	0	0
**1.14.12.18**	0	0	0	0	0	0	0	0	0	0	0	0	0	0	0	0	0	0	0
**1.14.13.-**	0	6	0	0	0	1	0	0	0	0	1	0	1	0	0	0	0	1	1
**1.14.13.1**	0	0	0	0	0	0	0	0	0	0	0	0	0	0	0	0	0	0	0
**1.14.13.2**	0	1	0	0	0	0	0	0	0	0	0	0	0	0	0	0	0	0	0
**1.14.13.50**	0	0	0	0	0	0	0	0	0	0	0	0	0	0	0	0	0	0	0
**1.14.13.7**	0	0	1	0	0	0	0	0	0	0	0	0	1	0	0	0	0	0	0
**1.14.13.8**	0	0	0	0	0	0	0	0	0	0	0	0	0	0	0	0	0	0	0
**1.14.13.82**	0	2	0	0	0	0	0	0	0	0	0	0	0	0	0	0	0	2	0
**1.14.99.-**	0	0	0	0	0	0	0	0	0	0	0	0	0	0	0	0	0	0	0
**1.17.99.1**	0	0	0	0	0	0	0	0	0	0	0	0	0	0	0	0	0	0	0
**1.18.6.1**	0	0	0	0	0	0	0	0	0	0	0	0	0	0	0	1	0	0	0
**1.2.1.-**	0	2	0	0	0	0	0	0	0	0	0	0	0	0	0	0	0	1	0
**1.2.1.10**	1	1	6	2	4	3	1	7	1	1	1	0	5	3	2	1	5	3	4
**1.2.1.3**	1	8	12	1	10	11	3	12	5	1	5	1	10	5	3	2	7	11	5
**1.2.1.39**	0	0	0	0	0	0	0	0	0	0	0	0	0	0	0	0	0	0	0
**1.2.7.1**	4	3	15	2	17	17	6	16	4	2	20	5	9	5	2	1	4	16	7
**1.2.99.2**	1	1	1	0	4	8	0	2	1	1	2	1	4	1	0	0	2	3	2
**1.3.1.-**	0	0	0	0	0	0	0	1	0	0	0	0	0	0	0	0	0	0	0
**1.3.1.2**	0	0	1	0	1	1	0	2	0	0	0	0	2	0	0	0	3	2	1
**1.3.1.25**	0	1	0	0	0	0	0	0	0	0	0	0	0	0	0	0	0	0	0
**1.3.99.-**	0	0	0	0	0	2	0	0	0	0	0	0	0	0	1	0	0	1	0
**1.6.5.-**	14	18	55	11	73	59	22	56	23	8	41	27	54	28	17	12	26	58	40
**1.7.1.-**	3	5	12	2	8	11	4	10	2	2	5	5	11	5	3	2	6	10	8
**1.8.99.3**	0	0	0	0	1	0	0	0	0	0	0	0	1	0	0	0	0	1	0
**2.1.1.-**	103	101	336	83	432	392	106	345	89	40	180	128	275	171	97	101	152	329	201
**2.3.1.-**	50	72	169	62	231	228	59	190	58	24	115	80	147	118	41	48	86	214	122
**2.3.1.16**	1	0	2	0	3	0	0	1	0	0	0	0	2	0	0	0	0	1	2
**2.3.1.5**	0	0	0	0	0	0	0	0	0	0	0	0	0	0	0	0	0	1	0
**2.3.1.9**	0	2	1	1	1	2	0	1	0	0	1	0	0	0	0	0	0	3	1
**2.4.2.10**	6	3	23	4	18	21	5	19	5	4	11	5	17	6	5	7	10	20	12
**2.4.2.3**	8	5	10	1	14	14	3	12	3	0	3	4	9	5	0	2	5	9	4
**2.4.2.4**	1	1	7	0	6	7	0	7	2	0	2	3	1	2	3	0	3	6	4
**2.4.2.8**	3	4	17	2	21	20	3	15	4	1	6	2	15	10	6	2	5	17	13
**2.5.1.-**	7	13	27	9	37	29	7	27	10	2	16	13	25	14	7	4	6	28	21

EC/KO	DA-AD-10	DA-AD-11	DA-AD-12	DA-AD-13	DA-AD-14	DA-AD-15	DA-AD-16	DA-AD-17	DA-AD-18	DA-AD-19	DA-AD-2	DA-AD-20	DA-AD-21	DA-AD-22	DA-AD-23	DA-AD-24	DA-AD-25	DA-AD-26	DA-AD-27
2.5.1.18	0	8	1	0	0	5	0	2	0	0	0	0	3	0	0	0	1	7	0
2.6.1.-	13	16	42	14	44	43	12	57	11	7	16	13	27	24	11	15	22	34	29
2.7.1.21	4	2	8	4	11	13	2	9	2	3	5	2	5	5	2	2	6	9	2
2.7.1.48	14	11	45	10	36	39	9	47	15	2	28	17	31	19	13	11	16	29	22
2.7.4.-	6	4	24	7	30	19	5	20	5	0	11	10	16	10	8	6	17	21	12
2.8.3.-	0	0	1	0	3	4	0	2	0	1	0	1	3	2	0	0	0	4	1
2.8.3.1	0	0	0	0	1	0	0	0	0	0	2	0	0	1	0	0	0	0	0
2.8.3.12	2	0	0	0	0	0	1	0	0	0	3	1	0	0	0	0	0	1	0
2.8.3.6	0	3	1	0	0	0	0	0	0	0	2	0	1	0	0	0	0	0	0
2.8.3.8	1	1	1	0	3	2	0	0	0	0	1	0	2	0	1	0	1	3	0
3.1.1.-	1	7	6	1	6	7	1	7	2	1	5	2	8	4	2	3	1	14	5
3.1.1.1	1	3	9	3	9	11	3	7	1	0	5	3	11	4	3	2	3	12	4
3.1.1.17	1	2	0	0	1	2	0	3	0	0	1	1	3	1	0	0	1	3	1
3.1.1.2	0	1	0	0	0	1	0	0	0	0	1	0	0	0	0	0	0	0	1
3.1.1.24	0	0	5	2	2	5	1	2	2	0	3	0	2	1	1	0	2	4	2
3.1.1.45	0	0	0	0	0	0	0	0	0	0	0	0	0	0	0	0	0	0	0
3.1.2.-	0	1	1	0	0	1	0	0	0	0	1	0	1	0	0	0	0	2	0
3.1.2.23	1	3	6	4	5	7	2	7	4	1	4	5	5	5	0	1	5	4	3
3.1.3.1	5	5	17	8	24	22	9	18	2	3	8	10	24	12	3	7	9	24	12
3.1.3.2	2	4	6	3	7	7	1	3	2	2	2	4	4	2	1	1	2	6	3
3.1.3.41	0	0	0	0	0	0	0	0	0	0	1	0	0	0	0	0	0	0	0
3.2.1.31	6	0	3	2	4	7	3	8	2	0	0	2	3	2	0	1	4	3	5
3.3.2.9	0	1	0	0	0	0	0	0	0	0	0	0	0	0	0	0	0	0	0
3.5.1.-	9	11	39	12	43	32	9	34	10	3	15	17	22	19	12	14	16	32	16
3.5.1.4	0	1	3	1	7	5	0	2	0	2	1	1	4	2	2	0	0	0	1
3.5.1.5	0	1	1	0	1	1	0	2	0	0	0	0	2	1	0	0	0	2	1
3.5.1.54	1	1	3	1	3	2	4	6	0	0	6	1	4	0	1	0	1	5	3
3.5.1.6	1	3	3	0	3	2	0	2	0	1	0	1	4	0	1	1	1	4	2
3.5.2.-	0	0	0	0	0	0	0	0	0	0	0	0	1	0	0	0	0	0	0
3.5.2.2	1	0	0	0	0	2	2	1	0	0	1	1	2	0	0	0	1	1	3
3.5.4.-	6	13	42	7	41	35	13	40	10	4	16	12	26	17	6	8	16	31	18
3.5.4.5	7	4	24	6	22	26	4	18	6	4	11	5	13	17	6	3	7	16	11
3.5.5.1	1	0	0	0	0	2	0	2	0	0	2	0	2	1	0	2	0	4	0
3.5.5.7	0	0	1	0	0	0	0	0	0	0	0	0	0	0	0	0	0	0	0
3.5.99.3	0	0	0	0	1	0	0	0	0	0	0	0	0	0	0	0	0	0	0
3.6.1.7	3	2	6	2	5	6	2	6	2	0	3	2	4	4	0	0	2	1	1
3.7.1.-	0	1	4	0	1	0	1	1	0	0	0	0	2	0	1	0	1	2	1
3.7.1.2	0	4	4	1	7	8	3	7	1	1	3	2	8	5	0	0	4	2	3
3.8.1.2	2	4	4	3	6	6	2	9	1	1	1	3	9	5	1	0	5	7	4
3.8.1.3	0	0	0	0	0	0	0	0	0	0	0	0	0	0	0	0	0	0	0
3.8.1.5	0	2	3	0	0	1	0	1	0	0	0	0	0	0	0	0	0	0	1
4.1.1.-	2	8	8	3	16	13	3	17	5	2	6	8	15	8	5	2	4	10	11
4.1.1.44	9	7	29	11	43	27	15	32	8	4	37	14	32	23	9	7	20	31	20
4.1.1.55	0	0	0	0	0	0	0	0	0	0	0	0	0	0	0	0	0	1	0
4.1.1.7	0	0	0	0	0	0	0	0	0	0	0	0	0	0	0	0	0	0	0
4.1.1.70	0	0	0	0	0	0	0	0	0	0	0	0	1	0	0	0	0	0	1
4.1.1.77	0	0	0	0	0	0	0	0	0	0	0	0	0	0	0	0	0	0	0
4.1.2.-	2	3	4	2	6	9	1	4	0	0	2	1	3	7	0	2	5	5	4
4.1.3.-	11	6	21	11	24	35	10	31	7	3	12	10	27	12	4	11	11	18	21

EC/KO	DA-AD-10	DA-AD-11	DA-AD-12	DA-AD-13	DA-AD-14	DA-AD-15	DA-AD-16	DA-AD-17	DA-AD-18	DA-AD-19	DA-AD-2	DA-AD-20	DA-AD-21	DA-AD-22	DA-AD-23	DA-AD-24	DA-AD-25	DA-AD-26	DA-AD-27
4.1.3.39	0	1	0	0	1	0	0	0	0	0	0	0	0	0	0	0	0	1	0
4.1.99.-	2	1	4	2	5	3	1	2	1	0	1	1	1	2	1	3	0	4	0
4.2.1.-	18	23	58	16	75	60	25	70	13	10	34	26	57	46	16	18	37	75	35
4.2.1.17	3	1	4	2	7	5	1	9	1	0	4	4	8	2	0	5	5	10	1
4.2.1.80	0	0	0	0	0	0	0	1	0	0	0	0	0	0	0	0	0	0	0
4.2.1.83	3	1	1	1	2	0	0	1	0	0	0	0	0	2	0	1	0	1	1
4.2.1.84	0	0	0	0	0	0	0	0	0	0	0	0	0	0	0	0	0	0	1
5.1.2.2	0	0	0	0	0	0	0	0	0	0	0	0	0	0	0	0	0	0	0
5.2.1.2	0	0	0	0	0	0	0	0	0	0	0	0	0	0	0	0	0	0	0
5.3.3.4	0	1	0	0	0	0	0	0	0	0	1	0	0	0	0	0	0	0	0
5.3.99.-	2	2	6	0	6	4	1	4	0	0	4	2	2	3	0	1	4	3	1
5.4.99.-	0	0	1	0	2	2	0	1	1	0	0	1	0	0	0	0	1	0	0
5.5.1.1	1	0	3	3	5	4	0	7	2	0	0	3	5	3	1	0	1	4	3
5.5.1.2	0	0	0	0	0	0	0	0	0	0	0	0	0	0	0	0	0	0	1
6.2.1.-	0	1	0	0	0	0	0	0	0	0	0	0	0	0	0	0	0	0	0
6.3.5.2	5	6	17	5	23	26	0	28	3	3	11	9	23	13	5	3	12	28	17
K00002	0	0	1	1	3	3	0	1	0	0	0	2	3	2	0	1	1	3	1
K00055	0	2	0	0	0	0	0	0	0	0	0	0	0	0	0	0	0	0	0
K00074	1	2	6	2	5	3	1	5	1	0	7	0	3	2	0	0	1	1	3
K00088	2	4	25	0	23	21	7	22	3	1	10	9	18	1	6	6	7	16	17
K00100	16	16	56	12	83	77	20	64	14	10	39	19	57	32	19	10	34	72	34
K00128	0	2	1	0	1	5	0	4	1	0	1	0	1	4	0	0	2	3	0
K00129	0	1	0	0	0	0	0	0	0	0	0	0	0	0	0	0	0	0	0
K00132	0	0	2	0	0	1	0	1	0	0	1	0	0	0	2	0	0	0	1
K00141	0	1	0	0	0	0	0	0	0	0	0	0	0	0	0	0	0	0	0
K00146	0	1	0	0	0	0	0	0	0	0	0	0	0	0	0	0	0	0	0
K00148	0	0	0	0	0	0	0	0	0	0	0	0	0	0	0	0	0	0	0
K00155	0	0	0	0	0	1	0	1	0	1	0	0	0	0	0	0	0	1	0
K00169	0	1	3	1	4	2	1	2	0	0	0	1	2	1	0	0	0	3	1
K00224	0	0	1	0	1	0	0	1	0	0	1	0	1	0	0	0	0	2	1
K00274	0	0	0	0	0	0	0	0	0	0	0	0	0	0	0	0	0	0	0
K00446	0	0	0	0	0	0	0	0	0	0	0	0	0	0	0	0	0	0	0
K00448	0	0	0	0	0	0	0	0	0	0	0	0	0	0	0	0	0	0	0
K00462	0	0	1	0	0	0	0	1	0	1	0	1	2	1	0	0	0	1	0
K00480	0	0	0	0	0	0	0	0	0	0	0	0	0	0	0	0	0	0	0
K00481	0	1	0	0	0	0	0	0	0	0	0	0	0	0	0	0	0	0	0
K00539	0	0	1	0	1	1	1	1	0	0	0	1	1	1	0	0	0	1	0
K00599	20	17	54	26	65	67	25	63	24	5	20	27	48	31	16	18	22	48	34
K00626	1	2	3	1	6	3	0	3	0	0	1	1	4	0	0	0	0	5	4
K00632	0	0	0	0	0	1	0	0	0	0	0	0	0	0	0	0	0	0	0
K00680	12	13	49	17	69	59	22	51	9	5	38	17	43	27	14	11	24	45	31
K00757	5	6	9	3	15	13	6	17	6	1	6	9	15	7	5	5	5	16	5
K00758	2	0	5	3	5	5	2	4	2	0	2	2	1	3	0	1	2	4	1
K00760	7	9	31	5	33	31	9	27	8	2	15	8	23	17	8	6	9	29	17
K00799	0	2	0	0	1	2	0	0	1	0	0	1	0	1	0	0	0	3	0
K00857	4	5	11	7	16	17	6	15	5	3	12	5	9	7	7	2	10	12	6
K00876	7	12	45	11	31	36	9	35	12	4	19	17	33	16	16	7	11	27	18
K01026	1	0	0	0	2	1	0	0	0	0	1	0	1	0	0	0	0	0	0
K01031	0	0	0	0	0	0	0	0	0	0	0	0	0	0	0	0	0	0	0

EC/KO	DA-AD-10	DA-AD-11	DA-AD-12	DA-AD-13	DA-AD-14	DA-AD-15	DA-AD-16	DA-AD-17	DA-AD-18	DA-AD-19	DA-AD-2	DA-AD-20	DA-AD-21	DA-AD-22	DA-AD-23	DA-AD-24	DA-AD-25	DA-AD-26	DA-AD-27
K01034	0	0	3	1	4	0	1	4	0	0	2	1	0	0	0	0	1	3	1
K01039	0	0	0	0	0	1	0	1	0	0	1	0	0	0	0	0	0	0	0
K01041	0	2	5	1	5	7	2	6	1	0	1	2	7	1	0	1	3	7	6
K01053	0	1	0	0	0	0	0	0	0	0	0	0	0	0	0	0	0	0	0
K01055	0	0	0	0	0	0	0	0	0	0	0	0	0	0	0	0	0	0	0
K01061	0	0	0	0	0	0	0	0	0	0	0	0	0	0	0	0	0	0	0
K01066	0	5	7	2	16	18	3	6	3	0	2	3	11	8	3	0	4	16	5
K01075	0	1	1	0	1	2	1	2	0	0	0	0	1	1	0	0	0	5	0
K01077	2	4	7	4	14	15	3	10	2	1	6	7	10	8	1	0	4	8	5
K01101	1	0	4	0	1	5	1	1	1	1	6	0	4	0	7	2	2	4	3
K01195	4	3	9	5	13	14	3	12	1	0	1	4	6	8	2	1	3	8	7
K01426	0	0	0	0	1	1	0	1	0	0	0	0	1	0	0	0	1	1	1
K01428	0	2	0	0	2	0	0	2	0	0	0	0	3	0	0	0	0	0	0
K01457	0	0	0	1	1	0	0	0	0	0	0	0	0	0	0	0	0	0	0
K01464	0	0	0	0	0	3	1	1	0	0	2	1	2	0	0	0	1	3	3
K01489	10	8	31	8	27	27	6	23	7	4	16	7	17	17	10	7	10	20	15
K01500	0	1	0	0	0	0	0	0	0	0	0	0	0	1	0	0	0	0	0
K01501	1	0	0	0	2	1	0	2	0	0	3	0	3	0	0	3	0	6	0
K01502	0	0	1	0	0	0	0	0	0	0	0	0	0	0	0	0	0	1	0
K01512	6	7	9	3	11	9	4	10	4	0	4	7	4	8	1	2	2	10	2
K01560	3	1	8	0	6	10	2	6	1	0	5	1	3	5	7	2	2	7	5
K01561	0	0	0	0	0	0	0	2	0	0	0	0	1	0	0	0	0	1	0
K01563	0	0	0	0	0	0	0	0	0	0	0	0	0	0	0	0	0	0	0
K01564	0	1	2	0	2	1	0	4	1	0	0	1	4	0	0	0	2	4	1
K01607	7	5	15	4	24	11	8	16	2	1	18	4	18	11	3	3	11	16	8
K01612	0	0	0	0	1	0	0	0	0	0	0	0	0	0	0	0	0	0	0
K01615	1	1	22	2	18	14	4	12	2	2	10	4	23	6	3	5	11	10	12
K01617	0	0	0	0	0	0	0	0	0	0	0	0	0	0	0	0	0	0	0
K01666	3	3	4	3	11	11	3	9	3	1	0	2	2	2	1	4	2	5	5
K01692	0	1	0	0	1	1	0	1	0	0	0	1	0	1	0	0	0	1	0
K01721	0	0	0	0	0	0	0	0	0	0	0	0	0	0	0	0	0	0	0
K01726	2	3	6	3	9	9	2	9	2	1	1	2	13	5	1	2	4	14	7
K01781	0	0	0	0	1	1	0	0	0	0	0	0	2	0	0	0	0	0	0
K01821	0	4	1	0	4	1	2	3	0	1	0	1	2	2	0	1	1	5	1
K01856	0	0	0	0	0	0	0	0	0	0	1	0	0	0	0	0	0	0	0
K01857	0	0	0	0	0	0	0	1	0	0	0	0	0	0	0	0	0	1	1
K01913	0	0	1	0	0	1	0	2	1	0	0	0	0	0	0	0	1	0	0
K01951	3	5	18	3	19	15	0	16	6	3	9	6	20	4	6	3	8	15	15
K02554	0	0	0	0	0	0	0	0	0	0	0	0	1	0	0	0	0	0	0
K03381	0	0	0	0	0	0	0	0	0	0	0	0	0	0	0	0	0	0	0
K03382	0	1	0	0	3	0	0	0	0	0	0	0	0	0	0	0	0	0	0
K03464	0	1	0	0	0	0	0	0	0	0	0	0	0	0	0	0	0	0	0
K03518	5	1	7	1	15	15	4	13	3	1	13	3	13	3	1	2	1	9	8
K03862	0	1	0	0	0	0	0	0	0	0	0	0	0	0	0	0	0	1	0
K04099	0	1	0	0	0	0	0	0	0	0	0	0	0	0	0	0	0	0	0
K04100	0	0	0	0	0	0	0	0	0	0	0	0	0	0	0	0	0	0	0
K04102	0	0	0	0	0	0	0	0	0	0	0	0	0	0	0	0	0	1	0
K04116	0	0	0	0	0	0	0	0	0	0	0	0	0	0	0	0	0	0	0
K05394	0	0	0	0	0	0	1	0	0	0	0	2	1	0	0	0	0	1	1

EC/KO	DA-AD-10	DA-AD-11	DA-AD-12	DA-AD-13	DA-AD-14	DA-AD-15	DA-AD-16	DA-AD-17	DA-AD-18	DA-AD-19	DA-AD-2	DA-AD-20	DA-AD-21	DA-AD-22	DA-AD-23	DA-AD-24	DA-AD-25	DA-AD-26	DA-AD-27
K05549	0	1	0	0	0	0	0	0	0	0	0	0	0	0	0	0	0	0	0
K05783	0	1	0	0	0	0	0	0	0	0	0	0	0	0	0	0	0	0	0
K05797	0	0	0	0	0	0	0	0	0	0	0	0	0	0	0	0	0	0	0
K06281	0	0	0	0	2	2	0	4	0	0	1	0	3	0	1	0	2	5	1
K06446	0	0	5	1	3	2	1	2	1	0	6	0	5	1	1	1	0	5	3
K06912	0	0	0	0	0	0	0	0	0	0	0	0	0	0	0	0	0	0	0
K07535	0	0	0	0	1	0	0	0	0	0	1	0	0	0	0	0	0	0	0
K07536	0	1	1	1	2	1	0	3	1	1	0	2	0	0	2	1	0	4	0
K08689	0	0	0	0	0	0	0	0	0	0	0	0	0	0	0	0	0	0	0
K08710	0	0	0	0	0	0	0	0	0	0	0	0	0	0	0	0	0	0	0
K09461	0	0	1	0	0	0	0	0	0	0	0	0	1	0	0	0	0	1	0
K10217	0	0	0	0	0	0	0	0	0	0	0	0	0	0	0	0	0	0	0
K10218	0	0	2	0	0	0	0	0	0	0	0	0	2	0	0	0	0	1	0
K10220	0	0	0	0	0	0	0	0	0	0	0	0	0	0	0	0	0	0	0
K11180	0	0	0	0	0	0	0	0	0	0	0	0	1	0	0	0	0	1	0
K13953	0	1	0	0	0	1	0	0	0	0	1	0	0	0	0	0	0	1	0
K14333	0	1	0	0	0	0	0	0	0	0	0	0	0	0	0	0	0	0	0
K14519	0	0	0	0	0	0	0	0	0	0	0	0	0	0	0	0	0	0	0
K15054	0	1	0	0	0	0	0	0	0	0	0	0	0	0	0	0	0	0	0
K16173	0	0	0	0	2	0	0	1	0	0	0	0	1	0	0	0	0	1	1
K16514	0	0	0	0	0	0	0	0	0	0	0	0	1	0	0	0	0	1	0
K16874	0	0	0	0	0	0	0	0	0	0	0	0	0	0	0	0	0	1	0

EC/KO	DA-AD-28	DA-AD-29	DA-AD-3	DA-AD-30	DA-AD-31	DA-AD-32	DA-AD-33	DA-AD-34	DA-AD-35	DA-AD-36	DA-AD-37	DA-AD-38	DA-AD-39	DA-AD-4	DA-AD-40	DA-AD-41	DA-AD-42	DA-AD-43	DA-AD-44
1.1.1.-	14	56	44	27	37	59	22	53	39	42	51	60	51	33	64	43	62	53	68
1.1.1.1	1	43	19	18	24	32	19	35	30	25	32	35	52	19	37	34	43	33	40
1.1.1.157	0	0	2	1	0	0	0	0	0	1	0	1	0	1	1	0	2	1	0
1.1.1.205	2	24	16	22	13	21	7	23	21	17	22	27	29	15	23	22	24	21	21
1.1.1.35	0	3	4	4	1	4	0	5	6	2	5	4	0	3	1	2	4	3	3
1.12.99.6	0	3	2	0	1	0	0	1	0	1	2	2	0	0	0	1	1	0	2
1.13.11.-	0	0	0	0	0	0	0	0	0	0	0	0	0	0	0	0	0	0	0
1.13.11.1	0	0	0	0	0	0	0	0	0	0	0	0	0	0	0	0	0	0	0
1.13.11.2	0	0	0	0	0	0	0	0	0	0	0	0	0	0	0	0	0	0	0
1.13.11.3	0	1	0	0	0	0	0	0	0	0	0	0	0	0	0	0	0	0	0
1.13.11.39	0	0	0	0	0	0	0	0	0	0	0	0	0	0	0	0	0	0	0
1.13.11.5	0	0	0	0	1	0	0	0	1	0	0	0	0	0	0	0	0	0	0
1.13.11.8	0	1	0	0	0	0	0	0	0	0	0	0	0	0	0	0	0	0	0
1.14.12.10	0	1	0	0	0	0	0	0	0	0	0	0	0	0	0	0	0	0	0
1.14.12.13	0	0	0	0	0	0	0	0	0	0	0	0	0	0	0	0	0	0	0
1.14.12.18	0	0	0	0	0	0	0	0	0	0	0	0	0	0	0	0	0	0	0
1.14.13.-	0	4	0	0	0	1	0	0	0	0	0	0	0	0	0	0	1	0	0
1.14.13.1	0	0	0	0	0	0	0	0	0	0	0	0	0	0	0	0	0	0	0
1.14.13.2	0	1	0	0	0	0	0	0	0	0	0	0	0	0	0	0	0	0	0
1.14.13.50	0	0	0	0	0	0	0	0	0	0	0	0	0	0	0	0	0	0	0
1.14.13.7	0	0	0	0	0	0	0	0	0	0	0	0	1	0	0	0	0	1	0
1.14.13.8	0	0	0	0	0	0	0	0	0	0	0	0	0	0	0	0	0	0	0
1.14.13.82	0	0	0	0	0	0	0	0	0	0	0	0	0	0	0	0	0	0	0
1.14.99.-	0	0	0	0	0	0	0	0	0	0	0	0	0	0	0	0	0	0	0
1.17.99.1	0	0	0	0	0	0	0	0	0	0	0	0	0	0	0	0	0	0	0
1.18.6.1	0	0	0	0	0	0	0	0	0	0	0	1	0	0	0	0	0	0	4
1.2.1.-	0	0	0	0	0	0	0	0	0	0	0	0	0	0	0	0	0	0	0
1.2.1.10	0	5	1	3	3	2	4	4	1	0	4	5	6	1	2	3	7	6	7
1.2.1.3	0	9	8	8	9	9	2	10	5	7	7	13	8	5	7	5	5	10	7
1.2.1.39	0	0	0	0	0	0	0	0	0	0	0	0	0	0	0	0	0	0	0
1.2.7.1	1	15	22	7	6	13	6	12	8	11	9	12	17	14	13	13	12	9	10
1.2.99.2	0	3	7	2	3	3	0	4	1	2	5	3	2	3	3	2	2	3	3
1.3.1.-	0	0	0	0	0	0	0	0	0	0	0	1	0	0	0	0	1	0	0
1.3.1.2	0	3	1	0	0	0	0	2	1	0	2	1	2	0	0	1	0	1	1
1.3.1.25	0	0	0	0	0	0	0	0	0	0	0	0	0	0	0	0	0	0	0
1.3.99.-	0	0	0	0	0	0	0	0	0	0	0	0	0	0	0	1	0	0	0
1.6.5.-	10	48	64	39	33	40	21	34	51	40	49	42	56	54	45	35	38	48	31
1.7.1.-	0	8	3	8	8	11	6	11	12	6	6	5	12	6	10	7	8	14	9
1.8.99.3	0	1	1	0	1	0	0	0	0	0	0	0	0	0	1	0	1	0	0
2.1.1.-	37	299	242	234	162	335	107	295	269	237	261	289	369	208	257	275	313	283	301
2.3.1.-	34	207	103	97	90	150	54	168	167	152	158	186	223	111	157	138	192	175	170
2.3.1.16	0	2	0	0	0	3	1	2	1	2	0	1	0	0	0	0	0	0	3
2.3.1.5	0	0	0	0	0	0	0	0	0	0	0	0	0	0	0	0	0	0	0
2.3.1.9	0	0	1	1	0	0	0	1	0	1	0	1	2	0	1	0	0	1	1
2.4.2.10	1	17	20	15	8	16	5	12	15	10	12	16	20	12	12	16	15	20	13
2.4.2.3	2	10	4	11	8	8	4	10	9	6	7	12	12	6	10	10	12	15	12
2.4.2.4	0	7	2	7	2	8	2	10	5	2	6	4	9	3	2	4	5	5	5
2.4.2.8	0	16	12	14	9	16	6	15	13	7	13	12	15	8	17	14	16	15	16
2.5.1.-	4	25	24	17	12	28	11	26	23	17	21	26	30	24	29	13	21	30	20

EC/KO	DA-AD-28	DA-AD-29	DA-AD-3	DA-AD-30	DA-AD-31	DA-AD-32	DA-AD-33	DA-AD-34	DA-AD-35	DA-AD-36	DA-AD-37	DA-AD-38	DA-AD-39	DA-AD-4	DA-AD-40	DA-AD-41	DA-AD-42	DA-AD-43	DA-AD-44
**2.5.1.18**	0	4	1	0	4	0	0	2	0	2	2	3	0	0	3	1	0	3	2
**2.6.1.-**	6	42	15	30	25	49	13	49	47	35	39	52	44	20	34	29	35	40	41
**2.7.1.21**	0	13	9	6	2	7	2	12	9	7	9	7	11	3	9	9	8	9	7
**2.7.1.48**	4	37	25	34	20	39	6	37	34	29	38	39	43	19	29	27	32	40	24
**2.7.4.-**	2	17	9	16	13	19	8	25	18	10	18	13	25	9	19	22	21	9	19
**2.8.3.-**	0	1	0	1	1	1	2	1	1	0	3	2	2	0	0	0	0	1	2
**2.8.3.1**	0	0	1	1	0	0	0	0	0	0	0	1	0	0	0	0	0	0	2
**2.8.3.12**	1	1	2	0	0	0	0	0	1	0	0	2	2	2	1	0	0	0	0
**2.8.3.6**	0	2	2	0	2	0	0	0	0	0	0	0	1	0	0	0	0	0	0
**2.8.3.8**	0	0	4	0	2	0	0	1	1	1	1	1	1	2	0	2	3	1	1
**3.1.1.-**	1	9	2	2	5	6	3	4	5	6	3	8	3	7	3	5	4	4	4
**3.1.1.1**	0	8	6	2	2	6	1	8	7	5	7	6	9	4	10	3	7	4	9
**3.1.1.17**	0	3	0	0	0	2	0	3	0	2	4	3	4	0	1	1	1	2	1
**3.1.1.2**	0	1	0	0	1	0	0	0	1	0	1	0	0	0	0	0	0	1	0
**3.1.1.24**	0	3	3	6	0	4	0	2	2	2	3	4	4	1	2	4	3	2	2
**3.1.1.45**	0	1	0	0	0	0	0	0	0	0	0	0	0	0	0	0	0	0	0
**3.1.2.-**	0	0	1	0	0	0	0	0	1	0	0	0	2	1	1	1	0	0	0
**3.1.2.23**	1	8	2	1	2	4	2	5	5	4	6	5	9	0	4	5	7	4	3
**3.1.3.1**	2	17	5	9	8	11	6	16	17	15	19	15	20	9	17	11	16	14	8
**3.1.3.2**	1	5	1	2	3	4	3	3	4	6	7	6	5	3	5	5	2	2	2
**3.1.3.41**	0	0	0	0	0	0	0	0	0	0	0	0	0	0	1	0	0	0	0
**3.2.1.31**	2	0	2	2	3	6	3	7	1	2	1	7	10	3	9	3	4	11	4
**3.3.2.9**	0	0	0	0	0	0	0	0	0	0	0	0	0	0	0	0	0	0	0
**3.5.1.-**	5	33	7	28	19	41	15	38	29	16	17	30	33	22	27	28	30	27	31
**3.5.1.4**	1	2	3	1	1	6	1	1	3	3	4	3	2	3	3	3	3	5	4
**3.5.1.5**	2	2	0	2	2	2	0	2	1	0	1	1	3	0	3	2	2	1	1
**3.5.1.54**	0	6	6	5	1	3	0	2	4	2	1	2	6	5	3	2	3	0	3
**3.5.1.6**	0	3	3	2	1	1	0	0	1	0	0	0	1	1	0	2	2	0	2
**3.5.2.-**	0	0	0	0	0	0	0	0	0	0	0	0	0	0	0	0	0	0	0
**3.5.2.2**	0	0	0	1	0	0	0	0	1	1	1	1	3	1	1	2	3	1	3
**3.5.4.-**	7	35	23	27	15	31	16	32	21	22	28	25	40	29	23	25	25	25	28
**3.5.4.5**	0	15	13	17	8	23	5	18	16	12	15	19	21	10	8	16	13	19	15
**3.5.5.1**	1	1	0	3	0	1	1	0	0	1	4	1	1	0	2	1	2	0	1
**3.5.5.7**	0	0	0	0	1	0	0	0	0	0	0	0	0	0	0	0	0	0	0
**3.5.99.3**	0	0	0	0	0	0	0	0	0	0	1	0	0	0	0	0	0	0	0
**3.6.1.7**	1	3	5	3	2	5	2	6	3	5	5	5	5	5	8	3	4	4	7
**3.7.1.-**	0	4	1	0	1	0	0	0	0	0	0	0	2	2	3	2	0	0	2
**3.7.1.2**	1	3	2	1	2	4	1	5	5	1	5	4	6	1	5	2	3	4	3
**3.8.1.2**	1	11	0	2	0	9	1	3	4	5	8	8	7	2	7	3	3	6	7
**3.8.1.3**	0	0	0	0	0	0	0	0	0	0	0	0	0	0	0	0	0	0	0
**3.8.1.5**	0	0	2	0	1	0	0	0	0	0	2	1	1	0	0	1	1	0	0
**4.1.1.-**	3	14	10	8	8	14	4	9	10	7	12	9	11	3	12	9	9	9	10
**4.1.1.44**	6	28	30	14	8	20	8	23	24	22	22	25	36	33	25	27	25	25	24
**4.1.1.55**	0	0	0	0	0	0	0	0	0	0	0	0	0	0	0	0	0	0	0
**4.1.1.7**	0	0	0	0	0	0	0	0	0	0	0	0	0	0	0	0	0	0	0
**4.1.1.70**	0	0	0	0	0	0	0	0	0	0	0	0	0	0	1	0	0	0	0
**4.1.1.77**	0	0	1	0	0	0	0	0	0	0	0	0	0	1	0	0	0	0	0
**4.1.2.-**	1	5	5	3	4	1	2	5	2	6	9	3	4	2	3	6	8	2	6
**4.1.3.-**	2	18	17	16	12	26	7	25	22	18	14	22	27	16	24	17	19	27	24

EC/KO	DA-AD-28	DA-AD-29	DA-AD-3	DA-AD-30	DA-AD-31	DA-AD-32	DA-AD-33	DA-AD-34	DA-AD-35	DA-AD-36	DA-AD-37	DA-AD-38	DA-AD-39	DA-AD-4	DA-AD-40	DA-AD-41	DA-AD-42	DA-AD-43	DA-AD-44
4.1.3.39	0	0	0	0	0	0	1	1	1	2	0	0	0	1	2	0	0	0	1
4.1.99.-	1	1	0	1	1	8	3	3	3	1	1	1	5	1	3	2	2	5	3
4.2.1.-	6	65	39	36	34	51	16	58	55	42	50	60	61	40	45	40	45	55	53
4.2.1.17	1	10	6	3	2	7	1	7	10	3	1	2	12	5	4	3	6	5	7
4.2.1.80	0	0	0	0	0	0	0	0	0	0	0	0	0	0	0	0	0	0	1
4.2.1.83	0	1	0	0	1	3	0	1	1	0	2	0	2	0	0	0	0	4	1
4.2.1.84	0	0	0	1	2	2	1	0	1	0	0	0	0	0	0	0	0	1	1
5.1.2.2	0	0	0	0	0	0	0	0	0	0	0	0	0	0	0	0	0	0	0
5.2.1.2	0	0	1	0	0	0	0	0	0	0	0	0	0	0	0	0	0	0	0
5.3.3.4	0	0	0	0	0	0	0	0	0	0	0	0	0	0	0	1	0	0	0
5.3.99.-	0	4	7	2	1	6	0	4	3	1	1	6	5	4	3	3	3	4	2
5.4.99.-	1	0	1	0	1	1	0	0	0	0	0	0	1	1	1	0	0	1	0
5.5.1.1	1	5	1	4	0	4	0	1	2	5	4	5	5	1	3	3	2	4	2
5.5.1.2	0	0	0	0	0	0	0	0	0	0	0	0	0	0	0	0	0	0	0
6.2.1.-	0	0	0	0	0	0	0	0	0	0	0	0	0	0	0	0	0	0	0
6.3.5.2	2	14	16	9	9	22	5	16	12	14	23	17	19	12	17	15	18	19	14
K00002	0	3	1	1	0	3	0	3	1	1	3	0	4	1	1	2	3	1	1
K00055	0	0	0	0	0	0	0	0	0	0	0	0	1	0	0	0	0	0	0
K00074	0	2	8	5	1	3	0	5	7	4	5	5	0	9	3	3	5	4	4
K00088	1	20	9	14	11	16	9	17	17	10	17	19	19	9	13	16	13	15	12
K00100	5	67	43	31	26	48	15	54	51	46	50	57	57	17	46	39	49	47	53
K00128	0	5	0	1	1	3	1	4	3	2	3	3	2	0	2	4	2	3	2
K00129	0	0	0	0	0	0	0	0	0	0	0	0	0	0	0	0	0	0	0
K00132	0	0	1	0	0	0	0	0	0	0	0	0	1	0	0	1	0	0	1
K00141	0	0	0	0	0	0	0	0	0	0	0	0	0	0	0	0	0	0	0
K00146	0	0	0	0	0	0	0	0	0	0	0	0	0	0	0	0	0	0	0
K00148	0	0	0	0	0	0	0	0	0	0	0	0	0	0	0	0	0	0	0
K00155	0	2	0	0	0	0	0	0	0	0	0	0	0	0	0	0	0	0	0
K00169	0	2	1	0	0	3	2	4	0	1	2	2	0	2	1	0	3	4	4
K00224	0	1	2	0	0	0	0	1	0	0	0	1	0	0	0	0	0	1	1
K00274	0	0	0	0	0	0	0	0	0	0	0	0	0	0	0	0	0	0	0
K00446	0	0	0	0	0	0	0	0	0	0	0	0	0	0	0	0	0	0	0
K00448	0	1	0	0	0	0	0	0	0	0	0	0	0	0	0	0	0	0	0
K00462	0	1	0	0	1	1	0	0	1	2	0	0	1	1	1	1	0	1	2
K00480	0	0	0	0	0	0	0	0	0	0	0	0	0	0	0	0	0	0	0
K00481	0	1	0	0	0	0	0	0	0	0	0	0	0	0	0	0	0	0	0
K00539	0	2	2	0	0	1	1	1	0	1	1	1	2	0	0	1	1	2	1
K00599	3	55	24	32	31	51	22	53	48	39	32	57	53	26	48	36	43	70	62
K00626	0	4	2	3	0	3	1	2	1	4	0	2	3	0	2	1	1	2	3
K00632	0	0	0	0	0	0	0	0	0	0	0	0	0	0	0	0	0	0	0
K00680	6	46	28	38	27	33	11	49	51	30	35	44	69	27	37	43	46	41	44
K00757	5	16	12	10	5	7	5	9	8	9	10	12	15	6	15	15	10	15	10
K00758	0	8	2	2	2	4	2	9	3	1	4	4	11	5	1	6	4	4	4
K00760	2	23	24	20	15	22	8	23	24	18	19	17	33	20	24	30	29	24	21
K00799	0	3	1	0	0	0	1	0	0	0	1	1	1	1	0	0	0	0	0
K00857	3	18	18	18	4	10	2	15	14	12	14	11	20	7	14	12	17	12	11
K00876	3	32	25	34	16	35	8	26	22	18	33	33	36	15	24	26	29	36	22
K01026	0	0	3	0	0	0	0	0	1	0	0	0	0	0	1	1	2	0	0
K01031	0	0	0	0	0	0	0	0	0	0	0	0	0	0	0	0	0	0	0

EC/KO	DA-AD-28	DA-AD-29	DA-AD-3	DA-AD-30	DA-AD-31	DA-AD-32	DA-AD-33	DA-AD-34	DA-AD-35	DA-AD-36	DA-AD-37	DA-AD-38	DA-AD-39	DA-AD-4	DA-AD-40	DA-AD-41	DA-AD-42	DA-AD-43	DA-AD-44
K01034	1	3	4	1	0	0	1	1	1	3	1	1	2	2	0	1	2	2	1
K01039	0	2	3	0	0	0	0	0	0	0	0	0	0	2	1	0	0	0	0
K01041	0	4	1	3	2	4	2	6	7	7	5	2	4	1	4	3	2	4	2
K01053	0	0	0	0	0	1	0	0	0	0	0	0	1	0	0	0	0	0	0
K01055	0	0	0	0	0	0	0	0	0	0	0	0	0	0	0	0	0	0	0
K01061	0	2	0	0	0	0	0	0	0	0	0	0	0	0	0	0	0	0	0
K01066	0	12	7	4	2	6	0	8	7	9	8	6	11	1	9	6	8	6	7
K01075	0	2	1	0	1	0	0	2	0	0	1	0	2	1	1	1	1	0	1
K01077	1	10	2	1	4	6	2	7	9	9	9	8	15	2	8	4	5	8	5
K01101	2	2	3	8	0	2	1	2	5	6	4	5	8	3	1	4	3	3	2
K01195	1	5	5	6	4	8	2	14	9	11	8	12	13	5	11	6	10	13	11
K01426	0	0	0	1	1	0	0	1	0	0	0	0	1	0	0	0	0	1	0
K01428	0	1	0	1	1	0	0	0	0	0	0	1	0	0	2	0	1	1	1
K01457	0	0	0	0	0	0	0	0	0	0	0	0	0	0	0	0	0	0	1
K01464	0	3	1	2	0	1	0	0	1	4	2	1	4	2	2	1	4	1	3
K01489	2	23	19	21	11	27	9	23	23	16	19	23	29	11	16	24	20	20	21
K01500	0	0	0	0	1	0	0	0	0	0	1	0	0	0	0	0	0	0	0
K01501	1	3	1	3	0	1	1	0	0	2	5	2	6	0	2	1	4	0	2
K01502	0	0	0	0	1	0	0	0	0	0	0	0	0	0	0	0	0	0	0
K01512	3	6	7	6	3	9	2	11	8	7	6	7	7	8	10	6	7	7	11
K01560	3	6	9	7	1	3	0	2	2	7	2	7	7	2	7	5	6	6	4
K01561	0	1	0	0	0	0	0	1	0	0	0	0	0	0	0	0	0	0	0
K01563	0	0	0	0	0	0	0	0	0	0	0	0	0	0	0	0	0	0	0
K01564	0	3	2	0	1	1	0	1	1	1	0	3	0	0	3	1	1	2	1
K01607	2	19	12	5	4	9	7	9	10	11	12	10	21	19	8	11	13	9	8
K01612	0	0	0	0	0	0	0	0	0	0	0	0	0	0	0	0	0	0	0
K01615	0	13	11	10	10	10	5	17	14	12	14	11	20	7	12	8	15	18	9
K01617	0	0	0	0	0	0	0	0	0	0	0	0	0	1	0	0	0	0	0
K01666	0	5	2	5	4	12	4	10	7	7	2	8	9	3	10	7	5	9	10
K01692	0	1	0	0	0	0	0	1	0	0	1	0	1	0	0	1	1	0	0
K01721	0	0	0	0	0	0	0	0	0	0	0	0	0	0	0	0	0	0	0
K01726	3	5	2	5	2	4	3	3	6	7	7	8	6	4	7	5	4	4	2
K01781	0	0	0	0	0	0	0	0	0	0	0	0	0	0	1	0	0	0	0
K01821	0	3	3	0	0	1	1	0	1	1	1	1	0	2	0	2	0	0	2
K01856	0	0	0	0	0	0	0	0	0	0	0	0	0	0	0	0	0	0	0
K01857	0	0	0	0	1	0	0	0	0	0	0	0	0	0	0	0	0	0	0
K01913	0	0	0	0	0	0	0	1	0	0	0	0	0	0	0	1	1	0	1
K01951	0	13	17	9	5	13	4	11	18	12	18	11	19	11	14	8	16	15	9
K02554	0	0	1	0	0	0	0	0	0	0	0	0	0	0	0	0	0	0	0
K03381	0	0	0	0	0	0	0	0	0	0	0	0	0	0	0	0	0	0	0
K03382	0	0	1	0	0	1	0	0	0	1	2	1	0	0	1	0	0	0	0
K03464	0	0	0	0	0	0	0	0	0	0	0	0	0	0	0	0	0	0	0
K03518	4	6	30	3	4	8	1	6	7	13	8	13	8	18	14	11	8	10	14
K03862	0	0	0	0	0	0	0	0	0	0	0	0	0	0	0	0	0	0	0
K04099	0	1	0	0	0	0	0	0	0	0	0	0	0	0	0	0	0	0	0
K04100	0	0	0	0	0	0	0	0	0	0	0	0	0	0	0	0	0	0	0
K04102	0	0	0	0	0	0	0	0	0	0	0	0	0	0	0	0	0	0	0
K04116	0	0	0	0	0	0	0	0	0	0	0	0	0	0	0	0	0	0	0
K05394	0	1	1	1	0	0	0	0	1	1	0	0	2	0	0	1	0	0	1

EC/KO	DA-AD-28	DA-AD-29	DA-AD-3	DA-AD-30	DA-AD-31	DA-AD-32	DA-AD-33	DA-AD-34	DA-AD-35	DA-AD-36	DA-AD-37	DA-AD-38	DA-AD-39	DA-AD-4	DA-AD-40	DA-AD-41	DA-AD-42	DA-AD-43	DA-AD-44
K05549	0	1	0	0	0	0	0	0	0	0	0	0	0	0	0	0	0	0	0
K05783	0	0	0	0	0	0	0	0	0	0	0	0	0	0	0	0	0	0	0
K05797	0	0	0	0	0	0	0	0	0	0	0	0	0	0	0	0	0	0	0
K06281	0	2	1	0	1	0	0	1	0	1	1	1	0	0	0	1	1	0	1
K06446	0	2	11	0	1	2	1	1	1	2	1	2	3	19	1	4	3	0	4
K06912	0	0	0	0	0	0	0	0	0	0	0	0	0	0	0	0	0	0	0
K07535	0	0	1	0	0	0	0	0	0	0	0	0	0	0	1	1	0	0	0
K07536	0	3	1	3	1	1	1	2	3	1	2	0	1	1	0	0	2	2	4
K08689	0	0	0	0	0	0	0	0	0	0	0	0	0	0	0	0	0	0	0
K08710	0	0	0	0	0	0	0	0	0	0	0	0	0	0	0	0	0	0	0
K09461	0	0	1	0	0	0	0	1	0	0	0	0	0	0	1	1	0	0	0
K10217	0	0	0	0	0	0	0	0	0	0	0	0	0	0	0	0	0	0	0
K10218	0	1	1	0	1	0	0	0	0	0	1	0	0	0	0	0	0	0	0
K10220	0	0	0	0	0	0	0	0	0	0	0	0	0	0	0	0	0	0	0
K11180	0	0	0	0	0	0	0	0	0	0	0	0	0	1	0	0	1	0	0
K13953	0	0	0	0	0	0	0	0	0	0	0	0	1	0	0	0	0	0	0
K14333	0	1	0	0	0	0	0	0	0	0	0	0	0	0	0	0	0	0	0
K14519	0	0	0	0	0	0	0	0	0	0	0	0	0	0	0	0	0	0	0
K15054	0	0	0	0	0	0	0	0	0	0	0	0	0	0	0	0	0	0	0
K16173	0	1	0	0	0	0	0	0	0	0	0	0	1	0	0	0	0	0	0
K16514	0	0	0	0	0	0	0	0	0	0	0	0	0	0	0	0	0	0	0
K16874	0	0	1	0	0	0	0	0	0	0	0	0	0	0	0	0	0	0	0

EC/KO	DA-AD-45	DA-AD-46	DA-AD-47	DA-AD-48	DA-AD-49	DA-AD-5	DA-AD-50	DA-AD-51	DA-AD-52	DA-AD-53	DA-AD-54	DA-AD-55	DA-AD-56	DA-AD-57	DA-AD-58	DA-AD-59	DA-AD-6	DA-AD-60	DA-AD-61
**1.1.1.-**	35	26	19	27	46	8	46	35	39	58	52	52	48	71	71	44	44	35	62
**1.1.1.1**	27	18	15	14	40	9	24	20	33	33	33	32	20	30	37	35	28	24	42
**1.1.1.157**	0	0	1	0	2	0	0	1	1	2	1	0	0	0	0	2	1	1	1
**1.1.1.205**	16	7	9	11	23	5	10	12	21	24	25	25	14	30	24	21	14	13	22
**1.1.1.35**	3	1	3	0	3	0	1	2	5	5	5	3	1	2	6	4	3	3	3
**1.12.99.6**	0	0	0	0	0	0	0	0	1	2	0	0	3	3	1	2	1	1	1
**1.13.11.-**	0	0	0	0	0	0	0	0	0	0	0	0	0	0	0	0	0	0	0
**1.13.11.1**	0	0	0	0	0	0	0	0	0	0	0	0	0	0	0	0	0	0	0
**1.13.11.2**	0	0	0	0	0	0	0	0	0	0	0	0	0	0	0	0	0	0	0
**1.13.11.3**	0	0	0	0	0	0	0	0	0	0	0	0	0	0	0	0	0	0	0
**1.13.11.39**	0	0	0	0	0	0	0	0	0	0	0	0	0	0	0	0	0	0	0
**1.13.11.5**	0	0	0	0	0	0	0	0	0	0	0	0	0	0	0	0	0	0	0
**1.13.11.8**	0	0	0	0	0	0	0	0	0	0	0	0	0	0	0	0	0	0	0
**1.14.12.10**	0	0	0	0	0	0	0	1	0	0	0	0	0	0	0	0	0	0	0
**1.14.12.13**	0	0	0	0	0	0	0	0	0	0	0	0	0	0	0	0	0	0	0
**1.14.12.18**	0	0	0	0	0	0	0	0	0	0	0	0	0	0	0	0	0	0	0
**1.14.13.-**	0	0	0	0	0	0	0	0	0	0	0	0	0	0	0	0	0	0	0
**1.14.13.1**	0	0	0	0	0	0	0	0	0	0	0	0	0	0	0	0	0	0	0
**1.14.13.2**	0	0	0	0	0	0	0	0	0	0	0	0	0	0	0	0	0	0	0
**1.14.13.50**	0	0	0	0	0	0	0	0	0	0	0	0	0	0	0	0	0	0	0
**1.14.13.7**	0	2	0	0	1	0	0	0	1	0	0	1	0	1	2	0	0	0	0
**1.14.13.8**	0	0	0	0	0	0	0	0	0	0	0	0	0	0	0	0	0	0	0
**1.14.13.82**	0	0	0	0	0	0	0	0	0	0	0	0	0	0	0	0	0	0	0
**1.14.99.-**	0	0	0	0	0	0	0	0	0	0	0	0	0	0	0	0	0	0	0
**1.17.99.1**	0	0	0	0	0	0	0	0	0	0	0	0	0	0	0	0	0	0	0
**1.18.6.1**	1	0	0	0	0	0	0	0	0	1	0	0	0	0	0	0	0	0	0
**1.2.1.-**	0	0	0	0	0	0	0	0	0	0	0	0	0	0	0	0	0	0	0
**1.2.1.10**	2	3	2	2	3	0	3	2	6	7	8	4	4	3	6	8	7	4	3
**1.2.1.3**	3	4	2	4	4	2	2	3	7	6	8	5	8	8	13	4	5	8	9
**1.2.1.39**	0	0	0	0	0	0	0	0	0	0	0	0	0	0	0	0	0	0	0
**1.2.7.1**	9	3	6	8	14	1	6	8	15	13	14	14	11	15	13	9	14	7	14
**1.2.99.2**	0	3	1	2	1	0	3	2	2	7	5	6	1	4	5	2	4	2	5
**1.3.1.-**	0	0	0	0	0	0	1	0	0	0	0	0	1	0	0	0	0	0	0
**1.3.1.2**	0	0	1	0	1	0	1	1	2	2	2	1	1	0	1	0	2	0	1
**1.3.1.25**	0	0	0	0	0	0	0	0	0	0	0	0	0	0	0	0	0	0	0
**1.3.99.-**	0	0	0	0	0	0	0	0	0	2	0	0	0	0	0	0	0	0	0
**1.6.5.-**	55	29	25	24	66	8	28	46	36	44	64	51	45	48	53	52	42	33	43
**1.7.1.-**	11	4	4	6	10	3	10	13	8	7	11	10	10	11	11	8	4	6	7
**1.8.99.3**	0	0	0	0	0	0	0	0	1	0	1	0	0	0	0	0	1	0	0
**2.1.1.-**	223	118	166	123	341	51	168	206	280	325	318	268	188	335	374	295	276	211	307
**2.3.1.-**	138	85	101	74	171	27	122	116	129	168	184	168	133	219	221	141	143	152	144
**2.3.1.16**	0	0	1	0	1	1	0	0	1	1	1	2	0	1	1	4	2	1	2
**2.3.1.5**	0	0	0	0	0	0	0	0	0	0	0	0	0	0	0	0	0	0	0
**2.3.1.9**	0	0	0	0	0	1	0	0	0	1	0	0	0	1	0	0	1	0	1
**2.4.2.10**	12	8	12	6	14	3	9	13	20	14	21	20	15	17	19	18	15	7	16
**2.4.2.3**	4	6	11	3	15	2	5	6	7	10	16	12	4	10	16	10	9	11	12
**2.4.2.4**	2	5	3	1	10	0	3	3	10	6	5	2	1	8	7	6	5	2	4
**2.4.2.8**	17	5	12	8	14	6	11	9	15	16	17	16	6	22	20	16	10	13	14
**2.5.1.-**	30	13	11	14	24	4	14	18	27	21	28	29	23	28	36	21	17	14	27

EC/KO	DA-AD-45	DA-AD-46	DA-AD-47	DA-AD-48	DA-AD-49	DA-AD-5	DA-AD-50	DA-AD-51	DA-AD-52	DA-AD-53	DA-AD-54	DA-AD-55	DA-AD-56	DA-AD-57	DA-AD-58	DA-AD-59	DA-AD-6	DA-AD-60	DA-AD-61
2.5.1.18	0	0	0	1	2	0	1	0	1	0	0	0	2	0	1	1	0	1	6
2.6.1.-	37	9	23	19	34	11	26	25	41	43	42	31	33	35	53	41	27	25	32
2.7.1.21	10	4	10	3	10	1	5	6	8	9	10	16	7	10	9	7	4	5	10
2.7.1.48	30	18	16	11	42	3	17	23	27	34	47	37	28	43	44	39	26	16	26
2.7.4.-	14	11	13	10	22	2	11	14	18	19	15	15	15	23	20	20	19	11	17
2.8.3.-	2	0	1	1	2	1	1	0	0	2	1	2	2	3	2	1	1	0	0
2.8.3.1	0	0	0	0	0	0	0	0	1	0	0	0	0	0	0	0	0	0	0
2.8.3.12	1	0	0	1	0	0	0	0	0	0	1	0	2	1	0	0	1	0	0
2.8.3.6	0	0	0	0	1	0	0	0	0	0	0	1	0	0	1	1	0	0	0
2.8.3.8	1	0	1	0	3	0	0	2	2	1	1	4	0	1	0	2	2	0	4
3.1.1.-	6	4	3	2	5	1	3	4	2	5	4	3	1	10	9	4	6	7	4
3.1.1.1	9	3	2	4	9	0	5	0	3	6	10	3	5	12	5	7	7	2	6
3.1.1.17	1	0	1	2	1	0	3	1	0	0	1	3	2	4	2	2	1	0	0
3.1.1.2	0	0	0	0	0	0	0	0	0	0	1	0	0	0	1	0	0	0	0
3.1.1.24	0	0	2	1	3	2	4	3	2	3	3	2	2	5	7	2	4	4	2
3.1.1.45	0	0	0	0	0	0	0	0	0	0	0	0	0	0	0	0	0	0	0
3.1.2.-	0	0	0	0	0	0	0	0	0	0	0	0	0	0	0	0	0	0	0
3.1.2.23	4	1	4	2	7	1	5	9	2	4	7	9	4	6	7	6	5	5	5
3.1.3.1	21	8	13	12	19	2	14	11	3	13	20	16	22	22	17	9	9	11	9
3.1.3.2	5	2	3	4	5	0	3	7	2	1	7	3	7	6	3	4	3	1	4
3.1.3.41	0	0	0	0	0	0	0	0	0	0	0	0	0	0	0	0	0	0	0
3.2.1.31	10	1	1	3	5	0	1	2	3	9	3	7	5	5	4	5	2	2	2
3.3.2.9	0	0	0	0	0	0	0	0	0	0	0	0	0	0	0	0	0	0	0
3.5.1.-	24	16	16	15	39	6	21	33	26	25	40	29	17	31	41	37	26	22	24
3.5.1.4	1	2	1	1	5	1	1	1	3	1	3	4	3	3	2	2	3	3	1
3.5.1.5	0	0	0	0	1	0	0	0	1	0	0	1	0	2	0	2	2	1	2
3.5.1.54	1	1	2	0	6	0	2	1	2	3	4	3	3	7	4	3	3	1	4
3.5.1.6	0	0	1	0	1	1	1	0	1	1	2	2	1	2	3	3	2	0	2
3.5.2.-	0	0	0	0	0	0	0	0	0	0	0	0	0	0	0	0	0	0	0
3.5.2.2	1	0	0	0	2	0	1	1	0	2	2	1	1	2	1	2	2	2	2
3.5.4.-	26	21	16	9	38	6	14	31	28	25	32	26	19	31	42	30	29	17	27
3.5.4.5	15	10	7	5	15	1	12	14	18	15	17	12	9	19	19	14	15	8	11
3.5.5.1	1	1	1	1	2	0	0	1	3	2	1	2	2	1	5	1	1	2	0
3.5.5.7	0	0	0	0	0	0	0	0	1	0	0	0	0	0	0	0	0	0	0
3.5.99.3	0	0	0	0	0	0	0	0	0	1	0	0	0	0	0	0	0	0	0
3.6.1.7	2	2	1	2	4	0	4	0	4	2	5	7	2	3	5	6	5	4	6
3.7.1.-	0	2	1	0	3	0	0	0	4	1	2	0	0	0	1	3	2	0	4
3.7.1.2	3	5	3	1	4	0	3	2	0	3	3	6	6	6	12	3	4	4	8
3.8.1.2	5	3	6	4	6	1	5	2	2	6	7	6	8	4	10	5	2	3	4
3.8.1.3	0	0	0	0	0	0	0	0	0	0	0	0	0	0	0	0	0	0	0
3.8.1.5	0	0	3	0	0	0	1	0	0	1	0	0	1	0	0	0	1	0	0
4.1.1.-	11	4	5	4	13	2	5	6	7	11	21	11	5	15	11	9	10	6	13
4.1.1.44	24	24	20	14	33	4	23	17	19	23	32	26	20	32	39	22	29	16	15
4.1.1.55	0	0	0	0	0	0	0	0	0	0	0	0	0	0	0	0	1	0	0
4.1.1.7	0	0	0	0	0	0	0	0	0	0	0	0	0	0	0	0	0	0	0
4.1.1.70	0	0	0	0	0	0	0	0	0	0	0	0	1	0	0	0	0	1	0
4.1.1.77	0	0	0	0	0	0	0	0	0	0	0	0	0	0	0	0	0	0	0
4.1.2.-	3	2	5	0	4	3	4	5	9	9	5	6	4	8	7	7	7	2	6
4.1.3.-	23	5	10	12	27	5	14	16	20	21	27	15	12	29	27	21	17	14	21

EC/KO	DA-AD-45	DA-AD-46	DA-AD-47	DA-AD-48	DA-AD-49	DA-AD-5	DA-AD-50	DA-AD-51	DA-AD-52	DA-AD-53	DA-AD-54	DA-AD-55	DA-AD-56	DA-AD-57	DA-AD-58	DA-AD-59	DA-AD-6	DA-AD-60	DA-AD-61
4.1.3.39	0	0	0	1	1	0	0	0	0	0	0	0	1	2	0	1	0	0	0
4.1.99.-	1	1	2	1	1	1	1	2	2	3	4	0	1	5	5	1	3	1	1
4.2.1.-	47	22	28	21	50	6	33	42	44	46	51	48	40	61	74	48	41	39	54
4.2.1.17	5	1	4	4	7	1	4	3	4	5	6	8	6	8	7	2	3	4	6
4.2.1.80	0	0	0	0	0	0	0	0	0	0	0	0	0	0	0	0	0	0	0
4.2.1.83	1	0	0	2	1	0	1	0	1	0	0	0	0	2	2	1	0	0	0
4.2.1.84	0	0	0	0	0	0	0	0	0	0	1	0	0	1	0	0	0	0	0
5.1.2.2	0	0	0	0	0	0	0	0	0	1	0	0	0	0	0	0	0	0	0
5.2.1.2	0	0	0	0	0	0	0	0	0	0	0	0	0	0	0	0	0	0	0
5.3.3.4	0	0	0	0	0	0	0	0	0	0	0	0	0	0	0	0	0	0	0
5.3.99.-	3	1	1	1	5	0	1	1	6	4	3	2	3	4	5	0	0	0	7
5.4.99.-	2	1	0	1	0	1	0	1	0	0	1	2	0	1	0	1	1	0	1
5.5.1.1	2	0	2	2	3	0	2	1	3	3	1	2	3	3	4	4	1	3	2
5.5.1.2	0	0	0	0	0	0	0	0	0	0	0	0	0	0	0	0	0	0	0
6.2.1.-	0	0	0	0	0	0	0	0	0	0	0	0	0	0	0	0	0	0	0
6.3.5.2	20	3	9	11	18	3	13	10	10	20	15	17	21	22	25	21	14	16	17
K00002	0	2	0	1	0	0	1	2	2	2	1	1	2	1	1	2	0	4	1
K00055	0	0	0	0	0	0	0	0	0	0	0	1	0	0	0	0	0	0	0
K00074	3	2	4	1	5	0	1	3	6	8	9	3	5	3	6	8	3	7	3
K00088	13	4	6	9	20	3	7	7	20	24	16	17	11	15	21	17	17	14	15
K00100	51	27	18	15	58	9	25	36	45	63	54	51	46	59	65	48	41	32	67
K00128	1	1	0	1	0	0	1	1	3	3	2	0	2	1	4	0	0	2	4
K00129	0	0	0	0	0	0	0	0	0	0	0	0	0	0	0	0	0	0	0
K00132	0	1	1	0	0	0	0	0	0	1	0	1	0	0	1	1	1	0	0
K00141	0	0	0	0	0	0	0	0	0	0	0	0	0	0	0	0	0	0	0
K00146	0	0	0	0	0	0	0	0	0	0	0	0	0	0	0	0	0	0	0
K00148	0	0	0	0	0	0	0	0	0	0	0	0	0	0	0	0	0	0	0
K00155	0	0	0	0	0	0	0	0	0	1	1	0	0	1	0	0	0	0	0
K00169	1	1	1	0	4	0	0	0	3	0	5	0	2	6	3	2	2	1	4
K00224	0	0	0	0	1	0	0	0	0	1	1	0	1	1	1	2	1	0	1
K00274	0	0	0	0	0	0	0	0	0	0	0	0	0	0	0	0	0	0	0
K00446	0	0	0	0	0	0	0	0	0	0	0	0	0	0	0	0	0	0	0
K00448	0	0	0	0	0	0	0	0	0	0	0	0	0	0	0	0	0	0	0
K00462	0	0	0	1	1	0	1	0	0	0	1	0	2	1	1	1	0	2	1
K00480	0	0	0	0	0	0	0	0	0	0	0	0	0	0	0	0	0	0	0
K00481	0	0	0	0	0	0	0	0	0	0	0	0	0	0	0	0	0	0	0
K00539	0	1	0	0	0	0	0	1	0	1	0	0	1	2	3	0	3	1	1
K00599	40	15	27	22	46	16	24	34	33	40	65	42	30	66	65	32	32	32	58
K00626	0	0	2	0	4	1	0	1	2	3	2	4	3	2	1	4	4	1	3
K00632	0	0	0	0	0	0	0	0	0	0	0	0	0	0	0	0	0	0	0
K00680	35	33	28	17	46	10	31	26	34	51	58	51	31	50	64	39	37	37	43
K00757	12	4	6	7	15	3	2	11	8	11	15	12	14	9	18	9	8	8	12
K00758	1	1	3	1	5	0	1	4	5	2	7	0	0	5	5	7	4	6	3
K00760	23	10	19	11	28	6	20	23	25	32	24	23	16	31	29	25	22	22	22
K00799	0	0	0	0	2	0	0	1	0	0	0	0	0	1	1	0	2	0	0
K00857	12	6	11	3	21	2	7	10	16	17	13	17	11	18	14	12	9	11	13
K00876	28	16	17	13	42	6	13	23	28	30	40	32	25	32	39	39	23	15	20
K01026	1	0	0	0	1	0	0	0	1	2	1	2	0	0	0	0	1	0	1
K01031	0	0	0	0	0	0	0	0	0	0	0	0	0	0	0	0	0	0	0

EC/KO	DA-AD-45	DA-AD-46	DA-AD-47	DA-AD-48	DA-AD-49	DA-AD-5	DA-AD-50	DA-AD-51	DA-AD-52	DA-AD-53	DA-AD-54	DA-AD-55	DA-AD-56	DA-AD-57	DA-AD-58	DA-AD-59	DA-AD-6	DA-AD-60	DA-AD-61
K01034	1	1	2	0	2	0	0	1	2	0	3	2	1	1	0	1	1	2	0
K01039	0	0	0	0	0	0	0	0	0	0	0	1	0	0	0	0	1	0	0
K01041	8	0	0	4	4	1	3	5	3	5	5	8	5	6	2	2	4	1	3
K01053	0	0	0	0	0	0	0	0	0	0	0	0	0	0	0	0	0	0	0
K01055	0	0	0	0	0	0	0	0	0	0	0	0	0	0	0	0	0	0	0
K01061	0	0	0	0	0	0	0	0	0	0	0	0	0	0	0	0	0	0	0
K01066	8	4	3	2	12	0	4	2	2	8	9	3	4	10	4	7	6	4	7
K01075	0	2	0	0	1	0	0	1	1	2	1	1	2	1	1	2	2	1	1
K01077	9	3	4	4	8	0	11	3	0	10	8	6	9	10	8	6	7	6	5
K01101	5	1	1	2	9	0	3	3	6	9	0	5	3	5	7	9	2	4	6
K01195	11	1	3	4	7	0	9	5	7	17	13	6	7	12	8	5	3	6	12
K01426	0	0	1	0	0	0	0	0	0	1	0	0	0	1	0	0	0	0	2
K01428	0	0	0	0	0	0	0	0	0	1	0	0	0	1	1	0	0	2	1
K01457	0	0	0	0	0	0	0	0	0	0	0	0	0	0	0	0	0	0	0
K01464	0	1	0	0	3	0	0	0	0	2	3	0	2	3	1	2	2	4	3
K01489	20	14	11	7	25	2	15	19	26	21	24	17	17	27	23	25	21	12	15
K01500	0	1	0	0	1	0	0	0	0	0	0	0	0	0	0	0	1	0	0
K01501	1	0	2	1	4	0	0	2	6	4	2	1	3	2	4	2	1	3	2
K01502	0	0	0	0	0	0	0	0	1	0	0	0	0	0	0	0	0	0	0
K01512	4	3	5	5	8	2	4	5	6	5	8	8	5	11	8	7	9	7	12
K01560	3	1	4	0	8	1	1	3	10	8	7	3	5	4	2	7	4	6	3
K01561	0	0	0	0	1	0	0	0	0	0	0	0	1	0	1	0	0	0	0
K01563	0	0	0	0	0	0	0	0	0	0	0	0	0	0	0	0	0	0	0
K01564	1	0	1	0	2	0	1	0	0	0	0	1	3	1	1	1	2	0	0
K01607	11	12	12	8	15	3	10	8	5	16	14	14	9	15	13	10	11	6	9
K01612	0	0	0	0	0	0	0	0	0	0	0	0	0	0	0	0	0	0	0
K01615	11	4	4	7	22	1	6	8	20	13	16	9	18	12	12	18	11	17	13
K01617	0	0	0	0	0	0	0	0	0	0	0	0	0	0	0	0	0	0	0
K01666	5	0	4	3	8	1	4	5	8	4	6	3	4	13	8	6	10	6	4
K01692	0	0	1	0	0	0	0	1	1	0	0	0	1	1	0	0	1	1	0
K01721	0	0	0	0	0	0	0	0	0	0	0	0	0	0	0	0	0	0	0
K01726	9	1	3	1	12	0	3	4	5	3	12	4	8	6	4	4	1	7	3
K01781	0	0	1	0	0	0	0	0	0	2	1	0	0	1	1	0	0	0	0
K01821	2	0	0	0	2	0	3	0	2	1	1	2	2	2	3	2	3	0	0
K01856	0	0	0	0	0	0	0	0	0	0	0	0	0	0	0	0	0	0	0
K01857	0	0	0	0	0	0	0	0	0	1	0	0	0	1	0	0	0	0	1
K01913	1	0	2	0	0	0	1	1	1	0	0	1	0	0	1	0	0	0	0
K01951	15	4	7	7	19	3	5	9	8	16	14	9	13	12	12	18	11	11	14
K02554	0	0	0	0	0	0	0	0	0	0	0	0	0	0	0	0	0	0	0
K03381	0	0	0	0	0	0	0	0	0	0	0	0	0	0	0	0	0	0	0
K03382	0	0	0	0	1	0	0	0	1	1	0	1	0	2	1	2	0	2	0
K03464	0	0	0	0	0	0	0	0	0	0	0	0	0	0	0	0	0	0	0
K03518	6	4	1	5	9	1	3	4	8	14	7	11	9	9	12	8	8	11	11
K03862	0	0	0	0	0	0	0	0	0	0	0	0	0	0	0	0	0	0	0
K04099	0	0	0	0	0	0	0	0	0	0	0	0	0	0	0	0	0	0	0
K04100	0	0	0	0	0	0	0	0	0	0	0	0	0	0	0	0	0	0	0
K04102	0	0	0	0	0	0	0	0	0	0	0	1	0	0	0	0	1	0	0
K04116	0	0	0	0	0	0	0	0	0	0	0	0	0	0	0	0	0	0	0
K05394	0	0	1	0	1	0	1	0	0	2	1	1	1	1	0	1	2	1	1

EC/KO	DA-AD-45	DA-AD-46	DA-AD-47	DA-AD-48	DA-AD-49	DA-AD-5	DA-AD-50	DA-AD-51	DA-AD-52	DA-AD-53	DA-AD-54	DA-AD-55	DA-AD-56	DA-AD-57	DA-AD-58	DA-AD-59	DA-AD-6	DA-AD-60	DA-AD-61
K05549	0	0	0	0	0	0	0	0	0	0	0	0	0	0	0	0	0	0	0
K05783	0	0	0	0	0	0	0	0	0	0	0	0	0	0	0	0	0	0	0
K05797	0	0	0	0	0	0	0	0	0	0	0	0	0	0	0	0	0	0	0
K06281	0	0	0	1	0	0	0	0	1	2	0	0	1	2	1	2	1	1	1
K06446	3	0	1	2	8	3	2	0	5	9	3	6	2	1	5	3	4	2	1
K06912	0	0	0	0	0	0	0	0	0	0	0	0	0	0	0	0	0	0	0
K07535	0	0	0	0	0	0	0	0	0	1	0	0	0	0	0	0	0	0	0
K07536	3	2	2	1	4	1	0	1	2	1	0	4	3	3	0	2	2	0	0
K08689	0	0	0	0	0	0	0	0	0	0	0	0	0	0	0	0	0	0	0
K08710	0	0	0	0	0	0	0	0	0	0	0	0	0	0	0	1	0	0	0
K09461	0	0	0	0	0	0	0	0	0	0	0	0	0	1	0	0	0	0	0
K10217	0	0	0	0	0	0	0	0	0	0	0	0	0	0	0	0	0	0	0
K10218	0	0	0	0	0	0	0	0	0	1	1	1	0	1	0	0	0	0	1
K10220	0	0	0	0	0	0	0	0	0	0	0	0	0	0	0	0	0	0	0
K11180	0	0	0	0	0	0	0	0	0	0	1	0	0	1	0	0	0	0	0
K13953	0	0	0	0	0	0	0	0	0	0	0	0	0	1	0	0	0	0	0
K14333	0	0	0	0	0	0	0	0	0	0	0	0	0	0	0	0	0	0	0
K14519	0	0	0	0	0	0	0	0	0	0	0	0	0	0	0	0	0	0	0
K15054	0	0	0	0	0	0	0	0	0	0	0	0	0	0	0	0	0	0	0
K16173	1	0	0	0	0	0	0	1	1	2	0	1	0	0	0	0	0	0	1
K16514	0	0	0	0	0	0	0	0	0	0	0	1	0	0	0	0	0	0	0
K16874	0	0	0	0	0	0	0	0	1	0	0	0	0	0	0	0	0	0	0

EC/KO	DA-AD-62	DA-AD-63	DA-AD-64	DA-AD-65	DA-AD-66	DA-AD-67	DA-AD-68	DA-AD-69	DA-AD-7	DA-AD-70	DA-AD-71	DA-AD-72	DA-AD-73	DA-AD-74	DA-AD-75	DA-AD-76	DA-AD-77	DA-AD-78	DA-AD-79
**1.1.1.-**	28	53	50	47	53	63	60	52	84	69	39	66	67	49	43	54	9	29	55
**1.1.1.1**	32	32	33	42	31	37	38	54	47	36	20	35	55	38	26	37	6	22	40
**1.1.1.157**	1	0	1	0	2	1	1	0	2	1	0	0	3	0	0	0	0	1	1
**1.1.1.205**	16	18	21	12	22	20	27	22	28	16	11	20	24	31	13	19	8	17	27
**1.1.1.35**	1	1	6	3	0	0	8	3	3	2	2	2	0	5	2	5	0	5	3
**1.12.99.6**	0	2	1	2	3	2	1	2	0	3	1	0	2	1	1	0	0	1	3
**1.13.11.-**	0	0	0	0	0	0	0	0	0	0	0	0	0	0	0	0	0	0	0
**1.13.11.1**	0	0	0	0	0	0	0	0	0	0	0	0	0	0	0	0	0	0	0
**1.13.11.2**	0	0	0	0	0	0	0	0	0	0	0	0	0	0	0	0	0	0	0
**1.13.11.3**	0	0	0	0	0	0	0	0	0	0	0	0	0	0	0	0	0	0	0
**1.13.11.39**	0	0	0	0	0	0	0	0	0	0	0	0	0	0	0	0	0	0	0
**1.13.11.5**	0	0	0	0	0	0	0	1	0	0	0	0	2	0	0	1	0	0	1
**1.13.11.8**	0	0	0	0	0	0	0	0	0	0	0	0	0	0	0	0	0	0	0
**1.14.12.10**	0	0	0	0	0	0	0	0	0	0	0	1	0	0	0	0	0	0	1
**1.14.12.13**	0	0	0	0	0	0	0	0	0	0	0	0	0	0	0	0	0	0	0
**1.14.12.18**	0	0	0	0	0	0	0	0	0	0	0	0	0	0	0	0	0	0	0
**1.14.13.-**	0	0	0	0	0	1	0	0	0	0	0	0	0	0	0	0	0	0	0
**1.14.13.1**	0	0	0	0	0	0	0	0	0	0	0	0	0	0	0	0	0	0	0
**1.14.13.2**	0	0	0	0	0	0	0	0	0	0	0	0	0	0	0	0	0	0	0
**1.14.13.50**	0	0	0	0	0	0	0	0	0	0	0	0	0	0	0	0	0	0	0
**1.14.13.7**	0	0	1	0	0	0	0	0	0	0	0	1	0	0	0	0	0	0	0
**1.14.13.8**	0	0	0	0	0	0	0	0	0	0	0	0	0	0	0	0	0	0	0
**1.14.13.82**	1	0	0	0	0	0	0	0	0	0	0	0	0	0	0	0	0	0	0
**1.14.99.-**	0	0	0	0	0	0	0	0	0	0	0	0	0	0	0	0	0	0	0
**1.17.99.1**	0	0	0	0	0	0	0	0	0	0	0	0	0	0	0	0	0	0	0
**1.18.6.1**	0	0	0	0	0	0	0	0	0	0	0	0	0	0	1	0	0	0	0
**1.2.1.-**	0	0	0	0	0	0	0	0	0	0	0	0	0	0	0	0	0	0	0
**1.2.1.10**	5	4	5	7	2	3	5	4	6	5	4	4	4	5	5	3	3	8	5
**1.2.1.3**	6	8	9	6	10	9	12	6	10	8	5	7	13	14	7	8	2	5	7
**1.2.1.39**	0	0	0	0	0	0	0	0	0	0	0	0	0	0	0	0	0	0	0
**1.2.7.1**	13	14	15	8	17	7	21	14	20	16	5	8	12	17	12	10	3	7	17
**1.2.99.2**	1	4	2	4	2	4	2	5	4	4	1	4	4	3	3	3	1	1	2
**1.3.1.-**	0	0	1	0	0	0	0	1	0	0	0	0	0	0	0	0	0	0	0
**1.3.1.2**	0	0	3	0	1	1	1	2	2	1	0	0	2	0	2	0	0	1	1
**1.3.1.25**	0	0	0	0	0	0	0	0	0	0	0	0	0	0	0	0	0	0	0
**1.3.99.-**	0	0	1	0	0	0	0	0	0	0	0	0	0	1	0	0	0	0	0
**1.6.5.-**	30	38	45	25	41	52	58	57	57	48	32	36	47	63	29	41	12	37	45
**1.7.1.-**	9	10	11	7	10	5	13	8	12	9	9	6	6	10	4	12	4	6	15
**1.8.99.3**	1	0	0	0	0	1	0	0	0	1	0	0	0	0	0	0	0	1	0
**2.1.1.-**	262	249	305	283	258	249	350	357	373	301	162	247	314	342	251	324	100	194	333
**2.3.1.-**	125	146	141	163	190	129	185	191	237	162	106	141	164	182	139	160	40	103	190
**2.3.1.16**	2	2	2	0	2	2	0	2	1	0	0	1	2	1	1	0	0	2	1
**2.3.1.5**	0	0	0	0	0	0	0	0	0	0	0	0	0	0	0	0	0	0	0
**2.3.1.9**	2	1	0	1	1	0	2	1	1	2	0	0	0	2	0	1	0	2	0
**2.4.2.10**	15	7	13	17	11	10	21	13	21	12	11	9	17	20	13	15	5	12	17
**2.4.2.3**	14	10	14	6	13	10	17	11	8	18	3	13	13	14	8	11	2	8	12
**2.4.2.4**	8	9	5	7	3	4	6	12	3	3	0	1	4	7	6	5	1	7	6
**2.4.2.8**	13	10	14	12	10	13	22	12	17	14	7	12	16	19	18	22	5	13	19
**2.5.1.-**	16	16	22	19	23	16	28	23	24	17	13	23	20	30	12	22	6	12	27

EC/KO	DA-AD-62	DA-AD-63	DA-AD-64	DA-AD-65	DA-AD-66	DA-AD-67	DA-AD-68	DA-AD-69	DA-AD-7	DA-AD-70	DA-AD-71	DA-AD-72	DA-AD-73	DA-AD-74	DA-AD-75	DA-AD-76	DA-AD-77	DA-AD-78	DA-AD-79
**2.5.1.18**	1	0	0	0	0	0	1	2	3	1	0	1	1	1	0	3	0	4	1
**2.6.1.-**	29	38	39	29	49	35	31	48	54	29	25	34	40	41	33	41	9	19	47
**2.7.1.21**	7	7	15	10	6	5	12	11	6	8	5	5	4	10	8	8	2	12	11
**2.7.1.48**	23	29	40	31	30	25	42	39	33	25	15	26	26	42	32	36	9	24	37
**2.7.4.-**	14	15	23	22	17	17	24	23	22	14	8	14	16	25	12	17	5	13	22
**2.8.3.-**	1	1	5	1	1	2	2	0	1	0	1	1	3	1	3	0	0	0	2
**2.8.3.1**	0	0	0	0	0	0	0	0	0	1	0	1	2	0	0	0	0	1	0
**2.8.3.12**	1	0	0	2	0	2	0	2	1	0	1	0	2	0	0	0	0	0	0
**2.8.3.6**	1	0	0	0	0	0	0	1	0	0	0	0	0	0	0	0	0	0	0
**2.8.3.8**	3	1	3	0	1	1	2	2	3	1	0	2	5	1	0	0	0	2	0
**3.1.1.-**	3	6	3	6	7	8	2	8	5	8	6	10	14	3	7	4	1	7	10
**3.1.1.1**	2	4	3	6	6	9	7	6	14	8	3	5	10	5	3	3	1	1	7
**3.1.1.17**	0	1	0	4	5	3	1	2	4	2	1	1	3	1	1	1	0	0	2
**3.1.1.2**	0	0	0	0	0	0	0	1	0	0	0	0	0	1	1	1	0	1	0
**3.1.1.24**	5	4	2	2	2	0	4	1	3	4	1	0	4	9	2	1	2	3	4
**3.1.1.45**	0	0	0	0	0	0	0	0	0	0	0	0	0	0	0	0	0	1	0
**3.1.2.-**	0	0	0	0	0	0	1	0	0	1	2	0	0	0	0	0	0	0	0
**3.1.2.23**	2	5	4	5	5	6	6	6	6	6	3	5	2	9	2	6	1	3	8
**3.1.3.1**	9	10	14	11	25	18	24	13	19	13	4	18	25	15	11	13	4	7	17
**3.1.3.2**	4	5	5	2	6	4	6	4	7	3	3	5	2	4	4	3	1	3	3
**3.1.3.41**	0	0	0	0	0	0	1	0	0	0	0	0	0	0	0	0	0	0	0
**3.2.1.31**	1	5	5	5	6	5	4	6	5	7	5	8	5	5	4	2	2	1	3
**3.3.2.9**	0	0	0	0	0	0	0	0	0	0	0	0	0	0	0	0	0	0	0
**3.5.1.-**	27	27	38	30	21	23	49	35	28	22	18	24	25	32	28	38	11	21	37
**3.5.1.4**	2	3	2	1	2	3	5	2	3	2	2	4	2	0	3	2	1	1	3
**3.5.1.5**	1	1	0	0	1	1	1	0	2	2	2	0	2	1	0	0	0	1	4
**3.5.1.54**	4	4	2	5	3	7	4	0	4	2	5	4	5	5	1	5	1	2	4
**3.5.1.6**	0	0	0	0	1	3	2	0	2	0	2	2	1	1	2	0	1	0	3
**3.5.2.-**	0	0	0	0	0	1	0	0	0	0	0	1	0	0	0	0	0	0	0
**3.5.2.2**	2	1	0	0	1	0	4	1	2	2	1	1	5	2	2	0	1	1	3
**3.5.4.-**	19	26	44	32	28	22	37	31	32	23	15	21	24	32	21	24	8	22	35
**3.5.4.5**	20	17	22	9	15	9	25	18	15	16	8	11	9	19	18	19	4	19	22
**3.5.5.1**	2	1	2	2	1	1	0	1	1	0	0	1	4	1	2	0	0	1	2
**3.5.5.7**	0	0	0	0	0	0	0	0	0	0	0	0	0	0	0	0	0	0	0
**3.5.99.3**	1	0	0	0	0	0	0	0	0	0	0	0	1	0	0	0	0	1	0
**3.6.1.7**	2	3	7	2	7	8	4	8	8	8	5	9	6	3	3	7	1	0	9
**3.7.1.-**	1	1	0	1	2	0	0	1	2	2	0	0	2	2	0	2	1	1	1
**3.7.1.2**	2	4	2	6	6	3	6	4	6	5	2	3	5	2	5	6	0	1	4
**3.8.1.2**	4	7	9	7	7	4	6	9	7	7	4	3	5	8	7	9	2	2	9
**3.8.1.3**	0	0	0	0	0	0	0	0	0	0	0	0	0	0	0	0	0	0	0
**3.8.1.5**	1	0	0	3	0	1	2	0	1	2	0	0	1	2	0	0	0	2	1
**4.1.1.-**	6	5	7	4	12	9	13	10	10	14	3	14	16	10	5	13	2	3	9
**4.1.1.44**	17	26	18	24	19	15	43	29	31	29	23	26	26	30	21	24	7	15	33
**4.1.1.55**	0	0	0	0	0	0	0	0	0	0	0	1	0	0	0	0	0	0	0
**4.1.1.7**	0	0	0	0	0	0	0	0	0	0	0	0	0	0	0	0	0	0	0
**4.1.1.70**	0	0	0	0	0	1	0	0	0	0	1	1	0	0	0	0	0	0	0
**4.1.1.77**	0	0	0	0	0	0	0	0	0	0	0	0	0	0	0	0	0	0	0
**4.1.2.-**	4	3	7	10	2	3	8	4	4	7	3	7	7	9	5	4	3	6	8
**4.1.3.-**	14	18	24	14	23	17	27	27	29	13	12	22	17	27	22	23	6	8	27

EC/KO	DA-AD-62	DA-AD-63	DA-AD-64	DA-AD-65	DA-AD-66	DA-AD-67	DA-AD-68	DA-AD-69	DA-AD-7	DA-AD-70	DA-AD-71	DA-AD-72	DA-AD-73	DA-AD-74	DA-AD-75	DA-AD-76	DA-AD-77	DA-AD-78	DA-AD-79
4.1.3.39	1	0	0	0	0	1	0	2	0	0	1	0	0	1	1	1	0	1	0
4.1.99.-	3	3	4	2	3	2	5	3	1	2	2	2	2	2	4	2	1	1	3
4.2.1.-	36	47	45	44	56	58	47	43	69	48	46	43	61	58	36	61	19	25	76
4.2.1.17	4	4	8	6	6	7	5	10	12	2	2	6	8	4	1	3	1	5	3
4.2.1.80	0	0	0	0	0	0	0	0	0	0	0	0	0	0	0	0	0	0	0
4.2.1.83	0	2	0	0	2	0	1	3	0	0	0	1	0	1	1	0	1	0	0
4.2.1.84	1	1	0	0	1	0	0	1	0	0	0	1	1	0	2	1	0	0	0
5.1.2.2	0	0	0	0	0	0	0	0	0	0	0	0	1	0	0	0	0	0	0
5.2.1.2	0	0	0	0	0	0	0	0	0	0	0	0	0	0	0	0	0	0	0
5.3.3.4	0	0	0	0	0	0	0	0	0	0	0	0	0	0	0	0	0	0	0
5.3.99.-	2	4	3	3	2	3	2	5	4	6	2	3	4	2	0	4	4	1	3
5.4.99.-	1	1	1	0	0	0	0	1	1	0	0	2	0	1	0	1	0	0	1
5.5.1.1	2	1	2	3	5	4	2	1	5	4	1	2	4	4	2	1	0	1	4
5.5.1.2	0	0	0	0	0	1	0	0	0	0	0	0	0	0	0	0	0	0	0
6.2.1.-	0	0	0	0	0	0	0	0	0	0	0	0	0	0	0	0	0	0	0
6.3.5.2	15	21	17	26	22	10	24	14	21	26	13	19	20	16	15	18	5	14	18
K00002	3	1	1	0	2	4	3	4	4	3	1	0	3	3	0	4	0	0	0
K00055	0	0	0	0	0	0	0	0	1	0	0	0	0	0	0	0	0	0	1
K00074	3	3	8	3	2	1	10	5	4	2	4	3	4	6	3	4	1	9	4
K00088	17	13	22	15	17	16	17	12	20	14	7	13	19	24	12	15	6	14	12
K00100	36	37	37	44	55	43	62	42	77	47	29	53	62	54	38	54	11	22	54
K00128	4	2	3	3	2	0	1	2	0	2	0	2	1	3	0	1	1	2	3
K00129	0	0	0	0	0	0	0	0	0	0	0	0	0	0	0	0	0	0	0
K00132	0	1	1	0	0	0	3	1	0	1	0	0	1	0	0	2	0	0	1
K00141	0	0	0	0	0	0	0	0	0	0	0	0	0	0	0	0	0	0	0
K00146	0	0	0	0	0	0	0	0	0	0	0	0	0	0	0	0	0	0	0
K00148	0	0	0	0	0	0	0	0	0	0	0	0	0	0	0	0	0	0	0
K00155	0	1	0	0	0	0	0	0	1	0	0	1	0	0	1	0	0	0	0
K00169	3	3	3	2	2	0	2	1	5	3	0	3	2	2	0	4	0	0	4
K00224	0	1	0	1	1	1	1	0	1	0	1	1	1	1	1	1	0	0	0
K00274	0	0	0	0	0	0	0	0	0	0	0	0	0	0	0	0	0	0	0
K00446	0	0	0	0	0	0	0	0	0	0	0	0	0	0	0	0	0	0	0
K00448	0	0	0	0	0	0	0	0	0	0	0	0	0	0	0	0	0	0	0
K00462	0	1	0	1	1	1	1	0	3	1	2	0	1	1	1	1	0	0	0
K00480	0	0	0	0	0	0	0	0	0	0	0	0	0	0	0	0	0	0	0
K00481	0	0	0	0	0	0	0	0	0	0	0	0	0	0	0	0	0	0	0
K00539	0	0	0	0	0	2	1	3	2	0	0	0	3	2	0	2	0	0	0
K00599	41	39	53	41	48	45	47	70	57	40	27	45	48	50	47	62	12	18	63
K00626	4	3	2	2	3	4	4	3	2	3	0	1	4	3	1	2	1	5	1
K00632	0	0	0	0	0	0	0	0	0	0	0	0	0	0	0	0	0	0	0
K00680	35	37	43	43	40	35	64	49	57	50	28	36	42	48	37	48	6	35	63
K00757	11	9	13	8	13	13	13	15	12	15	3	16	14	15	10	11	2	6	9
K00758	5	0	6	7	3	5	7	8	6	2	6	2	3	8	6	8	1	6	1
K00760	28	21	27	29	19	23	39	23	36	23	16	21	23	30	27	31	7	19	32
K00799	0	0	1	0	0	0	0	2	1	0	0	0	0	0	1	0	0	4	1
K00857	15	11	22	17	11	8	20	14	18	11	7	5	7	16	10	15	2	19	13
K00876	28	24	38	32	30	21	37	30	30	24	11	24	28	34	28	29	7	24	34
K01026	2	2	1	0	0	1	2	2	2	0	0	1	1	0	0	1	0	0	0
K01031	0	0	0	0	0	0	0	0	0	0	0	0	0	0	0	0	0	0	0

EC/KO	DA-AD-62	DA-AD-63	DA-AD-64	DA-AD-65	DA-AD-66	DA-AD-67	DA-AD-68	DA-AD-69	DA-AD-7	DA-AD-70	DA-AD-71	DA-AD-72	DA-AD-73	DA-AD-74	DA-AD-75	DA-AD-76	DA-AD-77	DA-AD-78	DA-AD-79
K01034	2	1	0	5	1	1	1	2	3	3	0	0	2	1	1	1	0	4	1
K01039	0	0	0	0	0	0	0	2	0	0	0	1	1	1	0	0	0	0	0
K01041	3	4	4	4	4	5	5	7	6	5	1	4	4	4	4	3	1	3	3
K01053	0	0	0	1	0	0	0	0	1	0	0	0	1	0	0	0	0	0	0
K01055	0	0	0	0	0	0	0	0	0	0	0	0	0	0	0	0	0	0	0
K01061	0	0	0	0	0	0	0	0	0	0	0	0	0	0	0	0	0	2	0
K01066	5	2	3	12	8	10	8	8	8	9	4	3	8	9	2	5	0	3	6
K01075	0	0	0	1	1	0	1	2	1	0	0	2	0	1	1	1	0	2	0
K01077	5	4	10	7	10	9	7	7	7	9	3	5	12	7	8	6	0	3	11
K01101	5	1	8	6	2	2	6	3	8	2	2	4	2	4	4	1	1	7	1
K01195	6	9	8	9	9	11	12	7	13	8	12	9	9	9	9	3	1	1	3
K01426	1	0	0	1	0	1	0	1	0	1	1	0	0	0	2	1	0	1	0
K01428	0	0	0	0	1	0	1	0	0	1	0	0	3	0	0	0	0	0	0
K01457	0	0	0	0	0	0	0	0	0	0	0	0	0	0	0	0	0	0	0
K01464	3	0	0	1	1	0	4	1	1	4	1	1	5	2	2	0	0	2	2
K01489	21	18	26	18	22	12	27	25	21	22	13	14	13	29	18	22	7	22	23
K01500	0	0	0	0	0	0	0	0	0	0	0	0	0	0	0	0	0	0	0
K01501	2	1	2	5	0	2	2	2	5	0	1	1	7	1	2	0	0	1	1
K01502	0	0	0	0	0	0	0	0	0	0	0	0	0	0	0	0	0	0	0
K01512	7	7	8	4	8	13	9	13	11	10	8	13	11	6	7	11	2	2	10
K01560	6	6	7	5	3	3	9	5	8	8	1	4	4	8	2	6	5	5	2
K01561	0	0	0	0	0	0	0	0	1	0	0	0	0	0	1	0	0	0	0
K01563	0	0	0	0	0	0	0	0	0	0	0	0	0	0	0	0	0	0	0
K01564	0	2	1	2	2	1	2	1	2	1	0	1	1	2	0	1	0	0	1
K01607	8	17	9	13	10	8	19	18	11	11	11	11	12	14	4	10	4	8	19
K01612	0	0	0	0	0	0	0	0	0	0	0	0	0	0	0	0	0	0	0
K01615	15	14	14	15	17	11	6	15	17	17	10	10	22	25	6	7	2	11	11
K01617	0	0	0	0	0	0	0	0	0	0	0	0	0	0	0	0	0	0	0
K01666	6	8	10	4	3	8	8	15	11	5	5	8	5	8	7	8	2	2	9
K01692	0	0	0	1	0	1	0	2	0	0	0	0	1	1	0	0	0	3	0
K01721	0	0	0	0	0	0	0	0	0	0	0	0	0	0	0	0	0	0	0
K01726	2	4	5	4	11	7	7	4	5	5	6	9	3	7	2	8	0	3	7
K01781	0	0	0	0	1	2	2	0	0	2	0	1	3	0	0	0	0	0	0
K01821	0	1	3	2	0	0	2	1	3	1	0	1	1	3	3	0	2	1	1
K01856	0	0	0	0	0	0	0	0	0	0	0	0	0	0	0	0	0	0	0
K01857	0	0	1	0	1	1	0	0	1	0	0	1	0	2	0	1	0	0	0
K01913	0	0	1	1	0	0	0	0	1	0	0	0	1	0	1	0	0	0	0
K01951	10	12	12	20	14	9	14	9	20	16	6	10	16	14	8	8	1	8	7
K02554	0	0	0	0	0	0	0	0	0	0	0	0	1	0	0	0	0	0	0
K03381	0	0	0	0	0	0	0	0	0	0	0	0	0	0	0	0	0	0	0
K03382	1	0	1	0	0	0	1	0	1	0	0	0	2	1	0	0	0	1	0
K03464	0	0	0	0	0	0	0	0	0	0	0	0	0	0	0	0	0	0	0
K03518	10	8	4	7	6	6	9	9	14	12	8	9	20	13	3	4	3	8	10
K03862	0	0	0	0	0	0	0	0	0	0	0	0	0	0	0	0	0	0	0
K04099	0	0	0	0	0	0	0	0	0	0	0	0	0	0	0	0	0	0	0
K04100	0	0	0	0	0	0	0	0	0	0	0	0	0	0	0	0	0	0	0
K04102	0	0	0	0	0	0	0	0	0	0	0	0	0	0	0	0	0	0	0
K04116	0	0	0	0	0	0	0	0	0	0	0	0	0	0	0	0	0	0	0
K05394	1	0	1	3	0	1	1	0	1	1	0	1	1	0	1	0	0	0	0

EC/KO	DA-AD-62	DA-AD-63	DA-AD-64	DA-AD-65	DA-AD-66	DA-AD-67	DA-AD-68	DA-AD-69	DA-AD-7	DA-AD-70	DA-AD-71	DA-AD-72	DA-AD-73	DA-AD-74	DA-AD-75	DA-AD-76	DA-AD-77	DA-AD-78	DA-AD-79
**K05549**	0	0	0	0	0	0	0	0	0	0	0	0	0	0	0	0	0	0	0
**K05783**	0	0	0	0	0	0	0	0	0	0	0	0	0	0	0	0	0	0	0
**K05797**	0	0	0	0	0	0	0	0	0	0	0	0	0	0	0	0	0	0	0
**K06281**	0	2	1	2	2	2	1	1	0	2	1	0	2	0	0	0	0	0	3
**K06446**	4	1	2	4	3	9	8	3	6	4	2	6	6	4	1	0	2	2	3
**K06912**	0	0	0	0	0	0	0	0	0	0	0	0	0	0	0	0	0	0	0
**K07535**	0	0	1	0	0	0	0	0	0	0	0	0	0	0	0	1	0	0	0
**K07536**	0	1	1	2	4	1	4	2	3	2	2	3	0	3	1	3	0	1	2
**K08689**	0	0	0	0	0	0	0	0	0	0	0	0	0	0	0	0	0	0	0
**K08710**	0	0	0	0	0	0	0	0	0	0	0	0	0	0	0	0	0	1	0
**K09461**	0	0	0	0	0	0	1	0	0	0	0	0	0	0	0	0	0	0	0
**K10217**	0	0	0	0	0	0	0	0	0	0	0	0	0	0	0	0	0	0	0
**K10218**	0	0	0	0	0	0	2	0	0	1	0	1	0	0	0	0	0	0	0
**K10220**	0	0	0	0	0	0	0	0	0	0	0	0	0	0	0	0	0	0	0
**K11180**	0	0	0	0	0	1	1	0	1	0	0	0	0	0	0	0	0	0	0
**K13953**	0	0	0	0	0	0	0	0	2	0	0	0	0	0	0	0	0	0	0
**K14333**	0	0	0	0	0	0	0	0	0	0	0	0	0	0	0	0	0	0	0
**K14519**	0	0	0	0	0	0	0	0	0	0	0	0	0	0	0	0	0	0	0
**K15054**	0	0	0	0	0	0	0	0	0	0	0	1	0	0	0	0	0	0	0
**K16173**	0	0	0	0	0	2	0	0	0	0	0	0	0	0	0	0	0	0	0
**K16514**	0	0	0	0	0	0	0	0	0	0	0	0	1	0	0	0	0	1	0
**K16874**	0	0	0	0	0	0	0	0	0	0	0	0	1	0	0	0	0	0	0

EC/KO	DA-AD-8	DA-AD-80	DA-AD-81	DA-AD-82	DA-AD-83	DA-AD-84	DA-AD-85	DA-AD-9	ES-AD-1	ES-AD-10	ES-AD-11	ES-AD-12	ES-AD-13	ES-AD-14	ES-AD-15	ES-AD-16	ES-AD-17	ES-AD-18	ES-AD-19
**1.1.1.-**	9	54	63	59	25	52	87	8	41	34	84	53	32	51	30	71	47	56	56
**1.1.1.1**	16	24	35	35	15	28	92	10	15	11	47	34	28	28	14	33	41	38	45
**1.1.1.157**	0	1	0	0	0	0	1	0	2	0	0	0	0	0	0	2	0	2	0
**1.1.1.205**	5	28	31	24	16	22	39	4	11	9	13	24	13	17	13	22	14	19	27
**1.1.1.35**	0	7	6	5	1	0	3	0	3	0	2	3	0	2	1	3	3	3	5
**1.12.99.6**	0	0	0	2	0	3	2	0	3	0	2	0	0	2	0	2	4	0	3
**1.13.11.-**	0	0	0	0	0	0	0	0	0	0	0	0	0	0	0	0	0	0	0
**1.13.11.1**	0	0	0	0	0	0	0	0	0	0	0	0	0	0	0	0	0	0	0
**1.13.11.2**	0	0	0	0	0	0	0	0	0	0	0	0	0	0	0	0	0	0	0
**1.13.11.3**	0	0	0	0	0	0	0	0	0	0	0	0	0	0	0	0	0	0	0
**1.13.11.39**	0	0	0	0	0	0	0	0	0	0	0	0	0	0	0	0	0	0	0
**1.13.11.5**	0	0	0	1	0	0	1	0	0	0	0	0	0	0	0	0	1	0	0
**1.13.11.8**	0	0	0	0	0	0	0	0	0	0	0	0	0	0	0	0	0	0	0
**1.14.12.10**	0	1	0	0	0	0	0	0	0	0	0	0	0	0	0	0	0	0	1
**1.14.12.13**	0	0	0	0	0	0	0	0	0	0	0	0	0	0	0	0	0	0	0
**1.14.12.18**	0	0	0	0	0	0	0	0	0	0	0	0	0	0	0	0	0	0	0
**1.14.13.-**	0	0	0	0	0	0	0	0	0	1	2	0	0	0	0	0	3	0	0
**1.14.13.1**	0	0	0	0	0	0	0	0	0	0	0	0	0	0	0	0	0	0	0
**1.14.13.2**	0	0	0	0	0	0	0	0	0	0	0	0	0	0	0	0	0	0	0
**1.14.13.50**	0	0	0	0	0	0	0	0	0	0	0	0	0	0	0	0	0	0	0
**1.14.13.7**	0	0	1	0	0	0	0	0	0	0	0	0	0	0	0	0	0	0	0
**1.14.13.8**	0	0	0	0	0	0	0	0	0	0	0	0	0	0	0	0	0	0	0
**1.14.13.82**	0	0	0	0	0	0	0	0	0	0	0	0	0	0	0	0	0	0	0
**1.14.99.-**	0	0	0	0	0	0	0	0	0	0	0	0	0	0	0	0	0	0	0
**1.17.99.1**	0	0	0	0	0	0	0	0	0	0	0	0	0	0	0	0	0	0	0
**1.18.6.1**	0	0	0	0	0	0	0	0	0	0	0	0	0	0	0	0	0	0	0
**1.2.1.-**	0	0	0	0	0	0	0	0	0	0	1	0	0	0	0	0	0	0	1
**1.2.1.10**	0	4	5	3	2	6	6	0	1	2	6	4	1	3	3	4	5	5	6
**1.2.1.3**	1	10	9	15	4	4	23	0	5	4	12	10	5	11	2	8	9	8	9
**1.2.1.39**	0	0	1	0	0	0	0	0	0	0	0	0	0	0	0	0	0	0	0
**1.2.7.1**	3	13	14	15	5	8	28	2	6	2	9	9	14	13	10	7	5	12	5
**1.2.99.2**	1	2	5	3	1	2	6	0	1	1	8	2	1	4	0	3	11	0	4
**1.3.1.-**	0	0	0	0	0	0	0	0	1	0	0	1	0	0	0	1	1	0	0
**1.3.1.2**	1	2	1	1	0	2	1	0	0	0	1	0	1	0	0	1	1	0	2
**1.3.1.25**	0	0	0	0	0	0	0	0	0	0	0	0	0	0	0	0	0	0	0
**1.3.99.-**	0	0	0	0	0	0	0	0	0	0	1	0	0	0	0	0	0	0	1
**1.6.5.-**	19	39	54	42	16	47	63	10	42	25	31	54	39	40	28	54	30	46	35
**1.7.1.-**	3	6	14	9	6	12	14	1	9	2	3	9	4	7	5	9	9	12	8
**1.8.99.3**	0	0	0	0	0	0	0	0	0	0	1	0	0	0	0	0	0	0	1
**2.1.1.-**	92	359	349	334	115	222	537	58	136	108	222	262	222	282	142	290	222	241	265
**2.3.1.-**	55	174	182	201	78	146	271	27	84	81	132	152	129	152	81	167	128	120	131
**2.3.1.16**	0	1	0	3	0	1	2	1	0	1	3	1	1	1	1	2	2	3	3
**2.3.1.5**	0	0	0	0	0	0	0	0	0	0	1	0	0	0	0	0	1	0	1
**2.3.1.9**	0	1	0	0	0	0	1	0	0	0	5	0	1	1	1	1	0	1	4
**2.4.2.10**	3	17	21	18	10	14	31	5	6	8	15	22	17	10	10	14	18	20	19
**2.4.2.3**	2	14	14	11	3	4	26	0	2	5	13	10	5	11	8	16	4	9	12
**2.4.2.4**	1	9	13	7	0	2	9	0	0	0	4	5	4	7	1	5	4	5	2
**2.4.2.8**	3	15	19	13	7	13	34	3	5	3	11	15	12	11	9	14	9	14	16
**2.5.1.-**	10	19	24	26	14	19	32	5	11	9	14	25	15	20	13	24	19	22	19

EC/KO	DA-AD-8	DA-AD-80	DA-AD-81	DA-AD-82	DA-AD-83	DA-AD-84	DA-AD-85	DA-AD-9	ES-AD-1	ES-AD-10	ES-AD-11	ES-AD-12	ES-AD-13	ES-AD-14	ES-AD-15	ES-AD-16	ES-AD-17	ES-AD-18	ES-AD-19
**2.5.1.18**	0	2	0	3	1	1	10	0	0	1	4	0	1	3	1	2	4	1	2
**2.6.1.-**	8	48	37	49	17	30	71	12	10	11	35	32	26	32	19	34	20	30	38
**2.7.1.21**	5	11	12	9	8	4	8	2	6	3	6	13	9	6	3	6	6	8	10
**2.7.1.48**	13	42	38	33	19	22	51	8	16	11	27	35	24	27	16	38	18	28	23
**2.7.4.-**	5	18	25	22	8	11	24	3	5	10	13	16	8	22	10	15	19	20	10
**2.8.3.-**	0	3	0	2	1	2	2	0	0	1	3	2	0	1	1	3	3	1	2
**2.8.3.1**	0	0	0	0	0	0	1	0	1	0	2	0	0	1	0	0	1	0	0
**2.8.3.12**	0	1	0	0	1	2	1	0	4	0	0	1	0	0	1	1	0	0	2
**2.8.3.6**	0	0	1	0	0	0	0	0	0	0	0	1	0	1	0	1	0	2	0
**2.8.3.8**	0	2	1	2	0	0	3	0	1	1	6	2	1	2	1	0	2	1	3
**3.1.1.-**	3	10	8	8	4	6	5	0	5	6	10	5	7	4	2	7	4	6	7
**3.1.1.1**	2	4	6	8	7	7	7	0	4	4	8	9	0	5	6	12	6	6	5
**3.1.1.17**	0	1	1	3	1	2	1	0	2	1	3	4	0	1	0	2	0	0	0
**3.1.1.2**	0	0	1	0	0	0	1	0	0	0	2	0	0	0	0	0	1	0	3
**3.1.1.24**	1	6	8	5	0	1	8	0	3	1	3	4	2	6	1	3	5	4	3
**3.1.1.45**	0	0	0	0	0	0	0	0	0	0	1	0	0	0	0	0	1	0	0
**3.1.2.-**	0	0	0	0	0	0	2	0	1	0	1	0	0	1	0	1	3	0	1
**3.1.2.23**	5	4	8	8	5	7	7	2	4	3	3	8	6	2	3	8	4	3	3
**3.1.3.1**	5	12	8	21	12	27	14	3	14	6	7	18	8	12	7	15	13	16	9
**3.1.3.2**	3	1	5	4	6	7	5	2	2	4	1	9	2	3	2	3	4	7	3
**3.1.3.41**	0	0	0	0	0	0	1	0	0	0	0	0	0	0	0	0	0	0	0
**3.2.1.31**	0	8	4	5	1	7	4	0	3	0	6	3	1	8	5	5	7	4	2
**3.3.2.9**	0	0	0	0	0	0	0	0	0	0	0	0	0	0	0	0	0	0	0
**3.5.1.-**	7	33	40	35	9	16	41	8	4	6	20	28	21	27	8	27	18	25	21
**3.5.1.4**	3	2	5	3	2	3	4	1	0	1	2	2	4	2	2	4	6	2	7
**3.5.1.5**	0	1	3	2	0	2	5	0	0	0	0	2	0	0	0	1	0	0	0
**3.5.1.54**	0	6	2	5	3	4	10	0	1	0	5	2	4	10	4	5	4	3	12
**3.5.1.6**	1	0	1	1	1	2	3	0	0	1	2	0	1	2	1	0	1	2	2
**3.5.2.-**	0	0	0	0	0	0	0	0	0	0	0	0	0	1	0	0	0	0	0
**3.5.2.2**	1	2	0	3	1	2	3	0	0	1	2	1	1	2	1	0	1	0	4
**3.5.4.-**	12	36	38	33	16	20	51	6	13	9	18	24	26	27	9	32	18	28	17
**3.5.4.5**	4	23	23	17	6	12	26	3	4	3	10	16	10	10	7	12	12	10	10
**3.5.5.1**	0	2	0	4	1	2	3	0	0	1	3	3	2	0	0	1	1	0	3
**3.5.5.7**	0	0	0	0	0	0	0	0	0	0	0	0	0	0	0	0	0	0	0
**3.5.99.3**	0	0	0	0	0	0	0	0	0	1	0	0	0	0	0	0	0	0	0
**3.6.1.7**	1	5	7	6	2	0	17	0	1	2	2	0	4	5	2	7	4	2	5
**3.7.1.-**	0	1	0	1	0	0	6	0	1	0	2	2	1	3	2	4	2	3	4
**3.7.1.2**	2	5	4	5	3	5	6	1	3	4	2	3	0	3	0	5	2	2	2
**3.8.1.2**	2	6	4	3	2	8	9	2	9	2	6	5	2	3	3	5	4	3	8
**3.8.1.3**	0	0	0	0	0	0	0	0	0	0	0	0	0	0	0	0	0	0	0
**3.8.1.5**	0	0	0	0	1	0	1	0	1	0	2	0	0	2	0	1	1	1	3
**4.1.1.-**	5	9	9	9	4	11	18	2	7	2	9	9	4	7	5	16	13	7	12
**4.1.1.44**	11	22	28	34	15	22	42	10	32	11	18	34	7	22	15	33	18	22	22
**4.1.1.55**	0	0	0	0	0	0	0	0	0	0	0	0	0	0	0	0	0	0	0
**4.1.1.7**	0	0	0	0	0	0	0	0	0	0	0	0	0	0	0	0	0	0	0
**4.1.1.70**	0	0	0	1	0	0	0	0	1	0	1	0	0	1	0	0	0	0	0
**4.1.1.77**	0	0	0	0	0	0	0	0	0	0	0	0	0	0	0	0	0	0	0
**4.1.2.-**	1	6	3	6	3	3	10	0	5	1	14	9	6	7	5	7	10	8	11
**4.1.3.-**	7	19	24	28	13	18	46	3	9	8	13	12	14	22	8	18	15	13	17

EC/KO	DA-AD-8	DA-AD-80	DA-AD-81	DA-AD-82	DA-AD-83	DA-AD-84	DA-AD-85	DA-AD-9	ES-AD-1	ES-AD-10	ES-AD-11	ES-AD-12	ES-AD-13	ES-AD-14	ES-AD-15	ES-AD-16	ES-AD-17	ES-AD-18	ES-AD-19
4.1.3.39	0	1	0	1	0	0	1	0	0	0	1	0	0	0	0	0	0	0	1
4.1.99.-	0	1	3	1	0	2	3	3	0	0	2	0	1	1	1	2	0	0	2
4.2.1.-	7	55	53	59	28	58	86	10	26	23	44	51	29	56	23	58	36	32	39
4.2.1.17	2	7	6	3	3	4	11	2	3	1	4	3	4	7	4	6	1	6	5
4.2.1.80	0	0	0	0	0	0	0	0	0	0	0	0	0	0	0	0	0	0	0
4.2.1.83	0	0	0	1	2	0	0	0	0	0	0	2	0	0	0	0	0	2	0
4.2.1.84	0	0	0	1	1	0	1	0	0	0	0	0	0	0	0	0	0	0	1
5.1.2.2	0	0	0	0	0	0	0	0	0	0	0	0	0	0	0	0	0	0	0
5.2.1.2	0	0	0	0	0	0	0	0	0	0	0	0	0	0	0	0	0	0	0
5.3.3.4	0	0	0	0	0	0	0	0	0	0	0	0	0	0	0	0	0	0	0
5.3.99.-	1	3	2	5	2	2	3	0	3	1	5	3	1	3	1	4	0	1	4
5.4.99.-	0	2	0	2	0	1	0	0	0	0	0	0	2	0	0	1	0	1	0
5.5.1.1	1	1	4	4	0	2	3	0	1	1	5	4	2	1	2	3	4	1	1
5.5.1.2	0	0	0	0	0	0	0	0	0	0	0	0	0	0	0	0	0	0	0
6.2.1.-	0	0	0	0	0	0	0	0	0	0	0	0	0	0	0	0	0	0	0
6.3.5.2	3	17	21	28	11	17	38	2	11	5	21	17	8	13	9	23	17	24	12
K00002	0	4	1	3	0	1	5	0	0	1	2	2	4	1	2	1	2	0	1
K00055	0	0	0	1	0	0	0	0	0	0	1	0	0	0	1	0	0	0	3
K00074	0	9	6	9	1	1	5	0	8	1	7	4	2	5	3	3	6	8	6
K00088	5	21	18	19	9	16	27	3	10	8	16	13	14	13	11	16	10	19	16
K00100	16	44	61	67	28	42	96	12	29	22	62	54	30	50	23	66	51	40	72
K00128	0	2	5	2	0	1	5	0	1	0	5	3	0	2	0	1	3	4	3
K00129	0	0	0	0	0	0	0	0	0	0	0	0	0	0	0	0	0	0	0
K00132	0	0	0	0	0	0	0	0	0	1	2	1	0	1	0	0	0	0	0
K00141	0	0	0	0	0	0	0	0	0	0	0	0	0	0	0	0	0	0	0
K00146	0	0	0	0	0	0	0	0	0	0	0	0	0	0	0	0	0	0	0
K00148	0	0	0	0	0	0	0	0	0	0	0	0	0	0	0	0	0	0	0
K00155	0	0	1	0	0	1	0	0	0	0	1	0	0	0	0	1	0	0	1
K00169	0	1	4	1	0	2	7	0	0	0	3	0	4	2	1	1	1	2	2
K00224	0	0	1	0	1	1	1	0	0	0	0	0	0	1	0	1	1	0	1
K00274	0	0	0	0	0	0	0	0	0	0	0	0	0	0	0	0	0	0	0
K00446	0	0	0	0	0	0	0	0	0	0	0	0	0	0	0	0	0	0	0
K00448	0	0	0	0	0	0	0	0	0	0	0	0	0	0	0	0	0	0	0
K00462	0	1	1	2	0	0	1	0	1	0	1	0	0	0	2	2	0	0	1
K00480	0	0	0	0	0	0	0	0	0	0	0	0	0	0	0	0	0	0	0
K00481	0	0	0	0	0	0	0	0	0	0	0	0	0	0	0	0	0	0	0
K00539	0	1	2	2	0	0	2	0	0	0	1	1	0	3	0	0	0	1	1
K00599	19	48	67	48	22	37	97	14	16	21	30	38	39	53	28	62	22	48	58
K00626	0	2	0	6	0	1	4	2	1	3	8	1	3	3	4	3	2	6	8
K00632	0	0	0	0	0	0	0	0	0	0	1	0	0	0	0	0	0	0	0
K00680	12	43	50	54	19	33	71	11	20	17	45	40	22	34	17	63	34	42	29
K00757	5	9	10	13	8	11	27	2	9	4	10	17	6	12	9	14	11	9	10
K00758	0	8	5	6	0	0	7	1	0	0	4	4	1	5	5	2	2	3	1
K00760	10	26	35	25	12	20	50	5	10	6	15	22	24	22	17	24	19	18	22
K00799	0	0	0	1	0	0	0	0	0	0	2	0	0	0	0	0	2	0	0
K00857	7	22	18	14	9	6	15	3	6	4	7	14	14	10	6	12	10	15	13
K00876	13	43	37	23	15	19	45	7	10	14	21	35	22	23	15	28	19	28	24
K01026	0	0	0	4	0	0	2	0	0	0	6	1	1	0	0	0	0	0	2
K01031	0	0	0	0	0	0	0	0	0	0	0	0	0	0	0	0	0	0	0

EC/KO	DA-AD-8	DA-AD-80	DA-AD-81	DA-AD-82	DA-AD-83	DA-AD-84	DA-AD-85	DA-AD-9	ES-AD-1	ES-AD-10	ES-AD-11	ES-AD-12	ES-AD-13	ES-AD-14	ES-AD-15	ES-AD-16	ES-AD-17	ES-AD-18	ES-AD-19
K01034	0	1	0	3	0	0	2	0	1	1	0	0	2	0	1	0	1	3	0
K01039	0	0	0	1	0	0	0	0	0	1	1	1	0	2	2	0	1	1	0
K01041	2	1	8	6	4	5	5	3	6	3	0	8	3	2	2	3	4	4	2
K01053	0	0	0	1	0	0	0	0	0	0	2	0	0	0	0	0	0	0	0
K01055	0	0	0	0	0	0	0	0	0	0	0	0	0	0	0	0	0	0	0
K01061	0	0	0	0	0	0	1	0	0	0	1	0	0	0	0	0	2	0	0
K01066	4	5	8	10	3	5	15	0	2	3	8	8	1	4	5	11	9	6	3
K01075	0	0	1	2	2	1	1	0	1	0	1	1	0	0	0	1	1	1	1
K01077	1	6	8	12	4	19	11	1	6	4	4	6	2	9	6	9	9	6	4
K01101	1	9	4	7	2	0	7	0	0	1	7	3	6	1	4	6	4	4	6
K01195	1	9	6	10	2	12	13	1	1	4	9	8	5	8	9	16	8	5	3
K01426	0	1	0	0	0	1	2	0	0	0	4	0	0	1	0	0	1	0	2
K01428	0	0	1	0	0	1	2	0	0	0	1	0	0	1	0	1	0	0	0
K01457	0	3	0	0	0	0	0	0	0	0	0	0	0	0	0	1	0	0	0
K01464	1	2	0	4	1	3	4	0	0	1	9	1	1	3	0	0	3	0	6
K01489	5	30	24	23	8	12	29	6	5	6	15	22	21	20	12	19	19	17	11
K01500	0	0	0	0	0	0	0	0	0	0	0	0	0	0	0	0	0	0	0
K01501	0	4	0	6	2	1	4	0	0	2	5	5	4	1	2	0	1	2	3
K01502	0	0	0	0	0	0	1	0	0	0	0	0	0	0	0	0	0	0	0
K01512	3	7	12	9	3	2	25	2	1	3	3	2	5	12	7	11	6	4	12
K01560	0	6	2	8	2	2	14	0	3	1	5	6	5	7	3	5	10	3	4
K01561	0	0	1	0	0	0	0	0	0	0	0	0	0	1	0	2	0	1	1
K01563	0	0	0	0	0	0	0	0	0	0	0	0	0	0	0	0	0	0	0
K01564	0	1	1	3	0	1	1	0	0	3	1	1	1	0	1	4	2	2	2
K01607	6	13	12	13	7	9	14	3	16	4	12	17	3	8	6	13	9	11	7
K01612	0	0	0	0	0	0	0	0	0	0	0	0	0	0	0	0	0	0	0
K01615	5	12	16	15	5	9	20	1	8	2	16	7	12	11	12	11	12	7	12
K01617	0	0	0	0	0	0	0	0	0	0	0	0	0	0	0	0	0	0	0
K01666	4	8	7	11	2	5	11	1	0	1	3	1	2	8	1	5	2	4	6
K01692	0	0	0	1	0	0	1	0	0	0	1	1	0	0	0	0	0	0	0
K01721	0	0	0	0	0	0	0	0	0	0	0	0	0	0	0	0	0	0	0
K01726	3	3	9	6	6	9	12	3	5	6	4	9	6	8	1	5	4	6	7
K01781	0	0	0	0	0	2	0	0	0	1	2	0	0	0	0	0	0	0	2
K01821	0	1	2	2	0	1	4	0	2	2	1	2	1	2	1	2	2	3	9
K01856	0	0	0	0	0	0	0	0	0	0	1	0	0	0	0	0	0	0	0
K01857	0	0	1	0	0	0	0	0	0	0	0	0	0	0	0	1	0	0	1
K01913	0	0	1	1	1	0	1	0	0	0	0	0	0	0	0	0	1	0	1
K01951	2	16	12	14	8	11	19	2	7	4	19	13	11	9	9	14	10	18	11
K02554	0	0	0	0	0	0	0	0	0	0	0	0	0	0	0	0	0	0	0
K03381	0	0	0	0	0	0	0	0	0	0	0	0	0	0	0	0	0	0	0
K03382	0	0	0	1	0	1	0	0	0	1	2	1	1	0	0	0	2	0	0
K03464	0	0	0	0	0	0	0	0	0	0	0	0	0	0	0	0	0	0	0
K03518	4	8	4	11	3	6	27	0	6	4	22	12	9	12	4	9	11	3	14
K03862	0	0	0	0	0	0	0	0	0	0	0	0	0	0	0	0	0	0	0
K04099	0	0	0	0	0	0	0	0	0	0	0	0	0	0	0	0	0	0	0
K04100	0	0	0	0	0	0	0	0	0	0	0	0	0	0	0	0	0	0	0
K04102	0	0	0	0	0	0	0	0	0	0	0	0	0	0	0	0	0	0	0
K04116	0	0	0	0	0	0	0	0	0	0	0	0	0	0	0	0	0	0	0
K05394	0	0	0	0	0	1	2	0	0	0	0	0	0	1	1	1	2	0	1

EC/KO	DA-AD-8	DA-AD-80	DA-AD-81	DA-AD-82	DA-AD-83	DA-AD-84	DA-AD-85	DA-AD-9	ES-AD-1	ES-AD-10	ES-AD-11	ES-AD-12	ES-AD-13	ES-AD-14	ES-AD-15	ES-AD-16	ES-AD-17	ES-AD-18	ES-AD-19
**K05549**	0	0	0	0	0	0	0	0	0	0	0	0	0	0	0	0	0	0	0
**K05783**	0	0	0	0	0	0	0	0	0	0	0	0	0	0	0	0	0	0	0
**K05797**	0	0	0	0	0	0	0	0	0	0	0	0	0	0	0	0	0	0	0
**K06281**	0	0	0	2	0	0	2	0	1	0	1	0	0	2	0	1	2	1	3
**K06446**	2	7	2	5	4	6	7	1	5	5	5	4	3	1	2	5	2	4	8
**K06912**	0	0	0	0	0	0	0	0	0	0	0	0	0	0	0	0	0	0	0
**K07535**	0	0	0	0	0	0	0	0	0	0	0	0	0	0	0	0	0	0	0
**K07536**	0	2	2	3	1	1	0	0	2	1	2	4	2	2	2	0	4	2	2
**K08689**	0	0	0	0	0	0	0	0	0	0	0	0	0	0	0	0	0	0	0
**K08710**	0	0	0	0	0	0	0	0	0	0	1	0	0	0	0	0	0	0	1
**K09461**	0	0	0	0	0	0	0	0	0	0	0	0	0	0	0	0	0	0	0
**K10217**	0	0	0	0	0	0	0	0	0	0	0	0	0	0	0	0	0	0	0
**K10218**	0	0	0	2	0	1	0	0	0	0	3	1	1	0	0	1	0	0	0
**K10220**	0	0	0	0	0	0	0	0	0	0	0	0	0	0	0	0	0	0	0
**K11180**	0	0	0	0	0	0	0	0	0	0	0	0	0	0	0	1	1	0	1
**K13953**	0	0	0	0	0	0	2	0	0	0	1	1	1	1	0	0	2	0	4
**K14333**	0	0	0	0	0	0	0	0	0	0	0	0	0	0	0	0	0	0	0
**K14519**	0	0	0	0	0	0	0	0	0	0	0	0	0	0	0	0	0	0	0
**K15054**	0	0	0	0	0	0	0	0	0	0	1	0	0	0	0	0	0	0	0
**K16173**	0	0	0	1	0	0	2	0	0	0	1	0	1	0	0	0	1	0	2
**K16514**	0	0	0	0	0	0	0	0	0	0	0	0	0	0	1	0	0	0	1
**K16874**	1	1	0	0	0	0	0	0	0	0	0	0	0	0	0	0	0	0	0

EC/KO	ES-AD-2	ES-AD-20	ES-AD-21	ES-AD-22	ES-AD-23	ES-AD-24	ES-AD-25	ES-AD-26	ES-AD-27	ES-AD-28	ES-AD-29	ES-AD-3	ES-AD-30	ES-AD-31	ES-AD-32	ES-AD-33	ES-AD-34	ES-AD-35	ES-AD-36
1.1.1.-	38	39	50	71	42	50	71	44	63	60	75	42	52	60	75	42	67	57	59
1.1.1.1	17	28	39	53	31	27	33	32	38	41	31	12	43	43	57	42	43	46	39
1.1.1.157	0	3	0	2	1	0	2	0	1	2	1	2	1	2	2	1	1	0	0
1.1.1.205	5	16	21	32	17	19	21	16	27	24	22	15	17	25	31	20	20	28	16
1.1.1.35	1	4	2	7	4	2	2	2	6	3	6	3	4	8	6	1	5	4	3
1.12.99.6	0	1	0	2	2	0	0	2	2	2	1	1	2	1	3	1	3	4	0
1.13.11.-	0	0	0	0	0	0	0	0	0	0	0	0	0	0	0	0	0	0	0
1.13.11.1	0	0	0	0	0	0	0	0	0	0	0	0	0	0	0	0	0	0	0
1.13.11.2	0	0	0	0	0	0	0	0	0	0	0	0	0	0	0	0	0	0	0
1.13.11.3	0	0	0	0	0	0	0	0	0	0	0	0	0	0	0	0	0	0	0
1.13.11.39	0	0	0	0	0	0	0	0	0	0	0	0	0	0	0	0	0	0	0
1.13.11.5	0	0	0	0	0	0	0	0	0	0	0	0	0	0	0	0	0	0	0
1.13.11.8	0	0	0	0	0	0	0	0	0	0	0	0	0	0	0	0	0	0	0
1.14.12.10	0	0	0	0	0	0	0	0	0	0	0	0	0	0	0	0	0	0	0
1.14.12.13	0	0	0	0	0	0	0	0	0	0	0	0	0	0	0	0	0	0	0
1.14.12.18	0	0	0	0	0	0	0	0	0	0	0	0	0	0	0	0	0	0	0
1.14.13.-	1	0	1	0	0	0	2	1	0	1	0	0	0	1	0	0	0	0	3
1.14.13.1	0	0	0	0	0	0	0	0	0	0	0	0	0	0	0	0	0	0	0
1.14.13.2	0	0	0	0	0	0	0	0	0	0	0	0	0	0	0	0	0	0	0
1.14.13.50	0	0	0	0	0	0	0	0	0	0	0	0	0	0	0	0	0	0	0
1.14.13.7	0	0	0	0	0	0	0	0	0	0	0	0	0	0	0	0	0	1	0
1.14.13.8	0	0	0	0	0	0	0	0	0	0	0	0	0	0	0	0	0	0	0
1.14.13.82	0	0	0	0	0	0	0	0	0	0	0	0	0	0	0	0	0	0	1
1.14.99.-	0	0	0	0	0	0	0	0	0	0	0	0	0	0	0	0	0	0	0
1.17.99.1	0	0	0	0	0	0	0	0	0	0	0	0	0	0	0	0	0	0	0
1.18.6.1	0	0	0	2	0	0	1	0	0	2	0	0	0	0	0	1	0	0	1
1.2.1.-	0	0	0	0	0	0	0	0	0	0	0	0	0	0	0	0	0	0	1
1.2.1.10	3	9	5	7	4	3	4	9	8	11	2	1	7	3	7	3	1	4	3
1.2.1.3	6	6	8	12	5	3	10	9	11	13	8	1	11	8	14	1	16	11	9
1.2.1.39	0	0	0	0	0	0	0	0	0	0	0	0	0	0	0	0	0	0	0
1.2.7.1	7	5	15	28	2	7	14	6	16	16	15	15	9	18	13	9	7	17	8
1.2.99.2	0	5	4	7	1	1	2	3	8	3	7	0	5	2	4	1	2	4	3
1.3.1.-	0	0	0	0	1	0	0	0	0	0	0	1	0	0	1	0	0	0	0
1.3.1.2	0	1	0	1	1	0	0	0	0	1	1	0	0	1	1	2	0	1	0
1.3.1.25	0	0	0	0	0	0	0	0	0	0	0	0	0	0	0	0	0	0	0
1.3.99.-	0	0	0	0	0	0	0	0	1	1	0	0	0	0	1	0	0	0	0
1.6.5.-	29	22	41	56	28	31	45	44	56	47	56	42	44	47	59	42	44	60	53
1.7.1.-	8	5	9	13	6	8	11	6	11	10	12	8	9	13	10	9	9	9	8
1.8.99.3	0	1	0	0	0	0	0	0	0	0	0	0	0	0	0	1	0	1	0
2.1.1.-	160	147	334	388	157	233	303	222	372	303	361	139	266	312	349	255	268	388	261
2.3.1.-	81	120	177	216	74	136	169	131	212	188	207	87	201	151	180	140	159	193	183
2.3.1.16	0	1	2	1	1	3	5	0	1	2	0	0	2	2	1	4	1	3	2
2.3.1.5	0	0	0	0	0	0	0	0	0	0	0	0	0	0	0	0	0	0	1
2.3.1.9	0	0	0	1	1	0	0	0	1	3	1	0	1	0	0	1	1	1	2
2.4.2.10	10	9	20	21	6	13	15	13	20	19	22	10	17	17	18	17	15	15	15
2.4.2.3	6	14	15	17	5	9	9	5	11	6	16	7	9	10	16	12	11	12	12
2.4.2.4	4	2	5	4	1	2	6	4	11	3	10	0	3	6	3	5	2	5	6
2.4.2.8	14	10	17	18	11	7	19	6	18	14	15	11	17	15	16	12	14	18	16
2.5.1.-	17	10	19	38	15	19	16	17	29	21	32	8	24	21	21	13	24	28	19

EC/KO	ES-AD-2	ES-AD-20	ES-AD-21	ES-AD-22	ES-AD-23	ES-AD-24	ES-AD-25	ES-AD-26	ES-AD-27	ES-AD-28	ES-AD-29	ES-AD-3	ES-AD-30	ES-AD-31	ES-AD-32	ES-AD-33	ES-AD-34	ES-AD-35	ES-AD-36
2.5.1.18	0	3	4	1	0	0	1	0	1	2	0	1	0	3	2	2	4	1	10
2.6.1.-	9	18	33	44	18	21	38	32	48	37	53	15	39	31	37	47	26	44	28
2.7.1.21	6	5	7	11	1	8	6	6	11	4	10	4	8	11	7	8	4	9	11
2.7.1.48	23	20	34	40	16	24	41	21	37	28	33	18	29	31	33	23	36	31	32
2.7.4.-	5	7	18	28	11	14	20	18	27	16	23	8	19	23	18	16	15	23	15
2.8.3.-	1	0	1	3	2	0	1	1	1	4	0	1	2	1	1	2	2	3	1
2.8.3.1	0	0	0	1	2	0	0	0	0	0	0	1	1	0	0	0	1	0	0
2.8.3.12	2	0	1	1	1	0	1	0	1	1	0	1	0	0	1	0	1	0	0
2.8.3.6	1	1	0	2	1	0	0	2	0	2	0	0	1	1	0	1	0	1	0
2.8.3.8	1	2	1	3	2	0	2	0	3	8	1	3	3	2	0	3	3	3	0
3.1.1.-	2	2	5	4	5	3	5	8	6	7	4	5	4	3	6	4	6	5	5
3.1.1.1	2	7	5	3	7	9	9	5	9	7	11	5	16	7	11	6	14	8	3
3.1.1.17	0	1	0	4	1	3	2	2	1	4	2	2	1	0	1	1	3	3	4
3.1.1.2	0	0	0	0	0	0	0	0	0	0	1	0	0	0	0	1	0	0	1
3.1.1.24	0	1	4	6	1	3	4	4	5	4	3	2	4	3	6	5	2	0	4
3.1.1.45	0	0	0	0	0	0	0	0	0	0	0	0	0	0	0	0	0	0	1
3.1.2.-	1	0	0	0	1	1	0	0	0	0	0	0	1	0	2	0	2	0	4
3.1.2.23	2	5	5	8	2	6	3	6	9	9	5	5	6	4	5	5	5	4	5
3.1.3.1	8	9	11	14	13	9	12	7	14	17	16	11	23	15	21	13	26	26	10
3.1.3.2	4	2	4	5	4	4	5	3	4	5	5	4	9	6	1	4	3	6	6
3.1.3.41	0	0	0	0	1	1	0	0	0	0	0	0	2	1	2	0	2	0	0
3.2.1.31	5	0	8	5	3	2	1	1	5	2	5	7	4	8	4	3	3	3	4
3.3.2.9	0	0	0	0	0	0	0	0	0	0	0	0	0	0	0	0	0	0	1
3.5.1.-	11	21	36	34	16	18	34	17	33	18	43	9	22	37	32	22	22	27	25
3.5.1.4	2	2	2	4	1	3	4	1	5	3	1	2	3	1	1	5	4	4	0
3.5.1.5	0	2	0	2	0	1	3	1	1	5	4	2	0	1	2	1	3	2	3
3.5.1.54	3	1	6	6	4	2	7	2	6	3	4	3	3	5	8	4	1	8	4
3.5.1.6	1	1	3	4	0	0	0	0	0	2	0	2	1	2	1	1	0	0	1
3.5.2.-	0	0	0	0	0	0	0	0	0	0	0	0	0	0	1	0	1	0	0
3.5.2.2	0	2	0	2	2	1	4	1	3	1	2	1	1	1	1	2	1	1	1
3.5.4.-	10	13	27	39	12	21	29	20	37	28	33	17	27	30	28	25	30	33	30
3.5.4.5	8	8	14	19	7	11	23	8	22	14	18	11	10	12	15	10	10	16	13
3.5.5.1	0	0	1	4	0	1	2	1	3	3	1	0	1	1	0	2	2	1	1
3.5.5.7	0	0	0	0	0	0	0	0	0	0	0	0	0	0	0	0	0	0	0
3.5.99.3	0	1	1	0	0	0	1	0	0	0	0	0	0	0	0	0	0	0	0
3.6.1.7	2	3	3	7	6	1	4	6	4	4	7	5	1	7	9	4	5	9	5
3.7.1.-	2	0	3	2	1	1	3	0	2	5	2	1	2	2	2	1	4	2	0
3.7.1.2	1	5	5	2	4	6	4	5	2	6	5	2	3	2	3	0	5	7	3
3.8.1.2	1	4	5	7	4	5	9	6	8	9	10	4	11	5	5	6	5	8	6
3.8.1.3	0	0	0	0	0	0	0	0	0	0	0	0	0	1	0	0	0	0	0
3.8.1.5	0	0	0	0	2	0	2	0	2	2	3	0	2	1	4	0	1	0	1
4.1.1.-	4	8	12	19	8	5	14	9	13	16	6	6	12	12	12	10	12	12	8
4.1.1.44	35	11	22	29	14	23	28	30	34	35	25	48	30	27	33	18	18	35	19
4.1.1.55	0	0	0	0	0	0	0	0	0	1	0	0	0	0	0	0	0	0	0
4.1.1.7	0	0	0	0	0	0	0	0	0	0	0	0	0	0	0	0	0	0	0
4.1.1.70	0	0	1	0	0	0	0	0	0	0	0	1	1	0	0	1	0	1	1
4.1.1.77	0	0	0	0	0	0	0	0	0	0	0	0	0	0	0	0	0	0	0
4.1.2.-	2	2	7	9	4	1	7	3	8	6	6	3	10	4	9	1	4	3	5
4.1.3.-	9	7	29	28	11	15	23	19	26	18	26	16	20	20	21	17	20	23	20

EC/KO	ES-AD-2	ES-AD-20	ES-AD-21	ES-AD-22	ES-AD-23	ES-AD-24	ES-AD-25	ES-AD-26	ES-AD-27	ES-AD-28	ES-AD-29	ES-AD-3	ES-AD-30	ES-AD-31	ES-AD-32	ES-AD-33	ES-AD-34	ES-AD-35	ES-AD-36
4.1.3.39	0	0	0	1	1	0	1	0	1	0	0	0	0	0	0	0	0	0	0
4.1.99.-	0	3	3	2	2	2	2	0	3	2	5	0	0	3	2	3	1	0	1
4.2.1.-	19	39	60	65	32	36	48	43	58	59	70	31	55	40	70	40	59	55	44
4.2.1.17	4	3	5	5	6	8	7	2	3	9	7	4	3	4	13	6	4	7	7
4.2.1.80	0	0	0	0	0	0	0	0	0	0	0	0	0	0	0	0	0	0	0
4.2.1.83	0	0	2	0	0	0	2	1	1	0	1	0	0	0	0	1	1	1	1
4.2.1.84	0	0	0	1	0	0	0	0	0	0	0	0	0	0	0	0	1	0	1
5.1.2.2	0	1	0	0	0	0	0	0	0	0	0	0	0	0	0	0	0	0	0
5.2.1.2	0	0	0	0	0	0	0	0	0	0	0	0	0	0	0	0	0	0	0
5.3.3.4	0	0	0	0	0	0	0	0	0	0	0	0	0	0	0	0	0	0	0
5.3.99.-	1	3	2	5	2	5	1	3	2	2	5	1	6	2	2	2	2	3	2
5.4.99.-	1	1	0	1	0	1	1	0	0	1	1	0	1	1	0	0	0	0	1
5.5.1.1	2	2	3	1	2	1	5	5	4	4	5	2	4	2	2	4	3	2	1
5.5.1.2	0	0	0	0	0	0	0	0	0	0	0	0	0	0	0	0	0	0	0
6.2.1.-	0	0	0	0	0	0	0	0	0	0	0	0	0	0	0	0	0	0	0
6.3.5.2	6	13	15	20	18	15	25	17	21	36	21	12	26	16	21	18	19	26	18
K00002	1	1	1	3	1	2	3	1	3	5	1	0	1	1	5	1	3	5	1
K00055	0	1	0	0	0	0	0	0	0	0	0	0	0	0	0	0	0	0	0
K00074	6	7	2	10	6	3	5	3	8	8	9	11	6	12	8	3	7	5	3
K00088	6	15	15	27	9	18	13	12	24	22	17	8	16	19	25	19	18	21	14
K00100	23	37	45	72	44	35	56	31	61	57	68	37	62	55	73	45	64	79	60
K00128	0	2	2	2	0	0	1	2	5	4	3	0	3	3	2	1	2	4	2
K00129	0	0	0	0	0	0	0	0	0	0	0	0	0	0	0	0	0	0	0
K00132	0	0	0	1	0	0	1	0	0	0	2	0	0	0	0	0	0	0	0
K00141	0	0	0	0	0	0	0	0	0	0	0	0	0	0	0	0	0	0	0
K00146	0	0	0	0	0	0	0	0	0	0	0	0	0	0	0	0	0	0	0
K00148	0	0	0	0	0	0	0	0	0	0	0	0	0	0	0	0	0	0	1
K00155	1	0	0	0	1	0	0	0	0	0	0	0	0	0	0	0	1	1	1
K00169	0	0	4	4	1	2	3	1	3	2	2	0	0	5	2	1	1	4	2
K00224	0	0	1	1	1	1	0	0	1	1	0	0	0	1	1	0	1	1	0
K00274	0	0	0	0	0	0	0	0	0	0	0	0	0	0	0	0	0	0	0
K00446	0	0	0	0	0	0	0	0	0	0	0	0	0	0	0	0	0	0	1
K00448	0	0	0	0	0	0	0	0	0	0	0	0	0	0	0	0	0	0	0
K00462	0	1	1	1	0	1	1	1	1	0	1	1	0	0	1	0	1	2	1
K00480	0	0	0	0	0	0	0	0	0	0	0	0	0	0	0	0	0	0	0
K00481	0	0	0	0	0	0	0	0	0	0	0	0	0	0	0	0	0	0	0
K00539	0	0	1	1	0	0	0	2	1	2	2	1	0	0	1	0	3	4	0
K00599	20	41	59	64	25	43	48	31	47	38	69	14	47	57	60	46	53	54	44
K00626	0	3	3	5	1	3	5	0	3	9	0	1	3	4	3	8	4	4	4
K00632	0	0	0	0	0	0	0	0	0	0	0	0	0	0	0	0	0	0	0
K00680	24	33	47	66	28	32	36	36	56	61	55	30	51	35	54	33	47	51	28
K00757	9	10	13	15	7	9	9	11	14	10	13	8	10	12	13	10	14	12	12
K00758	2	0	6	5	0	3	8	4	7	7	4	2	6	5	4	3	2	7	5
K00760	16	13	27	34	15	17	29	14	34	22	30	16	22	26	28	20	24	29	29
K00799	0	2	0	2	0	0	1	1	1	1	0	0	0	0	0	0	1	1	3
K00857	8	8	14	19	3	10	13	15	18	6	13	5	12	18	11	11	6	16	15
K00876	17	16	32	33	15	24	30	23	34	28	32	12	27	32	29	25	24	42	31
K01026	2	1	1	2	0	0	2	0	1	0	0	1	1	2	0	2	1	0	0
K01031	0	0	0	0	0	0	0	0	0	0	0	0	0	0	0	0	0	0	0

EC/KO	ES-AD-2	ES-AD-20	ES-AD-21	ES-AD-22	ES-AD-23	ES-AD-24	ES-AD-25	ES-AD-26	ES-AD-27	ES-AD-28	ES-AD-29	ES-AD-3	ES-AD-30	ES-AD-31	ES-AD-32	ES-AD-33	ES-AD-34	ES-AD-35	ES-AD-36
K01034	2	3	3	2	1	1	3	2	2	5	2	0	2	1	1	3	2	2	1
K01039	0	1	0	0	1	0	0	0	0	1	1	1	0	0	0	1	0	0	0
K01041	3	1	6	9	1	5	1	5	7	5	1	3	5	8	5	8	9	11	3
K01053	0	0	0	0	0	0	0	0	1	0	0	0	0	0	0	0	0	0	0
K01055	0	0	0	0	0	0	0	0	0	0	0	0	0	0	0	0	0	0	0
K01061	0	0	0	0	0	0	0	0	0	0	0	0	1	0	0	1	0	0	1
K01066	1	4	8	8	3	7	7	7	15	5	5	7	14	10	12	5	12	10	4
K01075	0	0	1	2	1	0	1	2	2	2	0	0	0	0	1	1	2	2	2
K01077	1	4	8	8	9	6	4	5	7	11	8	2	13	3	16	6	13	9	6
K01101	0	1	2	7	3	6	4	3	9	5	1	2	9	3	4	6	4	2	3
K01195	2	4	10	13	4	5	9	4	12	5	6	3	5	10	10	7	10	8	4
K01426	0	1	0	1	0	0	2	2	1	0	1	0	0	1	0	1	0	2	0
K01428	0	0	0	2	0	1	1	1	0	1	0	0	0	0	2	0	0	0	2
K01457	0	0	0	1	0	0	0	0	1	0	0	0	0	0	0	0	0	0	0
K01464	1	3	0	5	3	1	5	1	3	5	3	2	2	2	1	2	1	2	2
K01489	14	13	21	33	10	16	27	11	31	20	22	14	15	20	20	17	17	26	21
K01500	0	0	0	0	0	0	0	0	0	0	0	0	0	0	0	0	0	0	0
K01501	0	2	3	3	0	2	3	2	5	5	3	1	2	3	4	2	3	3	1
K01502	0	0	0	0	0	0	0	0	0	0	0	0	0	0	0	0	0	0	0
K01512	4	6	10	12	8	3	8	9	11	8	9	5	3	10	14	8	10	13	5
K01560	6	2	5	6	5	1	6	5	13	4	6	2	10	13	12	4	9	7	8
K01561	0	0	0	0	0	0	0	0	1	0	0	0	1	0	0	0	0	0	0
K01563	0	0	0	0	0	0	0	0	0	0	0	0	0	0	0	0	0	0	0
K01564	0	1	1	2	0	1	0	0	1	2	1	0	2	0	1	1	1	4	1
K01607	16	5	11	7	5	9	15	13	17	20	11	26	10	8	18	7	6	14	8
K01612	0	0	0	1	0	0	0	0	1	1	0	0	0	0	0	0	0	0	0
K01615	7	6	14	20	6	7	14	9	19	20	20	3	14	12	18	14	12	19	16
K01617	0	0	0	0	0	0	0	0	0	0	0	0	0	0	0	0	0	0	0
K01666	1	3	12	12	5	7	7	6	8	6	8	1	2	4	8	5	1	7	7
K01692	0	0	0	0	0	0	0	0	1	1	1	0	1	0	0	0	1	2	0
K01721	0	0	0	0	0	0	0	0	0	0	0	0	0	0	0	0	0	0	0
K01726	1	12	7	6	4	7	7	7	9	7	11	4	9	9	9	7	9	10	7
K01781	0	1	0	0	0	0	2	0	0	0	0	0	2	0	0	0	0	0	1
K01821	1	4	0	3	1	1	2	2	2	3	1	2	0	0	3	1	1	3	2
K01856	0	0	0	0	0	0	0	0	0	0	0	0	0	0	0	0	0	0	0
K01857	0	0	0	0	0	0	0	0	0	0	0	0	0	0	1	0	1	0	0
K01913	0	0	1	0	0	0	0	0	1	0	1	0	1	1	0	0	0	1	0
K01951	6	8	9	16	10	13	15	10	17	22	13	7	14	15	13	17	15	19	13
K02554	0	0	0	0	0	0	0	0	0	0	0	0	0	0	0	0	0	0	0
K03381	0	0	0	0	0	0	0	0	0	0	0	0	0	0	0	0	0	0	0
K03382	0	1	1	1	0	0	1	2	2	0	0	0	1	0	1	0	1	1	1
K03464	0	0	0	0	0	0	0	0	0	0	0	0	0	0	0	0	0	0	0
K03518	13	10	12	15	11	2	15	8	18	24	12	9	8	11	14	11	6	15	11
K03862	0	0	0	0	0	0	0	0	0	0	0	0	0	0	0	0	0	0	0
K04099	0	0	0	0	0	0	0	0	0	0	0	0	0	0	0	0	0	0	0
K04100	0	0	0	0	0	0	0	0	0	0	0	0	0	0	0	0	0	0	0
K04102	0	0	0	0	0	0	0	0	0	1	0	0	0	0	0	0	0	0	0
K04116	0	0	0	0	0	0	0	0	0	0	0	0	0	0	0	0	0	0	0
K05394	1	0	0	2	2	1	0	2	0	1	1	0	2	0	1	1	2	1	1

EC/KO	ES-AD-2	ES-AD-20	ES-AD-21	ES-AD-22	ES-AD-23	ES-AD-24	ES-AD-25	ES-AD-26	ES-AD-27	ES-AD-28	ES-AD-29	ES-AD-3	ES-AD-30	ES-AD-31	ES-AD-32	ES-AD-33	ES-AD-34	ES-AD-35	ES-AD-36
**K05549**	0	0	0	0	0	0	0	0	0	0	0	0	0	0	0	0	0	0	0
**K05783**	0	0	0	0	0	0	0	0	0	0	0	0	0	0	0	0	0	0	0
**K05797**	0	0	0	0	0	0	0	0	0	0	0	0	0	0	0	0	0	0	0
**K06281**	1	1	0	2	2	0	0	2	2	2	1	0	1	0	3	1	1	3	0
**K06446**	8	5	3	6	6	4	4	5	4	8	1	9	6	5	6	7	3	6	4
**K06912**	0	0	0	0	0	0	0	0	0	0	0	0	0	0	0	0	0	0	0
**K07535**	0	0	0	0	0	0	0	0	1	0	0	0	0	1	0	0	1	0	0
**K07536**	5	0	2	4	1	2	1	1	3	3	3	3	3	3	2	3	2	4	1
**K08689**	0	0	0	0	0	0	0	0	0	0	0	0	0	0	0	0	0	0	0
**K08710**	0	0	0	0	0	0	0	0	0	0	0	0	0	0	0	0	0	0	0
**K09461**	0	0	0	0	1	0	0	0	0	0	0	0	2	0	1	0	3	0	0
**K10217**	0	0	0	0	0	0	0	0	0	0	0	0	0	0	0	0	0	0	0
**K10218**	0	1	1	0	0	0	0	0	0	0	1	1	0	0	1	0	0	1	0
**K10220**	0	0	0	0	0	0	0	0	0	0	0	0	0	0	0	0	0	0	0
**K11180**	0	0	0	0	0	0	0	1	0	1	0	0	0	0	0	0	0	0	0
**K13953**	0	0	0	0	0	0	0	0	0	0	0	0	1	0	0	1	1	0	2
**K14333**	0	0	0	0	0	0	0	0	0	0	0	0	0	0	0	0	0	0	0
**K14519**	0	0	0	0	0	0	0	0	0	0	0	0	0	0	0	0	0	0	0
**K15054**	0	0	0	0	0	0	0	0	0	0	0	0	0	0	0	0	0	0	0
**K16173**	0	0	0	1	2	0	2	0	0	2	0	0	0	0	0	0	3	1	0
**K16514**	0	2	0	0	0	0	0	0	0	1	0	0	0	1	0	0	0	0	0
**K16874**	0	1	0	0	1	0	0	0	0	0	0	0	0	0	0	0	0	0	0

EC/KO	ES-AD-37	ES-AD-38	ES-AD-39	ES-AD-4	ES-AD-5	ES-AD-6	ES-AD-7	ES-AD-8	ES-AD-9	FR-AD-1	FR-AD-2	FR-AD-3	FR-AD-4	FR-AD-5	FR-AD-6	FR-AD-7	FR-AD-8	IN-1660SH	IN-1660SK	IN-199SD
1.1.1.-	49	81	61	43	37	37	45	13	13	41	25	37	40	32	41	31	32	16	17	24
1.1.1.1	29	51	48	22	31	14	34	14	12	27	12	9	14	25	17	20	11	10	11	10
1.1.1.157	0	1	0	0	0	0	0	0	0	1	0	0	3	1	1	0	0	0	3	0
1.1.1.205	17	24	26	21	12	13	25	8	7	11	9	7	7	8	7	11	7	14	9	19
1.1.1.35	0	4	5	4	2	2	4	1	1	4	0	0	2	1	5	0	2	0	1	0
1.12.99.6	2	3	1	2	0	0	1	1	0	0	1	1	0	0	0	1	1	0	0	0
1.13.11.-	0	0	0	0	0	0	0	0	0	0	0	0	0	0	0	0	0	0	0	0
1.13.11.1	0	0	0	0	0	0	0	0	0	0	0	0	0	0	0	0	0	0	0	0
1.13.11.2	0	0	0	0	0	0	0	0	0	0	0	0	0	0	0	0	0	0	0	0
1.13.11.3	0	0	0	0	0	0	0	0	0	0	0	0	0	0	0	0	0	0	0	0
1.13.11.39	0	0	0	0	0	0	0	0	0	0	0	0	0	0	0	0	0	0	0	0
1.13.11.5	0	0	0	0	0	0	0	0	0	0	0	0	0	0	0	0	0	0	0	0
1.13.11.8	0	0	0	0	0	0	0	0	0	0	0	0	0	0	0	0	0	0	0	0
1.14.12.10	0	0	0	0	0	1	0	0	0	0	0	0	0	0	0	0	0	0	0	0
1.14.12.13	0	0	0	0	0	0	0	0	0	0	0	0	0	0	0	0	0	0	0	0
1.14.12.18	0	0	0	0	0	0	0	0	0	0	0	0	0	0	0	0	0	0	0	0
1.14.13.-	1	1	1	0	0	0	0	0	0	0	0	0	0	0	0	0	0	0	0	0
1.14.13.1	0	0	0	0	0	0	0	0	0	0	0	0	0	0	0	0	0	0	0	0
1.14.13.2	0	0	0	0	0	0	0	0	0	0	0	0	0	0	0	0	0	0	0	0
1.14.13.50	0	0	0	0	0	0	0	0	0	0	0	0	0	0	0	0	0	0	0	0
1.14.13.7	0	0	0	0	0	0	0	0	0	0	0	0	0	0	0	0	0	0	0	1
1.14.13.8	0	0	0	0	0	0	0	0	0	0	0	0	0	0	0	0	0	0	0	0
1.14.13.82	0	0	0	0	0	0	0	0	0	0	0	0	0	0	0	0	0	0	0	0
1.14.99.-	0	0	0	0	0	0	0	0	0	0	0	0	0	0	0	0	0	0	0	0
1.17.99.1	0	0	0	0	0	0	0	0	0	0	0	0	0	0	0	0	0	0	0	0
1.18.6.1	0	0	0	2	0	1	1	0	0	1	0	0	0	0	0	1	0	0	0	0
1.2.1.-	0	0	0	0	0	0	0	0	0	0	0	0	0	0	0	0	0	0	0	0
1.2.1.10	1	5	6	3	1	1	4	1	2	2	0	1	3	3	2	3	2	0	0	1
1.2.1.3	3	13	7	6	5	6	9	1	2	2	3	2	8	5	3	2	1	0	3	0
1.2.1.39	0	0	0	0	0	0	0	0	0	1	0	0	0	0	1	0	0	0	0	0
1.2.7.1	7	16	24	10	5	5	16	6	8	10	7	7	8	8	6	7	10	5	10	7
1.2.99.2	2	2	3	3	3	5	6	0	2	7	1	2	1	1	2	2	2	0	1	0
1.3.1.-	0	0	0	0	0	0	1	1	0	0	0	0	0	0	0	0	0	0	0	0
1.3.1.2	0	0	0	0	0	1	2	0	1	0	0	1	0	0	0	0	0	0	0	0
1.3.1.25	0	0	0	0	0	0	0	0	0	0	0	0	0	0	0	0	0	0	0	0
1.3.99.-	0	0	1	0	0	0	0	0	0	0	0	0	0	0	0	0	0	0	0	0
1.6.5.-	34	64	50	51	18	32	58	14	12	44	31	24	30	25	39	35	24	30	41	40
1.7.1.-	7	11	13	7	6	9	11	6	6	8	7	8	7	8	3	11	1	8	10	14
1.8.99.3	0	0	0	1	0	0	0	0	0	0	2	0	0	0	0	0	0	0	1	0
2.1.1.-	170	433	353	214	161	193	297	96	103	160	131	114	161	138	104	142	132	89	130	102
2.3.1.-	99	167	164	128	61	80	149	56	32	91	80	89	91	74	65	87	77	32	49	46
2.3.1.16	0	2	1	0	2	1	2	3	2	0	0	0	1	0	0	0	0	0	1	0
2.3.1.5	0	0	0	0	0	0	0	0	0	0	0	0	0	0	0	0	0	0	0	0
2.3.1.9	1	1	1	0	1	1	1	0	0	1	0	0	0	3	1	1	0	0	0	0
2.4.2.10	11	22	20	13	10	13	20	8	5	11	7	7	10	12	11	6	14	4	8	6
2.4.2.3	6	16	12	9	9	8	10	3	4	9	4	3	9	3	4	7	7	2	4	2
2.4.2.4	0	8	5	1	2	2	5	2	2	1	1	0	0	2	0	0	0	0	1	0
2.4.2.8	10	17	15	12	13	13	15	7	5	6	6	2	8	6	11	8	7	10	6	9
2.5.1.-	17	32	20	16	13	18	27	8	8	14	8	12	13	14	7	19	7	18	14	23

EC/KO	ES-AD-37	ES-AD-38	ES-AD-39	ES-AD-4	ES-AD-5	ES-AD-6	ES-AD-7	ES-AD-8	ES-AD-9	FR-AD-1	FR-AD-2	FR-AD-3	FR-AD-4	FR-AD-5	FR-AD-6	FR-AD-7	FR-AD-8	IN-1660SH	IN-1660SK	IN-199SD
**2.5.1.18**	0	4	2	0	1	2	0	0	0	1	0	0	0	0	1	1	1	0	0	0
**2.6.1.-**	17	45	33	20	13	21	35	15	15	8	3	9	7	8	5	9	9	19	12	24
**2.7.1.21**	7	7	8	4	2	2	16	3	3	3	9	6	4	7	3	1	3	5	5	9
**2.7.1.48**	17	49	39	18	16	17	38	9	9	25	23	16	12	22	17	19	15	8	18	8
**2.7.4.-**	10	24	29	9	5	9	21	6	7	5	8	5	6	12	5	6	6	9	11	12
**2.8.3.-**	2	3	1	0	0	3	1	2	1	1	0	0	0	0	0	0	0	0	1	1
**2.8.3.1**	0	1	0	0	1	0	0	0	0	3	0	0	1	0	1	0	0	0	0	0
**2.8.3.12**	1	0	1	3	0	0	0	0	0	1	1	1	0	0	2	1	1	0	0	0
**2.8.3.6**	0	0	2	1	1	0	0	0	0	0	1	0	2	0	0	2	0	0	0	0
**2.8.3.8**	0	4	4	7	2	3	0	0	0	4	2	0	2	2	2	1	6	0	0	0
**3.1.1.-**	3	10	5	2	1	5	3	2	2	4	2	3	2	2	7	6	4	2	2	3
**3.1.1.1**	2	9	6	5	4	1	5	3	1	2	3	4	0	3	0	4	2	3	2	5
**3.1.1.17**	1	1	1	0	0	0	2	0	0	0	0	0	0	0	0	0	1	0	0	0
**3.1.1.2**	0	0	0	0	0	0	0	0	0	0	0	0	0	1	0	0	0	0	0	0
**3.1.1.24**	1	2	3	2	0	1	3	1	1	1	2	1	3	2	0	1	1	0	2	1
**3.1.1.45**	0	0	0	0	0	0	0	0	0	0	0	0	0	0	0	0	0	0	0	0
**3.1.2.-**	0	2	1	0	0	1	0	0	0	2	1	0	1	1	0	1	0	0	1	0
**3.1.2.23**	6	7	6	6	1	5	8	0	2	4	4	4	1	0	1	3	2	1	1	0
**3.1.3.1**	14	21	11	12	6	11	15	6	3	8	5	15	2	4	9	8	8	12	12	10
**3.1.3.2**	4	5	3	1	2	3	7	2	3	1	2	3	3	1	4	3	0	4	6	8
**3.1.3.41**	0	1	0	0	0	0	0	0	0	0	0	0	0	1	0	0	0	0	0	0
**3.2.1.31**	1	4	2	4	5	6	5	0	1	6	1	0	4	4	0	3	3	0	0	1
**3.3.2.9**	0	0	0	0	0	0	0	0	0	0	0	0	0	0	0	0	0	0	0	0
**3.5.1.-**	16	40	33	14	16	19	29	8	6	7	6	10	12	14	8	6	8	9	14	10
**3.5.1.4**	1	4	6	0	0	2	3	2	1	0	5	1	1	3	3	0	3	3	4	3
**3.5.1.5**	1	3	3	3	0	2	0	0	0	2	0	2	0	3	0	6	4	0	1	0
**3.5.1.54**	5	11	6	7	3	3	4	2	0	2	3	0	3	4	1	2	3	0	1	1
**3.5.1.6**	3	0	3	1	0	2	2	0	0	0	0	0	0	0	0	0	0	0	0	0
**3.5.2.-**	0	0	0	0	0	0	0	0	0	1	0	0	0	0	0	0	0	0	0	0
**3.5.2.2**	2	5	0	0	2	0	0	0	0	0	0	0	1	0	1	1	0	0	0	0
**3.5.4.-**	18	39	29	23	13	15	31	8	9	16	11	17	15	13	19	6	13	14	11	23
**3.5.4.5**	7	13	13	15	5	9	21	6	6	9	7	14	12	11	7	10	10	5	10	2
**3.5.5.1**	1	4	2	3	0	0	1	0	0	0	0	0	0	0	0	0	0	0	0	0
**3.5.5.7**	0	0	0	0	0	0	0	0	0	0	0	0	0	0	0	0	0	0	0	0
**3.5.99.3**	0	0	0	0	0	0	0	0	0	0	0	0	0	0	0	0	0	0	0	0
**3.6.1.7**	5	12	6	3	8	3	3	1	0	7	2	1	4	3	1	6	1	1	4	0
**3.7.1.-**	0	3	4	3	1	1	3	2	0	1	2	1	2	1	1	2	5	0	0	0
**3.7.1.2**	5	7	3	6	0	2	2	0	0	4	1	2	2	1	0	3	2	0	0	1
**3.8.1.2**	5	7	7	4	3	3	5	5	0	0	1	4	0	1	1	3	3	1	1	0
**3.8.1.3**	0	0	0	0	0	0	0	0	0	1	0	0	0	0	0	0	0	0	0	0
**3.8.1.5**	0	2	0	0	1	0	1	0	0	0	0	0	0	1	0	0	0	0	0	0
**4.1.1.-**	8	18	9	6	5	9	8	7	6	7	4	6	6	1	6	6	2	7	3	10
**4.1.1.44**	22	37	24	37	11	10	30	11	5	32	19	30	31	26	27	27	27	7	16	7
**4.1.1.55**	0	0	0	0	0	0	0	0	0	0	0	0	0	0	0	0	0	0	0	0
**4.1.1.7**	0	0	0	0	0	0	0	0	0	0	0	0	0	0	0	0	0	0	0	0
**4.1.1.70**	0	1	0	0	0	0	0	0	0	0	0	0	0	0	0	0	0	0	0	0
**4.1.1.77**	0	0	0	0	0	0	0	0	0	0	0	0	0	0	0	0	0	0	0	0
**4.1.2.-**	3	7	9	9	5	2	4	3	1	4	2	2	0	3	3	1	3	0	3	2
**4.1.3.-**	12	28	23	11	4	11	25	6	6	19	12	10	12	13	11	8	9	4	11	9

EC/KO	ES-AD-37	ES-AD-38	ES-AD-39	ES-AD-4	ES-AD-5	ES-AD-6	ES-AD-7	ES-AD-8	ES-AD-9	FR-AD-1	FR-AD-2	FR-AD-3	FR-AD-4	FR-AD-5	FR-AD-6	FR-AD-7	FR-AD-8	IN-1660SH	IN-1660SK	IN-199SD
4.1.3.39	0	0	1	0	0	0	1	0	1	0	0	2	0	0	0	0	0	2	1	2
4.1.99.-	1	2	2	0	0	1	2	1	2	0	0	0	0	2	0	1	1	0	0	0
4.2.1.-	35	68	53	45	19	33	52	15	13	28	13	18	25	19	25	26	18	10	20	20
4.2.1.17	9	6	9	2	4	4	8	4	3	7	2	4	2	4	1	5	3	2	5	1
4.2.1.80	0	0	0	0	0	0	0	0	0	0	0	0	0	0	0	0	0	0	0	0
4.2.1.83	1	0	1	1	1	0	1	0	0	0	0	0	1	1	1	0	2	0	0	0
4.2.1.84	1	0	1	0	1	0	0	0	0	0	0	0	0	0	0	0	1	0	0	0
5.1.2.2	0	0	0	0	0	0	0	0	0	0	0	0	0	0	0	0	0	0	0	0
5.2.1.2	0	0	0	0	0	0	0	0	0	0	0	0	0	0	0	0	0	0	0	0
5.3.3.4	0	0	0	0	0	1	0	0	0	0	0	0	0	0	0	0	0	0	0	0
5.3.99.-	2	4	4	5	3	1	2	0	0	2	1	1	4	5	2	2	4	0	0	0
5.4.99.-	0	1	0	2	0	0	0	0	0	1	1	0	0	1	0	0	0	0	0	1
5.5.1.1	2	3	4	2	1	2	3	1	0	0	1	1	0	0	0	0	0	0	0	0
5.5.1.2	0	0	0	0	0	0	0	0	0	0	0	0	0	0	0	0	0	0	0	0
6.2.1.-	0	0	0	0	0	0	0	0	0	0	0	0	0	0	0	0	0	0	0	0
6.3.5.2	13	31	26	17	8	12	22	9	4	8	3	4	6	10	5	8	7	5	9	11
K00002	3	2	4	0	0	3	0	2	0	1	0	0	1	0	0	0	0	0	0	0
K00055	0	0	0	0	0	0	0	0	0	1	0	0	0	0	0	0	0	0	0	0
K00074	1	10	7	6	1	3	6	3	1	9	6	1	3	3	6	2	5	1	2	0
K00088	13	19	16	15	7	16	18	7	7	10	9	8	3	5	6	7	5	10	12	13
K00100	41	70	63	42	28	19	52	18	16	36	19	29	20	30	27	18	19	20	22	22
K00128	0	2	2	0	1	1	4	1	1	1	0	0	1	0	0	0	0	0	0	0
K00129	0	0	0	0	0	0	0	0	0	0	0	0	0	0	0	0	0	0	0	0
K00132	0	1	0	0	0	1	0	1	0	0	0	0	0	0	0	0	0	0	0	0
K00141	0	0	0	0	0	0	0	0	0	0	0	0	0	0	0	0	0	0	0	0
K00146	0	0	0	0	0	0	0	0	0	0	0	0	0	0	0	0	0	0	0	0
K00148	0	0	0	0	0	0	0	0	0	0	0	0	0	0	0	0	0	0	0	0
K00155	1	0	0	1	0	0	0	0	0	0	0	0	0	0	0	0	0	0	0	0
K00169	1	6	2	0	3	0	1	0	1	0	1	0	0	0	0	0	0	0	2	0
K00224	1	1	0	0	0	0	0	0	0	0	0	0	0	0	0	1	0	0	0	0
K00274	0	0	0	0	0	0	0	0	0	0	0	0	0	0	0	0	0	0	0	0
K00446	0	0	0	0	0	0	0	0	0	0	0	0	0	0	0	0	0	0	0	0
K00448	0	0	0	0	0	0	0	0	0	0	0	0	0	0	0	0	0	0	0	0
K00462	1	0	1	0	0	0	0	1	0	0	0	0	0	0	0	0	0	0	0	0
K00480	0	0	0	0	0	0	0	0	0	0	0	0	0	0	0	0	0	0	0	0
K00481	0	0	0	0	0	0	0	0	0	0	0	0	0	0	0	0	0	0	0	0
K00539	1	0	0	0	0	0	0	0	0	0	0	1	0	0	0	0	0	0	1	0
K00599	44	73	59	20	31	24	48	13	21	18	14	18	22	19	15	16	13	19	21	25
K00626	2	4	1	2	3	4	3	4	3	1	0	0	1	4	3	0	0	0	2	1
K00632	0	0	0	0	0	0	0	0	0	0	0	0	0	0	0	0	0	0	0	0
K00680	22	53	34	33	27	19	34	15	12	31	21	21	28	18	11	21	18	10	15	19
K00757	13	15	11	12	7	8	13	6	4	12	7	2	5	6	5	9	4	8	5	10
K00758	3	4	7	8	0	1	4	0	1	0	1	1	5	4	0	2	1	0	2	1
K00760	15	26	33	27	16	17	26	15	7	14	11	12	15	15	15	11	11	6	6	2
K00799	0	0	0	0	0	0	1	0	0	1	0	0	0	0	1	0	0	0	1	0
K00857	8	11	15	10	4	3	16	4	4	6	12	6	9	9	5	2	10	6	8	8
K00876	18	36	29	21	12	18	33	11	10	16	16	15	7	14	8	10	8	18	22	23
K01026	0	1	1	0	2	1	0	0	0	0	2	1	1	0	0	0	2	0	0	0
K01031	0	0	0	0	0	0	0	0	0	0	0	0	0	0	0	0	0	0	0	0

EC/KO	ES-AD-37	ES-AD-38	ES-AD-39	ES-AD-4	ES-AD-5	ES-AD-6	ES-AD-7	ES-AD-8	ES-AD-9	FR-AD-1	FR-AD-2	FR-AD-3	FR-AD-4	FR-AD-5	FR-AD-6	FR-AD-7	FR-AD-8	IN-1660SH	IN-1660SK	IN-199SD
**K01034**	1	2	0	2	0	0	0	0	0	1	2	1	2	0	0	1	2	0	0	1
**K01039**	0	1	1	0	0	0	0	0	0	1	1	1	0	0	2	0	0	0	0	0
**K01041**	8	6	3	3	3	1	10	2	1	3	1	5	0	1	1	3	0	5	2	5
**K01053**	0	0	0	0	0	0	0	0	0	0	0	0	0	0	0	0	0	0	0	0
**K01055**	0	0	0	0	0	0	0	0	0	0	0	0	0	0	0	0	0	0	0	0
**K01061**	0	0	0	0	0	0	0	0	0	0	0	0	1	0	0	0	0	0	0	0
**K01066**	3	11	7	5	5	1	6	2	1	4	3	4	0	3	2	2	2	2	1	2
**K01075**	2	0	0	1	0	1	0	0	0	0	0	0	0	0	0	1	0	0	2	0
**K01077**	7	8	7	8	1	4	6	1	0	3	3	5	1	0	0	1	0	0	1	0
**K01101**	0	7	8	3	4	3	3	2	1	3	2	0	1	4	1	1	4	0	1	0
**K01195**	2	7	7	4	5	4	14	0	2	3	1	2	3	2	1	5	0	0	1	1
**K01426**	0	2	3	0	0	0	0	0	0	1	1	0	0	0	1	0	0	0	0	0
**K01428**	1	3	0	0	0	0	0	0	0	0	0	2	0	1	0	0	0	0	1	0
**K01457**	0	0	0	0	0	1	0	0	0	0	0	0	0	0	0	0	0	0	0	0
**K01464**	2	7	3	0	4	0	0	0	0	0	1	0	0	0	1	2	2	0	0	0
**K01489**	10	19	22	21	9	10	23	7	8	13	13	16	13	14	12	10	11	7	9	9
**K01500**	0	0	0	4	0	0	0	0	0	0	0	0	0	0	0	0	0	0	0	0
**K01501**	2	3	8	3	0	0	1	0	0	0	0	0	0	0	0	0	0	0	0	0
**K01502**	0	0	0	0	0	0	0	0	0	0	0	0	0	0	0	0	0	0	0	0
**K01512**	6	21	15	8	9	5	6	2	1	11	2	2	7	6	6	8	3	2	5	0
**K01560**	1	10	10	7	6	6	6	1	1	4	3	2	4	7	3	1	3	1	2	1
**K01561**	0	1	0	0	1	0	0	0	0	1	0	0	0	0	0	0	0	0	0	0
**K01563**	0	0	0	0	0	0	0	0	0	0	0	0	0	0	0	0	0	0	0	0
**K01564**	2	2	2	2	0	1	0	0	0	0	0	0	0	0	0	0	0	0	0	0
**K01607**	10	17	8	15	5	6	10	3	3	12	4	11	13	12	9	16	11	4	6	7
**K01612**	0	1	0	0	0	1	0	0	0	0	0	0	0	0	0	0	0	0	0	0
**K01615**	16	14	15	8	4	6	15	5	1	8	1	3	4	4	1	3	1	7	8	7
**K01617**	0	0	0	0	0	0	0	0	0	0	0	0	0	0	0	0	0	0	0	0
**K01666**	2	6	3	2	2	2	7	4	5	1	1	2	1	1	0	2	0	2	1	2
**K01692**	0	0	1	0	0	0	0	0	0	1	0	0	0	0	0	0	0	0	1	0
**K01721**	0	0	0	0	0	0	0	0	0	0	0	0	0	0	0	0	0	0	0	0
**K01726**	9	15	10	2	3	6	8	1	2	0	1	1	0	3	2	2	0	4	3	8
**K01781**	0	0	0	1	2	0	0	0	0	0	0	0	0	0	0	0	0	0	0	0
**K01821**	1	2	0	0	0	4	2	2	0	0	0	0	0	0	2	0	0	0	0	1
**K01856**	0	0	0	0	0	0	0	0	0	0	0	0	0	0	0	0	0	0	0	0
**K01857**	0	0	1	0	0	0	0	0	0	0	0	0	0	0	0	0	0	0	0	0
**K01913**	0	0	0	1	0	1	0	0	0	0	0	0	0	0	0	0	0	0	0	0
**K01951**	9	25	14	10	5	9	14	6	4	8	3	2	2	4	7	8	2	5	7	10
**K02554**	0	0	0	0	0	0	0	0	0	0	0	0	0	0	0	0	0	0	0	0
**K03381**	0	0	0	0	0	0	0	0	0	0	0	0	0	0	0	0	0	0	0	0
**K03382**	0	1	5	3	0	2	1	0	0	0	0	0	0	0	0	0	0	0	0	0
**K03464**	0	0	0	0	0	0	0	0	0	0	0	0	0	0	0	0	0	0	0	0
**K03518**	11	21	18	19	11	7	7	4	3	15	11	9	10	13	11	10	12	1	5	1
**K03862**	0	0	0	0	0	0	0	0	0	0	0	0	0	0	0	0	0	0	0	0
**K04099**	0	0	0	0	0	0	0	0	0	0	0	0	0	0	0	0	0	0	0	0
**K04100**	0	0	0	0	0	0	0	0	0	0	0	0	0	0	0	0	0	0	0	0
**K04102**	0	0	0	0	0	0	0	0	0	0	0	0	0	0	0	0	0	0	0	0
**K04116**	0	0	0	0	0	0	0	0	0	0	0	0	0	0	0	0	0	0	0	0
**K05394**	1	0	1	0	0	0	0	0	0	0	0	0	0	0	0	0	0	1	0	1

EC/KO	ES-AD-37	ES-AD-38	ES-AD-39	ES-AD-4	ES-AD-5	ES-AD-6	ES-AD-7	ES-AD-8	ES-AD-9	FR-AD-1	FR-AD-2	FR-AD-3	FR-AD-4	FR-AD-5	FR-AD-6	FR-AD-7	FR-AD-8	IN-1660SH	IN-1660SK	IN-199SD
**K05549**	0	0	0	0	0	0	0	0	0	0	0	0	0	0	0	0	0	0	0	0
**K05783**	0	0	0	0	0	0	0	0	0	0	0	0	0	0	0	0	0	0	0	0
**K05797**	0	0	0	0	0	0	0	0	0	0	0	0	0	0	0	0	0	0	0	0
**K06281**	2	2	3	1	0	0	1	1	0	0	1	0	0	0	0	0	0	0	0	0
**K06446**	5	2	4	13	4	1	3	1	3	8	3	2	3	4	4	4	1	0	4	1
**K06912**	0	0	0	0	0	0	0	0	0	0	0	0	0	0	0	0	0	0	0	0
**K07535**	0	0	0	0	0	0	1	0	0	0	0	0	0	0	0	1	0	0	0	0
**K07536**	1	5	5	3	0	1	1	0	2	1	3	5	1	0	2	1	1	5	5	6
**K08689**	0	0	0	0	0	0	0	0	0	0	0	0	0	0	0	0	0	0	0	0
**K08710**	0	0	0	0	0	0	0	0	0	0	0	0	0	0	0	0	0	0	0	0
**K09461**	0	1	0	0	0	0	0	0	0	1	1	0	0	0	0	0	0	0	0	0
**K10217**	0	0	0	0	0	0	0	0	0	0	0	0	0	0	1	0	0	0	0	0
**K10218**	0	0	0	1	0	0	1	1	0	0	0	0	1	1	1	0	0	0	0	0
**K10220**	0	0	0	0	0	0	0	0	0	0	0	0	0	0	0	0	0	0	0	0
**K11180**	0	0	0	0	0	0	0	0	0	0	0	0	0	0	0	1	1	0	0	0
**K13953**	0	0	0	0	0	1	0	0	0	1	0	0	0	0	1	0	0	0	0	0
**K14333**	0	0	0	0	0	0	0	0	0	0	0	0	0	0	0	0	0	0	0	0
**K14519**	0	0	0	0	0	0	0	0	0	0	0	0	0	0	0	0	0	0	0	0
**K15054**	0	0	0	0	0	0	0	0	0	0	0	0	0	0	0	0	0	0	0	0
**K16173**	0	1	0	0	1	0	0	0	0	0	0	0	1	0	0	0	0	0	0	0
**K16514**	0	0	0	0	0	0	0	0	0	0	0	0	0	0	0	0	0	0	0	0
**K16874**	0	1	0	1	0	0	0	0	0	0	0	0	0	0	0	0	0	0	0	0

EC/KO	IN-199SUBD	IN-683PD	IN-683RD	IN-882AK	IN-882RK	IN-976AB	IN-976DB	IN-A1	IN-AA6	IN-DB2	IN-IS8	IN-LB1	IN-SAK7	IN-SH3	IN-SK5	IN-SOH4	IN-U2	IT-AD-1	IT-AD-2	IT-AD-3	IT-AD-4	IT-AD-5
**1.1.1.-**	16	5	20	31	21	34	25	8	25	23	20	16	10	34	19	18	24	37	41	31	26	31
**1.1.1.1**	4	3	7	18	15	18	11	8	11	4	13	8	3	9	7	8	8	14	26	25	23	15
**1.1.1.157**	0	0	0	0	2	0	0	0	0	0	0	0	0	1	0	0	0	0	1	0	1	1
**1.1.1.205**	11	5	13	22	14	19	15	2	14	17	16	12	10	12	16	13	13	9	6	13	7	11
**1.1.1.35**	0	0	0	1	0	3	0	1	0	0	0	0	0	0	0	0	0	0	3	2	3	4
**1.12.99.6**	0	1	0	2	1	0	0	2	1	0	0	0	0	2	0	1	0	0	0	1	0	1
**1.13.11.-**	0	0	0	0	0	0	0	0	0	0	0	0	0	0	0	0	0	0	0	0	0	0
**1.13.11.1**	0	0	0	0	0	0	0	0	0	0	0	0	0	0	0	0	0	0	0	0	0	0
**1.13.11.2**	0	0	0	0	0	0	0	0	0	0	0	0	0	0	0	0	0	0	0	1	0	0
**1.13.11.3**	0	0	0	0	0	0	0	0	0	0	0	0	0	0	0	0	0	0	1	0	0	0
**1.13.11.39**	0	0	0	0	0	0	0	0	0	0	0	0	0	0	0	0	0	0	0	0	0	0
**1.13.11.5**	0	0	0	0	0	0	0	0	0	0	0	0	0	0	0	0	0	0	0	0	0	0
**1.13.11.8**	0	0	0	0	0	0	0	0	0	0	0	0	0	0	0	0	0	0	1	0	0	0
**1.14.12.10**	0	0	0	1	0	0	0	0	0	0	0	0	0	1	0	0	0	1	0	0	0	0
**1.14.12.13**	0	0	0	0	0	0	0	0	0	0	0	0	0	0	0	0	0	0	0	0	0	0
**1.14.12.18**	0	0	0	0	0	0	0	0	0	0	0	0	0	0	0	0	0	0	0	0	0	0
**1.14.13.-**	0	0	0	2	0	0	0	3	2	0	0	0	0	1	0	0	0	1	4	1	0	0
**1.14.13.1**	0	0	0	0	0	0	0	0	0	0	0	0	0	0	0	0	0	0	1	0	0	0
**1.14.13.2**	0	0	0	0	0	0	0	0	0	0	0	0	0	0	0	0	0	0	1	0	0	0
**1.14.13.50**	0	0	0	0	0	0	0	0	0	0	0	0	0	0	0	0	0	0	0	0	0	0
**1.14.13.7**	1	0	0	0	0	0	0	0	0	0	0	1	0	0	0	0	0	0	0	0	0	0
**1.14.13.8**	0	0	0	0	1	0	0	0	0	0	0	0	0	0	0	0	0	0	0	1	0	1
**1.14.13.82**	0	0	0	0	0	0	0	0	0	0	0	0	0	0	0	0	0	0	1	0	0	0
**1.14.99.-**	0	0	0	0	0	0	0	0	0	0	0	0	0	0	0	0	0	0	0	0	0	0
**1.17.99.1**	0	0	0	0	0	0	0	0	0	0	0	0	0	0	0	0	0	0	0	0	0	0
**1.18.6.1**	0	0	0	0	0	0	0	0	0	0	0	0	0	1	0	0	0	0	0	0	0	0
**1.2.1.-**	0	0	0	0	0	1	0	1	0	0	1	0	0	2	0	0	0	2	4	2	0	0
**1.2.1.10**	0	1	2	2	3	3	0	1	0	0	0	0	0	2	0	1	0	4	3	1	1	3
**1.2.1.3**	0	0	2	3	7	4	0	3	7	2	4	0	1	4	0	2	0	10	10	3	2	4
**1.2.1.39**	0	0	0	0	0	0	0	0	0	0	0	0	0	1	0	0	0	0	0	0	0	0
**1.2.7.1**	4	1	9	11	3	11	12	0	9	7	11	9	3	8	11	8	8	6	11	12	3	8
**1.2.99.2**	1	0	0	0	6	1	0	0	1	1	1	0	0	3	0	0	0	5	1	3	0	8
**1.3.1.-**	0	0	0	0	0	0	0	0	0	0	0	0	0	0	0	0	0	0	0	0	0	0
**1.3.1.2**	0	0	0	1	0	0	0	1	0	0	0	0	0	0	0	0	0	0	0	0	0	0
**1.3.1.25**	0	0	0	0	0	0	0	0	0	0	0	0	0	0	0	0	0	0	1	1	0	0
**1.3.99.-**	0	0	0	0	0	0	0	0	1	0	0	0	0	1	0	0	0	0	0	0	0	1
**1.6.5.-**	30	5	40	41	11	28	43	0	39	56	32	36	16	30	53	38	27	22	31	33	16	30
**1.7.1.-**	5	4	10	15	5	8	10	2	5	11	10	11	5	10	12	10	9	4	2	6	5	10
**1.8.99.3**	0	0	1	0	0	0	1	0	2	0	0	0	0	0	1	0	0	1	0	0	0	0
**2.1.1.-**	100	39	99	136	86	138	100	35	138	100	125	83	58	91	113	82	103	109	152	140	89	149
**2.3.1.-**	30	16	50	66	35	64	35	10	54	52	55	42	20	51	55	42	45	79	78	70	42	75
**2.3.1.16**	0	0	1	1	1	0	0	0	0	0	2	0	0	1	1	0	0	1	1	0	0	0
**2.3.1.5**	0	0	0	1	0	0	0	1	0	0	0	0	0	0	0	0	0	0	1	0	0	0
**2.3.1.9**	0	0	0	3	1	1	0	2	0	0	2	0	0	1	0	0	0	1	3	0	0	1
**2.4.2.10**	7	1	12	13	6	11	9	2	12	10	11	7	4	9	11	12	5	7	9	13	1	12
**2.4.2.3**	1	0	1	2	2	7	3	1	5	0	4	1	1	1	1	1	1	5	1	11	2	10
**2.4.2.4**	0	1	0	0	0	1	0	0	0	0	1	0	0	3	0	0	0	1	3	0	1	3
**2.4.2.8**	5	1	9	11	6	12	9	2	12	13	8	7	5	7	10	9	10	6	6	8	7	10
**2.5.1.-**	11	5	18	24	11	20	19	3	17	22	16	19	10	21	20	15	19	12	14	7	7	12

EC/KO	IN-199SUBD	IN-683PD	IN-683RD	IN-882AK	IN-882RK	IN-976AB	IN-976DB	IN-A1	IN-AA6	IN-DB2	IN-IS8	IN-LB1	IN-SAK7	IN-SH3	IN-SK5	IN-SOH4	IN-U2	IT-AD-1	IT-AD-2	IT-AD-3	IT-AD-4	IT-AD-5
**2.5.1.18**	0	0	0	3	0	1	0	4	1	2	0	0	0	4	0	0	0	2	9	4	2	1
**2.6.1.-**	8	7	17	29	10	24	17	2	15	18	26	24	11	17	27	16	21	10	5	8	7	9
**2.7.1.21**	5	3	7	7	5	7	6	0	6	7	6	8	2	7	11	8	4	3	4	3	2	2
**2.7.1.48**	11	5	12	13	4	7	6	0	18	14	11	10	3	8	6	9	9	10	20	19	10	13
**2.7.4.-**	9	2	9	12	8	14	10	0	13	13	12	10	8	10	13	12	13	8	9	6	3	4
**2.8.3.-**	1	0	1	3	1	0	1	1	1	0	0	0	0	1	0	0	0	0	1	0	0	0
**2.8.3.1**	0	0	0	0	0	0	1	0	0	0	0	0	0	0	0	1	0	0	1	0	0	2
**2.8.3.12**	2	0	0	1	0	0	0	0	1	0	1	0	0	0	0	0	0	1	1	1	0	0
**2.8.3.6**	1	0	0	0	0	0	0	0	0	0	0	0	0	0	1	0	0	1	1	0	0	0
**2.8.3.8**	1	0	0	1	1	1	1	2	0	0	0	0	0	0	0	1	0	1	1	1	0	7
**3.1.1.-**	1	0	3	4	4	1	1	5	6	4	5	0	2	4	4	0	1	2	9	6	2	7
**3.1.1.1**	1	1	3	2	0	3	6	0	1	6	3	5	1	4	4	2	3	4	5	3	0	1
**3.1.1.17**	0	0	0	0	0	0	0	0	0	0	0	0	0	0	0	0	0	0	1	0	0	1
**3.1.1.2**	0	0	0	0	1	0	0	0	1	1	0	0	0	1	1	0	0	0	2	0	0	1
**3.1.1.24**	1	0	0	2	0	0	0	1	0	0	0	0	0	0	0	0	0	0	3	1	0	0
**3.1.1.45**	0	0	0	0	0	0	0	1	0	0	0	0	0	1	0	0	0	1	2	0	0	0
**3.1.2.-**	0	0	0	0	2	2	0	1	2	1	0	0	0	1	1	0	0	1	4	2	0	1
**3.1.2.23**	0	1	1	1	0	1	0	0	2	2	1	1	2	0	1	1	2	4	1	2	0	0
**3.1.3.1**	3	6	11	16	4	7	6	2	9	12	15	11	7	8	12	14	12	6	3	4	5	1
**3.1.3.2**	3	2	7	8	3	3	6	1	1	9	7	7	7	8	8	6	9	1	2	3	3	1
**3.1.3.41**	0	0	0	0	0	1	0	1	0	0	0	0	0	0	0	0	0	1	1	1	0	0
**3.2.1.31**	0	0	0	1	0	0	0	2	3	0	0	0	0	2	0	0	0	2	0	3	2	0
**3.3.2.9**	0	0	0	0	0	0	0	0	0	0	0	0	0	0	0	0	0	0	1	1	0	0
**3.5.1.-**	11	2	14	18	9	11	11	1	13	15	16	11	7	12	14	13	11	10	7	12	5	7
**3.5.1.4**	4	0	4	5	3	4	3	1	2	4	2	3	2	5	3	2	3	3	2	0	3	3
**3.5.1.5**	0	0	1	2	4	2	1	3	1	0	1	0	0	0	2	0	0	0	4	0	1	0
**3.5.1.54**	4	0	1	0	2	0	1	2	1	1	0	0	0	2	1	0	1	4	5	5	2	3
**3.5.1.6**	0	0	0	0	0	0	0	0	0	0	0	0	0	0	1	0	0	0	2	1	0	0
**3.5.2.-**	0	0	0	0	0	0	0	0	0	0	0	0	0	0	0	0	0	0	0	0	0	0
**3.5.2.2**	0	0	0	0	0	0	0	0	0	0	0	0	0	1	0	0	0	0	0	1	0	1
**3.5.4.-**	12	7	13	24	14	19	12	2	24	16	18	24	9	12	22	14	9	14	13	21	7	20
**3.5.4.5**	6	2	3	4	3	7	4	0	4	4	2	5	1	2	0	4	6	10	11	8	4	10
**3.5.5.1**	1	0	0	0	0	0	0	0	1	0	0	0	0	0	1	0	0	0	0	0	0	0
**3.5.5.7**	0	0	0	0	2	0	0	0	0	0	0	0	0	0	0	0	0	0	0	0	0	0
**3.5.99.3**	0	0	0	0	0	0	0	0	0	0	0	0	0	0	0	0	0	1	0	0	0	0
**3.6.1.7**	1	1	2	3	2	1	0	0	2	1	1	1	0	0	1	0	1	4	3	5	4	2
**3.7.1.-**	2	2	0	0	1	2	0	0	0	0	0	0	0	0	0	0	0	1	3	1	2	1
**3.7.1.2**	0	0	0	0	0	0	0	0	0	0	0	0	0	1	1	0	0	1	3	2	0	2
**3.8.1.2**	1	0	1	3	1	6	0	1	0	0	1	1	1	2	1	1	0	5	1	5	0	2
**3.8.1.3**	0	0	0	0	0	0	0	0	0	0	0	0	0	0	0	0	0	0	0	0	0	0
**3.8.1.5**	0	0	0	0	0	0	0	1	0	0	0	0	0	0	0	0	0	0	1	0	0	1
**4.1.1.-**	2	1	7	11	14	4	7	1	8	9	5	6	4	6	9	6	9	5	9	8	2	4
**4.1.1.44**	16	3	11	12	10	14	10	0	12	12	11	10	4	9	11	5	8	16	25	18	19	16
**4.1.1.55**	0	0	0	0	0	0	0	0	0	0	0	0	0	0	0	0	0	0	0	0	0	0
**4.1.1.7**	0	0	0	0	0	0	0	0	0	0	0	0	0	0	0	0	0	0	0	0	0	0
**4.1.1.70**	0	0	0	0	0	0	0	0	0	0	0	0	0	0	0	0	0	0	0	0	0	0
**4.1.1.77**	0	0	0	0	0	0	0	0	0	0	0	0	0	0	0	0	0	0	0	0	0	0
**4.1.2.-**	2	0	2	0	3	0	2	3	5	0	1	0	1	4	2	4	2	3	2	7	2	5
**4.1.3.-**	8	4	11	14	6	13	8	0	11	10	8	7	4	7	10	8	12	11	10	11	10	8

EC/KO	IN-199SUBD	IN-683PD	IN-683RD	IN-882AK	IN-882RK	IN-976AB	IN-976DB	IN-A1	IN-AA6	IN-DB2	IN-IS8	IN-LB1	IN-SAK7	IN-SH3	IN-SK5	IN-SOH4	IN-U2	IT-AD-1	IT-AD-2	IT-AD-3	IT-AD-4	IT-AD-5
4.1.3.39	0	0	0	0	0	0	1	0	0	0	0	0	0	2	1	1	0	0	1	0	0	0
4.1.99.-	0	0	0	0	2	0	0	0	0	0	0	0	0	0	0	0	1	0	0	1	1	0
4.2.1.-	7	5	18	23	11	18	14	3	22	18	12	19	15	14	19	16	25	20	23	19	8	15
4.2.1.17	2	2	3	2	3	1	1	2	4	0	2	0	1	2	1	1	2	5	8	4	3	1
4.2.1.80	0	0	0	0	0	0	0	0	0	0	0	0	0	0	0	0	0	3	2	0	0	0
4.2.1.83	0	0	0	0	0	0	0	0	0	0	0	0	0	0	0	0	0	2	0	0	0	1
4.2.1.84	0	0	0	0	0	0	0	0	0	0	0	0	0	0	0	0	0	0	0	0	0	0
5.1.2.2	0	0	0	0	0	0	0	0	0	0	0	0	0	0	0	0	0	0	0	0	0	0
5.2.1.2	0	0	0	0	0	0	0	0	0	0	0	0	0	0	0	0	0	0	0	0	0	0
5.3.3.4	0	0	0	0	0	0	0	0	0	0	0	0	0	0	0	0	0	0	0	0	0	0
5.3.99.-	1	2	0	2	0	0	1	0	1	0	0	0	1	1	0	2	0	1	6	2	3	1
5.4.99.-	0	0	1	1	0	0	1	0	1	0	0	0	1	0	0	0	0	0	0	1	0	1
5.5.1.1	2	0	1	0	1	0	0	0	2	0	0	0	0	1	0	1	0	1	2	0	1	2
5.5.1.2	0	0	0	0	0	0	0	0	0	0	0	0	0	0	0	0	0	0	1	0	0	0
6.2.1.-	0	0	0	0	0	0	0	0	0	0	0	0	0	0	0	0	0	0	1	0	0	0
6.3.5.2	3	2	7	14	7	12	7	3	5	9	6	4	6	9	8	8	12	11	11	10	7	13
K00002	0	0	0	0	0	1	0	0	0	0	1	0	0	0	0	0	0	0	1	2	0	2
K00055	0	0	0	0	2	1	0	0	0	0	0	0	0	0	0	0	0	0	1	1	0	0
K00074	1	0	0	1	2	3	1	0	0	0	0	0	0	1	0	1	1	5	6	5	8	6
K00088	7	4	11	16	9	14	11	2	13	16	11	9	7	7	12	8	12	7	9	7	8	3
K00100	17	11	26	31	22	30	27	6	22	24	26	22	7	22	29	22	20	32	27	28	23	19
K00128	1	0	1	1	4	4	0	0	0	0	2	0	0	1	0	0	0	1	3	2	0	0
K00129	0	0	0	0	0	0	0	0	0	0	0	0	0	0	0	0	0	0	0	0	0	0
K00132	0	0	0	0	0	0	0	0	0	0	0	0	0	0	0	0	0	0	0	0	0	0
K00141	0	0	0	0	0	0	0	0	0	0	0	0	0	0	0	0	0	0	0	0	0	0
K00146	0	0	0	0	0	0	0	0	0	0	0	0	0	1	0	0	0	0	2	0	0	0
K00148	0	0	0	0	0	0	0	0	0	0	0	0	0	0	0	0	0	0	0	0	0	0
K00155	0	0	0	1	1	0	0	0	0	0	0	0	0	0	0	0	0	0	1	0	0	0
K00169	0	0	2	2	0	2	2	0	2	0	2	0	0	0	2	0	0	0	2	1	0	0
K00224	0	0	0	0	0	1	0	0	0	0	0	0	0	0	0	0	1	1	0	0	0	0
K00274	0	0	0	0	0	0	0	0	0	0	0	0	0	0	0	0	0	0	0	0	0	0
K00446	0	0	0	0	0	0	0	0	0	0	0	0	0	0	0	0	0	0	1	1	0	0
K00448	0	0	0	0	0	0	0	0	0	0	0	0	0	0	0	0	0	0	0	0	0	0
K00462	0	0	0	0	0	0	1	0	0	0	0	0	0	1	0	0	0	0	1	2	0	1
K00480	0	0	0	0	0	0	0	0	0	0	0	0	0	0	0	0	0	0	0	0	0	0
K00481	0	0	0	0	0	0	0	0	0	0	0	0	0	0	0	0	0	0	1	0	0	0
K00539	0	0	0	0	0	0	0	0	0	0	0	0	0	0	0	0	0	0	0	0	0	1
K00599	9	9	20	34	21	32	17	1	16	19	20	24	10	16	25	16	23	11	13	21	14	17
K00626	0	0	1	3	1	2	0	2	0	0	5	0	0	3	1	0	0	2	4	0	1	1
K00632	0	0	0	0	1	0	0	0	0	0	0	0	0	0	0	0	0	0	1	0	0	0
K00680	12	8	15	23	17	24	14	0	13	17	16	15	7	10	14	9	7	21	20	21	7	28
K00757	8	2	6	10	4	5	11	1	10	8	8	8	5	6	11	7	9	8	4	10	3	9
K00758	0	1	0	1	0	0	0	1	3	0	2	0	0	1	0	1	0	3	2	4	1	4
K00760	4	1	2	4	11	11	3	2	7	2	5	2	1	3	3	5	3	11	11	17	7	17
K00799	0	0	0	1	0	1	0	1	0	0	1	0	0	1	1	0	0	0	5	2	0	0
K00857	5	3	9	9	7	11	9	0	7	6	8	11	2	8	13	11	3	5	7	6	3	5
K00876	14	6	18	23	12	17	10	0	16	21	20	18	9	16	21	18	12	7	20	12	6	8
K01026	1	0	0	0	0	0	1	0	0	0	0	0	0	1	0	1	0	1	2	0	1	2
K01031	0	0	0	0	0	0	0	0	0	0	0	0	0	0	0	0	0	0	0	0	0	0

EC/KO	IN-199SUBD	IN-683PD	IN-683RD	IN-882AK	IN-882RK	IN-976AB	IN-976DB	IN-A1	IN-AA6	IN-DB2	IN-IS8	IN-LB1	IN-SAK7	IN-SH3	IN-SK5	IN-SOH4	IN-U2	IT-AD-1	IT-AD-2	IT-AD-3	IT-AD-4	IT-AD-5
K01034	1	0	0	0	0	0	0	1	0	0	0	0	0	0	0	1	0	1	0	0	0	2
K01039	0	0	0	0	0	0	0	0	0	0	0	0	0	0	0	0	0	1	0	1	0	0
K01041	3	2	6	10	3	5	6	1	7	7	7	5	4	6	6	4	4	2	1	2	2	0
K01053	0	0	0	0	0	0	0	0	0	0	0	0	0	0	0	0	0	0	0	0	0	0
K01055	0	0	0	0	0	0	0	0	0	0	0	0	0	0	0	0	0	0	0	0	0	0
K01061	0	0	0	0	0	0	0	1	1	0	0	0	0	2	0	0	0	1	5	0	0	0
K01066	2	0	2	0	2	1	2	2	2	5	2	1	0	3	2	1	1	3	7	3	0	1
K01075	0	0	0	1	0	0	2	0	1	0	0	0	0	1	0	0	0	0	1	1	0	1
K01077	0	2	1	1	2	3	0	2	1	1	2	0	0	1	1	1	2	1	3	1	0	1
K01101	1	0	0	1	3	3	1	0	1	0	0	0	0	0	0	1	1	1	6	3	0	4
K01195	0	0	0	2	0	1	0	1	3	2	1	0	0	1	1	0	0	2	1	2	1	1
K01426	0	0	0	0	1	2	0	0	0	0	0	0	0	0	0	0	0	2	2	0	0	2
K01428	0	0	1	0	3	1	1	1	1	0	0	0	0	0	1	0	0	0	1	0	0	0
K01457	0	0	0	0	0	0	0	0	0	0	0	0	0	0	0	0	0	0	0	0	0	0
K01464	1	0	0	0	0	1	0	1	0	0	1	0	0	1	0	1	0	0	1	2	0	2
K01489	8	3	8	10	3	8	6	2	8	7	7	4	5	6	7	7	9	11	13	10	7	18
K01500	0	0	1	0	0	0	0	0	1	0	1	0	1	0	0	0	0	0	2	1	0	0
K01501	2	0	0	0	0	0	0	0	1	0	1	0	0	0	1	1	1	0	0	0	0	0
K01502	0	0	0	0	2	0	0	0	0	0	0	0	0	0	0	0	0	0	0	0	0	0
K01512	1	1	2	4	6	5	1	0	3	1	2	1	2	0	2	0	2	5	4	8	5	5
K01560	1	1	3	3	1	0	1	2	1	1	3	0	0	0	1	0	2	6	6	2	4	4
K01561	0	0	0	0	0	0	0	0	0	0	0	0	0	0	0	0	0	0	1	0	0	0
K01563	0	0	0	0	0	0	0	0	0	0	0	0	0	0	0	0	0	0	0	0	0	0
K01564	0	0	0	0	0	0	0	1	0	0	0	0	0	1	0	0	0	1	0	1	0	0
K01607	7	2	11	7	2	8	5	2	6	11	5	7	3	7	7	4	8	8	7	6	8	3
K01612	0	0	0	0	0	0	0	0	0	0	0	0	0	0	0	0	0	0	0	0	0	0
K01615	5	2	9	7	0	7	5	1	7	6	6	5	6	5	9	4	7	5	5	7	4	7
K01617	0	0	0	0	0	0	0	0	0	0	0	0	0	0	0	0	0	0	0	0	0	0
K01666	0	1	0	0	0	0	1	0	0	0	1	0	0	2	1	1	0	1	2	2	0	0
K01692	0	0	0	0	0	0	0	0	0	0	0	0	0	0	0	0	0	0	2	0	0	0
K01721	0	0	0	0	0	0	0	0	0	0	0	0	0	0	0	0	0	0	0	0	0	0
K01726	3	3	7	11	2	6	6	1	5	5	7	8	4	3	8	4	8	3	2	0	0	1
K01781	0	0	0	0	0	0	0	0	0	0	0	0	0	0	0	0	0	0	0	0	0	0
K01821	1	0	0	0	5	4	0	0	1	1	0	0	0	0	0	0	0	2	2	3	1	1
K01856	0	0	0	0	0	0	0	0	0	0	0	0	0	0	0	0	0	0	0	0	0	0
K01857	0	0	0	0	0	0	0	0	0	0	0	0	0	0	0	1	0	0	1	0	0	0
K01913	0	0	1	0	0	0	0	0	1	0	0	0	0	0	0	0	0	0	0	0	0	0
K01951	6	2	8	17	5	15	8	3	4	10	8	6	6	9	7	8	10	6	9	5	3	8
K02554	0	0	0	0	0	0	0	0	0	0	0	0	0	0	0	0	0	2	2	0	0	0
K03381	0	0	0	0	0	0	0	0	0	0	0	0	0	0	0	0	0	0	0	0	0	0
K03382	0	0	0	0	0	1	0	0	0	0	0	0	0	0	0	0	0	2	1	0	0	0
K03464	0	0	0	0	0	0	0	0	0	0	0	0	0	0	0	0	0	0	0	0	0	0
K03518	4	1	0	0	3	3	0	3	4	1	1	0	1	0	2	0	1	7	18	5	5	12
K03862	0	0	0	0	0	0	0	0	0	0	0	0	0	0	0	0	0	0	0	0	0	0
K04099	0	0	0	0	0	0	0	0	0	0	0	0	0	0	0	0	0	0	1	0	0	0
K04100	0	0	0	0	0	0	0	0	0	0	0	0	0	0	0	0	0	0	0	0	0	0
K04102	0	0	0	0	0	0	0	0	0	0	0	0	0	0	0	0	0	0	0	0	0	0
K04116	0	0	0	0	0	0	0	0	0	0	0	0	0	0	0	0	0	1	0	0	0	0
K05394	0	0	1	1	0	0	0	0	1	0	1	0	1	0	0	1	1	0	1	1	0	0

EC/KO	IN-199SUBD	IN-683PD	IN-683RD	IN-882AK	IN-882RK	IN-976AB	IN-976DB	IN-A1	IN-AA6	IN-DB2	IN-IS8	IN-LB1	IN-SAK7	IN-SH3	IN-SK5	IN-SOH4	IN-U2	IT-AD-1	IT-AD-2	IT-AD-3	IT-AD-4	IT-AD-5
K05549	0	0	0	0	0	0	0	0	0	0	0	0	0	0	0	0	0	0	1	0	0	0
K05783	0	0	0	0	0	0	0	0	0	0	0	0	0	0	0	0	0	0	1	1	0	0
K05797	0	0	0	0	0	0	0	0	0	0	0	0	0	0	0	0	0	0	0	0	0	0
K06281	0	0	0	2	1	0	0	2	0	0	0	0	0	2	0	0	0	1	1	1	0	1
K06446	1	0	0	1	2	1	1	0	2	0	4	1	1	1	1	2	0	2	4	5	2	2
K06912	0	0	0	0	0	0	0	0	0	0	0	0	0	0	0	0	0	0	0	0	0	0
K07535	0	0	0	0	0	0	0	0	0	0	0	0	0	0	0	0	0	0	0	0	0	0
K07536	1	0	3	5	2	1	6	3	4	4	3	9	2	6	7	3	4	2	2	2	1	2
K08689	0	0	0	0	0	0	0	0	0	0	0	0	0	0	0	0	0	0	0	0	0	0
K08710	0	0	0	0	0	0	0	0	0	0	0	0	0	1	0	0	0	0	0	0	0	0
K09461	0	0	0	0	0	1	0	1	0	0	0	0	0	0	0	0	0	1	2	1	2	1
K10217	0	0	0	0	0	0	0	0	0	0	0	0	0	1	0	0	0	0	0	0	0	0
K10218	1	0	0	0	0	0	0	0	0	0	0	0	0	0	0	0	0	0	0	1	0	0
K10220	0	0	0	0	0	0	0	0	0	0	0	0	0	0	0	0	0	0	0	0	0	0
K11180	0	0	0	0	0	0	0	0	0	0	0	0	0	0	0	0	0	3	0	0	0	0
K13953	0	0	0	2	0	1	1	1	0	0	0	0	0	1	0	0	0	0	3	1	3	2
K14333	0	0	0	0	0	0	0	0	0	0	0	0	0	0	0	0	0	0	1	0	0	0
K14519	0	0	0	0	0	0	0	0	0	0	0	0	0	0	0	0	0	0	0	0	0	0
K15054	0	0	0	0	1	0	0	0	0	0	0	0	0	0	0	0	0	0	1	0	0	0
K16173	0	0	0	1	0	0	1	0	0	0	0	0	1	0	0	2	0	0	0	0	0	0
K16514	0	0	0	0	0	0	0	0	1	0	0	0	0	1	0	0	0	0	0	0	0	0
K16874	0	0	0	0	0	0	0	0	0	0	0	0	0	0	0	0	0	0	0	0	0	0

EC/KO	IT-AD-6	JP-AD-1	JP-AD-2	JP-AD-3	JP-AD-4	JP-AD-7	JP-AD-8	JP-AD-9	JP-CH-1	JP-CH-2	JP-IN-1	JP-IN-2	JP-IN-3	JP-IN-4
**1.1.1.-**	34	16	18	25	21	17	22	23	22	27	36	8	18	29
**1.1.1.1**	25	14	26	19	19	8	14	17	28	19	23	15	17	17
**1.1.1.157**	0	0	2	0	0	0	0	0	2	0	0	0	0	0
**1.1.1.205**	12	5	9	17	8	1	9	4	8	9	4	4	6	3
**1.1.1.35**	2	1	2	0	0	1	0	2	2	0	1	0	1	1
**1.12.99.6**	1	0	1	0	0	1	0	0	0	2	1	0	0	3
**1.13.11.-**	0	0	0	0	0	0	0	0	0	0	0	0	1	0
**1.13.11.1**	0	0	0	0	0	0	0	0	0	0	0	0	0	0
**1.13.11.2**	0	0	0	0	0	0	0	0	0	0	0	0	0	0
**1.13.11.3**	0	0	0	1	0	0	0	0	0	0	1	0	0	0
**1.13.11.39**	0	0	0	0	0	0	0	0	0	0	0	0	0	0
**1.13.11.5**	0	0	0	0	0	0	0	0	0	0	0	0	0	0
**1.13.11.8**	0	0	0	0	0	0	0	0	0	0	1	0	0	0
**1.14.12.10**	0	0	0	1	0	0	0	0	0	0	1	0	0	0
**1.14.12.13**	0	0	0	0	0	0	0	0	0	0	0	0	0	0
**1.14.12.18**	0	0	0	1	0	0	0	0	0	0	0	0	0	1
**1.14.13.-**	0	1	0	1	0	0	0	0	1	0	18	3	0	3
**1.14.13.1**	0	0	0	0	0	0	0	0	0	0	2	0	0	0
**1.14.13.2**	0	0	0	1	0	0	0	0	0	0	1	0	0	0
**1.14.13.50**	0	0	0	0	0	0	0	0	1	0	0	0	0	0
**1.14.13.7**	0	0	0	0	0	0	0	0	0	0	0	0	0	0
**1.14.13.8**	0	0	0	0	0	0	0	0	0	0	0	0	0	0
**1.14.13.82**	0	0	0	1	0	0	0	0	0	1	4	0	0	0
**1.14.99.-**	0	0	0	0	0	0	0	0	0	0	0	0	0	0
**1.17.99.1**	0	0	0	0	0	0	0	0	0	0	0	0	0	0
**1.18.6.1**	0	0	0	0	0	0	0	0	0	0	4	0	0	0
**1.2.1.-**	0	0	0	0	0	0	0	0	0	0	5	0	1	1
**1.2.1.10**	3	5	2	1	2	0	0	1	1	2	2	0	0	2
**1.2.1.3**	3	6	4	5	2	1	2	5	2	1	17	1	6	7
**1.2.1.39**	0	0	0	0	0	0	0	0	0	0	0	0	0	0
**1.2.7.1**	13	3	3	3	4	1	4	3	3	9	0	0	1	0
**1.2.99.2**	6	1	3	2	1	0	1	3	1	3	8	0	2	4
**1.3.1.-**	0	0	0	0	0	1	1	0	0	0	0	0	0	0
**1.3.1.2**	0	0	0	0	0	0	0	0	0	1	0	0	0	1
**1.3.1.25**	0	0	0	0	0	0	0	0	0	0	1	0	0	0
**1.3.99.-**	0	0	0	0	0	0	0	0	2	0	0	0	0	1
**1.6.5.-**	36	25	24	28	26	15	21	14	13	26	11	0	4	4
**1.7.1.-**	8	4	2	4	4	5	3	8	4	1	2	1	1	2
**1.8.99.3**	0	0	0	0	0	0	0	0	0	0	0	0	0	0
**2.1.1.-**	146	103	130	148	105	54	88	115	89	100	73	18	56	66
**2.3.1.-**	62	56	62	56	55	37	64	57	40	72	75	12	23	28
**2.3.1.16**	0	0	2	2	0	0	0	0	1	1	3	0	0	0
**2.3.1.5**	0	0	0	1	0	0	0	0	0	0	2	0	0	1
**2.3.1.9**	0	0	0	0	0	0	0	1	1	0	6	0	0	2
**2.4.2.10**	12	10	7	7	6	3	5	10	7	7	4	2	6	5
**2.4.2.3**	3	2	1	3	4	1	3	4	5	1	2	0	2	6
**2.4.2.4**	0	1	1	2	2	0	0	2	0	1	0	0	0	0
**2.4.2.8**	8	4	4	5	5	5	2	5	5	5	2	1	4	7
**2.5.1.-**	12	9	11	19	11	5	3	8	5	9	10	3	3	3

EC/KO	IT-AD-6	JP-AD-1	JP-AD-2	JP-AD-3	JP-AD-4	JP-AD-7	JP-AD-8	JP-AD-9	JP-CH-1	JP-CH-2	JP-IN-1	JP-IN-2	JP-IN-3	JP-IN-4
**2.5.1.18**	0	0	1	2	2	0	0	3	1	0	16	0	0	4
**2.6.1.-**	6	12	6	12	13	12	8	9	7	14	6	0	4	4
**2.7.1.21**	6	2	1	2	1	2	3	0	6	6	3	0	1	2
**2.7.1.48**	20	10	10	20	10	5	9	14	11	15	1	1	3	3
**2.7.4.-**	3	8	5	5	7	5	7	7	4	5	1	1	2	3
**2.8.3.-**	1	0	0	1	0	0	0	0	0	1	1	0	1	2
**2.8.3.1**	1	0	1	0	0	0	0	0	0	0	0	0	0	1
**2.8.3.12**	2	1	0	1	0	0	0	0	0	0	0	0	0	0
**2.8.3.6**	0	0	0	1	0	0	0	0	1	1	1	0	0	0
**2.8.3.8**	2	0	2	2	1	0	0	3	1	0	1	0	0	5
**3.1.1.-**	1	2	4	3	3	0	2	3	2	2	6	0	0	8
**3.1.1.1**	1	5	2	4	0	0	3	1	3	4	0	0	0	1
**3.1.1.17**	0	0	0	1	0	0	0	0	0	2	4	0	0	0
**3.1.1.2**	0	0	0	0	0	0	0	0	0	0	4	1	0	2
**3.1.1.24**	5	2	1	1	0	1	2	1	0	0	3	0	2	0
**3.1.1.45**	0	0	1	0	0	0	0	0	0	0	3	0	0	1
**3.1.2.-**	1	0	0	1	0	0	1	0	2	0	5	1	3	2
**3.1.2.23**	2	1	0	0	0	1	2	2	2	4	0	0	0	0
**3.1.3.1**	9	7	2	6	5	3	8	5	6	5	1	0	0	3
**3.1.3.2**	3	2	0	6	2	2	2	0	0	2	3	0	0	3
**3.1.3.41**	0	0	1	0	2	0	0	0	0	0	0	1	0	1
**3.2.1.31**	2	4	2	5	4	0	0	2	0	2	3	1	4	2
**3.3.2.9**	0	0	0	1	0	0	0	0	0	0	1	0	0	0
**3.5.1.-**	13	8	6	6	10	3	7	9	8	5	5	3	2	3
**3.5.1.4**	4	2	2	3	0	0	0	1	1	1	0	1	0	2
**3.5.1.5**	1	0	0	2	1	0	0	0	0	2	2	0	1	2
**3.5.1.54**	4	0	6	6	1	1	0	1	1	1	12	3	1	2
**3.5.1.6**	2	2	2	3	0	0	0	1	0	0	5	0	0	1
**3.5.2.-**	0	0	0	0	1	0	1	1	0	0	2	0	0	0
**3.5.2.2**	1	0	1	1	0	0	0	0	0	0	0	1	1	0
**3.5.4.-**	19	7	5	13	10	6	8	7	8	16	5	5	3	4
**3.5.4.5**	8	4	3	3	1	5	4	6	3	4	1	0	1	2
**3.5.5.1**	0	0	0	2	0	0	2	0	1	0	1	0	0	0
**3.5.5.7**	0	0	0	0	0	0	0	0	0	0	0	0	0	0
**3.5.99.3**	0	0	0	0	0	0	0	0	0	0	0	0	0	1
**3.6.1.7**	5	3	4	4	1	0	1	4	4	1	2	1	5	3
**3.7.1.-**	1	1	2	2	1	0	1	1	2	1	5	0	1	0
**3.7.1.2**	2	0	2	0	1	0	1	1	0	2	0	0	0	0
**3.8.1.2**	4	2	2	0	2	1	1	3	2	4	5	1	0	1
**3.8.1.3**	0	0	0	0	0	0	0	0	0	0	0	0	0	0
**3.8.1.5**	0	1	0	3	1	0	2	2	2	0	4	2	3	0
**4.1.1.-**	10	4	6	5	5	1	11	3	7	4	17	0	1	6
**4.1.1.44**	48	16	16	10	14	3	7	13	13	11	3	3	1	4
**4.1.1.55**	0	0	0	0	0	0	0	0	0	0	0	0	0	0
**4.1.1.7**	0	0	0	0	0	0	0	0	0	0	0	0	0	0
**4.1.1.70**	0	0	0	0	0	0	0	0	0	0	0	0	0	0
**4.1.1.77**	0	0	0	0	0	0	0	0	0	0	0	0	0	0
**4.1.2.-**	5	1	5	10	2	3	3	6	2	3	13	0	3	5
**4.1.3.-**	17	9	6	12	8	5	6	11	8	6	4	0	2	4

EC/KO	IT-AD-6	JP-AD-1	JP-AD-2	JP-AD-3	JP-AD-4	JP-AD-7	JP-AD-8	JP-AD-9	JP-CH-1	JP-CH-2	JP-IN-1	JP-IN-2	JP-IN-3	JP-IN-4
**4.1.3.39**	0	0	0	0	0	0	0	0	0	0	0	0	0	0
**4.1.99.-**	0	0	0	0	1	2	0	1	0	1	0	0	0	1
**4.2.1.-**	26	20	14	14	16	5	17	26	18	18	5	1	4	8
**4.2.1.17**	6	2	0	7	0	2	1	7	5	4	6	0	0	2
**4.2.1.80**	0	0	0	0	0	0	0	0	0	0	3	0	0	0
**4.2.1.83**	0	0	0	0	0	0	0	0	0	0	0	0	0	0
**4.2.1.84**	0	0	0	0	0	0	0	0	2	0	0	0	0	0
**5.1.2.2**	0	0	0	0	0	0	0	0	0	0	0	0	0	0
**5.2.1.2**	0	0	0	0	0	0	0	0	0	0	0	0	0	0
**5.3.3.4**	0	0	1	0	0	0	0	0	0	0	0	2	0	0
**5.3.99.-**	2	1	4	1	0	2	2	6	1	1	2	0	4	6
**5.4.99.-**	2	0	0	2	0	0	0	0	0	0	0	0	0	0
**5.5.1.1**	0	1	1	3	0	0	1	2	0	1	2	0	0	1
**5.5.1.2**	0	0	0	0	0	0	0	0	0	0	0	0	0	0
**6.2.1.-**	0	0	0	0	0	0	0	0	0	0	2	0	0	0
**6.3.5.2**	6	10	12	6	8	3	4	3	6	7	2	6	4	10
**K00002**	0	0	0	1	0	0	0	1	1	0	0	0	1	0
**K00055**	0	0	0	0	0	0	1	0	0	0	0	0	0	0
**K00074**	2	5	4	5	3	2	3	5	4	0	2	0	1	2
**K00088**	6	3	6	12	4	2	6	3	4	9	5	4	4	5
**K00100**	23	20	28	32	28	15	20	21	17	32	20	9	9	23
**K00128**	0	1	0	0	1	0	1	1	1	0	6	1	2	3
**K00129**	0	0	0	0	0	0	0	0	0	0	0	0	0	0
**K00132**	0	0	0	0	0	0	0	0	2	1	2	0	0	1
**K00141**	0	0	0	0	0	0	0	0	0	0	2	0	0	0
**K00146**	0	0	0	0	0	0	0	0	0	0	1	0	0	0
**K00148**	0	0	0	0	0	0	0	0	0	0	0	0	0	0
**K00155**	0	0	0	0	0	0	0	0	0	0	1	0	0	0
**K00169**	0	0	0	0	1	0	0	0	0	0	0	0	0	0
**K00224**	0	0	0	1	0	0	0	0	0	0	0	0	0	0
**K00274**	0	0	0	0	0	0	0	0	0	0	0	0	0	0
**K00446**	0	0	0	0	0	0	0	0	0	0	0	0	0	0
**K00448**	0	0	0	0	0	0	0	0	0	0	0	0	0	0
**K00462**	0	0	2	1	0	0	0	0	1	0	1	0	0	0
**K00480**	0	0	0	0	1	0	0	0	0	0	0	0	0	0
**K00481**	0	0	0	0	0	0	0	0	0	0	1	0	0	0
**K00539**	0	0	0	0	0	0	0	0	0	1	0	0	0	0
**K00599**	27	20	12	16	17	13	16	22	10	14	4	3	15	5
**K00626**	0	1	3	4	1	0	1	3	1	1	4	0	0	2
**K00632**	0	0	0	1	0	0	0	0	1	1	2	0	0	0
**K00680**	17	13	16	20	17	5	12	7	11	19	12	5	4	8
**K00757**	11	6	3	2	6	3	5	2	5	4	3	0	1	7
**K00758**	3	1	3	2	2	0	1	0	2	0	2	0	1	3
**K00760**	17	5	6	9	9	5	7	8	7	8	4	1	6	7
**K00799**	1	0	0	2	1	0	0	0	0	0	19	0	0	1
**K00857**	10	6	3	5	3	2	4	3	7	7	4	0	2	2
**K00876**	13	10	8	20	8	4	6	8	10	12	3	0	3	1
**K01026**	1	0	0	3	0	0	0	2	0	0	0	0	1	0
**K01031**	0	0	0	1	0	0	0	0	1	0	0	0	0	0

EC/KO	IT-AD-6	JP-AD-1	JP-AD-2	JP-AD-3	JP-AD-4	JP-AD-7	JP-AD-8	JP-AD-9	JP-CH-1	JP-CH-2	JP-IN-1	JP-IN-2	JP-IN-3	JP-IN-4
K01034	0	1	0	1	2	0	1	0	0	0	0	0	0	1
K01039	0	0	0	0	0	0	0	0	0	0	0	0	0	0
K01041	1	2	0	3	1	1	5	1	1	1	1	0	0	1
K01053	0	0	0	1	0	0	0	0	0	0	0	0	0	0
K01055	0	0	0	1	0	0	0	0	0	0	5	0	0	0
K01061	0	0	1	0	0	0	0	0	0	1	6	0	0	1
K01066	1	5	3	6	2	1	2	1	2	4	2	0	1	4
K01075	0	0	0	0	0	1	0	0	0	0	2	0	0	1
K01077	1	4	2	1	2	2	5	1	4	3	5	0	1	3
K01101	1	1	1	2	3	0	1	3	0	1	0	0	5	3
K01195	3	3	2	5	3	0	2	1	0	4	1	2	4	1
K01426	0	1	0	1	0	0	0	0	0	0	1	0	1	2
K01428	0	0	0	0	0	0	0	0	0	0	2	0	1	1
K01457	0	0	0	1	0	0	0	0	0	0	3	0	0	0
K01464	2	0	4	3	0	1	0	0	1	0	2	0	1	2
K01489	7	5	4	8	5	5	4	10	7	6	2	1	1	4
K01500	0	0	0	0	0	0	0	0	0	0	0	0	1	0
K01501	0	1	1	2	0	0	1	0	4	0	0	0	1	0
K01502	0	0	0	0	0	0	0	0	0	0	0	0	0	0
K01512	9	4	7	7	2	1	1	4	5	2	7	2	5	3
K01560	4	4	3	4	7	0	3	2	5	3	1	3	3	3
K01561	0	0	0	0	0	0	0	0	0	0	0	0	0	1
K01563	0	0	0	0	0	0	0	0	0	0	0	0	0	0
K01564	0	0	1	1	0	0	0	0	0	0	1	0	0	0
K01607	10	6	4	6	4	2	4	6	4	6	3	1	1	4
K01612	0	0	0	0	0	0	0	0	0	0	0	0	0	0
K01615	3	6	10	9	8	4	7	3	4	9	0	0	0	3
K01617	0	0	0	0	0	0	0	0	0	0	0	0	0	0
K01666	0	1	1	2	0	1	1	1	0	0	2	0	0	0
K01692	0	0	0	0	0	0	0	1	0	0	2	0	1	0
K01721	0	0	0	0	0	0	0	0	0	0	2	0	0	0
K01726	2	2	1	3	2	1	1	6	0	1	3	0	0	1
K01781	0	0	0	0	0	0	0	1	0	0	0	0	0	0
K01821	1	0	1	1	1	1	1	1	1	0	4	0	1	1
K01856	0	0	0	0	0	0	0	0	0	0	0	0	0	0
K01857	0	0	0	0	0	0	0	1	0	0	1	0	1	0
K01913	0	0	0	0	0	0	0	0	0	0	0	0	0	0
K01951	2	6	10	7	6	3	3	2	7	9	5	3	5	7
K02554	0	0	0	1	0	1	0	0	0	0	1	0	0	0
K03381	0	0	0	0	0	0	0	0	0	0	0	0	0	0
K03382	0	0	0	0	0	0	0	0	2	0	1	0	0	1
K03464	0	0	0	0	0	0	0	0	0	0	0	0	0	0
K03518	24	5	6	7	6	5	6	7	8	4	2	0	3	9
K03862	0	0	0	0	0	0	0	0	0	0	3	0	0	0
K04099	0	0	0	0	0	0	0	0	0	0	0	0	0	0
K04100	0	0	0	0	0	0	0	0	0	0	1	0	0	0
K04102	0	0	0	0	0	0	0	0	0	0	0	0	0	0
K04116	0	0	0	0	0	0	0	0	0	0	1	0	0	0
K05394	0	0	1	0	1	0	0	0	0	1	0	0	0	0

EC/KO	IT-AD-6	JP-AD-1	JP-AD-2	JP-AD-3	JP-AD-4	JP-AD-7	JP-AD-8	JP-AD-9	JP-CH-1	JP-CH-2	JP-IN-1	JP-IN-2	JP-IN-3	JP-IN-4
**K05549**	0	0	0	0	0	0	0	0	0	0	2	0	0	0
**K05783**	0	0	0	0	0	0	0	0	0	0	0	0	0	0
**K05797**	0	0	0	0	0	0	0	0	0	0	0	0	0	0
**K06281**	0	1	1	1	0	0	0	0	0	1	2	0	0	2
**K06446**	5	5	9	4	2	0	2	5	2	4	0	0	1	0
**K06912**	0	0	0	0	0	0	0	0	0	0	0	0	0	0
**K07535**	0	0	0	0	0	0	0	0	0	0	0	0	0	0
**K07536**	3	4	1	3	2	2	0	2	0	1	2	0	0	3
**K08689**	0	0	0	0	0	0	0	0	0	0	0	0	0	0
**K08710**	0	0	0	0	0	0	0	0	1	0	1	0	0	0
**K09461**	0	0	0	0	1	0	1	0	2	1	0	1	0	0
**K10217**	0	0	0	0	0	0	0	0	0	0	0	0	0	0
**K10218**	1	0	1	0	0	0	0	0	1	1	0	0	0	0
**K10220**	0	0	0	0	0	0	0	0	0	0	0	0	0	0
**K11180**	0	1	0	0	0	0	0	0	0	0	0	0	0	0
**K13953**	1	0	0	0	0	0	0	0	2	0	5	1	0	1
**K14333**	0	0	0	0	0	0	0	0	1	0	1	0	0	0
**K14519**	0	0	1	0	0	0	0	0	0	0	1	0	0	0
**K15054**	0	0	0	0	0	0	0	0	0	0	3	0	0	0
**K16173**	0	0	0	3	0	0	0	2	0	0	0	0	0	1
**K16514**	0	0	1	0	0	0	0	0	0	0	2	0	0	0
**K16874**	0	0	0	0	0	0	0	0	0	0	0	0	0	0
