# Supplementary material for: Xenobiotic Metabolism and Gut Microbiomes
Source: PLoS One. 2016 Oct 3;11(10):e0163099. doi: 10.1371/journal.pone.0163099 (PMC5047465; doi:10.1371/journal.pone.0163099)
Supplement: S9 Table — (PDF) [file pone.0163099.s028.pdf]

Microbial Family	Cluster number : Group specificity		Microbial Family	Cluster number : Group specificity		Microbial Family	Cluster number : Group specificity
Desulfobulbaceae	C1 : G2 Specific		Cellulomonadaceae	C1 : G2 Specific		Picrophilaceae	C3 : G1 specific
Deferribacteraceae	C1 : G2 Specific		Carnobacteriaceae	C1 : G2 Specific		Peptostreptococcaceae	C3 : G1 specific
Ectothiorhodospiraceae	C1 : G2 Specific		Cardiobacteriaceae	C1 : G2 Specific		Pentatomidae	C3 : G1 specific
Methylophilaceae	C1 : G2 Specific		Brachyspiraceae	C1 : G2 Specific		Parachlamydiaceae	C3 : G1 specific
Porphyromonadaceae	C1 : G2 Specific		Beutenbergiaceae	C1 : G2 Specific		Oscillospiraceae	C3 : G1 specific
Thermoanaerobacteraceae	C1 : G2 Specific		Bdellovibrionaceae	C1 : G2 Specific		Orobanchaceae	C3 : G1 specific
Peptococcaceae	C1 : G2 Specific		Bartonellaceae	C1 : G2 Specific		Opisthorchiidae	C3 : G1 specific
Enterobacteriaceae	C1 : G2 Specific		Bacteroidaceae	C1 : G2 Specific		Nitrosopumilaceae	C3 : G1 specific
Chlorobiaceae	C1 : G2 Specific		Bacteriovoracaceae	C1 : G2 Specific		Mycoplasmataceae	C3 : G1 specific
Aquificaceae	C1 : G2 Specific		Bacillidae	C1 : G2 Specific		Moritellaceae	C3 : G1 specific
Micrococcaceae	C1 : G2 Specific		Anaerolinaceae	C1 : G2 Specific		Microcystidae	C3 : G1 specific
Thermotogaceae	C1 : G2 Specific		Alicyclobacillaceae	C1 : G2 Specific		Microchaetaceae	C3 : G1 specific
Syntrophaceae	C1 : G2 Specific		Alcanivoracaceae	C1 : G2 Specific		Methylocystaceae	C3 : G1 specific
Pasteurellaceae	C1 : G2 Specific		Aerococcaceae	C1 : G2 Specific		Methanothermaceae	C3 : G1 specific
Nocardioidaceae	C1 : G2 Specific		Acidothermaceae	C1 : G2 Specific		Methanospirillaceae	C3 : G1 specific
Coriobacteriaceae	C1 : G2 Specific		Acidobacteriaceae	C1 : G2 Specific		Methanosarcinaceae	C3 : G1 specific
[Clostridiales]	C1 : G2 Specific		Acidithiobacillaceae	C1 : G2 Specific		Methanosaetaceae	C3 : G1 specific
Campylobacteraceae	C1 : G2 Specific		Acidimicrobiaceae	C1 : G2 Specific		Methanopyraceae	C3 : G1 specific
Aeromonadaceae	C1 : G2 Specific		Acholeplasmataceae	C1 : G2 Specific		Methanomicrobiaceae	C3 : G1 specific
Waddliaceae	C1 : G2 Specific		Actinosynnemataceae	C2 : G1 and G2		Methanocorpusculaceae	C3 : G1 specific
Vesicomyidae	C1 : G2 Specific		Actinomycetaceae	C2 : G1 and G2		Methanococcaceae	C3 : G1 specific
Verrucomicrobiaceae	C1 : G2 Specific		Alteromonadaceae	C2 : G1 and G2		Methanocaldococcaceae	C3 : G1 specific
Tsukamurellaceae	C1 : G2 Specific		Clostridiales	C2 : G1 and G2		Methanobacteriaceae	C3 : G1 specific
Trueperaceae	C1 : G2 Specific		Cryomorphaceae	C2 : G1 and G2		Lycoperdaceae	C3 : G1 specific
Thermomicrobiaceae	C1 : G2 Specific		Geobacteraceae	C2 : G1 and G2		Kineosporiaceae	C3 : G1 specific
Thermoanaerobacterales	C1 : G2 Specific		Halobacteroidaceae	C2 : G1 and G2		Halobacteriaceae	C3 : G1 specific
Thermaceae	C1 : G2 Specific		Halomonadaceae	C2 : G1 and G2		Ferroplasmaceae	C3 : G1 specific
Syntrophomonadaceae	C1 : G2 Specific		Heliobacteriaceae	C2 : G1 and G2		Erysipelotrichaceae	C3 : G1 specific
Streptococcaceae	C1 : G2 Specific		Lachnospiraceae	C2 : G1 and G2		Entomoplasmataceae	C3 : G1 specific
Staphylococcaceae	C1 : G2 Specific		Lactobacillaceae	C2 : G1 and G2		Desulfurococcaceae	C3 : G1 specific
Sphaerobacteraceae	C1 : G2 Specific		Legionellaceae	C2 : G1 and G2		Dermabacteraceae	C3 : G1 specific
Segniliparaceae	C1 : G2 Specific		Leptospiraceae	C2 : G1 and G2		Cyclobacteriaceae	C3 : G1 specific
Sanguibacteraceae	C1 : G2 Specific		Leuconostocaceae	C2 : G1 and G2		Cucurbitaceae	C3 : G1 specific

Microbial Family	Cluster number : Group specificity		Microbial Family	Cluster number : Group specificity		Microbial Family	Cluster number : Group specificity
Rhodothermaceae	C1 : G2 Specific		Listeriaceae	C2 : G1 and G2		Chlamydomonadaceae	C3 : G1 specific
Puniceicoccaceae	C1 : G2 Specific		Nitrospiraceae	C2 : G1 and G2		Chlamydiaceae	C3 : G1 specific
Psychromonadaceae	C1 : G2 Specific		Planococcaceae	C2 : G1 and G2		Cenarchaeaceae	C3 : G1 specific
Pseudococcidae	C1 : G2 Specific		Promicromonosporaceae	C2 : G1 and G2		Caricaceae	C3 : G1 specific
Pseudoalteromonadaceae	C1 : G2 Specific		Saprospiraceae	C2 : G1 and G2		Caldisphaeraceae	C3 : G1 specific
Proteidae	C1 : G2 Specific		Streptomycetaceae	C2 : G1 and G2		Caldilineaceae	C3 : G1 specific
Prochlorococcaceae	C1 : G2 Specific		Syntrophobacteraceae	C2 : G1 and G2		Brevibacteriaceae	C3 : G1 specific
Planctomycetaceae	C1 : G2 Specific		Thermodesulfobiaceae	C2 : G1 and G2		Bovidae	C3 : G1 specific
Pelobacteraceae	C1 : G2 Specific		Thermomonosporaceae	C2 : G1 and G2		Blattabacteriaceae	C3 : G1 specific
Parvularculaceae	C1 : G2 Specific		Flexibacteraceae	C2 : G1 and G2		Aurantimonadaceae	C3 : G1 specific
Opitutaceae	C1 : G2 Specific		Rhodospirillaceae	C2 : G1 and G2		Asteraceae	C3 : G1 specific
Nocardiopsaceae	C1 : G2 Specific		Microbacteriaceae	C2 : G1 and G2		Archaeoglobaceae	C3 : G1 specific
Nocardiaceae	C1 : G2 Specific		Fusobacteriaceae	C2 : G1 and G2		Xanthomonadaceae	C4 : G3 specific
Nitrosomonadaceae	C1 : G2 Specific		Bacillaceae	C2 : G1 and G2		Sphingomonadaceae	C4 : G3 specific
Nautiliaceae	C1 : G2 Specific		Paenibacillaceae	C2 : G1 and G2		Moraxellaceae	C4 : G3 specific
Natranaerobiaceae	C1 : G2 Specific		Vibrionaceae	C2 : G1 and G2		Desulfobacteraceae	C4 : G3 specific
Nakamurellaceae	C1 : G2 Specific		Flavobacteriaceae	C2 : G1 and G2		Pseudonocardiaceae	C4 : G3 specific
Mantidae	C1 : G2 Specific		Spirochaetaceae	C2 : G1 and G2		Sphingobacteriaceae	C4 : G3 specific
Lythraceae	C1 : G2 Specific		Neisseriaceae	C2 : G1 and G2		Chloroflexaceae	C4 : G3 specific
Jonesiaceae	C1 : G2 Specific		Methylococcaceae	C2 : G1 and G2		Caulobacteraceae	C4 : G3 specific
Idiomarinaceae	C1 : G2 Specific		Intrasporangiaceae	C2 : G1 and G2		Rhodocyclaceae	C4 : G3 specific
Hyphomicrobiaceae	C1 : G2 Specific		Geodermatophilaceae	C2 : G1 and G2		Rhizobiaceae	C4 : G3 specific
Hydrogenothermaceae	C1 : G2 Specific		Eubacteriaceae	C2 : G1 and G2		Phyllobacteriaceae	C4 : G3 specific
Hydrogenophilaceae	C1 : G2 Specific		Enterococcaceae	C2 : G1 and G2		Oceanospirillaceae	C4 : G3 specific
Helicobacteraceae	C1 : G2 Specific		Bifidobacteriaceae	C2 : G1 and G2		Oxalobacteraceae	C4 : G3 specific
Halothiobacillaceae	C1 : G2 Specific		Anaplasmatace	C2 : G1 and G2		Comamonadaceae	C4 : G3 specific
Haliangiaceae	C1 : G2 Specific		Rhodobacteraceae	C3 : G1 specific		Methylobacteriaceae	C4 : G3 specific
Halanaerobiaceae	C1 : G2 Specific		Piscirickettsiaceae	C3 : G1 specific		Alcaligenaceae	C4 : G3 specific
Glycomycetaceae	C1 : G2 Specific		Chromatiaceae	C3 : G1 specific		Burkholderiaceae	C4 : G3 specific
Gemmatimonadaceae	C1 : G2 Specific		Rickettsiaceae	C3 : G1 specific		Xanthobacteraceae	C4 : G3 specific
Gallionellaceae	C1 : G2 Specific		Nostocaceae	C3 : G1 specific		Bradyrhizobiaceae	C4 : G3 specific
Francisellaceae	C1 : G2 Specific		Ruminococcaceae	C3 : G1 specific		Beijerinckiaceae	C4 : G3 specific
Fibrobacteraceae	C1 : G2 Specific		Veillonellaceae	C3 : G1 specific		Hyphomonadaceae	C4 : G3 specific

Microbial Family	Cluster number : Group specificity		Microbial Family	Cluster number : Group specificity		Microbial Family	Cluster number : Group specificity
Ferrimonadaceae	C1 : G2 Specific		Thiotrichaceae	C3 : G1 specific		Myxococcaceae	C4 : G3 specific
Erythrobacteraceae	C1 : G2 Specific		Thermoproteaceae	C3 : G1 specific		Brucellaceae	C4 : G3 specific
Edwardsiidae	C1 : G2 Specific		Thermoplasmataceae	C3 : G1 specific		Pseudomonadaceae	C4 : G3 specific
Dictyoglomaceae	C1 : G2 Specific		Thermofilaceae	C3 : G1 specific		Micromonosporaceae	C4 : G3 specific
Desulfurobacteriaceae	C1 : G2 Specific		Thermodesulfobacteriaceae	C3 : G1 specific		Streptosporangiaceae	C4 : G3 specific
Desulfurellaceae	C1 : G2 Specific		Thermococcaceae	C3 : G1 specific		Shewanellaceae	C4 : G3 specific
Desulfovibrionaceae	C1 : G2 Specific		Theaceae	C3 : G1 specific		Rubrobacteraceae	C4 : G3 specific
Desulfomicrobiaceae	C1 : G2 Specific		Sulfolobaceae	C3 : G1 specific		Polyangiaceae	C4 : G3 specific
Desulfohalobiaceae	C1 : G2 Specific		Spiroplasmataceae	C3 : G1 specific		Mycobacteriaceae	C4 : G3 specific
Desulfarculaceae	C1 : G2 Specific		Simkaniaceae	C3 : G1 specific		Herpetosiphonaceae	C4 : G3 specific
Dermacoccaceae	C1 : G2 Specific		Scytonemataceae	C3 : G1 specific		Hahellaceae	C4 : G3 specific
Deinococcaceae	C1 : G2 Specific		Rivulariaceae	C3 : G1 specific		Frankiaceae	C4 : G3 specific
Coxiellaceae	C1 : G2 Specific		Rikenellaceae	C3 : G1 specific		Cystobacteraceae	C4 : G3 specific
Corynebacteriaceae	C1 : G2 Specific		Pyrodictiaceae	C3 : G1 specific		Conexibacteraceae	C4 : G3 specific
Colwelliaceae	C1 : G2 Specific		Propionibacteriaceae	C3 : G1 specific		Catenulisporaceae	C4 : G3 specific
Clostridiaceae	C1 : G2 Specific		Prevotellaceae	C3 : G1 specific		Acetobacteraceae	Equal propensity
