# Supplementary material for: Xenobiotic Metabolism and Gut Microbiomes
Source: PLoS One. 2016 Oct 3;11(10):e0163099. doi: 10.1371/journal.pone.0163099 (PMC5047465; doi:10.1371/journal.pone.0163099)
Supplement: S10 Table — (PDF) [file pone.0163099.s029.pdf]

Xenobiotic Degradation Pathway	Drugs in the category	Links
Felbamate Cyclophosphamide and Ifosfamide Degradation	Cytoxan (TN); Cyclophosphamide (INN); Ifex; Ifosfamide	1
Xylene Degradation	Lidocaine	2
Chlorocyclohexane and Chlorobenzene Degradation	Katasuccin; Succinic Acid; Assuccin;	3
Toluene Degradation	Benzyl Alcohol; Butylated Hydroxytoulene	4
Chloroalkane and Chloroalkene Degradation	Formalin; Oxomethane; Anadrol-50	5
Dioxin Degradation	Benzenecarboxylic acid; Phenylformic acid; Dracylic acid; o-Hydroxybenzoic acid	6
Ethylbenzene Degradation	Propanoic acid: Solubilizing agent in Drugs; Cumene; Benzenemethanol; Hydroxymethylbenzene	7
Naphthalene Degradation	Salicylic acid; o-Hydroxybenzoic acid; Terbinafine (USAN/INN); Lamasil (TN); Tolnaftate	8
Caprolactam Degradation	6-Aminocaproic acid; Epsilcapramine; 6-Aminohexanoate;	9
Bisphenol Degradation	Bisphenol A; Diethylstilbestrol	10
Codeine and Morphine Degradation	Codeine (USP); Codeine hydrate; Morphine (BAN); Substitol (TN)	11
Azathioprine and 6- Mercaptopurine Degradation	Azathioprine (JP16/USP/INN); Azasan (TN); Imuran (TN); Mercaptopurine (INN); Purinethol (TN)	12
Isoniazid Degradation	Isoniazid (JP16/USP/INN); Laniazid (TN)	13
Fluorouracil and Irinotecan Degradation	Fluorouracil, Adrucil, Carac; Campto (TN); Irinotecan hydrochloride (USAN); CPT 11;	14
Tamoxifen Degradation	Tamoxifen citrate (JP16/USP); Nolvadex (TN); Tamoplex (TN)	15
Aminobenzoate Degradation	Ethyl aminobenzoate (JP16);Benzocaine (USP/INN);Parathesin (TN)	16
Benzoate Degradation	Rizatriptan benzoate (JAN/USAN); Maxalt (TN); Phenylformic acid; o-Hydroxybenzoic acid	17
Citalopram Degradation	Citalopram (USP/INN); Citadur (TN)	18
Cyclophosphamide and Ifosfamide Degradation	Cytoxan (TN); Cyclophosphamide (INN); Ifex; Ifosfamide	19
Metabolism of Xenobiotics- CYP450	Generic: All Drugs	20
Steroid Degradation	Range of Steroid based drugs.	21
Furfural Degradation	5-hydroxymethyl-2-furfural	22
Fluorobenzoate Degradation	3-(p-Fluorobenzoyloxy)tropane; triphenyltin 4-fluorobenzoate;	23
Styrene Degradation	Polystyrene sulfonate based drugs;	24
Polycyclic Aromatic Hydrocarbon Degradation	Benzoctamine; Valrubicin; Lumefantrine; Halofantrine; Nalbuphine	25
Atrazine Degradation	NA	NA
DDT Degradation	NA	NA
Nitrotoluene Degradation	NA	NA

Links:

1. http ://www.genome.jp/dbget-bin/www_bget?dr:D07760 |http://www.genome.jp/dbget-bin/www_bget?dr:D00343|http://www.genome.jp/dbget-bin/www_bget?dg:DG01633|http://www.genome.jp/dbget-bin/www_bget?dg:DG01720
2. http://apps.who.int/iris/bitstream/10665/93142/1/EML_18_eng.pdf?ua=1
3. http://pubchem.ncbi.nlm.nih.gov/compound/succinic_acid#section=Top | http://www.ncbi.nlm.nih.gov/pubmed/20036722
4. http://pubs.acs.org/doi/abs/10.1021/ja00411a035|http://www.genome.jp/dbget-bin/www_bget?cpd:C00556|http://www.genome.jp/kegg-bin/get_htext?br08312+C00556+D00077
5. http://www.genome.jp/dbget-bin/www_bget?cpd:C00067 | http://www.medapharma.us/products/pi/Anadrol-50_PI.pdf
6. http://www.genome.jp/dbget-bin/www_bget?cpd:C00180|http://www.genome.jp/dbget-bin/www_bget?cpd:C00805
7. http://www.genome.jp/dbget-bin/www_bget?cpd:C00556|http://www.fda.gov/downloads/drugs/guidancecomplianceregulatoryinformation/guidances/ucm073395.pdf
8. http://www.genome.jp/dbget-bin/www_bget?cpd:C00805|http://www.genome.jp/dbget-bin/www_bget?D02375+D02219
9. http://www.genome.jp/dbget-bin/www_bget?cpd:C02378
10. http://www.genome.jp/dbget-bin/www_bget?cpd:C13624 | www.drugbank.ca
11. http://www.genome.jp/dbget-bin/www_bget?D00195+C06174|http://www.genome.jp/dbget-bin/www_bget?D08233+D00842+D02271
12. http://www.genome.jp/dbget-bin/www_bget?D00238+C06837
13. http://www.genome.jp/dbget-bin/www_bget?D00346+C07054
14, http://www.genome.jp/dbget-bin/www_bget?D00584+C07649|http://www.genome.jp/dbget-bin/www_bget?D08086+C16641
15. http://www.genome.jp/dbget-bin/www_bget?D00966+D08559
16. http://www.genome.jp/dbget-bin/www_bget?dr:D00552
17. http://www.genome.jp/dbget-bin/www_bget?dr:D00675
18. http://www.genome.jp/dbget-bin/www_bget?dr:D07704
19. http://www.genome.jp/dbget-bin/www_bget?dr:D07760|http://www.genome.jp/dbget-bin/www_bget?dr:D00343|http://www.genome.jp/dbget-bin/www_bget?dg:DG01633|http://www.genome.jp/dbget-bin/www_bget?dg:DG01720
20. http://www.genome.jp/kegg-bin/show_pathway?map00982
21. http://www.genome.jp/kegg/drug/br08317.htmll|http://www.genome.jp/dbget-bin/www_bget?dr:D00389|http://www.ncbi.nlm.nih.gov/books/NBK47237/|http://www.hindawi.com/journals/isrn/2012/985495/
22. http://www.ncbi.nlm.nih.gov/pubmed/15686467
23. http://www.ncbi.nlm.nih.gov/pubmed/7669950|https://www.ncbi.nlm.nih.gov/pubmed/21083227
24. https://microbewiki.kenyon.edu/index.php/Styrene_metabolism_in_bacteria|http://www.ncbi.nlm.nih.gov/pubmed/16823552
25. www.drugbank.ca
