# Supplementary material for: Xenobiotic Metabolism and Gut Microbiomes
Source: PLoS One. 2016 Oct 3;11(10):e0163099. doi: 10.1371/journal.pone.0163099 (PMC5047465; doi:10.1371/journal.pone.0163099)
Supplement: S11 Table — (PDF) [file pone.0163099.s030.pdf]

Drug	Genera (Cluster Affiliation)	References
Rutin	Bacteroides(2)| Bacillus(2)| Veillonella(1)	1
Catechin and epicatechin	Clostridium(2) | Eubacterium(2),Bifidobacterium(2),Escherichia (2)	2
Anthocyanins	Streptococcus(2) | Eubacterium(2) | Clostridium(2) | Bacteroides(2)	3
Genistin	Bacteroides(2) | Streptococcus(2) | Ruminococcus(2) |Enterococcus(1)|Lactobacillus(2) | Finegoldia(2) | Veillonella(1)|Clostridium(2) | Eubacterium(2) |Butyrivibrio(2)	3
Naringin	Clostridium (2) | Butyrivibrio (2)	3,4
Digoxin	Eggerthella (2) | Eubacterium(2)	5
Polyphenols	Butyrivibrio (2)| Bacteroides(2)	6
Gliadin	Bifidobacterium (2) | Enterobacteria (2)	7
Daidzein	Bacteroides (2)|Streptococcus (2)|Ruminococcus (2)|Enterococcus(1)|Lactobacillus (2)| Finegoldia (2)|Veillonella(1)|Clostridium (2)|Eubacterium(2)	3
Sulphasalazine	Escherichia (2)|Bacteroides (2)|Klebsiella(3)|Pseudomonas(3)|Staphylococcus (2)|Proteus (2)	8
Nitrazepam	Clostridium (2)| Bacteroides (2)| Eubacterium (2)	9
Sulphinpyrazone	Escherichia (2)|Enterobacter (2)|Providencia (1)|Klebsiella (3)	10
Sulindac	Escherichia (2)|Proteus (2)|Providencia (1)|Klebsiella (3)|Citrobacter (2)|Pseudomonas (3)| Enterococcus (2)| Bacteroides (2)| Clostridium (2)	10,11
Quercetin-3-glucoside	Eubacterium (2) | Enterococcus(1)	12

References:

1) Yang J. et. al. Identification of rutin deglycosylated metabolites produced by human intestinal bacteria using UPLC-Q-TOF/MS. *J Chromatogr B* **898,** 95-100 (2012).

2) Tzounis X. et. al. Flavanol monomer-induced changes to the human faecal microflora. *Br J Nutr.* **99,** 782-92 (2008).

3) Selma MV, Espín JC, Tomás-Barberán FA. Interaction between phenolics and gut microbiota: role in human health. *J Agric Food Chem.* **57,** 6485-501 (2009)

4) Cheng K. J., Krishnamurty H. G., Jones G. A., Simpson F. J. Identification of products produced by the anaerobic degradation of naringin by Butyrivibrio sp. C3. *Can. J. Microbiol.* **17**,129–131 (1971).

5) Saha J. R., Butler V. P. Jr., Neu H. C., Lindenbaum J. Digoxin-inactivating bacteria: identification in human gut flora. *Science.* **220**, 325-327 (1983).

6) Rowland I. R. Interactions of the gut microflora and the host in toxicology. *Toxicol Pathol.* **16**, 147-53 (1988)

7) Cinova J. et al. Role of Intestinal Bacteria in Gliadin-Induced Changes in Intestinal Mucosa: Study in Germ-Free Rats. Leulier F, ed. *PLoS ONE* **6,** e16169 (2011).

8) Azad Khan A. K., Guthrie G., Johnston H. H., Truelove S. C., Williamson D. H. Tissue and bacterial splitting of sulphasalazine. *Clin Sci (Lond).* **64**, 349-54 (1983).

9) Rafii F., Sutherland J. B., Hansen E. B. Jr., Cerniglia C. E. Reduction of nitrazepam by Clostridium leptum, a nitroreductase-producing bacterium isolated from the human intestinal tract. *Clin Infect Dis.* **25** Suppl 2,S121-2 (1997).

10) Strong H. A., Renwick A. G., George C. F., Liu Y. F., Hill M. J. The reduction of sulphinpyrazone and sulindac by intestinal bacteria. *Xenobiotica.* **17**, 685-96 (1987).

11) Strong H. A., Warner N. J., Renwick A. G., George C. F. Sulindac metabolism: the importance of an intact colon. *Clin Pharmacol Ther*. **38**, 387-93 (1985).

12) Sousa T., Paterson R., Moore V., Carlsson A., Abrahamsson B., Basit A. W. The gastrointestinal microbiota as a site for the biotransformation of drugs. *Int J Pharm.* **363**,1-25 (2008).
