# Supplementary material for: Xenobiotic Metabolism and Gut Microbiomes
Source: PLoS One. 2016 Oct 3;11(10):e0163099. doi: 10.1371/journal.pone.0163099 (PMC5047465; doi:10.1371/journal.pone.0163099)
Supplement: S12 Table — (PDF) [file pone.0163099.s031.pdf]

EC/KO	Pathways
1.1.1.-	Benzoate Degradation|Bisphenol Degradation|Chloroalkane and Chloroalkene Degradation|Furfural Degradation|Nitrotoluene Degradation
1.1.1.1	Chloroalkane and Chloroalkene Degradation|Felbamate Cyclophosphamide and Ifosfamide Degradation|Naphthalene Degradation
1.1.1.1.	Metabolism of Xenobiotics- CYP450
1.1.1.145	Steroid Degradation
1.1.1.146	Metabolism of Xenobiotics- CYP450
1.1.1.157	Benzoate Degradation
1.1.1.174	Caprolactam Degradation
1.1.1.184	Metabolism of Xenobiotics- CYP450
1.1.1.2	Caprolactam Degradation
1.1.1.205	Azathioprine and 6-Mercaptopurine Degradation
1.1.1.245	Caprolactam Degradation
1.1.1.256	Polycyclic Aromatic Hydrocarbon Degradation
1.1.1.257	Toluene Degradation
1.1.1.258	Caprolactam Degradation
1.1.1.259	Benzoate Degradation
1.1.1.311	Ethylbenzene Degradation
1.1.1.312	Aminobenzoate Degradation|Benzoate Degradation
1.1.1.35	Caprolactam Degradation|Toluene Degradation
1.1.1.368	Benzoate Degradation
1.1.1.51	Steroid Degradation
1.1.1.90	Caprolactam Degradation|Toluene Degradation|Xylene Degradation
1.1.1.97	Toluene Degradation
1.1.2.7	Chloroalkane and Chloroalkene Degradation
1.1.2.8	Chloroalkane and Chloroalkene Degradation
1.1.3.19	Aminobenzoate Degradation
1.1.3.38	Aminobenzoate Degradation
1.1.3.6	Steroid Degradation
1.1.99.31	Aminobenzoate Degradation
1.12.99.6	Nitrotoluene Degradation
1.13.11.-	Aminobenzoate Degradation|Benzoate Degradation|Chlorocyclohexane and Chlorobenzene Degradation|DDT Degradation|Dioxin Degradation|Ethylbenzene Degradation|Naphthalene Degradation|Polycyclic Aromatic Hydrocarbon Degradation|Toluene Degradation|Xylene Degradation
1.13.11.1	Benzoate Degradation|Chlorocyclohexane and Chlorobenzene Degradation|Fluorobenzoate Degradation|Toluene Degradation
1.13.11.13	Polycyclic Aromatic Hydrocarbon Degradation
1.13.11.14	Benzoate Degradation|Xylene Degradation
1.13.11.2	Benzoate Degradation|Chlorocyclohexane and Chlorobenzene Degradation|Styrene Degradation|Xylene Degradation
1.13.11.25	Steroid Degradation
1.13.11.3	Benzoate Degradation
1.13.11.37	Benzoate Degradation|Chlorocyclohexane and Chlorobenzene Degradation
1.13.11.38	Polycyclic Aromatic Hydrocarbon Degradation
1.13.11.39	Chlorocyclohexane and Chlorobenzene Degradation|Dioxin Degradation
1.13.11.41	Bisphenol Degradation
1.13.11.5	Styrene Degradation

1.13.11.56	Naphthalene Degradation
1.13.11.57	Aminobenzoate Degradation
1.13.11.66	Chlorocyclohexane and Chlorobenzene Degradation
1.13.11.74	Aminobenzoate Degradation
1.13.11.8	Aminobenzoate Degradation|Benzoate Degradation|Polycyclic Aromatic Hydrocarbon Degradation
1.13.12.-	Chloroalkane and Chloroalkene Degradation|Nitrotoluene Degradation
1.14.11.-	Chlorocyclohexane and Chlorobenzene Degradation
1.14.12.-	Aminobenzoate Degradation|Chlorocyclohexane and Chlorobenzene Degradation|Dioxin Degradation|Ethylbenzene Degradation|Fluorobenzoate Degradation|Naphthalene Degradation|Nitrotoluene Degradation|Polycyclic Aromatic Hydrocarbon Degradation|Styrene Degradation|Toluene Degradation|Xylene Degradation
1.14.12.1	Aminobenzoate Degradation
1.14.12.10	Benzoate Degradation|Fluorobenzoate Degradation
1.14.12.11	Chloroalkane and Chloroalkene Degradation|Toluene Degradation
1.14.12.12	Ethylbenzene Degradation|Naphthalene Degradation|Polycyclic Aromatic Hydrocarbon Degradation
1.14.12.13	Fluorobenzoate Degradation
1.14.12.14	Benzoate Degradation|Toluene Degradation
1.14.12.15	Polycyclic Aromatic Hydrocarbon Degradation
1.14.12.18	Dioxin Degradation
1.14.12.22	Dioxin Degradation
1.14.12.3	Benzoate Degradation|Chlorocyclohexane and Chlorobenzene Degradation
1.14.12.7	Polycyclic Aromatic Hydrocarbon Degradation
1.14.12.8	Toluene Degradation
1.14.13.-	Aminobenzoate Degradation|Benzoate Degradation|Bisphenol Degradation|Chloroalkane and Chloroalkene Degradation|Chlorocyclohexane and Chlorobenzene Degradation|Naphthalene Degradation|Polycyclic Aromatic Hydrocarbon Degradation|Styrene Degradation|Toluene Degradation|Xylene Degradation
1.14.13.1	Dioxin Degradation|Naphthalene Degradation|Polycyclic Aromatic Hydrocarbon Degradation
1.14.13.12	Aminobenzoate Degradation|Benzoate Degradation
1.14.13.141	Steroid Degradation
1.14.13.142	Steroid Degradation
1.14.13.167	Aminobenzoate Degradation
1.14.13.172	Naphthalene Degradation
1.14.13.2	Benzoate Degradation
1.14.13.20	Chlorocyclohexane and Chlorobenzene Degradation
1.14.13.22	Caprolactam Degradation
1.14.13.23	Benzoate Degradation|Polycyclic Aromatic Hydrocarbon Degradation
1.14.13.24	Benzoate Degradation
1.14.13.27	Aminobenzoate Degradation
1.14.13.29	Aminobenzoate Degradation
1.14.13.33	Benzoate Degradation
1.14.13.35	Aminobenzoate Degradation
1.14.13.40	Aminobenzoate Degradation
1.14.13.50	Chlorocyclohexane and Chlorobenzene Degradation|Fluorobenzoate Degradation
1.14.13.63	Styrene Degradation
1.14.13.64	Benzoate Degradation
1.14.13.69	Chloroalkane and Chloroalkene Degradation

1.14.13.7	Aminobenzoate Degradation|Chlorocyclohexane and Chlorobenzene Degradation|Toluene Degradation
1.14.13.8	Tamoxifen Degradation
1.14.13.82	Aminobenzoate Degradation
1.14.13.84	Bisphenol Degradation
1.14.14.1	Aminobenzoate Degradation|Metabolism of Xenobiotics- CYP450
1.14.14.11	Styrene Degradation
1.14.14.12	Steroid Degradation
1.14.15.-	Atrazine Degradation|Caprolactam Degradation
1.14.16.6	Aminobenzoate Degradation
1.14.99.-	DDT Degradation|Naphthalene Degradation
1.14.99.15	Benzoate Degradation
1.14.99.23	Benzoate Degradation
1.17.3.2	Azathioprine and 6-Mercaptopurine Degradation
1.17.99.1	Toluene Degradation
1.17.99.2	Ethylbenzene Degradation
1.18.6.1	Chloroalkane and Chloroalkene Degradation
1.2.1.-	Naphthalene Degradation|Polycyclic Aromatic Hydrocarbon Degradation
1.2.1.10	Benzoate Degradation|Dioxin Degradation|Xylene Degradation
1.2.1.28	Aminobenzoate Degradation|Toluene Degradation|Xylene Degradation
1.2.1.29	Xylene Degradation
1.2.1.3	Chloroalkane and Chloroalkene Degradation
1.2.1.39	Styrene Degradation
1.2.1.4	Caprolactam Degradation
1.2.1.46	Chloroalkane and Chloroalkene Degradation
1.2.1.5	Felbamate Cyclophosphamide and Ifosfamide Degradation|Metabolism of Xenobiotics- CYP450
1.2.1.61	Chlorocyclohexane and Chlorobenzene Degradation
1.2.1.62	Toluene Degradation
1.2.1.63	Caprolactam Degradation
1.2.1.64	Aminobenzoate Degradation|Toluene Degradation
1.2.1.65	Naphthalene Degradation
1.2.1.67	Aminobenzoate Degradation
1.2.1.7	Aminobenzoate Degradation|Toluene Degradation|Xylene Degradation
1.2.1.78	Polycyclic Aromatic Hydrocarbon Degradation
1.2.1.85	Benzoate Degradation|Xylene Degradation
1.2.3.-	Naphthalene Degradation
1.2.3.1	Citalopram Degradation
1.2.7.1	Nitrotoluene Degradation
1.2.99.2	Nitrotoluene Degradation
1.2.99.4	Chloroalkane and Chloroalkene Degradation
1.3.1.	Xylene Degradation
1.3.1.-	Chlorocyclohexane and Chlorobenzene Degradation|DDT Degradation|Fluorobenzoate Degradation|Polycyclic Aromatic Hydrocarbon Degradation|Toluene Degradation
1.3.1.19	Benzoate Degradation|Chlorocyclohexane and Chlorobenzene Degradation|Polycyclic Aromatic Hydrocarbon Degradation|Styrene Degradation|Toluene Degradation
1.3.1.2	Fluorouracil Degradation
1.3.1.20	Metabolism of Xenobiotics- CYP450

1.3.1.25	Benzoate Degradation|Fluorobenzoate Degradation
1.3.1.29	Naphthalene Degradation|Polycyclic Aromatic Hydrocarbon Degradation
1.3.1.32	Benzoate Degradation|Chlorocyclohexane and Chlorobenzene Degradation|Fluorobenzoate Degradation|Toluene Degradation
1.3.1.49	Polycyclic Aromatic Hydrocarbon Degradation
1.3.1.53	Polycyclic Aromatic Hydrocarbon Degradation
1.3.1.56	Dioxin Degradation
1.3.1.57	Aminobenzoate Degradation
1.3.1.58	Xylene Degradation
1.3.1.62	Benzoate Degradation
1.3.1.63	Fluorobenzoate Degradation
1.3.1.64	Polycyclic Aromatic Hydrocarbon Degradation
1.3.1.66	Ethylbenzene Degradation
1.3.1.67	Xylene Degradation
1.3.1.68	Xylene Degradation
1.3.7.8	Benzoate Degradation
1.3.7.9	Aminobenzoate Degradation|Benzoate Degradation
1.3.8.3	Toluene Degradation
1.3.99.-	Benzoate Degradation|Caprolactam Degradation|Naphthalene Degradation
1.3.99.32	Benzoate Degradation
1.3.99.4	Steroid Degradation
1.3.99.5	Steroid Degradation
1.3.99.8	Furfural Degradation
1.4.3.12	Caprolactam Degradation
1.4.3.4	Citalopram Degradation
1.5.1.37	Chlorocyclohexane and Chlorobenzene Degradation
1.6.5.-	Polycyclic Aromatic Hydrocarbon Degradation
1.6.5.6	Aminobenzoate Degradation
1.6.5.7	Aminobenzoate Degradation|Chlorocyclohexane and Chlorobenzene Degradation
1.7.1.-	Aminobenzoate Degradation|Nitrotoluene Degradation
1.8.99.3	Nitrotoluene Degradation
1.97.1.-	Bisphenol Degradation|Chloroalkane and Chloroalkene Degradation|Chlorocyclohexane and Chlorobenzene Degradation|DDT Degradation
1.97.1.2	Aminobenzoate Degradation
1.97.1.8	Chloroalkane and Chloroalkene Degradation
2.1.1.-	Aminobenzoate Degradation|Polycyclic Aromatic Hydrocarbon Degradation
2.1.1.67	Azathioprine and 6-Mercaptopurine Degradation
2.3.1.-	Aminobenzoate Degradation|Benzoate Degradation|Ethylbenzene Degradation|Naphthalene Degradation|Toluene Degradation
2.3.1.16	Benzoate Degradation|Ethylbenzene Degradation
2.3.1.174	Benzoate Degradation
2.3.1.5	Isoniazid Degradation|Nitrotoluene Degradation
2.3.1.9	Benzoate Degradation
2.4.1.17	Codeine and Morphine Degradation|Irinotecan Degradation|Metabolism of Xenobiotics- CYP450
2.4.2.10	Fluorouracil Degradation
2.4.2.3	Fluorouracil Degradation
2.4.2.4	Fluorouracil Degradation

2.4.2.8	Azathioprine and 6-Mercaptopurine Degradation
2.5.1.-	Chlorocyclohexane and Chlorobenzene Degradation
2.5.1.18	Cyclophosphamide and Ifosfamide Degradation|Metabolism of Xenobiotics- CYP450
2.6.1.-	Caprolactam Degradation
2.7.1.21	Fluorouracil Degradation
2.7.1.48	Fluorouracil Degradation
2.7.4.-	Azathioprine and 6- Mercaptopurine Degradation
2.7.9.-	Aminobenzoate Degradation
2.8.2.-	Polycyclic Aromatic Hydrocarbon Degradation
2.8.2.14	Metabolism of Xenobiotics- CYP450
2.8.3.-	Naphthalene Degradation
2.8.3.1	Styrene Degradation
2.8.3.12	Styrene Degradation
2.8.3.15	Toluene Degradation
2.8.3.6	Benzoate Degradation
2.8.3.8	Aminobenzoate Degradation
3.1.1.-	Bisphenol Degradation|Furfural Degradation|Toluene Degradation
3.1.1.1	Fluorouracil and Irinotecan Degradation
3.1.1.17	Caprolactam Degradation
3.1.1.2	Bisphenol Degradation
3.1.1.24	Benzoate Degradation
3.1.1.35	Polycyclic Aromatic Hydrocarbon Degradation
3.1.1.45	Chlorocyclohexane and Chlorobenzene Degradation|Fluorobenzoate Degradation|Toluene Degradation
3.1.1.57	Aminobenzoate Degradation|Benzoate Degradation
3.1.1.92	Aminobenzoate Degradation
3.1.2.-	Benzoate Degradation
3.1.2.23	Benzoate Degradation
3.1.3.1	Aminobenzoate Degradation
3.1.3.2	Aminobenzoate Degradation
3.1.3.41	Aminobenzoate Degradation
3.1.8.1	Aminobenzoate Degradation
3.10.1.2	Caprolactam Degradation
3.2.1.31	Irinotecan Degradation
3.3.2.-	Polycyclic Aromatic Hydrocarbon Degradation
3.3.2.10	Chloroalkane and Chloroalkene Degradation
3.3.2.9	Metabolism of Xenobiotics- CYP450
3.5.1.-	Caprolactam Degradation|Fluorobenzoate Degradation
3.5.1.4	Aminobenzoate Degradation|Styrene Degradation
3.5.1.5	Atrazine Degradation
3.5.1.54	Atrazine Degradation
3.5.1.6	Fluorouracil Degradation
3.5.1.84	Atrazine Degradation
3.5.2.-	Caprolactam Degradation
3.5.2.15	Atrazine Degradation
3.5.2.2	Fluorouracil Degradation
3.5.4.-	Atrazine Degradation

3.5.4.5	Fluorouracil Degradation
3.5.5.1	Aminobenzoate Degradation|Styrene Degradation
3.5.5.6	Fluorobenzoate Degradation
3.5.5.7	Styrene Degradation
3.5.99.-	Atrazine Degradation
3.5.99.3	Atrazine Degradation
3.5.99.4	Atrazine Degradation
3.6.1.19	Azathioprine and 6-Mercaptopurine Degradation
3.6.1.7	Aminobenzoate Degradation
3.7.1.-	Aminobenzoate Degradation|Benzoate Degradation|DDT Degradation|Dioxin Degradation|Ethylbenzene Degradation|Nitrotoluene Degradation|Xylene Degradation
3.7.1.11	Caprolactam Degradation
3.7.1.17	Steroid Degradation
3.7.1.2	Styrene Degradation
3.7.1.8	Dioxin Degradation
3.7.1.9	Benzoate Degradation|Styrene Degradation|Xylene Degradation
3.8.1.-	Atrazine Degradation|Chloroalkane and Chloroalkene Degradation
3.8.1.2	Chloroalkane and Chloroalkene Degradation|Chlorocyclohexane and Chlorobenzene Degradation
3.8.1.3	Chloroalkane and Chloroalkene Degradation|Chlorocyclohexane and Chlorobenzene Degradation
3.8.1.5	Chloroalkane and Chloroalkene Degradation|Chlorocyclohexane and Chlorobenzene Degradation
3.8.1.7	Fluorobenzoate Degradation
3.8.1.8	Atrazine Degradation
4.1.1.-	Benzoate Degradation|Chloroalkane and Chloroalkene Degradation|DDT Degradation|Dioxin Degradation|Naphthalene Degradation|Polycyclic Aromatic Hydrocarbon Degradation|Xylene Degradation
4.1.1.44	Benzoate Degradation
4.1.1.46	Aminobenzoate Degradation
4.1.1.55	Polycyclic Aromatic Hydrocarbon Degradation
4.1.1.59	Aminobenzoate Degradation
4.1.1.61	Aminobenzoate Degradation
4.1.1.63	Benzoate Degradation
4.1.1.69	Polycyclic Aromatic Hydrocarbon Degradation
4.1.1.7	Aminobenzoate Degradation
4.1.1.70	Benzoate Degradation
4.1.1.77	Benzoate Degradation|Dioxin Degradation|Xylene Degradation
4.1.2.-	Polycyclic Aromatic Hydrocarbon Degradation
4.1.2.34	Polycyclic Aromatic Hydrocarbon Degradation
4.1.2.45	Naphthalene Degradation
4.1.3.-	Polycyclic Aromatic Hydrocarbon Degradation
4.1.3.17	Benzoate Degradation
4.1.3.39	Benzoate Degradation|Dioxin Degradation|Xylene Degradation
4.1.99.-	Naphthalene Degradation
4.1.99.11	Toluene Degradation
4.2.1.-	Benzoate Degradation|Bisphenol Degradation|Naphthalene Degradation|Toluene Degradation
4.2.1.100	Benzoate Degradation
4.2.1.103	Caprolactam Degradation
4.2.1.112	Chloroalkane and Chloroalkene Degradation

4.2.1.17	Aminobenzoate Degradation|Benzoate Degradation|Caprolactam Degradation
4.2.1.54	Styrene Degradation
4.2.1.69	Atrazine Degradation
4.2.1.80	Benzoate Degradation|Dioxin Degradation|Xylene Degradation
4.2.1.83	Benzoate Degradation
4.2.1.84	Aminobenzoate Degradation|Fluorobenzoate Degradation|Styrene Degradation
4.5.1.-	Chlorocyclohexane and Chlorobenzene Degradation|DDT Degradation
4.5.1.1	DDT Degradation
4.5.1.3	Chloroalkane and Chloroalkene Degradation
4.99.1.7	Styrene Degradation
5.1.2.-	Polycyclic Aromatic Hydrocarbon Degradation
5.1.2.2	Aminobenzoate Degradation
5.2.1.10	Chlorocyclohexane and Chlorobenzene Degradation
5.2.1.2	Styrene Degradation
5.3.2.6	Benzoate Degradation|Dioxin Degradation|Xylene Degradation
5.3.2.8	Benzoate Degradation
5.3.3.1	Steroid Degradation
5.3.3.4	Benzoate Degradation
5.3.99.-	Naphthalene Degradation
5.3.99.7	Styrene Degradation
5.4.4.1	Aminobenzoate Degradation
5.4.4.3	Aminobenzoate Degradation
5.4.99.-	Aminobenzoate Degradation
5.4.99.14	Toluene Degradation
5.5.1.-	Aminobenzoate Degradation|Toluene Degradation
5.5.1.1	Benzoate Degradation|Chlorocyclohexane and Chlorobenzene Degradation|Fluorobenzoate Degradation|Toluene Degradation
5.5.1.11	Chlorocyclohexane and Chlorobenzene Degradation
5.5.1.2	Benzoate Degradation
5.5.1.5	Benzoate Degradation
5.5.1.7	Chlorocyclohexane and Chlorobenzene Degradation|Fluorobenzoate Degradation|Toluene Degradation
5.99.1.4	Naphthalene Degradation
6.2.1.-	Aminobenzoate Degradation|Benzoate Degradation|Caprolactam Degradation|Ethylbenzene Degradation|Fluorobenzoate Degradation
6.2.1.25	Aminobenzoate Degradation|Benzoate Degradation
6.2.1.27	Aminobenzoate Degradation|Benzoate Degradation
6.2.1.31	Furfural Degradation
6.2.1.32	Aminobenzoate Degradation
6.2.1.33	Fluorobenzoate Degradation
6.3.5.2	Azathioprine and 6-Mercaptopurine Degradation
6.4.1.-	Aminobenzoate Degradation
6.4.1.8	Ethylbenzene Degradation
K00002	Caprolactam Degradation
K00022	Caprolactam Degradation
K00055	Toluene Degradation|Xylene Degradation | Caprolactam Degradation
K00074	Benzoate Degradation

K00079	Metabolism of Xenobiotics- CYP450
K00088	Azathioprine and 6-Mercaptopurine Degradation
K00100	Bisphenol Degradation|Chloroalkane and Chloroalkene Degradation
K00106	Azathioprine and 6-Mercaptopurine Degradation
K00114	Chloroalkane and Chloroalkene Degradation
K00128	Chloroalkane and Chloroalkene Degradation
K00129	Felbamate Cyclophosphamide and Ifosfamide Degradation|Metabolism of Xenobiotics- CYP450
K00132	Benzoate Degradation|Dioxin Degradation|Xylene Degradation
K00141	Aminobenzoate Degradation|Toluene Degradation|Xylene Degradation
K00146	Styrene Degradation
K00148	Chloroalkane and Chloroalkene Degradation
K00152	Naphthalene Degradation
K00155	Naphthalene Degradation
K00157	Citalopram Degradation
K00169	Nitrotoluene Degradation
K00207	Fluorouracil Degradation
K00212	Metabolism of Xenobiotics- CYP450
K00217	Benzoate Degradation|Chlorocyclohexane and Chlorobenzene Degradation|Fluorobenzoate Degradation|Toluene Degradation
K00224	Polycyclic Aromatic Hydrocarbon Degradation|Chlorocyclohexane and Chlorobenzene Degradation|DDT Degradation
K00274	Citalopram Degradation
K00446	Benzoate Degradation|Chlorocyclohexane and Chlorobenzene Degradation|Styrene Degradation|Xylene Degradation
K00448	Polycyclic Aromatic Hydrocarbon Degradation|Benzoate Degradation
K00451	Styrene Degradation
K00462	Chlorocyclohexane and Chlorobenzene Degradation|Dioxin Degradation
K00480	Dioxin Degradation|Naphthalene Degradation|Polycyclic Aromatic Hydrocarbon Degradation
K00481	Benzoate Degradation
K00485	Tamoxifen Degradation
K00492	Bisphenol Degradation|Naphthalene Degradation|Polycyclic Aromatic Hydrocarbon Degradation
K00493	Aminobenzoate Degradation
K00531	Chloroalkane and Chloroalkene Degradation
K00539	Bisphenol Degradation|Chloroalkane and Chloroalkene Degradation|Chlorocyclohexane and Chlorobenzene Degradation|DDT Degradation
K00569	Azathioprine and 6-Mercaptopurine Degradation
K00599	Polycyclic Aromatic Hydrocarbon Degradation
K00622	Isoniazid Degradation|Nitrotoluene Degradation
K00626	Benzoate Degradation
K00632	Benzoate Degradation|Ethylbenzene Degradation
K00680	Aminobenzoate Degradation|Benzoate Degradation|Ethylbenzene Degradation
K00699	Codeine and Morphine Degradation|Irinotecan Degradation
K00757	Fluorouracil Degradation
K00758	Fluorouracil Degradation
K00760	Azathioprine and 6-Mercaptopurine Degradation
K00799	Cyclophosphamide and Ifosfamide Degradation|Metabolism of Xenobiotics- CYP450
K00857	Fluorouracil Degradation

K00876	Fluorouracil Degradation
K01026	Styrene Degradation
K01031	Benzoate Degradation
K01034	Aminobenzoate Degradation
K01039	Styrene Degradation
K01041	Naphthalene Degradation
K01044	Fluorouracil and Irinotecan Degradation
K01045	Bisphenol Degradation|Aminobenzoate Degradation
K01053	Caprolactam Degradation
K01055	Benzoate Degradation
K01061	Chlorocyclohexane and Chlorobenzene Degradation|Fluorobenzoate Degradation|Toluene Degradation
K01066	Bisphenol Degradation
K01075	Benzoate Degradation
K01077	Aminobenzoate Degradation
K01078	Aminobenzoate Degradation
K01101	Aminobenzoate Degradation
K01195	Irinotecan Degradation
K01253	Metabolism of Xenobiotics- CYP450
K01426	Aminobenzoate Degradation|Styrene Degradation
K01428	Atrazine Degradation
K01431	Fluorouracil Degradation
K01457	Atrazine Degradation
K01464	Fluorouracil Degradation
K01489	Fluorouracil Degradation
K01500	Atrazine Degradation
K01501	Aminobenzoate Degradation|Styrene Degradation
K01502	Styrene Degradation
K01512	Aminobenzoate Degradation
K01519	Azathioprine and 6-Mercaptopurine Degradation
K01555	Styrene Degradation
K01560	Chlorocyclohexane and Chlorobenzene Degradation | Chloroalkane and Chloroalkene Degradation
K01561	Chloroalkane and Chloroalkene Degradation|Chlorocyclohexane and Chlorobenzene Degradation
K01563	Chloroalkane and Chloroalkene Degradation|Chlorocyclohexane and Chlorobenzene Degradation
K01564	Atrazine Degradation|Chloroalkane and Chloroalkene Degradation
K01576	Aminobenzoate Degradation
K01607	Benzoate Degradation
K01612	Aminobenzoate Degradation
K01615	Benzoate Degradation
K01617	Benzoate Degradation|Dioxin Degradation|Xylene Degradation
K01666	Benzoate Degradation|Dioxin Degradation|Xylene Degradation
K01670	Naphthalene Degradation
K01692	Benzoate Degradation|Caprolactam Degradation | Aminobenzoate Degradation
K01721	Aminobenzoate Degradation|Fluorobenzoate Degradation|Styrene Degradation
K01726	Benzoate Degradation|Bisphenol Degradation|Naphthalene Degradation
K01781	Aminobenzoate Degradation
K01800	Styrene Degradation

K01821	Benzoate Degradation|Dioxin Degradation|Xylene Degradation
K01822	Steroid Degradation
K01856	Benzoate Degradation|Chlorocyclohexane and Chlorobenzene Degradation|Fluorobenzoate Degradation|Toluene Degradation
K01857	Benzoate Degradation
K01860	Chlorocyclohexane and Chlorobenzene Degradation|Fluorobenzoate Degradation|Toluene Degradation
K01913	Aminobenzoate Degradation|Caprolactam Degradation
K01951	Azathioprine and 6-Mercaptopurine Degradation
K02554	Benzoate Degradation|Dioxin Degradation|Xylene Degradation
K03268	Chloroalkane and Chloroalkene Degradation|Toluene Degradation|Benzoate Degradation|Chlorocyclohexane and Chlorobenzene Degradation
K03333	Steroid Degradation
K03379	Caprolactam Degradation
K03380	Chlorocyclohexane and Chlorobenzene Degradation |Aminobenzoate Degradation|Toluene Degradation
K03381	Benzoate Degradation|Chlorocyclohexane and Chlorobenzene Degradation|Fluorobenzoate Degradation|Toluene Degradation
K03382	Atrazine Degradation
K03383	Atrazine Degradation
K03384	Chlorocyclohexane and Chlorobenzene Degradation|Naphthalene Degradation|Polycyclic Aromatic Hydrocarbon Degradation|Toluene Degradation
K03391	Chlorocyclohexane and Chlorobenzene Degradation|Fluorobenzoate Degradation
K03464	Benzoate Degradation
K03518	Nitrotoluene Degradation
K03862	Aminobenzoate Degradation
K04098	Benzoate Degradation|Chlorocyclohexane and Chlorobenzene Degradation
K04099	Aminobenzoate Degradation
K04100	Aminobenzoate Degradation|Benzoate Degradation|Polycyclic Aromatic Hydrocarbon Degradation
K04102	Polycyclic Aromatic Hydrocarbon Degradation
K04105	Aminobenzoate Degradation|Benzoate Degradation
K04107	Benzoate Degradation | Aminobenzoate Degradation
K04110	Aminobenzoate Degradation|Benzoate Degradation
K04112	Benzoate Degradation
K04116	Benzoate Degradation
K04117	Benzoate Degradation
K04118	Benzoate Degradation
K05296	Steroid Degradation
K05394	Atrazine Degradation
K05549	Xylene Degradation|Benzoate Degradation|Fluorobenzoate Degradation
K05599	Aminobenzoate Degradation
K05783	Xylene Degradation|Benzoate Degradation|Fluorobenzoate Degradation
K05797	Toluene Degradation
K05898	Steroid Degradation
K05913	Bisphenol Degradation
K06035	Atrazine Degradation
K06281	Nitrotoluene Degradation

K06446	Caprolactam Degradation
K06912	Chlorocyclohexane and Chlorobenzene Degradation
K07408	Metabolism of Xenobiotics- CYP450
K07535	Benzoate Degradation
K07536	Benzoate Degradation
K07537	Benzoate Degradation
K07538	Benzoate Degradation
K07540	Toluene Degradation
K07543	Toluene Degradation
K07545	Toluene Degradation
K07546	Toluene Degradation
K07547	Toluene Degradation
K07549	Toluene Degradation
K07823	Benzoate Degradation
K07824	Aminobenzoate Degradation|Benzoate Degradation
K08295	Aminobenzoate Degradation
K08686	Fluorobenzoate Degradation
K08689	Dioxin Degradation
K08690	Dioxin Degradation
K08710	Atrazine Degradation
K08726	Chloroalkane and Chloroalkene Degradation
K09461	Aminobenzoate Degradation
K10215	Aminobenzoate Degradation
K10216	Benzoate Degradation|Styrene Degradation|Xylene Degradation
K10217	Benzoate Degradation|Xylene Degradation
K10218	Benzoate Degradation
K10219	Aminobenzoate Degradation|Benzoate Degradation
K10220	Benzoate Degradation
K10221	Aminobenzoate Degradation|Benzoate Degradation
K10222	Dioxin Degradation
K10437	Styrene Degradation
K10438	Styrene Degradation
K10566	Styrene Degradation
K10616	Xylene Degradation
K10617	Xylene Degradation
K10618	Xylene Degradation
K10619	Xylene Degradation
K10620	Xylene Degradation
K10621	Xylene Degradation|Benzoate Degradation
K10622	Benzoate Degradation|Xylene Degradation
K10623	Xylene Degradation
K10676	Chlorocyclohexane and Chlorobenzene Degradation
K10700	Ethylbenzene Degradation
K10701	Ethylbenzene Degradation
K10702	Xylene Degradation
K11180	Nitrotoluene Degradation
K11822	Metabolism of Xenobiotics- CYP450

K11946	Polycyclic Aromatic Hydrocarbon Degradation
K11947	Polycyclic Aromatic Hydrocarbon Degradation
K11948	Polycyclic Aromatic Hydrocarbon Degradation
K11949	Polycyclic Aromatic Hydrocarbon Degradation
K13421	Fluorouracil Degradation
K13951	Felbamate Cyclophosphamide and Ifosfamide Degradation | Metabolism of Xenobiotics- CYP450
K13953	Chloroalkane and Chloroalkene Degradation|Naphthalene Degradation
K14028	Chloroalkane and Chloroalkene Degradation
K14333	Aminobenzoate Degradation
K14334	Benzoate Degradation
K14417	Fluorobenzoate Degradation
K14418	Fluorobenzoate Degradation
K14419	Chloroalkane and Chloroalkene Degradation
K14421	Chloroalkane and Chloroalkene Degradation
K14422	Chloroalkane and Chloroalkene Degradation
K14481	Styrene Degradation
K14519	Caprolactam Degradation
K14520	Bisphenol Degradation
K14542	Atrazine Degradation
K14579	Ethylbenzene Degradation|Naphthalene Degradation|Polycyclic Aromatic Hydrocarbon Degradation
K14582	Naphthalene Degradation|Polycyclic Aromatic Hydrocarbon Degradation
K14583	Naphthalene Degradation
K14584	Naphthalene Degradation
K14585	Naphthalene Degradation
K14586	Naphthalene Degradation
K14599	Dioxin Degradation|Polycyclic Aromatic Hydrocarbon Degradation
K14601	Polycyclic Aromatic Hydrocarbon Degradation
K14602	Polycyclic Aromatic Hydrocarbon Degradation
K14604	Polycyclic Aromatic Hydrocarbon Degradation
K14746	Ethylbenzene Degradation
K14747	Ethylbenzene Degradation
K14748	Ethylbenzene Degradation
K14751	Ethylbenzene Degradation
K15054	Aminobenzoate Degradation
K15055	Aminobenzoate Degradation
K15056	Aminobenzoate Degradation
K15057	Aminobenzoate Degradation
K15058	Aminobenzoate Degradation
K15060	Aminobenzoate Degradation
K15061	Aminobenzoate Degradation
K15062	Aminobenzoate Degradation
K15063	Aminobenzoate Degradation
K15064	Aminobenzoate Degradation
K15065	Aminobenzoate Degradation
K15066	Aminobenzoate Degradation
K15236	Chlorocyclohexane and Chlorobenzene Degradation
K15237	Chlorocyclohexane and Chlorobenzene Degradation

K15238	Chlorocyclohexane and Chlorobenzene Degradation
K15239	Chlorocyclohexane and Chlorobenzene Degradation
K15240	Chlorocyclohexane and Chlorobenzene Degradation
K15241	Chlorocyclohexane and Chlorobenzene Degradation
K15242	Chlorocyclohexane and Chlorobenzene Degradation
K15243	Chlorocyclohexane and Chlorobenzene Degradation
K15244	Chlorocyclohexane and Chlorobenzene Degradation
K15245	Chlorocyclohexane and Chlorobenzene Degradation
K15246	Chlorocyclohexane and Chlorobenzene Degradation
K15247	Chlorocyclohexane and Chlorobenzene Degradation
K15248	Chlorocyclohexane and Chlorobenzene Degradation
K15252	Chlorocyclohexane and Chlorobenzene Degradation
K15253	Chlorocyclohexane and Chlorobenzene Degradation
K15569	Naphthalene Degradation
K15571	Naphthalene Degradation
K15572	Naphthalene Degradation
K15573	Naphthalene Degradation
K15574	Naphthalene Degradation
K15680	Metabolism of Xenobiotics- CYP450
K15749	Dioxin Degradation
K15751	Dioxin Degradation
K15754	Dioxin Degradation
K15756	Dioxin Degradation
K15757	Toluene Degradation|Xylene Degradation
K15766	Toluene Degradation
K15768	Toluene Degradation
K15981	Steroid Degradation
K15982	Steroid Degradation
K16045	Steroid Degradation
K16047	Steroid Degradation
K16049	Steroid Degradation
K16050	Steroid Degradation
K16051	Steroid Degradation
K16173	Benzoate Degradation
K16249	Benzoate Degradation|Chlorocyclohexane and Chlorobenzene Degradation|Toluene Degradation
K16269	Benzoate Degradation|Chlorocyclohexane and Chlorobenzene Degradation|Styrene Degradation|Toluene Degradation | Polycyclic Aromatic Hydrocarbon Degradation
K16270	Xylene Degradation
K16514	Benzoate Degradation
K16873	Furfural Degradation
K16874	Furfural Degradation
K16876	Furfural Degradation
K16877	Furfural Degradation
K16880	Furfural Degradation
K17067	Chloroalkane and Chloroalkene Degradation
K17070	Chloroalkane and Chloroalkene Degradation
K18067	Polycyclic Aromatic Hydrocarbon Degradation

K18068	Polycyclic Aromatic Hydrocarbon Degradation
K18074	Polycyclic Aromatic Hydrocarbon Degradation
K18076	Polycyclic Aromatic Hydrocarbon Degradation
K18092	Ethylbenzene Degradation
K18199	Caprolactam Degradation
K18242	Naphthalene Degradation
K18256	Polycyclic Aromatic Hydrocarbon Degradation
K18257	Polycyclic Aromatic Hydrocarbon Degradation
K18275	Polycyclic Aromatic Hydrocarbon Degradation
K18312	Styrene Degradation
