# Supplementary material for: Xenobiotic Metabolism and Gut Microbiomes
Source: PLoS One. 2016 Oct 3;11(10):e0163099. doi: 10.1371/journal.pone.0163099 (PMC5047465; doi:10.1371/journal.pone.0163099)
Supplement: S13 Table — (PDF) [file pone.0163099.s032.pdf]

Sample ID	Region	Age	Age Group		Sample ID	Region	Age	Age Group		Sample ID	Region	Age	Age Group
AM-AD-10	American	N/A	N/A		DOF007	Chinese	70	60_above		DA-AD-40	Danish	59	50_to_60
AM-AD-11	American	N/A	N/A		DOF008	Chinese	40	40_to_50		DA-AD-41	Danish	49	40_to_50
AM-AD-12	American	N/A	N/A		DOF009	Chinese	68	60_above		DA-AD-42	Danish	69	60_above
AM-AD-13	American	N/A	N/A		DOF010	Chinese	62	60_above		DA-AD-43	Danish	64	60_above
AM-AD-14	American	N/A	N/A		DOF011	Chinese	48	40_to_50		DA-AD-44	Danish	59	50_to_60
AM-AD-15	American	N/A	N/A		DOF012	Chinese	61	60_above		DA-AD-45	Danish	54	50_to_60
AM-AD-16	American	N/A	N/A		DOF013	Chinese	53	50_to_60		DA-AD-46	Danish	69	60_above
AM-AD-17	American	N/A	N/A		DOF014	Chinese	63	60_above		DA-AD-47	Danish	54	50_to_60
AM-AD-18	American	N/A	N/A		DOM001	Chinese	59	50_to_60		DA-AD-48	Danish	44	40_to_50
AM-AD-19	American	N/A	N/A		DOM003	Chinese	53	50_to_60		DA-AD-49	Danish	49	40_to_50
AM-AD-20	American	N/A	N/A		DOM005	Chinese	49	40_to_50		DA-AD-5	Danish	49	40_to_50
AM-AD-21	American	N/A	N/A		DOM008	Chinese	75	60_above		DA-AD-50	Danish	69	60_above
AM-AD-22	American	N/A	N/A		DOM010	Chinese	74	60_above		DA-AD-51	Danish	49	40_to_50
AM-AD-23	American	N/A	N/A		DOM012	Chinese	57	50_to_60		DA-AD-52	Danish	49	40_to_50
AM-AD-24	American	N/A	N/A		DOM013	Chinese	36	30_to_40		DA-AD-53	Danish	49	40_to_50
AM-AD-25	American	N/A	N/A		DOM014	Chinese	71	60_above		DA-AD-54	Danish	59	50_to_60
AM-AD-26	American	N/A	N/A		DOM015	Chinese	68	60_above		DA-AD-55	Danish	54	50_to_60
AM-AD-27	American	N/A	N/A		DOM016	Chinese	39	30_to_40		DA-AD-56	Danish	54	50_to_60
AM-AD-28	American	N/A	N/A		DOM017	Chinese	41	40_to_50		DA-AD-57	Danish	54	50_to_60
AM-AD-29	American	N/A	N/A		DOM018	Chinese	68	60_above		DA-AD-58	Danish	59	50_to_60
AM-AD-3	American	N/A	N/A		DOM019	Chinese	53	50_to_60		DA-AD-59	Danish	54	50_to_60
AM-AD-30	American	N/A	N/A		DOM020	Chinese	43	40_to_50		DA-AD-6	Danish	59	50_to_60
AM-AD-31	American	N/A	N/A		DOM021	Chinese	72	60_above		DA-AD-60	Danish	69	60_above
AM-AD-32	American	N/A	N/A		DOM022	Chinese	31	30_to_40		DA-AD-61	Danish	49	40_to_50
AM-AD-33	American	N/A	N/A		DOM023	Chinese	39	30_to_40		DA-AD-62	Danish	59	50_to_60
AM-AD-34	American	N/A	N/A		DOM024	Chinese	42	40_to_50		DA-AD-63	Danish	54	50_to_60
AM-AD-35	American	N/A	N/A		DOM025	Chinese	62	60_above		DA-AD-64	Danish	59	50_to_60
AM-AD-36	American	N/A	N/A		DOM026	Chinese	36	30_to_40		DA-AD-65	Danish	44	40_to_50
AM-AD-37	American	N/A	N/A		NLF001	Chinese	31	30_to_40		DA-AD-66	Danish	54	50_to_60
AM-AD-38	American	N/A	N/A		NLF002	Chinese	34	30_to_40		DA-AD-67	Danish	54	50_to_60
AM-AD-39	American	N/A	N/A		NLF005	Chinese	44	40_to_50		DA-AD-68	Danish	59	50_to_60
AM-AD-4	American	N/A	N/A		NLF006	Chinese	27	10_to_30		DA-AD-69	Danish	49	40_to_50
AM-AD-40	American	N/A	N/A		NLF007	Chinese	39	30_to_40		DA-AD-7	Danish	69	60_above
AM-AD-41	American	N/A	N/A		NLF008	Chinese	26	10_to_30		DA-AD-70	Danish	44	40_to_50
AM-AD-42	American	N/A	N/A		NLF009	Chinese	27	10_to_30		DA-AD-71	Danish	64	60_above
AM-AD-43	American	N/A	N/A		NLF010	Chinese	35	30_to_40		DA-AD-72	Danish	54	50_to_60

Sample ID	Region	Age	Age Group		Sample ID	Region	Age	Age Group		Sample ID	Region	Age	Age Group
AM-AD-44	American	N/A	N/A		NLF011	Chinese	25	10_to_30		DA-AD-73	Danish	49	40_to_50
AM-AD-45	American	N/A	N/A		NLF012	Chinese	27	10_to_30		DA-AD-74	Danish	64	60_above
AM-AD-46	American	N/A	N/A		NLF013	Chinese	32	30_to_40		DA-AD-75	Danish	69	60_above
AM-AD-47	American	N/A	N/A		NLF014	Chinese	37	30_to_40		DA-AD-76	Danish	49	40_to_50
AM-AD-48	American	N/A	N/A		NLF015	Chinese	59	50_to_60		DA-AD-77	Danish	49	40_to_50
AM-AD-49	American	N/A	N/A		NLM001	Chinese	32	30_to_40		DA-AD-78	Danish	64	60_above
AM-AD-5	American	N/A	N/A		NLM002	Chinese	23	10_to_30		DA-AD-79	Danish	59	50_to_60
AM-AD-50	American	N/A	N/A		NLM003	Chinese	23	10_to_30		DA-AD-8	Danish	59	50_to_60
AM-AD-51	American	N/A	N/A		NLM004	Chinese	24	10_to_30		DA-AD-80	Danish	49	40_to_50
AM-AD-52	American	N/A	N/A		NLM005	Chinese	42	40_to_50		DA-AD-81	Danish	59	50_to_60
AM-AD-53	American	N/A	N/A		NLM006	Chinese	24	10_to_30		DA-AD-82	Danish	54	50_to_60
AM-AD-54	American	N/A	N/A		NLM007	Chinese	22	10_to_30		DA-AD-83	Danish	64	60_above
AM-AD-55	American	N/A	N/A		NLM008	Chinese	22	10_to_30		DA-AD-84	Danish	59	50_to_60
AM-AD-56	American	N/A	N/A		NLM009	Chinese	24	10_to_30		DA-AD-85	Danish	59	50_to_60
AM-AD-57	American	N/A	N/A		NLM010	Chinese	23	10_to_30		DA-AD-9	Danish	64	60_above
AM-AD-58	American	N/A	N/A		NLM015	Chinese	23	10_to_30		ES-AD-10	Spanish	47	40_to_50
AM-AD-59	American	N/A	N/A		NLM016	Chinese	23	10_to_30		ES-AD-11	Spanish	56	50_to_60
AM-AD-6	American	N/A	N/A		NLM017	Chinese	23	10_to_30		ES-AD-12	Spanish	48	40_to_50
AM-AD-60	American	N/A	N/A		NLM021	Chinese	25	10_to_30		ES-AD-13	Spanish	42	40_to_50
AM-AD-61	American	N/A	N/A		NLM022	Chinese	29	10_to_30		ES-AD-14	Spanish	51	50_to_60
AM-AD-62	American	N/A	N/A		NLM023	Chinese	38	30_to_40		ES-AD-15	Spanish	49	40_to_50
AM-AD-63	American	N/A	N/A		NLM024	Chinese	23	10_to_30		ES-AD-16	Spanish	44	40_to_50
AM-AD-64	American	N/A	N/A		NLM025	Chinese	19	10_to_30		ES-AD-17	Spanish	44	40_to_50
AM-AD-65	American	N/A	N/A		NLM026	Chinese	24	10_to_30		ES-AD-18	Spanish	55	50_to_60
AM-AD-66	American	N/A	N/A		NLM027	Chinese	22	10_to_30		ES-AD-19	Spanish	62	60_above
AM-AD-67	American	N/A	N/A		NLM028	Chinese	21	10_to_30		ES-AD-20	Spanish	41	40_to_50
AM-AD-68	American	N/A	N/A		NLM029	Chinese	25	10_to_30		ES-AD-21	Spanish	68	60_above
AM-AD-69	American	N/A	N/A		NLM031	Chinese	33	30_to_40		ES-AD-22	Spanish	41	40_to_50
AM-AD-7	American	N/A	N/A		NLM032	Chinese	39	30_to_40		ES-AD-23	Spanish	34	30_to_40
AM-AD-70	American	N/A	N/A		NOF001	Chinese	45	40_to_50		ES-AD-24	Spanish	18	10_to_30
AM-AD-71	American	N/A	N/A		NOF002	Chinese	51	50_to_60		ES-AD-25	Spanish	46	40_to_50
AM-AD-72	American	N/A	N/A		NOF004	Chinese	33	30_to_40		ES-AD-26	Spanish	36	30_to_40
AM-AD-73	American	N/A	N/A		NOF005	Chinese	32	30_to_40		ES-AD-27	Spanish	51	50_to_60
AM-AD-74	American	N/A	N/A		NOF006	Chinese	35	30_to_40		ES-AD-28	Spanish	48	40_to_50
AM-AD-75	American	N/A	N/A		NOF007	Chinese	32	30_to_40		ES-AD-29	Spanish	45	40_to_50
AM-AD-76	American	N/A	N/A		NOF008	Chinese	30	30_to_40		ES-AD-30	Spanish	51	50_to_60
AM-AD-77	American	N/A	N/A		NOF009	Chinese	26	10_to_30		ES-AD-31	Spanish	53	50_to_60
AM-AD-78	American	N/A	N/A		NOF010	Chinese	40	40_to_50		ES-AD-32	Spanish	25	10_to_30
AM-AD-79	American	N/A	N/A		NOF011	Chinese	30	30_to_40		ES-AD-33	Spanish	41	40_to_50
AM-AD-8	American	N/A	N/A		NOF012	Chinese	38	30_to_40		ES-AD-34	Spanish	63	60_above
AM-AD-80	American	N/A	N/A		NOF013	Chinese	41	40_to_50		ES-AD-35	Spanish	37	30_to_40

Sample ID	Region	Age	Age Group		Sample ID	Region	Age	Age Group		Sample ID	Region	Age	Age Group
AM-AD-81	American	N/A	N/A		NOF014	Chinese	42	40_to_50		ES-AD-36	Spanish	62	60_above
AM-AD-82	American	N/A	N/A		NOM001	Chinese	48	40_to_50		ES-AD-37	Spanish	19	10_to_30
AM-AD-83	American	N/A	N/A		NOM002	Chinese	68	60_above		ES-AD-38	Spanish	22	10_to_30
AM-AD-84	American	N/A	N/A		NOM004	Chinese	32	30_to_40		ES-AD-39	Spanish	32	30_to_40
AM-AD-85	American	N/A	N/A		NOM005	Chinese	46	40_to_50		ES-AD-5	Spanish	37	30_to_40
AM-AD-86	American	N/A	N/A		NOM007	Chinese	72	60_above		ES-AD-6	Spanish	34	30_to_40
AM-AD-87	American	N/A	N/A		NOM008	Chinese	54	50_to_60		ES-AD-7	Spanish	43	40_to_50
AM-AD-88	American	N/A	N/A		NOM009	Chinese	33	30_to_40		ES-AD-8	Spanish	68	60_above
AM-AD-89	American	N/A	N/A		NOM010	Chinese	30	30_to_40		ES-AD-9	Spanish	31	30_to_40
AM-AD-9	American	N/A	N/A		NOM012	Chinese	32	30_to_40		FR-AD-1	French	63	60_above
AM-AD-90	American	N/A	N/A		NOM013	Chinese	42	40_to_50		FR-AD-2	French	61	60_above
AM-AD-91	American	N/A	N/A		NOM014	Chinese	41	40_to_50		FR-AD-3	French	60	60_above
AM-AD-92	American	N/A	N/A		NOM015	Chinese	32	30_to_40		FR-AD-4	French	60	60_above
DLF001	Chinese	59	50_to_60		NOM016	Chinese	37	30_to_40		FR-AD-5	French	64	60_above
DLF002	Chinese	43	40_to_50		NOM017	Chinese	39	30_to_40		FR-AD-6	French	63	60_above
DLF003	Chinese	46	40_to_50		NOM018	Chinese	43	40_to_50		FR-AD-7	French	62	60_above
DLF004	Chinese	25	10_to_30		NOM019	Chinese	26	10_to_30		FR-AD-8	French	60	60_above
DLF005	Chinese	60	60_above		NOM020	Chinese	24	10_to_30		1660SH	Indian	3.1666666667	0_to_10
DLF006	Chinese	71	60_above		NOM022	Chinese	23	10_to_30		1660SK	Indian	4.75	0_to_10
DLF007	Chinese	62	60_above		NOM023	Chinese	46	40_to_50		199SD	Indian	4	0_to_10
DLF008	Chinese	54	50_to_60		NOM025	Chinese	28	10_to_30		199SUBD	Indian	2.5	0_to_10
DLF009	Chinese	53	50_to_60		NOM026	Chinese	52	50_to_60		683PD	Indian	4	0_to_10
DLF010	Chinese	51	50_to_60		NOM027	Chinese	38	30_to_40		683RD	Indian	3	0_to_10
DLF012	Chinese	52	50_to_60		NOM028	Chinese	47	40_to_50		882AK	Indian	3.4166666667	0_to_10
DLF013	Chinese	49	40_to_50		NOM029	Chinese	14	10_to_30		882RK	Indian	5	0_to_10
DLF014	Chinese	61	60_above		DA-AD-10	Danish	69	60_above		976AB	Indian	2.9166666667	0_to_10
DLM001	Chinese	46	40_to_50		DA-AD-11	Danish	59	50_to_60		976DB	Indian	1.5833333333	0_to_10
DLM002	Chinese	29	10_to_30		DA-AD-12	Danish	64	60_above		A1	Indian	4	0_to_10
DLM003	Chinese	41	40_to_50		DA-AD-13	Danish	64	60_above		AA6	Indian	2.75	0_to_10
DLM004	Chinese	38	30_to_40		DA-AD-14	Danish	N/A	N/A		DB2	Indian	3.3333333333	0_to_10
DLM005	Chinese	35	30_to_40		DA-AD-15	Danish	54	50_to_60		IS8	Indian	2.75	0_to_10
DLM006	Chinese	28	10_to_30		DA-AD-16	Danish	59	50_to_60		LB1	Indian	5	0_to_10
DLM007	Chinese	43	40_to_50		DA-AD-17	Danish	49	40_to_50		SAK7	Indian	5	0_to_10
DLM008	Chinese	38	30_to_40		DA-AD-18	Danish	64	60_above		SH3	Indian	5	0_to_10
DLM009	Chinese	30	30_to_40		DA-AD-19	Danish	49	40_to_50		SK5	Indian	4	0_to_10
DLM010	Chinese	37	30_to_40		DA-AD-20	Danish	44	40_to_50		SOH4	Indian	3	0_to_10
DLM011	Chinese	49	40_to_50		DA-AD-21	Danish	63	60_above		U2	Indian	0.4166666667	0_to_10
DLM012	Chinese	55	50_to_60		DA-AD-22	Danish	49	40_to_50		IT-AD-1	Italian	84	60_above
DLM014	Chinese	48	40_to_50		DA-AD-23	Danish	64	60_above		IT-AD-2	Italian	87	60_above
DLM015	Chinese	56	50_to_60		DA-AD-24	Danish	69	60_above		IT-AD-3	Italian	77	60_above
DLM016	Chinese	70	60_above		DA-AD-25	Danish	59	50_to_60		IT-AD-4	Italian	80	60_above

Sample ID	Region	Age	Age Group		Sample ID	Region	Age	Age Group		Sample ID	Region	Age	Age Group
DLM017	Chinese	52	50_to_60		DA-AD-26	Danish	49	40_to_50		IT-AD-5	Italian	70	60_above
DLM018	Chinese	49	40_to_50		DA-AD-27	Danish	49	40_to_50		IT-AD-6	Italian	72	60_above
DLM019	Chinese	47	40_to_50		DA-AD-28	Danish	59	50_to_60		JP-AD-1	Japanese	30	30_to_40
DLM020	Chinese	41	40_to_50		DA-AD-29	Danish	44	40_to_50		JP-AD-2	Japanese	28	10_to_30
DLM021	Chinese	58	50_to_60		DA-AD-30	Danish	69	60_above		JP-AD-3	Japanese	37	30_to_40
DLM022	Chinese	53	50_to_60		DA-AD-31	Danish	69	60_above		JP-AD-4	Japanese	36	30_to_40
DLM023	Chinese	45	40_to_50		DA-AD-32	Danish	59	50_to_60		JP-AD-7	Japanese	45	40_to_50
DLM024	Chinese	39	30_to_40		DA-AD-33	Danish	54	50_to_60		JP-AD-8	Japanese	35	30_to_40
DLM027	Chinese	70	60_above		DA-AD-34	Danish	49	40_to_50		JP-AD-9	Japanese	24	10_to_30
DLM028	Chinese	63	60_above		DA-AD-35	Danish	64	60_above		JP-CH-1	Japanese	3	0_to_10
DOF002	Chinese	56	50_to_60		DA-AD-36	Danish	44	40_to_50		JP-CH-2	Japanese	1.5	0_to_10
DOF003	Chinese	59	50_to_60		DA-AD-37	Danish	54	50_to_60		JP-IN-1	Japanese	0.6	0_to_10
DOF004	Chinese	63	60_above		DA-AD-38	Danish	58	50_to_60		JP-IN-2	Japanese	0.5	0_to_10
DOF006	Chinese	51	50_to_60		DA-AD-39	Danish	67	60_above		JP-IN-3	Japanese	0.3	0_to_10
										JP-IN-4	Japanese	0.3	0_to_10
