# Supplementary material for: Xenobiotic Metabolism and Gut Microbiomes
Source: PLoS One. 2016 Oct 3;11(10):e0163099. doi: 10.1371/journal.pone.0163099 (PMC5047465; doi:10.1371/journal.pone.0163099)
Supplement: S1 Text — (PDF) [file pone.0163099.s033.pdf]

S1 TEXT

The current document describes the two different validation tests performed for identifying whether the various alignment based filters (specifically, percentage identity, subject coverage) used in the current study for expanding the reference database of xenobiotic degrading enzymes, as well as identifying homologues of such enzymes in the gut microbiomes, were sensitive and accurate.

VALIDATION TEST 1

METHODOLOGY: In this test, we selected 11 most frequently occurring E.C numbers in the reference database of xenobiotic degrading enzymes. Further, we also included sequences corresponding to 142 E.C. numbers, that were non xenobiotic degrading, but were functionally related to at least one of the above 11 (xenobiotic degrading) E.C. Numbers (i.e. having same three digits of the E.C number). This created a validation database of around 93568 sequences from 153 E.C numbers that were either xenobiotic degrading or at least functionally related (i.e. having homology to) to at least one major xenobiotic degrading enzyme category. We referred to this database as 'Validation Database 1'. Subsequently, for each sequence, a BLASTp search was performed against a modified version of the Validation Database 1 (obtained after removing all the sequences of the source genome of the sequence). This created a simulated scenario where in the query sequence originated from a genome that was absent in the Validation Database 1. This was repeated for all the sequences in this database. Only those hits were retained where in the subject (i.e. the hit sequence in the BLASTp) had more than 70% of its length covered in the alignment. The hits obtained for all the sequences were then collated The hits thus obtained were then categorized into two groups, namely 'CORRECT' (hits where in the query and the subject or hit sequence belonged to same E.C number) and 'INCORRECT' (hits where in the query and the subject or hit sequence had different E.C numbers). Finally, the distribution of percentage identity between these two category of hits were plotted and compared. It was observed that the maximum number of 'INCORRECT' hits had identities in the range of 30-40%. Less than 1% of 'INCORRECT' hits had identity values of greater than 90%. On the other hand, for 'CORRECT' hits, the identity values steadily increased after 40% (with the maximum between 90-100). This indicates that the identity threshold of 90% used for expanding the database was appropriate. We repeated the above validation further using simulated metagenomic scenarios, where in, for each sequence, sequences from the same species/genus/family/order/class (of the source organism of the sequence) were progressively removed. The pattern observed for these scenarios (namely, SPECIES ABSENT, GENUS ABSENT, FAMILY ABSENT, ORDER ABSENT. CLASS ABSENT) was more or less similar with less than 1% of the 'INCORRECT HITS' having identifies greater than 90%.

RESULTS: As observed in Figure ST1 of this document, the maximum number of 'INCORRECT' hits had identities in the range of 30-40%. Less than 1% of 'INCORRECT' hits had identity values of greater than 90%. On the other hand, for 'CORRECT' hits, the identity values steadily increased after 40% (with the maximum between 90-100). This indicates that the identity threshold of 90% used for expanding the database was appropriate.

Further, for 'CORRECT' hits, the identity values steadily increased after 40% (with the

maximum between 90-100). The percentage of 'INCORRECT HITS' at identity values greater than 40% was observed to be less than 5-6%. However, the sensitivity of detection in this range of 40-100% identity range (i.e. the percentage of 'CORRECT HITS' having identities more than 40%) was observed to be greater than 94%.

The pattern observed for these scenarios (namely, SPECIES ABSENT, GENUS ABSENT, FAMILY ABSENT, ORDER ABSENT. CLASS ABSENT) was more or less similar with less than 10% of the 'INCORRECT HITS' having identifies greater than 40% (Figures ST2-ST6). These results indicate that the 40% identity cut-off is an appropriate threshold that maximizes the detection sensitivity, while minimizing the false positive rate of detection.

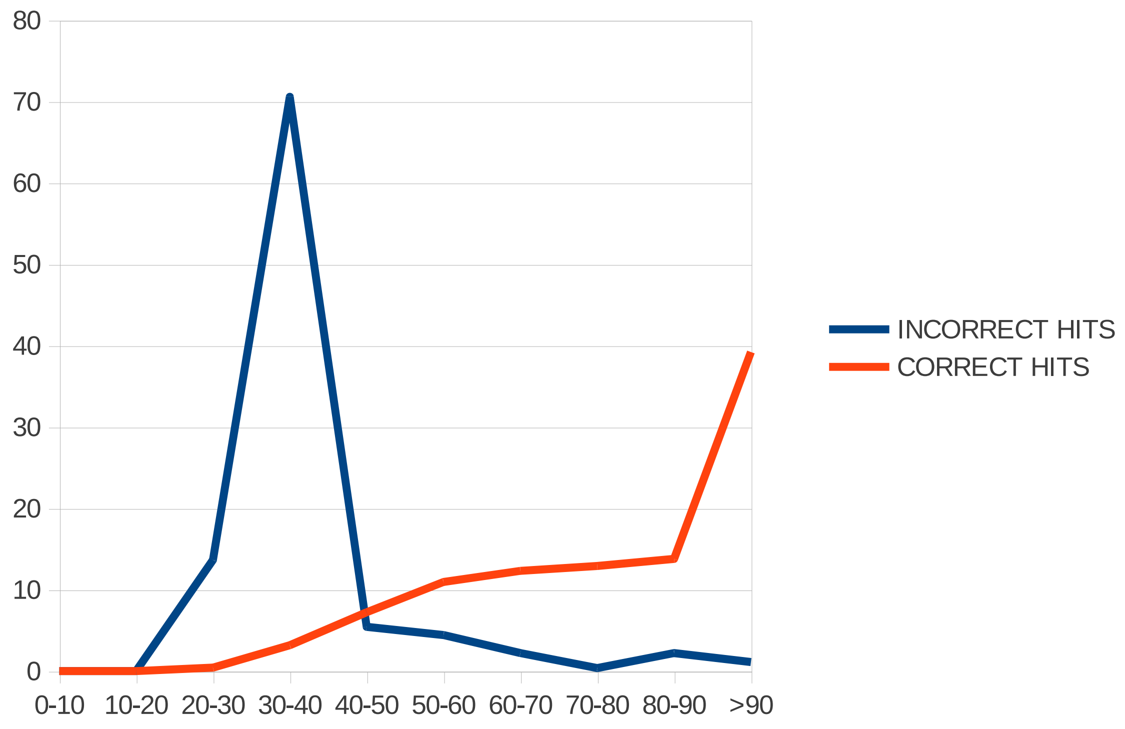

Figure ST1: Percentage of identity values in various ranges for 'INCORRECT' and 'CORRECT' hits obtained in the Validation Test 1 after removing the self-hits (hits in which the subject and the query are the same) as well as hits from the source genome of the sequence

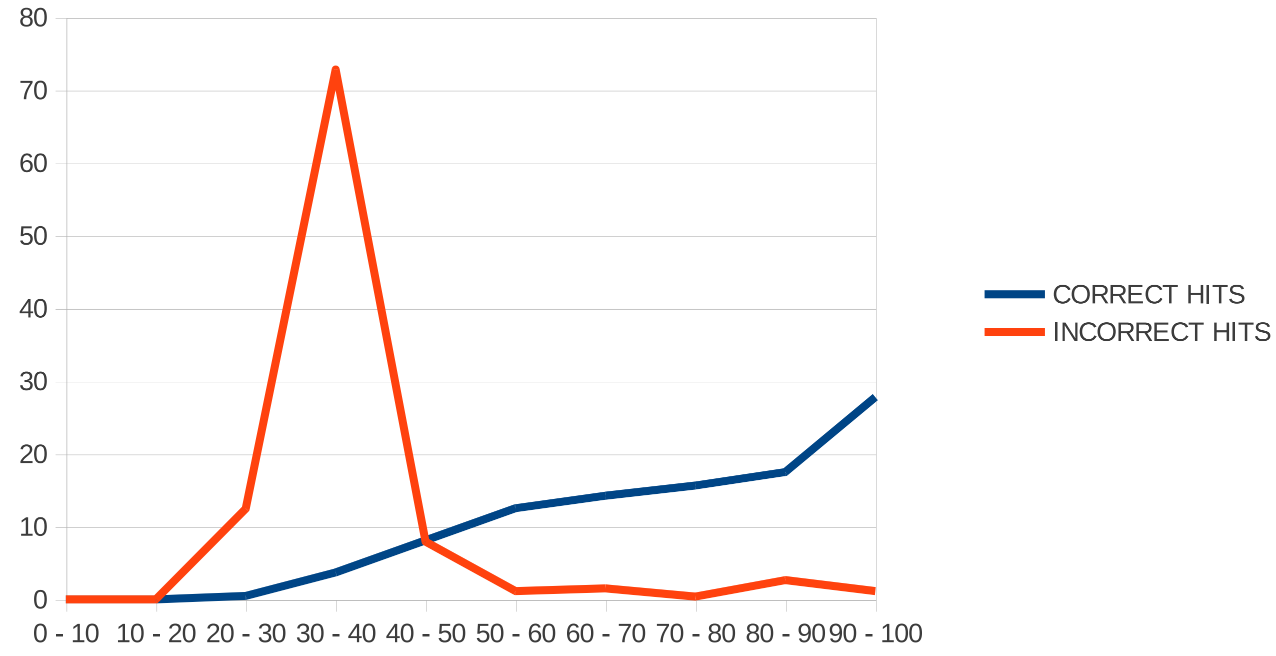

Figure ST2: Percentage of identity values in various ranges for 'INCORRECT' and 'CORRECT' hits obtained in the Validation Test 1 after removing all hits from the same species as the source genome of the sequence.

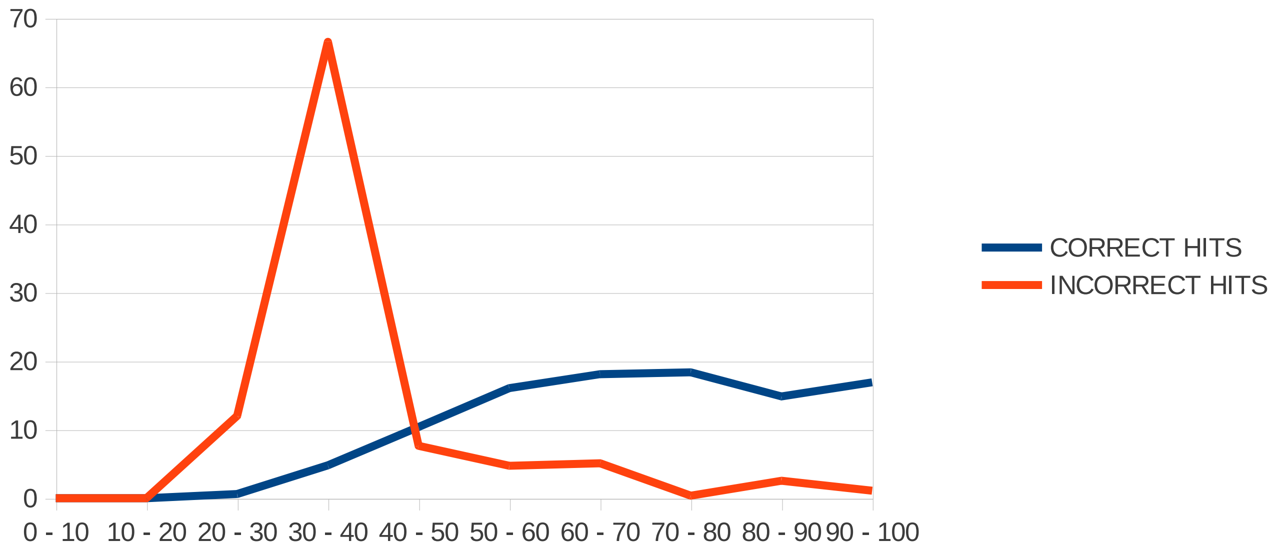

Figure ST3: Percentage of identity values in various ranges for 'INCORRECT' and 'CORRECT' hits obtained in the Validation Test 1 after removing all hits from the same genus as the source genome of the sequence.

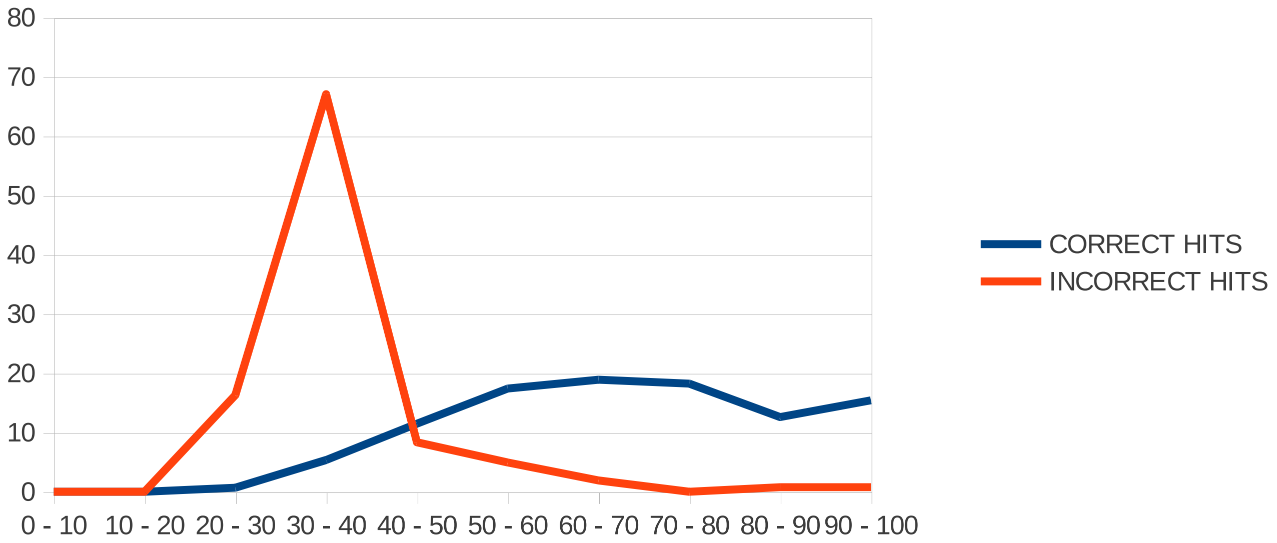

Figure ST4: Percentage of identity values in various ranges for 'INCORRECT' and 'CORRECT' hits obtained in the Validation Test 1 after removing all hits from the same family as the source genome of the sequence.

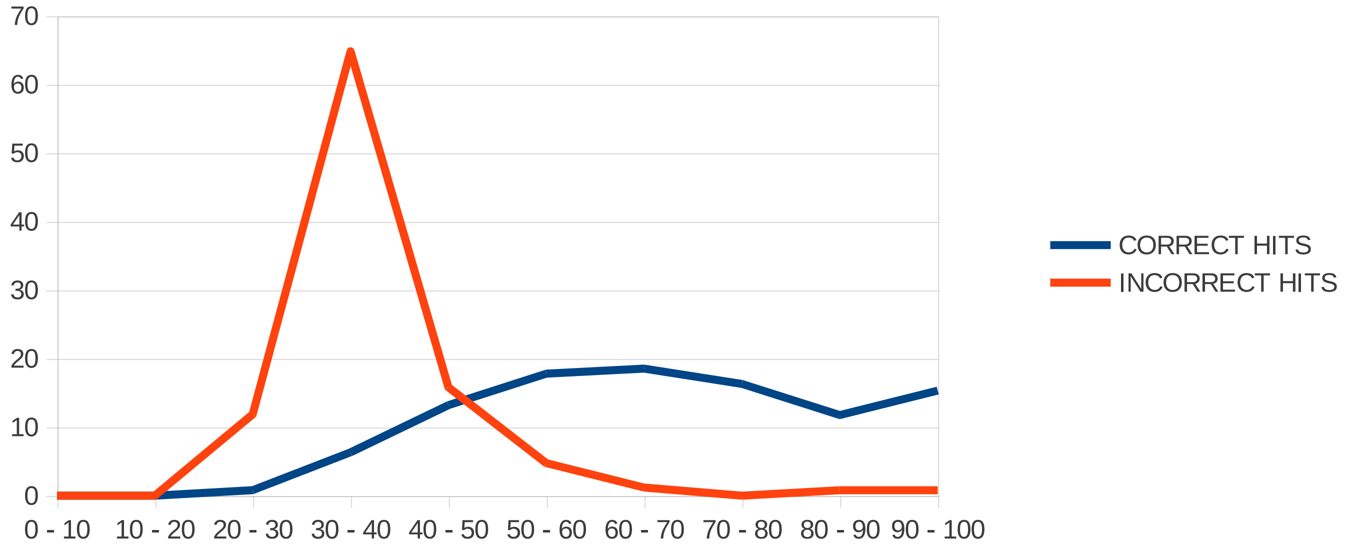

Figure ST5: Percentage of identity values in various ranges for 'INCORRECT' and 'CORRECT' hits obtained in the Validation Test 1 after removing all hits from the same order as the source genome of the sequence.

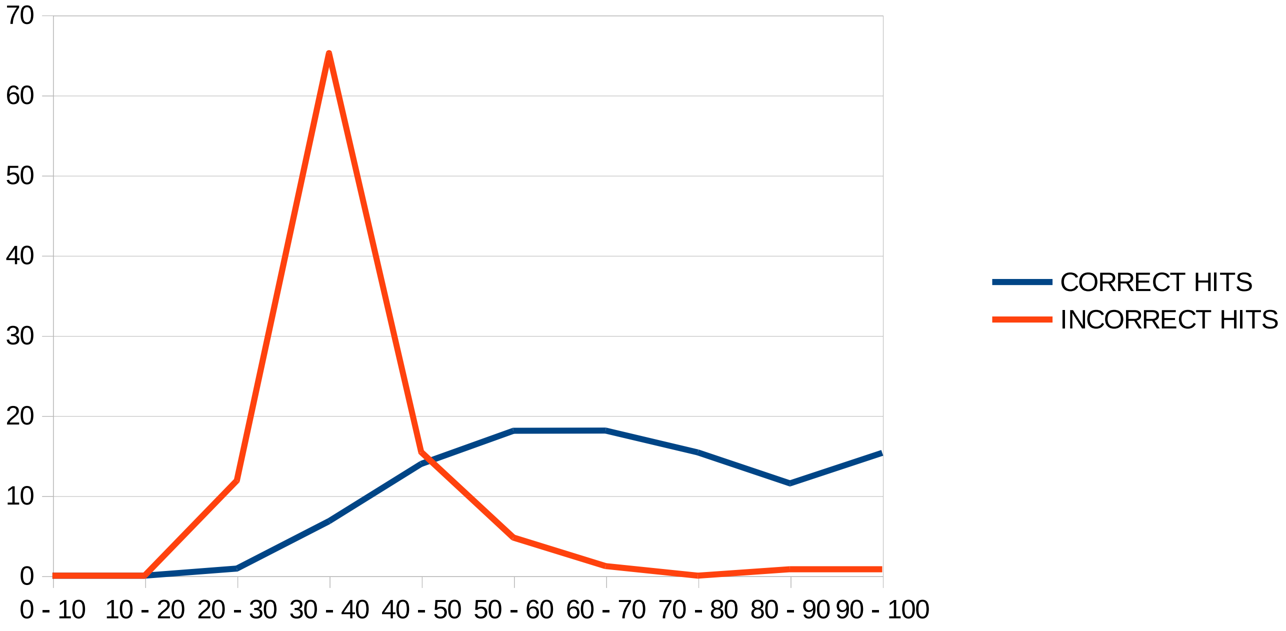

Figure ST6: Percentage of identity values in various ranges for 'INCORRECT' and 'CORRECT' hits obtained in the Validation Test 1 after removing all hits from the same class as the source genome of the sequence.

VALIDATION TEST 2

METHODOLOGY: The second test was much more comprehensive and was performed to investigate the rate of spurious hit detection using the 40% identity threshold. In this test, all the annotated E.C numbers in the reference database of xenobiotic degrading enzymes were selected. For each E.C number, a list of E.C numbers were obtained from the BRENDA database (http://www.brenda-enzymes.org/), such that the obtained list of 1062 E.C numbers are functionally related to the given (xenobiotic degrading) E.C number, but are not known to be involved in xenobiotic degradation. The sequences corresponding to these functionally related yet xenobiotic non-degrading enzymes were subsequently selected from the nr database. This was referred to as 'Validation Database 2'.

The above procedure was repeated for all the E.C numbers present in the reference database of xenobiotic degrading enzymes used in the current study. This resulted in the creation of a 'negative-control' dataset, containing sequences from functionally related enzymatic families that have the highest likelihood of being detected as spurious hits to the xenobiotic degrading enzymes.

Subsequently, a BLASTp search of the sequences in this negative-control dataset was performed against the reference database of xenobiotic degrading enzymes, to obtain the distribution of identity values of the resulting 'spurious' hits in various identity ranges.

RESULTS: As observed in Figure 7, the maximum number of such hits (> 50%) were observed to have identities between 20-30%. On the other hand, the percentage of such hits having identity

values of greater than 40% was observed to be only around 10%. This further indicates that using the identity threshold of greater than 40% (used in this study) is reasonably accurate in reducing the rate of false positives.

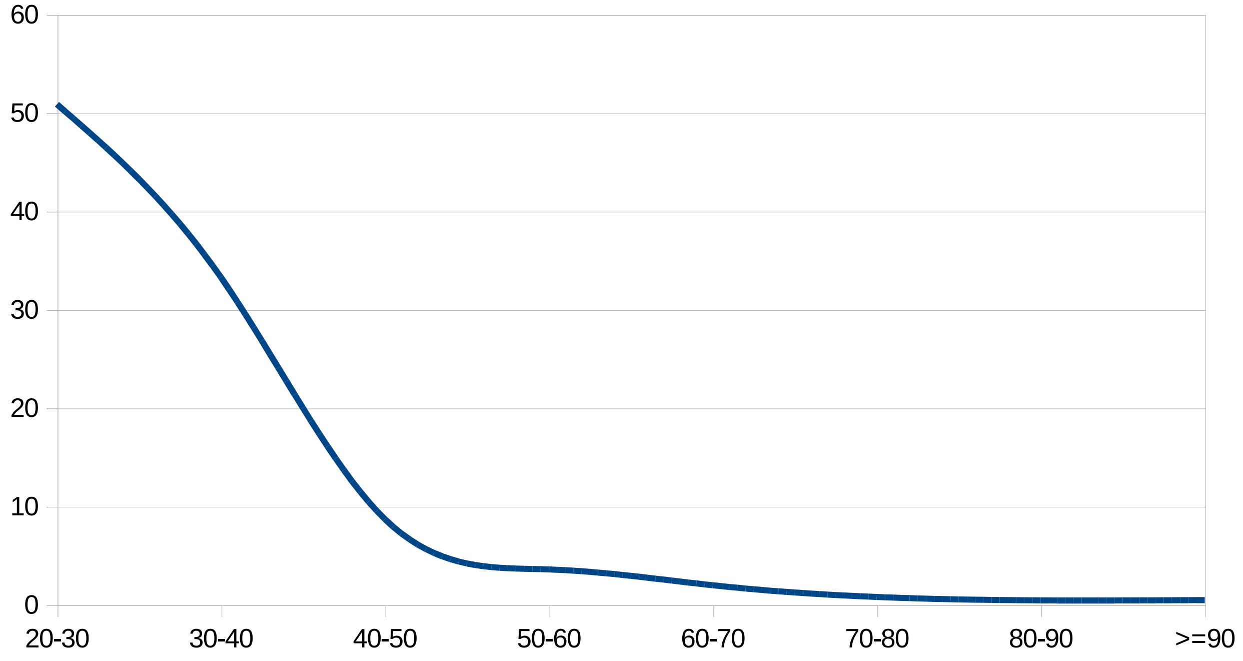

Figure ST7: Percentage of 'spurious' hits having identities in various ranges in the Validation Test 2
